# Data concerning statistical relation between obliquity and Dansgaard–Oeschger events

**DOI:** 10.1016/j.dib.2019.103727

**Published:** 2019-03-07

**Authors:** Jia Deng, Zhaohua Wu, Min Zhang, Norden E. Huang, Shizhu Wang, Fangli Qiao

**Affiliations:** a First Institute of Oceanography, Ministry of Natural Resources, Qingdao 266061, PR China; b Laboratory for Regional Oceanography and Numerical Modelling, Qingdao National Laboratory for Marine Science and Technology, Qingdao 266071, PR China; c Key Laboratory of Data Analysis and Applications, Ministry of Natural Resources, Qingdao 266061, PR China; d Department of Earth, Ocean and Atmospheric Science & Center for Ocean-Atmospheric Prediction Studies, Florida State University, Tallahassee FL 32306, USA; e Key Laboratory of Marine Sciences and Numerical Modelling, Ministry of Natural Resources, Qingdao 266061, PR China

**Keywords:** Dansgaard–Oeschger events, Obliquity, Time-varying Shannon entropy, Surrogate data

## Abstract

Data presented are related to the research article entitled “Using Holo-Hilbert spectral analysis to quantify the modulation of Dansgaard-Oeschger events by obliquity” (J. Deng et al., 2018). The datasets in Deng et al. (2018) are analyzed on the foundation of ensemble empirical mode decomposition (EEMD) (Z.H. Wu and N.E. Huang, 2009), and reveal more occurrences of Dansgaard-Oeschger (DO) events in the decreasing phase of obliquity. Here, we report the number of significant high Shannon entropy (SE) (C.E. Shannon and W. Weaver, 1949) of 95% significance level of DO events in the increasing and decreasing phases of obliquity, respectively. First, the proxy time series are filtered by EEMD to obtain DO events. Then, the time-varying SE of DO modes are calculated on the basis of principle of histogram. The 95% significance level is evaluated through surrogate data (T. Schreiber and A. Schmitz, 1996). Finally, a comparison between the numbers of SE values that are larger than 95% significance level in the increasing and decreasing phases of obliquity, respectively, is reported.

**Table:** Specifications table

Subject area	Earth and Planetary Sciences
More specific subject area	Pleistocene; Paleoclimatology; Data analysis.
Type of data	Table, figure, text files
How data was acquired	http://www.iceandclimate.nbi.ku.dk/data/, https://www.ncdc.noaa.gov/paleo-search/study/6080, https://www.ncdc.noaa.gov/paleo-search/study/2453, calibrated isotope data, and ftp://ftp.ncdc.noaa.gov/pub/data/paleo/insolation/, model data.
Data format	Raw and filtered.
Experimental factors	None.
Experimental features	A statistical relation between the phase of obliquity and occurrences of Dansgaard–Oeschger events.
Data source location	Greenland, Eastern Antarctic
Data accessibility	Data is with this article. Calibrated secondary data package title: NGRIP, GRIP and GISP2 $\delta^{18}O$ and calcium concentration in 20 years means on the GICC05 modelext timescale [5], [6] Resource link: http://www.iceandclimate.nbi.ku.dk/data/Identiﬁer: https://doi.org/10.1016/j.quascirev.2014.10.032https://doi.org/10.1016/j.quascirev.2014.09.007 Calibrated secondary data package title: EPICA Dome C Ice Core 800KYr Deuterium Data and Temperature Estimates [7] Resource link: https://www.ncdc.noaa.gov/paleo-search/study/6080 Identiﬁer: https://doi.org/10.1126/science.1141038 Calibrated secondary data package title: Orbital Variations and Insolation Database [8]. Resource link: ftp://ftp.ncdc.noaa.gov/pub/data/paleo/insolation/Identiﬁer: https://doi.org/10.1016/0277-3791(91)90033-Q
Related research article	Deng J, Wu ZH, Zhang M, Huang NE, Wang SZ, Qiao FL. Using Holo-Hilbert spectral analysis to quantify the modulation of Dansgaard-Oeschger events by obliquity. Quaternary Sci Rev. 2018 192: 282–299 [1].

**Table:** 

**Value of the data** • The Data provide an insight into the role of decreasing obliquity in Dansgaard-Oeschger events recorded in the Greenland region. • The Data help to investigate the role of obliquity in Dansgaard-Oeschger events recorded in the Antarctic region. • The Data would facilitate to distinguish Dansgaard-Oeschger events recorded in the Greenland region from those recorded in the eastern Antarctic region.

## 1 Data

Datasets reported here are related to the statistical relation between obliquity and Dansgaard-Oeschger (DO) events proposed in [1]. All the computations have been conducted on an ordinary laptop using Matlab software. Time-varying Shannon entropy (SE) [3] of DO events recorded in the oxygen isotope ($\delta^{18}\text{O}$) records from three Greenland ice cores (NGRIP, GRIP and GISP2 on GICC05 modelext timescale) [5], [6] and the deuterium isotope (δD) records from EPICA Dome C Ice Core (EDC) in Antarctic region have been calculated [7]. The $\delta^{18}\text{O}$ time series have time resolutions of Δt=20 years and δD time series has been interpolated onto a uniform grid with time span of Δt=20 years. All the isotope time series have been truncated to the range of 2–99 kyr. BP (kyr. BP = 1,000 years before present).

To contrast with the analysis in [1], we compare the number of significant high SE (i.e., the SE that is larger than the 95% significance level) in the decreasing obliquity phase with the number of significant high SE in the increasing obliquity phase. To track the change of SE over the whole temporal domain, a sliding window has been added to DO time series. The window size, M, is set as M=300 points (i.e., a window spans 6,000 years) and the length of overlap region between two consecutive windows, L, is set as L=30 points. By repeating the computations using different M and L satisfying M≫300 and L≫30, results will not be essentially changed.

## 2 Experimental design, materials, and methods

**Fig. 1:**
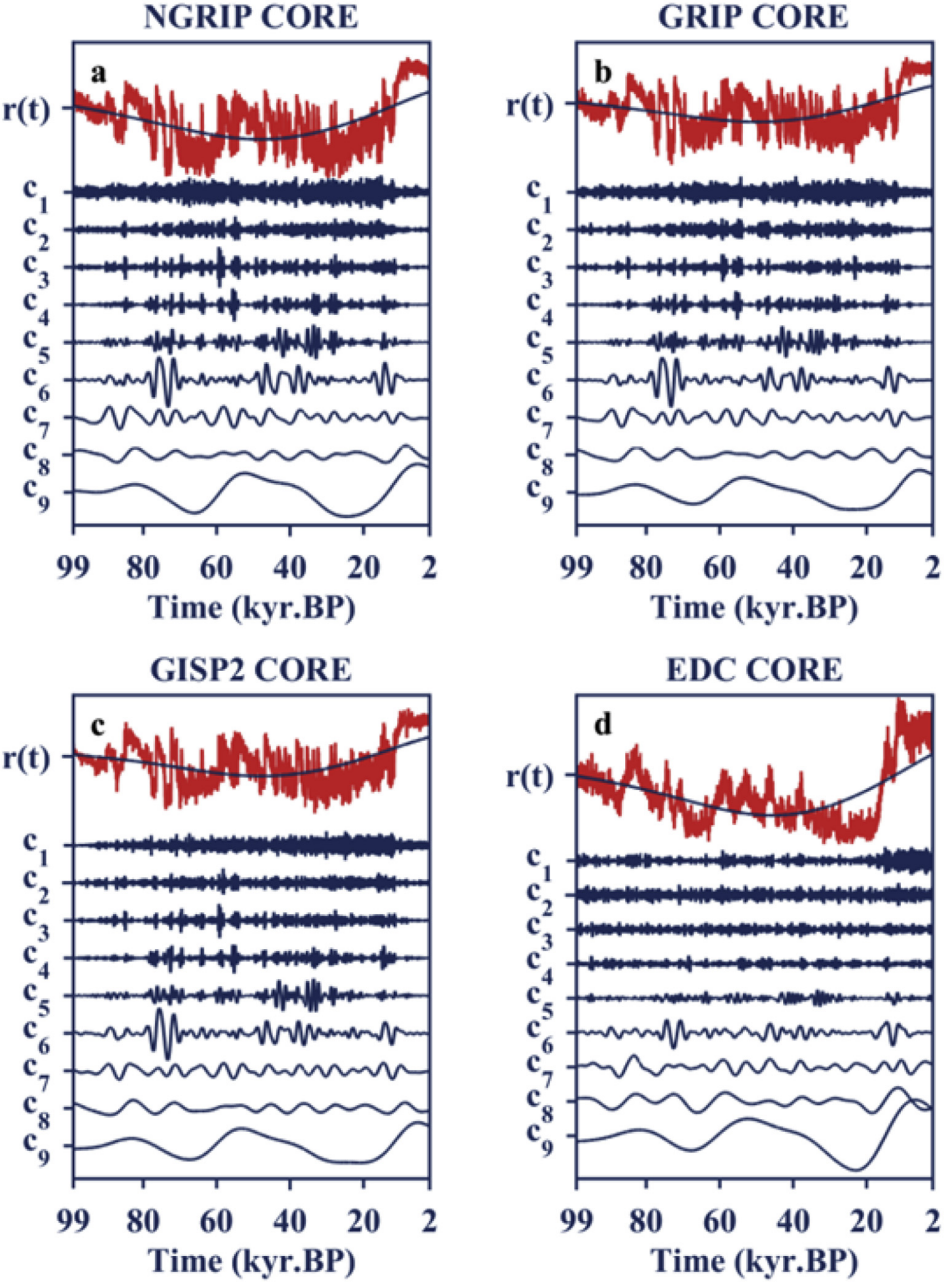
a–c: Ensemble empirical mode decomposition (EEMD) of $\delta^{18}\text{O}$ time series recorded in Greenland ice cores. d: EEMD of δD time series recorded in EPICA Dome C Ice Core (EDC) in the Antarctic. The solid red lines are calibrated isotope data. The solid blue lines are the decomposed intrinsic mode functions signed by $c_{j},\;j=1,2,\cdots ,9$ and the trend signed by r(t).

**Fig. 2:**
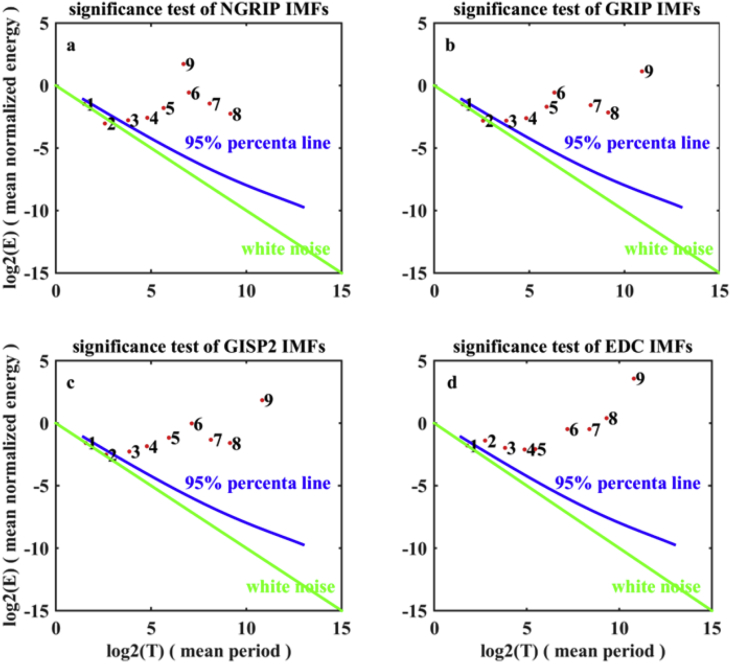
Significance tests of the intrinsic mode functions (IMFs) shown in Fig. 1.

Implementation details of counting the numbers of significant high SE during increasing and decreasing phases of obliquity, respectively, have been provided. In the first stage, EEMD [2] is applied to $\delta^{18}\text{O}$ and δD records to extract DO modes which are represented by the intrinsic oscillations (intrinsic mode function, IMF) with durations not exceeding 5000 years. The resultant decompositions are shown in Fig. 1 and the significance tests of decomposed components are given in Fig. 2. The mean periods of statistical significant IMFs are estimated via the Hilbert transform and presented in Table 1. In the second stage, applying a sliding window to DO modes to obtain a series of small segments and calculating the SE for each of these segments with time coordinate defined as the mean time of the corresponding window. The 95% significance level is estimated with the aid of surrogate data, following the idea of bootstrap method in [1]. The numbers of significant high SE in the increasing obliquity phase and decreasing obliquity phase, respectively, are shown in Table 2, Table 3, Table 4, Table 5.

**Fig. 3:**
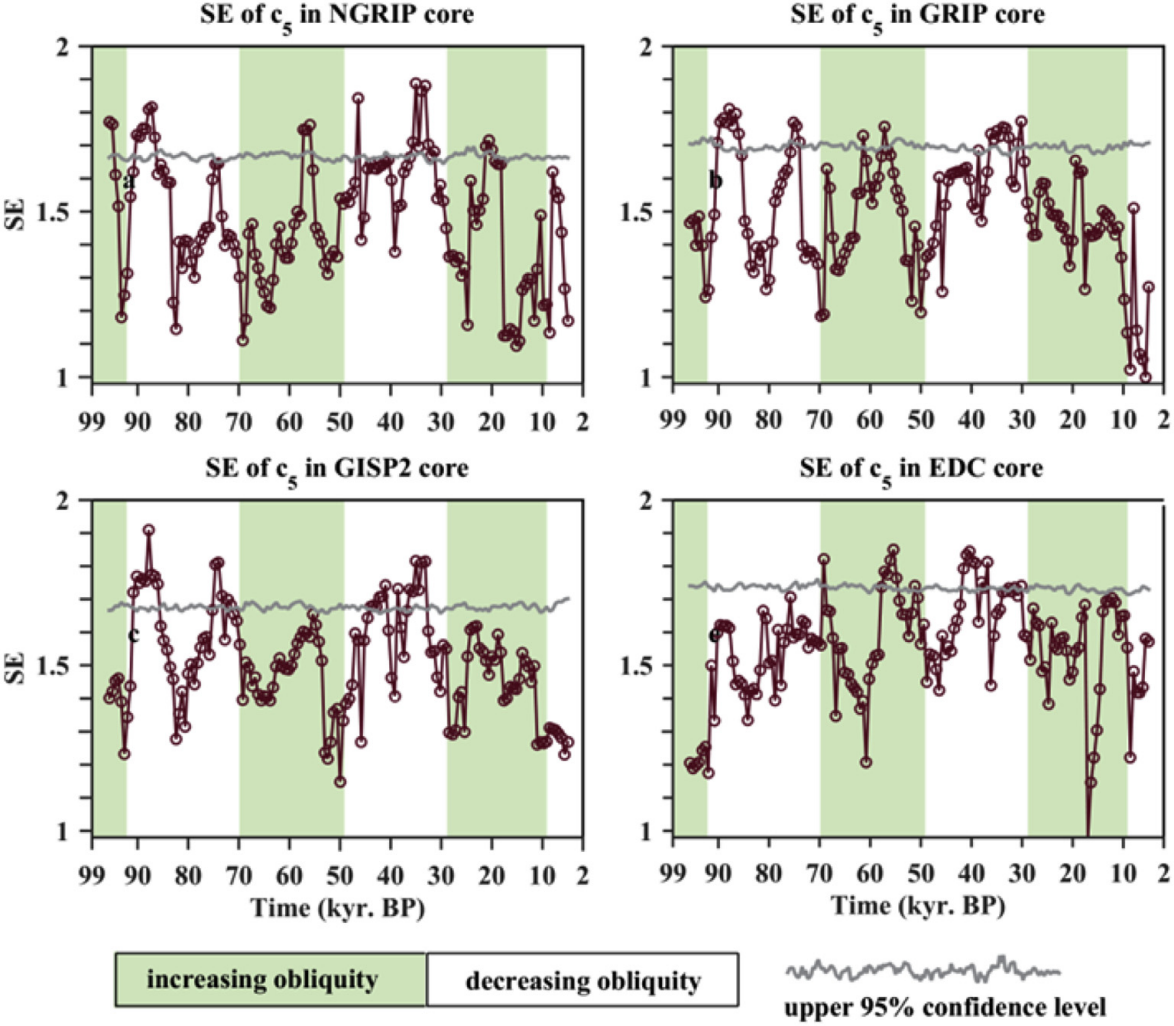
Time-varying Shannon entropy (SE) of the 5th mode, $c_{5}$ shown Fig. 1, of isotope time series.

**Table 1:** Mean Period (T years) of statistical significant intrinsic mode functions signed by $c_{j}$ and shown in Fig. 1, Fig. 2. Since the second modes, $c_{2}$, in the Greenland ice cores are statistical insignificant, their mean periods are not reported and replaced by the symbol “#”.

T (years)	$c_{2}$	$c_{3}$	$c_{4}$	$c_{5}$	$c_{6}$	$c_{7}$	$c_{8}$	$c_{9}$
NGRIP	#	280	614	1,330	2,700	6,087	12,225	49,883
GRIP	#	283	606	1,312	3,241	6,065	10,861	32,996
GISP2	#	299	630	1,311	3,136	5,730	10,859	32,968
EDC	153	295	618	1,260	3,350	8,120	12,219	32,936

**Table 2:** Numbers of significant high Shannon entropy (SE) of DO modes ($c_{3}∼c_{5}$ shown in Fig. 1) recorded in the NGRIP core (i.e., numbers of circles locating above the 95% significance level. As an example, see Fig. 3) during the decreasing obliquity (white regions) and increasing obliquity (light grey regions) stages.

Time (kyr. BP)	70–92	49–70	29–49	9–29
$c_{3}$	0	0	8	7
$c_{4}$	4	0	14	2
$c_{5}$	7	3	9	3
$c_{6}$	8	0	0	3

**Table 3:** Numbers of significant high Shannon entropy (SE) of DO modes ($c_{3}∼c_{5}$ shown in Fig. 1) recorded in the GRIP core (i.e., numbers of circles locating above the 95% significance level. As an example, see Fig. 3) during the decreasing obliquity (white regions) and increasing obliquity (light grey regions) stages.

Time (kyr. BP)	70–92	49–70	29–49	9–29
$c_{3}$	0	0	15	13
$c_{4}$	2	0	5	2
$c_{5}$	11	2	10	0
$c_{6}$	2	0	1	0

**Table 4:** Numbers of significant high Shannon entropy (SE) of DO modes ($c_{3}∼c_{5}$ shown in Fig. 1) recorded in the GISP2 core (i.e., numbers of circles locating above the 95% significance level. As an example, see Fig. 3) during the decreasing obliquity (white regions) and increasing obliquity (light grey regions) stages.

Time (kyr. BP)	70–92	49–70	29–49	9–29
$c_{3}$	0	1	15	9
$c_{4}$	3	0	9	5
$c_{5}$	14	0	14	0
$c_{6}$	8	3	2	0

**Table 5:** Numbers of significant high Shannon entropy (SE) of DO modes ($c_{2}∼c_{5}$ shown in Fig. 1) recorded in the EPICA Dome C Ice Core (i.e., numbers of circles locating above the 95% significance level. As an example, see Fig. 3) during the decreasing obliquity (white regions) and increasing obliquity (light grey regions) stages.

Time (kyr. BP)	70–92	49–70	29–49	9–29
$c_{2}$	1	0	0	1
$c_{3}$	0	0	2	0
$c_{4}$	2	0	2	3
$c_{5}$	0	7	10	0
$c_{6}$	1	8	2	0

### 2.1 Shannon entropy

Shannon entropy (SE) is widely defined using the occurrence probability of different states (values) of a time series over the whole data span, i.e.,

(1) $$SE=-p\left(x\right)\log_{10}p\left(x\right)$$

where *x* is a state of a time series and *p*(*x*) is the probability of the occurrence of state *x*. It can be verified that SE are small when only a few possible states can occur and large when the time series are evenly distributed at numerous states (ergodic).

### 2.2 Estimating 95% significance level

Surrogate data is one of the popular methods that are capable of evaluating whether a time series arises from a deterministic or a stochastic dynamic system. The technique has the idea of comparing a specific statistical property of the data with the empirical distribution of the same property generated by a set of surrogates (i.e., a constructed data that is comparable with original data but shares none of the tested property). In this data article, we use the common iterative amplitude adjusted Fourier transform (IAAFT) algorithm to produce B=1000 surrogates. Details of IAAFT can be found in [4]. Then, we calculate the time-varying SE for these surrogates, using the same M=300 and L=30, as was done for the original data, and obtain an empirical distribution of SE. The 95% significance level is defined as the α-quantiles (where we set α=0.05) of this distribution.
