# Supplementary material for: Data concerning statistical relation between obliquity and Dansgaard–Oeschger events
Source: Data Brief. 2019 Mar 7;23:103727. doi: 10.1016/j.dib.2019.103727 (PMC6660458; doi:10.1016/j.dib.2019.103727)
Supplement: Multimedia component 2 [file mmc2.pdf]

Processed oxygen isotope (d18O) data from GISP2 ice core on GICC05 modelext timescale
----
NAME OF DATA SET:
Processed oxygen isotope (d18O) data from GISP2 ice core on GICC05 modelext timescale

LAST UPDATE: 21/12/2018

ORIGINAL REFERENCE:
Jia Deng, Zhaohua Wu, Min Zhanga, Norden. E Huang, Shizhu Wang,Fangli Qiao. 2018.
Data concerning statistical relation between obliquity and Dansgaard-Oeschger events.
Data in Brief, in press.

MAIN REFERENCES:
Datafile of the original data accompanies the following two papers:

Rasmussen, S. O et al., 2014. A stratigraphic framework for abrupt climatic changes during the Last
Glacial period based on three synchronized Greenland ice-core records: refining and extending the
INTIMATE event stratigraphy. Quaternary. Sci. Rev. 106: 14-28.

Seierstad, I. K et al., 2014. Consistently dated records from the Greenland GRIP, GISP2 and NGRIP
ice cores for the past 104 ka reveal regional millennial-scale ヲﾄ18O gradients with possible Heinrich
event imprint. Quaternary. Sci. Rev. 106: 29-46.

ABSTRACT:
Data presented are related to the research article entitled 。-Using Holo-Hilbert spectral analysis to
quantify the modulation of Dansgaard-Oeschger events by obliquity。ｱ [1]. The datasets in Deng et al (2018)
are analyzed on the foundation of ensemble empirical mode decomposition (EEMD) [2], and reveal more occurrences
of Dansgaard-Oeschger (DO) events in the decreasing phase of obliquity. Here, we report the number of significant
high Shannon entropy (SE) [3] of 95% significance level of DO events in the increasing and decreasing phases of
obliquity, respectively. First, the proxy time series are filtered by EEMD to obtain DO events. Then, the
time-varying SE of DO modes are calculated on the basis of principle of histogram. The 95% significance level
is evaluated through surrogate data [4]. Finally, a comparison between the numbers of SE values that are larger
than 95% significance level in the increasing and decreasing phases of obliquity, respectively, is reported.

GEOGRAPHIC REGION: Greenland

PERIOD OF RECORD: 99 kyr. BP - 2 kyr.BP

FUNDING SOURCES:
This work was jointly supported by the National Basic Research Program of China (Grant 2012CB957802);
the National Natural Science Foundation of China (NSFC) (Grant 41506067); the Basic Scientific Fund
for National Public Research Institutes of China (Grant 2015G04); the US National Science Foundation
(Grant AGS-1723300); the NSFC-Shandong Joint Fund for Marine Science Research Centers (Grant U1406404);
and the National Programme on Global Change and Air-Sea Interaction (Grant GASI-IPOVAI-05).

DATA:
Calibrated GISP2 oxygen isotope (d18O) data on GICC05modeext timescale (Rasmussen et al, 2014; Seierstad et al, 2014).
Processed data by ensemble empirical mode decomposition (EEMD).

Column 1: Time (kyr. BP)
Column 2: (Calibrated secondary) d18O data (years before b2k)
Column 3: 1st EEMD component of d18O records
Column 4: 2nd EEMD component of d18O records
Column 5: 3rd EEMD component of d18O records
Column 6: 4th EEMD component of d18O records
Column 7: 5th EEMD component of d18O records
Column 8: 6th EEMD component of d18O records
Column 9: 8th EEMD component of d18O records
Column 10: 9th EEMD component of d18O records
Column 11: 10th EEMD component of d18O records
Column 12: EEMD trend of d18O records

Time	d18O data	IMF1	IMF2	IMF3	IMF4	IMF5	IMF6	IMF7	IMF8	IMF9	trend
2.0000	-34.5800	0.0390	-0.0222	0.0107	0.0388	0.0541	-0.0094	-0.0476	-0.2676	2.1011	-36.4601
2.0200	-34.8079	-0.0040	-0.1916	-0.0084	0.0411	0.0539	-0.0111	-0.0482	-0.2688	2.1047	-36.4623
2.0400	-34.8500	-0.0302	-0.2356	0.0022	0.0423	0.0533	-0.0129	-0.0488	-0.2699	2.1084	-36.4645
2.0600	-34.6300	-0.0065	-0.1236	0.0413	0.0412	0.0522	-0.0148	-0.0494	-0.2710	2.1120	-36.4666

2.0800 -34.5400 -0.0684 0.0694
0.0859 0.0372 0.0507 -0.0168 -0.0500
-0.2721 2.1157 -36.4688
2.1000 -34.0200 0.1791 0.2121
0.1115 0.0303 0.0488 -0.0189 -0.0506
-0.2732 2.1194 -36.4709
2.1200 -34.4300 -0.1226 0.2072
0.1061 0.0207 0.0467 -0.0211 -0.0512
-0.2743 2.1230 -36.4731
2.1400 -34.1700 0.2140 0.0734
0.0707 0.0094 0.0443 -0.0234 -0.0518
-0.2754 2.1267 -36.4752
2.1600 -34.9300 -0.1148 -0.1295
0.0225 -0.0027 0.0418 -0.0257 -0.0524
-0.2766 2.1303 -36.4774
2.1800 -35.1100 -0.0680 -0.2644 -
0.0192 -0.0143 0.0392 -0.0281 -0.0530
-0.2777 2.1340 -36.4796
2.2000 -34.8400 0.0906 -0.2166 -
0.0436 -0.0245 0.0366 -0.0305 -0.0537
-0.2789 2.1377 -36.4817
2.2200 -34.7000 -0.0044 -0.0034 -
0.0523 -0.0326 0.0339 -0.0330 -0.0543
-0.2800 2.1413 -36.4839
2.2400 -34.7500 -0.1902 0.2270 -
0.0538 -0.0380 0.0312 -0.0355 -0.0549
-0.2812 2.1450 -36.4860
2.2600 -34.3000 0.1869 0.2949 -
0.0555 -0.0401 0.0285 -0.0380 -0.0556
-0.2823 2.1487 -36.4882
2.2800 -34.6800 0.0667 0.0943 -
0.0591 -0.0389 0.0258 -0.0405 -0.0563
-0.2835 2.1523 -36.4904
2.3000 -35.0400 -0.0070 -0.2109 -
0.0540 -0.0345 0.0230 -0.0430 -0.0569
-0.2847 2.1560 -36.4925
2.3200 -35.2600 -0.3158 -0.3271 -
0.0293 -0.0273 0.0202 -0.0455 -0.0576
-0.2859 2.1596 -36.4947
2.3400 -34.4300 0.3923 -0.2074
0.0174 -0.0181 0.0173 -0.0479 -0.0583
-0.2871 2.1633 -36.4969
2.3600 -34.8900 -0.2738 -0.0016
0.0744 -0.0081 0.0143 -0.0502 -0.0590
-0.2883 2.1670 -36.4990
2.3800 -34.4300 -0.0286 0.1991
0.1174 0.0019 0.0112 -0.0525 -0.0597
-0.2895 2.1706 -36.5012
2.4000 -33.9700 0.3044 0.3305
0.1247 0.0106 0.0079 -0.0546 -0.0605
-0.2907 2.1743 -36.5033

2.4200 -34.4000 -0.1002 0.2533
0.0894 0.0172 0.0046 -0.0567 -0.0612
-0.2919 2.1779 -36.5055
2.4400 -34.7500 0.0038 0.0021
0.0261 0.0210 0.0012 -0.0586 -0.0619
-0.2932 2.1816 -36.5077
2.4600 -34.9900 -0.0592 -0.2065 -
0.0401 0.0218 -0.0023 -0.0604 -0.0627
-0.2944 2.1852 -36.5098
2.4800 -34.9600 0.1516 -0.2636 -
0.0823 0.0195 -0.0059 -0.0620 -0.0635
-0.2956 2.1889 -36.5120
2.5000 -35.2600 -0.2625 -0.1706 -
0.0841 0.0147 -0.0094 -0.0634 -0.0642
-0.2969 2.1925 -36.5142
2.5200 -34.4400 0.3878 -0.0211 -
0.0485 0.0083 -0.0128 -0.0647 -0.0650
-0.2982 2.1962 -36.5163
2.5400 -35.1500 -0.3795 0.0704
0.0051 0.0008 -0.0162 -0.0658 -0.0658
-0.2994 2.1998 -36.5185
2.5600 -34.4500 0.1547 0.1533
0.0508 -0.0071 -0.0195 -0.0667 -0.0666
-0.3007 2.2034 -36.5207
2.5800 -34.5200 0.0702 0.1978
0.0695 -0.0148 -0.0227 -0.0674 -0.0675
-0.3020 2.2071 -36.5228
2.6000 -34.6100 -0.0078 0.1215
0.0555 -0.0216 -0.0258 -0.0679 -0.0683
-0.3033 2.2107 -36.5250
2.6200 -34.9600 -0.1657 -0.0169
0.0188 -0.0269 -0.0287 -0.0682 -0.0691
-0.3046 2.2143 -36.5272
2.6400 -34.8300 0.1033 -0.0967 -
0.0206 -0.0301 -0.0316 -0.0683 -0.0700
-0.3059 2.2179 -36.5294
2.6600 -35.0100 -0.1381 -0.0968 -
0.0486 -0.0307 -0.0342 -0.0681 -0.0709
-0.3072 2.2215 -36.5315
2.6800 -34.7000 0.3351 -0.0993 -
0.0576 -0.0282 -0.0367 -0.0677 -0.0717
-0.3085 2.2251 -36.5337
2.7000 -35.1300 -0.2357 -0.1087 -
0.0500 -0.0225 -0.0390 -0.0671 -0.0726
-0.3098 2.2287 -36.5359
2.7200 -35.0700 -0.1731 0.0224 -
0.0375 -0.0138 -0.0411 -0.0663 -0.0735
-0.3111 2.2323 -36.5380
2.7400 -34.4400 0.1707 0.2145 -
0.0311 -0.0025 -0.0430 -0.0652 -0.0745
-0.3124 2.2359 -36.5402

2.7600 -34.2300 0.3527 0.2120 -
0.0342 0.0106 -0.0445 -0.0639 -0.0754
-0.3137 2.2395 -36.5424
2.7800 -35.0600 -0.1843 -0.0000 -
0.0421 0.0243 -0.0456 -0.0623 -0.0763
-0.3150 2.2430 -36.5446
2.8000 -35.1700 -0.1636 -0.1714 -
0.0443 0.0368 -0.0463 -0.0606 -0.0773
-0.3163 2.2466 -36.5467
2.8200 -34.7900 0.1466 -0.1543 -
0.0304 0.0464 -0.0465 -0.0586 -0.0783
-0.3176 2.2501 -36.5489
2.8400 -34.6800 0.0736 -0.0251
0.0034 0.0515 -0.0462 -0.0564 -0.0793
-0.3190 2.2537 -36.5511
2.8600 -34.7700 -0.1267 0.0791
0.0543 0.0515 -0.0453 -0.0540 -0.0803
-0.3203 2.2572 -36.5533
2.8800 -34.5400 0.0590 0.0811
0.1099 0.0460 -0.0437 -0.0514 -0.0813
-0.3216 2.2607 -36.5555
2.9000 -34.4500 0.1770 -0.0098
0.1500 0.0353 -0.0416 -0.0486 -0.0823
-0.3229 2.2643 -36.5576
2.9200 -35.1000 -0.4555 -0.0568
0.1584 0.0199 -0.0388 -0.0456 -0.0834
-0.3242 2.2678 -36.5598
2.9400 -34.3500 0.3540 0.0343
0.1289 0.0012 -0.0353 -0.0425 -0.0844
-0.3255 2.2713 -36.5620
2.9600 -34.5700 -0.0042 0.1114
0.0658 -0.0190 -0.0310 -0.0392 -0.0855
-0.3268 2.2747 -36.5642
2.9800 -34.6900 -0.0072 0.0236 -
0.0117 -0.0387 -0.0261 -0.0358 -0.0866
-0.3281 2.2782 -36.5664
3.0000 -35.0500 -0.0000 -0.1158 -
0.0807 -0.0558 -0.0205 -0.0322 -0.0877
-0.3294 2.2817 -36.5686
3.0200 -35.2500 -0.1522 -0.1334 -
0.1250 -0.0687 -0.0143 -0.0286 -0.0888
-0.3307 2.2851 -36.5707
3.0400 -34.9100 0.1076 -0.0308 -
0.1381 -0.0761 -0.0076 -0.0248 -0.0899
-0.3319 2.2886 -36.5729
3.0600 -34.6900 0.1211 0.0783 -
0.1240 -0.0772 -0.0005 -0.0210 -0.0911
-0.3332 2.2920 -36.5751
3.0800 -34.8600 -0.1259 0.1265 -
0.0938 -0.0717 0.0069 -0.0171 -0.0922
-0.3345 2.2954 -36.5773

3.1000 -34.7100 -0.0609 0.1015 -
0.0605 -0.0596 0.0144 -0.0132 -0.0934
-0.3358 2.2988 -36.5795
3.1200 -34.3900 0.3087 -0.0047 -
0.0305 -0.0419 0.0219 -0.0093 -0.0946
-0.3370 2.3022 -36.5817
3.1400 -34.8400 -0.0769 -0.1154 -
0.0070 -0.0203 0.0293 -0.0053 -0.0958
-0.3383 2.3055 -36.5839
3.1600 -35.1200 -0.4113 -0.0809
0.0093 0.0034 0.0363 -0.0014 -0.0971
-0.3395 2.3089 -36.5861
3.1800 -34.0800 0.4981 0.0399
0.0215 0.0267 0.0430 0.0024 -0.0983
-0.3407 2.3122 -36.5883
3.2000 -34.5600 -0.0879 0.0564
0.0334 0.0476 0.0490 0.0062 -0.0996
-0.3419 2.3156 -36.5905
3.2200 -34.7400 -0.1516 -0.0272
0.0510 0.0641 0.0544 0.0100 -0.1009
-0.3431 2.3189 -36.5927
3.2400 -34.5600 -0.0562 -0.0791
0.0761 0.0750 0.0589 0.0136 -0.1022
-0.3443 2.3221 -36.5949
3.2600 -34.4700 0.1829 -0.0850
0.1061 0.0795 0.0626 0.0171 -0.1035
-0.3455 2.3254 -36.5971
3.2800 -34.5900 -0.1183 -0.0510
0.1291 0.0769 0.0654 0.0205 -0.1048
-0.3467 2.3287 -36.5993
3.3000 -34.4500 -0.0901 0.0710
0.1310 0.0671 0.0673 0.0237 -0.1061
-0.3478 2.3319 -36.6015
3.3200 -34.0500 0.1709 0.2278
0.1029 0.0511 0.0682 0.0268 -0.1075
-0.3490 2.3351 -36.6037
3.3400 -34.3300 0.0601 0.2173
0.0451 0.0308 0.0681 0.0297 -0.1089
-0.3501 2.3383 -36.6059
3.3600 -34.6400 -0.1020 0.0152 -
0.0320 0.0089 0.0671 0.0324 -0.1103
-0.3512 2.3415 -36.6081
3.3800 -34.8500 0.0108 -0.1964 -
0.1059 -0.0120 0.0652 0.0348 -0.1117
-0.3523 2.3447 -36.6103
3.4000 -35.2300 -0.1040 -0.2500 -
0.1514 -0.0294 0.0622 0.0371 -0.1131
-0.3533 2.3478 -36.6125
3.4200 -34.9600 -0.0117 -0.1786 -
0.1531 -0.0412 0.0584 0.0391 -0.1145
-0.3544 2.3509 -36.6147

3.4400 -34.8162 0.0809 -0.0890 -
0.1122 -0.0457 0.0536 0.0408 -0.1159
-0.3554 2.3540 -36.6170
3.4600 -34.7111 0.0424 -0.0065 -
0.0455 -0.0426 0.0482 0.0423 -0.1174
-0.3564 2.3571 -36.6192
3.4800 -34.6300 -0.0854 0.1078
0.0199 -0.0325 0.0421 0.0436 -0.1189
-0.3574 2.3602 -36.6214
3.5000 -34.5700 -0.2365 0.2440
0.0605 -0.0166 0.0354 0.0445 -0.1203
-0.3584 2.3632 -36.6236
3.5200 -33.9000 0.4219 0.2634
0.0666 0.0032 0.0284 0.0452 -0.1218
-0.3593 2.3662 -36.6258
3.5400 -34.7400 -0.1700 0.0478
0.0450 0.0243 0.0211 0.0455 -0.1233
-0.3603 2.3692 -36.6280
3.5600 -34.9300 -0.0806 -0.2059
0.0140 0.0444 0.0136 0.0456 -0.1248
-0.3612 2.3722 -36.6303
3.5800 -35.0800 -0.2097 -0.2355 -
0.0061 0.0614 0.0061 0.0454 -0.1263
-0.3620 2.3751 -36.6325
3.6000 -34.4900 0.2354 -0.0674 -
0.0058 0.0738 -0.0014 0.0448 -0.1278
-0.3629 2.3781 -36.6347
3.6200 -34.5400 -0.1214 0.1048
0.0080 0.0804 -0.0085 0.0440 -0.1293
-0.3637 2.3810 -36.6369
3.6400 -34.3200 0.1583 0.1706
0.0226 0.0805 -0.0153 0.0429 -0.1309
-0.3645 2.3838 -36.6392
3.6600 -34.6500 -0.0634 0.1207
0.0277 0.0739 -0.0216 0.0414 -0.1324
-0.3653 2.3867 -36.6414
3.6800 -34.7300 -0.1006 0.0120
0.0201 0.0613 -0.0272 0.0396 -0.1339
-0.3660 2.3895 -36.6436
3.7000 -34.6000 0.1673 -0.0867
0.0063 0.0434 -0.0321 0.0376 -0.1354
-0.3667 2.3923 -36.6459
3.7200 -34.8800 -0.0106 -0.1485 -
0.0024 0.0217 -0.0359 0.0352 -0.1370
-0.3674 2.3951 -36.6481
3.7400 -35.0100 -0.1955 -0.1275
0.0033 -0.0025 -0.0387 0.0326 -0.1385
-0.3680 2.3978 -36.6503
3.7600 -34.7400 -0.0091 0.0068
0.0244 -0.0277 -0.0404 0.0297 -0.1400
-0.3687 2.4005 -36.6526

3.7800 -34.3900 0.2111 0.1635
0.0467 -0.0528 -0.0407 0.0266 -0.1416
-0.3692 2.4032 -36.6548
3.8000 -34.5800 0.0712 0.1656
0.0518 -0.0761 -0.0397 0.0232 -0.1431
-0.3698 2.4059 -36.6570
3.8200 -34.9000 -0.1628 0.0380
0.0327 -0.0963 -0.0373 0.0195 -0.1446
-0.3703 2.4085 -36.6593
3.8400 -34.7200 0.2467 -0.0736 -
0.0045 -0.1121 -0.0335 0.0157 -0.1461
-0.3708 2.4111 -36.6615
3.8600 -35.2000 -0.2108 -0.1045 -
0.0457 -0.1226 -0.0282 0.0117 -0.1476
-0.3712 2.4137 -36.6638
3.8800 -34.8200 0.3093 -0.0757 -
0.0752 -0.1266 -0.0215 0.0075 -0.1491
-0.3716 2.4163 -36.6660
3.9000 -35.3500 -0.4270 0.0115 -
0.0836 -0.1233 -0.0136 0.0031 -0.1506
-0.3720 2.4188 -36.6683
3.9200 -34.4600 0.4384 0.0873 -
0.0680 -0.1124 -0.0045 -0.0014 -0.1521
-0.3723 2.4213 -36.6705
3.9400 -35.1100 -0.1372 0.0164 -
0.0357 -0.0943 0.0055 -0.0060 -0.1536
-0.3726 2.4237 -36.6728
3.9600 -35.1400 -0.2229 -0.0902 -
0.0010 -0.0698 0.0160 -0.0107 -0.1551
-0.3729 2.4262 -36.6750
3.9800 -34.7700 0.0744 -0.0209
0.0270 -0.0407 0.0268 -0.0154 -0.1565
-0.3731 2.4286 -36.6773
4.0000 -34.5500 -0.0654 0.1473
0.0414 -0.0092 0.0376 -0.0203 -0.1580
-0.3733 2.4309 -36.6795
4.0200 -34.1300 0.4857 0.0898
0.0424 0.0225 0.0479 -0.0251 -0.1594
-0.3734 2.4333 -36.6818
4.0400 -35.3400 -0.5002 -0.1679
0.0362 0.0522 0.0577 -0.0300 -0.1608
-0.3735 2.4356 -36.6840
4.0600 -34.6500 0.3307 -0.2559
0.0316 0.0778 0.0666 -0.0349 -0.1622
-0.3735 2.4378 -36.6863
4.0800 -34.6000 0.1119 -0.0940
0.0296 0.0980 0.0743 -0.0398 -0.1635
-0.3735 2.4401 -36.6885
4.1000 -34.9100 -0.4580 0.1391
0.0272 0.1124 0.0805 -0.0446 -0.1649
-0.3735 2.4423 -36.6908

4.1200 -33.9900 0.4041 0.2410
0.0212 0.1208 0.0852 -0.0493 -0.1662
-0.3734 2.4445 -36.6931
4.1400 -34.3900 0.0456 0.1348
0.0118 0.1233 0.0880 -0.0540 -0.1675
-0.3732 2.4466 -36.6953
4.1600 -35.1400 -0.4643 -0.0404
0.0018 0.1201 0.0890 -0.0586 -0.1688
-0.3730 2.4487 -36.6976
4.1800 -34.3700 0.2532 -0.0789 -
0.0043 0.1117 0.0882 -0.0631 -0.1700
-0.3728 2.4508 -36.6999
4.2000 -34.7100 -0.0583 -0.0492 -
0.0013 0.0987 0.0855 -0.0674 -0.1713
-0.3725 2.4528 -36.7022
4.2200 -34.6800 0.1068 -0.1040
0.0145 0.0818 0.0811 -0.0716 -0.1725
-0.3721 2.4548 -36.7044
4.2400 -34.8100 0.0627 -0.1661
0.0391 0.0626 0.0751 -0.0756 -0.1737
-0.3717 2.4568 -36.7067
4.2600 -35.0100 -0.3537 -0.0488
0.0610 0.0424 0.0674 -0.0794 -0.1748
-0.3713 2.4587 -36.7090
4.2800 -34.1800 0.4143 0.1915
0.0687 0.0230 0.0584 -0.0830 -0.1759
-0.3708 2.4606 -36.7112
4.3000 -34.6600 -0.1459 0.2804
0.0541 0.0058 0.0480 -0.0864 -0.1770
-0.3702 2.4624 -36.7135
4.3200 -34.7100 -0.0128 0.1401
0.0178 -0.0078 0.0364 -0.0895 -0.1781
-0.3696 2.4642 -36.7158
4.3400 -34.7400 0.2346 -0.0980 -
0.0272 -0.0168 0.0238 -0.0924 -0.1791
-0.3689 2.4660 -36.7181
4.3600 -35.4600 -0.2800 -0.2797 -
0.0643 -0.0210 0.0103 -0.0950 -0.1801
-0.3682 2.4678 -36.7204
4.3800 -35.1400 0.1212 -0.2540 -
0.0823 -0.0209 -0.0037 -0.0974 -0.1810
-0.3674 2.4695 -36.7227
4.4000 -35.2800 -0.1530 -0.0356 -
0.0824 -0.0169 -0.0180 -0.0994 -0.1819
-0.3666 2.4711 -36.7249
4.4200 -34.5400 0.3445 0.1747 -
0.0713 -0.0101 -0.0323 -0.1011 -0.1828
-0.3657 2.4727 -36.7272
4.4400 -35.1200 -0.2968 0.1903 -
0.0551 -0.0016 -0.0462 -0.1025 -0.1837
-0.3648 2.4743 -36.7295

4.4600 -34.6400 0.3745 0.0295 -
0.0350 0.0071 -0.0595 -0.1035 -0.1845
-0.3638 2.4759 -36.7318
4.4800 -35.3900 -0.2592 -0.1465 -
0.0072 0.0142 -0.0719 -0.1042 -0.1852
-0.3627 2.4774 -36.7341
4.5000 -35.2287 -0.1517 -0.1721
0.0319 0.0183 -0.0831 -0.1045 -0.1860
-0.3616 2.4789 -36.7364
4.5200 -34.9200 0.0850 -0.0574
0.0760 0.0178 -0.0930 -0.1044 -0.1867
-0.3604 2.4803 -36.7387
4.5400 -34.5500 0.3167 0.0393
0.1132 0.0113 -0.1012 -0.1039 -0.1873
-0.3591 2.4817 -36.7410
4.5600 -35.1800 -0.3892 0.0864
0.1289 -0.0013 -0.1077 -0.1030 -0.1879
-0.3578 2.4830 -36.7433
4.5800 -34.6900 0.1250 0.1479
0.1102 -0.0196 -0.1123 -0.1017 -0.1885
-0.3564 2.4843 -36.7456
4.6000 -34.5000 0.3123 0.1806
0.0582 -0.0424 -0.1150 -0.1000 -0.1890
-0.3550 2.4856 -36.7479
4.6200 -35.4800 -0.4339 0.0714 -
0.0086 -0.0678 -0.1157 -0.0979 -0.1895
-0.3535 2.4868 -36.7502
4.6400 -34.7400 0.5142 -0.1872 -
0.0644 -0.0940 -0.1142 -0.0954 -0.1900
-0.3520 2.4880 -36.7525
4.6600 -35.8700 -0.4297 -0.3329 -
0.0931 -0.1185 -0.1106 -0.0926 -0.1904
-0.3503 2.4891 -36.7548
4.6800 -35.5200 -0.1659 -0.1525 -
0.0929 -0.1388 -0.1049 -0.0894 -0.1907
-0.3486 2.4902 -36.7571
4.7000 -34.6500 0.3150 0.1890 -
0.0779 -0.1522 -0.0970 -0.0859 -0.1911
-0.3469 2.4913 -36.7594
4.7200 -34.7700 0.1057 0.3601 -
0.0653 -0.1568 -0.0870 -0.0821 -0.1914
-0.3451 2.4923 -36.7617
4.7400 -34.9200 -0.0554 0.2339 -
0.0658 -0.1516 -0.0752 -0.0780 -0.1916
-0.3432 2.4932 -36.7641
4.7600 -35.3000 -0.1294 -0.0226 -
0.0746 -0.1366 -0.0616 -0.0737 -0.1918
-0.3412 2.4942 -36.7664
4.7800 -35.3100 0.0212 -0.2082 -
0.0805 -0.1123 -0.0467 -0.0691 -0.1920
-0.3392 2.4950 -36.7687

4.8000 -35.2163 0.0449 -0.2409 -
0.0700 -0.0803 -0.0307 -0.0643 -0.1922
-0.3371 2.4959 -36.7710
4.8200 -35.0292 0.0842 -0.1633 -
0.0347 -0.0426 -0.0141 -0.0593 -0.1923
-0.3350 2.4967 -36.7733
4.8400 -34.8900 -0.0444 -0.0272
0.0219 -0.0020 0.0027 -0.0541 -0.1923
-0.3328 2.4974 -36.7756
4.8600 -34.8400 -0.0684 0.0998
0.0867 0.0383 0.0194 -0.0489 -0.1924
-0.3305 2.4981 -36.7780
4.8800 -34.1700 0.3108 0.1113
0.1438 0.0748 0.0355 -0.0435 -0.1924
-0.3281 2.4988 -36.7803
4.9000 -34.7300 -0.3896 0.0070
0.1744 0.1048 0.0506 -0.0380 -0.1924
-0.3257 2.4994 -36.7826
4.9200 -34.2000 0.3173 -0.0353
0.1669 0.1265 0.0644 -0.0325 -0.1923
-0.3232 2.5000 -36.7849
4.9400 -34.8800 -0.3626 0.0310
0.1244 0.1398 0.0765 -0.0270 -0.1922
-0.3207 2.5005 -36.7873
4.9600 -34.1500 0.3451 0.0505
0.0631 0.1453 0.0867 -0.0215 -0.1921
-0.3180 2.5010 -36.7896
4.9800 -34.9000 -0.2342 -0.0250 -
0.0010 0.1441 0.0947 -0.0160 -0.1920
-0.3153 2.5014 -36.7919
5.0000 -34.7000 0.0503 -0.0704 -
0.0560 0.1381 0.1004 -0.0106 -0.1918
-0.3126 2.5018 -36.7943
5.0200 -34.7400 -0.0882 -0.0139 -
0.1008 0.1298 0.1036 -0.0052 -0.1916
-0.3097 2.5021 -36.7966
5.0400 -34.7800 -0.1227 0.1010 -
0.1367 0.1215 0.1042 0.0000 -0.1914
-0.3068 2.5024 -36.7989
5.0600 -34.3800 0.3163 0.0991 -
0.1600 0.1142 0.1024 0.0051 -0.1911
-0.3038 2.5027 -36.8013
5.0800 -34.7800 -0.0255 -0.0596 -
0.1602 0.1081 0.0982 0.0101 -0.1909
-0.3008 2.5029 -36.8036
5.1000 -35.0800 -0.2805 -0.1708 -
0.1273 0.1025 0.0919 0.0149 -0.1906
-0.2977 2.5030 -36.8060
5.1200 -34.7300 0.0518 -0.1115 -
0.0613 0.0965 0.0839 0.0195 -0.1902
-0.2945 2.5032 -36.8083

5.1400	-34.2000	0.3097	0.0131	
0.0254	0.0884	0.0744	0.0238	-0.1899
	-0.2912	2.5032	-36.8106	
5.1600	-34.8200	-0.4064	0.1066	
0.1091	0.0766	0.0639	0.0280	-0.1895
	-0.2879	2.5032	-36.8130	
5.1800	-34.0700	0.3370	0.1186	
0.1668	0.0595	0.0526	0.0319	-0.1892
	-0.2845	2.5032	-36.8153	
5.2000	-34.5700	-0.1070	0.0383	
0.1857	0.0363	0.0410	0.0355	-0.1888
	-0.2810	2.5031	-36.8177	
5.2200	-34.3900	0.1132	-0.0534	
0.1659	0.0070	0.0293	0.0388	-0.1883
	-0.2774	2.5030	-36.8200	
5.2400	-35.0500	-0.3625	-0.0655	
0.1191	-0.0271	0.0179	0.0418	-0.1879
	-0.2738	2.5028	-36.8224	
5.2600	-34.4100	0.3602	-0.0309	
0.0603	-0.0641	0.0070	0.0445	-0.1874
	-0.2701	2.5026	-36.8247	
5.2800	-34.8000	0.1058	-0.0468	
0.0020	-0.1015	-0.0032	0.0469	-0.1870
	-0.2664	2.5024	-36.8271	
5.3000	-35.1900	-0.2534	-0.0556	-
0.0504	-0.1360	-0.0124	0.0490	-0.1865
	-0.2625	2.5020	-36.8294	
5.3200	-34.8000	0.1711	0.0357	-
0.0955	-0.1644	-0.0204	0.0507	-0.1860
	-0.2586	2.5017	-36.8318	
5.3400	-35.1600	-0.2796	0.1666	-
0.1326	-0.1838	-0.0270	0.0521	-0.1855
	-0.2546	2.5013	-36.8342	
5.3600	-34.6100	0.4673	0.1172	-
0.1562	-0.1923	-0.0320	0.0531	-0.1850
	-0.2506	2.5008	-36.8365	
5.3800	-35.3900	-0.2540	-0.0947	-
0.1577	-0.1889	-0.0353	0.0538	-0.1845
	-0.2465	2.5003	-36.8389	
5.4000	-35.4800	-0.2885	-0.1865	-
0.1287	-0.1745	-0.0368	0.0542	-0.1840
	-0.2423	2.4998	-36.8412	
5.4200	-35.0500	0.0015	-0.0756	-
0.0718	-0.1502	-0.0368	0.0541	-0.1834
	-0.2381	2.4992	-36.8436	
5.4400	-34.5100	0.3925	0.0306	-
0.0025	-0.1177	-0.0352	0.0538	-0.1829
	-0.2337	2.4985	-36.8460	
5.4600	-34.9900	-0.2741	0.0247	
0.0583	-0.0793	-0.0323	0.0531	-0.1824
	-0.2293	2.4978	-36.8483	

5.4800 -34.6700 0.0032 -0.0065
0.0950 -0.0375 -0.0282 0.0521 -0.1818
-0.2249 2.4971 -36.8507
5.5000 -34.4100 0.1198 0.0161
0.1027 0.0046 -0.0233 0.0507 -0.1813
-0.2204 2.4963 -36.8531
5.5200 -34.5800 -0.0172 0.0363
0.0902 0.0436 -0.0176 0.0490 -0.1808
-0.2158 2.4955 -36.8554
5.5400 -34.5300 -0.0203 0.0202
0.0683 0.0769 -0.0114 0.0470 -0.1802
-0.2111 2.4946 -36.8578
5.5600 -34.5900 0.0127 0.0102
0.0470 0.1024 -0.0048 0.0447 -0.1797
-0.2064 2.4937 -36.8602
5.5800 -34.5700 -0.0345 0.0192
0.0341 0.1189 0.0019 0.0421 -0.1792
-0.2016 2.4927 -36.8626
5.6000 -34.5300 0.0667 0.0027
0.0290 0.1259 0.0087 0.0392 -0.1787
-0.1968 2.4916 -36.8649
5.6200 -34.6500 0.0390 -0.0448
0.0293 0.1236 0.0153 0.0361 -0.1781
-0.1918 2.4906 -36.8673
5.6400 -34.7500 -0.0582 -0.0529
0.0291 0.1130 0.0216 0.0327 -0.1776
-0.1869 2.4894 -36.8697
5.6600 -34.6900 -0.1198 0.0124
0.0233 0.0959 0.0274 0.0290 -0.1771
-0.1818 2.4883 -36.8721
5.6800 -34.3300 0.3337 0.0785
0.0099 0.0746 0.0328 0.0251 -0.1766
-0.1767 2.4871 -36.8745
5.7000 -34.9400 -0.3630 0.0675 -
0.0095 0.0513 0.0374 0.0210 -0.1761
-0.1715 2.4858 -36.8768
5.7200 -34.3300 0.3414 -0.0266 -
0.0307 0.0281 0.0412 0.0167 -0.1756
-0.1663 2.4845 -36.8792
5.7400 -35.2000 -0.3967 -0.0833 -
0.0504 0.0074 0.0442 0.0122 -0.1752
-0.1610 2.4831 -36.8816
5.7600 -34.6400 0.1748 -0.0001 -
0.0671 -0.0091 0.0461 0.0076 -0.1747
-0.1556 2.4817 -36.8840
5.7800 -34.6200 0.1001 0.0869 -
0.0772 -0.0202 0.0471 0.0028 -0.1742
-0.1502 2.4802 -36.8864
5.8000 -34.7300 0.0339 0.0424 -
0.0779 -0.0255 0.0471 -0.0021 -0.1738
-0.1447 2.4787 -36.8888

5.8200 -34.6600 0.1381 -0.0815 -
0.0652 -0.0257 0.0462 -0.0071 -0.1733
-0.1392 2.4772 -36.8912
5.8400 -35.3200 -0.4175 -0.0996 -
0.0385 -0.0214 0.0445 -0.0122 -0.1729
-0.1336 2.4756 -36.8936
5.8600 -34.2700 0.3439 0.0430 -
0.0012 -0.0143 0.0420 -0.0173 -0.1725
-0.1280 2.4739 -36.8959
5.8800 -34.7100 -0.1401 0.1145
0.0391 -0.0061 0.0389 -0.0225 -0.1721
-0.1222 2.4722 -36.8983
5.9000 -34.4100 0.1231 -0.0003
0.0756 0.0017 0.0351 -0.0277 -0.1717
-0.1165 2.4705 -36.9007
5.9200 -35.0700 -0.3088 -0.1215
0.1031 0.0074 0.0308 -0.0328 -0.1714
-0.1107 2.4687 -36.9031
5.9400 -34.5500 0.1466 -0.0899
0.1174 0.0099 0.0261 -0.0380 -0.1710
-0.1048 2.4669 -36.9055
5.9600 -34.4100 0.1749 0.0228
0.1146 0.0087 0.0210 -0.0430 -0.1707
-0.0989 2.4650 -36.9079
5.9800 -34.8400 -0.2423 0.1095
0.0913 0.0041 0.0156 -0.0480 -0.1704
-0.0929 2.4630 -36.9103
6.0000 -34.5700 0.0395 0.1544
0.0478 -0.0026 0.0099 -0.0529 -0.1701
-0.0868 2.4611 -36.9127
6.0200 -34.3100 0.3257 0.1490 -
0.0096 -0.0101 0.0039 -0.0577 -0.1698
-0.0807 2.4590 -36.9151
6.0400 -35.2500 -0.4600 0.0652 -
0.0672 -0.0169 -0.0024 -0.0623 -0.1695
-0.0746 2.4570 -36.9175
6.0600 -34.7400 0.1430 -0.0643 -
0.1080 -0.0222 -0.0090 -0.0667 -0.1693
-0.0684 2.4548 -36.9200
6.0800 -34.6300 0.4358 -0.2050 -
0.1188 -0.0248 -0.0157 -0.0710 -0.1691
-0.0622 2.4527 -36.9224
6.1000 -35.5200 -0.3848 -0.2975 -
0.0921 -0.0243 -0.0225 -0.0750 -0.1689
-0.0559 2.4504 -36.9248
6.1200 -35.1500 -0.0497 -0.1864 -
0.0339 -0.0207 -0.0294 -0.0788 -0.1687
-0.0496 2.4482 -36.9272
6.1400 -34.5200 0.2185 0.0731
0.0358 -0.0146 -0.0363 -0.0824 -0.1685
-0.0432 2.4459 -36.9296

6.1600	-34.4500	0.0467	0.2508	
0.0916	-0.0067	-0.0430	-0.0858	-0.1684
	-0.0368	2.4435	-36.9320	
6.1800	-34.4600	-0.0313	0.2661	
0.1117	0.0023	-0.0495	-0.0888	-0.1682
	-0.0303	2.4411	-36.9344	
6.2000	-34.6000	-0.0370	0.1703	
0.0883	0.0118	-0.0557	-0.0916	-0.1681
	-0.0238	2.4386	-36.9368	
6.2200	-34.8100	0.0887	0.0110	
0.0313	0.0215	-0.0614	-0.0941	-0.1680
	-0.0172	2.4361	-36.9393	
6.2400	-35.0500	-0.1291	-0.1399	-
0.0364	0.0310	-0.0665	-0.0963	-0.1680
	-0.0106	2.4336	-36.9417	
6.2600	-34.9800	0.1607	-0.2363	-
0.0882	0.0397	-0.0709	-0.0982	-0.1679
	-0.0040	2.4310	-36.9441	
6.2800	-35.1800	0.0127	-0.2500	-
0.1042	0.0469	-0.0742	-0.0999	-0.1679
	0.0027	2.4283	-36.9465	
6.3000	-35.2800	-0.3015	-0.1061	-
0.0829	0.0518	-0.0764	-0.1011	-0.1679
	0.0094	2.4257	-36.9489	
6.3200	-34.3700	0.4066	0.1264	-
0.0379	0.0538	-0.0774	-0.1021	-0.1679
	0.0162	2.4229	-36.9514	
6.3400	-34.6800	-0.1420	0.1923	
0.0095	0.0523	-0.0769	-0.1028	-0.1679
	0.0229	2.4201	-36.9538	
6.3600	-34.5100	0.0776	0.0817	
0.0432	0.0472	-0.0750	-0.1031	-0.1680
	0.0298	2.4173	-36.9562	
6.3800	-35.0600	-0.2982	-0.0122	
0.0577	0.0389	-0.0715	-0.1032	-0.1681
	0.0366	2.4144	-36.9586	
6.4000	-34.4400	0.3572	-0.0462	
0.0569	0.0279	-0.0665	-0.1029	-0.1681
	0.0435	2.4115	-36.9611	
6.4200	-34.7700	0.0096	-0.0661	
0.0479	0.0146	-0.0599	-0.1023	-0.1682
	0.0504	2.4085	-36.9635	
6.4400	-35.1000	-0.1709	-0.0737	
0.0369	-0.0012	-0.0518	-0.1014	-0.1684
	0.0574	2.4055	-36.9659	
6.4600	-34.9800	-0.1099	-0.0098	
0.0268	-0.0195	-0.0423	-0.1003	-0.1685
	0.0643	2.4024	-36.9684	
6.4800	-34.5800	0.1402	0.0854	
0.0199	-0.0400	-0.0314	-0.0988	-0.1687
	0.0713	2.3993	-36.9708	

6.5000 -34.7900 -0.0315 0.0994
0.0187 -0.0619 -0.0193 -0.0971 -0.1688
0.0784 2.3962 -36.9732
6.5200 -35.0000 -0.1633 0.0434
0.0227 -0.0843 -0.0062 -0.0951 -0.1690
0.0854 2.3930 -36.9757
6.5400 -34.5000 0.3191 -0.0359
0.0282 -0.1058 0.0076 -0.0928 -0.1692
0.0925 2.3897 -36.9781
6.5600 -35.0600 -0.1467 -0.0889
0.0313 -0.1249 0.0221 -0.0903 -0.1695
0.0996 2.3864 -36.9805
6.5800 -35.2600 -0.2413 -0.0393
0.0277 -0.1399 0.0370 -0.0875 -0.1697
0.1067 2.3831 -36.9830
6.6000 -34.4100 0.3789 0.0264
0.0114 -0.1490 0.0521 -0.0845 -0.1699
0.1138 2.3797 -36.9854
6.6200 -34.9500 -0.0857 0.0022 -
0.0203 -0.1510 0.0671 -0.0812 -0.1702
0.1209 2.3762 -36.9879
6.6400 -35.1700 -0.3255 -0.0068 -
0.0642 -0.1450 0.0819 -0.0778 -0.1705
0.1281 2.3728 -36.9903
6.6600 -34.7600 0.0619 0.0950 -
0.1104 -0.1307 0.0961 -0.0741 -0.1707
0.1353 2.3692 -36.9927
6.6800 -34.3700 0.3508 0.1564 -
0.1466 -0.1082 0.1095 -0.0702 -0.1710
0.1424 2.3657 -36.9952
6.7000 -35.0300 -0.2782 0.0182 -
0.1608 -0.0776 0.1216 -0.0662 -0.1713
0.1496 2.3620 -36.9976
6.7200 -34.6400 0.1865 -0.1891 -
0.1422 -0.0402 0.1323 -0.0620 -0.1716
0.1568 2.3584 -37.0001
6.7400 -35.0200 -0.1554 -0.2359 -
0.0868 0.0023 0.1413 -0.0576 -0.1719
0.1640 2.3547 -37.0025
6.7600 -34.7000 -0.0600 -0.0865 -
0.0027 0.0471 0.1483 -0.0532 -0.1722
0.1713 2.3509 -37.0050
6.7800 -33.9900 0.2933 0.0886
0.0886 0.0914 0.1530 -0.0485 -0.1725
0.1785 2.3471 -37.0074
6.8000 -34.2000 -0.1766 0.1372
0.1617 0.1321 0.1554 -0.0438 -0.1728
0.1857 2.3432 -37.0099
6.8200 -34.2600 -0.1258 0.1283
0.1978 0.1665 0.1552 -0.0390 -0.1731
0.1929 2.3393 -37.0123

6.8400 -33.9500 0.0735 0.1412
0.1908 0.1922 0.1523 -0.0341 -0.1734
0.2001 2.3354 -37.0148
6.8600 -33.7300 0.3297 0.0854
0.1496 0.2073 0.1465 -0.0291 -0.1737
0.2073 2.3314 -37.0173
6.8800 -34.7900 -0.5705 -0.0291
0.0909 0.2112 0.1380 -0.0241 -0.1739
0.2145 2.3274 -37.0197
6.9000 -34.3400 0.0210 -0.0756
0.0334 0.2038 0.1268 -0.0191 -0.1742
0.2217 2.3233 -37.0222
6.9200 -34.0600 0.3446 -0.0661 -
0.0125 0.1858 0.1130 -0.0141 -0.1745
0.2289 2.3192 -37.0246
6.9400 -34.3700 0.1700 -0.1095 -
0.0446 0.1584 0.0969 -0.0091 -0.1747
0.2361 2.3150 -37.0271
6.9600 -35.2300 -0.6226 -0.1238 -
0.0659 0.1232 0.0789 -0.0041 -0.1749
0.2432 2.3108 -37.0295
6.9800 -34.0600 0.5988 0.0346 -
0.0816 0.0824 0.0593 0.0007 -0.1751
0.2504 2.3065 -37.0320
7.0000 -35.0000 -0.5223 0.2108 -
0.0938 0.0382 0.0385 0.0055 -0.1753
0.2575 2.3022 -37.0345
7.0200 -34.2200 0.3108 0.1775 -
0.0998 -0.0072 0.0170 0.0102 -0.1755
0.2646 2.2979 -37.0369
7.0400 -34.8000 0.1808 -0.0320 -
0.0937 -0.0513 -0.0049 0.0147 -0.1756
0.2717 2.2935 -37.0394
7.0600 -35.3100 -0.3481 -0.2193 -
0.0686 -0.0921 -0.0268 0.0191 -0.1758
0.2788 2.2890 -37.0419
7.0800 -34.9100 0.1234 -0.1688 -
0.0234 -0.1275 -0.0481 0.0232 -0.1759
0.2858 2.2845 -37.0443
7.1000 -34.7280 0.0161 0.0332
0.0309 -0.1553 -0.0685 0.0271 -0.1759
0.2928 2.2800 -37.0468
7.1200 -34.6500 0.0279 0.1192
0.0779 -0.1741 -0.0876 0.0308 -0.1760
0.2998 2.2754 -37.0493
7.1400 -34.6600 0.0653 0.0653
0.1017 -0.1828 -0.1049 0.0342 -0.1760
0.3068 2.2708 -37.0517
7.1600 -35.3300 -0.4557 0.0297
0.0895 -0.1812 -0.1201 0.0374 -0.1759
0.3137 2.2661 -37.0542

7.1800 -34.3800 0.3928 0.0950
0.0407 -0.1698 -0.1330 0.0402 -0.1759
0.3206 2.2614 -37.0567
7.2000 -35.0200 -0.2442 0.0992 -
0.0282 -0.1498 -0.1432 0.0427 -0.1758
0.3275 2.2567 -37.0592
7.2200 -34.7400 0.2470 -0.0332 -
0.0933 -0.1228 -0.1506 0.0448 -0.1756
0.3343 2.2519 -37.0616
7.2400 -35.4900 -0.4036 -0.1338 -
0.1306 -0.0909 -0.1552 0.0466 -0.1755
0.3411 2.2470 -37.0641
7.2600 -34.6100 0.3612 -0.0926 -
0.1276 -0.0565 -0.1568 0.0480 -0.1752
0.3478 2.2421 -37.0666
7.2800 -35.0600 -0.2026 0.0047 -
0.0872 -0.0224 -0.1555 0.0490 -0.1750
0.3545 2.2372 -37.0691
7.3000 -34.6000 0.0584 0.0726 -
0.0215 0.0087 -0.1511 0.0497 -0.1747
0.3612 2.2322 -37.0716
7.3200 -34.6800 -0.1143 0.0828
0.0525 0.0343 -0.1438 0.0500 -0.1743
0.3678 2.2272 -37.0740
7.3400 -34.4200 0.0561 0.0471
0.1170 0.0525 -0.1337 0.0500 -0.1739
0.3743 2.2221 -37.0765
7.3600 -34.3800 0.1225 -0.0275
0.1560 0.0619 -0.1207 0.0496 -0.1734
0.3808 2.2170 -37.0790
7.3800 -34.8100 -0.1909 -0.0870
0.1618 0.0624 -0.1051 0.0489 -0.1729
0.3873 2.2119 -37.0815
7.4000 -34.5100 0.0280 -0.0673
0.1384 0.0547 -0.0871 0.0478 -0.1724
0.3937 2.2067 -37.0840
7.4200 -34.4300 0.0235 0.0521
0.0927 0.0407 -0.0669 0.0463 -0.1718
0.4000 2.2014 -37.0865
7.4400 -34.3500 0.0396 0.1670
0.0330 0.0226 -0.0448 0.0445 -0.1711
0.4063 2.1961 -37.0889
7.4600 -34.5400 -0.0296 0.1539 -
0.0324 0.0027 -0.0211 0.0424 -0.1703
0.4125 2.1908 -37.0914
7.4800 -34.6100 0.0360 0.0003 -
0.0914 -0.0168 0.0039 0.0399 -0.1695
0.4187 2.1854 -37.0939
7.5000 -34.9700 0.0734 -0.1760 -
0.1304 -0.0340 0.0297 0.0371 -0.1686
0.4248 2.1800 -37.0964

7.5200 -35.4000 -0.2628 -0.2102 -
0.1384 -0.0471 0.0560 0.0339 -0.1677
0.4308 2.1746 -37.0989
7.5400 -34.7800 0.1211 -0.1081 -
0.1136 -0.0545 0.0825 0.0303 -0.1666
0.4368 2.1691 -37.1014
7.5600 -34.5500 0.0379 0.0238 -
0.0716 -0.0554 0.1086 0.0265 -0.1655
0.4427 2.1635 -37.1039
7.5800 -34.3800 0.1127 0.1203 -
0.0292 -0.0502 0.1340 0.0222 -0.1644
0.4485 2.1579 -37.1064
7.6000 -34.6700 -0.2993 0.1396 -
0.0017 -0.0394 0.1583 0.0177 -0.1631
0.4543 2.1523 -37.1089
7.6200 -34.0000 0.4740 0.0419
0.0074 -0.0239 0.1810 0.0128 -0.1617
0.4600 2.1466 -37.1114
7.6400 -34.8700 -0.2946 -0.1023
0.0074 -0.0045 0.2019 0.0076 -0.1603
0.4656 2.1409 -37.1139
7.6600 -34.8300 -0.2651 -0.1314
0.0082 0.0178 0.2203 0.0020 -0.1587
0.4711 2.1352 -37.1164
7.6800 -34.0300 0.3342 -0.0245
0.0102 0.0415 0.2361 -0.0038 -0.1570
0.4765 2.1294 -37.1189
7.7000 -34.1100 0.1268 0.0654
0.0117 0.0652 0.2488 -0.0100 -0.1553
0.4819 2.1235 -37.1214
7.7200 -34.6600 -0.4427 0.1079
0.0102 0.0878 0.2580 -0.0164 -0.1534
0.4871 2.1176 -37.1239
7.7400 -33.8700 0.4002 0.1006
0.0060 0.1084 0.2634 -0.0231 -0.1514
0.4923 2.1117 -37.1264
7.7600 -34.5400 -0.1584 -0.0496
0.0088 0.1271 0.2648 -0.0300 -0.1493
0.4974 2.1057 -37.1289
7.7800 -34.2800 0.2150 -0.2181
0.0287 0.1441 0.2618 -0.0372 -0.1471
0.5025 2.0997 -37.1314
7.8000 -34.8500 -0.5223 -0.0786
0.0646 0.1596 0.2544 -0.0446 -0.1447
0.5074 2.0937 -37.1339
7.8200 -33.4000 0.6150 0.1936
0.0944 0.1734 0.2424 -0.0521 -0.1422
0.5122 2.0876 -37.1364
7.8400 -34.4900 -0.4367 0.1664
0.0962 0.1855 0.2259 -0.0598 -0.1396
0.5170 2.0815 -37.1389

7.8600 -34.2100 0.1035 0.0113
0.0665 0.1954 0.2051 -0.0675 -0.1368
0.5216 2.0753 -37.1414
7.8800 -34.5800 -0.1939 -0.0444
0.0194 0.2019 0.1803 -0.0753 -0.1339
0.5262 2.0691 -37.1439
7.9000 -34.3100 0.3502 -0.1255 -
0.0215 0.2038 0.1518 -0.0831 -0.1308
0.5306 2.0628 -37.1464
7.9200 -35.0600 -0.4298 -0.1812 -
0.0374 0.2000 0.1202 -0.0909 -0.1276
0.5350 2.0565 -37.1489
7.9400 -34.3500 0.2187 -0.0461 -
0.0219 0.1893 0.0859 -0.0985 -0.1242
0.5392 2.0502 -37.1514
7.9600 -34.4800 0.0068 0.1417
0.0149 0.1703 0.0497 -0.1061 -0.1207
0.5434 2.0438 -37.1540
7.9800 -34.2900 0.0319 0.2230
0.0553 0.1418 0.0121 -0.1134 -0.1170
0.5474 2.0373 -37.1565
8.0000 -34.5200 -0.0548 0.1324
0.0886 0.1037 -0.0261 -0.1205 -0.1131
0.5514 2.0309 -37.1590
8.0200 -34.5300 0.2547 -0.0872
0.1131 0.0565 -0.0643 -0.1273 -0.1091
0.5552 2.0244 -37.1615
8.0400 -35.1700 -0.1238 -0.2449
0.1318 0.0016 -0.1018 -0.1337 -0.1049
0.5590 2.0178 -37.1640
8.0600 -35.3700 -0.2986 -0.2020
0.1465 -0.0590 -0.1379 -0.1397 -0.1005
0.5626 2.0112 -37.1665
8.0800 -34.6000 0.4258 -0.0100
0.1514 -0.1223 -0.1719 -0.1453 -0.0959
0.5661 2.0046 -37.1691
8.1000 -34.9700 0.0383 0.1257
0.1314 -0.1845 -0.2031 -0.1503 -0.0912
0.5695 1.9980 -37.1716
8.1200 -35.1700 -0.0918 0.1844
0.0710 -0.2411 -0.2309 -0.1549 -0.0862
0.5728 1.9913 -37.1741
8.1400 -35.0800 0.1123 0.2407 -
0.0362 -0.2878 -0.2547 -0.1588 -0.0811
0.5760 1.9845 -37.1766
8.1600 -35.5800 -0.2001 0.2668 -
0.1711 -0.3213 -0.2739 -0.1620 -0.0758
0.5791 1.9777 -37.1791
8.1800 -35.4700 0.2164 0.1175 -
0.2982 -0.3386 -0.2880 -0.1645 -0.0703
0.5821 1.9709 -37.1817

8.2000 -36.0800 0.0567 -0.2257 -
0.3812 -0.3371 -0.2965 -0.1663 -0.0646
0.5849 1.9641 -37.1842
8.2200 -36.4100 -0.0288 -0.4966 -
0.3876 -0.3152 -0.2995 -0.1673 -0.0587
0.5876 1.9572 -37.1867
8.2400 -36.5600 -0.3848 -0.4194 -
0.2998 -0.2741 -0.2972 -0.1675 -0.0526
0.5903 1.9502 -37.1892
8.2600 -35.2000 0.2383 -0.0192 -
0.1394 -0.2181 -0.2900 -0.1669 -0.0463
0.5928 1.9433 -37.1918
8.2800 -34.8800 0.0473 0.3577
0.0449 -0.1521 -0.2783 -0.1655 -0.0398
0.5952 1.9362 -37.1943
8.3000 -34.3300 0.2619 0.4375
0.2033 -0.0814 -0.2626 -0.1634 -0.0331
0.5975 1.9292 -37.1968
8.3200 -34.8200 -0.3101 0.2736
0.3008 -0.0108 -0.2435 -0.1606 -0.0262
0.5996 1.9221 -37.1993
8.3400 -34.5800 0.0806 0.0615
0.3255 0.0549 -0.2213 -0.1571 -0.0192
0.6017 1.9150 -37.2019
8.3600 -34.5200 0.2804 -0.1093
0.2897 0.1124 -0.1967 -0.1530 -0.0119
0.6036 1.9078 -37.2044
8.3800 -35.0500 -0.1511 -0.2448
0.2171 0.1594 -0.1700 -0.1482 -0.0045
0.6055 1.9006 -37.2069
8.4000 -35.1200 -0.1743 -0.2580
0.1278 0.1941 -0.1418 -0.1429 0.0032
0.6072 1.8934 -37.2095
8.4200 -34.7700 0.0551 -0.1161
0.0391 0.2157 -0.1125 -0.1370 0.0110
0.6088 1.8861 -37.2120
8.4400 -34.5000 0.1355 0.0486 -
0.0381 0.2241 -0.0826 -0.1307 0.0190
0.6103 1.8788 -37.2145
8.4600 -34.6700 -0.0269 0.1168 -
0.0960 0.2200 -0.0525 -0.1239 0.0271
0.6116 1.8715 -37.2171
8.4800 -34.7300 -0.1072 0.1101 -
0.1300 0.2053 -0.0226 -0.1166 0.0355
0.6129 1.8641 -37.2196
8.5000 -34.5900 0.1045 0.0402 -
0.1396 0.1822 0.0066 -0.1090 0.0439
0.6140 1.8567 -37.2221
8.5200 -34.9000 -0.0214 -0.0828 -
0.1268 0.1533 0.0349 -0.1010 0.0526
0.6150 1.8492 -37.2247

8.5400 -34.8800 -0.0784 -0.1428 -
0.0935 0.1214 0.0617 -0.0927 0.0614
0.6159 1.8417 -37.2272
8.5600 -34.9100 -0.1800 -0.0564 -
0.0455 0.0887 0.0869 -0.0841 0.0703
0.6167 1.8342 -37.2298
8.5800 -34.3400 0.2682 0.0934
0.0042 0.0574 0.1100 -0.0753 0.0794
0.6174 1.8267 -37.2323
8.6000 -34.5000 -0.0393 0.1568
0.0403 0.0292 0.1309 -0.0662 0.0886
0.6180 1.8191 -37.2348
8.6200 -34.4700 -0.0200 0.0948
0.0522 0.0053 0.1493 -0.0571 0.0979
0.6185 1.8114 -37.2374
8.6400 -34.6600 -0.0365 -0.0208
0.0422 -0.0138 0.1654 -0.0478 0.1074
0.6188 1.8038 -37.2399
8.6600 -34.6700 0.0974 -0.1099
0.0207 -0.0281 0.1789 -0.0384 0.1169
0.6190 1.7961 -37.2425
8.6800 -34.8700 -0.1576 -0.1446
0.0019 -0.0381 0.1901 -0.0290 0.1266
0.6191 1.7884 -37.2450
8.7000 -34.4100 0.2491 -0.1211 -
0.0072 -0.0441 0.1988 -0.0196 0.1363
0.6192 1.7806 -37.2476
8.7200 -34.7800 -0.0836 -0.0302 -
0.0049 -0.0463 0.2053 -0.0102 0.1462
0.6190 1.7728 -37.2501
8.7400 -34.7300 -0.2761 0.0902
0.0011 -0.0448 0.2095 -0.0009 0.1561
0.6188 1.7650 -37.2527
8.7600 -33.9800 0.4643 0.1440 -
0.0013 -0.0403 0.2117 0.0083 0.1661
0.6185 1.7571 -37.2552
8.7800 -34.4400 -0.0288 0.0787 -
0.0202 -0.0334 0.2118 0.0173 0.1762
0.6181 1.7492 -37.2577
8.8000 -34.9300 -0.4916 0.0446 -
0.0510 -0.0248 0.2100 0.0262 0.1863
0.6175 1.7413 -37.2603
8.8200 -34.3200 0.2289 0.0858 -
0.0792 -0.0148 0.2065 0.0350 0.1965
0.6168 1.7334 -37.2628
8.8400 -34.1600 0.4037 0.0100 -
0.0849 -0.0040 0.2013 0.0435 0.2067
0.6161 1.7254 -37.2654
8.8600 -35.0600 -0.3392 -0.2056 -
0.0528 0.0071 0.1945 0.0518 0.2170
0.6152 1.7174 -37.2679

8.8800 -34.6500 -0.0100 -0.2996
0.0122 0.0177 0.1862 0.0599 0.2273
0.6142 1.7093 -37.2705
8.9000 -34.7400 -0.4155 -0.0536
0.0906 0.0266 0.1764 0.0678 0.2376
0.6131 1.7012 -37.2731
8.9200 -33.6000 0.3982 0.2963
0.1496 0.0328 0.1653 0.0754 0.2480
0.6118 1.6931 -37.2756
8.9400 -33.7100 0.2005 0.3480
0.1579 0.0355 0.1530 0.0827 0.2584
0.6105 1.6850 -37.2782
8.9600 -34.5600 -0.2886 0.1000
0.1035 0.0345 0.1395 0.0899 0.2687
0.6091 1.6768 -37.2807
8.9800 -34.5400 0.1358 -0.1251
0.0068 0.0302 0.1250 0.0968 0.2791
0.6075 1.6686 -37.2833
9.0000 -34.8400 -0.1507 -0.1506 -
0.0931 0.0236 0.1096 0.1034 0.2894
0.6059 1.6604 -37.2858
9.0200 -34.7700 0.0824 -0.1064 -
0.1574 0.0154 0.0936 0.1098 0.2997
0.6041 1.6521 -37.2884
9.0400 -34.6600 0.0979 -0.0867 -
0.1618 0.0065 0.0770 0.1160 0.3100
0.6023 1.6438 -37.2909
9.0600 -34.8900 -0.2573 -0.0681 -
0.1066 -0.0026 0.0601 0.1219 0.3203
0.6003 1.6355 -37.2935
9.0800 -34.2100 0.2748 -0.0229 -
0.0134 -0.0114 0.0432 0.1276 0.3304
0.5982 1.6271 -37.2961
9.1000 -34.5600 -0.0572 0.0322
0.0853 -0.0193 0.0263 0.1331 0.3406
0.5960 1.6188 -37.2986
9.1200 -34.7100 -0.2882 0.1173
0.1592 -0.0252 0.0097 0.1384 0.3506
0.5937 1.6103 -37.3012
9.1400 -33.9100 0.3732 0.1805
0.1875 -0.0283 -0.0064 0.1435 0.3606
0.5913 1.6019 -37.3037
9.1600 -34.5300 -0.0628 0.1073
0.1619 -0.0283 -0.0217 0.1483 0.3705
0.5888 1.5934 -37.3063
9.1800 -34.8100 -0.2108 -0.0209
0.0912 -0.0251 -0.0362 0.1530 0.3803
0.5861 1.5850 -37.3089
9.2000 -34.8700 -0.1036 -0.0211 -
0.0075 -0.0190 -0.0497 0.1574 0.3899
0.5834 1.5764 -37.3114

9.2200 -34.5200 0.1794 0.0634 -
0.1105 -0.0104 -0.0618 0.1617 0.3995
0.5806 1.5679 -37.3140
9.2400 -34.7100 0.1404 0.0424 -
0.1949 0.0000 -0.0727 0.1658 0.4089
0.5776 1.5593 -37.3166
9.2600 -35.0500 -0.1017 -0.0926 -
0.2423 0.0114 -0.0820 0.1698 0.4182
0.5746 1.5507 -37.3191
9.2800 -35.3700 -0.3618 -0.1771 -
0.2380 0.0227 -0.0897 0.1736 0.4273
0.5714 1.5421 -37.3217
9.3000 -34.7500 0.2134 -0.1513 -
0.1718 0.0328 -0.0959 0.1772 0.4363
0.5682 1.5334 -37.3243
9.3200 -34.6200 0.2609 -0.1039 -
0.0527 0.0405 -0.1003 0.1807 0.4450
0.5648 1.5248 -37.3268
9.3400 -35.0800 -0.4606 -0.0109
0.0921 0.0445 -0.1029 0.1841 0.4536
0.5614 1.5161 -37.3294
9.3600 -34.1200 0.2969 0.1524
0.2280 0.0437 -0.1037 0.1873 0.4621
0.5578 1.5073 -37.3320
9.3800 -34.1700 0.0432 0.2246
0.3206 0.0372 -0.1027 0.1904 0.4703
0.5541 1.4986 -37.3345
9.4000 -34.2400 -0.0844 0.1235
0.3452 0.0245 -0.1000 0.1933 0.4783
0.5504 1.4898 -37.3371
9.4200 -34.1000 0.2056 -0.0282
0.2952 0.0063 -0.0957 0.1961 0.4860
0.5465 1.4810 -37.3397
9.4400 -35.1100 -0.4626 -0.0439
0.1822 -0.0157 -0.0897 0.1988 0.4936
0.5426 1.4722 -37.3423
9.4600 -34.3300 0.3671 0.0594
0.0305 -0.0390 -0.0822 0.2013 0.5009
0.5385 1.4633 -37.3448
9.4800 -34.5900 0.2816 0.0535 -
0.1273 -0.0614 -0.0734 0.2037 0.5079
0.5343 1.4544 -37.3474
9.5000 -35.4200 -0.3107 -0.0742 -
0.2605 -0.0804 -0.0632 0.2060 0.5147
0.5301 1.4456 -37.3500
9.5200 -35.4400 -0.1431 -0.1439 -
0.3438 -0.0938 -0.0520 0.2081 0.5212
0.5257 1.4366 -37.3526
9.5400 -34.9500 0.2307 -0.0730 -
0.3567 -0.0997 -0.0400 0.2101 0.5274
0.5212 1.4277 -37.3551

9.5600 -35.2900 -0.2128 -0.0132 -
0.2971 -0.0977 -0.0272 0.2120 0.5333
0.5167 1.4187 -37.3577
9.5800 -34.7500 0.2385 -0.0799 -
0.1828 -0.0884 -0.0139 0.2138 0.5389
0.5121 1.4097 -37.3603
9.6000 -35.2300 -0.2765 -0.1357 -
0.0471 -0.0734 -0.0003 0.2154 0.5442
0.5073 1.4007 -37.3629
9.6200 -34.6900 0.0161 -0.0072
0.0753 -0.0542 0.0134 0.2170 0.5492
0.5025 1.3917 -37.3654
9.6400 -34.3100 0.0711 0.1851
0.1604 -0.0326 0.0270 0.2184 0.5538
0.4976 1.3826 -37.3680
9.6600 -34.1400 0.2029 0.2314
0.1984 -0.0101 0.0403 0.2197 0.5581
0.4926 1.3736 -37.3706
9.6800 -34.7100 -0.3250 0.1079
0.1937 0.0117 0.0531 0.2209 0.5621
0.4875 1.3645 -37.3732
9.7000 -34.4000 0.3230 -0.1107
0.1654 0.0307 0.0652 0.2220 0.5657
0.4823 1.3553 -37.3758
9.7200 -35.2200 -0.5945 -0.2188
0.1347 0.0452 0.0764 0.2230 0.5689
0.4770 1.3462 -37.3783
9.7400 -34.3600 0.2975 -0.1073
0.1109 0.0541 0.0866 0.2239 0.5718
0.4717 1.3370 -37.3809
9.7600 -34.1700 0.3066 0.0080
0.0908 0.0576 0.0955 0.2247 0.5742
0.4662 1.3279 -37.3835
9.7800 -34.4000 0.1823 -0.0119
0.0654 0.0564 0.1032 0.2255 0.5763
0.4607 1.3187 -37.3861
9.8000 -35.1700 -0.6052 0.0195
0.0224 0.0517 0.1094 0.2262 0.5779
0.4551 1.3094 -37.3887
9.8200 -34.5100 0.0306 0.1855 -
0.0407 0.0449 0.1143 0.2268 0.5792
0.4494 1.3002 -37.3913
9.8400 -33.9100 0.6528 0.2328 -
0.1096 0.0375 0.1176 0.2274 0.5800
0.4437 1.2909 -37.3938
9.8600 -35.2800 -0.4736 0.0018 -
0.1616 0.0308 0.1195 0.2280 0.5805
0.4378 1.2817 -37.3964
9.8800 -35.2400 -0.0325 -0.2420 -
0.1757 0.0257 0.1198 0.2285 0.5805
0.4319 1.2724 -37.3990

9.9000	-35.1100	0.0790	-0.2567	-
0.1412	0.0228	0.1185	0.2290	0.5800
	0.4259	1.2631	-37.4016	
9.9200	-35.0100	-0.1020	-0.0109	-
0.0646	0.0222	0.1156	0.2295	0.5792
	0.4198	1.2537	-37.4042	
9.9400	-34.9400	-0.4298	0.2667	
0.0262	0.0230	0.1112	0.2301	0.5779
	0.4137	1.2444	-37.4068	
9.9600	-33.6800	0.8282	0.2145	
0.0988	0.0240	0.1052	0.2307	0.5761
	0.4075	1.2350	-37.4094	
9.9800	-35.7000	-0.8687	-0.1261	
0.1330	0.0240	0.0978	0.2313	0.5739
	0.4012	1.2256	-37.4120	
10.0000	-34.2000	0.7164	-0.1759	
0.1286	0.0225	0.0891	0.2320	0.5713
	0.3948	1.2162	-37.4146	
10.0200	-34.9600	-0.0657	-0.0484	
0.1020	0.0198	0.0790	0.2328	0.5681
	0.3884	1.2068	-37.4171	
10.0400	-34.9100	-0.0820	-0.0096	
0.0655	0.0167	0.0679	0.2337	0.5646
	0.3819	1.1974	-37.4197	
10.0600	-35.3000	-0.4835	0.1367	
0.0176	0.0139	0.0557	0.2347	0.5606
	0.3754	1.1879	-37.4223	
10.0800	-34.3900	0.4125	0.2991	-
0.0460	0.0120	0.0427	0.2359	0.5561
	0.3688	1.1784	-37.4249	
10.1000	-34.8300	0.2086	0.1517	-
0.1157	0.0115	0.0291	0.2373	0.5511
	0.3621	1.1690	-37.4275	
10.1200	-35.9000	-0.4311	-0.2161	-
0.1660	0.0132	0.0150	0.2388	0.5457
	0.3553	1.1595	-37.4301	
10.1400	-35.6300	0.1139	-0.3935	-
0.1735	0.0171	0.0007	0.2406	0.5398
	0.3485	1.1499	-37.4327	
10.1600	-35.5100	-0.1294	-0.1650	-
0.1315	0.0226	-0.0137	0.2426	0.5334
	0.3417	1.1404	-37.4353	
10.1800	-35.2700	-0.1650	0.2228	-
0.0564	0.0280	-0.0280	0.2449	0.5266
	0.3347	1.1309	-37.4379	
10.2000	-34.4000	0.4776	0.3800	
0.0271	0.0316	-0.0421	0.2475	0.5193
	0.3278	1.1213	-37.4405	
10.2200	-35.1700	-0.1482	0.1486	
0.0997	0.0320	-0.0557	0.2505	0.5115
	0.3207	1.1117	-37.4431	

10.2400	-35.5100	-0.2281	-0.1714	
0.1519	0.0283	-0.0687	0.2538	0.5033
	0.3136	1.1022	-37.4457	
10.2600	-35.4600	-0.1024	-0.2609	
0.1773	0.0199	-0.0809	0.2574	0.4946
	0.3065	1.0926	-37.4483	
10.2800	-35.2210	0.2422	-0.1328	
0.1751	0.0071	-0.0923	0.2615	0.4854
	0.2993	1.0829	-37.4509	
10.3000	-35.0200	0.1361	0.0750	
0.1449	-0.0096	-0.1026	0.2660	0.4757
	0.2921	1.0733	-37.4535	
10.3200	-35.6100	-0.4316	0.2551	
0.0865	-0.0288	-0.1118	0.2709	0.4656
	0.2848	1.0637	-37.4561	
10.3400	-35.0100	0.3477	0.2613	
0.0044	-0.0488	-0.1197	0.2763	0.4551
	0.2775	1.0540	-37.4587	
10.3600	-35.7100	-0.0237	0.0373	-
0.0885	-0.0679	-0.1265	0.2822	0.4440
	0.2701	1.0444	-37.4613	
10.3800	-36.0500	-0.1013	-0.2144	-
0.1686	-0.0840	-0.1319	0.2886	0.4326
	0.2627	1.0347	-37.4639	
10.4000	-36.3200	-0.2182	-0.2505	-
0.2112	-0.0953	-0.1359	0.2955	0.4206
	0.2552	1.0250	-37.4665	
10.4200	-35.8500	0.1508	-0.0955	-
0.2047	-0.1002	-0.1386	0.3030	0.4083
	0.2477	1.0153	-37.4691	
10.4400	-35.7800	0.1422	0.0419	-
0.1514	-0.0982	-0.1399	0.3109	0.3954
	0.2402	1.0056	-37.4717	
10.4600	-35.9200	-0.1123	0.0568	-
0.0681	-0.0895	-0.1400	0.3195	0.3822
	0.2326	0.9959	-37.4743	
10.4800	-35.7000	0.0535	0.0341	
0.0193	-0.0749	-0.1391	0.3286	0.3685
	0.2250	0.9861	-37.4769	
10.5000	-35.4800	-0.0186	0.0604	
0.0872	-0.0557	-0.1371	0.3382	0.3545
	0.2174	0.9764	-37.4795	
10.5200	-35.7800	-0.1549	0.0926	
0.1224	-0.0334	-0.1345	0.3484	0.3400
	0.2097	0.9666	-37.4821	
10.5400	-35.2600	0.3700	0.0439	
0.1254	-0.0097	-0.1313	0.3592	0.3251
	0.2020	0.9569	-37.4847	
10.5600	-36.0100	-0.3434	-0.0490	
0.1069	0.0135	-0.1278	0.3705	0.3098
	0.1943	0.9471	-37.4873	

10.5800 -35.6900 0.0540 -0.0631
0.0818 0.0349 -0.1243 0.3823 0.2942
0.1865 0.9373 -37.4899
10.6000 -35.5700 0.1383 -0.0048
0.0603 0.0532 -0.1209 0.3947 0.2782
0.1787 0.9275 -37.4925
10.6200 -35.6100 0.1174 0.0005
0.0434 0.0683 -0.1179 0.4075 0.2618
0.1709 0.9177 -37.4951
10.6400 -36.0600 -0.2964 -0.0242
0.0254 0.0801 -0.1155 0.4208 0.2451
0.1631 0.9079 -37.4977
10.6600 -35.6800 0.1583 0.0122 -
0.0006 0.0890 -0.1138 0.4344 0.2281
0.1552 0.8981 -37.5003
10.6800 -35.8000 0.0002 0.0563 -
0.0377 0.0952 -0.1130 0.4484 0.2108
0.1473 0.8882 -37.5029
10.7000 -35.7600 0.2853 0.0092 -
0.0789 0.0991 -0.1130 0.4626 0.1932
0.1394 0.8784 -37.5056
10.7200 -36.4300 -0.4728 -0.0315 -
0.1109 0.1007 -0.1140 0.4770 0.1754
0.1315 0.8686 -37.5082
10.7400 -35.5400 0.5045 -0.0177 -
0.1179 0.0998 -0.1159 0.4916 0.1573
0.1236 0.8587 -37.5108
10.7600 -36.1700 -0.1169 -0.0534 -
0.0864 0.0957 -0.1187 0.5061 0.1389
0.1156 0.8489 -37.5134
10.7800 -36.5600 -0.3897 -0.0939 -
0.0223 0.0877 -0.1222 0.5206 0.1203
0.1077 0.8390 -37.5160
10.8000 -35.5000 0.4883 -0.0693
0.0550 0.0748 -0.1261 0.5349 0.1015
0.0997 0.8291 -37.5186
10.8200 -36.1900 -0.3336 0.0310
0.1204 0.0561 -0.1301 0.5488 0.0825
0.0917 0.8192 -37.5212
10.8400 -35.5000 0.2968 0.1356
0.1510 0.0312 -0.1340 0.5624 0.0634
0.0837 0.8094 -37.5238
10.8600 -36.0700 -0.1396 0.1832
0.1339 0.0010 -0.1372 0.5754 0.0441
0.0757 0.7995 -37.5264
10.8800 -36.1100 -0.1227 0.1582
0.0720 -0.0326 -0.1396 0.5878 0.0246
0.0677 0.7896 -37.5291
10.9000 -36.0700 0.1717 0.0397 -
0.0166 -0.0667 -0.1405 0.5994 0.0051
0.0597 0.7797 -37.5317

10.9200 -36.3500 0.1660 -0.1442 -
0.1044 -0.0985 -0.1398 0.6101 -0.0146
0.0517 0.7698 -37.5343

10.9400 -37.2500 -0.4615 -0.2691 -
0.1642 -0.1253 -0.1368 0.6199 -0.0344
0.0437 0.7598 -37.5369

10.9600 -36.5500 0.3064 -0.2183 -
0.1726 -0.1448 -0.1314 0.6286 -0.0543
0.0356 0.7499 -37.5395

10.9800 -36.7800 -0.0654 -0.0896 -
0.1250 -0.1559 -0.1231 0.6361 -0.0742
0.0276 0.7400 -37.5421

11.0000 -36.6900 -0.1250 0.0175 -
0.0389 -0.1589 -0.1117 0.6423 -0.0942
0.0196 0.7301 -37.5447

11.0200 -36.4500 -0.1712 0.1592
0.0534 -0.1551 -0.0970 0.6472 -0.1142
0.0116 0.7201 -37.5474

11.0400 -35.8200 0.4010 0.2416
0.1199 -0.1461 -0.0789 0.6506 -0.1342
0.0036 0.7102 -37.5500

11.0600 -36.3100 -0.0974 0.1412
0.1398 -0.1334 -0.0575 0.6525 -0.1542
-0.0044 0.7003 -37.5526

11.0800 -36.8400 -0.3922 -0.0207
0.1106 -0.1193 -0.0327 0.6527 -0.1742
-0.0124 0.6903 -37.5552

11.1000 -36.2700 0.3476 -0.0817
0.0476 -0.1059 -0.0047 0.6514 -0.1942
-0.0204 0.6804 -37.5578

11.1200 -36.4900 0.0358 -0.0553 -
0.0253 -0.0957 0.0263 0.6482 -0.2142
-0.0284 0.6705 -37.5604

11.1400 -36.8700 -0.2808 0.0080 -
0.0829 -0.0907 0.0601 0.6433 -0.2340
-0.0363 0.6605 -37.5631

11.1600 -36.5000 0.0950 0.0271 -
0.1034 -0.0921 0.0964 0.6366 -0.2539
-0.0443 0.6506 -37.5657

11.1800 -36.3400 0.3829 -0.0631 -
0.0745 -0.1008 0.1346 0.6279 -0.2736
-0.0522 0.6406 -37.5683

11.2000 -37.1700 -0.4281 -0.1791
0.0002 -0.1160 0.1741 0.6173 -0.2933
-0.0601 0.6306 -37.5709

11.2200 -36.7500 -0.0109 -0.1060
0.1078 -0.1360 0.2142 0.6048 -0.3128
-0.0680 0.6207 -37.5735

11.2400 -36.3600 -0.0722 0.1375
0.2274 -0.1579 0.2543 0.5901 -0.3323
-0.0759 0.6107 -37.5762

11.2600 -35.5700 0.5471 0.2176
0.3316 -0.1783 0.2935 0.5734 -0.3516
-0.0837 0.6008 -37.5788
11.2800 -36.4400 -0.0852 -0.0079
0.3934 -0.1933 0.3310 0.5546 -0.3707
-0.0916 0.5908 -37.5814
11.3000 -37.0800 -0.4049 -0.2237
0.3903 -0.1994 0.3661 0.5337 -0.3897
-0.0994 0.5809 -37.5840
11.3200 -36.5700 0.1078 -0.1290
0.3105 -0.1941 0.3979 0.5106 -0.4086
-0.1072 0.5709 -37.5867
11.3400 -36.4500 0.1589 0.1684
0.1552 -0.1764 0.4258 0.4853 -0.4273
-0.1149 0.5610 -37.5893
11.3600 -36.5700 0.0047 0.3114 -
0.0590 -0.1456 0.4491 0.4578 -0.4458
-0.1226 0.5510 -37.5919
11.3800 -36.6700 0.3667 0.1328 -
0.2987 -0.1017 0.4674 0.4282 -0.4641
-0.1303 0.5410 -37.5945
11.4000 -38.1200 -0.5057 -0.1894 -
0.5166 -0.0450 0.4802 0.3964 -0.4822
-0.1380 0.5311 -37.5971
11.4200 -37.5800 0.2832 -0.3569 -
0.6626 0.0238 0.4872 0.3625 -0.5000
-0.1456 0.5211 -37.5998
11.4400 -37.7700 0.0848 -0.2819 -
0.6986 0.1034 0.4881 0.3265 -0.5177
-0.1532 0.5112 -37.6024
11.4600 -37.9900 -0.3564 -0.0382 -
0.6146 0.1907 0.4829 0.2885 -0.5351
-0.1608 0.5012 -37.6050
11.4800 -36.6900 0.3436 0.2012 -
0.4234 0.2804 0.4715 0.2486 -0.5522
-0.1683 0.4913 -37.6076
11.5000 -36.8400 0.0909 0.1702 -
0.1543 0.3658 0.4536 0.2069 -0.5690
-0.1758 0.4813 -37.6103
11.5200 -36.8900 -0.1767 -0.0945
0.1471 0.4398 0.4295 0.1636 -0.5856
-0.1832 0.4714 -37.6129
11.5400 -36.8140 -0.1253 -0.2655
0.4263 0.4954 0.3990 0.1188 -0.6019
-0.1906 0.4615 -37.6155
11.5600 -36.6200 -0.0136 -0.1904
0.6368 0.5252 0.3623 0.0727 -0.6179
-0.1980 0.4515 -37.6182
11.5800 -36.0300 0.3045 0.0260
0.7472 0.5228 0.3200 0.0253 -0.6335
-0.2053 0.4416 -37.6208

11.6000 -36.6000 -0.2980 0.3293
0.7325 0.4848 0.2726 -0.0230 -0.6489
-0.2126 0.4317 -37.6234
11.6200 -36.5000 -0.2045 0.6424
0.5834 0.4130 0.2212 -0.0722 -0.6639
-0.2198 0.4217 -37.6260
11.6400 -35.9900 0.6026 0.7363
0.3227 0.3135 0.1668 -0.1220 -0.6785
-0.2270 0.4118 -37.6287
11.6600 -37.7700 -0.0277 0.3483
0.0048 0.1936 0.1105 -0.1725 -0.6928
-0.2341 0.4019 -37.6313
11.6800 -38.7300 0.3507 -0.4371 -
0.2948 0.0605 0.0534 -0.2232 -0.7067
-0.2412 0.3920 -37.6339
11.7000 -40.7700 -0.8791 -0.9325 -
0.4939 -0.0783 -0.0033 -0.2742 -0.7202
-0.2482 0.3821 -37.6365
11.7200 -39.7200 0.1960 -0.5476 -
0.5322 -0.2153 -0.0587 -0.3253 -0.7333
-0.2552 0.3722 -37.6392
11.7400 -39.5400 -0.1914 0.3181 -
0.4403 -0.3415 -0.1116 -0.3762 -0.7460
-0.2621 0.3623 -37.6418
11.7600 -38.1300 0.8989 0.6698 -
0.2818 -0.4475 -0.1609 -0.4269 -0.7583
-0.2689 0.3524 -37.6444
11.7800 -40.2000 -0.7027 0.2321 -
0.1268 -0.5241 -0.2054 -0.4771 -0.7701
-0.2757 0.3425 -37.6471
11.8000 -39.9100 0.0461 -0.1683 -
0.0362 -0.5631 -0.2441 -0.5268 -0.7815
-0.2825 0.3326 -37.6497
11.8200 -40.3700 -0.5536 0.0274 -
0.0442 -0.5609 -0.2762 -0.5757 -0.7924
-0.2891 0.3227 -37.6523
11.8400 -38.9700 0.7839 0.2330 -
0.1318 -0.5192 -0.3010 -0.6237 -0.8029
-0.2957 0.3129 -37.6550
11.8600 -40.5100 -0.4096 0.0471 -
0.2482 -0.4435 -0.3185 -0.6707 -0.8128
-0.3023 0.3030 -37.6576
11.8800 -40.9800 -0.4970 -0.1974 -
0.3414 -0.3414 -0.3291 -0.7164 -0.8223
-0.3087 0.2931 -37.6602
11.9000 -39.9300 0.5273 -0.2270 -
0.3720 -0.2218 -0.3332 -0.7609 -0.8313
-0.3152 0.2833 -37.6629
11.9200 -40.5600 -0.4068 -0.0719 -
0.3178 -0.0945 -0.3313 -0.8039 -0.8397
-0.3215 0.2735 -37.6655

11.9400 -39.5800 0.2363 0.0609 -
0.1784 0.0313 -0.3240 -0.8453 -0.8476
-0.3278 0.2636 -37.6681
11.9600 -39.5700 -0.0131 0.0187
0.0224 0.1474 -0.3117 -0.8852 -0.8550
-0.3340 0.2538 -37.6708
11.9800 -39.3600 0.1571 -0.1629
0.2429 0.2464 -0.2950 -0.9232 -0.8618
-0.3401 0.2440 -37.6734
12.0000 -39.2300 0.1076 -0.1793
0.4277 0.3215 -0.2745 -0.9594 -0.8680
-0.3461 0.2342 -37.6760
12.0200 -39.7500 -0.9069 0.1853
0.5269 0.3669 -0.2508 -0.9937 -0.8737
-0.3521 0.2244 -37.6787
12.0400 -37.5000 1.0538 0.5259
0.5068 0.3800 -0.2248 -1.0259 -0.8788
-0.3580 0.2146 -37.6813
12.0600 -39.0100 -0.1247 0.2947
0.3618 0.3639 -0.1971 -1.0560 -0.8832
-0.3638 0.2048 -37.6839
12.0800 -40.7400 -0.9620 -0.2063
0.1426 0.3256 -0.1688 -1.0839 -0.8871
-0.3695 0.1951 -37.6866
12.1000 -40.5100 -0.3278 -0.4452 -
0.0832 0.2739 -0.1408 -1.1095 -0.8903
-0.3752 0.1853 -37.6892
12.1200 -39.9900 0.4367 -0.3305 -
0.2613 0.2176 -0.1140 -1.1327 -0.8929
-0.3808 0.1756 -37.6918
12.1400 -39.3600 0.9615 -0.0642 -
0.3612 0.1654 -0.0894 -1.1535 -0.8948
-0.3863 0.1658 -37.6945
12.1600 -41.0100 -1.1378 0.3044 -
0.3889 0.1246 -0.0678 -1.1718 -0.8961
-0.3917 0.1561 -37.6971
12.1800 -38.9200 1.1490 0.3392 -
0.3544 0.1004 -0.0500 -1.1876 -0.8967
-0.3970 0.1464 -37.6997
12.2000 -41.5000 -1.1029 -0.1051 -
0.2702 0.0941 -0.0365 -1.2008 -0.8966
-0.4022 0.1367 -37.7024
12.2200 -39.5900 0.8101 -0.2742 -
0.1525 0.1037 -0.0275 -1.2115 -0.8958
-0.4073 0.1270 -37.7050
12.2400 -39.6500 0.3914 -0.1715 -
0.0132 0.1253 -0.0230 -1.2196 -0.8943
-0.4124 0.1173 -37.7076
12.2600 -39.8800 0.2113 -0.1558
0.1302 0.1539 -0.0226 -1.2251 -0.8921
-0.4174 0.1076 -37.7103

12.2800 -40.9000 -1.2528 0.1123
0.2320 0.1851 -0.0258 -1.2282 -0.8891
-0.4222 0.0980 -37.7129
12.3000 -38.5300 0.7859 0.4746
0.2595 0.2144 -0.0319 -1.2287 -0.8855
-0.4270 0.0883 -37.7156
12.3200 -38.0500 1.3087 0.3496
0.2114 0.2373 -0.0401 -1.2269 -0.8811
-0.4317 0.0787 -37.7182
12.3400 -41.5600 -1.3400 -0.3032
0.1187 0.2499 -0.0497 -1.2227 -0.8761
-0.4363 0.0691 -37.7208
12.3600 -40.5900 -0.0226 -0.5750
0.0306 0.2489 -0.0599 -1.2163 -0.8703
-0.4408 0.0595 -37.7235
12.3800 -40.6200 -0.5360 -0.0543 -
0.0143 0.2322 -0.0700 -1.2076 -0.8638
-0.4452 0.0499 -37.7261
12.4000 -38.8400 0.7439 0.4778 -
0.0218 0.1989 -0.0795 -1.1967 -0.8567
-0.4496 0.0403 -37.7287
12.4200 -39.1500 0.5883 0.4778 -
0.0094 0.1492 -0.0879 -1.1838 -0.8489
-0.4538 0.0307 -37.7314
12.4400 -40.5100 -0.3442 0.0401
0.0109 0.0841 -0.0948 -1.1688 -0.8404
-0.4579 0.0212 -37.7340
12.4600 -41.0300 -0.4641 -0.3476
0.0307 0.0055 -0.1001 -1.1519 -0.8313
-0.4620 0.0117 -37.7367
12.4800 -40.3400 0.2801 -0.3934
0.0414 -0.0833 -0.1035 -1.1331 -0.8216
-0.4659 0.0021 -37.7393
12.5000 -40.5100 0.1701 -0.2033
0.0417 -0.1783 -0.1050 -1.1124 -0.8112
-0.4698 -0.0074 -37.7419
12.5200 -40.6200 -0.2073 0.0449
0.0275 -0.2746 -0.1044 -1.0900 -0.8003
-0.4736 -0.0168 -37.7446
12.5400 -40.5600 -0.2232 0.2848 -
0.0162 -0.3662 -0.1015 -1.0659 -0.7888
-0.4773 -0.0263 -37.7472
12.5600 -40.2900 0.0528 0.3727 -
0.0962 -0.4472 -0.0963 -1.0402 -0.7767
-0.4808 -0.0358 -37.7499
12.5800 -40.0800 0.6545 0.1109 -
0.2006 -0.5120 -0.0884 -1.0130 -0.7640
-0.4843 -0.0452 -37.7525
12.6000 -41.9100 -0.7774 -0.2822 -
0.2953 -0.5562 -0.0780 -0.9844 -0.7509
-0.4877 -0.0546 -37.7551

12.6200 -40.4700 0.7039 -0.2781 -
0.3342 -0.5762 -0.0649 -0.9544 -0.7372
-0.4911 -0.0640 -37.7578
12.6400 -41.5600 -0.5434 -0.1486 -
0.2817 -0.5698 -0.0494 -0.9231 -0.7229
-0.4943 -0.0734 -37.7604
12.6600 -40.3300 0.5546 -0.1892 -
0.1448 -0.5369 -0.0317 -0.8906 -0.7082
-0.4974 -0.0828 -37.7631
12.6800 -41.0500 -0.5870 -0.0412
0.0226 -0.4791 -0.0124 -0.8569 -0.6930
-0.5004 -0.0922 -37.7657
12.7000 -39.8700 -0.0551 0.3219
0.1545 -0.3984 0.0083 -0.8222 -0.6774
-0.5034 -0.1015 -37.7684
12.7200 -38.8600 0.5755 0.5435
0.1994 -0.2974 0.0296 -0.7864 -0.6613
-0.5063 -0.1108 -37.7710
12.7400 -39.0900 0.4206 0.3124
0.1316 -0.1802 0.0512 -0.7496 -0.6447
-0.5090 -0.1201 -37.7736
12.7600 -40.6300 -0.6934 -0.1257 -
0.0152 -0.0525 0.0726 -0.7119 -0.6278
-0.5117 -0.1294 -37.7763
12.7800 -40.2500 -0.0844 -0.3464 -
0.1844 0.0790 0.0932 -0.6733 -0.6104
-0.5143 -0.1387 -37.7789
12.8000 -39.8900 0.1205 -0.3154 -
0.3182 0.2071 0.1126 -0.6339 -0.5926
-0.5168 -0.1479 -37.7816
12.8200 -39.3900 0.3981 -0.2519 -
0.3720 0.3256 0.1302 -0.5938 -0.5745
-0.5192 -0.1571 -37.7842
12.8400 -39.9400 -0.3765 -0.1818 -
0.3307 0.4297 0.1456 -0.5531 -0.5560
-0.5216 -0.1663 -37.7869
12.8600 -39.0100 0.0664 -0.0301 -
0.2060 0.5157 0.1583 -0.5117 -0.5371
-0.5238 -0.1755 -37.7895
12.8800 -38.3500 0.3329 0.1356 -
0.0268 0.5807 0.1678 -0.4699 -0.5179
-0.5260 -0.1847 -37.7921
12.9000 -38.3600 -0.2573 0.2153
0.1732 0.6221 0.1737 -0.4276 -0.4984
-0.5281 -0.1939 -37.7948
12.9200 -38.3600 -0.3285 0.2060
0.3687 0.6366 0.1759 -0.3849 -0.4786
-0.5301 -0.2030 -37.7974
12.9400 -37.2800 0.6698 0.0848
0.5413 0.6211 0.1744 -0.3419 -0.4584
-0.5320 -0.2121 -37.8001

12.9600 -38.6400 -0.5880 -0.1199
0.6699 0.5728 0.1693 -0.2988 -0.4380
-0.5338 -0.2212 -37.8027
12.9800 -38.3600 -0.2434 -0.2319
0.7305 0.4908 0.1613 -0.2556 -0.4173
-0.5356 -0.2302 -37.8054
13.0000 -37.4700 0.7375 -0.1607
0.7035 0.3770 0.1508 -0.2123 -0.3964
-0.5372 -0.2393 -37.8080
13.0200 -38.6600 -0.6289 0.0730
0.5744 0.2376 0.1383 -0.1691 -0.3753
-0.5388 -0.2483 -37.8106
13.0400 -38.1700 -0.0889 0.3834
0.3430 0.0823 0.1246 -0.1261 -0.3539
-0.5403 -0.2573 -37.8133
13.0600 -37.8600 0.6161 0.5387
0.0388 -0.0781 0.1101 -0.0833 -0.3323
-0.5418 -0.2663 -37.8159
13.0800 -39.4200 -0.3464 0.2891 -
0.2881 -0.2327 0.0955 -0.0408 -0.3105
-0.5431 -0.2752 -37.8186
13.1000 -39.7400 0.4533 -0.3223 -
0.5680 -0.3709 0.0812 0.0012 -0.2885
-0.5444 -0.2842 -37.8212
13.1200 -40.3800 0.3449 -0.7012 -
0.7242 -0.4818 0.0680 0.0428 -0.2664
-0.5456 -0.2931 -37.8239
13.1400 -41.5100 -1.0981 -0.3921 -
0.7071 -0.5568 0.0563 0.0838 -0.2441
-0.5467 -0.3020 -37.8265
13.1600 -38.4100 1.0483 0.4350 -
0.5560 -0.5923 0.0466 0.1243 -0.2217
-0.5477 -0.3108 -37.8292
13.1800 -39.7400 -0.5915 0.5217 -
0.3426 -0.5894 0.0395 0.1640 -0.1991
-0.5487 -0.3197 -37.8318
13.2000 -39.1200 0.3741 -0.1214 -
0.1391 -0.5509 0.0350 0.2031 -0.1765
-0.5496 -0.3285 -37.8344
13.2200 -39.9600 -0.5813 -0.3146
0.0018 -0.4804 0.0330 0.2415 -0.1537
-0.5504 -0.3373 -37.8371
13.2400 -38.4800 0.3805 0.0299
0.0729 -0.3838 0.0332 0.2792 -0.1309
-0.5511 -0.3460 -37.8397
13.2600 -38.4600 -0.0514 0.2416
0.1136 -0.2698 0.0351 0.3162 -0.1080
-0.5518 -0.3548 -37.8424
13.2800 -38.0400 0.4079 0.0099
0.1658 -0.1482 0.0384 0.3525 -0.0851
-0.5524 -0.3635 -37.8450

13.3000 -39.0900 -0.6102 -0.3046
0.2340 -0.0276 0.0428 0.3881 -0.0621
-0.5529 -0.3722 -37.8477
13.3200 -37.7800 0.4600 -0.2399
0.3044 0.0855 0.0480 0.4229 -0.0391
-0.5534 -0.3809 -37.8503
13.3400 -38.0900 -0.4158 0.1410
0.3497 0.1855 0.0534 0.4571 -0.0161
-0.5537 -0.3895 -37.8530
13.3600 -36.7500 0.6085 0.3503
0.3442 0.2678 0.0589 0.4905 0.0069
-0.5540 -0.3981 -37.8556
13.3800 -37.8900 -0.4078 0.1704
0.2828 0.3296 0.0641 0.5232 0.0299
-0.5543 -0.4067 -37.8583
13.4000 -37.4300 0.3087 -0.1342
0.1931 0.3703 0.0685 0.5552 0.0529
-0.5545 -0.4153 -37.8609
13.4200 -38.2500 -0.3198 -0.3063
0.1114 0.3909 0.0718 0.5865 0.0758
-0.5546 -0.4238 -37.8636
13.4400 -37.8100 0.0397 -0.2843
0.0666 0.3935 0.0735 0.6170 0.0986
-0.5546 -0.4323 -37.8662
13.4600 -37.6000 -0.0062 -0.0978
0.0640 0.3805 0.0733 0.6469 0.1214
-0.5546 -0.4408 -37.8688
13.4800 -37.2800 0.1076 0.1075
0.0880 0.3552 0.0709 0.6761 0.1440
-0.5545 -0.4493 -37.8715
13.5000 -37.2500 0.0258 0.2235
0.1107 0.3214 0.0659 0.7045 0.1666
-0.5543 -0.4577 -37.8741
13.5200 -37.4600 -0.2850 0.2789
0.1033 0.2829 0.0581 0.7323 0.1890
-0.5541 -0.4661 -37.8768
13.5400 -37.3200 0.0357 0.2520
0.0547 0.2434 0.0472 0.7594 0.2113
-0.5538 -0.4745 -37.8794
13.5600 -37.2200 0.3844 0.0375 -
0.0263 0.2065 0.0330 0.7858 0.2334
-0.5534 -0.4829 -37.8821
13.5800 -38.2100 -0.1677 -0.2389 -
0.1154 0.1755 0.0154 0.8115 0.2554
-0.5530 -0.4912 -37.8847
13.6000 -38.6600 -0.5231 -0.2978 -
0.1872 0.1528 -0.0055 0.8365 0.2771
-0.5525 -0.4995 -37.8874
13.6200 -37.8400 0.1403 -0.0810 -
0.2241 0.1402 -0.0297 0.8609 0.2987
-0.5519 -0.5078 -37.8900

13.6400 -37.1500 0.5436 0.1598 -
0.2221 0.1382 -0.0567 0.8846 0.3201
-0.5513 -0.5160 -37.8927
13.6600 -38.3000 -0.5922 0.2064 -
0.1864 0.1462 -0.0859 0.9077 0.3413
-0.5507 -0.5242 -37.8953
13.6800 -37.1400 0.5211 0.0695 -
0.1296 0.1618 -0.1164 0.9300 0.3622
-0.5499 -0.5324 -37.8980
13.7000 -38.1200 -0.3789 -0.1107 -
0.0655 0.1816 -0.1474 0.9516 0.3828
-0.5491 -0.5406 -37.9006
13.7200 -37.7200 -0.0170 -0.1620 -
0.0049 0.2021 -0.1777 0.9725 0.4032
-0.5483 -0.5487 -37.9033
13.7400 -37.3700 0.1336 0.0225
0.0448 0.2189 -0.2066 0.9927 0.4233
-0.5473 -0.5568 -37.9059
13.7600 -37.5000 -0.1733 0.2679
0.0802 0.2274 -0.2330 1.0121 0.4431
-0.5464 -0.5649 -37.9086
13.7800 -36.7000 0.4745 0.2699
0.1009 0.2229 -0.2562 1.0307 0.4626
-0.5453 -0.5729 -37.9112
13.8000 -37.4900 0.0080 -0.0223
0.1135 0.2008 -0.2754 1.0485 0.4818
-0.5442 -0.5809 -37.9138
13.8200 -38.2800 -0.5982 -0.3046
0.1312 0.1578 -0.2900 1.0655 0.5006
-0.5431 -0.5889 -37.9165
13.8400 -37.4600 0.5300 -0.3633
0.1696 0.0917 -0.2997 1.0816 0.5191
-0.5419 -0.5969 -37.9191
13.8600 -37.8400 -0.0989 -0.3522
0.2351 0.0025 -0.3043 1.0970 0.5372
-0.5406 -0.6048 -37.9218
13.8800 -38.0800 -0.2618 -0.2867
0.3081 -0.1070 -0.3039 1.1114 0.5550
-0.5393 -0.6127 -37.9244
13.9000 -37.3500 0.2298 -0.0138
0.3478 -0.2326 -0.2986 1.1251 0.5723
-0.5379 -0.6206 -37.9271
13.9200 -37.4700 -0.1874 0.4955
0.3177 -0.3687 -0.2886 1.1378 0.5892
-0.5365 -0.6285 -37.9297
13.9400 -37.5400 -0.4577 0.8423
0.2013 -0.5088 -0.2739 1.1496 0.6058
-0.5350 -0.6363 -37.9324
13.9600 -36.6800 1.0102 0.5358
0.0077 -0.6449 -0.2548 1.1606 0.6218
-0.5335 -0.6441 -37.9350

13.9800	-40.0000	-1.0555	-0.3238	-
0.2139	-0.7688	-0.2313	1.1706	0.6375
	-0.5319	-0.6518	-37.9377	
14.0000	-39.6700	-0.0244	-0.8668	-
0.3963	-0.8722	-0.2035	1.1797	0.6527
	-0.5303	-0.6596	-37.9403	
14.0200	-38.8700	0.8126	-0.8265	-
0.4797	-0.9470	-0.1716	1.1877	0.6674
	-0.5286	-0.6673	-37.9430	
14.0400	-39.3600	-0.2252	-0.3638	-
0.4519	-0.9869	-0.1357	1.1947	0.6816
	-0.5268	-0.6749	-37.9456	
14.0600	-38.8400	-0.3446	0.1522	-
0.3390	-0.9905	-0.0959	1.2006	0.6953
	-0.5250	-0.6826	-37.9483	
14.0800	-37.6400	0.2312	0.5309	-
0.1832	-0.9597	-0.0528	1.2053	0.7085
	-0.5232	-0.6902	-37.9509	
14.1000	-37.6600	-0.1357	0.6428	-
0.0292	-0.8978	-0.0067	1.2088	0.7212
	-0.5213	-0.6978	-37.9536	
14.1200	-37.1500	0.3714	0.3984	
0.0875	-0.8081	0.0417	1.2111	0.7334
	-0.5193	-0.7053	-37.9562	
14.1400	-38.0000	-0.2878	-0.0178	
0.1467	-0.6949	0.0917	1.2121	0.7450
	-0.5173	-0.7129	-37.9588	
14.1600	-37.7600	-0.1083	-0.2467	
0.1496	-0.5627	0.1428	1.2118	0.7561
	-0.5153	-0.7204	-37.9615	
14.1800	-37.2300	0.1563	-0.2111	
0.1258	-0.4170	0.1943	1.2101	0.7667
	-0.5132	-0.7278	-37.9641	
14.2000	-37.3800	-0.1972	-0.1097	
0.1166	-0.2636	0.2455	1.2069	0.7766
	-0.5111	-0.7353	-37.9668	
14.2200	-36.6600	0.4489	-0.1569	
0.1451	-0.1072	0.2960	1.2023	0.7861
	-0.5089	-0.7427	-37.9694	
14.2400	-37.1100	-0.2740	-0.2544	
0.2004	0.0497	0.3449	1.1962	0.7949
	-0.5066	-0.7501	-37.9721	
14.2600	-36.8500	-0.2908	-0.1120	
0.2490	0.2060	0.3916	1.1885	0.8032
	-0.5044	-0.7574	-37.9747	
14.2800	-36.0100	0.0869	0.2313	
0.2537	0.3610	0.4356	1.1792	0.8109
	-0.5020	-0.7647	-37.9774	
14.3000	-35.3100	0.4132	0.3907	
0.1851	0.5137	0.4760	1.1682	0.8181
	-0.4997	-0.7720	-37.9800	

14.3200 -36.0500 -0.1692 0.1722
0.0403 0.6623 0.5123 1.1556 0.8246
-0.4973 -0.7793 -37.9827
14.3400 -36.3300 0.0056 -0.1968 -
0.1421 0.8041 0.5437 1.1413 0.8307
-0.4948 -0.7865 -37.9853
14.3600 -36.8100 -0.3419 -0.3431 -
0.3045 0.9355 0.5696 1.1253 0.8362
-0.4923 -0.7937 -37.9880
14.3800 -35.9300 0.2813 -0.1975 -
0.3939 1.0531 0.5893 1.1075 0.8411
-0.4898 -0.8009 -37.9906
14.4000 -35.9900 -0.0131 0.0033 -
0.3878 1.1538 0.6023 1.0879 0.8455
-0.4872 -0.8081 -37.9933
14.4200 -36.0300 -0.1226 0.0579 -
0.2923 1.2349 0.6079 1.0666 0.8493
-0.4846 -0.8152 -37.9959
14.4400 -35.2600 0.3261 -0.0089 -
0.1349 1.2935 0.6055 1.0435 0.8526
-0.4819 -0.8223 -37.9985
14.4600 -35.8400 -0.3954 -0.0021
0.0465 1.3268 0.5947 1.0187 0.8554
-0.4792 -0.8293 -38.0012
14.4800 -35.0100 0.1224 0.1467
0.2200 1.3319 0.5753 0.9922 0.8577
-0.4765 -0.8364 -38.0038
14.5000 -34.9300 0.1835 0.2334
0.3629 1.3055 0.5477 0.9641 0.8594
-0.4737 -0.8434 -38.0065
14.5200 -34.9500 0.1873 0.1308
0.4652 1.2447 0.5122 0.9346 0.8607
-0.4709 -0.8503 -38.0091
14.5400 -35.8000 -0.3360 -0.0553
0.5283 1.1482 0.4698 0.9035 0.8614
-0.4681 -0.8573 -38.0118
14.5600 -36.1200 -0.3227 -0.2078
0.5554 1.0166 0.4211 0.8712 0.8617
-0.4652 -0.8642 -38.0144
14.5800 -35.7300 0.5181 -0.2869
0.5433 0.8532 0.3672 0.8375 0.8615
-0.4623 -0.8711 -38.0171
14.6000 -36.0900 0.2778 -0.2291
0.4818 0.6633 0.3088 0.8027 0.8608
-0.4593 -0.8779 -38.0197
14.6200 -37.3100 -0.7577 0.0640
0.3606 0.4534 0.2468 0.7668 0.8596
-0.4564 -0.8847 -38.0224
14.6400 -36.1200 0.5969 0.4871
0.1785 0.2305 0.1820 0.7299 0.8580
-0.4534 -0.8915 -38.0250

14.6600 -37.3000 -0.0873 0.5863 -
0.0538 0.0016 0.1154 0.6920 0.8559
-0.4503 -0.8983 -38.0277
14.6800 -38.3700 -0.1718 0.2465 -
0.3082 -0.2262 0.0479 0.6533 0.8534
-0.4473 -0.9050 -38.0303
14.7000 -38.9000 0.3378 -0.2824 -
0.5404 -0.4455 -0.0198 0.6139 0.8504
-0.4442 -0.9117 -38.0329
14.7200 -40.5900 -0.6135 -0.6159 -
0.7028 -0.6494 -0.0868 0.5738 0.8470
-0.4410 -0.9184 -38.0356
14.7400 -39.8200 0.5662 -0.5713 -
0.7557 -0.8307 -0.1522 0.5331 0.8432
-0.4379 -0.9251 -38.0382
14.7600 -40.9200 -0.8386 -0.1381 -
0.6878 -0.9835 -0.2151 0.4919 0.8389
-0.4347 -0.9317 -38.0409
14.7800 -39.3700 0.2879 0.3920 -
0.5182 -1.1035 -0.2747 0.4503 0.8342
-0.4315 -0.9383 -38.0435
14.8000 -38.5000 0.8808 0.5825 -
0.2776 -1.1885 -0.3301 0.4084 0.8291
-0.4282 -0.9448 -38.0462
14.8200 -39.8100 -0.2185 0.2262 -
0.0040 -1.2371 -0.3805 0.3662 0.8236
-0.4249 -0.9514 -38.0488
14.8400 -40.7600 -0.8417 -0.2198
0.2561 -1.2489 -0.4251 0.3238 0.8177
-0.4216 -0.9579 -38.0515
14.8600 -39.5500 0.1936 -0.2281
0.4565 -1.2231 -0.4633 0.2814 0.8114
-0.4183 -0.9643 -38.0541
14.8800 -39.5200 -0.2712 0.1776
0.5631 -1.1596 -0.4949 0.2390 0.8048
-0.4149 -0.9708 -38.0567
14.9000 -38.1800 0.8749 0.3574
0.5544 -1.0595 -0.5198 0.1967 0.7977
-0.4115 -0.9772 -38.0594
14.9200 -39.6000 -0.2118 0.1048
0.4233 -0.9254 -0.5381 0.1545 0.7903
-0.4081 -0.9836 -38.0620
14.9400 -40.3900 -0.5709 -0.1541
0.1926 -0.7633 -0.5498 0.1126 0.7825
-0.4047 -0.9899 -38.0647
14.9600 -40.5100 -0.6189 -0.0144 -
0.0959 -0.5805 -0.5553 0.0711 0.7743
-0.4012 -0.9963 -38.0673
14.9800 -39.0200 0.6078 0.3119 -
0.3766 -0.3850 -0.5547 0.0299 0.7658
-0.3977 -1.0026 -38.0700

15.0000 -39.1300 0.5445 0.3464 -
0.5755 -0.1848 -0.5482 -0.0108 0.7570
-0.3942 -1.0088 -38.0726
15.0200 -40.3800 -0.3018 -0.0391 -
0.6409 0.0126 -0.5359 -0.0510 0.7478
-0.3906 -1.0151 -38.0752
15.0400 -40.7500 -0.4897 -0.5469 -
0.5617 0.2003 -0.5182 -0.0906 0.7382
-0.3871 -1.0213 -38.0779
15.0600 -39.5500 0.6368 -0.7492 -
0.3509 0.3721 -0.4950 -0.1295 0.7284
-0.3835 -1.0275 -38.0805
15.0800 -39.7900 -0.5542 -0.2762 -
0.0497 0.5225 -0.4667 -0.1676 0.7182
-0.3798 -1.0336 -38.0832
15.1000 -38.8900 -0.7677 0.4668
0.2645 0.6457 -0.4335 -0.2050 0.7077
-0.3762 -1.0397 -38.0858
15.1200 -36.2300 1.1675 0.8151
0.5060 0.7362 -0.3954 -0.2415 0.6969
-0.3725 -1.0458 -38.0885
15.1400 -38.3300 -0.6537 0.3627
0.6093 0.7885 -0.3528 -0.2771 0.6859
-0.3688 -1.0519 -38.0911
15.1600 -39.2200 -0.8854 -0.3342
0.5853 0.7997 -0.3059 -0.3117 0.6745
-0.3651 -1.0579 -38.0937
15.1800 -38.0800 0.7523 -0.6518
0.4656 0.7719 -0.2552 -0.3453 0.6628
-0.3614 -1.0640 -38.0964
15.2000 -38.8300 0.0345 -0.4222
0.2843 0.7106 -0.2015 -0.3778 0.6508
-0.3576 -1.0699 -38.0990
15.2200 -38.9500 -0.2870 0.0377
0.0839 0.6227 -0.1457 -0.4091 0.6386
-0.3538 -1.0759 -38.1017
15.2400 -38.3900 0.2358 0.3856 -
0.0923 0.5153 -0.0886 -0.4391 0.6261
-0.3500 -1.0818 -38.1043
15.2600 -39.0900 -0.3062 0.4065 -
0.2137 0.3956 -0.0312 -0.4679 0.6133
-0.3462 -1.0877 -38.1069
15.2800 -38.8900 0.6032 0.0180 -
0.2735 0.2708 0.0256 -0.4954 0.6003
-0.3423 -1.0936 -38.1096
15.3000 -40.1100 -0.1683 -0.4016 -
0.2865 0.1481 0.0808 -0.5215 0.5870
-0.3385 -1.0994 -38.1122
15.3200 -40.8300 -0.9014 -0.3748 -
0.2685 0.0347 0.1336 -0.5462 0.5735
-0.3346 -1.1052 -38.1149

15.3400 -38.6700 0.8201 0.1700 -
0.2315 -0.0631 0.1828 -0.5694 0.5598
-0.3307 -1.1110 -38.1175
15.3600 -38.7700 0.4371 0.5242 -
0.1911 -0.1403 0.2276 -0.5911 0.5458
-0.3268 -1.1168 -38.1201
15.3800 -39.0200 0.5695 0.1572 -
0.1606 -0.1934 0.2670 -0.6112 0.5316
-0.3228 -1.1225 -38.1228
15.4000 -40.7000 -0.5293 -0.4543 -
0.1331 -0.2214 0.3004 -0.6297 0.5172
-0.3189 -1.1282 -38.1254
15.4200 -41.0500 -0.7208 -0.6306 -
0.0961 -0.2256 0.3273 -0.6466 0.5026
-0.3149 -1.1338 -38.1281
15.4400 -39.4600 0.3438 -0.1763 -
0.0437 -0.2087 0.3475 -0.6619 0.4878
-0.3109 -1.1395 -38.1307
15.4600 -38.6100 0.5623 0.4307
0.0133 -0.1737 0.3611 -0.6754 0.4727
-0.3069 -1.1451 -38.1333
15.4800 -39.3800 -0.5272 0.6697
0.0610 -0.1244 0.3682 -0.6874 0.4575
-0.3028 -1.1507 -38.1360
15.5000 -38.7600 0.3403 0.3034
0.0897 -0.0650 0.3689 -0.6976 0.4421
-0.2988 -1.1562 -38.1386
15.5200 -39.0300 0.6810 -0.3039
0.0983 -0.0004 0.3637 -0.7063 0.4265
-0.2947 -1.1618 -38.1413
15.5400 -41.3100 -1.1356 -0.6711
0.0911 0.0644 0.3528 -0.7133 0.4108
-0.2906 -1.1672 -38.1439
15.5600 -40.0400 -0.1464 -0.5144
0.0778 0.1246 0.3366 -0.7188 0.3949
-0.2865 -1.1727 -38.1465
15.5800 -38.3800 1.1036 -0.0951
0.0759 0.1759 0.3154 -0.7227 0.3788
-0.2824 -1.1782 -38.1492
15.6000 -38.1600 1.1312 0.1614
0.0957 0.2153 0.2899 -0.7252 0.3625
-0.2783 -1.1836 -38.1518
15.6200 -40.3100 -1.1488 0.1626
0.1330 0.2408 0.2603 -0.7262 0.3462
-0.2742 -1.1890 -38.1544
15.6400 -40.1000 -0.9914 0.2616
0.1716 0.2515 0.2272 -0.7259 0.3297
-0.2700 -1.1943 -38.1571
15.6600 -39.3500 -0.5545 0.6322
0.1866 0.2471 0.1911 -0.7241 0.3130
-0.2658 -1.1996 -38.1597

15.6800 -37.5400 1.2770 0.7537
0.1457 0.2285 0.1525 -0.7211 0.2962
-0.2617 -1.2049 -38.1623
15.7000 -40.4200 -1.1084 0.4318
0.0321 0.1978 0.1121 -0.7168 0.2794
-0.2575 -1.2102 -38.1650
15.7200 -39.3600 0.4548 0.1937 -
0.1369 0.1579 0.0704 -0.7114 0.2624
-0.2533 -1.2155 -38.1676
15.7400 -39.9400 0.2713 0.1085 -
0.3191 0.1124 0.0280 -0.7047 0.2453
-0.2490 -1.2207 -38.1703
15.7600 -40.5900 0.1009 -0.1891 -
0.4521 0.0651 -0.0143 -0.6970 0.2281
-0.2448 -1.2259 -38.1729
15.7800 -41.1200 0.1388 -0.6998 -
0.4917 0.0196 -0.0561 -0.6882 0.2109
-0.2406 -1.2310 -38.1755
15.8000 -42.2600 -0.5359 -1.0393 -
0.4163 -0.0209 -0.0966 -0.6784 0.1936
-0.2363 -1.2362 -38.1782
15.8200 -40.9000 0.6335 -0.9006 -
0.2252 -0.0554 -0.1351 -0.6677 0.1762
-0.2320 -1.2413 -38.1808
15.8400 -41.1800 -0.5816 -0.3049
0.0368 -0.0843 -0.1709 -0.6561 0.1588
-0.2278 -1.2463 -38.1834
15.8600 -39.4900 0.1792 0.5154
0.3031 -0.1082 -0.2035 -0.6437 0.1414
-0.2235 -1.2514 -38.1861
15.8800 -38.5600 0.3484 1.0773
0.5059 -0.1277 -0.2322 -0.6304 0.1240
-0.2192 -1.2564 -38.1887
15.9000 -38.8700 0.0785 0.9438
0.5923 -0.1432 -0.2564 -0.6165 0.1065
-0.2149 -1.2614 -38.1913
15.9200 -39.6700 0.2201 0.1644
0.5582 -0.1548 -0.2756 -0.6019 0.0891
-0.2106 -1.2664 -38.1940
15.9400 -41.4400 -0.5942 -0.6549
0.4311 -0.1626 -0.2893 -0.5867 0.0717
-0.2062 -1.2713 -38.1966
15.9600 -41.0700 0.2608 -0.8935
0.2442 -0.1675 -0.2974 -0.5709 0.0543
-0.2019 -1.2762 -38.1992
15.9800 -41.3400 -0.1870 -0.4570
0.0308 -0.1698 -0.2996 -0.5546 0.0369
-0.1976 -1.2811 -38.2019
16.0000 -41.0200 -0.4666 0.2440 -
0.1757 -0.1690 -0.2961 -0.5379 0.0197
-0.1932 -1.2860 -38.2045

16.0200 -39.4600 0.8985 0.6583 -
0.3446 -0.1630 -0.2868 -0.5208 0.0025
-0.1889 -1.2908 -38.2071
16.0400 -41.0500 -0.5359 0.5487 -
0.4575 -0.1498 -0.2721 -0.5034 -0.0147
-0.1845 -1.2956 -38.2097
16.0600 -41.6300 -0.6892 0.2394 -
0.5180 -0.1280 -0.2523 -0.4857 -0.0317
-0.1801 -1.3004 -38.2124
16.0800 -40.6300 0.5260 -0.0270 -
0.5236 -0.0978 -0.2279 -0.4678 -0.0486
-0.1758 -1.3051 -38.2150
16.1000 -40.8100 0.3803 -0.3032 -
0.4500 -0.0611 -0.1995 -0.4498 -0.0654
-0.1714 -1.3099 -38.2176
16.1200 -41.3800 -0.0627 -0.5980 -
0.2854 -0.0205 -0.1677 -0.4317 -0.0820
-0.1670 -1.3146 -38.2203
16.1400 -41.4700 -0.4528 -0.6115 -
0.0467 0.0209 -0.1334 -0.4136 -0.0985
-0.1626 -1.3192 -38.2229
16.1600 -40.4800 -0.1384 -0.1771
0.2247 0.0594 -0.0971 -0.3956 -0.1148
-0.1582 -1.3239 -38.2255
16.1800 -39.1800 0.0800 0.4523
0.4621 0.0914 -0.0596 -0.3777 -0.1309
-0.1538 -1.3285 -38.2281
16.2000 -38.4500 0.2724 0.7874
0.5922 0.1130 -0.0217 -0.3599 -0.1469
-0.1494 -1.3331 -38.2308
16.2200 -38.2800 0.6993 0.5546
0.5637 0.1214 0.0161 -0.3425 -0.1626
-0.1450 -1.3376 -38.2334
16.2400 -40.1800 -0.4874 -0.0118
0.4003 0.1171 0.0529 -0.3253 -0.1781
-0.1406 -1.3422 -38.2360
16.2600 -40.9700 -0.6664 -0.3908
0.1557 0.1036 0.0882 -0.3085 -0.1933
-0.1362 -1.3467 -38.2387
16.2800 -39.9800 0.4843 -0.3546 -
0.1081 0.0853 0.1213 -0.2921 -0.2083
-0.1318 -1.3511 -38.2413
16.3000 -40.4000 0.1504 -0.1899 -
0.3151 0.0663 0.1519 -0.2762 -0.2231
-0.1273 -1.3556 -38.2439
16.3200 -40.3200 0.3330 -0.2408 -
0.4010 0.0509 0.1793 -0.2609 -0.2375
-0.1229 -1.3600 -38.2465
16.3400 -40.9100 -0.2181 -0.3411 -
0.3638 0.0420 0.2031 -0.2462 -0.2517
-0.1185 -1.3644 -38.2492

16.3600 -40.7000 -0.2876 -0.1692 -
0.2407 0.0414 0.2230 -0.2321 -0.2655
-0.1141 -1.3688 -38.2518
16.3800 -40.1500 -0.4510 0.3137 -
0.0884 0.0499 0.2388 -0.2188 -0.2791
-0.1096 -1.3731 -38.2544
16.4000 -38.5700 0.7930 0.5333
0.0375 0.0673 0.2503 -0.2063 -0.2923
-0.1052 -1.3774 -38.2570
16.4200 -40.4900 -0.7021 0.0596
0.0970 0.0924 0.2575 -0.1945 -0.3052
-0.1008 -1.3817 -38.2597
16.4400 -39.8100 0.2794 -0.4007
0.1030 0.1220 0.2607 -0.1837 -0.3177
-0.0964 -1.3860 -38.2623
16.4600 -40.0300 0.0654 -0.2635
0.0839 0.1520 0.2601 -0.1737 -0.3298
-0.0919 -1.3902 -38.2649
16.4800 -40.1400 -0.5342 0.2214
0.0600 0.1784 0.2560 -0.1646 -0.3416
-0.0875 -1.3944 -38.2675
16.5000 -38.5900 0.9590 0.3118
0.0472 0.1977 0.2487 -0.1565 -0.3530
-0.0831 -1.3986 -38.2702
16.5200 -40.8200 -1.0614 -0.0012
0.0484 0.2072 0.2386 -0.1493 -0.3640
-0.0787 -1.4028 -38.2728
16.5400 -39.2800 0.6538 -0.1326
0.0556 0.2052 0.2263 -0.1430 -0.3746
-0.0742 -1.4069 -38.2754
16.5600 -39.1500 0.8554 -0.1406
0.0691 0.1913 0.2120 -0.1376 -0.3848
-0.0698 -1.4110 -38.2780
16.5800 -40.4300 -0.3378 -0.1869
0.0923 0.1660 0.1962 -0.1331 -0.3946
-0.0654 -1.4151 -38.2806
16.6000 -40.6100 -0.6091 -0.1749
0.1126 0.1306 0.1794 -0.1295 -0.4040
-0.0610 -1.4192 -38.2833
16.6200 -40.2900 -0.2999 0.0640
0.1105 0.0871 0.1620 -0.1268 -0.4129
-0.0566 -1.4232 -38.2859
16.6400 -39.9300 -0.2565 0.4284
0.0694 0.0372 0.1443 -0.1250 -0.4214
-0.0522 -1.4272 -38.2885
16.6600 -38.9700 0.7180 0.4885 -
0.0150 -0.0173 0.1266 -0.1240 -0.4295
-0.0477 -1.4312 -38.2911
16.6800 -40.2900 0.1612 0.0337 -
0.1264 -0.0749 0.1093 -0.1239 -0.4372
-0.0433 -1.4351 -38.2937

16.7000 -41.8300 -0.7188 -0.4869 -
0.2190 -0.1339 0.0927 -0.1246 -0.4444
-0.0389 -1.4390 -38.2964
16.7200 -40.6800 0.5162 -0.5166 -
0.2444 -0.1922 0.0768 -0.1260 -0.4511
-0.0346 -1.4429 -38.2990
16.7400 -41.0900 -0.3263 -0.0713 -
0.1817 -0.2475 0.0621 -0.1283 -0.4574
-0.0302 -1.4468 -38.3016
16.7600 -40.5500 -0.1727 0.3096 -
0.0519 -0.2969 0.0485 -0.1313 -0.4633
-0.0258 -1.4506 -38.3042
16.7800 -39.6900 0.6441 0.2946
0.1074 -0.3376 0.0363 -0.1349 -0.4687
-0.0214 -1.4545 -38.3068
16.8000 -41.0100 -0.5942 -0.0318
0.2460 -0.3666 0.0255 -0.1392 -0.4737
-0.0170 -1.4583 -38.3095
16.8200 -40.3600 0.2369 -0.1303
0.3131 -0.3817 0.0162 -0.1442 -0.4783
-0.0127 -1.4620 -38.3121
16.8400 -40.2900 0.0573 0.0759
0.2786 -0.3808 0.0084 -0.1497 -0.4824
-0.0083 -1.4658 -38.3147
16.8600 -40.4800 -0.1621 0.2729
0.1499 -0.3630 0.0020 -0.1557 -0.4861
-0.0040 -1.4695 -38.3173
16.8800 -40.2400 0.4169 0.2040 -
0.0436 -0.3279 -0.0032 -0.1622 -0.4894
0.0004 -1.4732 -38.3199
16.9000 -41.5100 -0.5063 -0.0371 -
0.2577 -0.2764 -0.0073 -0.1692 -0.4922
0.0047 -1.4768 -38.3225
16.9200 -41.2000 0.2081 -0.2180 -
0.4364 -0.2091 -0.0106 -0.1764 -0.4946
0.0091 -1.4805 -38.3251
16.9400 -40.8600 0.5686 -0.2893 -
0.5263 -0.1272 -0.0132 -0.1840 -0.4966
0.0134 -1.4841 -38.3278
16.9600 -42.2000 -0.9272 -0.2575 -
0.4996 -0.0328 -0.0154 -0.1918 -0.4982
0.0177 -1.4877 -38.3304
16.9800 -40.4600 0.5690 -0.2148 -
0.3556 0.0694 -0.0174 -0.1998 -0.4994
0.0220 -1.4912 -38.3330
17.0000 -39.7700 0.9494 -0.1832 -
0.1222 0.1730 -0.0193 -0.2080 -0.5001
0.0263 -1.4948 -38.3356
17.0200 -41.0900 -0.9775 -0.0762
0.1457 0.2711 -0.0214 -0.2162 -0.5005
0.0306 -1.4983 -38.3382

17.0400 -40.2300 -0.6657 0.1879
0.3861 0.3566 -0.0236 -0.2244 -0.5004
0.0349 -1.5018 -38.3408
17.0600 -38.2100 0.9924 0.4482
0.5448 0.4228 -0.0264 -0.2325 -0.5000
0.0391 -1.5052 -38.3434
17.0800 -39.7900 -0.6267 0.4175
0.5835 0.4631 -0.0297 -0.2405 -0.4992
0.0434 -1.5087 -38.3460
17.1000 -39.6900 -0.1184 0.1241
0.5015 0.4741 -0.0338 -0.2484 -0.4979
0.0477 -1.5121 -38.3486
17.1200 -39.5600 0.5526 -0.2038
0.3329 0.4570 -0.0386 -0.2561 -0.4963
0.0519 -1.5155 -38.3513
17.1400 -40.3500 -0.0098 -0.3591
0.1226 0.4148 -0.0440 -0.2634 -0.4943
0.0561 -1.5188 -38.3539
17.1600 -41.3700 -0.7537 -0.2370 -
0.0874 0.3511 -0.0499 -0.2705 -0.4920
0.0603 -1.5221 -38.3565
17.1800 -40.3400 0.1439 0.0585 -
0.2559 0.2697 -0.0562 -0.2772 -0.4892
0.0645 -1.5255 -38.3591
17.2000 -39.7700 0.9078 0.1800 -
0.3511 0.1745 -0.0626 -0.2835 -0.4862
0.0687 -1.5287 -38.3617
17.2200 -41.8600 -0.8446 -0.0321 -
0.3625 0.0698 -0.0690 -0.2894 -0.4827
0.0729 -1.5320 -38.3643
17.2400 -41.3700 -0.2362 -0.1391 -
0.2981 -0.0393 -0.0753 -0.2948 -0.4789
0.0771 -1.5352 -38.3669
17.2600 -41.2200 -0.2781 0.0900 -
0.1749 -0.1468 -0.0813 -0.2997 -0.4747
0.0813 -1.5385 -38.3695
17.2800 -39.9900 0.6953 0.2311 -
0.0262 -0.2460 -0.0867 -0.3042 -0.4702
0.0854 -1.5416 -38.3721
17.3000 -40.5300 0.3865 0.0459
0.1079 -0.3303 -0.0914 -0.3080 -0.4654
0.0895 -1.5448 -38.3747
17.3200 -41.9200 -0.8276 -0.2332
0.1952 -0.3936 -0.0952 -0.3113 -0.4602
0.0936 -1.5479 -38.3773
17.3400 -40.8300 0.3637 -0.2252
0.2108 -0.4318 -0.0980 -0.3140 -0.4547
0.0977 -1.5510 -38.3799
17.3600 -40.3700 0.5210 0.0568
0.1472 -0.4431 -0.0996 -0.3160 -0.4489
0.1018 -1.5541 -38.3825

17.3800 -41.6700 -0.7648 0.2830
0.0198 -0.4281 -0.1000 -0.3174 -0.4428
0.1059 -1.5572 -38.3851

17.4000 -40.2400 0.8165 0.1168 -
0.1377 -0.3883 -0.0991 -0.3182 -0.4364
0.1099 -1.5602 -38.3877

17.4200 -42.3200 -0.8178 -0.1715 -
0.2719 -0.3268 -0.0971 -0.3183 -0.4297
0.1140 -1.5633 -38.3903

17.4400 -40.6600 0.7754 -0.1657 -
0.3227 -0.2470 -0.0942 -0.3178 -0.4227
0.1180 -1.5662 -38.3929

17.4600 -41.8800 -0.6800 -0.0823 -
0.2617 -0.1543 -0.0903 -0.3166 -0.4154
0.1220 -1.5692 -38.3955

17.4800 -40.4400 0.4573 -0.0785 -
0.1086 -0.0550 -0.0857 -0.3148 -0.4078
0.1260 -1.5722 -38.3981

17.5000 -40.1700 0.2902 0.0292
0.0775 0.0446 -0.0805 -0.3123 -0.4000
0.1300 -1.5751 -38.4007

17.5200 -40.7100 -0.7139 0.2505
0.2333 0.1378 -0.0747 -0.3092 -0.3919
0.1339 -1.5780 -38.4033

17.5400 -39.0800 0.7293 0.3352
0.3175 0.2181 -0.0685 -0.3055 -0.3836
0.1378 -1.5808 -38.4059

17.5600 -40.4900 -0.4583 0.0348
0.3205 0.2803 -0.0619 -0.3011 -0.3751
0.1417 -1.5837 -38.4085

17.5800 -40.5100 -0.1397 -0.3258
0.2630 0.3217 -0.0551 -0.2962 -0.3663
0.1456 -1.5865 -38.4111

17.6000 -40.2600 0.1605 -0.3832
0.1683 0.3418 -0.0481 -0.2908 -0.3573
0.1495 -1.5893 -38.4137

17.6200 -40.5800 -0.3797 -0.0755
0.0548 0.3422 -0.0412 -0.2848 -0.3481
0.1534 -1.5921 -38.4163

17.6400 -39.5200 0.4736 0.3072 -
0.0640 0.3254 -0.0345 -0.2783 -0.3387
0.1572 -1.5948 -38.4189

17.6600 -40.3300 -0.3938 0.4505 -
0.1766 0.2944 -0.0279 -0.2713 -0.3292
0.1610 -1.5976 -38.4215

17.6800 -39.9900 0.2043 0.2694 -
0.2727 0.2524 -0.0218 -0.2638 -0.3194
0.1648 -1.6003 -38.4241

17.7000 -40.1200 0.5206 -0.1065 -
0.3356 0.2026 -0.0160 -0.2559 -0.3095
0.1685 -1.6029 -38.4267

17.7200 -41.2300 -0.3169 -0.4293 -
0.3409 0.1480 -0.0108 -0.2476 -0.2994
0.1723 -1.6056 -38.4293
17.7400 -41.5400 -0.5716 -0.3927 -
0.2712 0.0913 -0.0061 -0.2389 -0.2891
0.1760 -1.6082 -38.4319
17.7600 -39.9400 0.4934 -0.0214 -
0.1317 0.0349 -0.0019 -0.2299 -0.2788
0.1797 -1.6109 -38.4345
17.7800 -39.7700 0.3309 0.2464
0.0491 -0.0196 0.0019 -0.2205 -0.2682
0.1834 -1.6134 -38.4371
17.8000 -39.9400 0.0648 0.1669
0.2302 -0.0707 0.0054 -0.2109 -0.2576
0.1870 -1.6160 -38.4397
17.8200 -40.6400 -0.5407 0.0263
0.3640 -0.1171 0.0088 -0.2010 -0.2468
0.1906 -1.6185 -38.4423
17.8400 -39.6700 0.3016 0.1028
0.4142 -0.1570 0.0121 -0.1909 -0.2360
0.1942 -1.6211 -38.4449
17.8600 -40.0500 -0.1571 0.1659
0.3703 -0.1887 0.0156 -0.1807 -0.2250
0.1978 -1.6236 -38.4475
17.8800 -39.5900 0.7241 -0.1180
0.2484 -0.2112 0.0193 -0.1703 -0.2139
0.2014 -1.6260 -38.4500
17.9000 -41.6400 -0.9482 -0.2760
0.0843 -0.2238 0.0234 -0.1598 -0.2028
0.2049 -1.6285 -38.4526
17.9200 -39.6000 0.9882 0.0313 -
0.0833 -0.2261 0.0278 -0.1492 -0.1916
0.2084 -1.6309 -38.4552
17.9400 -40.9700 -0.6516 0.2514 -
0.2312 -0.2176 0.0327 -0.1386 -0.1803
0.2118 -1.6333 -38.4578
17.9600 -40.8600 -0.3895 0.1634 -
0.3466 -0.1976 0.0379 -0.1279 -0.1690
0.2153 -1.6357 -38.4604
17.9800 -40.2600 0.5302 -0.1176 -
0.4208 -0.1664 0.0437 -0.1173 -0.1576
0.2187 -1.6381 -38.4630
18.0000 -40.7800 0.2665 -0.3258 -
0.4444 -0.1251 0.0498 -0.1068 -0.1462
0.2221 -1.6404 -38.4656
18.0200 -41.3000 -0.5541 -0.2723 -
0.4103 -0.0762 0.0562 -0.0963 -0.1348
0.2254 -1.6427 -38.4682
18.0400 -40.7000 -0.2926 -0.0072 -
0.3127 -0.0230 0.0628 -0.0859 -0.1233
0.2287 -1.6450 -38.4707

18.0600 -39.2400 0.7662 0.1348 -
0.1515 0.0309 0.0693 -0.0756 -0.1119
0.2320 -1.6473 -38.4733
18.0800 -40.4700 -0.5817 -0.0481
0.0574 0.0816 0.0756 -0.0655 -0.1004
0.2353 -1.6495 -38.4759
18.1000 -39.9000 -0.2080 -0.1507
0.2693 0.1253 0.0815 -0.0556 -0.0889
0.2385 -1.6517 -38.4785
18.1200 -39.1100 0.1472 0.0780
0.4374 0.1583 0.0868 -0.0459 -0.0775
0.2418 -1.6540 -38.4811
18.1400 -38.9100 -0.1666 0.4589
0.5243 0.1769 0.0913 -0.0364 -0.0661
0.2449 -1.6561 -38.4837
18.1600 -38.5200 0.1465 0.5986
0.5035 0.1784 0.0947 -0.0271 -0.0547
0.2481 -1.6583 -38.4862
18.1800 -38.3400 0.6279 0.3297
0.3729 0.1631 0.0970 -0.0180 -0.0433
0.2512 -1.6604 -38.4888
18.2000 -40.3900 -0.7831 -0.1055
0.1697 0.1346 0.0979 -0.0092 -0.0320
0.2543 -1.6625 -38.4914
18.2200 -40.2300 -0.0448 -0.3044 -
0.0576 0.0973 0.0975 -0.0007 -0.0207
0.2573 -1.6646 -38.4940
18.2400 -39.5200 0.7475 -0.3141 -
0.2563 0.0558 0.0956 0.0076 -0.0095
0.2603 -1.6667 -38.4966
18.2600 -40.8600 -0.3509 -0.3568 -
0.3793 0.0144 0.0923 0.0156 0.0016
0.2633 -1.6688 -38.4991
18.2800 -41.0000 -0.3599 -0.4370 -
0.4021 -0.0227 0.0878 0.0234 0.0127
0.2663 -1.6708 -38.5017
18.3000 -40.0300 0.4578 -0.4027 -
0.3280 -0.0528 0.0819 0.0309 0.0236
0.2692 -1.6728 -38.5043
18.3200 -40.6400 -0.3726 -0.1406 -
0.1860 -0.0745 0.0747 0.0382 0.0345
0.2720 -1.6748 -38.5069
18.3400 -39.7000 -0.0996 0.2387 -
0.0239 -0.0871 0.0665 0.0452 0.0453
0.2749 -1.6768 -38.5094
18.3600 -39.1500 0.1208 0.4793
0.1088 -0.0902 0.0572 0.0519 0.0559
0.2777 -1.6787 -38.5120
18.3800 -38.7700 0.4995 0.3419
0.1728 -0.0840 0.0471 0.0584 0.0665
0.2805 -1.6806 -38.5146

18.4000 −40.3800 −0.6090 −0.0321
0.1599 −0.0697 0.0362 0.0647 0.0769
0.2832 −1.6825 −38.5172
18.4200 −39.8700 −0.0280 −0.1721
0.0863 −0.0501 0.0248 0.0708 0.0872
0.2859 −1.6844 −38.5197
18.4400 −39.4400 0.3272 −0.0475 −
0.0068 −0.0288 0.0131 0.0767 0.0974
0.2886 −1.6863 −38.5223
18.4600 −39.6700 0.0692 0.0315 −
0.0625 −0.0089 0.0012 0.0823 0.1075
0.2912 −1.6881 −38.5249
18.4800 −40.3900 −0.5210 −0.0815 −
0.0498 0.0072 −0.0107 0.0878 0.1174
0.2938 −1.6899 −38.5274
18.5000 −39.1500 0.8742 −0.2658
0.0079 0.0194 −0.0224 0.0930 0.1271
0.2963 −1.6917 −38.5300
18.5200 −40.6700 −1.0063 −0.0522
0.0585 0.0279 −0.0336 0.0981 0.1367
0.2989 −1.6935 −38.5326
18.5400 −38.2600 1.0272 0.4656
0.0556 0.0336 −0.0441 0.1030 0.1461
0.3013 −1.6953 −38.5352
18.5600 −39.9400 −0.5020 0.2250 −
0.0081 0.0373 −0.0539 0.1078 0.1554
0.3038 −1.6970 −38.5377
18.5800 −39.9800 0.2764 −0.5116 −
0.0768 0.0401 −0.0629 0.1124 0.1645
0.3062 −1.6987 −38.5403
18.6000 −41.1800 −1.1262 −0.3272 −
0.0893 0.0431 −0.0710 0.1169 0.1734
0.3085 −1.7004 −38.5429
18.6200 −38.2100 1.2102 0.3901 −
0.0465 0.0463 −0.0781 0.1212 0.1821
0.3108 −1.7021 −38.5454
18.6400 −40.3700 −1.0828 0.3799
0.0188 0.0483 −0.0843 0.1255 0.1906
0.3131 −1.7037 −38.5480
18.6600 −39.5500 −0.0333 −0.0626
0.0691 0.0478 −0.0896 0.1297 0.1990
0.3154 −1.7054 −38.5505
18.6800 −39.2600 0.5529 −0.2466
0.0762 0.0436 −0.0938 0.1338 0.2071
0.3175 −1.7070 −38.5531
18.7000 −40.1200 −0.4939 0.0399
0.0377 0.0349 −0.0968 0.1378 0.2151
0.3197 −1.7086 −38.5557
18.7200 −38.7500 0.6816 0.2193 −
0.0113 0.0220 −0.0987 0.1418 0.2228
0.3218 −1.7102 −38.5582

18.7400 -40.2400 -0.4093 -0.1295 -
0.0328 0.0050 -0.0992 0.1457 0.2304
0.3239 -1.7117 -38.5608
18.7600 -40.0200 0.0972 -0.4494 -
0.0226 -0.0143 -0.0983 0.1495 0.2377
0.3259 -1.7132 -38.5634
18.7800 -40.5900 -0.8862 -0.0586
0.0063 -0.0338 -0.0959 0.1533 0.2448
0.3279 -1.7148 -38.5659
18.8000 -37.9500 1.1570 0.5067
0.0243 -0.0509 -0.0919 0.1570 0.2517
0.3298 -1.7163 -38.5685
18.8200 -39.7600 -0.5601 0.4804
0.0062 -0.0636 -0.0863 0.1606 0.2583
0.3317 -1.7177 -38.5710
18.8400 -40.8200 -1.0897 0.0459 -
0.0442 -0.0708 -0.0790 0.1641 0.2647
0.3336 -1.7192 -38.5736
18.8600 -39.0100 1.1234 -0.3962 -
0.0938 -0.0728 -0.0699 0.1675 0.2709
0.3354 -1.7206 -38.5762
18.8800 -41.0800 -0.8421 -0.4932 -
0.1038 -0.0707 -0.0591 0.1708 0.2769
0.3371 -1.7220 -38.5787
18.9000 -40.0200 -0.1831 -0.2238 -
0.0548 -0.0659 -0.0466 0.1740 0.2826
0.3389 -1.7234 -38.5813
18.9200 -38.6900 0.6142 0.1424
0.0360 -0.0604 -0.0325 0.1770 0.2881
0.3405 -1.7248 -38.5838
18.9400 -39.3200 -0.2226 0.2560
0.1368 -0.0558 -0.0168 0.1799 0.2934
0.3422 -1.7262 -38.5864
18.9600 -38.9500 0.2721 0.1438
0.2006 -0.0534 0.0003 0.1826 0.2984
0.3438 -1.7275 -38.5889
18.9800 -39.4000 -0.3244 0.1550
0.1850 -0.0536 0.0185 0.1851 0.3031
0.3453 -1.7289 -38.5915
19.0000 -39.4200 -0.2723 0.2741
0.0902 -0.0553 0.0376 0.1873 0.3076
0.3468 -1.7302 -38.5940
19.0200 -38.7200 0.6384 0.2026 -
0.0434 -0.0563 0.0572 0.1894 0.3119
0.3482 -1.7314 -38.5966
19.0400 -40.5100 -0.6596 -0.2487 -
0.1564 -0.0545 0.0771 0.1912 0.3159
0.3496 -1.7327 -38.5991
19.0600 -39.5900 0.4657 -0.4487 -
0.1974 -0.0478 0.0967 0.1927 0.3196
0.3510 -1.7340 -38.6017

19.0800	-40.2300	-0.6680	-0.0354	-
0.1512	-0.0350	0.1159	0.1939	0.3231
	0.3523	-1.7352	-38.6042	
19.1000	-38.3000	0.8164	0.2874	-
0.0559	-0.0157	0.1340	0.1949	0.3263
	0.3535	-1.7364	-38.6068	
19.1200	-39.7600	-0.8018	0.1763	
0.0335	0.0095	0.1508	0.1954	0.3293
	0.3547	-1.7376	-38.6093	
19.1400	-38.9900	0.1123	0.0530	
0.0741	0.0400	0.1659	0.1957	0.3320
	0.3559	-1.7388	-38.6119	
19.1600	-38.4000	0.6933	0.0679	
0.0494	0.0740	0.1788	0.1956	0.3344
	0.3570	-1.7399	-38.6144	
19.1800	-38.9600	0.0795	0.1155	-
0.0255	0.1087	0.1892	0.1951	0.3366
	0.3580	-1.7411	-38.6170	
19.2000	-39.9000	-0.6889	0.0830	-
0.1076	0.1406	0.1968	0.1942	0.3386
	0.3590	-1.7422	-38.6195	
19.2200	-38.6400	0.6652	-0.0714	-
0.1507	0.1662	0.2014	0.1929	0.3402
	0.3600	-1.7433	-38.6221	
19.2400	-39.7600	-0.3083	-0.2894	-
0.1257	0.1822	0.2028	0.1912	0.3416
	0.3609	-1.7444	-38.6246	
19.2600	-39.6900	-0.2803	-0.3500	-
0.0313	0.1865	0.2007	0.1892	0.3428
	0.3617	-1.7455	-38.6271	
19.2800	-39.0900	-0.0554	-0.1106	
0.1089	0.1786	0.1953	0.1867	0.3437
	0.3625	-1.7465	-38.6297	
19.3000	-38.3900	0.3088	0.2177	
0.2511	0.1592	0.1865	0.1839	0.3443
	0.3633	-1.7476	-38.6322	
19.3200	-38.2000	0.2130	0.3751	
0.3522	0.1294	0.1749	0.1807	0.3447
	0.3640	-1.7486	-38.6348	
19.3400	-38.8100	-0.2491	0.3369	
0.3802	0.0908	0.1606	0.1772	0.3449
	0.3647	-1.7496	-38.6373	
19.3600	-38.8900	-0.1025	0.1835	
0.3278	0.0460	0.1440	0.1735	0.3448
	0.3653	-1.7506	-38.6398	
19.3800	-38.6300	0.5293	-0.0583	
0.2129	-0.0019	0.1257	0.1694	0.3444
	0.3658	-1.7516	-38.6424	
19.4000	-39.8600	-0.1203	-0.3284	
0.0625	-0.0501	0.1061	0.1652	0.3438
	0.3663	-1.7525	-38.6449	

19.4200 -40.4300 -0.4270 -0.4190 -
0.0975 -0.0957 0.0855 0.1607 0.3430
0.3668 -1.7534 -38.6475
19.4400 -40.2400 -0.2925 -0.1430 -
0.2428 -0.1359 0.0645 0.1561 0.3419
0.3672 -1.7544 -38.6500
19.4600 -39.2100 0.6488 0.2446 -
0.3535 -0.1683 0.0436 0.1513 0.3406
0.3675 -1.7553 -38.6525
19.4800 -39.8600 0.0008 0.2758 -
0.4145 -0.1903 0.0231 0.1464 0.3391
0.3678 -1.7562 -38.6551
19.5000 -40.1400 0.0986 -0.0569 -
0.4172 -0.2004 0.0036 0.1414 0.3373
0.3681 -1.7570 -38.6576
19.5200 -41.0200 -0.6644 -0.2786 -
0.3552 -0.1983 -0.0147 0.1363 0.3352
0.3683 -1.7579 -38.6601
19.5400 -40.1400 0.1021 -0.1875 -
0.2294 -0.1860 -0.0315 0.1312 0.3330
0.3685 -1.7587 -38.6627
19.5600 -39.2200 0.6589 0.0413 -
0.0570 -0.1661 -0.0467 0.1261 0.3305
0.3686 -1.7595 -38.6652
19.5800 -40.3900 -0.8087 0.1657
0.1354 -0.1416 -0.0601 0.1211 0.3278
0.3686 -1.7604 -38.6677
19.6000 -38.8700 0.5110 0.1040
0.3109 -0.1151 -0.0717 0.1161 0.3249
0.3686 -1.7611 -38.6702
19.6200 -38.8800 0.4659 -0.0281
0.4321 -0.0896 -0.0813 0.1112 0.3218
0.3686 -1.7619 -38.6728
19.6400 -40.1400 -0.7466 -0.0183
0.4692 -0.0678 -0.0890 0.1065 0.3185
0.3685 -1.7627 -38.6753
19.6600 -38.6900 0.6122 0.2102
0.4111 -0.0512 -0.0947 0.1020 0.3149
0.3684 -1.7634 -38.6778
19.6800 -39.4800 -0.3377 0.3286
0.2697 -0.0400 -0.0986 0.0977 0.3111
0.3682 -1.7642 -38.6803
19.7000 -39.2500 0.2265 0.1778
0.0761 -0.0333 -0.1008 0.0937 0.3072
0.3679 -1.7649 -38.6829
19.7200 -40.3300 -0.2430 -0.0580 -
0.1254 -0.0298 -0.1016 0.0901 0.3030
0.3676 -1.7656 -38.6854
19.7400 -40.2500 0.0886 -0.2746 -
0.2869 -0.0281 -0.1011 0.0868 0.2987
0.3673 -1.7663 -38.6879

19.7600	−40.3700	0.2885	−0.4550	−
0.3693	−0.0268	−0.0998	0.0839	0.2941
	0.3669	−1.7669	−38.6904	
19.7800	−41.2300	−0.4780	−0.4730	−
0.3555	−0.0247	−0.0979	0.0815	0.2894
	0.3665	−1.7676	−38.6930	
19.8000	−40.8900	−0.4873	−0.2488	−
0.2586	−0.0207	−0.0960	0.0795	0.2844
	0.3660	−1.7682	−38.6955	
19.8200	−38.9700	0.9443	0.0850	−
0.1145	−0.0137	−0.0943	0.0782	0.2793
	0.3655	−1.7689	−38.6980	
19.8400	−39.9400	−0.4149	0.3164	
0.0332	−0.0030	−0.0934	0.0774	0.2740
	0.3649	−1.7695	−38.7005	
19.8600	−40.2200	−0.8494	0.4420	
0.1426	0.0122	−0.0935	0.0772	0.2686
	0.3643	−1.7701	−38.7030	
19.8800	−38.1900	0.9340	0.4701	
0.1908	0.0322	−0.0950	0.0777	0.2629
	0.3636	−1.7706	−38.7055	
19.9000	−40.1500	−0.8928	0.3083	
0.1808	0.0569	−0.0981	0.0788	0.2571
	0.3629	−1.7712	−38.7081	
19.9200	−39.5600	0.1848	−0.0682	
0.1299	0.0853	−0.1028	0.0806	0.2511
	0.3621	−1.7718	−38.7106	
19.9400	−39.5100	0.7912	−0.4483	
0.0658	0.1163	−0.1090	0.0831	0.2450
	0.3613	−1.7723	−38.7131	
19.9600	−40.9300	−0.4992	−0.6025	
0.0126	0.1487	−0.1169	0.0862	0.2387
	0.3605	−1.7728	−38.7156	
19.9800	−40.9100	−0.6736	−0.3582	−
0.0179	0.1810	−0.1261	0.0900	0.2323
	0.3596	−1.7733	−38.7181	
20.0000	−39.1300	0.4133	0.2009	−
0.0281	0.2123	−0.1366	0.0943	0.2258
	0.3586	−1.7738	−38.7206	
20.0200	−38.8700	0.3334	0.5635	−
0.0284	0.2413	−0.1482	0.0992	0.2191
	0.3576	−1.7743	−38.7231	
20.0400	−39.0800	0.3017	0.3499	−
0.0285	0.2666	−0.1605	0.1045	0.2124
	0.3566	−1.7748	−38.7256	
20.0600	−40.4900	−0.5893	−0.1384	−
0.0303	0.2867	−0.1733	0.1104	0.2055
	0.3555	−1.7753	−38.7282	
20.0800	−39.7100	0.5122	−0.4460	−
0.0321	0.2997	−0.1862	0.1165	0.1985
	0.3543	−1.7757	−38.7307	

20.1000 -40.6700 -0.5570 -0.3383 -
0.0297 0.3037 -0.1987 0.1231 0.1914
0.3531 -1.7761 -38.7332
20.1200 -39.6500 0.0039 0.0630 -
0.0194 0.2967 -0.2106 0.1299 0.1842
0.3519 -1.7765 -38.7357
20.1400 -38.8900 0.5679 0.3965
0.0043 0.2771 -0.2214 0.1369 0.1770
0.3506 -1.7770 -38.7382
20.1600 -39.6500 -0.2064 0.3963
0.0454 0.2436 -0.2307 0.1441 0.1697
0.3493 -1.7773 -38.7407
20.1800 -40.2300 -0.4511 0.0705
0.1022 0.1960 -0.2381 0.1514 0.1623
0.3479 -1.7777 -38.7432
20.2000 -39.4500 0.6994 -0.3579
0.1625 0.1349 -0.2435 0.1587 0.1549
0.3465 -1.7781 -38.7457
20.2200 -41.0000 -0.6637 -0.4879
0.2084 0.0615 -0.2463 0.1660 0.1475
0.3450 -1.7784 -38.7482
20.2400 -39.5700 0.5047 -0.0973
0.2207 -0.0220 -0.2465 0.1733 0.1401
0.3435 -1.7788 -38.7507
20.2600 -40.0400 -0.3182 0.4122
0.1866 -0.1122 -0.2437 0.1804 0.1326
0.3420 -1.7791 -38.7532
20.2800 -39.4300 0.2783 0.5435
0.1003 -0.2044 -0.2378 0.1873 0.1251
0.3404 -1.7794 -38.7557
20.3000 -40.0800 0.1456 0.2347 -
0.0287 -0.2931 -0.2284 0.1940 0.1176
0.3387 -1.7797 -38.7582
20.3200 -41.1800 -0.3983 -0.2571 -
0.1687 -0.3730 -0.2153 0.2004 0.1101
0.3370 -1.7800 -38.7607
20.3400 -41.1700 0.2342 -0.5911 -
0.2825 -0.4385 -0.1985 0.2065 0.1027
0.3353 -1.7803 -38.7632
20.3600 -41.8000 -0.4068 -0.4870 -
0.3422 -0.4849 -0.1776 0.2122 0.0953
0.3335 -1.7806 -38.7657
20.3800 -41.4100 -0.5107 -0.0001 -
0.3429 -0.5079 -0.1527 0.2174 0.0879
0.3317 -1.7808 -38.7682
20.4000 -39.6300 0.8215 0.4340 -
0.2981 -0.5058 -0.1237 0.2221 0.0805
0.3298 -1.7811 -38.7707
20.4200 -40.7500 -0.4860 0.3713 -
0.2260 -0.4789 -0.0910 0.2262 0.0733
0.3279 -1.7813 -38.7731

20.4400 -40.7200 -0.2267 0.0851 -
0.1532 -0.4281 -0.0552 0.2298 0.0660
0.3260 -1.7815 -38.7756
20.4600 -41.1400 -0.7525 0.0986 -
0.1039 -0.3562 -0.0168 0.2327 0.0589
0.3240 -1.7817 -38.7781
20.4800 -39.8100 0.1750 0.2713 -
0.0799 -0.2673 0.0234 0.2349 0.0518
0.3219 -1.7819 -38.7806
20.5000 -38.9600 1.0353 0.2200 -
0.0607 -0.1672 0.0647 0.2363 0.0449
0.3198 -1.7821 -38.7831
20.5200 -39.9900 0.1846 -0.1724 -
0.0234 -0.0622 0.1066 0.2370 0.0380
0.3177 -1.7823 -38.7856
20.5400 -41.3500 -1.1442 -0.4959
0.0504 0.0413 0.1484 0.2368 0.0312
0.3155 -1.7825 -38.7881
20.5600 -38.9400 1.0354 -0.3772
0.1684 0.1381 0.1894 0.2358 0.0245
0.3133 -1.7826 -38.7906
20.5800 -39.6500 -0.4295 -0.0033
0.3104 0.2242 0.2288 0.2338 0.0180
0.3110 -1.7828 -38.7930
20.6000 -39.2800 -0.5378 0.2848
0.4393 0.2970 0.2662 0.2308 0.0115
0.3087 -1.7829 -38.7955
20.6200 -38.0400 0.5170 0.3522
0.5163 0.3541 0.3008 0.2269 0.0052
0.3064 -1.7830 -38.7980
20.6400 -38.7700 -0.0838 0.2072
0.5086 0.3940 0.3320 0.2218 -0.0010
0.3040 -1.7831 -38.8005
20.6600 -39.1100 -0.2048 0.0457
0.4026 0.4168 0.3592 0.2157 -0.0070
0.3016 -1.7832 -38.8030
20.6800 -38.9700 0.1533 0.0422
0.2093 0.4241 0.3821 0.2084 -0.0130
0.2991 -1.7833 -38.8055
20.7000 -39.2900 -0.0764 0.0744 -
0.0354 0.4183 0.4002 0.1999 -0.0187
0.2966 -1.7834 -38.8079
20.7200 -39.6200 0.1318 -0.0559 -
0.2802 0.4018 0.4134 0.1902 -0.0243
0.2940 -1.7835 -38.8104
20.7400 -39.9300 0.2440 -0.2722 -
0.4714 0.3769 0.4216 0.1793 -0.0298
0.2914 -1.7835 -38.8129
20.7600 -40.8600 -0.6500 -0.3104 -
0.5648 0.3459 0.4246 0.1671 -0.0351
0.2888 -1.7836 -38.8154

20.7800 -39.2500 0.7866 -0.0923 -
0.5355 0.3102 0.4225 0.1537 -0.0403
0.2861 -1.7836 -38.8178
20.8000 -40.5700 -0.7864 0.0954 -
0.3894 0.2708 0.4155 0.1392 -0.0453
0.2834 -1.7837 -38.8203
20.8200 -40.1100 -0.3795 0.0730 -
0.1606 0.2282 0.4039 0.1234 -0.0501
0.2806 -1.7837 -38.8228
20.8400 -38.5700 1.1474 -0.0840
0.0975 0.1828 0.3878 0.1067 -0.0548
0.2778 -1.7837 -38.8252
20.8600 -39.8400 -0.2289 -0.1623
0.3250 0.1349 0.3677 0.0889 -0.0593
0.2750 -1.7837 -38.8277
20.8800 -40.4500 -0.9956 -0.0353
0.4711 0.0851 0.3439 0.0702 -0.0636
0.2721 -1.7837 -38.8302
20.9000 -39.8600 -0.7697 0.3275
0.5019 0.0337 0.3167 0.0507 -0.0678
0.2692 -1.7837 -38.8327
20.9200 -38.0700 1.1040 0.5506
0.4023 -0.0180 0.2866 0.0304 -0.0718
0.2662 -1.7837 -38.8351
20.9400 -40.5200 -0.6953 0.1942
0.1938 -0.0684 0.2540 0.0094 -0.0756
0.2632 -1.7836 -38.8376
20.9600 -40.8200 -0.1182 -0.3467 -
0.0503 -0.1156 0.2192 -0.0121 -0.0793
0.2602 -1.7836 -38.8401
20.9800 -41.3000 -0.1260 -0.4841 -
0.2482 -0.1577 0.1827 -0.0341 -0.0828
0.2571 -1.7835 -38.8425
21.0000 -41.2100 -0.1928 -0.2415 -
0.3366 -0.1926 0.1447 -0.0566 -0.0861
0.2540 -1.7835 -38.8450
21.0200 -40.4000 0.5116 -0.0555 -
0.3001 -0.2184 0.1056 -0.0793 -0.0892
0.2508 -1.7834 -38.8474
21.0400 -41.7100 -0.5711 -0.1821 -
0.1630 -0.2337 0.0659 -0.1023 -0.0921
0.2476 -1.7833 -38.8499
21.0600 -40.3800 0.5796 -0.1761 -
0.0025 -0.2378 0.0259 -0.1255 -0.0949
0.2444 -1.7832 -38.8524
21.0800 -41.1800 -0.7752 0.3624
0.1022 -0.2308 -0.0140 -0.1487 -0.0974
0.2411 -1.7831 -38.8548
21.1000 -39.2000 0.8816 0.7106
0.1235 -0.2133 -0.0533 -0.1719 -0.0998
0.2378 -1.7830 -38.8573

21.1200	-41.4200	-0.6653	0.1509	
0.0669	-0.1871	-0.0916	-0.1950	-0.1020
	0.2345	-1.7829	-38.8597	
21.1400	-41.2400	0.3324	-0.5932	-
0.0182	-0.1549	-0.1283	-0.2179	-0.1039
	0.2311	-1.7828	-38.8622	
21.1600	-42.2200	-0.3674	-0.7326	-
0.0758	-0.1196	-0.1632	-0.2405	-0.1057
	0.2276	-1.7827	-38.8646	
21.1800	-41.2800	0.2679	-0.3702	-
0.0768	-0.0846	-0.1958	-0.2627	-0.1073
	0.2242	-1.7826	-38.8671	
21.2000	-40.9500	-0.0694	0.1840	-
0.0330	-0.0528	-0.2256	-0.2845	-0.1087
	0.2207	-1.7824	-38.8695	
21.2200	-40.4551	-0.1413	0.6216	
0.0312	-0.0263	-0.2524	-0.3058	-0.1099
	0.2171	-1.7823	-38.8720	
21.2400	-40.1400	0.3031	0.6371	
0.0907	-0.0061	-0.2758	-0.3264	-0.1109
	0.2136	-1.7821	-38.8744	
21.2600	-40.9700	-0.0280	0.1964	
0.1270	0.0075	-0.2957	-0.3463	-0.1117
	0.2100	-1.7820	-38.8769	
21.2800	-41.6200	-0.2624	-0.3155	
0.1322	0.0150	-0.3119	-0.3655	-0.1123
	0.2063	-1.7818	-38.8793	
21.3000	-41.6200	0.0226	-0.4993	
0.1017	0.0172	-0.3243	-0.3838	-0.1126
	0.2027	-1.7816	-38.8818	
21.3200	-41.3100	0.1432	-0.2606	
0.0409	0.0155	-0.3329	-0.4011	-0.1128
	0.1990	-1.7814	-38.8842	
21.3400	-41.5000	-0.2829	0.1659	-
0.0325	0.0113	-0.3378	-0.4175	-0.1128
	0.1952	-1.7813	-38.8867	
21.3600	-40.6400	0.5015	0.3643	-
0.1012	0.0060	-0.3390	-0.4327	-0.1125
	0.1914	-1.7811	-38.8891	
21.3800	-41.7600	-0.4390	0.1717	-
0.1549	0.0005	-0.3367	-0.4469	-0.1120
	0.1876	-1.7809	-38.8916	
21.4000	-41.4800	0.3042	-0.1009	-
0.1845	-0.0044	-0.3310	-0.4599	-0.1113
	0.1838	-1.7807	-38.8940	
21.4200	-41.7900	-0.0106	-0.2581	-
0.1731	-0.0091	-0.3220	-0.4717	-0.1105
	0.1799	-1.7804	-38.8964	
21.4400	-41.6000	0.0788	-0.2987	-
0.1068	-0.0147	-0.3098	-0.4822	-0.1093
	0.1761	-1.7802	-38.8989	

21.4600	-41.9900	-0.5248	-0.0680	
0.0044	-0.0222	-0.2948	-0.4915	-0.1080
	0.1721	-1.7800	-38.9013	
21.4800	-40.5400	0.4935	0.2792	
0.1243	-0.0324	-0.2771	-0.4994	-0.1065
	0.1682	-1.7798	-38.9038	
21.5000	-40.7000	0.2816	0.2631	
0.2111	-0.0456	-0.2570	-0.5061	-0.1047
	0.1642	-1.7795	-38.9062	
21.5200	-41.7100	-0.4824	-0.0655	
0.2421	-0.0618	-0.2348	-0.5115	-0.1027
	0.1602	-1.7793	-38.9086	
21.5400	-41.4500	0.0279	-0.2698	
0.2205	-0.0801	-0.2109	-0.5157	-0.1006
	0.1562	-1.7791	-38.9111	
21.5600	-41.1100	0.3005	-0.1159	
0.1559	-0.0990	-0.1854	-0.5185	-0.0982
	0.1522	-1.7788	-38.9135	
21.5800	-41.8900	-0.6982	0.2029	
0.0591	-0.1157	-0.1588	-0.5200	-0.0956
	0.1481	-1.7785	-38.9159	
21.6000	-40.3300	1.0758	0.1078	-
0.0517	-0.1268	-0.1313	-0.5203	-0.0927
	0.1440	-1.7783	-38.9184	
21.6200	-42.9300	-1.2550	-0.1587	-
0.1637	-0.1289	-0.1033	-0.5194	-0.0897
	0.1399	-1.7780	-38.9208	
21.6400	-41.3800	0.3214	0.0033	-
0.2622	-0.1192	-0.0751	-0.5172	-0.0865
	0.1358	-1.7777	-38.9232	
21.6600	-40.2400	1.1597	0.2298	-
0.3172	-0.0960	-0.0470	-0.5139	-0.0831
	0.1316	-1.7775	-38.9256	
21.6800	-41.7800	-0.3623	0.0716	-
0.3018	-0.0590	-0.0195	-0.5093	-0.0795
	0.1275	-1.7772	-38.9281	
21.7000	-42.5700	-0.8337	-0.2820	-
0.2086	-0.0110	0.0072	-0.5036	-0.0757
	0.1233	-1.7769	-38.9305	
21.7200	-41.0800	0.4976	-0.4288	-
0.0545	0.0437	0.0326	-0.4967	-0.0717
	0.1191	-1.7766	-38.9329	
21.7400	-41.2600	-0.2191	-0.0906	
0.1204	0.0997	0.0566	-0.4888	-0.0676
	0.1149	-1.7763	-38.9353	
21.7600	-40.5600	-0.3361	0.4130	
0.2653	0.1513	0.0789	-0.4797	-0.0633
	0.1107	-1.7760	-38.9378	
21.7800	-39.4000	0.5071	0.6078	
0.3315	0.1931	0.0992	-0.4695	-0.0589
	0.1065	-1.7757	-38.9402	

21.8000 -40.3700 -0.0428 0.2140
0.2944 0.2203 0.1172 -0.4582 -0.0543
0.1023 -1.7754 -38.9426
21.8200 -41.2000 -0.3032 -0.3574
0.1862 0.2302 0.1328 -0.4460 -0.0495
0.0980 -1.7751 -38.9450
21.8400 -41.1800 0.0522 -0.5435
0.0561 0.2217 0.1459 -0.4327 -0.0446
0.0938 -1.7748 -38.9474
21.8600 -40.9300 0.0543 -0.1734 -
0.0490 0.1947 0.1565 -0.4185 -0.0396
0.0896 -1.7745 -38.9498
21.8800 -41.1900 -0.6220 0.3722 -
0.1010 0.1503 0.1647 -0.4033 -0.0345
0.0853 -1.7742 -38.9523
21.9000 -39.5500 1.0343 0.3490 -
0.0880 0.0915 0.1705 -0.3872 -0.0293
0.0811 -1.7738 -38.9547
21.9200 -42.1800 -1.0759 -0.1950 -
0.0282 0.0237 0.1743 -0.3703 -0.0240
0.0769 -1.7735 -38.9571
21.9400 -40.7200 0.3834 -0.2986
0.0348 -0.0468 0.1763 -0.3526 -0.0185
0.0726 -1.7732 -38.9595
21.9600 -40.6800 0.2399 0.0280
0.0662 -0.1135 0.1767 -0.3340 -0.0130
0.0684 -1.7729 -38.9619
21.9800 -40.4200 0.4679 0.2142
0.0475 -0.1711 0.1758 -0.3148 -0.0075
0.0642 -1.7725 -38.9643
22.0000 -41.2600 -0.3226 0.1253 -
0.0203 -0.2154 0.1739 -0.2948 -0.0018
0.0599 -1.7722 -38.9667
22.0200 -41.3000 -0.0908 -0.0435 -
0.1146 -0.2438 0.1711 -0.2742 0.0039
0.0557 -1.7719 -38.9691
22.0400 -41.3338 0.0849 -0.1341 -
0.2054 -0.2546 0.1677 -0.2530 0.0097
0.0515 -1.7715 -38.9715
22.0600 -41.3661 -0.0096 -0.1290 -
0.2681 -0.2473 0.1638 -0.2311 0.0155
0.0473 -1.7712 -38.9739
22.0800 -41.3904 0.0252 -0.1081 -
0.2899 -0.2225 0.1594 -0.2088 0.0213
0.0431 -1.7708 -38.9763
22.1000 -41.4000 -0.0661 -0.0540 -
0.2676 -0.1828 0.1543 -0.1860 0.0271
0.0390 -1.7705 -38.9787
22.1200 -40.9100 0.0020 0.0629 -
0.2099 -0.1316 0.1485 -0.1626 0.0330
0.0348 -1.7701 -38.9811

22.1400 -40.7000 0.0159 0.1789 -
0.1319 -0.0731 0.1418 -0.1389 0.0389
0.0306 -1.7698 -38.9835
22.1600 -40.5800 -0.0871 0.1909 -
0.0452 -0.0116 0.1341 -0.1147 0.0448
0.0265 -1.7694 -38.9859
22.1800 -40.0300 0.3845 0.0642
0.0468 0.0486 0.1252 -0.0902 0.0507
0.0224 -1.7691 -38.9883
22.2000 -41.0400 -0.5324 -0.1435
0.1429 0.1034 0.1151 -0.0653 0.0565
0.0183 -1.7687 -38.9907
22.2200 -40.0600 0.3863 -0.3006
0.2382 0.1490 0.1035 -0.0401 0.0624
0.0143 -1.7684 -38.9931
22.2400 -40.1300 0.3140 -0.2687
0.3198 0.1830 0.0905 -0.0146 0.0682
0.0102 -1.7680 -38.9955
22.2600 -40.8500 -0.8387 0.0144
0.3708 0.2039 0.0758 0.0111 0.0739
0.0062 -1.7677 -38.9979
22.2800 -39.0300 0.5418 0.3996
0.3714 0.2116 0.0595 0.0371 0.0796
0.0022 -1.7673 -39.0003
22.3000 -38.9000 0.6997 0.5785
0.3052 0.2068 0.0415 0.0631 0.0853
-0.0018 -1.7669 -39.0027
22.3200 -40.4100 -0.6745 0.4170
0.1698 0.1921 0.0219 0.0893 0.0909
-0.0057 -1.7666 -39.0051
22.3400 -40.9800 -0.6225 0.0596 -
0.0102 0.1701 0.0008 0.1155 0.0964
-0.0096 -1.7662 -39.0074
22.3600 -40.0300 0.8879 -0.3070 -
0.1942 0.1440 -0.0217 0.1417 0.1018
-0.0135 -1.7659 -39.0098
22.3800 -42.0800 -0.7562 -0.5148 -
0.3371 0.1166 -0.0455 0.1678 0.1072
-0.0174 -1.7655 -39.0122
22.4000 -41.3600 0.0384 -0.4727 -
0.3992 0.0907 -0.0705 0.1938 0.1124
-0.0212 -1.7652 -39.0146
22.4200 -40.9600 0.2894 -0.3406 -
0.3627 0.0683 -0.0964 0.2197 0.1175
-0.0250 -1.7648 -39.0170
22.4400 -40.5400 0.4693 -0.2830 -
0.2388 0.0499 -0.1229 0.2452 0.1226
-0.0287 -1.7644 -39.0194
22.4600 -41.2600 -0.4888 -0.1228 -
0.0708 0.0346 -0.1496 0.2704 0.1275
-0.0324 -1.7641 -39.0217

22.4800 -40.8100 -0.4400 0.2447
0.0932 0.0210 -0.1760 0.2952 0.1322
-0.0361 -1.7637 -39.0241
22.5000 -39.1400 0.6928 0.5778
0.2144 0.0078 -0.2016 0.3195 0.1369
-0.0397 -1.7634 -39.0265
22.5200 -39.5500 0.1796 0.5000
0.2665 -0.0066 -0.2259 0.3432 0.1413
-0.0433 -1.7630 -39.0289
22.5400 -40.5300 -0.3801 0.1004
0.2526 -0.0234 -0.2483 0.3663 0.1457
-0.0469 -1.7626 -39.0312
22.5600 -41.1300 -0.4534 -0.2521
0.1944 -0.0433 -0.2683 0.3887 0.1498
-0.0504 -1.7623 -39.0336
22.5800 -40.2600 0.6334 -0.3523
0.1178 -0.0669 -0.2854 0.4103 0.1538
-0.0539 -1.7619 -39.0360
22.6000 -41.2200 -0.4328 -0.1833
0.0447 -0.0940 -0.2989 0.4310 0.1577
-0.0573 -1.7616 -39.0384
22.6200 -40.8900 -0.2712 0.0703 -
0.0102 -0.1239 -0.3087 0.4508 0.1613
-0.0607 -1.7612 -39.0407
22.6400 -40.2300 0.4769 0.1959 -
0.0418 -0.1547 -0.3143 0.4696 0.1647
-0.0640 -1.7609 -39.0431
22.6600 -40.4100 0.3441 0.0794 -
0.0547 -0.1839 -0.3153 0.4875 0.1680
-0.0673 -1.7605 -39.0455
22.6800 -41.5600 -0.6962 -0.0954 -
0.0567 -0.2084 -0.3118 0.5043 0.1710
-0.0705 -1.7602 -39.0478
22.7000 -40.5200 0.3821 -0.0888 -
0.0592 -0.2252 -0.3035 0.5200 0.1738
-0.0737 -1.7598 -39.0502
22.7200 -40.4300 0.4248 -0.0178 -
0.0752 -0.2310 -0.2903 0.5346 0.1764
-0.0768 -1.7595 -39.0525
22.7400 -41.1200 -0.2514 -0.0161 -
0.1138 -0.2234 -0.2724 0.5480 0.1788
-0.0799 -1.7591 -39.0549
22.7600 -40.8200 0.0767 -0.0056 -
0.1749 -0.2015 -0.2497 0.5602 0.1809
-0.0829 -1.7588 -39.0573
22.7800 -41.1100 -0.4603 0.1852 -
0.2444 -0.1660 -0.2225 0.5712 0.1828
-0.0859 -1.7584 -39.0596
22.8000 -40.5600 -0.1459 0.3691 -
0.2893 -0.1194 -0.1912 0.5810 0.1844
-0.0888 -1.7581 -39.0620

22.8200 -39.8400 0.7232 0.2011 –
0.2757 -0.0650 -0.1560 0.5895 0.1858
-0.0916 -1.7578 -39.0643
22.8400 -41.4700 -0.7204 -0.3378 –
0.1871 -0.0071 -0.1173 0.5967 0.1869
-0.0944 -1.7574 -39.0667
22.8600 -40.4300 0.4603 -0.6552 –
0.0268 0.0491 -0.0754 0.6025 0.1877
-0.0972 -1.7571 -39.0690
22.8800 -40.7500 -0.5048 -0.4010
0.1823 0.0984 -0.0307 0.6070 0.1883
-0.0998 -1.7568 -39.0714
22.9000 -39.1300 0.2702 0.1825
0.3910 0.1350 0.0165 0.6101 0.1886
-0.1025 -1.7565 -39.0737
22.9200 -38.9000 -0.1458 0.7063
0.5400 0.1531 0.0656 0.6118 0.1886
-0.1050 -1.7561 -39.0761
22.9400 -38.0000 0.5625 0.7529
0.5773 0.1480 0.1163 0.6120 0.1883
-0.1075 -1.7558 -39.0784
22.9600 -39.4900 -0.4397 0.3136
0.4892 0.1181 0.1677 0.6107 0.1878
-0.1100 -1.7555 -39.0808
22.9800 -40.0500 -0.2594 -0.2123
0.3163 0.0674 0.2194 0.6079 0.1869
-0.1123 -1.7552 -39.0831
23.0000 -39.6100 0.6389 -0.5300
0.1125 0.0014 0.2706 0.6035 0.1858
-0.1147 -1.7549 -39.0855
23.0200 -41.2500 -0.7369 -0.5065 –
0.0737 -0.0740 0.3207 0.5976 0.1845
-0.1169 -1.7545 -39.0878
23.0400 -39.6200 0.6389 -0.1951 –
0.2108 -0.1521 0.3690 0.5901 0.1828
-0.1191 -1.7542 -39.0902
23.0600 -40.6600 -0.4947 0.1714 –
0.2924 -0.2243 0.4147 0.5809 0.1809
-0.1212 -1.7539 -39.0925
23.0800 -40.2400 -0.2295 0.3804 –
0.3359 -0.2807 0.4573 0.5700 0.1787
-0.1233 -1.7536 -39.0948
23.1000 -39.6200 0.4911 0.3266 –
0.3663 -0.3118 0.4961 0.5574 0.1762
-0.1253 -1.7533 -39.0972
23.1200 -40.2100 0.2539 0.0433 –
0.4088 -0.3098 0.5304 0.5431 0.1735
-0.1272 -1.7530 -39.0995
23.1400 -41.3500 -0.6591 -0.1704 –
0.4815 -0.2720 0.5598 0.5270 0.1706
-0.1291 -1.7528 -39.1018

23.1600 -39.9400 0.5437 -0.0126 -
0.5728 -0.2008 0.5837 0.5092 0.1673
-0.1309 -1.7525 -39.1042
23.1800 -40.5200 -0.2644 0.2137 -
0.6257 -0.1025 0.6018 0.4897 0.1639
-0.1327 -1.7522 -39.1065
23.2000 -39.8700 0.3708 0.0452 -
0.5829 0.0142 0.6135 0.4684 0.1602
-0.1344 -1.7519 -39.1088
23.2200 -40.3400 0.0830 -0.3663 -
0.4224 0.1399 0.6184 0.4454 0.1563
-0.1360 -1.7516 -39.1112
23.2400 -40.7600 -0.5635 -0.5412 -
0.1514 0.2639 0.6162 0.4209 0.1521
-0.1376 -1.7514 -39.1135
23.2600 -39.3300 0.1201 -0.1856
0.1957 0.3760 0.6062 0.3948 0.1477
-0.1391 -1.7511 -39.1158
23.2800 -38.5100 0.0382 0.3743
0.5312 0.4656 0.5882 0.3672 0.1431
-0.1405 -1.7508 -39.1181
23.3000 -37.6700 0.4501 0.6150
0.7545 0.5223 0.5619 0.3384 0.1383
-0.1419 -1.7506 -39.1205
23.3200 -38.5400 -0.2225 0.3881
0.7916 0.5372 0.5271 0.3083 0.1333
-0.1432 -1.7503 -39.1228
23.3400 -38.9300 0.1468 -0.0302
0.6520 0.5081 0.4843 0.2772 0.1281
-0.1444 -1.7500 -39.1251
23.3600 -40.1100 -0.4938 -0.2727
0.3889 0.4412 0.4342 0.2451 0.1227
-0.1456 -1.7498 -39.1274
23.3800 -39.5800 0.4802 -0.1992
0.0687 0.3451 0.3779 0.2123 0.1171
-0.1467 -1.7496 -39.1297
23.4000 -40.9100 -0.6524 0.0728 -
0.2352 0.2286 0.3163 0.1787 0.1113
-0.1478 -1.7493 -39.1321
23.4200 -40.1300 0.6861 0.1120 -
0.4481 0.1003 0.2506 0.1447 0.1053
-0.1488 -1.7491 -39.1344
23.4400 -41.7100 -0.3150 -0.2360 -
0.5333 -0.0313 0.1816 0.1102 0.0992
-0.1497 -1.7488 -39.1367
23.4600 -42.1800 -0.2659 -0.4668 -
0.4908 -0.1582 0.1106 0.0755 0.0928
-0.1506 -1.7486 -39.1390
23.4800 -42.0200 -0.4086 -0.1403 -
0.3459 -0.2732 0.0384 0.0406 0.0864
-0.1514 -1.7484 -39.1413

23.5000 -40.4200 0.5961 0.4284 -
0.1557 -0.3695 -0.0339 0.0057 0.0797
-0.1522 -1.7482 -39.1436
23.5200 -41.3400 -0.2480 0.5533
0.0218 -0.4408 -0.1052 -0.0290 0.0729
-0.1528 -1.7480 -39.1459
23.5400 -41.1000 0.6269 -0.1057
0.1447 -0.4820 -0.1746 -0.0635 0.0660
-0.1535 -1.7478 -39.1482
23.5600 -43.1700 -0.8132 -0.6749
0.1930 -0.4904 -0.2411 -0.0975 0.0589
-0.1540 -1.7476 -39.1505
23.5800 -41.4500 0.6300 -0.2576
0.1602 -0.4667 -0.3039 -0.1311 0.0517
-0.1545 -1.7474 -39.1528
23.6000 -41.8900 -0.4928 0.5522
0.0673 -0.4143 -0.3622 -0.1639 0.0443
-0.1550 -1.7472 -39.1552
23.6200 -40.6200 0.6647 0.7576 -
0.0451 -0.3387 -0.4157 -0.1960 0.0368
-0.1554 -1.7470 -39.1575
23.6400 -42.4800 -0.6378 0.2328 -
0.1382 -0.2459 -0.4639 -0.2272 0.0292
-0.1557 -1.7468 -39.1598
23.6600 -42.3000 0.2967 -0.4986 -
0.1839 -0.1426 -0.5064 -0.2573 0.0215
-0.1559 -1.7466 -39.1620
23.6800 -42.3200 0.5458 -0.8510 -
0.1642 -0.0356 -0.5430 -0.2862 0.0137
-0.1561 -1.7464 -39.1643
23.7000 -43.4200 -0.9296 -0.5304 -
0.0772 0.0681 -0.5734 -0.3138 0.0057
-0.1563 -1.7463 -39.1666
23.7200 -41.8700 -0.3062 0.2502
0.0413 0.1618 -0.5973 -0.3400 -0.0023
-0.1564 -1.7461 -39.1689
23.7400 -39.8700 1.0147 0.7774
0.1407 0.2397 -0.6145 -0.3646 -0.0104
-0.1564 -1.7459 -39.1712
23.7600 -42.0400 -0.8816 0.5013
0.1792 0.2966 -0.6248 -0.3876 -0.0186
-0.1563 -1.7458 -39.1735
23.7800 -41.0900 0.6464 -0.1304
0.1561 0.3292 -0.6282 -0.4087 -0.0269
-0.1562 -1.7456 -39.1758
23.8000 -42.3300 -0.1240 -0.4559
0.0885 0.3368 -0.6247 -0.4280 -0.0353
-0.1561 -1.7455 -39.1781
23.8200 -42.7400 -0.4667 -0.4133
0.0049 0.3207 -0.6147 -0.4453 -0.0438
-0.1558 -1.7453 -39.1804

23.8400 -41.5600 0.5216 -0.1575 -
0.0584 0.2831 -0.5983 -0.4607 -0.0523
-0.1556 -1.7452 -39.1827
23.8600 -42.4500 -0.5066 0.1123 -
0.0789 0.2277 -0.5761 -0.4740 -0.0609
-0.1552 -1.7451 -39.1850
23.8800 -41.7600 0.1960 0.2339 -
0.0579 0.1589 -0.5486 -0.4853 -0.0695
-0.1548 -1.7449 -39.1872
23.9000 -41.5300 0.5305 0.1117 -
0.0140 0.0817 -0.5165 -0.4947 -0.0782
-0.1544 -1.7448 -39.1895
23.9200 -42.6900 -0.4824 -0.1159
0.0223 0.0012 -0.4803 -0.5020 -0.0869
-0.1538 -1.7447 -39.1918
23.9400 -42.5400 -0.1790 -0.1018
0.0250 -0.0777 -0.4407 -0.5075 -0.0957
-0.1533 -1.7446 -39.1941
23.9600 -41.9800 0.1521 0.1635 -
0.0116 -0.1512 -0.3983 -0.5111 -0.1045
-0.1526 -1.7445 -39.1963
23.9800 -42.0500 -0.0020 0.3196 -
0.0692 -0.2163 -0.3535 -0.5129 -0.1134
-0.1519 -1.7444 -39.1986
24.0000 -41.9700 0.4488 0.0895 -
0.1235 -0.2711 -0.3069 -0.5129 -0.1223
-0.1512 -1.7443 -39.2009
24.0200 -42.7000 0.0633 -0.3188 -
0.1485 -0.3144 -0.2587 -0.5112 -0.1312
-0.1504 -1.7442 -39.2032
24.0400 -43.6100 -0.6886 -0.4639 -
0.1193 -0.3458 -0.2095 -0.5078 -0.1401
-0.1495 -1.7441 -39.2054
24.0600 -42.3700 -0.0082 -0.1456 -
0.0298 -0.3658 -0.1596 -0.5029 -0.1490
-0.1486 -1.7440 -39.2077
24.0800 -41.2000 0.6505 0.2863
0.0850 -0.3754 -0.1095 -0.4964 -0.1580
-0.1476 -1.7440 -39.2100
24.1000 -41.1900 0.4271 0.3935
0.1808 -0.3753 -0.0593 -0.4885 -0.1669
-0.1466 -1.7439 -39.2122
24.1200 -42.4100 -0.6309 0.1584
0.2250 -0.3663 -0.0097 -0.4791 -0.1759
-0.1455 -1.7438 -39.2145
24.1400 -41.6700 0.4228 -0.1597
0.2067 -0.3484 0.0391 -0.4684 -0.1848
-0.1443 -1.7438 -39.2168
24.1600 -42.2700 -0.1374 -0.2423
0.1277 -0.3216 0.0866 -0.4564 -0.1937
-0.1431 -1.7437 -39.2190

24.1800 -42.2400 -0.3077 -0.0553
0.0090 -0.2853 0.1325 -0.4432 -0.2026
-0.1419 -1.7436 -39.2213
24.2000 -41.4200 0.2915 0.1926 -
0.1148 -0.2395 0.1764 -0.4287 -0.2115
-0.1405 -1.7436 -39.2235
24.2200 -41.6093 0.0940 0.1980 -
0.2075 -0.1838 0.2180 -0.4131 -0.2204
-0.1392 -1.7435 -39.2258
24.2400 -41.9607 -0.1068 -0.0483 -
0.2462 -0.1184 0.2568 -0.3964 -0.2292
-0.1377 -1.7435 -39.2281
24.2600 -42.1500 -0.0513 -0.3096 -
0.2319 -0.0437 0.2926 -0.3787 -0.2379
-0.1363 -1.7435 -39.2303
24.2800 -41.9200 -0.0196 -0.3874 -
0.1824 0.0387 0.3249 -0.3600 -0.2466
-0.1347 -1.7434 -39.2326
24.3000 -41.1900 0.5436 -0.2042 -
0.1205 0.1265 0.3533 -0.3404 -0.2552
-0.1331 -1.7434 -39.2348
24.3200 -41.7500 -0.8728 0.2594 -
0.0643 0.2165 0.3776 -0.3199 -0.2638
-0.1315 -1.7434 -39.2371
24.3400 -40.4400 -0.1141 0.6503 -
0.0229 0.3046 0.3974 -0.2986 -0.2723
-0.1298 -1.7434 -39.2393
24.3600 -39.0300 1.2826 0.5708
0.0045 0.3864 0.4123 -0.2766 -0.2807
-0.1280 -1.7434 -39.2416
24.3800 -41.9500 -1.0820 -0.1104
0.0308 0.4569 0.4221 -0.2540 -0.2890
-0.1262 -1.7433 -39.2438
24.4000 -42.2500 -0.8532 -0.7028
0.0743 0.5116 0.4265 -0.2308 -0.2973
-0.1244 -1.7433 -39.2460
24.4200 -40.3800 0.8495 -0.7166
0.1487 0.5467 0.4253 -0.2072 -0.3054
-0.1225 -1.7433 -39.2483
24.4400 -40.5300 -0.0145 -0.1681
0.2448 0.5596 0.4182 -0.1832 -0.3134
-0.1205 -1.7433 -39.2505
24.4600 -40.3700 -0.5173 0.4459
0.3281 0.5489 0.4055 -0.1590 -0.3213
-0.1185 -1.7433 -39.2528
24.4800 -39.0700 0.5644 0.6888
0.3612 0.5137 0.3874 -0.1348 -0.3291
-0.1164 -1.7433 -39.2550
24.5000 -39.5600 0.3961 0.4869
0.3179 0.4546 0.3647 -0.1106 -0.3368
-0.1143 -1.7433 -39.2572

24.5200 -41.0700 -0.5179 0.0738
0.2057 0.3755 0.3381 -0.0865 -0.3443
-0.1121 -1.7434 -39.2595
24.5400 -41.6000 -0.4604 -0.2140
0.0490 0.2812 0.3083 -0.0627 -0.3517
-0.1099 -1.7434 -39.2617
24.5600 -40.6800 0.7182 -0.2837 -
0.1187 0.1768 0.2761 -0.0392 -0.3589
-0.1077 -1.7434 -39.2639
24.5800 -42.3600 -0.5830 -0.2995 -
0.2595 0.0670 0.2424 -0.0163 -0.3660
-0.1053 -1.7434 -39.2662
24.6000 -41.7100 0.2350 -0.2595 -
0.3448 -0.0436 0.2078 0.0059 -0.3729
-0.1030 -1.7434 -39.2684
24.6200 -41.7600 0.0632 -0.0634 -
0.3635 -0.1511 0.1731 0.0275 -0.3797
-0.1005 -1.7435 -39.2706
24.6400 -41.8400 -0.1327 0.1892 -
0.3205 -0.2520 0.1392 0.0481 -0.3863
-0.0981 -1.7435 -39.2728
24.6600 -41.3200 0.1881 0.3004 -
0.2244 -0.3433 0.1069 0.0677 -0.3927
-0.0955 -1.7435 -39.2751
24.6800 -41.7200 -0.0404 0.1483 -
0.0891 -0.4224 0.0769 0.0862 -0.3989
-0.0930 -1.7436 -39.2773
24.7000 -42.0700 -0.0461 -0.1962
0.0687 -0.4881 0.0500 0.1035 -0.4049
-0.0904 -1.7436 -39.2795
24.7200 -41.9800 0.2244 -0.4995
0.2211 -0.5391 0.0271 0.1194 -0.4107
-0.0877 -1.7436 -39.2817
24.7400 -42.4600 -0.4773 -0.4477
0.3330 -0.5735 0.0087 0.1339 -0.4164
-0.0850 -1.7437 -39.2839
24.7600 -41.3800 0.1761 0.0851
0.3742 -0.5889 -0.0045 0.1468 -0.4218
-0.0822 -1.7437 -39.2862
24.7800 -41.0400 -0.1093 0.6733
0.3231 -0.5839 -0.0124 0.1581 -0.4270
-0.0794 -1.7438 -39.2884
24.8000 -40.6100 0.5762 0.6478
0.1707 -0.5583 -0.0149 0.1677 -0.4319
-0.0765 -1.7438 -39.2906
24.8200 -42.5800 -0.6205 0.0077 -
0.0552 -0.5129 -0.0123 0.1757 -0.4367
-0.0736 -1.7439 -39.2928
24.8400 -42.2300 0.3976 -0.5486 -
0.2898 -0.4486 -0.0050 0.1820 -0.4412
-0.0707 -1.7439 -39.2950

24.8600 -42.8500 -0.0361 -0.5625 -
0.4666 -0.3667 0.0067 0.1866 -0.4455
-0.0677 -1.7440 -39.2972
24.8800 -43.3600 -1.1000 -0.1722 -
0.5462 -0.2686 0.0222 0.1898 -0.4495
-0.0646 -1.7441 -39.2994
24.9000 -41.4300 0.4036 0.1893 -
0.5231 -0.1573 0.0409 0.1914 -0.4533
-0.0616 -1.7441 -39.3016
24.9200 -39.9800 1.4946 0.2573 -
0.4038 -0.0374 0.0623 0.1915 -0.4569
-0.0584 -1.7442 -39.3038
24.9400 -41.3200 0.1432 -0.0116 -
0.2114 0.0852 0.0857 0.1902 -0.4602
-0.0552 -1.7442 -39.3060
24.9600 -42.8800 -1.5817 -0.2173
0.0052 0.2048 0.1106 0.1875 -0.4633
-0.0520 -1.7443 -39.3082
24.9800 -40.6400 0.1316 -0.0224
0.1972 0.3154 0.1364 0.1835 -0.4661
-0.0487 -1.7444 -39.3104
25.0000 -39.0100 1.1858 0.2654
0.3438 0.4109 0.1625 0.1783 -0.4686
-0.0454 -1.7444 -39.3126
25.0200 -40.6400 -0.7038 0.2628
0.4360 0.4851 0.1882 0.1718 -0.4709
-0.0421 -1.7445 -39.3148
25.0400 -40.1300 -0.0660 0.0043
0.4735 0.5328 0.2131 0.1641 -0.4729
-0.0387 -1.7446 -39.3170
25.0600 -40.3000 0.0747 -0.1791
0.4637 0.5506 0.2365 0.1553 -0.4747
-0.0352 -1.7446 -39.3192
25.0800 -40.4400 0.0566 -0.2206
0.4152 0.5380 0.2578 0.1455 -0.4762
-0.0318 -1.7447 -39.3214
25.1000 -40.4100 0.0515 -0.1322
0.3342 0.4977 0.2765 0.1347 -0.4774
-0.0283 -1.7448 -39.3236
25.1200 -40.6700 -0.3278 0.1101
0.2140 0.4358 0.2920 0.1230 -0.4784
-0.0247 -1.7449 -39.3258
25.1400 -40.3900 0.0674 0.3615
0.0499 0.3600 0.3040 0.1103 -0.4792
-0.0211 -1.7449 -39.3279
25.1600 -40.2300 0.5766 0.3068 -
0.1433 0.2784 0.3120 0.0969 -0.4796
-0.0175 -1.7450 -39.3301
25.1800 -42.1800 -0.7225 -0.0850 -
0.3297 0.1987 0.3154 0.0827 -0.4798
-0.0138 -1.7451 -39.3323

25.2000 -41.2800 0.5902 -0.3225 -
0.4573 0.1280 0.3139 0.0678 -0.4797
-0.0101 -1.7452 -39.3345
25.2200 -42.4400 -0.6211 -0.2029 -
0.4888 0.0723 0.3070 0.0523 -0.4794
-0.0063 -1.7452 -39.3367
25.2400 -41.0100 0.7201 -0.0759 -
0.4190 0.0359 0.2943 0.0363 -0.4788
-0.0025 -1.7453 -39.3388
25.2600 -42.3600 -0.7786 -0.0036 -
0.2780 0.0192 0.2756 0.0199 -0.4779
0.0013 -1.7454 -39.3410
25.2800 -40.9400 0.2634 0.1881 -
0.1235 0.0203 0.2509 0.0032 -0.4768
0.0052 -1.7454 -39.3432
25.3000 -40.5000 0.5700 0.3091 -
0.0022 0.0357 0.2208 -0.0138 -0.4754
0.0090 -1.7455 -39.3454
25.3200 -40.5100 0.5956 0.0850
0.0648 0.0615 0.1858 -0.0310 -0.4737
0.0130 -1.7456 -39.3475
25.3400 -42.2700 -0.8751 -0.1922
0.0931 0.0933 0.1466 -0.0481 -0.4718
0.0169 -1.7457 -39.3497
25.3600 -41.6900 -0.3415 -0.1886
0.1067 0.1259 0.1040 -0.0653 -0.4696
0.0209 -1.7457 -39.3519
25.3800 -40.5800 0.7966 -0.0337
0.1236 0.1543 0.0588 -0.0822 -0.4671
0.0249 -1.7458 -39.3540
25.4000 -41.2500 0.0731 0.0142
0.1553 0.1736 0.0119 -0.0990 -0.4643
0.0290 -1.7459 -39.3562
25.4200 -41.7500 -0.4114 -0.0271
0.1975 0.1797 -0.0360 -0.1154 -0.4613
0.0330 -1.7459 -39.3584
25.4400 -41.2300 0.2013 -0.0082
0.2356 0.1699 -0.0839 -0.1313 -0.4580
0.0371 -1.7460 -39.3605
25.4600 -41.4400 -0.0672 0.0716
0.2556 0.1431 -0.1310 -0.1468 -0.4544
0.0413 -1.7461 -39.3627
25.4800 -41.0600 0.3454 0.0892
0.2459 0.0994 -0.1767 -0.1616 -0.4505
0.0454 -1.7462 -39.3648
25.5000 -42.3700 -0.7919 0.1087
0.2114 0.0399 -0.2201 -0.1757 -0.4464
0.0496 -1.7462 -39.3670
25.5200 -40.8900 0.9635 0.0549
0.1658 -0.0327 -0.2607 -0.1890 -0.4420
0.0538 -1.7463 -39.3691

25.5400 -42.5800 -0.4075 -0.1743
0.1258 -0.1151 -0.2978 -0.2014 -0.4374
0.0580 -1.7463 -39.3713
25.5600 -43.3600 -0.9438 -0.2834
0.1001 -0.2027 -0.3307 -0.2127 -0.4324
0.0622 -1.7464 -39.3734
25.5800 -41.2400 1.0314 -0.0443
0.0788 -0.2897 -0.3588 -0.2230 -0.4272
0.0665 -1.7465 -39.3756
25.6000 -43.1300 -0.9250 0.2836
0.0327 -0.3693 -0.3816 -0.2321 -0.4217
0.0707 -1.7465 -39.3777
25.6200 -41.5800 0.8641 0.1977 -
0.0657 -0.4351 -0.3983 -0.2399 -0.4159
0.0750 -1.7466 -39.3799
25.6400 -43.7600 -0.7337 -0.1121 -
0.2151 -0.4815 -0.4085 -0.2463 -0.4099
0.0793 -1.7466 -39.3820
25.6600 -42.8000 0.2802 -0.0997 -
0.3816 -0.5044 -0.4116 -0.2513 -0.4036
0.0836 -1.7467 -39.3842
25.6800 -42.6800 0.3345 0.0641 -
0.5018 -0.5005 -0.4073 -0.2548 -0.3970
0.0880 -1.7467 -39.3863
25.7000 -43.2500 -0.1457 -0.0436 -
0.5139 -0.4675 -0.3958 -0.2569 -0.3902
0.0923 -1.7468 -39.3885
25.7200 -42.9200 0.3257 -0.3916 -
0.3999 -0.4068 -0.3775 -0.2576 -0.3831
0.0966 -1.7468 -39.3906
25.7400 -43.6800 -0.9107 -0.2643 -
0.1853 -0.3246 -0.3533 -0.2569 -0.3758
0.1010 -1.7469 -39.3927
25.7600 -40.8200 0.9937 0.3168
0.0695 -0.2286 -0.3238 -0.2549 -0.3682
0.1054 -1.7469 -39.3949
25.7800 -41.1200 0.1727 0.4899
0.2907 -0.1268 -0.2898 -0.2517 -0.3603
0.1098 -1.7469 -39.3970
25.8000 -42.3400 -1.0049 0.1423
0.4263 -0.0271 -0.2520 -0.2474 -0.3523
0.1141 -1.7470 -39.3991
25.8200 -40.7100 0.9567 -0.3513
0.4738 0.0623 -0.2114 -0.2421 -0.3440
0.1185 -1.7470 -39.4013
25.8400 -42.4700 -0.9771 -0.3376
0.4595 0.1341 -0.1686 -0.2357 -0.3354
0.1229 -1.7470 -39.4034
25.8600 -40.5200 0.5610 0.0298
0.4212 0.1830 -0.1245 -0.2285 -0.3267
0.1273 -1.7471 -39.4055

25.8800 -39.9000 0.8297 0.2531
0.3879 0.2062 -0.0798 -0.2204 -0.3178
0.1317 -1.7471 -39.4076
25.9000 -41.7000 -0.8417 0.1042
0.3699 0.2039 -0.0352 -0.2117 -0.3086
0.1361 -1.7471 -39.4098
25.9200 -41.4100 -0.2968 -0.0416
0.3486 0.1800 0.0087 -0.2022 -0.2992
0.1405 -1.7471 -39.4119
25.9400 -40.8800 0.0683 0.0609
0.2975 0.1399 0.0516 -0.1921 -0.2897
0.1449 -1.7471 -39.4140
25.9600 -40.5300 0.4603 0.1733
0.2029 0.0904 0.0930 -0.1816 -0.2800
0.1493 -1.7472 -39.4161
25.9800 -40.8800 0.3007 0.0345
0.0625 0.0379 0.1323 -0.1705 -0.2701
0.1536 -1.7472 -39.4182
26.0000 -41.4600 0.1775 -0.2905 -
0.1098 -0.0117 0.1692 -0.1592 -0.2600
0.1580 -1.7472 -39.4203
26.0200 -42.7400 -0.7935 -0.3772 -
0.2933 -0.0531 0.2031 -0.1475 -0.2497
0.1624 -1.7472 -39.4224
26.0400 -41.3900 0.3239 0.0115 -
0.4583 -0.0819 0.2338 -0.1357 -0.2393
0.1667 -1.7472 -39.4246
26.0600 -40.9200 0.4820 0.4179 -
0.5723 -0.0945 0.2608 -0.1237 -0.2288
0.1711 -1.7472 -39.4267
26.0800 -41.7600 -0.3198 0.3407 -
0.6060 -0.0885 0.2838 -0.1116 -0.2181
0.1754 -1.7471 -39.4288
26.1000 -41.4000 0.5152 -0.2670 -
0.5407 -0.0647 0.3029 -0.0996 -0.2072
0.1798 -1.7471 -39.4309
26.1200 -42.7100 -0.7700 -0.6316 -
0.3726 -0.0262 0.3179 -0.0876 -0.1962
0.1841 -1.7471 -39.4330
26.1400 -40.6600 0.7362 -0.3265 -
0.1196 0.0219 0.3288 -0.0758 -0.1851
0.1884 -1.7471 -39.4351
26.1600 -40.9700 -0.4668 0.1273
0.1636 0.0738 0.3356 -0.0641 -0.1739
0.1927 -1.7470 -39.4372
26.1800 -40.0900 -0.1991 0.4426
0.4051 0.1235 0.3383 -0.0528 -0.1626
0.1969 -1.7470 -39.4393
26.2000 -39.6800 -0.2564 0.6774
0.5339 0.1650 0.3370 -0.0417 -0.1512
0.2012 -1.7470 -39.4414

26.2200 -38.7400 0.8057 0.4840
0.5261 0.1934 0.3318 -0.0309 -0.1396
0.2054 -1.7469 -39.4435
26.2400 -40.8600 -0.5660 -0.1751
0.4107 0.2071 0.3227 -0.0206 -0.1280
0.2096 -1.7469 -39.4456
26.2600 -41.4500 -0.4359 -0.6464
0.2463 0.2066 0.3100 -0.0106 -0.1163
0.2138 -1.7468 -39.4477
26.2800 -40.6900 0.3622 -0.5688
0.0897 0.1927 0.2939 -0.0011 -0.1045
0.2180 -1.7468 -39.4497
26.3000 -40.9600 -0.0964 -0.1440 -
0.0321 0.1668 0.2749 0.0080 -0.0927
0.2222 -1.7467 -39.4518
26.3200 -40.3700 0.2884 0.2144 -
0.1125 0.1316 0.2532 0.0167 -0.0807
0.2263 -1.7466 -39.4539
26.3400 -40.5900 -0.1425 0.3809 -
0.1593 0.0906 0.2293 0.0249 -0.0687
0.2304 -1.7466 -39.4560
26.3600 -40.3800 0.1291 0.3255 -
0.1856 0.0478 0.2035 0.0327 -0.0567
0.2344 -1.7465 -39.4581
26.3800 -40.8700 0.0849 0.0565 -
0.2047 0.0077 0.1761 0.0400 -0.0446
0.2385 -1.7464 -39.4602
26.4000 -41.2400 -0.0003 -0.2347 -
0.2206 -0.0261 0.1476 0.0468 -0.0325
0.2425 -1.7463 -39.4623
26.4200 -41.6800 -0.4050 -0.2691 -
0.2317 -0.0508 0.1182 0.0532 -0.0203
0.2465 -1.7462 -39.4643
26.4400 -41.1100 0.0008 0.0062 -
0.2322 -0.0656 0.0884 0.0591 -0.0082
0.2504 -1.7461 -39.4664
26.4600 -40.5100 0.3556 0.2569 -
0.2187 -0.0715 0.0585 0.0646 0.0041
0.2543 -1.7460 -39.4685
26.4800 -41.0400 -0.1061 0.2105 -
0.1899 -0.0706 0.0290 0.0697 0.0163
0.2582 -1.7459 -39.4706
26.5000 -40.9200 0.1777 -0.0836 -
0.1371 -0.0661 0.0003 0.0744 0.0285
0.2620 -1.7458 -39.4726
26.5200 -41.4500 -0.2263 -0.2961 -
0.0517 -0.0614 -0.0273 0.0786 0.0407
0.2658 -1.7456 -39.4747
26.5400 -41.2300 -0.1347 -0.2212
0.0645 -0.0598 -0.0532 0.0826 0.0530
0.2696 -1.7455 -39.4768

26.5600 -40.4900 0.1923 0.0380
0.1927 -0.0639 -0.0773 0.0861 0.0652
0.2733 -1.7454 -39.4788
26.5800 -40.4700 0.0092 0.2057
0.3025 -0.0760 -0.0992 0.0893 0.0774
0.2770 -1.7452 -39.4809
26.6000 -40.5600 -0.0487 0.1248
0.3592 -0.0969 -0.1189 0.0922 0.0896
0.2807 -1.7451 -39.4830
26.6200 -40.1900 0.4263 -0.0687
0.3380 -0.1260 -0.1365 0.0949 0.1017
0.2843 -1.7449 -39.4850
26.6400 -41.5100 -0.6839 -0.0525
0.2339 -0.1599 -0.1520 0.0972 0.1138
0.2878 -1.7447 -39.4871
26.6600 -41.4900 -0.6031 0.1716
0.0686 -0.1941 -0.1657 0.0994 0.1259
0.2914 -1.7446 -39.4891
26.6800 -40.0400 1.0513 0.2035 -
0.1146 -0.2227 -0.1778 0.1013 0.1379
0.2948 -1.7444 -39.4912
26.7000 -42.5400 -0.9778 -0.2207 -
0.2670 -0.2397 -0.1886 0.1031 0.1498
0.2982 -1.7442 -39.4932
26.7200 -41.5600 0.2244 -0.3941 -
0.3475 -0.2394 -0.1982 0.1048 0.1617
0.3016 -1.7440 -39.4953
26.7400 -41.7700 -0.4492 0.0189 -
0.3412 -0.2181 -0.2068 0.1063 0.1735
0.3049 -1.7438 -39.4973
26.7600 -40.2800 0.7180 0.2737 -
0.2749 -0.1757 -0.2146 0.1078 0.1852
0.3082 -1.7436 -39.4994
26.7800 -41.0700 0.0683 0.0624 -
0.1838 -0.1142 -0.2218 0.1092 0.1969
0.3114 -1.7434 -39.5014
26.8000 -41.8500 -0.6927 -0.1670 -
0.0984 -0.0369 -0.2285 0.1105 0.2084
0.3146 -1.7431 -39.5035
26.8200 -40.4200 0.4174 -0.0737 -
0.0320 0.0511 -0.2349 0.1119 0.2199
0.3177 -1.7429 -39.5055
26.8400 -40.5900 0.0795 0.0539
0.0309 0.1427 -0.2413 0.1132 0.2312
0.3207 -1.7427 -39.5076
26.8600 -40.1000 0.5319 -0.1316
0.1121 0.2301 -0.2477 0.1145 0.2425
0.3237 -1.7424 -39.5096
26.8800 -41.2800 -0.7299 -0.2302
0.2199 0.3060 -0.2543 0.1158 0.2536
0.3267 -1.7422 -39.5116

26.9000 -39.4200 0.6178 0.0267
0.3400 0.3657 -0.2613 0.1172 0.2646
0.3295 -1.7419 -39.5137
26.9200 -39.9800 -0.2662 0.2321
0.4305 0.4059 -0.2687 0.1185 0.2755
0.3323 -1.7416 -39.5157
26.9400 -39.5800 0.1070 0.2599
0.4422 0.4249 -0.2767 0.1197 0.2863
0.3351 -1.7413 -39.5177
26.9600 -39.8800 -0.2209 0.3029
0.3405 0.4230 -0.2851 0.1209 0.2969
0.3378 -1.7411 -39.5198
26.9800 -39.8400 0.0883 0.3368
0.1361 0.4023 -0.2939 0.1220 0.3074
0.3404 -1.7408 -39.5218
27.0000 -40.2200 0.1365 0.2099 -
0.1216 0.3671 -0.3027 0.1230 0.3178
0.3429 -1.7404 -39.5238
27.0200 -41.1200 -0.2349 -0.0798 -
0.3673 0.3213 -0.3114 0.1237 0.3281
0.3454 -1.7401 -39.5259
27.0400 -41.2100 0.2752 -0.4017 -
0.5411 0.2690 -0.3198 0.1242 0.3382
0.3478 -1.7398 -39.5279
27.0600 -42.1400 -0.3874 -0.5713 -
0.5925 0.2140 -0.3274 0.1244 0.3482
0.3502 -1.7395 -39.5299
27.0800 -41.6200 -0.0617 -0.4553 -
0.4980 0.1587 -0.3339 0.1243 0.3580
0.3524 -1.7391 -39.5319
27.1000 -40.4800 0.5841 -0.1110 -
0.2828 0.1036 -0.3387 0.1237 0.3677
0.3546 -1.7388 -39.5339
27.1200 -41.1700 -0.7282 0.3149 -
0.0026 0.0481 -0.3412 0.1228 0.3772
0.3567 -1.7384 -39.5360
27.1400 -39.2200 0.7293 0.5502
0.2760 -0.0084 -0.3408 0.1213 0.3866
0.3588 -1.7381 -39.5380
27.1600 -40.2300 -0.2430 0.3844
0.4932 -0.0662 -0.3367 0.1193 0.3959
0.3608 -1.7377 -39.5400
27.1800 -40.4900 -0.2489 0.0817
0.6127 -0.1258 -0.3283 0.1167 0.4049
0.3626 -1.7373 -39.5420
27.2000 -40.7500 -0.4469 0.0397
0.6214 -0.1875 -0.3151 0.1136 0.4139
0.3644 -1.7369 -39.5440
27.2200 -39.9700 0.1650 0.1762
0.5445 -0.2518 -0.2966 0.1099 0.4227
0.3662 -1.7365 -39.5460

27.2400 -39.5900 0.7719 0.1244
0.4348 -0.3190 -0.2723 0.1055 0.4313
0.3678 -1.7361 -39.5480
27.2600 -40.9200 -0.0662 -0.2361
0.3364 -0.3891 -0.2421 0.1006 0.4397
0.3694 -1.7356 -39.5500
27.2800 -42.2300 -0.8621 -0.5602
0.2620 -0.4605 -0.2060 0.0950 0.4480
0.3708 -1.7352 -39.5520
27.3000 -40.6300 0.7986 -0.5000
0.2096 -0.5301 -0.1641 0.0887 0.4562
0.3722 -1.7348 -39.5540
27.3200 -41.4600 -0.3540 -0.1386
0.1579 -0.5926 -0.1166 0.0819 0.4642
0.3735 -1.7343 -39.5560
27.3400 -41.3400 -0.4947 0.2546
0.0754 -0.6418 -0.0638 0.0745 0.4720
0.3747 -1.7338 -39.5580
27.3600 -39.8000 0.7367 0.4904 -
0.0686 -0.6723 -0.0061 0.0666 0.4796
0.3758 -1.7334 -39.5600
27.3800 -41.1900 -0.4456 0.5271 -
0.2876 -0.6801 0.0560 0.0582 0.4870
0.3769 -1.7329 -39.5620
27.4000 -41.7100 -0.5498 0.3059 -
0.5577 -0.6630 0.1218 0.0493 0.4943
0.3778 -1.7324 -39.5640
27.4200 -40.9400 0.9001 -0.1678 -
0.8230 -0.6203 0.1907 0.0400 0.5014
0.3786 -1.7319 -39.5660
27.4400 -43.1300 -0.8618 -0.5542 -
1.0159 -0.5513 0.2618 0.0304 0.5084
0.3794 -1.7314 -39.5680
27.4600 -41.7300 0.2618 -0.3960 -
1.0823 -0.4559 0.3340 0.0203 0.5151
0.3800 -1.7308 -39.5700
27.4800 -41.1100 0.1778 0.0263 -
1.0188 -0.3362 0.4062 0.0100 0.5216
0.3806 -1.7303 -39.5720
27.5000 -40.6585 0.0616 0.2418 -
0.8543 -0.1972 0.4771 -0.0006 0.5280
0.3810 -1.7297 -39.5739
27.5200 -40.2872 0.0180 0.1798 -
0.6259 -0.0447 0.5454 -0.0114 0.5341
0.3814 -1.7292 -39.5759
27.5400 -39.9428 -0.0547 0.0586 -
0.3721 0.1149 0.6099 -0.0224 0.5400
0.3816 -1.7286 -39.5779
27.5600 -39.5715 -0.0036 0.0303 -
0.1174 0.2750 0.6694 -0.0335 0.5457
0.3818 -1.7280 -39.5799

27.5800 -39.1200 -0.0817 0.0798
0.1316 0.4286 0.7225 -0.0448 0.5512
0.3818 -1.7274 -39.5819
27.6000 -38.5000 0.1223 0.0809
0.3796 0.5685 0.7681 -0.0561 0.5565
0.3818 -1.7268 -39.5838
27.6200 -38.3500 0.0895 -0.0825
0.6277 0.6875 0.8048 -0.0675 0.5615
0.3816 -1.7262 -39.5858
27.6400 -38.1100 0.1529 -0.3258
0.8575 0.7790 0.8315 -0.0789 0.5663
0.3813 -1.7256 -39.5878
27.6600 -38.5800 -0.4988 -0.3558
1.0394 0.8369 0.8470 -0.0902 0.5708
0.3810 -1.7249 -39.5898
27.6800 -37.4400 0.2521 -0.0161
1.1438 0.8563 0.8500 -0.1015 0.5751
0.3805 -1.7243 -39.5917
27.7000 -37.0600 0.1596 0.4745
1.1413 0.8339 0.8400 -0.1128 0.5792
0.3799 -1.7236 -39.5937
27.7200 -37.3500 -0.2180 0.7922
1.0027 0.7709 0.8173 -0.1238 0.5829
0.3792 -1.7230 -39.5957
27.7400 -37.3200 0.3103 0.6580
0.7182 0.6735 0.7827 -0.1347 0.5864
0.3784 -1.7223 -39.5976
27.7600 -38.3300 0.4698 0.0572
0.3369 0.5492 0.7371 -0.1452 0.5897
0.3775 -1.7216 -39.5996
27.7800 -40.5900 -0.5431 -0.6195 -
0.0611 0.4056 0.6813 -0.1555 0.5926
0.3765 -1.7209 -39.6015
27.8000 -41.2200 -0.3664 -0.8601 -
0.3927 0.2502 0.6163 -0.1653 0.5953
0.3754 -1.7202 -39.6035
27.8200 -40.5700 0.5637 -0.6041 -
0.5905 0.0908 0.5430 -0.1747 0.5977
0.3741 -1.7194 -39.6054
27.8400 -40.9000 0.0862 -0.2442 -
0.6303 -0.0653 0.4624 -0.1835 0.5997
0.3728 -1.7187 -39.6074
27.8600 -40.9300 0.0325 -0.0137 -
0.5373 -0.2107 0.3754 -0.1917 0.6015
0.3713 -1.7179 -39.6094
27.8800 -41.2400 -0.5378 0.2892 -
0.3858 -0.3377 0.2828 -0.1992 0.6030
0.3698 -1.7172 -39.6113
27.9000 -40.5000 -0.1188 0.6971 -
0.2527 -0.4379 0.1857 -0.2060 0.6041
0.3681 -1.7164 -39.6132

27.9200 -39.6000 0.7375 0.8548 -
0.1848 -0.5046 0.0849 -0.2120 0.6050
0.3663 -1.7156 -39.6152
27.9400 -40.9900 -0.0589 0.4954 -
0.2035 -0.5345 -0.0186 -0.2171 0.6055
0.3644 -1.7148 -39.6171
27.9600 -42.3100 -0.6463 -0.0525 -
0.2819 -0.5296 -0.1238 -0.2212 0.6057
0.3624 -1.7140 -39.6191
27.9800 -41.7500 0.3171 -0.3932 -
0.3765 -0.4944 -0.2296 -0.2244 0.6056
0.3603 -1.7132 -39.6210
28.0000 -42.1900 0.0773 -0.4780 -
0.4289 -0.4339 -0.3348 -0.2264 0.6051
0.3581 -1.7123 -39.6230
28.0200 -42.2600 0.1008 -0.5476 -
0.3861 -0.3535 -0.4381 -0.2273 0.6043
0.3558 -1.7115 -39.6249
28.0400 -42.0700 0.1779 -0.5981 -
0.2385 -0.2596 -0.5380 -0.2270 0.6031
0.3534 -1.7106 -39.6268
28.0600 -42.2500 -0.4779 -0.3849 -
0.0174 -0.1588 -0.6333 -0.2255 0.6016
0.3509 -1.7098 -39.6288
28.0800 -40.8100 0.2332 0.1582
0.2217 -0.0570 -0.7224 -0.2226 0.5998
0.3483 -1.7089 -39.6307
28.1000 -40.0600 0.1674 0.6426
0.4149 0.0398 -0.8039 -0.2184 0.5976
0.3455 -1.7080 -39.6326
28.1200 -40.1500 0.0235 0.7178
0.5046 0.1255 -0.8765 -0.2126 0.5951
0.3427 -1.7071 -39.6346
28.1400 -40.4400 0.2355 0.3726
0.4662 0.1947 -0.9388 -0.2054 0.5922
0.3398 -1.7061 -39.6365
28.1600 -41.8700 -0.7020 -0.1279
0.3238 0.2441 -0.9896 -0.1966 0.5890
0.3368 -1.7052 -39.6384
28.1800 -40.9500 0.9168 -0.5660
0.1178 0.2726 -1.0279 -0.1862 0.5854
0.3337 -1.7043 -39.6403
28.2000 -43.0600 -0.9793 -0.5406 -
0.1061 0.2792 -1.0532 -0.1741 0.5814
0.3305 -1.7033 -39.6423
28.2200 -41.8700 -0.2101 0.0670 -
0.2925 0.2637 -1.0653 -0.1604 0.5771
0.3272 -1.7023 -39.6442
28.2400 -40.1900 1.0111 0.5903 -
0.3794 0.2261 -1.0641 -0.1450 0.5724
0.3238 -1.7014 -39.6461

28.2600 -42.4700 -0.9420 0.3346 -
0.3249 0.1670 -1.0497 -0.1279 0.5674
0.3203 -1.7004 -39.6480
28.2800 -41.0800 0.8786 -0.3866 -
0.1519 0.0885 -1.0224 -0.1094 0.5620
0.3167 -1.6994 -39.6499
28.3000 -42.6900 -0.6793 -0.5542
0.0821 -0.0061 -0.9826 -0.0894 0.5562
0.3130 -1.6983 -39.6518
28.3200 -41.8200 -0.5713 -0.0504
0.3057 -0.1130 -0.9310 -0.0682 0.5501
0.3093 -1.6973 -39.6537
28.3400 -39.8800 0.8796 0.5054
0.4343 -0.2280 -0.8682 -0.0459 0.5437
0.3054 -1.6963 -39.6557
28.3600 -41.6400 -0.6442 0.3478
0.4024 -0.3465 -0.7951 -0.0225 0.5368
0.3015 -1.6952 -39.6576
28.3800 -41.0800 0.5110 -0.1908
0.2326 -0.4628 -0.7128 0.0016 0.5296
0.2975 -1.6941 -39.6595
28.4000 -42.7500 -0.8118 -0.2039
0.0086 -0.5705 -0.6224 0.0265 0.5221
0.2934 -1.6930 -39.6614
28.4200 -41.0100 0.8359 0.0017 -
0.1702 -0.6631 -0.5250 0.0519 0.5142
0.2893 -1.6920 -39.6633
28.4400 -42.7300 -0.7432 -0.1793 -
0.2407 -0.7337 -0.4219 0.0776 0.5060
0.2850 -1.6908 -39.6652
28.4600 -41.5100 0.4563 -0.2452 -
0.2315 -0.7750 -0.3143 0.1037 0.4974
0.2807 -1.6897 -39.6671
28.4800 -41.8500 -0.4367 0.1614 -
0.2050 -0.7810 -0.2032 0.1297 0.4884
0.2763 -1.6886 -39.6690
28.5000 -40.4700 0.4871 0.5114 -
0.2008 -0.7478 -0.0900 0.1558 0.4792
0.2718 -1.6874 -39.6709
28.5200 -41.1800 -0.2867 0.3708 -
0.2331 -0.6750 0.0241 0.1816 0.4695
0.2673 -1.6863 -39.6728
28.5400 -41.2600 0.0887 -0.1500 -
0.2796 -0.5654 0.1380 0.2070 0.4595
0.2627 -1.6851 -39.6746
28.5600 -40.9200 0.6168 -0.6195 -
0.2974 -0.4245 0.2503 0.2319 0.4492
0.2580 -1.6839 -39.6765
28.5800 -42.1300 -0.9785 -0.5614 -
0.2501 -0.2588 0.3595 0.2561 0.4386
0.2532 -1.6827 -39.6784

28.6000 -39.3900 0.8067 -0.0108 -
0.1413 -0.0766 0.4644 0.2794 0.4276
0.2484 -1.6815 -39.6803
28.6200 -38.7600 0.5592 0.4302
0.0017 0.1134 0.5635 0.3018 0.4163
0.2435 -1.6803 -39.6822
28.6400 -39.4900 -0.7173 0.5441
0.1403 0.3022 0.6556 0.3231 0.4046
0.2386 -1.6791 -39.6841
28.6600 -38.1300 0.3570 0.4867
0.2413 0.4810 0.7392 0.3430 0.3926
0.2335 -1.6778 -39.6860
28.6800 -38.3000 -0.0590 0.3923
0.2960 0.6417 0.8130 0.3615 0.3803
0.2285 -1.6766 -39.6878
28.7000 -38.3000 -0.1030 0.0656
0.3234 0.7769 0.8756 0.3784 0.3677
0.2233 -1.6753 -39.6897
28.7200 -38.2200 0.5525 -0.4898
0.3510 0.8800 0.9257 0.3936 0.3548
0.2182 -1.6740 -39.6916
28.7400 -39.7300 -0.7484 -0.8315
0.3952 0.9456 0.9618 0.4068 0.3415
0.2129 -1.6727 -39.6935
28.7600 -38.2700 0.2784 -0.5690
0.4596 0.9699 0.9829 0.4180 0.3279
0.2076 -1.6714 -39.6953
28.7800 -37.3800 0.5019 -0.0108
0.5235 0.9519 0.9885 0.4271 0.3141
0.2022 -1.6701 -39.6972
28.8000 -37.3900 0.2114 0.4452
0.5547 0.8928 0.9792 0.4340 0.2999
0.1968 -1.6687 -39.6991
28.8200 -38.1900 -0.7084 0.6884
0.5155 0.7961 0.9562 0.4386 0.2854
0.1914 -1.6674 -39.7009
28.8400 -37.2800 0.4746 0.6390
0.3909 0.6671 0.9210 0.4411 0.2707
0.1859 -1.6660 -39.7028
28.8600 -38.3900 0.2472 0.2249
0.2040 0.5129 0.8749 0.4414 0.2557
0.1803 -1.6646 -39.7047
28.8800 -40.0300 -0.4031 -0.3380
0.0020 0.3404 0.8192 0.4397 0.2404
0.1747 -1.6632 -39.7065
28.9000 -40.1900 0.1805 -0.6348 -
0.1651 0.1566 0.7554 0.4360 0.2248
0.1691 -1.6618 -39.7084
28.9200 -40.9400 -0.2585 -0.4898 -
0.2609 -0.0310 0.6849 0.4304 0.2090
0.1634 -1.6604 -39.7102

28.9400 -40.4100 0.0265 -0.0420 -
0.2841 -0.2145 0.6089 0.4229 0.1930
0.1576 -1.6590 -39.7121
28.9600 -40.4800 -0.0965 0.3869 -
0.2606 -0.3851 0.5289 0.4136 0.1768
0.1519 -1.6575 -39.7139
28.9800 -40.0300 0.6284 0.3672 -
0.2280 -0.5343 0.4462 0.4027 0.1603
0.1460 -1.6561 -39.7158
29.0000 -41.9100 -0.7430 0.0131 -
0.2262 -0.6540 0.3621 0.3901 0.1437
0.1402 -1.6546 -39.7176
29.0200 -41.0600 0.5421 -0.0892 -
0.2763 -0.7393 0.2782 0.3759 0.1269
0.1343 -1.6531 -39.7195
29.0400 -41.5600 0.1626 0.0035 -
0.3653 -0.7876 0.1955 0.3603 0.1100
0.1284 -1.6516 -39.7213
29.0600 -42.0300 -0.1289 0.0112 -
0.4605 -0.7985 0.1154 0.3432 0.0929
0.1224 -1.6501 -39.7232
29.0800 -42.2200 0.0344 -0.0689 -
0.5223 -0.7727 0.0387 0.3248 0.0757
0.1165 -1.6486 -39.7250
29.1000 -42.6600 -0.4963 -0.0302 -
0.5164 -0.7125 -0.0340 0.3051 0.0584
0.1104 -1.6470 -39.7269
29.1200 -41.7900 0.2134 0.0733 -
0.4194 -0.6228 -0.1022 0.2842 0.0410
0.1044 -1.6455 -39.7287
29.1400 -41.2400 0.6824 -0.0466 -
0.2232 -0.5106 -0.1657 0.2621 0.0236
0.0983 -1.6439 -39.7305
29.1600 -42.7900 -0.7991 -0.4196
0.0467 -0.3836 -0.2240 0.2391 0.0060
0.0922 -1.6423 -39.7324
29.1800 -41.5800 0.3046 -0.5727
0.3288 -0.2496 -0.2768 0.2150 -0.0116
0.0861 -1.6407 -39.7342
29.2000 -40.6300 0.4988 -0.1969
0.5579 -0.1163 -0.3238 0.1901 -0.0292
0.0800 -1.6391 -39.7360
29.2200 -40.9700 -0.7337 0.5135
0.6751 0.0088 -0.3646 0.1645 -0.0468
0.0738 -1.6375 -39.7379
29.2400 -39.3300 0.7728 0.8435
0.6291 0.1193 -0.3990 0.1382 -0.0644
0.0676 -1.6359 -39.7397
29.2600 -41.3600 -0.5788 0.3517
0.4063 0.2121 -0.4267 0.1113 -0.0820
0.0615 -1.6342 -39.7415

29.2800 -41.4600 0.2796 -0.3881
0.0890 0.2864 -0.4479 0.0840 -0.0996
0.0552 -1.6326 -39.7433
29.3000 -42.7300 -0.2775 -0.7213 -
0.2184 0.3419 -0.4627 0.0564 -0.1172
0.0490 -1.6309 -39.7452
29.3200 -42.1700 0.3977 -0.5504 -
0.4299 0.3788 -0.4713 0.0285 -0.1346
0.0428 -1.6292 -39.7470
29.3400 -42.7900 -0.6660 -0.0354 -
0.5101 0.3973 -0.4744 0.0005 -0.1520
0.0365 -1.6275 -39.7488
29.3600 -40.9500 0.8076 0.3304 -
0.4626 0.3977 -0.4722 -0.0275 -0.1694
0.0302 -1.6258 -39.7506
29.3800 -42.4600 -0.7594 0.3213 -
0.3207 0.3813 -0.4655 -0.0553 -0.1866
0.0240 -1.6240 -39.7524
29.4000 -41.7300 -0.1124 0.2703 -
0.1410 0.3505 -0.4546 -0.0828 -0.2037
0.0177 -1.6223 -39.7542
29.4200 -41.0000 0.6201 0.1983
0.0387 0.3076 -0.4400 -0.1100 -0.2206
0.0114 -1.6205 -39.7561
29.4400 -41.6500 0.0778 -0.0743
0.2038 0.2539 -0.4219 -0.1366 -0.2374
0.0051 -1.6187 -39.7579
29.4600 -42.6300 -0.5475 -0.3934
0.3418 0.1906 -0.4007 -0.1627 -0.2540
-0.0012 -1.6170 -39.7597
29.4800 -41.5000 0.6112 -0.4252
0.4328 0.1193 -0.3769 -0.1880 -0.2705
-0.0075 -1.6152 -39.7615
29.5000 -42.3900 -0.6869 -0.0016
0.4530 0.0426 -0.3509 -0.2125 -0.2867
-0.0138 -1.6133 -39.7633
29.5200 -41.1100 0.2538 0.4683
0.3875 -0.0358 -0.3232 -0.2360 -0.3027
-0.0201 -1.6115 -39.7651
29.5400 -40.9500 0.7325 0.4948
0.2341 -0.1115 -0.2942 -0.2585 -0.3185
-0.0264 -1.6097 -39.7669
29.5600 -43.0300 -0.5481 -0.0352
0.0229 -0.1796 -0.2646 -0.2799 -0.3341
-0.0327 -1.6078 -39.7687
29.5800 -43.4400 -0.0612 -0.5025 -
0.1916 -0.2352 -0.2348 -0.3000 -0.3493
-0.0389 -1.6059 -39.7705
29.6000 -43.8400 -0.5542 -0.3675 -
0.3570 -0.2738 -0.2052 -0.3188 -0.3644
-0.0452 -1.6040 -39.7723

29.6200 -42.6600 0.1896 0.0959 -
0.4507 -0.2916 -0.1762 -0.3360 -0.3791
-0.0515 -1.6021 -39.7741
29.6400 -42.2700 0.4527 0.3673 -
0.4660 -0.2864 -0.1482 -0.3517 -0.3935
-0.0577 -1.6002 -39.7759
29.6600 -42.8600 -0.1151 0.2186 -
0.4039 -0.2587 -0.1215 -0.3658 -0.4076
-0.0640 -1.5983 -39.7777
29.6800 -43.4400 -0.4877 -0.1013 -
0.2802 -0.2112 -0.0963 -0.3781 -0.4213
-0.0702 -1.5963 -39.7794
29.7000 -42.3500 0.6271 -0.3117 -
0.1156 -0.1486 -0.0730 -0.3886 -0.4347
-0.0764 -1.5944 -39.7812
29.7200 -43.2800 -0.6311 -0.2447
0.0714 -0.0773 -0.0520 -0.3972 -0.4477
-0.0826 -1.5924 -39.7830
29.7400 -42.1200 0.0637 0.0276
0.2539 -0.0034 -0.0335 -0.4039 -0.4604
-0.0888 -1.5904 -39.7848
29.7600 -41.0900 0.5242 0.2597
0.4007 0.0668 -0.0176 -0.4087 -0.4726
-0.0950 -1.5884 -39.7866
29.7800 -41.5200 0.1155 0.2369
0.4797 0.1276 -0.0045 -0.4116 -0.4845
-0.1011 -1.5864 -39.7884
29.8000 -42.2400 -0.6149 0.0718
0.4722 0.1739 0.0056 -0.4126 -0.4959
-0.1072 -1.5844 -39.7901
29.8200 -41.7400 0.1318 -0.0458
0.3869 0.2037 0.0126 -0.4117 -0.5069
-0.1133 -1.5823 -39.7919
29.8400 -41.7500 0.3610 -0.0901
0.2474 0.2174 0.0166 -0.4091 -0.5175
-0.1194 -1.5803 -39.7937
29.8600 -42.5800 -0.4065 -0.1121
0.0826 0.2168 0.0177 -0.4047 -0.5276
-0.1254 -1.5782 -39.7954
29.8800 -42.1400 0.2849 -0.0578 -
0.0801 0.2042 0.0162 -0.3987 -0.5372
-0.1314 -1.5761 -39.7972
29.9000 -42.9000 -0.4732 0.1190 -
0.2194 0.1819 0.0123 -0.3911 -0.5464
-0.1374 -1.5740 -39.7990
29.9200 -42.0900 0.3068 0.1768 -
0.3219 0.1526 0.0065 -0.3821 -0.5551
-0.1434 -1.5719 -39.8008
29.9400 -42.1600 0.5188 -0.0183 -
0.3770 0.1185 -0.0010 -0.3717 -0.5633
-0.1493 -1.5697 -39.8025

29.9600 -43.7300 -0.7170 -0.2221 -
0.3652 0.0813 -0.0098 -0.3602 -0.5710
-0.1552 -1.5676 -39.8043
29.9800 -42.1000 0.6436 -0.0305 -
0.2728 0.0425 -0.0193 -0.3475 -0.5783
-0.1610 -1.5654 -39.8060
30.0000 -43.0300 -0.6036 0.1777 -
0.1200 0.0034 -0.0292 -0.3338 -0.5850
-0.1668 -1.5632 -39.8078
30.0200 -41.8000 0.8370 -0.0163
0.0548 -0.0348 -0.0387 -0.3192 -0.5913
-0.1726 -1.5610 -39.8096
30.0400 -43.1500 -0.5828 -0.1680
0.2054 -0.0712 -0.0475 -0.3038 -0.5971
-0.1784 -1.5588 -39.8113
30.0600 -43.2700 -1.0588 -0.0273
0.2946 -0.1051 -0.0551 -0.2878 -0.6024
-0.1840 -1.5566 -39.8131
30.0800 -41.8200 0.1216 0.2888
0.3101 -0.1358 -0.0609 -0.2712 -0.6072
-0.1897 -1.5544 -39.8148
30.1000 -40.5700 1.5095 0.3040
0.2630 -0.1625 -0.0646 -0.2542 -0.6115
-0.1953 -1.5521 -39.8166
30.1200 -44.1500 -1.4433 -0.1884
0.1768 -0.1844 -0.0658 -0.2368 -0.6153
-0.2009 -1.5499 -39.8183
30.1400 -42.4200 0.7058 -0.4455
0.0753 -0.2008 -0.0643 -0.2192 -0.6187
-0.2064 -1.5476 -39.8201
30.1600 -42.2100 0.6538 -0.1841 -
0.0255 -0.2110 -0.0598 -0.2015 -0.6215
-0.2118 -1.5453 -39.8218
30.1800 -42.7700 -0.3401 0.2738 -
0.1231 -0.2138 -0.0522 -0.1837 -0.6238
-0.2173 -1.5430 -39.8235
30.2000 -42.5600 -0.1801 0.4443 -
0.2176 -0.2079 -0.0415 -0.1660 -0.6257
-0.2226 -1.5406 -39.8253
30.2200 -42.2100 0.5056 0.2153 -
0.3032 -0.1924 -0.0277 -0.1484 -0.6270
-0.2279 -1.5383 -39.8270
30.2400 -43.6600 -0.5762 -0.1865 -
0.3644 -0.1671 -0.0111 -0.1310 -0.6279
-0.2332 -1.5359 -39.8288
30.2600 -43.0900 0.1164 -0.4028 -
0.3829 -0.1329 0.0081 -0.1139 -0.6282
-0.2384 -1.5335 -39.8305
30.2800 -42.5100 0.6045 -0.3683 -
0.3446 -0.0912 0.0294 -0.0972 -0.6281
-0.2435 -1.5312 -39.8322

30.3000 -43.3800 -0.6930 -0.0893 -
0.2481 -0.0446 0.0526 -0.0808 -0.6274
-0.2486 -1.5288 -39.8340
30.3200 -41.6200 0.2654 0.3389 -
0.1087 0.0045 0.0770 -0.0650 -0.6263
-0.2537 -1.5263 -39.8357
30.3400 -41.1900 0.3366 0.5674
0.0526 0.0533 0.1024 -0.0497 -0.6247
-0.2586 -1.5239 -39.8374
30.3600 -41.2900 0.2651 0.2907
0.2113 0.0987 0.1281 -0.0351 -0.6225
-0.2635 -1.5214 -39.8391
30.3800 -41.7900 0.1417 -0.2588
0.3506 0.1380 0.1539 -0.0211 -0.6199
-0.2684 -1.5190 -39.8409
30.4000 -42.7300 -0.7064 -0.5708
0.4563 0.1686 0.1793 -0.0079 -0.6169
-0.2732 -1.5165 -39.8426
30.4200 -41.1600 0.6279 -0.4265
0.5168 0.1883 0.2038 0.0044 -0.6133
-0.2779 -1.5140 -39.8443
30.4400 -41.3600 0.1034 -0.0688
0.5240 0.1960 0.2270 0.0159 -0.6092
-0.2826 -1.5115 -39.8460
30.4600 -41.7200 -0.5988 0.2941
0.4696 0.1923 0.2487 0.0264 -0.6047
-0.2871 -1.5090 -39.8477
30.4800 -40.6000 0.3117 0.5322
0.3411 0.1791 0.2684 0.0359 -0.5997
-0.2917 -1.5064 -39.8495
30.5000 -40.9000 0.2122 0.5130
0.1422 0.1593 0.2860 0.0444 -0.5943
-0.2961 -1.5039 -39.8512
30.5200 -41.7400 0.0075 0.1782 -
0.1012 0.1362 0.3011 0.0517 -0.5883
-0.3005 -1.5013 -39.8529
30.5400 -42.4600 -0.1662 -0.2546 -
0.3425 0.1131 0.3135 0.0579 -0.5820
-0.3049 -1.4987 -39.8546
30.5600 -42.7000 -0.0394 -0.4712 -
0.5320 0.0931 0.3230 0.0629 -0.5751
-0.3091 -1.4961 -39.8563
30.5800 -42.9700 -0.3072 -0.3705 -
0.6318 0.0793 0.3295 0.0667 -0.5678
-0.3133 -1.4935 -39.8580
30.6000 -42.3700 0.1388 -0.1122 -
0.6314 0.0737 0.3327 0.0693 -0.5601
-0.3174 -1.4908 -39.8597
30.6200 -41.8000 0.4590 0.0794 -
0.5427 0.0762 0.3326 0.0706 -0.5520
-0.3215 -1.4882 -39.8614

30.6400 -41.5500 0.6059 0.0296 -
0.3881 0.0856 0.3294 0.0707 -0.5434
-0.3255 -1.4855 -39.8631
30.6600 -42.8900 -0.9233 -0.0703 -
0.1995 0.0996 0.3231 0.0697 -0.5344
-0.3294 -1.4828 -39.8648
30.6800 -41.5500 0.0409 0.0578 -
0.0110 0.1157 0.3138 0.0676 -0.5250
-0.3332 -1.4801 -39.8665
30.7000 -40.5900 0.8203 0.2743
0.1524 0.1314 0.3017 0.0644 -0.5152
-0.3370 -1.4774 -39.8682
30.7200 -41.5600 -0.4796 0.3364
0.2778 0.1438 0.2871 0.0601 -0.5050
-0.3407 -1.4747 -39.8699
30.7400 -41.2800 -0.1877 0.2171
0.3658 0.1501 0.2700 0.0549 -0.4945
-0.3443 -1.4719 -39.8716
30.7600 -41.0000 0.1328 0.0299
0.4204 0.1472 0.2508 0.0489 -0.4836
-0.3479 -1.4691 -39.8733
30.7800 -41.2100 0.2628 -0.2341
0.4500 0.1318 0.2297 0.0420 -0.4723
-0.3514 -1.4664 -39.8750
30.8000 -41.8300 0.0251 -0.4605
0.4543 0.1020 0.2070 0.0343 -0.4608
-0.3548 -1.4636 -39.8766
30.8200 -42.4300 -0.5769 -0.3536
0.4218 0.0585 0.1829 0.0259 -0.4489
-0.3582 -1.4608 -39.8783
30.8400 -41.5800 -0.0773 0.1323
0.3438 0.0036 0.1579 0.0170 -0.4367
-0.3615 -1.4579 -39.8800
30.8600 -40.6000 0.5902 0.5579
0.2179 -0.0591 0.1320 0.0075 -0.4242
-0.3647 -1.4551 -39.8817
30.8800 -41.7300 -0.1662 0.4847
0.0514 -0.1256 0.1057 -0.0025 -0.4115
-0.3678 -1.4522 -39.8834
30.9000 -42.2600 0.0936 -0.0050 -
0.1239 -0.1914 0.0791 -0.0128 -0.3984
-0.3709 -1.4493 -39.8850
30.9200 -43.5200 -0.4221 -0.4480 -
0.2637 -0.2523 0.0525 -0.0235 -0.3852
-0.3739 -1.4464 -39.8867
30.9400 -43.3300 -0.1029 -0.5438 -
0.3272 -0.3037 0.0261 -0.0343 -0.3717
-0.3768 -1.4435 -39.8884
30.9600 -43.4000 -0.4111 -0.2448 -
0.3054 -0.3415 0.0001 -0.0453 -0.3580
-0.3797 -1.4406 -39.8901

30.9800 -41.9800 0.4545 0.2288 -
0.2284 -0.3622 -0.0253 -0.0562 -0.3440
-0.3825 -1.4377 -39.8917
31.0000 -41.8600 0.2962 0.4728 -
0.1386 -0.3633 -0.0499 -0.0671 -0.3299
-0.3852 -1.4347 -39.8934
31.0200 -42.1000 0.2271 0.2711 -
0.0720 -0.3427 -0.0735 -0.0778 -0.3156
-0.3878 -1.4317 -39.8951
31.0400 -42.8100 -0.1164 -0.1397 -
0.0480 -0.2993 -0.0961 -0.0883 -0.3012
-0.3904 -1.4287 -39.8967
31.0600 -43.4100 -0.5391 -0.3383 -
0.0738 -0.2343 -0.1179 -0.0983 -0.2866
-0.3929 -1.4257 -39.8984
31.0800 -42.9200 -0.3150 -0.1120 -
0.1409 -0.1516 -0.1390 -0.1077 -0.2718
-0.3953 -1.4227 -39.9000
31.1000 -41.8400 0.5450 0.2423 -
0.2117 -0.0571 -0.1595 -0.1165 -0.2570
-0.3976 -1.4197 -39.9017
31.1200 -42.0900 0.0660 0.3041 -
0.2426 0.0428 -0.1796 -0.1246 -0.2420
-0.3999 -1.4166 -39.9034
31.1400 -42.5400 -0.1970 0.0276 -
0.2034 0.1417 -0.1997 -0.1318 -0.2269
-0.4021 -1.4136 -39.9050
31.1600 -42.5000 0.0633 -0.3407 -
0.0880 0.2338 -0.2197 -0.1380 -0.2117
-0.4042 -1.4105 -39.9067
31.1800 -42.4800 -0.0339 -0.5170
0.0916 0.3146 -0.2401 -0.1431 -0.1965
-0.4063 -1.4074 -39.9083
31.2000 -42.0500 -0.1689 -0.3218
0.3031 0.3799 -0.2609 -0.1469 -0.1812
-0.4083 -1.4042 -39.9100
31.2200 -41.6500 -0.5577 0.2595
0.4782 0.4259 -0.2824 -0.1494 -0.1659
-0.4102 -1.4011 -39.9116
31.2400 -39.7800 0.8501 0.6916
0.5352 0.4494 -0.3047 -0.1506 -0.1505
-0.4120 -1.3979 -39.9133
31.2600 -41.8300 -0.7290 0.3533
0.4193 0.4488 -0.3281 -0.1502 -0.1350
-0.4138 -1.3948 -39.9149
31.2800 -41.8100 0.2569 -0.2540
0.1757 0.4276 -0.3527 -0.1483 -0.1196
-0.4154 -1.3916 -39.9165
31.3000 -42.5200 0.0620 -0.4405 -
0.1177 0.3908 -0.3785 -0.1448 -0.1042
-0.4170 -1.3884 -39.9182

31.3200 -42.8800 -0.3472 -0.2102 -
0.3815 0.3436 -0.4054 -0.1398 -0.0888
-0.4186 -1.3852 -39.9198
31.3400 -42.2500 0.3587 0.0634 -
0.5430 0.2914 -0.4335 -0.1331 -0.0734
-0.4200 -1.3819 -39.9214
31.3600 -42.8500 -0.3021 0.0939 -
0.5590 0.2390 -0.4625 -0.1250 -0.0580
-0.4214 -1.3787 -39.9231
31.3800 -42.3700 0.2343 -0.0646 -
0.4328 0.1895 -0.4923 -0.1153 -0.0426
-0.4227 -1.3754 -39.9247
31.4000 -42.8700 -0.2106 -0.2607 -
0.2023 0.1433 -0.5226 -0.1043 -0.0273
-0.4239 -1.3721 -39.9263
31.4200 -42.1600 0.3329 -0.2702
0.0765 0.0999 -0.5533 -0.0918 -0.0121
-0.4251 -1.3688 -39.9280
31.4400 -42.4600 -0.6302 0.0985
0.3360 0.0589 -0.5838 -0.0781 0.0031
-0.4261 -1.3655 -39.9296
31.4600 -40.5900 0.8111 0.4253
0.5041 0.0199 -0.6138 -0.0632 0.0182
-0.4271 -1.3622 -39.9312
31.4800 -42.0400 -0.4124 0.2480
0.5332 -0.0173 -0.6427 -0.0472 0.0332
-0.4280 -1.3589 -39.9328
31.5000 -42.6600 -0.5065 -0.1003
0.4404 -0.0528 -0.6702 -0.0301 0.0482
-0.4289 -1.3555 -39.9345
31.5200 -42.1300 0.2429 -0.2410
0.2694 -0.0867 -0.6955 -0.0121 0.0630
-0.4297 -1.3521 -39.9361
31.5400 -42.1600 0.3207 -0.1719
0.0674 -0.1193 -0.7181 0.0069 0.0777
-0.4303 -1.3487 -39.9377
31.5600 -42.8700 -0.2386 -0.0358 -
0.1253 -0.1506 -0.7374 0.0266 0.0924
-0.4309 -1.3453 -39.9393
31.5800 -42.7700 0.0473 0.0948 -
0.2812 -0.1807 -0.7527 0.0471 0.1069
-0.4315 -1.3419 -39.9409
31.6000 -42.7700 -0.0613 0.1702 -
0.3836 -0.2093 -0.7634 0.0681 0.1212
-0.4319 -1.3384 -39.9425
31.6200 -42.7700 0.1101 0.0844 -
0.4220 -0.2357 -0.7689 0.0898 0.1355
-0.4323 -1.3350 -39.9442
31.6400 -42.7700 0.1869 -0.1750 -
0.3908 -0.2601 -0.7689 0.1119 0.1496
-0.4326 -1.3315 -39.9458

31.6600	-43.1400	-0.1412	-0.2878	-
0.2943	-0.2824	-0.7629	0.1343	0.1635
	-0.4328	-1.3280	-39.9474	
31.6800	-43.1600	-0.6554	0.0038	-
0.1479	-0.3032	-0.7505	0.1571	0.1773
	-0.4329	-1.3245	-39.9490	
31.7000	-41.1100	0.8985	0.3608	
0.0170	-0.3240	-0.7314	0.1801	0.1909
	-0.4330	-1.3210	-39.9506	
31.7200	-41.9600	0.0769	0.1922	
0.1671	-0.3462	-0.7052	0.2031	0.2043
	-0.4329	-1.3175	-39.9522	
31.7400	-42.3000	0.0175	-0.3005	
0.2830	-0.3712	-0.6719	0.2262	0.2175
	-0.4328	-1.3139	-39.9538	
31.7600	-43.1700	-0.9164	-0.3647	
0.3512	-0.3999	-0.6311	0.2493	0.2305
	-0.4327	-1.3104	-39.9554	
31.7800	-40.9500	0.8587	0.0884	
0.3556	-0.4320	-0.5830	0.2722	0.2434
	-0.4324	-1.3068	-39.9570	
31.8000	-41.5200	-0.0675	0.4154	
0.2874	-0.4661	-0.5274	0.2949	0.2560
	-0.4320	-1.3032	-39.9586	
31.8200	-42.0900	-0.4850	0.3570	
0.1479	-0.4995	-0.4645	0.3172	0.2684
	-0.4316	-1.2996	-39.9601	
31.8400	-41.4000	0.5324	0.0564	-
0.0306	-0.5285	-0.3946	0.3392	0.2806
	-0.4311	-1.2959	-39.9617	
31.8600	-42.8200	-0.5900	-0.1231	-
0.2014	-0.5498	-0.3179	0.3607	0.2925
	-0.4305	-1.2923	-39.9633	
31.8800	-41.7000	0.6011	-0.2203	-
0.3144	-0.5599	-0.2348	0.3817	0.3042
	-0.4298	-1.2886	-39.9649	
31.9000	-42.3200	-0.0338	-0.3402	-
0.3328	-0.5563	-0.1460	0.4020	0.3157
	-0.4291	-1.2850	-39.9665	
31.9200	-42.7400	-0.6619	-0.2916	-
0.2598	-0.5382	-0.0520	0.4216	0.3268
	-0.4282	-1.2813	-39.9681	
31.9400	-40.9700	0.5883	0.0208	-
0.1264	-0.5072	0.0462	0.4404	0.3378
	-0.4273	-1.2776	-39.9697	
31.9600	-41.0700	-0.1414	0.2855	
0.0239	-0.4659	0.1480	0.4583	0.3484
	-0.4263	-1.2739	-39.9712	
31.9800	-40.6100	-0.0946	0.2918	
0.1472	-0.4171	0.2523	0.4753	0.3588
	-0.4252	-1.2701	-39.9728	

32.0000 -40.5400 -0.0312 0.1167
0.2131 -0.3639 0.3584 0.4912 0.3688
-0.4240 -1.2664 -39.9744
32.0200 -40.0000 0.5624 -0.1507
0.2196 -0.3088 0.4653 0.5060 0.3786
-0.4227 -1.2626 -39.9760
32.0400 -41.2500 -0.7654 -0.2725
0.1831 -0.2541 0.5720 0.5196 0.3881
-0.4214 -1.2589 -39.9775
32.0600 -40.1300 0.0513 -0.1042
0.1260 -0.2012 0.6777 0.5319 0.3972
-0.4200 -1.2551 -39.9791
32.0800 -39.1500 0.7763 0.0879
0.0643 -0.1509 0.7812 0.5428 0.4061
-0.4185 -1.2513 -39.9807
32.1000 -40.6600 -0.7239 0.0779
0.0065 -0.1028 0.8814 0.5522 0.4146
-0.4169 -1.2475 -39.9822
32.1200 -39.3300 0.4540 0.0639 -
0.0487 -0.0555 0.9774 0.5602 0.4227
-0.4152 -1.2436 -39.9838
32.1400 -39.8300 -0.1854 0.0853 -
0.0996 -0.0063 1.0679 0.5665 0.4305
-0.4134 -1.2398 -39.9854
32.1600 -39.4300 0.1558 -0.0472 -
0.1370 0.0479 1.1516 0.5711 0.4380
-0.4115 -1.2359 -39.9869
32.1800 -39.2300 0.3638 -0.1882 -
0.1564 0.1101 1.2276 0.5739 0.4451
-0.4096 -1.2321 -39.9885
32.2000 -40.0500 -0.7678 -0.0712 -
0.1642 0.1822 1.2945 0.5748 0.4519
-0.4076 -1.2282 -39.9900
32.2200 -38.0500 0.8684 0.1806 -
0.1762 0.2658 1.3515 0.5738 0.4583
-0.4054 -1.2243 -39.9916
32.2400 -39.4700 -0.5735 0.1684 -
0.2074 0.3601 1.3975 0.5707 0.4643
-0.4032 -1.2204 -39.9931
32.2600 -39.3000 -0.3494 0.0397 -
0.2540 0.4617 1.4317 0.5655 0.4699
-0.4009 -1.2165 -39.9947
32.2800 -38.6400 0.3862 -0.0638 -
0.2954 0.5640 1.4533 0.5581 0.4752
-0.3986 -1.2125 -39.9962
32.3000 -38.7600 0.1413 -0.1611 -
0.2979 0.6587 1.4613 0.5485 0.4801
-0.3961 -1.2086 -39.9978
32.3200 -39.1000 -0.2564 -0.2118 -
0.2280 0.7370 1.4551 0.5366 0.4846
-0.3935 -1.2046 -39.9993

32.3400 -38.4900 0.1247 -0.0966 -
0.0744 0.7916 1.4340 0.5225 0.4887
-0.3909 -1.2006 -40.0009
32.3600 -38.2400 -0.0599 0.0844
0.1472 0.8163 1.3975 0.5063 0.4924
-0.3882 -1.1967 -40.0024
32.3800 -37.6500 0.2740 0.1141
0.3996 0.8056 1.3457 0.4880 0.4957
-0.3853 -1.1927 -40.0039
32.4000 -38.3400 -0.3908 0.0150
0.6211 0.7541 1.2789 0.4678 0.4987
-0.3824 -1.1886 -40.0055
32.4200 -37.5300 0.5482 -0.0185
0.7383 0.6585 1.1984 0.4458 0.5013
-0.3794 -1.1846 -40.0070
32.4400 -38.7800 -0.6529 0.1746
0.6992 0.5198 1.1053 0.4222 0.5035
-0.3763 -1.1806 -40.0085
32.4600 -37.8200 0.4931 0.4127
0.4917 0.3466 1.0009 0.3969 0.5053
-0.3732 -1.1765 -40.0101
32.4800 -38.9600 0.2696 0.3178
0.1488 0.1514 0.8866 0.3702 0.5067
-0.3699 -1.1725 -40.0116
32.5000 -40.8600 -0.4921 -0.1131 -
0.2453 -0.0524 0.7636 0.3422 0.5078
-0.3666 -1.1684 -40.0131
32.5200 -41.1200 0.2897 -0.4521 -
0.5829 -0.2515 0.6333 0.3129 0.5085
-0.3631 -1.1643 -40.0147
32.5400 -42.2500 -0.4731 -0.3814 -
0.7610 -0.4324 0.4969 0.2825 0.5089
-0.3596 -1.1602 -40.0162
32.5600 -41.2000 0.6713 -0.1701 -
0.7290 -0.5836 0.3558 0.2511 0.5089
-0.3560 -1.1561 -40.0177
32.5800 -42.5100 -0.7639 0.0208 -
0.5133 -0.6991 0.2112 0.2188 0.5085
-0.3523 -1.1520 -40.0192
32.6000 -40.7700 0.7635 0.1919 -
0.2016 -0.7778 0.0644 0.1856 0.5078
-0.3485 -1.1479 -40.0207
32.6200 -41.8900 -0.3477 0.1619
0.1094 -0.8200 -0.0833 0.1518 0.5067
-0.3446 -1.1437 -40.0223
32.6400 -41.7941 -0.3108 0.1128
0.3369 -0.8266 -0.2308 0.1175 0.5052
-0.3407 -1.1396 -40.0238
32.6600 -41.5600 -0.2094 0.2419
0.4304 -0.8000 -0.3770 0.0827 0.5035
-0.3366 -1.1354 -40.0253

32.6800 -40.9600 0.5319 0.2970
0.3993 -0.7443 -0.5208 0.0475 0.5014
-0.3325 -1.1313 -40.0268
32.7000 -41.4900 0.5013 0.0163
0.2816 -0.6646 -0.6610 0.0121 0.4989
-0.3283 -1.1271 -40.0283
32.7200 -42.3900 0.2022 -0.4447
0.1245 -0.5664 -0.7967 -0.0234 0.4961
-0.3240 -1.1229 -40.0298
32.7400 -44.0400 -1.0372 -0.5607 -
0.0239 -0.4543 -0.9267 -0.0589 0.4930
-0.3196 -1.1187 -40.0313
32.7600 -42.4400 0.1744 -0.0743 -
0.1261 -0.3332 -1.0499 -0.0943 0.4896
-0.3152 -1.1145 -40.0328
32.7800 -41.2200 0.9668 0.4614 -
0.1773 -0.2071 -1.1654 -0.1295 0.4858
-0.3106 -1.1102 -40.0343
32.8000 -42.6600 -0.4613 0.4651 -
0.1839 -0.0794 -1.2720 -0.1643 0.4817
-0.3060 -1.1060 -40.0358
32.8200 -42.5100 0.1129 -0.0018 -
0.1531 0.0469 -1.3689 -0.1987 0.4773
-0.3013 -1.1018 -40.0373
32.8400 -42.9100 0.0096 -0.3666 -
0.0935 0.1691 -1.4549 -0.2325 0.4726
-0.2965 -1.0975 -40.0388
32.8600 -43.6100 -0.7824 -0.3264 -
0.0164 0.2847 -1.5293 -0.2657 0.4676
-0.2917 -1.0933 -40.0403
32.8800 -42.5100 -0.1512 0.0135
0.0601 0.3915 -1.5911 -0.2981 0.4623
-0.2868 -1.0890 -40.0418
32.9000 -41.1300 0.8449 0.2804
0.1201 0.4871 -1.6395 -0.3296 0.4566
-0.2818 -1.0847 -40.0433
32.9200 -41.2700 0.7565 0.1951
0.1495 0.5687 -1.6739 -0.3600 0.4507
-0.2767 -1.0804 -40.0448
32.9400 -43.4300 -1.1527 -0.0374
0.1377 0.6326 -1.6933 -0.3893 0.4445
-0.2715 -1.0761 -40.0463
32.9600 -42.4200 0.0267 -0.0137
0.0809 0.6746 -1.6973 -0.4175 0.4380
-0.2663 -1.0718 -40.0478
32.9800 -41.7800 0.5555 0.1636 -
0.0064 0.6910 -1.6852 -0.4443 0.4313
-0.2610 -1.0675 -40.0492
33.0000 -41.7900 0.5078 0.1449 -
0.0961 0.6781 -1.6568 -0.4698 0.4242
-0.2556 -1.0632 -40.0507

33.0200 -42.7600 -0.0542 -0.1278 –
0.1571 0.6332 -1.6121 -0.4939 0.4169
-0.2502 -1.0588 -40.0522
33.0400 -43.5400 -0.4731 -0.3669 –
0.1624 0.5551 -1.5512 -0.5166 0.4093
-0.2447 -1.0545 -40.0537
33.0600 -42.7100 0.1610 -0.2681 –
0.0944 0.4452 -1.4750 -0.5380 0.4015
-0.2391 -1.0501 -40.0552
33.0800 -42.7600 -0.5049 0.1555
0.0379 0.3066 -1.3845 -0.5580 0.3934
-0.2335 -1.0458 -40.0566
33.1000 -41.5600 0.4780 0.4111
0.2058 0.1446 -1.2808 -0.5765 0.3850
-0.2277 -1.0414 -40.0581
33.1200 -41.4100 0.7455 0.1830
0.3731 -0.0344 -1.1650 -0.5937 0.3764
-0.2220 -1.0370 -40.0596
33.1400 -43.4400 -0.8298 -0.3188
0.4979 -0.2233 -1.0383 -0.6095 0.3676
-0.2161 -1.0326 -40.0610
33.1600 -42.4800 0.1948 -0.4037
0.5390 -0.4145 -0.9019 -0.6238 0.3586
-0.2103 -1.0283 -40.0625
33.1800 -42.4700 -0.0374 0.0276
0.4728 -0.5999 -0.7571 -0.6368 0.3493
-0.2043 -1.0239 -40.0640
33.2000 -41.7800 0.4604 0.4303
0.3005 -0.7701 -0.6051 -0.6483 0.3398
-0.1983 -1.0194 -40.0654
33.2200 -42.5200 -0.0247 0.4731
0.0393 -0.9156 -0.4472 -0.6585 0.3300
-0.1922 -1.0150 -40.0669
33.2400 -43.3500 -0.3682 0.1783 –
0.2673 -1.0268 -0.2846 -0.6672 0.3201
-0.1861 -1.0106 -40.0683
33.2600 -43.0000 0.6040 -0.2721 –
0.5485 -1.0940 -0.1188 -0.6745 0.3099
-0.1799 -1.0062 -40.0698
33.2800 -44.5800 -0.7179 -0.4986 –
0.7238 -1.1085 0.0488 -0.6804 0.2996
-0.1737 -1.0017 -40.0713
33.3000 -42.8400 0.6776 -0.2966 –
0.7324 -1.0644 0.2167 -0.6849 0.2891
-0.1674 -0.9973 -40.0727
33.3200 -43.3900 -0.5503 0.0051 –
0.5792 -0.9641 0.3835 -0.6879 0.2783
-0.1611 -0.9928 -40.0742
33.3400 -41.6700 0.4281 0.1197 –
0.3116 -0.8159 0.5478 -0.6896 0.2674
-0.1547 -0.9884 -40.0756

33.3600 -41.7200 -0.2232 0.0879 -
0.0011 -0.6298 0.7081 -0.6897 0.2563
-0.1483 -0.9839 -40.0771
33.3800 -40.7600 -0.0094 0.0723
0.2817 -0.4158 0.8630 -0.6885 0.2450
-0.1419 -0.9795 -40.0785
33.4000 -40.0200 0.1424 0.1345
0.4849 -0.1843 1.0112 -0.6858 0.2336
-0.1354 -0.9750 -40.0800
33.4200 -39.7900 -0.1384 0.1834
0.5819 0.0544 1.1512 -0.6817 0.2220
-0.1288 -0.9705 -40.0814
33.4400 -39.3100 0.0872 0.1330
0.5679 0.2903 1.2816 -0.6761 0.2103
-0.1222 -0.9660 -40.0828
33.4600 -39.0900 0.1151 -0.0073
0.4547 0.5146 1.4010 -0.6691 0.1984
-0.1156 -0.9615 -40.0843
33.4800 -39.4000 -0.1141 -0.1802
0.2723 0.7183 1.5080 -0.6607 0.1864
-0.1089 -0.9570 -40.0857
33.5000 -38.8400 0.3439 -0.1859
0.0610 0.8928 1.6012 -0.6508 0.1743
-0.1022 -0.9525 -40.0871
33.5200 -39.6600 -0.7523 0.1294 -
0.1346 1.0300 1.6792 -0.6394 0.1620
-0.0955 -0.9480 -40.0886
33.5400 -37.7700 0.8619 0.3502 -
0.2651 1.1228 1.7406 -0.6267 0.1497
-0.0888 -0.9435 -40.0900
33.5600 -39.6400 -0.7559 0.0142 -
0.2913 1.1666 1.7840 -0.6124 0.1372
-0.0820 -0.9389 -40.0914
33.5800 -38.7700 0.4404 -0.4237 -
0.2133 1.1607 1.8086 -0.5967 0.1247
-0.0752 -0.9344 -40.0929
33.6000 -39.2100 -0.0376 -0.4959 -
0.0542 1.1071 1.8141 -0.5796 0.1121
-0.0683 -0.9299 -40.0943
33.6200 -39.0400 -0.2764 -0.2033
0.1462 1.0097 1.8014 -0.5610 0.0994
-0.0615 -0.9253 -40.0957
33.6400 -38.3400 -0.0757 0.2665
0.3273 0.8736 1.7716 -0.5411 0.0867
-0.0546 -0.9208 -40.0971
33.6600 -37.7300 0.2965 0.6064
0.4225 0.7052 1.7260 -0.5197 0.0739
-0.0477 -0.9162 -40.0985
33.6800 -38.0900 0.2254 0.5232
0.3875 0.5125 1.6658 -0.4971 0.0611
-0.0407 -0.9117 -40.1000

33.7000 -39.0400 0.2803 0.0585
0.2422 0.3047 1.5924 -0.4732 0.0482
-0.0338 -0.9071 -40.1014
33.7200 -40.8100 -0.6348 -0.3529
0.0417 0.0907 1.5070 -0.4481 0.0354
-0.0268 -0.9025 -40.1028
33.7400 -41.2600 -0.5799 -0.3821 -
0.1531 -0.1210 1.4109 -0.4220 0.0225
-0.0199 -0.8980 -40.1042
33.7600 -40.0500 0.8237 -0.1524 -
0.2914 -0.3216 1.3055 -0.3948 0.0096
-0.0129 -0.8934 -40.1056
33.7800 -41.5500 -0.6564 0.0005 -
0.3452 -0.5027 1.1919 -0.3666 -0.0032
-0.0059 -0.8888 -40.1070
33.8000 -41.0000 0.1858 0.1057 -
0.3190 -0.6573 1.0715 -0.3376 -0.0161
0.0011 -0.8842 -40.1084
33.8200 -41.1500 0.0926 0.1830 -
0.2361 -0.7805 0.9455 -0.3077 -0.0289
0.0081 -0.8796 -40.1098
33.8400 -41.1600 0.2707 0.0138 -
0.1202 -0.8698 0.8153 -0.2771 -0.0417
0.0152 -0.8750 -40.1112
33.8600 -41.7900 -0.0300 -0.2134
0.0044 -0.9238 0.6822 -0.2458 -0.0545
0.0222 -0.8704 -40.1126
33.8800 -42.3900 -0.7129 -0.1599
0.1158 -0.9423 0.5473 -0.2139 -0.0672
0.0292 -0.8658 -40.1140
33.9000 -40.5300 0.8003 0.1338
0.1894 -0.9258 0.4117 -0.1815 -0.0798
0.0362 -0.8612 -40.1154
33.9200 -42.0700 -0.6740 0.2101
0.1982 -0.8754 0.2763 -0.1486 -0.0924
0.0432 -0.8566 -40.1168
33.9400 -41.3300 0.2532 0.1579
0.1230 -0.7932 0.1417 -0.1153 -0.1049
0.0503 -0.8520 -40.1182
33.9600 -41.6300 0.0332 0.1296 -
0.0225 -0.6834 0.0084 -0.0817 -0.1173
0.0573 -0.8474 -40.1196
33.9800 -42.0500 -0.0764 -0.0131 -
0.1914 -0.5513 -0.1230 -0.0479 -0.1296
0.0643 -0.8428 -40.1210
34.0000 -41.9200 0.4887 -0.3044 -
0.3216 -0.4034 -0.2519 -0.0138 -0.1418
0.0713 -0.8382 -40.1224
34.0200 -43.2200 -0.9299 -0.3222 -
0.3610 -0.2467 -0.3776 0.0204 -0.1539
0.0783 -0.8336 -40.1238

34.0400 -40.9300 1.0056 -0.0297 -
0.2898 -0.0883 -0.4995 0.0546 -0.1659
0.0853 -0.8289 -40.1251
34.0600 -41.2800 0.2679 0.0870 -
0.1311 0.0646 -0.6171 0.0889 -0.1778
0.0922 -0.8243 -40.1265
34.0800 -42.2100 -0.9488 0.0632
0.0666 0.2054 -0.7298 0.1230 -0.1895
0.0992 -0.8197 -40.1279
34.1000 -40.8700 0.1769 0.1381
0.2470 0.3272 -0.8369 0.1571 -0.2011
0.1061 -0.8150 -40.1293
34.1200 -40.5800 0.0889 0.2668
0.3655 0.4235 -0.9380 0.1911 -0.2125
0.1130 -0.8104 -40.1307
34.1400 -40.2700 0.6200 0.1506
0.3907 0.4891 -1.0327 0.2248 -0.2238
0.1199 -0.8058 -40.1320
34.1600 -41.4600 -0.1335 -0.1379
0.3218 0.5223 -1.1206 0.2583 -0.2349
0.1268 -0.8011 -40.1334
34.1800 -42.0000 -0.4624 -0.2433
0.1853 0.5258 -1.2019 0.2916 -0.2458
0.1337 -0.7965 -40.1348
34.2000 -42.0000 -0.5602 0.0125
0.0195 0.5049 -1.2764 0.3245 -0.2566
0.1405 -0.7918 -40.1361
34.2200 -40.3600 1.0914 0.2622 -
0.1298 0.4653 -1.3444 0.3571 -0.2671
0.1473 -0.7872 -40.1375
34.2400 -42.7100 -1.1104 0.1401 -
0.2237 0.4129 -1.4060 0.3894 -0.2775
0.1541 -0.7825 -40.1389
34.2600 -42.2600 -0.1344 -0.1807 -
0.2486 0.3529 -1.4616 0.4211 -0.2876
0.1608 -0.7779 -40.1402
34.2800 -41.3400 1.0480 -0.3943 -
0.2126 0.2897 -1.5114 0.4524 -0.2975
0.1675 -0.7732 -40.1416
34.3000 -43.2200 -0.9612 -0.2266 -
0.1359 0.2266 -1.5554 0.4832 -0.3072
0.1742 -0.7686 -40.1430
34.3200 -41.0300 0.7455 0.2252 -
0.0539 0.1664 -1.5939 0.5134 -0.3167
0.1809 -0.7639 -40.1443
34.3400 -41.5900 -0.0096 0.4015 -
0.0026 0.1109 -1.6267 0.5429 -0.3259
0.1875 -0.7593 -40.1457
34.3600 -41.7300 0.1680 0.1119
0.0043 0.0604 -1.6534 0.5717 -0.3348
0.1941 -0.7546 -40.1470

34.3800 -43.0700 -0.7481 -0.1545 -
0.0177 0.0136 -1.6737 0.5997 -0.3435
0.2006 -0.7500 -40.1484
34.4000 -41.7400 0.6297 -0.2162 -
0.0409 -0.0315 -1.6871 0.6268 -0.3520
0.2071 -0.7453 -40.1497
34.4200 -41.9300 0.4144 -0.2061 -
0.0369 -0.0774 -1.6931 0.6531 -0.3601
0.2136 -0.7407 -40.1511
34.4400 -43.2200 -0.9207 -0.1152
0.0098 -0.1256 -1.6912 0.6785 -0.3680
0.2200 -0.7360 -40.1524
34.4600 -41.8400 0.1627 0.0717
0.0956 -0.1761 -1.6810 0.7028 -0.3756
0.2264 -0.7314 -40.1538
34.4800 -41.0000 0.7926 0.1567
0.2003 -0.2271 -1.6622 0.7262 -0.3829
0.2328 -0.7267 -40.1551
34.5000 -42.4000 -0.4815 0.0183
0.2936 -0.2750 -1.6347 0.7485 -0.3899
0.2391 -0.7220 -40.1564
34.5200 -42.2600 -0.3135 -0.0639
0.3405 -0.3159 -1.5984 0.7697 -0.3966
0.2453 -0.7174 -40.1578
34.5400 -41.8700 -0.1483 0.1284
0.3135 -0.3462 -1.5534 0.7898 -0.4030
0.2515 -0.7127 -40.1591
34.5600 -41.1500 0.4635 0.2934
0.2016 -0.3632 -1.5000 0.8087 -0.4090
0.2577 -0.7081 -40.1604
34.5800 -41.2800 0.5574 0.1433
0.0100 -0.3646 -1.4383 0.8265 -0.4148
0.2638 -0.7034 -40.1618
34.6000 -43.0000 -0.7708 -0.1104 -
0.2261 -0.3493 -1.3685 0.8431 -0.4202
0.2698 -0.6987 -40.1631
34.6200 -42.4869 -0.1273 -0.0967 -
0.4569 -0.3168 -1.2909 0.8585 -0.4252
0.2758 -0.6941 -40.1644
34.6400 -41.8000 0.3833 0.0598 -
0.6239 -0.2669 -1.2060 0.8726 -0.4300
0.2818 -0.6894 -40.1658
34.6600 -41.5400 0.5808 0.0230 -
0.6757 -0.2002 -1.1142 0.8853 -0.4343
0.2877 -0.6848 -40.1671
34.6800 -42.6300 -0.6658 -0.2376 -
0.5886 -0.1194 -1.0160 0.8968 -0.4384
0.2935 -0.6801 -40.1684
34.7000 -41.2600 0.4923 -0.4318 -
0.3700 -0.0301 -0.9120 0.9069 -0.4421
0.2993 -0.6755 -40.1697

34.7200 -41.6700 -0.5228 -0.2442 -
0.0603 0.0606 -0.8027 0.9156 -0.4454
0.3050 -0.6708 -40.1710
34.7400 -40.1400 -0.0263 0.2138
0.2713 0.1451 -0.6887 0.9228 -0.4484
0.3107 -0.6662 -40.1724
34.7600 -38.9200 0.3874 0.5372
0.5515 0.2162 -0.5705 0.9286 -0.4510
0.3163 -0.6615 -40.1737
34.7800 -38.9500 0.0886 0.4416
0.7171 0.2663 -0.4488 0.9329 -0.4532
0.3218 -0.6569 -40.1750
34.8000 -39.3400 -0.0837 0.0461
0.7455 0.2890 -0.3240 0.9357 -0.4551
0.3273 -0.6522 -40.1763
34.8200 -39.7100 -0.2385 -0.2901
0.6555 0.2811 -0.1969 0.9370 -0.4566
0.3327 -0.6476 -40.1776
34.8400 -39.6500 -0.0005 -0.2963
0.4786 0.2438 -0.0683 0.9366 -0.4578
0.3381 -0.6429 -40.1789
34.8600 -39.4800 0.0082 -0.0656
0.2541 0.1813 0.0611 0.9347 -0.4586
0.3434 -0.6383 -40.1802
34.8800 -39.2700 0.2441 0.1064
0.0220 0.0994 0.1903 0.9311 -0.4590
0.3486 -0.6337 -40.1815
34.9000 -39.8000 -0.0547 0.1158 -
0.1903 0.0046 0.3186 0.9258 -0.4591
0.3538 -0.6290 -40.1828
34.9200 -40.2600 -0.3560 0.1080 -
0.3713 -0.0954 0.4448 0.9188 -0.4588
0.3589 -0.6244 -40.1841
34.9400 -39.6000 0.4301 0.1113 -
0.5106 -0.1932 0.5681 0.9102 -0.4582
0.3639 -0.6198 -40.1854
34.9600 -40.3900 -0.1717 -0.0219 -
0.5911 -0.2816 0.6875 0.8997 -0.4573
0.3689 -0.6151 -40.1867
34.9800 -40.2600 0.2909 -0.2866 -
0.5961 -0.3552 0.8022 0.8875 -0.4560
0.3738 -0.6105 -40.1880
35.0000 -40.8200 -0.4100 -0.3660 -
0.5208 -0.4100 0.9112 0.8734 -0.4543
0.3786 -0.6059 -40.1893
35.0200 -39.9800 -0.1423 -0.0744 -
0.3790 -0.4438 1.0140 0.8575 -0.4523
0.3834 -0.6013 -40.1906
35.0400 -38.8800 0.3392 0.3148 -
0.2069 -0.4556 1.1098 0.8398 -0.4501
0.3880 -0.5966 -40.1919

35.0600 -38.8800 0.0435 0.4215 -
0.0465 -0.4450 1.1980 0.8202 -0.4474
0.3927 -0.5920 -40.1932
35.0800 -38.9500 0.0720 0.1765
0.0708 -0.4121 1.2780 0.7987 -0.4445
0.3972 -0.5874 -40.1945
35.1000 -39.1400 -0.0872 -0.1706
0.1370 -0.3581 1.3494 0.7753 -0.4413
0.4017 -0.5828 -40.1957
35.1200 -39.1200 -0.1317 -0.3254
0.1593 -0.2848 1.4116 0.7499 -0.4377
0.4061 -0.5782 -40.1970
35.1400 -38.8700 -0.0697 -0.2279
0.1525 -0.1950 1.4639 0.7226 -0.4338
0.4104 -0.5736 -40.1983
35.1600 -38.3400 0.2826 -0.0556
0.1272 -0.0914 1.5059 0.6933 -0.4297
0.4147 -0.5690 -40.1996
35.1800 -38.2000 0.2341 0.0694
0.0865 0.0226 1.5371 0.6620 -0.4253
0.4189 -0.5644 -40.2008
35.2000 -38.8400 -0.5646 0.1871
0.0241 0.1438 1.5569 0.6288 -0.4206
0.4230 -0.5598 -40.2021
35.2200 -37.5600 0.5835 0.2235 -
0.0648 0.2680 1.5648 0.5937 -0.4156
0.4270 -0.5552 -40.2034
35.2400 -38.6100 -0.2217 0.0139 -
0.1700 0.3895 1.5603 0.5566 -0.4103
0.4310 -0.5506 -40.2047
35.2600 -38.7200 -0.0835 -0.2433 -
0.2595 0.5013 1.5430 0.5178 -0.4048
0.4349 -0.5461 -40.2059
35.2800 -39.0600 -0.4557 -0.2180 -
0.2947 0.5970 1.5125 0.4772 -0.3990
0.4387 -0.5415 -40.2072
35.3000 -37.7800 0.5952 0.0129 -
0.2499 0.6710 1.4686 0.4350 -0.3929
0.4424 -0.5369 -40.2085
35.3200 -38.7900 -0.6122 0.1425 -
0.1190 0.7190 1.4112 0.3913 -0.3866
0.4461 -0.5324 -40.2097
35.3400 -37.5400 0.5993 0.0277
0.0823 0.7374 1.3404 0.3461 -0.3801
0.4497 -0.5278 -40.2110
35.3600 -38.0000 0.2828 -0.1578
0.3097 0.7230 1.2571 0.2997 -0.3733
0.4532 -0.5232 -40.2122
35.3800 -39.0000 -0.7808 -0.1822
0.5050 0.6733 1.1623 0.2520 -0.3663
0.4566 -0.5187 -40.2135

35.4000 -37.4000 0.6748 0.0780
0.6160 0.5872 1.0573 0.2034 -0.3590
0.4600 -0.5141 -40.2147
35.4200 -38.0700 0.0235 0.3660
0.6059 0.4669 0.9436 0.1537 -0.3515
0.4633 -0.5096 -40.2160
35.4400 -38.8800 -0.4766 0.3973
0.4656 0.3192 0.8225 0.1033 -0.3438
0.4665 -0.5051 -40.2172
35.4600 -38.8900 0.4775 0.1116
0.2227 0.1537 0.6957 0.0522 -0.3359
0.4696 -0.5005 -40.2185
35.4800 -40.5600 -0.2109 -0.2487 -
0.0694 -0.0187 0.5645 0.0005 -0.3278
0.4727 -0.4960 -40.2197
35.5000 -41.4000 -0.3060 -0.3510 -
0.3505 -0.1872 0.4306 -0.0516 -0.3195
0.4757 -0.4915 -40.2210
35.5200 -41.0700 0.3059 -0.1226 -
0.5636 -0.3410 0.2953 -0.1041 -0.3110
0.4786 -0.4870 -40.2222
35.5400 -41.8500 -0.1914 0.0743 -
0.6624 -0.4694 0.1601 -0.1568 -0.3022
0.4814 -0.4825 -40.2235
35.5600 -41.4300 0.5609 -0.0768 -
0.6267 -0.5638 0.0265 -0.2095 -0.2933
0.4841 -0.4780 -40.2247
35.5800 -42.4600 -0.1240 -0.3587 -
0.4753 -0.6200 -0.1040 -0.2623 -0.2842
0.4868 -0.4735 -40.2259
35.6000 -43.1300 -0.7997 -0.3895 -
0.2456 -0.6385 -0.2300 -0.3148 -0.2750
0.4894 -0.4690 -40.2272
35.6200 -41.7400 -0.0242 0.0017
0.0113 -0.6223 -0.3504 -0.3671 -0.2655
0.4919 -0.4645 -40.2284
35.6400 -40.6800 0.5723 0.4406
0.2346 -0.5743 -0.4644 -0.4191 -0.2559
0.4943 -0.4600 -40.2296
35.6600 -40.3700 0.7383 0.5042
0.3683 -0.4981 -0.5713 -0.4705 -0.2462
0.4967 -0.4556 -40.2309
35.6800 -41.6100 -0.2882 0.2485
0.3837 -0.3984 -0.6706 -0.5212 -0.2362
0.4990 -0.4511 -40.2321
35.7000 -42.4500 -0.6958 -0.0147
0.2933 -0.2831 -0.7617 -0.5712 -0.2262
0.5011 -0.4466 -40.2333
35.7200 -41.4400 0.5222 -0.1046
0.1353 -0.1624 -0.8441 -0.6204 -0.2159
0.5033 -0.4422 -40.2345

35.7400 -42.3200 -0.1892 -0.1327 -
0.0327 -0.0471 -0.9173 -0.6685 -0.2056
0.5053 -0.4377 -40.2358
35.7600 -42.2700 0.2163 -0.2657 -
0.1517 0.0512 -0.9808 -0.7156 -0.1951
0.5073 -0.4333 -40.2370
35.7800 -42.8300 -0.2553 -0.3500 -
0.1783 0.1228 -1.0344 -0.7615 -0.1844
0.5091 -0.4289 -40.2382
35.8000 -42.2800 0.1824 -0.1885 -
0.0965 0.1620 -1.0780 -0.8060 -0.1737
0.5109 -0.4245 -40.2394
35.8200 -42.1500 -0.2464 0.1025
0.0702 0.1696 -1.1115 -0.8492 -0.1628
0.5127 -0.4200 -40.2406
35.8400 -41.6500 0.0661 0.1871
0.2766 0.1499 -1.1356 -0.8908 -0.1518
0.5143 -0.4156 -40.2418
35.8600 -40.8900 0.9446 -0.0168
0.4571 0.1085 -1.1508 -0.9308 -0.1406
0.5158 -0.4112 -40.2430
35.8800 -43.1100 -1.1782 -0.1221
0.5394 0.0513 -1.1580 -0.9690 -0.1294
0.5173 -0.4068 -40.2443
35.9000 -40.8500 0.8012 0.2450
0.4804 -0.0149 -1.1580 -1.0055 -0.1181
0.5187 -0.4025 -40.2455
35.9200 -40.8500 0.7911 0.5827
0.2871 -0.0833 -1.1518 -1.0400 -0.1066
0.5200 -0.3981 -40.2467
35.9400 -41.8800 0.2371 0.4292 -
0.0023 -0.1461 -1.1402 -1.0725 -0.0951
0.5213 -0.3937 -40.2479
35.9600 -43.5200 -0.6382 -0.0202 -
0.3133 -0.1960 -1.1241 -1.1030 -0.0835
0.5224 -0.3894 -40.2491
35.9800 -43.7239 -0.1701 -0.4396 -
0.5664 -0.2253 -1.1044 -1.1313 -0.0718
0.5235 -0.3850 -40.2503
36.0000 -43.7900 0.1818 -0.6731 -
0.6867 -0.2267 -1.0821 -1.1574 -0.0600
0.5245 -0.3807 -40.2515
36.0200 -43.6300 0.3185 -0.6768 -
0.6218 -0.1958 -1.0580 -1.1812 -0.0481
0.5254 -0.3763 -40.2527
36.0400 -43.9400 -0.7893 -0.2952 -
0.3993 -0.1360 -1.0325 -1.2028 -0.0362
0.5262 -0.3720 -40.2539
36.0600 -42.4600 -0.3043 0.3317 -
0.0945 -0.0555 -1.0058 -1.2219 -0.0241
0.5269 -0.3677 -40.2550

36.0800 -40.3900 1.0036 0.7317
0.2104 0.0370 -0.9781 -1.2387 -0.0120
0.5276 -0.3634 -40.2562
36.1000 -41.6100 -0.3738 0.5263
0.4410 0.1330 -0.9494 -1.2530 0.0001
0.5282 -0.3591 -40.2574
36.1200 -42.1000 -0.7607 0.0629
0.5548 0.2237 -0.9198 -1.2649 0.0123
0.5287 -0.3548 -40.2586
36.1400 -41.0900 0.5494 -0.2070
0.5379 0.3014 -0.8891 -1.2744 0.0246
0.5291 -0.3505 -40.2598
36.1600 -41.4900 0.0378 -0.1310
0.4055 0.3608 -0.8574 -1.2815 0.0369
0.5294 -0.3462 -40.2610
36.1800 -42.0600 -0.5268 0.0419
0.2077 0.3991 -0.8248 -1.2862 0.0493
0.5297 -0.3420 -40.2621
36.2000 -40.9400 0.6815 0.0061
0.0082 0.4146 -0.7913 -1.2886 0.0617
0.5298 -0.3377 -40.2633
36.2200 -42.6000 -0.6448 -0.1719 -
0.1489 0.4063 -0.7568 -1.2887 0.0742
0.5299 -0.3335 -40.2645
36.2400 -41.9400 0.0682 -0.1240 -
0.2453 0.3742 -0.7213 -1.2865 0.0867
0.5299 -0.3292 -40.2657
36.2600 -41.5800 0.2748 0.0501 -
0.2841 0.3202 -0.6850 -1.2821 0.0992
0.5298 -0.3250 -40.2668
36.2800 -41.3600 0.5165 0.0316 -
0.2772 0.2476 -0.6479 -1.2756 0.1118
0.5297 -0.3208 -40.2680
36.3000 -42.1000 -0.1320 -0.1018 -
0.2370 0.1607 -0.6102 -1.2670 0.1244
0.5294 -0.3166 -40.2692
36.3200 -42.7800 -0.8343 -0.0647 -
0.1703 0.0650 -0.5720 -1.2564 0.1370
0.5291 -0.3124 -40.2703
36.3400 -40.7600 0.8345 0.1575 -
0.0838 -0.0349 -0.5334 -1.2438 0.1496
0.5286 -0.3082 -40.2715
36.3600 -41.7200 0.0340 0.1284
0.0136 -0.1347 -0.4945 -1.2292 0.1622
0.5281 -0.3040 -40.2727
36.3800 -41.8900 -0.0271 -0.2021
0.1080 -0.2294 -0.4554 -1.2127 0.1748
0.5275 -0.2998 -40.2738
36.4000 -42.5700 -0.6792 -0.3129
0.1804 -0.3135 -0.4162 -1.1943 0.1874
0.5268 -0.2957 -40.2750

36.4200 -41.6400 -0.0231 -0.0240
0.2121 -0.3820 -0.3772 -1.1741 0.2000
0.5261 -0.2915 -40.2761
36.4400 -40.6400 0.5582 0.3086
0.1858 -0.4312 -0.3384 -1.1521 0.2125
0.5252 -0.2874 -40.2773
36.4600 -41.2200 -0.0244 0.3412
0.0922 -0.4581 -0.3001 -1.1284 0.2251
0.5243 -0.2832 -40.2784
36.4800 -41.4400 0.1309 0.1156 -
0.0543 -0.4613 -0.2623 -1.1029 0.2376
0.5233 -0.2791 -40.2796
36.5000 -42.3600 -0.5714 -0.0828 -
0.2201 -0.4398 -0.2254 -1.0758 0.2500
0.5222 -0.2750 -40.2807
36.5200 -41.6400 0.2672 -0.1614 -
0.3626 -0.3934 -0.1896 -1.0470 0.2624
0.5210 -0.2709 -40.2819
36.5400 -41.6000 0.2701 -0.2118 -
0.4389 -0.3226 -0.1554 -1.0165 0.2747
0.5197 -0.2668 -40.2830
36.5600 -42.1200 -0.3946 -0.2513 -
0.4246 -0.2303 -0.1229 -0.9845 0.2869
0.5183 -0.2628 -40.2842
36.5800 -41.3400 0.0501 -0.1918 -
0.3214 -0.1211 -0.0927 -0.9508 0.2991
0.5169 -0.2587 -40.2853
36.6000 -40.8000 0.1113 -0.0129 -
0.1544 -0.0016 -0.0650 -0.9157 0.3112
0.5153 -0.2546 -40.2865
36.6200 -40.3500 -0.0845 0.1660
0.0408 0.1214 -0.0402 -0.8790 0.3232
0.5137 -0.2506 -40.2876
36.6400 -39.9300 -0.0591 0.2448
0.2215 0.2410 -0.0186 -0.8408 0.3350
0.5120 -0.2466 -40.2887
36.6600 -39.3100 0.1832 0.2616
0.3483 0.3500 -0.0007 -0.8013 0.3468
0.5102 -0.2425 -40.2899
36.6800 -39.5000 -0.3082 0.2942
0.4013 0.4416 0.0133 -0.7603 0.3584
0.5083 -0.2385 -40.2910
36.7000 -39.0500 0.0707 0.3097
0.3908 0.5096 0.0230 -0.7179 0.3700
0.5064 -0.2345 -40.2921
36.7200 -38.8300 0.4792 0.1280
0.3519 0.5492 0.0282 -0.6743 0.3813
0.5043 -0.2305 -40.2932
36.7400 -40.0600 -0.4599 -0.2918
0.3194 0.5579 0.0284 -0.6294 0.3926
0.5021 -0.2265 -40.2944

36.7600 -40.4900 -0.5095 -0.6299
0.3124 0.5344 0.0238 -0.5834 0.4036
0.4999 -0.2226 -40.2955
36.7800 -39.1700 0.7165 -0.5277
0.3357 0.4800 0.0147 -0.5362 0.4145
0.4976 -0.2186 -40.2966
36.8000 -40.0300 -0.9541 0.1604
0.3666 0.3991 0.0014 -0.4880 0.4253
0.4952 -0.2147 -40.2977
36.8200 -37.5300 1.0157 0.7506
0.3614 0.2977 -0.0154 -0.4388 0.4358
0.4927 -0.2107 -40.2989
36.8400 -39.5400 -0.6027 0.5918
0.2824 0.1829 -0.0351 -0.3887 0.4462
0.4901 -0.2068 -40.3000
36.8600 -40.3300 -0.6444 0.0657
0.1319 0.0619 -0.0572 -0.3378 0.4564
0.4874 -0.2029 -40.3011
36.8800 -39.9300 0.3938 -0.3741 -
0.0617 -0.0586 -0.0811 -0.2862 0.4663
0.4847 -0.1990 -40.3022
36.9000 -40.3200 0.4664 -0.5603 -
0.2558 -0.1722 -0.1060 -0.2340 0.4761
0.4818 -0.1951 -40.3033
36.9200 -41.3600 -0.3454 -0.4669 -
0.4135 -0.2731 -0.1315 -0.1813 0.4856
0.4789 -0.1912 -40.3044
36.9400 -41.0600 -0.2391 -0.1156 -
0.5186 -0.3556 -0.1569 -0.1282 0.4949
0.4758 -0.1873 -40.3055
36.9600 -40.2400 0.2512 0.2991 -
0.5674 -0.4155 -0.1815 -0.0747 0.5040
0.4727 -0.1834 -40.3066
36.9800 -40.1700 0.2351 0.4094 -
0.5588 -0.4495 -0.2049 -0.0210 0.5128
0.4695 -0.1796 -40.3077
37.0000 -40.5100 0.1623 0.1068 -
0.4991 -0.4567 -0.2264 0.0328 0.5214
0.4662 -0.1757 -40.3088
37.0200 -41.0200 -0.2743 -0.2775 -
0.4030 -0.4387 -0.2459 0.0866 0.5297
0.4629 -0.1719 -40.3099
37.0400 -41.3600 -0.6371 -0.3251 -
0.2870 -0.3985 -0.2629 0.1403 0.5377
0.4594 -0.1681 -40.3110
37.0600 -39.7400 0.3264 0.0182 -
0.1653 -0.3402 -0.2775 0.1939 0.5455
0.4559 -0.1643 -40.3121
37.0800 -39.1800 0.2939 0.3031 -
0.0504 -0.2684 -0.2896 0.2473 0.5529
0.4522 -0.1605 -40.3132

37.1000 -38.7400 0.7220 0.1197
0.0498 -0.1879 -0.2990 0.3003 0.5601
0.4485 -0.1567 -40.3143
37.1200 -40.5500 -0.8578 -0.2472
0.1378 -0.1035 -0.3058 0.3528 0.5669
0.4447 -0.1529 -40.3154
37.1400 -39.1700 0.2254 -0.2633
0.2143 -0.0199 -0.3099 0.4048 0.5735
0.4408 -0.1492 -40.3165
37.1600 -38.6300 0.4000 -0.0086
0.2705 0.0583 -0.3113 0.4563 0.5797
0.4368 -0.1454 -40.3175
37.1800 -38.6700 -0.1249 0.2163
0.2970 0.1271 -0.3101 0.5070 0.5856
0.4327 -0.1416 -40.3186
37.2000 -38.4000 0.0812 0.2433
0.2877 0.1833 -0.3061 0.5570 0.5911
0.4285 -0.1379 -40.3197
37.2200 -38.5700 -0.1324 0.1194
0.2438 0.2242 -0.2996 0.6061 0.5963
0.4243 -0.1342 -40.3208
37.2400 -38.3300 0.4306 -0.1328
0.1818 0.2487 -0.2904 0.6544 0.6011
0.4200 -0.1305 -40.3219
37.2600 -39.5800 -0.5827 -0.3174
0.1225 0.2564 -0.2787 0.7017 0.6056
0.4155 -0.1268 -40.3229
37.2800 -38.4500 0.2961 -0.1459
0.0754 0.2480 -0.2644 0.7479 0.6097
0.4110 -0.1231 -40.3240
37.3000 -38.3200 0.1013 0.1755
0.0401 0.2258 -0.2478 0.7930 0.6134
0.4064 -0.1194 -40.3251
37.3200 -38.3674 -0.0491 0.3150
0.0065 0.1929 -0.2288 0.8368 0.6167
0.4018 -0.1157 -40.3262
37.3400 -38.4859 0.0261 0.2273 -
0.0332 0.1529 -0.2077 0.8794 0.6196
0.3970 -0.1121 -40.3272
37.3600 -38.6400 -0.0551 0.0223 -
0.0771 0.1092 -0.1844 0.9206 0.6221
0.3922 -0.1084 -40.3283
37.3800 -38.7941 0.0723 -0.2116 -
0.1096 0.0648 -0.1593 0.9603 0.6241
0.3872 -0.1048 -40.3293
37.4000 -38.9126 -0.0077 -0.3634 -
0.1123 0.0224 -0.1323 0.9985 0.6258
0.3822 -0.1011 -40.3304
37.4200 -38.9600 -0.1005 -0.3014 -
0.0755 -0.0164 -0.1037 1.0350 0.6270
0.3771 -0.0975 -40.3315

37.4400 -38.7100 -0.1914 -0.0415 -
0.0070 -0.0506 -0.0734 1.0699 0.6277
0.3720 -0.0939 -40.3325
37.4600 -37.7300 0.4181 0.2182
0.0693 -0.0806 -0.0416 1.1029 0.6280
0.3667 -0.0903 -40.3336
37.4800 -38.1300 -0.1639 0.3377
0.1215 -0.1069 -0.0082 1.1341 0.6279
0.3614 -0.0867 -40.3346
37.5000 -38.1900 -0.2532 0.3258
0.1284 -0.1302 0.0270 1.1632 0.6272
0.3560 -0.0831 -40.3357
37.5200 -37.6900 0.3268 0.2054
0.0883 -0.1507 0.0639 1.1903 0.6261
0.3505 -0.0795 -40.3367
37.5400 -38.2000 0.1353 -0.0438
0.0180 -0.1686 0.1028 1.2153 0.6245
0.3449 -0.0760 -40.3378
37.5600 -38.7300 -0.1564 -0.3158 -
0.0549 -0.1837 0.1438 1.2379 0.6224
0.3393 -0.0724 -40.3388
37.5800 -38.9600 -0.2276 -0.3698 -
0.1053 -0.1956 0.1870 1.2583 0.6198
0.3335 -0.0689 -40.3399
37.6000 -38.4200 -0.1124 -0.1059 -
0.1193 -0.2042 0.2324 1.2762 0.6167
0.3278 -0.0654 -40.3409
37.6200 -37.6900 0.3051 0.2078 -
0.0988 -0.2098 0.2800 1.2916 0.6130
0.3219 -0.0618 -40.3420
37.6400 -37.6400 0.1789 0.2747 -
0.0546 -0.2131 0.3298 1.3044 0.6088
0.3160 -0.0583 -40.3430
37.6600 -38.1700 -0.2123 0.0864
0.0001 -0.2149 0.3816 1.3146 0.6041
0.3100 -0.0548 -40.3440
37.6800 -38.0000 -0.0185 -0.1134
0.0549 -0.2158 0.4352 1.3219 0.5988
0.3039 -0.0514 -40.3451
37.7000 -37.9900 -0.0983 -0.1476
0.1039 -0.2163 0.4902 1.3265 0.5930
0.2978 -0.0479 -40.3461
37.7200 -37.7700 0.0581 -0.0736
0.1469 -0.2169 0.5461 1.3281 0.5867
0.2916 -0.0444 -40.3471
37.7400 -37.4800 0.1159 -0.0107
0.1813 -0.2173 0.6026 1.3268 0.5798
0.2853 -0.0409 -40.3482
37.7600 -37.7400 -0.1618 0.0469
0.1984 -0.2169 0.6588 1.3224 0.5723
0.2790 -0.0375 -40.3492

37.7800 -37.4500 -0.1287 0.1124
0.1895 -0.2143 0.7141 1.3148 0.5644
0.2726 -0.0341 -40.3502
37.8000 -36.9900 0.5548 0.1151
0.1507 -0.2075 0.7675 1.3041 0.5559
0.2662 -0.0306 -40.3512
37.8200 -37.8000 -0.1787 -0.0019
0.0877 -0.1941 0.8179 1.2901 0.5470
0.2597 -0.0272 -40.3522
37.8400 -38.2100 -0.4757 -0.0837
0.0108 -0.1721 0.8641 1.2727 0.5375
0.2532 -0.0238 -40.3533
37.8600 -37.4300 0.3324 -0.0405 -
0.0693 -0.1394 0.9049 1.2520 0.5276
0.2466 -0.0204 -40.3543
37.8800 -37.2800 0.3549 -0.0004 -
0.1454 -0.0937 0.9392 1.2279 0.5172
0.2399 -0.0170 -40.3553
37.9000 -38.1800 -0.3935 -0.0205 -
0.2141 -0.0329 0.9661 1.2004 0.5064
0.2333 -0.0136 -40.3563
37.9200 -37.7300 -0.0492 0.0011 -
0.2754 0.0447 0.9844 1.1695 0.4951
0.2265 -0.0102 -40.3573
37.9400 -37.7400 -0.0999 0.0972 -
0.3264 0.1385 0.9935 1.1353 0.4834
0.2197 -0.0069 -40.3583
37.9600 -37.1600 0.4551 0.0677 -
0.3644 0.2453 0.9925 1.0978 0.4714
0.2129 -0.0035 -40.3593
37.9800 -38.1500 -0.4077 -0.1150 -
0.3779 0.3594 0.9809 1.0572 0.4589
0.2060 -0.0002 -40.3603
38.0000 -37.6500 0.0939 -0.1326 -
0.3491 0.4736 0.9582 1.0135 0.4461
0.1991 0.0031 -40.3613
38.0200 -37.6400 -0.0686 0.0129 -
0.2626 0.5793 0.9238 0.9670 0.4330
0.1922 0.0065 -40.3623
38.0400 -37.0200 0.2387 0.0245 -
0.1082 0.6680 0.8775 0.9179 0.4195
0.1852 0.0098 -40.3633
38.0600 -37.5900 -0.2361 -0.1427
0.1095 0.7312 0.8191 0.8662 0.4057
0.1781 0.0131 -40.3643
38.0800 -37.3900 -0.0293 -0.2904
0.3596 0.7615 0.7487 0.8123 0.3916
0.1711 0.0164 -40.3653
38.1000 -36.8800 0.3808 -0.2920
0.5912 0.7521 0.6670 0.7564 0.3772
0.1640 0.0197 -40.3663

38.1200 -37.5100 -0.4241 -0.0620
0.7452 0.6978 0.5749 0.6987 0.3625
0.1568 0.0229 -40.3673
38.1400 -37.2400 -0.2728 0.3908
0.7698 0.5966 0.4741 0.6394 0.3476
0.1497 0.0262 -40.3683
38.1600 -36.6500 0.4444 0.7801
0.6359 0.4528 0.3664 0.5787 0.3325
0.1425 0.0295 -40.3693
38.1800 -37.4600 0.3103 0.7032
0.3498 0.2774 0.2539 0.5169 0.3171
0.1353 0.0327 -40.3703
38.2000 -38.6300 0.5010 0.0024 -
0.0241 0.0835 0.1382 0.4542 0.3015
0.1280 0.0359 -40.3713
38.2200 -41.5700 -0.7698 -0.7731 -
0.3860 -0.1153 0.0215 0.3907 0.2858
0.1208 0.0392 -40.3722
38.2400 -41.4600 0.0729 -0.8882 -
0.6374 -0.3058 -0.0943 0.3268 0.2698
0.1135 0.0424 -40.3732
38.2600 -41.4500 0.0163 -0.3778 -
0.7233 -0.4750 -0.2075 0.2626 0.2537
0.1062 0.0456 -40.3742
38.2800 -40.6200 0.5588 0.2500 -
0.6617 -0.6129 -0.3160 0.1984 0.2375
0.0988 0.0488 -40.3752
38.3000 -41.5200 -0.7137 0.6352 -
0.5067 -0.7133 -0.4179 0.1344 0.2211
0.0915 0.0520 -40.3762
38.3200 -40.1400 0.8999 0.5221 -
0.3234 -0.7723 -0.5114 0.0707 0.2046
0.0841 0.0552 -40.3771
38.3400 -42.2600 -0.8096 0.1686 -
0.1641 -0.7892 -0.5951 0.0077 0.1881
0.0768 0.0583 -40.3781
38.3600 -42.2200 -0.5614 -0.0541 -
0.0512 -0.7669 -0.6676 -0.0545 0.1714
0.0694 0.0615 -40.3791
38.3800 -40.7400 0.9442 -0.1619
0.0291 -0.7115 -0.7283 -0.1156 0.1547
0.0620 0.0646 -40.3800
38.4000 -41.4000 0.4916 -0.3188
0.1017 -0.6307 -0.7768 -0.1755 0.1379
0.0546 0.0678 -40.3810
38.4200 -42.7900 -0.8574 -0.3776
0.1774 -0.5322 -0.8130 -0.2339 0.1211
0.0471 0.0709 -40.3820
38.4400 -41.0700 0.4702 -0.1135
0.2536 -0.4223 -0.8373 -0.2906 0.1043
0.0397 0.0740 -40.3829

38.4600 -41.1900 -0.0942 0.2657
0.3036 -0.3054 -0.8499 -0.3456 0.0875
0.0323 0.0772 -40.3839
38.4800 -40.6200 0.4339 0.3243
0.3011 -0.1854 -0.8517 -0.3987 0.0706
0.0248 0.0803 -40.3848
38.5000 -41.6100 -0.4384 0.1419
0.2420 -0.0668 -0.8433 -0.4498 0.0539
0.0174 0.0834 -40.3858
38.5200 -41.9200 -0.4964 -0.0046
0.1401 0.0462 -0.8256 -0.4987 0.0371
0.0099 0.0865 -40.3867
38.5400 -41.3100 0.1467 -0.0541
0.0224 0.1497 -0.7995 -0.5455 0.0205
0.0025 0.0895 -40.3877
38.5600 -40.5400 1.0801 -0.1352 -
0.0724 0.2406 -0.7657 -0.5900 0.0039
-0.0049 0.0926 -40.3886
38.5800 -41.5300 0.1535 -0.2989 -
0.1122 0.3161 -0.7252 -0.6322 -0.0126
-0.0124 0.0957 -40.3896
38.6000 -42.9300 -1.1616 -0.3298 -
0.0874 0.3745 -0.6789 -0.6721 -0.0290
-0.0198 0.0987 -40.3905
38.6200 -41.5500 -0.2496 -0.0459 -
0.0105 0.4156 -0.6277 -0.7097 -0.0453
-0.0272 0.1018 -40.3915
38.6400 -40.0300 0.8074 0.2933
0.0841 0.4393 -0.5724 -0.7449 -0.0614
-0.0346 0.1048 -40.3924
38.6600 -40.0400 0.7155 0.3358
0.1573 0.4463 -0.5139 -0.7777 -0.0774
-0.0420 0.1078 -40.3933
38.6800 -41.7400 -0.7061 0.0527
0.1813 0.4374 -0.4530 -0.8081 -0.0932
-0.0494 0.1108 -40.3943
38.7000 -40.9100 0.4586 -0.1644
0.1484 0.4144 -0.3905 -0.8360 -0.1089
-0.0568 0.1139 -40.3952
38.7200 -42.0200 -0.8786 0.0859
0.0708 0.3796 -0.3272 -0.8614 -0.1243
-0.0642 0.1169 -40.3962
38.7400 -40.1200 0.9800 0.2156 -
0.0141 0.3355 -0.2638 -0.8844 -0.1395
-0.0715 0.1198 -40.3971
38.7600 -41.1400 0.4269 -0.1013 -
0.0723 0.2851 -0.2007 -0.9050 -0.1545
-0.0789 0.1228 -40.3980
38.7800 -42.9700 -1.1630 -0.3981 -
0.0912 0.2306 -0.1386 -0.9231 -0.1693
-0.0862 0.1258 -40.3989

38.8000 -40.9600 0.6708 -0.1431 -
0.0702 0.1740 -0.0781 -0.9388 -0.1838
-0.0935 0.1288 -40.3999
38.8200 -40.7400 0.4441 0.2667 -
0.0257 0.1167 -0.0195 -0.9522 -0.1980
-0.1007 0.1317 -40.4008
38.8400 -40.8500 0.3258 0.2462
0.0228 0.0599 0.0366 -0.9634 -0.2120
-0.1080 0.1347 -40.4017
38.8600 -41.2200 0.3041 -0.1350
0.0633 0.0053 0.0901 -0.9723 -0.2256
-0.1152 0.1376 -40.4026
38.8800 -42.5800 -0.8189 -0.3571
0.0928 -0.0453 0.1406 -0.9793 -0.2389
-0.1224 0.1405 -40.4035
38.9000 -40.7000 0.7955 -0.1082
0.1104 -0.0902 0.1879 -0.9842 -0.2519
-0.1295 0.1434 -40.4045
38.9200 -41.8000 -0.6460 0.2379
0.1108 -0.1279 0.2318 -0.9873 -0.2646
-0.1367 0.1464 -40.4054
38.9400 -41.1600 -0.0510 0.3253
0.0859 -0.1568 0.2721 -0.9886 -0.2769
-0.1438 0.1493 -40.4063
38.9600 -40.7300 0.7531 0.1523
0.0309 -0.1749 0.3087 -0.9882 -0.2888
-0.1508 0.1522 -40.4072
38.9800 -41.9900 -0.2257 -0.1121 -
0.0494 -0.1808 0.3412 -0.9862 -0.3004
-0.1579 0.1550 -40.4081
39.0000 -42.6200 -0.7945 -0.2234 -
0.1446 -0.1737 0.3695 -0.9826 -0.3115
-0.1649 0.1579 -40.4090
39.0200 -41.6800 0.0698 -0.0660 -
0.2376 -0.1536 0.3933 -0.9775 -0.3222
-0.1718 0.1608 -40.4099
39.0400 -40.8100 0.7688 0.1049 -
0.3100 -0.1212 0.4125 -0.9709 -0.3325
-0.1787 0.1636 -40.4108
39.0600 -42.1500 -0.4804 0.0644 -
0.3465 -0.0780 0.4270 -0.9628 -0.3423
-0.1856 0.1665 -40.4117
39.0800 -41.9200 -0.2308 -0.0447 -
0.3416 -0.0263 0.4367 -0.9533 -0.3517
-0.1925 0.1693 -40.4126
39.1000 -41.3100 0.2610 -0.0266 -
0.2953 0.0310 0.4416 -0.9423 -0.3607
-0.1992 0.1722 -40.4135
39.1200 -41.7000 -0.2834 0.1014 -
0.2110 0.0906 0.4416 -0.9300 -0.3691
-0.2060 0.1750 -40.4144

39.1400 -40.9400 0.2090 0.1113 -
0.0941 0.1490 0.4367 -0.9163 -0.3771
-0.2127 0.1778 -40.4153
39.1600 -40.6800 0.4908 -0.0684
0.0489 0.2024 0.4268 -0.9014 -0.3846
-0.2193 0.1806 -40.4162
39.1800 -41.6100 -0.5349 -0.2265
0.2099 0.2469 0.4119 -0.8852 -0.3916
-0.2259 0.1834 -40.4171
39.2000 -41.3000 -0.4787 -0.1265
0.3732 0.2787 0.3922 -0.8678 -0.3981
-0.2325 0.1862 -40.4180
39.2200 -39.6500 0.7275 0.1334
0.5118 0.2948 0.3678 -0.8494 -0.4042
-0.2390 0.1890 -40.4188
39.2400 -40.9500 -0.6405 0.1926
0.5900 0.2928 0.3388 -0.8299 -0.4097
-0.2454 0.1918 -40.4197
39.2600 -40.0300 0.4422 0.0371
0.5810 0.2715 0.3057 -0.8094 -0.4148
-0.2518 0.1946 -40.4206
39.2800 -40.6600 0.0843 -0.0442
0.4757 0.2325 0.2691 -0.7879 -0.4195
-0.2581 0.1973 -40.4215
39.3000 -41.4500 -0.4589 0.0487
0.2836 0.1794 0.2294 -0.7656 -0.4237
-0.2644 0.2001 -40.4224
39.3200 -40.7000 0.3814 0.1847
0.0393 0.1165 0.1872 -0.7424 -0.4274
-0.2706 0.2028 -40.4232
39.3400 -41.3400 0.2918 0.1264 -
0.2089 0.0485 0.1431 -0.7184 -0.4307
-0.2768 0.2055 -40.4241
39.3600 -42.0800 0.1112 -0.1956 -
0.4184 -0.0203 0.0976 -0.6937 -0.4336
-0.2829 0.2083 -40.4250
39.3800 -43.1100 -0.5648 -0.4397 -
0.5587 -0.0851 0.0514 -0.6682 -0.4361
-0.2889 0.2110 -40.4258
39.4000 -42.9900 -0.4104 -0.2836 -
0.6104 -0.1413 0.0049 -0.6421 -0.4381
-0.2948 0.2137 -40.4267
39.4200 -41.6200 0.6181 0.1475 -
0.5806 -0.1856 -0.0412 -0.6154 -0.4398
-0.3007 0.2164 -40.4276
39.4400 -41.6100 0.3641 0.4143 -
0.4919 -0.2164 -0.0862 -0.5882 -0.4411
-0.3066 0.2191 -40.4284
39.4600 -42.6400 -0.6316 0.3022 -
0.3664 -0.2340 -0.1295 -0.5604 -0.4420
-0.3123 0.2218 -40.4293

39.4800 -41.8300 0.4549 -0.0958 -
0.2178 -0.2404 -0.1702 -0.5322 -0.4425
-0.3180 0.2245 -40.4302
39.5000 -42.1900 0.0332 -0.3833 -
0.0562 -0.2380 -0.2077 -0.5036 -0.4427
-0.3237 0.2271 -40.4310
39.5200 -42.5800 -0.4474 -0.3492
0.1100 -0.2298 -0.2414 -0.4747 -0.4425
-0.3292 0.2298 -40.4319
39.5400 -41.3200 0.3936 -0.0526
0.2706 -0.2185 -0.2705 -0.4454 -0.4420
-0.3347 0.2325 -40.4327
39.5600 -41.2200 0.1043 0.1550
0.4100 -0.2071 -0.2946 -0.4159 -0.4411
-0.3402 0.2351 -40.4336
39.5800 -41.5200 -0.2278 0.1473
0.5064 -0.1983 -0.3131 -0.3862 -0.4400
-0.3455 0.2377 -40.4344
39.6000 -41.1900 0.0935 0.1138
0.5352 -0.1949 -0.3255 -0.3563 -0.4385
-0.3508 0.2404 -40.4353
39.6200 -41.5900 -0.3860 0.2176
0.4838 -0.1987 -0.3315 -0.3262 -0.4367
-0.3560 0.2430 -40.4361
39.6400 -40.6900 0.4523 0.2659
0.3629 -0.2104 -0.3312 -0.2961 -0.4347
-0.3611 0.2456 -40.4370
39.6600 -41.5300 -0.0142 0.0310
0.2022 -0.2293 -0.3243 -0.2660 -0.4323
-0.3662 0.2482 -40.4378
39.6800 -42.1600 -0.0724 -0.3087
0.0432 -0.2541 -0.3112 -0.2359 -0.4297
-0.3712 0.2508 -40.4386
39.7000 -42.7500 -0.4218 -0.3831 -
0.0728 -0.2832 -0.2921 -0.2059 -0.4269
-0.3761 0.2534 -40.4395
39.7200 -41.7000 0.2981 -0.1017 -
0.1237 -0.3138 -0.2675 -0.1761 -0.4237
-0.3809 0.2560 -40.4403
39.7400 -41.7300 -0.0401 0.1486 -
0.1159 -0.3421 -0.2376 -0.1464 -0.4204
-0.3857 0.2586 -40.4411
39.7600 -41.0000 0.7298 0.0541 -
0.0687 -0.3632 -0.2032 -0.1170 -0.4168
-0.3903 0.2611 -40.4420
39.7800 -42.1300 -0.3958 -0.1601 -
0.0131 -0.3721 -0.1648 -0.0878 -0.4129
-0.3949 0.2637 -40.4428
39.8000 -42.5900 -0.9903 -0.1702
0.0173 -0.3642 -0.1229 -0.0589 -0.4089
-0.3995 0.2662 -40.4436

39.8200 -41.1700 0.1871 0.1270
0.0026 -0.3356 -0.0780 -0.0305 -0.4046
-0.4039 0.2688 -40.4445
39.8400 -40.0600 0.8528 0.4319 -
0.0597 -0.2844 -0.0310 -0.0024 -0.4001
-0.4083 0.2713 -40.4453
39.8600 -39.6800 1.1626 0.3894 -
0.1607 -0.2109 0.0176 0.0253 -0.3954
-0.4126 0.2739 -40.4461
39.8800 -42.0900 -0.8826 0.0580 -
0.2749 -0.1180 0.0669 0.0525 -0.3906
-0.4168 0.2764 -40.4469
39.9000 -42.3500 -1.0102 -0.2482 -
0.3707 -0.0098 0.1161 0.0791 -0.3855
-0.4209 0.2789 -40.4477
39.9200 -40.6400 0.6621 -0.3671 -
0.4172 0.1087 0.1644 0.1053 -0.3802
-0.4249 0.2814 -40.4485
39.9400 -40.3100 0.8152 -0.3285 -
0.3923 0.2320 0.2110 0.1308 -0.3748
-0.4289 0.2839 -40.4494
39.9600 -41.4600 -0.7676 -0.2033 -
0.2885 0.3547 0.2549 0.1556 -0.3692
-0.4328 0.2864 -40.4502
39.9800 -40.0900 0.1132 -0.0832 -
0.1147 0.4710 0.2955 0.1798 -0.3635
-0.4365 0.2889 -40.4510
40.0000 -39.4400 0.3283 -0.0209
0.1025 0.5755 0.3318 0.2033 -0.3576
-0.4403 0.2914 -40.4518
40.0200 -39.4200 -0.0941 -0.0185
0.3236 0.6623 0.3629 0.2260 -0.3516
-0.4439 0.2938 -40.4526
40.0400 -39.0800 -0.0573 0.0462
0.4992 0.7258 0.3882 0.2479 -0.3454
-0.4474 0.2963 -40.4534
40.0600 -38.7200 -0.0837 0.2846
0.5804 0.7605 0.4066 0.2689 -0.3391
-0.4509 0.2988 -40.4542
40.0800 -38.4300 0.0168 0.5266
0.5362 0.7627 0.4177 0.2891 -0.3327
-0.4543 0.3012 -40.4550
40.1000 -38.2800 0.3150 0.4836
0.3593 0.7337 0.4210 0.3084 -0.3262
-0.4576 0.3036 -40.4558
40.1200 -39.2400 -0.0121 0.0972
0.0868 0.6778 0.4166 0.3267 -0.3196
-0.4608 0.3061 -40.4566
40.1400 -40.3500 -0.1890 -0.3359 -
0.2088 0.6001 0.4049 0.3441 -0.3129
-0.4639 0.3085 -40.4574

40.1600 -40.9700 -0.3997 -0.4454 -
0.4492 0.5055 0.3863 0.3605 -0.3061
-0.4669 0.3109 -40.4582
40.1800 -40.3500 0.1477 -0.2176 -
0.5694 0.3991 0.3612 0.3760 -0.2992
-0.4699 0.3133 -40.4590
40.2000 -40.0100 0.4011 -0.0133 -
0.5442 0.2857 0.3302 0.3906 -0.2922
-0.4727 0.3157 -40.4598
40.2200 -40.8300 -0.3536 -0.0365 -
0.3867 0.1699 0.2938 0.4043 -0.2852
-0.4755 0.3181 -40.4605
40.2400 -40.0000 0.2784 -0.0400 -
0.1445 0.0562 0.2524 0.4171 -0.2781
-0.4782 0.3205 -40.4613
40.2600 -40.6300 -0.5985 0.2271
0.1215 -0.0504 0.2066 0.4290 -0.2710
-0.4808 0.3229 -40.4621
40.2800 -39.1200 0.8210 0.2569
0.3527 -0.1449 0.1567 0.4400 -0.2639
-0.4833 0.3253 -40.4629
40.3000 -41.1800 -0.8450 -0.1389
0.4922 -0.2225 0.1034 0.4501 -0.2567
-0.4857 0.3277 -40.4637
40.3200 -39.9700 0.3923 -0.1958
0.5011 -0.2804 0.0472 0.4594 -0.2495
-0.4880 0.3300 -40.4644
40.3400 -40.1000 0.0836 0.1809
0.3729 -0.3178 -0.0113 0.4679 -0.2423
-0.4903 0.3324 -40.4652
40.3600 -40.2400 -0.0193 0.3568
0.1385 -0.3367 -0.0712 0.4755 -0.2350
-0.4924 0.3347 -40.4660
40.3800 -40.2800 0.6890 -0.0081 -
0.1426 -0.3398 -0.1318 0.4823 -0.2278
-0.4945 0.3371 -40.4668
40.4000 -42.6000 -0.8631 -0.4328 -
0.3836 -0.3302 -0.1921 0.4884 -0.2206
-0.4965 0.3394 -40.4675
40.4200 -41.1500 0.5166 -0.2884 -
0.5060 -0.3111 -0.2512 0.4936 -0.2134
-0.4983 0.3417 -40.4683
40.4400 -41.2000 0.2344 -0.0069 -
0.4873 -0.2861 -0.3080 0.4981 -0.2062
-0.5001 0.3441 -40.4691
40.4600 -40.9900 0.4221 -0.0502 -
0.3488 -0.2590 -0.3615 0.5018 -0.1991
-0.5018 0.3464 -40.4698
40.4800 -41.8600 -0.6833 -0.0663 -
0.1449 -0.2339 -0.4107 0.5048 -0.1920
-0.5034 0.3487 -40.4706

40.5000 -40.6200 0.0596 0.1926
0.0599 -0.2145 -0.4546 0.5071 -0.1849
-0.5049 0.3510 -40.4713
40.5200 -40.2000 0.1720 0.4387
0.2268 -0.2047 -0.4923 0.5087 -0.1779
-0.5063 0.3533 -40.4721
40.5400 -39.8800 0.4418 0.3014
0.3373 -0.2075 -0.5232 0.5096 -0.1709
-0.5077 0.3556 -40.4729
40.5600 -40.7100 0.1575 -0.1813
0.3876 -0.2253 -0.5467 0.5098 -0.1640
-0.5089 0.3578 -40.4736
40.5800 -41.9100 -0.5584 -0.5654
0.3795 -0.2591 -0.5624 0.5095 -0.1571
-0.5100 0.3601 -40.4744
40.6000 -41.1300 0.0831 -0.3920
0.3161 -0.3078 -0.5702 0.5085 -0.1503
-0.5110 0.3624 -40.4751
40.6200 -40.9400 -0.1817 0.2247
0.2056 -0.3688 -0.5700 0.5070 -0.1436
-0.5120 0.3646 -40.4759
40.6400 -39.8800 0.6721 0.6671
0.0653 -0.4384 -0.5618 0.5049 -0.1370
-0.5128 0.3669 -40.4766
40.6600 -41.3100 -0.4105 0.5701 -
0.0837 -0.5123 -0.5457 0.5023 -0.1304
-0.5136 0.3692 -40.4773
40.6800 -41.4800 -0.0174 0.1295 -
0.2179 -0.5859 -0.5216 0.4993 -0.1240
-0.5142 0.3714 -40.4781
40.7000 -41.8600 0.3303 -0.3779 -
0.3144 -0.6545 -0.4899 0.4958 -0.1176
-0.5148 0.3736 -40.4788
40.7200 -42.8600 -0.3675 -0.7403 -
0.3509 -0.7138 -0.4505 0.4919 -0.1114
-0.5152 0.3759 -40.4796
40.7400 -42.5500 -0.1809 -0.7219 -
0.3151 -0.7596 -0.4039 0.4876 -0.1052
-0.5156 0.3781 -40.4803
40.7600 -42.1000 -0.2269 -0.2353 -
0.2242 -0.7896 -0.3502 0.4831 -0.0992
-0.5158 0.3803 -40.4810
40.7800 -40.8600 0.2009 0.3795 -
0.1147 -0.8022 -0.2899 0.4782 -0.0932
-0.5160 0.3825 -40.4818
40.8000 -39.9700 0.6679 0.6540 -
0.0263 -0.7964 -0.2235 0.4731 -0.0874
-0.5161 0.3847 -40.4825
40.8200 -41.4900 -0.8576 0.4822
0.0137 -0.7716 -0.1517 0.4677 -0.0818
-0.5160 0.3869 -40.4832

40.8400 -40.4200 0.5305 0.1311
0.0027 -0.7284 -0.0753 0.4621 -0.0762
-0.5159 0.3891 -40.4839
40.8600 -40.7000 0.3555 -0.1884 -
0.0445 -0.6685 0.0051 0.4563 -0.0708
-0.5156 0.3913 -40.4847
40.8800 -41.7400 -0.6106 -0.3512 -
0.0995 -0.5950 0.0884 0.4504 -0.0655
-0.5153 0.3935 -40.4854
40.9000 -40.8500 0.0291 -0.3073 -
0.1311 -0.5113 0.1741 0.4443 -0.0603
-0.5149 0.3957 -40.4861
40.9200 -40.4300 0.2564 -0.1820 -
0.1135 -0.4213 0.2611 0.4381 -0.0553
-0.5143 0.3978 -40.4868
40.9400 -40.2500 0.0915 -0.1180 -
0.0355 -0.3284 0.3486 0.4318 -0.0505
-0.5137 0.4000 -40.4875
40.9600 -40.1700 -0.1580 -0.0666
0.0912 -0.2354 0.4358 0.4255 -0.0458
-0.5130 0.4021 -40.4882
40.9800 -39.7400 -0.3548 0.1270
0.2395 -0.1440 0.5219 0.4191 -0.0412
-0.5121 0.4043 -40.4890
41.0000 -38.5600 0.4306 0.3063
0.3811 -0.0558 0.6060 0.4127 -0.0368
-0.5112 0.4064 -40.4897
41.0200 -38.8800 0.0402 0.1720
0.4899 0.0281 0.6873 0.4063 -0.0326
-0.5101 0.4086 -40.4904
41.0400 -39.1900 -0.2779 -0.1176
0.5466 0.1069 0.7649 0.4000 -0.0285
-0.5090 0.4107 -40.4911
41.0600 -38.7700 0.0423 -0.2021
0.5400 0.1801 0.8379 0.3937 -0.0246
-0.5078 0.4128 -40.4918
41.0800 -38.7700 -0.1496 -0.0157
0.4639 0.2478 0.9056 0.3876 -0.0209
-0.5064 0.4150 -40.4925
41.1000 -38.0800 0.3334 0.1771
0.3197 0.3102 0.9670 0.3815 -0.0173
-0.5050 0.4171 -40.4932
41.1200 -38.7000 -0.2742 0.1908
0.1153 0.3680 1.0214 0.3756 -0.0140
-0.5034 0.4192 -40.4939
41.1400 -38.6700 0.1019 0.0898 -
0.1273 0.4219 1.0677 0.3698 -0.0108
-0.5018 0.4213 -40.4946
41.1600 -39.1500 -0.1278 -0.0573 -
0.3722 0.4725 1.1053 0.3642 -0.0078
-0.5001 0.4234 -40.4953

41.1800 -39.1800 0.1107 -0.1608 -
0.5783 0.5198 1.1333 0.3588 -0.0049
-0.4982 0.4255 -40.4960
41.2000 -39.6900 -0.3137 -0.1374 -
0.7077 0.5634 1.1510 0.3536 -0.0023
-0.4963 0.4276 -40.4966
41.2200 -39.0300 0.2439 -0.0947 -
0.7382 0.6026 1.1576 0.3487 0.0002
-0.4942 0.4297 -40.4973
41.2400 -38.7400 0.4605 -0.1153 -
0.6671 0.6364 1.1529 0.3440 0.0024
-0.4921 0.4318 -40.4980
41.2600 -39.6500 -0.7666 -0.0624 -
0.5102 0.6630 1.1364 0.3396 0.0045
-0.4899 0.4339 -40.4987
41.2800 -38.4800 0.1520 0.1665 -
0.2928 0.6804 1.1083 0.3355 0.0064
-0.4875 0.4359 -40.4994
41.3000 -37.5300 0.6723 0.2746 -
0.0305 0.6864 1.0687 0.3316 0.0080
-0.4851 0.4380 -40.5001
41.3200 -37.8700 0.3738 0.0297
0.2537 0.6785 1.0180 0.3280 0.0095
-0.4826 0.4401 -40.5007
41.3400 -39.0700 -0.6932 -0.2920
0.5183 0.6545 0.9569 0.3248 0.0108
-0.4800 0.4421 -40.5014
41.3600 -37.8300 0.4521 -0.2707
0.7167 0.6117 0.8860 0.3218 0.0119
-0.4772 0.4442 -40.5021
41.3800 -37.9500 -0.1560 0.1181
0.8099 0.5483 0.8062 0.3192 0.0128
-0.4744 0.4462 -40.5028
41.4000 -37.8700 -0.0845 0.4479
0.7720 0.4647 0.7182 0.3169 0.0135
-0.4715 0.4483 -40.5034
41.4200 -37.7100 0.5445 0.3909
0.5982 0.3645 0.6231 0.3149 0.0140
-0.4685 0.4503 -40.5041
41.4400 -39.1600 0.0052 -0.0323
0.3249 0.2531 0.5218 0.3133 0.0144
-0.4654 0.4523 -40.5048
41.4600 -40.5600 -0.5965 -0.3973
0.0124 0.1361 0.4150 0.3120 0.0145
-0.4622 0.4544 -40.5054
41.4800 -40.3600 0.1970 -0.3583 -
0.2772 0.0193 0.3038 0.3110 0.0144
-0.4589 0.4564 -40.5061
41.5000 -40.3900 0.3342 -0.0969 -
0.4946 -0.0918 0.1890 0.3105 0.0142
-0.4555 0.4584 -40.5068

41.5200 -40.8800 -0.0047 0.0136 -
0.6096 -0.1916 0.0716 0.3102 0.0137
-0.4521 0.4605 -40.5074
41.5400 -41.1100 0.1102 -0.1124 -
0.6158 -0.2751 -0.0476 0.3104 0.0131
-0.4485 0.4625 -40.5081
41.5600 -41.6800 -0.3702 -0.1775 -
0.5309 -0.3386 -0.1676 0.3109 0.0122
-0.4448 0.4645 -40.5087
41.5800 -41.1900 0.0388 -0.0213 -
0.3810 -0.3801 -0.2877 0.3119 0.0112
-0.4411 0.4665 -40.5094
41.6000 -40.7400 0.1937 0.1708 -
0.1963 -0.3993 -0.4069 0.3132 0.0100
-0.4373 0.4685 -40.5100
41.6200 -40.9700 -0.0611 0.1755 -
0.0078 -0.3969 -0.5245 0.3149 0.0086
-0.4333 0.4705 -40.5107
41.6400 -40.9200 0.0673 0.0168
0.1534 -0.3740 -0.6397 0.3171 0.0070
-0.4293 0.4725 -40.5113
41.6600 -41.2900 -0.2001 -0.1037
0.2589 -0.3330 -0.7519 0.3196 0.0052
-0.4252 0.4745 -40.5120
41.6800 -41.0300 -0.0523 -0.0192
0.2902 -0.2767 -0.8604 0.3226 0.0032
-0.4210 0.4765 -40.5126
41.7000 -40.7100 0.1804 0.1925
0.2491 -0.2094 -0.9643 0.3261 0.0011
-0.4168 0.4785 -40.5132
41.7200 -41.0200 -0.0372 0.2601
0.1574 -0.1359 -1.0630 0.3299 -0.0013
-0.4124 0.4805 -40.5139
41.7400 -40.6800 0.5693 -0.0192
0.0492 -0.0612 -1.1557 0.3343 -0.0038
-0.4080 0.4825 -40.5145
41.7600 -42.4300 -0.6894 -0.4014 -
0.0377 0.0098 -1.2416 0.3392 -0.0064
-0.4035 0.4844 -40.5152
41.7800 -41.5100 0.2709 -0.3825 -
0.0749 0.0723 -1.3200 0.3445 -0.0093
-0.3989 0.4864 -40.5158
41.8000 -41.6000 -0.1369 0.0176 -
0.0625 0.1229 -1.3903 0.3503 -0.0123
-0.3942 0.4884 -40.5164
41.8200 -40.8700 0.1492 0.3784 -
0.0242 0.1594 -1.4518 0.3567 -0.0155
-0.3894 0.4904 -40.5170
41.8400 -40.8100 0.2255 0.3788
0.0152 0.1801 -1.5039 0.3636 -0.0188
-0.3846 0.4923 -40.5177

41.8600 -41.3500 -0.0597 0.0736
0.0425 0.1839 -1.5463 0.3710 -0.0223
-0.3797 0.4943 -40.5183
41.8800 -41.9900 -0.3305 -0.2276
0.0555 0.1708 -1.5786 0.3790 -0.0259
-0.3747 0.4963 -40.5189
41.9000 -41.4300 0.3177 -0.3107
0.0565 0.1417 -1.6008 0.3874 -0.0297
-0.3696 0.4982 -40.5195
41.9200 -41.4600 0.1789 -0.1297
0.0518 0.0988 -1.6128 0.3964 -0.0336
-0.3645 0.5002 -40.5202
41.9400 -41.9900 -0.7237 0.2115
0.0485 0.0442 -1.6148 0.4058 -0.0376
-0.3593 0.5021 -40.5208
41.9600 -40.4000 0.8778 0.3091
0.0504 -0.0196 -1.6068 0.4157 -0.0418
-0.3540 0.5041 -40.5214
41.9800 -42.3800 -0.7989 -0.0111
0.0548 -0.0893 -1.5892 0.4259 -0.0460
-0.3487 0.5060 -40.5220
42.0000 -41.0600 0.6937 -0.2414
0.0599 -0.1607 -1.5621 0.4366 -0.0504
-0.3433 0.5080 -40.5226
42.0200 -42.2200 -0.4037 -0.2275
0.0630 -0.2290 -1.5259 0.4475 -0.0548
-0.3378 0.5099 -40.5232
42.0400 -41.7900 -0.0456 -0.1026
0.0557 -0.2884 -1.4809 0.4587 -0.0594
-0.3322 0.5119 -40.5238
42.0600 -41.6000 -0.1476 0.1644
0.0225 -0.3339 -1.4276 0.4702 -0.0640
-0.3266 0.5138 -40.5244
42.0800 -41.0500 0.3382 0.3809 -
0.0502 -0.3603 -1.3667 0.4819 -0.0687
-0.3210 0.5158 -40.5250
42.1000 -41.5000 -0.0922 0.3104 -
0.1593 -0.3640 -1.2988 0.4936 -0.0735
-0.3153 0.5177 -40.5256
42.1200 -41.6300 0.0778 0.0203 -
0.2776 -0.3426 -1.2248 0.5055 -0.0783
-0.3095 0.5196 -40.5262
42.1400 -41.9300 -0.0206 -0.2625 -
0.3610 -0.2964 -1.1456 0.5175 -0.0832
-0.3037 0.5216 -40.5268
42.1600 -42.0800 -0.1236 -0.3699 -
0.3668 -0.2275 -1.0619 0.5294 -0.0881
-0.2978 0.5235 -40.5274
42.1800 -41.5900 -0.0154 -0.2549 -
0.2767 -0.1407 -0.9746 0.5413 -0.0931
-0.2918 0.5254 -40.5280

42.2000	-40.4800	0.3880	-0.0486	-
0.1084	-0.0425	-0.8848	0.5531	-0.0981
	-0.2858	0.5274	-40.5286	
42.2200	-40.7800	-0.4762	0.1547	
0.0960	0.0593	-0.7933	0.5647	-0.1031
	-0.2798	0.5293	-40.5292	
42.2400	-39.2400	0.4298	0.3050	
0.2838	0.1571	-0.7010	0.5762	-0.1082
	-0.2737	0.5312	-40.5298	
42.2600	-39.7700	-0.2735	0.2518	
0.4079	0.2429	-0.6088	0.5875	-0.1132
	-0.2676	0.5332	-40.5304	
42.2800	-39.1400	0.4242	0.0135	
0.4413	0.3096	-0.5177	0.5984	-0.1183
	-0.2614	0.5351	-40.5309	
42.3000	-39.9500	-0.5275	-0.1107	
0.3864	0.3515	-0.4284	0.6091	-0.1234
	-0.2552	0.5370	-40.5315	
42.3200	-39.2900	0.1896	0.0415	
0.2675	0.3664	-0.3420	0.6194	-0.1284
	-0.2489	0.5389	-40.5321	
42.3400	-39.0900	0.1941	0.2130	
0.1247	0.3548	-0.2588	0.6293	-0.1335
	-0.2426	0.5409	-40.5327	
42.3600	-39.1400	0.2414	0.0881	-
0.0003	0.3190	-0.1791	0.6387	-0.1385
	-0.2363	0.5428	-40.5333	
42.3800	-40.2800	-0.4652	-0.2070	-
0.0802	0.2622	-0.1028	0.6477	-0.1435
	-0.2299	0.5447	-40.5338	
42.4000	-40.0500	-0.0106	-0.3312	-
0.1045	0.1885	-0.0298	0.6561	-0.1485
	-0.2235	0.5466	-40.5344	
42.4200	-39.6200	0.1254	-0.1687	-
0.0813	0.1029	0.0403	0.6639	-0.1534
	-0.2171	0.5485	-40.5350	
42.4400	-39.8400	-0.3280	0.1298	-
0.0370	0.0115	0.1077	0.6710	-0.1583
	-0.2106	0.5505	-40.5355	
42.4600	-38.9600	0.4183	0.2956	-
0.0051	-0.0797	0.1727	0.6775	-0.1631
	-0.2041	0.5524	-40.5361	
42.4800	-38.9100	0.5152	0.2035	-
0.0084	-0.1649	0.2357	0.6833	-0.1679
	-0.1975	0.5543	-40.5367	
42.5000	-40.1900	-0.4790	-0.0513	-
0.0487	-0.2398	0.2970	0.6883	-0.1726
	-0.1910	0.5562	-40.5372	
42.5200	-40.3300	-0.5391	-0.1694	-
0.1130	-0.3010	0.3569	0.6925	-0.1773
	-0.1844	0.5581	-40.5378	

42.5400 -39.5200 0.2769 -0.0661 -
0.1814 -0.3460 0.4158 0.6959 -0.1819
-0.1778 0.5601 -40.5383
42.5600 -39.1800 0.5608 0.0230 -
0.2283 -0.3735 0.4739 0.6984 -0.1864
-0.1711 0.5620 -40.5389
42.5800 -40.3200 -0.5540 -0.0688 -
0.2345 -0.3829 0.5315 0.6999 -0.1908
-0.1645 0.5639 -40.5395
42.6000 -39.3300 0.3589 -0.1620 -
0.2005 -0.3753 0.5890 0.7005 -0.1951
-0.1578 0.5658 -40.5400
42.6200 -39.7600 -0.2525 -0.0564 -
0.1417 -0.3529 0.6464 0.7000 -0.1994
-0.1511 0.5677 -40.5406
42.6400 -39.4800 -0.1819 0.1328 -
0.0779 -0.3182 0.7040 0.6985 -0.2035
-0.1444 0.5697 -40.5411
42.6600 -38.7900 0.1654 0.2507 -
0.0277 -0.2743 0.7619 0.6960 -0.2076
-0.1377 0.5716 -40.5416
42.6800 -38.4400 0.4241 0.1435
0.0007 -0.2244 0.8203 0.6923 -0.2115
-0.1310 0.5735 -40.5422
42.7000 -39.6300 -0.4426 -0.1192
0.0119 -0.1722 0.8794 0.6875 -0.2153
-0.1242 0.5754 -40.5427
42.7200 -39.1000 -0.1451 -0.2149
0.0186 -0.1218 0.9393 0.6814 -0.2191
-0.1175 0.5773 -40.5433
42.7400 -38.4700 0.3114 -0.0661
0.0337 -0.0767 1.0001 0.6742 -0.2227
-0.1107 0.5793 -40.5438
42.7600 -38.5300 -0.0940 0.1010
0.0628 -0.0402 1.0618 0.6656 -0.2262
-0.1040 0.5812 -40.5443
42.7800 -38.2800 0.1066 0.0720
0.1046 -0.0152 1.1244 0.6558 -0.2295
-0.0972 0.5831 -40.5449
42.8000 -38.3800 0.0477 -0.0919
0.1483 -0.0039 1.1875 0.6447 -0.2328
-0.0904 0.5850 -40.5454
42.8200 -38.6300 -0.1999 -0.1516
0.1787 -0.0064 1.2508 0.6322 -0.2359
-0.0836 0.5870 -40.5459
42.8400 -38.2600 -0.0384 -0.0004
0.1823 -0.0219 1.3133 0.6183 -0.2389
-0.0769 0.5889 -40.5465
42.8600 -37.7700 0.2327 0.2228
0.1487 -0.0478 1.3744 0.6029 -0.2418
-0.0701 0.5908 -40.5470

42.8800 -38.1200 -0.1467 0.2707
0.0780 -0.0802 1.4327 0.5862 -0.2445
-0.0633 0.5927 -40.5475
42.9000 -38.0400 0.2830 0.0809 -
0.0169 -0.1142 1.4873 0.5680 -0.2471
-0.0566 0.5947 -40.5480
42.9200 -38.7500 -0.1243 -0.1758 -
0.1095 -0.1454 1.5368 0.5483 -0.2496
-0.0498 0.5966 -40.5485
42.9400 -38.9300 -0.1172 -0.2983 -
0.1719 -0.1694 1.5802 0.5271 -0.2519
-0.0430 0.5985 -40.5491
42.9600 -38.6000 0.0405 -0.1919 -
0.1871 -0.1820 1.6162 0.5044 -0.2541
-0.0363 0.6005 -40.5496
42.9800 -38.3500 0.0316 0.0527 -
0.1622 -0.1795 1.6438 0.4802 -0.2561
-0.0295 0.6024 -40.5501
43.0000 -38.1200 0.0404 0.2307 -
0.1199 -0.1584 1.6623 0.4545 -0.2580
-0.0228 0.6043 -40.5506
43.0200 -38.1000 -0.0029 0.2436 -
0.0879 -0.1167 1.6707 0.4273 -0.2598
-0.0161 0.6063 -40.5511
43.0400 -38.3400 -0.1980 0.1395 -
0.0846 -0.0536 1.6687 0.3987 -0.2614
-0.0094 0.6082 -40.5516
43.0600 -38.0500 0.2692 -0.0366 -
0.1082 0.0286 1.6558 0.3686 -0.2629
-0.0027 0.6101 -40.5521
43.0800 -38.2800 0.1350 -0.2540 -
0.1453 0.1257 1.6320 0.3371 -0.2642
0.0040 0.6121 -40.5526
43.1000 -38.8500 -0.3295 -0.3199 -
0.1803 0.2320 1.5970 0.3043 -0.2654
0.0106 0.6140 -40.5531
43.1200 -38.5600 -0.2624 -0.0736 -
0.1973 0.3412 1.5506 0.2703 -0.2664
0.0173 0.6160 -40.5536
43.1400 -37.5200 0.3726 0.2946 -
0.1853 0.4468 1.4930 0.2350 -0.2673
0.0239 0.6179 -40.5541
43.1600 -37.4100 0.3057 0.4235 -
0.1328 0.5420 1.4239 0.1987 -0.2680
0.0305 0.6199 -40.5546
43.1800 -37.7500 0.3341 0.1150 -
0.0352 0.6199 1.3434 0.1614 -0.2685
0.0371 0.6218 -40.5551
43.2000 -38.9600 -0.6191 -0.3350
0.1027 0.6740 1.2520 0.1231 -0.2689
0.0436 0.6238 -40.5556

43.2200 -38.1500 0.2314 -0.4949
0.2680 0.6980 1.1499 0.0840 -0.2692
0.0502 0.6257 -40.5561
43.2400 -37.9500 0.2797 -0.3983
0.4402 0.6866 1.0378 0.0442 -0.2693
0.0567 0.6277 -40.5566
43.2600 -38.3200 -0.2742 -0.1680
0.5847 0.6363 0.9169 0.0038 -0.2692
0.0631 0.6297 -40.5571
43.2800 -37.6300 0.2432 0.2340
0.6458 0.5465 0.7884 -0.0371 -0.2689
0.0696 0.6316 -40.5576
43.3000 -38.4600 -0.6064 0.6759
0.5723 0.4205 0.6538 -0.0785 -0.2685
0.0760 0.6336 -40.5580
43.3200 -37.6600 0.8694 0.5823
0.3517 0.2666 0.5148 -0.1203 -0.2679
0.0824 0.6356 -40.5585
43.3400 -39.2000 0.5853 -0.0785
0.0381 0.0958 0.3728 -0.1623 -0.2672
0.0888 0.6375 -40.5590
43.3600 -42.2300 -1.1527 -0.6440 -
0.2770 -0.0807 0.2294 -0.2044 -0.2662
0.0951 0.6395 -40.5595
43.3800 -40.4200 1.0572 -0.4723 -
0.5062 -0.2515 0.0862 -0.2467 -0.2651
0.1014 0.6415 -40.5599
43.4000 -42.2200 -0.7878 0.0146 -
0.6084 -0.4056 -0.0554 -0.2889 -0.2639
0.1076 0.6435 -40.5604
43.4200 -41.3300 0.1380 0.2725 -
0.5869 -0.5345 -0.1938 -0.3310 -0.2624
0.1139 0.6454 -40.5609
43.4400 -41.3900 0.2445 0.2767 -
0.4779 -0.6321 -0.3274 -0.3728 -0.2608
0.1201 0.6474 -40.5614
43.4600 -41.9800 -0.1209 0.1508 -
0.3269 -0.6949 -0.4549 -0.4144 -0.2590
0.1262 0.6494 -40.5618
43.4800 -42.0800 -0.1456 0.0376 -
0.1683 -0.7222 -0.5750 -0.4555 -0.2570
0.1323 0.6514 -40.5623
43.5000 -41.8800 0.0973 -0.0412 -
0.0174 -0.7162 -0.6865 -0.4962 -0.2549
0.1384 0.6534 -40.5627
43.5200 -41.6900 0.2897 -0.1585
0.1197 -0.6810 -0.7887 -0.5363 -0.2526
0.1444 0.6554 -40.5632
43.5400 -42.4900 -0.4795 -0.2054
0.2348 -0.6205 -0.8807 -0.5757 -0.2501
0.1504 0.6574 -40.5637

43.5600 -42.0200 -0.0936 -0.0128
0.3127 -0.5380 -0.9622 -0.6143 -0.2474
0.1564 0.6594 -40.5641
43.5800 -41.1700 0.3185 0.2583
0.3315 -0.4367 -1.0329 -0.6520 -0.2445
0.1623 0.6614 -40.5646
43.6000 -41.1600 0.4247 0.3214
0.2759 -0.3206 -1.0931 -0.6888 -0.2414
0.1681 0.6634 -40.5650
43.6200 -41.8000 -0.1331 0.1320
0.1557 -0.1946 -1.1428 -0.7246 -0.2381
0.1740 0.6655 -40.5655
43.6400 -42.5500 -0.4793 -0.0850
0.0020 -0.0634 -1.1826 -0.7592 -0.2347
0.1797 0.6675 -40.5659
43.6600 -42.1600 -0.0007 -0.1317 -
0.1478 0.0677 -1.2128 -0.7927 -0.2311
0.1854 0.6695 -40.5664
43.6800 -41.8600 0.2312 -0.0961 -
0.2491 0.1939 -1.2338 -0.8248 -0.2272
0.1911 0.6715 -40.5668
43.7000 -42.0400 0.1188 -0.1843 -
0.2659 0.3109 -1.2459 -0.8555 -0.2232
0.1968 0.6736 -40.5672
43.7200 -42.3400 -0.1390 -0.3828 -
0.1934 0.4149 -1.2496 -0.8848 -0.2190
0.2023 0.6756 -40.5677
43.7400 -42.2200 -0.1282 -0.4454 -
0.0559 0.5023 -1.2450 -0.9125 -0.2146
0.2079 0.6776 -40.5681
43.7600 -41.5600 -0.1082 -0.1457
0.1047 0.5704 -1.2323 -0.9386 -0.2100
0.2133 0.6797 -40.5686
43.7800 -41.1600 -0.3789 0.4262
0.2424 0.6166 -1.2117 -0.9631 -0.2052
0.2188 0.6817 -40.5690
43.8000 -39.6500 0.6738 0.7803
0.3132 0.6387 -1.1833 -0.9860 -0.2002
0.2241 0.6838 -40.5694
43.8200 -40.3500 0.2898 0.5005
0.2866 0.6351 -1.1473 -1.0071 -0.1950
0.2295 0.6859 -40.5699
43.8400 -42.0900 -0.7168 -0.1876
0.1842 0.6071 -1.1040 -1.0266 -0.1896
0.2347 0.6879 -40.5703
43.8600 -41.4900 0.5421 -0.7346
0.0444 0.5569 -1.0539 -1.0444 -0.1840
0.2399 0.6900 -40.5707
43.8800 -42.7000 -0.6294 -0.6624 -
0.0964 0.4875 -0.9975 -1.0605 -0.1782
0.2451 0.6921 -40.5711

43.9000 -41.2400 0.5340 -0.1065 -
0.2110 0.4022 -0.9355 -1.0750 -0.1723
0.2502 0.6941 -40.5716
43.9200 -41.2100 0.1296 0.4504 -
0.2856 0.3045 -0.8684 -1.0879 -0.1661
0.2552 0.6962 -40.5720
43.9400 -41.4700 -0.4391 0.6839 -
0.3144 0.1985 -0.7972 -1.0992 -0.1597
0.2602 0.6983 -40.5724
43.9600 -40.9800 0.4087 0.4802 -
0.2967 0.0881 -0.7224 -1.1089 -0.1531
0.2652 0.7004 -40.5728
43.9800 -41.4400 0.3535 -0.0447 -
0.2313 -0.0231 -0.6447 -1.1172 -0.1464
0.2700 0.7025 -40.5732
44.0000 -42.5700 -0.5504 -0.5276 -
0.1169 -0.1317 -0.5649 -1.1240 -0.1395
0.2748 0.7046 -40.5736
44.0200 -41.9200 0.1152 -0.5646
0.0442 -0.2342 -0.4835 -1.1294 -0.1324
0.2796 0.7067 -40.5740
44.0400 -41.5100 0.0754 -0.2306
0.2318 -0.3268 -0.4012 -1.1334 -0.1251
0.2843 0.7088 -40.5744
44.0600 -40.7900 0.0511 0.1567
0.4068 -0.4060 -0.3185 -1.1362 -0.1176
0.2889 0.7109 -40.5749
44.0800 -40.7400 0.0409 0.3251
0.5251 -0.4683 -0.2360 -1.1377 -0.1099
0.2935 0.7130 -40.5753
44.1000 -40.5200 -0.0318 0.2889
0.5478 -0.5110 -0.1543 -1.1380 -0.1021
0.2980 0.7152 -40.5757
44.1200 -40.8400 -0.1113 0.1659
0.4596 -0.5322 -0.0738 -1.1372 -0.0941
0.3024 0.7173 -40.5761
44.1400 -40.7400 0.2906 -0.0397
0.2752 -0.5316 0.0049 -1.1353 -0.0860
0.3068 0.7194 -40.5765
44.1600 -41.2400 0.1126 -0.2021
0.0257 -0.5102 0.0813 -1.1323 -0.0777
0.3111 0.7216 -40.5769
44.1800 -42.0500 -0.7283 -0.1254 -
0.2459 -0.4697 0.1551 -1.1283 -0.0692
0.3154 0.7237 -40.5772
44.2000 -40.5400 0.8078 0.0900 -
0.4930 -0.4120 0.2259 -1.1233 -0.0606
0.3196 0.7259 -40.5776
44.2200 -41.7000 -0.4052 0.0929 -
0.6762 -0.3390 0.2932 -1.1174 -0.0518
0.3237 0.7280 -40.5780

44.2400 -41.7100 -0.3725 -0.0268 -
0.7658 -0.2528 0.3569 -1.1105 -0.0429
0.3277 0.7302 -40.5784
44.2600 -41.3400 -0.0234 -0.0597 -
0.7488 -0.1560 0.4165 -1.1028 -0.0338
0.3317 0.7324 -40.5788
44.2800 -40.8700 -0.0324 0.0154 -
0.6284 -0.0527 0.4716 -1.0942 -0.0246
0.3356 0.7346 -40.5792
44.3000 -40.3100 0.1441 0.0233 -
0.4240 0.0525 0.5220 -1.0849 -0.0152
0.3395 0.7367 -40.5796
44.3200 -39.8700 0.2554 -0.1041 -
0.1623 0.1551 0.5671 -1.0747 -0.0057
0.3433 0.7389 -40.5799
44.3400 -40.2200 -0.5112 -0.1765
0.1264 0.2502 0.6068 -1.0639 0.0039
0.3470 0.7411 -40.5803
44.3600 -39.1100 0.1679 -0.0019
0.4079 0.3334 0.6404 -1.0523 0.0136
0.3506 0.7433 -40.5807
44.3800 -38.3900 0.1430 0.2213
0.6484 0.3998 0.6678 -1.0401 0.0235
0.3542 0.7455 -40.5811
44.4000 -38.1200 0.1760 0.1985
0.8160 0.4449 0.6886 -1.0272 0.0335
0.3577 0.7478 -40.5814
44.4200 -38.5900 -0.1730 -0.0486
0.8812 0.4648 0.7025 -1.0138 0.0436
0.3611 0.7500 -40.5818
44.4400 -38.5200 0.0998 -0.2093
0.8281 0.4578 0.7093 -0.9998 0.0538
0.3644 0.7522 -40.5822
44.4600 -38.9400 -0.4371 0.0510
0.6543 0.4257 0.7092 -0.9853 0.0642
0.3677 0.7544 -40.5825
44.4800 -37.9500 0.5614 0.4643
0.3837 0.3728 0.7025 -0.9703 0.0746
0.3709 0.7567 -40.5829
44.5000 -39.2200 -0.4534 0.4675
0.0603 0.3044 0.6898 -0.9549 0.0851
0.3741 0.7589 -40.5833
44.5200 -39.2100 0.3064 0.1081 -
0.2645 0.2253 0.6715 -0.9391 0.0957
0.3771 0.7612 -40.5836
44.5400 -40.3300 -0.0439 -0.3397 -
0.5379 0.1407 0.6482 -0.9229 0.1064
0.3801 0.7634 -40.5840
44.5600 -40.6300 0.2631 -0.7035 -
0.7055 0.0555 0.6205 -0.9063 0.1172
0.3830 0.7657 -40.5843

44.5800 -41.6100 -0.6564 -0.6401 -
0.7285 -0.0255 0.5890 -0.8895 0.1281
0.3858 0.7680 -40.5847
44.6000 -39.9100 0.7326 -0.1744 -
0.6131 -0.0989 0.5541 -0.8723 0.1390
0.3886 0.7703 -40.5851
44.6200 -40.0500 -0.1774 0.2600 -
0.4060 -0.1628 0.5165 -0.8548 0.1500
0.3912 0.7726 -40.5854
44.6400 -40.0600 -0.5966 0.5142 -
0.1649 -0.2160 0.4769 -0.8371 0.1610
0.3938 0.7748 -40.5858
44.6600 -39.3200 -0.0127 0.5874
0.0568 -0.2571 0.4356 -0.8192 0.1721
0.3963 0.7771 -40.5861
44.6800 -38.7300 0.4884 0.4550
0.2245 -0.2855 0.3933 -0.8010 0.1833
0.3988 0.7795 -40.5864
44.7000 -39.6700 -0.2823 0.1118
0.3232 -0.3007 0.3506 -0.7827 0.1945
0.4011 0.7818 -40.5868
44.7200 -40.0100 -0.1229 -0.2407
0.3574 -0.3035 0.3080 -0.7642 0.2057
0.4034 0.7841 -40.5871
44.7400 -39.8900 0.0096 -0.3879
0.3388 -0.2953 0.2660 -0.7455 0.2169
0.4056 0.7864 -40.5875
44.7600 -39.9500 0.0375 -0.3069
0.2856 -0.2786 0.2252 -0.7268 0.2282
0.4077 0.7888 -40.5878
44.7800 -39.8400 -0.1888 -0.0500
0.2119 -0.2560 0.1859 -0.7079 0.2394
0.4097 0.7911 -40.5881
44.8000 -39.1700 0.3714 0.2108
0.1251 -0.2294 0.1485 -0.6890 0.2507
0.4117 0.7935 -40.5885
44.8200 -39.8200 -0.2626 0.2447
0.0322 -0.2005 0.1132 -0.6700 0.2619
0.4135 0.7958 -40.5888
44.8400 -39.6400 0.1682 0.0682 -
0.0556 -0.1709 0.0802 -0.6509 0.2731
0.4153 0.7982 -40.5891
44.8600 -40.2300 -0.1837 -0.1088 -
0.1236 -0.1413 0.0497 -0.6318 0.2843
0.4170 0.8006 -40.5894
44.8800 -40.2100 -0.1715 -0.1605 -
0.1545 -0.1120 0.0215 -0.6126 0.2955
0.4186 0.8030 -40.5898
44.9000 -39.7100 0.3757 -0.1517 -
0.1393 -0.0827 -0.0043 -0.5934 0.3066
0.4201 0.8054 -40.5901

44.9200 -39.7300 0.2567 -0.1507 -
0.0876 -0.0527 -0.0276 -0.5740 0.3177
0.4215 0.8078 -40.5904
44.9400 -40.3100 -0.5591 -0.0556 -
0.0261 -0.0214 -0.0487 -0.5547 0.3287
0.4229 0.8102 -40.5907
44.9600 -39.8000 -0.3594 0.2071
0.0171 0.0117 -0.0676 -0.5352 0.3397
0.4241 0.8126 -40.5910
44.9800 -38.5200 0.5547 0.4254
0.0254 0.0463 -0.0844 -0.5157 0.3505
0.4253 0.8150 -40.5914
45.0000 -38.8800 0.3800 0.3073 -
0.0061 0.0815 -0.0992 -0.4960 0.3613
0.4264 0.8174 -40.5917
45.0200 -40.0000 -0.5107 -0.0948 -
0.0586 0.1160 -0.1120 -0.4763 0.3720
0.4274 0.8199 -40.5920
45.0400 -40.0700 -0.1287 -0.4407 -
0.1014 0.1484 -0.1229 -0.4564 0.3826
0.4283 0.8223 -40.5923
45.0600 -39.8000 0.1458 -0.4893 -
0.1073 0.1771 -0.1319 -0.4364 0.3931
0.4291 0.8248 -40.5926
45.0800 -39.3300 0.2077 -0.2805 -
0.0685 0.2003 -0.1392 -0.4162 0.4035
0.4298 0.8272 -40.5929
45.1000 -39.6500 -0.4940 0.1097
0.0015 0.2161 -0.1447 -0.3959 0.4138
0.4305 0.8297 -40.5932
45.1200 -38.2300 0.3962 0.4850
0.0776 0.2227 -0.1484 -0.3753 0.4239
0.4310 0.8322 -40.5935
45.1400 -38.5700 -0.0480 0.5270
0.1364 0.2186 -0.1505 -0.3546 0.4339
0.4314 0.8347 -40.5938
45.1600 -38.7700 0.0673 0.1823
0.1664 0.2032 -0.1510 -0.3336 0.4437
0.4318 0.8372 -40.5941
45.1800 -39.1600 0.1482 -0.2728
0.1703 0.1766 -0.1500 -0.3123 0.4534
0.4321 0.8397 -40.5944
45.2000 -39.7700 -0.3329 -0.4960
0.1547 0.1402 -0.1475 -0.2907 0.4629
0.4323 0.8422 -40.5947
45.2200 -39.6300 -0.2913 -0.3061
0.1231 0.0957 -0.1436 -0.2688 0.4723
0.4323 0.8447 -40.5950
45.2400 -38.5500 0.3467 0.1201
0.0769 0.0459 -0.1385 -0.2465 0.4814
0.4323 0.8472 -40.5953

45.2600 -38.6100 0.2113 0.3896
0.0153 -0.0062 -0.1323 -0.2239 0.4904
0.4322 0.8497 -40.5955
45.2800 -38.9500 -0.0148 0.3392 -
0.0614 -0.0576 -0.1252 -0.2007 0.4992
0.4320 0.8523 -40.5958
45.3000 -39.4400 -0.2444 0.1382 -
0.1446 -0.1048 -0.1173 -0.1771 0.5078
0.4318 0.8548 -40.5961
45.3200 -39.5300 -0.0158 -0.0251 -
0.2199 -0.1450 -0.1088 -0.1530 0.5162
0.4314 0.8574 -40.5964
45.3400 -39.4500 0.0923 -0.1409 -
0.2651 -0.1757 -0.0998 -0.1283 0.5244
0.4309 0.8599 -40.5967
45.3600 -39.6100 0.0445 -0.2616 -
0.2579 -0.1955 -0.0905 -0.1030 0.5323
0.4303 0.8625 -40.5969
45.3800 -39.4400 0.1705 -0.3372 -
0.1905 -0.2036 -0.0811 -0.0771 0.5400
0.4297 0.8651 -40.5972
45.4000 -39.7200 -0.3111 -0.2539 -
0.0763 -0.2009 -0.0718 -0.0506 0.5475
0.4289 0.8677 -40.5975
45.4200 -38.8500 0.1764 0.0126
0.0566 -0.1888 -0.0629 -0.0233 0.5548
0.4281 0.8703 -40.5978
45.4400 -38.3800 0.0735 0.2849
0.1726 -0.1692 -0.0544 0.0048 0.5618
0.4272 0.8729 -40.5980
45.4600 -38.4000 -0.1916 0.4007
0.2402 -0.1439 -0.0467 0.0336 0.5686
0.4261 0.8755 -40.5983
45.4800 -38.1000 0.1911 0.3225
0.2443 -0.1147 -0.0398 0.0632 0.5751
0.4250 0.8781 -40.5985
45.5000 -38.7200 -0.1993 0.0848
0.1922 -0.0832 -0.0342 0.0936 0.5814
0.4238 0.8807 -40.5988
45.5200 -38.5700 0.1546 -0.2138
0.1077 -0.0505 -0.0300 0.1249 0.5874
0.4225 0.8834 -40.5991
45.5400 -38.9100 -0.0545 -0.3725
0.0184 -0.0176 -0.0274 0.1571 0.5931
0.4210 0.8860 -40.5993
45.5600 -39.0900 -0.3308 -0.2510 -
0.0536 0.0145 -0.0267 0.1901 0.5985
0.4195 0.8886 -40.5996
45.5800 -38.3700 0.0875 0.0374 -
0.0959 0.0449 -0.0282 0.2240 0.6037
0.4179 0.8913 -40.5998

45.6000 -37.8900 0.3920 0.1871 -
0.1065 0.0732 -0.0319 0.2588 0.6086
0.4163 0.8940 -40.6001
45.6200 -38.5500 -0.1837 0.1027 -
0.0954 0.0989 -0.0381 0.2944 0.6132
0.4145 0.8966 -40.6003
45.6400 -38.6100 -0.2976 0.0094 -
0.0774 0.1216 -0.0469 0.3309 0.6175
0.4126 0.8993 -40.6006
45.6600 -38.0600 0.0854 0.0514 -
0.0613 0.1405 -0.0583 0.3681 0.6215
0.4106 0.9020 -40.6008
45.6800 -38.0600 -0.0020 0.0880 -
0.0433 0.1542 -0.0724 0.4060 0.6252
0.4086 0.9047 -40.6011
45.7000 -37.9500 0.3161 -0.0500 -
0.0139 0.1614 -0.0892 0.4445 0.6285
0.4064 0.9074 -40.6013
45.7200 -38.5300 -0.2529 -0.2242
0.0291 0.1608 -0.1084 0.4836 0.6316
0.4041 0.9101 -40.6016
45.7400 -38.4000 -0.2713 -0.1755
0.0795 0.1516 -0.1298 0.5231 0.6344
0.4018 0.9128 -40.6018
45.7600 -37.8400 0.0976 0.0926
0.1217 0.1339 -0.1530 0.5631 0.6368
0.3993 0.9155 -40.6020
45.7800 -37.3900 0.2848 0.2792
0.1351 0.1083 -0.1775 0.6032 0.6389
0.3968 0.9182 -40.6023
45.8000 -37.9200 -0.0683 0.1880
0.1055 0.0761 -0.2029 0.6436 0.6406
0.3942 0.9210 -40.6025
45.8200 -38.2200 -0.0776 -0.0618
0.0435 0.0392 -0.2286 0.6839 0.6421
0.3915 0.9237 -40.6027
45.8400 -38.5400 -0.0926 -0.2252 -
0.0288 -0.0002 -0.2539 0.7242 0.6431
0.3886 0.9264 -40.6030
45.8600 -38.5800 -0.0616 -0.1644 -
0.0873 -0.0399 -0.2783 0.7643 0.6439
0.3857 0.9292 -40.6032
45.8800 -38.2900 0.1445 -0.0004 -
0.1138 -0.0777 -0.3011 0.8040 0.6442
0.3827 0.9320 -40.6034
45.9000 -38.2900 -0.0209 0.1002 -
0.1046 -0.1115 -0.3217 0.8432 0.6442
0.3797 0.9347 -40.6036
45.9200 -38.2900 -0.1175 0.1010 -
0.0691 -0.1398 -0.3395 0.8818 0.6439
0.3765 0.9375 -40.6038

45.9400 -38.2900 -0.0308 0.0417 -
0.0195 -0.1617 -0.3541 0.9197 0.6432
0.3732 0.9403 -40.6041
45.9600 -38.2300 0.0326 -0.0451
0.0315 -0.1761 -0.3648 0.9568 0.6421
0.3698 0.9431 -40.6043
45.9800 -38.0000 0.2199 -0.1113
0.0735 -0.1826 -0.3715 0.9929 0.6406
0.3664 0.9459 -40.6045
46.0000 -38.6200 -0.3944 -0.0743
0.0987 -0.1811 -0.3737 1.0280 0.6387
0.3628 0.9487 -40.6047
46.0200 -37.5700 0.3856 0.1033
0.0982 -0.1715 -0.3713 1.0618 0.6365
0.3592 0.9515 -40.6049
46.0400 -38.0500 -0.3042 0.2494
0.0675 -0.1542 -0.3640 1.0944 0.6339
0.3555 0.9543 -40.6051
46.0600 -37.5300 0.4201 0.1231
0.0154 -0.1297 -0.3518 1.1256 0.6308
0.3517 0.9571 -40.6053
46.0800 -38.4300 -0.2483 -0.1843 -
0.0379 -0.0987 -0.3346 1.1553 0.6274
0.3478 0.9599 -40.6055
46.1000 -38.4000 -0.2009 -0.3388 -
0.0695 -0.0625 -0.3125 1.1834 0.6236
0.3438 0.9628 -40.6057
46.1200 -37.9400 0.1803 -0.1711 -
0.0684 -0.0227 -0.2854 1.2099 0.6193
0.3397 0.9656 -40.6059
46.1400 -37.6400 0.0400 0.0999 -
0.0434 0.0183 -0.2534 1.2346 0.6147
0.3356 0.9685 -40.6061
46.1600 -37.2400 0.2685 0.1934 -
0.0102 0.0577 -0.2166 1.2575 0.6096
0.3314 0.9713 -40.6063
46.1800 -37.5100 -0.1171 0.1047
0.0168 0.0924 -0.1750 1.2784 0.6041
0.3270 0.9742 -40.6065
46.2000 -37.5800 -0.2418 0.0318
0.0305 0.1192 -0.1288 1.2974 0.5982
0.3227 0.9770 -40.6067
46.2200 -37.4000 -0.0721 0.0409
0.0317 0.1355 -0.0781 1.3142 0.5918
0.3182 0.9799 -40.6069
46.2400 -36.6800 0.5053 -0.0170
0.0302 0.1387 -0.0232 1.3288 0.5850
0.3137 0.9828 -40.6071
46.2600 -37.5500 -0.1928 -0.1664
0.0373 0.1272 0.0355 1.3411 0.5778
0.3090 0.9857 -40.6073

46.2800 -37.7100 -0.3755 -0.1935
0.0553 0.1004 0.0976 1.3511 0.5702
0.3044 0.9885 -40.6074
46.3000 -36.7100 0.4004 -0.0101
0.0749 0.0591 0.1624 1.3585 0.5621
0.2996 0.9914 -40.6076
46.3200 -37.2000 -0.2972 0.2114
0.0805 0.0053 0.2289 1.3634 0.5536
0.2948 0.9943 -40.6078
46.3400 -36.5600 0.2986 0.2953
0.0554 -0.0583 0.2963 1.3656 0.5446
0.2899 0.9972 -40.6080
46.3600 -37.1100 -0.1676 0.2209 -
0.0051 -0.1274 0.3633 1.3651 0.5353
0.2849 1.0002 -40.6082
46.3800 -37.2600 -0.0055 0.0632 -
0.0860 -0.1967 0.4290 1.3617 0.5255
0.2799 1.0031 -40.6083
46.4000 -37.5900 -0.0601 -0.1130 -
0.1557 -0.2605 0.4919 1.3553 0.5153
0.2748 1.0060 -40.6085
46.4200 -37.3900 0.2835 -0.2970 -
0.1819 -0.3133 0.5510 1.3458 0.5046
0.2696 1.0089 -40.6087
46.4400 -38.0500 -0.2406 -0.3712 -
0.1469 -0.3497 0.6050 1.3332 0.4936
0.2644 1.0119 -40.6088
46.4600 -37.9000 -0.3284 -0.1792 -
0.0569 -0.3650 0.6528 1.3173 0.4823
0.2591 1.0148 -40.6090
46.4800 -36.5600 0.3502 0.1742
0.0567 -0.3555 0.6935 1.2981 0.4705
0.2538 1.0177 -40.6092
46.5000 -36.5700 0.1012 0.3595
0.1573 -0.3187 0.7264 1.2755 0.4584
0.2484 1.0207 -40.6093
46.5200 -36.9100 -0.1873 0.2683
0.2122 -0.2526 0.7510 1.2495 0.4460
0.2430 1.0236 -40.6095
46.5400 -36.9100 -0.0146 0.0325
0.2006 -0.1573 0.7670 1.2200 0.4332
0.2375 1.0266 -40.6096
46.5600 -36.6200 0.4593 -0.1777
0.1183 -0.0356 0.7741 1.1870 0.4201
0.2319 1.0296 -40.6098
46.5800 -37.5600 -0.3795 -0.1964 -
0.0230 0.1060 0.7723 1.1508 0.4067
0.2263 1.0325 -40.6100
46.6000 -37.7200 -0.6677 -0.0077 -
0.1981 0.2590 0.7612 1.1113 0.3930
0.2207 1.0355 -40.6101

46.6200 -36.0300 0.8937 0.1822 -
0.3680 0.4133 0.7407 1.0687 0.3791
0.2150 1.0385 -40.6103
46.6400 -37.1100 0.1067 0.0257 -
0.4881 0.5585 0.7106 1.0233 0.3649
0.2093 1.0415 -40.6104
46.6600 -37.8700 -0.2941 -0.3174 -
0.5136 0.6843 0.6709 0.9751 0.3504
0.2035 1.0445 -40.6105
46.6800 -38.1900 -0.7129 -0.3485 -
0.4111 0.7822 0.6216 0.9245 0.3357
0.1977 1.0475 -40.6107
46.7000 -36.4300 0.6377 -0.0761 -
0.1791 0.8460 0.5627 0.8715 0.3208
0.1918 1.0505 -40.6108
46.7200 -36.6800 -0.0513 0.0459
0.1478 0.8711 0.4949 0.8163 0.3057
0.1859 1.0535 -40.6110
46.7400 -36.7400 -0.0410 -0.0882
0.5058 0.8538 0.4187 0.7592 0.2904
0.1800 1.0565 -40.6111
46.7600 -36.7500 -0.1090 -0.1523
0.8029 0.7904 0.3353 0.7004 0.2749
0.1740 1.0595 -40.6112
46.7800 -36.6200 -0.1028 0.1392
0.9503 0.6787 0.2460 0.6400 0.2592
0.1680 1.0625 -40.6114
46.8000 -36.5700 -0.3255 0.6366
0.8980 0.5212 0.1522 0.5782 0.2434
0.1620 1.0656 -40.6115
46.8200 -36.1900 0.6699 0.7504
0.6391 0.3275 0.0555 0.5152 0.2274
0.1559 1.0686 -40.6116
46.8400 -38.6600 -0.3906 0.1698
0.2274 0.1104 -0.0429 0.4512 0.2113
0.1498 1.0716 -40.6118
46.8600 -39.7600 -0.0337 -0.5216 -
0.2340 -0.1166 -0.1413 0.3864 0.1951
0.1437 1.0747 -40.6119
46.8800 -40.9100 -0.1945 -0.7553 -
0.6341 -0.3401 -0.2385 0.3209 0.1788
0.1375 1.0777 -40.6120
46.9000 -41.0400 0.3061 -0.5126 -
0.8812 -0.5466 -0.3328 0.2550 0.1624
0.1313 1.0807 -40.6121
46.9200 -41.4700 -0.2580 -0.0633 -
0.9392 -0.7235 -0.4230 0.1889 0.1459
0.1251 1.0838 -40.6123
46.9400 -40.8100 0.2524 0.2703 -
0.8180 -0.8608 -0.5075 0.1228 0.1294
0.1189 1.0868 -40.6124

46.9600 -41.2100 -0.1948 0.3099 -
0.5531 -0.9542 -0.5849 0.0567 0.1128
0.1127 1.0899 -40.6125
46.9800 -40.8700 0.2780 0.0647 -
0.2010 -1.0041 -0.6538 -0.0090 0.0962
0.1064 1.0930 -40.6126
47.0000 -41.3200 -0.1363 -0.2538
0.1585 -1.0128 -0.7129 -0.0741 0.0796
0.1001 1.0960 -40.6127
47.0200 -41.4700 -0.3170 -0.2951
0.4414 -0.9828 -0.7613 -0.1386 0.0629
0.0938 1.0991 -40.6128
47.0400 -40.7300 -0.0713 0.0445
0.5978 -0.9167 -0.7986 -0.2022 0.0462
0.0875 1.1022 -40.6129
47.0600 -39.9800 0.2967 0.4195
0.6166 -0.8178 -0.8247 -0.2647 0.0296
0.0812 1.1053 -40.6130
47.0800 -40.0500 0.2803 0.4575
0.5069 -0.6911 -0.8402 -0.3259 0.0130
0.0748 1.1083 -40.6131
47.1000 -40.9300 -0.2171 0.1367
0.3065 -0.5435 -0.8455 -0.3857 -0.0035
0.0685 1.1114 -40.6132
47.1200 -41.1800 0.2036 -0.2793
0.0762 -0.3828 -0.8414 -0.4440 -0.0200
0.0621 1.1145 -40.6133
47.1400 -42.0400 -0.4955 -0.4278 -
0.1196 -0.2172 -0.8285 -0.5005 -0.0365
0.0558 1.1176 -40.6134
47.1600 -40.9700 0.3436 -0.1614 -
0.2376 -0.0540 -0.8075 -0.5553 -0.0528
0.0494 1.1207 -40.6135
47.1800 -40.9100 -0.1084 0.2348 -
0.2785 0.1009 -0.7792 -0.6083 -0.0691
0.0431 1.1238 -40.6136
47.2000 -40.2100 0.3975 0.3054 -
0.2606 0.2439 -0.7443 -0.6593 -0.0852
0.0367 1.1269 -40.6137
47.2200 -41.2500 -0.5169 0.0554 -
0.2041 0.3727 -0.7034 -0.7083 -0.1012
0.0303 1.1300 -40.6138
47.2400 -40.4100 0.3561 -0.1568 -
0.1249 0.4857 -0.6572 -0.7552 -0.1171
0.0239 1.1331 -40.6139
47.2600 -40.6500 0.0335 -0.1701 -
0.0370 0.5813 -0.6064 -0.8000 -0.1329
0.0176 1.1362 -40.6140
47.2800 -40.7100 -0.4124 -0.0464
0.0455 0.6585 -0.5516 -0.8427 -0.1484
0.0112 1.1394 -40.6140

47.3000 -39.7400 0.3037 0.0945
0.1107 0.7166 -0.4935 -0.8830 -0.1638
0.0049 1.1425 -40.6141
47.3200 -39.7900 0.1736 0.1217
0.1478 0.7553 -0.4327 -0.9210 -0.1790
-0.0015 1.1456 -40.6142
47.3400 -40.3200 -0.2676 0.0681
0.1470 0.7744 -0.3700 -0.9567 -0.1940
-0.0078 1.1487 -40.6143
47.3600 -40.1900 -0.2093 0.0535
0.1055 0.7742 -0.3059 -0.9900 -0.2088
-0.0142 1.1519 -40.6144
47.3800 -39.8300 0.3393 0.0353
0.0379 0.7560 -0.2413 -1.0208 -0.2233
-0.0205 1.1550 -40.6144
47.4000 -40.1600 0.1222 -0.0474 -
0.0366 0.7221 -0.1767 -1.0491 -0.2377
-0.0268 1.1581 -40.6145
47.4200 -40.9500 -0.3734 -0.1125 -
0.0968 0.6747 -0.1129 -1.0749 -0.2517
-0.0331 1.1613 -40.6146
47.4400 -40.3100 0.1815 -0.0791 -
0.1271 0.6165 -0.0506 -1.0982 -0.2655
-0.0393 1.1644 -40.6146
47.4600 -40.2000 0.3489 -0.0128 -
0.1218 0.5495 0.0096 -1.1188 -0.2790
-0.0456 1.1675 -40.6147
47.4800 -40.6000 -0.3845 0.0502 -
0.0839 0.4762 0.0668 -1.1368 -0.2922
-0.0518 1.1707 -40.6148
47.5000 -40.4200 -0.0709 0.1165 -
0.0202 0.3986 0.1205 -1.1521 -0.3051
-0.0580 1.1738 -40.6148
47.5200 -39.8500 0.4578 0.0892
0.0577 0.3184 0.1700 -1.1648 -0.3177
-0.0642 1.1770 -40.6149
47.5400 -40.7800 -0.3132 -0.0881
0.1308 0.2371 0.2149 -1.1747 -0.3299
-0.0704 1.1801 -40.6149
47.5600 -40.4900 0.0833 -0.1978
0.1799 0.1569 0.2546 -1.1819 -0.3418
-0.0765 1.1833 -40.6150
47.5800 -40.8100 -0.2684 -0.0307
0.1872 0.0804 0.2885 -1.1864 -0.3533
-0.0826 1.1865 -40.6150
47.6000 -40.0900 0.1753 0.2404
0.1441 0.0104 0.3159 -1.1880 -0.3645
-0.0887 1.1896 -40.6151
47.6200 -40.1400 0.2597 0.2621
0.0556 -0.0501 0.3365 -1.1869 -0.3753
-0.0948 1.1928 -40.6151

47.6400 -41.2100 -0.3177 -0.0113 -
0.0598 -0.0982 0.3497 -1.1829 -0.3857
-0.1008 1.1960 -40.6152
47.6600 -41.2300 0.0596 -0.2742 -
0.1763 -0.1316 0.3552 -1.1762 -0.3956
-0.1068 1.1991 -40.6152
47.6800 -41.2700 0.0461 -0.2298 -
0.2676 -0.1486 0.3529 -1.1667 -0.4052
-0.1127 1.2023 -40.6153
47.7000 -41.7300 -0.6022 0.0889 -
0.3127 -0.1486 0.3429 -1.1546 -0.4144
-0.1186 1.2055 -40.6153
47.7200 -40.0900 0.7589 0.2581 -
0.3021 -0.1323 0.3257 -1.1398 -0.4232
-0.1245 1.2086 -40.6154
47.7400 -40.8600 0.2231 0.0055 -
0.2372 -0.1020 0.3017 -1.1225 -0.4317
-0.1304 1.2118 -40.6154
47.7600 -41.9500 -0.8009 -0.2829 -
0.1346 -0.0611 0.2716 -1.1027 -0.4397
-0.1361 1.2150 -40.6154
47.7800 -40.5400 0.5153 -0.1187 -
0.0154 -0.0136 0.2362 -1.0807 -0.4473
-0.1419 1.2182 -40.6155
47.8000 -40.7000 -0.2536 0.2926
0.1003 0.0362 0.1963 -1.0565 -0.4545
-0.1476 1.2213 -40.6155
47.8200 -39.6100 0.7159 0.2931
0.1968 0.0834 0.1526 -1.0303 -0.4614
-0.1533 1.2245 -40.6155
47.8400 -41.0000 -0.4193 -0.0751
0.2698 0.1232 0.1061 -1.0020 -0.4679
-0.1589 1.2277 -40.6155
47.8600 -41.4500 -0.6398 -0.3327
0.3221 0.1517 0.0576 -0.9720 -0.4740
-0.1644 1.2309 -40.6156
47.8800 -40.7100 0.0635 -0.2412
0.3552 0.1656 0.0079 -0.9402 -0.4798
-0.1700 1.2341 -40.6156
47.9000 -39.6600 0.7236 -0.0052
0.3614 0.1636 -0.0420 -0.9068 -0.4851
-0.1754 1.2373 -40.6156
47.9200 -39.9200 0.4113 0.1322
0.3340 0.1462 -0.0914 -0.8719 -0.4902
-0.1808 1.2405 -40.6156
47.9400 -41.3400 -0.8873 0.2068
0.2627 0.1152 -0.1396 -0.8356 -0.4949
-0.1862 1.2437 -40.6156
47.9600 -40.7100 -0.2274 0.3234
0.1392 0.0734 -0.1858 -0.7981 -0.4992
-0.1915 1.2469 -40.6157

47.9800 -40.2300 0.4685 0.3656 -
0.0236 0.0245 -0.2295 -0.7594 -0.5033
-0.1967 1.2501 -40.6157
48.0000 -40.6400 0.5132 0.1237 -
0.1930 -0.0282 -0.2701 -0.7197 -0.5069
-0.2019 1.2532 -40.6157
48.0200 -41.9900 -0.1894 -0.2910 -
0.3272 -0.0813 -0.3069 -0.6791 -0.5103
-0.2071 1.2564 -40.6157
48.0400 -42.6500 -0.6019 -0.5030 -
0.3826 -0.1311 -0.3395 -0.6377 -0.5133
-0.2121 1.2596 -40.6157
48.0600 -41.5900 0.2934 -0.2927 -
0.3330 -0.1748 -0.3673 -0.5956 -0.5161
-0.2171 1.2628 -40.6157
48.0800 -41.3100 0.0704 0.0765 -
0.1997 -0.2105 -0.3898 -0.5531 -0.5185
-0.2221 1.2660 -40.6157
48.1000 -40.6400 0.3563 0.1761 -
0.0273 -0.2372 -0.4068 -0.5101 -0.5206
-0.2270 1.2692 -40.6157
48.1200 -40.5500 0.5572 -0.0003
0.1298 -0.2543 -0.4178 -0.4669 -0.5224
-0.2318 1.2724 -40.6157
48.1400 -42.0800 -1.0700 -0.0667
0.2213 -0.2611 -0.4229 -0.4235 -0.5239
-0.2365 1.2757 -40.6157
48.1600 -39.8400 0.8240 0.1801
0.2223 -0.2572 -0.4219 -0.3800 -0.5252
-0.2412 1.2789 -40.6157
48.1800 -39.9800 0.7355 0.2970
0.1458 -0.2425 -0.4149 -0.3365 -0.5261
-0.2459 1.2821 -40.6157
48.2000 -40.9200 0.1029 0.0180
0.0229 -0.2178 -0.4023 -0.2931 -0.5268
-0.2504 1.2853 -40.6157
48.2200 -42.3700 -1.0068 -0.2669 -
0.1038 -0.1839 -0.3845 -0.2498 -0.5272
-0.2549 1.2885 -40.6157
48.2400 -40.4500 0.8798 -0.2534 -
0.1945 -0.1415 -0.3618 -0.2068 -0.5273
-0.2593 1.2917 -40.6156
48.2600 -41.5700 -0.5480 -0.0540 -
0.2327 -0.0915 -0.3349 -0.1640 -0.5272
-0.2637 1.2949 -40.6156
48.2800 -40.7800 -0.0787 0.1286 -
0.2220 -0.0356 -0.3043 -0.1215 -0.5268
-0.2679 1.2981 -40.6156
48.3000 -40.1000 0.3481 0.2096 -
0.1726 0.0243 -0.2707 -0.0794 -0.5262
-0.2722 1.3013 -40.6156

48.3200 -40.4600 -0.1516 0.1001 -
0.0973 0.0855 -0.2345 -0.0377 -0.5253
-0.2763 1.3045 -40.6156
48.3400 -40.1300 0.0765 -0.1147 -
0.0090 0.1448 -0.1965 0.0035 -0.5242
-0.2803 1.3077 -40.6155
48.3600 -40.3200 -0.1050 -0.2461
0.0780 0.1992 -0.1570 0.0441 -0.5229
-0.2843 1.3109 -40.6155
48.3800 -39.8900 0.0262 -0.1859
0.1483 0.2450 -0.1166 0.0842 -0.5213
-0.2882 1.3141 -40.6155
48.4000 -39.6900 -0.1378 0.0390
0.1899 0.2784 -0.0758 0.1236 -0.5194
-0.2921 1.3173 -40.6154
48.4200 -39.1200 0.1000 0.2530
0.1957 0.2961 -0.0351 0.1623 -0.5174
-0.2958 1.3206 -40.6154
48.4400 -39.0500 0.1782 0.2662
0.1608 0.2959 0.0051 0.2002 -0.5151
-0.2995 1.3238 -40.6154
48.4600 -39.3400 0.1209 0.0497
0.0927 0.2771 0.0444 0.2373 -0.5127
-0.3031 1.3270 -40.6153
48.4800 -40.0500 -0.4234 -0.1764
0.0122 0.2407 0.0826 0.2735 -0.5100
-0.3067 1.3302 -40.6153
48.5000 -39.7500 -0.0045 -0.1624 -
0.0570 0.1890 0.1193 0.3087 -0.5071
-0.3101 1.3334 -40.6153
48.5200 -39.7000 -0.0137 0.0136 -
0.0989 0.1249 0.1544 0.3428 -0.5040
-0.3135 1.3366 -40.6152
48.5400 -39.0500 0.3938 0.0671 -
0.1060 0.0519 0.1878 0.3757 -0.5007
-0.3168 1.3398 -40.6152
48.5600 -39.7100 -0.2027 -0.0146 -
0.0850 -0.0258 0.2194 0.4075 -0.4973
-0.3200 1.3430 -40.6151
48.5800 -39.9100 -0.3452 -0.0198 -
0.0498 -0.1037 0.2489 0.4379 -0.4936
-0.3231 1.3462 -40.6151
48.6000 -38.9900 0.5001 0.0694 -
0.0103 -0.1772 0.2764 0.4670 -0.4898
-0.3262 1.3494 -40.6150
48.6200 -39.8500 -0.3703 0.0251
0.0268 -0.2422 0.3017 0.4947 -0.4858
-0.3292 1.3526 -40.6150
48.6400 -39.2000 0.4485 -0.1693
0.0571 -0.2949 0.3248 0.5208 -0.4816
-0.3321 1.3558 -40.6149

48.6600 -40.1200 -0.5051 -0.1897
0.0729 -0.3321 0.3457 0.5453 -0.4772
-0.3349 1.3590 -40.6149
48.6800 -39.2300 0.1311 0.0355
0.0626 -0.3518 0.3644 0.5681 -0.4727
-0.3376 1.3623 -40.6148
48.7000 -38.6400 0.5237 0.2146
0.0180 -0.3532 0.3809 0.5893 -0.4680
-0.3402 1.3655 -40.6147
48.7200 -39.7800 -0.4963 0.1682 -
0.0595 -0.3370 0.3952 0.6087 -0.4632
-0.3428 1.3687 -40.6147
48.7400 -39.3300 0.0464 0.0802 -
0.1534 -0.3051 0.4072 0.6262 -0.4582
-0.3452 1.3719 -40.6146
48.7600 -39.4200 0.0717 0.0234 -
0.2361 -0.2601 0.4169 0.6419 -0.4531
-0.3476 1.3751 -40.6146
48.7800 -39.1700 0.3416 -0.1383 -
0.2730 -0.2055 0.4242 0.6557 -0.4479
-0.3499 1.3783 -40.6145
48.8000 -39.6900 -0.0535 -0.3222 -
0.2401 -0.1449 0.4291 0.6676 -0.4425
-0.3521 1.3815 -40.6144
48.8200 -40.0800 -0.6550 -0.2473 -
0.1336 -0.0814 0.4316 0.6776 -0.4370
-0.3542 1.3847 -40.6143
48.8400 -38.2800 0.5257 0.0869
0.0262 -0.0176 0.4315 0.6856 -0.4313
-0.3563 1.3879 -40.6143
48.8600 -37.9100 0.4849 0.2669
0.2034 0.0445 0.4288 0.6918 -0.4255
-0.3582 1.3911 -40.6142
48.8800 -38.7200 -0.4258 0.1515
0.3608 0.1029 0.4233 0.6960 -0.4197
-0.3601 1.3943 -40.6141
48.9000 -38.2100 -0.0519 -0.0136
0.4692 0.1555 0.4150 0.6984 -0.4137
-0.3618 1.3974 -40.6140
48.9200 -38.2600 -0.0535 -0.0121
0.5091 0.2007 0.4037 0.6990 -0.4076
-0.3635 1.4006 -40.6139
48.9400 -37.6700 0.3868 0.0658
0.4676 0.2375 0.3894 0.6978 -0.4014
-0.3651 1.4038 -40.6139
48.9600 -38.5900 -0.4282 0.1563
0.3364 0.2661 0.3719 0.6948 -0.3951
-0.3666 1.4070 -40.6138
48.9800 -38.2900 -0.0440 0.2643
0.1240 0.2879 0.3514 0.6902 -0.3887
-0.3680 1.4102 -40.6137

49.0000 -38.1900 0.3801 0.2109 -
0.1330 0.3044 0.3279 0.6839 -0.3823
-0.3693 1.4134 -40.6136
49.0200 -38.7900 0.3521 -0.1194 -
0.3861 0.3169 0.3013 0.6761 -0.3757
-0.3705 1.4166 -40.6135
49.0400 -40.1500 -0.4634 -0.4382 -
0.5792 0.3270 0.2719 0.6669 -0.3691
-0.3717 1.4198 -40.6134
49.0600 -40.3200 -0.6073 -0.4123 -
0.6652 0.3360 0.2397 0.6562 -0.3625
-0.3727 1.4229 -40.6133
49.0800 -38.8500 0.7198 -0.1209 -
0.6320 0.3441 0.2051 0.6443 -0.3558
-0.3736 1.4261 -40.6132
49.1000 -39.0400 0.1757 0.0880 -
0.4949 0.3501 0.1686 0.6311 -0.3490
-0.3745 1.4293 -40.6131
49.1200 -39.4000 -0.3740 0.1108 -
0.2809 0.3517 0.1307 0.6169 -0.3422
-0.3753 1.4325 -40.6130
49.1400 -39.2600 -0.4512 0.1120 -
0.0212 0.3463 0.0917 0.6016 -0.3354
-0.3759 1.4357 -40.6129
49.1600 -37.9800 0.5856 0.1284
0.2525 0.3306 0.0523 0.5854 -0.3286
-0.3765 1.4388 -40.6128
49.1800 -38.6800 -0.1988 0.0416
0.5071 0.3014 0.0130 0.5683 -0.3218
-0.3770 1.4420 -40.6127
49.2000 -38.4500 -0.0205 -0.0449
0.6945 0.2560 -0.0258 0.5505 -0.3149
-0.3773 1.4452 -40.6126
49.2200 -38.5400 -0.2757 0.1145
0.7666 0.1931 -0.0635 0.5320 -0.3081
-0.3776 1.4483 -40.6125
49.2400 -38.1800 -0.0141 0.4412
0.7028 0.1133 -0.0996 0.5129 -0.3013
-0.3778 1.4515 -40.6123
49.2600 -37.8800 0.4778 0.5784
0.5074 0.0197 -0.1337 0.4934 -0.2945
-0.3779 1.4547 -40.6122
49.2800 -38.9800 0.1243 0.2703
0.2092 -0.0823 -0.1653 0.4735 -0.2877
-0.3779 1.4578 -40.6121
49.3000 -40.3600 -0.2459 -0.2562 -
0.1220 -0.1867 -0.1940 0.4532 -0.2810
-0.3778 1.4610 -40.6120
49.3200 -40.9800 -0.1872 -0.5620 -
0.4056 -0.2875 -0.2193 0.4328 -0.2743
-0.3776 1.4641 -40.6119

49.3400 -41.2600 -0.2220 -0.4533 -
0.5740 -0.3786 -0.2407 0.4122 -0.2676
-0.3773 1.4673 -40.6117
49.3600 -40.6600 0.2608 -0.1535 -
0.6073 -0.4543 -0.2578 0.3916 -0.2611
-0.3769 1.4704 -40.6116
49.3800 -40.5000 0.2292 0.0127 -
0.5264 -0.5093 -0.2701 0.3711 -0.2546
-0.3764 1.4735 -40.6115
49.4000 -40.6300 -0.1824 0.0517 -
0.3832 -0.5397 -0.2774 0.3507 -0.2481
-0.3758 1.4767 -40.6113
49.4200 -40.8300 -0.4209 0.1342 -
0.2424 -0.5425 -0.2794 0.3304 -0.2418
-0.3751 1.4798 -40.6112
49.4400 -39.4700 0.6875 0.2402 -
0.1533 -0.5151 -0.2762 0.3104 -0.2356
-0.3743 1.4830 -40.6111
49.4600 -40.2900 -0.3334 0.2564 -
0.1364 -0.4573 -0.2679 0.2907 -0.2294
-0.3734 1.4861 -40.6109
49.4800 -40.5900 -0.5252 0.1659 -
0.1759 -0.3726 -0.2552 0.2713 -0.2234
-0.3725 1.4892 -40.6108
49.5000 -39.3800 0.7188 -0.0066 -
0.2274 -0.2675 -0.2387 0.2523 -0.2175
-0.3714 1.4923 -40.6107
49.5200 -40.9100 -0.4960 -0.2520 -
0.2384 -0.1502 -0.2190 0.2337 -0.2118
-0.3702 1.4954 -40.6105
49.5400 -40.1000 0.0741 -0.3375 -
0.1745 -0.0285 -0.1967 0.2155 -0.2061
-0.3689 1.4985 -40.6104
49.5600 -39.8200 -0.0955 -0.1232 -
0.0341 0.0901 -0.1726 0.1979 -0.2006
-0.3675 1.5017 -40.6102
49.5800 -39.1400 0.0296 0.1784
0.1468 0.1988 -0.1471 0.1807 -0.1953
-0.3660 1.5048 -40.6101
49.6000 -38.6200 0.2126 0.2908
0.3149 0.2914 -0.1210 0.1640 -0.1902
-0.3645 1.5079 -40.6099
49.6200 -38.7500 -0.0535 0.1745
0.4237 0.3617 -0.0948 0.1480 -0.1852
-0.3628 1.5109 -40.6098
49.6400 -38.7500 0.0060 0.0153
0.4516 0.4052 -0.0693 0.1326 -0.1803
-0.3610 1.5140 -40.6096
49.6600 -39.0200 -0.2083 -0.0517
0.4036 0.4203 -0.0449 0.1177 -0.1757
-0.3591 1.5171 -40.6095

49.6800 -38.6200 0.2812 -0.0396
0.3024 0.4089 -0.0221 0.1036 -0.1713
-0.3571 1.5202 -40.6093
49.7000 -39.0400 -0.0384 -0.0008
0.1796 0.3746 -0.0012 0.0902 -0.1670
-0.3550 1.5233 -40.6091
49.7200 -39.2900 -0.1922 0.0539
0.0635 0.3220 0.0176 0.0775 -0.1630
-0.3529 1.5263 -40.6090
49.7400 -39.1000 0.0994 0.0653 -
0.0235 0.2561 0.0343 0.0655 -0.1591
-0.3506 1.5294 -40.6088
49.7600 -39.2100 0.2736 -0.0742 -
0.0657 0.1821 0.0487 0.0543 -0.1555
-0.3482 1.5325 -40.6086
49.7800 -40.0400 -0.2765 -0.2523 -
0.0680 0.1052 0.0606 0.0439 -0.1521
-0.3457 1.5355 -40.6085
49.8000 -39.8400 -0.2008 -0.1711 -
0.0480 0.0305 0.0699 0.0344 -0.1489
-0.3432 1.5385 -40.6083
49.8200 -39.2000 0.1226 0.1414 -
0.0340 -0.0374 0.0766 0.0256 -0.1459
-0.3405 1.5416 -40.6081
49.8400 -39.1400 0.1871 0.3408 -
0.0511 -0.0944 0.0806 0.0177 -0.1431
-0.3377 1.5446 -40.6079
49.8600 -39.3300 0.2161 0.1877 -
0.1095 -0.1382 0.0819 0.0106 -0.1406
-0.3349 1.5476 -40.6078
49.8800 -40.0800 -0.1501 -0.1463 -
0.1887 -0.1675 0.0805 0.0043 -0.1383
-0.3319 1.5507 -40.6076
49.9000 -40.6000 -0.4582 -0.2615 -
0.2533 -0.1822 0.0767 -0.0011 -0.1361
-0.3289 1.5537 -40.6074
49.9200 -39.4200 0.6098 -0.1085 -
0.2714 -0.1831 0.0707 -0.0058 -0.1342
-0.3257 1.5567 -40.6072
49.9400 -40.3100 -0.5135 0.0302 -
0.2286 -0.1721 0.0627 -0.0098 -0.1325
-0.3225 1.5597 -40.6070
49.9600 -39.4000 0.3683 0.0729 -
0.1340 -0.1522 0.0531 -0.0131 -0.1310
-0.3192 1.5627 -40.6068
49.9800 -39.9400 -0.2934 0.0228 -
0.0088 -0.1260 0.0423 -0.0158 -0.1297
-0.3158 1.5657 -40.6067
50.0000 -39.2400 0.3525 -0.1009
0.1209 -0.0964 0.0308 -0.0179 -0.1286
-0.3123 1.5686 -40.6065

50.0200 -39.9800 -0.5063 -0.1064
0.2308 -0.0664 0.0190 -0.0195 -0.1276
-0.3088 1.5716 -40.6063
50.0400 -39.0000 0.2614 0.0505
0.3001 -0.0385 0.0074 -0.0207 -0.1268
-0.3051 1.5746 -40.6061
50.0600 -38.4900 0.5853 0.1708
0.3102 -0.0147 -0.0037 -0.0215 -0.1262
-0.3014 1.5775 -40.6059
50.0800 -39.3800 -0.2201 0.1367
0.2516 0.0041 -0.0139 -0.0219 -0.1257
-0.2976 1.5805 -40.6057
50.1000 -39.9500 -0.7509 0.0853
0.1357 0.0178 -0.0228 -0.0221 -0.1254
-0.2937 1.5834 -40.6055
50.1200 -38.5800 0.8094 0.0411 -
0.0128 0.0269 -0.0301 -0.0221 -0.1252
-0.2897 1.5863 -40.6053
50.1400 -40.2200 -0.5512 -0.1403 -
0.1599 0.0321 -0.0356 -0.0220 -0.1251
-0.2857 1.5893 -40.6051
50.1600 -39.9800 0.0319 -0.2535 -
0.2710 0.0339 -0.0390 -0.0219 -0.1252
-0.2816 1.5922 -40.6048
50.1800 -40.0000 -0.1616 -0.0950 -
0.3213 0.0327 -0.0403 -0.0217 -0.1254
-0.2774 1.5951 -40.6046
50.2000 -39.5500 0.1390 0.1352 -
0.3042 0.0288 -0.0393 -0.0215 -0.1256
-0.2732 1.5980 -40.6044
50.2200 -39.3300 0.0817 0.1885 -
0.2245 0.0226 -0.0361 -0.0215 -0.1259
-0.2689 1.6009 -40.6042
50.2400 -39.5100 -0.0940 0.0531 -
0.0980 0.0143 -0.0308 -0.0216 -0.1263
-0.2645 1.6037 -40.6040
50.2600 -39.5500 -0.0696 -0.1140
0.0493 0.0041 -0.0235 -0.0218 -0.1268
-0.2601 1.6066 -40.6038
50.2800 -39.3600 0.0724 -0.1811
0.1882 -0.0078 -0.0144 -0.0223 -0.1273
-0.2556 1.6095 -40.6036
50.3000 -39.1100 0.2022 -0.1096
0.2889 -0.0211 -0.0038 -0.0230 -0.1279
-0.2511 1.6123 -40.6033
50.3200 -39.3700 -0.3847 0.0991
0.3289 -0.0353 0.0080 -0.0240 -0.1285
-0.2465 1.6152 -40.6031
50.3400 -38.9100 -0.0481 0.3226
0.2961 -0.0494 0.0207 -0.0252 -0.1291
-0.2418 1.6180 -40.6029

50.3600	-38.4400	0.4607	0.3355	
0.1941	-0.0623	0.0339	-0.0268	-0.1297
	-0.2371	1.6208	-40.6027	
50.3800	-39.3800	0.0522	0.0278	
0.0491	-0.0721	0.0473	-0.0287	-0.1303
	-0.2323	1.6236	-40.6024	
50.4000	-40.3100	-0.5384	-0.3058	-
0.1008	-0.0769	0.0606	-0.0310	-0.1309
	-0.2275	1.6264	-40.6022	
50.4200	-39.6300	0.4079	-0.3389	-
0.2182	-0.0748	0.0733	-0.0336	-0.1315
	-0.2227	1.6292	-40.6020	
50.4400	-39.9600	-0.2486	-0.0859	-
0.2778	-0.0644	0.0853	-0.0367	-0.1320
	-0.2178	1.6320	-40.6017	
50.4600	-39.3900	0.0103	0.1711	-
0.2756	-0.0454	0.0962	-0.0401	-0.1325
	-0.2128	1.6348	-40.6015	
50.4800	-38.9000	0.3903	0.1926	-
0.2163	-0.0186	0.1058	-0.0439	-0.1329
	-0.2079	1.6376	-40.6012	
50.5000	-39.4300	-0.1502	0.0057	-
0.1165	0.0140	0.1140	-0.0481	-0.1333
	-0.2029	1.6403	-40.6010	
50.5200	-39.9400	-0.4392	-0.1206	-
0.0032	0.0498	0.1204	-0.0527	-0.1336
	-0.1978	1.6431	-40.6008	
50.5400	-38.6900	0.4434	-0.0699	
0.0957	0.0859	0.1247	-0.0578	-0.1338
	-0.1927	1.6458	-40.6005	
50.5600	-38.6000	0.2943	0.0515	
0.1596	0.1189	0.1267	-0.0632	-0.1340
	-0.1876	1.6485	-40.6003	
50.5800	-39.3500	-0.6784	0.1541	
0.1742	0.1453	0.1262	-0.0689	-0.1340
	-0.1825	1.6512	-40.6000	
50.6000	-38.0800	0.7592	0.1145	
0.1445	0.1614	0.1228	-0.0750	-0.1339
	-0.1773	1.6539	-40.5998	
50.6200	-39.7300	-0.6284	-0.1378	
0.0986	0.1644	0.1167	-0.0815	-0.1338
	-0.1721	1.6566	-40.5995	
50.6400	-38.8400	0.3651	-0.2025	
0.0701	0.1523	0.1079	-0.0881	-0.1335
	-0.1669	1.6593	-40.5993	
50.6600	-39.3000	-0.4665	0.1104	
0.0762	0.1252	0.0967	-0.0951	-0.1331
	-0.1617	1.6619	-40.5990	
50.6800	-38.2100	0.6251	0.2364	
0.1029	0.0851	0.0833	-0.1022	-0.1325
	-0.1564	1.6646	-40.5987	

50.7000 -39.6200 -0.4536 -0.0536
0.1177 0.0365 0.0684 -0.1096 -0.1318
-0.1511 1.6672 -40.5985
50.7200 -39.4800 0.0167 -0.2183
0.0867 -0.0147 0.0522 -0.1170 -0.1310
-0.1458 1.6699 -40.5982
50.7400 -39.3600 -0.0342 -0.0040 -
0.0062 -0.0630 0.0354 -0.1246 -0.1300
-0.1405 1.6725 -40.5979
50.7600 -38.9300 0.3019 0.2885 -
0.1359 -0.1042 0.0184 -0.1322 -0.1289
-0.1352 1.6751 -40.5977
50.7800 -39.7600 -0.3337 0.2951 -
0.2588 -0.1355 0.0018 -0.1398 -0.1277
-0.1299 1.6777 -40.5974
50.8000 -39.3600 0.6323 -0.1625 -
0.3311 -0.1549 -0.0141 -0.1475 -0.1262
-0.1246 1.6803 -40.5971
50.8200 -41.0900 -0.7082 -0.6155 -
0.3140 -0.1615 -0.0289 -0.1550 -0.1246
-0.1192 1.6828 -40.5969
50.8400 -39.8100 0.3188 -0.4159 -
0.1885 -0.1562 -0.0420 -0.1625 -0.1228
-0.1139 1.6854 -40.5966
50.8600 -39.4500 -0.1800 0.1931
0.0005 -0.1423 -0.0532 -0.1699 -0.1209
-0.1085 1.6879 -40.5963
50.8800 -38.3600 0.5986 0.5500
0.1872 -0.1237 -0.0621 -0.1771 -0.1187
-0.1032 1.6905 -40.5960
50.9000 -38.9200 -0.0728 0.3597
0.3118 -0.1043 -0.0687 -0.1841 -0.1164
-0.0978 1.6930 -40.5957
50.9200 -38.7700 0.4944 -0.1707
0.3452 -0.0875 -0.0727 -0.1909 -0.1139
-0.0925 1.6955 -40.5955
50.9400 -40.4500 -0.9399 -0.3678
0.2813 -0.0756 -0.0741 -0.1975 -0.1112
-0.0871 1.6980 -40.5952
50.9600 -39.6700 -0.2741 -0.0262
0.1365 -0.0688 -0.0731 -0.2038 -0.1083
-0.0818 1.7004 -40.5949
50.9800 -38.1500 0.9884 0.3103 -
0.0341 -0.0662 -0.0697 -0.2098 -0.1051
-0.0765 1.7029 -40.5946
51.0000 -40.3000 -0.8306 0.0992 -
0.1713 -0.0661 -0.0642 -0.2155 -0.1018
-0.0712 1.7053 -40.5943
51.0200 -39.6000 0.2363 -0.2704 -
0.2408 -0.0670 -0.0569 -0.2208 -0.0982
-0.0658 1.7078 -40.5940

51.0400 -40.0000 -0.1828 -0.2256 -
0.2318 -0.0667 -0.0482 -0.2259 -0.0945
-0.0606 1.7102 -40.5937
51.0600 -39.5400 -0.2176 0.1053 -
0.1646 -0.0631 -0.0384 -0.2306 -0.0905
-0.0553 1.7126 -40.5934
51.0800 -38.7300 0.4558 0.2819 -
0.0758 -0.0545 -0.0280 -0.2349 -0.0863
-0.0500 1.7150 -40.5931
51.1000 -39.1700 -0.0127 0.1288 -
0.0014 -0.0397 -0.0173 -0.2389 -0.0819
-0.0447 1.7174 -40.5928
51.1200 -39.6600 -0.4477 -0.0886
0.0412 -0.0179 -0.0067 -0.2425 -0.0772
-0.0395 1.7197 -40.5925
51.1400 -39.3100 -0.0042 -0.1193
0.0491 0.0108 0.0033 -0.2457 -0.0723
-0.0343 1.7221 -40.5922
51.1600 -38.8200 0.4251 -0.0390
0.0330 0.0453 0.0125 -0.2486 -0.0672
-0.0291 1.7244 -40.5919
51.1800 -39.2500 -0.2848 -0.0002
0.0121 0.0838 0.0207 -0.2511 -0.0618
-0.0239 1.7267 -40.5916
51.2000 -39.0200 0.0234 0.0121
0.0050 0.1234 0.0276 -0.2532 -0.0562
-0.0187 1.7290 -40.5913
51.2200 -39.1700 -0.2648 0.0560
0.0207 0.1612 0.0330 -0.2550 -0.0504
-0.0136 1.7313 -40.5910
51.2400 -38.4000 0.5163 -0.0030
0.0566 0.1942 0.0369 -0.2564 -0.0444
-0.0085 1.7336 -40.5907
51.2600 -38.7000 0.1598 -0.1912
0.1006 0.2194 0.0390 -0.2574 -0.0381
-0.0034 1.7358 -40.5903
51.2800 -39.4900 -0.5744 -0.2525
0.1421 0.2344 0.0393 -0.2581 -0.0316
0.0017 1.7381 -40.5900
51.3000 -38.2400 0.3463 0.0188
0.1678 0.2370 0.0377 -0.2584 -0.0248
0.0067 1.7403 -40.5897
51.3200 -38.3100 0.0350 0.3317
0.1631 0.2259 0.0342 -0.2583 -0.0178
0.0117 1.7425 -40.5894
51.3400 -38.2500 0.1869 0.3315
0.1225 0.2008 0.0290 -0.2579 -0.0106
0.0167 1.7447 -40.5890
51.3600 -38.9000 -0.1376 0.0573
0.0573 0.1624 0.0223 -0.2572 -0.0032
0.0217 1.7469 -40.5887

51.3800 -39.1600 0.1715 -0.2608 -
0.0042 0.1125 0.0145 -0.2561 0.0045
0.0267 1.7490 -40.5884
51.4000 -39.5300 -0.1452 -0.3770 -
0.0348 0.0535 0.0057 -0.2547 0.0124
0.0316 1.7512 -40.5881
51.4200 -39.4500 -0.1989 -0.2056 -
0.0209 -0.0117 -0.0036 -0.2530 0.0205
0.0365 1.7533 -40.5877
51.4400 -38.7900 0.1943 0.1013
0.0223 -0.0787 -0.0131 -0.2509 0.0287
0.0413 1.7554 -40.5874
51.4600 -38.6200 0.2012 0.2351
0.0678 -0.1426 -0.0225 -0.2485 0.0372
0.0461 1.7575 -40.5871
51.4800 -38.8400 0.1379 0.1273
0.0829 -0.1982 -0.0314 -0.2459 0.0459
0.0509 1.7596 -40.5867
51.5000 -39.8500 -0.6483 0.0584
0.0409 -0.2410 -0.0397 -0.2429 0.0548
0.0557 1.7616 -40.5864
51.5200 -38.5500 0.6038 0.1435 -
0.0616 -0.2679 -0.0471 -0.2397 0.0639
0.0605 1.7637 -40.5860
51.5400 -39.5400 -0.2071 0.0725 -
0.1942 -0.2778 -0.0531 -0.2362 0.0732
0.0652 1.7657 -40.5857
51.5600 -39.5100 0.1389 -0.2038 -
0.3142 -0.2702 -0.0578 -0.2324 0.0826
0.0699 1.7677 -40.5853
51.5800 -40.3300 -0.4829 -0.2691 -
0.3820 -0.2452 -0.0609 -0.2284 0.0923
0.0745 1.7697 -40.5850
51.6000 -39.2100 0.2636 -0.0444 -
0.3722 -0.2039 -0.0625 -0.2242 0.1021
0.0791 1.7717 -40.5846
51.6200 -39.1000 0.1359 0.0818 -
0.2826 -0.1496 -0.0625 -0.2198 0.1120
0.0837 1.7736 -40.5843
51.6400 -39.0200 0.1233 -0.0437 -
0.1251 -0.0868 -0.0612 -0.2152 0.1222
0.0883 1.7756 -40.5839
51.6600 -39.2900 -0.2926 -0.1831
0.0698 -0.0204 -0.0585 -0.2104 0.1324
0.0928 1.7775 -40.5836
51.6800 -38.5300 0.1329 -0.1393
0.2616 0.0445 -0.0546 -0.2055 0.1429
0.0973 1.7794 -40.5832
51.7000 -38.1500 0.0832 0.0838
0.4106 0.1028 -0.0497 -0.2005 0.1534
0.1017 1.7813 -40.5829

51.7200 -37.9500 -0.1442 0.3437
0.4829 0.1500 -0.0437 -0.1955 0.1641
0.1062 1.7831 -40.5825
51.7400 -37.6000 0.0045 0.4675
0.4529 0.1825 -0.0369 -0.1903 0.1750
0.1106 1.7850 -40.5821
51.7600 -37.5800 0.3414 0.3423
0.3180 0.1990 -0.0293 -0.1851 0.1859
0.1149 1.7868 -40.5818
51.7800 -38.7900 -0.4314 0.0046
0.1100 0.2006 -0.0211 -0.1799 0.1970
0.1192 1.7886 -40.5814
51.8000 -38.5200 0.3524 -0.2847 -
0.1156 0.1893 -0.0125 -0.1748 0.2082
0.1235 1.7904 -40.5810
51.8200 -39.3300 -0.2821 -0.3428 -
0.2961 0.1673 -0.0035 -0.1696 0.2195
0.1278 1.7922 -40.5807
51.8400 -39.0600 -0.0302 -0.2433 -
0.3832 0.1370 0.0056 -0.1646 0.2308
0.1320 1.7939 -40.5803
51.8600 -38.5800 0.3427 -0.1541 -
0.3560 0.1006 0.0146 -0.1596 0.2423
0.1361 1.7956 -40.5799
51.8800 -39.1600 -0.3799 -0.1109 -
0.2285 0.0610 0.0235 -0.1547 0.2539
0.1403 1.7973 -40.5795
51.9000 -38.4300 0.0972 0.0044 -
0.0514 0.0208 0.0321 -0.1500 0.2656
0.1444 1.7990 -40.5792
51.9200 -37.9800 0.1532 0.2053
0.1169 -0.0173 0.0403 -0.1455 0.2773
0.1485 1.8007 -40.5788
51.9400 -37.9900 -0.0952 0.3410
0.2276 -0.0506 0.0479 -0.1412 0.2891
0.1525 1.8024 -40.5784
51.9600 -37.8600 0.0886 0.2897
0.2515 -0.0767 0.0548 -0.1370 0.3009
0.1565 1.8040 -40.5780
51.9800 -38.2100 0.0249 0.0783
0.1932 -0.0942 0.0609 -0.1332 0.3128
0.1604 1.8056 -40.5776
52.0000 -38.6700 -0.2071 -0.0948
0.0808 -0.1030 0.0662 -0.1295 0.3248
0.1643 1.8072 -40.5772
52.0200 -38.5200 -0.0224 -0.1362 -
0.0472 -0.1035 0.0705 -0.1262 0.3367
0.1682 1.8088 -40.5769
52.0400 -38.3400 0.2570 -0.1052 -
0.1518 -0.0969 0.0738 -0.1231 0.3488
0.1720 1.8103 -40.5765

52.0600 -38.7800 -0.1299 -0.1225 -
0.2000 -0.0843 0.0760 -0.1204 0.3608
0.1758 1.8118 -40.5761
52.0800 -38.4300 0.0833 -0.1856 -
0.1793 -0.0673 0.0772 -0.1179 0.3728
0.1796 1.8133 -40.5757
52.1000 -38.8500 -0.3354 -0.1631 -
0.1020 -0.0475 0.0773 -0.1158 0.3848
0.1833 1.8148 -40.5753
52.1200 -37.9800 0.1712 0.0267
0.0002 -0.0261 0.0763 -0.1139 0.3968
0.1869 1.8163 -40.5749
52.1400 -38.1900 -0.2291 0.2448
0.0916 -0.0046 0.0741 -0.1124 0.4088
0.1905 1.8177 -40.5745
52.1600 -37.3100 0.4030 0.3052
0.1400 0.0157 0.0708 -0.1111 0.4208
0.1941 1.8192 -40.5741
52.1800 -38.2100 -0.3442 0.1656
0.1305 0.0338 0.0662 -0.1101 0.4327
0.1977 1.8206 -40.5737
52.2000 -37.7800 0.3101 -0.0873
0.0751 0.0491 0.0606 -0.1094 0.4445
0.2011 1.8219 -40.5733
52.2200 -38.4800 -0.1425 -0.2800
0.0008 0.0616 0.0539 -0.1090 0.4563
0.2046 1.8233 -40.5728
52.2400 -38.5800 -0.2974 -0.2431 -
0.0615 0.0713 0.0461 -0.1087 0.4680
0.2080 1.8246 -40.5724
52.2600 -38.0500 0.1608 0.0057 -
0.0885 0.0789 0.0375 -0.1087 0.4796
0.2114 1.8259 -40.5720
52.2800 -37.4900 0.3625 0.1990 -
0.0769 0.0850 0.0279 -0.1088 0.4911
0.2147 1.8272 -40.5716
52.3000 -38.2400 -0.2940 0.1443 -
0.0348 0.0900 0.0177 -0.1091 0.5025
0.2179 1.8285 -40.5712
52.3200 -37.8100 0.1741 -0.0262
0.0193 0.0944 0.0068 -0.1095 0.5138
0.2212 1.8298 -40.5708
52.3400 -38.2000 -0.1965 -0.1081
0.0625 0.0980 -0.0047 -0.1100 0.5249
0.2243 1.8310 -40.5704
52.3600 -37.8700 0.0757 -0.0414
0.0770 0.1008 -0.0165 -0.1105 0.5358
0.2275 1.8322 -40.5699
52.3800 -38.0600 -0.2618 0.1374
0.0563 0.1027 -0.0286 -0.1110 0.5466
0.2305 1.8334 -40.5695

52.4000 -37.2700 0.4297 0.2315
0.0047 0.1033 -0.0407 -0.1115 0.5572
0.2336 1.8345 -40.5691
52.4200 -37.9700 -0.0363 0.0731 -
0.0637 0.1018 -0.0527 -0.1118 0.5676
0.2365 1.8357 -40.5687
52.4400 -38.4900 -0.2300 -0.1421 -
0.1237 0.0974 -0.0644 -0.1120 0.5779
0.2395 1.8368 -40.5682
52.4600 -38.5800 -0.2475 -0.1603 -
0.1519 0.0892 -0.0755 -0.1121 0.5878
0.2424 1.8379 -40.5678
52.4800 -37.8900 0.3809 -0.0578 -
0.1349 0.0768 -0.0860 -0.1119 0.5976
0.2452 1.8389 -40.5674
52.5000 -37.8000 0.2575 -0.0361 -
0.0720 0.0597 -0.0956 -0.1114 0.6071
0.2480 1.8400 -40.5669
52.5200 -38.4800 -0.3337 -0.0663
0.0222 0.0377 -0.1041 -0.1106 0.6163
0.2507 1.8410 -40.5665
52.5400 -38.3100 -0.4421 0.0122
0.1256 0.0106 -0.1112 -0.1094 0.6253
0.2534 1.8420 -40.5660
52.5600 -37.2900 0.3793 0.1830
0.2117 -0.0216 -0.1169 -0.1079 0.6340
0.2560 1.8429 -40.5656
52.5800 -37.4600 0.1730 0.1992
0.2536 -0.0581 -0.1209 -0.1059 0.6424
0.2586 1.8439 -40.5652
52.6000 -37.9200 -0.0855 0.0298
0.2343 -0.0974 -0.1231 -0.1034 0.6505
0.2611 1.8448 -40.5647
52.6200 -37.9900 0.0809 -0.1241
0.1562 -0.1372 -0.1234 -0.1004 0.6582
0.2636 1.8457 -40.5643
52.6400 -38.6400 -0.3833 -0.0956
0.0379 -0.1743 -0.1220 -0.0968 0.6657
0.2660 1.8466 -40.5638
52.6600 -38.0100 0.2539 0.0564 -
0.0885 -0.2053 -0.1188 -0.0926 0.6728
0.2684 1.8474 -40.5634
52.6800 -38.0100 0.3028 0.0969 -
0.1908 -0.2267 -0.1140 -0.0878 0.6795
0.2707 1.8482 -40.5629
52.7000 -38.9400 -0.4980 -0.0794 -
0.2482 -0.2357 -0.1076 -0.0824 0.6859
0.2730 1.8490 -40.5625
52.7200 -38.0300 0.6572 -0.3291 -
0.2556 -0.2301 -0.1000 -0.0763 0.6918
0.2752 1.8498 -40.5620

52.7400 -39.1100 -0.5579 -0.2566 -
0.2234 -0.2095 -0.0912 -0.0694 0.6975
0.2773 1.8506 -40.5616
52.7600 -38.5600 -0.4800 0.1441 -
0.1730 -0.1746 -0.0817 -0.0618 0.7027
0.2794 1.8513 -40.5611
52.7800 -36.8500 0.8976 0.4601 -
0.1215 -0.1274 -0.0718 -0.0535 0.7075
0.2815 1.8520 -40.5606
52.8000 -38.2600 -0.5569 0.2671 -
0.0787 -0.0706 -0.0619 -0.0443 0.7119
0.2834 1.8526 -40.5602
52.8200 -38.3000 -0.3435 -0.1618 -
0.0379 -0.0075 -0.0523 -0.0344 0.7159
0.2854 1.8533 -40.5597
52.8400 -37.8900 0.1693 -0.3896
0.0121 0.0576 -0.0433 -0.0237 0.7195
0.2872 1.8539 -40.5592
52.8600 -37.7600 0.0069 -0.2735
0.0763 0.1201 -0.0353 -0.0122 0.7226
0.2890 1.8545 -40.5588
52.8800 -37.6000 -0.1709 0.0506
0.1495 0.1760 -0.0287 0.0002 0.7253
0.2908 1.8551 -40.5583
52.9000 -36.7700 0.1808 0.3340
0.2155 0.2213 -0.0237 0.0133 0.7276
0.2925 1.8556 -40.5578
52.9200 -36.6600 0.1063 0.3626
0.2558 0.2533 -0.0207 0.0273 0.7295
0.2941 1.8561 -40.5574
52.9400 -37.0500 0.0173 0.1277
0.2577 0.2701 -0.0199 0.0420 0.7309
0.2957 1.8566 -40.5569
52.9600 -37.5500 -0.2501 -0.1112
0.2200 0.2717 -0.0215 0.0575 0.7318
0.2972 1.8571 -40.5564
52.9800 -37.3900 -0.0301 -0.1428
0.1500 0.2590 -0.0255 0.0737 0.7323
0.2987 1.8575 -40.5559
53.0000 -36.9900 0.3403 -0.0273
0.0632 0.2332 -0.0318 0.0905 0.7324
0.3001 1.8579 -40.5554
53.0200 -37.8300 -0.3428 0.0417 -
0.0220 0.1963 -0.0402 0.1079 0.7320
0.3014 1.8583 -40.5550
53.0400 -37.2900 0.2217 0.0092 -
0.0922 0.1504 -0.0505 0.1259 0.7311
0.3027 1.8586 -40.5545
53.0600 -37.7700 -0.0448 -0.0481 -
0.1378 0.0980 -0.0622 0.1444 0.7297
0.3039 1.8590 -40.5540

53.0800	-37.9100	-0.1165	-0.0543	-
0.1512	0.0416	-0.0750	0.1632	0.7279
	0.3050	1.8593	-40.5535	
53.1000	-37.7800	-0.0414	0.0228	-
0.1309	-0.0163	-0.0884	0.1824	0.7256
	0.3061	1.8595	-40.5530	
53.1200	-37.5200	0.1489	0.0699	-
0.0836	-0.0729	-0.1019	0.2017	0.7229
	0.3072	1.8598	-40.5525	
53.1400	-37.5200	0.2440	-0.0413	-
0.0210	-0.1256	-0.1151	0.2212	0.7196
	0.3082	1.8600	-40.5520	
53.1600	-38.2600	-0.4450	-0.1644	
0.0395	-0.1718	-0.1277	0.2407	0.7159
	0.3091	1.8602	-40.5515	
53.1800	-37.7000	0.0844	-0.0847	
0.0790	-0.2096	-0.1392	0.2600	0.7117
	0.3099	1.8603	-40.5510	
53.2000	-37.5800	0.0983	0.0975	
0.0882	-0.2371	-0.1492	0.2792	0.7070
	0.3107	1.8605	-40.5505	
53.2200	-37.5500	0.0321	0.1624	
0.0639	-0.2530	-0.1575	0.2982	0.7018
	0.3115	1.8605	-40.5500	
53.2400	-37.5300	0.1296	0.0632	
0.0111	-0.2564	-0.1637	0.3167	0.6961
	0.3122	1.8606	-40.5495	
53.2600	-38.1500	-0.2443	-0.0701	-
0.0555	-0.2469	-0.1676	0.3348	0.6899
	0.3128	1.8607	-40.5490	
53.2800	-38.0500	-0.0462	-0.0923	-
0.1186	-0.2242	-0.1690	0.3524	0.6831
	0.3134	1.8607	-40.5485	
53.3000	-37.7500	0.1759	-0.0402	-
0.1590	-0.1887	-0.1678	0.3694	0.6759
	0.3139	1.8607	-40.5480	
53.3200	-37.8200	0.1108	-0.0448	-
0.1615	-0.1415	-0.1638	0.3857	0.6682
	0.3144	1.8606	-40.5475	
53.3400	-38.0600	-0.4094	-0.0445	-
0.1251	-0.0846	-0.1570	0.4012	0.6600
	0.3148	1.8605	-40.5470	
53.3600	-37.4200	0.1444	0.0262	-
0.0619	-0.0212	-0.1474	0.4159	0.6513
	0.3152	1.8604	-40.5465	
53.3800	-36.8700	0.5113	0.0391	
0.0084	0.0452	-0.1349	0.4297	0.6420
	0.3155	1.8603	-40.5460	
53.4000	-37.2800	0.0359	-0.0369	
0.0643	0.1105	-0.1195	0.4426	0.6323
	0.3158	1.8601	-40.5455	

53.4200	-37.6900	-0.7002	-0.0315	
0.0949	0.1704	-0.1013	0.4545	0.6220
	0.3160	1.8599	-40.5449	
53.4400	-36.3100	0.6981	0.1041	
0.0985	0.2204	-0.0802	0.4654	0.6112
	0.3162	1.8597	-40.5444	
53.4600	-37.2200	-0.3046	0.1177	
0.0815	0.2567	-0.0562	0.4752	0.6000
	0.3163	1.8595	-40.5439	
53.4800	-37.3200	-0.2452	-0.0504	
0.0573	0.2764	-0.0292	0.4839	0.5882
	0.3164	1.8592	-40.5434	
53.5000	-36.8000	0.3550	-0.1693	
0.0394	0.2775	0.0006	0.4914	0.5759
	0.3164	1.8589	-40.5429	
53.5200	-37.5700	-0.5467	-0.0369	
0.0392	0.2600	0.0332	0.4978	0.5631
	0.3164	1.8585	-40.5423	
53.5400	-36.2900	0.5217	0.1680	
0.0568	0.2249	0.0683	0.5029	0.5499
	0.3164	1.8582	-40.5418	
53.5600	-37.1800	-0.2882	0.1101	
0.0845	0.1745	0.1056	0.5067	0.5362
	0.3163	1.8578	-40.5413	
53.5800	-36.9000	0.1186	-0.0916	
0.1073	0.1120	0.1449	0.5091	0.5219
	0.3161	1.8573	-40.5407	
53.6000	-37.2700	-0.2214	-0.1438	
0.1092	0.0417	0.1855	0.5102	0.5073
	0.3160	1.8569	-40.5402	
53.6200	-36.8600	0.2551	-0.0159	
0.0807	-0.0318	0.2271	0.5098	0.4921
	0.3157	1.8564	-40.5397	
53.6400	-37.0100	0.0670	0.1676	
0.0163	-0.1033	0.2691	0.5080	0.4766
	0.3155	1.8559	-40.5391	
53.6600	-37.5300	-0.4324	0.2377	-
0.0807	-0.1684	0.3112	0.5045	0.4606
	0.3152	1.8553	-40.5386	
53.6800	-36.8500	0.5763	0.1132	-
0.1933	-0.2233	0.3526	0.4996	0.4441
	0.3149	1.8547	-40.5381	
53.7000	-38.1700	-0.4814	-0.1536	-
0.2879	-0.2650	0.3931	0.4930	0.4273
	0.3145	1.8541	-40.5375	
53.7200	-37.8800	0.0063	-0.2741	-
0.3262	-0.2912	0.4321	0.4847	0.4101
	0.3141	1.8535	-40.5370	
53.7400	-37.6100	0.1036	-0.1541	-
0.2831	-0.3007	0.4691	0.4748	0.3924
	0.3137	1.8528	-40.5364	

53.7600 -37.4100 0.1510 -0.0170 -
0.1574 -0.2936 0.5038 0.4632 0.3745
0.3132 1.8521 -40.5359
53.7800 -37.2200 0.0700 -0.0245
0.0301 -0.2711 0.5357 0.4498 0.3561
0.3127 1.8514 -40.5353
53.8000 -37.2300 -0.1798 -0.0719
0.2390 -0.2349 0.5646 0.4348 0.3375
0.3121 1.8506 -40.5348
53.8200 -36.8300 -0.0760 0.0117
0.4230 -0.1866 0.5899 0.4180 0.3185
0.3116 1.8498 -40.5342
53.8400 -36.5100 -0.0154 0.1786
0.5397 -0.1277 0.6115 0.3994 0.2992
0.3110 1.8490 -40.5337
53.8600 -35.9500 0.3870 0.2126
0.5601 -0.0604 0.6288 0.3792 0.2797
0.3103 1.8481 -40.5331
53.8800 -36.8200 -0.1941 0.0295
0.4714 0.0128 0.6415 0.3572 0.2599
0.3097 1.8472 -40.5325
53.9000 -37.2100 -0.2554 -0.0864
0.2828 0.0898 0.6491 0.3336 0.2398
0.3090 1.8463 -40.5320
53.9200 -37.0600 -0.1111 0.0683
0.0207 0.1681 0.6510 0.3083 0.2196
0.3083 1.8454 -40.5314
53.9400 -36.5100 0.5126 0.2149 -
0.2720 0.2453 0.6467 0.2815 0.1991
0.3075 1.8444 -40.5309
53.9600 -37.9600 -0.4408 0.0645 -
0.5389 0.3187 0.6358 0.2531 0.1784
0.3067 1.8434 -40.5303
53.9800 -37.4300 0.4976 -0.2938 -
0.7218 0.3857 0.6176 0.2233 0.1575
0.3060 1.8423 -40.5297
54.0000 -38.9000 -0.7009 -0.3616 -
0.7756 0.4440 0.5918 0.1921 0.1365
0.3051 1.8412 -40.5292
54.0200 -37.3500 0.4509 0.0285 -
0.6811 0.4907 0.5582 0.1598 0.1153
0.3043 1.8401 -40.5286
54.0400 -37.0700 0.0971 0.3796 -
0.4655 0.5236 0.5167 0.1265 0.0940
0.3034 1.8390 -40.5280
54.0600 -37.2300 -0.2216 0.3178 -
0.1675 0.5408 0.4675 0.0922 0.0726
0.3025 1.8378 -40.5275
54.0800 -37.0200 0.1896 -0.0627
0.1702 0.5403 0.4110 0.0573 0.0511
0.3016 1.8366 -40.5269

54.1000 -37.5100 -0.2878 -0.4011
0.4961 0.5193 0.3476 0.0217 0.0296
0.3007 1.8354 -40.5263
54.1200 -36.9200 0.3681 -0.4827
0.7590 0.4752 0.2781 -0.0142 0.0079
0.2997 1.8341 -40.5257
54.1400 -37.3000 -0.0975 -0.1931
0.9104 0.4065 0.2036 -0.0504 -0.0137
0.2988 1.8328 -40.5251
54.1600 -37.3400 -0.5863 0.3720
0.9171 0.3137 0.1249 -0.0867 -0.0354
0.2978 1.8315 -40.5246
54.1800 -36.0500 0.7291 0.7791
0.7638 0.2000 0.0432 -0.1228 -0.0571
0.2968 1.8301 -40.5240
54.2000 -37.7200 -0.1424 0.5860
0.4609 0.0714 -0.0404 -0.1588 -0.0788
0.2958 1.8288 -40.5234
54.2200 -39.3600 -0.5611 -0.0272
0.0695 -0.0649 -0.1247 -0.1943 -0.1004
0.2947 1.8273 -40.5228
54.2400 -39.6500 0.5987 -0.6450 -
0.3311 -0.2015 -0.2087 -0.2293 -0.1220
0.2937 1.8259 -40.5222
54.2600 -41.5400 -0.5639 -0.8351 -
0.6622 -0.3313 -0.2912 -0.2636 -0.1436
0.2926 1.8244 -40.5216
54.2800 -41.0900 0.0472 -0.5770 -
0.8616 -0.4469 -0.3710 -0.2970 -0.1651
0.2915 1.8229 -40.5210
54.3000 -40.5600 0.4699 -0.1815 -
0.9074 -0.5419 -0.4471 -0.3293 -0.1864
0.2905 1.8213 -40.5204
54.3200 -40.8000 0.0400 0.0903 -
0.8143 -0.6118 -0.5184 -0.3604 -0.2077
0.2894 1.8197 -40.5199
54.3400 -41.0300 -0.4775 0.2784 -
0.6222 -0.6556 -0.5838 -0.3902 -0.2288
0.2883 1.8181 -40.5193
54.3600 -40.3700 -0.1234 0.4999 -
0.3769 -0.6750 -0.6425 -0.4184 -0.2498
0.2871 1.8165 -40.5187
54.3800 -39.5300 0.4485 0.6271 -
0.1134 -0.6731 -0.6936 -0.4449 -0.2706
0.2860 1.8148 -40.5181
54.4000 -39.8100 0.2287 0.4365
0.1410 -0.6535 -0.7366 -0.4696 -0.2912
0.2849 1.8131 -40.5175
54.4200 -40.4700 -0.1849 -0.0084
0.3617 -0.6203 -0.7710 -0.4922 -0.3116
0.2837 1.8113 -40.5169

54.4400 -40.7700 -0.4027 -0.3576
0.5277 -0.5779 -0.7962 -0.5127 -0.3319
0.2826 1.8096 -40.5162
54.4600 -40.7500 -0.3802 -0.3093
0.6238 -0.5304 -0.8121 -0.5309 -0.3518
0.2814 1.8077 -40.5156
54.4800 -40.0400 -0.1643 0.0613
0.6421 -0.4820 -0.8182 -0.5467 -0.3716
0.2803 1.8059 -40.5150
54.5000 -38.9500 0.6742 0.4048
0.5821 -0.4361 -0.8144 -0.5601 -0.3910
0.2791 1.8040 -40.5144
54.5200 -39.4800 0.4314 0.3703
0.4506 -0.3949 -0.8010 -0.5709 -0.4102
0.2779 1.8021 -40.5138
54.5400 -40.3300 -0.0764 0.0196
0.2721 -0.3596 -0.7785 -0.5794 -0.4291
0.2768 1.8002 -40.5132
54.5600 -41.5800 -0.8584 -0.2562
0.0814 -0.3307 -0.7477 -0.5855 -0.4476
0.2756 1.7982 -40.5126
54.5800 -40.4600 0.2774 -0.2520 -
0.0859 -0.3080 -0.7098 -0.5892 -0.4658
0.2744 1.7962 -40.5120
54.6000 -40.2900 0.5390 -0.1584 -
0.1968 -0.2905 -0.6663 -0.5905 -0.4837
0.2732 1.7942 -40.5114
54.6200 -40.7800 0.0817 -0.1915 -
0.2319 -0.2747 -0.6187 -0.5897 -0.5012
0.2721 1.7921 -40.5107
54.6400 -41.3600 -0.4977 -0.2092 -
0.2042 -0.2542 -0.5685 -0.5865 -0.5184
0.2709 1.7900 -40.5101
54.6600 -40.4600 -0.0087 -0.0281 -
0.1451 -0.2213 -0.5171 -0.5812 -0.5351
0.2697 1.7879 -40.5095
54.6800 -39.7500 0.3007 0.2336 -
0.0958 -0.1688 -0.4659 -0.5738 -0.5515
0.2686 1.7857 -40.5089
54.7000 -39.8000 0.0651 0.3686 -
0.0946 -0.0908 -0.4162 -0.5644 -0.5676
0.2674 1.7835 -40.5082
54.7200 -40.1200 -0.3537 0.3359 -
0.1595 0.0157 -0.3687 -0.5529 -0.5832
0.2662 1.7812 -40.5076
54.7400 -39.6300 0.3780 0.1429 -
0.2801 0.1487 -0.3240 -0.5394 -0.5984
0.2651 1.7790 -40.5070
54.7600 -40.3700 -0.1659 -0.2115 -
0.4223 0.3032 -0.2823 -0.5241 -0.6132
0.2640 1.7767 -40.5064

54.7800 -40.5600 -0.1017 -0.4851 -
0.5404 0.4731 -0.2437 -0.5070 -0.6276
0.2628 1.7743 -40.5057
54.8000 -40.1900 -0.0116 -0.4475 -
0.5834 0.6511 -0.2083 -0.4881 -0.6416
0.2617 1.7720 -40.5051
54.8200 -39.6200 0.2191 -0.2795 -
0.5106 0.8296 -0.1765 -0.4676 -0.6552
0.2606 1.7696 -40.5044
54.8400 -39.5000 -0.1757 -0.2984 -
0.3044 0.9993 -0.1483 -0.4454 -0.6684
0.2595 1.7671 -40.5038
54.8600 -38.7400 0.3051 -0.4873
0.0054 1.1498 -0.1238 -0.4217 -0.6811
0.2584 1.7647 -40.5032
54.8800 -38.9300 -0.3887 -0.5207
0.3549 1.2701 -0.1033 -0.3966 -0.6935
0.2573 1.7622 -40.5025
54.9000 -37.7500 -0.0299 -0.1397
0.6752 1.3495 -0.0869 -0.3701 -0.7053
0.2562 1.7596 -40.5019
54.9200 -36.6300 0.2575 0.5143
0.8999 1.3771 -0.0746 -0.3425 -0.7168
0.2551 1.7571 -40.5012
54.9400 -36.5300 -0.2389 1.0192
0.9652 1.3432 -0.0666 -0.3137 -0.7278
0.2541 1.7545 -40.5006
54.9600 -35.8500 0.7664 0.8987
0.8236 1.2439 -0.0625 -0.2841 -0.7384
0.2530 1.7518 -40.5000
54.9800 -38.0400 -0.2755 0.1723
0.5114 1.0865 -0.0617 -0.2537 -0.7485
0.2520 1.7492 -40.4993
55.0000 -39.7500 -0.6830 -0.5571
0.1180 0.8810 -0.0635 -0.2228 -0.7582
0.2510 1.7465 -40.4987
55.0200 -40.1200 -0.1539 -0.7706 -
0.2606 0.6377 -0.0671 -0.1915 -0.7675
0.2500 1.7437 -40.4980
55.0400 -39.9800 0.3470 -0.5740 -
0.5375 0.3671 -0.0717 -0.1601 -0.7763
0.2491 1.7410 -40.4974
55.0600 -40.0100 0.5166 -0.3821 -
0.6517 0.0794 -0.0765 -0.1286 -0.7846
0.2481 1.7382 -40.4967
55.0800 -40.6900 -0.1528 -0.2924 -
0.6002 -0.2154 -0.0809 -0.0973 -0.7925
0.2472 1.7353 -40.4960
55.1000 -41.1600 -0.6231 -0.1418 -
0.4277 -0.5073 -0.0839 -0.0664 -0.7999
0.2463 1.7325 -40.4954

55.1200	-39.7800	0.6168	0.0592	-
0.1911	-0.7855	-0.0848	-0.0360	-0.8068
	0.2454	1.7295	-40.4947	
55.1400	-40.3600	-0.1222	0.1457	
0.0532	-1.0390	-0.0829	-0.0064	-0.8133
	0.2445	1.7266	-40.4941	
55.1600	-40.5300	-0.3351	0.2132	
0.2477	-1.2568	-0.0774	0.0222	-0.8194
	0.2436	1.7236	-40.4934	
55.1800	-40.0500	0.0919	0.3615	
0.3470	-1.4293	-0.0674	0.0498	-0.8249
	0.2428	1.7206	-40.4927	
55.2000	-39.9700	0.1467	0.4179	
0.3333	-1.5492	-0.0522	0.0760	-0.8300
	0.2420	1.7176	-40.4921	
55.2200	-40.2900	0.2356	0.1829	
0.2178	-1.6130	-0.0313	0.1008	-0.8346
	0.2412	1.7145	-40.4914	
55.2400	-40.8900	0.1619	-0.2489	
0.0335	-1.6200	-0.0042	0.1240	-0.8387
	0.2405	1.7114	-40.4907	
55.2600	-41.9100	-0.4386	-0.5259	-
0.1807	-1.5708	0.0289	0.1455	-0.8424
	0.2397	1.7083	-40.4901	
55.2800	-41.7200	-0.4913	-0.3001	-
0.3833	-1.4678	0.0675	0.1653	-0.8456
	0.2390	1.7051	-40.4894	
55.3000	-41.1800	-0.6080	0.3498	-
0.5348	-1.3144	0.1106	0.1834	-0.8482
	0.2383	1.7019	-40.4887	
55.3200	-39.0000	1.0642	0.6979	-
0.5956	-1.1165	0.1574	0.1997	-0.8504
	0.2377	1.6987	-40.4880	
55.3400	-40.8000	-0.4932	0.1924	-
0.5375	-0.8818	0.2069	0.2142	-0.8521
	0.2370	1.6954	-40.4874	
55.3600	-41.2400	-0.7425	-0.6070	-
0.3685	-0.6194	0.2583	0.2269	-0.8534
	0.2364	1.6921	-40.4867	
55.3800	-39.9000	0.3744	-0.8623	-
0.1143	-0.3394	0.3106	0.2378	-0.8541
	0.2358	1.6887	-40.4860	
55.4000	-39.5900	-0.6740	-0.1675	
0.1812	-0.0520	0.3629	0.2470	-0.8543
	0.2353	1.6853	-40.4853	
55.4200	-36.4800	0.8589	0.6461	
0.4456	0.2325	0.4143	0.2544	-0.8541
	0.2347	1.6819	-40.4846	
55.4400	-36.6700	0.0221	0.7797	
0.6050	0.5037	0.4638	0.2600	-0.8533
	0.2342	1.6785	-40.4840	

55.4600 -37.5400 -0.6387 0.4100
0.6169 0.7512 0.5106 0.2639 -0.8520
0.2337 1.6750 -40.4833
55.4800 -36.9100 0.2223 -0.0428
0.4984 0.9662 0.5537 0.2660 -0.8503
0.2333 1.6715 -40.4826
55.5000 -37.1200 0.2827 -0.3554
0.2944 1.1424 0.5921 0.2664 -0.8481
0.2328 1.6680 -40.4819
55.5200 -38.0100 -0.3528 -0.4619
0.0600 1.2763 0.6248 0.2651 -0.8453
0.2324 1.6644 -40.4812
55.5400 -37.4600 0.2169 -0.3797 -
0.1518 1.3660 0.6508 0.2621 -0.8421
0.2320 1.6608 -40.4805
55.5600 -37.5400 0.0168 -0.1630 -
0.2989 1.4104 0.6690 0.2574 -0.8384
0.2317 1.6571 -40.4798
55.5800 -37.6500 -0.3179 0.1108 -
0.3593 1.4096 0.6786 0.2511 -0.8343
0.2313 1.6534 -40.4791
55.6000 -36.9400 0.2399 0.2608 -
0.3242 1.3645 0.6791 0.2431 -0.8296
0.2310 1.6497 -40.4784
55.6200 -37.1700 0.2002 0.1599 -
0.1975 1.2772 0.6709 0.2337 -0.8245
0.2307 1.6460 -40.4777
55.6400 -37.7900 -0.2646 -0.1333 -
0.0071 1.1520 0.6543 0.2227 -0.8190
0.2304 1.6422 -40.4770
55.6600 -37.7400 0.0170 -0.3703
0.2017 0.9945 0.6300 0.2104 -0.8130
0.2302 1.6384 -40.4763
55.6800 -37.7400 0.0731 -0.2923
0.3821 0.8111 0.5987 0.1969 -0.8065
0.2300 1.6345 -40.4756
55.7000 -37.8100 -0.3365 0.1041
0.4993 0.6083 0.5608 0.1821 -0.7996
0.2298 1.6306 -40.4749
55.7200 -36.9300 0.3282 0.5200
0.5267 0.3930 0.5172 0.1663 -0.7922
0.2296 1.6267 -40.4742
55.7400 -37.1200 0.3335 0.6660
0.4490 0.1725 0.4684 0.1496 -0.7844
0.2294 1.6228 -40.4735
55.7600 -38.3700 -0.3435 0.5138
0.2763 -0.0457 0.4150 0.1319 -0.7762
0.2293 1.6188 -40.4728
55.7800 -39.0400 -0.0688 0.2061
0.0436 -0.2536 0.3578 0.1136 -0.7675
0.2292 1.6148 -40.4721

55.8000 -39.6700 0.2460 -0.1759 -
0.2000 -0.4438 0.2973 0.0945 -0.7585
0.2291 1.6107 -40.4714
55.8200 -40.5400 0.0492 -0.5812 -
0.4014 -0.6082 0.2342 0.0750 -0.7490
0.2290 1.6066 -40.4707
55.8400 -41.6800 -0.4169 -0.7929 -
0.5174 -0.7394 0.1691 0.0549 -0.7391
0.2289 1.6025 -40.4700
55.8600 -41.3100 -0.1895 -0.5819 -
0.5222 -0.8309 0.1027 0.0346 -0.7288
0.2289 1.5983 -40.4692
55.8800 -40.6500 -0.0113 -0.0182 -
0.4322 -0.8796 0.0356 0.0139 -0.7182
0.2289 1.5942 -40.4685
55.9000 -39.9800 -0.0136 0.5267 -
0.2844 -0.8869 -0.0316 -0.0069 -0.7071
0.2289 1.5899 -40.4678
55.9200 -39.1700 0.7212 0.5992 -
0.1154 -0.8571 -0.0982 -0.0277 -0.6957
0.2289 1.5857 -40.4671
55.9400 -40.5000 -0.3979 0.1686
0.0452 -0.7959 -0.1635 -0.0484 -0.6840
0.2289 1.5814 -40.4664
55.9600 -40.8600 -0.3739 -0.2528
0.1708 -0.7094 -0.2268 -0.0691 -0.6718
0.2290 1.5771 -40.4656
55.9800 -40.2700 0.0793 -0.3179
0.2392 -0.6045 -0.2872 -0.0894 -0.6593
0.2291 1.5727 -40.4649
56.0000 -40.1700 -0.0048 -0.1058
0.2454 -0.4881 -0.3440 -0.1094 -0.6465
0.2292 1.5684 -40.4642
56.0200 -39.8500 -0.0055 0.1270
0.2054 -0.3673 -0.3964 -0.1289 -0.6334
0.2293 1.5639 -40.4635
56.0400 -39.5600 0.1346 0.2263
0.1445 -0.2493 -0.4437 -0.1479 -0.6199
0.2294 1.5595 -40.4627
56.0600 -39.8200 -0.0225 0.1468
0.0849 -0.1409 -0.4851 -0.1662 -0.6061
0.2295 1.5550 -40.4620
56.0800 -40.0600 0.0198 -0.0594
0.0461 -0.0479 -0.5199 -0.1838 -0.5920
0.2296 1.5505 -40.4613
56.1000 -40.1800 0.0156 -0.2427
0.0387 0.0257 -0.5472 -0.2005 -0.5776
0.2298 1.5459 -40.4605
56.1200 -40.2300 -0.1091 -0.2937
0.0633 0.0774 -0.5665 -0.2163 -0.5629
0.2300 1.5414 -40.4598

56.1400 -39.6600 0.2868 -0.1776
0.1056 0.1071 -0.5773 -0.2310 -0.5480
0.2302 1.5367 -40.4591
56.1600 -39.8600 -0.1740 0.0474
0.1429 0.1156 -0.5795 -0.2446 -0.5327
0.2304 1.5321 -40.4583
56.1800 -39.8900 -0.3184 0.2752
0.1509 0.1051 -0.5733 -0.2571 -0.5172
0.2306 1.5274 -40.4576
56.2000 -38.8600 0.5255 0.3643
0.1110 0.0787 -0.5588 -0.2683 -0.5015
0.2308 1.5227 -40.4568
56.2200 -39.9300 -0.2172 0.2012
0.0232 0.0400 -0.5366 -0.2783 -0.4856
0.2310 1.5180 -40.4561
56.2400 -40.3800 -0.2769 -0.0251 -
0.0871 -0.0073 -0.5071 -0.2871 -0.4694
0.2313 1.5132 -40.4553
56.2600 -40.4800 -0.2705 -0.1187 -
0.1863 -0.0599 -0.4710 -0.2947 -0.4530
0.2315 1.5084 -40.4546
56.2800 -39.8800 0.6147 -0.1984 -
0.2387 -0.1146 -0.4290 -0.3012 -0.4363
0.2318 1.5036 -40.4539
56.3000 -40.9900 -0.4662 -0.3087 -
0.2207 -0.1678 -0.3821 -0.3065 -0.4195
0.2321 1.4987 -40.4531
56.3200 -40.6300 -0.2474 -0.2477 -
0.1357 -0.2169 -0.3310 -0.3108 -0.4025
0.2323 1.4938 -40.4524
56.3400 -39.4000 0.4739 -0.0165 -
0.0093 -0.2596 -0.2765 -0.3139 -0.3854
0.2326 1.4889 -40.4516
56.3600 -39.7600 -0.1471 0.2005
0.1176 -0.2939 -0.2194 -0.3160 -0.3680
0.2329 1.4839 -40.4509
56.3800 -39.4000 -0.1778 0.2846
0.2071 -0.3180 -0.1606 -0.3170 -0.3505
0.2332 1.4789 -40.4501
56.4000 -39.1600 0.2230 0.1865
0.2349 -0.3300 -0.1006 -0.3171 -0.3329
0.2336 1.4739 -40.4493
56.4200 -39.5200 0.0601 -0.0523
0.1963 -0.3291 -0.0404 -0.3162 -0.3151
0.2339 1.4688 -40.4486
56.4400 -39.8300 -0.0788 -0.1965
0.1001 -0.3151 0.0195 -0.3143 -0.2971
0.2342 1.4637 -40.4478
56.4600 -40.1700 -0.5577 -0.0509 -
0.0328 -0.2878 0.0784 -0.3115 -0.2791
0.2345 1.4586 -40.4471

56.4800 -38.7800 0.6324 0.1855 -
0.1696 -0.2476 0.1355 -0.3078 -0.2609
0.2349 1.4535 -40.4463
56.5000 -39.7100 -0.2632 0.1290 -
0.2747 -0.1949 0.1904 -0.3033 -0.2426
0.2352 1.4483 -40.4455
56.5200 -39.5900 -0.0652 -0.1018 -
0.3215 -0.1310 0.2423 -0.2979 -0.2242
0.2355 1.4431 -40.4448
56.5400 -39.5400 0.0670 -0.2385 -
0.2976 -0.0583 0.2907 -0.2917 -0.2057
0.2359 1.4378 -40.4440
56.5600 -39.6100 -0.3172 -0.1645 -
0.2106 0.0199 0.3350 -0.2847 -0.1871
0.2362 1.4326 -40.4432
56.5800 -38.5900 0.1955 0.0538 -
0.0865 0.0992 0.3746 -0.2770 -0.1685
0.2366 1.4273 -40.4425
56.6000 -38.2300 0.1006 0.2342
0.0429 0.1752 0.4089 -0.2685 -0.1498
0.2369 1.4220 -40.4417
56.6200 -38.2900 -0.2072 0.2277
0.1481 0.2436 0.4374 -0.2593 -0.1310
0.2373 1.4166 -40.4409
56.6400 -37.8500 0.3464 0.0383
0.2091 0.2998 0.4594 -0.2495 -0.1122
0.2377 1.4112 -40.4402
56.6600 -38.6400 -0.4215 -0.1441
0.2271 0.3400 0.4745 -0.2391 -0.0933
0.2380 1.4058 -40.4394
56.6800 -37.9300 0.2312 -0.1390
0.2174 0.3613 0.4822 -0.2281 -0.0744
0.2384 1.4003 -40.4386
56.7000 -37.8500 0.1497 -0.0591
0.1973 0.3620 0.4824 -0.2165 -0.0554
0.2387 1.3949 -40.4378
56.7200 -38.0800 -0.1532 -0.0221
0.1767 0.3417 0.4750 -0.2044 -0.0365
0.2391 1.3894 -40.4371
56.7400 -37.8900 0.2488 -0.0588
0.1522 0.3021 0.4604 -0.1918 -0.0175
0.2394 1.3838 -40.4363
56.7600 -38.6300 -0.4163 -0.0171
0.1105 0.2464 0.4392 -0.1788 0.0015
0.2398 1.3783 -40.4355
56.7800 -38.0600 0.0238 0.1462
0.0426 0.1793 0.4122 -0.1653 0.0205
0.2401 1.3727 -40.4347
56.8000 -37.6300 0.4560 0.2436 -
0.0465 0.1054 0.3799 -0.1515 0.0394
0.2405 1.3671 -40.4339

56.8200 -38.8500 -0.3356 0.0821 -
0.1362 0.0292 0.3432 -0.1374 0.0584
0.2408 1.3614 -40.4331
56.8400 -38.9700 0.0105 -0.1848 -
0.1989 -0.0456 0.3029 -0.1230 0.0773
0.2411 1.3557 -40.4324
56.8600 -39.3500 -0.1522 -0.3048 -
0.2149 -0.1153 0.2598 -0.1085 0.0962
0.2415 1.3500 -40.4316
56.8800 -39.2200 0.0083 -0.1893 -
0.1858 -0.1770 0.2146 -0.0938 0.1150
0.2418 1.3443 -40.4308
56.9000 -38.8400 0.1386 0.0636 -
0.1308 -0.2286 0.1682 -0.0790 0.1338
0.2421 1.3386 -40.4300
56.9200 -38.9000 -0.0721 0.2877 -
0.0768 -0.2683 0.1214 -0.0642 0.1526
0.2424 1.3328 -40.4292
56.9400 -38.7200 -0.0905 0.3859 -
0.0437 -0.2953 0.0748 -0.0494 0.1712
0.2427 1.3270 -40.4284
56.9600 -38.6400 0.1073 0.2754 -
0.0341 -0.3094 0.0292 -0.0348 0.1898
0.2430 1.3211 -40.4276
56.9800 -38.9700 0.1785 -0.0471 -
0.0352 -0.3117 -0.0148 -0.0203 0.2083
0.2433 1.3152 -40.4268
57.0000 -39.5300 -0.1496 -0.3826 -
0.0298 -0.3038 -0.0567 -0.0060 0.2267
0.2436 1.3094 -40.4260
57.0200 -39.6700 -0.2351 -0.4583 -
0.0058 -0.2873 -0.0962 0.0080 0.2450
0.2439 1.3034 -40.4252
57.0400 -39.1400 0.0005 -0.1959
0.0358 -0.2636 -0.1327 0.0216 0.2632
0.2442 1.2975 -40.4244
57.0600 -38.6200 0.0377 0.1877
0.0795 -0.2340 -0.1659 0.0349 0.2812
0.2444 1.2915 -40.4236
57.0800 -38.3500 0.0884 0.4161
0.1050 -0.1999 -0.1956 0.0476 0.2992
0.2447 1.2855 -40.4228
57.1000 -38.3200 0.1688 0.3361
0.0996 -0.1629 -0.2216 0.0599 0.3170
0.2449 1.2795 -40.4220
57.1200 -38.4500 0.3151 -0.0139
0.0650 -0.1246 -0.2437 0.0717 0.3346
0.2451 1.2734 -40.4212
57.1400 -39.6800 -0.6405 -0.2800
0.0119 -0.0861 -0.2618 0.0829 0.3521
0.2453 1.2674 -40.4204

57.1600	-39.1600	-0.1969	-0.1743	-
0.0436	-0.0480	-0.2759	0.0934	0.3694
	0.2455	1.2613	-40.4196	
57.1800	-38.1900	0.4645	0.0769	-
0.0788	-0.0110	-0.2861	0.1034	0.3866
	0.2457	1.2551	-40.4188	
57.2000	-38.1000	0.5968	0.0579	-
0.0764	0.0244	-0.2924	0.1127	0.4036
	0.2459	1.2490	-40.4180	
57.2200	-39.5800	-0.6777	-0.1826	-
0.0346	0.0569	-0.2949	0.1213	0.4204
	0.2460	1.2428	-40.4172	
57.2400	-38.9100	-0.1051	-0.2201	
0.0343	0.0853	-0.2937	0.1293	0.4370
	0.2462	1.2366	-40.4163	
57.2600	-38.6400	-0.2239	0.0455	
0.1069	0.1077	-0.2887	0.1367	0.4534
	0.2463	1.2304	-40.4155	
57.2800	-37.5500	0.6254	0.2347	
0.1506	0.1223	-0.2801	0.1435	0.4696
	0.2464	1.2241	-40.4147	
57.3000	-38.2300	-0.0846	0.1936	
0.1419	0.1272	-0.2678	0.1496	0.4855
	0.2465	1.2179	-40.4139	
57.3200	-38.6600	-0.5065	0.0987	
0.0831	0.1221	-0.2520	0.1552	0.5012
	0.2466	1.2116	-40.4131	
57.3400	-38.0200	0.4525	0.0599	-
0.0050	0.1073	-0.2327	0.1601	0.5167
	0.2467	1.2052	-40.4122	
57.3600	-38.3800	0.1821	-0.0782	-
0.0844	0.0839	-0.2100	0.1645	0.5319
	0.2467	1.1989	-40.4114	
57.3800	-38.7300	0.0915	-0.3278	-
0.1169	0.0532	-0.1843	0.1683	0.5469
	0.2467	1.1925	-40.4106	
57.4000	-39.5000	-0.7469	-0.2806	-
0.0874	0.0177	-0.1557	0.1716	0.5616
	0.2467	1.1861	-40.4098	
57.4200	-37.6000	0.7196	0.0872	-
0.0161	-0.0197	-0.1244	0.1743	0.5760
	0.2467	1.1797	-40.4090	
57.4400	-38.0700	0.0508	0.2098	
0.0605	-0.0555	-0.0907	0.1766	0.5901
	0.2467	1.1733	-40.4081	
57.4600	-38.3700	-0.0782	0.0368	
0.1068	-0.0862	-0.0549	0.1784	0.6039
	0.2466	1.1668	-40.4073	
57.4800	-38.6600	-0.3580	-0.0781	
0.0997	-0.1089	-0.0173	0.1797	0.6173
	0.2466	1.1603	-40.4065	

57.5000 -38.5200 -0.3494 0.0897
0.0352 -0.1214 0.0216 0.1806 0.6305
0.2465 1.1538 -40.4056
57.5200 -38.3100 -0.3709 0.3339 -
0.0553 -0.1225 0.0615 0.1812 0.6434
0.2463 1.1473 -40.4048
57.5400 -37.2900 0.9277 0.1699 -
0.1259 -0.1112 0.1020 0.1813 0.6559
0.2462 1.1408 -40.4040
57.5600 -39.6000 -0.9052 -0.4056 -
0.1454 -0.0874 0.1425 0.1812 0.6680
0.2460 1.1342 -40.4031
57.5800 -38.4900 0.2403 -0.5860 -
0.1040 -0.0517 0.1825 0.1807 0.6798
0.2459 1.1276 -40.4023
57.6000 -38.1300 -0.0301 -0.1664 -
0.0144 -0.0064 0.2214 0.1799 0.6913
0.2456 1.1210 -40.4015
57.6200 -37.4000 0.0421 0.4034
0.0830 0.0455 0.2587 0.1789 0.7023
0.2454 1.1143 -40.4006
57.6400 -36.7200 0.3788 0.6261
0.1434 0.1009 0.2938 0.1777 0.7131
0.2451 1.1077 -40.3998
57.6600 -37.3500 -0.0486 0.3524
0.1387 0.1568 0.3263 0.1763 0.7234
0.2449 1.1010 -40.3989
57.6800 -37.7100 -0.1050 -0.0995
0.0817 0.2107 0.3555 0.1746 0.7333
0.2445 1.0943 -40.3981
57.7000 -37.8900 0.0631 -0.3741
0.0039 0.2605 0.3809 0.1728 0.7428
0.2442 1.0876 -40.3973
57.7200 -37.9000 -0.0992 -0.4073 -
0.0540 0.3043 0.4019 0.1709 0.7520
0.2438 1.0808 -40.3964
57.7400 -37.5800 0.0892 -0.2650 -
0.0631 0.3400 0.4181 0.1688 0.7607
0.2435 1.0741 -40.3956
57.7600 -37.4900 -0.1686 0.0016 -
0.0232 0.3665 0.4289 0.1666 0.7690
0.2430 1.0673 -40.3947
57.7800 -36.8000 0.2850 0.1965
0.0443 0.3832 0.4339 0.1643 0.7769
0.2426 1.0605 -40.3939
57.8000 -37.2500 -0.1777 0.2014
0.1074 0.3900 0.4327 0.1619 0.7844
0.2421 1.0537 -40.3930
57.8200 -36.9800 -0.0104 0.1526
0.1427 0.3878 0.4251 0.1595 0.7914
0.2416 1.0468 -40.3922

57.8400 -37.1300 -0.0343 0.1284
0.1459 0.3780 0.4111 0.1569 0.7980
0.2411 1.0400 -40.3913
57.8600 -37.0200 0.2461 0.0663
0.1324 0.3618 0.3906 0.1544 0.8042
0.2405 1.0331 -40.3905
57.8800 -37.7800 -0.3657 -0.0885
0.1218 0.3408 0.3636 0.1518 0.8099
0.2399 1.0262 -40.3896
57.9000 -37.2500 0.4643 -0.3229
0.1238 0.3173 0.3304 0.1493 0.8152
0.2393 1.0193 -40.3887
57.9200 -38.3400 -0.3854 -0.4727
0.1292 0.2940 0.2911 0.1468 0.8200
0.2386 1.0124 -40.3879
57.9400 -38.2000 -0.3270 -0.2769
0.1186 0.2738 0.2460 0.1443 0.8244
0.2379 1.0054 -40.3870
57.9600 -37.3100 0.0171 0.2889
0.0752 0.2589 0.1955 0.1419 0.8283
0.2372 0.9984 -40.3862
57.9800 -36.8400 0.1310 0.8548 -
0.0120 0.2508 0.1402 0.1396 0.8318
0.2364 0.9915 -40.3853
58.0000 -36.8500 0.2393 0.9519 -
0.1483 0.2499 0.0806 0.1374 0.8348
0.2356 0.9845 -40.3844
58.0200 -37.4200 0.5100 0.3905 -
0.3248 0.2555 0.0173 0.1354 0.8374
0.2348 0.9774 -40.3836
58.0400 -39.5700 -0.6000 -0.4931 -
0.4994 0.2665 -0.0489 0.1335 0.8395
0.2340 0.9704 -40.3827
58.0600 -39.5100 0.1158 -1.0963 -
0.6214 0.2814 -0.1172 0.1318 0.8411
0.2331 0.9633 -40.3818
58.0800 -39.9200 0.0802 -1.2140 -
0.6420 0.2981 -0.1869 0.1303 0.8423
0.2321 0.9563 -40.3810
58.1000 -40.1300 -0.3483 -1.0480 -
0.5323 0.3136 -0.2571 0.1290 0.8430
0.2312 0.9492 -40.3801
58.1200 -39.0500 0.2929 -0.8648 -
0.3003 0.3238 -0.3269 0.1280 0.8432
0.2302 0.9421 -40.3792
58.1400 -38.4300 0.6223 -0.7098
0.0140 0.3235 -0.3954 0.1272 0.8430
0.2292 0.9349 -40.3784
58.1600 -39.3300 -0.8503 -0.3471
0.3436 0.3076 -0.4620 0.1267 0.8423
0.2281 0.9278 -40.3775

58.1800 -37.4300 0.1611 0.4191
0.6219 0.2711 -0.5257 0.1265 0.8412
0.2270 0.9206 -40.3766
58.2000 -36.0500 0.6468 1.2321
0.7950 0.2096 -0.5859 0.1266 0.8395
0.2259 0.9135 -40.3757
58.2200 -36.5300 -0.1130 1.5609
0.8153 0.1199 -0.6419 0.1270 0.8375
0.2247 0.9063 -40.3749
58.2400 -36.6700 0.4992 1.0967
0.6628 0.0035 -0.6931 0.1278 0.8349
0.2235 0.8991 -40.3740
58.2600 -38.5600 0.1204 0.0968
0.3883 -0.1326 -0.7390 0.1289 0.8319
0.2223 0.8919 -40.3731
58.2800 -40.7400 -0.5284 -0.8512
0.0653 -0.2803 -0.7791 0.1305 0.8284
0.2210 0.8846 -40.3722
58.3000 -41.2700 -0.3029 -1.1750 -
0.2329 -0.4313 -0.8127 0.1324 0.8245
0.2197 0.8774 -40.3713
58.3200 -40.8700 0.0852 -0.8166 -
0.4483 -0.5774 -0.8394 0.1348 0.8201
0.2184 0.8701 -40.3705
58.3400 -40.3600 0.3478 -0.2001 -
0.5626 -0.7105 -0.8586 0.1376 0.8153
0.2170 0.8628 -40.3696
58.3600 -40.2500 0.0902 0.2540 -
0.5758 -0.8228 -0.8697 0.1408 0.8101
0.2156 0.8555 -40.3687
58.3800 -40.5900 -0.3460 0.4385 -
0.5080 -0.9075 -0.8723 0.1444 0.8044
0.2142 0.8482 -40.3678
58.4000 -39.9200 0.2689 0.4094 -
0.4017 -0.9588 -0.8658 0.1486 0.7982
0.2127 0.8409 -40.3669
58.4200 -40.4600 -0.1043 0.2182 -
0.3008 -0.9727 -0.8500 0.1531 0.7917
0.2112 0.8336 -40.3660
58.4400 -40.4500 -0.0482 -0.0218 -
0.2340 -0.9470 -0.8248 0.1580 0.7847
0.2097 0.8262 -40.3651
58.4600 -40.2600 0.1186 -0.1264 -
0.2072 -0.8823 -0.7904 0.1632 0.7773
0.2081 0.8189 -40.3642
58.4800 -40.3900 -0.2259 -0.0598 -
0.2076 -0.7826 -0.7477 0.1687 0.7695
0.2065 0.8115 -40.3633
58.5000 -39.7200 0.1451 0.0615 -
0.2137 -0.6544 -0.6974 0.1744 0.7613
0.2049 0.8041 -40.3625

58.5200 -39.5700 0.2263 0.0527 -
0.2015 -0.5048 -0.6402 0.1804 0.7527
0.2032 0.7967 -40.3616
58.5400 -39.7500 -0.1146 -0.1167 -
0.1467 -0.3416 -0.5768 0.1864 0.7437
0.2015 0.7893 -40.3607
58.5600 -39.7400 -0.3514 -0.2051 -
0.0349 -0.1720 -0.5081 0.1926 0.7343
0.1998 0.7819 -40.3598
58.5800 -38.5900 0.2643 -0.0820
0.1334 -0.0021 -0.4348 0.1987 0.7246
0.1980 0.7745 -40.3589
58.6000 -38.3500 -0.1542 0.1454
0.3345 0.1632 -0.3578 0.2049 0.7145
0.1963 0.7670 -40.3580
58.6200 -37.8200 -0.1815 0.2764
0.5292 0.3195 -0.2777 0.2110 0.7040
0.1944 0.7595 -40.3571
58.6400 -36.8600 0.5266 0.1393
0.6746 0.4623 -0.1954 0.2170 0.6932
0.1926 0.7521 -40.3562
58.6600 -37.4500 0.0191 -0.1625
0.7265 0.5873 -0.1116 0.2228 0.6820
0.1907 0.7446 -40.3553
58.6800 -38.0500 -0.5433 -0.2457
0.6516 0.6904 -0.0272 0.2284 0.6705
0.1888 0.7371 -40.3544
58.7000 -37.4000 -0.2587 0.0904
0.4452 0.7695 0.0569 0.2337 0.6587
0.1869 0.7296 -40.3534
58.7200 -36.3600 0.6386 0.4751
0.1360 0.8249 0.1400 0.2387 0.6465
0.1850 0.7221 -40.3525
58.7400 -37.4500 -0.1797 0.4367 -
0.2342 0.8576 0.2211 0.2433 0.6341
0.1830 0.7146 -40.3516
58.7600 -38.0100 -0.0199 0.0152 -
0.6018 0.8686 0.2996 0.2475 0.6214
0.1810 0.7070 -40.3507
58.7800 -38.7300 0.0824 -0.4521 -
0.8862 0.8589 0.3747 0.2513 0.6083
0.1789 0.6995 -40.3498
58.8000 -38.8800 0.0646 -0.6499 -
1.0042 0.8296 0.4460 0.2545 0.5950
0.1769 0.6919 -40.3489
58.8200 -39.2900 -0.7373 -0.3229 -
0.9091 0.7819 0.5129 0.2572 0.5814
0.1748 0.6844 -40.3480
58.8400 -36.8600 0.7996 0.2858 -
0.6343 0.7172 0.5752 0.2592 0.5676
0.1727 0.6768 -40.3471

58.8600	-37.7300	-0.4784	0.4353	-
0.2456	0.6373	0.6328	0.2606	0.5535
	0.1705	0.6692	-40.3462	
58.8800	-37.2200	-0.0729	0.1791	
0.1843	0.5446	0.6856	0.2612	0.5391
	0.1684	0.6616	-40.3452	
58.9000	-37.1500	-0.1967	0.0155	
0.5921	0.4413	0.7334	0.2611	0.5246
	0.1662	0.6540	-40.3443	
58.9200	-36.5800	0.1987	-0.0008	
0.9393	0.3282	0.7761	0.2602	0.5098
	0.1639	0.6464	-40.3434	
58.9400	-36.7400	-0.0623	-0.0476	
1.2091	0.2060	0.8137	0.2584	0.4948
	0.1617	0.6388	-40.3425	
58.9600	-36.6500	-0.0560	-0.1307	
1.3810	0.0766	0.8459	0.2559	0.4795
	0.1595	0.6312	-40.3416	
58.9800	-36.5700	0.1259	-0.0508	
1.4225	-0.0568	0.8728	0.2524	0.4641
	0.1572	0.6235	-40.3406	
59.0000	-37.1900	-0.5581	0.2632	
1.3009	-0.1901	0.8942	0.2480	0.4485
	0.1549	0.6159	-40.3397	
59.0200	-36.4300	0.3933	0.5313	
0.9992	-0.3190	0.9103	0.2426	0.4327
	0.1525	0.6082	-40.3388	
59.0400	-36.8700	0.6357	0.4069	
0.5296	-0.4387	0.9209	0.2364	0.4168
	0.1502	0.6006	-40.3379	
59.0600	-39.0200	-0.2189	-0.1103	-
0.0457	-0.5447	0.9260	0.2291	0.4007
	0.1478	0.5929	-40.3369	
59.0800	-40.4600	-0.7114	-0.4891	-
0.6363	-0.6321	0.9257	0.2208	0.3845
	0.1454	0.5852	-40.3360	
59.1000	-40.3000	0.0106	-0.3685	-
1.1541	-0.6962	0.9199	0.2116	0.3681
	0.1430	0.5776	-40.3351	
59.1200	-40.1500	0.1968	-0.0018	-
1.5422	-0.7322	0.9088	0.2015	0.3516
	0.1406	0.5699	-40.3342	
59.1400	-40.0400	0.5639	0.1547	-
1.7738	-0.7356	0.8922	0.1904	0.3349
	0.1381	0.5622	-40.3332	
59.1600	-41.2800	-0.5621	0.0494	-
1.8499	-0.7032	0.8704	0.1784	0.3182
	0.1357	0.5545	-40.3323	
59.1800	-40.9000	0.0158	-0.0647	-
1.7871	-0.6348	0.8437	0.1656	0.3014
	0.1332	0.5468	-40.3314	

59.2000 -40.4800 0.2172 -0.1033 -
1.6023 -0.5345 0.8121 0.1519 0.2845
0.1306 0.5391 -40.3304
59.2200 -40.0400 0.3139 -0.1895 -
1.2983 -0.4094 0.7760 0.1375 0.2675
0.1281 0.5313 -40.3295
59.2400 -40.6100 -0.6170 -0.3102 -
0.8761 -0.2676 0.7355 0.1225 0.2505
0.1256 0.5236 -40.3285
59.2600 -39.4900 0.0513 -0.4102 -
0.3515 -0.1169 0.6909 0.1067 0.2334
0.1230 0.5159 -40.3276
59.2800 -38.2300 0.7736 -0.4757
0.2372 0.0349 0.6423 0.0904 0.2162
0.1204 0.5082 -40.3267
59.3000 -38.9900 -0.8275 -0.3676
0.8255 0.1799 0.5900 0.0736 0.1990
0.1178 0.5004 -40.3257
59.3200 -36.3900 0.6837 0.1024
1.3316 0.3105 0.5343 0.0563 0.1818
0.1152 0.4927 -40.3248
59.3400 -36.2300 0.1148 0.6648
1.6737 0.4187 0.4752 0.0387 0.1646
0.1126 0.4849 -40.3238
59.3600 -36.5300 -0.6657 0.9709
1.7759 0.4970 0.4133 0.0207 0.1473
0.1099 0.4772 -40.3229
59.3800 -36.2400 0.0984 0.8162
1.5950 0.5405 0.3488 0.0025 0.1301
0.1072 0.4694 -40.3220
59.4000 -36.7100 0.7109 0.2272
1.1861 0.5515 0.2821 -0.0159 0.1128
0.1046 0.4617 -40.3210
59.4200 -39.4500 -0.5567 -0.4821
0.6440 0.5346 0.2137 -0.0344 0.0956
0.1019 0.4539 -40.3201
59.4400 -40.5300 -0.6505 -0.8402
0.0666 0.4940 0.1441 -0.0529 0.0784
0.0992 0.4461 -40.3191
59.4600 -40.7100 -0.2786 -0.6430 -
0.4560 0.4343 0.0736 -0.0715 0.0612
0.0964 0.4384 -40.3182
59.4800 -39.7600 0.8219 -0.2233 -
0.8546 0.3599 0.0029 -0.0899 0.0441
0.0937 0.4306 -40.3172
59.5000 -40.9100 -0.1263 -0.0241 -
1.0788 0.2750 -0.0678 -0.1082 0.0271
0.0909 0.4228 -40.3163
59.5200 -41.3300 -0.2693 -0.0932 -
1.1145 0.1840 -0.1379 -0.1263 0.0101
0.0882 0.4150 -40.3153

59.5400 -41.6300 -0.4194 -0.1644 -
0.9860 0.0908 -0.2070 -0.1441 -0.0069
0.0854 0.4072 -40.3144
59.5600 -40.8300 0.3012 -0.0925 -
0.7419 -0.0003 -0.2746 -0.1616 -0.0237
0.0826 0.3995 -40.3134
59.5800 -40.9500 -0.0009 0.0343 -
0.4382 -0.0849 -0.3403 -0.1787 -0.0405
0.0798 0.3917 -40.3124
59.6000 -41.0100 -0.3138 0.1773 -
0.1293 -0.1581 -0.4036 -0.1953 -0.0571
0.0770 0.3839 -40.3115
59.6200 -40.3600 -0.0199 0.3300
0.1410 -0.2155 -0.4640 -0.2114 -0.0737
0.0741 0.3761 -40.3105
59.6400 -40.0600 0.2123 0.3270
0.3379 -0.2534 -0.5211 -0.2270 -0.0901
0.0713 0.3683 -40.3096
59.6600 -40.0200 0.5978 0.0348
0.4418 -0.2696 -0.5744 -0.2420 -0.1064
0.0685 0.3605 -40.3086
59.6800 -41.7800 -0.8517 -0.2470
0.4509 -0.2643 -0.6233 -0.2564 -0.1226
0.0656 0.3527 -40.3077
59.7000 -40.7500 0.1923 -0.0828
0.3750 -0.2399 -0.6670 -0.2700 -0.1386
0.0627 0.3449 -40.3067
59.7200 -40.3000 0.4014 0.3078
0.2333 -0.2007 -0.7049 -0.2829 -0.1545
0.0598 0.3371 -40.3057
59.7400 -40.2000 0.7267 0.3563
0.0565 -0.1528 -0.7363 -0.2950 -0.1702
0.0570 0.3293 -40.3048
59.7600 -41.9700 -0.5574 0.0116 -
0.1138 -0.1023 -0.7606 -0.3062 -0.1858
0.0541 0.3215 -40.3038
59.7800 -42.2300 -0.4648 -0.2593 -
0.2346 -0.0553 -0.7774 -0.3165 -0.2011
0.0511 0.3137 -40.3028
59.8000 -41.9500 -0.2171 -0.1797 -
0.2726 -0.0170 -0.7862 -0.3259 -0.2163
0.0482 0.3059 -40.3019
59.8200 -41.0100 0.6138 -0.0724 -
0.2236 0.0086 -0.7865 -0.3342 -0.2313
0.0453 0.2981 -40.3009
59.8400 -41.5900 0.0029 -0.1422 -
0.1040 0.0203 -0.7783 -0.3416 -0.2461
0.0424 0.2903 -40.2999
59.8600 -41.8900 -0.4622 -0.1786
0.0506 0.0184 -0.7615 -0.3479 -0.2606
0.0394 0.2825 -40.2990

59. 8800 -41. 0500 0. 1314 0. 0096
0. 1972 0. 0038 -0. 7365 -0. 3531 -0. 2749
0. 0365 0. 2747 -40. 2980
59. 9000 -40. 8100 -0. 0684 0. 2811
0. 2978 -0. 0219 -0. 7039 -0. 3572 -0. 2890
0. 0335 0. 2669 -40. 2970
59. 9200 -40. 5200 0. 2116 0. 3369
0. 3264 -0. 0565 -0. 6641 -0. 3604 -0. 3029
0. 0306 0. 2591 -40. 2960
59. 9400 -41. 1800 -0. 0140 0. 1172
0. 2741 -0. 0965 -0. 6181 -0. 3624 -0. 3165
0. 0276 0. 2513 -40. 2951
59. 9600 -41. 4700 -0. 0818 -0. 1058
0. 1551 -0. 1371 -0. 5665 -0. 3635 -0. 3299
0. 0247 0. 2435 -40. 2941
59. 9800 -41. 7000 -0. 1401 -0. 1143 -
0. 0075 -0. 1735 -0. 5101 -0. 3636 -0. 3430
0. 0217 0. 2357 -40. 2931
60. 0000 -41. 3700 0. 1153 0. 0434 -
0. 1768 -0. 2010 -0. 4497 -0. 3627 -0. 3559
0. 0187 0. 2279 -40. 2921
60. 0200 -41. 4600 0. 1902 0. 1391 -
0. 3062 -0. 2150 -0. 3861 -0. 3609 -0. 3684
0. 0157 0. 2201 -40. 2912
60. 0400 -41. 6300 -0. 0554 0. 0092 -
0. 3612 -0. 2115 -0. 3199 -0. 3582 -0. 3808
0. 0128 0. 2123 -40. 2902
60. 0600 -42. 0000 -0. 1210 -0. 2234 -
0. 3296 -0. 1890 -0. 2521 -0. 3545 -0. 3928
0. 0098 0. 2046 -40. 2892
60. 0800 -41. 7400 0. 0162 -0. 3072 -
0. 2163 -0. 1492 -0. 1833 -0. 3500 -0. 4046
0. 0068 0. 1968 -40. 2882
60. 1000 -41. 3700 -0. 2195 -0. 1256 -
0. 0481 -0. 0959 -0. 1144 -0. 3446 -0. 4161
0. 0038 0. 1890 -40. 2872
60. 1200 -40. 3800 0. 3018 0. 1415
0. 1301 -0. 0337 -0. 0460 -0. 3384 -0. 4273
0. 0008 0. 1812 -40. 2862
60. 1400 -40. 3200 -0. 0283 0. 2247
0. 2729 0. 0328 0. 0211 -0. 3314 -0. 4382
-0. 0022 0. 1734 -40. 2853
60. 1600 -40. 3000 -0. 0885 0. 1515
0. 3487 0. 0989 0. 0860 -0. 3236 -0. 4489
-0. 0052 0. 1657 -40. 2843
60. 1800 -40. 3300 -0. 0217 0. 0682
0. 3453 0. 1606 0. 1481 -0. 3151 -0. 4593
-0. 0082 0. 1579 -40. 2833
60. 2000 -40. 4100 -0. 1094 -0. 0068
0. 2725 0. 2145 0. 2066 -0. 3057 -0. 4693
-0. 0112 0. 1501 -40. 2823

60.2200 -40.3400 0.0886 -0.1041
0.1542 0.2585 0.2608 -0.2957 -0.4791
-0.0142 0.1424 -40.2813
60.2400 -40.1700 0.3099 -0.1648
0.0173 0.2916 0.3099 -0.2849 -0.4886
-0.0172 0.1346 -40.2803
60.2600 -41.0300 -0.6011 -0.0636 -
0.1143 0.3133 0.3534 -0.2735 -0.4978
-0.0202 0.1268 -40.2793
60.2800 -40.1400 0.1511 0.1284 -
0.2187 0.3237 0.3906 -0.2614 -0.5068
-0.0232 0.1191 -40.2783
60.3000 -39.6700 0.6324 0.1691 -
0.2750 0.3230 0.4211 -0.2487 -0.5154
-0.0262 0.1113 -40.2773
60.3200 -41.1300 -0.6213 -0.0148 -
0.2699 0.3115 0.4448 -0.2353 -0.5237
-0.0292 0.1036 -40.2763
60.3400 -40.4500 0.0787 -0.1651 -
0.2059 0.2902 0.4614 -0.2213 -0.5318
-0.0322 0.0959 -40.2754
60.3600 -40.2600 0.2126 -0.1382 -
0.0984 0.2602 0.4714 -0.2067 -0.5395
-0.0351 0.0881 -40.2744
60.3800 -40.4600 -0.0931 0.0075
0.0265 0.2227 0.4750 -0.1916 -0.5470
-0.0381 0.0804 -40.2734
60.4000 -40.1800 -0.0456 0.1752
0.1357 0.1789 0.4725 -0.1760 -0.5542
-0.0411 0.0727 -40.2724
60.4200 -39.9200 -0.0501 0.2816
0.1989 0.1301 0.4646 -0.1598 -0.5611
-0.0441 0.0650 -40.2714
60.4400 -39.6500 0.4580 0.1747
0.2029 0.0779 0.4516 -0.1432 -0.5677
-0.0470 0.0573 -40.2704
60.4600 -40.8500 -0.3899 -0.1281
0.1593 0.0237 0.4343 -0.1263 -0.5740
-0.0500 0.0495 -40.2694
60.4800 -40.7800 0.0677 -0.2926
0.0896 -0.0308 0.4130 -0.1089 -0.5800
-0.0530 0.0418 -40.2684
60.5000 -40.7500 0.1145 -0.2102
0.0124 -0.0839 0.3884 -0.0913 -0.5857
-0.0559 0.0342 -40.2674
60.5200 -40.9300 -0.1352 -0.0275 -
0.0592 -0.1337 0.3611 -0.0734 -0.5911
-0.0588 0.0265 -40.2663
60.5400 -40.7400 0.0767 0.1200 -
0.1180 -0.1785 0.3316 -0.0553 -0.5963
-0.0618 0.0188 -40.2653

60.5600 -40.7600 0.1060 0.1872 -
0.1608 -0.2166 0.3005 -0.0371 -0.6012
-0.0647 0.0111 -40.2643
60.5800 -41.2200 -0.1810 0.1711 -
0.1829 -0.2463 0.2683 -0.0188 -0.6057
-0.0676 0.0034 -40.2633
60.6000 -41.0300 0.1063 0.0457 -
0.1747 -0.2669 0.2356 -0.0006 -0.6100
-0.0705 -0.0042 -40.2623
60.6200 -41.1700 0.1265 -0.1731 -
0.1288 -0.2787 0.2030 0.0177 -0.6140
-0.0734 -0.0119 -40.2613
60.6400 -41.6700 -0.2969 -0.2845 -
0.0476 -0.2825 0.1709 0.0358 -0.6178
-0.0763 -0.0195 -40.2603
60.6600 -41.0800 -0.0445 -0.1162
0.0566 -0.2796 0.1399 0.0537 -0.6212
-0.0791 -0.0272 -40.2593
60.6800 -40.7800 0.1753 0.1531
0.1601 -0.2715 0.1105 0.0715 -0.6244
-0.0820 -0.0348 -40.2583
60.7000 -40.3400 0.1955 0.2259
0.2367 -0.2593 0.0831 0.0889 -0.6273
-0.0849 -0.0424 -40.2573
60.7200 -40.8700 -0.1206 0.0914
0.2677 -0.2441 0.0581 0.1060 -0.6299
-0.0877 -0.0500 -40.2563
60.7400 -41.0300 -0.0310 -0.0528
0.2466 -0.2267 0.0356 0.1227 -0.6323
-0.0905 -0.0576 -40.2552
60.7600 -41.0100 0.0295 -0.0839
0.1748 -0.2071 0.0159 0.1390 -0.6344
-0.0933 -0.0652 -40.2542
60.7800 -41.0300 -0.0192 -0.0347
0.0632 -0.1851 -0.0010 0.1547 -0.6362
-0.0961 -0.0728 -40.2532
60.8000 -41.0500 0.0050 -0.0019 -
0.0678 -0.1604 -0.0152 0.1700 -0.6378
-0.0989 -0.0804 -40.2522
60.8200 -41.2400 0.1063 -0.0730 -
0.1947 -0.1328 -0.0267 0.1846 -0.6391
-0.1017 -0.0880 -40.2512
60.8400 -41.4800 0.0891 -0.1521 -
0.2948 -0.1015 -0.0358 0.1987 -0.6402
-0.1044 -0.0955 -40.2502
60.8600 -41.9600 -0.6231 -0.0444 -
0.3471 -0.0660 -0.0426 0.2122 -0.6410
-0.1071 -0.1031 -40.2491
60.8800 -40.9600 0.1500 0.1605 -
0.3352 -0.0272 -0.0474 0.2251 -0.6416
-0.1099 -0.1106 -40.2481

60.9000 -40.2500 0.7586 0.1459 -
0.2551 0.0134 -0.0503 0.2374 -0.6419
-0.1126 -0.1182 -40.2471
60.9200 -41.6600 -0.5511 -0.1738 -
0.1153 0.0541 -0.0516 0.2490 -0.6420
-0.1152 -0.1257 -40.2461
60.9400 -41.5800 -0.3851 -0.3814
0.0597 0.0933 -0.0514 0.2600 -0.6419
-0.1179 -0.1332 -40.2450
60.9600 -40.6600 0.1635 -0.2180
0.2354 0.1289 -0.0501 0.2704 -0.6415
-0.1206 -0.1407 -40.2440
60.9800 -40.1500 0.1385 0.1414
0.3756 0.1590 -0.0477 0.2802 -0.6409
-0.1232 -0.1482 -40.2430
61.0000 -39.6400 0.1890 0.4227
0.4495 0.1816 -0.0446 0.2893 -0.6401
-0.1258 -0.1557 -40.2420
61.0200 -39.9500 -0.2861 0.5568
0.4341 0.1957 -0.0409 0.2979 -0.6391
-0.1284 -0.1631 -40.2409
61.0400 -40.0500 -0.1209 0.5145
0.3280 0.2015 -0.0370 0.3059 -0.6379
-0.1309 -0.1706 -40.2399
61.0600 -39.8000 0.5408 0.2337
0.1564 0.2005 -0.0331 0.3132 -0.6365
-0.1335 -0.1781 -40.2389
61.0800 -40.8100 0.1126 -0.2520 -
0.0407 0.1944 -0.0294 0.3200 -0.6348
-0.1360 -0.1855 -40.2378
61.1000 -42.2300 -0.6053 -0.6643 -
0.2193 0.1849 -0.0261 0.3262 -0.6330
-0.1385 -0.1929 -40.2368
61.1200 -41.6900 0.0531 -0.6946 -
0.3406 0.1736 -0.0233 0.3319 -0.6310
-0.1410 -0.2003 -40.2358
61.1400 -41.4200 0.1656 -0.3633 -
0.3886 0.1620 -0.0213 0.3369 -0.6289
-0.1434 -0.2077 -40.2347
61.1600 -40.9800 0.0613 0.0922 -
0.3723 0.1509 -0.0202 0.3414 -0.6265
-0.1458 -0.2151 -40.2337
61.1800 -40.7000 -0.1855 0.4896 -
0.3116 0.1409 -0.0200 0.3454 -0.6240
-0.1482 -0.2225 -40.2327
61.2000 -39.9700 0.3569 0.6620 -
0.2267 0.1323 -0.0206 0.3488 -0.6213
-0.1506 -0.2299 -40.2316
61.2200 -40.5100 -0.0230 0.4084 -
0.1313 0.1249 -0.0220 0.3516 -0.6185
-0.1530 -0.2372 -40.2306

61.2400 -40.8900 0.2416 -0.1656 -
0.0343 0.1174 -0.0238 0.3538 -0.6154
-0.1553 -0.2446 -40.2296
61.2600 -41.7600 -0.5717 -0.5562
0.0591 0.1078 -0.0259 0.3555 -0.6122
-0.1576 -0.2519 -40.2285
61.2800 -41.3600 -0.2386 -0.4653
0.1470 0.0940 -0.0280 0.3566 -0.6089
-0.1599 -0.2592 -40.2275
61.3000 -39.9200 0.8561 -0.1351
0.2335 0.0739 -0.0299 0.3571 -0.6054
-0.1621 -0.2665 -40.2264
61.3200 -40.6300 -0.0378 0.0624
0.3181 0.0465 -0.0312 0.3570 -0.6017
-0.1643 -0.2738 -40.2254
61.3400 -40.9800 -0.6417 0.1559
0.3779 0.0117 -0.0318 0.3563 -0.5979
-0.1665 -0.2811 -40.2244
61.3600 -40.9000 -0.6428 0.3329
0.3827 -0.0299 -0.0316 0.3550 -0.5940
-0.1687 -0.2884 -40.2233
61.3800 -40.2700 -0.0620 0.4903
0.3169 -0.0768 -0.0303 0.3530 -0.5898
-0.1708 -0.2956 -40.2223
61.4000 -39.5300 1.1256 0.3095
0.1849 -0.1262 -0.0278 0.3505 -0.5856
-0.1730 -0.3029 -40.2212
61.4200 -41.8800 -0.5766 -0.2024
0.0162 -0.1747 -0.0242 0.3474 -0.5811
-0.1750 -0.3101 -40.2202
61.4400 -42.6600 -0.7425 -0.5065 -
0.1420 -0.2186 -0.0192 0.3436 -0.5765
-0.1771 -0.3173 -40.2191
61.4600 -41.4000 0.3374 -0.3168 -
0.2514 -0.2541 -0.0128 0.3392 -0.5718
-0.1791 -0.3245 -40.2181
61.4800 -41.3500 0.2517 0.0722 -
0.3046 -0.2777 -0.0051 0.3341 -0.5669
-0.1811 -0.3317 -40.2170
61.5000 -41.2300 0.1044 0.2571 -
0.3110 -0.2867 0.0040 0.3284 -0.5619
-0.1831 -0.3388 -40.2160
61.5200 -41.3700 0.0332 0.1587 -
0.2889 -0.2795 0.0144 0.3220 -0.5567
-0.1851 -0.3460 -40.2149
61.5400 -41.5300 -0.0880 -0.0167 -
0.2582 -0.2556 0.0258 0.3149 -0.5514
-0.1870 -0.3531 -40.2139
61.5600 -41.5100 -0.0485 -0.0501 -
0.2325 -0.2166 0.0382 0.3072 -0.5459
-0.1889 -0.3603 -40.2128

61.5800 -41.5000 -0.4156 0.0973 -
0.2031 -0.1655 0.0511 0.2988 -0.5403
-0.1907 -0.3674 -40.2118
61.6000 -40.5000 0.5859 0.1607 -
0.1488 -0.1065 0.0643 0.2897 -0.5345
-0.1926 -0.3745 -40.2107
61.6200 -40.6400 0.4791 -0.0566 -
0.0547 -0.0438 0.0775 0.2798 -0.5286
-0.1944 -0.3815 -40.2097
61.6400 -41.8600 -0.7356 -0.3311
0.0752 0.0185 0.0903 0.2693 -0.5225
-0.1962 -0.3886 -40.2086
61.6600 -41.3300 -0.3868 -0.2866
0.2224 0.0769 0.1024 0.2580 -0.5163
-0.1980 -0.3957 -40.2076
61.6800 -39.9000 0.5296 0.0513
0.3515 0.1282 0.1135 0.2461 -0.5099
-0.1997 -0.4027 -40.2065
61.7000 -39.9000 0.2272 0.3383
0.4242 0.1697 0.1232 0.2334 -0.5034
-0.2014 -0.4097 -40.2055
61.7200 -39.9000 0.1293 0.2996
0.4125 0.1996 0.1314 0.2201 -0.4967
-0.2031 -0.4167 -40.2044
61.7400 -40.5600 -0.1127 0.0116
0.3217 0.2177 0.1378 0.2060 -0.4899
-0.2048 -0.4237 -40.2033
61.7600 -40.9300 -0.1444 -0.2144
0.1820 0.2253 0.1422 0.1912 -0.4830
-0.2064 -0.4307 -40.2023
61.7800 -41.1700 -0.1190 -0.1802
0.0286 0.2244 0.1445 0.1758 -0.4759
-0.2080 -0.4376 -40.2012
61.8000 -41.0700 -0.1197 0.0189 -
0.1098 0.2166 0.1447 0.1597 -0.4687
-0.2096 -0.4446 -40.2002
61.8200 -40.4800 0.3939 0.1159 -
0.2127 0.2040 0.1427 0.1430 -0.4613
-0.2112 -0.4515 -40.1991
61.8400 -40.9300 0.1803 0.0088 -
0.2718 0.1886 0.1384 0.1257 -0.4538
-0.2127 -0.4584 -40.1980
61.8600 -41.7100 -0.4734 -0.1137 -
0.2945 0.1725 0.1320 0.1079 -0.4462
-0.2143 -0.4653 -40.1970
61.8800 -41.3000 0.1507 -0.0326 -
0.2911 0.1573 0.1233 0.0895 -0.4384
-0.2158 -0.4721 -40.1959
61.9000 -40.9000 0.1980 0.1470 -
0.2651 0.1439 0.1126 0.0706 -0.4305
-0.2172 -0.4790 -40.1948

61.9200 -41.1300 -0.0523 0.1588 -
0.2119 0.1320 0.0999 0.0513 -0.4224
-0.2187 -0.4858 -40.1938
61.9400 -41.3300 0.0605 -0.0400 -
0.1288 0.1207 0.0857 0.0316 -0.4142
-0.2201 -0.4927 -40.1927
61.9600 -41.4000 0.0085 -0.2422 -
0.0177 0.1085 0.0700 0.0115 -0.4059
-0.2216 -0.4995 -40.1916
61.9800 -41.4800 -0.1405 -0.2562
0.1132 0.0942 0.0533 -0.0088 -0.3975
-0.2229 -0.5062 -40.1906
62.0000 -40.9300 0.0730 -0.0509
0.2445 0.0767 0.0358 -0.0294 -0.3889
-0.2243 -0.5130 -40.1895
62.0200 -40.7000 -0.0218 0.2254
0.3456 0.0554 0.0179 -0.0502 -0.3802
-0.2257 -0.5198 -40.1884
62.0400 -40.6600 -0.0172 0.3325
0.3867 0.0298 0.0000 -0.0712 -0.3714
-0.2270 -0.5265 -40.1873
62.0600 -40.6600 0.4163 0.1452
0.3501 -0.0005 -0.0176 -0.0922 -0.3625
-0.2283 -0.5332 -40.1863
62.0800 -41.8100 -0.4138 -0.1227
0.2447 -0.0344 -0.0346 -0.1133 -0.3534
-0.2296 -0.5399 -40.1852
62.1000 -41.8100 -0.1236 -0.1650
0.0945 -0.0701 -0.0507 -0.1343 -0.3443
-0.2309 -0.5466 -40.1841
62.1200 -41.6100 0.1004 -0.0062 -
0.0664 -0.1053 -0.0657 -0.1553 -0.3350
-0.2321 -0.5533 -40.1830
62.1400 -41.5900 0.3422 0.0094 -
0.2020 -0.1378 -0.0793 -0.1762 -0.3256
-0.2333 -0.5599 -40.1820
62.1600 -42.4100 -0.2815 -0.1543 -
0.2897 -0.1653 -0.0911 -0.1970 -0.3161
-0.2345 -0.5665 -40.1809
62.1800 -42.4100 -0.1064 -0.1956 -
0.3221 -0.1860 -0.1011 -0.2175 -0.3065
-0.2357 -0.5731 -40.1798
62.2000 -42.0500 0.0805 -0.0129 -
0.3038 -0.1990 -0.1088 -0.2378 -0.2967
-0.2369 -0.5797 -40.1787
62.2200 -42.0500 -0.0786 0.1949 -
0.2455 -0.2039 -0.1142 -0.2577 -0.2869
-0.2381 -0.5863 -40.1777
62.2400 -41.6900 0.0816 0.2077 -
0.1593 -0.2018 -0.1171 -0.2773 -0.2770
-0.2392 -0.5928 -40.1766

62.2600 -41.7400 0.1910 0.0054 -
0.0580 -0.1938 -0.1174 -0.2965 -0.2670
-0.2403 -0.5994 -40.1755
62.2800 -42.1900 -0.0951 -0.2298
0.0474 -0.1816 -0.1151 -0.3152 -0.2568
-0.2414 -0.6059 -40.1744
62.3000 -42.1200 -0.1663 -0.2590
0.1440 -0.1671 -0.1102 -0.3333 -0.2466
-0.2425 -0.6124 -40.1733
62.3200 -41.5200 0.1751 -0.0508
0.2178 -0.1520 -0.1028 -0.3509 -0.2363
-0.2435 -0.6189 -40.1723
62.3400 -41.5200 -0.0798 0.2012
0.2572 -0.1374 -0.0931 -0.3679 -0.2259
-0.2446 -0.6253 -40.1712
62.3600 -41.2800 0.0242 0.2760
0.2557 -0.1238 -0.0812 -0.3842 -0.2154
-0.2456 -0.6318 -40.1701
62.3800 -41.2800 0.1887 0.1168
0.2142 -0.1112 -0.0675 -0.3998 -0.2048
-0.2466 -0.6382 -40.1690
62.4000 -42.0800 -0.2232 -0.0795
0.1396 -0.0993 -0.0521 -0.4147 -0.1941
-0.2476 -0.6446 -40.1679
62.4200 -42.1700 -0.3377 -0.0964
0.0431 -0.0875 -0.0355 -0.4288 -0.1833
-0.2486 -0.6510 -40.1668
62.4400 -41.5000 0.2454 0.0333 -
0.0570 -0.0750 -0.0180 -0.4421 -0.1725
-0.2496 -0.6573 -40.1657
62.4600 -41.4200 0.5187 0.0502 -
0.1445 -0.0604 0.0001 -0.4545 -0.1616
-0.2505 -0.6637 -40.1647
62.4800 -42.4000 -0.3374 -0.1052 -
0.2067 -0.0425 0.0184 -0.4661 -0.1506
-0.2515 -0.6700 -40.1636
62.5000 -42.6500 -0.5455 -0.1399 -
0.2352 -0.0206 0.0366 -0.4767 -0.1395
-0.2524 -0.6763 -40.1625
62.5200 -41.6800 0.2817 0.0410 -
0.2280 0.0053 0.0542 -0.4864 -0.1283
-0.2533 -0.6826 -40.1614
62.5400 -41.2700 0.2949 0.1692 -
0.1901 0.0344 0.0710 -0.4951 -0.1171
-0.2542 -0.6888 -40.1603
62.5600 -41.6200 -0.0101 0.0685 -
0.1265 0.0656 0.0866 -0.5027 -0.1058
-0.2550 -0.6951 -40.1592
62.5800 -41.9000 -0.1795 -0.1279 -
0.0447 0.0970 0.1007 -0.5093 -0.0945
-0.2559 -0.7013 -40.1581

62.6000 -41.7100 -0.1027 -0.2015
0.0438 0.1264 0.1130 -0.5149 -0.0831
-0.2567 -0.7075 -40.1570
62.6200 -41.3400 0.1222 -0.0641
0.1259 0.1515 0.1233 -0.5192 -0.0716
-0.2576 -0.7137 -40.1559
62.6400 -41.2200 -0.1012 0.1502
0.1873 0.1698 0.1311 -0.5225 -0.0600
-0.2584 -0.7198 -40.1548
62.6600 -40.7900 0.0782 0.2470
0.2153 0.1797 0.1365 -0.5245 -0.0485
-0.2592 -0.7260 -40.1537
62.6800 -40.9000 0.0851 0.1502
0.2039 0.1797 0.1390 -0.5253 -0.0368
-0.2600 -0.7321 -40.1526
62.7000 -41.3200 0.0162 -0.0371
0.1602 0.1697 0.1387 -0.5249 -0.0252
-0.2607 -0.7382 -40.1515
62.7200 -41.4000 -0.0087 -0.1963
0.0983 0.1506 0.1356 -0.5233 -0.0134
-0.2615 -0.7443 -40.1504
62.7400 -41.7800 -0.1157 -0.2422
0.0327 0.1235 0.1297 -0.5204 -0.0017
-0.2622 -0.7503 -40.1493
62.7600 -41.7400 -0.0953 -0.1345 -
0.0250 0.0899 0.1212 -0.5162 0.0101
-0.2630 -0.7564 -40.1482
62.7800 -41.3900 0.1680 0.0754 -
0.0682 0.0515 0.1103 -0.5108 0.0219
-0.2637 -0.7624 -40.1471
62.8000 -41.3900 0.0640 0.2358 -
0.0926 0.0101 0.0971 -0.5041 0.0337
-0.2644 -0.7684 -40.1460
62.8200 -41.4200 -0.0129 0.2103 -
0.0963 -0.0323 0.0819 -0.4962 0.0456
-0.2651 -0.7743 -40.1449
62.8400 -41.5100 0.2467 -0.0285 -
0.0814 -0.0734 0.0649 -0.4870 0.0574
-0.2658 -0.7803 -40.1438
62.8600 -42.3300 -0.2375 -0.2911 -
0.0509 -0.1112 0.0464 -0.4766 0.0692
-0.2665 -0.7862 -40.1427
62.8800 -42.2300 -0.2712 -0.2939 -
0.0104 -0.1441 0.0265 -0.4650 0.0811
-0.2671 -0.7921 -40.1416
62.9000 -41.4400 0.2497 -0.0129
0.0292 -0.1705 0.0057 -0.4523 0.0929
-0.2678 -0.7980 -40.1405
62.9200 -41.4100 0.0007 0.2968
0.0543 -0.1891 -0.0159 -0.4384 0.1047
-0.2684 -0.8039 -40.1394

62.9400 −41.2700 0.0771 0.4026
0.0528 −0.1990 −0.0379 −0.4234 0.1165
−0.2690 −0.8097 −40.1383
62.9600 −41.4300 0.1266 0.2332
0.0239 −0.1996 −0.0603 −0.4074 0.1283
−0.2696 −0.8155 −40.1372
62.9800 −42.0500 −0.1636 −0.1176 −
0.0195 −0.1905 −0.0826 −0.3903 0.1401
−0.2702 −0.8213 −40.1361
63.0000 −42.0800 0.0149 −0.3962 −
0.0552 −0.1720 −0.1048 −0.3722 0.1518
−0.2708 −0.8271 −40.1350
63.0200 −42.1800 0.0076 −0.4028 −
0.0624 −0.1445 −0.1267 −0.3532 0.1634
−0.2714 −0.8328 −40.1339
63.0400 −42.0300 −0.0810 −0.2053 −
0.0344 −0.1089 −0.1481 −0.3333 0.1750
−0.2719 −0.8386 −40.1328
63.0600 −41.4200 0.1757 0.0232
0.0183 −0.0665 −0.1689 −0.3125 0.1866
−0.2725 −0.8443 −40.1317
63.0800 −41.4000 −0.0747 0.2208
0.0704 −0.0191 −0.1889 −0.2909 0.1981
−0.2730 −0.8500 −40.1305
63.1000 −41.3100 −0.1824 0.3984
0.0897 0.0316 −0.2080 −0.2685 0.2096
−0.2736 −0.8556 −40.1294
63.1200 −41.0700 −0.1492 0.4574
0.0565 0.0837 −0.2261 −0.2454 0.2209
−0.2741 −0.8613 −40.1283
63.1400 −40.5100 0.6322 0.2223 −
0.0214 0.1353 −0.2431 −0.2215 0.2322
−0.2746 −0.8669 −40.1272
63.1600 −41.4700 0.1418 −0.2472 −
0.1116 0.1846 −0.2589 −0.1970 0.2434
−0.2751 −0.8725 −40.1261
63.1800 −42.9200 −0.8835 −0.5383 −
0.1679 0.2299 −0.2734 −0.1719 0.2546
−0.2756 −0.8780 −40.1250
63.2000 −41.7400 −0.1033 −0.3238 −
0.1535 0.2699 −0.2864 −0.1462 0.2656
−0.2760 −0.8836 −40.1239
63.2200 −40.5700 0.6164 0.1013 −
0.0753 0.3030 −0.2978 −0.1200 0.2766
−0.2765 −0.8891 −40.1227
63.2400 −40.7200 −0.0210 0.3039
0.0400 0.3282 −0.3074 −0.0934 0.2874
−0.2770 −0.8946 −40.1216
63.2600 −40.8000 −0.0794 0.1792
0.1567 0.3448 −0.3151 −0.0663 0.2981
−0.2774 −0.9001 −40.1205

63.2800 −40.7300 0.0240 −0.0423
0.2320 0.3523 −0.3208 −0.0388 0.3087
−0.2778 −0.9055 −40.1194
63.3000 −40.7700 0.1348 −0.1610
0.2331 0.3504 −0.3242 −0.0111 0.3192
−0.2783 −0.9109 −40.1183
63.3200 −41.0500 −0.2382 −0.0463
0.1492 0.3392 −0.3252 0.0169 0.3296
−0.2787 −0.9163 −40.1172
63.3400 −40.7000 −0.0302 0.2600 −
0.0017 0.3188 −0.3237 0.0451 0.3398
−0.2791 −0.9217 −40.1160
63.3600 −40.3000 0.3010 0.4386 −
0.1820 0.2899 −0.3196 0.0733 0.3500
−0.2795 −0.9271 −40.1149
63.3800 −41.1700 −0.0799 0.1772 −
0.3468 0.2531 −0.3127 0.1016 0.3599
−0.2798 −0.9324 −40.1138
63.4000 −41.5700 0.1058 −0.3306 −
0.4495 0.2088 −0.3028 0.1299 0.3697
−0.2802 −0.9377 −40.1127
63.4200 −42.2700 −0.3676 −0.5855 −
0.4459 0.1575 −0.2898 0.1580 0.3794
−0.2806 −0.9430 −40.1115
63.4400 −41.6000 0.0389 −0.3942 −
0.3128 0.0999 −0.2735 0.1859 0.3889
−0.2809 −0.9482 −40.1104
63.4600 −40.9300 0.1187 0.0179 −
0.0858 0.0363 −0.2537 0.2135 0.3983
−0.2813 −0.9535 −40.1093
63.4800 −40.4600 0.0370 0.3484
0.1760 −0.0330 −0.2304 0.2407 0.4074
−0.2816 −0.9587 −40.1082
63.5000 −40.2800 −0.1610 0.4555
0.4109 −0.1076 −0.2035 0.2675 0.4164
−0.2820 −0.9639 −40.1071
63.5200 −39.8500 0.2146 0.3185
0.5699 −0.1871 −0.1728 0.2938 0.4253
−0.2823 −0.9690 −40.1059
63.5400 −40.3800 −0.0574 0.0209
0.6320 −0.2709 −0.1385 0.3195 0.4339
−0.2826 −0.9742 −40.1048
63.5600 −40.6400 0.0016 −0.2389
0.6009 −0.3573 −0.1007 0.3445 0.4423
−0.2829 −0.9793 −40.1037
63.5800 −40.9900 −0.1156 −0.2634
0.4919 −0.4443 −0.0597 0.3687 0.4506
−0.2832 −0.9843 −40.1025
63.6000 −40.8200 0.0264 −0.0807
0.3305 −0.5292 −0.0159 0.3922 0.4586
−0.2835 −0.9894 −40.1014

63.6200 -40.5900 0.2207 0.0730
0.1524 -0.6085 0.0303 0.4147 0.4665
-0.2838 -0.9944 -40.1003
63.6400 -41.1100 0.0340 0.0090 -
0.0175 -0.6776 0.0784 0.4364 0.4741
-0.2841 -0.9994 -40.0992
63.6600 -41.4000 -0.0379 -0.1806 -
0.1786 -0.7304 0.1280 0.4570 0.4815
-0.2843 -1.0044 -40.0980
63.6800 -41.7700 -0.2613 -0.2190 -
0.3415 -0.7599 0.1785 0.4765 0.4887
-0.2846 -1.0094 -40.0969
63.7000 -41.5500 -0.0856 0.0341 -
0.5138 -0.7598 0.2294 0.4949 0.4957
-0.2849 -1.0143 -40.0958
63.7200 -40.7900 0.2816 0.3899 -
0.6831 -0.7258 0.2804 0.5122 0.5024
-0.2851 -1.0192 -40.0946
63.7400 -40.8900 0.1059 0.5159 -
0.8242 -0.6555 0.3308 0.5281 0.5089
-0.2854 -1.0241 -40.0935
63.7600 -40.9700 0.0909 0.2218 -
0.9059 -0.5478 0.3799 0.5427 0.5152
-0.2856 -1.0289 -40.0924
63.7800 -41.3100 0.3072 -0.3824 -
0.8921 -0.4042 0.4270 0.5560 0.5212
-0.2858 -1.0338 -40.0912
63.8000 -41.8000 -0.1202 -0.8285 -
0.7537 -0.2293 0.4714 0.5678 0.5269
-0.2861 -1.0386 -40.0901
63.8200 -41.7200 -0.8626 -0.6216 -
0.4835 -0.0317 0.5121 0.5781 0.5324
-0.2863 -1.0433 -40.0890
63.8400 -38.7500 0.7935 0.1916 -
0.1359 0.1781 0.5485 0.5868 0.5376
-0.2865 -1.0481 -40.0878
63.8600 -38.4400 -0.0693 0.7539
0.2102 0.3894 0.5797 0.5939 0.5426
-0.2867 -1.0528 -40.0867
63.8800 -37.9400 0.0430 0.6270
0.4826 0.5914 0.6049 0.5993 0.5473
-0.2870 -1.0575 -40.0856
63.9000 -38.0900 -0.0590 0.1697
0.6502 0.7734 0.6233 0.6029 0.5517
-0.2872 -1.0621 -40.0844
63.9200 -38.4600 -0.2806 -0.1855
0.7161 0.9248 0.6341 0.6049 0.5559
-0.2874 -1.0668 -40.0833
63.9400 -37.8500 0.3126 -0.3362
0.7143 1.0358 0.6364 0.6050 0.5597
-0.2876 -1.0714 -40.0821

63.9600 -38.3000 -0.0827 -0.4087
0.7009 1.0975 0.6295 0.6033 0.5633
-0.2878 -1.0760 -40.0810
63.9800 -38.3100 -0.0450 -0.4265
0.7160 1.1034 0.6127 0.5999 0.5666
-0.2880 -1.0805 -40.0799
64.0000 -38.1100 0.0580 -0.2995
0.7571 1.0532 0.5861 0.5948 0.5697
-0.2881 -1.0851 -40.0787
64.0200 -38.2300 -0.2730 0.0223
0.7838 0.9528 0.5505 0.5881 0.5724
-0.2883 -1.0896 -40.0776
64.0400 -37.7100 0.1195 0.3851
0.7452 0.8114 0.5069 0.5797 0.5749
-0.2885 -1.0940 -40.0764
64.0600 -37.8800 0.1654 0.5029
0.6017 0.6391 0.4563 0.5699 0.5772
-0.2887 -1.0985 -40.0753
64.0800 -38.9100 -0.0495 0.3115
0.3494 0.4458 0.3997 0.5586 0.5791
-0.2889 -1.1029 -40.0741
64.1000 -39.8700 -0.1694 -0.0049
0.0276 0.2409 0.3380 0.5460 0.5809
-0.2891 -1.1073 -40.0730
64.1200 -40.2900 0.3358 -0.2858 -
0.3065 0.0332 0.2722 0.5321 0.5823
-0.2892 -1.1117 -40.0719
64.1400 -41.6300 -0.3164 -0.3703 -
0.5996 -0.1687 0.2035 0.5170 0.5835
-0.2894 -1.1160 -40.0707
64.1600 -41.8200 -0.2235 -0.1790 -
0.8135 -0.3562 0.1327 0.5009 0.5845
-0.2896 -1.1203 -40.0696
64.1800 -41.3100 0.4731 0.0546 -
0.9269 -0.5208 0.0608 0.4837 0.5853
-0.2897 -1.1246 -40.0684
64.2000 -42.0000 0.0665 0.0636 -
0.9358 -0.6558 -0.0111 0.4655 0.5858
-0.2899 -1.1289 -40.0673
64.2200 -42.5700 -0.3902 -0.0630 -
0.8505 -0.7575 -0.0821 0.4464 0.5860
-0.2901 -1.1331 -40.0661
64.2400 -42.3200 -0.0295 -0.1191 -
0.6884 -0.8261 -0.1511 0.4266 0.5861
-0.2902 -1.1373 -40.0650
64.2600 -41.8700 0.1810 -0.0733 -
0.4718 -0.8638 -0.2174 0.4060 0.5859
-0.2904 -1.1415 -40.0638
64.2800 -41.8300 0.0764 -0.0121 -
0.2251 -0.8739 -0.2800 0.3848 0.5856
-0.2906 -1.1456 -40.0627

64.3000 -41.8200 -0.1632 0.0146
0.0281 -0.8600 -0.3382 0.3631 0.5850
-0.2907 -1.1497 -40.0615
64.3200 -41.4900 0.1237 0.0100
0.2631 -0.8255 -0.3915 0.3408 0.5842
-0.2909 -1.1538 -40.0604
64.3400 -41.3900 -0.0550 -0.0135
0.4516 -0.7737 -0.4393 0.3181 0.5832
-0.2911 -1.1579 -40.0592
64.3600 -41.2200 -0.0092 0.0030
0.5623 -0.7073 -0.4812 0.2952 0.5820
-0.2912 -1.1619 -40.0581
64.3800 -41.1000 -0.0788 0.1425
0.5734 -0.6289 -0.5168 0.2719 0.5806
-0.2914 -1.1659 -40.0569
64.4000 -41.0300 -0.1028 0.3428
0.4806 -0.5410 -0.5458 0.2485 0.5790
-0.2916 -1.1698 -40.0558
64.4200 -40.9000 0.1513 0.3790
0.3016 -0.4469 -0.5682 0.2250 0.5773
-0.2917 -1.1738 -40.0546
64.4400 -41.0900 0.3731 0.0926
0.0772 -0.3502 -0.5838 0.2015 0.5753
-0.2919 -1.1777 -40.0535
64.4600 -42.6200 -0.4754 -0.3222 -
0.1396 -0.2548 -0.5926 0.1780 0.5732
-0.2921 -1.1816 -40.0523
64.4800 -42.6200 -0.2621 -0.4852 -
0.2952 -0.1642 -0.5947 0.1546 0.5709
-0.2922 -1.1854 -40.0512
64.5000 -41.8700 0.2901 -0.3124 -
0.3557 -0.0818 -0.5902 0.1313 0.5685
-0.2924 -1.1893 -40.0500
64.5200 -41.6500 0.0382 -0.0168 -
0.3207 -0.0098 -0.5791 0.1082 0.5659
-0.2926 -1.1931 -40.0489
64.5400 -41.4500 0.0068 0.1758 -
0.2143 0.0514 -0.5618 0.0854 0.5632
-0.2927 -1.1968 -40.0477
64.5600 -41.1900 -0.0099 0.2104 -
0.0783 0.1028 -0.5386 0.0628 0.5603
-0.2929 -1.2006 -40.0465
64.5800 -41.1500 -0.0578 0.1952
0.0400 0.1453 -0.5099 0.0404 0.5572
-0.2931 -1.2043 -40.0454
64.6000 -40.8200 0.0512 0.2357
0.1089 0.1804 -0.4761 0.0183 0.5541
-0.2933 -1.2080 -40.0442
64.6200 -40.8200 0.0035 0.2867
0.1174 0.2092 -0.4379 -0.0034 0.5507
-0.2934 -1.2116 -40.0431

64.6400 -40.9600 -0.0728 0.2139
0.0732 0.2329 -0.3959 -0.0249 0.5473
-0.2936 -1.2152 -40.0419
64.6600 -41.0000 0.1553 -0.0160
0.0003 0.2523 -0.3507 -0.0459 0.5438
-0.2938 -1.2188 -40.0408
64.6800 -41.4600 0.0542 -0.3161 -
0.0681 0.2679 -0.3029 -0.0667 0.5401
-0.2940 -1.2224 -40.0396
64.7000 -41.9200 -0.2076 -0.4871 -
0.1039 0.2801 -0.2531 -0.0870 0.5363
-0.2942 -1.2259 -40.0384
64.7200 -41.7100 -0.0666 -0.4007 -
0.0937 0.2887 -0.2019 -0.1069 0.5324
-0.2944 -1.2294 -40.0373
64.7400 -40.9700 0.2413 -0.1372 -
0.0425 0.2932 -0.1499 -0.1264 0.5284
-0.2945 -1.2329 -40.0361
64.7600 -40.9700 -0.2114 0.1690
0.0297 0.2926 -0.0977 -0.1455 0.5243
-0.2947 -1.2363 -40.0349
64.7800 -40.2900 0.1109 0.4487
0.0987 0.2862 -0.0459 -0.1640 0.5201
-0.2949 -1.2398 -40.0338
64.8000 -40.2900 -0.0510 0.5988
0.1476 0.2737 0.0050 -0.1821 0.5159
-0.2951 -1.2431 -40.0326
64.8200 -40.3600 -0.0368 0.4460
0.1700 0.2552 0.0544 -0.1997 0.5115
-0.2953 -1.2465 -40.0315
64.8400 -40.3700 0.4800 -0.0578
0.1667 0.2318 0.1018 -0.2168 0.5071
-0.2955 -1.2498 -40.0303
64.8600 -41.7700 -0.4255 -0.5988
0.1424 0.2048 0.1466 -0.2334 0.5026
-0.2957 -1.2531 -40.0291
64.8800 -41.9500 -0.4237 -0.7029
0.1027 0.1761 0.1883 -0.2494 0.4981
-0.2960 -1.2564 -40.0280
64.9000 -40.8000 0.3185 -0.2448
0.0523 0.1480 0.2266 -0.2648 0.4935
-0.2962 -1.2596 -40.0268
64.9200 -40.5100 0.0652 0.3885 -
0.0105 0.1229 0.2610 -0.2797 0.4888
-0.2964 -1.2628 -40.0256
64.9400 -40.2800 0.1248 0.6753 -
0.0886 0.1031 0.2912 -0.2940 0.4841
-0.2966 -1.2660 -40.0245
64.9600 -40.2200 0.5578 0.4048 -
0.1784 0.0901 0.3170 -0.3078 0.4794
-0.2968 -1.2692 -40.0233

64.9800 -41.8300 -0.4562 -0.1499 -
0.2597 0.0847 0.3382 -0.3209 0.4746
-0.2970 -1.2723 -40.0221
65.0000 -42.0100 -0.2592 -0.4399 -
0.3081 0.0870 0.3549 -0.3335 0.4698
-0.2973 -1.2754 -40.0210
65.0200 -41.5900 0.0768 -0.3154 -
0.3031 0.0964 0.3669 -0.3455 0.4649
-0.2975 -1.2784 -40.0198
65.0400 -40.9700 0.3560 -0.1246 -
0.2364 0.1118 0.3743 -0.3570 0.4601
-0.2977 -1.2814 -40.0186
65.0600 -41.2200 0.0534 -0.1077 -
0.1097 0.1316 0.3773 -0.3678 0.4552
-0.2980 -1.2844 -40.0175
65.0800 -41.5200 -0.4158 -0.1307
0.0569 0.1536 0.3759 -0.3781 0.4503
-0.2982 -1.2874 -40.0163
65.1000 -40.8100 0.0341 -0.0244
0.2325 0.1756 0.3704 -0.3878 0.4454
-0.2985 -1.2904 -40.0151
65.1200 -40.2200 0.2861 0.1170
0.3885 0.1954 0.3612 -0.3970 0.4405
-0.2987 -1.2933 -40.0139
65.1400 -40.4500 -0.0004 0.2021
0.4949 0.2104 0.3484 -0.4055 0.4356
-0.2990 -1.2961 -40.0128
65.1600 -40.5500 -0.3582 0.2859
0.5229 0.2186 0.3324 -0.4136 0.4307
-0.2992 -1.2990 -40.0116
65.1800 -40.2900 0.0152 0.3253
0.4550 0.2186 0.3135 -0.4211 0.4259
-0.2995 -1.3018 -40.0104
65.2000 -40.1800 0.4588 0.2006
0.2990 0.2105 0.2921 -0.4280 0.4210
-0.2997 -1.3046 -40.0093
65.2200 -41.3600 -0.2626 -0.0458
0.0833 0.1951 0.2684 -0.4344 0.4162
-0.3000 -1.3074 -40.0081
65.2400 -41.8600 -0.3387 -0.1620 -
0.1538 0.1734 0.2430 -0.4402 0.4114
-0.3003 -1.3101 -40.0069
65.2600 -41.5800 0.0796 -0.0375 -
0.3719 0.1462 0.2160 -0.4455 0.4066
-0.3005 -1.3128 -40.0057
65.2800 -41.4400 0.4201 0.0453 -
0.5313 0.1144 0.1880 -0.4503 0.4019
-0.3008 -1.3155 -40.0046
65.3000 -42.2000 -0.0490 -0.1322 -
0.6025 0.0787 0.1591 -0.4546 0.3971
-0.3011 -1.3181 -40.0034

65.3200 -42.7000 -0.2848 -0.3610 -
0.5715 0.0403 0.1298 -0.4584 0.3925
-0.3013 -1.3208 -40.0022
65.3400 -42.4000 -0.0523 -0.3668 -
0.4376 0.0001 0.1005 -0.4616 0.3879
-0.3016 -1.3233 -40.0010
65.3600 -42.0300 0.1008 -0.1876 -
0.2195 -0.0406 0.0713 -0.4644 0.3833
-0.3019 -1.3259 -39.9999
65.3800 -41.7800 -0.0583 0.0036
0.0463 -0.0804 0.0426 -0.4667 0.3788
-0.3022 -1.3284 -39.9987
65.4000 -41.2900 0.0196 0.1665
0.3114 -0.1181 0.0148 -0.4684 0.3743
-0.3025 -1.3309 -39.9975
65.4200 -41.2400 -0.0833 0.3201
0.5252 -0.1525 -0.0120 -0.4697 0.3699
-0.3027 -1.3334 -39.9963
65.4400 -40.8200 0.0432 0.3797
0.6471 -0.1823 -0.0376 -0.4706 0.3655
-0.3030 -1.3358 -39.9951
65.4600 -40.8200 0.3662 0.2276
0.6564 -0.2069 -0.0616 -0.4710 0.3612
-0.3033 -1.3382 -39.9940
65.4800 -41.8700 -0.3522 -0.0473
0.5539 -0.2254 -0.0839 -0.4709 0.3569
-0.3036 -1.3406 -39.9928
65.5000 -42.1700 -0.3746 -0.1111
0.3557 -0.2371 -0.1045 -0.4704 0.3527
-0.3039 -1.3430 -39.9916
65.5200 -41.8900 0.0203 0.1174
0.0934 -0.2411 -0.1231 -0.4694 0.3486
-0.3042 -1.3453 -39.9904
65.5400 -41.6100 0.4254 0.2708 -
0.1841 -0.2367 -0.1397 -0.4679 0.3445
-0.3045 -1.3476 -39.9893
65.5600 -42.0600 0.5634 0.0411 -
0.4256 -0.2230 -0.1543 -0.4661 0.3405
-0.3048 -1.3499 -39.9881
65.5800 -43.6400 -0.6349 -0.3372 -
0.5894 -0.1991 -0.1669 -0.4637 0.3365
-0.3050 -1.3521 -39.9869
65.6000 -43.6400 -0.3953 -0.4143 -
0.6421 -0.1643 -0.1775 -0.4609 0.3326
-0.3053 -1.3543 -39.9857
65.6200 -42.3800 0.4425 -0.1706 -
0.5758 -0.1190 -0.1864 -0.4576 0.3287
-0.3056 -1.3565 -39.9845
65.6400 -42.2400 0.1174 0.0726 -
0.4176 -0.0651 -0.1936 -0.4538 0.3249
-0.3059 -1.3587 -39.9833

65.6600 -42.0600 -0.0021 0.1038 -
0.2068 -0.0058 -0.1993 -0.4495 0.3212
-0.3062 -1.3608 -39.9822
65.6800 -41.9700 -0.0828 0.0647
0.0127 0.0556 -0.2036 -0.4446 0.3175
-0.3065 -1.3629 -39.9810
65.7000 -41.7400 -0.2540 0.1266
0.2023 0.1155 -0.2067 -0.4393 0.3138
-0.3067 -1.3650 -39.9798
65.7200 -41.0300 0.2872 0.1934
0.3389 0.1702 -0.2088 -0.4333 0.3102
-0.3070 -1.3670 -39.9786
65.7400 -41.0300 0.2450 0.0778
0.4135 0.2162 -0.2100 -0.4269 0.3067
-0.3073 -1.3690 -39.9774
65.7600 -41.7000 -0.3358 -0.1457
0.4265 0.2500 -0.2105 -0.4198 0.3032
-0.3075 -1.3710 -39.9762
65.7800 -41.7000 -0.1354 -0.1621
0.3835 0.2698 -0.2104 -0.4121 0.2997
-0.3078 -1.3730 -39.9751
65.8000 -41.3000 0.0402 0.0790
0.2977 0.2748 -0.2098 -0.4039 0.2963
-0.3081 -1.3749 -39.9739
65.8200 -41.0600 0.2343 0.2787
0.1895 0.2654 -0.2087 -0.3950 0.2929
-0.3083 -1.3768 -39.9727
65.8400 -41.4100 0.0110 0.2138
0.0799 0.2426 -0.2071 -0.3855 0.2895
-0.3085 -1.3787 -39.9715
65.8600 -42.0000 -0.1851 -0.0554 -
0.0139 0.2075 -0.2051 -0.3754 0.2862
-0.3088 -1.3805 -39.9703
65.8800 -42.0000 0.2224 -0.3455 -
0.0789 0.1622 -0.2024 -0.3648 0.2829
-0.3090 -1.3823 -39.9691
65.9000 -42.5900 -0.1274 -0.4566 -
0.1087 0.1094 -0.1990 -0.3535 0.2797
-0.3092 -1.3841 -39.9679
65.9200 -42.5900 -0.3470 -0.2198 -
0.1111 0.0528 -0.1949 -0.3417 0.2765
-0.3094 -1.3859 -39.9667
65.9400 -41.8100 0.0405 0.2633 -
0.1046 -0.0033 -0.1900 -0.3293 0.2732
-0.3096 -1.3876 -39.9656
65.9600 -41.2300 0.2381 0.5751 -
0.1100 -0.0550 -0.1842 -0.3164 0.2701
-0.3098 -1.3893 -39.9644
65.9800 -41.2300 0.5252 0.3981 -
0.1412 -0.0994 -0.1774 -0.3030 0.2669
-0.3100 -1.3910 -39.9632

66.0000 -42.8000 -0.5331 -0.0944 -
0.1828 -0.1353 -0.1695 -0.2892 0.2637
-0.3102 -1.3927 -39.9620
66.0200 -42.8000 -0.1111 -0.4414 -
0.2076 -0.1625 -0.1606 -0.2749 0.2606
-0.3103 -1.3943 -39.9608
66.0400 -42.4800 0.1201 -0.4447 -
0.1913 -0.1809 -0.1505 -0.2602 0.2575
-0.3105 -1.3959 -39.9596
66.0600 -42.3200 0.0550 -0.2454 -
0.1243 -0.1904 -0.1393 -0.2452 0.2543
-0.3106 -1.3975 -39.9584
66.0800 -42.0700 -0.0201 -0.0447 -
0.0161 -0.1916 -0.1269 -0.2298 0.2512
-0.3107 -1.3990 -39.9572
66.1000 -41.6500 0.1637 0.0899
0.1074 -0.1856 -0.1133 -0.2141 0.2481
-0.3108 -1.4006 -39.9560
66.1200 -41.5900 -0.1275 0.1782
0.2135 -0.1735 -0.0987 -0.1981 0.2449
-0.3109 -1.4021 -39.9548
66.1400 -41.2000 0.0279 0.2294
0.2671 -0.1563 -0.0830 -0.1818 0.2418
-0.3110 -1.4035 -39.9536
66.1600 -41.2000 0.0059 0.2694
0.2461 -0.1354 -0.0663 -0.1654 0.2387
-0.3110 -1.4050 -39.9525
66.1800 -41.3400 -0.0791 0.3105
0.1512 -0.1118 -0.0488 -0.1487 0.2355
-0.3110 -1.4064 -39.9513
66.2000 -41.3400 0.1488 0.2709
0.0030 -0.0864 -0.0305 -0.1319 0.2323
-0.3111 -1.4078 -39.9501
66.2200 -41.7600 -0.0897 0.0745 -
0.1617 -0.0596 -0.0117 -0.1150 0.2291
-0.3111 -1.4091 -39.9489
66.2400 -41.8800 0.2074 -0.2550 -
0.2999 -0.0319 0.0076 -0.0980 0.2259
-0.3110 -1.4105 -39.9477
66.2600 -42.3800 -0.0705 -0.5286 -
0.3715 -0.0039 0.0270 -0.0810 0.2226
-0.3110 -1.4118 -39.9465
66.2800 -42.7800 -0.4500 -0.4972 -
0.3518 0.0235 0.0465 -0.0639 0.2193
-0.3109 -1.4131 -39.9453
66.3000 -41.7300 0.0159 -0.0809 -
0.2476 0.0494 0.0657 -0.0469 0.2160
-0.3108 -1.4143 -39.9441
66.3200 -40.4800 0.5773 0.3659 -
0.0900 0.0722 0.0844 -0.0299 0.2127
-0.3107 -1.4156 -39.9429

66.3400 -40.7600 0.0549 0.4117
0.0853 0.0904 0.1025 -0.0130 0.2093
-0.3106 -1.4168 -39.9417
66.3600 -41.0300 -0.0937 0.0833
0.2429 0.1026 0.1198 0.0038 0.2058
-0.3105 -1.4179 -39.9405
66.3800 -41.3700 -0.2333 -0.2091
0.3478 0.1077 0.1359 0.0204 0.2023
-0.3103 -1.4191 -39.9393
66.4000 -41.6000 -0.6641 -0.0945
0.3746 0.1048 0.1508 0.0369 0.1988
-0.3101 -1.4202 -39.9381
66.4200 -40.3800 0.1822 0.2798
0.3247 0.0941 0.1642 0.0531 0.1952
-0.3099 -1.4213 -39.9369
66.4400 -39.5700 0.9211 0.4501
0.2132 0.0775 0.1759 0.0691 0.1915
-0.3096 -1.4224 -39.9357
66.4600 -40.9400 0.0841 0.1311
0.0640 0.0573 0.1857 0.0848 0.1878
-0.3094 -1.4235 -39.9345
66.4800 -42.3100 -0.7414 -0.3047 -
0.0856 0.0363 0.1933 0.1002 0.1840
-0.3090 -1.4245 -39.9333
66.5000 -41.8700 -0.1221 -0.4144 -
0.1988 0.0173 0.1987 0.1152 0.1802
-0.3087 -1.4255 -39.9321
66.5200 -41.3500 0.1860 -0.2130 -
0.2518 0.0028 0.2015 0.1299 0.1763
-0.3084 -1.4265 -39.9309
66.5400 -41.2300 0.0816 0.0075 -
0.2413 -0.0054 0.2018 0.1441 0.1723
-0.3080 -1.4274 -39.9297
66.5600 -41.1200 0.1375 0.0438 -
0.1780 -0.0061 0.1994 0.1579 0.1682
-0.3076 -1.4283 -39.9285
66.5800 -41.2100 0.0179 0.0027 -
0.0923 0.0012 0.1943 0.1712 0.1640
-0.3071 -1.4293 -39.9273
66.6000 -41.3100 -0.3531 0.0908 -
0.0184 0.0163 0.1867 0.1840 0.1598
-0.3066 -1.4301 -39.9261
66.6200 -40.6900 0.2463 0.2683
0.0238 0.0388 0.1767 0.1963 0.1554
-0.3061 -1.4310 -39.9249
66.6400 -40.2700 0.4254 0.2670
0.0278 0.0672 0.1645 0.2081 0.1510
-0.3056 -1.4318 -39.9237
66.6600 -41.1400 -0.0791 0.0309 -
0.0007 0.0991 0.1505 0.2194 0.1465
-0.3050 -1.4326 -39.9225

66.6800 -41.6300 -0.4348 -0.1718 -
0.0439 0.1313 0.1349 0.2301 0.1418
-0.3044 -1.4334 -39.9213
66.7000 -41.0700 0.1800 -0.1708 -
0.0783 0.1600 0.1180 0.2403 0.1371
-0.3038 -1.4342 -39.9201
66.7200 -40.9300 0.2598 -0.0990 -
0.0803 0.1812 0.1002 0.2500 0.1322
-0.3031 -1.4349 -39.9189
66.7400 -41.2500 -0.0544 -0.1262 -
0.0351 0.1916 0.0817 0.2591 0.1273
-0.3024 -1.4356 -39.9177
66.7600 -41.2900 -0.2659 -0.1480
0.0506 0.1892 0.0629 0.2677 0.1222
-0.3016 -1.4363 -39.9165
66.7800 -40.7800 0.1779 -0.0438
0.1558 0.1732 0.0439 0.2757 0.1170
-0.3008 -1.4369 -39.9153
66.8000 -40.7800 -0.0090 0.1200
0.2524 0.1439 0.0252 0.2832 0.1117
-0.3000 -1.4376 -39.9141
66.8200 -40.5700 0.0158 0.2272
0.3102 0.1021 0.0069 0.2902 0.1063
-0.2992 -1.4382 -39.9129
66.8400 -40.5500 0.1087 0.2425
0.3059 0.0495 -0.0106 0.2965 0.1007
-0.2983 -1.4388 -39.9117
66.8600 -40.9500 -0.1200 0.1640
0.2371 -0.0111 -0.0271 0.3024 0.0950
-0.2973 -1.4393 -39.9105
66.8800 -41.0500 0.1976 -0.0135
0.1214 -0.0761 -0.0421 0.3076 0.0892
-0.2964 -1.4399 -39.9093
66.9000 -41.7400 -0.1680 -0.2386 -
0.0126 -0.1421 -0.0555 0.3123 0.0832
-0.2953 -1.4404 -39.9081
66.9200 -42.0400 0.0434 -0.3290 -
0.1366 -0.2053 -0.0668 0.3165 0.0772
-0.2943 -1.4409 -39.9069
66.9400 -42.0000 0.0928 -0.1895 -
0.2311 -0.2617 -0.0758 0.3201 0.0709
-0.2932 -1.4413 -39.9057
66.9600 -41.9700 -0.1671 0.0917 -
0.2892 -0.3075 -0.0821 0.3231 0.0646
-0.2920 -1.4418 -39.9045
66.9800 -41.7600 -0.0279 0.3660 -
0.3134 -0.3391 -0.0856 0.3256 0.0581
-0.2909 -1.4422 -39.9032
67.0000 -41.3400 0.2003 0.3725 -
0.3040 -0.3544 -0.0858 0.3274 0.0514
-0.2896 -1.4426 -39.9020

67.0200 -41.4900 0.4335 0.0240 -
0.2581 -0.3535 -0.0827 0.3288 0.0446
-0.2884 -1.4430 -39.9008
67.0400 -42.7200 -0.5019 -0.3829 -
0.1766 -0.3373 -0.0764 0.3295 0.0377
-0.2871 -1.4433 -39.8996
67.0600 -42.7200 -0.4929 -0.4868 -
0.0637 -0.3075 -0.0670 0.3297 0.0306
-0.2857 -1.4437 -39.8984
67.0800 -41.2300 0.5957 -0.3033
0.0680 -0.2665 -0.0549 0.3294 0.0234
-0.2843 -1.4440 -39.8972
67.1000 -41.2300 0.2082 -0.0962
0.2023 -0.2171 -0.0404 0.3284 0.0161
-0.2829 -1.4443 -39.8960
67.1200 -41.3800 -0.2433 0.0487
0.3143 -0.1623 -0.0239 0.3268 0.0086
-0.2814 -1.4445 -39.8948
67.1400 -41.3800 -0.5988 0.2997
0.3721 -0.1049 -0.0058 0.3246 0.0009
-0.2799 -1.4448 -39.8936
67.1600 -40.2300 0.3714 0.5748
0.3537 -0.0476 0.0135 0.3219 -0.0068
-0.2783 -1.4450 -39.8924
67.1800 -40.1000 0.4018 0.5553
0.2567 0.0073 0.0337 0.3185 -0.0147
-0.2767 -1.4452 -39.8912
67.2000 -41.3700 -0.3988 0.1614
0.1022 0.0586 0.0542 0.3145 -0.0227
-0.2750 -1.4454 -39.8900
67.2200 -41.3700 0.2086 -0.3465 -
0.0664 0.1055 0.0747 0.3098 -0.0309
-0.2733 -1.4455 -39.8888
67.2400 -42.0600 -0.2115 -0.5843 -
0.2020 0.1471 0.0948 0.3046 -0.0392
-0.2716 -1.4456 -39.8875
67.2600 -41.8700 -0.0997 -0.4155 -
0.2692 0.1829 0.1139 0.2987 -0.0476
-0.2698 -1.4457 -39.8863
67.2800 -41.0000 0.3267 -0.0240 -
0.2627 0.2130 0.1317 0.2921 -0.0561
-0.2679 -1.4458 -39.8851
67.3000 -41.0800 -0.1839 0.3012 -
0.2010 0.2379 0.1475 0.2850 -0.0647
-0.2660 -1.4459 -39.8839
67.3200 -41.1700 -0.3888 0.3765 -
0.1108 0.2584 0.1610 0.2771 -0.0734
-0.2641 -1.4459 -39.8827
67.3400 -40.4800 0.4208 0.1414 -
0.0197 0.2752 0.1717 0.2687 -0.0823
-0.2621 -1.4460 -39.8815

67.3600 -40.2200 0.8928 -0.2017
0.0443 0.2888 0.1792 0.2595 -0.0912
-0.2600 -1.4459 -39.8803
67.3800 -42.3400 -1.2068 -0.2732
0.0627 0.2986 0.1835 0.2497 -0.1003
-0.2580 -1.4459 -39.8791
67.4000 -41.4200 -0.5966 0.0569
0.0406 0.3033 0.1842 0.2393 -0.1094
-0.2558 -1.4459 -39.8779
67.4200 -39.3400 1.2875 0.3590
0.0074 0.3011 0.1816 0.2283 -0.1186
-0.2537 -1.4458 -39.8766
67.4400 -41.0100 -0.1562 0.1618 -
0.0068 0.2907 0.1757 0.2166 -0.1279
-0.2514 -1.4457 -39.8754
67.4600 -42.2000 -0.8074 -0.2870
0.0100 0.2707 0.1669 0.2043 -0.1373
-0.2492 -1.4456 -39.8742
67.4800 -41.7100 -0.1293 -0.5128
0.0582 0.2403 0.1553 0.1915 -0.1467
-0.2468 -1.4455 -39.8730
67.5000 -41.5100 -0.0933 -0.3320
0.1287 0.1996 0.1413 0.1782 -0.1563
-0.2445 -1.4453 -39.8718
67.5200 -40.7700 0.2981 0.0431
0.1963 0.1494 0.1253 0.1644 -0.1658
-0.2421 -1.4452 -39.8706
67.5400 -40.8000 0.0201 0.3871
0.2334 0.0913 0.1077 0.1501 -0.1755
-0.2396 -1.4450 -39.8694
67.5600 -40.8800 -0.0391 0.5611
0.2169 0.0277 0.0889 0.1355 -0.1851
-0.2371 -1.4448 -39.8682
67.5800 -41.0600 -0.0838 0.4894
0.1378 -0.0386 0.0692 0.1205 -0.1948
-0.2345 -1.4445 -39.8669
67.6000 -41.3200 0.1617 0.1750
0.0146 -0.1037 0.0490 0.1051 -0.2046
-0.2319 -1.4443 -39.8657
67.6200 -42.0100 0.1798 -0.2671 -
0.1165 -0.1638 0.0287 0.0895 -0.2144
-0.2293 -1.4440 -39.8645
67.6400 -43.0400 -0.3085 -0.5695 -
0.2167 -0.2150 0.0085 0.0737 -0.2242
-0.2265 -1.4437 -39.8633
67.6600 -42.7300 -0.1281 -0.4835 -
0.2543 -0.2538 -0.0110 0.0577 -0.2340
-0.2238 -1.4434 -39.8621
67.6800 -42.1100 0.3208 -0.1182 -
0.2197 -0.2778 -0.0298 0.0415 -0.2438
-0.2210 -1.4430 -39.8609

67.7000 -42.0900 -0.0380 0.1932 -
0.1308 -0.2867 -0.0473 0.0253 -0.2537
-0.2181 -1.4427 -39.8596
67.7200 -41.9200 -0.0396 0.2786 -
0.0183 -0.2813 -0.0633 0.0090 -0.2635
-0.2152 -1.4423 -39.8584
67.7400 -41.9200 -0.0537 0.2120
0.0771 -0.2626 -0.0779 -0.0072 -0.2733
-0.2123 -1.4419 -39.8572
67.7600 -41.8600 -0.0128 0.1438
0.1192 -0.2322 -0.0907 -0.0234 -0.2831
-0.2093 -1.4415 -39.8560
67.7800 -41.8600 0.0813 0.1460
0.0966 -0.1921 -0.1019 -0.0394 -0.2929
-0.2062 -1.4411 -39.8548
67.8000 -42.0700 -0.0407 0.1190
0.0287 -0.1453 -0.1114 -0.0553 -0.3027
-0.2031 -1.4406 -39.8536
67.8200 -42.1000 -0.0510 -0.0177 -
0.0469 -0.0951 -0.1193 -0.0709 -0.3124
-0.2000 -1.4401 -39.8524
67.8400 -42.3200 0.1053 -0.1971 -
0.0921 -0.0451 -0.1256 -0.0862 -0.3221
-0.1968 -1.4396 -39.8511
67.8600 -42.4300 -0.1633 -0.3098 -
0.0813 0.0016 -0.1304 -0.1011 -0.3317
-0.1935 -1.4391 -39.8499
67.8800 -42.2400 0.0862 -0.2907 -
0.0112 0.0430 -0.1338 -0.1157 -0.3413
-0.1902 -1.4386 -39.8487
67.9000 -42.0900 0.0229 -0.1216
0.0940 0.0783 -0.1359 -0.1297 -0.3508
-0.1869 -1.4380 -39.8475
67.9200 -41.7300 -0.0319 0.1124
0.1949 0.1070 -0.1369 -0.1432 -0.3602
-0.1835 -1.4375 -39.8463
67.9400 -41.2900 0.1289 0.3051
0.2494 0.1287 -0.1369 -0.1561 -0.3696
-0.1800 -1.4369 -39.8451
67.9600 -41.4300 -0.0362 0.3601
0.2256 0.1431 -0.1361 -0.1683 -0.3788
-0.1765 -1.4363 -39.8438
67.9800 -41.7200 -0.1127 0.2657
0.1204 0.1501 -0.1345 -0.1799 -0.3880
-0.1730 -1.4356 -39.8426
68.0000 -41.7200 0.1948 0.0899 -
0.0369 0.1502 -0.1323 -0.1907 -0.3971
-0.1694 -1.4350 -39.8414
68.0200 -42.5400 -0.1445 -0.0894 -
0.2010 0.1439 -0.1297 -0.2006 -0.4061
-0.1657 -1.4343 -39.8402

68.0400 -42.5400 0.0735 -0.2708 -
0.3227 0.1318 -0.1268 -0.2097 -0.4149
-0.1620 -1.4337 -39.8390
68.0600 -42.8300 0.0305 -0.3979 -
0.3603 0.1149 -0.1236 -0.2180 -0.4237
-0.1583 -1.4330 -39.8377
68.0800 -42.9800 -0.2710 -0.3194 -
0.2976 0.0942 -0.1204 -0.2252 -0.4323
-0.1545 -1.4322 -39.8365
68.1000 -42.2600 0.0752 -0.0190 -
0.1489 0.0704 -0.1172 -0.2315 -0.4408
-0.1507 -1.4315 -39.8353
68.1200 -41.6900 0.1534 0.2607
0.0470 0.0444 -0.1141 -0.2368 -0.4491
-0.1468 -1.4307 -39.8341
68.1400 -41.7300 -0.0423 0.2882
0.2437 0.0171 -0.1111 -0.2411 -0.4573
-0.1428 -1.4300 -39.8329
68.1600 -41.7800 -0.0407 0.1315
0.3931 -0.0102 -0.1084 -0.2443 -0.4653
-0.1388 -1.4292 -39.8316
68.1800 -41.8200 -0.0661 -0.0214
0.4603 -0.0364 -0.1059 -0.2464 -0.4732
-0.1348 -1.4284 -39.8304
68.2000 -41.9500 0.0022 -0.0718
0.4241 -0.0600 -0.1037 -0.2474 -0.4809
-0.1307 -1.4275 -39.8292
68.2200 -41.9800 0.0736 -0.0288
0.2912 -0.0797 -0.1018 -0.2473 -0.4884
-0.1266 -1.4267 -39.8280
68.2400 -42.2400 -0.1927 0.0477
0.0915 -0.0945 -0.1002 -0.2461 -0.4957
-0.1224 -1.4258 -39.8268
68.2600 -42.2400 0.1926 0.1077 -
0.1357 -0.1034 -0.0989 -0.2437 -0.5028
-0.1181 -1.4249 -39.8255
68.2800 -42.6600 -0.1082 0.0437 -
0.3482 -0.1059 -0.0981 -0.2402 -0.5098
-0.1138 -1.4240 -39.8243
68.3000 -42.6600 0.3406 -0.1778 -
0.5072 -0.1011 -0.0978 -0.2356 -0.5165
-0.1095 -1.4231 -39.8231
68.3200 -43.5800 -0.4838 -0.3034 -
0.5858 -0.0885 -0.0981 -0.2299 -0.5230
-0.1051 -1.4222 -39.8219
68.3400 -43.6800 -0.6803 -0.1672 -
0.5701 -0.0685 -0.0991 -0.2230 -0.5293
-0.1007 -1.4212 -39.8207
68.3600 -41.9500 0.5841 0.0941 -
0.4654 -0.0423 -0.1011 -0.2151 -0.5353
-0.0962 -1.4202 -39.8194

68.3800 -41.4600 0.8759 0.1687 -
0.2852 -0.0122 -0.1040 -0.2061 -0.5412
-0.0917 -1.4192 -39.8182
68.4000 -42.3500 -0.1047 -0.0233 -
0.0534 0.0191 -0.1081 -0.1960 -0.5467
-0.0871 -1.4182 -39.8170
68.4200 -42.9500 -0.7703 -0.2054
0.1909 0.0491 -0.1135 -0.1850 -0.5521
-0.0825 -1.4172 -39.8158
68.4400 -42.0200 -0.1387 -0.1037
0.4060 0.0752 -0.1201 -0.1729 -0.5571
-0.0778 -1.4162 -39.8146
68.4600 -40.8500 0.3316 0.2361
0.5585 0.0948 -0.1280 -0.1599 -0.5620
-0.0731 -1.4151 -39.8133
68.4800 -40.8500 0.0935 0.5571
0.6189 0.1055 -0.1374 -0.1461 -0.5665
-0.0683 -1.4140 -39.8121
68.5000 -40.8400 0.0114 0.6025
0.5655 0.1059 -0.1481 -0.1314 -0.5708
-0.0635 -1.4129 -39.8109
68.5200 -41.0000 0.2859 0.3202
0.4026 0.0970 -0.1600 -0.1159 -0.5748
-0.0586 -1.4118 -39.8097
68.5400 -42.2900 -0.3836 -0.0758
0.1730 0.0811 -0.1731 -0.0997 -0.5785
-0.0537 -1.4107 -39.8084
68.5600 -42.2900 0.1824 -0.2904 -
0.0697 0.0602 -0.1871 -0.0829 -0.5820
-0.0488 -1.4096 -39.8072
68.5800 -42.6900 -0.0605 -0.3630 -
0.2690 0.0365 -0.2019 -0.0655 -0.5851
-0.0438 -1.4084 -39.8060
68.6000 -42.6900 0.1290 -0.4269 -
0.3787 0.0122 -0.2172 -0.0476 -0.5880
-0.0387 -1.4072 -39.8048
68.6200 -42.8900 -0.0266 -0.4087 -
0.3833 -0.0108 -0.2327 -0.0294 -0.5905
-0.0336 -1.4060 -39.8036
68.6400 -42.9200 -0.3256 -0.1873 -
0.2996 -0.0302 -0.2483 -0.0108 -0.5927
-0.0285 -1.4048 -39.8023
68.6600 -41.9100 0.2894 0.1439 -
0.1632 -0.0438 -0.2636 0.0079 -0.5946
-0.0233 -1.4036 -39.8011
68.6800 -41.4800 0.2823 0.3182 -
0.0166 -0.0498 -0.2784 0.0267 -0.5962
-0.0181 -1.4023 -39.7999
68.7000 -41.8500 -0.1108 0.2584
0.0941 -0.0463 -0.2922 0.0456 -0.5974
-0.0128 -1.4011 -39.7987

68.7200 -42.0900 -0.3673 0.1860
0.1322 -0.0323 -0.3050 0.0644 -0.5983
-0.0075 -1.3998 -39.7974
68.7400 -41.7300 0.0291 0.2151
0.0898 -0.0080 -0.3163 0.0830 -0.5989
-0.0022 -1.3985 -39.7962
68.7600 -41.4900 0.2973 0.2001 -
0.0075 0.0243 -0.3257 0.1013 -0.5991
0.0032 -1.3972 -39.7950
68.7800 -42.0900 0.0407 -0.0234 -
0.1172 0.0617 -0.3329 0.1192 -0.5989
0.0086 -1.3959 -39.7938
68.8000 -42.6900 -0.1782 -0.3522 -
0.1969 0.1012 -0.3373 0.1367 -0.5984
0.0141 -1.3945 -39.7925
68.8200 -42.5600 -0.0385 -0.5072 -
0.2145 0.1396 -0.3385 0.1537 -0.5975
0.0196 -1.3931 -39.7913
68.8400 -42.4300 -0.1801 -0.3172 -
0.1573 0.1738 -0.3363 0.1701 -0.5963
0.0252 -1.3918 -39.7901
68.8600 -41.6400 0.0708 0.0627 -
0.0468 0.2007 -0.3301 0.1857 -0.5946
0.0307 -1.3904 -39.7889
68.8800 -40.8500 0.3971 0.3226
0.0830 0.2176 -0.3198 0.2006 -0.5926
0.0363 -1.3890 -39.7877
68.9000 -41.1300 -0.0492 0.3620
0.1959 0.2214 -0.3051 0.2147 -0.5902
0.0420 -1.3875 -39.7864
68.9200 -41.3100 -0.3944 0.3448
0.2607 0.2099 -0.2858 0.2278 -0.5874
0.0477 -1.3861 -39.7852
68.9400 -40.8800 0.0227 0.3784
0.2620 0.1812 -0.2618 0.2400 -0.5843
0.0534 -1.3846 -39.7840
68.9600 -40.6300 0.3915 0.3232
0.2058 0.1356 -0.2331 0.2512 -0.5807
0.0591 -1.3832 -39.7828
68.9800 -41.5600 -0.2080 0.0608
0.1142 0.0747 -0.2000 0.2612 -0.5767
0.0649 -1.3817 -39.7815
69.0000 -41.9100 -0.1257 -0.2496
0.0177 0.0009 -0.1626 0.2702 -0.5723
0.0707 -1.3802 -39.7803
69.0200 -42.0900 0.0185 -0.4774 -
0.0527 -0.0834 -0.1213 0.2779 -0.5675
0.0765 -1.3786 -39.7791
69.0400 -42.1400 0.1699 -0.6370 -
0.0715 -0.1751 -0.0765 0.2845 -0.5622
0.0824 -1.3771 -39.7779

69.0600 -42.3200 -0.0551 -0.6152 -
0.0344 -0.2710 -0.0284 0.2897 -0.5566
0.0883 -1.3755 -39.7766
69.0800 -42.3700 -0.5568 -0.2098
0.0335 -0.3670 0.0224 0.2936 -0.5505
0.0942 -1.3740 -39.7754
69.1000 -40.7200 0.4393 0.4321
0.0973 -0.4584 0.0756 0.2961 -0.5440
0.1001 -1.3724 -39.7742
69.1200 -40.3100 0.4036 0.7864
0.1223 -0.5403 0.1307 0.2972 -0.5370
0.1060 -1.3708 -39.7730
69.1400 -41.2900 -0.2386 0.5656
0.0842 -0.6074 0.1871 0.2968 -0.5297
0.1120 -1.3691 -39.7717
69.1600 -41.5400 0.1315 -0.0214 -
0.0055 -0.6550 0.2442 0.2949 -0.5219
0.1180 -1.3675 -39.7705
69.1800 -42.3500 -0.1624 -0.5196 -
0.1184 -0.6796 0.3015 0.2914 -0.5137
0.1240 -1.3659 -39.7693
69.2000 -42.5800 -0.2443 -0.5915 -
0.2265 -0.6785 0.3584 0.2863 -0.5050
0.1300 -1.3642 -39.7681
69.2200 -41.8100 0.1350 -0.2458 -
0.3117 -0.6499 0.4142 0.2796 -0.4960
0.1360 -1.3625 -39.7668
69.2400 -41.4200 0.0507 0.1658 -
0.3747 -0.5927 0.4683 0.2713 -0.4865
0.1421 -1.3608 -39.7656
69.2600 -41.3100 0.0113 0.3123 -
0.4240 -0.5066 0.5203 0.2612 -0.4766
0.1482 -1.3591 -39.7644
69.2800 -41.2700 0.0087 0.2056 -
0.4739 -0.3921 0.5694 0.2493 -0.4663
0.1542 -1.3574 -39.7632
69.3000 -41.3200 -0.0933 0.0720 -
0.5353 -0.2518 0.6151 0.2357 -0.4557
0.1603 -1.3556 -39.7619
69.3200 -41.3400 -0.1565 0.0427 -
0.6055 -0.0904 0.6569 0.2204 -0.4446
0.1664 -1.3539 -39.7607
69.3400 -40.7900 0.3389 0.0055 -
0.6594 0.0851 0.6941 0.2032 -0.4332
0.1725 -1.3521 -39.7595
69.3600 -40.8600 0.0847 -0.1490 -
0.6631 0.2668 0.7261 0.1841 -0.4214
0.1786 -1.3503 -39.7583
69.3800 -41.2300 -0.3709 -0.2961 -
0.5915 0.4468 0.7523 0.1632 -0.4093
0.1847 -1.3485 -39.7570

69.4000 -40.3500 0.0880 -0.2683 -
0.4333 0.6181 0.7721 0.1405 -0.3968
0.1908 -1.3467 -39.7558
69.4200 -39.8600 0.0449 -0.1235 -
0.1905 0.7741 0.7849 0.1159 -0.3840
0.1970 -1.3448 -39.7546
69.4400 -39.4400 -0.1073 -0.0085
0.1184 0.9088 0.7900 0.0894 -0.3709
0.2031 -1.3430 -39.7534
69.4600 -38.8600 0.1178 0.0073
0.4644 1.0165 0.7869 0.0612 -0.3575
0.2092 -1.3411 -39.7521
69.4800 -38.6300 -0.0009 -0.0869
0.8049 1.0912 0.7747 0.0313 -0.3438
0.2153 -1.3392 -39.7509
69.5000 -38.1500 0.1907 -0.1947
1.0845 1.1271 0.7531 -0.0002 -0.3298
0.2215 -1.3373 -39.7497
69.5200 -38.5500 -0.4080 -0.0674
1.2495 1.1184 0.7218 -0.0332 -0.3156
0.2276 -1.3354 -39.7485
69.5400 -37.8500 0.0371 0.3473
1.2616 1.0612 0.6812 -0.0676 -0.3011
0.2337 -1.3335 -39.7472
69.5600 -37.6700 0.2130 0.7299
1.0976 0.9576 0.6321 -0.1031 -0.2864
0.2398 -1.3315 -39.7460
69.5800 -38.2500 0.1543 0.7317
0.7538 0.8150 0.5754 -0.1397 -0.2715
0.2459 -1.3296 -39.7448
69.6000 -39.6200 -0.0388 0.3333
0.2771 0.6423 0.5121 -0.1773 -0.2564
0.2520 -1.3276 -39.7436
69.6200 -40.9100 -0.1016 -0.1990 -
0.2503 0.4482 0.4431 -0.2155 -0.2410
0.2581 -1.3256 -39.7423
69.6400 -42.0000 0.0050 -0.5934 -
0.7390 0.2417 0.3694 -0.2544 -0.2256
0.2641 -1.3236 -39.7411
69.6600 -42.8100 -0.0590 -0.7241 -
1.1024 0.0315 0.2918 -0.2938 -0.2099
0.2702 -1.3216 -39.7399
69.6800 -43.0300 0.0749 -0.6284 -
1.2751 -0.1735 0.2115 -0.3335 -0.1941
0.2762 -1.3196 -39.7386
69.7000 -43.3500 -0.1487 -0.3625 -
1.2377 -0.3654 0.1291 -0.3734 -0.1782
0.2823 -1.3175 -39.7374
69.7200 -43.0000 -0.1372 0.0128 -
1.0141 -0.5393 0.0459 -0.4133 -0.1621
0.2883 -1.3155 -39.7362

69.7400 -42.2000 0.2290 0.2990 -
0.6555 -0.6921 -0.0375 -0.4531 -0.1460
0.2943 -1.3134 -39.7350
69.7600 -42.2000 0.0646 0.2840 -
0.2216 -0.8211 -0.1199 -0.4926 -0.1297
0.3002 -1.3113 -39.7337
69.7800 -42.2900 -0.1120 0.0613
0.2129 -0.9235 -0.2005 -0.5317 -0.1134
0.3062 -1.3092 -39.7325
69.8000 -42.2900 -0.1066 -0.0562
0.5689 -0.9967 -0.2782 -0.5703 -0.0971
0.3121 -1.3071 -39.7313
69.8200 -41.8500 0.0382 0.0546
0.7851 -1.0381 -0.3523 -0.6081 -0.0806
0.3181 -1.3049 -39.7301
69.8400 -41.5900 0.1302 0.3006
0.8281 -1.0461 -0.4219 -0.6451 -0.0642
0.3239 -1.3028 -39.7288
69.8600 -41.7100 -0.0385 0.4990
0.7032 -1.0218 -0.4863 -0.6812 -0.0477
0.3298 -1.3006 -39.7276
69.8800 -42.1300 -0.1920 0.4881
0.4462 -0.9688 -0.5451 -0.7160 -0.0312
0.3357 -1.2984 -39.7264
69.9000 -42.1300 0.5448 0.1639
0.1188 -0.8918 -0.5981 -0.7496 -0.0147
0.3415 -1.2962 -39.7252
69.9200 -43.6700 -0.3617 -0.3139 -
0.2033 -0.7956 -0.6451 -0.7818 0.0017
0.3473 -1.2940 -39.7239
69.9400 -44.1100 -0.2801 -0.5177 -
0.4462 -0.6850 -0.6862 -0.8123 0.0182
0.3530 -1.2918 -39.7227
69.9600 -43.5700 0.0069 -0.2841 -
0.5609 -0.5648 -0.7215 -0.8412 0.0345
0.3588 -1.2896 -39.7215
69.9800 -42.8900 0.2266 0.0985 -
0.5532 -0.4396 -0.7511 -0.8682 0.0509
0.3645 -1.2873 -39.7203
70.0000 -42.7900 -0.0507 0.2955 -
0.4514 -0.3131 -0.7751 -0.8932 0.0671
0.3701 -1.2851 -39.7190
70.0200 -42.4300 0.1441 0.2179 -
0.2914 -0.1882 -0.7937 -0.9162 0.0833
0.3758 -1.2828 -39.7178
70.0400 -42.4100 -0.0582 0.0000 -
0.1070 -0.0679 -0.8069 -0.9369 0.0993
0.3814 -1.2805 -39.7166
70.0600 -42.2700 0.1158 -0.1831
0.0741 0.0449 -0.8148 -0.9554 0.1153
0.3869 -1.2782 -39.7154

70.0800 -42.2700 -0.2944 -0.1503
0.2285 0.1473 -0.8175 -0.9716 0.1311
0.3924 -1.2759 -39.7141
70.1000 -41.3900 0.1957 0.0610
0.3383 0.2365 -0.8151 -0.9853 0.1468
0.3979 -1.2736 -39.7129
70.1200 -41.2800 0.0097 0.1993
0.3931 0.3101 -0.8078 -0.9965 0.1624
0.4034 -1.2712 -39.7117
70.1400 -41.3000 -0.0658 0.1369
0.3902 0.3667 -0.7956 -1.0052 0.1778
0.4088 -1.2688 -39.7105
70.1600 -41.3000 0.0910 -0.0631
0.3394 0.4054 -0.7788 -1.0113 0.1931
0.4141 -1.2665 -39.7092
70.1800 -41.6800 0.0033 -0.2303
0.2577 0.4272 -0.7578 -1.0148 0.2082
0.4195 -1.2641 -39.7080
70.2000 -41.8700 -0.2148 -0.1802
0.1611 0.4339 -0.7330 -1.0156 0.2231
0.4247 -1.2617 -39.7068
70.2200 -41.3600 -0.0135 0.0836
0.0588 0.4285 -0.7047 -1.0137 0.2378
0.4300 -1.2593 -39.7056
70.2400 -41.0200 0.3212 0.2829 -
0.0459 0.4146 -0.6737 -1.0091 0.2524
0.4351 -1.2568 -39.7043
70.2600 -41.5600 -0.1040 0.2063 -
0.1489 0.3960 -0.6405 -1.0018 0.2668
0.4403 -1.2544 -39.7031
70.2800 -41.7900 -0.0794 -0.0190 -
0.2395 0.3762 -0.6056 -0.9919 0.2809
0.4454 -1.2519 -39.7019
70.3000 -41.8900 -0.0772 -0.1791 -
0.3009 0.3582 -0.5695 -0.9793 0.2949
0.4504 -1.2495 -39.7007
70.3200 -41.9100 -0.0567 -0.1912 -
0.3183 0.3444 -0.5329 -0.9640 0.3087
0.4554 -1.2470 -39.6994
70.3400 -41.5500 0.1552 -0.1492 -
0.2850 0.3362 -0.4959 -0.9462 0.3222
0.4603 -1.2445 -39.6982
70.3600 -41.5000 0.0074 -0.0839 -
0.2079 0.3335 -0.4589 -0.9258 0.3356
0.4652 -1.2420 -39.6970
70.3800 -41.2800 -0.2342 0.0862 -
0.1071 0.3347 -0.4220 -0.9028 0.3487
0.4700 -1.2395 -39.6958
70.4000 -40.7200 -0.0754 0.3142 -
0.0062 0.3375 -0.3849 -0.8774 0.3617
0.4748 -1.2369 -39.6945

70.4200 -39.9300 0.5905 0.3292
0.0795 0.3390 -0.3477 -0.8496 0.3744
0.4795 -1.2344 -39.6933
70.4400 -40.8200 -0.2449 0.0133
0.1439 0.3358 -0.3101 -0.8195 0.3869
0.4842 -1.2318 -39.6921
70.4600 -41.2600 -0.4348 -0.2926
0.1944 0.3246 -0.2720 -0.7871 0.3991
0.4888 -1.2292 -39.6909
70.4800 -40.6000 0.2112 -0.2896
0.2416 0.3025 -0.2332 -0.7525 0.4112
0.4933 -1.2266 -39.6896
70.5000 -40.3600 -0.0075 -0.0543
0.2856 0.2675 -0.1937 -0.7159 0.4230
0.4978 -1.2240 -39.6884
70.5200 -39.9700 0.2554 0.1159
0.3184 0.2193 -0.1532 -0.6773 0.4346
0.5022 -1.2214 -39.6872
70.5400 -40.3300 -0.1917 0.1010
0.3298 0.1588 -0.1118 -0.6368 0.4459
0.5065 -1.2188 -39.6860
70.5600 -40.2500 -0.0441 0.0371
0.3125 0.0880 -0.0694 -0.5944 0.4571
0.5108 -1.2161 -39.6847
70.5800 -39.9500 0.2088 0.0048
0.2625 0.0099 -0.0261 -0.5503 0.4679
0.5150 -1.2135 -39.6835
70.6000 -40.4000 -0.2009 0.0115
0.1794 -0.0717 0.0180 -0.5046 0.4786
0.5192 -1.2108 -39.6823
70.6200 -40.4300 -0.1485 0.0646
0.0713 -0.1523 0.0628 -0.4574 0.4889
0.5232 -1.2081 -39.6811
70.6400 -40.0900 0.2543 0.1000 -
0.0456 -0.2270 0.1081 -0.4086 0.4991
0.5272 -1.2055 -39.6798
70.6600 -40.3700 0.1816 -0.0151 -
0.1537 -0.2909 0.1540 -0.3585 0.5089
0.5312 -1.2027 -39.6786
70.6800 -40.6200 0.1265 -0.2349 -
0.2421 -0.3393 0.2001 -0.3071 0.5185
0.5351 -1.2000 -39.6774
70.7000 -41.2600 -0.5136 -0.2804 -
0.3068 -0.3688 0.2464 -0.2545 0.5279
0.5388 -1.1973 -39.6762
70.7200 -40.2200 0.1701 -0.0062 -
0.3498 -0.3768 0.2926 -0.2007 0.5370
0.5426 -1.1946 -39.6749
70.7400 -39.6700 0.2813 0.3063 -
0.3720 -0.3630 0.3384 -0.1457 0.5458
0.5462 -1.1918 -39.6737

70.7600	-39.7300	0.1023	0.3360	-
0.3694	-0.3281	0.3833	-0.0897	0.5544
	0.5498	-1.1890	-39.6725	
70.7800	-40.2000	-0.2907	0.0732	-
0.3335	-0.2738	0.4267	-0.0328	0.5626
	0.5533	-1.1862	-39.6713	
70.8000	-40.0000	0.0826	-0.2862	-
0.2561	-0.2037	0.4680	0.0252	0.5706
	0.5567	-1.1834	-39.6701	
70.8200	-39.6500	0.3142	-0.4698	-
0.1365	-0.1225	0.5066	0.0840	0.5784
	0.5600	-1.1806	-39.6688	
70.8400	-39.8500	-0.5238	-0.2681	
0.0100	-0.0359	0.5418	0.1437	0.5858
	0.5633	-1.1778	-39.6676	
70.8600	-39.1600	-0.6470	0.1955	
0.1548	0.0502	0.5731	0.2041	0.5930
	0.5665	-1.1750	-39.6664	
70.8800	-37.7000	0.1355	0.5724	
0.2647	0.1298	0.5999	0.2652	0.5998
	0.5695	-1.1721	-39.6652	
70.9000	-36.6000	1.2146	0.4104	
0.3133	0.1968	0.6215	0.3270	0.6064
	0.5725	-1.1693	-39.6639	
70.9200	-39.5900	-1.1750	-0.2896	
0.3008	0.2466	0.6373	0.3894	0.6127
	0.5755	-1.1664	-39.6627	
70.9400	-37.6400	0.7671	-0.5078	
0.2476	0.2764	0.6468	0.4522	0.6187
	0.5783	-1.1635	-39.6615	
70.9600	-38.3600	-0.3410	-0.0523	
0.1778	0.2853	0.6495	0.5155	0.6244
	0.5811	-1.1606	-39.6603	
70.9800	-37.7000	-0.0931	0.3393	
0.1087	0.2746	0.6450	0.5791	0.6297
	0.5837	-1.1577	-39.6591	
71.0000	-37.3200	0.3464	0.3340	
0.0490	0.2466	0.6333	0.6429	0.6348
	0.5863	-1.1548	-39.6578	
71.0200	-38.0000	0.1026	-0.0086	-
0.0024	0.2049	0.6143	0.7068	0.6395
	0.5888	-1.1519	-39.6566	
71.0400	-38.8700	-0.4864	-0.2709	-
0.0480	0.1544	0.5884	0.7707	0.6440
	0.5912	-1.1489	-39.6554	
71.0600	-37.9400	0.2733	-0.1456	-
0.0854	0.1002	0.5560	0.8345	0.6481
	0.5935	-1.1460	-39.6542	
71.0800	-37.9000	0.2341	0.0583	-
0.1068	0.0470	0.5177	0.8980	0.6519
	0.5957	-1.1430	-39.6529	

71.1000 -38.1700 -0.0963 0.0664 -
0.1097 -0.0012 0.4740 0.9611 0.6553
0.5978 -1.1400 -39.6517
71.1200 -38.4600 -0.1184 -0.0095 -
0.1038 -0.0413 0.4255 1.0237 0.6584
0.5998 -1.1371 -39.6505
71.1400 -38.3100 -0.1390 0.0079 -
0.1012 -0.0710 0.3730 1.0858 0.6612
0.6018 -1.1341 -39.6493
71.1600 -37.9700 0.1420 0.1055 -
0.1069 -0.0898 0.3174 1.1471 0.6636
0.6036 -1.1310 -39.6481
71.1800 -37.9700 0.1014 0.1020 -
0.1176 -0.0985 0.2593 1.2076 0.6657
0.6053 -1.1280 -39.6468
71.2000 -38.0500 0.3046 -0.1095 -
0.1219 -0.0996 0.1998 1.2670 0.6674
0.6070 -1.1250 -39.6456
71.2200 -39.0900 -0.5838 -0.2540 -
0.0981 -0.0958 0.1395 1.3253 0.6687
0.6085 -1.1219 -39.6444
71.2400 -38.0000 0.3078 -0.0608 -
0.0327 -0.0896 0.0794 1.3823 0.6697
0.6099 -1.1189 -39.6432
71.2600 -37.7300 0.1802 0.1431
0.0669 -0.0834 0.0202 1.4379 0.6703
0.6113 -1.1158 -39.6420
71.2800 -37.9400 -0.1184 0.0658
0.1779 -0.0792 -0.0372 1.4919 0.6705
0.6125 -1.1127 -39.6407
71.3000 -37.9400 0.0861 -0.1034
0.2569 -0.0786 -0.0920 1.5443 0.6703
0.6136 -1.1096 -39.6395
71.3200 -38.0800 -0.2046 0.0080
0.2596 -0.0826 -0.1435 1.5948 0.6697
0.6147 -1.1065 -39.6383
71.3400 -37.9600 -0.3512 0.3153
0.1799 -0.0912 -0.1911 1.6433 0.6687
0.6156 -1.1034 -39.6371
71.3600 -37.1300 0.6802 0.3742
0.0409 -0.1033 -0.2341 1.6897 0.6674
0.6164 -1.1003 -39.6359
71.3800 -38.7700 -0.5211 -0.0675 -
0.1170 -0.1173 -0.2719 1.7340 0.6656
0.6172 -1.0971 -39.6346
71.4000 -38.7700 -0.0230 -0.4713 -
0.2345 -0.1317 -0.3041 1.7759 0.6634
0.6178 -1.0940 -39.6334
71.4200 -38.9400 -0.2743 -0.4075 -
0.2586 -0.1449 -0.3302 1.8154 0.6608
0.6183 -1.0908 -39.6322

71.4400 -38.1200 0.2668 -0.0858 -
0.1843 -0.1563 -0.3499 1.8524 0.6577
0.6187 -1.0876 -39.6310
71.4600 -37.7700 0.1679 0.1698 -
0.0467 -0.1662 -0.3630 1.8867 0.6542
0.6191 -1.0844 -39.6298
71.4800 -37.9000 -0.0899 0.2537
0.1023 -0.1757 -0.3693 1.9184 0.6503
0.6193 -1.0812 -39.6285
71.5000 -37.6200 -0.0936 0.2899
0.2093 -0.1857 -0.3685 1.9472 0.6460
0.6194 -1.0780 -39.6273
71.5200 -37.2800 0.1878 0.3133
0.2402 -0.1969 -0.3608 1.9732 0.6412
0.6194 -1.0748 -39.6261
71.5400 -37.5300 0.0484 0.2840
0.1931 -0.2095 -0.3460 1.9963 0.6359
0.6193 -1.0716 -39.6249
71.5600 -37.9800 -0.3643 0.1486
0.0940 -0.2227 -0.3243 2.0163 0.6303
0.6191 -1.0683 -39.6237
71.5800 -37.6500 0.3470 -0.0834 -
0.0229 -0.2362 -0.2960 2.0334 0.6241
0.6188 -1.0651 -39.6225
71.6000 -38.2400 0.0427 -0.3172 -
0.1227 -0.2495 -0.2611 2.0473 0.6176
0.6184 -1.0618 -39.6212
71.6200 -38.8800 -0.3442 -0.4210 -
0.1790 -0.2624 -0.2202 2.0582 0.6106
0.6179 -1.0585 -39.6200
71.6400 -37.9600 0.3125 -0.2889 -
0.1857 -0.2747 -0.1734 2.0660 0.6031
0.6173 -1.0552 -39.6188
71.6600 -38.2100 -0.3334 0.0414 -
0.1545 -0.2864 -0.1213 2.0705 0.5952
0.6166 -1.0519 -39.6176
71.6800 -37.3000 0.2219 0.3749 -
0.1054 -0.2976 -0.0641 2.0720 0.5869
0.6158 -1.0486 -39.6164
71.7000 -37.2100 0.1348 0.4314 -
0.0573 -0.3079 -0.0023 2.0701 0.5781
0.6149 -1.0453 -39.6152
71.7200 -37.4600 0.1741 0.1206 -
0.0195 -0.3170 0.0638 2.0651 0.5689
0.6139 -1.0420 -39.6139
71.7400 -38.3100 -0.4279 -0.2435
0.0133 -0.3246 0.1336 2.0567 0.5593
0.6128 -1.0386 -39.6127
71.7600 -38.0000 -0.0120 -0.3428
0.0504 -0.3304 0.2067 2.0450 0.5493
0.6117 -1.0353 -39.6115

71.7800	-37.2100	0.4093	-0.2118	
0.0981	-0.3341	0.2824	2.0299	0.5389
	0.6104	-1.0319	-39.6103	
71.8000	-37.3800	-0.0393	-0.0444	
0.1528	-0.3349	0.3600	2.0113	0.5281
	0.6090	-1.0285	-39.6091	
71.8200	-37.4900	-0.3501	0.0791	
0.1980	-0.3319	0.4387	1.9893	0.5170
	0.6075	-1.0251	-39.6079	
71.8400	-36.7200	0.3328	0.1482	
0.2127	-0.3242	0.5176	1.9638	0.5054
	0.6060	-1.0217	-39.6066	
71.8600	-36.9500	0.0781	0.1357	
0.1816	-0.3105	0.5959	1.9347	0.4936
	0.6043	-1.0183	-39.6054	
71.8800	-37.0700	-0.0156	0.0540	
0.1013	-0.2891	0.6725	1.9021	0.4813
	0.6026	-1.0149	-39.6042	
71.9000	-37.4900	-0.2096	-0.0271	-
0.0188	-0.2584	0.7464	1.8659	0.4688
	0.6008	-1.0115	-39.6030	
71.9200	-37.1700	0.1038	-0.0322	-
0.1570	-0.2165	0.8167	1.8261	0.4559
	0.5988	-1.0080	-39.6018	
71.9400	-37.4600	-0.1273	0.0289	-
0.2870	-0.1615	0.8822	1.7827	0.4427
	0.5968	-1.0046	-39.6006	
71.9600	-37.2600	0.0890	0.0472	-
0.3846	-0.0912	0.9421	1.7357	0.4292
	0.5947	-1.0011	-39.5994	
71.9800	-37.2400	0.2052	-0.0610	-
0.4322	-0.0042	0.9956	1.6852	0.4154
	0.5925	-0.9976	-39.5981	
72.0000	-37.6500	-0.2141	-0.1873	-
0.4225	0.1001	1.0417	1.6312	0.4014
	0.5903	-0.9942	-39.5969	
72.0200	-37.3900	-0.0882	-0.1658	-
0.3634	0.2206	1.0801	1.5736	0.3870
	0.5879	-0.9907	-39.5957	
72.0400	-36.8600	0.0395	0.0419	-
0.2778	0.3556	1.1099	1.5126	0.3725
	0.5854	-0.9872	-39.5945	
72.0600	-36.5100	-0.0283	0.2883	-
0.1949	0.5027	1.1308	1.4482	0.3577
	0.5829	-0.9836	-39.5933	
72.0800	-36.0800	0.2358	0.3538	-
0.1398	0.6585	1.1422	1.3804	0.3426
	0.5803	-0.9801	-39.5921	
72.1000	-36.7000	-0.1407	0.1223	-
0.1191	0.8172	1.1435	1.3095	0.3273
	0.5776	-0.9766	-39.5909	

72.1200 -36.3400 0.3820 -0.2509 -
0.1163 0.9703 1.1342 1.2354 0.3119
0.5748 -0.9730 -39.5896
72.1400 -37.4300 -0.7640 -0.3650 -
0.1007 1.1075 1.1139 1.1584 0.2962
0.5719 -0.9695 -39.5884
72.1600 -36.2700 0.1958 -0.0982 -
0.0396 1.2187 1.0823 1.0786 0.2803
0.5690 -0.9659 -39.5872
72.1800 -35.4000 0.7385 0.1560
0.0911 1.2943 1.0391 0.9961 0.2643
0.5659 -0.9623 -39.5860
72.2000 -35.7400 0.4629 0.0128
0.2973 1.3278 0.9844 0.9112 0.2481
0.5628 -0.9587 -39.5848
72.2200 -36.7000 -0.2248 -0.4030
0.5412 1.3153 0.9184 0.8241 0.2318
0.5596 -0.9551 -39.5836
72.2400 -37.1500 -0.4879 -0.5877
0.7609 1.2542 0.8421 0.7348 0.2154
0.5564 -0.9515 -39.5824
72.2600 -36.9200 -0.4407 -0.2089
0.9001 1.1434 0.7563 0.6436 0.1988
0.5530 -0.9479 -39.5812
72.2800 -35.4600 0.6022 0.4904
0.9286 0.9847 0.6625 0.5507 0.1821
0.5496 -0.9443 -39.5800
72.3000 -36.3300 -0.1054 0.9980
0.8247 0.7839 0.5618 0.4562 0.1653
0.5461 -0.9406 -39.5787
72.3200 -36.7900 0.0870 1.0088
0.5799 0.5497 0.4556 0.3603 0.1484
0.5425 -0.9370 -39.5775
72.3400 -37.8100 0.4031 0.5119
0.2289 0.2924 0.3451 0.2633 0.1315
0.5388 -0.9333 -39.5763
72.3600 -40.1300 -0.2531 -0.2344 -
0.1668 0.0222 0.2318 0.1652 0.1144
0.5351 -0.9297 -39.5751
72.3800 -41.7900 -0.5867 -0.8188 -
0.5409 -0.2507 0.1168 0.0663 0.0974
0.5313 -0.9260 -39.5739
72.4000 -42.1600 0.2356 -1.0112 -
0.8286 -0.5159 0.0014 -0.0333 0.0803
0.5274 -0.9223 -39.5727
72.4200 -42.1600 0.7323 -0.9021 -
0.9807 -0.7631 -0.1130 -0.1334 0.0632
0.5235 -0.9186 -39.5715
72.4400 -43.4200 -0.5533 -0.5643 -
0.9824 -0.9830 -0.2251 -0.2338 0.0461
0.5194 -0.9149 -39.5703

72.4600 -43.4200 -0.7442 -0.0417 -
0.8493 -1.1689 -0.3337 -0.3343 0.0289
0.5153 -0.9112 -39.5691
72.4800 -41.7600 0.4721 0.4942 -
0.6133 -1.3177 -0.4374 -0.4348 0.0118
0.5112 -0.9074 -39.5679
72.5000 -41.5200 0.6539 0.7162 -
0.3122 -1.4283 -0.5350 -0.5350 -0.0053
0.5069 -0.9037 -39.5667
72.5200 -42.1200 0.1505 0.4678
0.0098 -1.5004 -0.6253 -0.6348 -0.0223
0.5026 -0.8999 -39.5654
72.5400 -43.1200 -0.5925 0.0763
0.3030 -1.5342 -0.7072 -0.7341 -0.0393
0.4983 -0.8962 -39.5642
72.5600 -43.1200 -0.4196 -0.0553
0.5203 -1.5307 -0.7798 -0.8325 -0.0563
0.4938 -0.8924 -39.5630
72.5800 -42.3000 0.2764 0.0958
0.6358 -1.4918 -0.8425 -0.9301 -0.0731
0.4893 -0.8887 -39.5618
72.6000 -42.1800 0.3010 0.2217
0.6539 -1.4204 -0.8948 -1.0264 -0.0899
0.4847 -0.8849 -39.5606
72.6200 -42.5400 0.2787 0.0501
0.5930 -1.3205 -0.9367 -1.1215 -0.1066
0.4801 -0.8811 -39.5594
72.6400 -43.6300 -0.3689 -0.2283
0.4722 -1.1966 -0.9684 -1.2152 -0.1231
0.4754 -0.8773 -39.5582
72.6600 -43.6300 -0.1940 -0.2637
0.3077 -1.0531 -0.9902 -1.3072 -0.1396
0.4706 -0.8735 -39.5570
72.6800 -43.2300 0.1291 -0.0130
0.1178 -0.8945 -1.0026 -1.3974 -0.1559
0.4658 -0.8696 -39.5558
72.7000 -43.1000 0.0982 0.2814 -
0.0792 -0.7255 -1.0061 -1.4858 -0.1721
0.4609 -0.8658 -39.5546
72.7200 -43.1000 0.1155 0.3633 -
0.2639 -0.5508 -1.0011 -1.5721 -0.1881
0.4559 -0.8620 -39.5534
72.7400 -43.6400 -0.2075 0.2143 -
0.4171 -0.3752 -0.9882 -1.6564 -0.2039
0.4509 -0.8581 -39.5522
72.7600 -43.6400 0.0242 -0.0480 -
0.5144 -0.2030 -0.9679 -1.7385 -0.2195
0.4458 -0.8543 -39.5510
72.7800 -43.6900 0.1423 -0.3122 -
0.5288 -0.0386 -0.9407 -1.8183 -0.2350
0.4407 -0.8504 -39.5498

72.8000 -43.7500 -0.0483 -0.4360 -
0.4445 0.1158 -0.9071 -1.8957 -0.2502
0.4355 -0.8465 -39.5486
72.8200 -43.7500 -0.3456 -0.2619 -
0.2711 0.2588 -0.8677 -1.9707 -0.2652
0.4303 -0.8426 -39.5474
72.8400 -42.3300 0.3226 0.1438 -
0.0457 0.3898 -0.8231 -2.0432 -0.2800
0.4250 -0.8388 -39.5461
72.8600 -41.8500 0.1366 0.4134
0.1812 0.5082 -0.7738 -2.1132 -0.2945
0.4196 -0.8349 -39.5449
72.8800 -41.8500 -0.0332 0.3683
0.3596 0.6136 -0.7205 -2.1804 -0.3088
0.4142 -0.8310 -39.5437
72.9000 -41.9400 -0.0668 0.1697
0.4549 0.7053 -0.6638 -2.2449 -0.3228
0.4087 -0.8270 -39.5425
72.9200 -41.9400 0.0330 -0.0650
0.4623 0.7827 -0.6044 -2.3066 -0.3365
0.4032 -0.8231 -39.5413
72.9400 -42.1600 0.0378 -0.2763
0.3947 0.8455 -0.5431 -2.3653 -0.3499
0.3976 -0.8192 -39.5401
72.9600 -42.4500 -0.1127 -0.3278
0.2655 0.8946 -0.4805 -2.4212 -0.3630
0.3919 -0.8152 -39.5389
72.9800 -42.4500 -0.1837 -0.1105
0.0941 0.9316 -0.4174 -2.4740 -0.3758
0.3863 -0.8113 -39.5377
73.0000 -42.0400 0.0079 0.2563 -
0.0925 0.9584 -0.3546 -2.5238 -0.3883
0.3805 -0.8073 -39.5365
73.0200 -41.9000 0.2024 0.4256 -
0.2673 0.9773 -0.2929 -2.5705 -0.4004
0.3747 -0.8034 -39.5353
73.0400 -42.0800 0.3145 0.1828 -
0.4066 0.9902 -0.2331 -2.6140 -0.4121
0.3689 -0.7994 -39.5341
73.0600 -43.3000 -0.5106 -0.2454 -
0.4845 0.9987 -0.1759 -2.6543 -0.4235
0.3630 -0.7954 -39.5329
73.0800 -43.3000 -0.0637 -0.4627 -
0.4773 1.0041 -0.1220 -2.6914 -0.4345
0.3571 -0.7914 -39.5317
73.1000 -42.8300 0.2460 -0.4153 -
0.3729 1.0063 -0.0717 -2.7252 -0.4452
0.3511 -0.7874 -39.5305
73.1200 -42.4700 0.0378 -0.2207 -
0.1875 1.0043 -0.0256 -2.7556 -0.4554
0.3451 -0.7834 -39.5293

73.1400 −42.4700 −0.2930 0.0642
0.0351 0.9962 0.0160 −2.7827 −0.4652
0.3390 −0.7794 −39.5281
73.1600 −41.3800 0.1922 0.3398
0.2457 0.9802 0.0531 −2.8065 −0.4746
0.3329 −0.7754 −39.5269
73.1800 −41.3800 0.0077 0.4259
0.4000 0.9541 0.0854 −2.8269 −0.4836
0.3267 −0.7714 −39.5257
73.2000 −41.6100 0.1259 0.2455
0.4712 0.9159 0.1129 −2.8440 −0.4922
0.3205 −0.7673 −39.5245
73.2200 −42.1900 −0.1702 −0.0620
0.4662 0.8641 0.1356 −2.8577 −0.5003
0.3143 −0.7633 −39.5233
73.2400 −42.1900 0.0670 −0.2453
0.4067 0.7984 0.1533 −2.8680 −0.5079
0.3080 −0.7593 −39.5221
73.2600 −42.3200 −0.0186 −0.1870
0.3131 0.7195 0.1663 −2.8751 −0.5151
0.3016 −0.7552 −39.5209
73.2800 −42.4000 −0.0092 −0.0304
0.2004 0.6298 0.1745 −2.8788 −0.5219
0.2953 −0.7511 −39.5197
73.3000 −42.4000 0.0826 0.0668
0.0761 0.5330 0.1780 −2.8793 −0.5281
0.2889 −0.7471 −39.5185
73.3200 −42.8300 −0.0885 0.0893 −
0.0534 0.4336 0.1768 −2.8765 −0.5339
0.2824 −0.7430 −39.5173
73.3400 −42.8900 0.0680 0.0915 −
0.1810 0.3359 0.1710 −2.8705 −0.5393
0.2759 −0.7389 −39.5161
73.3600 −43.0900 0.1249 0.0395 −
0.2936 0.2442 0.1605 −2.8613 −0.5441
0.2694 −0.7348 −39.5149
73.3800 −43.6900 −0.2176 −0.0580 −
0.3744 0.1627 0.1456 −2.8489 −0.5485
0.2628 −0.7307 −39.5137
73.4000 −43.6900 0.0738 −0.1313 −
0.4060 0.0952 0.1262 −2.8334 −0.5525
0.2563 −0.7266 −39.5125
73.4200 −43.7500 0.1202 −0.1654 −
0.3815 0.0449 0.1025 −2.8148 −0.5560
0.2496 −0.7225 −39.5113
73.4400 −43.8000 0.0212 −0.1495 −
0.3094 0.0133 0.0746 −2.7933 −0.5590
0.2430 −0.7184 −39.5102
73.4600 −43.8000 −0.2623 −0.0243 −
0.2084 0.0001 0.0427 −2.7690 −0.5616
0.2363 −0.7143 −39.5090

73.4800 -43.0200 0.2245 0.2039 -
0.1048 0.0037 0.0073 -2.7418 -0.5637
0.2295 -0.7102 -39.5078
73.5000 -42.8900 0.0169 0.3277 -
0.0203 0.0214 -0.0310 -2.7118 -0.5654
0.2228 -0.7060 -39.5066
73.5200 -43.0100 0.0669 0.1894
0.0342 0.0496 -0.0716 -2.6793 -0.5667
0.2160 -0.7019 -39.5054
73.5400 -43.3600 -0.1644 -0.0824
0.0655 0.0834 -0.1136 -2.6442 -0.5676
0.2092 -0.6977 -39.5042
73.5600 -43.3600 -0.0601 -0.2458
0.0892 0.1174 -0.1563 -2.6066 -0.5680
0.2023 -0.6936 -39.5030
73.5800 -43.2500 0.1042 -0.2291
0.1217 0.1462 -0.1987 -2.5667 -0.5681
0.1955 -0.6894 -39.5018
73.6000 -43.1300 0.0427 -0.1282
0.1711 0.1650 -0.2400 -2.5244 -0.5677
0.1886 -0.6853 -39.5006
73.6200 -43.1300 -0.1502 -0.0097
0.2291 0.1708 -0.2794 -2.4799 -0.5670
0.1816 -0.6811 -39.4994
73.6400 -42.7500 0.0087 0.1201
0.2743 0.1616 -0.3161 -2.4332 -0.5658
0.1747 -0.6769 -39.4982
73.6600 -42.6200 0.1185 0.1984
0.2844 0.1362 -0.3494 -2.3845 -0.5643
0.1677 -0.6727 -39.4970
73.6800 -42.7600 0.0695 0.1635
0.2449 0.0943 -0.3788 -2.3337 -0.5624
0.1607 -0.6686 -39.4958
73.7000 -43.1900 -0.1707 0.0695
0.1561 0.0369 -0.4038 -2.2810 -0.5602
0.1537 -0.6644 -39.4946
73.7200 -43.1900 0.0437 0.0249
0.0321 -0.0334 -0.4242 -2.2265 -0.5575
0.1466 -0.6602 -39.4934
73.7400 -43.4400 -0.0383 0.0257 -
0.0967 -0.1130 -0.4396 -2.1702 -0.5545
0.1395 -0.6560 -39.4923
73.7600 -43.5200 0.0433 -0.0367 -
0.1930 -0.1982 -0.4497 -2.1122 -0.5512
0.1324 -0.6518 -39.4911
73.7800 -43.6100 0.1988 -0.2228 -
0.2300 -0.2852 -0.4544 -2.0527 -0.5476
0.1253 -0.6475 -39.4899
73.8000 -44.1800 -0.1347 -0.4094 -
0.1957 -0.3701 -0.4535 -1.9916 -0.5436
0.1182 -0.6433 -39.4887

73.8200 -44.1800 -0.3480 -0.3408 -
0.1002 -0.4498 -0.4466 -1.9291 -0.5392
0.1110 -0.6391 -39.4875
73.8400 -43.2000 0.1442 0.0243
0.0249 -0.5220 -0.4337 -1.8653 -0.5346
0.1039 -0.6349 -39.4863
73.8600 -42.6100 0.1903 0.3901
0.1382 -0.5842 -0.4146 -1.8003 -0.5296
0.0967 -0.6306 -39.4851
73.8800 -42.6300 -0.1080 0.4794
0.2005 -0.6342 -0.3893 -1.7341 -0.5244
0.0895 -0.6264 -39.4839
73.9000 -42.7600 -0.0506 0.2593
0.1922 -0.6699 -0.3580 -1.6668 -0.5188
0.0823 -0.6222 -39.4827
73.9200 -42.7600 0.3472 -0.0980
0.1228 -0.6889 -0.3208 -1.5984 -0.5130
0.0751 -0.6179 -39.4815
73.9400 -43.6100 -0.1848 -0.3776
0.0135 -0.6898 -0.2783 -1.5291 -0.5068
0.0678 -0.6137 -39.4804
73.9600 -43.8900 -0.4713 -0.3853 -
0.1101 -0.6716 -0.2307 -1.4589 -0.5004
0.0606 -0.6094 -39.4792
73.9800 -43.0000 0.0129 -0.1293 -
0.2201 -0.6344 -0.1788 -1.3879 -0.4937
0.0533 -0.6051 -39.4780
74.0000 -42.1100 0.6066 0.1153 -
0.2930 -0.5786 -0.1231 -1.3161 -0.4868
0.0461 -0.6009 -39.4768
74.0200 -42.3600 0.2751 0.1000 -
0.3137 -0.5050 -0.0642 -1.2437 -0.4796
0.0388 -0.5966 -39.4756
74.0400 -42.8500 -0.3593 -0.0841 -
0.2881 -0.4157 -0.0029 -1.1706 -0.4722
0.0315 -0.5923 -39.4744
74.0600 -42.5500 -0.3899 -0.1051 -
0.2355 -0.3129 0.0601 -1.0970 -0.4645
0.0242 -0.5881 -39.4732
74.0800 -41.3700 0.2283 0.1237 -
0.1773 -0.1993 0.1241 -1.0229 -0.4566
0.0169 -0.5838 -39.4721
74.1000 -41.1500 0.0049 0.3583 -
0.1248 -0.0785 0.1883 -0.9484 -0.4484
0.0096 -0.5795 -39.4709
74.1200 -40.5800 0.1831 0.3257 -
0.0815 0.0454 0.2521 -0.8736 -0.4401
0.0023 -0.5752 -39.4697
74.1400 -40.8000 -0.0487 0.0356 -
0.0408 0.1670 0.3147 -0.7985 -0.4315
-0.0050 -0.5709 -39.4685

74.1600 -41.0200 -0.1403 -0.3125
0.0128 0.2809 0.3753 -0.7233 -0.4228
-0.0123 -0.5666 -39.4673
74.1800 -40.6200 -0.0376 -0.5001
0.0916 0.3822 0.4333 -0.6479 -0.4138
-0.0196 -0.5623 -39.4661
74.2000 -40.3200 0.0456 -0.4277
0.1931 0.4666 0.4878 -0.5724 -0.4046
-0.0269 -0.5580 -39.4649
74.2200 -39.7100 -0.0141 -0.1462
0.2989 0.5310 0.5383 -0.4970 -0.3953
-0.0341 -0.5537 -39.4638
74.2400 -39.1500 -0.0283 0.2103
0.3800 0.5731 0.5839 -0.4217 -0.3858
-0.0414 -0.5494 -39.4626
74.2600 -38.7400 -0.0649 0.4832
0.4050 0.5912 0.6241 -0.3466 -0.3761
-0.0487 -0.5451 -39.4614
74.2800 -38.2600 0.4407 0.4824
0.3515 0.5846 0.6585 -0.2717 -0.3663
-0.0560 -0.5408 -39.4602
74.3000 -39.4300 -0.5690 0.2028
0.2218 0.5556 0.6869 -0.1971 -0.3563
-0.0633 -0.5364 -39.4590
74.3200 -39.2200 0.1306 -0.1250
0.0481 0.5081 0.7093 -0.1229 -0.3462
-0.0705 -0.5321 -39.4578
74.3400 -39.3400 0.3997 -0.3371 -
0.1286 0.4462 0.7256 -0.0491 -0.3359
-0.0778 -0.5278 -39.4567
74.3600 -40.3400 -0.4829 -0.3510 -
0.2680 0.3745 0.7360 0.0241 -0.3255
-0.0850 -0.5235 -39.4555
74.3800 -39.4400 0.2952 -0.1678 -
0.3403 0.2973 0.7405 0.0968 -0.3150
-0.0922 -0.5191 -39.4543
74.4000 -39.4200 0.0971 0.0243 -
0.3358 0.2185 0.7393 0.1689 -0.3043
-0.0995 -0.5148 -39.4531
74.4200 -39.6700 -0.3027 0.0893 -
0.2641 0.1412 0.7327 0.2404 -0.2936
-0.1067 -0.5105 -39.4519
74.4400 -39.1300 0.2465 0.0615 -
0.1475 0.0674 0.7208 0.3111 -0.2827
-0.1138 -0.5061 -39.4508
74.4600 -39.1700 0.1226 0.0048 -
0.0160 -0.0013 0.7039 0.3811 -0.2718
-0.1210 -0.5018 -39.4496
74.4800 -39.4700 -0.3179 -0.0308
0.1009 -0.0633 0.6826 0.4503 -0.2608
-0.1282 -0.4974 -39.4484

74.5000 -39.0200 0.1567 0.0100
0.1824 -0.1182 0.6571 0.5187 -0.2496
-0.1353 -0.4931 -39.4472
74.5200 -38.9400 0.0242 0.0457
0.2172 -0.1658 0.6280 0.5862 -0.2385
-0.1424 -0.4887 -39.4460
74.5400 -38.9500 0.0773 0.0176
0.2014 -0.2065 0.5956 0.6529 -0.2272
-0.1495 -0.4844 -39.4449
74.5600 -39.3400 -0.1090 0.0162
0.1421 -0.2407 0.5605 0.7186 -0.2159
-0.1566 -0.4800 -39.4437
74.5800 -39.0100 -0.0033 0.0874
0.0554 -0.2686 0.5231 0.7835 -0.2046
-0.1637 -0.4757 -39.4425
74.6000 -39.1200 0.1176 0.1143 -
0.0356 -0.2906 0.4839 0.8473 -0.1932
-0.1707 -0.4713 -39.4413
74.6200 -39.3800 -0.1234 -0.0006 -
0.1064 -0.3069 0.4435 0.9102 -0.1817
-0.1777 -0.4669 -39.4402
74.6400 -39.4500 0.1340 -0.1593 -
0.1380 -0.3172 0.4022 0.9721 -0.1703
-0.1847 -0.4626 -39.4390
74.6600 -39.8000 -0.3074 -0.1809 -
0.1242 -0.3207 0.3603 1.0330 -0.1588
-0.1916 -0.4582 -39.4378
74.6800 -39.1600 0.0797 -0.0362 -
0.0777 -0.3165 0.3183 1.0929 -0.1473
-0.1986 -0.4539 -39.4366
74.7000 -38.7600 0.2373 0.1331 -
0.0234 -0.3040 0.2763 1.1517 -0.1358
-0.2055 -0.4495 -39.4355
74.7200 -38.9300 -0.0932 0.1792
0.0135 -0.2828 0.2346 1.2094 -0.1244
-0.2123 -0.4451 -39.4343
74.7400 -39.0100 -0.1765 0.1092
0.0211 -0.2534 0.1935 1.2661 -0.1129
-0.2192 -0.4407 -39.4331
74.7600 -38.6700 0.2704 0.0060
0.0045 -0.2168 0.1530 1.3217 -0.1014
-0.2260 -0.4364 -39.4319
74.7800 -39.0600 -0.0352 -0.1079 -
0.0178 -0.1745 0.1132 1.3763 -0.0900
-0.2327 -0.4320 -39.4308
74.8000 -39.0500 -0.1875 -0.1484 -
0.0280 -0.1283 0.0743 1.4298 -0.0786
-0.2395 -0.4276 -39.4296
74.8200 -38.7000 0.1359 -0.0764 -
0.0183 -0.0804 0.0362 1.4822 -0.0673
-0.2462 -0.4232 -39.4284

74.8400 -38.6000 0.0306 -0.0057
0.0078 -0.0332 -0.0010 1.5336 -0.0560
-0.2528 -0.4189 -39.4272
74.8600 -38.4600 0.0266 0.0186
0.0364 0.0108 -0.0374 1.5839 -0.0447
-0.2595 -0.4145 -39.4261
74.8800 -38.4000 0.0456 0.0478
0.0533 0.0496 -0.0729 1.6332 -0.0335
-0.2661 -0.4101 -39.4249
74.9000 -38.4200 -0.1440 0.1024
0.0513 0.0812 -0.1075 1.6815 -0.0224
-0.2726 -0.4057 -39.4237
74.9200 -38.1800 -0.0196 0.1529
0.0335 0.1045 -0.1413 1.7287 -0.0114
-0.2791 -0.4013 -39.4226
74.9400 -37.9400 0.3926 0.0699
0.0128 0.1188 -0.1743 1.7749 -0.0005
-0.2856 -0.3970 -39.4214
74.9600 -38.9600 -0.4582 -0.1413
0.0059 0.1238 -0.2064 1.8202 0.0104
-0.2920 -0.3926 -39.4202
74.9800 -38.1500 0.4655 -0.2581
0.0223 0.1201 -0.2377 1.8644 0.0211
-0.2984 -0.3882 -39.4190
75.0000 -38.8700 -0.4579 -0.1194
0.0580 0.1089 -0.2682 1.9077 0.0318
-0.3047 -0.3838 -39.4179
75.0200 -37.9000 0.2554 0.1563
0.0891 0.0925 -0.2978 1.9501 0.0423
-0.3110 -0.3794 -39.4167
75.0400 -37.6200 0.3130 0.3127
0.0901 0.0735 -0.3267 1.9915 0.0527
-0.3173 -0.3750 -39.4155
75.0600 -38.0500 0.0025 0.2180
0.0517 0.0541 -0.3546 2.0320 0.0630
-0.3235 -0.3706 -39.4144
75.0800 -38.6300 -0.4197 0.0237 -
0.0156 0.0365 -0.3817 2.0716 0.0731
-0.3296 -0.3663 -39.4132
75.1000 -38.7500 -0.1999 -0.1025 -
0.0893 0.0223 -0.4078 2.1103 0.0831
-0.3357 -0.3619 -39.4120
75.1200 -38.0700 0.5663 -0.1852 -
0.1408 0.0129 -0.4329 2.1480 0.0929
-0.3418 -0.3575 -39.4109
75.1400 -39.0100 -0.3640 -0.2411 -
0.1512 0.0086 -0.4569 2.1848 0.1026
-0.3478 -0.3531 -39.4097
75.1600 -38.8300 -0.3270 -0.1783 -
0.1159 0.0089 -0.4798 2.2206 0.1121
-0.3537 -0.3487 -39.4085

75.1800 -37.8900 0.4080 0.0024 -
0.0489 0.0125 -0.5015 2.2554 0.1215
-0.3596 -0.3443 -39.4074
75.2000 -38.0400 -0.1233 0.1587
0.0252 0.0172 -0.5219 2.2892 0.1307
-0.3654 -0.3399 -39.4062
75.2200 -37.9600 -0.0990 0.2001
0.0816 0.0210 -0.5409 2.3220 0.1397
-0.3712 -0.3356 -39.4050
75.2400 -37.7200 0.2294 0.1612
0.1026 0.0222 -0.5582 2.3536 0.1485
-0.3769 -0.3312 -39.4039
75.2600 -38.0400 -0.1019 0.0491
0.0889 0.0193 -0.5739 2.3842 0.1571
-0.3826 -0.3268 -39.4027
75.2800 -38.1600 0.0454 -0.1048
0.0557 0.0118 -0.5878 2.4136 0.1655
-0.3882 -0.3224 -39.4015
75.3000 -38.4600 -0.0774 -0.1853
0.0187 -0.0003 -0.5998 2.4418 0.1737
-0.3937 -0.3180 -39.4004
75.3200 -38.3500 -0.0754 -0.0995 -
0.0119 -0.0165 -0.6097 2.4688 0.1817
-0.3992 -0.3136 -39.3992
75.3400 -37.9000 0.0513 0.0950 -
0.0308 -0.0353 -0.6174 2.4945 0.1895
-0.4046 -0.3092 -39.3980
75.3600 -37.8100 0.1273 0.2033 -
0.0357 -0.0549 -0.6228 2.5188 0.1970
-0.4100 -0.3049 -39.3969
75.3800 -38.0600 -0.0346 0.0875 -
0.0274 -0.0732 -0.6257 2.5418 0.2043
-0.4153 -0.3005 -39.3957
75.4000 -38.2600 -0.0453 -0.1501 -
0.0109 -0.0888 -0.6260 2.5633 0.2114
-0.4205 -0.2961 -39.3946
75.4200 -38.1000 0.1837 -0.2385
0.0041 -0.1001 -0.6234 2.5833 0.2182
-0.4257 -0.2917 -39.3934
75.4400 -38.6800 -0.6125 -0.0120
0.0096 -0.1060 -0.6180 2.6017 0.2248
-0.4307 -0.2873 -39.3922
75.4600 -37.1000 0.6777 0.2711
0.0047 -0.1055 -0.6097 2.6186 0.2311
-0.4358 -0.2830 -39.3911
75.4800 -37.8400 -0.0962 0.1891 -
0.0089 -0.0974 -0.5984 2.6338 0.2372
-0.4407 -0.2786 -39.3899
75.5000 -38.3500 -0.3462 -0.1272 -
0.0234 -0.0815 -0.5840 2.6473 0.2430
-0.4456 -0.2742 -39.3887

75.5200 -38.1800 -0.0992 -0.2859 –
0.0284 -0.0582 -0.5664 2.6590 0.2485
-0.4504 -0.2698 -39.3876
75.5400 -37.9800 -0.1335 -0.1222 –
0.0215 -0.0287 -0.5455 2.6690 0.2538
-0.4552 -0.2654 -39.3864
75.5600 -37.3400 0.1006 0.1436 –
0.0100 0.0055 -0.5212 2.6770 0.2588
-0.4598 -0.2611 -39.3853
75.5800 -37.1400 0.2422 0.2273 –
0.0036 0.0422 -0.4933 2.6831 0.2636
-0.4644 -0.2567 -39.3841
75.6000 -37.4200 0.0959 0.0644 –
0.0074 0.0789 -0.4618 2.6873 0.2680
-0.4689 -0.2523 -39.3830
75.6200 -37.8600 -0.3286 -0.1287 –
0.0177 0.1128 -0.4264 2.6894 0.2722
-0.4734 -0.2480 -39.3818
75.6400 -37.5700 -0.0319 -0.1411 –
0.0241 0.1408 -0.3871 2.6894 0.2761
-0.4777 -0.2436 -39.3806
75.6600 -37.0600 0.2651 -0.0284 –
0.0168 0.1604 -0.3437 2.6872 0.2798
-0.4820 -0.2392 -39.3795
75.6800 -37.2000 -0.0751 0.0367
0.0096 0.1690 -0.2962 2.6829 0.2831
-0.4862 -0.2349 -39.3783
75.7000 -37.0500 0.0382 0.0068
0.0479 0.1645 -0.2445 2.6762 0.2862
-0.4904 -0.2305 -39.3772
75.7200 -37.1500 -0.1302 0.0081
0.0841 0.1454 -0.1888 2.6672 0.2889
-0.4944 -0.2261 -39.3760
75.7400 -36.9000 0.0876 0.0749
0.1061 0.1113 -0.1291 2.6558 0.2914
-0.4984 -0.2218 -39.3749
75.7600 -36.9900 0.0579 0.0993
0.1057 0.0627 -0.0657 2.6420 0.2936
-0.5022 -0.2174 -39.3737
75.7800 -36.9400 0.1218 -0.0116
0.0848 0.0015 0.0010 2.6256 0.2956
-0.5060 -0.2131 -39.3725
75.8000 -37.5500 -0.2168 -0.1305
0.0501 -0.0692 0.0704 2.6066 0.2972
-0.5098 -0.2087 -39.3714
75.8200 -37.4300 -0.1417 -0.1001
0.0105 -0.1453 0.1420 2.5849 0.2986
-0.5134 -0.2044 -39.3702
75.8400 -36.9600 0.2246 0.0424 –
0.0271 -0.2227 0.2151 2.5605 0.2997
-0.5170 -0.2000 -39.3691

75.8600 -37.2600 -0.1073 0.1128 -
0.0600 -0.2967 0.2890 2.5333 0.3005
-0.5204 -0.1957 -39.3679
75.8800 -37.4600 -0.0572 0.0730 -
0.0876 -0.3628 0.3631 2.5032 0.3010
-0.5238 -0.1913 -39.3668
75.9000 -37.3100 0.0112 0.0056 -
0.1059 -0.4171 0.4366 2.4702 0.3013
-0.5271 -0.1870 -39.3656
75.9200 -37.5200 -0.0440 -0.0409 -
0.1120 -0.4562 0.5088 2.4343 0.3013
-0.5303 -0.1827 -39.3645
75.9400 -37.3100 0.1628 -0.0729 -
0.1024 -0.4779 0.5792 2.3954 0.3011
-0.5334 -0.1783 -39.3633
75.9600 -37.5300 -0.0326 -0.0690 -
0.0766 -0.4808 0.6471 2.3534 0.3006
-0.5365 -0.1740 -39.3622
75.9800 -37.5400 -0.3175 0.0016 -
0.0368 -0.4647 0.7119 2.3083 0.2998
-0.5394 -0.1697 -39.3610
76.0000 -36.5800 0.4493 0.0703
0.0125 -0.4300 0.7733 2.2601 0.2988
-0.5423 -0.1653 -39.3599
76.0200 -37.2100 -0.2092 0.0143
0.0656 -0.3778 0.8308 2.2088 0.2976
-0.5450 -0.1610 -39.3587
76.0400 -37.1400 -0.1486 -0.1079
0.1154 -0.3093 0.8837 2.1543 0.2961
-0.5477 -0.1567 -39.3576
76.0600 -36.7900 0.1774 -0.1270
0.1500 -0.2259 0.9316 2.0966 0.2944
-0.5503 -0.1524 -39.3564
76.0800 -36.9400 -0.1723 -0.0045
0.1559 -0.1286 0.9737 2.0357 0.2924
-0.5528 -0.1481 -39.3553
76.1000 -36.3600 0.1878 0.1547
0.1184 -0.0189 1.0095 1.9716 0.2902
-0.5552 -0.1437 -39.3541
76.1200 -36.3700 0.0506 0.1933
0.0309 0.1006 1.0383 1.9045 0.2878
-0.5576 -0.1394 -39.3530
76.1400 -36.7300 -0.0306 0.0647 -
0.0947 0.2255 1.0595 1.8342 0.2851
-0.5598 -0.1351 -39.3518
76.1600 -36.8800 0.0754 -0.1010 -
0.2314 0.3509 1.0724 1.7610 0.2823
-0.5620 -0.1308 -39.3507
76.1800 -37.1200 -0.1300 -0.1582 -
0.3447 0.4715 1.0765 1.6848 0.2792
-0.5640 -0.1265 -39.3495

76.2000 -36.8100 0.1416 -0.0872 -
0.4051 0.5822 1.0713 1.6058 0.2759
-0.5660 -0.1222 -39.3484
76.2200 -36.9200 -0.0790 0.0003 -
0.3932 0.6783 1.0564 1.5241 0.2724
-0.5679 -0.1179 -39.3472
76.2400 -36.6300 0.1860 0.0229 -
0.2999 0.7554 1.0313 1.4399 0.2687
-0.5697 -0.1136 -39.3461
76.2600 -36.9400 -0.2012 -0.0076 -
0.1337 0.8100 0.9961 1.3533 0.2648
-0.5714 -0.1093 -39.3449
76.2800 -36.5500 0.1426 -0.0663
0.0825 0.8385 0.9508 1.2645 0.2607
-0.5730 -0.1051 -39.3438
76.3000 -36.6700 -0.0057 -0.1141
0.3193 0.8380 0.8956 1.1738 0.2565
-0.5745 -0.1008 -39.3427
76.3200 -36.6400 -0.1680 -0.0622
0.5404 0.8057 0.8311 1.0812 0.2520
-0.5760 -0.0965 -39.3415
76.3400 -36.2900 0.2521 0.0896
0.7096 0.7397 0.7580 0.9871 0.2474
-0.5773 -0.0922 -39.3404
76.3600 -36.5600 -0.0860 0.2687
0.7944 0.6393 0.6774 0.8916 0.2426
-0.5786 -0.0880 -39.3392
76.3800 -36.8600 -0.1566 0.4510
0.7687 0.5069 0.5903 0.7948 0.2376
-0.5798 -0.0837 -39.3381
76.4000 -37.0100 -0.0221 0.5971
0.6242 0.3479 0.4980 0.6971 0.2325
-0.5809 -0.0794 -39.3369
76.4200 -37.3900 0.2560 0.5689
0.3755 0.1698 0.4017 0.5986 0.2272
-0.5818 -0.0752 -39.3358
76.4400 -38.6200 -0.0137 0.2903
0.0593 -0.0190 0.3025 0.4995 0.2217
-0.5828 -0.0709 -39.3347
76.4600 -39.9800 -0.0904 -0.1510 -
0.2756 -0.2097 0.2016 0.3999 0.2161
-0.5836 -0.0667 -39.3335
76.4800 -40.8300 -0.0030 -0.5488 -
0.5788 -0.3938 0.1002 0.3002 0.2103
-0.5843 -0.0624 -39.3324
76.5000 -41.6300 -0.0441 -0.7462 -
0.8009 -0.5626 -0.0004 0.2005 0.2044
-0.5850 -0.0582 -39.3312
76.5200 -42.0200 0.1422 -0.7330 -
0.9053 -0.7077 -0.0991 0.1010 0.1984
-0.5855 -0.0539 -39.3301

76.5400 -42.3600 -0.1790 -0.5501 -
0.8849 -0.8215 -0.1947 0.0018 0.1922
-0.5860 -0.0497 -39.3290
76.5600 -42.0800 -0.1239 -0.1938 -
0.7632 -0.8992 -0.2860 -0.0967 0.1859
-0.5864 -0.0455 -39.3278
76.5800 -41.4300 0.1394 0.2848 -
0.5790 -0.9396 -0.3718 -0.1945 0.1795
-0.5867 -0.0413 -39.3267
76.6000 -41.3100 -0.1944 0.6782 -
0.3733 -0.9435 -0.4513 -0.2912 0.1729
-0.5869 -0.0370 -39.3255
76.6200 -40.8900 0.2703 0.7103 -
0.1832 -0.9130 -0.5238 -0.3868 0.1663
-0.5870 -0.0328 -39.3244
76.6400 -41.0500 0.3235 0.3057 -
0.0316 -0.8512 -0.5890 -0.4810 0.1595
-0.5870 -0.0286 -39.3233
76.6600 -42.3500 -0.4204 -0.2645
0.0823 -0.7635 -0.6469 -0.5735 0.1526
-0.5870 -0.0244 -39.3221
76.6800 -42.3500 -0.2037 -0.5302
0.1694 -0.6566 -0.6972 -0.6644 0.1456
-0.5869 -0.0202 -39.3210
76.7000 -41.6500 0.3582 -0.3828
0.2463 -0.5381 -0.7403 -0.7532 0.1385
-0.5866 -0.0160 -39.3199
76.7200 -41.6500 -0.0252 -0.0974
0.3279 -0.4158 -0.7760 -0.8399 0.1313
-0.5863 -0.0118 -39.3187
76.7400 -41.1900 0.0172 0.1008
0.4171 -0.2962 -0.8046 -0.9243 0.1241
-0.5859 -0.0076 -39.3176
76.7600 -41.1300 -0.0557 0.1600
0.5059 -0.1843 -0.8262 -1.0063 0.1167
-0.5855 -0.0034 -39.3164
76.7800 -41.0700 -0.0079 0.1355
0.5738 -0.0837 -0.8410 -1.0856 0.1093
-0.5849 0.0008 -39.3153
76.8000 -41.0500 0.0041 0.0878
0.5968 0.0029 -0.8492 -1.1621 0.1018
-0.5842 0.0049 -39.3142
76.8200 -41.2100 -0.0168 0.0574
0.5588 0.0744 -0.8514 -1.2358 0.0942
-0.5835 0.0091 -39.3130
76.8400 -41.3100 -0.0365 0.0941
0.4545 0.1311 -0.8480 -1.3064 0.0865
-0.5827 0.0133 -39.3119
76.8600 -41.3800 -0.0374 0.2067
0.2925 0.1749 -0.8398 -1.3738 0.0788
-0.5818 0.0174 -39.3108

76.8800 -41.5200 0.0110 0.2785
0.0951 0.2081 -0.8273 -1.4378 0.0710
-0.5808 0.0216 -39.3096
76.9000 -41.7300 0.1853 0.1722 -
0.1080 0.2335 -0.8113 -1.4984 0.0632
-0.5797 0.0257 -39.3085
76.9200 -42.4700 -0.2116 -0.1072 -
0.2843 0.2539 -0.7924 -1.5555 0.0554
-0.5786 0.0299 -39.3074
76.9400 -42.5300 0.1700 -0.4482 -
0.4044 0.2722 -0.7715 -1.6088 0.0474
-0.5774 0.0340 -39.3063
76.9600 -43.0200 -0.1223 -0.6578 -
0.4422 0.2907 -0.7493 -1.6583 0.0395
-0.5760 0.0382 -39.3051
76.9800 -43.0200 -0.2324 -0.5752 -
0.3833 0.3113 -0.7266 -1.7038 0.0315
-0.5746 0.0423 -39.3040
77.0000 -42.0600 0.3197 -0.1863 -
0.2509 0.3349 -0.7042 -1.7453 0.0235
-0.5732 0.0464 -39.3029
77.0200 -41.9400 -0.2704 0.3178 -
0.0872 0.3618 -0.6824 -1.7825 0.0154
-0.5716 0.0505 -39.3017
77.0400 -41.0600 0.0916 0.7021
0.0608 0.3922 -0.6619 -1.8156 0.0074
-0.5700 0.0546 -39.3006
77.0600 -40.6800 0.1983 0.7645
0.1502 0.4260 -0.6427 -1.8442 -0.0007
-0.5682 0.0587 -39.2995
77.0800 -41.3300 -0.0488 0.4778
0.1628 0.4628 -0.6249 -1.8685 -0.0088
-0.5664 0.0628 -39.2984
77.1000 -41.7700 -0.2127 0.0584
0.1104 0.5009 -0.6086 -1.8884 -0.0169
-0.5645 0.0669 -39.2972
77.1200 -41.9800 0.0666 -0.2576
0.0199 0.5377 -0.5936 -1.9039 -0.0250
-0.5626 0.0710 -39.2961
77.1400 -42.2400 0.0874 -0.4466 -
0.0715 0.5705 -0.5795 -1.9150 -0.0332
-0.5605 0.0751 -39.2950
77.1600 -42.2900 0.1412 -0.5502 -
0.1285 0.5964 -0.5661 -1.9219 -0.0413
-0.5584 0.0792 -39.2938
77.1800 -42.4000 -0.1516 -0.4802 -
0.1312 0.6127 -0.5530 -1.9247 -0.0494
-0.5562 0.0833 -39.2927
77.2000 -42.1100 -0.2882 -0.1786 -
0.0832 0.6170 -0.5397 -1.9235 -0.0575
-0.5540 0.0873 -39.2916

77.2200 -41.1100 0.3661 0.1835 -
0.0048 0.6077 -0.5259 -1.9185 -0.0656
-0.5516 0.0914 -39.2905
77.2400 -41.1100 0.1078 0.3322
0.0791 0.5833 -0.5113 -1.9097 -0.0737
-0.5492 0.0955 -39.2893
77.2600 -41.4700 -0.1872 0.2399
0.1442 0.5427 -0.4954 -1.8974 -0.0818
-0.5467 0.0995 -39.2882
77.2800 -41.4700 -0.0907 0.1214
0.1740 0.4851 -0.4779 -1.8817 -0.0898
-0.5441 0.1035 -39.2871
77.3000 -41.5200 0.0142 0.0846
0.1664 0.4109 -0.4587 -1.8627 -0.0979
-0.5414 0.1076 -39.2860
77.3200 -41.5400 0.1327 0.0100
0.1339 0.3211 -0.4375 -1.8406 -0.1059
-0.5387 0.1116 -39.2848
77.3400 -41.8500 -0.0253 -0.1394
0.0923 0.2179 -0.4142 -1.8156 -0.1139
-0.5359 0.1156 -39.2837
77.3600 -42.2300 -0.1223 -0.2189
0.0551 0.1040 -0.3887 -1.7878 -0.1218
-0.5330 0.1197 -39.2826
77.3800 -42.1200 -0.0760 -0.0950
0.0321 -0.0174 -0.3608 -1.7574 -0.1298
-0.5301 0.1237 -39.2815
77.4000 -41.7200 0.1543 0.1066
0.0220 -0.1424 -0.3306 -1.7245 -0.1377
-0.5271 0.1277 -39.2804
77.4200 -41.7200 0.2324 0.1311
0.0145 -0.2665 -0.2978 -1.6893 -0.1455
-0.5240 0.1317 -39.2792
77.4400 -42.3600 -0.3469 -0.0124 -
0.0036 -0.3852 -0.2625 -1.6520 -0.1534
-0.5208 0.1357 -39.2781
77.4600 -42.3600 -0.1030 -0.0441 -
0.0395 -0.4939 -0.2246 -1.6127 -0.1612
-0.5176 0.1397 -39.2770
77.4800 -42.1100 0.1453 0.0907 -
0.0903 -0.5881 -0.1839 -1.5717 -0.1689
-0.5143 0.1436 -39.2759
77.5000 -42.0200 0.1024 0.1541 -
0.1497 -0.6642 -0.1406 -1.5289 -0.1766
-0.5109 0.1476 -39.2748
77.5200 -42.3400 0.0043 0.0098 -
0.2041 -0.7187 -0.0946 -1.4847 -0.1843
-0.5075 0.1516 -39.2736
77.5400 -42.7400 -0.2302 -0.1662 -
0.2360 -0.7492 -0.0460 -1.4390 -0.1919
-0.5040 0.1555 -39.2725

77.5600 -42.4900 -0.0549 -0.1661 -
0.2358 -0.7539 0.0047 -1.3921 -0.1994
-0.5004 0.1595 -39.2714
77.5800 -41.9200 0.1952 -0.0570 -
0.2011 -0.7327 0.0573 -1.3440 -0.2070
-0.4967 0.1634 -39.2703
77.6000 -41.8600 -0.0299 -0.0109 -
0.1379 -0.6869 0.1113 -1.2948 -0.2144
-0.4930 0.1674 -39.2692
77.6200 -41.6400 0.0008 -0.0176 -
0.0613 -0.6190 0.1661 -1.2447 -0.2218
-0.4892 0.1713 -39.2681
77.6400 -41.4400 -0.2295 0.0604
0.0082 -0.5321 0.2213 -1.1936 -0.2291
-0.4854 0.1752 -39.2669
77.6600 -40.6200 0.2557 0.1790
0.0543 -0.4299 0.2764 -1.1417 -0.2364
-0.4815 0.1792 -39.2658
77.6800 -40.6900 0.0613 0.1873
0.0705 -0.3161 0.3308 -1.0891 -0.2435
-0.4775 0.1831 -39.2647
77.7000 -40.8600 -0.4238 0.0620
0.0632 -0.1948 0.3839 -1.0360 -0.2506
-0.4735 0.1870 -39.2636
77.7200 -40.4300 -0.0289 -0.1326
0.0490 -0.0703 0.4353 -0.9823 -0.2577
-0.4694 0.1909 -39.2625
77.7400 -39.6900 0.8269 -0.2996
0.0461 0.0535 0.4845 -0.9281 -0.2646
-0.4652 0.1948 -39.2614
77.7600 -40.4100 -0.1380 -0.3127
0.0676 0.1730 0.5310 -0.8737 -0.2715
-0.4610 0.1986 -39.2603
77.7800 -40.8200 -1.1022 -0.0437
0.1082 0.2855 0.5742 -0.8190 -0.2783
-0.4567 0.2025 -39.2591
77.8000 -38.0100 1.0794 0.3473
0.1467 0.3889 0.6136 -0.7641 -0.2850
-0.4523 0.2064 -39.2580
77.8200 -38.7100 0.1510 0.3669
0.1609 0.4815 0.6487 -0.7092 -0.2916
-0.4479 0.2102 -39.2569
77.8400 -39.5100 -0.5616 0.0561
0.1425 0.5615 0.6790 -0.6544 -0.2981
-0.4435 0.2141 -39.2558
77.8600 -39.2400 -0.1436 -0.2458
0.1011 0.6270 0.7041 -0.5997 -0.3046
-0.4389 0.2179 -39.2547
77.8800 -39.1400 -0.1509 -0.2311
0.0512 0.6765 0.7234 -0.5452 -0.3109
-0.4343 0.2218 -39.2536

77.9000 -38.7700 0.0076 -0.0194
0.0070 0.7084 0.7367 -0.4911 -0.3171
-0.4297 0.2256 -39.2525
77.9200 -38.4400 0.2409 0.1212 -
0.0159 0.7219 0.7435 -0.4373 -0.3232
-0.4250 0.2294 -39.2514
77.9400 -38.7600 -0.1754 0.1069 -
0.0086 0.7164 0.7439 -0.3841 -0.3293
-0.4202 0.2333 -39.2503
77.9600 -38.5000 0.0267 0.0627
0.0271 0.6917 0.7377 -0.3314 -0.3352
-0.4154 0.2371 -39.2492
77.9800 -38.5200 0.0748 0.0196
0.0850 0.6478 0.7253 -0.2794 -0.3409
-0.4106 0.2409 -39.2480
78.0000 -38.5900 -0.0029 -0.0636
0.1553 0.5854 0.7069 -0.2281 -0.3466
-0.4056 0.2447 -39.2469
78.0200 -38.6200 0.0347 -0.1547
0.2240 0.5060 0.6831 -0.1776 -0.3522
-0.4007 0.2484 -39.2458
78.0400 -38.6400 -0.0037 -0.1492
0.2745 0.4118 0.6544 -0.1279 -0.3576
-0.3956 0.2522 -39.2447
78.0600 -38.7600 -0.1663 0.0108
0.2902 0.3056 0.6213 -0.0790 -0.3629
-0.3905 0.2560 -39.2436
78.0800 -38.4600 0.0693 0.2168
0.2569 0.1908 0.5843 -0.0312 -0.3680
-0.3854 0.2597 -39.2425
78.1000 -38.5800 0.1144 0.2642
0.1688 0.0713 0.5441 0.0158 -0.3731
-0.3802 0.2635 -39.2414
78.1200 -39.0600 0.0214 0.1029
0.0367 -0.0486 0.5012 0.0616 -0.3780
-0.3750 0.2672 -39.2403
78.1400 -39.7000 -0.2355 -0.0801 -
0.1127 -0.1642 0.4561 0.1064 -0.3827
-0.3697 0.2710 -39.2392
78.1600 -39.7400 0.1197 -0.1543 -
0.2467 -0.2707 0.4093 0.1501 -0.3874
-0.3643 0.2747 -39.2381
78.1800 -40.0800 -0.0470 -0.1904 -
0.3343 -0.3634 0.3614 0.1927 -0.3918
-0.3589 0.2784 -39.2370
78.2000 -40.1700 0.0480 -0.2271 -
0.3569 -0.4381 0.3130 0.2340 -0.3962
-0.3535 0.2821 -39.2359
78.2200 -40.0400 0.1663 -0.2149 -
0.3134 -0.4922 0.2645 0.2742 -0.4004
-0.3480 0.2858 -39.2348

78.2400 -40.4000 -0.3565 -0.0618 -
0.2232 -0.5250 0.2166 0.3130 -0.4044
-0.3425 0.2895 -39.2337
78.2600 -39.8800 -0.1414 0.2022 -
0.1163 -0.5378 0.1697 0.3506 -0.4082
-0.3369 0.2932 -39.2326
78.2800 -38.8400 0.5925 0.3600 -
0.0237 -0.5326 0.1244 0.3868 -0.4120
-0.3313 0.2969 -39.2315
78.3000 -39.7100 -0.1560 0.1953
0.0330 -0.5119 0.0814 0.4217 -0.4155
-0.3256 0.3005 -39.2304
78.3200 -39.9500 -0.2118 -0.1146
0.0558 -0.4795 0.0410 0.4551 -0.4189
-0.3199 0.3042 -39.2293
78.3400 -40.0100 -0.2449 -0.2431
0.0582 -0.4398 0.0038 0.4871 -0.4221
-0.3141 0.3079 -39.2282
78.3600 -39.4900 0.2221 -0.1577
0.0567 -0.3973 -0.0298 0.5177 -0.4251
-0.3083 0.3115 -39.2271
78.3800 -39.4300 -0.0341 -0.0348
0.0656 -0.3559 -0.0594 0.5467 -0.4280
-0.3024 0.3151 -39.2260
78.4000 -39.2900 0.0098 0.0135
0.0921 -0.3188 -0.0845 0.5743 -0.4307
-0.2966 0.3187 -39.2249
78.4200 -39.1200 0.0864 -0.0069
0.1301 -0.2881 -0.1049 0.6003 -0.4332
-0.2906 0.3224 -39.2238
78.4400 -39.3100 -0.0878 -0.0075
0.1650 -0.2649 -0.1203 0.6248 -0.4355
-0.2846 0.3260 -39.2227
78.4600 -39.0900 -0.0314 0.0514
0.1810 -0.2492 -0.1306 0.6477 -0.4377
-0.2786 0.3296 -39.2216
78.4800 -38.9900 -0.0034 0.1051
0.1679 -0.2400 -0.1360 0.6691 -0.4396
-0.2726 0.3331 -39.2205
78.5000 -38.9800 0.1707 0.0803
0.1228 -0.2352 -0.1365 0.6889 -0.4414
-0.2665 0.3367 -39.2194
78.5200 -39.2800 -0.1142 -0.0220
0.0542 -0.2323 -0.1324 0.7071 -0.4429
-0.2604 0.3403 -39.2183
78.5400 -39.3400 -0.0121 -0.1165 -
0.0171 -0.2285 -0.1241 0.7238 -0.4443
-0.2542 0.3439 -39.2172
78.5600 -39.3600 -0.0121 -0.1246 -
0.0695 -0.2210 -0.1120 0.7388 -0.4455
-0.2480 0.3474 -39.2161

78.5800 -39.3200 -0.0563 -0.0378 -
0.0917 -0.2079 -0.0965 0.7523 -0.4465
-0.2417 0.3509 -39.2150
78.6000 -39.0900 -0.0281 0.0570 -
0.0873 -0.1874 -0.0781 0.7643 -0.4472
-0.2355 0.3545 -39.2139
78.6200 -38.7900 0.1920 0.0895 -
0.0704 -0.1591 -0.0573 0.7747 -0.4478
-0.2292 0.3580 -39.2128
78.6400 -38.9200 -0.1089 0.1134 -
0.0576 -0.1234 -0.0345 0.7836 -0.4482
-0.2228 0.3615 -39.2117
78.6600 -39.0200 -0.3510 0.1421 -
0.0568 -0.0814 -0.0100 0.7910 -0.4483
-0.2164 0.3650 -39.2106
78.6800 -38.2300 0.5920 0.0598 -
0.0584 -0.0352 0.0157 0.7970 -0.4483
-0.2100 0.3685 -39.2096
78.7000 -39.3300 -0.4740 -0.1848 -
0.0475 0.0125 0.0424 0.8016 -0.4481
-0.2036 0.3720 -39.2085
78.7200 -39.0900 -0.1808 -0.2964 -
0.0125 0.0587 0.0697 0.8047 -0.4476
-0.1971 0.3754 -39.2074
78.7400 -38.5500 -0.0641 -0.0820
0.0475 0.1002 0.0972 0.8065 -0.4470
-0.1906 0.3789 -39.2063
78.7600 -37.9900 0.0758 0.2173
0.1135 0.1343 0.1248 0.8070 -0.4462
-0.1841 0.3824 -39.2052
78.7800 -37.4700 0.4071 0.2724
0.1570 0.1587 0.1519 0.8062 -0.4451
-0.1775 0.3858 -39.2041
78.8000 -38.4300 -0.4097 0.0955
0.1568 0.1716 0.1784 0.8041 -0.4439
-0.1709 0.3892 -39.2030
78.8200 -38.1100 0.0778 -0.0337
0.1147 0.1727 0.2038 0.8008 -0.4424
-0.1643 0.3927 -39.2019
78.8400 -38.0800 0.0783 -0.0307
0.0513 0.1635 0.2280 0.7962 -0.4408
-0.1576 0.3961 -39.2008
78.8600 -38.2000 0.0243 -0.0335 -
0.0086 0.1462 0.2506 0.7906 -0.4390
-0.1510 0.3995 -39.1997
78.8800 -38.3900 0.0209 -0.1117 -
0.0430 0.1235 0.2713 0.7838 -0.4369
-0.1443 0.4029 -39.1987
78.9000 -38.5500 -0.1087 -0.1543 -
0.0413 0.0983 0.2899 0.7759 -0.4347
-0.1375 0.4062 -39.1976

78.9200 -38.3800 -0.1993 -0.0453 -
0.0175 0.0744 0.3061 0.7669 -0.4323
-0.1308 0.4096 -39.1965
78.9400 -37.6400 0.4867 0.1180
0.0055 0.0557 0.3198 0.7570 -0.4297
-0.1240 0.4130 -39.1954
78.9600 -38.4900 -0.4125 0.1420
0.0057 0.0455 0.3306 0.7460 -0.4270
-0.1172 0.4163 -39.1943
78.9800 -38.0900 0.1309 0.0882 -
0.0276 0.0462 0.3384 0.7342 -0.4240
-0.1104 0.4197 -39.1932
79.0000 -38.0500 0.1895 0.0312 -
0.0908 0.0579 0.3430 0.7214 -0.4209
-0.1035 0.4230 -39.1921
79.0200 -38.2900 0.0130 -0.0346 -
0.1694 0.0790 0.3442 0.7078 -0.4177
-0.0967 0.4263 -39.1911
79.0400 -38.5000 0.0528 -0.0869 -
0.2385 0.1070 0.3421 0.6933 -0.4142
-0.0898 0.4296 -39.1900
79.0600 -38.7100 -0.3123 -0.0738 -
0.2698 0.1390 0.3365 0.6781 -0.4106
-0.0829 0.4329 -39.1889
79.0800 -38.2300 0.2033 -0.0443 -
0.2414 0.1720 0.3276 0.6622 -0.4068
-0.0760 0.4362 -39.1878
79.1000 -38.0200 0.2961 -0.1124 -
0.1424 0.2028 0.3154 0.6456 -0.4029
-0.0690 0.4395 -39.1867
79.1200 -38.5800 -0.3047 -0.2261
0.0131 0.2284 0.2999 0.6284 -0.3988
-0.0620 0.4427 -39.1856
79.1400 -38.3600 -0.2444 -0.2022
0.1880 0.2457 0.2813 0.6106 -0.3946
-0.0551 0.4460 -39.1846
79.1600 -37.7200 -0.0598 0.0492
0.3417 0.2522 0.2596 0.5922 -0.3903
-0.0481 0.4492 -39.1835
79.1800 -37.2900 0.0280 0.3687
0.4344 0.2451 0.2351 0.5734 -0.3858
-0.0410 0.4524 -39.1824
79.2000 -37.1400 0.1414 0.4625
0.4324 0.2227 0.2079 0.5542 -0.3811
-0.0340 0.4557 -39.1813
79.2200 -37.2500 0.4286 0.1963
0.3281 0.1861 0.1786 0.5346 -0.3764
-0.0269 0.4589 -39.1802
79.2400 -38.6600 -0.2788 -0.2150
0.1585 0.1392 0.1474 0.5147 -0.3715
-0.0199 0.4621 -39.1792

79.2600 -39.1400 -0.3661 -0.3824 -
0.0290 0.0864 0.1148 0.4945 -0.3664
-0.0128 0.4652 -39.1781
79.2800 -39.2000 -0.2988 -0.2234 -
0.1929 0.0323 0.0813 0.4740 -0.3613
-0.0057 0.4684 -39.1770
79.3000 -38.2000 0.7347 -0.0201 -
0.3063 -0.0188 0.0473 0.4534 -0.3560
0.0014 0.4716 -39.1759
79.3200 -39.3000 -0.4419 0.0864 -
0.3602 -0.0631 0.0132 0.4327 -0.3507
0.0085 0.4747 -39.1748
79.3400 -39.4100 -0.4088 0.1327 -
0.3601 -0.0981 -0.0206 0.4119 -0.3452
0.0156 0.4779 -39.1738
79.3600 -38.3100 0.5987 0.1059 -
0.3179 -0.1225 -0.0537 0.3910 -0.3396
0.0228 0.4810 -39.1727
79.3800 -39.3200 -0.1794 -0.0133 -
0.2447 -0.1358 -0.0856 0.3702 -0.3339
0.0299 0.4841 -39.1716
79.4000 -39.4300 -0.2384 -0.1100 -
0.1492 -0.1394 -0.1159 0.3494 -0.3281
0.0371 0.4872 -39.1705
79.4200 -39.1200 -0.0584 -0.0948 -
0.0372 -0.1356 -0.1443 0.3288 -0.3222
0.0443 0.4903 -39.1695
79.4400 -38.6700 0.2366 -0.0730
0.0793 -0.1266 -0.1705 0.3083 -0.3163
0.0515 0.4934 -39.1684
79.4600 -38.8100 0.0147 -0.0947
0.1819 -0.1141 -0.1941 0.2880 -0.3102
0.0587 0.4965 -39.1673
79.4800 -38.9700 -0.2519 -0.0318
0.2491 -0.0997 -0.2148 0.2679 -0.3041
0.0659 0.4995 -39.1662
79.5000 -38.4000 0.0767 0.1713
0.2658 -0.0848 -0.2325 0.2482 -0.2979
0.0731 0.5026 -39.1652
79.5200 -38.4100 0.0639 0.3042
0.2280 -0.0705 -0.2470 0.2287 -0.2916
0.0803 0.5056 -39.1641
79.5400 -38.4800 0.2049 0.1553
0.1452 -0.0577 -0.2582 0.2097 -0.2853
0.0875 0.5086 -39.1630
79.5600 -39.2000 -0.1057 -0.1699
0.0442 -0.0469 -0.2660 0.1911 -0.2788
0.0947 0.5116 -39.1620
79.5800 -39.4900 -0.2670 -0.3265 -
0.0423 -0.0388 -0.2705 0.1729 -0.2724
0.1020 0.5146 -39.1609

79.6000 -39.5700 -0.3916 -0.1806 -
0.0863 -0.0338 -0.2716 0.1552 -0.2658
0.1092 0.5176 -39.1598
79.6200 -38.3700 0.6535 0.0116 -
0.0806 -0.0328 -0.2694 0.1380 -0.2593
0.1164 0.5206 -39.1587
79.6400 -39.0500 -0.0177 -0.0033 -
0.0328 -0.0354 -0.2642 0.1213 -0.2526
0.1237 0.5235 -39.1577
79.6600 -39.4100 -0.5419 -0.0685
0.0308 -0.0409 -0.2560 0.1051 -0.2460
0.1309 0.5265 -39.1566
79.6800 -38.5700 0.2082 0.0673
0.0770 -0.0482 -0.2452 0.0895 -0.2392
0.1382 0.5294 -39.1555
79.7000 -38.3800 0.0761 0.3188
0.0822 -0.0563 -0.2321 0.0743 -0.2325
0.1454 0.5323 -39.1545
79.7200 -38.3500 0.1973 0.3836
0.0453 -0.0636 -0.2168 0.0597 -0.2257
0.1527 0.5352 -39.1534
79.7400 -38.8700 -0.2161 0.1982 -
0.0208 -0.0689 -0.1998 0.0456 -0.2189
0.1599 0.5381 -39.1523
79.7600 -38.9900 0.0136 -0.0924 -
0.0918 -0.0709 -0.1813 0.0321 -0.2121
0.1672 0.5410 -39.1513
79.7800 -39.1800 0.2865 -0.3931 -
0.1393 -0.0683 -0.1618 0.0191 -0.2052
0.1744 0.5439 -39.1502
79.8000 -39.8400 -0.4463 -0.4891 -
0.1401 -0.0606 -0.1415 0.0066 -0.1984
0.1817 0.5467 -39.1491
79.8200 -39.0500 -0.0135 -0.2065 -
0.0937 -0.0478 -0.1208 -0.0054 -0.1915
0.1889 0.5496 -39.1481
79.8400 -38.4600 0.1130 0.2441 -
0.0220 -0.0308 -0.0999 -0.0169 -0.1846
0.1961 0.5524 -39.1470
79.8600 -37.7700 0.4431 0.4477
0.0476 -0.0108 -0.0792 -0.0279 -0.1777
0.2034 0.5552 -39.1459
79.8800 -38.3500 -0.1266 0.2608
0.0915 0.0110 -0.0588 -0.0384 -0.1708
0.2106 0.5580 -39.1449
79.9000 -38.7300 -0.1990 -0.0736
0.1033 0.0331 -0.0390 -0.0484 -0.1639
0.2178 0.5608 -39.1438
79.9200 -38.9700 -0.2733 -0.2325
0.0896 0.0538 -0.0201 -0.0580 -0.1570
0.2251 0.5636 -39.1427

79.9400 -38.4500 0.1732 -0.1637
0.0611 0.0720 -0.0023 -0.0671 -0.1500
0.2323 0.5664 -39.1417
79.9600 -38.3200 0.0373 -0.0445
0.0266 0.0865 0.0144 -0.0757 -0.1431
0.2395 0.5691 -39.1406
79.9800 -38.3800 0.0663 0.0154 -
0.0061 0.0969 0.0297 -0.0838 -0.1362
0.2467 0.5718 -39.1396
80.0000 -38.5900 -0.2709 0.0524 -
0.0338 0.1028 0.0434 -0.0916 -0.1293
0.2539 0.5746 -39.1385
80.0200 -38.0100 0.3040 0.0930 -
0.0547 0.1047 0.0555 -0.0989 -0.1224
0.2611 0.5773 -39.1374
80.0400 -38.4400 -0.1449 0.0863 -
0.0660 0.1026 0.0660 -0.1058 -0.1155
0.2683 0.5800 -39.1364
80.0600 -38.2700 0.0968 -0.0097 -
0.0601 0.0968 0.0747 -0.1123 -0.1086
0.2754 0.5827 -39.1353
80.0800 -38.3600 0.1155 -0.1619 -
0.0319 0.0873 0.0818 -0.1184 -0.1018
0.2826 0.5853 -39.1343
80.1000 -38.8200 -0.2907 -0.1880
0.0140 0.0746 0.0873 -0.1241 -0.0949
0.2897 0.5880 -39.1332
80.1200 -38.0400 0.2073 -0.0027
0.0640 0.0592 0.0912 -0.1294 -0.0880
0.2969 0.5906 -39.1321
80.1400 -37.8400 -0.0118 0.2556
0.0981 0.0415 0.0937 -0.1344 -0.0812
0.3040 0.5933 -39.1311
80.1600 -37.8500 -0.0124 0.3711
0.0995 0.0223 0.0947 -0.1391 -0.0743
0.3111 0.5959 -39.1300
80.1800 -38.0100 0.1640 0.2338
0.0623 0.0022 0.0945 -0.1434 -0.0675
0.3182 0.5985 -39.1290
80.2000 -38.4700 -0.0623 -0.0688
0.0002 -0.0176 0.0932 -0.1474 -0.0606
0.3253 0.6011 -39.1279
80.2200 -38.8000 -0.1495 -0.3198 -
0.0608 -0.0360 0.0910 -0.1511 -0.0538
0.3323 0.6036 -39.1268
80.2400 -38.8300 0.0183 -0.4018 -
0.0940 -0.0520 0.0879 -0.1545 -0.0470
0.3394 0.6062 -39.1258
80.2600 -38.7500 -0.0277 -0.3182 -
0.0847 -0.0648 0.0842 -0.1577 -0.0402
0.3464 0.6087 -39.1247

80.2800 -38.6200 -0.1190 -0.1020 -
0.0401 -0.0737 0.0799 -0.1605 -0.0333
0.3535 0.6113 -39.1237
80.3000 -37.9700 0.1521 0.1863
0.0168 -0.0783 0.0753 -0.1632 -0.0265
0.3605 0.6138 -39.1226
80.3200 -37.8400 -0.0175 0.4112
0.0583 -0.0786 0.0703 -0.1655 -0.0197
0.3674 0.6163 -39.1216
80.3400 -37.8700 -0.0244 0.4159
0.0638 -0.0748 0.0652 -0.1677 -0.0129
0.3744 0.6188 -39.1205
80.3600 -37.9000 0.1977 0.1477
0.0298 -0.0674 0.0598 -0.1696 -0.0060
0.3814 0.6213 -39.1195
80.3800 -38.6100 -0.2324 -0.1927 -
0.0273 -0.0566 0.0544 -0.1712 0.0008
0.3883 0.6237 -39.1184
80.4000 -38.6500 0.0336 -0.3243 -
0.0823 -0.0433 0.0490 -0.1727 0.0076
0.3952 0.6262 -39.1174
80.4200 -38.4600 0.1295 -0.2372 -
0.1107 -0.0283 0.0435 -0.1740 0.0144
0.4021 0.6286 -39.1163
80.4400 -38.3500 0.0498 -0.1268 -
0.0981 -0.0126 0.0380 -0.1751 0.0213
0.4090 0.6310 -39.1153
80.4600 -38.4300 -0.2198 -0.0334 -
0.0513 0.0024 0.0325 -0.1759 0.0281
0.4158 0.6334 -39.1142
80.4800 -38.0600 -0.0085 0.0923
0.0120 0.0157 0.0271 -0.1766 0.0349
0.4226 0.6358 -39.1132
80.5000 -37.7500 0.1595 0.1791
0.0707 0.0262 0.0218 -0.1771 0.0418
0.4294 0.6382 -39.1121
80.5200 -37.6600 0.1312 0.1239
0.1069 0.0330 0.0166 -0.1773 0.0486
0.4362 0.6405 -39.1111
80.5400 -38.2100 -0.2291 0.0071
0.1131 0.0355 0.0116 -0.1774 0.0555
0.4430 0.6429 -39.1100
80.5600 -38.1400 -0.1074 0.0201
0.0913 0.0335 0.0068 -0.1773 0.0623
0.4497 0.6452 -39.1090
80.5800 -37.6900 0.1769 0.1329
0.0493 0.0276 0.0022 -0.1769 0.0691
0.4564 0.6475 -39.1079
80.6000 -37.7400 0.1966 0.0973
0.0025 0.0189 -0.0022 -0.1764 0.0760
0.4631 0.6498 -39.1069

80.6200 -38.5100 -0.2371 -0.1213 -
0.0322 0.0083 -0.0063 -0.1756 0.0828
0.4697 0.6521 -39.1058
80.6400 -38.3100 0.0530 -0.2625 -
0.0443 -0.0032 -0.0103 -0.1746 0.0897
0.4763 0.6544 -39.1048
80.6600 -38.3500 -0.0312 -0.1766 -
0.0323 -0.0146 -0.0140 -0.1733 0.0966
0.4829 0.6566 -39.1037
80.6800 -38.0500 -0.0970 0.0348 -
0.0075 -0.0247 -0.0174 -0.1719 0.1034
0.4895 0.6588 -39.1027
80.7000 -37.8700 0.0679 0.1996
0.0135 -0.0324 -0.0207 -0.1702 0.1103
0.4960 0.6611 -39.1016
80.7200 -37.7900 0.1307 0.1977
0.0174 -0.0367 -0.0237 -0.1683 0.1171
0.5026 0.6633 -39.1006
80.7400 -37.8700 0.2041 0.0468
0.0020 -0.0370 -0.0265 -0.1662 0.1240
0.5090 0.6655 -39.0995
80.7600 -38.5000 -0.4211 -0.0911 -
0.0236 -0.0329 -0.0290 -0.1638 0.1308
0.5155 0.6676 -39.0985
80.7800 -37.6800 0.4726 -0.1069 -
0.0467 -0.0248 -0.0313 -0.1612 0.1377
0.5219 0.6698 -39.0975
80.8000 -38.2900 -0.2387 -0.0734 -
0.0597 -0.0130 -0.0333 -0.1583 0.1445
0.5283 0.6719 -39.0964
80.8200 -38.2500 -0.2071 -0.0005 -
0.0583 0.0015 -0.0350 -0.1552 0.1513
0.5346 0.6741 -39.0954
80.8400 -37.7600 0.1877 0.0666 -
0.0410 0.0175 -0.0364 -0.1518 0.1582
0.5409 0.6762 -39.0943
80.8600 -37.6500 0.2768 -0.0044 -
0.0086 0.0329 -0.0376 -0.1482 0.1650
0.5472 0.6783 -39.0933
80.8800 -38.3300 -0.4320 -0.1030
0.0306 0.0458 -0.0386 -0.1443 0.1718
0.5535 0.6803 -39.0923
80.9000 -37.8100 -0.0544 -0.0156
0.0647 0.0547 -0.0394 -0.1402 0.1786
0.5597 0.6824 -39.0912
80.9200 -37.1700 0.3473 0.1803
0.0855 0.0581 -0.0401 -0.1359 0.1854
0.5659 0.6845 -39.0902
80.9400 -37.4600 -0.0608 0.2372
0.0920 0.0556 -0.0405 -0.1313 0.1921
0.5720 0.6865 -39.0891

80.9600 -37.7000 -0.0046 0.0508
0.0880 0.0471 -0.0408 -0.1265 0.1989
0.5781 0.6885 -39.0881
80.9800 -37.7800 0.1206 -0.2094
0.0803 0.0331 -0.0410 -0.1214 0.2056
0.5842 0.6905 -39.0871
81.0000 -38.1100 -0.1447 -0.3001
0.0742 0.0149 -0.0411 -0.1161 0.2123
0.5902 0.6925 -39.0860
81.0200 -38.1400 -0.4020 -0.0978
0.0692 -0.0059 -0.0411 -0.1106 0.2191
0.5962 0.6945 -39.0850
81.0400 -37.0500 0.3993 0.2456
0.0530 -0.0268 -0.0410 -0.1048 0.2257
0.6022 0.6964 -39.0839
81.0600 -37.2500 0.1605 0.3647
0.0118 -0.0448 -0.0409 -0.0989 0.2324
0.6081 0.6984 -39.0829
81.0800 -37.5600 0.0639 0.1527 -
0.0550 -0.0578 -0.0408 -0.0927 0.2391
0.6139 0.7003 -39.0819
81.1000 -38.0000 0.0000 -0.1576 -
0.1297 -0.0644 -0.0406 -0.0864 0.2457
0.6198 0.7022 -39.0808
81.1200 -38.5100 -0.4077 -0.2764 -
0.1878 -0.0638 -0.0405 -0.0800 0.2523
0.6256 0.7041 -39.0798
81.1400 -37.7900 0.2530 -0.1560 -
0.2066 -0.0556 -0.0403 -0.0734 0.2588
0.6313 0.7059 -39.0788
81.1600 -37.6200 0.1809 -0.0127 -
0.1759 -0.0399 -0.0402 -0.0666 0.2654
0.6370 0.7078 -39.0777
81.1800 -37.7800 -0.0963 -0.0230 -
0.0961 -0.0181 -0.0401 -0.0598 0.2719
0.6427 0.7096 -39.0767
81.2000 -37.6800 -0.0772 -0.0979
0.0140 0.0075 -0.0400 -0.0529 0.2784
0.6483 0.7114 -39.0757
81.2200 -37.3900 0.0058 -0.0626
0.1218 0.0341 -0.0399 -0.0460 0.2848
0.6538 0.7132 -39.0746
81.2400 -37.2400 -0.1002 0.0980
0.1947 0.0589 -0.0397 -0.0390 0.2913
0.6593 0.7150 -39.0736
81.2600 -36.6400 0.2785 0.2276
0.2149 0.0793 -0.0394 -0.0321 0.2976
0.6648 0.7168 -39.0726
81.2800 -37.1400 -0.1027 0.1853
0.1810 0.0937 -0.0389 -0.0252 0.3040
0.6702 0.7186 -39.0715

81.3000 -37.1800 0.0701 -0.0054
0.1091 0.1009 -0.0383 -0.0183 0.3103
0.6756 0.7203 -39.0705
81.3200 -37.4800 0.0568 -0.1835
0.0275 0.1004 -0.0373 -0.0116 0.3165
0.6810 0.7220 -39.0695
81.3400 -37.7300 -0.2127 -0.1831 -
0.0392 0.0924 -0.0360 -0.0049 0.3228
0.6862 0.7237 -39.0684
81.3600 -37.4300 -0.0005 -0.0081 -
0.0773 0.0774 -0.0343 0.0015 0.3289
0.6915 0.7254 -39.0674
81.3800 -36.8700 0.3469 0.1364 -
0.0854 0.0565 -0.0322 0.0078 0.3351
0.6966 0.7271 -39.0664
81.4000 -37.2700 -0.0731 0.0952 -
0.0702 0.0311 -0.0295 0.0139 0.3412
0.7018 0.7287 -39.0654
81.4200 -37.6700 -0.3350 -0.0235 -
0.0426 0.0033 -0.0263 0.0198 0.3472
0.7068 0.7303 -39.0643
81.4400 -37.4100 -0.0697 -0.0401 -
0.0129 -0.0249 -0.0225 0.0254 0.3532
0.7119 0.7320 -39.0633
81.4600 -37.0300 0.2577 -0.0088
0.0094 -0.0515 -0.0182 0.0307 0.3591
0.7168 0.7336 -39.0623
81.4800 -37.0700 0.2268 -0.0336
0.0184 -0.0750 -0.0132 0.0357 0.3650
0.7217 0.7351 -39.0612
81.5000 -37.6000 -0.2703 -0.0577
0.0137 -0.0939 -0.0075 0.0403 0.3708
0.7266 0.7367 -39.0602
81.5200 -37.5200 -0.1488 0.0073
0.0001 -0.1072 -0.0013 0.0446 0.3765
0.7314 0.7382 -39.0592
81.5400 -36.8900 0.2447 0.0835 -
0.0165 -0.1143 0.0056 0.0485 0.3822
0.7361 0.7398 -39.0582
81.5600 -37.2000 -0.0381 0.0450 -
0.0277 -0.1151 0.0131 0.0520 0.3878
0.7408 0.7413 -39.0571
81.5800 -37.4000 -0.0070 -0.0743 -
0.0268 -0.1099 0.0210 0.0551 0.3934
0.7454 0.7428 -39.0561
81.6000 -37.3200 -0.0744 -0.1237 -
0.0134 -0.0996 0.0294 0.0577 0.3989
0.7500 0.7443 -39.0551
81.6200 -37.1800 0.0695 -0.0629
0.0071 -0.0852 0.0382 0.0599 0.4043
0.7545 0.7457 -39.0541

81.6400 -37.0600 0.0403 0.0042
0.0262 -0.0675 0.0473 0.0617 0.4097
0.7590 0.7472 -39.0530
81.6600 -37.0000 -0.0103 0.0555
0.0363 -0.0474 0.0565 0.0629 0.4150
0.7633 0.7486 -39.0520
81.6800 -37.1100 -0.2184 0.1181
0.0326 -0.0260 0.0658 0.0637 0.4202
0.7677 0.7500 -39.0510
81.7000 -36.5500 0.2650 0.1325
0.0151 -0.0045 0.0749 0.0640 0.4253
0.7719 0.7514 -39.0500
81.7200 -36.8500 0.0830 0.0306 -
0.0083 0.0160 0.0837 0.0638 0.4304
0.7761 0.7527 -39.0490
81.7400 -37.3100 -0.3197 -0.1170 -
0.0262 0.0347 0.0922 0.0631 0.4354
0.7803 0.7541 -39.0479
81.7600 -37.1400 -0.1130 -0.1429 -
0.0269 0.0508 0.1002 0.0618 0.4403
0.7843 0.7554 -39.0469
81.7800 -36.7500 0.1015 -0.0207 -
0.0060 0.0638 0.1075 0.0600 0.4451
0.7883 0.7567 -39.0459
81.8000 -36.5600 0.1175 0.0915
0.0293 0.0733 0.1141 0.0578 0.4499
0.7923 0.7580 -39.0449
81.8200 -36.4400 0.1064 0.0870
0.0651 0.0791 0.1198 0.0549 0.4546
0.7961 0.7593 -39.0439
81.8400 -36.5800 -0.0173 0.0083
0.0878 0.0813 0.1245 0.0516 0.4592
0.7999 0.7606 -39.0428
81.8600 -37.0200 -0.2190 -0.0577
0.0891 0.0799 0.1281 0.0477 0.4638
0.8036 0.7618 -39.0418
81.8800 -36.6300 0.1969 -0.0487
0.0643 0.0754 0.1305 0.0433 0.4682
0.8073 0.7630 -39.0408
81.9000 -36.8900 -0.1707 0.0535
0.0166 0.0682 0.1316 0.0384 0.4726
0.8109 0.7642 -39.0398
81.9200 -36.5200 0.0898 0.1563 -
0.0437 0.0589 0.1314 0.0330 0.4770
0.8144 0.7654 -39.0388
81.9400 -36.4800 0.2753 0.1093 -
0.0971 0.0487 0.1297 0.0272 0.4812
0.8178 0.7666 -39.0378
81.9600 -37.0700 -0.0844 -0.0841 -
0.1236 0.0389 0.1265 0.0209 0.4854
0.8212 0.7677 -39.0367

81.9800 -37.5100 -0.4012 -0.2143 -
0.1128 0.0309 0.1218 0.0142 0.4895
0.8245 0.7689 -39.0357
82.0000 -36.6000 0.4493 -0.1807 -
0.0648 0.0254 0.1157 0.0071 0.4936
0.8277 0.7700 -39.0347
82.0200 -37.0800 -0.1928 -0.0703
0.0049 0.0228 0.1080 -0.0004 0.4975
0.8308 0.7711 -39.0337
82.0400 -36.8400 -0.1696 0.0893
0.0689 0.0229 0.0990 -0.0082 0.5015
0.8339 0.7721 -39.0327
82.0600 -36.4200 0.1029 0.2502
0.1021 0.0251 0.0888 -0.0163 0.5053
0.8369 0.7732 -39.0317
82.0800 -36.4400 0.1154 0.2648
0.0895 0.0287 0.0774 -0.0246 0.5091
0.8398 0.7742 -39.0307
82.1000 -36.7200 0.0284 0.0629
0.0379 0.0330 0.0650 -0.0332 0.5129
0.8426 0.7752 -39.0296
82.1200 -37.0000 -0.0169 -0.1783 -
0.0273 0.0365 0.0518 -0.0419 0.5166
0.8453 0.7762 -39.0286
82.1400 -37.3800 -0.2206 -0.2334 -
0.0785 0.0383 0.0379 -0.0508 0.5202
0.8480 0.7772 -39.0276
82.1600 -36.8900 0.2164 -0.1255 -
0.0948 0.0373 0.0236 -0.0597 0.5238
0.8506 0.7782 -39.0266
82.1800 -37.1100 -0.1059 -0.0325 -
0.0668 0.0329 0.0090 -0.0687 0.5273
0.8530 0.7791 -39.0256
82.2000 -36.8500 0.1238 0.0080 -
0.0049 0.0246 -0.0056 -0.0776 0.5308
0.8555 0.7800 -39.0246
82.2200 -36.9100 -0.1551 0.0491
0.0667 0.0121 -0.0201 -0.0866 0.5343
0.8578 0.7809 -39.0236
82.2400 -36.6600 0.1099 0.1089
0.1207 -0.0044 -0.0342 -0.0954 0.5377
0.8600 0.7818 -39.0226
82.2600 -36.6000 0.1149 0.1415
0.1409 -0.0243 -0.0478 -0.1041 0.5411
0.8622 0.7827 -39.0216
82.2800 -36.9700 -0.2492 0.1455
0.1220 -0.0467 -0.0606 -0.1127 0.5444
0.8642 0.7835 -39.0206
82.3000 -36.8700 -0.0514 0.1204
0.0687 -0.0703 -0.0726 -0.1210 0.5477
0.8662 0.7843 -39.0195

82.3200 -36.7400 0.3616 0.0270 -
0.0034 -0.0939 -0.0834 -0.1291 0.5510
0.8681 0.7851 -39.0185
82.3400 -37.6700 -0.3466 -0.1042 -
0.0707 -0.1159 -0.0931 -0.1369 0.5543
0.8699 0.7859 -39.0175
82.3600 -37.5700 0.0127 -0.1929 -
0.1132 -0.1341 -0.1014 -0.1444 0.5575
0.8716 0.7867 -39.0165
82.3800 -37.2300 0.2770 -0.1886 -
0.1196 -0.1467 -0.1083 -0.1516 0.5607
0.8732 0.7874 -39.0155
82.4000 -37.6000 -0.2226 -0.0811 -
0.0918 -0.1520 -0.1137 -0.1583 0.5639
0.8747 0.7882 -39.0145
82.4200 -37.2900 -0.0313 0.0950 -
0.0492 -0.1488 -0.1176 -0.1647 0.5671
0.8761 0.7889 -39.0135
82.4400 -36.9700 0.1121 0.2053 -
0.0139 -0.1365 -0.1198 -0.1706 0.5703
0.8774 0.7895 -39.0125
82.4600 -36.9600 0.1094 0.1721 -
0.0020 -0.1150 -0.1206 -0.1760 0.5734
0.8786 0.7902 -39.0115
82.4800 -37.2100 -0.1000 0.0625 -
0.0145 -0.0851 -0.1197 -0.1810 0.5766
0.8798 0.7909 -39.0105
82.5000 -37.2500 -0.1492 -0.0075 -
0.0407 -0.0487 -0.1175 -0.1854 0.5797
0.8808 0.7915 -39.0095
82.5200 -37.0600 0.1526 -0.0481 -
0.0611 -0.0085 -0.1138 -0.1894 0.5829
0.8817 0.7921 -39.0085
82.5400 -37.1000 0.1426 -0.1441 -
0.0585 0.0327 -0.1089 -0.1929 0.5860
0.8826 0.7927 -39.0075
82.5600 -37.4200 -0.2337 -0.2209 -
0.0281 0.0722 -0.1028 -0.1958 0.5892
0.8833 0.7932 -39.0065
82.5800 -37.0900 -0.1315 -0.1136
0.0229 0.1074 -0.0954 -0.1983 0.5923
0.8840 0.7938 -39.0055
82.6000 -36.6000 0.0249 0.1510
0.0777 0.1358 -0.0870 -0.2002 0.5955
0.8845 0.7943 -39.0045
82.6200 -36.2400 0.1581 0.3231
0.1185 0.1550 -0.0776 -0.2016 0.5987
0.8850 0.7948 -39.0035
82.6400 -36.2100 0.3090 0.1836
0.1339 0.1631 -0.0673 -0.2025 0.6019
0.8853 0.7953 -39.0025

82.6600 -37.2300 -0.3560 -0.1689
0.1243 0.1592 -0.0562 -0.2030 0.6051
0.8855 0.7958 -39.0015
82.6800 -37.0100 0.0428 -0.3643
0.0982 0.1436 -0.0444 -0.2029 0.6083
0.8857 0.7962 -39.0005
82.7000 -37.0600 -0.0244 -0.2111
0.0628 0.1175 -0.0321 -0.2024 0.6115
0.8857 0.7966 -38.9995
82.7200 -36.9100 -0.1350 0.1481
0.0226 0.0825 -0.0194 -0.2015 0.6147
0.8856 0.7970 -38.9985
82.7400 -36.5000 0.0504 0.3764 −
0.0180 0.0407 -0.0064 -0.2001 0.6180
0.8854 0.7974 -38.9975
82.7600 -36.0700 0.6941 0.1684 −
0.0534 -0.0053 0.0066 -0.1983 0.6213
0.8852 0.7978 -38.9965
82.7800 -38.0300 -0.7925 -0.2810 −
0.0733 -0.0521 0.0195 -0.1961 0.6246
0.8848 0.7981 -38.9955
82.8000 -36.9200 0.3630 -0.3385 −
0.0700 -0.0962 0.0321 -0.1935 0.6279
0.8843 0.7985 -38.9945
82.8200 -36.8900 0.1715 -0.0371 −
0.0471 -0.1339 0.0443 -0.1906 0.6312
0.8837 0.7988 -38.9935
82.8400 -36.8200 -0.0916 0.2270 −
0.0206 -0.1618 0.0558 -0.1873 0.6346
0.8830 0.7991 -38.9925
82.8600 -36.6200 0.0963 0.2326 −
0.0094 -0.1767 0.0666 -0.1837 0.6380
0.8822 0.7993 -38.9915
82.8800 -36.9900 -0.1005 0.1054 −
0.0315 -0.1765 0.0764 -0.1798 0.6414
0.8813 0.7996 -38.9905
82.9000 -37.0800 -0.0200 0.0061 −
0.0893 -0.1603 0.0853 -0.1756 0.6448
0.8803 0.7998 -38.9895
82.9200 -36.9900 0.1008 -0.0495 −
0.1626 -0.1292 0.0930 -0.1712 0.6483
0.8791 0.8000 -38.9885
82.9400 -37.1100 -0.0467 -0.0831 −
0.2223 -0.0851 0.0996 -0.1665 0.6518
0.8779 0.8002 -38.9875
82.9600 -37.2000 -0.1287 -0.0841 −
0.2394 -0.0313 0.1050 -0.1616 0.6553
0.8766 0.8003 -38.9865
82.9800 -36.7800 0.3074 -0.0849 −
0.1982 0.0286 0.1092 -0.1565 0.6588
0.8752 0.8005 -38.9856

83.0000 -37.0500 -0.2588 -0.1266 -
0.0977 0.0903 0.1122 -0.1512 0.6623
0.8736 0.8006 -38.9846
83.0200 -36.6400 -0.0157 -0.0968
0.0459 0.1492 0.1139 -0.1458 0.6659
0.8720 0.8007 -38.9836
83.0400 -36.2300 0.0696 0.0454
0.1998 0.2005 0.1143 -0.1401 0.6695
0.8702 0.8008 -38.9826
83.0600 -35.8900 0.0259 0.1858
0.3261 0.2397 0.1134 -0.1344 0.6731
0.8684 0.8009 -38.9816
83.0800 -35.8900 -0.1576 0.2235
0.3912 0.2627 0.1111 -0.1285 0.6767
0.8664 0.8009 -38.9806
83.1000 -35.8100 0.1402 0.1268
0.3779 0.2668 0.1076 -0.1224 0.6803
0.8644 0.8009 -38.9796
83.1200 -36.1500 -0.0165 -0.0343
0.2916 0.2520 0.1028 -0.1163 0.6839
0.8622 0.8009 -38.9786
83.1400 -36.5100 -0.0928 -0.1227
0.1562 0.2206 0.0970 -0.1101 0.6875
0.8599 0.8009 -38.9776
83.1600 -36.6200 -0.0814 -0.0650
0.0049 0.1760 0.0903 -0.1038 0.6911
0.8576 0.8009 -38.9766
83.1800 -36.4000 0.2405 0.0168 -
0.1292 0.1219 0.0828 -0.0974 0.6947
0.8551 0.8008 -38.9757
83.2000 -36.8400 -0.1087 -0.0238 -
0.2222 0.0618 0.0750 -0.0909 0.6983
0.8525 0.8007 -38.9747
83.2200 -37.1600 -0.1701 -0.0919 -
0.2625 -0.0008 0.0669 -0.0844 0.7019
0.8499 0.8007 -38.9737
83.2400 -37.0500 0.0372 -0.0576 -
0.2470 -0.0628 0.0588 -0.0779 0.7055
0.8471 0.8005 -38.9727
83.2600 -36.7300 0.1875 0.0156 -
0.1861 -0.1213 0.0510 -0.0712 0.7090
0.8442 0.8004 -38.9717
83.2800 -37.2200 -0.3467 0.0378 -
0.1007 -0.1733 0.0436 -0.0646 0.7125
0.8412 0.8003 -38.9707
83.3000 -36.6200 0.3389 0.0099 -
0.0173 -0.2160 0.0370 -0.0578 0.7160
0.8382 0.8001 -38.9697
83.3200 -36.9700 -0.1603 -0.0225
0.0417 -0.2470 0.0312 -0.0511 0.7193
0.8350 0.7999 -38.9688

83.3400	-36.9600	-0.0612	0.0320	
0.0609	-0.2645	0.0264	-0.0442	0.7227
	0.8318	0.7997	-38.9678	
83.3600	-36.5300	0.0632	0.1480	
0.0364	-0.2676	0.0229	-0.0373	0.7260
	0.8284	0.7995	-38.9668	
83.3800	-36.7500	0.0353	0.1766	-
0.0249	-0.2569	0.0206	-0.0303	0.7292
	0.8249	0.7992	-38.9658	
83.4000	-36.8900	0.0562	0.0406	-
0.1060	-0.2334	0.0195	-0.0231	0.7323
	0.8214	0.7989	-38.9648	
83.4200	-37.2600	-0.1484	-0.1375	-
0.1816	-0.1984	0.0194	-0.0159	0.7353
	0.8177	0.7987	-38.9638	
83.4400	-37.2700	-0.0340	-0.1889	-
0.2274	-0.1535	0.0202	-0.0085	0.7382
	0.8140	0.7984	-38.9629	
83.4600	-36.7900	0.2424	-0.1669	-
0.2257	-0.1011	0.0216	-0.0009	0.7410
	0.8101	0.7980	-38.9619	
83.4800	-37.0100	-0.1111	-0.1550	-
0.1726	-0.0440	0.0234	0.0068	0.7437
	0.8062	0.7977	-38.9609	
83.5000	-36.6100	0.0444	-0.1001	-
0.0814	0.0147	0.0252	0.0148	0.7463
	0.8022	0.7973	-38.9599	
83.5200	-36.5300	-0.2579	0.0962	
0.0232	0.0718	0.0269	0.0231	0.7487
	0.7980	0.7970	-38.9589	
83.5400	-35.9800	0.0669	0.3077	
0.1155	0.1243	0.0282	0.0317	0.7509
	0.7938	0.7966	-38.9580	
83.5600	-35.5200	0.3861	0.2912	
0.1759	0.1689	0.0287	0.0407	0.7530
	0.7895	0.7962	-38.9570	
83.5800	-36.4600	-0.3221	0.0295	
0.2010	0.2027	0.0284	0.0500	0.7550
	0.7851	0.7957	-38.9560	
83.6000	-36.4200	-0.0084	-0.1865	
0.2043	0.2238	0.0269	0.0598	0.7568
	0.7806	0.7953	-38.9550	
83.6200	-36.2200	0.0785	-0.2018	
0.1997	0.2313	0.0241	0.0700	0.7584
	0.7760	0.7948	-38.9540	
83.6400	-36.2200	0.0879	-0.1156	
0.1960	0.2255	0.0197	0.0806	0.7598
	0.7714	0.7943	-38.9531	
83.6600	-36.2100	-0.1040	-0.0456	
0.1964	0.2070	0.0137	0.0918	0.7610
	0.7666	0.7938	-38.9521	

83.6800 -36.1600 -0.0389 0.0233
0.1899 0.1777 0.0060 0.1035 0.7620
0.7618 0.7933 -38.9511
83.7000 -36.1300 -0.0374 0.1180
0.1611 0.1406 -0.0032 0.1157 0.7628
0.7568 0.7928 -38.9501
83.7200 -36.0200 0.1349 0.1653
0.1055 0.0991 -0.0141 0.1284 0.7633
0.7518 0.7922 -38.9492
83.7400 -36.3700 -0.0436 0.1098
0.0267 0.0565 -0.0266 0.1416 0.7637
0.7467 0.7916 -38.9482
83.7600 -36.6400 -0.1749 0.0041 -
0.0640 0.0160 -0.0405 0.1552 0.7638
0.7415 0.7911 -38.9472
83.7800 -36.5600 0.2493 -0.0862 -
0.1517 -0.0189 -0.0559 0.1693 0.7636
0.7362 0.7904 -38.9462
83.8000 -37.0200 -0.0493 -0.1418 -
0.2212 -0.0454 -0.0726 0.1838 0.7632
0.7308 0.7898 -38.9453
83.8200 -37.0600 -0.0897 -0.1044 -
0.2590 -0.0614 -0.0903 0.1986 0.7626
0.7254 0.7892 -38.9443
83.8400 -37.0100 -0.1351 0.0088 -
0.2590 -0.0656 -0.1088 0.2137 0.7617
0.7198 0.7885 -38.9433
83.8600 -36.6600 0.1777 0.0704 -
0.2236 -0.0579 -0.1278 0.2291 0.7605
0.7142 0.7878 -38.9424
83.8800 -36.6600 0.1696 0.0022 -
0.1600 -0.0401 -0.1467 0.2446 0.7591
0.7085 0.7871 -38.9414
83.9000 -37.1900 -0.3451 -0.0676 -
0.0787 -0.0149 -0.1650 0.2604 0.7573
0.7027 0.7864 -38.9404
83.9200 -36.4700 0.1012 -0.0167
0.0078 0.0141 -0.1822 0.2762 0.7553
0.6968 0.7857 -38.9394
83.9400 -36.1700 0.2199 0.0648
0.0851 0.0427 -0.1975 0.2920 0.7530
0.6909 0.7850 -38.9385
83.9600 -36.4500 -0.1749 0.0781
0.1421 0.0663 -0.2106 0.3079 0.7504
0.6849 0.7842 -38.9375
83.9800 -36.3600 -0.0318 0.0624
0.1723 0.0813 -0.2206 0.3237 0.7474
0.6788 0.7834 -38.9365
84.0000 -36.1900 0.2123 0.0309
0.1800 0.0850 -0.2273 0.3393 0.7442
0.6726 0.7826 -38.9356

84.0200 -36.5300 -0.1312 -0.0210
0.1697 0.0761 -0.2302 0.3548 0.7406
0.6663 0.7818 -38.9346
84.0400 -36.6300 -0.1717 -0.0370
0.1460 0.0546 -0.2292 0.3701 0.7368
0.6600 0.7810 -38.9336
84.0600 -36.1800 0.3456 -0.0056
0.1136 0.0213 -0.2241 0.3850 0.7325
0.6536 0.7802 -38.9327
84.0800 -36.5800 -0.1762 0.0555
0.0725 -0.0217 -0.2150 0.3997 0.7280
0.6471 0.7793 -38.9317
84.1000 -36.6900 -0.1244 0.1317
0.0211 -0.0714 -0.2021 0.4139 0.7231
0.6406 0.7784 -38.9307
84.1200 -36.4300 0.1285 0.1410 -
0.0371 -0.1243 -0.1854 0.4277 0.7178
0.6339 0.7775 -38.9298
84.1400 -36.5900 0.2491 -0.0201 -
0.0948 -0.1770 -0.1651 0.4410 0.7122
0.6273 0.7766 -38.9288
84.1600 -37.4000 -0.2932 -0.2277 -
0.1383 -0.2258 -0.1414 0.4537 0.7062
0.6205 0.7757 -38.9278
84.1800 -37.2500 -0.0829 -0.2197 -
0.1541 -0.2670 -0.1144 0.4658 0.6999
0.6137 0.7748 -38.9269
84.2000 -36.7100 0.1321 0.0092 -
0.1402 -0.2983 -0.0845 0.4772 0.6932
0.6068 0.7738 -38.9259
84.2200 -36.5100 0.0831 0.2114 -
0.1083 -0.3180 -0.0519 0.4879 0.6860
0.5998 0.7728 -38.9249
84.2400 -36.5100 0.1790 0.2116 -
0.0779 -0.3259 -0.0171 0.4977 0.6785
0.5928 0.7718 -38.9240
84.2600 -36.8500 -0.1350 0.0540 -
0.0602 -0.3221 0.0196 0.5067 0.6707
0.5857 0.7708 -38.9230
84.2800 -36.8500 -0.0810 -0.1063 -
0.0564 -0.3074 0.0578 0.5148 0.6624
0.5786 0.7698 -38.9220
84.3000 -36.8300 -0.1550 -0.1279 -
0.0585 -0.2826 0.0968 0.5219 0.6537
0.5714 0.7688 -38.9211
84.3200 -36.4200 0.1784 -0.0269 -
0.0550 -0.2486 0.1361 0.5279 0.6446
0.5641 0.7677 -38.9201
84.3400 -36.3400 0.1194 0.0320 -
0.0395 -0.2062 0.1751 0.5328 0.6351
0.5568 0.7667 -38.9192

84.3600 -36.4600 -0.0562 -0.0243 -
0.0123 -0.1561 0.2132 0.5366 0.6251
0.5494 0.7656 -38.9182
84.3800 -36.5300 -0.2519 -0.0548
0.0205 -0.0991 0.2497 0.5391 0.6148
0.5419 0.7645 -38.9172
84.4000 -36.3000 -0.1434 0.0477
0.0472 -0.0358 0.2840 0.5404 0.6040
0.5344 0.7634 -38.9163
84.4200 -35.5800 0.4418 0.1511
0.0542 0.0329 0.3155 0.5403 0.5928
0.5269 0.7623 -38.9153
84.4400 -36.2400 -0.3400 0.0975
0.0321 0.1059 0.3437 0.5389 0.5812
0.5193 0.7611 -38.9144
84.4600 -36.1000 -0.0273 -0.0381 -
0.0181 0.1812 0.3679 0.5360 0.5692
0.5116 0.7600 -38.9134
84.4800 -36.0000 0.1686 -0.1193 -
0.0832 0.2557 0.3875 0.5316 0.5568
0.5039 0.7588 -38.9124
84.5000 -36.2500 -0.0398 -0.0884 -
0.1457 0.3253 0.4021 0.5257 0.5439
0.4962 0.7576 -38.9115
84.5200 -36.0500 -0.0992 0.0498 -
0.1840 0.3862 0.4112 0.5182 0.5307
0.4884 0.7564 -38.9105
84.5400 -35.7100 0.1021 0.1582 -
0.1736 0.4347 0.4142 0.5093 0.5171
0.4805 0.7552 -38.9096
84.5600 -35.6900 0.0572 0.0926 -
0.0970 0.4674 0.4109 0.4987 0.5031
0.4726 0.7540 -38.9086
84.5800 -35.9200 0.0530 -0.1045
0.0489 0.4817 0.4010 0.4867 0.4887
0.4647 0.7527 -38.9077
84.6000 -35.9300 -0.0942 -0.2978
0.2517 0.4764 0.3849 0.4733 0.4740
0.4567 0.7515 -38.9067
84.6200 -35.7000 0.0681 -0.3275
0.4850 0.4508 0.3626 0.4584 0.4589
0.4487 0.7502 -38.9058
84.6400 -35.6400 -0.0987 -0.1522
0.7072 0.4052 0.3349 0.4423 0.4434
0.4406 0.7489 -38.9048
84.6600 -35.2100 -0.0321 0.1593
0.8690 0.3403 0.3022 0.4250 0.4277
0.4325 0.7476 -38.9038
84.6800 -34.8600 0.2655 0.4415
0.9178 0.2577 0.2653 0.4066 0.4116
0.4243 0.7463 -38.9029

84.7000	-35.3500	-0.1071	0.5071	
0.8140	0.1609	0.2249	0.3871	0.3952
	0.4161	0.7450	-38.9019	
84.7200	-35.8800	0.1013	0.2811	
0.5641	0.0551	0.1819	0.3666	0.3785
	0.4079	0.7437	-38.9010	
84.7400	-36.5200	0.3359	-0.1290	
0.2132	-0.0537	0.1368	0.3454	0.3616
	0.3997	0.7423	-38.9000	
84.7600	-38.2700	-0.5637	-0.4348	-
0.1784	-0.1597	0.0905	0.3233	0.3443
	0.3914	0.7409	-38.8991	
84.7800	-38.6100	-0.2968	-0.3920	-
0.5512	-0.2571	0.0437	0.3005	0.3268
	0.3831	0.7396	-38.8981	
84.8000	-37.9400	0.5711	-0.1213	-
0.8541	-0.3400	-0.0029	0.2771	0.3091
	0.3747	0.7382	-38.8972	
84.8200	-38.5800	0.0765	0.0644	-
1.0494	-0.4024	-0.0485	0.2532	0.2911
	0.3663	0.7367	-38.8962	
84.8400	-39.0700	-0.2691	0.0916	-
1.1192	-0.4392	-0.0925	0.2289	0.2729
	0.3579	0.7353	-38.8953	
84.8600	-39.0600	-0.1447	0.0817	-
1.0618	-0.4480	-0.1341	0.2042	0.2545
	0.3495	0.7339	-38.8943	
84.8800	-38.6300	0.2096	0.0325	-
0.8806	-0.4305	-0.1728	0.1793	0.2358
	0.3410	0.7324	-38.8934	
84.9000	-38.8100	-0.0026	-0.1519	-
0.5875	-0.3911	-0.2082	0.1541	0.2170
	0.3325	0.7310	-38.8924	
84.9200	-38.5000	0.0686	-0.3979	-
0.2154	-0.3355	-0.2401	0.1289	0.1980
	0.3240	0.7295	-38.8915	
84.9400	-38.5400	-0.1141	-0.4942	
0.1899	-0.2689	-0.2683	0.1037	0.1788
	0.3154	0.7280	-38.8905	
84.9600	-38.0900	-0.3778	-0.2563	
0.5725	-0.1970	-0.2926	0.0786	0.1594
	0.3069	0.7265	-38.8896	
84.9800	-36.9700	-0.1251	0.2861	
0.8710	-0.1249	-0.3129	0.0536	0.1399
	0.2983	0.7250	-38.8886	
85.0000	-35.6300	0.5620	0.7598	
1.0238	-0.0581	-0.3290	0.0289	0.1203
	0.2897	0.7235	-38.8877	
85.0200	-36.2000	0.1830	0.7435	
0.9836	-0.0011	-0.3409	0.0045	0.1005
	0.2811	0.7219	-38.8867	

85.0400 -37.1200 -0.0102 0.2649
0.7658 0.0437 -0.3485 -0.0195 0.0807
0.2724 0.7204 -38.8858
85.0600 -38.1800 -0.1773 -0.3108
0.4381 0.0768 -0.3521 -0.0430 0.0607
0.2638 0.7188 -38.8849
85.0800 -38.8000 -0.1854 -0.6136
0.0798 0.0991 -0.3517 -0.0660 0.0406
0.2551 0.7172 -38.8839
85.1000 -38.9100 0.0613 -0.5454 -
0.2354 0.1117 -0.3476 -0.0884 0.0204
0.2464 0.7157 -38.8830
85.1200 -38.9900 0.0391 -0.2786 -
0.4569 0.1157 -0.3400 -0.1102 0.0002
0.2377 0.7141 -38.8820
85.1400 -38.8800 -0.0476 -0.0288 -
0.5621 0.1122 -0.3295 -0.1313 -0.0201
0.2290 0.7124 -38.8811
85.1600 -38.8400 -0.0795 0.1076 -
0.5489 0.1027 -0.3162 -0.1517 -0.0405
0.2203 0.7108 -38.8801
85.1800 -38.6800 0.1167 0.1157 -
0.4370 0.0887 -0.3008 -0.1713 -0.0609
0.2116 0.7092 -38.8792
85.2000 -38.7400 0.0593 0.0139 -
0.2660 0.0728 -0.2837 -0.1901 -0.0813
0.2028 0.7075 -38.8783
85.2200 -38.8700 -0.1855 -0.0884 -
0.0836 0.0580 -0.2655 -0.2080 -0.1017
0.1941 0.7059 -38.8773
85.2400 -38.4900 0.0294 -0.0676
0.0688 0.0472 -0.2465 -0.2250 -0.1221
0.1853 0.7042 -38.8764
85.2600 -38.2100 0.1045 0.0412
0.1653 0.0432 -0.2274 -0.2411 -0.1426
0.1766 0.7025 -38.8754
85.2800 -38.3900 -0.0142 0.1130
0.1945 0.0471 -0.2086 -0.2563 -0.1630
0.1678 0.7008 -38.8745
85.3000 -38.4200 -0.0954 0.0872
0.1673 0.0581 -0.1904 -0.2704 -0.1833
0.1591 0.6991 -38.8735
85.3200 -38.5200 -0.0324 -0.0044
0.1089 0.0738 -0.1731 -0.2836 -0.2037
0.1503 0.6974 -38.8726
85.3400 -38.6100 0.1390 -0.0802
0.0467 0.0909 -0.1570 -0.2958 -0.2239
0.1416 0.6956 -38.8717
85.3600 -38.7600 -0.1221 -0.0775 -
0.0005 0.1063 -0.1421 -0.3070 -0.2442
0.1328 0.6939 -38.8707

85.3800 -38.6000 -0.0654 0.0266 -
0.0263 0.1172 -0.1283 -0.3172 -0.2643
0.1241 0.6921 -38.8698
85.4000 -38.4600 0.0515 0.1564 -
0.0339 0.1216 -0.1154 -0.3265 -0.2843
0.1153 0.6904 -38.8689
85.4200 -38.5700 0.0476 0.1557 -
0.0277 0.1184 -0.1035 -0.3349 -0.3043
0.1066 0.6886 -38.8679
85.4400 -38.5900 0.1799 -0.0268 -
0.0087 0.1071 -0.0921 -0.3423 -0.3241
0.0979 0.6868 -38.8670
85.4600 -39.2100 -0.3138 -0.2178
0.0220 0.0881 -0.0812 -0.3489 -0.3438
0.0891 0.6850 -38.8660
85.4800 -39.0800 -0.0472 -0.1947
0.0594 0.0629 -0.0706 -0.3546 -0.3634
0.0804 0.6832 -38.8651
85.5000 -38.4700 0.2900 -0.0007
0.0918 0.0328 -0.0600 -0.3596 -0.3828
0.0717 0.6814 -38.8642
85.5200 -38.6100 -0.0243 0.1438
0.1042 -0.0004 -0.0494 -0.3639 -0.4021
0.0630 0.6795 -38.8632
85.5400 -38.9700 -0.2175 0.1831
0.0848 -0.0349 -0.0385 -0.3676 -0.4211
0.0543 0.6777 -38.8623
85.5600 -38.9700 0.0012 0.1573
0.0324 -0.0683 -0.0273 -0.3706 -0.4400
0.0457 0.6758 -38.8614
85.5800 -38.9700 0.2050 0.0343 -
0.0382 -0.0981 -0.0156 -0.3730 -0.4587
0.0370 0.6740 -38.8604
85.6000 -39.4000 0.0442 -0.1833 -
0.1054 -0.1219 -0.0033 -0.3750 -0.4772
0.0284 0.6721 -38.8595
85.6200 -40.0400 -0.3224 -0.3196 -
0.1477 -0.1376 0.0097 -0.3766 -0.4955
0.0197 0.6702 -38.8586
85.6400 -39.6300 -0.0798 -0.1979 -
0.1544 -0.1439 0.0236 -0.3777 -0.5135
0.0111 0.6683 -38.8576
85.6600 -39.0200 0.2986 0.0816 -
0.1328 -0.1405 0.0382 -0.3786 -0.5313
0.0026 0.6664 -38.8567
85.6800 -39.0100 -0.0615 0.2822 -
0.0982 -0.1279 0.0536 -0.3792 -0.5489
-0.0060 0.6645 -38.8558
85.7000 -38.9900 -0.0369 0.2921 -
0.0643 -0.1073 0.0694 -0.3796 -0.5661
-0.0145 0.6625 -38.8548

85.7200 -39.0600 -0.0097 0.1570 -
0.0373 -0.0805 0.0857 -0.3798 -0.5831
-0.0231 0.6606 -38.8539
85.7400 -39.1700 -0.0348 -0.0325 -
0.0134 -0.0495 0.1020 -0.3799 -0.5998
-0.0315 0.6587 -38.8530
85.7600 -39.2300 0.0410 -0.1924
0.0161 -0.0167 0.1181 -0.3799 -0.6162
-0.0400 0.6567 -38.8521
85.7800 -39.3100 0.0454 -0.2790
0.0616 0.0154 0.1339 -0.3799 -0.6323
-0.0484 0.6547 -38.8511
85.8000 -39.2700 0.0370 -0.2931
0.1251 0.0443 0.1490 -0.3799 -0.6481
-0.0569 0.6528 -38.8502
85.8200 -39.1700 -0.1335 -0.1886
0.1945 0.0681 0.1634 -0.3798 -0.6636
-0.0652 0.6508 -38.8493
85.8400 -38.8900 -0.0913 0.0864
0.2459 0.0855 0.1768 -0.3799 -0.6787
-0.0736 0.6488 -38.8483
85.8600 -38.2300 0.2403 0.4121
0.2549 0.0958 0.1890 -0.3801 -0.6935
-0.0819 0.6468 -38.8474
85.8800 -38.3600 0.0070 0.5303
0.2071 0.0990 0.2000 -0.3803 -0.7079
-0.0902 0.6447 -38.8465
85.9000 -38.6700 -0.0985 0.3113
0.1046 0.0960 0.2095 -0.3808 -0.7219
-0.0984 0.6427 -38.8456
85.9200 -39.1000 0.2242 -0.1271 -
0.0260 0.0884 0.2177 -0.3815 -0.7356
-0.1067 0.6407 -38.8446
85.9400 -40.1100 -0.2880 -0.4752 -
0.1480 0.0773 0.2244 -0.3824 -0.7489
-0.1148 0.6386 -38.8437
85.9600 -39.8800 -0.0248 -0.4621 -
0.2269 0.0640 0.2299 -0.3836 -0.7617
-0.1230 0.6366 -38.8428
85.9800 -39.4700 0.0260 -0.1404 -
0.2456 0.0499 0.2342 -0.3851 -0.7742
-0.1311 0.6345 -38.8419
86.0000 -39.2400 0.0377 0.2048 -
0.2100 0.0360 0.2374 -0.3869 -0.7863
-0.1392 0.6324 -38.8409
86.0200 -38.8900 0.1613 0.3304 -
0.1348 0.0230 0.2396 -0.3891 -0.7980
-0.1472 0.6304 -38.8400
86.0400 -39.2100 -0.0957 0.1964 -
0.0415 0.0116 0.2409 -0.3916 -0.8093
-0.1552 0.6283 -38.8391

86.0600 -39.4700 -0.0296 -0.0478
0.0492 0.0021 0.2414 -0.3945 -0.8201
-0.1631 0.6262 -38.8382
86.0800 -39.4300 -0.0200 -0.1815
0.1194 -0.0055 0.2413 -0.3977 -0.8305
-0.1710 0.6241 -38.8372
86.1000 -39.4100 -0.0797 -0.0886
0.1537 -0.0113 0.2407 -0.4014 -0.8405
-0.1788 0.6219 -38.8363
86.1200 -39.0600 0.1125 0.1210
0.1482 -0.0155 0.2398 -0.4055 -0.8500
-0.1866 0.6198 -38.8354
86.1400 -39.0600 0.1945 0.1952
0.1076 -0.0184 0.2385 -0.4099 -0.8591
-0.1944 0.6177 -38.8345
86.1600 -39.4500 -0.0239 0.0608
0.0427 -0.0203 0.2371 -0.4149 -0.8677
-0.2021 0.6155 -38.8335
86.1800 -39.6100 0.0046 -0.1289 -
0.0264 -0.0213 0.2356 -0.4202 -0.8759
-0.2097 0.6134 -38.8326
86.2000 -40.0500 -0.2257 -0.1683 -
0.0808 -0.0216 0.2340 -0.4259 -0.8836
-0.2173 0.6112 -38.8317
86.2200 -39.8000 0.0164 -0.0529 -
0.1096 -0.0212 0.2324 -0.4321 -0.8909
-0.2249 0.6091 -38.8308
86.2400 -39.5000 0.2002 0.0583 -
0.1131 -0.0203 0.2308 -0.4387 -0.8977
-0.2324 0.6069 -38.8299
86.2600 -39.6600 0.0203 0.0508 -
0.0968 -0.0188 0.2293 -0.4458 -0.9041
-0.2398 0.6047 -38.8289
86.2800 -39.8600 -0.1492 -0.0119 -
0.0647 -0.0170 0.2279 -0.4532 -0.9100
-0.2472 0.6025 -38.8280
86.3000 -39.6800 -0.0581 -0.0199 -
0.0199 -0.0152 0.2265 -0.4610 -0.9154
-0.2545 0.6003 -38.8271
86.3200 -39.4900 0.1746 0.0107
0.0317 -0.0141 0.2252 -0.4692 -0.9204
-0.2618 0.5981 -38.8262
86.3400 -39.5800 0.0186 0.0052
0.0797 -0.0141 0.2238 -0.4777 -0.9249
-0.2690 0.5959 -38.8253
86.3600 -39.6600 -0.0508 0.0016
0.1128 -0.0158 0.2223 -0.4865 -0.9290
-0.2761 0.5937 -38.8244
86.3800 -39.6600 -0.0398 0.0523
0.1225 -0.0194 0.2206 -0.4956 -0.9326
-0.2832 0.5914 -38.8234

86.4000	-39.6500	-0.0732	0.1387	
0.1051	-0.0247	0.2185	-0.5049	-0.9357
	-0.2902	0.5892	-38.8225	
86.4200	-39.5900	-0.0477	0.1845	
0.0657	-0.0311	0.2158	-0.5144	-0.9384
	-0.2972	0.5869	-38.8216	
86.4400	-39.5300	0.2155	0.0983	
0.0153	-0.0375	0.2125	-0.5241	-0.9406
	-0.3041	0.5847	-38.8207	
86.4600	-39.9900	0.0261	-0.1298	-
0.0345	-0.0424	0.2084	-0.5339	-0.9424
	-0.3109	0.5824	-38.8198	
86.4800	-40.4400	-0.2758	-0.3121	-
0.0720	-0.0441	0.2032	-0.5437	-0.9437
	-0.3177	0.5801	-38.8189	
86.5000	-40.1500	-0.0024	-0.2281	-
0.0883	-0.0413	0.1970	-0.5536	-0.9446
	-0.3244	0.5779	-38.8180	
86.5200	-39.8500	0.1406	0.0612	-
0.0855	-0.0330	0.1895	-0.5636	-0.9450
	-0.3310	0.5756	-38.8170	
86.5400	-39.6900	0.0199	0.2884	-
0.0740	-0.0187	0.1807	-0.5734	-0.9450
	-0.3376	0.5733	-38.8161	
86.5600	-39.5800	0.2663	0.2359	-
0.0619	0.0008	0.1705	-0.5832	-0.9445
	-0.3441	0.5710	-38.8152	
86.5800	-40.1300	-0.1698	-0.0327	-
0.0516	0.0245	0.1589	-0.5929	-0.9436
	-0.3505	0.5687	-38.8143	
86.6000	-40.5000	-0.1977	-0.1978	-
0.0376	0.0506	0.1458	-0.6025	-0.9422
	-0.3569	0.5664	-38.8134	
86.6200	-40.0200	0.0462	-0.1028	-
0.0141	0.0772	0.1313	-0.6118	-0.9404
	-0.3632	0.5641	-38.8125	
86.6400	-39.7000	0.1356	0.0824	
0.0200	0.1025	0.1153	-0.6210	-0.9382
	-0.3694	0.5617	-38.8116	
86.6600	-39.7100	-0.0163	0.1371	
0.0623	0.1244	0.0979	-0.6298	-0.9355
	-0.3756	0.5594	-38.8107	
86.6800	-39.7100	0.1277	0.0126	
0.1058	0.1412	0.0791	-0.6384	-0.9324
	-0.3817	0.5571	-38.8097	
86.7000	-39.9900	0.0796	-0.1567	
0.1375	0.1516	0.0590	-0.6466	-0.9288
	-0.3877	0.5547	-38.8088	
86.7200	-40.4000	-0.3889	-0.1702	
0.1439	0.1552	0.0377	-0.6544	-0.9248
	-0.3937	0.5524	-38.8079	

86.7400 -40.1600 -0.2834 0.0632
0.1172 0.1518 0.0152 -0.6619 -0.9204
-0.3995 0.5500 -38.8070
86.7600 -39.3300 0.4499 0.3183
0.0575 0.1418 -0.0082 -0.6688 -0.9156
-0.4054 0.5477 -38.8061
86.7800 -39.6300 0.3367 0.2767 -
0.0272 0.1255 -0.0324 -0.6753 -0.9103
-0.4111 0.5453 -38.8052
86.8000 -40.8400 -0.4048 -0.0533 -
0.1125 0.1033 -0.0572 -0.6812 -0.9047
-0.4168 0.5429 -38.8043
86.8200 -40.8300 -0.0613 -0.3352 -
0.1678 0.0756 -0.0823 -0.6866 -0.8986
-0.4224 0.5405 -38.8034
86.8400 -40.7700 0.0556 -0.3133 -
0.1688 0.0427 -0.1075 -0.6913 -0.8921
-0.4280 0.5381 -38.8025
86.8600 -40.7700 -0.2821 -0.0381 -
0.1127 0.0052 -0.1325 -0.6954 -0.8852
-0.4334 0.5357 -38.8016
86.8800 -39.9700 0.3575 0.2032 -
0.0196 -0.0357 -0.1571 -0.6989 -0.8779
-0.4389 0.5333 -38.8007
86.9000 -39.9700 0.3029 0.2327
0.0809 -0.0786 -0.1811 -0.7015 -0.8701
-0.4442 0.5309 -38.7998
86.9200 -40.8100 -0.4743 0.1256
0.1640 -0.1220 -0.2040 -0.7034 -0.8620
-0.4495 0.5285 -38.7989
86.9400 -40.8100 -0.3957 0.0665
0.2140 -0.1643 -0.2257 -0.7045 -0.8535
-0.4547 0.5261 -38.7980
86.9600 -40.1500 0.3366 0.0738
0.2250 -0.2037 -0.2459 -0.7048 -0.8447
-0.4599 0.5237 -38.7971
86.9800 -40.1500 0.3859 -0.0238
0.2002 -0.2378 -0.2643 -0.7042 -0.8354
-0.4649 0.5213 -38.7961
87.0000 -41.2200 -0.3557 -0.2335
0.1449 -0.2642 -0.2806 -0.7026 -0.8257
-0.4700 0.5188 -38.7952
87.0200 -41.2200 -0.1253 -0.2907
0.0605 -0.2805 -0.2946 -0.7001 -0.8157
-0.4749 0.5164 -38.7943
87.0400 -40.8800 0.0091 -0.0344 -
0.0489 -0.2845 -0.3062 -0.6967 -0.8054
-0.4798 0.5140 -38.7934
87.0600 -40.8400 -0.2471 0.3804 -
0.1679 -0.2744 -0.3151 -0.6922 -0.7946
-0.4846 0.5115 -38.7925

87.0800 -40.3700 0.2452 0.5543 -
0.2755 -0.2492 -0.3213 -0.6867 -0.7835
-0.4894 0.5090 -38.7916
87.1000 -40.3100 0.6039 0.2497 -
0.3504 -0.2091 -0.3247 -0.6803 -0.7721
-0.4941 0.5066 -38.7907
87.1200 -41.7000 -0.2257 -0.3566 -
0.3734 -0.1557 -0.3254 -0.6728 -0.7604
-0.4987 0.5041 -38.7898
87.1400 -42.2900 -0.5994 -0.7618 -
0.3245 -0.0916 -0.3234 -0.6643 -0.7483
-0.5033 0.5017 -38.7889
87.1600 -41.2500 0.1710 -0.6264 -
0.1924 -0.0211 -0.3188 -0.6548 -0.7359
-0.5078 0.4992 -38.7880
87.1800 -40.4100 0.2671 -0.1165 -
0.0030 0.0510 -0.3118 -0.6443 -0.7232
-0.5123 0.4967 -38.7871
87.2000 -39.9300 -0.1666 0.4001
0.1994 0.1192 -0.3024 -0.6329 -0.7102
-0.5167 0.4942 -38.7862
87.2200 -38.9700 0.1814 0.6488
0.3669 0.1782 -0.2908 -0.6206 -0.6969
-0.5210 0.4917 -38.7853
87.2400 -39.0200 0.0860 0.6092
0.4566 0.2226 -0.2770 -0.6074 -0.6833
-0.5253 0.4893 -38.7844
87.2600 -39.3800 -0.1807 0.4189
0.4492 0.2482 -0.2612 -0.5933 -0.6695
-0.5295 0.4868 -38.7835
87.2800 -39.3800 0.1699 0.2119
0.3567 0.2534 -0.2436 -0.5784 -0.6553
-0.5336 0.4843 -38.7826
87.3000 -39.9900 -0.1199 -0.0252
0.2137 0.2397 -0.2242 -0.5627 -0.6410
-0.5377 0.4818 -38.7817
87.3200 -39.9900 0.2931 -0.3299
0.0623 0.2092 -0.2032 -0.5462 -0.6263
-0.5417 0.4793 -38.7808
87.3400 -40.8900 -0.2841 -0.5370 -
0.0628 0.1646 -0.1807 -0.5291 -0.6115
-0.5457 0.4767 -38.7800
87.3600 -41.0000 -0.4541 -0.4193 -
0.1342 0.1086 -0.1569 -0.5112 -0.5963
-0.5496 0.4742 -38.7791
87.3800 -40.0100 0.2179 -0.0254 -
0.1485 0.0448 -0.1320 -0.4927 -0.5810
-0.5534 0.4717 -38.7782
87.4000 -39.5800 0.3684 0.2881 -
0.1228 -0.0219 -0.1063 -0.4735 -0.5655
-0.5572 0.4692 -38.7773

87.4200 -39.9200 -0.0382 0.2670 -
0.0814 -0.0861 -0.0799 -0.4538 -0.5497
-0.5609 0.4667 -38.7764
87.4400 -40.1900 -0.2208 0.0751 -
0.0481 -0.1415 -0.0530 -0.4336 -0.5337
-0.5646 0.4641 -38.7755
87.4600 -40.1200 0.0002 -0.0357 -
0.0446 -0.1823 -0.0260 -0.4128 -0.5176
-0.5682 0.4616 -38.7746
87.4800 -39.9800 0.0998 -0.0396 -
0.0809 -0.2046 0.0011 -0.3916 -0.5012
-0.5718 0.4591 -38.7737
87.5000 -40.0000 0.0415 -0.0033 -
0.1539 -0.2066 0.0279 -0.3700 -0.4847
-0.5753 0.4565 -38.7728
87.5200 -40.0700 -0.0348 0.0459 -
0.2498 -0.1885 0.0544 -0.3480 -0.4680
-0.5787 0.4540 -38.7719
87.5400 -40.0400 -0.0586 0.1063 -
0.3490 -0.1522 0.0803 -0.3257 -0.4512
-0.5821 0.4514 -38.7710
87.5600 -39.8200 0.1135 0.1136 -
0.4276 -0.1006 0.1055 -0.3030 -0.4342
-0.5854 0.4489 -38.7701
87.5800 -39.8500 0.1184 -0.0143 -
0.4580 -0.0375 0.1298 -0.2801 -0.4171
-0.5887 0.4463 -38.7692
87.6000 -40.1300 -0.1052 -0.1869 -
0.4183 0.0326 0.1532 -0.2570 -0.3998
-0.5919 0.4438 -38.7683
87.6200 -40.0100 -0.2695 -0.2137 -
0.3015 0.1054 0.1754 -0.2337 -0.3824
-0.5951 0.4412 -38.7674
87.6400 -39.0400 0.3018 -0.0918 -
0.1194 0.1766 0.1963 -0.2102 -0.3649
-0.5982 0.4386 -38.7666
87.6600 -38.9700 -0.1256 0.0227
0.1012 0.2419 0.2159 -0.1867 -0.3473
-0.6012 0.4361 -38.7657
87.6800 -38.3800 0.0390 0.0970
0.3251 0.2972 0.2339 -0.1631 -0.3296
-0.6042 0.4335 -38.7648
87.7000 -38.2200 -0.0878 0.1692
0.5147 0.3382 0.2503 -0.1394 -0.3117
-0.6072 0.4309 -38.7639
87.7200 -37.7800 0.1167 0.1854
0.6383 0.3610 0.2649 -0.1158 -0.2938
-0.6101 0.4284 -38.7630
87.7400 -37.9200 -0.0038 0.1149
0.6763 0.3623 0.2776 -0.0922 -0.2759
-0.6129 0.4258 -38.7621

87.7600 −38.1100 −0.0465 −0.0253
0.6295 0.3411 0.2885 −0.0687 −0.2578
−0.6157 0.4232 −38.7612
87.7800 −38.3500 −0.0212 −0.1461
0.5106 0.2991 0.2975 −0.0454 −0.2397
−0.6184 0.4206 −38.7603
87.8000 −38.4900 0.0257 −0.1505
0.3381 0.2403 0.3047 −0.0222 −0.2216
−0.6211 0.4180 −38.7594
87.8200 −38.6800 −0.0676 −0.0634
0.1339 0.1694 0.3103 0.0008 −0.2034
−0.6237 0.4155 −38.7586
87.8400 −38.7400 0.0337 0.0473 −
0.0810 0.0909 0.3143 0.0235 −0.1852
−0.6263 0.4129 −38.7577
87.8600 −38.9700 −0.0088 0.1339 −
0.2880 0.0096 0.3169 0.0460 −0.1669
−0.6288 0.4103 −38.7568
87.8800 −39.0800 0.0410 0.1313 −
0.4680 −0.0699 0.3182 0.0682 −0.1486
−0.6313 0.4077 −38.7559
87.9000 −39.3900 0.0944 0.0063 −
0.5989 −0.1430 0.3184 0.0900 −0.1304
−0.6337 0.4051 −38.7550
87.9200 −39.7700 −0.1933 −0.1312 −
0.6589 −0.2060 0.3176 0.1115 −0.1121
−0.6361 0.4025 −38.7541
87.9400 −39.6600 0.0564 −0.1506 −
0.6326 −0.2560 0.3160 0.1326 −0.0938
−0.6384 0.3999 −38.7533
87.9600 −39.4200 0.0713 −0.1038 −
0.5174 −0.2922 0.3135 0.1532 −0.0755
−0.6407 0.3973 −38.7524
87.9800 −39.3700 −0.1196 −0.0714 −
0.3267 −0.3150 0.3105 0.1734 −0.0573
−0.6429 0.3947 −38.7515
88.0000 −38.9700 0.0705 −0.0337 −
0.0923 −0.3258 0.3069 0.1931 −0.0391
−0.6451 0.3921 −38.7506
88.0200 −38.8800 −0.1463 0.0571
0.1423 −0.3258 0.3029 0.2123 −0.0210
−0.6472 0.3895 −38.7497
88.0400 −38.0900 0.2247 0.1830
0.3330 −0.3164 0.2985 0.2310 −0.0029
−0.6493 0.3869 −38.7488
88.0600 −38.0900 −0.0383 0.2224
0.4495 −0.2987 0.2939 0.2492 0.0152
−0.6513 0.3843 −38.7480
88.0800 −38.1600 0.0361 0.1155
0.4805 −0.2742 0.2890 0.2668 0.0332
−0.6533 0.3817 −38.7471

88.1000 -38.1600 0.0355 -0.0578
0.4359 -0.2446 0.2840 0.2839 0.0511
-0.6552 0.3791 -38.7462
88.1200 -38.4300 -0.0823 -0.1757
0.3400 -0.2116 0.2788 0.3005 0.0689
-0.6571 0.3765 -38.7453
88.1400 -38.4300 0.0279 -0.1503
0.2168 -0.1763 0.2733 0.3166 0.0867
-0.6589 0.3738 -38.7444
88.1600 -38.3700 -0.0173 -0.0030
0.0828 -0.1393 0.2674 0.3321 0.1043
-0.6607 0.3712 -38.7436
88.1800 -38.3700 -0.0339 0.1517 -
0.0478 -0.1006 0.2610 0.3471 0.1218
-0.6624 0.3686 -38.7427
88.2000 -38.3700 0.0048 0.1726 -
0.1611 -0.0602 0.2540 0.3617 0.1392
-0.6641 0.3660 -38.7418
88.2200 -38.4000 0.1274 0.0435 -
0.2433 -0.0185 0.2462 0.3757 0.1565
-0.6658 0.3634 -38.7409
88.2400 -38.6700 -0.1527 -0.1272 -
0.2780 0.0242 0.2374 0.3892 0.1737
-0.6674 0.3608 -38.7400
88.2600 -38.6400 -0.0646 -0.1954 -
0.2535 0.0669 0.2275 0.4023 0.1907
-0.6689 0.3582 -38.7392
88.2800 -38.3500 0.0943 -0.1417 -
0.1752 0.1086 0.2165 0.4149 0.2076
-0.6705 0.3555 -38.7383
88.3000 -38.2300 -0.0810 -0.0143 -
0.0645 0.1481 0.2042 0.4271 0.2243
-0.6719 0.3529 -38.7374
88.3200 -37.7500 0.0747 0.1168
0.0479 0.1842 0.1905 0.4388 0.2409
-0.6734 0.3503 -38.7365
88.3400 -37.7100 0.0363 0.1986
0.1333 0.2155 0.1755 0.4500 0.2573
-0.6747 0.3477 -38.7357
88.3600 -37.6300 -0.0758 0.2025
0.1784 0.2409 0.1589 0.4609 0.2735
-0.6761 0.3451 -38.7348
88.3800 -37.6700 -0.0025 0.0959
0.1842 0.2595 0.1408 0.4714 0.2895
-0.6774 0.3424 -38.7339
88.4000 -37.7200 0.0284 -0.0727
0.1616 0.2709 0.1211 0.4814 0.3053
-0.6786 0.3398 -38.7330
88.4200 -37.9500 0.0061 -0.1924
0.1261 0.2749 0.0998 0.4911 0.3210
-0.6798 0.3372 -38.7322

88.4400 -38.1000 -0.1511 -0.1692
0.0887 0.2718 0.0768 0.5003 0.3364
-0.6810 0.3346 -38.7313
88.4600 -37.8100 0.1016 -0.0101
0.0515 0.2623 0.0522 0.5092 0.3516
-0.6821 0.3320 -38.7304
88.4800 -37.7500 -0.0394 0.1421
0.0134 0.2474 0.0262 0.5178 0.3666
-0.6832 0.3293 -38.7295
88.5000 -37.8400 0.0012 0.1600 -
0.0265 0.2284 -0.0011 0.5259 0.3814
-0.6842 0.3267 -38.7287
88.5200 -37.9000 0.1098 0.0470 -
0.0656 0.2060 -0.0295 0.5337 0.3959
-0.6852 0.3241 -38.7278
88.5400 -38.3800 -0.1749 -0.0660 -
0.0965 0.1809 -0.0587 0.5411 0.4102
-0.6862 0.3215 -38.7269
88.5600 -38.3100 -0.0946 -0.0844 -
0.1084 0.1534 -0.0885 0.5482 0.4243
-0.6871 0.3189 -38.7260
88.5800 -38.0100 0.2343 -0.0551 -
0.0920 0.1239 -0.1185 0.5549 0.4381
-0.6880 0.3162 -38.7252
88.6000 -38.1300 0.0801 -0.0667 -
0.0473 0.0924 -0.1485 0.5612 0.4516
-0.6888 0.3136 -38.7243
88.6200 -38.3400 -0.1762 -0.0831
0.0170 0.0590 -0.1781 0.5672 0.4649
-0.6896 0.3110 -38.7234
88.6400 -38.1800 -0.0079 -0.0308
0.0864 0.0236 -0.2070 0.5728 0.4778
-0.6904 0.3084 -38.7226
88.6600 -38.0500 0.0254 0.0755
0.1445 -0.0138 -0.2349 0.5780 0.4905
-0.6911 0.3058 -38.7217
88.6800 -37.9900 0.0352 0.1347
0.1783 -0.0528 -0.2614 0.5829 0.5029
-0.6918 0.3032 -38.7208
88.7000 -37.9700 0.1282 0.0925
0.1799 -0.0928 -0.2862 0.5874 0.5151
-0.6924 0.3005 -38.7200
88.7200 -38.4000 -0.1774 0.0243
0.1455 -0.1328 -0.3091 0.5916 0.5269
-0.6930 0.2979 -38.7191
88.7400 -38.4900 -0.2011 0.0317
0.0784 -0.1709 -0.3297 0.5955 0.5384
-0.6936 0.2953 -38.7182
88.7600 -38.3300 0.1404 0.0600 -
0.0100 -0.2051 -0.3478 0.5989 0.5495
-0.6941 0.2927 -38.7174

88.7800 -38.3100 0.3560 -0.0199 －
0.1031 -0.2332 -0.3630 0.6021 0.5604
-0.6946 0.2901 -38.7165
88.8000 -39.1400 -0.2631 -0.1274 －
0.1834 -0.2530 -0.3751 0.6049 0.5709
-0.6950 0.2875 -38.7156
88.8200 -39.1400 -0.1811 -0.1038 －
0.2370 -0.2627 -0.3840 0.6073 0.5811
-0.6954 0.2849 -38.7148
88.8400 -38.6300 0.1783 0.0059 －
0.2562 -0.2611 -0.3894 0.6095 0.5910
-0.6958 0.2823 -38.7139
88.8600 -38.6300 0.1352 0.0311 －
0.2400 -0.2482 -0.3914 0.6113 0.6005
-0.6962 0.2797 -38.7130
88.8800 -38.8800 -0.0946 -0.0498 －
0.1932 -0.2250 -0.3900 0.6127 0.6097
-0.6965 0.2771 -38.7122
88.9000 -38.8800 -0.1490 -0.0909 －
0.1249 -0.1936 -0.3853 0.6139 0.6185
-0.6967 0.2745 -38.7113
88.9200 -38.4300 0.1202 -0.0099 －
0.0445 -0.1563 -0.3775 0.6147 0.6270
-0.6970 0.2719 -38.7104
88.9400 -38.3800 -0.1197 0.1221
0.0388 -0.1158 -0.3667 0.6152 0.6351
-0.6972 0.2693 -38.7096
88.9600 -37.8600 0.2521 0.1636
0.1150 -0.0750 -0.3530 0.6153 0.6429
-0.6974 0.2667 -38.7087
88.9800 -37.9300 -0.1334 0.0559
0.1769 -0.0366 -0.3367 0.6151 0.6503
-0.6975 0.2641 -38.7078
89.0000 -38.2500 -0.0135 -0.0877
0.2215 -0.0034 -0.3180 0.6146 0.6573
-0.6976 0.2615 -38.7070
89.0200 -38.0700 -0.0598 -0.1373
0.2492 0.0225 -0.2969 0.6137 0.6640
-0.6976 0.2589 -38.7061
89.0400 -37.7500 0.1875 -0.0742
0.2581 0.0397 -0.2738 0.6124 0.6704
-0.6977 0.2563 -38.7052
89.0600 -37.8000 -0.1025 0.0280
0.2425 0.0483 -0.2488 0.6107 0.6763
-0.6977 0.2537 -38.7044
89.0800 -37.8300 -0.0722 0.1076
0.1978 0.0491 -0.2221 0.6087 0.6819
-0.6976 0.2511 -38.7035
89.1000 -37.7100 0.0692 0.1315
0.1251 0.0441 -0.1941 0.6063 0.6872
-0.6976 0.2485 -38.7027

89.1200 -37.7900 0.1851 0.0535
0.0313 0.0351 -0.1649 0.6035 0.6921
-0.6975 0.2460 -38.7018
89.1400 -38.2900 -0.2028 -0.0826 -
0.0720 0.0243 -0.1348 0.6002 0.6966
-0.6973 0.2434 -38.7009
89.1600 -38.2500 0.0726 -0.1396 -
0.1697 0.0137 -0.1040 0.5966 0.7008
-0.6972 0.2408 -38.7001
89.1800 -38.1900 -0.0658 -0.0676 -
0.2454 0.0055 -0.0729 0.5925 0.7046
-0.6970 0.2382 -38.6992
89.2000 -38.0600 0.0098 0.0507 -
0.2850 0.0012 -0.0416 0.5880 0.7080
-0.6968 0.2356 -38.6984
89.2200 -38.0100 0.0539 0.0818 -
0.2813 0.0019 -0.0104 0.5830 0.7112
-0.6965 0.2331 -38.6975
89.2400 -38.1300 0.0289 -0.0104 -
0.2338 0.0080 0.0205 0.5776 0.7139
-0.6962 0.2305 -38.6966
89.2600 -38.1200 0.0170 -0.1231 -
0.1492 0.0186 0.0509 0.5717 0.7164
-0.6959 0.2279 -38.6958
89.2800 -38.0900 -0.2262 -0.1146 -
0.0387 0.0321 0.0807 0.5653 0.7185
-0.6955 0.2254 -38.6949
89.3000 -37.5900 0.0893 0.0441
0.0795 0.0466 0.1097 0.5583 0.7202
-0.6952 0.2228 -38.6941
89.3200 -37.0900 0.2109 0.1775
0.1824 0.0600 0.1378 0.5509 0.7217
-0.6947 0.2203 -38.6932
89.3400 -37.3500 -0.1192 0.1391
0.2492 0.0702 0.1650 0.5429 0.7228
-0.6943 0.2177 -38.6924
89.3600 -37.4000 -0.0877 0.0192
0.2671 0.0757 0.1910 0.5343 0.7235
-0.6938 0.2152 -38.6915
89.3800 -37.3500 0.0531 -0.0445
0.2346 0.0760 0.2158 0.5252 0.7240
-0.6933 0.2126 -38.6906
89.4000 -37.3900 0.0800 -0.0583
0.1642 0.0713 0.2391 0.5155 0.7241
-0.6928 0.2101 -38.6898
89.4200 -37.8000 -0.1238 -0.0790
0.0754 0.0627 0.2608 0.5053 0.7239
-0.6922 0.2075 -38.6889
89.4400 -37.7700 0.0105 -0.0635 -
0.0123 0.0513 0.2807 0.4944 0.7235
-0.6916 0.2050 -38.6881

89.4600 -37.6600 0.0895 0.0089 -
0.0833 0.0388 0.2987 0.4829 0.7227
-0.6910 0.2025 -38.6872
89.4800 -37.6800 -0.0337 0.0613 -
0.1325 0.0270 0.3146 0.4709 0.7216
-0.6904 0.1999 -38.6864
89.5000 -37.7200 -0.0141 0.0446 -
0.1582 0.0178 0.3281 0.4582 0.7202
-0.6897 0.1974 -38.6855
89.5200 -37.8000 0.0144 0.0086 -
0.1612 0.0130 0.3392 0.4449 0.7186
-0.6890 0.1949 -38.6847
89.5400 -37.9300 -0.0633 0.0017 -
0.1438 0.0133 0.3476 0.4311 0.7166
-0.6883 0.1924 -38.6838
89.5600 -37.8000 0.0564 -0.0010 -
0.1069 0.0188 0.3532 0.4167 0.7144
-0.6875 0.1899 -38.6830
89.5800 -37.6500 0.0406 -0.0213 -
0.0538 0.0289 0.3559 0.4017 0.7119
-0.6867 0.1873 -38.6821
89.6000 -37.6900 -0.0212 -0.0385
0.0110 0.0426 0.3556 0.3862 0.7091
-0.6859 0.1848 -38.6813
89.6200 -37.7200 -0.1238 -0.0309
0.0786 0.0589 0.3522 0.3702 0.7061
-0.6850 0.1823 -38.6804
89.6400 -37.5200 0.0692 0.0087
0.1357 0.0761 0.3456 0.3537 0.7028
-0.6842 0.1798 -38.6796
89.6600 -37.4300 0.0377 0.0522
0.1687 0.0927 0.3359 0.3368 0.6993
-0.6833 0.1773 -38.6787
89.6800 -37.5300 -0.1166 0.0854
0.1695 0.1070 0.3230 0.3195 0.6955
-0.6823 0.1748 -38.6779
89.7000 -37.5500 0.0644 0.0494
0.1388 0.1183 0.3071 0.3018 0.6915
-0.6814 0.1723 -38.6770
89.7200 -37.7900 -0.0291 -0.0367
0.0849 0.1264 0.2884 0.2838 0.6872
-0.6804 0.1699 -38.6762
89.7400 -37.8200 0.1225 -0.0775
0.0152 0.1314 0.2669 0.2655 0.6827
-0.6794 0.1674 -38.6753
89.7600 -38.1100 -0.1624 -0.0524 -
0.0596 0.1338 0.2430 0.2469 0.6780
-0.6784 0.1649 -38.6745
89.7800 -38.1400 -0.0863 0.0597 -
0.1300 0.1341 0.2168 0.2281 0.6731
-0.6773 0.1624 -38.6736

89.8000 -37.9900 0.0630 0.1401 -
0.1869 0.1330 0.1886 0.2091 0.6679
-0.6762 0.1600 -38.6728
89.8200 -37.9700 0.1938 0.0693 -
0.2211 0.1305 0.1587 0.1899 0.6626
-0.6751 0.1575 -38.6719
89.8400 -38.5700 -0.1193 -0.0833 -
0.2229 0.1268 0.1274 0.1707 0.6570
-0.6740 0.1550 -38.6711
89.8600 -38.6500 -0.0579 -0.1544 -
0.1852 0.1215 0.0950 0.1514 0.6512
-0.6728 0.1526 -38.6702
89.8800 -38.4700 0.0750 -0.1600 -
0.1048 0.1134 0.0619 0.1320 0.6453
-0.6716 0.1501 -38.6694
89.9000 -38.4200 0.0715 -0.2026
0.0165 0.1014 0.0284 0.1127 0.6391
-0.6704 0.1477 -38.6685
89.9200 -38.4200 -0.0294 -0.2221
0.1598 0.0843 -0.0050 0.0934 0.6327
-0.6692 0.1453 -38.6677
89.9400 -38.4200 -0.1802 -0.0668
0.2928 0.0615 -0.0379 0.0742 0.6262
-0.6679 0.1428 -38.6668
89.9600 -37.8400 0.0409 0.2703
0.3785 0.0325 -0.0699 0.0552 0.6195
-0.6666 0.1404 -38.6660
89.9800 -37.7000 0.0339 0.5364
0.3870 -0.0030 -0.1005 0.0363 0.6126
-0.6653 0.1380 -38.6651
90.0000 -37.9300 0.0010 0.5620
0.3031 -0.0443 -0.1294 0.0176 0.6056
-0.6640 0.1355 -38.6643
90.0200 -38.4000 -0.0937 0.3441
0.1417 -0.0890 -0.1562 -0.0009 0.5984
-0.6627 0.1331 -38.6635
90.0400 -38.6600 0.4111 -0.0841 -
0.0575 -0.1337 -0.1807 -0.0190 0.5910
-0.6613 0.1307 -38.6626
90.0600 -40.2300 -0.4244 -0.5520 -
0.2436 -0.1746 -0.2025 -0.0368 0.5835
-0.6599 0.1283 -38.6618
90.0800 -40.2300 0.0160 -0.7163 -
0.3669 -0.2083 -0.2212 -0.0543 0.5758
-0.6585 0.1259 -38.6609
90.1000 -40.2300 -0.2124 -0.4627 -
0.3961 -0.2317 -0.2368 -0.0713 0.5680
-0.6570 0.1235 -38.6601
90.1200 -39.3200 0.3922 -0.0787 -
0.3365 -0.2430 -0.2489 -0.0880 0.5601
-0.6556 0.1211 -38.6592

90.1400 -39.3200 0.0277 0.1918 -
0.2135 -0.2428 -0.2576 -0.1041 0.5520
-0.6541 0.1187 -38.6584
90.1600 -39.3200 -0.3560 0.3435 -
0.0651 -0.2325 -0.2626 -0.1197 0.5438
-0.6526 0.1164 -38.6576
90.1800 -38.8600 -0.0741 0.4317
0.0674 -0.2139 -0.2643 -0.1348 0.5355
-0.6511 0.1140 -38.6567
90.2000 -38.6700 0.0854 0.4114
0.1571 -0.1890 -0.2626 -0.1494 0.5270
-0.6495 0.1116 -38.6559
90.2200 -38.6700 0.2160 0.1915
0.1924 -0.1594 -0.2577 -0.1633 0.5185
-0.6480 0.1093 -38.6550
90.2400 -39.3400 -0.1906 -0.1276
0.1773 -0.1266 -0.2500 -0.1767 0.5098
-0.6464 0.1069 -38.6542
90.2600 -39.4400 -0.0152 -0.2889
0.1329 -0.0919 -0.2396 -0.1895 0.5010
-0.6448 0.1045 -38.6534
90.2800 -39.3800 -0.1175 -0.1980
0.0845 -0.0569 -0.2269 -0.2016 0.4922
-0.6432 0.1022 -38.6525
90.3000 -39.0300 0.1247 -0.0134
0.0463 -0.0228 -0.2122 -0.2132 0.4832
-0.6415 0.0999 -38.6517
90.3200 -39.0300 0.0151 0.0818
0.0226 0.0095 -0.1960 -0.2240 0.4742
-0.6398 0.0975 -38.6508
90.3400 -39.0300 -0.0778 0.0649
0.0080 0.0405 -0.1785 -0.2343 0.4650
-0.6382 0.0952 -38.6500
90.3600 -39.0400 0.0488 0.0217 -
0.0043 0.0705 -0.1604 -0.2439 0.4558
-0.6365 0.0929 -38.6492
90.3800 -39.0400 -0.0280 -0.0128 -
0.0183 0.0997 -0.1420 -0.2528 0.4465
-0.6348 0.0906 -38.6483
90.4000 -39.0300 0.0062 -0.0311 -
0.0375 0.1280 -0.1237 -0.2611 0.4372
-0.6330 0.0882 -38.6475
90.4200 -39.0300 -0.0189 -0.0293 -
0.0597 0.1546 -0.1059 -0.2687 0.4277
-0.6313 0.0859 -38.6467
90.4400 -39.0300 -0.0477 -0.0018 -
0.0797 0.1784 -0.0888 -0.2756 0.4182
-0.6295 0.0836 -38.6458
90.4600 -38.9000 0.0437 0.0323 -
0.0908 0.1979 -0.0726 -0.2819 0.4087
-0.6277 0.0813 -38.6450

90.4800 -38.8800 -0.0140 0.0487 -
0.0839 0.2113 -0.0575 -0.2875 0.3991
-0.6259 0.0791 -38.6442
90.5000 -38.8800 -0.0388 0.0402 -
0.0537 0.2171 -0.0435 -0.2925 0.3894
-0.6241 0.0768 -38.6433
90.5200 -38.9100 -0.0169 0.0201 -
0.0003 0.2139 -0.0307 -0.2969 0.3797
-0.6222 0.0745 -38.6425
90.5400 -38.9100 -0.0186 -0.0405
0.0692 0.2009 -0.0191 -0.3006 0.3699
-0.6204 0.0722 -38.6416
90.5600 -38.8900 0.0243 -0.1246
0.1400 0.1778 -0.0087 -0.3037 0.3601
-0.6185 0.0700 -38.6408
90.5800 -38.8600 -0.0436 -0.1238
0.1943 0.1448 0.0006 -0.3063 0.3503
-0.6166 0.0677 -38.6400
90.6000 -38.8600 -0.1115 0.0065
0.2161 0.1027 0.0088 -0.3083 0.3404
-0.6147 0.0655 -38.6391
90.6200 -38.7300 0.0236 0.1905
0.1965 0.0533 0.0160 -0.3097 0.3305
-0.6128 0.0632 -38.6383
90.6400 -38.6800 0.1086 0.2858
0.1344 -0.0008 0.0223 -0.3106 0.3206
-0.6109 0.0610 -38.6375
90.6600 -38.6800 0.3805 0.1360
0.0389 -0.0558 0.0278 -0.3111 0.3106
-0.6090 0.0588 -38.6366
90.6800 -39.9900 -0.3084 -0.1768 -
0.0694 -0.1078 0.0328 -0.3111 0.3006
-0.6070 0.0565 -38.6358
90.7000 -39.9900 -0.1741 -0.3344 -
0.1664 -0.1529 0.0372 -0.3106 0.2907
-0.6050 0.0543 -38.6350
90.7200 -39.8400 -0.0034 -0.1861 -
0.2313 -0.1873 0.0412 -0.3098 0.2807
-0.6031 0.0521 -38.6342
90.7400 -39.4700 0.1855 0.0553 -
0.2555 -0.2085 0.0450 -0.3085 0.2707
-0.6011 0.0499 -38.6333
90.7600 -39.4700 -0.0367 0.1703 -
0.2399 -0.2150 0.0486 -0.3069 0.2607
-0.5991 0.0477 -38.6325
90.7800 -39.4600 -0.0309 0.1473 -
0.1895 -0.2067 0.0522 -0.3050 0.2507
-0.5970 0.0455 -38.6317
90.8000 -39.4600 -0.0002 0.0498 -
0.1146 -0.1852 0.0558 -0.3028 0.2407
-0.5950 0.0434 -38.6308

90.8200 -39.4600 0.0044 -0.0671 -
0.0278 -0.1527 0.0594 -0.3003 0.2307
-0.5930 0.0412 -38.6300
90.8400 -39.3400 0.0931 -0.1388
0.0572 -0.1122 0.0631 -0.2976 0.2208
-0.5909 0.0390 -38.6292
90.8600 -39.3200 -0.0900 -0.0930
0.1264 -0.0667 0.0667 -0.2947 0.2108
-0.5888 0.0369 -38.6283
90.8800 -39.2100 -0.2334 0.0996
0.1686 -0.0194 0.0704 -0.2917 0.2009
-0.5868 0.0347 -38.6275
90.9000 -38.5700 0.2371 0.2877
0.1761 0.0263 0.0741 -0.2884 0.1910
-0.5847 0.0326 -38.6267
90.9200 -38.5700 0.1389 0.2661
0.1488 0.0674 0.0777 -0.2851 0.1811
-0.5826 0.0304 -38.6259
90.9400 -39.2400 -0.1976 0.0144
0.1016 0.1010 0.0813 -0.2816 0.1713
-0.5805 0.0283 -38.6250
90.9600 -39.3500 -0.0171 -0.2480
0.0570 0.1254 0.0848 -0.2781 0.1615
-0.5783 0.0262 -38.6242
90.9800 -39.3500 0.0001 -0.3381
0.0366 0.1387 0.0882 -0.2745 0.1517
-0.5762 0.0241 -38.6234
91.0000 -39.1000 0.0703 -0.2243
0.0492 0.1405 0.0914 -0.2709 0.1420
-0.5741 0.0219 -38.6226
91.0200 -39.0200 0.0041 -0.0202
0.0861 0.1317 0.0944 -0.2672 0.1323
-0.5719 0.0198 -38.6217
91.0400 -38.9800 -0.0711 0.1411
0.1257 0.1141 0.0972 -0.2635 0.1227
-0.5698 0.0178 -38.6209
91.0600 -38.6700 0.0712 0.2281
0.1404 0.0899 0.0996 -0.2597 0.1132
-0.5676 0.0157 -38.6201
91.0800 -38.6700 0.1384 0.2279
0.1125 0.0617 0.1016 -0.2560 0.1036
-0.5654 0.0136 -38.6193
91.1000 -39.0000 -0.0438 0.1128
0.0401 0.0321 0.1030 -0.2523 0.0942
-0.5633 0.0115 -38.6184
91.1200 -39.4300 -0.1405 -0.0547 -
0.0609 0.0033 0.1039 -0.2486 0.0848
-0.5611 0.0095 -38.6176
91.1400 -39.4300 0.1115 -0.1664 -
0.1636 -0.0224 0.1040 -0.2450 0.0755
-0.5589 0.0074 -38.6168

91.1600 -39.7400 -0.0712 -0.1774 -
0.2404 -0.0430 0.1033 -0.2413 0.0662
-0.5567 0.0054 -38.6160
91.1800 -39.7400 -0.0977 -0.0909 -
0.2692 -0.0570 0.1017 -0.2377 0.0570
-0.5545 0.0033 -38.6151
91.2000 -39.4800 0.0579 0.0414 -
0.2425 -0.0638 0.0992 -0.2341 0.0479
-0.5522 0.0013 -38.6143
91.2200 -39.3200 0.0978 0.1008 -
0.1661 -0.0637 0.0958 -0.2306 0.0389
-0.5500 -0.0007 -38.6135
91.2400 -39.3400 0.0594 0.0111 -
0.0541 -0.0575 0.0915 -0.2271 0.0299
-0.5478 -0.0028 -38.6127
91.2600 -39.4000 -0.0433 -0.1194
0.0712 -0.0466 0.0862 -0.2236 0.0210
-0.5456 -0.0048 -38.6118
91.2800 -39.4000 -0.2415 -0.1151
0.1843 -0.0323 0.0800 -0.2201 0.0122
-0.5433 -0.0068 -38.6110
91.3000 -38.8600 0.1334 0.0643
0.2617 -0.0162 0.0729 -0.2167 0.0035
-0.5411 -0.0088 -38.6102
91.3200 -38.6800 0.1648 0.2207
0.2879 0.0002 0.0650 -0.2133 -0.0052
-0.5388 -0.0107 -38.6094
91.3400 -38.8800 0.1166 0.1357
0.2589 0.0154 0.0564 -0.2100 -0.0138
-0.5366 -0.0127 -38.6086
91.3600 -39.4700 -0.2041 -0.1120
0.1850 0.0285 0.0471 -0.2066 -0.0223
-0.5343 -0.0147 -38.6077
91.3800 -39.4700 -0.0423 -0.2201
0.0838 0.0391 0.0372 -0.2032 -0.0307
-0.5320 -0.0166 -38.6069
91.4000 -39.4000 0.0060 -0.0825 -
0.0285 0.0471 0.0266 -0.1999 -0.0390
-0.5297 -0.0186 -38.6061
91.4200 -39.3800 0.0286 0.1235 -
0.1372 0.0529 0.0157 -0.1966 -0.0472
-0.5275 -0.0205 -38.6053
91.4400 -39.4200 -0.0570 0.1969 -
0.2300 0.0565 0.0043 -0.1932 -0.0554
-0.5252 -0.0225 -38.6045
91.4600 -39.5500 -0.0326 0.1320 -
0.2923 0.0580 -0.0072 -0.1899 -0.0635
-0.5229 -0.0244 -38.6037
91.4800 -39.5500 0.1402 0.0048 -
0.3094 0.0576 -0.0189 -0.1865 -0.0714
-0.5206 -0.0263 -38.6028

91.5000 -39.8000 -0.0526 -0.1509 -
0.2663 0.0554 -0.0305 -0.1831 -0.0793
-0.5183 -0.0282 -38.6020
91.5200 -39.9000 -0.0689 -0.2534 -
0.1597 0.0513 -0.0420 -0.1797 -0.0871
-0.5160 -0.0301 -38.6012
91.5400 -39.7900 -0.0906 -0.2134 -
0.0064 0.0448 -0.0531 -0.1763 -0.0948
-0.5137 -0.0320 -38.6004
91.5600 -39.0600 0.1710 -0.0447
0.1605 0.0355 -0.0638 -0.1729 -0.1024
-0.5114 -0.0339 -38.5996
91.5800 -39.0600 -0.0748 0.1417
0.3011 0.0230 -0.0738 -0.1694 -0.1100
-0.5090 -0.0358 -38.5987
91.6000 -38.9500 -0.0190 0.2703
0.3749 0.0067 -0.0830 -0.1660 -0.1174
-0.5067 -0.0376 -38.5979
91.6200 -38.8700 -0.0378 0.3019
0.3595 -0.0131 -0.0914 -0.1625 -0.1247
-0.5044 -0.0395 -38.5971
91.6400 -38.8700 0.3305 0.1565
0.2633 -0.0351 -0.0986 -0.1589 -0.1320
-0.5021 -0.0414 -38.5963
91.6600 -39.8200 -0.2856 -0.0990
0.1201 -0.0575 -0.1047 -0.1554 -0.1391
-0.4997 -0.0432 -38.5955
91.6800 -39.8200 0.0659 -0.2703 -
0.0266 -0.0782 -0.1096 -0.1518 -0.1462
-0.4974 -0.0450 -38.5947
91.7000 -39.9100 0.0966 -0.2956 -
0.1383 -0.0958 -0.1132 -0.1482 -0.1531
-0.4951 -0.0469 -38.5939
91.7200 -40.0000 -0.0243 -0.2270 -
0.1960 -0.1081 -0.1153 -0.1446 -0.1599
-0.4927 -0.0487 -38.5930
91.7400 -40.0000 -0.0615 -0.0690 -
0.2035 -0.1137 -0.1161 -0.1410 -0.1667
-0.4904 -0.0505 -38.5922
91.7600 -39.4900 0.1064 0.1476 -
0.1821 -0.1114 -0.1154 -0.1374 -0.1733
-0.4880 -0.0523 -38.5914
91.7800 -39.4900 0.0869 0.2639 -
0.1568 -0.1004 -0.1133 -0.1338 -0.1799
-0.4857 -0.0541 -38.5906
91.8000 -39.5900 -0.0822 0.2410 -
0.1433 -0.0809 -0.1097 -0.1301 -0.1863
-0.4833 -0.0558 -38.5898
91.8200 -39.6600 -0.0534 0.1464 -
0.1409 -0.0543 -0.1048 -0.1265 -0.1926
-0.4809 -0.0576 -38.5890

91.8400 -39.7100 0.0503 -0.0122 -
0.1349 -0.0225 -0.0985 -0.1229 -0.1988
-0.4786 -0.0594 -38.5882
91.8600 -39.7300 0.1784 -0.2455 -
0.1081 0.0117 -0.0911 -0.1193 -0.2049
-0.4762 -0.0611 -38.5874
91.8800 -40.0500 -0.1523 -0.3899 -
0.0517 0.0457 -0.0826 -0.1158 -0.2109
-0.4738 -0.0629 -38.5865
91.9000 -40.1300 -0.4857 -0.2105
0.0331 0.0766 -0.0731 -0.1123 -0.2168
-0.4715 -0.0646 -38.5857
91.9200 -38.7100 0.4137 0.2136
0.1268 0.1020 -0.0627 -0.1089 -0.2225
-0.4691 -0.0663 -38.5849
91.9400 -38.4000 0.2689 0.4638
0.2028 0.1200 -0.0516 -0.1056 -0.2282
-0.4667 -0.0681 -38.5841
91.9600 -39.0800 -0.2726 0.3344
0.2393 0.1289 -0.0399 -0.1023 -0.2337
-0.4644 -0.0698 -38.5833
91.9800 -39.1700 0.0485 -0.0289
0.2307 0.1276 -0.0276 -0.0992 -0.2391
-0.4620 -0.0715 -38.5825
92.0000 -39.6500 -0.1117 -0.3331
0.1827 0.1162 -0.0148 -0.0962 -0.2444
-0.4596 -0.0732 -38.5817
92.0200 -39.7000 -0.1526 -0.3575
0.1048 0.0957 -0.0017 -0.0933 -0.2496
-0.4572 -0.0748 -38.5809
92.0400 -39.3900 0.0448 -0.0936
0.0090 0.0680 0.0117 -0.0905 -0.2547
-0.4548 -0.0765 -38.5801
92.0600 -39.3100 -0.0788 0.2422 -
0.0881 0.0352 0.0252 -0.0879 -0.2596
-0.4524 -0.0782 -38.5793
92.0800 -39.1000 0.0812 0.3668 -
0.1680 -0.0002 0.0389 -0.0855 -0.2644
-0.4500 -0.0798 -38.5785
92.1000 -39.0700 0.3824 0.1608 -
0.2164 -0.0360 0.0524 -0.0833 -0.2691
-0.4477 -0.0815 -38.5776
92.1200 -40.1700 -0.2948 -0.1971 -
0.2249 -0.0698 0.0659 -0.0813 -0.2737
-0.4453 -0.0831 -38.5768
92.1400 -40.4400 -0.3800 -0.3730 -
0.1894 -0.0998 0.0791 -0.0794 -0.2781
-0.4429 -0.0848 -38.5760
92.1600 -39.7000 0.1289 -0.2016 -
0.1133 -0.1240 0.0920 -0.0779 -0.2824
-0.4405 -0.0864 -38.5752

92.1800 -39.3800 0.0058 0.1463 -
0.0152 -0.1413 0.1043 -0.0765 -0.2866
-0.4381 -0.0880 -38.5744
92.2000 -38.9800 0.1931 0.3305
0.0771 -0.1508 0.1160 -0.0754 -0.2907
-0.4357 -0.0896 -38.5736
92.2200 -38.8100 0.3096 0.2086
0.1373 -0.1520 0.1270 -0.0745 -0.2946
-0.4333 -0.0912 -38.5728
92.2400 -39.5200 -0.0641 -0.0780
0.1538 -0.1448 0.1370 -0.0738 -0.2984
-0.4309 -0.0928 -38.5720
92.2600 -39.8800 -0.3873 -0.2298
0.1279 -0.1297 0.1460 -0.0734 -0.3021
-0.4285 -0.0943 -38.5712
92.2800 -39.3400 0.0862 -0.0788
0.0681 -0.1076 0.1539 -0.0733 -0.3056
-0.4261 -0.0959 -38.5704
92.3000 -38.9800 0.2152 0.1323 -
0.0064 -0.0794 0.1606 -0.0734 -0.3090
-0.4237 -0.0975 -38.5696
92.3200 -39.2900 0.0365 0.1292 -
0.0725 -0.0465 0.1661 -0.0737 -0.3123
-0.4213 -0.0990 -38.5688
92.3400 -39.5000 0.0103 -0.0948 -
0.1125 -0.0102 0.1701 -0.0743 -0.3155
-0.4189 -0.1006 -38.5680
92.3600 -39.7400 -0.0324 -0.2827 -
0.1187 0.0278 0.1725 -0.0751 -0.3185
-0.4165 -0.1021 -38.5672
92.3800 -39.8800 -0.4058 -0.1902 -
0.0918 0.0662 0.1733 -0.0762 -0.3214
-0.4141 -0.1036 -38.5664
92.4000 -38.7200 0.3520 0.1003 -
0.0425 0.1032 0.1724 -0.0774 -0.3242
-0.4117 -0.1051 -38.5656
92.4200 -38.7000 0.1953 0.2816
0.0106 0.1371 0.1695 -0.0789 -0.3269
-0.4093 -0.1066 -38.5648
92.4400 -39.1100 -0.2443 0.2195
0.0493 0.1662 0.1647 -0.0806 -0.3294
-0.4069 -0.1081 -38.5640
92.4600 -39.0800 -0.1184 0.0698
0.0647 0.1893 0.1578 -0.0825 -0.3318
-0.4045 -0.1096 -38.5632
92.4800 -38.9500 0.1789 -0.0432
0.0605 0.2053 0.1488 -0.0846 -0.3340
-0.4021 -0.1111 -38.5624
92.5000 -39.1300 0.1335 -0.1705
0.0488 0.2135 0.1377 -0.0868 -0.3361
-0.3997 -0.1126 -38.5616

92.5200 -39.6000 -0.2456 -0.2375
0.0400 0.2135 0.1247 -0.0891 -0.3381
-0.3973 -0.1140 -38.5608
92.5400 -39.4100 -0.1109 -0.0943
0.0366 0.2056 0.1098 -0.0916 -0.3400
-0.3949 -0.1155 -38.5600
92.5600 -38.9500 0.1622 0.1256
0.0341 0.1906 0.0932 -0.0942 -0.3417
-0.3925 -0.1169 -38.5592
92.5800 -38.9900 0.0985 0.1795
0.0266 0.1697 0.0750 -0.0969 -0.3433
-0.3901 -0.1184 -38.5584
92.6000 -39.3800 -0.0928 0.0516
0.0110 0.1440 0.0554 -0.0996 -0.3448
-0.3877 -0.1198 -38.5576
92.6200 -39.4000 -0.0666 -0.0749 -
0.0098 0.1144 0.0349 -0.1024 -0.3462
-0.3853 -0.1212 -38.5568
92.6400 -39.4800 0.0244 -0.0831 -
0.0276 0.0819 0.0136 -0.1052 -0.3474
-0.3829 -0.1226 -38.5560
92.6600 -39.4800 0.1114 -0.0350 -
0.0360 0.0472 -0.0082 -0.1080 -0.3485
-0.3805 -0.1240 -38.5552
92.6800 -39.5800 0.0410 -0.0057 -
0.0312 0.0108 -0.0300 -0.1108 -0.3494
-0.3781 -0.1254 -38.5544
92.7000 -39.5800 -0.1235 0.0443 -
0.0119 -0.0261 -0.0516 -0.1135 -0.3503
-0.3757 -0.1268 -38.5536
92.7200 -39.5000 0.0048 0.0941
0.0200 -0.0626 -0.0727 -0.1162 -0.3510
-0.3733 -0.1282 -38.5528
92.7400 -39.4900 0.1414 0.0278
0.0581 -0.0975 -0.0927 -0.1188 -0.3515
-0.3709 -0.1295 -38.5520
92.7600 -39.8600 -0.0906 -0.1300
0.0913 -0.1297 -0.1116 -0.1212 -0.3520
-0.3685 -0.1309 -38.5512
92.7800 -40.0500 -0.2046 -0.1473
0.1036 -0.1582 -0.1288 -0.1235 -0.3523
-0.3661 -0.1322 -38.5504
92.8000 -39.7300 0.0053 0.0586
0.0850 -0.1815 -0.1441 -0.1257 -0.3525
-0.3637 -0.1336 -38.5496
92.8200 -39.3200 0.2474 0.2822
0.0371 -0.1986 -0.1574 -0.1277 -0.3526
-0.3613 -0.1349 -38.5488
92.8400 -39.5200 0.0483 0.2739 -
0.0321 -0.2085 -0.1683 -0.1294 -0.3526
-0.3589 -0.1362 -38.5480

92.8600 -40.2100 -0.2793 0.0389 -
0.1053 -0.2105 -0.1767 -0.1310 -0.3524
-0.3565 -0.1375 -38.5472
92.8800 -40.2100 0.1425 -0.2613 -
0.1592 -0.2042 -0.1824 -0.1323 -0.3521
-0.3541 -0.1388 -38.5464
92.9000 -40.5500 0.0128 -0.4410 -
0.1720 -0.1896 -0.1856 -0.1334 -0.3517
-0.3517 -0.1401 -38.5456
92.9200 -40.5900 -0.2444 -0.3634 -
0.1338 -0.1673 -0.1861 -0.1342 -0.3512
-0.3493 -0.1414 -38.5448
92.9400 -39.8000 0.1118 -0.0503 -
0.0580 -0.1387 -0.1842 -0.1347 -0.3506
-0.3469 -0.1427 -38.5440
92.9600 -39.4100 0.1145 0.2670
0.0281 -0.1056 -0.1800 -0.1349 -0.3498
-0.3445 -0.1440 -38.5432
92.9800 -39.3300 -0.1125 0.4074
0.0941 -0.0696 -0.1736 -0.1349 -0.3490
-0.3421 -0.1452 -38.5425
93.0000 -39.1600 0.0248 0.3606
0.1169 -0.0327 -0.1653 -0.1347 -0.3480
-0.3397 -0.1465 -38.5417
93.0200 -39.1600 0.2475 0.1345
0.0989 0.0037 -0.1552 -0.1341 -0.3469
-0.3373 -0.1478 -38.5409
93.0400 -39.9400 -0.1563 -0.1798
0.0572 0.0379 -0.1435 -0.1333 -0.3457
-0.3349 -0.1490 -38.5401
93.0600 -39.9400 -0.0390 -0.3649
0.0113 0.0688 -0.1304 -0.1322 -0.3445
-0.3325 -0.1502 -38.5393
93.0800 -39.7000 0.0734 -0.3084 -
0.0200 0.0955 -0.1161 -0.1309 -0.3431
-0.3302 -0.1514 -38.5385
93.1000 -39.6300 0.0222 -0.0845 -
0.0285 0.1170 -0.1008 -0.1294 -0.3416
-0.3278 -0.1527 -38.5377
93.1200 -39.2900 -0.1279 0.1503 -
0.0164 0.1326 -0.0848 -0.1276 -0.3400
-0.3254 -0.1539 -38.5369
93.1400 -39.0100 0.2248 0.2353
0.0084 0.1417 -0.0683 -0.1256 -0.3383
-0.3230 -0.1551 -38.5361
93.1600 -39.1500 -0.0082 0.1384
0.0357 0.1438 -0.0514 -0.1234 -0.3365
-0.3206 -0.1563 -38.5353
93.1800 -39.4400 -0.1139 -0.0262
0.0574 0.1388 -0.0344 -0.1210 -0.3346
-0.3182 -0.1575 -38.5345

93.2000 -39.4100 -0.0312 -0.0986
0.0696 0.1269 -0.0174 -0.1184 -0.3326
-0.3158 -0.1586 -38.5337
93.2200 -39.1800 0.0615 -0.0448
0.0746 0.1083 -0.0008 -0.1156 -0.3306
-0.3134 -0.1598 -38.5330
93.2400 -39.1800 0.0982 0.0305
0.0755 0.0840 0.0155 -0.1126 -0.3284
-0.3110 -0.1610 -38.5322
93.2600 -39.2700 -0.0479 0.0490
0.0714 0.0554 0.0311 -0.1095 -0.3262
-0.3086 -0.1621 -38.5314
93.2800 -39.2800 0.0170 0.0246
0.0614 0.0245 0.0461 -0.1062 -0.3239
-0.3062 -0.1633 -38.5306
93.3000 -39.3600 0.0539 0.0071
0.0431 -0.0069 0.0602 -0.1029 -0.3215
-0.3038 -0.1644 -38.5298
93.3200 -39.3900 -0.0653 0.0033
0.0141 -0.0366 0.0733 -0.0994 -0.3191
-0.3014 -0.1655 -38.5290
93.3400 -39.4100 0.0280 -0.0279 -
0.0233 -0.0623 0.0854 -0.0957 -0.3166
-0.2990 -0.1667 -38.5282
93.3600 -39.5300 0.0720 -0.0584 -
0.0634 -0.0821 0.0963 -0.0920 -0.3140
-0.2966 -0.1678 -38.5274
93.3800 -39.5300 -0.0784 -0.0553 -
0.0973 -0.0942 0.1059 -0.0882 -0.3113
-0.2942 -0.1689 -38.5267
93.4000 -39.4500 -0.0289 0.0090 -
0.1204 -0.0975 0.1144 -0.0843 -0.3086
-0.2918 -0.1700 -38.5259
93.4200 -39.3400 0.0661 0.0939 -
0.1304 -0.0919 0.1215 -0.0804 -0.3058
-0.2893 -0.1711 -38.5251
93.4400 -39.3400 -0.0305 0.1150 -
0.1282 -0.0780 0.1274 -0.0764 -0.3029
-0.2869 -0.1722 -38.5243
93.4600 -39.3500 0.0711 0.0423 -
0.1146 -0.0575 0.1320 -0.0724 -0.3000
-0.2845 -0.1732 -38.5235
93.4800 -39.3700 -0.0187 -0.0648 -
0.0870 -0.0326 0.1353 -0.0683 -0.2970
-0.2821 -0.1743 -38.5227
93.5000 -39.3700 -0.0535 -0.1212 -
0.0415 -0.0059 0.1375 -0.0642 -0.2940
-0.2797 -0.1754 -38.5219
93.5200 -39.1900 0.1155 -0.1174
0.0205 0.0204 0.1386 -0.0601 -0.2910
-0.2773 -0.1764 -38.5212

93.5400 -39.0500 0.0416 -0.0762
0.0900 0.0440 0.1385 -0.0560 -0.2879
-0.2749 -0.1775 -38.5204
93.5600 -39.0500 -0.1498 -0.0014
0.1537 0.0629 0.1374 -0.0519 -0.2847
-0.2724 -0.1785 -38.5196
93.5800 -38.8000 -0.0418 0.1203
0.1962 0.0755 0.1351 -0.0479 -0.2815
-0.2700 -0.1796 -38.5188
93.6000 -38.6200 0.1923 0.1866
0.2080 0.0805 0.1318 -0.0439 -0.2783
-0.2676 -0.1806 -38.5180
93.6200 -38.6200 0.1874 0.0873
0.1853 0.0775 0.1274 -0.0399 -0.2750
-0.2651 -0.1816 -38.5172
93.6400 -39.1300 -0.1480 -0.1007
0.1358 0.0670 0.1220 -0.0359 -0.2717
-0.2627 -0.1826 -38.5165
93.6600 -39.3300 -0.0750 -0.2016
0.0744 0.0504 0.1156 -0.0321 -0.2684
-0.2603 -0.1837 -38.5157
93.6800 -39.3300 -0.1084 -0.1311
0.0153 0.0294 0.1084 -0.0283 -0.2651
-0.2578 -0.1847 -38.5149
93.7000 -39.0800 0.0839 0.0494 -
0.0349 0.0062 0.1002 -0.0245 -0.2617
-0.2554 -0.1857 -38.5141
93.7200 -38.9800 0.1029 0.1849 -
0.0769 -0.0167 0.0913 -0.0209 -0.2583
-0.2529 -0.1866 -38.5133
93.7400 -38.9800 0.1152 0.1565 -
0.1139 -0.0371 0.0816 -0.0173 -0.2549
-0.2505 -0.1876 -38.5125
93.7600 -39.4300 -0.1332 -0.0021 -
0.1462 -0.0529 0.0712 -0.0138 -0.2515
-0.2480 -0.1886 -38.5118
93.7800 -39.6100 -0.0104 -0.1408 -
0.1708 -0.0628 0.0602 -0.0105 -0.2480
-0.2456 -0.1896 -38.5110
93.8000 -39.6100 -0.0297 -0.1244 -
0.1807 -0.0656 0.0487 -0.0072 -0.2446
-0.2431 -0.1905 -38.5102
93.8200 -39.3100 -0.0209 0.0083 -
0.1694 -0.0612 0.0368 -0.0040 -0.2411
-0.2406 -0.1915 -38.5094
93.8400 -39.1900 0.0800 0.1122 -
0.1343 -0.0500 0.0246 -0.0010 -0.2376
-0.2381 -0.1924 -38.5086
93.8600 -39.1900 -0.0123 0.0854 -
0.0784 -0.0334 0.0123 0.0020 -0.2342
-0.2357 -0.1934 -38.5079

93.8800 -39.2900 0.0574 -0.0590 -
0.0102 -0.0130 -0.0001 0.0048 -0.2307
-0.2332 -0.1943 -38.5071
93.9000 -39.3100 0.0017 -0.1851
0.0588 0.0091 -0.0123 0.0074 -0.2273
-0.2307 -0.1953 -38.5063
93.9200 -39.3100 -0.2195 -0.1347
0.1184 0.0310 -0.0243 0.0100 -0.2238
-0.2282 -0.1962 -38.5055
93.9400 -38.6600 0.2119 0.0581
0.1612 0.0505 -0.0358 0.0124 -0.2203
-0.2257 -0.1971 -38.5047
93.9600 -38.6600 0.0930 0.1884
0.1791 0.0658 -0.0467 0.0147 -0.2169
-0.2231 -0.1980 -38.5040
93.9800 -38.6600 0.0813 0.1512
0.1664 0.0755 -0.0570 0.0169 -0.2135
-0.2206 -0.1989 -38.5032
94.0000 -39.1200 -0.1923 0.0169
0.1234 0.0791 -0.0665 0.0189 -0.2101
-0.2181 -0.1998 -38.5024
94.0200 -39.1200 -0.0076 -0.0632
0.0591 0.0762 -0.0751 0.0208 -0.2067
-0.2156 -0.2007 -38.5016
94.0400 -39.1200 -0.0272 -0.0496 -
0.0110 0.0671 -0.0827 0.0225 -0.2033
-0.2130 -0.2016 -38.5009
94.0600 -39.2600 0.0154 -0.0283 -
0.0711 0.0525 -0.0891 0.0241 -0.1999
-0.2105 -0.2025 -38.5001
94.0800 -39.2600 0.0338 -0.0438 -
0.1101 0.0332 -0.0942 0.0256 -0.1966
-0.2079 -0.2034 -38.4993
94.1000 -39.2600 -0.0241 -0.0414 -
0.1222 0.0107 -0.0980 0.0269 -0.1933
-0.2054 -0.2042 -38.4985
94.1200 -39.2500 -0.0105 0.0217 -
0.1084 -0.0141 -0.1004 0.0280 -0.1900
-0.2028 -0.2051 -38.4978
94.1400 -39.2500 0.0533 0.0639 -
0.0718 -0.0396 -0.1012 0.0290 -0.1868
-0.2002 -0.2060 -38.4970
94.1600 -39.2600 -0.0329 0.0142 -
0.0191 -0.0645 -0.1007 0.0299 -0.1836
-0.1976 -0.2068 -38.4962
94.1800 -39.3100 -0.0963 -0.0673
0.0366 -0.0872 -0.0986 0.0307 -0.1804
-0.1950 -0.2077 -38.4954
94.2000 -39.3100 0.0073 -0.0851
0.0811 -0.1065 -0.0951 0.0313 -0.1772
-0.1924 -0.2085 -38.4947

94.2200 -39.2100 0.0458 -0.0263
0.1031 -0.1210 -0.0902 0.0318 -0.1742
-0.1898 -0.2093 -38.4939
94.2400 -38.9700 -0.0295 0.0720
0.0952 -0.1302 -0.0840 0.0322 -0.1711
-0.1872 -0.2102 -38.4931
94.2600 -38.9700 0.0530 0.1663
0.0550 -0.1337 -0.0766 0.0324 -0.1681
-0.1846 -0.2110 -38.4923
94.2800 -39.0900 0.0017 0.1384 -
0.0117 -0.1314 -0.0681 0.0326 -0.1651
-0.1819 -0.2118 -38.4916
94.3000 -39.3800 -0.0791 0.0052 -
0.0889 -0.1238 -0.0587 0.0326 -0.1622
-0.1793 -0.2126 -38.4908
94.3200 -39.3800 -0.0300 -0.0777 -
0.1547 -0.1114 -0.0484 0.0326 -0.1593
-0.1766 -0.2134 -38.4900
94.3400 -39.3800 0.0872 -0.1159 -
0.1845 -0.0952 -0.0374 0.0324 -0.1565
-0.1739 -0.2142 -38.4892
94.3600 -39.3700 0.1081 -0.1682 -
0.1606 -0.0763 -0.0259 0.0322 -0.1538
-0.1713 -0.2150 -38.4885
94.3800 -39.3700 -0.1020 -0.1644 -
0.0825 -0.0560 -0.0140 0.0319 -0.1511
-0.1686 -0.2158 -38.4877
94.4000 -39.2100 -0.2538 -0.0050
0.0281 -0.0356 -0.0018 0.0314 -0.1484
-0.1659 -0.2166 -38.4869
94.4200 -38.3900 0.2253 0.1990
0.1373 -0.0159 0.0104 0.0309 -0.1459
-0.1632 -0.2174 -38.4862
94.4400 -38.3900 0.0257 0.2455
0.2125 0.0021 0.0226 0.0304 -0.1433
-0.1604 -0.2182 -38.4854
94.4600 -38.4500 0.1290 0.1239
0.2321 0.0180 0.0345 0.0297 -0.1409
-0.1577 -0.2189 -38.4846
94.4800 -38.8100 -0.1689 -0.0248
0.1947 0.0316 0.0458 0.0290 -0.1385
-0.1550 -0.2197 -38.4838
94.5000 -38.8100 0.0154 -0.0880
0.1150 0.0438 0.0564 0.0282 -0.1361
-0.1522 -0.2205 -38.4831
94.5200 -38.8100 0.0663 -0.0855
0.0176 0.0556 0.0661 0.0274 -0.1339
-0.1495 -0.2212 -38.4823
94.5400 -38.8800 -0.0092 -0.0477 -
0.0746 0.0682 0.0746 0.0265 -0.1317
-0.1467 -0.2220 -38.4815

94.5600 -38.8800 -0.0273 -0.0039 -
0.1450 0.0825 0.0818 0.0256 -0.1296
-0.1439 -0.2227 -38.4808
94.5800 -38.8800 -0.0305 0.0400 -
0.1853 0.0993 0.0876 0.0247 -0.1275
-0.1411 -0.2234 -38.4800
94.6000 -38.8700 -0.0472 0.0741 -
0.1953 0.1188 0.0918 0.0237 -0.1256
-0.1383 -0.2242 -38.4792
94.6200 -38.8200 0.0308 0.0466 -
0.1780 0.1407 0.0943 0.0227 -0.1237
-0.1355 -0.2249 -38.4785
94.6400 -38.8200 0.0196 -0.0485 -
0.1396 0.1640 0.0951 0.0218 -0.1218
-0.1326 -0.2256 -38.4777
94.6600 -38.8200 0.0217 -0.1308 -
0.0824 0.1873 0.0941 0.0208 -0.1201
-0.1298 -0.2264 -38.4769
94.6800 -38.8200 -0.1044 -0.0980 -
0.0108 0.2084 0.0914 0.0199 -0.1184
-0.1269 -0.2271 -38.4762
94.7000 -38.4000 0.0803 0.0377
0.0661 0.2249 0.0868 0.0189 -0.1168
-0.1241 -0.2278 -38.4754
94.7200 -38.4000 0.0169 0.1337
0.1350 0.2345 0.0805 0.0180 -0.1153
-0.1212 -0.2285 -38.4746
94.7400 -38.4000 -0.0440 0.1119
0.1809 0.2353 0.0726 0.0172 -0.1139
-0.1183 -0.2292 -38.4739
94.7600 -38.4000 0.0014 0.0213
0.1928 0.2261 0.0630 0.0164 -0.1126
-0.1154 -0.2299 -38.4731
94.7800 -38.6000 0.0079 -0.0526
0.1705 0.2064 0.0521 0.0157 -0.1113
-0.1125 -0.2306 -38.4723
94.8000 -38.7400 -0.0333 -0.0810
0.1251 0.1767 0.0400 0.0150 -0.1102
-0.1095 -0.2313 -38.4716
94.8200 -38.7400 -0.0300 -0.0425
0.0702 0.1382 0.0269 0.0145 -0.1091
-0.1066 -0.2320 -38.4708
94.8400 -38.7400 0.0412 -0.0021
0.0171 0.0925 0.0133 0.0140 -0.1081
-0.1036 -0.2326 -38.4700
94.8600 -38.8800 0.0129 -0.0031 -
0.0257 0.0419 -0.0007 0.0137 -0.1072
-0.1007 -0.2333 -38.4693
94.8800 -38.9800 0.0190 0.0077 -
0.0566 -0.0110 -0.0147 0.0135 -0.1064
-0.0977 -0.2340 -38.4685

94.9000 -38.9800 -0.1106 0.0495 -
0.0765 -0.0636 -0.0284 0.0133 -0.1057
-0.0947 -0.2347 -38.4677
94.9200 -38.9800 0.1373 0.0595 -
0.0858 -0.1132 -0.0416 0.0134 -0.1050
-0.0917 -0.2353 -38.4670
94.9400 -39.1100 -0.0079 0.0016 -
0.0883 -0.1575 -0.0539 0.0135 -0.1045
-0.0887 -0.2360 -38.4662
94.9600 -39.3000 -0.0840 -0.0696 -
0.0855 -0.1950 -0.0650 0.0139 -0.1040
-0.0856 -0.2366 -38.4654
94.9800 -39.3000 0.0390 -0.0740 -
0.0771 -0.2244 -0.0748 0.0143 -0.1037
-0.0826 -0.2373 -38.4647
95.0000 -39.3000 0.0097 -0.0146 -
0.0659 -0.2450 -0.0830 0.0149 -0.1034
-0.0795 -0.2379 -38.4639
95.0200 -39.2400 -0.0662 0.0514 -
0.0549 -0.2566 -0.0895 0.0157 -0.1032
-0.0765 -0.2386 -38.4632
95.0400 -39.1600 0.0445 0.0895 -
0.0472 -0.2591 -0.0940 0.0167 -0.1031
-0.0734 -0.2392 -38.4624
95.0600 -39.1600 0.0474 0.0887 -
0.0433 -0.2530 -0.0967 0.0178 -0.1031
-0.0703 -0.2398 -38.4616
95.0800 -39.1600 -0.0189 0.0367 -
0.0366 -0.2394 -0.0975 0.0191 -0.1031
-0.0672 -0.2405 -38.4609
95.1000 -39.2000 0.0000 -0.0666 -
0.0171 -0.2197 -0.0964 0.0205 -0.1033
-0.0640 -0.2411 -38.4601
95.1200 -39.2500 -0.0657 -0.1814
0.0197 -0.1956 -0.0936 0.0222 -0.1035
-0.0609 -0.2417 -38.4593
95.1400 -39.2500 0.0679 -0.2520
0.0740 -0.1683 -0.0892 0.0239 -0.1039
-0.0577 -0.2423 -38.4586
95.1600 -39.2500 -0.0441 -0.2105
0.1358 -0.1392 -0.0833 0.0258 -0.1043
-0.0546 -0.2430 -38.4578
95.1800 -38.8800 -0.0626 -0.0147
0.1826 -0.1090 -0.0761 0.0279 -0.1048
-0.0514 -0.2436 -38.4571
95.2000 -38.3300 0.1069 0.2752
0.1923 -0.0785 -0.0678 0.0300 -0.1054
-0.0482 -0.2442 -38.4563
95.2200 -38.3300 -0.0446 0.4735
0.1525 -0.0484 -0.0585 0.0323 -0.1060
-0.0450 -0.2448 -38.4555

95.2400 -38.3300 0.0821 0.3882
0.0666 -0.0191 -0.0485 0.0347 -0.1068
-0.0418 -0.2454 -38.4548
95.2600 -38.7600 0.0773 0.0282 -
0.0424 0.0094 -0.0379 0.0372 -0.1076
-0.0385 -0.2460 -38.4540
95.2800 -39.4000 -0.1362 -0.3647 -
0.1447 0.0371 -0.0270 0.0397 -0.1085
-0.0353 -0.2466 -38.4533
95.3000 -39.4000 0.0053 -0.5170 -
0.2108 0.0641 -0.0160 0.0424 -0.1095
-0.0320 -0.2472 -38.4525
95.3200 -39.4000 -0.0941 -0.3522 -
0.2219 0.0904 -0.0051 0.0450 -0.1105
-0.0288 -0.2477 -38.4518
95.3400 -38.9600 -0.0720 -0.0273 -
0.1821 0.1158 0.0056 0.0478 -0.1116
-0.0255 -0.2483 -38.4510
95.3600 -38.2900 0.1892 0.2392 -
0.1088 0.1398 0.0157 0.0505 -0.1128
-0.0222 -0.2489 -38.4502
95.3800 -38.2900 0.0551 0.3304 -
0.0243 0.1616 0.0252 0.0533 -0.1141
-0.0189 -0.2495 -38.4495
95.4000 -38.2900 -0.0726 0.2333
0.0494 0.1803 0.0339 0.0560 -0.1154
-0.0155 -0.2500 -38.4487
95.4200 -38.3800 -0.0146 0.0669
0.0972 0.1949 0.0417 0.0588 -0.1168
-0.0122 -0.2506 -38.4480
95.4400 -38.5200 -0.0297 -0.0539
0.1131 0.2045 0.0483 0.0615 -0.1182
-0.0089 -0.2512 -38.4472
95.4600 -38.5200 0.0065 -0.0902
0.1022 0.2078 0.0538 0.0642 -0.1198
-0.0055 -0.2517 -38.4464
95.4800 -38.5200 0.0124 -0.0654
0.0735 0.2041 0.0581 0.0668 -0.1213
-0.0021 -0.2523 -38.4457
95.5000 -38.5200 0.0927 -0.0214
0.0403 0.1927 0.0613 0.0693 -0.1230
0.0012 -0.2529 -38.4449
95.5200 -38.5200 -0.0111 0.0168
0.0139 0.1736 0.0635 0.0717 -0.1246
0.0046 -0.2534 -38.4442
95.5400 -38.5200 -0.0641 0.0349 -
0.0010 0.1474 0.0646 0.0740 -0.1264
0.0080 -0.2540 -38.4434
95.5600 -38.5200 0.0794 0.0254 -
0.0024 0.1149 0.0648 0.0762 -0.1281
0.0114 -0.2545 -38.4427

95.5800 -38.6000 -0.0657 -0.0025
0.0074 0.0773 0.0643 0.0782 -0.1300
0.0149 -0.2551 -38.4419
95.6000 -38.7300 -0.0873 -0.0224
0.0200 0.0362 0.0631 0.0800 -0.1318
0.0183 -0.2556 -38.4412
95.6200 -38.7300 0.0821 -0.0275
0.0283 -0.0066 0.0614 0.0817 -0.1337
0.0217 -0.2561 -38.4404
95.6400 -38.7300 0.0044 -0.0273
0.0301 -0.0487 0.0592 0.0831 -0.1357
0.0252 -0.2567 -38.4396
95.6600 -38.7300 -0.0500 -0.0207
0.0241 -0.0879 0.0566 0.0844 -0.1376
0.0286 -0.2572 -38.4389
95.6800 -38.7300 0.0001 0.0073
0.0078 -0.1221 0.0537 0.0853 -0.1396
0.0321 -0.2577 -38.4381
95.7000 -38.7300 0.0456 0.0612 -
0.0201 -0.1491 0.0507 0.0861 -0.1417
0.0356 -0.2582 -38.4374
95.7200 -38.7300 0.0508 0.0843 -
0.0547 -0.1676 0.0475 0.0865 -0.1437
0.0391 -0.2588 -38.4366
95.7400 -38.9500 0.0185 0.0093 -
0.0858 -0.1765 0.0443 0.0867 -0.1458
0.0426 -0.2593 -38.4359
95.7600 -39.0900 0.0203 -0.1323 -
0.0981 -0.1753 0.0411 0.0866 -0.1479
0.0461 -0.2598 -38.4351
95.7800 -39.0900 -0.0100 -0.2048 -
0.0815 -0.1640 0.0380 0.0862 -0.1501
0.0496 -0.2603 -38.4344
95.8000 -39.0900 -0.0824 -0.1049 -
0.0402 -0.1435 0.0350 0.0854 -0.1522
0.0531 -0.2608 -38.4336
95.8200 -38.6300 0.1572 0.0894
0.0117 -0.1154 0.0322 0.0844 -0.1543
0.0566 -0.2613 -38.4329
95.8400 -38.5200 -0.0196 0.2143
0.0538 -0.0813 0.0297 0.0831 -0.1565
0.0602 -0.2619 -38.4321
95.8600 -38.5200 0.0017 0.1857
0.0710 -0.0432 0.0273 0.0814 -0.1587
0.0637 -0.2624 -38.4314
95.8800 -38.5200 -0.0614 0.0689
0.0601 -0.0031 0.0252 0.0794 -0.1608
0.0672 -0.2629 -38.4306
95.9000 -38.7000 0.0698 -0.0548
0.0294 0.0368 0.0234 0.0771 -0.1630
0.0708 -0.2634 -38.4299

95.9200	-38.7500	0.0038	-0.1522	-
0.0063	0.0746	0.0218	0.0745	-0.1652
	0.0744	-0.2639	-38.4291	
95.9400	-38.7500	-0.0224	-0.1923	-
0.0282	0.1082	0.0204	0.0716	-0.1673
	0.0779	-0.2643	-38.4284	
95.9600	-38.7500	-0.0883	-0.1137	-
0.0274	0.1355	0.0193	0.0683	-0.1695
	0.0815	-0.2648	-38.4276	
95.9800	-38.4700	0.1645	0.0228	-
0.0063	0.1549	0.0184	0.0648	-0.1716
	0.0851	-0.2653	-38.4269	
96.0000	-38.4700	-0.0493	0.0848	
0.0274	0.1650	0.0177	0.0610	-0.1737
	0.0886	-0.2658	-38.4261	
96.0200	-38.4700	-0.0645	0.0493	
0.0623	0.1650	0.0172	0.0569	-0.1759
	0.0922	-0.2663	-38.4254	
96.0400	-38.4800	0.0515	-0.0064	
0.0862	0.1546	0.0168	0.0525	-0.1780
	0.0958	-0.2668	-38.4246	
96.0600	-38.5300	0.0053	-0.0246	
0.0919	0.1340	0.0166	0.0479	-0.1800
	0.0994	-0.2673	-38.4239	
96.0800	-38.5300	-0.0994	0.0238	
0.0763	0.1046	0.0164	0.0430	-0.1821
	0.1030	-0.2677	-38.4231	
96.1000	-38.5300	0.0052	0.0890	
0.0419	0.0682	0.0164	0.0378	-0.1841
	0.1066	-0.2682	-38.4224	
96.1200	-38.6700	0.0482	0.0531	-
0.0055	0.0275	0.0165	0.0325	-0.1861
	0.1102	-0.2687	-38.4216	
96.1400	-38.8900	-0.0646	-0.0567	-
0.0532	-0.0148	0.0168	0.0269	-0.1881
	0.1138	-0.2692	-38.4209	
96.1600	-38.8900	-0.0185	-0.0960	-
0.0864	-0.0558	0.0172	0.0212	-0.1900
	0.1174	-0.2696	-38.4201	
96.1800	-38.8900	-0.0070	-0.0368	-
0.0965	-0.0930	0.0178	0.0152	-0.1919
	0.1210	-0.2701	-38.4194	
96.2000	-38.9100	0.0268	0.0252	-
0.0840	-0.1241	0.0186	0.0091	-0.1937
	0.1246	-0.2706	-38.4186	
96.2200	-38.9100	-0.0307	0.0399	-
0.0546	-0.1473	0.0197	0.0028	-0.1955
	0.1282	-0.2710	-38.4179	
96.2400	-38.9100	0.0095	0.0075	-
0.0191	-0.1608	0.0210	-0.0036	-0.1973
	0.1318	-0.2715	-38.4171	

96.2600 -38.9000 0.0154 -0.0158
0.0085 -0.1638 0.0226 -0.0102 -0.1990
0.1354 -0.2719 -38.4164
96.2800 -38.8700 -0.0170 0.0263
0.0174 -0.1562 0.0245 -0.0168 -0.2007
0.1391 -0.2724 -38.4156
96.3000 -38.8700 0.0085 0.0611
0.0012 -0.1386 0.0264 -0.0236 -0.2023
0.1427 -0.2728 -38.4149
96.3200 -38.8700 -0.0191 0.0246 -
0.0378 -0.1121 0.0285 -0.0305 -0.2038
0.1463 -0.2733 -38.4141
96.3400 -38.8900 -0.0234 -0.0239 -
0.0895 -0.0781 0.0305 -0.0373 -0.2053
0.1499 -0.2737 -38.4134
96.3600 -38.9100 0.0840 -0.0356 -
0.1368 -0.0389 0.0323 -0.0443 -0.2067
0.1535 -0.2742 -38.4126
96.3800 -38.9100 -0.0295 -0.0305 -
0.1604 0.0031 0.0337 -0.0512 -0.2081
0.1571 -0.2746 -38.4119
96.4000 -38.8700 -0.1336 -0.0357 -
0.1474 0.0451 0.0347 -0.0581 -0.2094
0.1608 -0.2751 -38.4111
96.4200 -38.6900 0.1029 -0.0325 -
0.0949 0.0844 0.0351 -0.0650 -0.2106
0.1644 -0.2755 -38.4104
96.4400 -38.6900 0.0379 -0.0406 -
0.0075 0.1187 0.0347 -0.0718 -0.2118
0.1680 -0.2760 -38.4097
96.4600 -38.6900 -0.0871 -0.0374
0.0990 0.1458 0.0335 -0.0785 -0.2129
0.1716 -0.2764 -38.4089
96.4800 -38.3800 0.0289 0.0321
0.2014 0.1638 0.0314 -0.0852 -0.2139
0.1752 -0.2768 -38.4082
96.5000 -38.1700 -0.0004 0.1110
0.2771 0.1710 0.0284 -0.0916 -0.2148
0.1788 -0.2773 -38.4074
96.5200 -38.1700 -0.0160 0.1051
0.3114 0.1665 0.0245 -0.0980 -0.2157
0.1825 -0.2777 -38.4067
96.5400 -38.1700 0.1500 0.0361
0.2962 0.1501 0.0197 -0.1041 -0.2165
0.1861 -0.2781 -38.4059
96.5600 -38.5700 -0.0563 -0.0192
0.2311 0.1234 0.0141 -0.1101 -0.2172
0.1897 -0.2785 -38.4052
96.5800 -38.6700 -0.0931 0.0246
0.1213 0.0888 0.0077 -0.1158 -0.2178
0.1933 -0.2790 -38.4044

96.6000 -38.6700 0.0075 0.1125 -
0.0159 0.0497 0.0008 -0.1213 -0.2183
0.1969 -0.2794 -38.4037
96.6200 -38.6700 0.1648 0.0809 -
0.1553 0.0098 -0.0066 -0.1265 -0.2187
0.2005 -0.2798 -38.4030
96.6400 -39.3400 -0.1409 -0.0941 -
0.2701 -0.0274 -0.0143 -0.1315 -0.2190
0.2041 -0.2802 -38.4022
96.6600 -39.5100 -0.0654 -0.2521 -
0.3374 -0.0584 -0.0221 -0.1361 -0.2193
0.2077 -0.2807 -38.4015
96.6800 -39.5100 0.1062 -0.2488 -
0.3412 -0.0806 -0.0300 -0.1405 -0.2194
0.2113 -0.2811 -38.4007
96.7000 -39.5100 -0.1597 -0.0904 -
0.2809 -0.0929 -0.0377 -0.1445 -0.2195
0.2149 -0.2815 -38.4000
96.7200 -38.9200 0.1188 0.1070 -
0.1753 -0.0956 -0.0450 -0.1482 -0.2195
0.2185 -0.2819 -38.3992
96.7400 -38.7700 0.0502 0.2026 -
0.0526 -0.0904 -0.0518 -0.1515 -0.2193
0.2221 -0.2823 -38.3985
96.7600 -38.7700 -0.0432 0.1760
0.0586 -0.0790 -0.0580 -0.1544 -0.2191
0.2257 -0.2827 -38.3978
96.7800 -38.7700 -0.0052 0.0803
0.1390 -0.0636 -0.0634 -0.1569 -0.2188
0.2292 -0.2831 -38.3970
96.8000 -38.7700 -0.0062 -0.0184
0.1813 -0.0465 -0.0680 -0.1591 -0.2183
0.2328 -0.2836 -38.3963
96.8200 -38.7700 0.0345 -0.0598
0.1906 -0.0300 -0.0716 -0.1609 -0.2178
0.2364 -0.2840 -38.3955
96.8400 -38.7700 0.0041 -0.0275
0.1760 -0.0160 -0.0742 -0.1622 -0.2172
0.2400 -0.2844 -38.3948
96.8600 -38.7700 0.0438 0.0184
0.1473 -0.0061 -0.0756 -0.1632 -0.2164
0.2435 -0.2848 -38.3940
96.8800 -38.8400 -0.0287 0.0139
0.1146 -0.0014 -0.0759 -0.1637 -0.2156
0.2471 -0.2852 -38.3933
96.9000 -38.8800 -0.0219 -0.0203
0.0856 -0.0025 -0.0750 -0.1639 -0.2146
0.2507 -0.2856 -38.3926
96.9200 -38.8800 0.0045 -0.0326
0.0635 -0.0093 -0.0730 -0.1636 -0.2136
0.2542 -0.2860 -38.3918

96.9400 −38.8800 −0.0195 −0.0329
0.0459 −0.0209 −0.0697 −0.1630 −0.2125
0.2578 −0.2864 −38.3911
96.9600 −38.8600 0.0653 −0.0242
0.0287 −0.0353 −0.0654 −0.1620 −0.2113
0.2613 −0.2868 −38.3903
96.9800 −38.8400 −0.0372 0.0123
0.0078 −0.0503 −0.0601 −0.1606 −0.2099
0.2649 −0.2872 −38.3896
97.0000 −38.8400 0.0323 0.0430 −
0.0194 −0.0637 −0.0539 −0.1588 −0.2085
0.2684 −0.2876 −38.3889
97.0200 −38.8400 −0.0226 0.0472 −
0.0507 −0.0737 −0.0469 −0.1567 −0.2070
0.2719 −0.2880 −38.3881
97.0400 −38.9000 −0.0598 0.0514 −
0.0844 −0.0789 −0.0393 −0.1543 −0.2054
0.2754 −0.2884 −38.3874
97.0600 −38.9800 0.0049 0.0341 −
0.1180 −0.0780 −0.0313 −0.1515 −0.2037
0.2790 −0.2887 −38.3866
97.0800 −38.9800 0.0287 −0.0088 −
0.1491 −0.0703 −0.0229 −0.1485 −0.2020
0.2825 −0.2891 −38.3859
97.1000 −38.9800 −0.0142 −0.0028 −
0.1733 −0.0554 −0.0143 −0.1451 −0.2001
0.2860 −0.2895 −38.3852
97.1200 −38.9600 0.0121 0.0071 −
0.1802 −0.0335 −0.0058 −0.1415 −0.1981
0.2895 −0.2899 −38.3844
97.1400 −38.9000 0.0156 −0.0566 −
0.1568 −0.0060 0.0026 −0.1376 −0.1961
0.2929 −0.2903 −38.3837
97.1600 −38.9000 0.0073 −0.1634 −
0.0960 0.0254 0.0106 −0.1335 −0.1940
0.2964 −0.2907 −38.3829
97.1800 −38.9000 −0.0798 −0.1680 −
0.0036 0.0582 0.0183 −0.1292 −0.1918
0.2999 −0.2911 −38.3822
97.2000 −38.5800 −0.1097 −0.0171
0.1030 0.0896 0.0254 −0.1246 −0.1895
0.3033 −0.2915 −38.3815
97.2200 −38.1100 0.1591 0.1694
0.1986 0.1169 0.0319 −0.1199 −0.1872
0.3068 −0.2918 −38.3807
97.2400 −38.1100 −0.0009 0.2220
0.2561 0.1375 0.0378 −0.1150 −0.1848
0.3102 −0.2922 −38.3800
97.2600 −38.1100 0.0476 0.1182
0.2561 0.1492 0.0429 −0.1100 −0.1823
0.3137 −0.2926 −38.3792

97.2800	-38.3400	-0.0778	-0.0363	
0.2021	0.1513	0.0473	-0.1049	-0.1797
	0.3171	-0.2930	-38.3785	
97.3000	-38.4500	-0.0055	-0.1201	
0.1141	0.1440	0.0509	-0.0996	-0.1771
	0.3205	-0.2934	-38.3778	
97.3200	-38.4500	-0.0207	-0.0996	
0.0173	0.1285	0.0537	-0.0943	-0.1744
	0.3239	-0.2937	-38.3770	
97.3400	-38.4500	0.0809	-0.0369	-
0.0677	0.1060	0.0558	-0.0889	-0.1716
	0.3273	-0.2941	-38.3763	
97.3600	-38.5800	-0.0566	-0.0116	-
0.1239	0.0779	0.0572	-0.0835	-0.1688
	0.3307	-0.2945	-38.3756	
97.3800	-38.5800	0.0527	-0.0096	-
0.1408	0.0457	0.0580	-0.0780	-0.1659
	0.3340	-0.2949	-38.3748	
97.4000	-38.5800	-0.0751	0.0115	-
0.1181	0.0111	0.0581	-0.0726	-0.1630
	0.3374	-0.2952	-38.3741	
97.4200	-38.5800	-0.0544	0.0615	-
0.0699	-0.0238	0.0578	-0.0671	-0.1600
	0.3408	-0.2956	-38.3733	
97.4400	-38.5100	0.0227	0.0849	-
0.0169	-0.0569	0.0569	-0.0616	-0.1570
	0.3441	-0.2960	-38.3726	
97.4600	-38.4900	0.0126	0.0438	
0.0222	-0.0862	0.0557	-0.0562	-0.1539
	0.3474	-0.2964	-38.3719	
97.4800	-38.4900	-0.0127	0.0136	
0.0355	-0.1099	0.0541	-0.0507	-0.1507
	0.3507	-0.2967	-38.3711	
97.5000	-38.4900	0.0792	0.0329	
0.0201	-0.1271	0.0522	-0.0454	-0.1476
	0.3540	-0.2971	-38.3704	
97.5200	-38.4900	0.0344	0.0313	-
0.0152	-0.1371	0.0501	-0.0401	-0.1443
	0.3573	-0.2975	-38.3697	
97.5400	-38.7000	-0.0932	-0.0049	-
0.0541	-0.1396	0.0480	-0.0348	-0.1411
	0.3606	-0.2979	-38.3689	
97.5600	-38.7000	-0.0631	-0.0462	-
0.0820	-0.1347	0.0458	-0.0297	-0.1377
	0.3638	-0.2982	-38.3682	
97.5800	-38.7000	0.0447	-0.0787	-
0.0900	-0.1224	0.0437	-0.0246	-0.1344
	0.3671	-0.2986	-38.3674	
97.6000	-38.7000	-0.0607	-0.0918	-
0.0775	-0.1033	0.0415	-0.0197	-0.1310
	0.3703	-0.2990	-38.3667	

97.6200 -38.6000 0.0093 -0.0462 -
0.0490 -0.0787 0.0394 -0.0148 -0.1276
0.3735 -0.2993 -38.3660
97.6400 -38.3100 0.0024 0.0359 -
0.0126 -0.0498 0.0373 -0.0101 -0.1242
0.3767 -0.2997 -38.3652
97.6600 -38.3100 0.0402 0.0906
0.0197 -0.0185 0.0352 -0.0054 -0.1207
0.3799 -0.3001 -38.3645
97.6800 -38.3100 -0.0613 0.0772
0.0415 0.0135 0.0330 -0.0009 -0.1172
0.3831 -0.3004 -38.3638
97.7000 -38.3100 -0.0614 0.0184
0.0532 0.0442 0.0307 0.0035 -0.1137
0.3863 -0.3008 -38.3630
97.7200 -38.2700 -0.0457 -0.0307
0.0564 0.0718 0.0283 0.0077 -0.1101
0.3894 -0.3012 -38.3623
97.7400 -38.1600 0.0572 -0.0388
0.0539 0.0946 0.0256 0.0119 -0.1066
0.3925 -0.3015 -38.3616
97.7600 -38.1600 -0.0550 -0.0095
0.0497 0.1111 0.0227 0.0159 -0.1030
0.3957 -0.3019 -38.3608
97.7800 -38.1600 0.0463 0.0258
0.0464 0.1205 0.0195 0.0198 -0.0994
0.3988 -0.3023 -38.3601
97.8000 -38.1600 0.0253 0.0287
0.0463 0.1221 0.0160 0.0236 -0.0959
0.4018 -0.3026 -38.3593
97.8200 -38.1800 -0.0178 0.0008
0.0487 0.1158 0.0122 0.0273 -0.0923
0.4049 -0.3030 -38.3586
97.8400 -38.2400 0.0044 -0.0260
0.0510 0.1020 0.0082 0.0308 -0.0887
0.4080 -0.3033 -38.3579
97.8600 -38.2400 -0.0478 -0.0275
0.0486 0.0821 0.0038 0.0343 -0.0851
0.4110 -0.3037 -38.3571
97.8800 -38.2400 -0.0046 -0.0125
0.0362 0.0578 -0.0007 0.0376 -0.0815
0.4140 -0.3041 -38.3564
97.9000 -38.2400 0.0475 0.0242
0.0147 0.0311 -0.0053 0.0408 -0.0779
0.4170 -0.3044 -38.3557
97.9200 -38.2600 -0.0167 0.0507 -
0.0101 0.0041 -0.0100 0.0440 -0.0743
0.4200 -0.3048 -38.3549
97.9400 -38.3400 -0.0273 0.0196 -
0.0334 -0.0211 -0.0146 0.0470 -0.0708
0.4230 -0.3052 -38.3542

97.9600 -38.3400 0.0233 -0.0640 -
0.0519 -0.0423 -0.0192 0.0499 -0.0672
0.4259 -0.3055 -38.3535
97.9800 -38.3400 0.0031 -0.0928 -
0.0638 -0.0583 -0.0235 0.0527 -0.0637
0.4288 -0.3059 -38.3527
98.0000 -38.3400 0.0664 -0.0120 -
0.0670 -0.0684 -0.0276 0.0554 -0.0602
0.4317 -0.3062 -38.3520
98.0200 -38.3600 -0.0872 0.0697 -
0.0640 -0.0725 -0.0313 0.0580 -0.0567
0.4346 -0.3066 -38.3513
98.0400 -38.3600 0.0598 0.0709 -
0.0593 -0.0711 -0.0344 0.0605 -0.0532
0.4375 -0.3070 -38.3505
98.0600 -38.3600 -0.0345 0.0313 -
0.0516 -0.0650 -0.0371 0.0629 -0.0498
0.4404 -0.3073 -38.3498
98.0800 -38.3300 0.0216 -0.0237 -
0.0375 -0.0556 -0.0391 0.0652 -0.0464
0.4432 -0.3077 -38.3491
98.1000 -38.3200 0.0320 -0.0873 -
0.0132 -0.0443 -0.0404 0.0674 -0.0430
0.4460 -0.3080 -38.3483
98.1200 -38.3200 -0.0395 -0.1054
0.0216 -0.0329 -0.0411 0.0696 -0.0397
0.4488 -0.3084 -38.3476
98.1400 -38.3200 -0.1604 -0.0475
0.0635 -0.0230 -0.0412 0.0716 -0.0364
0.4516 -0.3088 -38.3469
98.1600 -37.9500 0.1109 0.0528
0.1009 -0.0156 -0.0406 0.0736 -0.0331
0.4543 -0.3091 -38.3461
98.1800 -37.9500 -0.0209 0.1154
0.1204 -0.0113 -0.0393 0.0756 -0.0299
0.4571 -0.3095 -38.3454
98.2000 -37.9500 -0.0735 0.0874
0.1141 -0.0100 -0.0375 0.0774 -0.0267
0.4598 -0.3098 -38.3447
98.2200 -38.0600 0.0718 -0.0018
0.0833 -0.0111 -0.0350 0.0792 -0.0236
0.4625 -0.3102 -38.3439
98.2400 -38.2300 -0.0565 -0.0857
0.0362 -0.0137 -0.0320 0.0809 -0.0206
0.4651 -0.3105 -38.3432
98.2600 -38.2300 -0.0191 -0.0917 -
0.0159 -0.0165 -0.0285 0.0825 -0.0176
0.4678 -0.3109 -38.3425
98.2800 -38.2300 -0.0056 -0.0262 -
0.0615 -0.0185 -0.0247 0.0841 -0.0146
0.4704 -0.3113 -38.3417

98.3000	-38.2500	0.0180	0.0305	-
0.0945	-0.0184	-0.0205	0.0856	-0.0117
	0.4730	-0.3116	-38.3410	
98.3200	-38.2500	0.0596	0.0391	-
0.1125	-0.0153	-0.0161	0.0870	-0.0089
	0.4756	-0.3120	-38.3402	
98.3400	-38.2500	0.0123	0.0160	-
0.1152	-0.0083	-0.0116	0.0884	-0.0062
	0.4782	-0.3123	-38.3395	
98.3600	-38.2500	-0.1134	-0.0061	-
0.1022	0.0026	-0.0070	0.0897	-0.0034
	0.4807	-0.3127	-38.3388	
98.3800	-38.1400	0.1218	-0.0052	-
0.0714	0.0170	-0.0024	0.0909	-0.0008
	0.4833	-0.3131	-38.3380	
98.4000	-38.1400	0.0612	-0.0279	-
0.0218	0.0340	0.0022	0.0921	0.0018
	0.4858	-0.3134	-38.3373	
98.4200	-38.1400	-0.1715	-0.0553	
0.0396	0.0522	0.0065	0.0932	0.0043
	0.4882	-0.3138	-38.3366	
98.4400	-37.9200	-0.0057	-0.0331	
0.0972	0.0699	0.0106	0.0942	0.0067
	0.4907	-0.3141	-38.3358	
98.4600	-37.7100	0.1159	0.0040	
0.1371	0.0853	0.0143	0.0952	0.0090
	0.4931	-0.3145	-38.3351	
98.4800	-37.7100	0.0242	0.0200	
0.1490	0.0965	0.0176	0.0961	0.0113
	0.4955	-0.3149	-38.3344	
98.5000	-37.7100	-0.0172	0.0239	
0.1293	0.1022	0.0203	0.0969	0.0135
	0.4979	-0.3152	-38.3336	
98.5200	-37.8500	-0.0470	0.0323	
0.0824	0.1019	0.0225	0.0977	0.0156
	0.5003	-0.3156	-38.3329	
98.5400	-37.8500	0.0892	0.0408	
0.0191	0.0954	0.0242	0.0984	0.0177
	0.5026	-0.3159	-38.3322	
98.5600	-37.8500	0.0212	0.0224	-
0.0420	0.0832	0.0253	0.0990	0.0197
	0.5049	-0.3163	-38.3314	
98.5800	-38.0000	-0.0630	-0.0337	-
0.0819	0.0662	0.0258	0.0996	0.0216
	0.5072	-0.3167	-38.3307	
98.6000	-38.1500	0.0486	-0.0965	-
0.0905	0.0454	0.0259	0.1001	0.0234
	0.5095	-0.3170	-38.3300	
98.6200	-38.1500	0.0051	-0.1253	-
0.0674	0.0222	0.0255	0.1005	0.0251
	0.5117	-0.3174	-38.3292	

98.6400 -38.1500 -0.1161 -0.0811 -
0.0238 -0.0023 0.0247 0.1008 0.0268
0.5139 -0.3178 -38.3285
98.6600 -37.8100 0.0710 0.0510
0.0228 -0.0264 0.0237 0.1011 0.0284
0.5161 -0.3181 -38.3278
98.6800 -37.8100 -0.0001 0.1881
0.0546 -0.0487 0.0225 0.1013 0.0299
0.5183 -0.3185 -38.3270
98.7000 -37.8100 0.0588 0.1869
0.0613 -0.0679 0.0214 0.1014 0.0313
0.5205 -0.3188 -38.3263
98.7200 -37.9800 0.0633 0.0087
0.0439 -0.0831 0.0205 0.1014 0.0326
0.5226 -0.3192 -38.3256
98.7400 -38.3200 -0.1292 -0.1862
0.0124 -0.0935 0.0200 0.1013 0.0339
0.5247 -0.3196 -38.3248
98.7600 -38.3200 0.0774 -0.2214 -
0.0231 -0.0983 0.0200 0.1012 0.0351
0.5268 -0.3199 -38.3241
98.7800 -38.3200 -0.0932 -0.0840 -
0.0578 -0.0971 0.0205 0.1009 0.0362
0.5288 -0.3203 -38.3234
98.8000 -37.9500 0.1306 0.1215 -
0.0879 -0.0895 0.0217 0.1005 0.0373
0.5309 -0.3207 -38.3226
98.8200 -37.8800 -0.0692 0.2556 -
0.1100 -0.0751 0.0234 0.1000 0.0383
0.5329 -0.3210 -38.3219
98.8400 -37.8800 0.0855 0.2045 -
0.1167 -0.0543 0.0257 0.0994 0.0392
0.5349 -0.3214 -38.3212
98.8600 -38.0700 0.0042 -0.0105 -
0.1025 -0.0283 0.0285 0.0987 0.0400
0.5369 -0.3218 -38.3204
98.8800 -38.2600 0.0468 -0.2544 -
0.0625 0.0011 0.0317 0.0979 0.0409
0.5388 -0.3221 -38.3197
98.9000 -38.2600 -0.0221 -0.3632
0.0057 0.0317 0.0352 0.0970 0.0416
0.5408 -0.3225 -38.3190
98.9200 -38.2600 -0.3624 -0.2027
0.0961 0.0602 0.0386 0.0960 0.0423
0.5427 -0.3229 -38.3182
98.9400 -37.1500 0.3613 0.1641
0.1845 0.0831 0.0420 0.0950 0.0430
0.5446 -0.3232 -38.3175
98.9600 -37.1500 0.0041 0.4188
0.2348 0.0974 0.0449 0.0938 0.0437
0.5465 -0.3236 -38.3168

98.9800	-37.1500	0.0976	0.3395	0.2167	0.1012	0.0474	0.0926	0.0443	0.5484	-0.3240	-38.3160
99.0000	-37.6900	-0.0504	0.0179	0.1353	0.0974	0.0494	0.0914	0.0449	0.5503	-0.3243	-38.3153

DATA:
Time-varying Shannon entropy (SE) and 95% significance level of DO modes extracted by EEMD
(shown in the 5-8 columns in the above).

Column 1: Time (kyr. BP)
Column 2: Shannon entropy of IMF3 (SE3)
Column 3: 95% significance level of SE of IMF3 (95% SL3)
Column 4: Shannon entropy of IMF4 (SE4)
Column 5: 95% significance level of SE of IMF4 (95% SL4)
Column 6: Shannon entropy of IMF5 (SE5)
Column 7: 95% significance level of SE of IMF5 (95% SL5)
Column 8: Shannon entropy of IMF6 (SE6)
Column 9: 95% significance level of SE of IMF6 (95% SL6)

Time	SE3	95% SL3	SE4	95% SL4	SE5	95% SL5	SE6	95% SL6
4.9900	1.5698	1.3959	1.4488	1.4703	1.2678	1.7015	1.6116	1.8941
5.5900	0.6996	1.3847	1.1890	1.4624	1.2312	1.6996	1.7144	1.8869
6.1900	0.7112	1.3860	1.1816	1.4640	1.2800	1.6917	1.5229	1.8907
6.7900	1.0359	1.3881	1.1830	1.4581	1.2942	1.6926	1.5996	1.8934
7.3900	1.0571	1.4064	1.1558	1.4651	1.3037	1.6883	1.6756	1.8942
7.9900	1.0695	1.4147	1.1620	1.4602	1.3077	1.6682	1.5573	1.8847
8.5900	0.6002	1.4095	0.8496	1.4618	1.3120	1.6639	1.6445	1.8911
9.1900	1.0363	1.4049	0.8608	1.4654	1.2697	1.6773	1.2780	1.8940

9.7900	1.0577	1.4028	1.1610	1.4676	1.2653	1.6651	1.3684	1.8753
10.3900	1.2750	1.3837	1.2762	1.4707	1.2691	1.6595	1.5656	1.8779
10.9900	1.2940	1.3921	1.1255	1.4720	1.2609	1.6751	1.6703	1.8791
11.5900	1.3545	1.3847	1.1196	1.4728	1.4987	1.6870	1.6727	1.8857
12.1900	1.5550	1.3882	1.3260	1.4773	1.4487	1.6795	1.6780	1.8913
12.7900	1.5551	1.3909	1.3501	1.4794	1.4961	1.6694	1.7650	1.8923
13.3900	1.5901	1.3967	1.3561	1.4809	1.5126	1.6763	1.8776	1.8896
13.9900	1.5933	1.3821	1.3692	1.4848	1.5382	1.6901	1.8632	1.8883
14.5900	1.4122	1.3796	1.3808	1.4810	1.4582	1.6904	1.7214	1.9012
15.1900	1.4025	1.3925	1.3666	1.4783	1.4280	1.6834	1.6854	1.9013
15.7900	1.3991	1.4072	1.3481	1.4803	1.4305	1.6836	1.5920	1.9082
16.3900	1.3747	1.4016	1.2915	1.4723	1.4353	1.6702	1.5198	1.8927
16.9900	1.3810	1.4021	1.1235	1.4642	1.4015	1.6804	1.4394	1.8861
17.5900	1.3661	1.3976	1.1474	1.4673	1.3946	1.6844	1.3514	1.8828
18.1900	1.7219	1.3752	1.0929	1.4766	1.5388	1.6770	1.6017	1.8937
18.7900	1.5796	1.3855	1.4664	1.4606	1.5928	1.6766	1.6680	1.8882
19.3900	1.5530	1.3875	1.4714	1.4721	1.5155	1.6782	1.6623	1.8913

19.9900 1.2789 1.3969 1.3412
1.4790 1.5270 1.6779 1.5383
1.8952
20.5900 1.1369 1.4044 1.2099
1.4799 1.4718 1.6632 1.5658
1.9012
21.1900 1.1071 1.3964 1.4565
1.4840 1.5139 1.6720 1.5399
1.8888
21.7900 1.1253 1.4021 1.4469
1.4825 1.5415 1.6662 1.5684
1.8839
22.3900 1.1511 1.4086 1.5550
1.4723 1.5515 1.6709 1.5934
1.8858
22.9900 1.1576 1.4105 1.5649
1.4746 1.6175 1.6660 1.6226
1.8769
23.5900 1.1740 1.4074 1.5424
1.4757 1.6157 1.6728 1.6307
1.8874
24.1900 1.1960 1.3981 1.5384
1.4714 1.6078 1.6731 1.6042
1.8801
24.7900 1.0637 1.3833 1.2115
1.4739 1.5271 1.6728 1.6423
1.8925
25.3900 1.0815 1.3908 1.2223
1.4874 1.2985 1.6898 1.6200
1.8987
25.9900 1.0799 1.3813 1.3771
1.4953 1.4184 1.6825 1.5987
1.8968
26.5900 1.0700 1.3886 1.3868
1.4728 1.4054 1.6822 1.6092
1.8927
27.1900 1.0757 1.4040 1.3791
1.4501 1.3044 1.6809 1.6110
1.9030
27.7900 1.0881 1.3822 1.3457
1.4539 1.2915 1.6704 1.6312
1.9013
28.3900 1.0853 1.3766 1.3428
1.4588 1.2965 1.6713 1.6158
1.8945
28.9900 1.0808 1.3593 1.3530
1.4691 1.5508 1.6741 1.5991
1.8915
29.5900 1.0981 1.3878 1.3879
1.4743 1.5613 1.6649 1.6877
1.8802

30.1900	1.0925	1.4037	1.4747
1.4808	1.4215	1.6572	1.6296
1.8919			
30.7900	1.2766	1.4109	1.3874
1.4859	1.4637	1.6739	1.8000
1.8802			
31.3900	1.2690	1.4063	1.4183
1.4913	1.5436	1.6770	1.7526
1.8809			
31.9900	1.4579	1.4102	1.2537
1.4823	1.5393	1.6834	1.7586
1.8843			
32.5900	1.4632	1.4047	1.2602
1.4824	1.6046	1.6672	1.8384
1.8874			
33.1900	1.5908	1.4128	1.2707
1.4930	1.8139	1.6701	1.6086
1.8850			
33.7900	1.5872	1.4020	1.2843
1.4683	1.8113	1.6733	1.7714
1.8828			
34.3900	1.5887	1.3958	1.2817
1.4637	1.7271	1.6743	1.7654
1.8860			
34.9900	1.5762	1.3900	1.2679
1.4972	1.8161	1.6836	1.7914
1.8874			
35.5900	1.5039	1.3852	1.2698
1.4758	1.7219	1.6710	1.8055
1.8784			
36.1900	1.4956	1.3740	1.2458
1.4903	1.7294	1.6622	1.8892
1.8793			
36.7900	1.4971	1.3615	1.3815
1.4893	1.6658	1.6597	1.9003
1.8882			
37.3900	1.5052	1.3703	1.3458
1.4784	1.5243	1.6756	1.8408
1.8909			
37.9900	1.2512	1.3825	1.4832
1.4837	1.6146	1.6653	1.8071
1.9001			
38.5900	1.3288	1.3874	1.6626
1.4830	1.7311	1.6736	1.7668
1.8979			
39.1900	1.3029	1.4162	1.5603
1.4690	1.4072	1.6727	1.7068
1.8896			
39.7900	1.2918	1.4054	1.5493
1.4668	1.4627	1.6690	1.6707
1.8882			

40.3900 1.4287 1.4057 1.5510
1.4757 1.6062 1.6641 1.4904
1.8963
40.9900 1.4479 1.3999 1.6446
1.4733 1.7427 1.6668 1.5092
1.8981
41.5900 1.4402 1.3917 1.6344
1.4668 1.7084 1.6605 1.6595
1.8961
42.1900 1.4412 1.3747 1.6263
1.4734 1.7058 1.6560 1.6919
1.9010
42.7900 1.4196 1.3739 1.6270
1.4737 1.6777 1.6681 1.7465
1.9022
43.3900 1.3320 1.3808 1.5057
1.4755 1.6815 1.6635 1.6607
1.9014
43.9900 1.3797 1.3965 1.1570
1.4805 1.6774 1.6711 1.7400
1.8929
44.5900 1.0411 1.4032 1.2845
1.4994 1.6435 1.6693 1.8156
1.8894
45.1900 1.0539 1.4051 1.2756
1.4866 1.5751 1.6767 1.8098
1.8768
45.7900 1.0488 1.3934 1.2686
1.4761 1.2686 1.6792 1.8287
1.8881
46.3900 1.3794 1.3915 1.2713
1.4910 1.5746 1.6662 1.8176
1.8805
46.9900 1.2807 1.3896 1.1282
1.4974 1.5965 1.6772 1.8270
1.8778
47.5900 1.0475 1.3998 1.0923
1.4878 1.4406 1.6801 1.7934
1.8832
48.1900 1.0429 1.4049 1.0770
1.4835 1.3991 1.6738 1.7102
1.8813
48.7900 1.0580 1.4040 1.0859
1.4886 1.3857 1.6809 1.6858
1.8919
49.3900 1.0691 1.4313 1.0815
1.4945 1.3332 1.6695 1.4772
1.8933
49.9900 1.2454 1.4338 0.9089
1.4698 1.1481 1.6859 1.4477
1.8970

50.5900 0.8475 1.4277 1.1172
1.4709 1.3694 1.6730 1.4181
1.8927
51.1900 1.0231 1.4234 1.1621
1.4748 1.3588 1.6716 1.6252
1.8963
51.7900 1.1788 1.3974 1.3986
1.4789 1.2691 1.6717 1.4907
1.9038
52.3900 1.3452 1.4085 0.9923
1.4613 1.2187 1.6582 1.6044
1.8989
52.9900 1.3672 1.4079 1.1268
1.4700 1.2367 1.6590 1.5957
1.8975
53.5900 1.3548 1.4002 1.1498
1.4738 1.5145 1.6696 1.6210
1.8948
54.1900 1.3495 1.3957 1.1661
1.4705 1.5709 1.6599 1.6382
1.8960
54.7900 1.3306 1.3948 1.1653
1.4742 1.6222 1.6652 1.6336
1.9044
55.3900 1.3865 1.3959 1.2138
1.4793 1.6551 1.6748 1.6285
1.8949
55.9900 0.9539 1.4025 1.2709
1.4616 1.5857 1.6803 1.5972
1.8883
56.5900 1.0018 1.4025 1.2960
1.4582 1.5967 1.6717 1.5433
1.9079
57.1900 0.9894 1.4057 1.2845
1.4385 1.6021 1.6832 1.4717
1.9011
57.7900 0.9542 1.3888 1.2604
1.4523 1.5847 1.6938 1.4570
1.8886
58.3900 0.9317 1.3899 1.2288
1.4656 1.5658 1.6921 1.6978
1.8995
58.9900 0.9041 1.4018 1.3620
1.4787 1.5337 1.6888 1.7109
1.8857
59.5900 0.9123 1.4145 1.3179
1.4710 1.5003 1.6829 1.6982
1.8921
60.1900 0.9111 1.4045 1.3045
1.4848 1.4877 1.6711 1.6899
1.8931

60.7900 0.9507 1.4080 1.3443
1.4861 1.4890 1.6760 1.6368
1.8997
61.3900 0.9753 1.3846 1.4202
1.4734 1.4971 1.6743 1.8241
1.8918
61.9900 0.8619 1.3924 1.0705
1.4791 1.5216 1.6760 1.9091
1.8891
62.5900 1.2558 1.3995 1.0079
1.4755 1.4971 1.6903 1.8931
1.8840
63.1900 1.3882 1.3986 1.0081
1.4624 1.4334 1.6807 1.8980
1.8840
63.7900 1.3907 1.4022 0.9901
1.4817 1.3941 1.6701 1.8766
1.8825
64.3900 1.3777 1.3990 1.0151
1.4628 1.4055 1.6774 1.8636
1.8805
64.9900 1.3685 1.4031 1.0188
1.4681 1.4069 1.6604 1.8116
1.8812
65.5900 1.3911 1.3911 1.0111
1.4821 1.3937 1.6692 1.8095
1.8783
66.1900 1.3983 1.4047 1.0265
1.4898 1.4126 1.6780 1.7797
1.8806
66.7900 0.8151 1.4061 1.0779
1.4925 1.4642 1.6860 1.6454
1.8911
67.3900 0.7775 1.3993 0.9183
1.4745 1.4373 1.6930 1.4757
1.8981
67.9900 0.7828 1.3995 0.9319
1.4826 1.4953 1.6825 1.3636
1.8891
68.5900 0.7434 1.4108 0.9319
1.4884 1.5089 1.6914 1.4290
1.8825
69.1900 0.7364 1.4032 1.1041
1.5005 1.3959 1.6672 1.4904
1.8757
69.7900 1.0946 1.4000 1.2776
1.4888 1.5620 1.6681 1.4264
1.8752
70.3900 1.1123 1.4074 1.3378
1.4754 1.6348 1.6764 1.5638
1.8887

70.9900 1.1155 1.4063 1.3531
1.4846 1.6550 1.6701 1.6554
1.8843
71.5900 1.0981 1.4201 1.3816
1.4684 1.6824 1.6748 1.7529
1.8734
72.1900 1.0923 1.4108 1.3702
1.4674 1.6985 1.6620 1.8935
1.8819
72.7900 0.9529 1.3996 1.3196
1.4848 1.5771 1.6732 1.8666
1.8828
73.3900 0.9329 1.4100 1.3097
1.4958 1.7105 1.6807 1.8794
1.8766
73.9900 1.0756 1.4027 1.3377
1.4959 1.8108 1.6788 1.9066
1.8867
74.5900 1.0758 1.4073 1.3723
1.4818 1.8048 1.6739 1.9024
1.8930
75.1900 1.0608 1.3912 1.2508
1.4869 1.6668 1.6710 1.9340
1.8859
75.7900 0.9053 1.3859 1.5400
1.4922 1.5323 1.6718 1.9220
1.8871
76.3900 0.8386 1.3925 1.4665
1.5040 1.5853 1.6734 1.9009
1.8874
76.9900 0.8174 1.3906 1.4489
1.5018 1.5811 1.6766 1.8069
1.8939
77.5900 0.7716 1.4005 1.4030
1.4972 1.5530 1.6831 1.7683
1.8989
78.1900 0.7773 1.4046 1.3766
1.4705 1.5064 1.6798 1.5097
1.8932
78.7900 0.7841 1.4035 1.3747
1.4916 1.4417 1.6739 1.4167
1.8936
79.3900 0.7702 1.3971 1.3005
1.4979 1.5032 1.6834 1.4909
1.8917
79.9900 1.0156 1.3838 1.3217
1.4725 1.4751 1.6728 1.3685
1.8928
80.5900 1.2001 1.3924 1.1135
1.4766 1.3153 1.6724 1.2144
1.8913

81.1900 1.2023 1.3975 1.1481
1.4760 1.4217 1.6639 1.5594
1.8896
81.7900 0.7055 1.4003 1.1418
1.4695 1.3540 1.6675 1.5971
1.8946
82.3900 0.6163 1.3973 1.0846
1.4619 1.2771 1.6847 1.5705
1.8821
82.9900 0.6222 1.3988 1.0862
1.4643 1.4579 1.6712 1.6250
1.8873
83.5900 0.6290 1.4036 1.0769
1.4608 1.4944 1.6717 1.7242
1.8928
84.1900 0.6562 1.3950 1.1144
1.4645 1.5470 1.6666 1.7583
1.8885
84.7900 0.7253 1.3951 1.2340
1.4660 1.5757 1.6882 1.8544
1.8937
85.3900 0.7970 1.3992 1.2644
1.4710 1.6176 1.6791 1.9480
1.8951
85.9900 0.8000 1.3960 1.2769
1.4839 1.7453 1.6670 1.8613
1.8889
86.5900 0.8014 1.3993 1.2449
1.4945 1.7695 1.6708 1.7332
1.8937
87.1900 0.8139 1.3970 1.2459
1.4921 1.7742 1.6778 1.8109
1.8851
87.7900 0.7818 1.3832 1.2118
1.5005 1.9094 1.6628 1.8111
1.8924
88.3900 0.9955 1.3725 1.2314
1.4903 1.7555 1.6713 1.7773
1.8848
88.9900 1.0119 1.3865 1.2237
1.4856 1.7627 1.6727 1.7728
1.8916
89.5900 1.0129 1.3833 1.2436
1.4913 1.7493 1.6754 1.6504
1.8821
90.1900 1.0001 1.3708 1.2386
1.4835 1.7693 1.6626 1.6066
1.8852
90.7900 0.9986 1.3818 1.6615
1.4801 1.7211 1.6740 1.6077
1.8919

91.3900 1.1427 1.3785 1.4403
1.4732 1.4372 1.6772 1.4783
1.8916
91.9900 1.1409 1.3834 1.6748
1.4774 1.3439 1.6848 1.4211
1.8885
92.5900 1.1165 1.3982 1.7646
1.4891 1.2331 1.6810 1.3191
1.8851
93.1900 1.1245 1.3938 1.6853
1.4725 1.3905 1.6909 1.8585
1.8875
93.7900 1.2088 1.4015 1.6764
1.4737 1.4612 1.6781 1.8718
1.8929
94.3900 1.1988 1.3948 1.6735
1.4784 1.4536 1.6793 1.7967
1.8937
94.9900 1.0471 1.3744 1.6780
1.4744 1.4203 1.6676 1.7105
1.8850
95.5900 1.0389 1.3978 1.4815
1.4711 1.4011 1.6662 1.7876
1.8859
