# Supplementary material for: Data concerning statistical relation between obliquity and Dansgaard–Oeschger events
Source: Data Brief. 2019 Mar 7;23:103727. doi: 10.1016/j.dib.2019.103727 (PMC6660458; doi:10.1016/j.dib.2019.103727)
Supplement: Multimedia component 3 [file mmc3.pdf]

Processed oxygen isotope (d18O) data from GRIP ice core on GICC05 modelext timescale
----

NAME OF DATA SET:
Processed oxygen isotope (d18O) data from GRIP ice core on GICC05 modelext timescale

LAST UPDATE: 21/12/2018

ORIGINAL REFERENCE:
Jia Deng, Zhaohua Wu, Min Zhanga, Norden. E Huang, Shizhu Wang,Fangli Qiao. 2018.
Data concerning statistical relation between obliquity and Dansgaard-Oeschger events.
Data in Brief, in press.

MAIN REFERENCES:
Datafile of the original data accompanies the following two papers:

Rasmussen, S. O et al., 2014. A stratigraphic framework for abrupt climatic changes during the Last
Glacial period based on three synchronized Greenland ice-core records: refining and extending the
INTIMATE event stratigraphy. Quaternary. Sci. Rev. 106: 14-28.

Seierstad, I. K et al., 2014. Consistently dated records from the Greenland GRIP, GISP2 and NGRIP
ice cores for the past 104 ka reveal regional millennial-scale ヲﾄ18O gradients with possible Heinrich
event imprint. Quaternary. Sci. Rev. 106: 29-46.

ABSTRACT:
Data presented are related to the research article entitled 。- Using Holo-Hilbert spectral analysis to
quantify the modulation of Dansgaard-Oeschger events by obliquity。7 [1]. The datasets in Deng et al (2018)
are analyzed on the foundation of ensemble empirical mode decomposition (EEMD) [2], and reveal more occurrences
of Dansgaard-Oeschger (DO) events in the decreasing phase of obliquity. Here, we report the number of significant
high Shannon entropy (SE) [3] of 95% significance level of DO events in the increasing and decreasing phases of
obliquity, respectively. First, the proxy time series are filtered by EEMD to obtain DO events. Then, the
time-varying SE of DO modes are calculated on the basis of principle of histogram. The 95% significance level
is evaluated through surrogate data [4]. Finally, a comparison between the numbers of SE values that are larger
than 95% significance level in the increasing and decreasing phases of obliquity, respectively, is reported.

GEOGRAPHIC REGION: Greenland

PERIOD OF RECORD: 99 kyr. BP - 2 kyr.BP

FUNDING SOURCES:
This work was jointly supported by the National Basic Research Program of China (Grant 2012CB957802);
the National Natural Science Foundation of China (NSFC) (Grant 41506067); the Basic Scientific Fund
for National Public Research Institutes of China (Grant 2015G04); the US National Science Foundation
(Grant AGS-1723300); the NSFC-Shandong Joint Fund for Marine Science Research Centers (Grant U1406404);
and the National Programme on Global Change and Air-Sea Interaction (Grant GASI-IPOVAI-05).

DATA:
Calibrated GRIP oxygen isotope (d180) data on GICC05modeext timescale (Rasmussen et al, 2014; Seierstad et al, 2014).
Processed data by ensemble empirical mode decomposition (EEMD).

Column 1: Time (kyr. BP)
Column 2: (Calibrated secondary) d180 data (years before b2k)
Column 3: 1st EEMD component of d180 records
Column 4: 2nd EEMD component of d180 records
Column 5: 3rd EEMD component of d180 records
Column 6: 4th EEMD component of d180 records
Column 7: 5th EEMD component of d180 records
Column 8: 6th EEMD component of d180 records
Column 9: 8th EEMD component of d180 records
Column 10: 9th EEMD component of d180 records
Column 11: 10th EEMD component of d180 records
Column 12: EEMD trend of d180 records

Time	d180 data	IMF1	IMF2	IMF3	IMF4	IMF5	IMF6	IMF7	IMF8	IMF9	trend
2.0000	-35.0300	0.1043	0.0132	0.0391	-0.0602	-0.0782	-0.0753	0.0100	-0.2594	1.8986	-36.6211
2.0200	-34.5700	0.5337	0.0412	0.0296	-0.0640	-0.0779	-0.0749	0.0105	-0.2590	1.9021	-36.6231
2.0400	-35.5900	-0.4578	-0.0641	0.0252	-0.0681	-0.0768	-0.0744	0.0110	-0.2586	1.9056	-36.6251
2.0600	-35.4300	-0.1777	-0.0815	0.0219	-0.0711	-0.0746	-0.0738	0.0116	-0.2582	1.9091	-36.6272

2.0800	-34.9000	0.3071	0.0109	
0.0110	-0.0716	-0.0711	-0.0729	0.0121
	-0.2578	1.9126	-36.6292	
2.1000	-34.8000	0.2897	0.0548	-
0.0097	-0.0690	-0.0665	-0.0720	0.0127
	-0.2575	1.9161	-36.6312	
2.1200	-35.6600	-0.5436	0.0714	-
0.0336	-0.0637	-0.0609	-0.0708	0.0133
	-0.2571	1.9196	-36.6332	
2.1400	-34.8100	0.2673	0.0707	-
0.0498	-0.0563	-0.0543	-0.0696	0.0139
	-0.2567	1.9231	-36.6353	
2.1600	-34.9600	0.3025	-0.0505	-
0.0501	-0.0477	-0.0468	-0.0682	0.0145
	-0.2563	1.9266	-36.6373	
2.1800	-35.4000	-0.1862	-0.2320	-
0.0307	-0.0388	-0.0385	-0.0667	0.0151
	-0.2559	1.9301	-36.6393	
2.2000	-35.5000	-0.3043	-0.2126	
0.0034	-0.0300	-0.0297	-0.0651	0.0158
	-0.2556	1.9336	-36.6414	
2.2200	-34.8100	0.2438	0.0517	
0.0427	-0.0218	-0.0204	-0.0634	0.0164
	-0.2552	1.9371	-36.6434	
2.2400	-34.5300	0.1626	0.2901	
0.0726	-0.0149	-0.0107	-0.0616	0.0171
	-0.2548	1.9406	-36.6454	
2.2600	-34.5700	0.0537	0.2747	
0.0793	-0.0094	-0.0010	-0.0597	0.0178
	-0.2545	1.9441	-36.6475	
2.2800	-34.9300	-0.0446	0.0307	
0.0620	-0.0049	0.0088	-0.0578	0.0185
	-0.2541	1.9476	-36.6495	
2.3000	-35.0900	0.0280	-0.2227	
0.0306	-0.0007	0.0183	-0.0558	0.0192
	-0.2538	1.9511	-36.6515	
2.3200	-35.4900	-0.1977	-0.2976	-
0.0018	0.0041	0.0275	-0.0539	0.0199
	-0.2534	1.9546	-36.6536	
2.3400	-34.9800	0.1320	-0.1417	-
0.0267	0.0101	0.0362	-0.0519	0.0206
	-0.2531	1.9581	-36.6556	
2.3600	-35.0100	-0.1432	0.1069	-
0.0419	0.0177	0.0442	-0.0498	0.0213
	-0.2527	1.9616	-36.6576	
2.3800	-34.6500	0.0307	0.2474	-
0.0535	0.0267	0.0515	-0.0478	0.0221
	-0.2524	1.9650	-36.6597	
2.4000	-34.4100	0.3718	0.1586	-
0.0644	0.0373	0.0579	-0.0458	0.0228
	-0.2520	1.9685	-36.6617	

2.4200 -35.4500 -0.4954 -0.0361 -
0.0702 0.0490 0.0633 -0.0438 0.0236
-0.2517 1.9720 -36.6637
2.4400 -34.7000 0.4087 -0.1209 -
0.0620 0.0608 0.0676 -0.0419 0.0243
-0.2514 1.9755 -36.6658
2.4600 -35.0900 -0.1250 -0.1635 -
0.0354 0.0719 0.0709 -0.0399 0.0251
-0.2510 1.9790 -36.6678
2.4800 -34.7400 0.1098 -0.1520
0.0092 0.0809 0.0730 -0.0381 0.0258
-0.2507 1.9825 -36.6698
2.5000 -35.0600 -0.3699 0.0556
0.0591 0.0869 0.0739 -0.0362 0.0266
-0.2504 1.9860 -36.6719
2.5200 -34.0700 0.3246 0.2527
0.0970 0.0889 0.0736 -0.0345 0.0273
-0.2501 1.9895 -36.6739
2.5400 -34.3300 0.0894 0.1930
0.1095 0.0866 0.0721 -0.0327 0.0281
-0.2498 1.9930 -36.6759
2.5600 -34.9100 -0.1932 -0.0163
0.0955 0.0800 0.0694 -0.0311 0.0288
-0.2495 1.9965 -36.6780
2.5800 -35.0300 -0.1690 -0.1419
0.0648 0.0698 0.0656 -0.0296 0.0296
-0.2491 2.0000 -36.6800
2.6000 -34.7600 0.1635 -0.1224
0.0316 0.0569 0.0608 -0.0281 0.0303
-0.2488 2.0035 -36.6821
2.6200 -34.8000 0.0584 -0.0470
0.0048 0.0422 0.0551 -0.0267 0.0310
-0.2485 2.0070 -36.6841
2.6400 -35.0500 -0.0764 0.0042 -
0.0122 0.0269 0.0486 -0.0254 0.0317
-0.2482 2.0105 -36.6861
2.6600 -34.8300 -0.0061 0.0249 -
0.0230 0.0118 0.0414 -0.0241 0.0324
-0.2479 2.0139 -36.6882
2.6800 -34.8100 0.0970 0.0452 -
0.0337 -0.0017 0.0339 -0.0230 0.0331
-0.2476 2.0174 -36.6902
2.7000 -34.9300 -0.0164 0.0722 -
0.0447 -0.0129 0.0260 -0.0219 0.0338
-0.2473 2.0209 -36.6922
2.7200 -34.9600 0.0463 0.0391 -
0.0531 -0.0215 0.0180 -0.0209 0.0344
-0.2471 2.0244 -36.6943
2.7400 -35.0400 0.0603 -0.0516 -
0.0544 -0.0271 0.0099 -0.0200 0.0351
-0.2468 2.0279 -36.6963

2.7600	-35.1100	0.0112	-0.1409	-
0.0447	-0.0299	0.0021	-0.0192	0.0357
	-0.2465	2.0314	-36.6984	
2.7800	-35.3100	-0.2352	-0.1364	-
0.0239	-0.0298	-0.0054	-0.0184	0.0363
	-0.2462	2.0348	-36.7004	
2.8000	-34.9900	-0.0019	0.0223	
0.0005	-0.0273	-0.0125	-0.0178	0.0369
	-0.2459	2.0383	-36.7025	
2.8200	-34.6300	0.0806	0.2053	
0.0166	-0.0227	-0.0189	-0.0172	0.0375
	-0.2456	2.0418	-36.7045	
2.8400	-34.4600	0.1995	0.2055	
0.0173	-0.0168	-0.0245	-0.0167	0.0380
	-0.2453	2.0452	-36.7065	
2.8600	-35.2000	-0.1936	0.0241	
0.0062	-0.0103	-0.0294	-0.0163	0.0385
	-0.2451	2.0487	-36.7086	
2.8800	-35.2900	-0.1949	-0.1498	-
0.0049	-0.0044	-0.0334	-0.0159	0.0390
	-0.2448	2.0522	-36.7106	
2.9000	-34.7000	0.3797	-0.1935	-
0.0044	0.0002	-0.0364	-0.0157	0.0395
	-0.2445	2.0556	-36.7127	
2.9200	-35.4100	-0.3589	-0.0972	
0.0108	0.0023	-0.0384	-0.0154	0.0399
	-0.2442	2.0591	-36.7147	
2.9400	-34.5900	0.2449	0.0610	
0.0363	0.0013	-0.0394	-0.0152	0.0404
	-0.2439	2.0626	-36.7168	
2.9600	-34.5900	0.1121	0.1504	
0.0589	-0.0032	-0.0393	-0.0151	0.0408
	-0.2437	2.0660	-36.7188	
2.9800	-34.9600	-0.3190	0.1004	
0.0677	-0.0110	-0.0381	-0.0150	0.0411
	-0.2434	2.0695	-36.7209	
3.0000	-34.6100	0.2660	-0.0231	
0.0619	-0.0217	-0.0359	-0.0149	0.0414
	-0.2431	2.0729	-36.7229	
3.0200	-35.1400	-0.1165	-0.1267	
0.0475	-0.0341	-0.0327	-0.0149	0.0417
	-0.2428	2.0763	-36.7250	
3.0400	-35.3700	-0.3164	-0.1139	
0.0300	-0.0468	-0.0286	-0.0148	0.0420
	-0.2425	2.0798	-36.7270	
3.0600	-34.5700	0.3766	-0.0017	
0.0110	-0.0581	-0.0237	-0.0148	0.0422
	-0.2423	2.0832	-36.7291	
3.0800	-35.1100	-0.1701	0.0560	-
0.0083	-0.0660	-0.0181	-0.0148	0.0424
	-0.2420	2.0866	-36.7311	

3.1000	-34.9900	-0.0317	0.0182	-
0.0285	-0.0687	-0.0120	-0.0147	0.0426
	-0.2417	2.0901	-36.7332	
3.1200	-34.9600	0.0230	-0.0441	-
0.0504	-0.0650	-0.0055	-0.0147	0.0427
	-0.2414	2.0935	-36.7352	
3.1400	-35.0800	0.1076	-0.0578	-
0.0743	-0.0546	0.0013	-0.0147	0.0428
	-0.2411	2.0969	-36.7373	
3.1600	-35.3900	-0.3562	0.0277	-
0.0969	-0.0377	0.0080	-0.0147	0.0428
	-0.2408	2.1003	-36.7393	
3.1800	-34.4400	0.3768	0.1284	-
0.1137	-0.0153	0.0147	-0.0147	0.0428
	-0.2405	2.1037	-36.7414	
3.2000	-34.9000	-0.0751	0.0614	-
0.1160	0.0106	0.0210	-0.0147	0.0428
	-0.2402	2.1071	-36.7435	
3.2200	-35.2100	-0.1151	-0.0927	-
0.0947	0.0378	0.0268	-0.0147	0.0427
	-0.2399	2.1105	-36.7455	
3.2400	-35.0700	-0.0776	-0.1507	-
0.0468	0.0641	0.0321	-0.0147	0.0426
	-0.2396	2.1138	-36.7476	
3.2600	-34.5900	0.1463	-0.0942	
0.0228	0.0874	0.0365	-0.0148	0.0424
	-0.2393	2.1172	-36.7496	
3.2800	-34.6100	-0.0975	-0.0031	
0.1009	0.1058	0.0401	-0.0148	0.0422
	-0.2390	2.1206	-36.7517	
3.3000	-34.2400	0.1614	0.0674	
0.1655	0.1173	0.0427	-0.0149	0.0419
	-0.2386	2.1239	-36.7538	
3.3200	-34.6000	-0.2458	0.1274	
0.1949	0.1207	0.0442	-0.0150	0.0416
	-0.2383	2.1273	-36.7558	
3.3400	-34.0500	0.2915	0.1510	
0.1778	0.1152	0.0446	-0.0151	0.0413
	-0.2380	2.1306	-36.7579	
3.3600	-34.7100	-0.2511	0.0561	
0.1158	0.1012	0.0438	-0.0152	0.0409
	-0.2376	2.1339	-36.7600	
3.3800	-34.6500	0.1378	-0.0610	
0.0263	0.0804	0.0419	-0.0153	0.0405
	-0.2373	2.1373	-36.7620	
3.4000	-34.8500	-0.0271	-0.1065	-
0.0667	0.0550	0.0391	-0.0155	0.0400
	-0.2369	2.1406	-36.7641	
3.4200	-35.0100	-0.0269	-0.0979	-
0.1367	0.0276	0.0354	-0.0157	0.0395
	-0.2366	2.1439	-36.7662	

3.4400 -34.8100 0.2399 -0.0988 -
0.1611 0.0006 0.0310 -0.0159 0.0389
-0.2362 2.1472 -36.7682
3.4600 -35.2600 -0.1737 -0.1171 -
0.1311 -0.0241 0.0261 -0.0161 0.0383
-0.2358 2.1505 -36.7703
3.4800 -35.1000 -0.0520 -0.0684 -
0.0625 -0.0450 0.0206 -0.0163 0.0376
-0.2354 2.1537 -36.7724
3.5000 -34.9100 -0.1556 0.0800
0.0179 -0.0611 0.0149 -0.0166 0.0369
-0.2350 2.1570 -36.7744
3.5200 -34.2200 0.4094 0.2166
0.0815 -0.0715 0.0090 -0.0168 0.0361
-0.2346 2.1602 -36.7765
3.5400 -34.8400 -0.2490 0.1819
0.1010 -0.0754 0.0032 -0.0171 0.0353
-0.2342 2.1635 -36.7786
3.5600 -34.7100 0.0729 0.0449
0.0694 -0.0727 -0.0024 -0.0174 0.0345
-0.2337 2.1667 -36.7806
3.5800 -34.8400 0.0362 -0.0557
0.0040 -0.0641 -0.0078 -0.0177 0.0336
-0.2333 2.1699 -36.7827
3.6000 -35.2500 -0.1321 -0.0833 -
0.0653 -0.0510 -0.0126 -0.0179 0.0326
-0.2328 2.1731 -36.7848
3.6200 -34.9900 0.0481 -0.0912 -
0.1100 -0.0346 -0.0169 -0.0182 0.0316
-0.2324 2.1763 -36.7869
3.6400 -35.0100 0.1597 -0.1274 -
0.1131 -0.0165 -0.0204 -0.0185 0.0306
-0.2319 2.1795 -36.7889
3.6600 -35.2100 -0.2035 -0.1266 -
0.0722 0.0016 -0.0233 -0.0188 0.0295
-0.2314 2.1827 -36.7910
3.6800 -34.8700 -0.0568 0.0135 -
0.0014 0.0183 -0.0253 -0.0191 0.0283
-0.2309 2.1858 -36.7931
3.7000 -34.4400 0.1232 0.1846
0.0740 0.0323 -0.0264 -0.0194 0.0272
-0.2303 2.1890 -36.7952
3.7200 -34.4400 0.0576 0.1956
0.1291 0.0425 -0.0266 -0.0197 0.0259
-0.2298 2.1921 -36.7973
3.7400 -34.4900 0.1319 0.0429
0.1470 0.0479 -0.0260 -0.0200 0.0247
-0.2292 2.1952 -36.7993
3.7600 -35.1900 -0.2967 -0.1112
0.1258 0.0482 -0.0245 -0.0203 0.0233
-0.2287 2.1983 -36.8014

3.7800 -34.7500 0.2518 -0.1455
0.0753 0.0436 -0.0223 -0.0206 0.0220
-0.2281 2.2013 -36.8035
3.8000 -34.8800 -0.0004 -0.0979
0.0126 0.0349 -0.0194 -0.0209 0.0206
-0.2275 2.2044 -36.8056
3.8200 -35.1100 -0.1965 0.0026 -
0.0462 0.0236 -0.0159 -0.0212 0.0191
-0.2268 2.2074 -36.8077
3.8400 -34.6200 0.1626 0.1100 -
0.0867 0.0111 -0.0118 -0.0215 0.0176
-0.2262 2.2105 -36.8098
3.8600 -34.9500 -0.2005 0.1130 -
0.1035 -0.0007 -0.0074 -0.0219 0.0161
-0.2255 2.2135 -36.8119
3.8800 -34.4200 0.4862 -0.0368 -
0.0991 -0.0105 -0.0026 -0.0223 0.0145
-0.2248 2.2165 -36.8139
3.9000 -35.5500 -0.5608 -0.1460 -
0.0798 -0.0170 0.0025 -0.0227 0.0129
-0.2241 2.2194 -36.8160
3.9200 -34.9200 0.0980 -0.0507 -
0.0544 -0.0199 0.0077 -0.0231 0.0112
-0.2234 2.2224 -36.8181
3.9400 -34.4200 0.3514 0.0865 -
0.0296 -0.0188 0.0130 -0.0237 0.0095
-0.2226 2.2253 -36.8202
3.9600 -34.5500 0.1001 0.0908 -
0.0076 -0.0137 0.0182 -0.0242 0.0078
-0.2218 2.2282 -36.8223
3.9800 -35.1000 -0.3501 0.0194
0.0129 -0.0055 0.0234 -0.0248 0.0060
-0.2210 2.2311 -36.8244
4.0000 -34.6200 0.1795 -0.0373
0.0324 0.0049 0.0283 -0.0255 0.0042
-0.2202 2.2340 -36.8265
4.0200 -34.7400 0.0101 -0.0440
0.0521 0.0158 0.0329 -0.0262 0.0024
-0.2193 2.2369 -36.8286
4.0400 -34.8000 -0.1564 -0.0092
0.0714 0.0256 0.0372 -0.0270 0.0005
-0.2185 2.2397 -36.8307
4.0600 -34.5400 0.1172 0.0289
0.0862 0.0329 0.0411 -0.0278 -0.0014
-0.2176 2.2425 -36.8328
4.0800 -34.5200 0.1822 0.0278
0.0905 0.0367 0.0445 -0.0287 -0.0034
-0.2166 2.2453 -36.8349
4.1000 -34.9300 -0.2928 0.0314
0.0803 0.0361 0.0474 -0.0296 -0.0053
-0.2157 2.2481 -36.8370

4.1200 -34.3000 0.3198 0.0605
0.0553 0.0310 0.0497 -0.0306 -0.0074
-0.2147 2.2508 -36.8391
4.1400 -34.9300 -0.2149 0.0428
0.0238 0.0218 0.0514 -0.0316 -0.0094
-0.2137 2.2536 -36.8412
4.1600 -34.7100 0.1231 -0.0405 -
0.0038 0.0100 0.0524 -0.0327 -0.0115
-0.2126 2.2563 -36.8433
4.1800 -34.9700 0.0282 -0.1534 -
0.0207 -0.0030 0.0528 -0.0337 -0.0136
-0.2115 2.2589 -36.8454
4.2000 -35.1600 -0.2075 -0.1724 -
0.0268 -0.0156 0.0526 -0.0349 -0.0157
-0.2104 2.2616 -36.8475
4.2200 -34.9500 -0.0554 0.0036 -
0.0265 -0.0263 0.0518 -0.0360 -0.0179
-0.2093 2.2642 -36.8496
4.2400 -34.5100 0.2662 0.1788 -
0.0266 -0.0344 0.0503 -0.0372 -0.0201
-0.2081 2.2668 -36.8517
4.2600 -34.8400 -0.1441 0.1575 -
0.0315 -0.0393 0.0483 -0.0384 -0.0223
-0.2069 2.2694 -36.8538
4.2800 -34.9100 -0.0685 0.0033 -
0.0406 -0.0404 0.0457 -0.0396 -0.0245
-0.2057 2.2720 -36.8559
4.3000 -34.8300 0.1645 -0.1028 -
0.0496 -0.0375 0.0426 -0.0408 -0.0268
-0.2044 2.2745 -36.8580
4.3200 -35.0900 -0.1439 -0.0670 -
0.0525 -0.0303 0.0390 -0.0420 -0.0291
-0.2031 2.2770 -36.8602
4.3400 -34.7900 0.0274 0.0085 -
0.0414 -0.0192 0.0349 -0.0432 -0.0314
-0.2018 2.2795 -36.8623
4.3600 -34.6700 0.2132 -0.0098 -
0.0143 -0.0050 0.0303 -0.0444 -0.0337
-0.2004 2.2819 -36.8644
4.3800 -35.1500 -0.2825 -0.0638
0.0207 0.0111 0.0253 -0.0456 -0.0361
-0.1990 2.2844 -36.8665
4.4000 -34.9000 -0.0996 -0.0389
0.0484 0.0278 0.0200 -0.0468 -0.0384
-0.1975 2.2868 -36.8686
4.4200 -34.3000 0.3407 0.0389
0.0577 0.0440 0.0144 -0.0479 -0.0408
-0.1960 2.2891 -36.8707
4.4400 -34.9500 -0.3378 0.0777
0.0474 0.0582 0.0086 -0.0490 -0.0432
-0.1945 2.2915 -36.8728

4.4600 -34.4400 0.2640 0.0734
0.0221 0.0696 0.0027 -0.0501 -0.0456
-0.1930 2.2938 -36.8750
4.4800 -35.0000 -0.0644 0.0084 -
0.0073 0.0773 -0.0031 -0.0512 -0.0480
-0.1914 2.2961 -36.8771
4.5000 -34.8800 0.0276 -0.0819 -
0.0291 0.0802 -0.0089 -0.0522 -0.0504
-0.1897 2.2983 -36.8792
4.5200 -35.0900 -0.1365 -0.0990 -
0.0360 0.0779 -0.0144 -0.0532 -0.0528
-0.1880 2.3005 -36.8813
4.5400 -34.7500 0.2034 -0.0270 -
0.0275 0.0704 -0.0195 -0.0540 -0.0552
-0.1863 2.3027 -36.8834
4.5600 -34.9200 -0.1953 0.0903 -
0.0107 0.0582 -0.0241 -0.0549 -0.0577
-0.1846 2.3049 -36.8856
4.5800 -34.8200 -0.1566 0.1538
0.0060 0.0420 -0.0280 -0.0556 -0.0601
-0.1828 2.3070 -36.8877
4.6000 -34.5500 0.3693 0.0566
0.0189 0.0225 -0.0313 -0.0563 -0.0625
-0.1809 2.3091 -36.8898
4.6200 -35.3300 -0.2618 -0.1385
0.0272 0.0005 -0.0339 -0.0569 -0.0650
-0.1790 2.3112 -36.8919
4.6400 -35.0700 0.0210 -0.2129
0.0309 -0.0226 -0.0356 -0.0574 -0.0674
-0.1771 2.3132 -36.8941
4.6600 -35.1000 -0.0812 -0.0701
0.0290 -0.0459 -0.0365 -0.0578 -0.0699
-0.1751 2.3152 -36.8962
4.6800 -34.8100 -0.0148 0.1559
0.0189 -0.0681 -0.0364 -0.0582 -0.0723
-0.1731 2.3172 -36.8983
4.7000 -34.5000 0.3362 0.2106 -
0.0016 -0.0878 -0.0354 -0.0584 -0.0748
-0.1710 2.3191 -36.9005
4.7200 -35.2400 -0.2608 0.0408 -
0.0314 -0.1034 -0.0335 -0.0586 -0.0772
-0.1689 2.3210 -36.9026
4.7400 -35.0900 0.0983 -0.1205 -
0.0628 -0.1136 -0.0306 -0.0586 -0.0796
-0.1668 2.3229 -36.9047
4.7600 -35.3100 -0.0743 -0.1324 -
0.0818 -0.1170 -0.0268 -0.0586 -0.0821
-0.1646 2.3247 -36.9069
4.7800 -35.3300 -0.1530 -0.0211 -
0.0788 -0.1127 -0.0223 -0.0584 -0.0845
-0.1623 2.3265 -36.9090

4.8000 -34.6600 0.2660 0.0640 -
0.0544 -0.1006 -0.0170 -0.0582 -0.0869
-0.1600 2.3283 -36.9111
4.8200 -34.8100 0.1930 0.0171 -
0.0142 -0.0810 -0.0112 -0.0579 -0.0893
-0.1576 2.3300 -36.9133
4.8400 -35.3400 -0.4273 -0.0577
0.0292 -0.0551 -0.0050 -0.0576 -0.0917
-0.1553 2.3317 -36.9154
4.8600 -34.4700 0.4135 -0.0196
0.0598 -0.0244 0.0014 -0.0571 -0.0941
-0.1528 2.3333 -36.9175
4.8800 -34.9300 -0.1450 0.0443
0.0668 0.0092 0.0078 -0.0566 -0.0965
-0.1503 2.3350 -36.9197
4.9000 -34.7700 -0.0161 0.0303
0.0522 0.0433 0.0141 -0.0560 -0.0989
-0.1478 2.3365 -36.9218
4.9200 -34.7700 0.1301 -0.0143
0.0241 0.0756 0.0201 -0.0553 -0.1012
-0.1452 2.3381 -36.9240
4.9400 -35.1500 -0.4205 -0.0146 -
0.0040 0.1041 0.0255 -0.0546 -0.1036
-0.1425 2.3396 -36.9261
4.9600 -34.3100 0.4341 0.0270 -
0.0194 0.1273 0.0303 -0.0538 -0.1059
-0.1398 2.3410 -36.9283
4.9800 -35.0900 -0.3811 0.0051 -
0.0177 0.1440 0.0342 -0.0530 -0.1083
-0.1371 2.3425 -36.9304
5.0000 -34.4200 0.3103 -0.0524 -
0.0008 0.1537 0.0371 -0.0521 -0.1106
-0.1342 2.3438 -36.9325
5.0200 -34.9400 -0.2418 -0.0577
0.0222 0.1564 0.0390 -0.0512 -0.1129
-0.1314 2.3452 -36.9347
5.0400 -34.6600 0.1038 -0.0038
0.0400 0.1523 0.0398 -0.0503 -0.1152
-0.1285 2.3465 -36.9368
5.0600 -34.4700 0.0815 0.0562
0.0453 0.1416 0.0395 -0.0493 -0.1175
-0.1255 2.3478 -36.9390
5.0800 -34.7900 -0.1581 0.0763
0.0378 0.1250 0.0382 -0.0483 -0.1198
-0.1225 2.3490 -36.9411
5.1000 -34.5900 0.1119 0.0392
0.0224 0.1034 0.0360 -0.0473 -0.1220
-0.1194 2.3502 -36.9433
5.1200 -34.8200 -0.0768 -0.0516
0.0081 0.0779 0.0329 -0.0463 -0.1243
-0.1163 2.3513 -36.9454

5.1400 -34.8000 0.0909 -0.1200
0.0008 0.0498 0.0290 -0.0453 -0.1265
-0.1131 2.3524 -36.9476
5.1600 -35.2700 -0.3464 -0.0675
0.0035 0.0203 0.0245 -0.0444 -0.1287
-0.1099 2.3535 -36.9497
5.1800 -34.3700 0.3843 0.0655
0.0140 -0.0092 0.0196 -0.0434 -0.1309
-0.1066 2.3545 -36.9519
5.2000 -34.8400 -0.0560 0.0974
0.0235 -0.0377 0.0144 -0.0424 -0.1331
-0.1032 2.3555 -36.9541
5.2200 -34.8700 -0.1226 0.0403
0.0240 -0.0641 0.0090 -0.0415 -0.1353
-0.0998 2.3564 -36.9562
5.2400 -35.1100 -0.1262 0.0293
0.0129 -0.0873 0.0038 -0.0405 -0.1374
-0.0964 2.3573 -36.9584
5.2600 -34.9200 0.0259 0.0475 -
0.0086 -0.1064 -0.0011 -0.0396 -0.1395
-0.0929 2.3582 -36.9605
5.2800 -34.8300 0.2570 -0.0165 -
0.0294 -0.1209 -0.0056 -0.0388 -0.1417
-0.0893 2.3590 -36.9627
5.3000 -35.4500 -0.3682 -0.1020 -
0.0399 -0.1301 -0.0094 -0.0379 -0.1438
-0.0857 2.3597 -36.9648
5.3200 -34.9600 0.1254 -0.0590 -
0.0383 -0.1340 -0.0125 -0.0371 -0.1459
-0.0820 2.3604 -36.9670
5.3400 -35.1500 -0.0136 0.0347 -
0.0250 -0.1327 -0.0146 -0.0363 -0.1479
-0.0783 2.3611 -36.9692
5.3600 -34.8600 0.0673 0.0374 -
0.0032 -0.1262 -0.0159 -0.0356 -0.1500
-0.0745 2.3617 -36.9713
5.3800 -34.8000 0.0979 -0.0208
0.0200 -0.1151 -0.0161 -0.0350 -0.1520
-0.0706 2.3623 -36.9735
5.4000 -35.3500 -0.4307 -0.0093
0.0367 -0.1000 -0.0154 -0.0343 -0.1540
-0.0667 2.3629 -36.9757
5.4200 -34.4100 0.4550 0.0496
0.0431 -0.0817 -0.0137 -0.0338 -0.1560
-0.0627 2.3633 -36.9778
5.4400 -34.8900 -0.0302 0.0186
0.0386 -0.0611 -0.0111 -0.0333 -0.1580
-0.0587 2.3638 -36.9800
5.4600 -35.2700 -0.3724 -0.0346
0.0265 -0.0392 -0.0077 -0.0329 -0.1600
-0.0546 2.3642 -36.9822

5.4800 -34.6800 0.2612 -0.0396
0.0112 -0.0167 -0.0035 -0.0325 -0.1619
-0.0505 2.3645 -36.9843
5.5000 -34.8400 0.0926 -0.0216 -
0.0012 0.0054 0.0013 -0.0322 -0.1638
-0.0463 2.3648 -36.9865
5.5200 -35.1500 -0.2814 -0.0088 -
0.0078 0.0262 0.0066 -0.0320 -0.1657
-0.0421 2.3651 -36.9887
5.5400 -34.5500 0.3620 0.0275 -
0.0075 0.0449 0.0122 -0.0319 -0.1676
-0.0378 2.3653 -36.9908
5.5600 -34.9300 -0.2033 0.0615 -
0.0018 0.0609 0.0181 -0.0320 -0.1694
-0.0334 2.3655 -36.9930
5.5800 -34.9300 -0.2515 0.0345
0.0042 0.0734 0.0240 -0.0321 -0.1713
-0.0290 2.3656 -36.9952
5.6000 -34.2800 0.5032 -0.0280
0.0051 0.0821 0.0298 -0.0323 -0.1731
-0.0245 2.3657 -36.9974
5.6200 -35.3300 -0.5673 -0.0888 -
0.0011 0.0869 0.0354 -0.0326 -0.1749
-0.0200 2.3657 -36.9995
5.6400 -34.3100 0.4298 -0.0209 -
0.0121 0.0882 0.0405 -0.0330 -0.1766
-0.0154 2.3657 -37.0017
5.6600 -34.7600 -0.1009 0.0819 -
0.0230 0.0870 0.0452 -0.0336 -0.1784
-0.0108 2.3656 -37.0039
5.6800 -34.8200 -0.1414 0.0975 -
0.0270 0.0844 0.0492 -0.0343 -0.1801
-0.0061 2.3655 -37.0061
5.7000 -34.6700 0.0294 0.0309 -
0.0215 0.0810 0.0526 -0.0351 -0.1818
-0.0013 2.3653 -37.0083
5.7200 -34.6600 0.1742 -0.0700 -
0.0097 0.0768 0.0551 -0.0360 -0.1835
0.0035 2.3651 -37.0104
5.7400 -34.8700 -0.1158 -0.1462
0.0028 0.0720 0.0569 -0.0371 -0.1852
0.0083 2.3648 -37.0126
5.7600 -34.8500 0.0273 -0.0981
0.0107 0.0664 0.0577 -0.0382 -0.1868
0.0132 2.3645 -37.0148
5.7800 -34.9200 -0.1947 0.0737
0.0116 0.0601 0.0577 -0.0395 -0.1884
0.0182 2.3641 -37.0170
5.8000 -34.6400 -0.0355 0.2008
0.0052 0.0530 0.0568 -0.0409 -0.1900
0.0232 2.3637 -37.0192

5.8200 -34.1600 0.4752 0.1325 -
0.0037 0.0451 0.0551 -0.0424 -0.1916
0.0283 2.3633 -37.0214
5.8400 -35.2600 -0.3897 -0.0874 -
0.0071 0.0361 0.0526 -0.0439 -0.1931
0.0334 2.3627 -37.0235
5.8600 -35.2700 -0.2699 -0.1857
0.0039 0.0255 0.0494 -0.0456 -0.1946
0.0385 2.3622 -37.0257
5.8800 -34.7600 0.2385 -0.0797
0.0328 0.0134 0.0456 -0.0474 -0.1961
0.0437 2.3616 -37.0279
5.9000 -34.7100 -0.0994 0.0828
0.0672 -0.0000 0.0414 -0.0493 -0.1976
0.0490 2.3609 -37.0301
5.9200 -34.2800 0.3194 0.1243
0.0880 -0.0144 0.0368 -0.0513 -0.1991
0.0543 2.3602 -37.0323
5.9400 -34.9200 -0.2219 0.0676
0.0800 -0.0288 0.0319 -0.0533 -0.2005
0.0597 2.3594 -37.0345
5.9600 -34.7500 0.0780 0.0338
0.0405 -0.0420 0.0270 -0.0554 -0.2019
0.0651 2.3586 -37.0367
5.9800 -34.9000 0.0068 0.0120 -
0.0181 -0.0529 0.0220 -0.0576 -0.2033
0.0705 2.3577 -37.0389
6.0000 -35.0400 0.0102 -0.0618 -
0.0730 -0.0606 0.0172 -0.0599 -0.2047
0.0760 2.3568 -37.0411
6.0200 -35.0900 0.0557 -0.1237 -
0.1060 -0.0641 0.0125 -0.0621 -0.2061
0.0816 2.3559 -37.0432
6.0400 -35.3900 -0.2607 -0.0882 -
0.1081 -0.0629 0.0082 -0.0644 -0.2074
0.0872 2.3549 -37.0454
6.0600 -34.9900 -0.0089 0.0287 -
0.0793 -0.0572 0.0042 -0.0667 -0.2087
0.0928 2.3538 -37.0476
6.0800 -34.4800 0.4753 0.0821 -
0.0300 -0.0475 0.0006 -0.0691 -0.2100
0.0985 2.3527 -37.0498
6.1000 -35.2400 -0.4093 0.0017
0.0225 -0.0344 -0.0026 -0.0713 -0.2112
0.1042 2.3515 -37.0520
6.1200 -34.9000 -0.0688 -0.0574
0.0647 -0.0193 -0.0052 -0.0736 -0.2125
0.1099 2.3503 -37.0542
6.1400 -34.6800 0.2277 -0.0180
0.0872 -0.0032 -0.0073 -0.0757 -0.2137
0.1157 2.3490 -37.0564

6.1600 -34.7200 0.0149 0.0738
0.0863 0.0125 -0.0090 -0.0779 -0.2149
0.1216 2.3477 -37.0586
6.1800 -34.8800 -0.2342 0.1228
0.0649 0.0267 -0.0103 -0.0799 -0.2161
0.1274 2.3463 -37.0608
6.2000 -34.3300 0.4070 0.0362
0.0319 0.0382 -0.0112 -0.0817 -0.2172
0.1333 2.3449 -37.0630
6.2200 -35.3100 -0.3194 -0.1227 -
0.0001 0.0462 -0.0117 -0.0835 -0.2183
0.1393 2.3434 -37.0652
6.2400 -35.0100 -0.0105 -0.1337 -
0.0204 0.0503 -0.0120 -0.0851 -0.2194
0.1453 2.3419 -37.0674
6.2600 -35.0000 -0.1178 0.0164 -
0.0291 0.0504 -0.0119 -0.0865 -0.2205
0.1513 2.3403 -37.0696
6.2800 -34.3600 0.4023 0.1046 -
0.0321 0.0470 -0.0115 -0.0878 -0.2215
0.1573 2.3387 -37.0718
6.3000 -35.1100 -0.2782 0.0528 -
0.0335 0.0404 -0.0108 -0.0888 -0.2226
0.1634 2.3370 -37.0740
6.3200 -35.1900 -0.2061 -0.0252 -
0.0325 0.0312 -0.0099 -0.0896 -0.2236
0.1695 2.3353 -37.0762
6.3400 -34.5300 0.4621 -0.0640 -
0.0296 0.0203 -0.0088 -0.0902 -0.2245
0.1757 2.3335 -37.0785
6.3600 -34.9700 -0.0803 -0.0694 -
0.0254 0.0085 -0.0074 -0.0906 -0.2255
0.1818 2.3317 -37.0807
6.3800 -35.3500 -0.4352 -0.0238 -
0.0187 -0.0035 -0.0059 -0.0908 -0.2264
0.1880 2.3298 -37.0829
6.4000 -34.4200 0.5191 0.0549 -
0.0122 -0.0152 -0.0044 -0.0906 -0.2273
0.1943 2.3279 -37.0851
6.4200 -35.2600 -0.4661 0.0605 -
0.0088 -0.0258 -0.0027 -0.0903 -0.2281
0.2005 2.3259 -37.0873
6.4400 -34.5000 0.3101 0.0152 -
0.0080 -0.0346 -0.0010 -0.0896 -0.2290
0.2068 2.3238 -37.0895
6.4600 -35.1800 -0.1854 -0.0097 -
0.0076 -0.0406 0.0007 -0.0887 -0.2298
0.2131 2.3218 -37.0917
6.4800 -34.8900 -0.0070 -0.0017 -
0.0040 -0.0433 0.0024 -0.0875 -0.2305
0.2194 2.3196 -37.0939

6.5000 -34.8700 0.0595 0.0012
0.0039 -0.0425 0.0041 -0.0860 -0.2313
0.2257 2.3174 -37.0961
6.5200 -34.7100 0.2589 -0.0589
0.0160 -0.0383 0.0058 -0.0843 -0.2320
0.2321 2.3152 -37.0984
6.5400 -35.2500 -0.3502 -0.0712
0.0304 -0.0314 0.0074 -0.0822 -0.2327
0.2384 2.3129 -37.1006
6.5600 -34.4700 0.2538 0.0558
0.0411 -0.0228 0.0089 -0.0799 -0.2333
0.2448 2.3105 -37.1028
6.5800 -34.6500 0.0666 0.1299
0.0400 -0.0129 0.0102 -0.0772 -0.2339
0.2512 2.3081 -37.1050
6.6000 -34.7500 0.0306 0.0543
0.0210 -0.0023 0.0112 -0.0743 -0.2345
0.2576 2.3057 -37.1072
6.6200 -35.0200 -0.2043 -0.0120 -
0.0118 0.0086 0.0119 -0.0712 -0.2350
0.2641 2.3032 -37.1094
6.6400 -34.7800 0.1513 0.0279 -
0.0468 0.0192 0.0122 -0.0677 -0.2356
0.2705 2.3006 -37.1117
6.6600 -34.7400 0.0474 0.0338 -
0.0712 0.0285 0.0120 -0.0640 -0.2360
0.2769 2.2980 -37.1139
6.6800 -34.7300 0.0844 -0.0863 -
0.0734 0.0357 0.0113 -0.0601 -0.2364
0.2834 2.2953 -37.1161
6.7000 -35.1200 -0.1612 -0.1847 -
0.0472 0.0408 0.0101 -0.0559 -0.2368
0.2898 2.2926 -37.1183
6.7200 -34.8700 -0.0762 -0.1300
0.0038 0.0436 0.0084 -0.0515 -0.2372
0.2963 2.2899 -37.1205
6.7400 -34.5400 0.1625 0.0216
0.0639 0.0444 0.0061 -0.0469 -0.2375
0.3027 2.2870 -37.1228
6.7600 -34.6400 -0.1165 0.1103
0.1112 0.0437 0.0033 -0.0421 -0.2377
0.3092 2.2842 -37.1250
6.7800 -34.2600 0.3193 0.0935
0.1250 0.0418 -0.0000 -0.0371 -0.2379
0.3156 2.2813 -37.1272
6.8000 -35.1100 -0.5158 0.0806
0.0956 0.0391 -0.0038 -0.0320 -0.2381
0.3221 2.2783 -37.1294
6.8200 -34.1300 0.5567 0.0593
0.0324 0.0359 -0.0081 -0.0267 -0.2382
0.3285 2.2753 -37.1317

6.8400 -35.1400 -0.3961 -0.0413 -
0.0393 0.0323 -0.0128 -0.0212 -0.2383
0.3350 2.2722 -37.1339
6.8600 -34.7500 0.1753 -0.1064 -
0.0954 0.0283 -0.0178 -0.0157 -0.2383
0.3414 2.2690 -37.1361
6.8800 -35.0700 -0.0652 -0.0999 -
0.1183 0.0235 -0.0229 -0.0101 -0.2383
0.3478 2.2659 -37.1383
6.9000 -34.9700 -0.0446 -0.0474 -
0.1028 0.0180 -0.0281 -0.0045 -0.2382
0.3542 2.2626 -37.1406
6.9200 -34.8400 -0.0285 0.0139 -
0.0577 0.0116 -0.0333 0.0012 -0.2380
0.3606 2.2594 -37.1428
6.9400 -34.6200 0.0608 0.0516
0.0004 0.0042 -0.0383 0.0068 -0.2378
0.3670 2.2560 -37.1450
6.9600 -34.8800 -0.2866 0.0879
0.0536 -0.0042 -0.0429 0.0125 -0.2376
0.3733 2.2526 -37.1473
6.9800 -34.2400 0.4200 0.0768
0.0918 -0.0137 -0.0472 0.0181 -0.2373
0.3796 2.2492 -37.1495
7.0000 -35.0700 -0.3791 -0.0227
0.1115 -0.0242 -0.0508 0.0236 -0.2369
0.3860 2.2457 -37.1517
7.0200 -34.7600 -0.0360 -0.0851
0.1130 -0.0354 -0.0538 0.0290 -0.2365
0.3923 2.2422 -37.1540
7.0400 -34.5400 0.2932 -0.0544
0.0995 -0.0468 -0.0560 0.0342 -0.2361
0.3985 2.2386 -37.1562
7.0600 -34.7600 -0.0504 0.0304
0.0725 -0.0573 -0.0573 0.0393 -0.2355
0.4048 2.2349 -37.1584
7.0800 -34.9200 -0.1951 0.1131
0.0314 -0.0656 -0.0576 0.0442 -0.2349
0.4110 2.2312 -37.1607
7.1000 -34.5800 0.0635 0.1156 -
0.0182 -0.0703 -0.0569 0.0488 -0.2343
0.4171 2.2275 -37.1629
7.1200 -34.6500 0.3118 -0.0033 -
0.0662 -0.0702 -0.0552 0.0532 -0.2336
0.4233 2.2237 -37.1651
7.1400 -35.1500 -0.0029 -0.1508 -
0.1020 -0.0644 -0.0524 0.0574 -0.2328
0.4294 2.2198 -37.1674
7.1600 -35.6700 -0.6450 -0.1429 -
0.1159 -0.0526 -0.0485 0.0612 -0.2320
0.4355 2.2159 -37.1696

7.1800 -34.3700 0.5524 0.0281 -
0.1034 -0.0354 -0.0436 0.0647 -0.2310
0.4415 2.2120 -37.1719
7.2000 -34.5100 0.2380 0.1146 -
0.0704 -0.0139 -0.0378 0.0679 -0.2301
0.4475 2.2080 -37.1741
7.2200 -35.1600 -0.5708 0.0511 -
0.0265 0.0105 -0.0311 0.0707 -0.2290
0.4535 2.2039 -37.1763
7.2400 -34.5000 0.2046 -0.0434
0.0192 0.0358 -0.0236 0.0732 -0.2279
0.4594 2.1998 -37.1786
7.2600 -34.5000 0.1141 -0.0414
0.0593 0.0603 -0.0155 0.0752 -0.2267
0.4652 2.1957 -37.1808
7.2800 -34.7100 -0.2307 0.0316
0.0874 0.0819 -0.0067 0.0769 -0.2255
0.4711 2.1915 -37.1831
7.3000 -34.3600 0.0669 0.0742
0.0982 0.0987 0.0025 0.0781 -0.2242
0.4768 2.1872 -37.1853
7.3200 -34.1700 0.3139 0.0236
0.0916 0.1088 0.0122 0.0789 -0.2228
0.4826 2.1829 -37.1875
7.3400 -34.5900 -0.1584 -0.0710
0.0722 0.1112 0.0223 0.0793 -0.2213
0.4882 2.1785 -37.1898
7.3600 -34.8000 -0.2512 -0.0754
0.0454 0.1055 0.0327 0.0791 -0.2198
0.4938 2.1741 -37.1920
7.3800 -34.2200 0.3490 -0.0047
0.0165 0.0922 0.0433 0.0785 -0.2182
0.4994 2.1697 -37.1943
7.4000 -34.6100 -0.0638 0.0252 -
0.0083 0.0722 0.0542 0.0774 -0.2165
0.5049 2.1652 -37.1965
7.4200 -34.7700 -0.1161 0.0275 -
0.0277 0.0471 0.0653 0.0758 -0.2148
0.5103 2.1606 -37.1988
7.4400 -34.4700 0.0590 0.0452 -
0.0420 0.0186 0.0766 0.0737 -0.2130
0.5157 2.1560 -37.2010
7.4600 -34.6200 0.0141 0.0604 -
0.0494 -0.0112 0.0879 0.0711 -0.2111
0.5210 2.1513 -37.2033
7.4800 -34.6600 -0.0242 0.0475 -
0.0469 -0.0404 0.0992 0.0679 -0.2091
0.5263 2.1466 -37.2055
7.5000 -34.7100 -0.0599 -0.0026 -
0.0337 -0.0671 0.1103 0.0641 -0.2071
0.5314 2.1419 -37.2078

7.5200 -34.6200 0.1807 -0.0850 -
0.0116 -0.0896 0.1212 0.0599 -0.2050
0.5365 2.1371 -37.2100
7.5400 -34.8800 -0.1172 -0.1391
0.0134 -0.1069 0.1318 0.0550 -0.2028
0.5416 2.1322 -37.2123
7.5600 -34.5700 0.0636 -0.0654
0.0297 -0.1181 0.1417 0.0496 -0.2005
0.5465 2.1273 -37.2145
7.5800 -34.7900 -0.1970 0.1019
0.0277 -0.1230 0.1508 0.0436 -0.1982
0.5514 2.1223 -37.2168
7.6000 -34.2200 0.3271 0.1797
0.0064 -0.1213 0.1590 0.0371 -0.1958
0.5562 2.1173 -37.2190
7.6200 -34.8800 -0.3243 0.0815 -
0.0274 -0.1136 0.1660 0.0300 -0.1933
0.5610 2.1123 -37.2213
7.6400 -34.5800 0.1737 -0.0716 -
0.0573 -0.1001 0.1717 0.0224 -0.1907
0.5656 2.1071 -37.2235
7.6600 -34.8300 0.0297 -0.2013 -
0.0659 -0.0813 0.1760 0.0142 -0.1880
0.5702 2.1020 -37.2258
7.6800 -34.9000 0.0097 -0.2357 -
0.0424 -0.0579 0.1788 0.0056 -0.1852
0.5747 2.0968 -37.2281
7.7000 -34.8700 -0.3100 -0.0562
0.0065 -0.0310 0.1798 -0.0035 -0.1823
0.5791 2.0915 -37.2303
7.7200 -34.2300 0.1617 0.2219
0.0609 -0.0017 0.1788 -0.0130 -0.1793
0.5834 2.0862 -37.2326
7.7400 -33.9600 0.2276 0.2825
0.1012 0.0292 0.1758 -0.0229 -0.1763
0.5876 2.0809 -37.2348
7.7600 -34.5000 -0.1442 0.0583
0.1145 0.0608 0.1704 -0.0331 -0.1731
0.5917 2.0755 -37.2371
7.7800 -34.5700 0.0486 -0.1716
0.0983 0.0926 0.1625 -0.0437 -0.1698
0.5958 2.0700 -37.2394
7.8000 -34.8300 -0.2098 -0.1805
0.0577 0.1239 0.1520 -0.0545 -0.1664
0.5997 2.0645 -37.2416
7.8200 -34.3700 0.0698 0.0086
0.0003 0.1542 0.1388 -0.0655 -0.1629
0.6036 2.0590 -37.2439
7.8400 -34.4300 -0.0330 0.1880 -
0.0622 0.1825 0.1230 -0.0767 -0.1593
0.6073 2.0534 -37.2461

7.8600 -34.3000 0.2152 0.1503 -
0.1155 0.2074 0.1048 -0.0880 -0.1556
0.6110 2.0477 -37.2484
7.8800 -34.9000 -0.1643 -0.0480 -
0.1443 0.2272 0.0845 -0.0994 -0.1517
0.6145 2.0420 -37.2507
7.9000 -34.8500 -0.0275 -0.1822 -
0.1376 0.2402 0.0622 -0.1107 -0.1478
0.6180 2.0363 -37.2529
7.9200 -34.8800 0.0921 -0.1800 -
0.0892 0.2445 0.0384 -0.1221 -0.1437
0.6213 2.0305 -37.2552
7.9400 -34.5900 0.0597 -0.0871 -
0.0071 0.2386 0.0134 -0.1333 -0.1395
0.6246 2.0247 -37.2575
7.9600 -34.7600 -0.2130 0.0672
0.0880 0.2213 -0.0123 -0.1444 -0.1351
0.6277 2.0188 -37.2597
7.9800 -34.2600 0.1639 0.2036
0.1701 0.1919 -0.0384 -0.1552 -0.1306
0.6307 2.0129 -37.2620
8.0000 -34.2900 0.1921 0.1823
0.2175 0.1505 -0.0644 -0.1657 -0.1260
0.6336 2.0069 -37.2642
8.0200 -34.7000 -0.0576 -0.0166
0.2240 0.0977 -0.0898 -0.1760 -0.1213
0.6365 2.0009 -37.2665
8.0400 -35.1100 -0.1016 -0.2071
0.2010 0.0354 -0.1142 -0.1858 -0.1164
0.6391 1.9948 -37.2688
8.0600 -35.1600 -0.0545 -0.1981
0.1626 -0.0340 -0.1371 -0.1952 -0.1114
0.6417 1.9887 -37.2711
8.0800 -35.2600 -0.0927 -0.0098
0.1182 -0.1069 -0.1580 -0.2040 -0.1062
0.6442 1.9825 -37.2733
8.1000 -34.8600 0.2874 0.1359
0.0691 -0.1786 -0.1766 -0.2124 -0.1009
0.6465 1.9763 -37.2756
8.1200 -35.1200 0.0929 0.0978
0.0114 -0.2442 -0.1923 -0.2201 -0.0954
0.6488 1.9701 -37.2779
8.1400 -35.9200 -0.3064 -0.0057 -
0.0637 -0.2986 -0.2048 -0.2271 -0.0898
0.6509 1.9638 -37.2801
8.1600 -36.0100 -0.3033 0.0296 -
0.1573 -0.3375 -0.2137 -0.2334 -0.0840
0.6529 1.9574 -37.2824
8.1800 -35.1700 0.5175 0.1211 -
0.2489 -0.3573 -0.2187 -0.2389 -0.0781
0.6548 1.9510 -37.2847

8.2000 -35.9500 -0.1407 -0.0029 -
0.3055 -0.3555 -0.2197 -0.2436 -0.0720
0.6565 1.9446 -37.2869
8.2200 -36.0500 0.0700 -0.3042 -
0.2988 -0.3310 -0.2165 -0.2475 -0.0658
0.6582 1.9381 -37.2892
8.2400 -36.6300 -0.5089 -0.3704 -
0.2163 -0.2853 -0.2096 -0.2505 -0.0594
0.6597 1.9316 -37.2915
8.2600 -35.2800 0.2325 -0.0412 -
0.0707 -0.2222 -0.1993 -0.2527 -0.0529
0.6611 1.9250 -37.2938
8.2800 -34.8700 0.1128 0.2969
0.0924 -0.1469 -0.1859 -0.2540 -0.0461
0.6624 1.9184 -37.2960
8.3000 -34.6700 0.1164 0.3142
0.2215 -0.0650 -0.1701 -0.2545 -0.0393
0.6636 1.9118 -37.2983
8.3200 -34.8300 -0.1013 0.1018
0.2834 0.0181 -0.1520 -0.2542 -0.0322
0.6646 1.9051 -37.3006
8.3400 -34.9000 -0.0877 -0.0761
0.2698 0.0973 -0.1323 -0.2530 -0.0250
0.6655 1.8983 -37.3029
8.3600 -35.0400 -0.1273 -0.0883
0.1925 0.1685 -0.1113 -0.2511 -0.0176
0.6663 1.8915 -37.3051
8.3800 -34.4600 0.3256 -0.0169
0.0809 0.2286 -0.0893 -0.2484 -0.0101
0.6670 1.8847 -37.3074
8.4000 -34.9700 -0.0884 0.0133 -
0.0311 0.2749 -0.0669 -0.2450 -0.0024
0.6675 1.8778 -37.3097
8.4200 -35.0400 -0.1242 -0.0274 -
0.1144 0.3055 -0.0443 -0.2409 0.0054
0.6680 1.8709 -37.3120
8.4400 -34.6200 0.3327 -0.0600 -
0.1544 0.3195 -0.0220 -0.2361 0.0135
0.6683 1.8640 -37.3143
8.4600 -35.2800 -0.4610 -0.0248 -
0.1507 0.3170 -0.0002 -0.2306 0.0216
0.6685 1.8570 -37.3165
8.4800 -34.5200 0.2738 0.0643 -
0.1124 0.2990 0.0208 -0.2245 0.0300
0.6685 1.8499 -37.3188
8.5000 -34.6300 0.0723 0.0619 -
0.0519 0.2671 0.0408 -0.2177 0.0385
0.6684 1.8429 -37.3211
8.5200 -34.8500 -0.1580 -0.0479
0.0128 0.2236 0.0596 -0.2104 0.0471
0.6683 1.8357 -37.3234

8.5400 -34.7900 0.0045 -0.1096
0.0660 0.1717 0.0771 -0.2024 0.0559
0.6680 1.8286 -37.3257
8.5600 -34.6900 -0.1316 -0.0238
0.0991 0.1146 0.0932 -0.1940 0.0648
0.6675 1.8214 -37.3279
8.5800 -34.4600 0.1174 0.1184
0.1072 0.0551 0.1077 -0.1850 0.0739
0.6670 1.8141 -37.3302
8.6000 -34.4000 0.1270 0.1490
0.0900 -0.0042 0.1207 -0.1755 0.0830
0.6663 1.8068 -37.3325
8.6200 -34.7200 0.0387 0.0133
0.0558 -0.0607 0.1320 -0.1656 0.0924
0.6655 1.7995 -37.3348
8.6400 -35.1800 -0.1500 -0.1262
0.0170 -0.1119 0.1417 -0.1552 0.1018
0.6646 1.7921 -37.3371
8.6600 -35.1600 0.0106 -0.1394 -
0.0135 -0.1555 0.1496 -0.1444 0.1113
0.6636 1.7847 -37.3394
8.6800 -35.1400 -0.0823 -0.0827 -
0.0262 -0.1891 0.1558 -0.1333 0.1210
0.6624 1.7773 -37.3417
8.7000 -34.6900 0.3936 -0.0882 -
0.0194 -0.2110 0.1603 -0.1218 0.1307
0.6611 1.7698 -37.3439
8.7200 -35.5400 -0.5173 -0.0478 -
0.0019 -0.2200 0.1630 -0.1099 0.1406
0.6597 1.7623 -37.3462
8.7400 -34.4100 0.4519 0.1254
0.0114 -0.2156 0.1641 -0.0978 0.1505
0.6582 1.7547 -37.3485
8.7600 -34.8600 -0.1019 0.1672
0.0029 -0.1978 0.1637 -0.0855 0.1605
0.6566 1.7471 -37.3508
8.7800 -34.7200 0.1137 0.0255 -
0.0337 -0.1677 0.1619 -0.0729 0.1706
0.6548 1.7395 -37.3531
8.8000 -35.2400 -0.3033 -0.0397 -
0.0882 -0.1272 0.1588 -0.0601 0.1807
0.6529 1.7318 -37.3554
8.8200 -34.8700 -0.0485 -0.0088 -
0.1356 -0.0796 0.1546 -0.0471 0.1909
0.6509 1.7241 -37.3577
8.8400 -34.4600 0.4429 -0.0469 -
0.1458 -0.0285 0.1492 -0.0339 0.2011
0.6488 1.7164 -37.3600
8.8600 -35.2600 -0.4302 -0.1791 -
0.0995 0.0221 0.1429 -0.0207 0.2114
0.6466 1.7086 -37.3623

8.8800 -34.5300 0.1126 -0.1534 -
0.0001 0.0686 0.1358 -0.0073 0.2217
0.6442 1.7008 -37.3645
8.9000 -34.4100 -0.0008 0.0355
0.1230 0.1079 0.1278 0.0061 0.2321
0.6418 1.6929 -37.3668
8.9200 -34.1600 0.0201 0.1695
0.2274 0.1377 0.1192 0.0196 0.2424
0.6392 1.6850 -37.3691
8.9400 -33.9300 0.2227 0.1763
0.2721 0.1559 0.1100 0.0331 0.2528
0.6365 1.6771 -37.3714
8.9600 -34.4700 -0.3123 0.1437
0.2346 0.1616 0.1003 0.0466 0.2631
0.6336 1.6691 -37.3737
8.9800 -33.9500 0.3434 0.1188
0.1222 0.1559 0.0902 0.0600 0.2735
0.6307 1.6612 -37.3760
9.0000 -34.8400 -0.2804 0.0075 -
0.0316 0.1406 0.0797 0.0734 0.2838
0.6276 1.6531 -37.3783
9.0200 -34.7000 0.2270 -0.1401 -
0.1758 0.1178 0.0690 0.0867 0.2941
0.6245 1.6451 -37.3806
9.0400 -35.2500 -0.2175 -0.2035 -
0.2646 0.0901 0.0581 0.0999 0.3044
0.6212 1.6370 -37.3829
9.0600 -34.9100 0.0675 -0.1875 -
0.2701 0.0596 0.0472 0.1130 0.3146
0.6178 1.6288 -37.3852
9.0800 -34.7800 0.1791 -0.1860 -
0.1884 0.0283 0.0364 0.1259 0.3247
0.6143 1.6207 -37.3875
9.1000 -34.7700 0.0627 -0.1599 -
0.0443 -0.0022 0.0259 0.1387 0.3348
0.6106 1.6125 -37.3898
9.1200 -35.0000 -0.5051 0.0436
0.1155 -0.0306 0.0157 0.1512 0.3449
0.6069 1.6042 -37.3921
9.1400 -33.5800 0.6173 0.2929
0.2409 -0.0555 0.0062 0.1636 0.3548
0.6030 1.5960 -37.3944
9.1600 -34.5700 -0.3782 0.2732
0.2910 -0.0757 -0.0027 0.1757 0.3647
0.5990 1.5877 -37.3967
9.1800 -34.4100 -0.0021 0.1251
0.2537 -0.0905 -0.0108 0.1876 0.3745
0.5949 1.5793 -37.3990
9.2000 -34.4300 0.1211 0.0663
0.1430 -0.0996 -0.0179 0.1992 0.3842
0.5907 1.5710 -37.4013

9.2200 -34.9900 -0.2380 0.0813 -
0.0071 -0.1028 -0.0239 0.2105 0.3938
0.5864 1.5626 -37.4036
9.2400 -34.7500 0.1735 -0.0031 -
0.1519 -0.1001 -0.0289 0.2216 0.4032
0.5820 1.5542 -37.4059
9.2600 -35.0400 0.2791 -0.2234 -
0.2525 -0.0915 -0.0326 0.2324 0.4125
0.5775 1.5457 -37.4082
9.2800 -35.8600 -0.5145 -0.3471 -
0.2875 -0.0773 -0.0351 0.2429 0.4217
0.5728 1.5372 -37.4105
9.3000 -34.9100 0.1862 -0.1582 -
0.2533 -0.0583 -0.0364 0.2530 0.4308
0.5680 1.5287 -37.4128
9.3200 -34.4500 0.2754 0.1197 -
0.1702 -0.0357 -0.0364 0.2629 0.4397
0.5632 1.5202 -37.4151
9.3400 -34.5000 -0.0056 0.2131 -
0.0672 -0.0114 -0.0352 0.2724 0.4484
0.5582 1.5116 -37.4174
9.3600 -34.4700 -0.0147 0.1295
0.0320 0.0132 -0.0328 0.2816 0.4570
0.5531 1.5030 -37.4197
9.3800 -34.8300 -0.2458 0.0218
0.1107 0.0365 -0.0293 0.2905 0.4654
0.5479 1.4944 -37.4220
9.4000 -34.1800 0.2238 -0.0333
0.1621 0.0569 -0.0248 0.2990 0.4736
0.5426 1.4857 -37.4243
9.4200 -34.4000 -0.0350 -0.0606
0.1859 0.0728 -0.0195 0.3071 0.4816
0.5372 1.4770 -37.4266
9.4400 -34.6200 -0.1100 -0.0471
0.1817 0.0831 -0.0135 0.3150 0.4894
0.5316 1.4683 -37.4289
9.4600 -34.3300 0.0942 -0.0025
0.1518 0.0870 -0.0071 0.3224 0.4970
0.5260 1.4596 -37.4312
9.4800 -34.3600 0.0624 0.0628
0.0998 0.0849 -0.0002 0.3295 0.5044
0.5203 1.4508 -37.4335
9.5000 -34.6000 -0.1737 0.1340
0.0308 0.0774 0.0069 0.3362 0.5115
0.5144 1.4420 -37.4358
9.5200 -34.4400 0.1054 0.1013 -
0.0443 0.0655 0.0141 0.3425 0.5184
0.5084 1.4332 -37.4381
9.5400 -34.5300 0.3068 -0.0819 -
0.1123 0.0501 0.0214 0.3485 0.5251
0.5024 1.4243 -37.4404

9.5600 -35.4700 -0.5109 -0.2146 -
0.1551 0.0325 0.0286 0.3541 0.5315
0.4962 1.4154 -37.4427
9.5800 -34.6300 0.1694 -0.0903 -
0.1586 0.0137 0.0357 0.3593 0.5376
0.4900 1.4065 -37.4450
9.6000 -34.4300 0.1415 0.0816 -
0.1200 -0.0050 0.0425 0.3642 0.5434
0.4836 1.3976 -37.4473
9.6200 -34.7100 -0.0101 0.0614 -
0.0499 -0.0221 0.0489 0.3687 0.5489
0.4771 1.3887 -37.4496
9.6400 -34.8100 0.0027 -0.0679
0.0305 -0.0367 0.0548 0.3728 0.5542
0.4706 1.3797 -37.4519
9.6600 -34.8800 -0.2947 -0.0602
0.0949 -0.0480 0.0602 0.3765 0.5591
0.4639 1.3707 -37.4543
9.6800 -34.4000 0.0982 0.1050
0.1236 -0.0556 0.0648 0.3799 0.5637
0.4571 1.3617 -37.4566
9.7000 -34.0400 0.4019 0.2067
0.1098 -0.0593 0.0687 0.3829 0.5680
0.4503 1.3526 -37.4589
9.7200 -34.9900 -0.4401 0.1091
0.0614 -0.0588 0.0718 0.3856 0.5719
0.4433 1.3435 -37.4612
9.7400 -34.5700 0.2558 -0.0588 -
0.0053 -0.0542 0.0739 0.3879 0.5755
0.4362 1.3344 -37.4635
9.7600 -34.9200 0.0757 -0.1807 -
0.0674 -0.0455 0.0750 0.3900 0.5787
0.4291 1.3253 -37.4658
9.7800 -35.0100 -0.0465 -0.1879 -
0.1029 -0.0330 0.0751 0.3917 0.5816
0.4219 1.3162 -37.4681
9.8000 -35.1000 -0.2677 -0.0478 -
0.1021 -0.0172 0.0741 0.3930 0.5840
0.4145 1.3070 -37.4704
9.8200 -34.2500 0.3966 0.1273 -
0.0728 0.0012 0.0721 0.3941 0.5861
0.4071 1.2978 -37.4727
9.8400 -34.8100 -0.2612 0.1563 -
0.0312 0.0207 0.0689 0.3949 0.5878
0.3996 1.2886 -37.4751
9.8600 -34.6300 -0.0390 0.0936
0.0059 0.0402 0.0646 0.3955 0.5891
0.3920 1.2794 -37.4774
9.8800 -34.6300 0.0328 0.0376
0.0331 0.0581 0.0593 0.3958 0.5900
0.3843 1.2701 -37.4797

9.9000 -34.6000 0.1046 -0.0559
0.0576 0.0729 0.0530 0.3960 0.5905
0.3766 1.2608 -37.4820
9.9200 -34.7300 0.1828 -0.1947
0.0862 0.0834 0.0459 0.3959 0.5905
0.3687 1.2516 -37.4843
9.9400 -35.4400 -0.5515 -0.1568
0.1124 0.0888 0.0379 0.3957 0.5901
0.3608 1.2422 -37.4866
9.9600 -34.0500 0.5908 0.1112
0.1230 0.0891 0.0292 0.3953 0.5893
0.3528 1.2329 -37.4889
9.9800 -34.8300 -0.3378 0.2656
0.1033 0.0846 0.0201 0.3949 0.5881
0.3447 1.2236 -37.4912
10.0000 -34.6000 0.0273 0.1830
0.0486 0.0762 0.0105 0.3943 0.5864
0.3365 1.2142 -37.4936
10.0200 -34.8200 0.1189 -0.0070 -
0.0277 0.0652 0.0007 0.3937 0.5842
0.3282 1.2048 -37.4959
10.0400 -35.2400 -0.1504 -0.1618 -
0.1025 0.0527 -0.0092 0.3931 0.5816
0.3199 1.1954 -37.4982
10.0600 -35.3100 0.0065 -0.2256 -
0.1473 0.0398 -0.0191 0.3925 0.5785
0.3115 1.1859 -37.5005
10.0800 -35.4000 -0.0637 -0.1803 -
0.1460 0.0271 -0.0288 0.3918 0.5750
0.3030 1.1765 -37.5028
10.1000 -35.4200 -0.0905 -0.0600 -
0.1016 0.0153 -0.0381 0.3913 0.5710
0.2944 1.1670 -37.5051
10.1200 -34.7000 0.4097 0.0338 -
0.0320 0.0043 -0.0468 0.3908 0.5665
0.2858 1.1575 -37.5075
10.1400 -35.6500 -0.4559 0.0857
0.0394 -0.0061 -0.0548 0.3904 0.5615
0.2771 1.1480 -37.5098
10.1600 -34.9000 0.1095 0.1607
0.0921 -0.0163 -0.0618 0.3902 0.5560
0.2683 1.1385 -37.5121
10.1800 -34.6900 0.3373 0.1831
0.1129 -0.0266 -0.0676 0.3902 0.5501
0.2595 1.1290 -37.5144
10.2000 -35.3800 -0.2255 0.0857
0.1015 -0.0372 -0.0721 0.3904 0.5436
0.2506 1.1194 -37.5167
10.2200 -35.3000 -0.0139 -0.0661
0.0677 -0.0475 -0.0753 0.3908 0.5367
0.2417 1.1099 -37.5191

10.2400 -35.5300 0.0685 -0.1728
0.0270 -0.0569 -0.0770 0.3915 0.5292
0.2327 1.1003 -37.5214
10.2600 -35.7000 -0.0978 -0.1773 –
0.0093 -0.0644 -0.0774 0.3926 0.5212
0.2236 1.0907 -37.5237
10.2800 -35.4900 0.0397 -0.0782 –
0.0367 -0.0694 -0.0765 0.3940 0.5127
0.2144 1.0811 -37.5260
10.3000 -35.4600 -0.1085 0.0931 –
0.0587 -0.0712 -0.0744 0.3959 0.5037
0.2053 1.0715 -37.5283
10.3200 -35.2300 0.1837 0.2512 –
0.0780 -0.0692 -0.0713 0.3982 0.4942
0.1960 1.0618 -37.5307
10.3400 -35.5800 -0.2050 0.2444 –
0.0897 -0.0637 -0.0673 0.4009 0.4842
0.1867 1.0522 -37.5330
10.3600 -35.2600 0.4431 -0.0237 –
0.0878 -0.0549 -0.0627 0.4042 0.4737
0.1774 1.0425 -37.5353
10.3800 -36.4100 -0.4222 -0.3526 –
0.0650 -0.0437 -0.0577 0.4080 0.4627
0.1680 1.0328 -37.5376
10.4000 -36.0600 -0.1365 -0.3702 –
0.0196 -0.0307 -0.0525 0.4123 0.4511
0.1586 1.0231 -37.5399
10.4200 -35.4700 0.1998 -0.0388
0.0366 -0.0165 -0.0474 0.4173 0.4391
0.1491 1.0134 -37.5423
10.4400 -35.1000 0.0880 0.3080
0.0829 -0.0018 -0.0426 0.4229 0.4266
0.1396 1.0037 -37.5446
10.4600 -35.1700 -0.0124 0.3828
0.1017 0.0127 -0.0383 0.4291 0.4135
0.1300 0.9940 -37.5469
10.4800 -35.2200 0.0793 0.2136
0.0895 0.0265 -0.0348 0.4359 0.4000
0.1204 0.9842 -37.5492
10.5000 -35.5300 -0.0511 -0.0164
0.0567 0.0390 -0.0323 0.4434 0.3861
0.1108 0.9745 -37.5516
10.5200 -35.9200 -0.1377 -0.1749
0.0184 0.0500 -0.0310 0.4515 0.3716
0.1011 0.9647 -37.5539
10.5400 -35.6400 0.2829 -0.2508 –
0.0103 0.0593 -0.0311 0.4603 0.3567
0.0914 0.9549 -37.5562
10.5600 -36.3100 -0.2949 -0.2345 –
0.0238 0.0669 -0.0327 0.4696 0.3414
0.0817 0.9451 -37.5585

10.5800 -35.8200 0.0192 -0.0530 -
0.0265 0.0723 -0.0359 0.4796 0.3256
0.0719 0.9353 -37.5609
10.6000 -35.4700 0.1553 0.1800 -
0.0275 0.0755 -0.0409 0.4901 0.3094
0.0621 0.9255 -37.5632
10.6200 -35.4400 0.1930 0.2158 -
0.0307 0.0764 -0.0477 0.5011 0.2928
0.0523 0.9157 -37.5655
10.6400 -36.0900 -0.2261 0.0463 -
0.0323 0.0747 -0.0562 0.5126 0.2758
0.0425 0.9059 -37.5678
10.6600 -35.8300 0.1319 -0.0961 -
0.0260 0.0697 -0.0664 0.5244 0.2584
0.0326 0.8960 -37.5702
10.6800 -36.2700 -0.2554 -0.0934 -
0.0054 0.0609 -0.0781 0.5366 0.2406
0.0227 0.8862 -37.5725
10.7000 -35.7100 0.3091 -0.0548
0.0278 0.0479 -0.0911 0.5489 0.2226
0.0129 0.8763 -37.5748
10.7200 -36.1400 -0.1090 -0.0369
0.0645 0.0309 -0.1051 0.5614 0.2041
0.0029 0.8665 -37.5771
10.7400 -36.1000 -0.2336 0.0302
0.0930 0.0106 -0.1198 0.5739 0.1854
-0.0070 0.8566 -37.5795
10.7600 -35.6200 0.3277 0.1280
0.1004 -0.0117 -0.1348 0.5863 0.1664
-0.0169 0.8467 -37.5818
10.7800 -36.1900 -0.2193 0.1284
0.0782 -0.0350 -0.1497 0.5986 0.1471
-0.0268 0.8368 -37.5841
10.8000 -35.9900 0.2457 -0.0159
0.0292 -0.0582 -0.1640 0.6107 0.1275
-0.0368 0.8269 -37.5865
10.8200 -36.6500 -0.2331 -0.1426 -
0.0306 -0.0797 -0.1774 0.6224 0.1077
-0.0467 0.8170 -37.5888
10.8400 -36.5400 0.0541 -0.1083 -
0.0834 -0.0978 -0.1893 0.6337 0.0877
-0.0567 0.8071 -37.5911
10.8600 -36.3300 0.1376 0.0079 -
0.1171 -0.1108 -0.1992 0.6444 0.0674
-0.0666 0.7972 -37.5934
10.8800 -36.7000 -0.1320 0.0810 -
0.1258 -0.1175 -0.2068 0.6545 0.0470
-0.0766 0.7873 -37.5958
10.9000 -36.4400 0.1064 0.0858 -
0.1110 -0.1169 -0.2116 0.6639 0.0264
-0.0865 0.7774 -37.5981

10.9200	-36.8000	-0.1922	0.0468	-
0.0783	-0.1087	-0.2132	0.6725	0.0056
	-0.0965	0.7674	-37.6004	
10.9400	-36.4400	0.2383	-0.0550	-
0.0275	-0.0937	-0.2113	0.6801	-0.0153
	-0.1064	0.7575	-37.6028	
10.9600	-36.8700	-0.1161	-0.1728	
0.0394	-0.0739	-0.2055	0.6868	-0.0363
	-0.1163	0.7476	-37.6051	
10.9800	-36.9300	-0.2686	-0.1478	
0.1116	-0.0514	-0.1956	0.6923	-0.0574
	-0.1263	0.7376	-37.6074	
11.0000	-36.1200	0.2559	0.0496	
0.1665	-0.0287	-0.1814	0.6966	-0.0787
	-0.1362	0.7277	-37.6097	
11.0200	-36.3400	-0.1167	0.2183	
0.1825	-0.0081	-0.1629	0.6996	-0.0999
	-0.1461	0.7177	-37.6121	
11.0400	-36.3700	-0.1107	0.1903	
0.1462	0.0076	-0.1398	0.7013	-0.1213
	-0.1559	0.7078	-37.6144	
11.0600	-36.1500	0.4571	0.0100	
0.0627	0.0166	-0.1123	0.7015	-0.1426
	-0.1658	0.6978	-37.6167	
11.0800	-37.3200	-0.5597	-0.1076	-
0.0475	0.0174	-0.0805	0.7002	-0.1640
	-0.1756	0.6878	-37.6191	
11.1000	-36.8800	0.0780	-0.0791	-
0.1546	0.0094	-0.0448	0.6972	-0.1854
	-0.1855	0.6779	-37.6214	
11.1200	-36.3400	0.5573	-0.0352	-
0.2241	-0.0081	-0.0054	0.6925	-0.2068
	-0.1953	0.6679	-37.6237	
11.1400	-37.5600	-0.5678	-0.0650	-
0.2300	-0.0348	0.0369	0.6861	-0.2282
	-0.2050	0.6580	-37.6261	
11.1600	-36.6600	0.3047	-0.0559	-
0.1654	-0.0696	0.0817	0.6779	-0.2496
	-0.2148	0.6480	-37.6284	
11.1800	-36.8800	0.0215	-0.0180	-
0.0421	-0.1093	0.1282	0.6677	-0.2708
	-0.2245	0.6380	-37.6307	
11.2000	-36.6900	0.1781	-0.0884	
0.1148	-0.1505	0.1756	0.6556	-0.2921
	-0.2342	0.6280	-37.6331	
11.2200	-37.1300	-0.3969	-0.1142	
0.2715	-0.1893	0.2230	0.6415	-0.3132
	-0.2438	0.6181	-37.6354	
11.2400	-36.3800	0.0533	0.0514	
0.3951	-0.2215	0.2697	0.6252	-0.3342
	-0.2534	0.6081	-37.6377	

11.2600 -36.0200 0.2504 0.2190
0.4542 -0.2436 0.3148 0.6069 -0.3552
-0.2630 0.5981 -37.6401
11.2800 -36.1100 0.2834 0.1646
0.4254 -0.2527 0.3574 0.5863 -0.3760
-0.2725 0.5882 -37.6424
11.3000 -36.9600 -0.2990 0.0151
0.3061 -0.2474 0.3969 0.5636 -0.3967
-0.2820 0.5782 -37.6447
11.3200 -37.0100 -0.1653 -0.0192
0.1141 -0.2273 0.4323 0.5385 -0.4172
-0.2915 0.5682 -37.6471
11.3400 -36.9000 0.3082 0.0139 -
0.1218 -0.1930 0.4632 0.5112 -0.4376
-0.3009 0.5583 -37.6494
11.3600 -37.3500 0.1265 -0.0383 -
0.3645 -0.1453 0.4889 0.4817 -0.4578
-0.3103 0.5483 -37.6517
11.3800 -38.0200 -0.3100 -0.1106 -
0.5751 -0.0851 0.5091 0.4498 -0.4778
-0.3196 0.5383 -37.6541
11.4000 -37.8800 0.0010 -0.0773 -
0.7193 -0.0132 0.5233 0.4157 -0.4977
-0.3289 0.5284 -37.6564
11.4200 -37.8200 0.0630 -0.0070 -
0.7694 0.0690 0.5312 0.3793 -0.5173
-0.3381 0.5184 -37.6587
11.4400 -37.4400 0.3421 -0.0330 -
0.7134 0.1590 0.5325 0.3407 -0.5367
-0.3472 0.5085 -37.6611
11.4600 -38.1700 -0.5762 -0.0739 -
0.5585 0.2527 0.5270 0.3000 -0.5558
-0.3563 0.4985 -37.6634
11.4800 -36.9100 0.5188 -0.0562 -
0.3291 0.3446 0.5144 0.2573 -0.5747
-0.3654 0.4886 -37.6658
11.5000 -37.0400 0.1233 -0.0257 -
0.0545 0.4288 0.4946 0.2126 -0.5933
-0.3744 0.4786 -37.6681
11.5200 -37.2600 -0.5402 0.0303
0.2351 0.4995 0.4673 0.1662 -0.6116
-0.3833 0.4687 -37.6704
11.5400 -35.9500 0.5195 0.0982
0.5071 0.5508 0.4326 0.1181 -0.6297
-0.3922 0.4588 -37.6728
11.5600 -36.6900 -0.2530 0.0452
0.7260 0.5769 0.3905 0.0687 -0.6474
-0.4010 0.4488 -37.6751
11.5800 -36.3200 0.2850 -0.0926
0.8528 0.5726 0.3416 0.0180 -0.6648
-0.4097 0.4389 -37.6774

11.6000 -37.1800 -0.4848 -0.0314
0.8573 0.5345 0.2864 -0.0339 -0.6819
-0.4184 0.4290 -37.6798
11.6200 -36.7200 0.1410 0.2301
0.7294 0.4634 0.2261 -0.0866 -0.6986
-0.4270 0.4191 -37.6821
11.6400 -36.6600 0.4273 0.3836
0.4857 0.3647 0.1620 -0.1401 -0.7149
-0.4355 0.4092 -37.6845
11.6600 -37.8400 0.1357 0.1701
0.1660 0.2456 0.0953 -0.1943 -0.7309
-0.4439 0.3993 -37.6868
11.6800 -38.8500 0.2498 -0.2654 -
0.1664 0.1139 0.0273 -0.2488 -0.7464
-0.4523 0.3894 -37.6891
11.7000 -40.5100 -0.6923 -0.4901 -
0.4434 -0.0231 -0.0407 -0.3037 -0.7616
-0.4606 0.3795 -37.6915
11.7200 -40.3800 -0.4048 -0.2948 -
0.6107 -0.1577 -0.1076 -0.3586 -0.7763
-0.4688 0.3696 -37.6938
11.7400 -39.3300 0.7592 0.0909 -
0.6568 -0.2821 -0.1719 -0.4135 -0.7906
-0.4769 0.3597 -37.6961
11.7600 -39.6700 0.4011 0.2909 -
0.5993 -0.3895 -0.2325 -0.4681 -0.8044
-0.4850 0.3498 -37.6985
11.7800 -40.9700 -0.7284 0.1914 -
0.4728 -0.4736 -0.2881 -0.5223 -0.8178
-0.4929 0.3400 -37.7008
11.8000 -40.6600 -0.1588 -0.0548 -
0.3249 -0.5296 -0.3374 -0.5760 -0.8307
-0.5008 0.3301 -37.7032
11.8200 -39.7400 0.8334 -0.1510 -
0.2033 -0.5538 -0.3793 -0.6290 -0.8431
-0.5086 0.3203 -37.7055
11.8400 -41.4000 -1.0118 0.0805 -
0.1308 -0.5457 -0.4129 -0.6810 -0.8550
-0.5163 0.3105 -37.7078
11.8600 -39.3100 1.0557 0.2310 -
0.0917 -0.5085 -0.4378 -0.7320 -0.8663
-0.5239 0.3006 -37.7102
11.8800 -41.1800 -0.6198 -0.0268 -
0.0604 -0.4469 -0.4541 -0.7818 -0.8771
-0.5314 0.2908 -37.7125
11.9000 -41.6200 -0.7680 -0.2814 -
0.0195 -0.3676 -0.4619 -0.8303 -0.8874
-0.5388 0.2810 -37.7149
11.9200 -40.4100 0.2560 -0.2020
0.0399 -0.2779 -0.4617 -0.8773 -0.8971
-0.5461 0.2712 -37.7172

11.9400 -40.0000 0.2850 0.1136
0.1051 -0.1847 -0.4538 -0.9227 -0.9063
-0.5533 0.2615 -37.7195
11.9600 -40.0900 -0.0462 0.2946
0.1552 -0.0944 -0.4388 -0.9665 -0.9148
-0.5604 0.2517 -37.7219
11.9800 -39.6600 0.3921 0.0880
0.1770 -0.0124 -0.4171 -1.0085 -0.9227
-0.5674 0.2419 -37.7242
12.0000 -40.8500 -0.4477 -0.2213
0.1750 0.0572 -0.3895 -1.0486 -0.9301
-0.5743 0.2322 -37.7266
12.0200 -40.2900 0.1660 -0.2396
0.1617 0.1121 -0.3565 -1.0869 -0.9367
-0.5811 0.2224 -37.7289
12.0400 -40.1700 0.0768 -0.0515
0.1417 0.1512 -0.3190 -1.1231 -0.9428
-0.5877 0.2127 -37.7312
12.0600 -40.0700 0.0155 0.1312
0.1116 0.1750 -0.2779 -1.1573 -0.9481
-0.5943 0.2030 -37.7336
12.0800 -40.1300 -0.1252 0.2660
0.0579 0.1851 -0.2342 -1.1893 -0.9528
-0.6008 0.1933 -37.7359
12.1000 -39.8200 0.2608 0.2597 -
0.0175 0.1836 -0.1892 -1.2191 -0.9568
-0.6071 0.1836 -37.7383
12.1200 -40.6400 -0.1748 -0.0072 -
0.0939 0.1733 -0.1440 -1.2467 -0.9601
-0.6133 0.1740 -37.7406
12.1400 -40.6800 0.0533 -0.2769 -
0.1387 0.1575 -0.0998 -1.2719 -0.9627
-0.6194 0.1643 -37.7429
12.1600 -41.5000 -0.7542 -0.2404 -
0.1251 0.1399 -0.0580 -1.2948 -0.9645
-0.6254 0.1547 -37.7453
12.1800 -39.4800 1.1620 -0.1795 -
0.0523 0.1237 -0.0198 -1.3152 -0.9656
-0.6313 0.1450 -37.7476
12.2000 -41.7000 -1.2363 -0.1165
0.0533 0.1114 0.0138 -1.3331 -0.9659
-0.6370 0.1354 -37.7500
12.2200 -40.7800 -0.7641 0.1992
0.1461 0.1051 0.0417 -1.3485 -0.9655
-0.6426 0.1258 -37.7523
12.2400 -38.6000 1.2486 0.4170
0.1857 0.1061 0.0631 -1.3613 -0.9643
-0.6481 0.1162 -37.7547
12.2600 -41.0800 -0.7676 0.0414
0.1511 0.1149 0.0775 -1.3714 -0.9622
-0.6535 0.1067 -37.7570

12.2800 -40.8700 -0.0329 -0.3794
0.0576 0.1314 0.0846 -1.3789 -0.9594
-0.6587 0.0971 -37.7593
12.3000 -40.9000 -0.4485 -0.0471 -
0.0616 0.1549 0.0847 -1.3838 -0.9558
-0.6638 0.0876 -37.7617
12.3200 -39.3900 0.6690 0.4072 -
0.1644 0.1839 0.0779 -1.3860 -0.9514
-0.6688 0.0781 -37.7640
12.3400 -41.2400 -0.7380 0.1433 -
0.2093 0.2161 0.0650 -1.3857 -0.9462
-0.6736 0.0685 -37.7664
12.3600 -40.1800 0.9160 -0.4448 -
0.1724 0.2486 0.0467 -1.3828 -0.9402
-0.6783 0.0591 -37.7687
12.3800 -41.9000 -1.1192 -0.3140 -
0.0559 0.2771 0.0239 -1.3775 -0.9334
-0.6829 0.0496 -37.7711
12.4000 -38.8300 1.2219 0.2538
0.0978 0.2969 -0.0025 -1.3698 -0.9258
-0.6873 0.0401 -37.7734
12.4200 -40.3100 -0.3866 0.3404
0.2380 0.3028 -0.0315 -1.3597 -0.9175
-0.6916 0.0307 -37.7757
12.4400 -41.1100 -1.0700 0.1176
0.3178 0.2907 -0.0619 -1.3474 -0.9084
-0.6958 0.0213 -37.7781
12.4600 -39.6200 0.5769 0.0023
0.3100 0.2573 -0.0930 -1.3329 -0.8986
-0.6999 0.0119 -37.7804
12.4800 -40.1700 0.2465 0.1041
0.2152 0.2023 -0.1238 -1.3162 -0.8881
-0.7038 0.0025 -37.7828
12.5000 -40.8800 -0.3812 0.1608
0.0682 0.1279 -0.1536 -1.2975 -0.8769
-0.7075 -0.0068 -37.7851
12.5200 -40.5300 0.3114 -0.0170 -
0.0787 0.0375 -0.1816 -1.2767 -0.8650
-0.7112 -0.0162 -37.7875
12.5400 -41.6000 -0.2602 -0.2831 -
0.1752 -0.0647 -0.2071 -1.2541 -0.8524
-0.7147 -0.0255 -37.7898
12.5600 -41.5800 -0.0840 -0.3609 -
0.1845 -0.1730 -0.2294 -1.2295 -0.8392
-0.7181 -0.0348 -37.7921
12.5800 -41.1000 0.3064 -0.1025 -
0.1034 -0.2805 -0.2479 -1.2031 -0.8253
-0.7213 -0.0441 -37.7945
12.6000 -41.6100 -0.6839 0.2997
0.0156 -0.3786 -0.2618 -1.1750 -0.8109
-0.7244 -0.0533 -37.7968

12.6200 -39.9500 1.0051 0.2615
0.0975 -0.4589 -0.2706 -1.1453 -0.7958
-0.7274 -0.0626 -37.7992
12.6400 -42.3700 -1.0430 -0.1143
0.0791 -0.5142 -0.2736 -1.1140 -0.7801
-0.7303 -0.0718 -37.8015
12.6600 -40.5500 0.8983 -0.0979 -
0.0596 -0.5402 -0.2704 -1.0812 -0.7639
-0.7330 -0.0810 -37.8039
12.6800 -41.7700 -0.1765 0.0381 -
0.2738 -0.5364 -0.2609 -1.0470 -0.7472
-0.7356 -0.0902 -37.8062
12.7000 -41.7200 -0.0024 -0.0606 -
0.4849 -0.5032 -0.2454 -1.0116 -0.7299
-0.7380 -0.0993 -37.8086
12.7200 -42.0700 -0.2723 -0.1510 -
0.6228 -0.4420 -0.2246 -0.9750 -0.7121
-0.7403 -0.1084 -37.8109
12.7400 -41.8300 -0.2922 -0.0460 -
0.6376 -0.3550 -0.1992 -0.9372 -0.6938
-0.7425 -0.1175 -37.8132
12.7600 -40.5600 0.5122 0.0908 -
0.5315 -0.2468 -0.1699 -0.8984 -0.6750
-0.7446 -0.1266 -37.8156
12.7800 -40.5900 0.1618 0.0373 -
0.3308 -0.1230 -0.1373 -0.8586 -0.6558
-0.7465 -0.1357 -37.8179
12.8000 -41.0400 -0.6981 -0.0407 -
0.0785 0.0098 -0.1024 -0.8178 -0.6361
-0.7484 -0.1447 -37.8203
12.8200 -39.1500 0.6252 0.0739
0.1798 0.1447 -0.0657 -0.7762 -0.6160
-0.7500 -0.1537 -37.8226
12.8400 -39.6300 -0.3797 0.0930
0.4103 0.2746 -0.0281 -0.7338 -0.5955
-0.7516 -0.1627 -37.8250
12.8600 -38.7400 0.4025 -0.0770
0.5937 0.3920 0.0097 -0.6906 -0.5746
-0.7530 -0.1717 -37.8273
12.8800 -39.3300 -0.6379 -0.0586
0.7253 0.4898 0.0469 -0.6467 -0.5534
-0.7543 -0.1806 -37.8297
12.9000 -37.9000 0.6003 0.0735
0.8063 0.5616 0.0828 -0.6023 -0.5317
-0.7555 -0.1895 -37.8320
12.9200 -38.0000 0.2114 0.0689
0.8352 0.6026 0.1167 -0.5572 -0.5098
-0.7566 -0.1984 -37.8343
12.9400 -38.9600 -0.7346 0.0011
0.8140 0.6105 0.1480 -0.5118 -0.4875
-0.7575 -0.2073 -37.8367

12.9600 -37.6900 0.4956 0.0155
0.7472 0.5853 0.1763 -0.4659 -0.4650
-0.7584 -0.2161 -37.8390
12.9800 -38.2400 0.1676 0.0157
0.6413 0.5302 0.2017 -0.4197 -0.4421
-0.7591 -0.2250 -37.8414
13.0000 -38.7900 -0.1826 -0.0458
0.5012 0.4500 0.2243 -0.3733 -0.4190
-0.7596 -0.2337 -37.8437
13.0200 -38.7200 -0.1496 -0.0243
0.3294 0.3507 0.2443 -0.3267 -0.3956
-0.7601 -0.2425 -37.8461
13.0400 -38.7800 0.1363 0.0923
0.1298 0.2385 0.2617 -0.2800 -0.3720
-0.7604 -0.2512 -37.8484
13.0600 -38.9600 0.0291 0.1480 -
0.0908 0.1197 0.2768 -0.2333 -0.3482
-0.7607 -0.2600 -37.8508
13.0800 -39.4300 -0.0072 0.0557 -
0.3188 0.0003 0.2898 -0.1866 -0.3242
-0.7608 -0.2686 -37.8531
13.1000 -39.6500 0.0786 -0.1164 -
0.5306 -0.1138 0.3007 -0.1400 -0.3000
-0.7608 -0.2773 -37.8554
13.1200 -40.5200 -0.4053 -0.1617 -
0.7025 -0.2169 0.3099 -0.0936 -0.2756
-0.7606 -0.2859 -37.8578
13.1400 -40.1900 -0.0789 -0.0376 -
0.8151 -0.3038 0.3174 -0.0475 -0.2511
-0.7604 -0.2945 -37.8601
13.1600 -39.4800 0.4596 0.0447 -
0.8555 -0.3694 0.3235 -0.0016 -0.2265
-0.7601 -0.3031 -37.8625
13.1800 -39.9000 0.2266 -0.0395 -
0.8217 -0.4106 0.3284 0.0440 -0.2017
-0.7596 -0.3117 -37.8648
13.2000 -40.5800 -0.6537 -0.1163 -
0.7262 -0.4265 0.3322 0.0892 -0.1769
-0.7590 -0.3202 -37.8672
13.2200 -39.1100 0.6021 -0.0116 -
0.5867 -0.4185 0.3351 0.1340 -0.1519
-0.7583 -0.3287 -37.8695
13.2400 -39.4000 -0.0779 0.1301 -
0.4254 -0.3895 0.3369 0.1785 -0.1270
-0.7575 -0.3372 -37.8719
13.2600 -39.4800 -0.4709 0.0940 -
0.2633 -0.3428 0.3376 0.2225 -0.1019
-0.7566 -0.3456 -37.8742
13.2800 -38.3400 0.6466 -0.1045 -
0.1165 -0.2818 0.3368 0.2660 -0.0769
-0.7556 -0.3540 -37.8766

13.3000 -39.4800 -0.6987 -0.1623
0.0042 -0.2098 0.3344 0.3091 -0.0518
-0.7545 -0.3624 -37.8789
13.3200 -38.1600 0.1729 0.0636
0.0945 -0.1304 0.3302 0.3517 -0.0268
-0.7532 -0.3708 -37.8812
13.3400 -37.4100 0.4633 0.2602
0.1496 -0.0471 0.3240 0.3938 -0.0018
-0.7519 -0.3791 -37.8836
13.3600 -38.1700 -0.3629 0.1368
0.1696 0.0357 0.3157 0.4354 0.0232
-0.7504 -0.3874 -37.8859
13.3800 -37.8400 0.2106 -0.1140
0.1724 0.1132 0.3051 0.4766 0.0481
-0.7489 -0.3957 -37.8883
13.4000 -38.3200 -0.4615 -0.1569
0.1798 0.1796 0.2921 0.5171 0.0729
-0.7472 -0.4039 -37.8906
13.4200 -37.2600 0.5229 -0.1640
0.2151 0.2302 0.2765 0.5572 0.0977
-0.7454 -0.4121 -37.8930
13.4400 -37.9600 -0.0508 -0.2755
0.2884 0.2619 0.2583 0.5967 0.1223
-0.7435 -0.4203 -37.8953
13.4600 -38.0800 -0.3912 -0.2845
0.3792 0.2737 0.2373 0.6357 0.1468
-0.7416 -0.4285 -37.8977
13.4800 -37.5200 -0.2242 -0.0012
0.4512 0.2663 0.2134 0.6741 0.1711
-0.7395 -0.4366 -37.9000
13.5000 -36.3100 0.5610 0.3925
0.4678 0.2412 0.1865 0.7120 0.1953
-0.7373 -0.4447 -37.9023
13.5200 -37.0800 -0.3213 0.5824
0.3987 0.2009 0.1564 0.7493 0.2192
-0.7350 -0.4528 -37.9047
13.5400 -37.2100 -0.1449 0.5377
0.2294 0.1504 0.1231 0.7860 0.2430
-0.7326 -0.4609 -37.9070
13.5600 -37.6200 -0.0395 0.2865 -
0.0093 0.0963 0.0867 0.8221 0.2666
-0.7301 -0.4689 -37.9094
13.5800 -37.6600 0.6884 -0.2456 -
0.2485 0.0458 0.0470 0.8576 0.2899
-0.7275 -0.4769 -37.9117
13.6000 -39.6500 -0.6475 -0.7145 -
0.4153 0.0062 0.0042 0.8924 0.3129
-0.7248 -0.4849 -37.9141
13.6200 -39.1400 -0.2403 -0.6065 -
0.4527 -0.0158 -0.0418 0.9266 0.3357
-0.7220 -0.4928 -37.9164

13.6400	-37.7200	0.5835	-0.0600	-
0.3684	-0.0168	-0.0906	0.9600	0.3582
	-0.7191	-0.5007	-37.9188	
13.6600	-37.8300	-0.0501	0.3452	-
0.2117	0.0025	-0.1417	0.9926	0.3803
	-0.7162	-0.5086	-37.9211	
13.6800	-37.4000	0.3623	0.2566	-
0.0396	0.0394	-0.1943	1.0244	0.4022
	-0.7131	-0.5164	-37.9235	
13.7000	-38.1800	-0.4640	0.0540	
0.0939	0.0904	-0.2472	1.0554	0.4237
	-0.7099	-0.5243	-37.9258	
13.7200	-37.4700	0.0875	0.0682	
0.1500	0.1506	-0.2993	1.0854	0.4448
	-0.7066	-0.5321	-37.9281	
13.7400	-37.1600	0.3271	0.1517	
0.1269	0.2132	-0.3494	1.1145	0.4656
	-0.7032	-0.5398	-37.9305	
13.7600	-37.6200	-0.0522	0.0901	
0.0544	0.2696	-0.3964	1.1425	0.4860
	-0.6998	-0.5476	-37.9328	
13.7800	-37.8800	-0.1658	-0.0757	-
0.0245	0.3103	-0.4391	1.1696	0.5059
	-0.6962	-0.5553	-37.9352	
13.8000	-37.8800	-0.0680	-0.1968	-
0.0667	0.3264	-0.4763	1.1955	0.5255
	-0.6926	-0.5629	-37.9375	
13.8200	-37.7100	0.1099	-0.1872	-
0.0398	0.3112	-0.5073	1.2202	0.5446
	-0.6888	-0.5706	-37.9399	
13.8400	-38.1700	-0.4104	-0.0719	
0.0600	0.2625	-0.5314	1.2438	0.5632
	-0.6850	-0.5782	-37.9422	
13.8600	-36.9100	0.7263	-0.0490	
0.1990	0.1820	-0.5484	1.2661	0.5814
	-0.6810	-0.5858	-37.9446	
13.8800	-38.3900	-0.7649	-0.0745	
0.3180	0.0738	-0.5583	1.2871	0.5990
	-0.6770	-0.5934	-37.9469	
13.9000	-36.9000	0.5063	0.1742	
0.3622	-0.0565	-0.5611	1.3069	0.6162
	-0.6729	-0.6009	-37.9492	
13.9200	-37.2700	-0.0554	0.4770	
0.3086	-0.2019	-0.5569	1.3254	0.6328
	-0.6687	-0.6084	-37.9516	
13.9400	-37.4500	0.0712	0.5128	
0.1624	-0.3535	-0.5457	1.3425	0.6489
	-0.6644	-0.6159	-37.9539	
13.9600	-38.1200	-0.0516	0.2493	-
0.0424	-0.5017	-0.5277	1.3583	0.6645
	-0.6600	-0.6234	-37.9563	

13.9800	-38.6100	0.2619	-0.2274	–
0.2524	-0.6366	-0.5030	1.3727	0.6795
	-0.6555	-0.6308	-37.9586	
14.0000	-39.9700	-0.4945	-0.6563	–
0.4103	-0.7483	-0.4715	1.3856	0.6939
	-0.6510	-0.6382	-37.9610	
14.0200	-39.2000	0.4970	-0.7828	–
0.4666	-0.8275	-0.4334	1.3971	0.7077
	-0.6463	-0.6455	-37.9633	
14.0400	-39.4200	-0.1751	-0.5420	–
0.4006	-0.8686	-0.3887	1.4070	0.7209
	-0.6416	-0.6529	-37.9656	
14.0600	-39.0500	-0.5241	0.0165	–
0.2465	-0.8725	-0.3379	1.4154	0.7335
	-0.6368	-0.6602	-37.9680	
14.0800	-37.0300	0.6034	0.6193	–
0.0625	-0.8430	-0.2814	1.4221	0.7454
	-0.6319	-0.6675	-37.9703	
14.1000	-37.3500	-0.0857	0.7887	
0.0958	-0.7846	-0.2200	1.4272	0.7567
	-0.6269	-0.6747	-37.9727	
14.1200	-37.1600	0.1070	0.4302	
0.1925	-0.7022	-0.1544	1.4305	0.7674
	-0.6218	-0.6819	-37.9750	
14.1400	-37.5500	0.1592	-0.0775	
0.2234	-0.6008	-0.0855	1.4320	0.7773
	-0.6167	-0.6891	-37.9774	
14.1600	-38.2200	-0.5131	-0.3468	
0.2012	-0.4849	-0.0141	1.4317	0.7866
	-0.6114	-0.6963	-37.9797	
14.1800	-37.1400	0.3026	-0.2831	
0.1540	-0.3594	0.0589	1.4294	0.7953
	-0.6061	-0.7034	-37.9821	
14.2000	-36.9600	0.2522	-0.1477	
0.1207	-0.2291	0.1326	1.4252	0.8032
	-0.6007	-0.7105	-37.9844	
14.2200	-37.2400	-0.3645	-0.1210	
0.1287	-0.0983	0.2062	1.4189	0.8105
	-0.5953	-0.7176	-37.9867	
14.2400	-36.4800	0.2466	-0.1407	
0.1721	0.0306	0.2787	1.4106	0.8170
	-0.5897	-0.7247	-37.9891	
14.2600	-36.6300	-0.1815	-0.0463	
0.2222	0.1573	0.3491	1.4001	0.8229
	-0.5841	-0.7317	-37.9914	
14.2800	-35.9700	-0.0896	0.1781	
0.2428	0.2821	0.4166	1.3873	0.8282
	-0.5784	-0.7387	-37.9938	
14.3000	-35.6300	0.0467	0.3199	
0.2020	0.4055	0.4802	1.3723	0.8327
	-0.5726	-0.7456	-37.9961	

14.3200 -35.4200 0.3600 0.1915
0.0842 0.5271 0.5390 1.3549 0.8366
-0.5668 -0.7526 -37.9985
14.3400 -36.2800 -0.2219 -0.0873 -
0.0911 0.6450 0.5920 1.3351 0.8398
-0.5608 -0.7595 -38.0008
14.3600 -36.5700 -0.3141 -0.2064 -
0.2793 0.7562 0.6382 1.3128 0.8424
-0.5548 -0.7664 -38.0031
14.3800 -35.7800 0.3881 -0.1188 -
0.4279 0.8574 0.6769 1.2880 0.8443
-0.5487 -0.7732 -38.0055
14.4000 -36.3300 -0.1746 -0.0263 -
0.4955 0.9455 0.7070 1.2606 0.8456
-0.5426 -0.7800 -38.0078
14.4200 -35.9200 0.1381 -0.0782 -
0.4620 1.0181 0.7278 1.2305 0.8463
-0.5364 -0.7868 -38.0102
14.4400 -35.7300 0.2751 -0.1331 -
0.3341 1.0730 0.7385 1.1978 0.8463
-0.5301 -0.7936 -38.0125
14.4600 -36.2700 -0.6245 -0.0164 -
0.1424 1.1077 0.7385 1.1626 0.8458
-0.5238 -0.8003 -38.0148
14.4800 -34.6900 0.5852 0.2143
0.0709 1.1198 0.7275 1.1249 0.8446
-0.5173 -0.8070 -38.0172
14.5000 -35.0100 0.0874 0.2245
0.2686 1.1062 0.7057 1.0849 0.8429
-0.5109 -0.8137 -38.0195
14.5200 -35.6700 -0.4281 0.0055
0.4299 1.0632 0.6735 1.0426 0.8406
-0.5043 -0.8203 -38.0219
14.5400 -35.0600 0.3066 -0.1633
0.5488 0.9879 0.6317 0.9983 0.8377
-0.4977 -0.8270 -38.0242
14.5600 -35.8600 -0.3501 -0.1050
0.6222 0.8786 0.5814 0.9520 0.8343
-0.4910 -0.8336 -38.0266
14.5800 -35.2500 0.5755 -0.0559
0.6461 0.7371 0.5237 0.9040 0.8304
-0.4843 -0.8401 -38.0289
14.6000 -36.3400 -0.2424 -0.0251
0.6031 0.5676 0.4597 0.8544 0.8259
-0.4775 -0.8467 -38.0312
14.6200 -36.9300 -0.6342 0.1389
0.4775 0.3765 0.3906 0.8033 0.8209
-0.4706 -0.8532 -38.0336
14.6400 -35.8100 0.7833 0.3805
0.2727 0.1719 0.3176 0.7509 0.8154
-0.4637 -0.8596 -38.0359

14.6600	-37.5800	-0.2672	0.3827	
0.0121	-0.0376	0.2418	0.6973	0.8095
	-0.4568	-0.8661	-38.0383	
14.6800	-38.6000	-0.4554	0.1107	-
0.2643	-0.2430	0.1642	0.6428	0.8030
	-0.4498	-0.8725	-38.0406	
14.7000	-38.5600	0.5201	-0.2898	-
0.5089	-0.4354	0.0861	0.5873	0.7960
	-0.4427	-0.8789	-38.0429	
14.7200	-39.8900	0.1126	-0.5944	-
0.6727	-0.6059	0.0086	0.5312	0.7886
	-0.4356	-0.8853	-38.0453	
14.7400	-40.5000	-0.2244	-0.6384	-
0.7181	-0.7461	-0.0671	0.4745	0.7808
	-0.4284	-0.8916	-38.0476	
14.7600	-40.4600	-0.4807	-0.2755	-
0.6395	-0.8499	-0.1400	0.4174	0.7725
	-0.4211	-0.8979	-38.0500	
14.7800	-39.1300	0.4037	0.2992	-
0.4796	-0.9149	-0.2088	0.3600	0.7637
	-0.4139	-0.9042	-38.0523	
14.8000	-38.7300	0.4542	0.6348	-
0.2957	-0.9410	-0.2728	0.3025	0.7546
	-0.4065	-0.9105	-38.0546	
14.8200	-39.9600	-0.6818	0.5457	-
0.1447	-0.9297	-0.3310	0.2450	0.7450
	-0.3992	-0.9167	-38.0570	
14.8400	-39.3300	0.3288	0.2483	-
0.0584	-0.8850	-0.3827	0.1878	0.7351
	-0.3918	-0.9229	-38.0593	
14.8600	-39.5500	0.3126	-0.1359	-
0.0094	-0.8142	-0.4275	0.1309	0.7247
	-0.3843	-0.9291	-38.0616	
14.8800	-39.7000	0.6141	-0.6193	
0.0556	-0.7267	-0.4647	0.0744	0.7140
	-0.3768	-0.9352	-38.0640	
14.9000	-41.2800	-1.0665	-0.5335	
0.1690	-0.6314	-0.4942	0.0186	0.7029
	-0.3692	-0.9414	-38.0663	
14.9200	-38.1500	1.0418	0.2386	
0.3268	-0.5356	-0.5155	-0.0364	0.6915
	-0.3617	-0.9474	-38.0687	
14.9400	-39.4300	-0.5159	0.4543	
0.4718	-0.4448	-0.5286	-0.0905	0.6797
	-0.3540	-0.9535	-38.0710	
14.9600	-39.1500	-0.0264	0.0955	
0.5376	-0.3632	-0.5332	-0.1436	0.6676
	-0.3464	-0.9596	-38.0733	
14.9800	-39.7600	-0.6464	0.0618	
0.4889	-0.2930	-0.5294	-0.1956	0.6551
	-0.3387	-0.9656	-38.0757	

15.0000 -38.5500 0.8157 0.0843
0.3486 -0.2338 -0.5177 -0.2464 0.6424
-0.3309 -0.9715 -38.0780
15.0200 -40.5700 -0.8269 -0.1377
0.1628 -0.1837 -0.4988 -0.2958 0.6293
-0.3232 -0.9775 -38.0803
15.0400 -39.6200 0.3926 -0.2453 -
0.0256 -0.1396 -0.4737 -0.3439 0.6160
-0.3154 -0.9834 -38.0827
15.0600 -39.5100 0.5091 -0.1531 -
0.1828 -0.0982 -0.4434 -0.3906 0.6024
-0.3075 -0.9893 -38.0850
15.0800 -40.5000 -0.6586 0.0360 -
0.2896 -0.0562 -0.4089 -0.4357 0.5885
-0.2997 -0.9952 -38.0874
15.1000 -39.7400 0.0779 0.1653 -
0.3388 -0.0111 -0.3712 -0.4792 0.5744
-0.2918 -1.0010 -38.0897
15.1200 -39.3100 0.5158 0.1424 -
0.3265 0.0389 -0.3313 -0.5211 0.5600
-0.2838 -1.0069 -38.0920
15.1400 -40.1800 -0.3162 -0.0228 -
0.2544 0.0941 -0.2902 -0.5613 0.5454
-0.2759 -1.0127 -38.0944
15.1600 -40.1600 -0.2917 -0.1730 -
0.1356 0.1534 -0.2486 -0.5997 0.5305
-0.2679 -1.0184 -38.0967
15.1800 -39.4100 0.3992 -0.1873
0.0039 0.2145 -0.2072 -0.6363 0.5155
-0.2599 -1.0242 -38.0990
15.2000 -39.3200 0.0151 -0.0752
0.1309 0.2745 -0.1663 -0.6710 0.5002
-0.2519 -1.0299 -38.1014
15.2200 -39.5400 -0.3834 0.1003
0.2151 0.3304 -0.1265 -0.7037 0.4848
-0.2438 -1.0356 -38.1037
15.2400 -38.5800 0.3092 0.2580
0.2356 0.3790 -0.0881 -0.7345 0.4692
-0.2358 -1.0412 -38.1060
15.2600 -38.5700 0.2300 0.2473
0.1890 0.4169 -0.0515 -0.7633 0.4534
-0.2277 -1.0469 -38.1084
15.2800 -39.6100 -0.3794 0.0199
0.1006 0.4414 -0.0171 -0.7900 0.4375
-0.2196 -1.0525 -38.1107
15.3000 -39.1200 0.3332 -0.2230
0.0089 0.4497 0.0149 -0.8147 0.4214
-0.2114 -1.0580 -38.1131
15.3200 -40.0100 -0.3574 -0.2622 -
0.0495 0.4399 0.0440 -0.8373 0.4052
-0.2033 -1.0636 -38.1154

15.3400 -39.2200 0.2552 -0.0641 -
0.0569 0.4109 0.0703 -0.8578 0.3889
-0.1951 -1.0691 -38.1177
15.3600 -39.4900 -0.1536 0.2187 -
0.0207 0.3643 0.0936 -0.8763 0.3725
-0.1870 -1.0746 -38.1201
15.3800 -39.2700 -0.0676 0.3299
0.0428 0.3033 0.1139 -0.8927 0.3561
-0.1788 -1.0801 -38.1224
15.4000 -39.0300 0.3351 0.1296
0.1119 0.2320 0.1313 -0.9071 0.3395
-0.1706 -1.0855 -38.1247
15.4200 -40.1500 -0.3411 -0.2728
0.1604 0.1549 0.1460 -0.9195 0.3229
-0.1624 -1.0910 -38.1271
15.4400 -39.7500 0.2943 -0.4914
0.1630 0.0766 0.1581 -0.9299 0.3062
-0.1542 -1.0964 -38.1294
15.4600 -40.4700 -0.4783 -0.2662
0.1053 0.0014 0.1676 -0.9383 0.2895
-0.1459 -1.1017 -38.1317
15.4800 -39.4000 0.3599 0.2489 -
0.0066 -0.0659 0.1747 -0.9449 0.2728
-0.1377 -1.1071 -38.1341
15.5000 -39.1700 0.3295 0.6560 -
0.1469 -0.1214 0.1795 -0.9496 0.2561
-0.1295 -1.1124 -38.1364
15.5200 -40.5800 -0.7113 0.5296 -
0.2811 -0.1615 0.1821 -0.9525 0.2394
-0.1212 -1.1177 -38.1387
15.5400 -39.8100 0.8925 -0.2241 -
0.3681 -0.1834 0.1825 -0.9536 0.2227
-0.1130 -1.1229 -38.1410
15.5600 -42.2000 -0.9029 -0.8263 -
0.3623 -0.1853 0.1806 -0.9530 0.2060
-0.1048 -1.1282 -38.1434
15.5800 -40.2000 0.7977 -0.5647 -
0.2325 -0.1684 0.1766 -0.9507 0.1894
-0.0965 -1.1334 -38.1457
15.6000 -40.7000 -0.6547 0.0411 -
0.0155 -0.1374 0.1704 -0.9467 0.1729
-0.0883 -1.1386 -38.1480
15.6200 -39.0200 0.3682 0.4938
0.2120 -0.0974 0.1621 -0.9411 0.1564
-0.0800 -1.1437 -38.1504
15.6400 -39.0500 0.0224 0.6523
0.3665 -0.0542 0.1516 -0.9340 0.1400
-0.0718 -1.1489 -38.1527
15.6600 -38.7000 0.4868 0.4888
0.3802 -0.0131 0.1393 -0.9254 0.1236
-0.0636 -1.1540 -38.1550

15.6800 -40.5900 -1.2452 0.4269
0.2454 0.0233 0.1251 -0.9153 0.1074
-0.0553 -1.1591 -38.1574
15.7000 -38.4900 1.1396 0.2947
0.0264 0.0549 0.1090 -0.9040 0.0913
-0.0471 -1.1641 -38.1597
15.7200 -39.1200 1.3160 -0.2461 -
0.1800 0.0826 0.0911 -0.8913 0.0754
-0.0389 -1.1691 -38.1620
15.7400 -42.5200 -1.5071 -0.7802 -
0.3028 0.1073 0.0712 -0.8774 0.0595
-0.0307 -1.1741 -38.1644
15.7600 -39.6100 1.1574 -0.4633 -
0.3005 0.1293 0.0493 -0.8624 0.0439
-0.0225 -1.1791 -38.1667
15.7800 -40.0900 -0.2212 0.2884 -
0.2053 0.1482 0.0257 -0.8463 0.0284
-0.0143 -1.1841 -38.1690
15.8000 -39.7700 -0.2437 0.5322 -
0.0736 0.1638 0.0003 -0.8292 0.0130
-0.0061 -1.1890 -38.1713
15.8200 -39.5700 0.3472 0.2036
0.0490 0.1760 -0.0262 -0.8112 -0.0021
0.0020 -1.1939 -38.1737
15.8400 -40.5200 -0.4003 -0.1915
0.1452 0.1829 -0.0535 -0.7923 -0.0171
0.0102 -1.1988 -38.1760
15.8600 -39.7600 0.3971 -0.2519
0.2139 0.1804 -0.0808 -0.7726 -0.0318
0.0183 -1.2037 -38.1783
15.8800 -40.3800 -0.4861 -0.0851
0.2723 0.1644 -0.1076 -0.7523 -0.0464
0.0264 -1.2085 -38.1806
15.9000 -39.3000 0.4971 0.0428
0.3327 0.1319 -0.1335 -0.7314 -0.0606
0.0345 -1.2133 -38.1830
15.9200 -40.3400 -0.5528 0.0262
0.3805 0.0832 -0.1577 -0.7100 -0.0747
0.0426 -1.2181 -38.1853
15.9400 -39.4200 0.3819 0.0950
0.3747 0.0215 -0.1798 -0.6882 -0.0885
0.0506 -1.2228 -38.1876
15.9600 -40.2500 -0.3359 0.2310
0.2899 -0.0492 -0.1992 -0.6661 -0.1020
0.0586 -1.2275 -38.1900
15.9800 -39.6900 0.6239 0.0656
0.1294 -0.1238 -0.2152 -0.6438 -0.1152
0.0666 -1.2322 -38.1923
16.0000 -41.5000 -0.5973 -0.2707 -
0.0634 -0.1962 -0.2273 -0.6213 -0.1282
0.0746 -1.2369 -38.1946

16.0200 -41.1100 -0.1021 -0.2767 -
0.2423 -0.2603 -0.2349 -0.5987 -0.1408
0.0826 -1.2416 -38.1969
16.0400 -40.6600 0.3211 0.0312 -
0.3829 -0.3099 -0.2375 -0.5761 -0.1532
0.0905 -1.2462 -38.1993
16.0600 -40.5800 0.2574 0.2878 -
0.4731 -0.3398 -0.2346 -0.5536 -0.1652
0.0984 -1.2508 -38.2016
16.0800 -41.3900 -0.5510 0.2773 -
0.5063 -0.3460 -0.2260 -0.5312 -0.1769
0.1063 -1.2554 -38.2039
16.1000 -40.5000 0.5959 -0.0214 -
0.4817 -0.3273 -0.2116 -0.5089 -0.1882
0.1142 -1.2599 -38.2062
16.1200 -41.1600 0.1774 -0.3227 -
0.3973 -0.2862 -0.1918 -0.4870 -0.1991
0.1220 -1.2645 -38.2086
16.1400 -41.8200 -0.8653 -0.2996 -
0.2576 -0.2274 -0.1673 -0.4653 -0.2097
0.1298 -1.2690 -38.2109
16.1600 -39.5700 0.7287 0.1257 -
0.0803 -0.1563 -0.1386 -0.4440 -0.2200
0.1375 -1.2735 -38.2132
16.1800 -39.3400 0.3797 0.3744
0.1030 -0.0788 -0.1066 -0.4231 -0.2298
0.1452 -1.2779 -38.2155
16.2000 -39.3300 0.4491 0.0814
0.2612 -0.0005 -0.0720 -0.4027 -0.2392
0.1529 -1.2824 -38.2178
16.2200 -40.4400 -0.7001 -0.2660
0.3831 0.0727 -0.0355 -0.3829 -0.2482
0.1606 -1.2868 -38.2202
16.2400 -39.9200 -0.3897 -0.2217
0.4643 0.1358 0.0022 -0.3636 -0.2568
0.1682 -1.2911 -38.2225
16.2600 -38.4900 0.6266 0.0808
0.5014 0.1848 0.0402 -0.3450 -0.2649
0.1758 -1.2955 -38.2248
16.2800 -38.8900 0.0452 0.2129
0.4915 0.2177 0.0780 -0.3271 -0.2726
0.1833 -1.2998 -38.2271
16.3000 -39.0900 0.0122 0.1095
0.4330 0.2345 0.1148 -0.3098 -0.2799
0.1908 -1.3041 -38.2295
16.3200 -39.4900 -0.3663 0.0470
0.3239 0.2369 0.1503 -0.2934 -0.2867
0.1983 -1.3084 -38.2318
16.3400 -39.4400 -0.1896 0.0487
0.1705 0.2273 0.1839 -0.2778 -0.2930
0.2057 -1.3127 -38.2341

16.3600	-38.7800	0.7744	-0.0157	-
0.0087	0.2089	0.2151	-0.2632	-0.2988
	0.2131	-1.3169	-38.2364	
16.3800	-40.7000	-0.9159	-0.1006	-
0.1872	0.1849	0.2436	-0.2494	-0.3041
	0.2204	-1.3212	-38.2387	
16.4000	-39.1000	0.7672	0.0095	-
0.3396	0.1593	0.2690	-0.2366	-0.3089
	0.2277	-1.3253	-38.2411	
16.4200	-39.5500	0.2741	0.0666	-
0.4443	0.1353	0.2908	-0.2249	-0.3133
	0.2349	-1.3295	-38.2434	
16.4400	-40.3800	-0.2841	-0.0792	-
0.4833	0.1150	0.3089	-0.2142	-0.3171
	0.2421	-1.3337	-38.2457	
16.4600	-40.5900	-0.4249	-0.2365	-
0.4443	0.0996	0.3229	-0.2046	-0.3204
	0.2493	-1.3378	-38.2480	
16.4800	-40.0300	-0.0616	-0.2653	-
0.3253	0.0896	0.3327	-0.1961	-0.3231
	0.2564	-1.3419	-38.2503	
16.5000	-39.2200	0.5797	-0.1927	-
0.1400	0.0845	0.3385	-0.1887	-0.3254
	0.2634	-1.3459	-38.2526	
16.5200	-40.0600	-0.6817	0.0003	
0.0793	0.0831	0.3402	-0.1825	-0.3271
	0.2704	-1.3500	-38.2550	
16.5400	-38.2200	0.6096	0.2753	
0.2786	0.0837	0.3381	-0.1773	-0.3283
	0.2774	-1.3540	-38.2573	
16.5600	-38.7800	-0.1611	0.3999	
0.4061	0.0845	0.3323	-0.1732	-0.3290
	0.2843	-1.3580	-38.2596	
16.5800	-38.9700	-0.3068	0.3084	
0.4300	0.0841	0.3231	-0.1702	-0.3291
	0.2911	-1.3620	-38.2619	
16.6000	-38.5400	0.4178	0.0816	
0.3471	0.0814	0.3108	-0.1682	-0.3288
	0.2979	-1.3660	-38.2642	
16.6200	-39.8100	-0.3345	-0.1133	
0.1837	0.0759	0.2956	-0.1671	-0.3279
	0.3047	-1.3699	-38.2665	
16.6400	-39.6000	0.0845	-0.1361	-
0.0196	0.0675	0.2780	-0.1670	-0.3265
	0.3114	-1.3738	-38.2689	
16.6600	-39.8700	-0.0784	-0.0024	-
0.2191	0.0558	0.2582	-0.1677	-0.3247
	0.3180	-1.3777	-38.2712	
16.6800	-39.6100	0.1522	0.1224	-
0.3754	0.0411	0.2365	-0.1692	-0.3223
	0.3246	-1.3815	-38.2735	

16.7000 -39.7000 0.2319 0.0114 -
0.4500 0.0233 0.2132 -0.1715 -0.3195
0.3311 -1.3854 -38.2758
16.7200 -40.2700 0.1370 -0.3424 -
0.4213 0.0028 0.1888 -0.1745 -0.3162
0.3375 -1.3892 -38.2781
16.7400 -40.5800 -0.2085 -0.5310 -
0.2854 -0.0200 0.1634 -0.1781 -0.3125
0.3439 -1.3930 -38.2804
16.7600 -40.8900 -0.9685 -0.2173 -
0.0618 -0.0445 0.1374 -0.1823 -0.3083
0.3503 -1.3968 -38.2827
16.7800 -38.1500 1.2061 0.3089
0.1879 -0.0696 0.1109 -0.1869 -0.3037
0.3566 -1.4005 -38.2851
16.8000 -39.5100 -0.4888 0.4315
0.3906 -0.0943 0.0842 -0.1920 -0.2987
0.3628 -1.4042 -38.2874
16.8200 -40.2300 -0.9755 0.2217
0.4845 -0.1177 0.0574 -0.1974 -0.2932
0.3690 -1.4079 -38.2897
16.8400 -38.5900 0.9164 -0.0246
0.4425 -0.1388 0.0306 -0.2031 -0.2874
0.3751 -1.4116 -38.2920
16.8600 -40.6100 -0.7687 -0.0471
0.2763 -0.1560 0.0038 -0.2090 -0.2812
0.3811 -1.4153 -38.2943
16.8800 -39.5300 0.3395 0.0646
0.0349 -0.1672 -0.0229 -0.2150 -0.2746
0.3871 -1.4189 -38.2966
16.9000 -40.2300 -0.0513 0.0531 -
0.2074 -0.1703 -0.0495 -0.2211 -0.2677
0.3930 -1.4225 -38.2989
16.9200 -40.3900 0.4193 -0.2392 -
0.3777 -0.1630 -0.0759 -0.2271 -0.2604
0.3989 -1.4261 -38.3012
16.9400 -41.4300 -0.5447 -0.4126 -
0.4261 -0.1436 -0.1019 -0.2330 -0.2528
0.4047 -1.4297 -38.3036
16.9600 -40.0400 0.3574 -0.1270 -
0.3412 -0.1118 -0.1276 -0.2388 -0.2449
0.4104 -1.4332 -38.3059
16.9800 -40.1100 -0.0315 0.2164 -
0.1701 -0.0698 -0.1526 -0.2443 -0.2366
0.4161 -1.4367 -38.3082
17.0000 -39.4600 0.2691 0.2048
0.0239 -0.0214 -0.1770 -0.2494 -0.2281
0.4217 -1.4402 -38.3105
17.0200 -39.6000 0.2989 -0.0076
0.1819 0.0297 -0.2003 -0.2541 -0.2193
0.4272 -1.4437 -38.3128

17.0400 -40.5800 -0.8125 -0.0324
0.2566 0.0794 -0.2225 -0.2584 -0.2102
0.4327 -1.4472 -38.3151
17.0600 -39.0600 0.3305 0.2465
0.2346 0.1239 -0.2433 -0.2621 -0.2009
0.4381 -1.4506 -38.3174
17.0800 -38.6800 0.7083 0.3760
0.1515 0.1598 -0.2626 -0.2653 -0.1913
0.4434 -1.4540 -38.3197
17.1000 -39.6400 0.2028 0.0003
0.0544 0.1842 -0.2802 -0.2678 -0.1815
0.4487 -1.4574 -38.3220
17.1200 -41.1000 -0.8383 -0.4873 -
0.0208 0.1944 -0.2959 -0.2695 -0.1715
0.4539 -1.4608 -38.3243
17.1400 -40.0300 0.4195 -0.5156 -
0.0460 0.1888 -0.3096 -0.2706 -0.1613
0.4591 -1.4641 -38.3266
17.1600 -40.1100 -0.1257 0.0067 -
0.0163 0.1670 -0.3212 -0.2708 -0.1509
0.4641 -1.4675 -38.3289
17.1800 -39.7600 -0.4805 0.5627
0.0417 0.1311 -0.3305 -0.2701 -0.1403
0.4691 -1.4708 -38.3312
17.2000 -38.3100 0.9913 0.5921
0.0957 0.0843 -0.3371 -0.2687 -0.1296
0.4741 -1.4740 -38.3335
17.2200 -40.2100 -0.3144 -0.0113
0.1245 0.0300 -0.3410 -0.2663 -0.1187
0.4789 -1.4773 -38.3358
17.2400 -41.3800 -0.8436 -0.5828
0.1245 -0.0284 -0.3417 -0.2631 -0.1077
0.4837 -1.4805 -38.3382
17.2600 -39.9600 0.6241 -0.5446
0.0952 -0.0873 -0.3391 -0.2589 -0.0966
0.4885 -1.4838 -38.3405
17.2800 -40.3400 -0.2060 -0.0705
0.0392 -0.1431 -0.3329 -0.2539 -0.0854
0.4931 -1.4870 -38.3428
17.3000 -39.8200 0.0340 0.3650 -
0.0357 -0.1919 -0.3230 -0.2480 -0.0740
0.4977 -1.4901 -38.3451
17.3200 -39.7200 -0.0228 0.6085 -
0.1248 -0.2302 -0.3090 -0.2413 -0.0626
0.5022 -1.4933 -38.3474
17.3400 -39.5700 0.3337 0.5114 -
0.2161 -0.2545 -0.2908 -0.2339 -0.0512
0.5067 -1.4964 -38.3497
17.3600 -40.6600 -0.2495 0.0580 -
0.2887 -0.2622 -0.2684 -0.2257 -0.0396
0.5110 -1.4995 -38.3520

17.3800 -40.7100 0.1116 -0.4432 -
0.3199 -0.2520 -0.2419 -0.2168 -0.0281
0.5154 -1.5026 -38.3543
17.4000 -41.0700 -0.1035 -0.6923 -
0.2871 -0.2244 -0.2117 -0.2072 -0.0164
0.5196 -1.5057 -38.3566
17.4200 -40.9500 -0.3092 -0.5602 -
0.1823 -0.1814 -0.1781 -0.1972 -0.0048
0.5237 -1.5088 -38.3589
17.4400 -39.7200 0.2787 -0.1296 -
0.0293 -0.1271 -0.1417 -0.1866 0.0069
0.5278 -1.5118 -38.3612
17.4600 -39.4200 -0.1854 0.3391
0.1320 -0.0662 -0.1032 -0.1755 0.0185
0.5318 -1.5148 -38.3635
17.4800 -38.6400 0.0549 0.6086
0.2583 -0.0036 -0.0632 -0.1641 0.0302
0.5358 -1.5178 -38.3658
17.5000 -38.4500 0.1109 0.5570
0.3148 0.0559 -0.0221 -0.1523 0.0418
0.5396 -1.5208 -38.3681
17.5200 -38.7000 0.1017 0.2093
0.2956 0.1081 0.0193 -0.1403 0.0534
0.5434 -1.5237 -38.3704
17.5400 -39.3600 -0.1567 -0.2008
0.2222 0.1501 0.0604 -0.1280 0.0650
0.5471 -1.5266 -38.3727
17.5600 -39.4900 0.0229 -0.4109
0.1259 0.1800 0.1006 -0.1156 0.0765
0.5508 -1.5295 -38.3750
17.5800 -39.2900 0.0791 -0.4049
0.0402 0.1966 0.1392 -0.1030 0.0880
0.5543 -1.5324 -38.3772
17.6000 -39.0000 0.2765 -0.2649 -
0.0111 0.2002 0.1757 -0.0905 0.0995
0.5578 -1.5353 -38.3795
17.6200 -39.7100 -0.8374 -0.0027 -
0.0268 0.1924 0.2095 -0.0779 0.1108
0.5612 -1.5382 -38.3818
17.6400 -38.0000 0.7778 0.1999 -
0.0170 0.1763 0.2400 -0.0654 0.1221
0.5646 -1.5410 -38.3841
17.6600 -37.6100 1.0368 0.2131
0.0001 0.1554 0.2667 -0.0530 0.1333
0.5678 -1.5438 -38.3864
17.6800 -39.8500 -1.1658 0.1064 -
0.0028 0.1338 0.2892 -0.0408 0.1444
0.5710 -1.5466 -38.3887
17.7000 -39.8400 -1.0646 0.1049 -
0.0467 0.1151 0.3070 -0.0289 0.1553
0.5741 -1.5494 -38.3910

17.7200 -38.1200 0.5924 0.2260 -
0.1251 0.1014 0.3197 -0.0172 0.1662
0.5771 -1.5521 -38.3933
17.7400 -37.3800 1.2707 0.2955 -
0.2128 0.0940 0.3273 -0.0059 0.1769
0.5801 -1.5549 -38.3956
17.7600 -38.9200 -0.0414 0.1533 -
0.2828 0.0929 0.3296 0.0051 0.1875
0.5830 -1.5576 -38.3979
17.7800 -40.3500 -1.1750 -0.1499 -
0.3081 0.0973 0.3269 0.0158 0.1980
0.5858 -1.5603 -38.4002
17.8000 -38.5200 0.9119 -0.4318 -
0.2650 0.1056 0.3194 0.0260 0.2083
0.5885 -1.5630 -38.4025
17.8200 -38.5700 0.6794 -0.3918 -
0.1462 0.1152 0.3076 0.0357 0.2184
0.5911 -1.5656 -38.4048
17.8400 -39.8500 -1.1297 -0.0328
0.0241 0.1227 0.2919 0.0451 0.2284
0.5937 -1.5683 -38.4071
17.8600 -38.0300 0.1465 0.3637
0.1944 0.1250 0.2728 0.0540 0.2382
0.5961 -1.5709 -38.4093
17.8800 -37.0000 1.0527 0.4122
0.3106 0.1189 0.2509 0.0625 0.2478
0.5985 -1.5735 -38.4116
17.9000 -39.3300 -0.9263 -0.0172
0.3446 0.1020 0.2268 0.0706 0.2572
0.6009 -1.5761 -38.4139
17.9200 -38.1000 0.7028 -0.3181
0.3074 0.0737 0.2010 0.0782 0.2664
0.6031 -1.5786 -38.4162
17.9400 -39.3200 -0.5351 -0.1565
0.2246 0.0347 0.1743 0.0854 0.2754
0.6052 -1.5812 -38.4185
17.9600 -38.3800 0.2897 0.0571
0.1292 -0.0130 0.1472 0.0921 0.2842
0.6073 -1.5837 -38.4208
17.9800 -38.7200 0.2178 0.0277
0.0395 -0.0658 0.1203 0.0983 0.2927
0.6093 -1.5862 -38.4231
18.0000 -39.4400 -0.2583 -0.0829 -
0.0560 -0.1187 0.0942 0.1041 0.3011
0.6112 -1.5887 -38.4254
18.0200 -39.4000 -0.1838 0.0846 -
0.1695 -0.1668 0.0693 0.1094 0.3092
0.6130 -1.5912 -38.4276
18.0400 -39.4800 -0.5193 0.4690 -
0.2853 -0.2069 0.0464 0.1143 0.3170
0.6148 -1.5937 -38.4299

18.0600 -38.1400 0.9893 0.3863 -
0.3637 -0.2374 0.0257 0.1187 0.3247
0.6165 -1.5961 -38.4322
18.0800 -40.9200 -0.9816 -0.3598 -
0.3679 -0.2570 0.0076 0.1225 0.3320
0.6180 -1.5986 -38.4345
18.1000 -40.1900 0.0610 -0.7736 -
0.2734 -0.2655 -0.0076 0.1260 0.3392
0.6195 -1.6010 -38.4368
18.1200 -40.1000 -0.3817 -0.3099 -
0.0800 -0.2634 -0.0197 0.1289 0.3460
0.6210 -1.6034 -38.4391
18.1400 -38.0800 0.6308 0.4136
0.1450 -0.2526 -0.0286 0.1314 0.3526
0.6223 -1.6057 -38.4414
18.1600 -37.6100 0.5062 0.7329
0.3192 -0.2352 -0.0343 0.1335 0.3590
0.6235 -1.6081 -38.4436
18.1800 -38.2600 0.0540 0.4979
0.3783 -0.2131 -0.0369 0.1352 0.3651
0.6247 -1.6104 -38.4459
18.2000 -39.0900 -0.2044 0.0098
0.3210 -0.1880 -0.0365 0.1365 0.3710
0.6258 -1.6128 -38.4482
18.2200 -39.1200 0.1930 -0.3607
0.1765 -0.1604 -0.0334 0.1374 0.3765
0.6268 -1.6151 -38.4505
18.2400 -39.5000 -0.0257 -0.3975 -
0.0145 -0.1305 -0.0279 0.1381 0.3818
0.6277 -1.6174 -38.4528
18.2600 -40.3100 -0.8811 -0.1190 -
0.2006 -0.0982 -0.0205 0.1386 0.3869
0.6285 -1.6196 -38.4550
18.2800 -38.8500 0.3252 0.1726 -
0.3253 -0.0634 -0.0118 0.1388 0.3917
0.6293 -1.6219 -38.4573
18.3000 -38.0200 1.0497 0.1756 -
0.3443 -0.0261 -0.0021 0.1390 0.3962
0.6299 -1.6241 -38.4596
18.3200 -39.9700 -0.7071 -0.1303 -
0.2460 0.0132 0.0078 0.1390 0.4004
0.6305 -1.6264 -38.4619
18.3400 -40.0500 -0.8075 -0.3228 -
0.0672 0.0533 0.0174 0.1389 0.4044
0.6310 -1.6286 -38.4641
18.3600 -37.9300 0.8639 -0.1699
0.1379 0.0928 0.0261 0.1389 0.4081
0.6314 -1.6308 -38.4664
18.3800 -38.5800 -0.2332 0.1001
0.3183 0.1300 0.0334 0.1389 0.4115
0.6317 -1.6329 -38.4687

18.4000 -38.2800 -0.2370 0.2316
0.4267 0.1633 0.0387 0.1390 0.4147
0.6319 -1.6351 -38.4710
18.4200 -38.1300 -0.2023 0.2989
0.4264 0.1910 0.0416 0.1392 0.4176
0.6321 -1.6372 -38.4733
18.4400 -37.8000 0.3123 0.2196
0.3156 0.2123 0.0418 0.1397 0.4202
0.6321 -1.6394 -38.4755
18.4600 -38.2800 0.4305 -0.1263
0.1289 0.2269 0.0390 0.1403 0.4225
0.6321 -1.6415 -38.4778
18.4800 -39.8500 -0.6919 -0.4386 -
0.0808 0.2354 0.0333 0.1411 0.4246
0.6320 -1.6436 -38.4801
18.5000 -38.5800 0.5476 -0.2646 -
0.2660 0.2393 0.0245 0.1422 0.4264
0.6318 -1.6457 -38.4823
18.5200 -38.6900 0.2716 0.1261 -
0.3977 0.2402 0.0126 0.1436 0.4279
0.6315 -1.6477 -38.4846
18.5400 -39.0800 -0.2235 0.3582 -
0.4634 0.2398 -0.0020 0.1453 0.4291
0.6312 -1.6498 -38.4869
18.5600 -39.8600 -1.0408 0.3027 -
0.4618 0.2372 -0.0193 0.1472 0.4301
0.6307 -1.6518 -38.4892
18.5800 -37.8600 1.4889 -0.2087 -
0.3810 0.2303 -0.0387 0.1494 0.4308
0.6302 -1.6538 -38.4914
18.6000 -41.1100 -1.5950 -0.5195 -
0.2134 0.2162 -0.0597 0.1518 0.4312
0.6296 -1.6559 -38.4937
18.6200 -37.3900 1.5155 -0.0344
0.0300 0.1921 -0.0816 0.1545 0.4313
0.6288 -1.6578 -38.4960
18.6400 -38.3400 -0.1610 0.3447
0.2908 0.1558 -0.1038 0.1574 0.4312
0.6281 -1.6598 -38.4982
18.6600 -38.8900 -0.6434 0.2511
0.4962 0.1062 -0.1256 0.1605 0.4308
0.6272 -1.6618 -38.5005
18.6800 -39.0100 -0.4740 -0.0086
0.5921 0.0433 -0.1463 0.1638 0.4301
0.6262 -1.6637 -38.5028
18.7000 -38.1800 0.5294 -0.1065
0.5532 -0.0305 -0.1653 0.1673 0.4292
0.6252 -1.6657 -38.5050
18.7200 -39.3100 -0.6135 0.1306
0.3841 -0.1100 -0.1822 0.1709 0.4279
0.6241 -1.6676 -38.5073

18.7400 -38.2400 0.6285 0.2500
0.1277 -0.1881 -0.1962 0.1747 0.4264
0.6229 -1.6695 -38.5096
18.7600 -40.0500 -0.3792 -0.0370 -
0.1548 -0.2573 -0.2071 0.1786 0.4247
0.6216 -1.6714 -38.5118
18.7800 -40.3800 -0.2705 -0.2754 -
0.4044 -0.3102 -0.2141 0.1827 0.4226
0.6202 -1.6732 -38.5141
18.8000 -40.1400 0.2464 -0.2296 -
0.5655 -0.3391 -0.2168 0.1868 0.4203
0.6188 -1.6751 -38.5164
18.8200 -40.0700 0.1205 -0.1677 -
0.5949 -0.3377 -0.2148 0.1909 0.4178
0.6172 -1.6769 -38.5186
18.8400 -40.2700 -0.1092 -0.2807 -
0.4776 -0.3056 -0.2081 0.1950 0.4150
0.6156 -1.6787 -38.5209
18.8600 -39.7100 0.3148 -0.3947 -
0.2500 -0.2481 -0.1969 0.1991 0.4119
0.6140 -1.6806 -38.5232
18.8800 -40.0300 -0.5284 -0.1656
0.0234 -0.1727 -0.1817 0.2032 0.4086
0.6122 -1.6824 -38.5254
18.9000 -38.9700 -0.3326 0.3451
0.2674 -0.0870 -0.1629 0.2071 0.4050
0.6104 -1.6841 -38.5277
18.9200 -37.2600 0.8176 0.6208
0.4124 0.0011 -0.1411 0.2110 0.4012
0.6084 -1.6859 -38.5300
18.9400 -39.2300 -0.7757 0.2332
0.4152 0.0843 -0.1167 0.2146 0.3971
0.6064 -1.6877 -38.5322
18.9600 -38.8000 0.2091 -0.2845
0.3084 0.1565 -0.0903 0.2181 0.3928
0.6044 -1.6894 -38.5345
18.9800 -38.6700 0.3594 -0.3473
0.1482 0.2130 -0.0624 0.2214 0.3883
0.6022 -1.6911 -38.5367
19.0000 -39.5800 -0.7750 -0.0046 -
0.0043 0.2494 -0.0336 0.2244 0.3835
0.6000 -1.6928 -38.5390
19.0200 -37.7900 0.9449 0.1249 -
0.0981 0.2628 -0.0044 0.2271 0.3785
0.5977 -1.6945 -38.5412
19.0400 -39.9200 -0.8339 -0.0980 -
0.1123 0.2522 0.0247 0.2294 0.3733
0.5954 -1.6962 -38.5435
19.0600 -39.4100 -0.4913 -0.1635 -
0.0666 0.2200 0.0532 0.2314 0.3679
0.5929 -1.6979 -38.5458

19.0800 -38.5000 0.2347 0.0223 -
0.0005 0.1708 0.0809 0.2330 0.3622
0.5904 -1.6996 -38.5480
19.1000 -37.8600 0.7371 0.1484
0.0521 0.1108 0.1074 0.2341 0.3563
0.5879 -1.7012 -38.5503
19.1200 -39.3600 -0.6618 0.0824
0.0718 0.0464 0.1324 0.2348 0.3503
0.5852 -1.7028 -38.5525
19.1400 -38.4200 0.4436 0.0082
0.0535 -0.0168 0.1556 0.2349 0.3440
0.5825 -1.7044 -38.5548
19.1600 -39.2800 -0.3892 -0.0030
0.0041 -0.0734 0.1768 0.2345 0.3375
0.5797 -1.7060 -38.5570
19.1800 -38.6200 0.5301 -0.0612 -
0.0610 -0.1193 0.1956 0.2336 0.3308
0.5768 -1.7076 -38.5593
19.2000 -39.7700 -0.7393 0.0340 -
0.1266 -0.1511 0.2119 0.2320 0.3240
0.5739 -1.7092 -38.5615
19.2200 -38.3600 0.8404 0.1257 -
0.1778 -0.1663 0.2255 0.2299 0.3169
0.5709 -1.7108 -38.5638
19.2400 -39.9700 -0.6363 -0.0657 -
0.1988 -0.1639 0.2363 0.2272 0.3097
0.5679 -1.7123 -38.5660
19.2600 -39.7500 -0.2838 -0.1831 -
0.1772 -0.1452 0.2442 0.2238 0.3023
0.5647 -1.7139 -38.5683
19.2800 -38.9800 0.2766 -0.0648 -
0.1091 -0.1130 0.2494 0.2199 0.2947
0.5615 -1.7154 -38.5705
19.3000 -38.7800 0.2999 0.1127
0.0000 -0.0713 0.2519 0.2154 0.2870
0.5583 -1.7169 -38.5728
19.3200 -39.1700 -0.4191 0.1025
0.1386 -0.0249 0.2518 0.2103 0.2791
0.5550 -1.7184 -38.5750
19.3400 -38.3900 0.6257 -0.1574
0.2776 0.0213 0.2490 0.2047 0.2710
0.5516 -1.7199 -38.5773
19.3600 -39.6900 -0.8309 -0.2044
0.3717 0.0624 0.2438 0.1985 0.2629
0.5481 -1.7213 -38.5795
19.3800 -37.4900 0.8925 0.1520
0.3798 0.0948 0.2362 0.1918 0.2545
0.5446 -1.7228 -38.5818
19.4000 -39.0800 -0.7969 0.4187
0.2838 0.1170 0.2262 0.1847 0.2461
0.5411 -1.7242 -38.5840

19.4200 -38.6700 -0.0901 0.3333
0.0918 0.1292 0.2141 0.1771 0.2375
0.5374 -1.7257 -38.5863
19.4400 -38.5300 0.7381 -0.0441 -
0.1467 0.1332 0.1998 0.1690 0.2288
0.5337 -1.7271 -38.5885
19.4600 -40.5500 -0.6662 -0.4356 -
0.3617 0.1307 0.1835 0.1606 0.2200
0.5300 -1.7285 -38.5908
19.4800 -39.7400 0.3101 -0.4354 -
0.4856 0.1235 0.1655 0.1518 0.2111
0.5262 -1.7299 -38.5930
19.5000 -40.0600 -0.2464 -0.0879 -
0.4801 0.1130 0.1459 0.1427 0.2021
0.5223 -1.7312 -38.5952
19.5200 -38.8400 0.6300 0.1295 -
0.3560 0.0997 0.1249 0.1333 0.1930
0.5184 -1.7326 -38.5975
19.5400 -40.0900 -0.6760 0.1702 -
0.1590 0.0836 0.1030 0.1237 0.1839
0.5144 -1.7340 -38.5997
19.5600 -39.1500 -0.0535 0.2134
0.0611 0.0647 0.0804 0.1139 0.1746
0.5104 -1.7353 -38.6020
19.5800 -38.3900 0.6539 0.1649
0.2643 0.0428 0.0575 0.1040 0.1653
0.5063 -1.7366 -38.6042
19.6000 -39.4700 -0.2014 -0.0575
0.4160 0.0175 0.0346 0.0940 0.1560
0.5021 -1.7379 -38.6065
19.6200 -39.7700 -0.4141 -0.2096
0.4846 -0.0104 0.0120 0.0839 0.1466
0.4979 -1.7392 -38.6087
19.6400 -39.4900 -0.1493 -0.0478
0.4474 -0.0398 -0.0100 0.0739 0.1371
0.4936 -1.7405 -38.6109
19.6600 -38.6800 0.5527 0.2254
0.3067 -0.0695 -0.0310 0.0639 0.1277
0.4893 -1.7418 -38.6132
19.6800 -40.1200 -0.5447 0.2236
0.0834 -0.0978 -0.0510 0.0541 0.1181
0.4850 -1.7431 -38.6154
19.7000 -39.4300 0.7993 -0.0999 -
0.1730 -0.1231 -0.0695 0.0443 0.1086
0.4806 -1.7443 -38.6176
19.7200 -41.7700 -1.1963 -0.1835 -
0.3957 -0.1437 -0.0865 0.0348 0.0991
0.4761 -1.7456 -38.6199
19.7400 -39.2100 1.4336 -0.0563 -
0.5224 -0.1582 -0.1018 0.0256 0.0895
0.4716 -1.7468 -38.6221

19.7600 -40.7400 0.0948 -0.0925 -
0.5181 -0.1654 -0.1151 0.0166 0.0799
0.4670 -1.7480 -38.6243
19.7800 -42.0800 -1.5437 -0.1463 -
0.3871 -0.1654 -0.1265 0.0080 0.0704
0.4624 -1.7492 -38.6266
19.8000 -39.4600 0.8404 -0.0117 -
0.1647 -0.1584 -0.1360 -0.0002 0.0608
0.4577 -1.7504 -38.6288
19.8200 -38.9000 1.0710 0.1109
0.0978 -0.1455 -0.1438 -0.0079 0.0513
0.4530 -1.7516 -38.6310
19.8400 -40.7200 -0.8631 0.0369
0.3424 -0.1278 -0.1500 -0.0152 0.0418
0.4482 -1.7528 -38.6333
19.8600 -39.6000 0.1657 -0.0660
0.5072 -0.1066 -0.1548 -0.0220 0.0324
0.4434 -1.7539 -38.6355
19.8800 -39.6400 -0.0293 0.0986
0.5481 -0.0829 -0.1583 -0.0281 0.0229
0.4386 -1.7551 -38.6377
19.9000 -39.5500 -0.1116 0.3459
0.4613 -0.0573 -0.1610 -0.0337 0.0136
0.4337 -1.7562 -38.6400
19.9200 -39.1000 0.4913 0.3026
0.2689 -0.0303 -0.1630 -0.0386 0.0042
0.4287 -1.7573 -38.6422
19.9400 -40.1400 0.1054 -0.0756
0.0183 -0.0016 -0.1645 -0.0429 -0.0050
0.4237 -1.7585 -38.6444
19.9600 -41.3900 -0.5735 -0.4248 -
0.2274 0.0290 -0.1657 -0.0465 -0.0142
0.4187 -1.7596 -38.6466
19.9800 -41.2300 -0.3496 -0.3824 -
0.4081 0.0618 -0.1668 -0.0493 -0.0233
0.4136 -1.7607 -38.6489
20.0000 -40.3100 0.4097 -0.0475 -
0.4877 0.0970 -0.1679 -0.0515 -0.0324
0.4085 -1.7617 -38.6511
20.0200 -40.0000 0.4927 0.1631 -
0.4628 0.1340 -0.1690 -0.0529 -0.0414
0.4034 -1.7628 -38.6533
20.0400 -40.0000 0.3342 0.0262 -
0.3485 0.1716 -0.1702 -0.0535 -0.0502
0.3982 -1.7639 -38.6555
20.0600 -41.1300 -0.6658 -0.1287 -
0.1840 0.2080 -0.1715 -0.0534 -0.0590
0.3930 -1.7649 -38.6578
20.0800 -40.2400 -0.0812 -0.0019 -
0.0127 0.2409 -0.1728 -0.0526 -0.0677
0.3877 -1.7659 -38.6600

20.1000 -39.1100 0.5133 0.2075
0.1416 0.2678 -0.1740 -0.0511 -0.0762
0.3824 -1.7670 -38.6622
20.1200 -40.0000 -0.2979 0.1561
0.2615 0.2857 -0.1750 -0.0489 -0.0847
0.3770 -1.7680 -38.6644
20.1400 -39.3700 0.4726 -0.1295
0.3303 0.2919 -0.1756 -0.0460 -0.0930
0.3717 -1.7690 -38.6666
20.1600 -40.4800 -0.5372 -0.1526
0.3407 0.2840 -0.1756 -0.0426 -0.1012
0.3662 -1.7700 -38.6689
20.1800 -40.1400 -0.4617 0.1415
0.2988 0.2606 -0.1750 -0.0385 -0.1092
0.3608 -1.7709 -38.6711
20.2000 -38.7200 0.8522 0.3354
0.2191 0.2210 -0.1735 -0.0339 -0.1171
0.3553 -1.7719 -38.6733
20.2200 -40.7800 -0.7517 0.0500
0.1224 0.1658 -0.1710 -0.0288 -0.1249
0.3498 -1.7729 -38.6755
20.2400 -40.3100 0.2427 -0.2820
0.0366 0.0967 -0.1673 -0.0233 -0.1325
0.3442 -1.7738 -38.6777
20.2600 -40.6800 0.0803 -0.3118 -
0.0159 0.0166 -0.1622 -0.0173 -0.1399
0.3387 -1.7748 -38.6800
20.2800 -40.6500 0.0359 -0.1363 -
0.0324 -0.0703 -0.1557 -0.0110 -0.1472
0.3330 -1.7757 -38.6822
20.3000 -40.7800 -0.2404 0.1262 -
0.0285 -0.1583 -0.1476 -0.0044 -0.1543
0.3274 -1.7766 -38.6844
20.3200 -40.2200 0.1400 0.3285 -
0.0264 -0.2417 -0.1377 0.0024 -0.1612
0.3217 -1.7775 -38.6866
20.3400 -40.2700 0.2856 0.2675 -
0.0429 -0.3145 -0.1260 0.0095 -0.1679
0.3160 -1.7784 -38.6888
20.3600 -40.8500 0.1131 -0.0713 -
0.0816 -0.3720 -0.1123 0.0166 -0.1745
0.3103 -1.7793 -38.6910
20.3800 -41.9200 -0.6132 -0.3075 -
0.1341 -0.4102 -0.0966 0.0239 -0.1808
0.3045 -1.7802 -38.6932
20.4000 -40.7100 0.5001 -0.1144 -
0.1867 -0.4262 -0.0788 0.0311 -0.1869
0.2988 -1.7810 -38.6954
20.4200 -41.2500 -0.4400 0.2067 -
0.2289 -0.4181 -0.0588 0.0384 -0.1929
0.2929 -1.7819 -38.6977

20.4400 -40.2400 0.5862 0.1866 -
0.2538 -0.3858 -0.0370 0.0455 -0.1986
0.2871 -1.7827 -38.6999
20.4600 -41.2500 -0.2657 -0.0701 -
0.2542 -0.3305 -0.0135 0.0524 -0.2041
0.2813 -1.7836 -38.7021
20.4800 -41.5300 -0.4237 -0.1851 -
0.2260 -0.2552 0.0111 0.0592 -0.2094
0.2754 -1.7844 -38.7043
20.5000 -40.2800 0.4387 -0.0985 -
0.1664 -0.1645 0.0365 0.0657 -0.2144
0.2695 -1.7852 -38.7065
20.5200 -40.3800 0.0667 -0.0268 -
0.0776 -0.0641 0.0621 0.0718 -0.2192
0.2635 -1.7860 -38.7087
20.5400 -40.5400 -0.2879 -0.0071
0.0276 0.0395 0.0876 0.0776 -0.2238
0.2576 -1.7868 -38.7109
20.5600 -39.8000 0.1590 -0.0040
0.1307 0.1400 0.1124 0.0829 -0.2281
0.2516 -1.7876 -38.7131
20.5800 -39.5400 0.2296 0.0366
0.2103 0.2315 0.1362 0.0878 -0.2321
0.2456 -1.7884 -38.7153
20.6000 -40.1800 -0.6479 0.1242
0.2486 0.3088 0.1585 0.0921 -0.2359
0.2396 -1.7892 -38.7175
20.6200 -38.7000 0.7810 0.1147
0.2354 0.3678 0.1789 0.0958 -0.2395
0.2336 -1.7899 -38.7197
20.6400 -39.2500 0.4102 -0.0292
0.1724 0.4053 0.1969 0.0989 -0.2428
0.2276 -1.7907 -38.7219
20.6600 -40.6700 -0.8883 -0.1007
0.0738 0.4203 0.2121 0.1012 -0.2458
0.2215 -1.7914 -38.7241
20.6800 -40.2000 -0.3714 0.0541 -
0.0366 0.4135 0.2242 0.1028 -0.2486
0.2154 -1.7921 -38.7263
20.7000 -38.8700 0.9039 0.1795 -
0.1217 0.3862 0.2332 0.1036 -0.2511
0.2093 -1.7929 -38.7285
20.7200 -40.6900 -0.6097 -0.0068 -
0.1477 0.3402 0.2389 0.1036 -0.2533
0.2032 -1.7936 -38.7307
20.7400 -40.1900 0.0499 -0.2880 -
0.1057 0.2780 0.2415 0.1027 -0.2552
0.1971 -1.7943 -38.7329
20.7600 -40.1900 0.1789 -0.3517 -
0.0072 0.2033 0.2411 0.1010 -0.2569
0.1910 -1.7950 -38.7351

20.7800 -40.3000 -0.2032 -0.1100
0.1156 0.1208 0.2380 0.0983 -0.2583
0.1849 -1.7957 -38.7373
20.8000 -40.3000 -0.5808 0.2950
0.2104 0.0358 0.2322 0.0948 -0.2594
0.1787 -1.7963 -38.7395
20.8200 -38.7600 0.8077 0.5045
0.2281 -0.0464 0.2240 0.0904 -0.2602
0.1725 -1.7970 -38.7417
20.8400 -39.3100 0.6931 0.2657
0.1488 -0.1211 0.2135 0.0851 -0.2608
0.1664 -1.7977 -38.7439
20.8600 -41.8500 -1.0873 -0.1800
0.0072 -0.1847 0.2009 0.0789 -0.2611
0.1602 -1.7983 -38.7461
20.8800 -41.2500 -0.2301 -0.3434 -
0.1439 -0.2341 0.1863 0.0720 -0.2612
0.1540 -1.7989 -38.7483
20.9000 -40.2900 0.8540 -0.1817 -
0.2503 -0.2667 0.1698 0.0642 -0.2610
0.1478 -1.7996 -38.7505
20.9200 -41.6000 -0.6630 0.0564 -
0.2736 -0.2809 0.1518 0.0557 -0.2605
0.1416 -1.8002 -38.7526
20.9400 -40.3900 0.5686 0.0465 -
0.2058 -0.2766 0.1322 0.0465 -0.2598
0.1354 -1.8008 -38.7548
20.9600 -41.2700 -0.3543 -0.1042 -
0.0781 -0.2555 0.1114 0.0366 -0.2588
0.1292 -1.8014 -38.7570
20.9800 -40.4300 0.3455 -0.0856
0.0573 -0.2208 0.0897 0.0261 -0.2576
0.1230 -1.8020 -38.7592
21.0000 -41.1000 -0.7247 0.2168
0.1520 -0.1762 0.0674 0.0150 -0.2561
0.1168 -1.8026 -38.7614
21.0200 -39.3800 0.9158 0.3065
0.1821 -0.1258 0.0448 0.0034 -0.2544
0.1106 -1.8032 -38.7636
21.0400 -41.4000 -0.7373 -0.0654
0.1521 -0.0731 0.0222 -0.0087 -0.2525
0.1043 -1.8038 -38.7658
21.0600 -41.5300 -0.5724 -0.3314
0.0930 -0.0215 -0.0002 -0.0212 -0.2504
0.0981 -1.8044 -38.7680
21.0800 -41.1000 -0.2404 -0.1485
0.0366 0.0259 -0.0222 -0.0341 -0.2480
0.0919 -1.8049 -38.7701
21.1000 -40.2400 0.3312 0.1854 -
0.0071 0.0668 -0.0435 -0.0474 -0.2454
0.0857 -1.8055 -38.7723

21.1200 -39.8200 0.7121 0.2696 -
0.0314 0.0997 -0.0638 -0.0609 -0.2426
0.0795 -1.8060 -38.7745
21.1400 -40.8500 0.0222 -0.0003 -
0.0290 0.1229 -0.0831 -0.0746 -0.2396
0.0733 -1.8065 -38.7767
21.1600 -41.5600 -0.4500 -0.3087 -
0.0051 0.1355 -0.1012 -0.0886 -0.2364
0.0671 -1.8071 -38.7789
21.1800 -41.0700 0.0855 -0.3381
0.0284 0.1374 -0.1178 -0.1026 -0.2331
0.0609 -1.8076 -38.7810
21.2000 -41.0500 -0.1319 -0.0404
0.0562 0.1294 -0.1330 -0.1168 -0.2295
0.0547 -1.8081 -38.7832
21.2200 -40.4900 0.1325 0.3404
0.0684 0.1133 -0.1466 -0.1310 -0.2257
0.0485 -1.8086 -38.7854
21.2400 -40.2100 0.1860 0.5131
0.0631 0.0911 -0.1585 -0.1452 -0.2218
0.0424 -1.8091 -38.7876
21.2600 -40.9300 -0.2071 0.2890
0.0419 0.0651 -0.1688 -0.1593 -0.2176
0.0362 -1.8096 -38.7898
21.2800 -40.8900 0.4313 -0.2679
0.0140 0.0378 -0.1773 -0.1733 -0.2133
0.0300 -1.8101 -38.7919
21.3000 -42.3500 -0.5886 -0.6696 -
0.0102 0.0116 -0.1840 -0.1871 -0.2089
0.0239 -1.8106 -38.7941
21.3200 -41.6200 -0.0024 -0.4736 -
0.0247 -0.0114 -0.1888 -0.2007 -0.2043
0.0178 -1.8110 -38.7963
21.3400 -40.8900 0.3027 0.0932 -
0.0377 -0.0294 -0.1917 -0.2140 -0.1995
0.0117 -1.8115 -38.7984
21.3600 -40.7200 0.0997 0.5529 -
0.0617 -0.0412 -0.1926 -0.2271 -0.1946
0.0055 -1.8119 -38.8006
21.3800 -40.9600 -0.0890 0.6241 -
0.1055 -0.0462 -0.1916 -0.2398 -0.1895
-0.0005 -1.8124 -38.8028
21.4000 -41.0300 0.0414 0.3377 -
0.1635 -0.0450 -0.1885 -0.2521 -0.1843
-0.0066 -1.8128 -38.8050
21.4200 -41.4500 0.0475 -0.0609 -
0.2180 -0.0388 -0.1833 -0.2640 -0.1790
-0.0127 -1.8133 -38.8071
21.4400 -42.3900 -0.6001 -0.3371 -
0.2436 -0.0291 -0.1761 -0.2753 -0.1735
-0.0187 -1.8137 -38.8093

21.4600 -41.3600 0.8202 -0.4918 -
0.2143 -0.0175 -0.1668 -0.2862 -0.1679
-0.0247 -1.8141 -38.8115
21.4800 -42.1200 -0.2222 -0.5083 -
0.1169 -0.0056 -0.1555 -0.2965 -0.1622
-0.0307 -1.8145 -38.8136
21.5000 -42.2400 -0.8506 -0.2242
0.0320 0.0053 -0.1422 -0.3062 -0.1563
-0.0367 -1.8149 -38.8158
21.5200 -39.9800 0.8384 0.3012
0.1969 0.0137 -0.1269 -0.3152 -0.1504
-0.0427 -1.8153 -38.8180
21.5400 -40.4100 -0.0051 0.5551
0.3419 0.0185 -0.1099 -0.3236 -0.1444
-0.0486 -1.8157 -38.8201
21.5600 -40.7100 -0.2604 0.3114
0.4344 0.0191 -0.0911 -0.3312 -0.1382
-0.0545 -1.8161 -38.8223
21.5800 -40.7500 0.1259 -0.1811
0.4552 0.0150 -0.0708 -0.3381 -0.1320
-0.0604 -1.8165 -38.8245
21.6000 -40.9900 0.1902 -0.4642
0.3946 0.0070 -0.0492 -0.3442 -0.1257
-0.0663 -1.8168 -38.8266
21.6200 -41.8200 -0.6663 -0.2448
0.2537 -0.0036 -0.0263 -0.3496 -0.1194
-0.0722 -1.8172 -38.8288
21.6400 -40.1900 0.6705 0.2834
0.0564 -0.0152 -0.0025 -0.3541 -0.1129
-0.0780 -1.8176 -38.8309
21.6600 -41.3000 -0.3831 0.4424 -
0.1606 -0.0261 0.0221 -0.3579 -0.1064
-0.0838 -1.8179 -38.8331
21.6800 -41.2000 0.1783 0.1353 -
0.3590 -0.0346 0.0471 -0.3607 -0.0998
-0.0895 -1.8183 -38.8352
21.7000 -42.2200 -0.2067 -0.2449 -
0.4998 -0.0392 0.0723 -0.3627 -0.0932
-0.0953 -1.8186 -38.8374
21.7200 -42.0100 0.0022 -0.3737 -
0.5471 -0.0386 0.0973 -0.3638 -0.0865
-0.1010 -1.8189 -38.8396
21.7400 -41.9000 -0.0965 -0.2275 -
0.4858 -0.0324 0.1219 -0.3639 -0.0798
-0.1066 -1.8193 -38.8417
21.7600 -41.1800 0.1676 -0.0024 -
0.3275 -0.0217 0.1458 -0.3630 -0.0731
-0.1123 -1.8196 -38.8439
21.7800 -41.1700 -0.0746 0.0953 -
0.1052 -0.0082 0.1687 -0.3612 -0.0663
-0.1179 -1.8199 -38.8460

21.8000 -40.4300 0.3078 0.0756
0.1333 0.0058 0.1903 -0.3584 -0.0595
-0.1235 -1.8202 -38.8482
21.8200 -41.0900 -0.4916 0.1073
0.3371 0.0183 0.2102 -0.3545 -0.0527
-0.1290 -1.8205 -38.8503
21.8400 -40.0200 0.3001 0.2477
0.4645 0.0272 0.2282 -0.3496 -0.0459
-0.1345 -1.8208 -38.8525
21.8600 -39.6700 0.3921 0.3151
0.4932 0.0307 0.2440 -0.3436 -0.0391
-0.1400 -1.8211 -38.8546
21.8800 -40.4100 -0.0183 0.1966
0.4243 0.0281 0.2573 -0.3365 -0.0323
-0.1454 -1.8214 -38.8568
21.9000 -41.2000 -0.5979 0.0802
0.2831 0.0202 0.2680 -0.3283 -0.0255
-0.1508 -1.8217 -38.8589
21.9200 -41.3900 -0.6982 0.1258
0.1055 0.0086 0.2757 -0.3190 -0.0187
-0.1562 -1.8220 -38.8611
21.9400 -40.4500 0.3315 0.1767 -
0.0670 -0.0049 0.2803 -0.3087 -0.0120
-0.1615 -1.8223 -38.8632
21.9600 -39.9600 1.1270 0.0131 -
0.1994 -0.0187 0.2815 -0.2973 -0.0053
-0.1668 -1.8225 -38.8654
21.9800 -41.8500 -0.3468 -0.3583 -
0.2708 -0.0309 0.2792 -0.2848 0.0014
-0.1720 -1.8228 -38.8675
22.0000 -42.7500 -1.0623 -0.5502 -
0.2751 -0.0390 0.2733 -0.2713 0.0081
-0.1772 -1.8230 -38.8696
22.0200 -41.0300 0.3633 -0.2281 -
0.2219 -0.0409 0.2635 -0.2568 0.0147
-0.1823 -1.8233 -38.8718
22.0400 -40.7200 -0.0333 0.3575 -
0.1469 -0.0345 0.2497 -0.2414 0.0213
-0.1874 -1.8235 -38.8739
22.0600 -39.5200 0.8405 0.5404 -
0.0914 -0.0180 0.2321 -0.2251 0.0278
-0.1925 -1.8238 -38.8761
22.0800 -41.0300 -0.2451 0.1830 -
0.0792 0.0093 0.2105 -0.2079 0.0342
-0.1975 -1.8240 -38.8782
22.1000 -41.7800 -0.5488 -0.2247 -
0.1008 0.0449 0.1851 -0.1900 0.0406
-0.2025 -1.8242 -38.8803
22.1200 -41.4200 -0.1905 -0.2920 -
0.1351 0.0847 0.1561 -0.1713 0.0469
-0.2074 -1.8245 -38.8825

22.1400 -40.1500 0.8634 -0.1300 -
0.1556 0.1232 0.1238 -0.1520 0.0531
-0.2123 -1.8247 -38.8846
22.1600 -41.5900 -0.7755 0.0813 -
0.1336 0.1550 0.0885 -0.1321 0.0592
-0.2171 -1.8249 -38.8868
22.1800 -41.1000 -0.5712 0.1754 -
0.0523 0.1755 0.0506 -0.1117 0.0652
-0.2218 -1.8251 -38.8889
22.2000 -39.7800 0.8658 0.0811
0.0824 0.1817 0.0103 -0.0908 0.0712
-0.2266 -1.8253 -38.8910
22.2200 -40.0800 0.6393 -0.1406
0.2353 0.1726 -0.0318 -0.0695 0.0770
-0.2312 -1.8255 -38.8932
22.2400 -41.8200 -1.0584 -0.2550
0.3595 0.1479 -0.0754 -0.0478 0.0827
-0.2358 -1.8257 -38.8953
22.2600 -40.3000 0.2797 -0.0245
0.4176 0.1080 -0.1198 -0.0259 0.0883
-0.2404 -1.8259 -38.8974
22.2800 -39.7800 0.4280 0.3317
0.3873 0.0547 -0.1644 -0.0038 0.0938
-0.2449 -1.8261 -38.8995
22.3000 -39.9100 0.5193 0.4059
0.2586 -0.0079 -0.2086 0.0184 0.0991
-0.2493 -1.8263 -38.9017
22.3200 -40.9000 0.1777 0.1080
0.0543 -0.0734 -0.2516 0.0407 0.1043
-0.2537 -1.8265 -38.9038
22.3400 -42.2700 -0.6940 -0.2538 -
0.1672 -0.1347 -0.2928 0.0630 0.1094
-0.2581 -1.8267 -38.9059
22.3600 -41.3500 0.6280 -0.3524 -
0.3421 -0.1848 -0.3315 0.0852 0.1143
-0.2623 -1.8268 -38.9081
22.3800 -42.3100 -0.4101 -0.1759 -
0.4239 -0.2172 -0.3671 0.1072 0.1191
-0.2666 -1.8270 -38.9102
22.4000 -41.6700 -0.0347 0.0698 -
0.3996 -0.2276 -0.3990 0.1291 0.1237
-0.2707 -1.8272 -38.9123
22.4200 -41.2700 0.3260 0.1601 -
0.2871 -0.2161 -0.4266 0.1507 0.1281
-0.2748 -1.8273 -38.9144
22.4400 -41.7500 -0.2999 0.0552 -
0.1257 -0.1859 -0.4496 0.1720 0.1324
-0.2789 -1.8275 -38.9166
22.4600 -40.8400 0.3320 0.0167
0.0353 -0.1415 -0.4678 0.1929 0.1365
-0.2828 -1.8276 -38.9187

22.4800 -41.5800 -0.5859 0.1489
0.1590 -0.0884 -0.4810 0.2134 0.1404
-0.2867 -1.8278 -38.9208
22.5000 -40.1600 0.6535 0.1341
0.2386 -0.0326 -0.4889 0.2334 0.1441
-0.2906 -1.8279 -38.9229
22.5200 -40.5300 0.5168 -0.1019
0.2797 0.0200 -0.4916 0.2528 0.1476
-0.2944 -1.8281 -38.9250
22.5400 -42.1100 -1.0278 -0.2935
0.2879 0.0644 -0.4890 0.2717 0.1510
-0.2981 -1.8282 -38.9271
22.5600 -39.9300 1.0096 -0.1703
0.2593 0.0974 -0.4810 0.2900 0.1541
-0.3018 -1.8284 -38.9293
22.5800 -41.5800 -0.9140 0.1325
0.1846 0.1180 -0.4677 0.3076 0.1571
-0.3054 -1.8285 -38.9314
22.6000 -39.6400 0.7858 0.4980
0.0639 0.1269 -0.4492 0.3246 0.1598
-0.3089 -1.8286 -38.9335
22.6200 -40.8400 -0.4680 0.5432 -
0.0827 0.1256 -0.4258 0.3408 0.1623
-0.3124 -1.8287 -38.9356
22.6400 -40.4400 0.6407 -0.0493 -
0.2215 0.1159 -0.3978 0.3564 0.1646
-0.3158 -1.8289 -38.9377
22.6600 -41.8300 0.0484 -0.6985 -
0.3146 0.0999 -0.3655 0.3711 0.1667
-0.3192 -1.8290 -38.9398
22.6800 -42.9400 -0.9031 -0.8432 -
0.3339 0.0802 -0.3294 0.3850 0.1686
-0.3224 -1.8291 -38.9419
22.7000 -41.8100 -0.6106 -0.2183 -
0.2780 0.0605 -0.2899 0.3981 0.1702
-0.3256 -1.8292 -38.9440
22.7200 -39.2500 1.0750 0.6280 -
0.1887 0.0449 -0.2475 0.4104 0.1716
-0.3288 -1.8293 -38.9462
22.7400 -39.8400 -0.0110 0.9042 -
0.1157 0.0371 -0.2027 0.4218 0.1727
-0.3319 -1.8294 -38.9483
22.7600 -40.4300 -0.1763 0.5490 -
0.0923 0.0396 -0.1560 0.4322 0.1736
-0.3349 -1.8295 -38.9504
22.7800 -40.9700 -0.3012 -0.0197 -
0.1117 0.0509 -0.1076 0.4418 0.1743
-0.3378 -1.8296 -38.9525
22.8000 -41.0900 0.0987 -0.4559 -
0.1451 0.0674 -0.0577 0.4504 0.1747
-0.3407 -1.8297 -38.9546

22.8200 -41.5400 -0.3821 -0.5345 -
0.1519 0.0851 -0.0067 0.4580 0.1749
-0.3435 -1.8298 -38.9567
22.8400 -40.5000 0.3270 -0.4068 -
0.0780 0.0999 0.0452 0.4645 0.1748
-0.3462 -1.8299 -38.9588
22.8600 -40.6200 0.0753 -0.3954
0.1125 0.1086 0.0978 0.4700 0.1744
-0.3489 -1.8300 -38.9609
22.8800 -40.4100 -0.0779 -0.4963
0.3994 0.1091 0.1507 0.4744 0.1738
-0.3515 -1.8301 -38.9630
22.9000 -40.0300 -0.2051 -0.3833
0.7173 0.0993 0.2038 0.4776 0.1730
-0.3540 -1.8302 -38.9651
22.9200 -39.5300 -0.4141 0.0891
0.9875 0.0772 0.2566 0.4797 0.1718
-0.3565 -1.8302 -38.9672
22.9400 -37.8500 0.5640 0.5980
1.1356 0.0409 0.3088 0.4805 0.1705
-0.3589 -1.8303 -38.9693
22.9600 -38.3400 0.0991 0.6851
1.0978 -0.0107 0.3599 0.4801 0.1688
-0.3612 -1.8304 -38.9714
22.9800 -39.3400 -0.3404 0.3264
0.8606 -0.0758 0.4096 0.4784 0.1669
-0.3634 -1.8305 -38.9735
23.0000 -39.5300 0.3321 -0.2146
0.4859 -0.1493 0.4574 0.4753 0.1648
-0.3656 -1.8305 -38.9756
23.0200 -40.6200 -0.0698 -0.4595
0.0555 -0.2251 0.5031 0.4709 0.1624
-0.3678 -1.8306 -38.9777
23.0400 -41.5900 -0.6852 -0.2325 -
0.3565 -0.2973 0.5462 0.4650 0.1598
-0.3698 -1.8307 -38.9797
23.0600 -40.0400 0.8031 0.1493 -
0.7030 -0.3595 0.5864 0.4577 0.1570
-0.3718 -1.8307 -38.9818
23.0800 -41.9900 -0.7631 0.1315 -
0.9603 -0.4048 0.6234 0.4489 0.1539
-0.3737 -1.8308 -38.9839
23.1000 -40.8300 0.6871 -0.0154 -
1.1465 -0.4263 0.6568 0.4385 0.1506
-0.3755 -1.8309 -38.9860
23.1200 -41.9300 -0.4725 0.1478 -
1.2848 -0.4184 0.6864 0.4267 0.1471
-0.3773 -1.8309 -38.9881
23.1400 -41.6800 -0.2834 0.2839 -
1.3541 -0.3785 0.7118 0.4132 0.1434
-0.3790 -1.8310 -38.9902

23.1600	-40.8000	0.7960	0.0656	-
1.3156	-0.3084	0.7327	0.3983	0.1395
	-0.3806	-1.8310	-38.9923	
23.1800	-42.5300	-0.8045	-0.4126	-
1.1367	-0.2131	0.7486	0.3818	0.1354
	-0.3822	-1.8311	-38.9944	
23.2000	-41.0800	0.3708	-0.5659	-
0.8054	-0.0999	0.7591	0.3638	0.1311
	-0.3837	-1.8311	-38.9964	
23.2200	-40.4600	0.1559	-0.3559	-
0.3477	0.0230	0.7637	0.3443	0.1267
	-0.3851	-1.8312	-38.9985	
23.2400	-39.7700	-0.0575	-0.0434	
0.1718	0.1478	0.7621	0.3234	0.1220
	-0.3865	-1.8312	-39.0006	
23.2600	-39.2100	-0.3383	0.2984	
0.6665	0.2664	0.7537	0.3012	0.1173
	-0.3878	-1.8313	-39.0027	
23.2800	-37.7500	0.3817	0.5382	
1.0503	0.3710	0.7382	0.2778	0.1123
	-0.3890	-1.8313	-39.0048	
23.3000	-37.4500	0.4617	0.4897	
1.2553	0.4536	0.7150	0.2532	0.1072
	-0.3902	-1.8313	-39.0068	
23.3200	-38.5300	-0.3116	0.2073	
1.2603	0.5073	0.6840	0.2276	0.1020
	-0.3912	-1.8314	-39.0089	
23.3400	-39.3400	-0.5485	-0.0023	
1.0891	0.5295	0.6451	0.2010	0.0966
	-0.3923	-1.8314	-39.0110	
23.3600	-39.8900	-0.8659	0.0570	
0.7862	0.5230	0.5985	0.1736	0.0911
	-0.3932	-1.8314	-39.0131	
23.3800	-38.3500	1.1937	0.0840	
0.4136	0.4916	0.5449	0.1456	0.0855
	-0.3941	-1.8315	-39.0151	
23.4000	-40.6700	-0.4636	-0.1599	
0.0386	0.4391	0.4849	0.1169	0.0797
	-0.3949	-1.8315	-39.0172	
23.4200	-41.9600	-0.9808	-0.3661	-
0.2849	0.3694	0.4193	0.0878	0.0739
	-0.3956	-1.8315	-39.0193	
23.4400	-41.5400	-0.2896	-0.2552	-
0.5157	0.2862	0.3489	0.0582	0.0680
	-0.3963	-1.8316	-39.0214	
23.4600	-40.4100	0.8977	-0.0382	-
0.6364	0.1935	0.2745	0.0285	0.0620
	-0.3969	-1.8316	-39.0234	
23.4800	-42.2100	-0.6543	0.0453	-
0.6496	0.0955	0.1970	-0.0014	0.0559
	-0.3975	-1.8316	-39.0255	

23.5000 -41.4900 0.1454 0.0607 -
0.5765 -0.0029 0.1172 -0.0314 0.0497
-0.3980 -1.8316 -39.0276
23.5200 -41.6000 0.1000 0.0801 -
0.4494 -0.0963 0.0359 -0.0613 0.0435
-0.3984 -1.8316 -39.0296
23.5400 -41.6300 0.1944 -0.0298 -
0.3026 -0.1790 -0.0459 -0.0910 0.0372
-0.3987 -1.8317 -39.0317
23.5600 -42.2700 -0.3154 -0.1377 -
0.1685 -0.2457 -0.1273 -0.1203 0.0309
-0.3990 -1.8317 -39.0338
23.5800 -42.0200 -0.1975 0.0894 -
0.0757 -0.2929 -0.2076 -0.1493 0.0245
-0.3992 -1.8317 -39.0358
23.6000 -41.6800 -0.0923 0.5294 -
0.0284 -0.3205 -0.2858 -0.1777 0.0181
-0.3994 -1.8317 -39.0379
23.6200 -40.7000 0.8759 0.6409 -
0.0139 -0.3302 -0.3611 -0.2055 0.0117
-0.3994 -1.8317 -39.0399
23.6400 -42.8900 -0.9618 0.2182 -
0.0130 -0.3250 -0.4328 -0.2326 0.0053
-0.3995 -1.8317 -39.0420
23.6600 -41.9100 0.8445 -0.3812 -
0.0075 -0.3084 -0.5001 -0.2587 -0.0012
-0.3994 -1.8317 -39.0441
23.6800 -43.2900 -0.0563 -0.8901
0.0200 -0.2829 -0.5622 -0.2839 -0.0076
-0.3993 -1.8317 -39.0461
23.7000 -44.0600 -0.6609 -1.0569
0.0769 -0.2504 -0.6185 -0.3080 -0.0141
-0.3991 -1.8317 -39.0482
23.7200 -42.5000 0.3973 -0.5930
0.1539 -0.2125 -0.6684 -0.3309 -0.0205
-0.3989 -1.8317 -39.0502
23.7400 -42.5300 -0.6187 0.4449
0.2210 -0.1709 -0.7116 -0.3525 -0.0269
-0.3986 -1.8317 -39.0523
23.7600 -40.1200 0.9007 1.2307
0.2438 -0.1278 -0.7475 -0.3726 -0.0333
-0.3982 -1.8317 -39.0543
23.7800 -41.7700 -0.6646 1.2367
0.1934 -0.0853 -0.7760 -0.3912 -0.0397
-0.3978 -1.8317 -39.0564
23.8000 -42.4800 -0.7203 0.7283
0.0714 -0.0458 -0.7967 -0.4082 -0.0460
-0.3973 -1.8317 -39.0584
23.8200 -41.7600 0.9230 -0.0102 -
0.0822 -0.0111 -0.8096 -0.4234 -0.0522
-0.3968 -1.8317 -39.0605

23.8400 -43.6300 -0.1337 -0.7413 -
0.2147 0.0174 -0.8147 -0.4367 -0.0584
-0.3961 -1.8317 -39.0625
23.8600 -44.8900 -0.7626 -1.1446 -
0.2744 0.0387 -0.8121 -0.4482 -0.0645
-0.3955 -1.8317 -39.0646
23.8800 -42.9100 0.7323 -0.8541 -
0.2265 0.0520 -0.8020 -0.4577 -0.0706
-0.3947 -1.8317 -39.0666
23.9000 -43.3500 -0.7673 0.0636 -
0.0905 0.0563 -0.7846 -0.4653 -0.0765
-0.3939 -1.8316 -39.0687
23.9200 -41.0200 0.8345 0.6935
0.0833 0.0509 -0.7601 -0.4709 -0.0824
-0.3931 -1.8316 -39.0707
23.9400 -42.4500 -0.6466 0.5219
0.2374 0.0350 -0.7287 -0.4746 -0.0881
-0.3921 -1.8316 -39.0728
23.9600 -42.0000 0.0457 0.2093
0.3090 0.0083 -0.6911 -0.4764 -0.0938
-0.3911 -1.8316 -39.0748
23.9800 -42.0400 0.0528 0.1877
0.2579 -0.0284 -0.6475 -0.4764 -0.0994
-0.3901 -1.8315 -39.0769
24.0000 -41.9300 0.2641 0.2557
0.1066 -0.0709 -0.5986 -0.4747 -0.1049
-0.3890 -1.8315 -39.0789
24.0200 -42.7300 -0.2700 0.2027 -
0.0835 -0.1135 -0.5452 -0.4713 -0.1102
-0.3878 -1.8315 -39.0809
24.0400 -42.5300 0.2835 -0.0107 -
0.2505 -0.1503 -0.4877 -0.4663 -0.1155
-0.3866 -1.8314 -39.0830
24.0600 -43.0500 0.1683 -0.3382 -
0.3410 -0.1757 -0.4271 -0.4597 -0.1206
-0.3853 -1.8314 -39.0850
24.0800 -43.4600 -0.0381 -0.5587 -
0.3234 -0.1851 -0.3638 -0.4517 -0.1257
-0.3839 -1.8313 -39.0870
24.1000 -43.9200 -1.0245 -0.3470 -
0.1960 -0.1766 -0.2987 -0.4422 -0.1307
-0.3825 -1.8313 -39.0891
24.1200 -42.1100 -0.0080 0.2279 -
0.0132 -0.1511 -0.2324 -0.4314 -0.1355
-0.3810 -1.8313 -39.0911
24.1400 -40.1800 1.1909 0.6011
0.1463 -0.1102 -0.1658 -0.4192 -0.1403
-0.3795 -1.8312 -39.0931
24.1600 -42.3000 -0.8814 0.3512
0.2164 -0.0563 -0.0994 -0.4059 -0.1449
-0.3779 -1.8312 -39.0952

24.1800 -42.3400 -0.5672 -0.1443
0.1936 0.0076 -0.0340 -0.3914 -0.1495
-0.3762 -1.8311 -39.0972
24.2000 -41.7800 0.2757 -0.4185
0.1074 0.0763 0.0296 -0.3757 -0.1540
-0.3745 -1.8310 -39.0992
24.2200 -41.4800 0.4084 -0.3417
0.0011 0.1441 0.0908 -0.3591 -0.1584
-0.3727 -1.8310 -39.1013
24.2400 -42.0700 -0.5125 -0.0421 -
0.0770 0.2053 0.1489 -0.3414 -0.1627
-0.3709 -1.8309 -39.1033
24.2600 -40.7800 0.4433 0.2047 -
0.0841 0.2550 0.2031 -0.3229 -0.1669
-0.3690 -1.8309 -39.1053
24.2800 -41.2100 0.0535 0.0941 -
0.0133 0.2903 0.2528 -0.3035 -0.1710
-0.3670 -1.8308 -39.1073
24.3000 -41.0300 0.3150 -0.2571
0.1011 0.3104 0.2975 -0.2834 -0.1750
-0.3650 -1.8307 -39.1093
24.3200 -41.8800 -0.8744 -0.1860
0.2028 0.3170 0.3366 -0.2625 -0.1790
-0.3629 -1.8306 -39.1114
24.3400 -39.5200 0.9190 0.3066
0.2374 0.3127 0.3698 -0.2410 -0.1829
-0.3607 -1.8306 -39.1134
24.3600 -40.6600 -0.2276 0.3982
0.1738 0.3000 0.3967 -0.2189 -0.1867
-0.3585 -1.8305 -39.1154
24.3800 -41.2800 -0.4661 0.1271
0.0270 0.2814 0.4173 -0.1964 -0.1904
-0.3563 -1.8304 -39.1174
24.4000 -41.5000 -0.2517 -0.1078 -
0.1491 0.2593 0.4316 -0.1734 -0.1940
-0.3539 -1.8303 -39.1194
24.4200 -40.7000 0.7945 -0.3104 -
0.2898 0.2356 0.4398 -0.1500 -0.1975
-0.3515 -1.8302 -39.1215
24.4400 -42.1600 -0.4934 -0.3826 -
0.3391 0.2121 0.4423 -0.1265 -0.2010
-0.3491 -1.8301 -39.1235
24.4600 -41.9900 -0.6221 -0.1401 -
0.2850 0.1891 0.4395 -0.1028 -0.2044
-0.3466 -1.8300 -39.1255
24.4800 -40.2700 0.5306 0.2687 -
0.1465 0.1657 0.4322 -0.0791 -0.2077
-0.3440 -1.8299 -39.1275
24.5000 -40.1800 0.5076 0.3451
0.0444 0.1397 0.4210 -0.0554 -0.2110
-0.3414 -1.8298 -39.1295

24.5200 -41.3900 -0.5628 -0.0106
0.2363 0.1091 0.4068 -0.0321 -0.2142
-0.3387 -1.8297 -39.1315
24.5400 -40.5800 0.3870 -0.2783
0.3603 0.0717 0.3902 -0.0090 -0.2173
-0.3359 -1.8296 -39.1335
24.5600 -41.5700 -0.8145 0.0578
0.3647 0.0259 0.3720 0.0136 -0.2204
-0.3331 -1.8295 -39.1355
24.5800 -40.7100 -0.3336 0.5214
0.2529 -0.0280 0.3529 0.0356 -0.2234
-0.3302 -1.8293 -39.1375
24.6000 -39.3900 1.3432 0.5554
0.0584 -0.0880 0.3334 0.0569 -0.2263
-0.3273 -1.8292 -39.1395
24.6200 -41.4500 0.0528 -0.0620 -
0.1600 -0.1513 0.3141 0.0773 -0.2292
-0.3243 -1.8291 -39.1416
24.6400 -43.8300 -1.5072 -0.6774 -
0.3225 -0.2150 0.2955 0.0968 -0.2320
-0.3213 -1.8290 -39.1436
24.6600 -40.9800 1.4324 -0.6084 -
0.3588 -0.2759 0.2779 0.1153 -0.2347
-0.3182 -1.8288 -39.1456
24.6800 -43.1600 -1.3040 -0.1823 -
0.2572 -0.3308 0.2618 0.1325 -0.2374
-0.3150 -1.8287 -39.1476
24.7000 -41.4900 0.1306 0.0189 -
0.0573 -0.3763 0.2476 0.1484 -0.2400
-0.3118 -1.8285 -39.1496
24.7200 -40.1300 1.1073 0.1135
0.1663 -0.4090 0.2356 0.1630 -0.2426
-0.3085 -1.8284 -39.1516
24.7400 -40.4800 0.4349 0.3092
0.3292 -0.4256 0.2263 0.1760 -0.2452
-0.3052 -1.8282 -39.1536
24.7600 -41.9400 -1.4355 0.5864
0.3818 -0.4231 0.2199 0.1874 -0.2476
-0.3018 -1.8281 -39.1555
24.7800 -39.0600 1.4564 0.6634
0.3127 -0.4000 0.2166 0.1972 -0.2501
-0.2983 -1.8279 -39.1575
24.8000 -40.9000 0.2142 0.2087
0.1456 -0.3569 0.2165 0.2053 -0.2525
-0.2948 -1.8278 -39.1595
24.8200 -43.1000 -1.2681 -0.4034 -
0.0682 -0.2960 0.2192 0.2117 -0.2548
-0.2912 -1.8276 -39.1615
24.8400 -42.3800 -0.1206 -0.6788 -
0.2737 -0.2203 0.2246 0.2165 -0.2571
-0.2876 -1.8274 -39.1635

24.8600 -41.2600 0.9203 -0.4967 -
0.4234 -0.1332 0.2321 0.2196 -0.2593
-0.2839 -1.8272 -39.1655
24.8800 -42.0900 -0.5498 0.0330 -
0.4931 -0.0381 0.2415 0.2212 -0.2616
-0.2802 -1.8271 -39.1675
24.9000 -41.4700 -0.4482 0.4511 -
0.4761 0.0613 0.2524 0.2212 -0.2637
-0.2764 -1.8269 -39.1695
24.9200 -40.0400 0.7214 0.4883 -
0.3714 0.1608 0.2643 0.2198 -0.2658
-0.2726 -1.8267 -39.1715
24.9400 -40.2900 0.5283 0.1145 -
0.1917 0.2557 0.2768 0.2171 -0.2679
-0.2687 -1.8265 -39.1735
24.9600 -41.5100 -0.5657 -0.3507
0.0289 0.3412 0.2896 0.2130 -0.2699
-0.2647 -1.8263 -39.1754
24.9800 -41.7500 -0.8795 -0.4405
0.2502 0.4125 0.3022 0.2076 -0.2719
-0.2607 -1.8261 -39.1774
25.0000 -39.2100 1.0091 -0.0952
0.4402 0.4649 0.3142 0.2011 -0.2738
-0.2567 -1.8258 -39.1794
25.0200 -40.1000 -0.3737 0.2510
0.5694 0.4939 0.3250 0.1935 -0.2757
-0.2526 -1.8256 -39.1814
25.0400 -40.0600 -0.4586 0.3623
0.6057 0.4955 0.3344 0.1848 -0.2775
-0.2484 -1.8254 -39.1834
25.0600 -39.4400 0.3995 0.2852
0.5308 0.4688 0.3418 0.1752 -0.2792
-0.2442 -1.8252 -39.1853
25.0800 -40.1900 0.0243 0.1261
0.3537 0.4175 0.3470 0.1647 -0.2809
-0.2400 -1.8249 -39.1873
25.1000 -40.9100 -0.3276 -0.0124
0.1110 0.3479 0.3497 0.1535 -0.2826
-0.2356 -1.8247 -39.1893
25.1200 -40.5900 0.3895 -0.0694 -
0.1413 0.2664 0.3496 0.1415 -0.2842
-0.2313 -1.8244 -39.1913
25.1400 -41.8000 -0.4338 -0.1266 -
0.3426 0.1793 0.3465 0.1289 -0.2857
-0.2269 -1.8242 -39.1932
25.1600 -41.1200 0.5939 -0.3105 -
0.4470 0.0932 0.3401 0.1157 -0.2872
-0.2225 -1.8239 -39.1952
25.1800 -42.7100 -0.7473 -0.4246 -
0.4458 0.0148 0.3301 0.1021 -0.2886
-0.2180 -1.8237 -39.1972

25.2000	-41.2100	0.6056	-0.2131	-
0.3693	-0.0495	0.3163	0.0881	-0.2899
	-0.2134	-1.8234	-39.1992	
25.2200	-40.9400	0.5625	0.1330	-
0.2711	-0.0937	0.2986	0.0738	-0.2912
	-0.2089	-1.8231	-39.2011	
25.2400	-41.0800	0.0275	0.3661	-
0.2002	-0.1126	0.2767	0.0593	-0.2924
	-0.2042	-1.8228	-39.2031	
25.2600	-42.2400	-1.2154	0.4742	-
0.1782	-0.1041	0.2509	0.0446	-0.2935
	-0.1996	-1.8225	-39.2051	
25.2800	-40.8300	0.3535	0.3864	-
0.1974	-0.0705	0.2212	0.0299	-0.2946
	-0.1949	-1.8222	-39.2070	
25.3000	-40.2800	1.3039	0.0475	-
0.2227	-0.0176	0.1881	0.0152	-0.2956
	-0.1901	-1.8219	-39.2090	
25.3200	-43.1900	-1.3029	-0.4193	-
0.2165	0.0475	0.1520	0.0005	-0.2966
	-0.1853	-1.8216	-39.2109	
25.3400	-40.9100	1.1389	-0.5487	-
0.1534	0.1176	0.1136	-0.0140	-0.2975
	-0.1805	-1.8213	-39.2129	
25.3600	-42.2200	-0.6163	-0.2893	-
0.0275	0.1861	0.0732	-0.0283	-0.2983
	-0.1757	-1.8210	-39.2149	
25.3800	-41.5200	-0.4611	0.0862	
0.1377	0.2469	0.0316	-0.0423	-0.2990
	-0.1708	-1.8206	-39.2168	
25.4000	-40.1000	0.4647	0.4125	
0.3046	0.2947	-0.0109	-0.0560	-0.2997
	-0.1658	-1.8203	-39.2188	
25.4200	-40.3100	0.1353	0.4661	
0.4317	0.3243	-0.0535	-0.0693	-0.3003
	-0.1609	-1.8200	-39.2207	
25.4400	-40.4200	0.2626	0.1527	
0.4919	0.3314	-0.0957	-0.0821	-0.3008
	-0.1559	-1.8196	-39.2227	
25.4600	-41.5200	-0.2859	-0.2530	
0.4874	0.3142	-0.1371	-0.0943	-0.3013
	-0.1508	-1.8192	-39.2246	
25.4800	-41.5900	-0.2827	-0.3352	
0.4325	0.2728	-0.1772	-0.1059	-0.3016
	-0.1458	-1.8189	-39.2266	
25.5000	-42.1200	-0.9355	0.0494	
0.3477	0.2095	-0.2153	-0.1168	-0.3020
	-0.1407	-1.8185	-39.2285	
25.5200	-41.4300	-0.3576	0.4293	
0.2538	0.1278	-0.2511	-0.1270	-0.3022
	-0.1355	-1.8181	-39.2305	

25.5400 -40.0600 1.3652 0.3139
0.1687 0.0326 -0.2841 -0.1363 -0.3023
-0.1304 -1.8177 -39.2324
25.5600 -43.5400 -1.2916 -0.3083
0.0931 -0.0705 -0.3136 -0.1447 -0.3024
-0.1252 -1.8173 -39.2344
25.5800 -41.7900 0.7969 -0.4645
0.0170 -0.1747 -0.3394 -0.1521 -0.3024
-0.1200 -1.8169 -39.2363
25.6000 -42.0000 0.3599 -0.0173 -
0.0709 -0.2738 -0.3609 -0.1584 -0.3024
-0.1148 -1.8165 -39.2383
25.6200 -42.4600 -0.3496 0.3944 -
0.1726 -0.3616 -0.3775 -0.1637 -0.3022
-0.1095 -1.8161 -39.2402
25.6400 -42.0200 0.4632 0.2707 -
0.2829 -0.4327 -0.3889 -0.1677 -0.3020
-0.1042 -1.8157 -39.2422
25.6600 -43.4600 -0.5494 -0.0496 -
0.3841 -0.4826 -0.3946 -0.1706 -0.3016
-0.0989 -1.8152 -39.2441
25.6800 -42.7500 0.3956 -0.2043 -
0.4485 -0.5082 -0.3941 -0.1722 -0.3012
-0.0936 -1.8148 -39.2461
25.7000 -42.9200 0.3565 -0.2614 -
0.4511 -0.5077 -0.3872 -0.1727 -0.3007
-0.0883 -1.8143 -39.2480
25.7200 -43.8300 -0.6240 -0.2603 -
0.3798 -0.4821 -0.3741 -0.1719 -0.3001
-0.0829 -1.8139 -39.2499
25.7400 -42.8800 -0.1281 -0.1094 -
0.2401 -0.4351 -0.3551 -0.1701 -0.2994
-0.0776 -1.8134 -39.2519
25.7600 -41.5100 0.6981 0.0929 -
0.0624 -0.3724 -0.3309 -0.1672 -0.2986
-0.0722 -1.8129 -39.2538
25.7800 -42.1600 -0.2368 0.1197
0.1137 -0.2995 -0.3019 -0.1634 -0.2976
-0.0668 -1.8125 -39.2557
25.8000 -42.1200 -0.2792 0.0373
0.2520 -0.2223 -0.2686 -0.1587 -0.2966
-0.0614 -1.8120 -39.2577
25.8200 -41.8500 -0.3326 0.1257
0.3228 -0.1467 -0.2316 -0.1533 -0.2954
-0.0559 -1.8115 -39.2596
25.8400 -40.7900 0.5731 0.2045
0.3202 -0.0775 -0.1916 -0.1472 -0.2941
-0.0505 -1.8109 -39.2615
25.8600 -41.9000 -0.4738 -0.0037
0.2556 -0.0178 -0.1491 -0.1404 -0.2926
-0.0450 -1.8104 -39.2635

25.8800 -41.3300 0.2633 -0.1918
0.1571 0.0303 -0.1048 -0.1332 -0.2910
-0.0396 -1.8099 -39.2654
25.9000 -41.8300 -0.2925 -0.1283
0.0549 0.0658 -0.0594 -0.1255 -0.2893
-0.0341 -1.8094 -39.2673
25.9200 -40.9900 0.4642 -0.0102 -
0.0325 0.0892 -0.0133 -0.1175 -0.2874
-0.0287 -1.8088 -39.2692
25.9400 -41.7500 -0.4463 0.1300 -
0.0999 0.1030 0.0326 -0.1092 -0.2854
-0.0232 -1.8082 -39.2712
25.9600 -41.3400 -0.1608 0.2830 -
0.1407 0.1098 0.0776 -0.1008 -0.2832
-0.0177 -1.8077 -39.2731
25.9800 -40.7100 0.4611 0.2195 -
0.1425 0.1125 0.1211 -0.0922 -0.2808
-0.0123 -1.8071 -39.2750
26.0000 -40.9400 0.5415 -0.1659 -
0.1048 0.1133 0.1623 -0.0836 -0.2783
-0.0068 -1.8065 -39.2769
26.0200 -42.4200 -0.7957 -0.4915 -
0.0373 0.1143 0.2005 -0.0751 -0.2756
-0.0013 -1.8059 -39.2788
26.0400 -40.7300 0.6483 -0.2691
0.0427 0.1169 0.2351 -0.0668 -0.2727
0.0041 -1.8053 -39.2808
26.0600 -40.7300 -0.0670 0.2948
0.1041 0.1219 0.2656 -0.0587 -0.2696
0.0096 -1.8047 -39.2827
26.0800 -40.9000 -0.5905 0.5610
0.1146 0.1298 0.2915 -0.0510 -0.2663
0.0151 -1.8041 -39.2846
26.1000 -39.9800 0.7373 0.1887
0.0615 0.1403 0.3124 -0.0436 -0.2629
0.0205 -1.8034 -39.2865
26.1200 -42.0900 -0.7289 -0.4142 -
0.0169 0.1535 0.3282 -0.0368 -0.2593
0.0260 -1.8028 -39.2884
26.1400 -40.9500 0.4027 -0.4096 -
0.0710 0.1684 0.3385 -0.0305 -0.2554
0.0314 -1.8021 -39.2903
26.1600 -41.2100 -0.4470 0.0971 -
0.0736 0.1837 0.3433 -0.0248 -0.2514
0.0368 -1.8015 -39.2922
26.1800 -39.8600 0.7553 0.2773 -
0.0341 0.1974 0.3426 -0.0198 -0.2471
0.0422 -1.8008 -39.2941
26.2000 -41.3700 -0.7937 0.0956
0.0080 0.2076 0.3365 -0.0155 -0.2426
0.0476 -1.8001 -39.2961

26.2200 -39.9900 0.7433 0.0854
0.0182 0.2122 0.3251 -0.0118 -0.2379
0.0530 -1.7994 -39.2980
26.2400 -41.2200 -0.5964 0.1183
0.0011 0.2100 0.3087 -0.0089 -0.2330
0.0584 -1.7987 -39.2999
26.2600 -40.3500 0.6093 -0.1446 -
0.0128 0.1998 0.2877 -0.0067 -0.2279
0.0637 -1.7980 -39.3018
26.2800 -41.7300 -0.5468 -0.3960
0.0012 0.1799 0.2626 -0.0051 -0.2226
0.0691 -1.7973 -39.3037
26.3000 -40.8300 0.1155 -0.1971
0.0444 0.1499 0.2340 -0.0041 -0.2170
0.0744 -1.7965 -39.3056
26.3200 -40.6700 -0.1575 0.3300
0.0942 0.1105 0.2025 -0.0038 -0.2112
0.0797 -1.7958 -39.3075
26.3400 -39.6600 0.6089 0.6067
0.1228 0.0635 0.1689 -0.0039 -0.2052
0.0850 -1.7950 -39.3094
26.3600 -40.9100 -0.2812 0.3378
0.1107 0.0116 0.1337 -0.0047 -0.1990
0.0902 -1.7943 -39.3113
26.3800 -41.1400 0.1723 -0.2125
0.0686 -0.0416 0.0977 -0.0059 -0.1925
0.0954 -1.7935 -39.3132
26.4000 -41.9800 -0.0899 -0.6582
0.0156 -0.0927 0.0617 -0.0076 -0.1859
0.1006 -1.7927 -39.3151
26.4200 -42.3100 -0.2639 -0.7512 -
0.0290 -0.1378 0.0262 -0.0098 -0.1790
0.1058 -1.7919 -39.3170
26.4400 -42.3200 -0.6171 -0.3561 -
0.0553 -0.1739 -0.0080 -0.0124 -0.1720
0.1110 -1.7911 -39.3188
26.4600 -41.0300 0.0964 0.3756 -
0.0708 -0.1986 -0.0402 -0.0153 -0.1647
0.1161 -1.7903 -39.3207
26.4800 -40.3400 0.3401 0.8879 -
0.0871 -0.2105 -0.0699 -0.0186 -0.1573
0.1212 -1.7894 -39.3226
26.5000 -40.0800 0.8798 0.5838 -
0.1102 -0.2094 -0.0966 -0.0222 -0.1496
0.1263 -1.7886 -39.3245
26.5200 -42.7600 -0.9484 -0.2653 -
0.1284 -0.1961 -0.1201 -0.0261 -0.1417
0.1313 -1.7877 -39.3264
26.5400 -42.2600 -0.0397 -0.7168 -
0.1256 -0.1724 -0.1402 -0.0301 -0.1337
0.1363 -1.7868 -39.3283

26.5600 -42.2600 -0.2247 -0.4972 -
0.0897 -0.1406 -0.1572 -0.0343 -0.1255
0.1413 -1.7859 -39.3302
26.5800 -40.9500 0.6040 -0.1169 -
0.0208 -0.1035 -0.1712 -0.0386 -0.1171
0.1462 -1.7851 -39.3321
26.6000 -40.7000 0.6215 0.1352
0.0738 -0.0639 -0.1823 -0.0428 -0.1085
0.1511 -1.7841 -39.3339
26.6200 -41.9800 -1.0549 0.2837
0.1635 -0.0252 -0.1911 -0.0470 -0.0997
0.1559 -1.7832 -39.3358
26.6400 -40.3900 0.2431 0.4043
0.2131 0.0092 -0.1977 -0.0510 -0.0908
0.1608 -1.7823 -39.3377
26.6600 -39.6900 0.9273 0.3754
0.2044 0.0367 -0.2026 -0.0547 -0.0816
0.1655 -1.7813 -39.3396
26.6800 -40.1700 0.8351 0.0706
0.1363 0.0561 -0.2060 -0.0581 -0.0724
0.1703 -1.7804 -39.3415
26.7000 -41.7500 -0.2850 -0.2973
0.0383 0.0676 -0.2084 -0.0610 -0.0629
0.1750 -1.7794 -39.3433
26.7200 -42.7500 -1.1238 -0.3419 -
0.0521 0.0727 -0.2099 -0.0634 -0.0534
0.1796 -1.7784 -39.3452
26.7400 -40.3400 1.0572 0.0065 -
0.1149 0.0734 -0.2109 -0.0653 -0.0436
0.1842 -1.7774 -39.3471
26.7600 -41.5000 -0.3373 0.1398 -
0.1474 0.0717 -0.2118 -0.0664 -0.0338
0.1888 -1.7764 -39.3489
26.7800 -41.5200 -0.1402 -0.0226 -
0.1517 0.0691 -0.2128 -0.0669 -0.0237
0.1933 -1.7754 -39.3508
26.8000 -41.7900 -0.5318 0.0833 -
0.1335 0.0667 -0.2142 -0.0665 -0.0136
0.1978 -1.7744 -39.3527
26.8200 -40.3600 0.7080 0.1789 -
0.0911 0.0648 -0.2163 -0.0653 -0.0033
0.2022 -1.7733 -39.3546
26.8400 -41.8800 -0.6180 -0.1051 -
0.0263 0.0636 -0.2194 -0.0632 0.0071
0.2066 -1.7723 -39.3564
26.8600 -41.3200 0.0163 -0.2691
0.0448 0.0624 -0.2237 -0.0601 0.0176
0.2109 -1.7712 -39.3583
26.8800 -40.5900 0.3828 -0.0187
0.1067 0.0610 -0.2295 -0.0562 0.0282
0.2151 -1.7701 -39.3601

26.9000 -41.2600 -0.6039 0.3847
0.1509 0.0596 -0.2368 -0.0514 0.0389
0.2193 -1.7690 -39.3620
26.9200 -39.7700 0.8838 0.2086
0.1738 0.0593 -0.2457 -0.0458 0.0497
0.2235 -1.7679 -39.3639
26.9400 -42.0500 -0.6956 -0.4156
0.1718 0.0618 -0.2562 -0.0394 0.0605
0.2276 -1.7668 -39.3657
26.9600 -42.2400 -0.6898 -0.5979
0.1360 0.0685 -0.2680 -0.0323 0.0715
0.2316 -1.7657 -39.3676
26.9800 -40.2600 0.7050 -0.0395
0.0608 0.0798 -0.2809 -0.0246 0.0825
0.2356 -1.7645 -39.3694
27.0000 -40.7200 -0.2884 0.6482 -
0.0482 0.0958 -0.2942 -0.0164 0.0936
0.2395 -1.7634 -39.3713
27.0200 -39.8700 0.7024 0.6461 -
0.1780 0.1154 -0.3073 -0.0077 0.1047
0.2434 -1.7622 -39.3732
27.0400 -41.7700 -0.6165 0.1151 -
0.2983 0.1365 -0.3194 0.0012 0.1158
0.2472 -1.7610 -39.3750
27.0600 -41.2700 0.5298 -0.4478 -
0.3645 0.1567 -0.3299 0.0103 0.1270
0.2510 -1.7598 -39.3769
27.0800 -42.5000 -0.5595 -0.7920 -
0.3289 0.1738 -0.3379 0.0196 0.1382
0.2546 -1.7586 -39.3787
27.1000 -41.5000 0.1003 -0.6501 -
0.1694 0.1851 -0.3427 0.0288 0.1495
0.2583 -1.7573 -39.3806
27.1200 -40.6700 0.0492 -0.0108
0.0796 0.1871 -0.3436 0.0379 0.1607
0.2618 -1.7561 -39.3824
27.1400 -40.3400 -0.5944 0.6799
0.3455 0.1759 -0.3401 0.0469 0.1719
0.2653 -1.7548 -39.3843
27.1600 -38.5700 1.0034 0.6892
0.5504 0.1479 -0.3316 0.0555 0.1831
0.2687 -1.7535 -39.3861
27.1800 -41.1300 -1.0254 0.0659
0.6331 0.0995 -0.3177 0.0638 0.1943
0.2720 -1.7523 -39.3879
27.2000 -40.2200 0.3101 -0.1712
0.5729 0.0296 -0.2982 0.0717 0.2054
0.2753 -1.7510 -39.3898
27.2200 -40.1600 0.2936 0.0435
0.3943 -0.0582 -0.2729 0.0790 0.2165
0.2785 -1.7496 -39.3916

27.2400 -40.2300 0.5647 0.0284
0.1667 -0.1586 -0.2419 0.0858 0.2276
0.2816 -1.7483 -39.3935
27.2600 -41.7200 -0.3400 -0.3128 –
0.0462 -0.2657 -0.2052 0.0919 0.2385
0.2847 -1.7470 -39.3953
27.2800 -41.6700 0.0668 -0.5527 –
0.2105 -0.3737 -0.1630 0.0974 0.2494
0.2877 -1.7456 -39.3971
27.3000 -42.1600 -0.4095 -0.3423 –
0.3142 -0.4761 -0.1154 0.1022 0.2603
0.2906 -1.7442 -39.3990
27.3200 -41.0400 0.3173 0.1423 –
0.3774 -0.5651 -0.0624 0.1063 0.2710
0.2934 -1.7428 -39.4008
27.3400 -40.7400 0.3795 0.4888 –
0.4324 -0.6322 -0.0042 0.1097 0.2816
0.2962 -1.7414 -39.4027
27.3600 -41.8500 -0.8431 0.5208 –
0.5089 -0.6698 0.0588 0.1124 0.2921
0.2988 -1.7400 -39.4045
27.3800 -40.6000 0.7391 0.2185 –
0.6090 -0.6728 0.1264 0.1143 0.3025
0.3014 -1.7386 -39.4063
27.4000 -41.4000 0.3915 -0.2152 –
0.7106 -0.6401 0.1979 0.1154 0.3127
0.3039 -1.7371 -39.4081
27.4200 -42.8200 -0.8469 -0.4787 –
0.7808 -0.5731 0.2726 0.1158 0.3228
0.3063 -1.7356 -39.4100
27.4400 -42.3200 -0.7055 -0.3288 –
0.7884 -0.4749 0.3491 0.1153 0.3327
0.3087 -1.7342 -39.4118
27.4600 -40.0000 0.9384 0.0590 –
0.7234 -0.3507 0.4261 0.1140 0.3424
0.3109 -1.7327 -39.4136
27.4800 -40.0000 0.5328 0.1716 –
0.5936 -0.2062 0.5022 0.1119 0.3520
0.3131 -1.7311 -39.4154
27.5000 -39.7300 0.7742 -0.2565 –
0.4167 -0.0477 0.5760 0.1090 0.3614
0.3152 -1.7296 -39.4173
27.5200 -41.5600 -1.3274 -0.4158 –
0.2186 0.1183 0.6463 0.1052 0.3706
0.3172 -1.7281 -39.4191
27.5400 -38.3900 0.9492 0.1158 –
0.0221 0.2853 0.7116 0.1005 0.3795
0.3191 -1.7265 -39.4209
27.5600 -37.3800 0.9006 0.6582
0.1656 0.4466 0.7706 0.0949 0.3883
0.3209 -1.7249 -39.4227

27.5800	-38.8000	-0.9202	0.7213	
0.3425	0.5957	0.8219	0.0884	0.3968
	0.3226	-1.7233	-39.4245	
27.6000	-38.1100	-0.2074	0.2963	
0.5070	0.7257	0.8641	0.0809	0.4050
	0.3243	-1.7217	-39.4264	
27.6200	-37.7000	0.5719	-0.2897	
0.6544	0.8303	0.8960	0.0726	0.4130
	0.3258	-1.7201	-39.4282	
27.6400	-38.7200	-0.2163	-0.7671	
0.7814	0.9032	0.9160	0.0633	0.4208
	0.3273	-1.7185	-39.4300	
27.6600	-38.4400	0.1117	-0.9254	
0.8805	0.9396	0.9230	0.0530	0.4282
	0.3286	-1.7168	-39.4318	
27.6800	-38.3900	-0.3029	-0.5804	
0.9362	0.9366	0.9160	0.0418	0.4354
	0.3299	-1.7152	-39.4336	
27.7000	-37.5500	-0.1118	0.1537	
0.9261	0.8940	0.8950	0.0298	0.4423
	0.3310	-1.7135	-39.4354	
27.7200	-37.0500	-0.1235	0.8714	
0.8233	0.8146	0.8607	0.0171	0.4489
	0.3321	-1.7118	-39.4372	
27.7400	-36.5100	0.6053	1.0592	
0.6045	0.7040	0.8142	0.0037	0.4552
	0.3330	-1.7100	-39.4390	
27.7600	-38.0700	0.1710	0.4930	
0.2826	0.5695	0.7565	-0.0102	0.4612
	0.3339	-1.7083	-39.4408	
27.7800	-40.2100	-0.5469	-0.3445	-
0.0813	0.4185	0.6888	-0.0245	0.4669
	0.3346	-1.7066	-39.4427	
27.8000	-40.9600	-0.2068	-0.8076	-
0.4144	0.2586	0.6122	-0.0390	0.4722
	0.3353	-1.7048	-39.4445	
27.8200	-41.0200	0.1595	-0.7795	-
0.6466	0.0973	0.5277	-0.0537	0.4772
	0.3358	-1.7030	-39.4463	
27.8400	-40.7500	0.4793	-0.5423	-
0.7291	-0.0581	0.4364	-0.0684	0.4819
	0.3363	-1.7012	-39.4481	
27.8600	-41.2600	-0.1238	-0.2375	-
0.6618	-0.2016	0.3394	-0.0829	0.4863
	0.3366	-1.6994	-39.4499	
27.8800	-41.5400	-0.7120	0.1371	-
0.4979	-0.3285	0.2379	-0.0973	0.4903
	0.3369	-1.6976	-39.4517	
27.9000	-40.7400	-0.2508	0.4985	-
0.2991	-0.4347	0.1327	-0.1112	0.4939
	0.3370	-1.6957	-39.4534	

27.9200 -39.1700 1.2519 0.5983 -
0.1189 -0.5158 0.0250 -0.1247 0.4972
0.3370 -1.6939 -39.4552
27.9400 -40.3700 0.4133 0.3297 -
0.0033 -0.5685 -0.0842 -0.1376 0.5002
0.3369 -1.6920 -39.4570
27.9600 -42.6400 -1.4621 0.0200
0.0311 -0.5915 -0.1939 -0.1497 0.5027
0.3368 -1.6901 -39.4588
27.9800 -41.6600 -0.2763 -0.0045 -
0.0099 -0.5860 -0.3029 -0.1610 0.5049
0.3365 -1.6882 -39.4606
28.0000 -39.9500 1.4950 0.1205 -
0.0939 -0.5553 -0.4099 -0.1713 0.5068
0.3361 -1.6862 -39.4624
28.0200 -42.8900 -1.3850 0.1152 -
0.1718 -0.5039 -0.5137 -0.1805 0.5082
0.3355 -1.6843 -39.4642
28.0400 -42.2500 -0.2967 -0.1869 -
0.2015 -0.4367 -0.6128 -0.1884 0.5093
0.3349 -1.6823 -39.4660
28.0600 -41.0000 1.2937 -0.5060 -
0.1680 -0.3582 -0.7059 -0.1950 0.5099
0.3342 -1.6804 -39.4678
28.0800 -43.1800 -1.0663 -0.4661 -
0.0749 -0.2727 -0.7919 -0.2000 0.5102
0.3334 -1.6784 -39.4696
28.1000 -42.0400 -0.4715 0.0176
0.0461 -0.1840 -0.8697 -0.2034 0.5101
0.3324 -1.6764 -39.4713
28.1200 -39.9700 0.9229 0.5136
0.1435 -0.0961 -0.9382 -0.2050 0.5096
0.3314 -1.6743 -39.4731
28.1400 -41.3500 -0.4643 0.5016
0.1728 -0.0134 -0.9965 -0.2048 0.5087
0.3302 -1.6723 -39.4749
28.1600 -41.6000 -0.2972 0.0406
0.1318 0.0595 -1.0435 -0.2025 0.5074
0.3289 -1.6703 -39.4767
28.1800 -41.3000 0.5641 -0.3722
0.0524 0.1172 -1.0780 -0.1982 0.5057
0.3276 -1.6682 -39.4785
28.2000 -42.6400 -0.9174 -0.2701 -
0.0195 0.1547 -1.0988 -0.1917 0.5036
0.3261 -1.6661 -39.4802
28.2200 -40.5600 0.8799 0.0141 -
0.0297 0.1679 -1.1052 -0.1830 0.5011
0.3245 -1.6640 -39.4820
28.2400 -40.5500 0.8049 0.0328
0.0559 0.1539 -1.0967 -0.1723 0.4982
0.3228 -1.6619 -39.4838

28.2600 -42.6800 -1.2957 -0.1034
0.2090 0.1125 -1.0734 -0.1594 0.4948
0.3210 -1.6598 -39.4855
28.2800 -40.4400 0.6222 0.0643
0.3653 0.0450 -1.0358 -0.1447 0.4912
0.3191 -1.6576 -39.4873
28.3000 -40.0600 0.6726 0.2964
0.4573 -0.0460 -0.9848 -0.1281 0.4871
0.3171 -1.6554 -39.4891
28.3200 -40.1200 0.9117 0.1549
0.4329 -0.1564 -0.9214 -0.1099 0.4826
0.3151 -1.6533 -39.4909
28.3400 -40.9900 0.4427 -0.1597
0.3020 -0.2792 -0.8470 -0.0903 0.4777
0.3129 -1.6511 -39.4926
28.3600 -43.1700 -1.4012 -0.2585
0.1126 -0.4062 -0.7627 -0.0694 0.4725
0.3106 -1.6489 -39.4944
28.3800 -41.0300 0.6836 -0.0397 -
0.0807 -0.5293 -0.6698 -0.0474 0.4668
0.3082 -1.6466 -39.4962
28.4000 -40.5000 1.2475 0.1135 -
0.2392 -0.6398 -0.5696 -0.0245 0.4608
0.3057 -1.6444 -39.4979
28.4200 -42.9200 -1.0083 -0.0265 -
0.3406 -0.7292 -0.4634 -0.0009 0.4544
0.3032 -1.6421 -39.4997
28.4400 -42.6300 -0.6015 -0.1642 -
0.4022 -0.7891 -0.3524 0.0233 0.4477
0.3005 -1.6399 -39.5014
28.4600 -41.7900 0.0502 -0.0210 -
0.4521 -0.8125 -0.2380 0.0480 0.4406
0.2978 -1.6376 -39.5032
28.4800 -41.3800 0.0908 0.3085 -
0.5053 -0.7949 -0.1213 0.0728 0.4331
0.2949 -1.6353 -39.5049
28.5000 -41.0100 0.2131 0.4245 -
0.5593 -0.7348 -0.0037 0.0978 0.4253
0.2920 -1.6330 -39.5067
28.5200 -41.2600 0.0287 0.1383 -
0.5986 -0.6339 0.1134 0.1226 0.4171
0.2890 -1.6307 -39.5085
28.5400 -41.7600 -0.2727 -0.2971 -
0.5933 -0.4970 0.2287 0.1472 0.4085
0.2859 -1.6283 -39.5102
28.5600 -40.9100 0.4026 -0.5431 -
0.5133 -0.3306 0.3408 0.1713 0.3996
0.2828 -1.6260 -39.5120
28.5800 -41.3400 -0.6822 -0.3311 -
0.3496 -0.1427 0.4484 0.1948 0.3904
0.2795 -1.6236 -39.5137

28.6000	-39.5800	0.0850	0.1636	-
0.1331	0.0581	0.5499	0.2175	0.3808
	0.2762	-1.6212	-39.5155	
28.6200	-37.9700	0.7929	0.4869	
0.0863	0.2633	0.6442	0.2392	0.3709
	0.2728	-1.6188	-39.5172	
28.6400	-39.0000	-0.5872	0.3637	
0.2621	0.4643	0.7297	0.2599	0.3607
	0.2693	-1.6164	-39.5190	
28.6600	-38.6600	-0.2636	0.0964	
0.3687	0.6524	0.8051	0.2792	0.3501
	0.2657	-1.6140	-39.5207	
28.6800	-37.7800	0.3929	-0.0709	
0.4051	0.8183	0.8691	0.2971	0.3393
	0.2621	-1.6115	-39.5224	
28.7000	-38.1100	-0.0572	-0.1879	
0.4031	0.9528	0.9202	0.3133	0.3281
	0.2584	-1.6091	-39.5242	
28.7200	-38.0500	0.1584	-0.3533	
0.4039	1.0469	0.9572	0.3277	0.3166
	0.2546	-1.6066	-39.5259	
28.7400	-38.4900	-0.3853	-0.3925	
0.4348	1.0938	0.9786	0.3401	0.3048
	0.2508	-1.6041	-39.5277	
28.7600	-37.9200	-0.0330	-0.2288	
0.5036	1.0904	0.9837	0.3505	0.2927
	0.2469	-1.6016	-39.5294	
28.7800	-37.0900	0.5722	-0.0403	
0.5979	1.0367	0.9727	0.3587	0.2803
	0.2429	-1.5991	-39.5311	
28.8000	-37.8500	-0.1652	0.0200	
0.6866	0.9352	0.9469	0.3647	0.2677
	0.2389	-1.5966	-39.5329	
28.8200	-38.2800	-0.6185	0.1216	
0.7176	0.7911	0.9080	0.3685	0.2547
	0.2348	-1.5940	-39.5346	
28.8400	-37.6600	0.0276	0.4016	
0.6444	0.6117	0.8574	0.3704	0.2415
	0.2307	-1.5915	-39.5363	
28.8600	-37.4600	0.5915	0.6009	
0.4481	0.4074	0.7967	0.3702	0.2281
	0.2265	-1.5889	-39.5381	
28.8800	-38.8000	-0.0811	0.4208	
0.1392	0.1896	0.7276	0.3680	0.2144
	0.2222	-1.5863	-39.5398	
28.9000	-39.7700	0.2643	-0.0891	-
0.2242	-0.0297	0.6516	0.3640	0.2005
	0.2179	-1.5837	-39.5415	
28.9200	-41.5100	-0.4662	-0.5177	-
0.5637	-0.2385	0.5703	0.3582	0.1863
	0.2135	-1.5811	-39.5432	

28.9400 -41.8500 -0.1427 -0.5663 -
0.8018 -0.4250 0.4853 0.3506 0.1720
0.2091 -1.5785 -39.5450
28.9600 -41.8600 -0.1131 -0.2905 -
0.8844 -0.5776 0.3982 0.3414 0.1575
0.2046 -1.5759 -39.5467
28.9800 -41.3500 0.2795 -0.0685 -
0.7998 -0.6866 0.3105 0.3305 0.1428
0.2001 -1.5732 -39.5484
29.0000 -41.3000 0.4215 -0.1316 -
0.5781 -0.7495 0.2239 0.3181 0.1279
0.1955 -1.5705 -39.5501
29.0200 -42.3500 -0.6848 -0.2518 -
0.2892 -0.7676 0.1397 0.3042 0.1129
0.1909 -1.5679 -39.5519
29.0400 -40.6500 0.5717 -0.0718 -
0.0171 -0.7436 0.0589 0.2889 0.0978
0.1862 -1.5652 -39.5536
29.0600 -40.7300 0.0624 0.3092
0.1794 -0.6800 -0.0177 0.2722 0.0825
0.1815 -1.5625 -39.5553
29.0800 -41.2300 -0.7025 0.5949
0.2631 -0.5802 -0.0894 0.2543 0.0672
0.1768 -1.5598 -39.5570
29.1000 -40.4800 0.0688 0.5249
0.2208 -0.4509 -0.1555 0.2351 0.0518
0.1720 -1.5570 -39.5587
29.1200 -40.2900 0.8914 0.0131
0.0973 -0.3041 -0.2154 0.2148 0.0363
0.1671 -1.5543 -39.5604
29.1400 -42.6500 -0.9264 -0.5622 -
0.0301 -0.1542 -0.2685 0.1933 0.0207
0.1623 -1.5515 -39.5621
29.1600 -40.9800 0.7430 -0.5678 -
0.0883 -0.0156 -0.3140 0.1709 0.0051
0.1574 -1.5488 -39.5639
29.1800 -41.5100 -0.0309 -0.2761 -
0.0398 0.0993 -0.3513 0.1475 -0.0105
0.1524 -1.5460 -39.5656
29.2000 -41.1900 -0.2046 -0.0867
0.1043 0.1829 -0.3799 0.1232 -0.0261
0.1475 -1.5432 -39.5673
29.2200 -40.5000 0.1760 0.0919
0.2786 0.2321 -0.3994 0.0982 -0.0417
0.1425 -1.5404 -39.5690
29.2400 -40.9400 -0.7180 0.5034
0.4067 0.2458 -0.4099 0.0724 -0.0574
0.1375 -1.5376 -39.5707
29.2600 -39.2600 0.7178 0.7408
0.4418 0.2248 -0.4118 0.0461 -0.0729
0.1324 -1.5347 -39.5724

29.2800 -40.3200 0.2919 0.3014
0.3707 0.1740 -0.4060 0.0194 -0.0884
0.1273 -1.5319 -39.5741
29.3000 -42.3200 -0.7037 -0.4364
0.2337 0.1024 -0.3937 -0.0077 -0.1039
0.1222 -1.5290 -39.5758
29.3200 -41.9600 0.2623 -0.7787
0.0822 0.0200 -0.3761 -0.0348 -0.1193
0.1171 -1.5262 -39.5775
29.3400 -42.2600 -0.2183 -0.4992 -
0.0449 -0.0629 -0.3544 -0.0620 -0.1346
0.1119 -1.5233 -39.5792
29.3600 -41.5900 0.0956 0.1012 -
0.1382 -0.1367 -0.3299 -0.0889 -0.1498
0.1068 -1.5204 -39.5809
29.3800 -41.4000 -0.1923 0.6708 -
0.2041 -0.1928 -0.3039 -0.1154 -0.1648
0.1016 -1.5175 -39.5826
29.4000 -40.8600 0.4764 0.6870 -
0.2456 -0.2243 -0.2776 -0.1414 -0.1798
0.0964 -1.5146 -39.5843
29.4200 -41.3500 0.7490 0.0267 -
0.2641 -0.2269 -0.2522 -0.1667 -0.1945
0.0912 -1.5116 -39.5860
29.4400 -43.7900 -0.9871 -0.7067 -
0.2538 -0.1998 -0.2285 -0.1912 -0.2092
0.0860 -1.5087 -39.5877
29.4600 -41.8400 0.8058 -0.6573 -
0.2105 -0.1453 -0.2073 -0.2145 -0.2236
0.0807 -1.5057 -39.5893
29.4800 -41.9800 -0.0718 -0.0145 -
0.1489 -0.0697 -0.1886 -0.2368 -0.2378
0.0755 -1.5028 -39.5910
29.5000 -41.7800 -0.4825 0.5468 -
0.0913 0.0187 -0.1728 -0.2577 -0.2519
0.0702 -1.4998 -39.5927
29.5200 -40.5500 0.5485 0.6552 -
0.0623 0.1106 -0.1597 -0.2772 -0.2657
0.0649 -1.4968 -39.5944
29.5400 -41.7900 -0.6019 0.3862 -
0.0681 0.1972 -0.1494 -0.2951 -0.2793
0.0597 -1.4938 -39.5961
29.5600 -41.0700 0.6930 -0.1448 -
0.0665 0.2700 -0.1417 -0.3115 -0.2926
0.0544 -1.4908 -39.5978
29.5800 -42.8900 -0.7507 -0.7109 -
0.0073 0.3217 -0.1366 -0.3262 -0.3056
0.0491 -1.4878 -39.5994
29.6000 -41.2800 0.7104 -0.6258
0.1243 0.3468 -0.1338 -0.3392 -0.3184
0.0438 -1.4847 -39.6011

29.6200 -41.7200 -0.7753 0.2691
0.2845 0.3426 -0.1330 -0.3506 -0.3309
0.0386 -1.4817 -39.6028
29.6400 -39.5000 0.8810 0.8306
0.3805 0.3085 -0.1337 -0.3603 -0.3431
0.0333 -1.4786 -39.6045
29.6600 -41.8200 -0.6834 0.2100
0.3370 0.2468 -0.1357 -0.3683 -0.3549
0.0280 -1.4756 -39.6062
29.6800 -41.7400 0.5316 -0.6524
0.1843 0.1636 -0.1386 -0.3747 -0.3665
0.0228 -1.4725 -39.6078
29.7000 -43.2100 -0.7852 -0.5391 -
0.0206 0.0675 -0.1420 -0.3794 -0.3776
0.0175 -1.4694 -39.6095
29.7200 -41.3400 0.7000 0.1936 -
0.2165 -0.0327 -0.1457 -0.3827 -0.3885
0.0122 -1.4663 -39.6112
29.7400 -42.2100 -0.3287 0.5063 -
0.3350 -0.1285 -0.1494 -0.3844 -0.3989
0.0070 -1.4632 -39.6128
29.7600 -42.0700 0.2507 0.2001 -
0.3412 -0.2115 -0.1526 -0.3847 -0.4090
0.0018 -1.4600 -39.6145
29.7800 -43.0400 -0.2757 -0.2912 -
0.2585 -0.2750 -0.1551 -0.3836 -0.4187
-0.0034 -1.4569 -39.6162
29.8000 -42.9100 0.1172 -0.5316 -
0.1226 -0.3158 -0.1566 -0.3811 -0.4280
-0.0086 -1.4538 -39.6178
29.8200 -43.1600 -0.6039 -0.2513
0.0275 -0.3325 -0.1568 -0.3773 -0.4369
-0.0138 -1.4506 -39.6195
29.8400 -41.3700 0.5427 0.2982
0.1544 -0.3246 -0.1555 -0.3722 -0.4455
-0.0190 -1.4474 -39.6212
29.8600 -40.8500 0.7011 0.5943
0.2219 -0.2919 -0.1530 -0.3659 -0.4536
-0.0241 -1.4442 -39.6228
29.8800 -42.6800 -1.0339 0.3939
0.2045 -0.2351 -0.1492 -0.3584 -0.4613
-0.0293 -1.4411 -39.6245
29.9000 -42.8700 -0.5806 -0.1584
0.1130 -0.1574 -0.1446 -0.3499 -0.4686
-0.0344 -1.4379 -39.6261
29.9200 -41.4800 1.3091 -0.6519 -
0.0183 -0.0645 -0.1391 -0.3403 -0.4755
-0.0394 -1.4346 -39.6278
29.9400 -43.2500 -0.5448 -0.5794 -
0.1499 0.0362 -0.1330 -0.3298 -0.4820
-0.0445 -1.4314 -39.6295

29.9600	-43.3600	-1.2470	0.0459	-
0.2488	0.1374	-0.1265	-0.3184	-0.4882
	-0.0495	-1.4282	-39.6311	
29.9800	-40.1900	1.1710	0.6821	-
0.2835	0.2327	-0.1196	-0.3061	-0.4939
	-0.0545	-1.4249	-39.6328	
30.0000	-42.3500	-0.7714	0.3741	-
0.2322	0.3170	-0.1124	-0.2931	-0.4992
	-0.0595	-1.4217	-39.6344	
30.0200	-41.9000	0.2751	-0.4424	-
0.1058	0.3862	-0.1052	-0.2794	-0.5041
	-0.0644	-1.4184	-39.6361	
30.0400	-42.5900	-0.5046	-0.5892	
0.0683	0.4367	-0.0979	-0.2652	-0.5086
	-0.0693	-1.4151	-39.6377	
30.0600	-42.0600	-0.7519	-0.0689	
0.2532	0.4655	-0.0908	-0.2504	-0.5128
	-0.0742	-1.4119	-39.6394	
30.0800	-40.3400	0.2392	0.5442	
0.4048	0.4701	-0.0838	-0.2352	-0.5165
	-0.0790	-1.4086	-39.6410	
30.1000	-38.9800	1.3446	0.6734	
0.4788	0.4484	-0.0769	-0.2196	-0.5199
	-0.0838	-1.4053	-39.6426	
30.1200	-41.7000	-0.6889	0.0788	
0.4450	0.4003	-0.0700	-0.2037	-0.5229
	-0.0886	-1.4019	-39.6443	
30.1400	-43.0800	-1.2955	-0.5227	
0.3139	0.3294	-0.0629	-0.1876	-0.5255
	-0.0933	-1.3986	-39.6459	
30.1600	-41.0200	1.0137	-0.4862	
0.1084	0.2409	-0.0556	-0.1714	-0.5277
	-0.0980	-1.3953	-39.6476	
30.1800	-41.5600	0.2884	0.0087	-
0.1339	0.1403	-0.0478	-0.1551	-0.5296
	-0.1026	-1.3919	-39.6492	
30.2000	-42.1500	-0.3467	0.3873	-
0.3615	0.0335	-0.0395	-0.1388	-0.5311
	-0.1072	-1.3886	-39.6508	
30.2200	-42.0800	0.0671	0.3488	-
0.5302	-0.0739	-0.0305	-0.1225	-0.5322
	-0.1117	-1.3852	-39.6525	
30.2400	-42.5500	0.0240	0.0265	-
0.6142	-0.1764	-0.0208	-0.1064	-0.5330
	-0.1162	-1.3818	-39.6541	
30.2600	-42.7900	0.2452	-0.3654	-
0.5933	-0.2692	-0.0101	-0.0905	-0.5334
	-0.1207	-1.3785	-39.6557	
30.2800	-43.5300	-0.4209	-0.5066	-
0.4545	-0.3490	0.0016	-0.0748	-0.5334
	-0.1251	-1.3751	-39.6574	

30.3000 -42.7100 -0.1631 -0.1659 -
0.2088 -0.4142 0.0146 -0.0593 -0.5331
-0.1295 -1.3717 -39.6590
30.3200 -41.2300 0.5775 0.2689
0.0916 -0.4643 0.0291 -0.0443 -0.5325
-0.1338 -1.3683 -39.6606
30.3400 -41.9700 -0.2522 0.2340
0.3813 -0.4988 0.0452 -0.0296 -0.5315
-0.1380 -1.3648 -39.6623
30.3600 -41.1700 0.4596 -0.1433
0.5954 -0.5172 0.0632 -0.0153 -0.5302
-0.1422 -1.3614 -39.6639
30.3800 -42.5900 -0.9485 -0.1099
0.6868 -0.5189 0.0831 -0.0015 -0.5286
-0.1464 -1.3580 -39.6655
30.4000 -40.7500 0.4596 0.2314
0.6478 -0.5036 0.1049 0.0118 -0.5266
-0.1505 -1.3545 -39.6671
30.4200 -40.0600 1.1049 0.2892
0.5029 -0.4719 0.1284 0.0246 -0.5243
-0.1545 -1.3511 -39.6688
30.4400 -42.9000 -1.0955 -0.1281
0.2914 -0.4252 0.1533 0.0367 -0.5216
-0.1585 -1.3476 -39.6704
30.4600 -41.9900 0.0656 -0.2969
0.0489 -0.3653 0.1790 0.0482 -0.5187
-0.1625 -1.3441 -39.6720
30.4800 -41.9100 0.0332 0.0010 -
0.1922 -0.2932 0.2052 0.0590 -0.5154
-0.1663 -1.3406 -39.6736
30.5000 -41.2400 0.6274 0.2020 -
0.4056 -0.2096 0.2312 0.0691 -0.5118
-0.1702 -1.3371 -39.6752
30.5200 -41.7900 0.2023 0.0843 -
0.5704 -0.1160 0.2567 0.0783 -0.5079
-0.1739 -1.3336 -39.6768
30.5400 -42.6600 -0.6362 -0.1183 -
0.6592 -0.0143 0.2812 0.0867 -0.5036
-0.1776 -1.3301 -39.6784
30.5600 -42.3200 -0.2868 -0.1332 -
0.6502 0.0934 0.3042 0.0941 -0.4991
-0.1813 -1.3266 -39.6801
30.5800 -41.4800 0.1095 -0.0333 -
0.5449 0.2039 0.3251 0.1006 -0.4942
-0.1849 -1.3231 -39.6817
30.6000 -40.3300 0.9551 -0.0238 -
0.3623 0.3127 0.3437 0.1061 -0.4891
-0.1884 -1.3195 -39.6833
30.6200 -41.7100 -0.8133 -0.0460 -
0.1320 0.4140 0.3593 0.1105 -0.4836
-0.1919 -1.3160 -39.6849

30.6400 -41.2000 -0.6891 0.0680
0.1084 0.5016 0.3716 0.1139 -0.4779
-0.1953 -1.3124 -39.6865
30.6600 -39.1700 0.9386 0.1928
0.3249 0.5693 0.3800 0.1163 -0.4719
-0.1987 -1.3089 -39.6881
30.6800 -40.6800 -0.5909 -0.0177
0.4865 0.6111 0.3841 0.1175 -0.4656
-0.2020 -1.3053 -39.6897
30.7000 -39.9300 0.3834 -0.2766
0.5594 0.6218 0.3835 0.1176 -0.4590
-0.2052 -1.3017 -39.6913
30.7200 -40.6200 -0.5931 -0.0078
0.5247 0.5985 0.3779 0.1166 -0.4522
-0.2084 -1.2981 -39.6929
30.7400 -39.5600 0.1635 0.4624
0.3961 0.5425 0.3674 0.1146 -0.4451
-0.2115 -1.2945 -39.6945
30.7600 -39.2500 0.6441 0.5148
0.2082 0.4579 0.3523 0.1117 -0.4378
-0.2146 -1.2909 -39.6961
30.7800 -41.4900 -0.6091 -0.0872
0.0113 0.3501 0.3332 0.1078 -0.4302
-0.2176 -1.2873 -39.6977
30.8000 -41.4900 0.3847 -0.6974 -
0.1389 0.2254 0.3103 0.1030 -0.4224
-0.2205 -1.2837 -39.6993
30.8200 -42.5300 -0.5821 -0.5573 -
0.1992 0.0906 0.2842 0.0975 -0.4144
-0.2233 -1.2801 -39.7009
30.8400 -41.9500 -0.5839 0.1412 -
0.1795 -0.0464 0.2554 0.0912 -0.4061
-0.2262 -1.2764 -39.7025
30.8600 -40.0600 0.8965 0.6633 -
0.1281 -0.1770 0.2242 0.0842 -0.3977
-0.2289 -1.2728 -39.7041
30.8800 -42.0800 -0.7332 0.3648 -
0.0949 -0.2927 0.1912 0.0766 -0.3891
-0.2316 -1.2692 -39.7056
30.9000 -41.5800 0.5671 -0.2683 -
0.0991 -0.3864 0.1568 0.0684 -0.3803
-0.2342 -1.2655 -39.7072
30.9200 -42.9800 -0.6460 -0.4110 -
0.1344 -0.4529 0.1213 0.0597 -0.3713
-0.2367 -1.2618 -39.7088
30.9400 -41.6900 0.4953 -0.0887 -
0.1772 -0.4894 0.0854 0.0506 -0.3621
-0.2392 -1.2582 -39.7104
30.9600 -42.2000 -0.2719 0.1911 -
0.1937 -0.4955 0.0494 0.0411 -0.3528
-0.2417 -1.2545 -39.7120

30.9800 -41.8300 0.1190 0.1260 -
0.1629 -0.4731 0.0135 0.0313 -0.3433
-0.2440 -1.2508 -39.7136
31.0000 -42.1200 0.1718 -0.1832 -
0.0908 -0.4259 -0.0221 0.0212 -0.3337
-0.2463 -1.2471 -39.7151
31.0200 -42.7000 -0.3531 -0.4030 -
0.0019 -0.3597 -0.0570 0.0109 -0.3240
-0.2486 -1.2434 -39.7167
31.0400 -42.0900 -0.1200 -0.2131
0.0750 -0.2806 -0.0912 0.0005 -0.3141
-0.2507 -1.2397 -39.7183
31.0600 -41.2200 0.2104 0.2874
0.1213 -0.1945 -0.1243 -0.0099 -0.3042
-0.2528 -1.2360 -39.7199
31.0800 -41.2000 -0.1278 0.6422
0.1268 -0.1069 -0.1563 -0.0203 -0.2941
-0.2549 -1.2322 -39.7214
31.1000 -40.5500 0.5507 0.4822
0.0880 -0.0224 -0.1870 -0.0306 -0.2840
-0.2569 -1.2285 -39.7230
31.1200 -42.3800 -0.6654 -0.0164
0.0238 0.0553 -0.2160 -0.0407 -0.2737
-0.2588 -1.2248 -39.7246
31.1400 -41.4900 0.6815 -0.4733 -
0.0331 0.1233 -0.2435 -0.0505 -0.2634
-0.2606 -1.2210 -39.7262
31.1600 -42.9800 -0.5939 -0.6943 -
0.0482 0.1797 -0.2693 -0.0599 -0.2530
-0.2624 -1.2173 -39.7277
31.1800 -41.9900 0.0437 -0.5351 -
0.0054 0.2240 -0.2938 -0.0689 -0.2426
-0.2642 -1.2135 -39.7293
31.2000 -40.8100 0.6316 -0.0655
0.0733 0.2573 -0.3171 -0.0772 -0.2321
-0.2658 -1.2097 -39.7308
31.2200 -41.5200 -0.6232 0.4875
0.1469 0.2816 -0.3397 -0.0849 -0.2216
-0.2674 -1.2060 -39.7324
31.2400 -41.1800 -0.4734 0.7055
0.1689 0.2992 -0.3618 -0.0919 -0.2110
-0.2689 -1.2022 -39.7340
31.2600 -40.1200 1.0124 0.3495
0.1097 0.3121 -0.3840 -0.0981 -0.2005
-0.2704 -1.1984 -39.7355
31.2800 -42.9700 -0.9709 -0.3445 -
0.0104 0.3217 -0.4065 -0.1034 -0.1899
-0.2718 -1.1946 -39.7371
31.3000 -41.4200 0.7122 -0.4116 -
0.1422 0.3282 -0.4296 -0.1078 -0.1793
-0.2732 -1.1908 -39.7387

31.3200 -42.3200 -0.5204 0.0210 -
0.2281 0.3315 -0.4535 -0.1111 -0.1687
-0.2744 -1.1870 -39.7402
31.3400 -41.1400 0.6624 0.0643 -
0.2237 0.3312 -0.4781 -0.1134 -0.1581
-0.2757 -1.1832 -39.7418
31.3600 -42.4700 -0.4706 -0.1497 -
0.1309 0.3257 -0.5033 -0.1147 -0.1475
-0.2768 -1.1794 -39.7433
31.3800 -42.1900 -0.3603 -0.1462
0.0094 0.3132 -0.5290 -0.1149 -0.1370
-0.2779 -1.1755 -39.7449
31.4000 -41.2800 0.3192 0.0504
0.1551 0.2911 -0.5549 -0.1141 -0.1265
-0.2789 -1.1717 -39.7464
31.4200 -40.7900 0.4641 0.1592
0.2674 0.2573 -0.5808 -0.1123 -0.1160
-0.2799 -1.1679 -39.7480
31.4400 -41.9900 -0.6242 0.1365
0.3144 0.2102 -0.6064 -0.1095 -0.1055
-0.2808 -1.1640 -39.7495
31.4600 -41.2500 0.2534 0.1026
0.2850 0.1496 -0.6311 -0.1057 -0.0952
-0.2816 -1.1602 -39.7511
31.4800 -41.7800 0.0293 0.0164
0.1903 0.0776 -0.6544 -0.1010 -0.0848
-0.2823 -1.1563 -39.7526
31.5000 -41.6900 0.5276 -0.1756
0.0627 -0.0016 -0.6757 -0.0954 -0.0746
-0.2830 -1.1525 -39.7541
31.5200 -43.1900 -0.8182 -0.1875 -
0.0585 -0.0832 -0.6945 -0.0890 -0.0644
-0.2837 -1.1486 -39.7557
31.5400 -41.5700 0.8478 0.0068 -
0.1479 -0.1620 -0.7099 -0.0817 -0.0543
-0.2842 -1.1447 -39.7572
31.5600 -43.0200 -0.6767 0.0871 -
0.1972 -0.2330 -0.7215 -0.0736 -0.0443
-0.2848 -1.1408 -39.7588
31.5800 -42.6500 -0.2143 0.1175 -
0.2196 -0.2916 -0.7285 -0.0647 -0.0343
-0.2852 -1.1369 -39.7603
31.6000 -41.8700 0.5162 0.1291 -
0.2292 -0.3344 -0.7304 -0.0551 -0.0245
-0.2856 -1.1330 -39.7618
31.6200 -42.7800 -0.2569 0.0322 -
0.2286 -0.3596 -0.7266 -0.0448 -0.0147
-0.2859 -1.1291 -39.7634
31.6400 -42.4700 0.2888 -0.2179 -
0.2100 -0.3678 -0.7166 -0.0339 -0.0050
-0.2861 -1.1252 -39.7649

31.6600 -43.3000 -0.6577 -0.2587 -
0.1641 -0.3619 -0.7000 -0.0224 0.0045
-0.2863 -1.1213 -39.7664
31.6800 -41.9300 0.2952 0.0439 -
0.0893 -0.3459 -0.6768 -0.0103 0.0139
-0.2864 -1.1174 -39.7680
31.7000 -41.3200 0.3925 0.2927
0.0037 -0.3243 -0.6466 0.0023 0.0233
-0.2865 -1.1135 -39.7695
31.7200 -41.1300 0.7301 0.1322
0.0971 -0.3012 -0.6095 0.0153 0.0325
-0.2864 -1.1096 -39.7710
31.7400 -42.5300 -0.6278 -0.2069
0.1725 -0.2804 -0.5653 0.0287 0.0416
-0.2864 -1.1056 -39.7725
31.7600 -42.6700 -0.7470 -0.2657
0.2126 -0.2650 -0.5140 0.0424 0.0505
-0.2862 -1.1017 -39.7741
31.7800 -40.9900 0.4908 0.0491
0.2060 -0.2572 -0.4558 0.0563 0.0594
-0.2860 -1.0978 -39.7756
31.8000 -40.7200 0.4280 0.3423
0.1492 -0.2581 -0.3909 0.0704 0.0681
-0.2857 -1.0938 -39.7771
31.8200 -40.8600 0.6132 0.1397
0.0487 -0.2674 -0.3197 0.0846 0.0766
-0.2854 -1.0899 -39.7786
31.8400 -42.4900 -0.7299 -0.2357 -
0.0580 -0.2833 -0.2427 0.0989 0.0850
-0.2850 -1.0859 -39.7802
31.8600 -41.6500 0.1760 -0.2349 -
0.1322 -0.3036 -0.1606 0.1131 0.0933
-0.2845 -1.0820 -39.7817
31.8800 -41.2900 0.2032 -0.0177 -
0.1524 -0.3255 -0.0741 0.1273 0.1014
-0.2840 -1.0780 -39.7832
31.9000 -41.1500 0.2384 0.0301 -
0.1184 -0.3468 0.0161 0.1413 0.1093
-0.2833 -1.0740 -39.7847
31.9200 -41.5700 -0.4919 0.0283 -
0.0574 -0.3650 0.1091 0.1551 0.1171
-0.2827 -1.0701 -39.7862
31.9400 -40.6800 0.3075 0.1031 -
0.0014 -0.3783 0.2041 0.1687 0.1247
-0.2819 -1.0661 -39.7877
31.9600 -40.8000 0.0193 0.1094
0.0316 -0.3851 0.3002 0.1818 0.1322
-0.2811 -1.0621 -39.7892
31.9800 -40.7100 0.0199 0.0039
0.0361 -0.3842 0.3965 0.1946 0.1395
-0.2802 -1.0581 -39.7907

32.0000 −40.9200 −0.2000 −0.0875
0.0159 −0.3745 0.4923 0.2070 0.1466
−0.2793 −1.0541 −39.7922
32.0200 −40.3100 0.2002 −0.0648 −
0.0191 −0.3548 0.5866 0.2188 0.1535
−0.2783 −1.0501 −39.7937
32.0400 −40.5800 −0.2265 0.0468 −
0.0606 −0.3242 0.6786 0.2300 0.1602
−0.2772 −1.0461 −39.7953
32.0600 −40.0400 0.1620 0.1277 −
0.1005 −0.2815 0.7673 0.2406 0.1668
−0.2761 −1.0421 −39.7968
32.0800 −39.9600 0.1681 0.0444 −
0.1285 −0.2267 0.8520 0.2505 0.1731
−0.2749 −1.0381 −39.7983
32.1000 −40.3900 −0.1557 −0.1498 −
0.1334 −0.1602 0.9319 0.2596 0.1793
−0.2736 −1.0341 −39.7998
32.1200 −40.0300 −0.0529 −0.2040 −
0.1068 −0.0829 1.0060 0.2679 0.1852
−0.2722 −1.0301 −39.8012
32.1400 −39.8100 −0.1244 −0.0238 −
0.0521 0.0033 1.0738 0.2753 0.1909
−0.2708 −1.0261 −39.8027
32.1600 −38.8200 0.3667 0.1399
0.0135 0.0967 1.1342 0.2817 0.1965
−0.2693 −1.0220 −39.8042
32.1800 −39.2400 −0.2285 0.1003
0.0686 0.1949 1.1866 0.2871 0.2018
−0.2678 −1.0180 −39.8057
32.2000 −39.0400 −0.1155 0.0228
0.0952 0.2956 1.2301 0.2915 0.2069
−0.2662 −1.0140 −39.8072
32.2200 −38.6900 0.0965 0.0306
0.0820 0.3961 1.2640 0.2947 0.2118
−0.2645 −1.0100 −39.8087
32.2400 −38.3600 0.4026 0.0136
0.0334 0.4931 1.2874 0.2967 0.2164
−0.2627 −1.0059 −39.8102
32.2600 −39.3900 −0.5889 −0.0219 −
0.0322 0.5827 1.2996 0.2974 0.2208
−0.2609 −1.0019 −39.8117
32.2800 −38.2800 0.3107 0.0154 −
0.0924 0.6607 1.2998 0.2969 0.2250
−0.2591 −0.9978 −39.8132
32.3000 −38.2400 0.3741 0.0220 −
0.1224 0.7225 1.2873 0.2950 0.2290
−0.2571 −0.9938 −39.8147
32.3200 −39.0800 −0.3705 −0.0733 −
0.0985 0.7635 1.2616 0.2918 0.2327
−0.2551 −0.9898 −39.8161

32.3400 -38.8800 -0.1543 -0.1793 -
0.0118 0.7799 1.2223 0.2874 0.2362
-0.2530 -0.9857 -39.8176
32.3600 -38.4600 0.1609 -0.2235
0.1268 0.7686 1.1696 0.2819 0.2395
-0.2509 -0.9817 -39.8191
32.3800 -38.5700 0.1204 -0.2350
0.2891 0.7279 1.1041 0.2751 0.2425
-0.2486 -0.9776 -39.8206
32.4000 -38.6800 -0.0749 -0.1820
0.4346 0.6566 1.0267 0.2674 0.2453
-0.2464 -0.9735 -39.8221
32.4200 -38.6700 -0.2070 0.0693
0.5124 0.5551 0.9386 0.2586 0.2478
-0.2440 -0.9695 -39.8235
32.4400 -38.2800 -0.0299 0.5010
0.4882 0.4265 0.8411 0.2488 0.2501
-0.2416 -0.9654 -39.8250
32.4600 -37.7600 0.5844 0.7671
0.3523 0.2768 0.7355 0.2382 0.2522
-0.2391 -0.9614 -39.8265
32.4800 -39.2200 -0.1223 0.6160
0.1211 0.1151 0.6231 0.2267 0.2540
-0.2366 -0.9573 -39.8279
32.5000 -40.9100 -0.7844 0.0975 -
0.1481 -0.0490 0.5051 0.2145 0.2556
-0.2340 -0.9532 -39.8294
32.5200 -40.2900 0.9602 -0.5926 -
0.3800 -0.2057 0.3828 0.2015 0.2570
-0.2313 -0.9491 -39.8309
32.5400 -42.9600 -0.9584 -0.8791 -
0.5014 -0.3454 0.2575 0.1878 0.2581
-0.2286 -0.9451 -39.8324
32.5600 -40.8000 0.8265 -0.3234 -
0.4731 -0.4595 0.1306 0.1736 0.2590
-0.2258 -0.9410 -39.8338
32.5800 -41.5900 -0.5497 0.4617 -
0.3301 -0.5429 0.0032 0.1588 0.2597
-0.2229 -0.9369 -39.8353
32.6000 -40.6700 0.1197 0.6958 -
0.1324 -0.5934 -0.1234 0.1435 0.2601
-0.2200 -0.9328 -39.8367
32.6200 -40.5900 0.4387 0.3249
0.0578 -0.6101 -0.2478 0.1277 0.2603
-0.2170 -0.9288 -39.8382
32.6400 -42.0900 -0.5697 -0.1882
0.1801 -0.5933 -0.3692 0.1116 0.2603
-0.2140 -0.9247 -39.8397
32.6600 -41.3900 0.2350 -0.1823
0.1908 -0.5453 -0.4864 0.0952 0.2601
-0.2109 -0.9206 -39.8411

32.6800 -41.7500 -0.4397 0.2614
0.1021 -0.4714 -0.5988 0.0785 0.2596
-0.2077 -0.9165 -39.8426
32.7000 -40.7500 0.6980 0.3430 -
0.0207 -0.3791 -0.7056 0.0617 0.2589
-0.2045 -0.9124 -39.8440
32.7200 -40.9700 1.0087 -0.0791 -
0.1145 -0.2764 -0.8058 0.0447 0.2580
-0.2013 -0.9083 -39.8455
32.7400 -43.7600 -1.3485 -0.5480 -
0.1462 -0.1713 -0.8988 0.0276 0.2568
-0.1979 -0.9042 -39.8469
32.7600 -41.8000 0.5127 -0.4663 -
0.1000 -0.0716 -0.9838 0.0104 0.2555
-0.1945 -0.9002 -39.8484
32.7800 -40.5000 1.2442 -0.0075
0.0010 0.0170 -1.0599 -0.0066 0.2539
-0.1911 -0.8961 -39.8498
32.8000 -42.3100 -1.1566 0.4319
0.1122 0.0895 -1.1267 -0.0236 0.2521
-0.1876 -0.8920 -39.8513
32.8200 -40.8100 0.2234 0.5030
0.1890 0.1422 -1.1836 -0.0405 0.2501
-0.1841 -0.8879 -39.8527
32.8400 -40.6000 0.7521 0.2410
0.2054 0.1726 -1.2305 -0.0571 0.2480
-0.1805 -0.8838 -39.8542
32.8600 -42.3400 -0.5445 -0.1685
0.1761 0.1801 -1.2670 -0.0735 0.2456
-0.1768 -0.8797 -39.8556
32.8800 -42.0000 0.0493 -0.3762
0.1327 0.1656 -1.2934 -0.0896 0.2430
-0.1731 -0.8756 -39.8570
32.9000 -42.0600 0.0952 -0.2969
0.1048 0.1310 -1.3097 -0.1053 0.2402
-0.1694 -0.8715 -39.8585
32.9200 -41.6000 0.3731 -0.1308
0.1073 0.0796 -1.3161 -0.1205 0.2372
-0.1656 -0.8674 -39.8599
32.9400 -42.5500 -0.7890 0.1054
0.1252 0.0163 -1.3128 -0.1353 0.2340
-0.1617 -0.8633 -39.8614
32.9600 -40.8300 1.0211 0.2112
0.1220 -0.0525 -1.3001 -0.1496 0.2307
-0.1578 -0.8592 -39.8628
32.9800 -43.1200 -1.1008 0.1285
0.0642 -0.1208 -1.2783 -0.1634 0.2272
-0.1539 -0.8551 -39.8642
33.0000 -41.4200 0.8063 0.1438 -
0.0615 -0.1835 -1.2477 -0.1766 0.2235
-0.1499 -0.8510 -39.8657

33.0200 -41.7300 0.6929 0.1278 -
0.2351 -0.2370 -1.2084 -0.1893 0.2196
-0.1459 -0.8469 -39.8671
33.0400 -43.3400 -0.5740 -0.0463 -
0.4012 -0.2793 -1.1608 -0.2013 0.2156
-0.1418 -0.8428 -39.8685
33.0600 -43.6200 -0.5188 -0.2795 -
0.4969 -0.3091 -1.1050 -0.2127 0.2115
-0.1377 -0.8387 -39.8699
33.0800 -42.3700 0.6134 -0.3684 -
0.4762 -0.3266 -1.0411 -0.2235 0.2071
-0.1335 -0.8346 -39.8714
33.1000 -43.3800 -0.7469 -0.1047 -
0.3262 -0.3344 -0.9691 -0.2336 0.2027
-0.1294 -0.8305 -39.8728
33.1200 -41.0800 0.8481 0.1688 -
0.0812 -0.3367 -0.8892 -0.2431 0.1981
-0.1251 -0.8264 -39.8742
33.1400 -42.3100 -0.5972 0.1025
0.2003 -0.3378 -0.8015 -0.2520 0.1933
-0.1209 -0.8223 -39.8756
33.1600 -42.0000 -0.4366 0.0247
0.4492 -0.3421 -0.7061 -0.2603 0.1884
-0.1165 -0.8182 -39.8771
33.1800 -41.2200 -0.2075 0.2119
0.6014 -0.3537 -0.6033 -0.2679 0.1834
-0.1122 -0.8141 -39.8785
33.2000 -40.2000 0.6372 0.3304
0.6197 -0.3758 -0.4936 -0.2749 0.1783
-0.1078 -0.8100 -39.8799
33.2200 -41.2300 -0.1647 0.1358
0.4979 -0.4076 -0.3779 -0.2813 0.1731
-0.1034 -0.8059 -39.8813
33.2400 -41.9200 -0.3844 -0.1298
0.2759 -0.4445 -0.2573 -0.2871 0.1677
-0.0990 -0.8018 -39.8827
33.2600 -41.2100 0.4159 -0.1998
0.0049 -0.4798 -0.1330 -0.2923 0.1623
-0.0945 -0.7977 -39.8841
33.2800 -41.9200 -0.0372 -0.1531 -
0.2607 -0.5066 -0.0064 -0.2969 0.1567
-0.0900 -0.7936 -39.8855
33.3000 -41.9300 0.0569 -0.0313 -
0.4735 -0.5173 0.1214 -0.3009 0.1511
-0.0855 -0.7896 -39.8870
33.3200 -42.3500 -0.9197 0.2856 -
0.5999 -0.5050 0.2492 -0.3043 0.1453
-0.0809 -0.7855 -39.8884
33.3400 -40.3000 1.2090 0.1779 -
0.6015 -0.4656 0.3758 -0.3071 0.1395
-0.0764 -0.7814 -39.8898

33.3600 -42.8800 -1.2274 -0.4109 -
0.4654 -0.3987 0.4999 -0.3094 0.1336
-0.0718 -0.7773 -39.8912
33.3800 -40.1500 0.9836 -0.3162 -
0.2206 -0.3068 0.6204 -0.3111 0.1276
-0.0671 -0.7732 -39.8926
33.4000 -40.3600 -0.2510 0.2578
0.0635 -0.1937 0.7358 -0.3122 0.1216
-0.0625 -0.7691 -39.8940
33.4200 -39.6400 -0.1993 0.4566
0.2989 -0.0637 0.8450 -0.3128 0.1155
-0.0578 -0.7650 -39.8954
33.4400 -39.0800 0.3121 0.1687
0.4161 0.0790 0.9466 -0.3128 0.1093
-0.0531 -0.7609 -39.8968
33.4600 -39.6600 -0.2963 -0.2263
0.4026 0.2297 1.0394 -0.3123 0.1031
-0.0484 -0.7569 -39.8982
33.4800 -39.3500 0.1687 -0.3699
0.2786 0.3820 1.1220 -0.3112 0.0969
-0.0437 -0.7528 -39.8996
33.5000 -39.3300 -0.0173 -0.1897
0.0859 0.5273 1.1931 -0.3096 0.0906
-0.0390 -0.7487 -39.9010
33.5200 -39.2900 -0.3436 0.1782 -
0.1147 0.6562 1.2516 -0.3075 0.0843
-0.0342 -0.7446 -39.9024
33.5400 -38.1000 0.5510 0.4221 -
0.2540 0.7597 1.2961 -0.3049 0.0779
-0.0294 -0.7406 -39.9037
33.5600 -39.2900 -0.3953 0.1793 -
0.2828 0.8308 1.3256 -0.3017 0.0716
-0.0246 -0.7365 -39.9051
33.5800 -38.8700 0.3988 -0.3817 -
0.1979 0.8662 1.3390 -0.2980 0.0652
-0.0199 -0.7324 -39.9065
33.6000 -39.8700 -0.4768 -0.6639 -
0.0210 0.8654 1.3359 -0.2938 0.0588
-0.0151 -0.7284 -39.9079
33.6200 -38.8900 0.0967 -0.3724
0.2126 0.8285 1.3167 -0.2892 0.0525
-0.0102 -0.7243 -39.9093
33.6400 -38.2000 -0.0235 0.2061
0.4483 0.7567 1.2823 -0.2841 0.0461
-0.0054 -0.7202 -39.9107
33.6600 -37.4400 0.2691 0.6372
0.6224 0.6512 1.2341 -0.2785 0.0397
-0.0006 -0.7162 -39.9121
33.6800 -37.6900 0.0454 0.7404
0.6757 0.5152 1.1734 -0.2725 0.0334
0.0042 -0.7121 -39.9134

33.7000 -38.4300 -0.2188 0.5979
0.5836 0.3542 1.1015 -0.2660 0.0270
0.0091 -0.7080 -39.9148
33.7200 -38.6800 0.2796 0.3166
0.3737 0.1766 1.0198 -0.2590 0.0207
0.0139 -0.7040 -39.9162
33.7400 -39.8800 -0.0926 -0.0630
0.0990 -0.0082 0.9297 -0.2516 0.0144
0.0187 -0.6999 -39.9176
33.7600 -41.0800 -0.1362 -0.4700 -
0.1827 -0.1910 0.8325 -0.2438 0.0082
0.0236 -0.6959 -39.9189
33.7800 -41.2900 0.2632 -0.7198 -
0.4178 -0.3626 0.7298 -0.2355 0.0020
0.0284 -0.6919 -39.9203
33.8000 -42.3400 -0.5104 -0.5739 -
0.5646 -0.5138 0.6227 -0.2268 -0.0041
0.0332 -0.6878 -39.9217
33.8200 -41.1400 0.3559 -0.0170 -
0.6203 -0.6367 0.5128 -0.2177 -0.0102
0.0380 -0.6838 -39.9231
33.8400 -41.3500 -0.1108 0.4798 -
0.6024 -0.7249 0.4014 -0.2083 -0.0162
0.0429 -0.6797 -39.9244
33.8600 -41.0200 0.2197 0.5331 -
0.5283 -0.7750 0.2898 -0.1984 -0.0222
0.0477 -0.6757 -39.9258
33.8800 -41.5200 -0.0060 0.2278 -
0.4129 -0.7869 0.1793 -0.1882 -0.0281
0.0525 -0.6717 -39.9272
33.9000 -42.1100 -0.3120 -0.1255 -
0.2752 -0.7645 0.0708 -0.1776 -0.0339
0.0573 -0.6677 -39.9285
33.9200 -41.5400 0.2728 -0.3080 -
0.1285 -0.7130 -0.0349 -0.1666 -0.0396
0.0620 -0.6636 -39.9299
33.9400 -42.0900 -0.2914 -0.2920
0.0150 -0.6383 -0.1375 -0.1553 -0.0453
0.0668 -0.6596 -39.9312
33.9600 -41.3800 0.0467 -0.0997
0.1460 -0.5464 -0.2364 -0.1436 -0.0508
0.0716 -0.6556 -39.9326
33.9800 -41.1400 -0.0319 0.1826
0.2554 -0.4429 -0.3313 -0.1316 -0.0563
0.0763 -0.6516 -39.9340
34.0000 -40.6400 0.1452 0.3375
0.3365 -0.3337 -0.4219 -0.1193 -0.0616
0.0811 -0.6476 -39.9353
34.0200 -40.0600 0.7404 0.1514
0.3858 -0.2244 -0.5078 -0.1067 -0.0668
0.0858 -0.6436 -39.9367

34.0400 -40.8200 0.4046 -0.2823
0.4061 -0.1200 -0.5886 -0.0937 -0.0719
0.0905 -0.6396 -39.9380
34.0600 -42.4700 -1.1113 -0.5121
0.4029 -0.0248 -0.6642 -0.0804 -0.0769
0.0952 -0.6356 -39.9394
34.0800 -41.0400 -0.0208 -0.1977
0.3790 0.0579 -0.7343 -0.0668 -0.0818
0.0998 -0.6316 -39.9407
34.1000 -39.6700 0.9810 0.2954
0.3276 0.1262 -0.7986 -0.0527 -0.0865
0.1045 -0.6276 -39.9421
34.1200 -40.4000 0.0802 0.5172
0.2403 0.1795 -0.8571 -0.0383 -0.0911
0.1091 -0.6236 -39.9434
34.1400 -41.3000 -0.5201 0.3799
0.1171 0.2186 -0.9098 -0.0234 -0.0956
0.1137 -0.6196 -39.9448
34.1600 -41.1300 0.1404 0.0695 -
0.0245 0.2448 -0.9569 -0.0081 -0.0999
0.1183 -0.6157 -39.9461
34.1800 -41.7600 -0.1378 -0.1884 -
0.1527 0.2596 -0.9986 0.0077 -0.1040
0.1228 -0.6117 -39.9475
34.2000 -41.8700 0.1131 -0.3631 -
0.2320 0.2647 -1.0351 0.0240 -0.1080
0.1273 -0.6077 -39.9488
34.2200 -42.1400 -0.1406 -0.4081 -
0.2366 0.2623 -1.0670 0.0409 -0.1118
0.1318 -0.6038 -39.9501
34.2400 -41.7000 0.1094 -0.2383 -
0.1719 0.2545 -1.0944 0.0583 -0.1155
0.1363 -0.5998 -39.9515
34.2600 -41.5100 -0.3594 0.1294 -
0.0727 0.2429 -1.1178 0.0763 -0.1189
0.1407 -0.5959 -39.9528
34.2800 -40.2900 0.6301 0.3368
0.0183 0.2290 -1.1376 0.0950 -0.1222
0.1451 -0.5919 -39.9541
34.3000 -41.5900 -0.4942 0.1430
0.0623 0.2138 -1.1541 0.1143 -0.1253
0.1495 -0.5880 -39.9555
34.3200 -41.3300 0.0087 -0.0237
0.0407 0.1980 -1.1676 0.1342 -0.1282
0.1539 -0.5840 -39.9568
34.3400 -41.5200 -0.2891 0.1237 -
0.0372 0.1813 -1.1780 0.1549 -0.1309
0.1582 -0.5801 -39.9581
34.3600 -40.6300 0.5686 0.2068 -
0.1309 0.1624 -1.1855 0.1761 -0.1335
0.1624 -0.5762 -39.9595

34.3800 -41.8500 -0.3366 -0.0455 -
0.1926 0.1398 -1.1897 0.1979 -0.1358
0.1667 -0.5723 -39.9608
34.4000 -42.0100 -0.1023 -0.3264 -
0.1847 0.1121 -1.1906 0.2201 -0.1379
0.1709 -0.5684 -39.9621
34.4200 -41.8800 -0.1510 -0.3334 -
0.0938 0.0784 -1.1880 0.2427 -0.1398
0.1751 -0.5644 -39.9634
34.4400 -40.8400 0.6295 -0.1358
0.0608 0.0392 -1.1816 0.2656 -0.1415
0.1792 -0.5605 -39.9648
34.4600 -41.7800 -0.8704 0.1927
0.2297 -0.0038 -1.1712 0.2887 -0.1430
0.1833 -0.5566 -39.9661
34.4800 -39.5400 1.0502 0.4103
0.3606 -0.0487 -1.1567 0.3119 -0.1443
0.1874 -0.5527 -39.9674
34.5000 -41.4900 -0.8566 0.3133
0.4178 -0.0933 -1.1379 0.3351 -0.1453
0.1914 -0.5489 -39.9687
34.5200 -41.8500 -0.9866 0.0712
0.3910 -0.1348 -1.1145 0.3582 -0.1462
0.1954 -0.5450 -39.9700
34.5400 -40.8000 0.3692 -0.1606
0.2912 -0.1705 -1.0863 0.3811 -0.1468
0.1993 -0.5411 -39.9713
34.5600 -40.2400 1.3164 -0.3517
0.1417 -0.1980 -1.0534 0.4037 -0.1472
0.2032 -0.5372 -39.9727
34.5800 -41.3900 0.2790 -0.4421 -
0.0363 -0.2147 -1.0154 0.4260 -0.1474
0.2070 -0.5334 -39.9740
34.6000 -42.7600 -1.2410 -0.1837 -
0.2312 -0.2180 -0.9722 0.4478 -0.1473
0.2109 -0.5295 -39.9753
34.6200 -39.9900 1.0544 0.4183 -
0.4232 -0.2056 -0.9236 0.4691 -0.1471
0.2146 -0.5257 -39.9766
34.6400 -41.4200 -0.4526 0.5337 -
0.5783 -0.1765 -0.8696 0.4898 -0.1466
0.2183 -0.5218 -39.9779
34.6600 -41.2100 0.3109 -0.0026 -
0.6570 -0.1313 -0.8102 0.5098 -0.1458
0.2220 -0.5180 -39.9792
34.6800 -42.3700 -0.6202 -0.4071 -
0.6189 -0.0725 -0.7454 0.5291 -0.1449
0.2256 -0.5141 -39.9805
34.7000 -40.6300 0.7651 -0.4714 -
0.4357 -0.0050 -0.6755 0.5475 -0.1437
0.2292 -0.5103 -39.9818

34.7200 -41.5700 -0.8745 -0.2003 -
0.1280 0.0647 -0.6006 0.5651 -0.1423
0.2328 -0.5065 -39.9831
34.7400 -38.9100 0.8591 0.2541
0.2287 0.1294 -0.5210 0.5817 -0.1407
0.2363 -0.5027 -39.9844
34.7600 -39.4900 -0.2885 0.3506
0.5409 0.1822 -0.4368 0.5972 -0.1388
0.2397 -0.4989 -39.9857
34.7800 -39.1500 -0.2023 0.1550
0.7233 0.2158 -0.3483 0.6117 -0.1368
0.2431 -0.4951 -39.9870
34.8000 -38.8100 0.1488 0.0540
0.7236 0.2245 -0.2558 0.6250 -0.1345
0.2464 -0.4913 -39.9883
34.8200 -39.2700 -0.2519 0.1537
0.5512 0.2064 -0.1596 0.6370 -0.1319
0.2497 -0.4875 -39.9896
34.8400 -38.9100 0.2067 0.1597
0.2664 0.1657 -0.0603 0.6478 -0.1292
0.2530 -0.4837 -39.9909
34.8600 -39.3900 0.3061 -0.0848 -
0.0485 0.1082 0.0414 0.6572 -0.1262
0.2562 -0.4800 -39.9922
34.8800 -40.7000 -0.5771 -0.3228 -
0.3208 0.0401 0.1448 0.6652 -0.1231
0.2593 -0.4762 -39.9935
34.9000 -39.6100 0.5073 -0.2188 -
0.4972 -0.0323 0.2491 0.6717 -0.1197
0.2624 -0.4724 -39.9947
34.9200 -40.3900 -0.4590 0.0417 -
0.5601 -0.1030 0.3535 0.6766 -0.1161
0.2654 -0.4687 -39.9960
34.9400 -39.1800 0.5594 0.0951 -
0.5219 -0.1669 0.4571 0.6799 -0.1123
0.2684 -0.4649 -39.9973
34.9600 -40.3700 -0.5523 -0.0469 -
0.4100 -0.2204 0.5590 0.6815 -0.1083
0.2713 -0.4612 -39.9986
34.9800 -39.1900 0.3968 -0.1341 -
0.2593 -0.2612 0.6585 0.6814 -0.1041
0.2742 -0.4575 -39.9999
35.0000 -39.2400 -0.0127 -0.0774 -
0.1021 -0.2876 0.7546 0.6794 -0.0997
0.2770 -0.4538 -40.0012
35.0200 -39.2000 -0.2668 0.0603
0.0357 -0.2993 0.8466 0.6755 -0.0951
0.2798 -0.4501 -40.0024
35.0400 -38.2300 0.3613 0.1630
0.1407 -0.2963 0.9337 0.6697 -0.0904
0.2825 -0.4464 -40.0037

35.0600 -38.8600 -0.3739 0.1536
0.2117 -0.2796 1.0150 0.6619 -0.0854
0.2852 -0.4427 -40.0050
35.0800 -38.0800 0.2464 0.0458
0.2551 -0.2504 1.0900 0.6520 -0.0803
0.2878 -0.4390 -40.0063
35.1000 -38.5500 -0.0569 -0.0955
0.2832 -0.2106 1.1579 0.6400 -0.0750
0.2903 -0.4353 -40.0075
35.1200 -38.5200 -0.2127 -0.1550
0.3032 -0.1616 1.2182 0.6258 -0.0696
0.2928 -0.4316 -40.0088
35.1400 -38.0100 0.2771 -0.0971
0.3102 -0.1045 1.2701 0.6093 -0.0640
0.2953 -0.4280 -40.0101
35.1600 -38.4800 -0.4198 0.0287
0.2881 -0.0392 1.3130 0.5905 -0.0583
0.2976 -0.4243 -40.0113
35.1800 -37.1100 0.7119 0.0562
0.2155 0.0344 1.3460 0.5695 -0.0524
0.3000 -0.4207 -40.0126
35.2000 -38.9100 -0.7729 0.0402
0.0871 0.1152 1.3685 0.5460 -0.0464
0.3022 -0.4170 -40.0139
35.2200 -37.2800 0.7239 0.0902 -
0.0853 0.2009 1.3797 0.5203 -0.0402
0.3044 -0.4134 -40.0151
35.2400 -38.7600 -0.5011 0.0168 -
0.2761 0.2875 1.3791 0.4922 -0.0339
0.3066 -0.4098 -40.0164
35.2600 -38.0700 0.3151 -0.0987 -
0.4406 0.3701 1.3661 0.4619 -0.0275
0.3087 -0.4062 -40.0176
35.2800 -38.8000 -0.1380 -0.1428 -
0.5312 0.4438 1.3403 0.4295 -0.0209
0.3108 -0.4026 -40.0189
35.3000 -38.3900 0.0524 -0.0886 -
0.5112 0.5035 1.3013 0.3951 -0.0143
0.3127 -0.3990 -40.0201
35.3200 -38.6300 -0.5069 0.1251 -
0.3702 0.5446 1.2492 0.3588 -0.0075
0.3147 -0.3954 -40.0214
35.3400 -37.1700 0.7485 0.1933 -
0.1225 0.5633 1.1841 0.3207 -0.0006
0.3165 -0.3918 -40.0227
35.3600 -37.8700 0.1755 -0.0791
0.1857 0.5562 1.1069 0.2810 0.0064
0.3184 -0.3882 -40.0239
35.3800 -39.0500 -0.8705 -0.3185
0.4780 0.5202 1.0187 0.2398 0.0135
0.3201 -0.3847 -40.0252

35.4000 -37.1600 0.7323 -0.1006
0.6806 0.4525 0.9207 0.1973 0.0207
0.3218 -0.3811 -40.0264
35.4200 -37.9100 -0.2495 0.3125
0.7470 0.3527 0.8145 0.1535 0.0280
0.3235 -0.3776 -40.0276
35.4400 -38.0500 -0.1446 0.5241
0.6507 0.2248 0.7014 0.1087 0.0354
0.3251 -0.3741 -40.0289
35.4600 -38.3600 0.0595 0.4830
0.3966 0.0781 0.5831 0.0629 0.0428
0.3266 -0.3705 -40.0301
35.4800 -38.9900 0.3477 0.1807
0.0401 -0.0761 0.4611 0.0163 0.0504
0.3281 -0.3670 -40.0314
35.5000 -40.6400 -0.1332 -0.2841 -
0.3370 -0.2265 0.3368 -0.0309 0.0580
0.3295 -0.3635 -40.0326
35.5200 -41.7100 -0.2810 -0.5842 -
0.6426 -0.3617 0.2119 -0.0787 0.0657
0.3308 -0.3600 -40.0339
35.5400 -42.0100 -0.2968 -0.5357 -
0.7947 -0.4705 0.0877 -0.1268 0.0735
0.3321 -0.3566 -40.0351
35.5600 -41.1500 0.6933 -0.3381 -
0.7608 -0.5433 -0.0342 -0.1753 0.0813
0.3334 -0.3531 -40.0363
35.5800 -42.3500 -0.6868 -0.1587 -
0.5669 -0.5770 -0.1524 -0.2238 0.0893
0.3346 -0.3496 -40.0376
35.6000 -40.8400 0.4975 0.1051 -
0.2833 -0.5736 -0.2656 -0.2723 0.0972
0.3357 -0.3462 -40.0388
35.6200 -40.8800 -0.1581 0.4106
0.0106 -0.5374 -0.3730 -0.3207 0.1052
0.3368 -0.3427 -40.0400
35.6400 -40.6000 -0.1097 0.5405
0.2505 -0.4728 -0.4740 -0.3688 0.1133
0.3378 -0.3393 -40.0412
35.6600 -40.1500 0.3801 0.3490
0.3964 -0.3851 -0.5682 -0.4165 0.1214
0.3387 -0.3359 -40.0425
35.6800 -41.2500 -0.2166 -0.0887
0.4435 -0.2811 -0.6550 -0.4636 0.1296
0.3396 -0.3325 -40.0437
35.7000 -41.1700 0.1317 -0.3862
0.4098 -0.1694 -0.7340 -0.5100 0.1378
0.3405 -0.3291 -40.0449
35.7200 -41.7200 -0.3850 -0.2885
0.3225 -0.0602 -0.8047 -0.5556 0.1461
0.3412 -0.3257 -40.0461

35.7400	-40.8900	0.0998	0.1237	
0.2183	0.0360	-0.8668	-0.6002	0.1543
	0.3420	-0.3223	-40.0474	
35.7600	-40.7800	-0.0703	0.4891	
0.1386	0.1098	-0.9200	-0.6438	0.1626
	0.3426	-0.3189	-40.0486	
35.7800	-40.1300	0.6985	0.3881	
0.1165	0.1547	-0.9641	-0.6861	0.1710
	0.3432	-0.3156	-40.0498	
35.8000	-41.3200	0.1051	-0.1823	
0.1529	0.1687	-0.9993	-0.7272	0.1793
	0.3438	-0.3122	-40.0510	
35.8200	-42.9000	-0.9191	-0.7330	
0.2202	0.1553	-1.0261	-0.7668	0.1877
	0.3443	-0.3089	-40.0522	
35.8400	-41.7900	0.1901	-0.7578	
0.2846	0.1207	-1.0453	-0.8048	0.1961
	0.3447	-0.3056	-40.0534	
35.8600	-40.6900	0.9422	-0.2787	
0.3132	0.0727	-1.0577	-0.8412	0.2045
	0.3451	-0.3023	-40.0547	
35.8800	-41.5300	-0.4833	0.4403	
0.2828	0.0194	-1.0642	-0.8759	0.2129
	0.3454	-0.2990	-40.0559	
35.9000	-41.4400	-0.7794	0.9682	
0.1731	-0.0314	-1.0657	-0.9087	0.2213
	0.3456	-0.2957	-40.0571	
35.9200	-40.1400	0.8693	0.9302	-
0.0200	-0.0738	-1.0629	-0.9396	0.2298
	0.3458	-0.2924	-40.0583	
35.9400	-42.5000	-0.5193	0.1673	-
0.2578	-0.1025	-1.0568	-0.9684	0.2382
	0.3459	-0.2891	-40.0595	
35.9600	-43.3400	-0.2037	-0.7392	-
0.4703	-0.1128	-1.0482	-0.9952	0.2466
	0.3460	-0.2858	-40.0607	
35.9800	-43.3000	0.2193	-1.0241	-
0.5860	-0.1001	-1.0377	-1.0198	0.2551
	0.3460	-0.2826	-40.0619	
36.0000	-43.4000	-0.5820	-0.4062	-
0.5623	-0.0618	-1.0259	-1.0421	0.2635
	0.3460	-0.2794	-40.0631	
36.0200	-41.0300	0.6540	0.4770	-
0.4197	-0.0014	-1.0129	-1.0620	0.2719
	0.3459	-0.2761	-40.0643	
36.0400	-41.4300	-0.3213	0.6965	-
0.1960	0.0739	-0.9988	-1.0796	0.2803
	0.3457	-0.2729	-40.0655	
36.0600	-41.2000	0.1068	0.2594	
0.0616	0.1563	-0.9835	-1.0947	0.2886
	0.3455	-0.2697	-40.0667	

36.0800 -41.3600 0.1191 -0.1903
0.2900 0.2381 -0.9669 -1.1073 0.2970
0.3452 -0.2665 -40.0679
36.1000 -41.8600 -0.6974 -0.2149
0.4354 0.3115 -0.9492 -1.1175 0.3053
0.3449 -0.2633 -40.0691
36.1200 -40.6200 0.2145 0.0815
0.4867 0.3696 -0.9302 -1.1251 0.3136
0.3445 -0.2602 -40.0703
36.1400 -39.9600 0.6928 0.2677
0.4584 0.4075 -0.9097 -1.1302 0.3219
0.3441 -0.2570 -40.0714
36.1600 -41.2900 -0.5873 0.1297
0.3705 0.4227 -0.8879 -1.1329 0.3302
0.3436 -0.2539 -40.0726
36.1800 -41.4600 -0.4049 -0.0948
0.2473 0.4148 -0.8644 -1.1332 0.3384
0.3430 -0.2507 -40.0738
36.2000 -40.5500 0.7585 -0.1808
0.1125 0.3846 -0.8393 -1.1311 0.3466
0.3424 -0.2476 -40.0750
36.2200 -41.5600 -0.2586 -0.0527 -
0.0155 0.3342 -0.8124 -1.1268 0.3547
0.3417 -0.2445 -40.0762
36.2400 -41.9500 -0.6796 0.1295 -
0.1266 0.2669 -0.7837 -1.1202 0.3628
0.3409 -0.2414 -40.0774
36.2600 -40.5500 0.7452 0.1593 -
0.2144 0.1865 -0.7532 -1.1115 0.3708
0.3401 -0.2383 -40.0786
36.2800 -41.4300 0.2161 -0.0303 -
0.2747 0.0974 -0.7210 -1.1007 0.3788
0.3392 -0.2352 -40.0797
36.3000 -42.6100 -0.7921 -0.1552 -
0.3044 0.0039 -0.6872 -1.0879 0.3867
0.3383 -0.2322 -40.0809
36.3200 -41.1100 0.5437 0.0568 -
0.3041 -0.0897 -0.6519 -1.0733 0.3946
0.3373 -0.2291 -40.0821
36.3400 -41.6900 -0.2161 0.2334 -
0.2741 -0.1791 -0.6150 -1.0568 0.4024
0.3363 -0.2261 -40.0833
36.3600 -41.2500 0.4233 0.0041 -
0.2149 -0.2601 -0.5768 -1.0385 0.4101
0.3352 -0.2230 -40.0844
36.3800 -42.3100 -0.3893 -0.3191 -
0.1347 -0.3291 -0.5372 -1.0185 0.4178
0.3340 -0.2200 -40.0856
36.4000 -41.8400 0.0234 -0.3641 -
0.0494 -0.3828 -0.4963 -0.9970 0.4253
0.3328 -0.2170 -40.0868

36.4200 -41.2600 0.1702 -0.0906
0.0193 -0.4184 -0.4544 -0.9739 0.4328
0.3315 -0.2140 -40.0879
36.4400 -41.3300 -0.3252 0.3073
0.0483 -0.4336 -0.4117 -0.9494 0.4402
0.3302 -0.2110 -40.0891
36.4600 -40.1800 0.4988 0.5118
0.0206 -0.4271 -0.3686 -0.9235 0.4476
0.3288 -0.2080 -40.0903
36.4800 -41.4800 -0.4751 0.3166 -
0.0643 -0.3991 -0.3254 -0.8962 0.4548
0.3273 -0.2051 -40.0914
36.5000 -40.7700 0.4990 -0.1449 -
0.1754 -0.3507 -0.2826 -0.8676 0.4619
0.3258 -0.2021 -40.0926
36.5200 -41.9200 -0.2762 -0.4696 -
0.2697 -0.2839 -0.2405 -0.8378 0.4690
0.3242 -0.1992 -40.0937
36.5400 -41.7200 -0.3361 -0.3765 -
0.3127 -0.2011 -0.1996 -0.8068 0.4759
0.3226 -0.1962 -40.0949
36.5600 -40.4400 0.4006 0.0113 -
0.2958 -0.1060 -0.1602 -0.7746 0.4827
0.3209 -0.1933 -40.0961
36.5800 -40.5300 -0.2345 0.2411 -
0.2291 -0.0029 -0.1227 -0.7413 0.4894
0.3191 -0.1904 -40.0972
36.6000 -39.6400 0.6253 0.0579 -
0.1318 0.1031 -0.0876 -0.7070 0.4960
0.3173 -0.1875 -40.0984
36.6200 -40.9700 -0.8856 -0.0896 -
0.0280 0.2070 -0.0552 -0.6716 0.5025
0.3155 -0.1846 -40.0995
36.6400 -38.9200 0.8006 0.0091
0.0669 0.3033 -0.0259 -0.6352 0.5088
0.3135 -0.1817 -40.1007
36.6600 -38.7000 0.8079 0.0578
0.1510 0.3861 -0.0000 -0.5979 0.5150
0.3115 -0.1789 -40.1018
36.6800 -39.4200 -0.0066 -0.0594
0.2243 0.4497 0.0219 -0.5597 0.5211
0.3095 -0.1760 -40.1030
36.7000 -40.3600 -1.2062 -0.0946
0.2835 0.4894 0.0397 -0.5207 0.5270
0.3074 -0.1732 -40.1041
36.7200 -38.0400 0.8861 0.0525
0.3236 0.5021 0.0530 -0.4808 0.5328
0.3052 -0.1703 -40.1052
36.7400 -38.2000 0.6936 0.1594
0.3341 0.4869 0.0616 -0.4403 0.5385
0.3030 -0.1675 -40.1064

36.7600 −39.3700 −0.4547 0.1433
0.3036 0.4452 0.0657 −0.3990 0.5440
0.3007 −0.1647 −40.1075
36.7800 −39.3300 −0.3412 0.1228
0.2260 0.3801 0.0657 −0.3571 0.5493
0.2984 −0.1619 −40.1087
36.8000 −39.2000 −0.1672 0.2249
0.1040 0.2968 0.0618 −0.3146 0.5545
0.2960 −0.1591 −40.1098
36.8200 −38.8700 0.3747 0.2658 −
0.0470 0.2010 0.0546 −0.2715 0.5596
0.2935 −0.1563 −40.1109
36.8400 −39.4400 0.0843 0.0830 −
0.1998 0.0986 0.0444 −0.2279 0.5644
0.2910 −0.1536 −40.1121
36.8600 −40.1700 0.0053 −0.2073 −
0.3195 −0.0046 0.0319 −0.1839 0.5692
0.2885 −0.1508 −40.1132
36.8800 −40.8800 −0.4728 −0.3989 −
0.3734 −0.1032 0.0174 −0.1394 0.5737
0.2858 −0.1481 −40.1143
36.9000 −39.7200 0.6137 −0.4413 −
0.3403 −0.1921 0.0015 −0.0946 0.5780
0.2831 −0.1453 −40.1155
36.9200 −40.8300 −0.6380 −0.2840 −
0.2333 −0.2676 −0.0152 −0.0496 0.5822
0.2804 −0.1426 −40.1166
36.9400 −39.6800 0.0238 0.1222 −
0.0906 −0.3272 −0.0322 −0.0043 0.5862
0.2776 −0.1399 −40.1177
36.9600 −39.0800 0.1954 0.4960
0.0372 −0.3687 −0.0490 0.0411 0.5900
0.2747 −0.1372 −40.1188
36.9800 −38.7500 0.4316 0.4693
0.1045 −0.3903 −0.0650 0.0867 0.5936
0.2718 −0.1345 −40.1200
37.0000 −39.4900 0.1036 0.0657
0.0958 −0.3905 −0.0800 0.1323 0.5970
0.2689 −0.1318 −40.1211
37.0200 −40.4700 −0.5638 −0.2688
0.0267 −0.3698 −0.0937 0.1778 0.6002
0.2658 −0.1291 −40.1222
37.0400 −39.6000 0.2164 −0.1923 −
0.0740 −0.3301 −0.1058 0.2233 0.6032
0.2628 −0.1264 −40.1233
37.0600 −39.5700 −0.0654 0.0698 −
0.1650 −0.2748 −0.1163 0.2687 0.6060
0.2596 −0.1238 −40.1244
37.0800 −39.1200 0.4905 0.0239 −
0.2072 −0.2074 −0.1252 0.3139 0.6086
0.2565 −0.1211 −40.1255

37.1000 -40.2500 -0.5410 -0.2145 -
0.1863 -0.1319 -0.1324 0.3589 0.6109
0.2532 -0.1185 -40.1266
37.1200 -39.1700 0.2570 -0.2009 -
0.1157 -0.0528 -0.1378 0.4037 0.6131
0.2499 -0.1159 -40.1278
37.1400 -39.0200 -0.0653 0.0442 -
0.0187 0.0256 -0.1416 0.4481 0.6150
0.2466 -0.1132 -40.1289
37.1600 -38.7800 -0.1574 0.2605
0.0784 0.0993 -0.1437 0.4921 0.6166
0.2432 -0.1106 -40.1300
37.1800 -38.0600 0.3303 0.2611
0.1521 0.1642 -0.1442 0.5357 0.6181
0.2397 -0.1080 -40.1311
37.2000 -38.4600 -0.0184 0.0290
0.1908 0.2170 -0.1432 0.5788 0.6193
0.2362 -0.1054 -40.1322
37.2200 -39.0800 -0.4605 -0.2413
0.1991 0.2551 -0.1407 0.6214 0.6202
0.2327 -0.1029 -40.1333
37.2400 -38.2600 0.5362 -0.3028
0.1867 0.2772 -0.1368 0.6633 0.6209
0.2291 -0.1003 -40.1344
37.2600 -38.4900 -0.0862 -0.0873
0.1597 0.2834 -0.1316 0.7047 0.6213
0.2254 -0.0977 -40.1355
37.2800 -38.6800 -0.5372 0.2318
0.1155 0.2752 -0.1253 0.7454 0.6215
0.2217 -0.0952 -40.1366
37.3000 -37.4600 0.6468 0.3180
0.0517 0.2555 -0.1178 0.7853 0.6214
0.2180 -0.0926 -40.1377
37.3200 -38.9800 -0.4628 0.0050 -
0.0278 0.2274 -0.1094 0.8245 0.6211
0.2142 -0.0901 -40.1388
37.3400 -38.8000 -0.0067 -0.2725 -
0.1041 0.1948 -0.1002 0.8628 0.6205
0.2103 -0.0876 -40.1399
37.3600 -38.8000 -0.0583 -0.1963 -
0.1555 0.1613 -0.0903 0.9002 0.6196
0.2064 -0.0851 -40.1410
37.3800 -38.5300 0.0338 0.0485 -
0.1707 0.1297 -0.0797 0.9366 0.6184
0.2025 -0.0826 -40.1421
37.4000 -38.3000 0.0205 0.1842 -
0.1514 0.1019 -0.0685 0.9719 0.6169
0.1985 -0.0801 -40.1431
37.4200 -38.1200 0.2877 0.1139 -
0.1045 0.0783 -0.0565 1.0060 0.6152
0.1945 -0.0776 -40.1442

37.4400 -38.7900 -0.3776 -0.0809 -
0.0423 0.0584 -0.0434 1.0389 0.6131
0.1904 -0.0751 -40.1453
37.4600 -38.2400 0.2995 -0.1750
0.0209 0.0411 -0.0289 1.0704 0.6107
0.1863 -0.0727 -40.1464
37.4800 -38.6300 -0.3706 -0.0467
0.0742 0.0251 -0.0126 1.1005 0.6081
0.1822 -0.0702 -40.1475
37.5000 -37.9000 0.1091 0.1685
0.1134 0.0085 0.0059 1.1289 0.6051
0.1780 -0.0677 -40.1486
37.5200 -37.8300 0.0754 0.2237
0.1352 -0.0104 0.0270 1.1558 0.6018
0.1738 -0.0653 -40.1496
37.5400 -37.5800 0.5260 -0.0311
0.1395 -0.0332 0.0511 1.1808 0.5982
0.1695 -0.0629 -40.1507
37.5600 -39.1200 -0.7216 -0.3064
0.1300 -0.0605 0.0785 1.2040 0.5943
0.1652 -0.0604 -40.1518
37.5800 -37.7900 0.4593 -0.1795
0.1109 -0.0922 0.1094 1.2252 0.5900
0.1609 -0.0580 -40.1529
37.6000 -38.2300 -0.1945 0.1869
0.0754 -0.1275 0.1439 1.2444 0.5855
0.1565 -0.0556 -40.1539
37.6200 -37.5200 0.3285 0.3661
0.0150 -0.1649 0.1819 1.2614 0.5806
0.1521 -0.0532 -40.1550
37.6400 -38.1300 -0.0763 0.2275 -
0.0675 -0.2023 0.2235 1.2763 0.5753
0.1476 -0.0508 -40.1561
37.6600 -38.2900 0.1102 -0.0748 -
0.1478 -0.2376 0.2683 1.2888 0.5698
0.1432 -0.0485 -40.1571
37.6800 -39.0100 -0.3215 -0.3267 -
0.1909 -0.2688 0.3161 1.2990 0.5639
0.1387 -0.0461 -40.1582
37.7000 -38.2900 0.3218 -0.3525 -
0.1695 -0.2940 0.3666 1.3068 0.5576
0.1341 -0.0437 -40.1593
37.7200 -38.8300 -0.5486 -0.0745 -
0.0837 -0.3124 0.4194 1.3121 0.5510
0.1295 -0.0414 -40.1603
37.7400 -37.2400 0.6173 0.2468
0.0339 -0.3241 0.4741 1.3148 0.5441
0.1249 -0.0390 -40.1614
37.7600 -37.8400 -0.1436 0.2830
0.1440 -0.3295 0.5303 1.3150 0.5369
0.1203 -0.0367 -40.1624

37.7800 −38.0400 −0.4384 0.1355
0.2159 −0.3281 0.5873 1.3124 0.5293
0.1157 −0.0343 −40.1635
37.8000 −37.4400 0.2379 −0.0027
0.2337 −0.3191 0.6446 1.3071 0.5214
0.1110 −0.0320 −40.1646
37.8200 −37.4400 0.3444 −0.1230
0.2012 −0.3010 0.7013 1.2990 0.5132
0.1063 −0.0297 −40.1656
37.8400 −38.2000 −0.4233 −0.1842
0.1322 −0.2719 0.7563 1.2880 0.5046
0.1016 −0.0274 −40.1667
37.8600 −37.7900 0.0440 −0.0886
0.0396 −0.2308 0.8087 1.2740 0.4958
0.0968 −0.0251 −40.1677
37.8800 −37.5400 0.1134 0.1093 −
0.0652 −0.1763 0.8572 1.2570 0.4867
0.0920 −0.0228 −40.1688
37.9000 −37.5700 0.0313 0.1932 −
0.1760 −0.1077 0.9008 1.2370 0.4772
0.0872 −0.0205 −40.1698
37.9200 −37.7100 −0.0132 0.0932 −
0.2861 −0.0250 0.9385 1.2138 0.4675
0.0824 −0.0182 −40.1709
37.9400 −38.2100 −0.2947 −0.0702 −
0.3797 0.0696 0.9692 1.1874 0.4576
0.0776 −0.0160 −40.1719
37.9600 −37.4200 0.5202 −0.1630 −
0.4338 0.1721 0.9919 1.1579 0.4473
0.0727 −0.0137 −40.1729
37.9800 −38.3600 −0.5569 −0.1298 −
0.4275 0.2773 1.0058 1.1251 0.4369
0.0678 −0.0115 −40.1740
38.0000 −37.0100 0.5723 0.0174 −
0.3510 0.3791 1.0100 1.0892 0.4261
0.0630 −0.0092 −40.1750
38.0200 −37.8000 −0.4744 0.0696 −
0.2097 0.4711 1.0037 1.0502 0.4152
0.0581 −0.0070 −40.1761
38.0400 −36.8900 0.4246 −0.0315 −
0.0193 0.5471 0.9860 1.0083 0.4040
0.0531 −0.0047 −40.1771
38.0600 −37.3900 −0.2286 −0.1431
0.1993 0.6003 0.9566 0.9635 0.3926
0.0482 −0.0025 −40.1781
38.0800 −37.1800 −0.1033 −0.1651
0.4145 0.6236 0.9152 0.9161 0.3810
0.0432 −0.0003 −40.1792
38.1000 −36.5700 0.3004 −0.0795
0.5892 0.6111 0.8619 0.8662 0.3692
0.0383 0.0019 −40.1802

38.1200 -36.7300 0.0384 0.0909
0.6818 0.5593 0.7976 0.8140 0.3572
0.0333 0.0041 -40.1812
38.1400 -37.2000 -0.3440 0.3426
0.6508 0.4694 0.7235 0.7597 0.3450
0.0283 0.0063 -40.1823
38.1600 -37.1900 -0.2220 0.5650
0.4808 0.3476 0.6409 0.7035 0.3327
0.0233 0.0085 -40.1833
38.1800 -37.2700 0.5056 0.4985
0.1958 0.2040 0.5512 0.6455 0.3202
0.0183 0.0107 -40.1843
38.2000 -38.6300 0.3016 -0.0120 -
0.1417 0.0493 0.4557 0.5860 0.3075
0.0133 0.0129 -40.1853
38.2200 -40.8400 -0.7239 -0.6104 -
0.4499 -0.1056 0.3559 0.5252 0.2947
0.0083 0.0150 -40.1863
38.2400 -40.5700 0.2398 -0.7311 -
0.6496 -0.2499 0.2531 0.4631 0.2818
0.0033 0.0172 -40.1874
38.2600 -40.8100 -0.0868 -0.2511 -
0.6933 -0.3732 0.1488 0.4001 0.2687
-0.0017 0.0193 -40.1884
38.2800 -40.6400 -0.4034 0.3950 -
0.5955 -0.4679 0.0442 0.3363 0.2555
-0.0068 0.0215 -40.1894
38.3000 -39.2500 0.7643 0.6116 -
0.3927 -0.5305 -0.0591 0.2719 0.2422
-0.0118 0.0236 -40.1904
38.3200 -39.7100 0.6032 0.2456 -
0.1370 -0.5603 -0.1598 0.2071 0.2288
-0.0168 0.0258 -40.1914
38.3400 -41.6200 -0.8632 -0.2406
0.1083 -0.5578 -0.2568 0.1420 0.2153
-0.0219 0.0279 -40.1924
38.3600 -40.5200 0.2641 -0.2328
0.2813 -0.5246 -0.3489 0.0769 0.2017
-0.0269 0.0300 -40.1934
38.3800 -40.2100 0.1252 0.1085
0.3468 -0.4635 -0.4353 0.0119 0.1880
-0.0319 0.0321 -40.1945
38.4000 -39.9300 0.5079 0.2815
0.3104 -0.3802 -0.5150 -0.0527 0.1743
-0.0370 0.0342 -40.1955
38.4200 -41.3500 -0.6672 0.2001
0.2058 -0.2823 -0.5873 -0.1169 0.1605
-0.0420 0.0363 -40.1965
38.4400 -40.3900 0.7266 -0.1331
0.0794 -0.1787 -0.6514 -0.1803 0.1467
-0.0470 0.0384 -40.1975

38.4600 -42.2600 -0.7151 -0.4349 -
0.0214 -0.0789 -0.7066 -0.2428 0.1328
-0.0521 0.0405 -40.1985
38.4800 -41.1400 0.3092 -0.2645 -
0.0617 0.0085 -0.7523 -0.3043 0.1189
-0.0571 0.0426 -40.1995
38.5000 -40.6800 0.3603 0.1730 -
0.0416 0.0773 -0.7880 -0.3645 0.1050
-0.0621 0.0447 -40.2005
38.5200 -41.3100 -0.5168 0.4562
0.0201 0.1243 -0.8135 -0.4234 0.0910
-0.0671 0.0467 -40.2015
38.5400 -40.3800 0.4977 0.3153
0.0977 0.1487 -0.8286 -0.4806 0.0771
-0.0721 0.0488 -40.2025
38.5600 -40.9900 0.2877 -0.1171
0.1679 0.1518 -0.8337 -0.5361 0.0631
-0.0771 0.0509 -40.2035
38.5800 -42.0700 -0.2266 -0.5233
0.2147 0.1361 -0.8292 -0.5899 0.0492
-0.0821 0.0529 -40.2044
38.6000 -42.7000 -0.8461 -0.4743
0.2250 0.1054 -0.8157 -0.6417 0.0353
-0.0870 0.0550 -40.2054
38.6200 -40.7600 0.6234 0.0938
0.1928 0.0641 -0.7941 -0.6916 0.0214
-0.0920 0.0570 -40.2064
38.6400 -40.6200 0.5128 0.5560
0.1180 0.0173 -0.7650 -0.7394 0.0075
-0.0969 0.0590 -40.2074
38.6600 -40.9200 0.3326 0.4797
0.0068 -0.0301 -0.7292 -0.7852 -0.0063
-0.1019 0.0611 -40.2084
38.6800 -42.2400 -0.2664 0.0656 -
0.1188 -0.0727 -0.6876 -0.8288 -0.0200
-0.1068 0.0631 -40.2094
38.7000 -43.1900 -0.7632 -0.3121 -
0.2292 -0.1054 -0.6409 -0.8703 -0.0337
-0.1117 0.0651 -40.2104
38.7200 -42.1300 0.5600 -0.4516 -
0.2946 -0.1232 -0.5901 -0.9096 -0.0473
-0.1166 0.0671 -40.2113
38.7400 -42.0200 0.5576 -0.3530 -
0.2953 -0.1226 -0.5359 -0.9465 -0.0609
-0.1214 0.0691 -40.2123
38.7600 -43.0000 -0.8191 -0.0460 -
0.2337 -0.1033 -0.4794 -0.9812 -0.0743
-0.1263 0.0711 -40.2133
38.7800 -40.8900 0.7794 0.2972 -
0.1328 -0.0680 -0.4214 -1.0136 -0.0876
-0.1311 0.0731 -40.2143

38.8000 -42.2700 -0.6816 0.3147 -
0.0241 -0.0205 -0.3628 -1.0436 -0.1009
-0.1359 0.0751 -40.2152
38.8200 -41.1900 0.3830 0.0331
0.0642 0.0346 -0.3043 -1.0711 -0.1140
-0.1407 0.0771 -40.2162
38.8400 -41.1900 0.4197 -0.1776
0.1140 0.0926 -0.2467 -1.0962 -0.1270
-0.1455 0.0791 -40.2172
38.8600 -42.3300 -0.6873 -0.1179
0.1248 0.1482 -0.1908 -1.1189 -0.1399
-0.1502 0.0810 -40.2182
38.8800 -42.0000 -0.7068 0.0701
0.1148 0.1956 -0.1373 -1.1390 -0.1526
-0.1549 0.0830 -40.2191
38.9000 -40.0300 1.1564 0.1171
0.1034 0.2303 -0.0871 -1.1565 -0.1652
-0.1596 0.0850 -40.2201
38.9200 -42.5300 -1.0948 -0.0794
0.1055 0.2490 -0.0407 -1.1715 -0.1776
-0.1643 0.0869 -40.2211
38.9400 -41.5400 -0.0602 -0.1423
0.1277 0.2501 0.0012 -1.1839 -0.1899
-0.1689 0.0889 -40.2220
38.9600 -40.6300 0.6803 -0.0552
0.1691 0.2340 0.0380 -1.1937 -0.2020
-0.1736 0.0908 -40.2230
38.9800 -41.1300 0.1693 -0.0124
0.2155 0.2031 0.0696 -1.2009 -0.2139
-0.1781 0.0928 -40.2239
39.0000 -41.6800 -0.5127 -0.0018
0.2395 0.1617 0.0958 -1.2054 -0.2256
-0.1827 0.0947 -40.2249
39.0200 -40.9800 0.2589 0.1472
0.2110 0.1155 0.1167 -1.2074 -0.2371
-0.1872 0.0967 -40.2258
39.0400 -41.3300 -0.1628 0.3032
0.1092 0.0706 0.1323 -1.2068 -0.2484
-0.1917 0.0986 -40.2268
39.0600 -41.0200 0.6740 0.0156 -
0.0564 0.0327 0.1429 -1.2036 -0.2595
-0.1962 0.1005 -40.2277
39.0800 -43.0800 -0.8025 -0.3505 -
0.2342 0.0060 0.1484 -1.1980 -0.2704
-0.2006 0.1024 -40.2287
39.1000 -41.6100 0.5337 -0.1316 -
0.3662 -0.0065 0.1492 -1.1899 -0.2811
-0.2050 0.1044 -40.2296
39.1200 -41.6700 0.1713 0.2019 -
0.4165 -0.0032 0.1455 -1.1794 -0.2916
-0.2094 0.1063 -40.2306

39.1400 -41.8000 0.1288 0.0640 -
0.3719 0.0151 0.1377 -1.1667 -0.3018
-0.2137 0.1082 -40.2315
39.1600 -41.9900 0.2607 -0.3247 -
0.2493 0.0450 0.1263 -1.1517 -0.3118
-0.2180 0.1101 -40.2325
39.1800 -42.6800 -0.6534 -0.2772 -
0.0855 0.0808 0.1117 -1.1347 -0.3216
-0.2222 0.1120 -40.2334
39.2000 -40.4300 0.6950 0.3396
0.0841 0.1163 0.0944 -1.1156 -0.3311
-0.2265 0.1139 -40.2344
39.2200 -41.5300 -0.5760 0.4268
0.2405 0.1452 0.0749 -1.0948 -0.3404
-0.2306 0.1158 -40.2353
39.2400 -40.4000 1.0084 -0.3415
0.3669 0.1622 0.0538 -1.0721 -0.3495
-0.2348 0.1177 -40.2362
39.2600 -42.6200 -0.9356 -0.6537
0.4406 0.1625 0.0316 -1.0479 -0.3584
-0.2389 0.1195 -40.2372
39.2800 -42.8200 -1.5614 -0.1265
0.4401 0.1439 0.0089 -1.0221 -0.3670
-0.2429 0.1214 -40.2381
39.3000 -38.9000 1.7179 0.6751
0.3507 0.1073 -0.0137 -0.9950 -0.3754
-0.2469 0.1233 -40.2390
39.3200 -41.6200 -0.6215 0.6656
0.1713 0.0574 -0.0358 -0.9665 -0.3835
-0.2509 0.1252 -40.2400
39.3400 -42.8700 -1.2386 0.0831 -
0.0497 -0.0003 -0.0569 -0.9369 -0.3914
-0.2548 0.1270 -40.2409
39.3600 -42.6400 -0.1388 -0.4260 -
0.2462 -0.0597 -0.0766 -0.9062 -0.3991
-0.2587 0.1289 -40.2418
39.3800 -42.7700 -0.1463 -0.4767 -
0.3579 -0.1148 -0.0944 -0.8745 -0.4065
-0.2625 0.1307 -40.2427
39.4000 -41.9900 0.5728 -0.3260 -
0.3647 -0.1604 -0.1100 -0.8419 -0.4137
-0.2663 0.1326 -40.2437
39.4200 -42.7900 -0.5619 0.0086 -
0.2944 -0.1932 -0.1230 -0.8086 -0.4206
-0.2700 0.1345 -40.2446
39.4400 -41.1300 0.5967 0.4168 -
0.1967 -0.2113 -0.1331 -0.7745 -0.4273
-0.2737 0.1363 -40.2455
39.4600 -41.5400 0.0933 0.4505 -
0.1066 -0.2137 -0.1401 -0.7399 -0.4338
-0.2774 0.1381 -40.2464

39.4800 -41.4600 0.3198 0.0496 -
0.0412 -0.2012 -0.1439 -0.7048 -0.4400
-0.2810 0.1400 -40.2474
39.5000 -42.7200 -0.6489 -0.3034
0.0018 -0.1762 -0.1445 -0.6694 -0.4459
-0.2845 0.1418 -40.2483
39.5200 -42.8100 -0.7647 -0.2907
0.0323 -0.1427 -0.1419 -0.6336 -0.4517
-0.2880 0.1437 -40.2492
39.5400 -40.4900 1.1598 -0.0418
0.0595 -0.1056 -0.1360 -0.5976 -0.4572
-0.2914 0.1455 -40.2501
39.5600 -41.3200 0.0947 0.0612
0.0958 -0.0699 -0.1269 -0.5615 -0.4624
-0.2948 0.1473 -40.2510
39.5800 -42.1800 -0.8067 0.0353
0.1466 -0.0400 -0.1146 -0.5252 -0.4674
-0.2982 0.1491 -40.2519
39.6000 -42.1300 -0.9900 0.1556
0.2058 -0.0191 -0.0991 -0.4889 -0.4722
-0.3015 0.1510 -40.2528
39.6200 -39.6400 1.2324 0.2524
0.2590 -0.0093 -0.0806 -0.4527 -0.4767
-0.3047 0.1528 -40.2537
39.6400 -41.3300 -0.2646 0.0589
0.2956 -0.0121 -0.0591 -0.4164 -0.4810
-0.3079 0.1546 -40.2546
39.6600 -42.2400 -0.9722 -0.2034
0.3160 -0.0273 -0.0350 -0.3803 -0.4851
-0.3110 0.1564 -40.2555
39.6800 -40.3000 0.8821 -0.2550
0.3226 -0.0533 -0.0085 -0.3442 -0.4889
-0.3141 0.1582 -40.2564
39.7000 -41.4100 -0.3218 -0.0618
0.3115 -0.0866 0.0199 -0.3083 -0.4925
-0.3171 0.1600 -40.2573
39.7200 -41.4600 -0.6164 0.1356
0.2726 -0.1234 0.0497 -0.2726 -0.4959
-0.3200 0.1618 -40.2582
39.7400 -40.4300 0.3730 0.1720
0.1956 -0.1592 0.0805 -0.2371 -0.4990
-0.3229 0.1636 -40.2591
39.7600 -40.5700 0.5423 0.0414
0.0755 -0.1896 0.1116 -0.2018 -0.5018
-0.3258 0.1654 -40.2600
39.7800 -42.1900 -0.7693 -0.0921 -
0.0822 -0.2101 0.1426 -0.1668 -0.5045
-0.3286 0.1672 -40.2609
39.8000 -41.2300 0.1708 0.0073 -
0.2595 -0.2170 0.1730 -0.1320 -0.5069
-0.3313 0.1690 -40.2618

39.8200 -40.8200 0.5704 0.1290 -
0.4246 -0.2072 0.2023 -0.0976 -0.5091
-0.3340 0.1708 -40.2627
39.8400 -41.9000 -0.4063 -0.0053 -
0.5436 -0.1785 0.2301 -0.0636 -0.5110
-0.3367 0.1726 -40.2636
39.8600 -41.4900 0.2678 -0.2684 -
0.5936 -0.1302 0.2561 -0.0299 -0.5127
-0.3392 0.1744 -40.2645
39.8800 -41.9700 -0.4241 -0.2447 -
0.5668 -0.0634 0.2799 0.0033 -0.5142
-0.3417 0.1761 -40.2653
39.9000 -41.2700 -0.1516 0.0566 -
0.4747 0.0185 0.3012 0.0360 -0.5154
-0.3442 0.1779 -40.2662
39.9200 -39.8900 0.5913 0.2983 -
0.3424 0.1112 0.3198 0.0683 -0.5164
-0.3466 0.1797 -40.2671
39.9400 -40.8100 -0.4794 0.2280 -
0.1903 0.2101 0.3351 0.1000 -0.5172
-0.3489 0.1815 -40.2680
39.9600 -40.4400 -0.2870 0.0536 -
0.0284 0.3097 0.3470 0.1311 -0.5178
-0.3512 0.1832 -40.2688
39.9800 -40.1400 -0.1697 -0.0410
0.1393 0.4045 0.3550 0.1616 -0.5181
-0.3535 0.1850 -40.2697
40.0000 -38.9900 0.8730 -0.2013
0.3115 0.4888 0.3589 0.1915 -0.5182
-0.3556 0.1868 -40.2706
40.0200 -39.3100 0.4395 -0.3998
0.4793 0.5573 0.3583 0.2207 -0.5181
-0.3577 0.1886 -40.2715
40.0400 -40.4200 -0.8777 -0.3824
0.6169 0.6059 0.3530 0.2492 -0.5177
-0.3598 0.1903 -40.2723
40.0600 -40.2000 -1.0265 0.0196
0.6947 0.6311 0.3425 0.2770 -0.5171
-0.3618 0.1921 -40.2732
40.0800 -37.3600 1.1199 0.5495
0.6886 0.6305 0.3270 0.3040 -0.5164
-0.3637 0.1938 -40.2741
40.1000 -38.0200 0.4257 0.7008
0.5793 0.6045 0.3064 0.3303 -0.5153
-0.3656 0.1956 -40.2749
40.1200 -39.5700 -0.5470 0.4177
0.3711 0.5568 0.2810 0.3557 -0.5141
-0.3674 0.1974 -40.2758
40.1400 -39.7900 -0.0456 -0.0647
0.1017 0.4926 0.2513 0.3803 -0.5127
-0.3692 0.1991 -40.2767

40.1600 -40.9100 -0.2764 -0.4200 -
0.1756 0.4175 0.2175 0.4042 -0.5110
-0.3709 0.2009 -40.2775
40.1800 -40.5600 0.4957 -0.6452 -
0.3973 0.3373 0.1801 0.4272 -0.5092
-0.3725 0.2026 -40.2784
40.2000 -41.7600 -0.4602 -0.6582 -
0.5196 0.2576 0.1396 0.4493 -0.5071
-0.3741 0.2044 -40.2792
40.2200 -41.0100 0.0762 -0.2907 -
0.5385 0.1834 0.0963 0.4707 -0.5048
-0.3756 0.2061 -40.2801
40.2400 -40.4200 0.0495 0.2411 -
0.4803 0.1188 0.0507 0.4912 -0.5023
-0.3771 0.2079 -40.2809
40.2600 -39.9700 0.2741 0.5722 -
0.3853 0.0667 0.0032 0.5108 -0.4997
-0.3785 0.2096 -40.2818
40.2800 -40.4900 -0.2670 0.5529 -
0.2926 0.0292 -0.0456 0.5296 -0.4968
-0.3798 0.2114 -40.2826
40.3000 -40.2400 0.2168 0.2711 -
0.2177 0.0062 -0.0954 0.5476 -0.4937
-0.3811 0.2131 -40.2835
40.3200 -40.9400 -0.1694 -0.1139 -
0.1570 -0.0048 -0.1455 0.5646 -0.4905
-0.3823 0.2148 -40.2843
40.3400 -40.6600 0.4062 -0.4118 -
0.0904 -0.0080 -0.1954 0.5809 -0.4870
-0.3834 0.2166 -40.2852
40.3600 -41.6700 -0.7614 -0.3196 -
0.0033 -0.0084 -0.2444 0.5962 -0.4834
-0.3845 0.2183 -40.2860
40.3800 -39.5200 0.8903 0.0242
0.1007 -0.0107 -0.2920 0.6108 -0.4796
-0.3856 0.2201 -40.2869
40.4000 -40.3100 -0.0259 0.1777
0.2032 -0.0188 -0.3375 0.6244 -0.4756
-0.3865 0.2218 -40.2877
40.4200 -41.1700 -0.8672 0.1553
0.2812 -0.0359 -0.3803 0.6373 -0.4715
-0.3874 0.2235 -40.2885
40.4400 -40.3800 -0.0573 0.1299
0.3182 -0.0643 -0.4200 0.6492 -0.4671
-0.3883 0.2253 -40.2894
40.4600 -39.6700 0.8013 0.0692
0.3019 -0.1048 -0.4559 0.6604 -0.4626
-0.3890 0.2270 -40.2902
40.4800 -41.4500 -0.6080 -0.0898
0.2309 -0.1569 -0.4877 0.6707 -0.4580
-0.3898 0.2288 -40.2910

40.5000 -40.5500 0.3959 -0.1660
0.1135 -0.2186 -0.5148 0.6802 -0.4532
-0.3904 0.2305 -40.2919
40.5200 -41.2800 -0.2250 -0.0172 -
0.0272 -0.2869 -0.5368 0.6889 -0.4482
-0.3910 0.2322 -40.2927
40.5400 -40.9500 0.1451 0.1829 -
0.1500 -0.3582 -0.5534 0.6968 -0.4431
-0.3915 0.2340 -40.2935
40.5600 -41.0000 0.1451 0.1402 -
0.2091 -0.4282 -0.5642 0.7039 -0.4378
-0.3920 0.2357 -40.2944
40.5800 -41.9000 -0.2860 -0.2502 -
0.1847 -0.4927 -0.5690 0.7102 -0.4324
-0.3924 0.2374 -40.2952
40.6000 -41.4800 0.6395 -0.7345 -
0.0921 -0.5481 -0.5675 0.7158 -0.4269
-0.3927 0.2392 -40.2960
40.6200 -42.9000 -0.9523 -0.6047
0.0333 -0.5922 -0.5595 0.7206 -0.4212
-0.3930 0.2409 -40.2968
40.6400 -41.6400 -0.7526 0.2553
0.1440 -0.6236 -0.5448 0.7247 -0.4154
-0.3932 0.2426 -40.2976
40.6600 -38.8400 1.2081 1.0446
0.1920 -0.6416 -0.5233 0.7281 -0.4095
-0.3933 0.2443 -40.2985
40.6800 -40.9100 -0.6293 0.8775
0.1438 -0.6463 -0.4950 0.7309 -0.4035
-0.3934 0.2461 -40.2993
40.7000 -41.6000 -0.5410 0.1015
0.0249 -0.6383 -0.4599 0.7330 -0.3974
-0.3934 0.2478 -40.3001
40.7200 -41.9800 -0.1015 -0.6271 -
0.1049 -0.6185 -0.4183 0.7345 -0.3912
-0.3934 0.2495 -40.3009
40.7400 -41.7700 0.5482 -1.0201 -
0.1796 -0.5884 -0.3703 0.7353 -0.3849
-0.3932 0.2513 -40.3017
40.7600 -42.3900 -0.4188 -0.8428 -
0.1535 -0.5498 -0.3163 0.7356 -0.3785
-0.3931 0.2530 -40.3025
40.7800 -41.8300 -0.7183 -0.1602 -
0.0450 -0.5052 -0.2566 0.7352 -0.3721
-0.3928 0.2547 -40.3033
40.8000 -39.1500 0.9462 0.5290
0.0937 -0.4569 -0.1917 0.7344 -0.3655
-0.3925 0.2565 -40.3041
40.8200 -40.6600 -0.9005 0.6362
0.2037 -0.4070 -0.1222 0.7330 -0.3589
-0.3921 0.2582 -40.3049

40.8400	-39.2600	0.6221	0.3611	
0.2345	-0.3571	-0.0486	0.7311	-0.3523
	-0.3917	0.2599	-40.3057	
40.8600	-39.9900	0.1460	0.0460	
0.1695	-0.3087	0.0286	0.7287	-0.3455
	-0.3911	0.2616	-40.3065	
40.8800	-40.7200	-0.4577	-0.1821	
0.0269	-0.2623	0.1086	0.7258	-0.3388
	-0.3906	0.2634	-40.3073	
40.9000	-40.0300	0.3746	-0.2153	-
0.1417	-0.2185	0.1909	0.7225	-0.3320
	-0.3899	0.2651	-40.3081	
40.9200	-40.7700	-0.4689	-0.0353	-
0.2663	-0.1780	0.2750	0.7188	-0.3251
	-0.3892	0.2668	-40.3089	
40.9400	-39.3700	0.6326	0.0465	-
0.2871	-0.1411	0.3600	0.7146	-0.3182
	-0.3884	0.2686	-40.3097	
40.9600	-40.5700	-0.4954	-0.1466	-
0.1928	-0.1077	0.4453	0.7101	-0.3113
	-0.3876	0.2703	-40.3105	
40.9800	-39.7400	0.0715	-0.2337	-
0.0134	-0.0773	0.5303	0.7053	-0.3044
	-0.3867	0.2720	-40.3113	
41.0000	-39.1300	0.1173	-0.0566	
0.1981	-0.0490	0.6141	0.7001	-0.2975
	-0.3857	0.2738	-40.3121	
41.0200	-38.8800	-0.1582	0.2014	
0.3742	-0.0220	0.6959	0.6946	-0.2906
	-0.3847	0.2755	-40.3129	
41.0400	-38.2500	0.2979	0.2669	
0.4544	0.0045	0.7750	0.6887	-0.2837
	-0.3835	0.2772	-40.3137	
41.0600	-38.7600	-0.0979	0.0984	
0.4143	0.0316	0.8504	0.6827	-0.2768
	-0.3824	0.2790	-40.3145	
41.0800	-39.1400	-0.2154	-0.0678	
0.2683	0.0602	0.9215	0.6763	-0.2699
	-0.3811	0.2807	-40.3152	
41.1000	-39.0400	-0.0303	-0.0874	
0.0546	0.0915	0.9872	0.6697	-0.2630
	-0.3798	0.2824	-40.3160	
41.1200	-38.6200	0.4276	-0.1076	-
0.1697	0.1268	1.0469	0.6629	-0.2562
	-0.3784	0.2842	-40.3168	
41.1400	-39.5600	-0.3076	-0.1899	-
0.3530	0.1673	1.0996	0.6559	-0.2494
	-0.3770	0.2859	-40.3176	
41.1600	-39.3000	-0.0153	-0.1549	-
0.4667	0.2148	1.1444	0.6487	-0.2426
	-0.3755	0.2876	-40.3183	

41.1800 -39.0300 0.0174 0.0238 -
0.5066 0.2704 1.1807 0.6414 -0.2359
-0.3739 0.2894 -40.3191
41.2000 -38.6300 0.0913 0.1447 -
0.4898 0.3341 1.2076 0.6338 -0.2292
-0.3723 0.2911 -40.3199
41.2200 -38.4100 0.3816 0.0507 -
0.4400 0.4049 1.2246 0.6262 -0.2226
-0.3706 0.2928 -40.3207
41.2400 -39.1400 -0.3387 -0.1099 -
0.3737 0.4805 1.2308 0.6185 -0.2161
-0.3688 0.2946 -40.3214
41.2600 -38.8300 -0.3212 -0.0587 -
0.2942 0.5570 1.2258 0.6106 -0.2096
-0.3670 0.2963 -40.3222
41.2800 -37.8600 0.4472 0.1079 -
0.1918 0.6288 1.2090 0.6027 -0.2032
-0.3651 0.2981 -40.3230
41.3000 -38.3800 -0.1416 0.0476 -
0.0496 0.6895 1.1799 0.5948 -0.1968
-0.3631 0.2998 -40.3237
41.3200 -38.1800 0.1165 -0.2399
0.1315 0.7328 1.1381 0.5868 -0.1906
-0.3610 0.3016 -40.3245
41.3400 -38.7100 -0.3921 -0.3908
0.3276 0.7531 1.0834 0.5788 -0.1844
-0.3589 0.3033 -40.3252
41.3600 -37.5400 0.4100 -0.2383
0.5017 0.7458 1.0159 0.5708 -0.1784
-0.3568 0.3050 -40.3260
41.3800 -37.6400 0.0849 0.1135
0.6166 0.7064 0.9363 0.5628 -0.1724
-0.3545 0.3068 -40.3267
41.4000 -37.9000 -0.5437 0.4880
0.6357 0.6330 0.8455 0.5549 -0.1665
-0.3522 0.3085 -40.3275
41.4200 -37.4100 0.1626 0.6338
0.5311 0.5281 0.7447 0.5470 -0.1608
-0.3499 0.3103 -40.3283
41.4400 -37.5800 0.6953 0.3417
0.3108 0.3996 0.6353 0.5392 -0.1552
-0.3475 0.3120 -40.3290
41.4600 -40.0000 -0.6836 -0.2523
0.0346 0.2564 0.5185 0.5316 -0.1496
-0.3450 0.3138 -40.3298
41.4800 -40.4400 -0.2059 -0.5554 -
0.2219 0.1079 0.3955 0.5241 -0.1443
-0.3424 0.3156 -40.3305
41.5000 -40.3600 0.0864 -0.4052 -
0.3914 -0.0367 0.2677 0.5167 -0.1390
-0.3398 0.3173 -40.3312

41.5200 -40.0600 0.5963 -0.1871 -
0.4441 -0.1687 0.1363 0.5096 -0.1339
-0.3371 0.3191 -40.3320
41.5400 -40.5700 0.2282 -0.1228 -
0.3886 -0.2806 0.0026 0.5026 -0.1289
-0.3344 0.3208 -40.3327
41.5600 -41.5900 -0.7487 -0.0447 -
0.2780 -0.3674 -0.1322 0.4959 -0.1241
-0.3316 0.3226 -40.3335
41.5800 -41.0000 -0.4201 0.2385 -
0.1741 -0.4249 -0.2667 0.4894 -0.1194
-0.3287 0.3244 -40.3342
41.6000 -39.5100 0.9920 0.4753 -
0.1073 -0.4516 -0.4000 0.4831 -0.1148
-0.3258 0.3261 -40.3349
41.6200 -41.6300 -0.9097 0.3174 -
0.0848 -0.4491 -0.5308 0.4772 -0.1105
-0.3228 0.3279 -40.3357
41.6400 -40.4200 0.7836 -0.1453 -
0.0880 -0.4216 -0.6583 0.4715 -0.1062
-0.3197 0.3296 -40.3364
41.6600 -41.9000 -0.1711 -0.4980 -
0.0880 -0.3752 -0.7816 0.4662 -0.1022
-0.3166 0.3314 -40.3371
41.6800 -42.0400 -0.3431 -0.4297 -
0.0647 -0.3167 -0.8997 0.4612 -0.0983
-0.3134 0.3332 -40.3379
41.7000 -41.3800 -0.3314 0.1331 -
0.0133 -0.2528 -1.0115 0.4566 -0.0946
-0.3102 0.3350 -40.3386
41.7200 -39.9100 0.5734 0.6371
0.0585 -0.1887 -1.1162 0.4524 -0.0911
-0.3069 0.3367 -40.3393
41.7400 -40.5100 0.2525 0.4778
0.1408 -0.1287 -1.2129 0.4486 -0.0877
-0.3035 0.3385 -40.3401
41.7600 -42.1600 -0.8127 -0.2319
0.2143 -0.0766 -1.3006 0.4451 -0.0845
-0.3001 0.3403 -40.3408
41.7800 -40.9900 0.8760 -0.7868
0.2510 -0.0352 -1.3789 0.4422 -0.0815
-0.2966 0.3421 -40.3415
41.8000 -42.5100 -1.0874 -0.2485
0.2263 -0.0065 -1.4469 0.4397 -0.0787
-0.2931 0.3438 -40.3422
41.8200 -39.4600 1.1207 0.7180
0.1387 0.0091 -1.5045 0.4376 -0.0761
-0.2895 0.3456 -40.3429
41.8400 -41.4600 -0.7804 0.7396 -
0.0001 0.0118 -1.5512 0.4361 -0.0736
-0.2859 0.3474 -40.3437

41.8600 -41.9600 -0.3290 0.0551 -
0.1548 0.0021 -1.5869 0.4350 -0.0714
-0.2822 0.3492 -40.3444
41.8800 -41.9700 0.2635 -0.5669 -
0.2693 -0.0190 -1.6116 0.4345 -0.0693
-0.2784 0.3510 -40.3451
41.9000 -42.8700 -0.3779 -0.6646 -
0.2865 -0.0504 -1.6252 0.4345 -0.0674
-0.2746 0.3528 -40.3458
41.9200 -41.9000 0.3352 -0.3934 -
0.1849 -0.0904 -1.6280 0.4350 -0.0657
-0.2708 0.3546 -40.3465
41.9400 -42.1200 -0.4773 0.0328 -
0.0017 -0.1372 -1.6202 0.4361 -0.0642
-0.2669 0.3564 -40.3472
41.9600 -40.4700 0.5826 0.4345
0.1897 -0.1891 -1.6023 0.4376 -0.0628
-0.2629 0.3581 -40.3479
41.9800 -41.4600 -0.4552 0.4242
0.3124 -0.2439 -1.5747 0.4396 -0.0617
-0.2589 0.3599 -40.3486
42.0000 -40.7400 0.5200 0.0891
0.3123 -0.2991 -1.5379 0.4421 -0.0607
-0.2549 0.3617 -40.3493
42.0200 -42.2900 -0.7857 0.0020
0.1847 -0.3502 -1.4925 0.4449 -0.0598
-0.2508 0.3635 -40.3500
42.0400 -40.8800 0.8066 0.0603 -
0.0191 -0.3919 -1.4392 0.4481 -0.0592
-0.2466 0.3654 -40.3507
42.0600 -42.6900 -0.7270 -0.1333 -
0.2138 -0.4183 -1.3787 0.4517 -0.0587
-0.2424 0.3672 -40.3514
42.0800 -41.8400 0.4110 -0.3313 -
0.3309 -0.4232 -1.3119 0.4555 -0.0583
-0.2382 0.3690 -40.3521
42.1000 -42.3100 -0.0461 -0.3797 -
0.3402 -0.4031 -1.2396 0.4595 -0.0581
-0.2339 0.3708 -40.3528
42.1200 -41.9100 -0.0086 -0.2956 -
0.2604 -0.3579 -1.1627 0.4638 -0.0581
-0.2296 0.3726 -40.3535
42.1400 -41.6500 -0.2538 -0.0410 -
0.1430 -0.2902 -1.0820 0.4683 -0.0582
-0.2252 0.3744 -40.3542
42.1600 -40.0200 0.6737 0.3312 -
0.0452 -0.2041 -0.9983 0.4729 -0.0584
-0.2208 0.3762 -40.3549
42.1800 -41.0700 -0.9567 0.6663
0.0008 -0.1050 -0.9124 0.4775 -0.0588
-0.2163 0.3780 -40.3556

42.2000 -39.0200 1.2127 0.3109
0.0063 -0.0007 -0.8252 0.4823 -0.0593
-0.2118 0.3799 -40.3563
42.2200 -42.2200 -1.2258 -0.6089
0.0179 0.0998 -0.7373 0.4870 -0.0599
-0.2073 0.3817 -40.3569
42.2400 -40.3700 0.5279 -0.7862
0.0727 0.1879 -0.6496 0.4917 -0.0606
-0.2028 0.3835 -40.3576
42.2600 -40.0300 -0.0824 -0.1448
0.1779 0.2564 -0.5628 0.4964 -0.0615
-0.1982 0.3853 -40.3583
42.2800 -38.9800 0.1583 0.5070
0.2924 0.3011 -0.4777 0.5009 -0.0624
-0.1935 0.3872 -40.3590
42.3000 -38.4800 0.3091 0.7359
0.3651 0.3191 -0.3949 0.5053 -0.0634
-0.1889 0.3890 -40.3597
42.3200 -38.8100 0.1506 0.4583
0.3620 0.3090 -0.3150 0.5096 -0.0646
-0.1842 0.3908 -40.3603
42.3400 -39.6600 -0.1291 -0.0707
0.2882 0.2720 -0.2381 0.5135 -0.0658
-0.1795 0.3927 -40.3610
42.3600 -40.1600 -0.1787 -0.4430
0.1618 0.2124 -0.1641 0.5173 -0.0671
-0.1747 0.3945 -40.3617
42.3800 -40.1400 -0.0204 -0.4143
0.0039 0.1358 -0.0929 0.5207 -0.0685
-0.1699 0.3964 -40.3623
42.4000 -39.6900 0.1961 -0.1045 -
0.1532 0.0485 -0.0241 0.5237 -0.0699
-0.1651 0.3982 -40.3630
42.4200 -40.0200 -0.2045 0.1978 -
0.2705 -0.0431 0.0426 0.5264 -0.0714
-0.1603 0.4000 -40.3637
42.4400 -39.3300 0.4007 0.2259 -
0.3191 -0.1324 0.1074 0.5286 -0.0730
-0.1554 0.4019 -40.3643
42.4600 -40.3800 -0.2938 -0.0588 -
0.2932 -0.2137 0.1708 0.5304 -0.0746
-0.1505 0.4038 -40.3650
42.4800 -39.9900 0.2736 -0.3426 -
0.2085 -0.2829 0.2331 0.5316 -0.0763
-0.1456 0.4056 -40.3657
42.5000 -40.5300 -0.5504 -0.2425 -
0.0920 -0.3369 0.2946 0.5322 -0.0780
-0.1407 0.4075 -40.3663
42.5200 -39.6100 -0.1232 0.1374
0.0205 -0.3733 0.3558 0.5323 -0.0797
-0.1357 0.4093 -40.3670

42.5400 -38.4900 0.7255 0.3770
0.0904 -0.3904 0.4170 0.5317 -0.0815
-0.1308 0.4112 -40.3676
42.5600 -39.7400 -0.5407 0.2160
0.0889 -0.3878 0.4785 0.5304 -0.0834
-0.1258 0.4131 -40.3683
42.5800 -39.5500 -0.0805 -0.0201
0.0209 -0.3671 0.5406 0.5284 -0.0852
-0.1208 0.4149 -40.3689
42.6000 -39.4700 0.1403 -0.1163 -
0.0815 -0.3321 0.6034 0.5256 -0.0871
-0.1158 0.4168 -40.3696
42.6200 -39.3700 0.2048 -0.2323 -
0.1682 -0.2877 0.6670 0.5219 -0.0890
-0.1107 0.4187 -40.3702
42.6400 -39.7700 -0.1119 -0.3299 -
0.1981 -0.2392 0.7316 0.5175 -0.0909
-0.1057 0.4205 -40.3709
42.6600 -39.6000 -0.2623 -0.2058 -
0.1576 -0.1914 0.7972 0.5121 -0.0928
-0.1006 0.4224 -40.3715
42.6800 -38.7600 0.2382 0.0444 -
0.0576 -0.1485 0.8640 0.5059 -0.0947
-0.0955 0.4243 -40.3722
42.7000 -38.2800 0.3236 0.1632
0.0716 -0.1138 0.9319 0.4987 -0.0967
-0.0905 0.4262 -40.3728
42.7200 -38.9800 -0.5874 0.1569
0.1898 -0.0900 1.0009 0.4904 -0.0986
-0.0854 0.4281 -40.3735
42.7400 -37.8900 0.2708 0.2322
0.2637 -0.0794 1.0708 0.4812 -0.1005
-0.0803 0.4300 -40.3741
42.7600 -37.8900 0.1346 0.3160
0.2780 -0.0829 1.1411 0.4709 -0.1024
-0.0752 0.4319 -40.3747
42.7800 -37.8500 0.3049 0.1367
0.2378 -0.0989 1.2111 0.4595 -0.1043
-0.0701 0.4338 -40.3754
42.8000 -38.8700 -0.2465 -0.2220
0.1683 -0.1244 1.2802 0.4469 -0.1062
-0.0649 0.4357 -40.3760
42.8200 -39.0800 -0.2598 -0.4057
0.0927 -0.1549 1.3472 0.4332 -0.1081
-0.0598 0.4376 -40.3766
42.8400 -38.5000 0.1983 -0.2494
0.0231 -0.1852 1.4112 0.4182 -0.1099
-0.0547 0.4395 -40.3773
42.8600 -38.3800 0.0812 0.0849 -
0.0429 -0.2094 1.4711 0.4021 -0.1118
-0.0496 0.4414 -40.3779

42.8800 -38.4800 -0.2705 0.3149 -
0.1128 -0.2221 1.5258 0.3846 -0.1136
-0.0444 0.4433 -40.3785
42.9000 -37.7800 0.5508 0.2309 -
0.1848 -0.2184 1.5744 0.3659 -0.1153
-0.0393 0.4452 -40.3791
42.9200 -39.2100 -0.6543 -0.0353 -
0.2446 -0.1951 1.6157 0.3458 -0.1171
-0.0342 0.4471 -40.3798
42.9400 -38.5200 0.2973 -0.1976 -
0.2739 -0.1503 1.6491 0.3245 -0.1187
-0.0291 0.4490 -40.3804
42.9600 -38.2400 0.4486 -0.2229 -
0.2549 -0.0846 1.6738 0.3017 -0.1204
-0.0240 0.4510 -40.3810
42.9800 -38.9900 -0.5448 -0.1470 -
0.1809 -0.0003 1.6894 0.2777 -0.1220
-0.0188 0.4529 -40.3816
43.0000 -37.6600 0.5065 -0.0068 -
0.0650 0.0986 1.6956 0.2523 -0.1235
-0.0137 0.4548 -40.3822
43.0200 -38.0800 -0.1776 0.0895
0.0625 0.2082 1.6920 0.2255 -0.1250
-0.0086 0.4567 -40.3828
43.0400 -37.6900 -0.1643 0.1268
0.1649 0.3238 1.6783 0.1975 -0.1264
-0.0035 0.4587 -40.3835
43.0600 -37.7000 -0.1915 0.1982
0.2108 0.4406 1.6541 0.1683 -0.1277
0.0015 0.4606 -40.3841
43.0800 -36.7900 0.4931 0.1630
0.1890 0.5530 1.6192 0.1380 -0.1290
0.0066 0.4626 -40.3847
43.1000 -37.9500 -0.2550 -0.0891
0.1109 0.6552 1.5733 0.1065 -0.1302
0.0117 0.4645 -40.3853
43.1200 -38.1700 -0.0971 -0.2592
0.0065 0.7416 1.5162 0.0741 -0.1312
0.0167 0.4665 -40.3859
43.1400 -38.1700 -0.2440 -0.1170 -
0.0902 0.8071 1.4478 0.0407 -0.1322
0.0218 0.4684 -40.3865
43.1600 -37.9100 -0.0628 0.1710 -
0.1503 0.8470 1.3683 0.0065 -0.1331
0.0268 0.4704 -40.3871
43.1800 -37.4400 0.4567 0.2438 -
0.1480 0.8572 1.2780 -0.0285 -0.1339
0.0318 0.4723 -40.3877
43.2000 -38.3700 -0.2426 -0.0293 -
0.0736 0.8351 1.1778 -0.0641 -0.1346
0.0368 0.4743 -40.3883

43.2200	-38.6100	-0.0945	-0.3163	
0.0528	0.7803	1.0686	-0.1003	-0.1352
	0.0418	0.4762	-40.3889	
43.2400	-38.6000	0.0721	-0.3298	
0.1997	0.6939	0.9517	-0.1370	-0.1356
	0.0467	0.4782	-40.3895	
43.2600	-38.2100	0.2509	-0.0461	
0.3237	0.5780	0.8282	-0.1741	-0.1359
	0.0516	0.4802	-40.3901	
43.2800	-38.9300	-0.6392	0.3900	
0.3780	0.4364	0.6996	-0.2116	-0.1361
	0.0566	0.4822	-40.3907	
43.3000	-37.6900	0.8334	0.4835	
0.3300	0.2744	0.5671	-0.2493	-0.1362
	0.0615	0.4841	-40.3912	
43.3200	-39.1200	0.4174	0.0386	
0.1868	0.0991	0.4321	-0.2872	-0.1361
	0.0663	0.4861	-40.3918	
43.3400	-41.5200	-0.9369	-0.4167	
0.0020	-0.0811	0.2959	-0.3252	-0.1359
	0.0712	0.4881	-40.3924	
43.3600	-40.2100	0.7025	-0.3076	-
0.1622	-0.2575	0.1598	-0.3632	-0.1355
	0.0760	0.4901	-40.3930	
43.3800	-41.1400	-0.1702	0.0477	-
0.2642	-0.4216	0.0251	-0.4012	-0.1349
	0.0808	0.4921	-40.3936	
43.4000	-40.9600	0.1617	0.1322	-
0.2896	-0.5656	-0.1068	-0.4389	-0.1342
	0.0856	0.4941	-40.3942	
43.4200	-41.6600	-0.1610	0.0406	-
0.2526	-0.6832	-0.2346	-0.4764	-0.1334
	0.0903	0.4961	-40.3947	
43.4400	-41.6300	0.1457	-0.0545	-
0.1825	-0.7696	-0.3570	-0.5137	-0.1323
	0.0951	0.4981	-40.3953	
43.4600	-42.1600	-0.2140	-0.0611	-
0.1085	-0.8220	-0.4728	-0.5505	-0.1311
	0.0998	0.5001	-40.3959	
43.4800	-41.8800	0.0146	0.0815	-
0.0491	-0.8397	-0.5808	-0.5868	-0.1297
	0.1044	0.5021	-40.3965	
43.5000	-42.1800	-0.3020	0.2061	-
0.0118	-0.8243	-0.6800	-0.6225	-0.1281
	0.1091	0.5041	-40.3970	
43.5200	-41.1100	1.0023	0.0512	
0.0069	-0.7790	-0.7700	-0.6576	-0.1263
	0.1137	0.5061	-40.3976	
43.5400	-43.1300	-0.8366	-0.2037	
0.0182	-0.7078	-0.8502	-0.6920	-0.1244
	0.1183	0.5082	-40.3982	

43.5600 -43.3800 -1.1015 -0.2328
0.0347 -0.6156 -0.9206 -0.7255 -0.1222
0.1228 0.5102 -40.3987
43.5800 -41.3400 0.8572 0.0100
0.0593 -0.5075 -0.9811 -0.7581 -0.1198
0.1273 0.5122 -40.3993
43.6000 -40.8100 1.1066 0.1927
0.0851 -0.3880 -1.0318 -0.7898 -0.1172
0.1318 0.5142 -40.3999
43.6200 -42.6800 -0.8904 0.1046
0.1015 -0.2614 -1.0732 -0.8204 -0.1144
0.1362 0.5163 -40.4004
43.6400 -42.3300 -0.4030 -0.0507
0.0906 -0.1315 -1.1056 -0.8498 -0.1113
0.1406 0.5183 -40.4010
43.6600 -41.3000 0.5591 -0.0064
0.0439 -0.0014 -1.1293 -0.8781 -0.1080
0.1450 0.5204 -40.4015
43.6800 -42.0600 -0.4982 0.2567 -
0.0297 0.1256 -1.1450 -0.9050 -0.1045
0.1493 0.5224 -40.4021
43.7000 -40.9600 0.6317 0.1928 -
0.1034 0.2463 -1.1530 -0.9305 -0.1008
0.1536 0.5245 -40.4027
43.7200 -42.5200 -0.5555 -0.2410 -
0.1439 0.3572 -1.1538 -0.9545 -0.0968
0.1579 0.5265 -40.4032
43.7400 -41.7600 0.2754 -0.4089 -
0.1285 0.4547 -1.1480 -0.9770 -0.0925
0.1621 0.5286 -40.4038
43.7600 -41.6600 0.0880 -0.2836 -
0.0516 0.5355 -1.1359 -0.9979 -0.0881
0.1663 0.5306 -40.4043
43.7800 -41.0400 0.2994 -0.1285
0.0640 0.5962 -1.1179 -1.0171 -0.0833
0.1705 0.5327 -40.4049
43.8000 -41.7200 -0.7883 0.1837
0.1672 0.6338 -1.0945 -1.0345 -0.0783
0.1746 0.5348 -40.4054
43.8200 -40.2000 0.2909 0.5231
0.2107 0.6464 -1.0659 -1.0502 -0.0730
0.1786 0.5368 -40.4059
43.8400 -39.7000 0.8867 0.5127
0.1772 0.6334 -1.0325 -1.0642 -0.0675
0.1827 0.5389 -40.4065
43.8600 -41.6200 -0.4530 0.0575
0.0842 0.5967 -0.9948 -1.0763 -0.0617
0.1867 0.5410 -40.4070
43.8800 -42.3300 -0.7099 -0.3810 -
0.0256 0.5396 -0.9530 -1.0866 -0.0556
0.1906 0.5431 -40.4076

43.9000 -41.9600 -0.2139 -0.4038 -
0.1100 0.4663 -0.9077 -1.0951 -0.0492
0.1945 0.5452 -40.4081
43.9200 -41.2700 0.2266 -0.1306 -
0.1459 0.3812 -0.8592 -1.1019 -0.0426
0.1984 0.5473 -40.4086
43.9400 -40.8000 0.4763 0.0904 -
0.1303 0.2890 -0.8081 -1.1069 -0.0358
0.2022 0.5494 -40.4092
43.9600 -41.6200 -0.2785 0.0976 -
0.0777 0.1938 -0.7545 -1.1102 -0.0287
0.2059 0.5515 -40.4097
43.9800 -41.4300 -0.1322 0.0501 -
0.0145 0.0996 -0.6989 -1.1120 -0.0213
0.2097 0.5536 -40.4102
44.0000 -41.6600 -0.3483 0.1204
0.0344 0.0095 -0.6415 -1.1122 -0.0137
0.2133 0.5557 -40.4108
44.0200 -41.1800 0.1593 0.1124
0.0529 -0.0744 -0.5825 -1.1109 -0.0058
0.2170 0.5578 -40.4113
44.0400 -40.6000 0.8951 -0.0931
0.0404 -0.1508 -0.5218 -1.1083 0.0023
0.2206 0.5599 -40.4118
44.0600 -42.6400 -1.0596 -0.1946
0.0136 -0.2197 -0.4595 -1.1044 0.0106
0.2241 0.5620 -40.4123
44.0800 -40.3000 0.9844 0.1232 -
0.0027 -0.2811 -0.3954 -1.0992 0.0191
0.2276 0.5642 -40.4128
44.1000 -41.9800 -0.7521 0.1865
0.0073 -0.3354 -0.3296 -1.0930 0.0279
0.2311 0.5663 -40.4134
44.1200 -41.0900 0.3728 -0.1609
0.0377 -0.3822 -0.2619 -1.0856 0.0369
0.2345 0.5684 -40.4139
44.1400 -41.1400 0.2813 -0.2595
0.0629 -0.4200 -0.1926 -1.0773 0.0461
0.2378 0.5706 -40.4144
44.1600 -41.8000 -0.8000 0.0635
0.0618 -0.4469 -0.1220 -1.0681 0.0555
0.2411 0.5727 -40.4149
44.1800 -40.5300 0.2166 0.4080
0.0268 -0.4601 -0.0507 -1.0581 0.0650
0.2444 0.5749 -40.4154
44.2000 -39.7800 0.9312 0.3167 -
0.0380 -0.4569 0.0208 -1.0473 0.0748
0.2476 0.5770 -40.4159
44.2200 -42.0900 -0.9363 -0.2660 -
0.1081 -0.4349 0.0918 -1.0359 0.0848
0.2508 0.5792 -40.4164

44.2400 −40.8200 0.5006 −0.4659 −
0.1513 −0.3926 0.1617 −1.0238 0.0950
0.2539 0.5813 −40.4169
44.2600 −41.2000 −0.5163 0.0448 −
0.1476 −0.3298 0.2299 −1.0112 0.1053
0.2569 0.5835 −40.4174
44.2800 −39.3900 0.6718 0.4374 −
0.1187 −0.2477 0.2956 −0.9981 0.1159
0.2599 0.5857 −40.4179
44.3000 −40.4300 −0.3097 0.1997 −
0.0927 −0.1497 0.3581 −0.9846 0.1266
0.2629 0.5878 −40.4184
44.3200 −40.1800 0.1123 −0.2579 −
0.0791 −0.0403 0.4166 −0.9708 0.1374
0.2658 0.5900 −40.4189
44.3400 −40.3000 −0.0855 −0.3440 −
0.0760 0.0739 0.4704 −0.9567 0.1485
0.2686 0.5922 −40.4194
44.3600 −40.1100 −0.3426 −0.0262 −
0.0634 0.1853 0.5186 −0.9424 0.1596
0.2714 0.5944 −40.4199
44.3800 −38.5900 0.6362 0.2640 −
0.0149 0.2855 0.5606 −0.9279 0.1710
0.2742 0.5966 −40.4204
44.4000 −39.5400 −0.4538 0.1033
0.0804 0.3667 0.5954 −0.9134 0.1824
0.2769 0.5988 −40.4209
44.4200 −39.2500 −0.0813 −0.2604
0.2013 0.4229 0.6224 −0.8989 0.1940
0.2795 0.6010 −40.4214
44.4400 −38.7400 0.3253 −0.3339
0.3088 0.4492 0.6409 −0.8844 0.2058
0.2821 0.6032 −40.4219
44.4600 −39.2500 −0.6462 0.0945
0.3613 0.4425 0.6505 −0.8701 0.2176
0.2846 0.6054 −40.4224
44.4800 −37.5600 0.7752 0.5053
0.3278 0.4022 0.6513 −0.8559 0.2296
0.2871 0.6076 −40.4229
44.5000 −39.4100 −0.6688 0.2667
0.1970 0.3329 0.6440 −0.8420 0.2418
0.2895 0.6098 −40.4234
44.5200 −38.9400 0.5362 −0.2678
0.0082 0.2426 0.6292 −0.8282 0.2540
0.2919 0.6121 −40.4238
44.5400 −40.5100 −0.5289 −0.4738 −
0.1741 0.1405 0.6079 −0.8148 0.2663
0.2942 0.6143 −40.4243
44.5600 −39.5400 0.3179 −0.1886 −
0.2928 0.0358 0.5808 −0.8015 0.2788
0.2965 0.6165 −40.4248

44.5800 -39.9700 -0.3430 0.2894 -
0.3295 -0.0631 0.5488 -0.7885 0.2913
0.2987 0.6188 -40.4253
44.6000 -38.9300 0.6301 0.2932 -
0.2853 -0.1488 0.5129 -0.7758 0.3039
0.3008 0.6210 -40.4257
44.6200 -40.7300 -0.6637 -0.1538 -
0.1820 -0.2166 0.4738 -0.7632 0.3166
0.3029 0.6233 -40.4262
44.6400 -39.6500 0.4698 -0.3089 -
0.0611 -0.2643 0.4325 -0.7509 0.3293
0.3049 0.6255 -40.4267
44.6600 -40.3200 -0.4845 -0.0222
0.0385 -0.2908 0.3896 -0.7388 0.3421
0.3068 0.6278 -40.4272
44.6800 -39.0300 0.4948 0.2564
0.0897 -0.2956 0.3459 -0.7268 0.3550
0.3087 0.6300 -40.4276
44.7000 -39.7100 -0.1770 0.2613
0.0764 -0.2804 0.3021 -0.7151 0.3678
0.3106 0.6323 -40.4281
44.7200 -39.6200 0.1184 0.0253
0.0134 -0.2492 0.2587 -0.7035 0.3807
0.3124 0.6346 -40.4286
44.7400 -40.2800 -0.0948 -0.2351 -
0.0626 -0.2075 0.2160 -0.6921 0.3937
0.3141 0.6369 -40.4290
44.7600 -40.1500 -0.1297 -0.2625 -
0.1087 -0.1610 0.1747 -0.6808 0.4066
0.3158 0.6391 -40.4295
44.7800 -39.5800 0.2017 -0.0653 -
0.0996 -0.1150 0.1350 -0.6697 0.4195
0.3173 0.6414 -40.4299
44.8000 -39.5000 0.0657 0.1106 -
0.0388 -0.0735 0.0973 -0.6586 0.4323
0.3189 0.6437 -40.4304
44.8200 -39.6700 -0.2054 0.1087
0.0548 -0.0391 0.0620 -0.6476 0.4452
0.3204 0.6460 -40.4308
44.8400 -39.4000 0.0113 0.0134
0.1511 -0.0129 0.0294 -0.6366 0.4579
0.3218 0.6483 -40.4313
44.8600 -39.3300 0.1352 -0.0546
0.2174 0.0051 -0.0006 -0.6257 0.4707
0.3231 0.6506 -40.4317
44.8800 -39.5500 -0.1743 -0.0196
0.2299 0.0158 -0.0277 -0.6146 0.4833
0.3244 0.6529 -40.4322
44.9000 -39.0500 0.1759 0.1474
0.1766 0.0210 -0.0522 -0.6035 0.4958
0.3256 0.6552 -40.4326

44.9200	-39.3400	-0.0915	0.2661	
0.0678	0.0239	-0.0743	-0.5922	0.5083
	0.3268	0.6576	-40.4331	
44.9400	-39.4800	0.0286	0.1403	-
0.0678	0.0274	-0.0944	-0.5807	0.5206
	0.3279	0.6599	-40.4335	
44.9600	-39.5700	0.4353	-0.1934	-
0.1924	0.0344	-0.1127	-0.5689	0.5328
	0.3289	0.6622	-40.4340	
44.9800	-40.8300	-0.6857	-0.3851	-
0.2650	0.0469	-0.1295	-0.5568	0.5449
	0.3299	0.6646	-40.4344	
45.0000	-39.5600	0.3606	-0.2134	-
0.2562	0.0663	-0.1452	-0.5444	0.5568
	0.3308	0.6669	-40.4349	
45.0200	-39.1800	0.4987	0.0178	-
0.1740	0.0916	-0.1597	-0.5316	0.5685
	0.3316	0.6692	-40.4353	
45.0400	-39.7400	-0.3583	0.0879	-
0.0471	0.1201	-0.1732	-0.5182	0.5801
	0.3324	0.6716	-40.4357	
45.0600	-39.3800	-0.1988	0.1183	
0.0860	0.1484	-0.1855	-0.5044	0.5914
	0.3331	0.6739	-40.4362	
45.0800	-38.4900	0.4433	0.1777	
0.1907	0.1729	-0.1964	-0.4900	0.6026
	0.3338	0.6763	-40.4366	
45.1000	-39.2100	-0.4063	0.1604	
0.2434	0.1898	-0.2057	-0.4750	0.6135
	0.3344	0.6787	-40.4370	
45.1200	-38.7400	0.2092	0.0275	
0.2389	0.1955	-0.2131	-0.4594	0.6242
	0.3349	0.6810	-40.4374	
45.1400	-38.7600	0.3729	-0.1219	
0.1945	0.1873	-0.2183	-0.4431	0.6347
	0.3353	0.6834	-40.4379	
45.1600	-39.8100	-0.5177	-0.1597	
0.1364	0.1642	-0.2214	-0.4261	0.6449
	0.3357	0.6858	-40.4383	
45.1800	-39.8700	-0.6284	-0.0368	
0.0883	0.1264	-0.2220	-0.4084	0.6549
	0.3360	0.6882	-40.4387	
45.2000	-38.1200	1.0234	0.1011	
0.0622	0.0761	-0.2201	-0.3900	0.6646
	0.3363	0.6906	-40.4391	
45.2200	-40.2000	-0.9864	0.0396	
0.0546	0.0173	-0.2157	-0.3708	0.6740
	0.3365	0.6930	-40.4396	
45.2400	-39.1300	0.1824	0.0008	
0.0476	-0.0452	-0.2088	-0.3509	0.6831
	0.3366	0.6954	-40.4400	

45.2600	-38.8400	0.5223	0.0732	
0.0190	-0.1058	-0.1994	-0.3301	0.6919
	0.3366	0.6978	-40.4404	
45.2800	-39.4000	-0.0948	0.1109	-
0.0409	-0.1590	-0.1874	-0.3084	0.7005
	0.3366	0.7002	-40.4408	
45.3000	-39.4300	0.0390	0.0332	-
0.1274	-0.2001	-0.1728	-0.2859	0.7087
	0.3365	0.7026	-40.4412	
45.3200	-39.6000	-0.0311	-0.0740	-
0.2229	-0.2256	-0.1558	-0.2625	0.7166
	0.3364	0.7050	-40.4416	
45.3400	-39.6300	0.0348	-0.0892	-
0.2988	-0.2331	-0.1364	-0.2381	0.7242
	0.3362	0.7074	-40.4420	
45.3600	-39.8600	-0.3008	-0.0516	-
0.3238	-0.2213	-0.1149	-0.2128	0.7314
	0.3359	0.7098	-40.4424	
45.3800	-39.1000	0.4798	-0.0820	-
0.2803	-0.1913	-0.0919	-0.1865	0.7383
	0.3355	0.7123	-40.4428	
45.4000	-39.7400	-0.3744	-0.1801	-
0.1687	-0.1462	-0.0679	-0.1592	0.7449
	0.3351	0.7147	-40.4432	
45.4200	-39.0800	0.0378	-0.1393	-
0.0113	-0.0907	-0.0436	-0.1310	0.7512
	0.3346	0.7172	-40.4436	
45.4400	-38.7200	-0.0522	0.0440	
0.1510	-0.0297	-0.0197	-0.1017	0.7570
	0.3341	0.7196	-40.4440	
45.4600	-38.1300	0.1226	0.1876	
0.2739	0.0316	0.0033	-0.0713	0.7626
	0.3335	0.7221	-40.4444	
45.4800	-37.9700	0.1012	0.2217	
0.3226	0.0882	0.0246	-0.0399	0.7678
	0.3328	0.7245	-40.4448	
45.5000	-38.1300	-0.1403	0.1907	
0.2808	0.1360	0.0435	-0.0075	0.7726
	0.3321	0.7270	-40.4452	
45.5200	-38.2300	-0.1130	0.1039	
0.1665	0.1720	0.0595	0.0261	0.7770
	0.3312	0.7294	-40.4456	
45.5400	-37.7900	0.4818	-0.0971	
0.0267	0.1947	0.0718	0.0607	0.7811
	0.3304	0.7319	-40.4460	
45.5600	-39.1900	-0.5499	-0.3083	-
0.0878	0.2032	0.0799	0.0963	0.7848
	0.3294	0.7344	-40.4464	
45.5800	-38.2500	0.4064	-0.2534	-
0.1386	0.1971	0.0835	0.1330	0.7882
	0.3284	0.7369	-40.4468	

45.6000	-38.7100	-0.3885	0.0331	-
0.1191	0.1771	0.0826	0.1707	0.7912
	0.3273	0.7393	-40.4472	
45.6200	-37.5500	0.4310	0.2179	-
0.0521	0.1453	0.0770	0.2094	0.7938
	0.3262	0.7418	-40.4476	
45.6400	-37.9800	0.0250	0.1520	
0.0306	0.1051	0.0672	0.2489	0.7960
	0.3249	0.7443	-40.4479	
45.6600	-38.4400	-0.3606	0.0030	
0.0999	0.0607	0.0533	0.2892	0.7978
	0.3237	0.7468	-40.4483	
45.6800	-37.9800	0.0778	-0.0600	
0.1357	0.0157	0.0358	0.3302	0.7992
	0.3223	0.7493	-40.4487	
45.7000	-37.9500	0.2699	-0.0600	
0.1294	-0.0264	0.0150	0.3719	0.8002
	0.3209	0.7518	-40.4491	
45.7200	-38.4900	-0.2918	-0.0337	
0.0835	-0.0630	-0.0085	0.4140	0.8009
	0.3194	0.7543	-40.4494	
45.7400	-38.0200	0.2257	0.0458	
0.0071	-0.0925	-0.0342	0.4565	0.8011
	0.3179	0.7569	-40.4498	
45.7600	-38.6100	-0.3944	0.1569	-
0.0815	-0.1139	-0.0615	0.4993	0.8009
	0.3162	0.7594	-40.4502	
45.7800	-37.9500	0.2995	0.1133	-
0.1525	-0.1261	-0.0898	0.5423	0.8003
	0.3146	0.7619	-40.4506	
45.8000	-38.1000	0.5511	-0.1236	-
0.1795	-0.1287	-0.1185	0.5853	0.7993
	0.3128	0.7644	-40.4509	
45.8200	-39.4900	-0.8079	-0.3097	-
0.1476	-0.1220	-0.1470	0.6282	0.7979
	0.3110	0.7670	-40.4513	
45.8400	-37.9000	0.4871	-0.0966	-
0.0601	-0.1074	-0.1749	0.6710	0.7960
	0.3091	0.7695	-40.4516	
45.8600	-38.0000	0.0144	0.2178	
0.0475	-0.0867	-0.2014	0.7133	0.7937
	0.3072	0.7720	-40.4520	
45.8800	-37.5600	0.0817	0.2665	
0.1309	-0.0620	-0.2262	0.7552	0.7910
	0.3051	0.7746	-40.4524	
45.9000	-37.6900	0.0263	0.1130	
0.1613	-0.0349	-0.2487	0.7965	0.7879
	0.3031	0.7771	-40.4527	
45.9200	-38.0800	-0.1792	-0.0182	
0.1336	-0.0068	-0.2684	0.8370	0.7843
	0.3009	0.7797	-40.4531	

45.9400 -37.8300 0.1181 -0.0424
0.0615 0.0214 -0.2848 0.8767 0.7803
0.2987 0.7822 -40.4534
45.9600 -37.7900 0.1305 -0.0576 -
0.0255 0.0489 -0.2975 0.9155 0.7758
0.2964 0.7848 -40.4538
45.9800 -38.3100 -0.1950 -0.1267 -
0.0940 0.0753 -0.3062 0.9532 0.7709
0.2941 0.7874 -40.4541
46.0000 -38.1000 0.0604 -0.1779 -
0.1212 0.0997 -0.3105 0.9897 0.7656
0.2917 0.7900 -40.4545
46.0200 -38.0300 0.0025 -0.1049 -
0.1055 0.1215 -0.3101 1.0249 0.7598
0.2893 0.7925 -40.4548
46.0400 -37.9100 -0.2720 0.0803 -
0.0618 0.1399 -0.3047 1.0587 0.7535
0.2867 0.7951 -40.4552
46.0600 -37.1000 0.3185 0.2135 -
0.0113 0.1541 -0.2941 1.0911 0.7468
0.2841 0.7977 -40.4555
46.0800 -37.4700 -0.0264 0.1378
0.0298 0.1627 -0.2781 1.1220 0.7396
0.2815 0.8003 -40.4559
46.1000 -37.6200 -0.0651 -0.0792
0.0539 0.1644 -0.2566 1.1512 0.7319
0.2788 0.8029 -40.4562
46.1200 -37.7100 -0.0115 -0.2012
0.0649 0.1577 -0.2295 1.1786 0.7238
0.2760 0.8055 -40.4565
46.1400 -37.7000 -0.1098 -0.1130
0.0705 0.1414 -0.1969 1.2043 0.7152
0.2732 0.8081 -40.4569
46.1600 -37.0300 0.2375 0.0382
0.0751 0.1152 -0.1591 1.2281 0.7061
0.2704 0.8107 -40.4572
46.1800 -37.4100 -0.1158 0.0989
0.0735 0.0790 -0.1163 1.2500 0.6965
0.2674 0.8133 -40.4575
46.2000 -37.5300 -0.1768 0.1083
0.0583 0.0335 -0.0688 1.2697 0.6865
0.2644 0.8159 -40.4579
46.2200 -37.1200 0.1568 0.1258
0.0260 -0.0199 -0.0172 1.2874 0.6760
0.2614 0.8185 -40.4582
46.2400 -37.5000 -0.1038 0.0726 -
0.0149 -0.0790 0.0379 1.3028 0.6650
0.2583 0.8211 -40.4585
46.2600 -37.5000 0.1194 -0.0922 -
0.0514 -0.1413 0.0958 1.3158 0.6535
0.2552 0.8238 -40.4588

46.2800 -37.6500 0.2077 -0.2940 -
0.0704 -0.2043 0.1559 1.3265 0.6415
0.2520 0.8264 -40.4592
46.3000 -38.1600 -0.3569 -0.3321 -
0.0644 -0.2651 0.2173 1.3347 0.6291
0.2487 0.8290 -40.4595
46.3200 -37.6700 -0.1369 -0.0617 -
0.0367 -0.3216 0.2792 1.3403 0.6162
0.2454 0.8317 -40.4598
46.3400 -36.7900 0.2453 0.3221 -
0.0003 -0.3715 0.3407 1.3432 0.6028
0.2421 0.8343 -40.4601
46.3600 -36.6200 0.2778 0.4768
0.0336 -0.4126 0.4010 1.3434 0.5889
0.2387 0.8369 -40.4604
46.3800 -37.4100 -0.3168 0.2829
0.0604 -0.4429 0.4594 1.3408 0.5746
0.2353 0.8396 -40.4608
46.4000 -37.0900 0.3045 -0.1082
0.0838 -0.4605 0.5150 1.3352 0.5599
0.2318 0.8422 -40.4611
46.4200 -37.7600 -0.0531 -0.4567
0.1097 -0.4632 0.5671 1.3266 0.5447
0.2283 0.8449 -40.4614
46.4400 -38.0500 -0.2452 -0.5094
0.1388 -0.4493 0.6151 1.3150 0.5291
0.2248 0.8476 -40.4617
46.4600 -37.0900 0.1939 -0.1625
0.1621 -0.4166 0.6583 1.3002 0.5131
0.2212 0.8502 -40.4620
46.4800 -37.1000 -0.3606 0.3354
0.1561 -0.3633 0.6961 1.2821 0.4967
0.2175 0.8529 -40.4623
46.5000 -36.1000 0.5251 0.5384
0.0934 -0.2883 0.7279 1.2609 0.4800
0.2138 0.8556 -40.4626
46.5200 -36.9300 -0.1182 0.2863 -
0.0388 -0.1919 0.7531 1.2363 0.4629
0.2101 0.8582 -40.4629
46.5400 -37.7800 -0.4353 -0.1086 -
0.2156 -0.0768 0.7713 1.2085 0.4455
0.2064 0.8609 -40.4632
46.5600 -37.3200 0.3341 -0.3108 -
0.3944 0.0530 0.7821 1.1775 0.4277
0.2026 0.8636 -40.4635
46.5800 -37.8600 -0.1895 -0.2968 -
0.5289 0.1928 0.7850 1.1433 0.4097
0.1988 0.8663 -40.4638
46.6000 -37.3400 0.1772 -0.2035 -
0.5830 0.3372 0.7799 1.1059 0.3913
0.1949 0.8689 -40.4641

46.6200	-37.5500	-0.1538	-0.1110	-
0.5446	0.4808	0.7663	1.0656	0.3726
	0.1910	0.8716	-40.4644	
46.6400	-36.7800	0.2613	-0.0011	-
0.4271	0.6180	0.7439	1.0224	0.3537
	0.1871	0.8743	-40.4647	
46.6600	-37.1400	-0.4185	0.1228	-
0.2530	0.7426	0.7124	0.9765	0.3346
	0.1831	0.8770	-40.4650	
46.6800	-36.2300	0.3554	0.1230	-
0.0365	0.8475	0.6717	0.9281	0.3152
	0.1791	0.8797	-40.4653	
46.7000	-36.5300	0.0915	-0.0813	
0.2106	0.9247	0.6216	0.8773	0.2956
	0.1751	0.8824	-40.4655	
46.7200	-36.9400	-0.3188	-0.3134	
0.4643	0.9662	0.5625	0.8243	0.2757
	0.1711	0.8851	-40.4658	
46.7400	-36.4500	0.1099	-0.3326	
0.6910	0.9644	0.4948	0.7694	0.2557
	0.1670	0.8878	-40.4661	
46.7600	-36.4700	-0.0567	-0.1002	
0.8496	0.9142	0.4192	0.7126	0.2355
	0.1629	0.8905	-40.4664	
46.7800	-35.9900	0.1143	0.3208	
0.8997	0.8140	0.3369	0.6542	0.2152
	0.1588	0.8933	-40.4667	
46.8000	-36.3600	-0.2411	0.7755	
0.8083	0.6677	0.2492	0.5944	0.1947
	0.1547	0.8960	-40.4669	
46.8200	-36.1100	0.4985	0.9632	
0.5675	0.4835	0.1574	0.5332	0.1740
	0.1505	0.8987	-40.4672	
46.8400	-37.9500	-0.2867	0.6489	
0.2099	0.2727	0.0628	0.4710	0.1533
	0.1463	0.9014	-40.4675	
46.8600	-39.3500	-0.1681	-0.0785	-
0.1904	0.0473	-0.0333	0.4079	0.1324
	0.1421	0.9041	-40.4678	
46.8800	-40.0000	0.7277	-0.8512	-
0.5480	-0.1807	-0.1295	0.3440	0.1115
	0.1379	0.9069	-40.4680	
46.9000	-42.4900	-1.0099	-1.0491	-
0.7814	-0.3995	-0.2246	0.2796	0.0905
	0.1336	0.9096	-40.4683	
46.9200	-40.6600	0.6766	-0.4802	-
0.8428	-0.5976	-0.3171	0.2148	0.0694
	0.1294	0.9123	-40.4686	
46.9400	-40.1300	0.8069	0.2717	-
0.7570	-0.7661	-0.4059	0.1498	0.0483
	0.1251	0.9151	-40.4688	

46.9600 -41.0900 -0.4851 0.7064 -
0.5712 -0.8993 -0.4895 0.0848 0.0272
0.1208 0.9178 -40.4691
46.9800 -40.8400 -0.3781 0.7105 -
0.3343 -0.9942 -0.5666 0.0200 0.0060
0.1165 0.9206 -40.4693
47.0000 -40.5500 0.4115 0.4037 -
0.0968 -1.0496 -0.6361 -0.0445 -0.0151
0.1122 0.9233 -40.4696
47.0200 -41.3700 -0.0985 -0.0022
0.1023 -1.0654 -0.6968 -0.1084 -0.0363
0.1078 0.9260 -40.4699
47.0400 -41.4800 0.0367 -0.2702
0.2363 -1.0435 -0.7480 -0.1717 -0.0574
0.1035 0.9288 -40.4701
47.0600 -41.6900 -0.2049 -0.2636
0.2981 -0.9874 -0.7891 -0.2340 -0.0784
0.0991 0.9315 -40.4704
47.0800 -40.9600 0.3469 -0.1094
0.3073 -0.9024 -0.8200 -0.2953 -0.0994
0.0948 0.9343 -40.4706
47.1000 -41.7200 -0.4999 0.0391
0.2899 -0.7951 -0.8407 -0.3554 -0.1203
0.0904 0.9371 -40.4709
47.1200 -40.8700 0.3495 0.0710
0.2646 -0.6718 -0.8516 -0.4140 -0.1412
0.0860 0.9398 -40.4711
47.1400 -40.7900 0.5321 -0.0613
0.2313 -0.5379 -0.8530 -0.4712 -0.1619
0.0816 0.9426 -40.4713
47.1600 -42.0900 -0.7463 -0.1728
0.1766 -0.3972 -0.8457 -0.5267 -0.1825
0.0772 0.9453 -40.4716
47.1800 -40.6900 0.3967 0.0799
0.0929 -0.2535 -0.8303 -0.5805 -0.2030
0.0728 0.9481 -40.4718
47.2000 -40.8800 -0.2011 0.4707 -
0.0134 -0.1104 -0.8074 -0.6325 -0.2233
0.0684 0.9509 -40.4721
47.2200 -40.3100 0.5647 0.3776 -
0.1238 0.0290 -0.7780 -0.6826 -0.2434
0.0640 0.9536 -40.4723
47.2400 -40.9600 0.5324 -0.1996 -
0.2107 0.1617 -0.7429 -0.7306 -0.2634
0.0596 0.9564 -40.4725
47.2600 -42.7700 -0.8609 -0.6975 -
0.2396 0.2853 -0.7028 -0.7766 -0.2832
0.0552 0.9592 -40.4728
47.2800 -41.2300 0.3486 -0.5062 -
0.1878 0.3984 -0.6585 -0.8205 -0.3028
0.0508 0.9620 -40.4730

47.3000 -40.8900 -0.2292 0.1418 -
0.0806 0.4992 -0.6109 -0.8622 -0.3221
0.0464 0.9647 -40.4732
47.3200 -39.7700 0.2822 0.6397
0.0379 0.5864 -0.5605 -0.9016 -0.3412
0.0420 0.9675 -40.4735
47.3400 -39.4400 0.2807 0.6947
0.1247 0.6578 -0.5082 -0.9386 -0.3601
0.0376 0.9703 -40.4737
47.3600 -40.2900 -0.1889 0.3294
0.1594 0.7116 -0.4547 -0.9733 -0.3787
0.0332 0.9731 -40.4739
47.3800 -40.4200 0.1692 -0.1822
0.1510 0.7469 -0.4008 -1.0055 -0.3970
0.0288 0.9759 -40.4741
47.4000 -41.3200 -0.4308 -0.5160
0.1165 0.7637 -0.3472 -1.0353 -0.4150
0.0245 0.9786 -40.4744
47.4200 -40.7900 0.2900 -0.6134
0.0762 0.7628 -0.2948 -1.0626 -0.4327
0.0201 0.9814 -40.4746
47.4400 -40.6000 0.3766 -0.5596
0.0484 0.7458 -0.2444 -1.0873 -0.4500
0.0157 0.9842 -40.4748
47.4600 -41.2900 -0.6131 -0.2884
0.0312 0.7151 -0.1965 -1.1094 -0.4670
0.0114 0.9870 -40.4750
47.4800 -40.4300 -0.1543 0.2400
0.0161 0.6736 -0.1518 -1.1290 -0.4837
0.0070 0.9898 -40.4752
47.5000 -39.1200 0.7574 0.7197 -
0.0016 0.6242 -0.1108 -1.1459 -0.5000
0.0027 0.9926 -40.4754
47.5200 -39.7300 0.1583 0.7708 -
0.0236 0.5699 -0.0739 -1.1602 -0.5159
-0.0016 0.9954 -40.4757
47.5400 -41.3400 -0.8472 0.3456 -
0.0469 0.5137 -0.0416 -1.1719 -0.5314
-0.0059 0.9982 -40.4759
47.5600 -40.3800 0.8162 -0.3527 -
0.0658 0.4584 -0.0140 -1.1810 -0.5465
-0.0102 1.0010 -40.4761
47.5800 -42.2800 -0.5809 -0.8150 -
0.0748 0.4058 0.0088 -1.1875 -0.5611
-0.0145 1.0038 -40.4763
47.6000 -42.6500 -0.9428 -0.7429 -
0.0691 0.3571 0.0265 -1.1913 -0.5753
-0.0187 1.0066 -40.4765
47.6200 -41.0500 -0.0153 -0.1220 -
0.0511 0.3130 0.0393 -1.1927 -0.5891
-0.0229 1.0094 -40.4767

47.6400 -40.2700 0.1746 0.6325 -
0.0301 0.2737 0.0470 -1.1915 -0.6024
-0.0272 1.0122 -40.4769
47.6600 -39.3900 0.7852 0.9182 -
0.0172 0.2392 0.0499 -1.1878 -0.6153
-0.0313 1.0150 -40.4771
47.6800 -39.5200 1.0575 0.4679 -
0.0199 0.2093 0.0481 -1.1818 -0.6277
-0.0355 1.0178 -40.4773
47.7000 -42.4500 -1.0240 -0.3281 -
0.0281 0.1829 0.0418 -1.1733 -0.6396
-0.0397 1.0206 -40.4775
47.7200 -42.5200 -0.7016 -0.7696 -
0.0267 0.1588 0.0313 -1.1625 -0.6510
-0.0438 1.0234 -40.4777
47.7400 -41.2500 0.5742 -0.6194 -
0.0021 0.1352 0.0169 -1.1496 -0.6620
-0.0479 1.0262 -40.4778
47.7600 -41.4300 -0.1674 -0.1292
0.0443 0.1103 -0.0010 -1.1344 -0.6725
-0.0520 1.0290 -40.4780
47.7800 -40.5100 0.3838 0.2525
0.1002 0.0830 -0.0220 -1.1171 -0.6825
-0.0560 1.0318 -40.4782
47.8000 -40.9100 -0.2753 0.4953
0.1449 0.0527 -0.0455 -1.0979 -0.6921
-0.0601 1.0347 -40.4784
47.8200 -40.4600 0.1626 0.5412
0.1565 0.0192 -0.0711 -1.0767 -0.7012
-0.0640 1.0375 -40.4786
47.8400 -40.9900 -0.0302 0.2731
0.1264 -0.0164 -0.0982 -1.0537 -0.7098
-0.0680 1.0403 -40.4788
47.8600 -41.4500 0.0360 -0.1816
0.0637 -0.0523 -0.1263 -1.0289 -0.7180
-0.0720 1.0431 -40.4789
47.8800 -42.4100 -0.5166 -0.4205 -
0.0150 -0.0862 -0.1550 -1.0024 -0.7257
-0.0759 1.0459 -40.4791
47.9000 -41.5300 0.2492 -0.1478 -
0.0926 -0.1159 -0.1837 -0.9744 -0.7330
-0.0797 1.0487 -40.4793
47.9200 -41.7100 -0.2531 0.2562 -
0.1512 -0.1394 -0.2119 -0.9449 -0.7399
-0.0836 1.0515 -40.4795
47.9400 -40.4800 1.1257 0.0580 -
0.1752 -0.1549 -0.2392 -0.9141 -0.7463
-0.0874 1.0543 -40.4796
47.9600 -43.4500 -1.2962 -0.3728 -
0.1586 -0.1617 -0.2649 -0.8819 -0.7523
-0.0911 1.0572 -40.4798

47.9800 -41.9900 -0.0137 -0.2883 -
0.1016 -0.1605 -0.2888 -0.8485 -0.7579
-0.0949 1.0600 -40.4800
48.0000 -40.6700 0.8148 0.1026 -
0.0205 -0.1530 -0.3104 -0.8140 -0.7630
-0.0986 1.0628 -40.4801
48.0200 -40.1600 1.0342 0.2782
0.0591 -0.1411 -0.3294 -0.7785 -0.7678
-0.1022 1.0656 -40.4803
48.0400 -42.1300 -0.7766 0.1274
0.1151 -0.1269 -0.3455 -0.7421 -0.7721
-0.1058 1.0684 -40.4805
48.0600 -42.0000 -0.6672 -0.0771
0.1378 -0.1124 -0.3586 -0.7049 -0.7761
-0.1094 1.0712 -40.4806
48.0800 -40.6500 0.7936 -0.1078
0.1260 -0.0995 -0.3684 -0.6670 -0.7797
-0.1130 1.0740 -40.4808
48.1000 -41.6300 -0.4614 0.0531
0.0865 -0.0895 -0.3748 -0.6284 -0.7829
-0.1165 1.0769 -40.4809
48.1200 -41.6800 -0.3814 0.0890
0.0381 -0.0836 -0.3776 -0.5893 -0.7857
-0.1199 1.0797 -40.4811
48.1400 -40.5200 0.8897 -0.0618 -
0.0050 -0.0822 -0.3768 -0.5498 -0.7881
-0.1233 1.0825 -40.4813
48.1600 -42.4100 -1.0090 -0.1264 -
0.0353 -0.0853 -0.3725 -0.5099 -0.7902
-0.1267 1.0853 -40.4814
48.1800 -40.5800 0.6570 0.0765 -
0.0514 -0.0922 -0.3649 -0.4697 -0.7919
-0.1300 1.0881 -40.4816
48.2000 -40.2900 0.7887 0.2000 -
0.0568 -0.1017 -0.3539 -0.4293 -0.7933
-0.1333 1.0909 -40.4817
48.2200 -41.7100 -0.5616 0.0364 -
0.0530 -0.1126 -0.3400 -0.3887 -0.7943
-0.1365 1.0938 -40.4819
48.2400 -41.7200 -0.3704 -0.1727 -
0.0356 -0.1227 -0.3232 -0.3480 -0.7950
-0.1397 1.0966 -40.4820
48.2600 -41.4200 -0.2396 -0.1417 -
0.0039 -0.1294 -0.3039 -0.3073 -0.7953
-0.1429 1.0994 -40.4821
48.2800 -40.5100 0.5228 -0.0329
0.0314 -0.1304 -0.2823 -0.2666 -0.7953
-0.1460 1.1022 -40.4823
48.3000 -41.0000 -0.1721 -0.0209
0.0506 -0.1240 -0.2587 -0.2260 -0.7950
-0.1490 1.1050 -40.4824

48.3200 -40.7700 0.0252 0.0358
0.0347 -0.1089 -0.2333 -0.1855 -0.7944
-0.1521 1.1078 -40.4826
48.3400 -40.7100 -0.1740 0.2486 -
0.0257 -0.0848 -0.2064 -0.1452 -0.7934
-0.1550 1.1106 -40.4827
48.3600 -40.5200 -0.1296 0.4022 -
0.1175 -0.0527 -0.1782 -0.1051 -0.7921
-0.1579 1.1134 -40.4828
48.3800 -39.8400 0.7995 0.1639 -
0.2108 -0.0147 -0.1489 -0.0654 -0.7906
-0.1608 1.1163 -40.4830
48.4000 -41.9100 -0.8847 -0.3457 -
0.2708 0.0261 -0.1188 -0.0260 -0.7887
-0.1636 1.1191 -40.4831
48.4200 -40.6700 0.5121 -0.5958 -
0.2669 0.0667 -0.0880 0.0130 -0.7866
-0.1664 1.1219 -40.4832
48.4400 -40.4500 0.5715 -0.5553 -
0.1857 0.1039 -0.0568 0.0515 -0.7842
-0.1691 1.1247 -40.4833
48.4600 -40.6000 -0.1613 -0.3370 -
0.0339 0.1343 -0.0255 0.0894 -0.7814
-0.1718 1.1275 -40.4835
48.4800 -40.3300 -0.5341 0.0451
0.1551 0.1548 0.0059 0.1268 -0.7785
-0.1744 1.1303 -40.4836
48.5000 -39.0100 0.1397 0.5138
0.3344 0.1621 0.0370 0.1634 -0.7752
-0.1770 1.1331 -40.4837
48.5200 -38.2800 0.2455 0.8347
0.4546 0.1531 0.0677 0.1992 -0.7717
-0.1795 1.1359 -40.4838
48.5400 -38.4200 0.1363 0.8132
0.4752 0.1255 0.0980 0.2342 -0.7679
-0.1820 1.1387 -40.4839
48.5600 -39.1800 -0.2925 0.5368
0.3874 0.0802 0.1278 0.2682 -0.7639
-0.1844 1.1415 -40.4841
48.5800 -39.4300 -0.0380 0.2481
0.2133 0.0217 0.1572 0.3013 -0.7596
-0.1868 1.1443 -40.4842
48.6000 -39.8600 -0.0127 -0.0220 -
0.0013 -0.0443 0.1862 0.3332 -0.7551
-0.1891 1.1471 -40.4843
48.6200 -40.1000 0.3282 -0.4320 -
0.2016 -0.1120 0.2148 0.3639 -0.7504
-0.1913 1.1499 -40.4844
48.6400 -40.6300 0.4125 -0.8758 -
0.3443 -0.1753 0.2430 0.3934 -0.7454
-0.1935 1.1527 -40.4845

48.6600 -42.0100 -0.9130 -0.8801 -
0.4050 -0.2289 0.2709 0.4216 -0.7402
-0.1957 1.1555 -40.4846
48.6800 -39.7300 0.6865 -0.2074 -
0.3854 -0.2689 0.2985 0.4483 -0.7348
-0.1978 1.1583 -40.4847
48.7000 -39.1500 0.4413 0.5324 -
0.3135 -0.2937 0.3256 0.4735 -0.7292
-0.1999 1.1611 -40.4848
48.7200 -39.5400 -0.4192 0.8133 -
0.2216 -0.3025 0.3522 0.4971 -0.7234
-0.2019 1.1639 -40.4849
48.7400 -39.4800 -0.3187 0.5636 -
0.1349 -0.2952 0.3781 0.5191 -0.7174
-0.2038 1.1667 -40.4850
48.7600 -39.0500 0.5888 0.0086 -
0.0601 -0.2728 0.4029 0.5395 -0.7112
-0.2057 1.1694 -40.4851
48.7800 -39.7300 0.1084 -0.4876
0.0035 -0.2375 0.4263 0.5580 -0.7048
-0.2075 1.1722 -40.4852
48.8000 -40.3600 -0.6118 -0.5736
0.0585 -0.1928 0.4481 0.5749 -0.6982
-0.2093 1.1750 -40.4853
48.8200 -39.0400 0.3369 -0.1452
0.1109 -0.1425 0.4679 0.5898 -0.6914
-0.2110 1.1778 -40.4854
48.8400 -38.7100 0.0349 0.3228
0.1698 -0.0906 0.4854 0.6030 -0.6845
-0.2127 1.1806 -40.4855
48.8600 -38.3900 0.1286 0.3880
0.2413 -0.0402 0.5004 0.6142 -0.6773
-0.2143 1.1833 -40.4856
48.8800 -38.4100 0.2608 0.0601
0.3190 0.0075 0.5125 0.6235 -0.6701
-0.2158 1.1861 -40.4856
48.9000 -39.1800 -0.2292 -0.2982
0.3845 0.0530 0.5214 0.6309 -0.6627
-0.2173 1.1889 -40.4857
48.9200 -39.5200 -0.6031 -0.3418
0.4173 0.0971 0.5267 0.6363 -0.6551
-0.2188 1.1917 -40.4858
48.9400 -37.8700 0.5905 -0.0448
0.4021 0.1410 0.5279 0.6398 -0.6474
-0.2202 1.1944 -40.4859
48.9600 -38.0000 0.2387 0.2467
0.3314 0.1860 0.5247 0.6414 -0.6395
-0.2215 1.1972 -40.4860
48.9800 -38.8200 -0.6346 0.3540
0.1982 0.2332 0.5167 0.6410 -0.6315
-0.2228 1.1999 -40.4860

49.0000 -38.2000 0.2045 0.3092 -
0.0042 0.2825 0.5037 0.6388 -0.6234
-0.2240 1.2027 -40.4861
49.0200 -38.6300 0.1620 0.1403 -
0.2547 0.3325 0.4853 0.6347 -0.6151
-0.2251 1.2055 -40.4862
49.0400 -38.9000 0.4551 -0.2425 -
0.5014 0.3813 0.4616 0.6289 -0.6068
-0.2262 1.2082 -40.4863
49.0600 -40.4100 -0.5579 -0.6009 -
0.6773 0.4265 0.4326 0.6213 -0.5983
-0.2272 1.2110 -40.4863
49.0800 -40.1000 -0.1553 -0.5714 -
0.7208 0.4661 0.3983 0.6121 -0.5897
-0.2282 1.2137 -40.4864
49.1000 -39.0100 0.3499 -0.2210 -
0.6119 0.4979 0.3592 0.6013 -0.5810
-0.2291 1.2164 -40.4865
49.1200 -38.5400 0.4192 0.1186 -
0.3740 0.5187 0.3157 0.5891 -0.5723
-0.2300 1.2192 -40.4865
49.1400 -39.0800 -0.6208 0.2684 -
0.0471 0.5251 0.2683 0.5756 -0.5634
-0.2308 1.2219 -40.4866
49.1600 -37.8600 0.3802 0.2236
0.3124 0.5137 0.2178 0.5608 -0.5545
-0.2315 1.2247 -40.4866
49.1800 -37.9400 0.1841 0.0485
0.6383 0.4809 0.1647 0.5448 -0.5455
-0.2322 1.2274 -40.4867
49.2000 -38.5000 -0.3604 -0.0738
0.8602 0.4236 0.1100 0.5278 -0.5365
-0.2329 1.2301 -40.4868
49.2200 -38.0000 0.2276 0.0720
0.9235 0.3390 0.0543 0.5099 -0.5274
-0.2334 1.2328 -40.4868
49.2400 -38.3200 -0.2818 0.4841
0.8072 0.2281 -0.0014 0.4911 -0.5182
-0.2339 1.2356 -40.4869
49.2600 -38.0500 0.0399 0.8019
0.5290 0.0979 -0.0564 0.4716 -0.5091
-0.2344 1.2383 -40.4869
49.2800 -38.4000 0.6308 0.6128
0.1409 -0.0430 -0.1098 0.4514 -0.4999
-0.2347 1.2410 -40.4870
49.3000 -40.6400 -0.3946 -0.0976 -
0.2707 -0.1854 -0.1608 0.4307 -0.4906
-0.2351 1.2437 -40.4870
49.3200 -41.3900 0.0398 -0.7298 -
0.6075 -0.3204 -0.2086 0.4096 -0.4814
-0.2353 1.2464 -40.4871

49.3400 -42.5500 -0.7700 -0.7139 -
0.7835 -0.4391 -0.2523 0.3881 -0.4722
-0.2355 1.2491 -40.4871
49.3600 -40.7000 0.6132 -0.1850 -
0.7758 -0.5331 -0.2911 0.3663 -0.4629
-0.2357 1.2518 -40.4871
49.3800 -40.4100 0.5198 0.2215 -
0.6195 -0.5978 -0.3243 0.3444 -0.4537
-0.2358 1.2545 -40.4872
49.4000 -40.6300 0.1148 0.2012 -
0.3719 -0.6319 -0.3511 0.3225 -0.4445
-0.2358 1.2572 -40.4872
49.4200 -41.4400 -0.7691 0.1186 -
0.1097 -0.6359 -0.3709 0.3006 -0.4353
-0.2357 1.2598 -40.4873
49.4400 -39.7500 0.5517 0.1955
0.0954 -0.6109 -0.3836 0.2789 -0.4262
-0.2356 1.2625 -40.4873
49.4600 -39.7900 0.2969 0.2716
0.2112 -0.5582 -0.3891 0.2573 -0.4171
-0.2355 1.2652 -40.4873
49.4800 -40.6800 -0.6152 0.1689
0.2413 -0.4810 -0.3879 0.2361 -0.4081
-0.2352 1.2679 -40.4874
49.5000 -39.7600 0.5529 -0.1260
0.2081 -0.3843 -0.3806 0.2152 -0.3991
-0.2350 1.2705 -40.4874
49.5200 -41.0400 -0.6060 -0.3471
0.1432 -0.2744 -0.3677 0.1946 -0.3902
-0.2346 1.2732 -40.4874
49.5400 -39.7100 0.5555 -0.2328
0.0755 -0.1576 -0.3499 0.1745 -0.3813
-0.2342 1.2758 -40.4874
49.5600 -40.4100 -0.4497 0.1094
0.0207 -0.0401 -0.3278 0.1549 -0.3726
-0.2337 1.2785 -40.4875
49.5800 -39.7600 -0.1248 0.3471 -
0.0129 0.0727 -0.3021 0.1357 -0.3639
-0.2332 1.2811 -40.4875
49.6000 -38.9400 0.5811 0.2787 -
0.0216 0.1762 -0.2735 0.1171 -0.3554
-0.2326 1.2837 -40.4875
49.6200 -39.7900 -0.0328 -0.1039 -
0.0029 0.2665 -0.2427 0.0991 -0.3470
-0.2319 1.2864 -40.4875
49.6400 -40.2100 -0.2453 -0.4575
0.0425 0.3406 -0.2102 0.0818 -0.3386
-0.2312 1.2890 -40.4875
49.6600 -40.0200 -0.2272 -0.3932
0.1077 0.3962 -0.1766 0.0651 -0.3305
-0.2305 1.2916 -40.4875

49.6800 −39.2000 −0.0236 0.0674
0.1743 0.4315 −0.1426 0.0491 −0.3224
−0.2296 1.2942 −40.4876
49.7000 −38.3300 0.3967 0.4652
0.2178 0.4454 −0.1087 0.0339 −0.3145
−0.2287 1.2968 −40.4876
49.7200 −39.0900 −0.2597 0.3874
0.2173 0.4378 −0.0754 0.0195 −0.3068
−0.2278 1.2994 −40.4876
49.7400 −38.9100 0.2477 −0.0512
0.1758 0.4097 −0.0432 0.0059 −0.2992
−0.2268 1.3020 −40.4876
49.7600 −40.0100 −0.4412 −0.3666
0.1166 0.3632 −0.0125 −0.0067 −0.2918
−0.2257 1.3046 −40.4876
49.7800 −39.1500 0.5041 −0.3416
0.0657 0.3014 0.0163 −0.0185 −0.2845
−0.2245 1.3072 −40.4876
49.8000 −39.9800 −0.5224 −0.0735
0.0331 0.2283 0.0427 −0.0293 −0.2774
−0.2234 1.3098 −40.4876
49.8200 −39.0800 0.0862 0.2435
0.0113 0.1490 0.0665 −0.0391 −0.2705
−0.2221 1.3123 −40.4876
49.8400 −38.6900 0.6353 0.3668 −
0.0159 0.0692 0.0872 −0.0479 −0.2638
−0.2208 1.3149 −40.4876
49.8600 −39.8500 −0.5288 0.1922 −
0.0627 −0.0058 0.1047 −0.0557 −0.2573
−0.2194 1.3174 −40.4876
49.8800 −39.7500 0.0394 −0.0737 −
0.1304 −0.0705 0.1185 −0.0624 −0.2510
−0.2180 1.3200 −40.4876
49.9000 −39.6600 0.4291 −0.2390 −
0.2064 −0.1209 0.1284 −0.0681 −0.2448
−0.2166 1.3225 −40.4876
49.9200 −40.7200 −0.4583 −0.2266 −
0.2652 −0.1539 0.1342 −0.0728 −0.2389
−0.2150 1.3251 −40.4876
49.9400 −40.1100 −0.0968 −0.0148 −
0.2826 −0.1683 0.1360 −0.0765 −0.2331
−0.2134 1.3276 −40.4876
49.9600 −39.3300 0.4424 0.1646 −
0.2443 −0.1652 0.1339 −0.0792 −0.2275
−0.2118 1.3301 −40.4875
49.9800 −39.6000 0.1929 0.0471 −
0.1475 −0.1477 0.1283 −0.0810 −0.2221
−0.2101 1.3326 −40.4875
50.0000 −40.5500 −0.5548 −0.1840 −
0.0097 −0.1202 0.1195 −0.0820 −0.2169
−0.2084 1.3351 −40.4875

50.0200 -39.4000 0.1754 -0.1220
0.1402 -0.0874 0.1083 -0.0821 -0.2118
-0.2066 1.3376 -40.4875
50.0400 -39.4000 -0.4157 0.2244
0.2649 -0.0543 0.0953 -0.0815 -0.2069
-0.2048 1.3401 -40.4875
50.0600 -38.3200 0.7009 0.2905
0.3341 -0.0256 0.0812 -0.0803 -0.2022
-0.2029 1.3426 -40.4874
50.0800 -39.7900 -0.6073 -0.0166
0.3331 -0.0051 0.0667 -0.0785 -0.1976
-0.2010 1.3450 -40.4874
50.1000 -39.3500 0.0924 -0.1686
0.2683 0.0054 0.0524 -0.0761 -0.1931
-0.1990 1.3475 -40.4874
50.1200 -39.1000 0.2207 -0.0595
0.1582 0.0061 0.0389 -0.0733 -0.1888
-0.1970 1.3500 -40.4874
50.1400 -39.3200 0.1458 0.0277
0.0297 -0.0011 0.0267 -0.0702 -0.1846
-0.1950 1.3524 -40.4873
50.1600 -39.7200 -0.1029 -0.0184 -
0.0913 -0.0137 0.0161 -0.0667 -0.1805
-0.1929 1.3549 -40.4873
50.1800 -40.0100 -0.0969 -0.0900 -
0.1842 -0.0290 0.0075 -0.0631 -0.1765
-0.1907 1.3573 -40.4873
50.2000 -40.0500 -0.0712 -0.0765 -
0.2339 -0.0444 0.0011 -0.0593 -0.1725
-0.1886 1.3597 -40.4872
50.2200 -39.7600 0.1353 -0.0496 -
0.2313 -0.0578 -0.0031 -0.0555 -0.1687
-0.1864 1.3621 -40.4872
50.2400 -39.7200 0.1941 -0.0719 -
0.1813 -0.0675 -0.0051 -0.0516 -0.1649
-0.1841 1.3645 -40.4872
50.2600 -40.1600 -0.3398 -0.0935 -
0.1004 -0.0726 -0.0048 -0.0479 -0.1612
-0.1818 1.3669 -40.4871
50.2800 -39.6400 -0.0800 -0.0133 -
0.0114 -0.0729 -0.0024 -0.0442 -0.1576
-0.1795 1.3693 -40.4871
50.3000 -39.3200 0.0520 0.1242
0.0621 -0.0688 0.0019 -0.0408 -0.1540
-0.1772 1.3717 -40.4870
50.3200 -38.8600 0.3738 0.1422
0.0978 -0.0606 0.0081 -0.0375 -0.1504
-0.1748 1.3741 -40.4870
50.3400 -39.9000 -0.5079 0.0309
0.0891 -0.0487 0.0157 -0.0345 -0.1469
-0.1724 1.3764 -40.4870

50.3600 -39.0900 0.4645 -0.0405
0.0462 -0.0343 0.0246 -0.0318 -0.1433
-0.1700 1.3788 -40.4869
50.3800 -39.6900 -0.2345 -0.0677 -
0.0107 -0.0184 0.0344 -0.0295 -0.1398
-0.1675 1.3811 -40.4869
50.4000 -39.5100 0.1059 -0.1094 -
0.0611 -0.0023 0.0446 -0.0274 -0.1362
-0.1650 1.3834 -40.4868
50.4200 -39.4900 0.0565 -0.1153 -
0.0875 0.0131 0.0550 -0.0258 -0.1327
-0.1625 1.3858 -40.4867
50.4400 -39.7300 -0.3230 -0.0182 -
0.0813 0.0273 0.0653 -0.0246 -0.1291
-0.1600 1.3881 -40.4867
50.4600 -39.1700 0.1930 0.1168 -
0.0503 0.0396 0.0750 -0.0238 -0.1254
-0.1574 1.3904 -40.4866
50.4800 -38.7500 0.3864 0.1705 -
0.0081 0.0496 0.0840 -0.0235 -0.1217
-0.1549 1.3927 -40.4866
50.5000 -39.7600 -0.7198 0.1501
0.0329 0.0569 0.0921 -0.0237 -0.1180
-0.1523 1.3949 -40.4865
50.5200 -38.6800 0.4925 0.0720
0.0620 0.0615 0.0988 -0.0243 -0.1142
-0.1497 1.3972 -40.4864
50.5400 -38.6900 0.5636 -0.0820
0.0776 0.0632 0.1043 -0.0255 -0.1103
-0.1470 1.3995 -40.4864
50.5600 -39.7300 -0.4310 -0.2227
0.0816 0.0621 0.1082 -0.0271 -0.1064
-0.1444 1.4017 -40.4863
50.5800 -39.8700 -0.6333 -0.1592
0.0768 0.0587 0.1105 -0.0293 -0.1023
-0.1417 1.4040 -40.4862
50.6000 -38.5600 0.5533 0.0871
0.0624 0.0532 0.1113 -0.0320 -0.0981
-0.1391 1.4062 -40.4862
50.6200 -38.9600 0.0304 0.1868
0.0368 0.0461 0.1105 -0.0352 -0.0939
-0.1364 1.4084 -40.4861
50.6400 -39.0200 0.2253 0.0054
0.0006 0.0376 0.1082 -0.0389 -0.0895
-0.1337 1.4106 -40.4860
50.6600 -39.7500 -0.3980 -0.1593 -
0.0368 0.0282 0.1046 -0.0431 -0.0850
-0.1310 1.4128 -40.4860
50.6800 -39.6100 -0.1919 -0.1152 -
0.0662 0.0187 0.0998 -0.0478 -0.0804
-0.1283 1.4150 -40.4859

50.7000 -38.6900 0.5431 0.0190 -
0.0830 0.0097 0.0939 -0.0529 -0.0757
-0.1256 1.4172 -40.4858
50.7200 -39.3100 -0.1178 0.0694 -
0.0845 0.0019 0.0871 -0.0584 -0.0708
-0.1229 1.4194 -40.4857
50.7400 -39.6100 -0.3011 0.0692 -
0.0729 -0.0041 0.0796 -0.0643 -0.0658
-0.1202 1.4215 -40.4856
50.7600 -39.6600 -0.5420 0.1454 -
0.0506 -0.0082 0.0717 -0.0705 -0.0607
-0.1174 1.4237 -40.4855
50.7800 -38.3300 0.7800 0.1280 -
0.0154 -0.0108 0.0634 -0.0770 -0.0554
-0.1147 1.4258 -40.4855
50.8000 -39.4900 -0.1397 -0.1344
0.0315 -0.0122 0.0549 -0.0839 -0.0500
-0.1120 1.4279 -40.4854
50.8200 -40.2600 -0.7235 -0.3502
0.0780 -0.0133 0.0464 -0.0909 -0.0444
-0.1093 1.4300 -40.4853
50.8400 -39.0100 0.3983 -0.2203
0.1091 -0.0145 0.0380 -0.0981 -0.0387
-0.1065 1.4321 -40.4852
50.8600 -38.8400 0.0709 0.1647
0.1125 -0.0161 0.0300 -0.1055 -0.0329
-0.1038 1.4342 -40.4851
50.8800 -39.1200 -0.3989 0.4548
0.0824 -0.0186 0.0223 -0.1130 -0.0269
-0.1011 1.4363 -40.4850
50.9000 -38.4900 0.4253 0.3471
0.0220 -0.0219 0.0152 -0.1205 -0.0207
-0.0984 1.4384 -40.4849
50.9200 -39.1000 0.4089 -0.1021 -
0.0481 -0.0255 0.0087 -0.1281 -0.0144
-0.0957 1.4404 -40.4848
50.9400 -40.5500 -0.6830 -0.4759 -
0.0968 -0.0290 0.0027 -0.1356 -0.0079
-0.0930 1.4424 -40.4847
50.9600 -39.4500 0.3142 -0.3377 -
0.0994 -0.0322 -0.0026 -0.1431 -0.0013
-0.0903 1.4445 -40.4846
50.9800 -39.3000 -0.0062 0.0991 -
0.0618 -0.0349 -0.0073 -0.1504 0.0055
-0.0876 1.4465 -40.4845
51.0000 -38.7300 0.2351 0.3118 -
0.0056 -0.0373 -0.0114 -0.1576 0.0124
-0.0849 1.4485 -40.4844
51.0200 -38.9100 0.1597 0.1943
0.0433 -0.0396 -0.0150 -0.1646 0.0195
-0.0823 1.4505 -40.4843

51.0400 -39.4900 -0.2854 0.0072
0.0647 -0.0414 -0.0181 -0.1714 0.0268
-0.0796 1.4525 -40.4842
51.0600 -39.4200 -0.1995 0.0055
0.0474 -0.0420 -0.0209 -0.1778 0.0342
-0.0769 1.4544 -40.4841
51.0800 -38.8400 0.3871 0.0809
0.0064 -0.0403 -0.0234 -0.1840 0.0418
-0.0743 1.4564 -40.4840
51.1000 -39.6100 -0.3200 -0.0368 -
0.0281 -0.0360 -0.0258 -0.1897 0.0495
-0.0717 1.4583 -40.4838
51.1200 -39.3500 0.3233 -0.2844 -
0.0383 -0.0286 -0.0281 -0.1951 0.0574
-0.0691 1.4602 -40.4837
51.1400 -40.0200 -0.5110 -0.2486 -
0.0243 -0.0177 -0.0304 -0.2000 0.0655
-0.0665 1.4622 -40.4836
51.1600 -38.9100 0.2598 0.1192 -
0.0050 -0.0026 -0.0327 -0.2045 0.0737
-0.0639 1.4641 -40.4835
51.1800 -38.5300 0.3292 0.3779 -
0.0062 0.0173 -0.0351 -0.2085 0.0821
-0.0613 1.4659 -40.4834
51.2000 -38.9700 -0.0422 0.2564 -
0.0447 0.0416 -0.0377 -0.2121 0.0907
-0.0588 1.4678 -40.4832
51.2200 -39.4500 -0.0861 -0.0724 -
0.1067 0.0694 -0.0405 -0.2151 0.0994
-0.0562 1.4697 -40.4831
51.2400 -39.6000 -0.0713 -0.2684 -
0.1621 0.0985 -0.0434 -0.2176 0.1082
-0.0537 1.4715 -40.4830
51.2600 -39.8500 -0.3320 -0.2249 -
0.1786 0.1261 -0.0464 -0.2196 0.1173
-0.0512 1.4734 -40.4829
51.2800 -38.8200 0.5361 -0.1102 -
0.1412 0.1495 -0.0494 -0.2210 0.1265
-0.0487 1.4752 -40.4827
51.3000 -38.9100 0.1422 -0.0219 -
0.0561 0.1660 -0.0524 -0.2218 0.1358
-0.0462 1.4770 -40.4826
51.3200 -39.8400 -0.8675 0.0631
0.0572 0.1733 -0.0553 -0.2221 0.1453
-0.0438 1.4788 -40.4825
51.3400 -37.7400 0.9155 0.1431
0.1694 0.1697 -0.0580 -0.2219 0.1549
-0.0413 1.4806 -40.4823
51.3600 -39.5000 -0.8682 0.1161
0.2555 0.1539 -0.0605 -0.2210 0.1647
-0.0389 1.4823 -40.4822

51.3800 -38.0000 0.6532 0.0419
0.3006 0.1252 -0.0625 -0.2197 0.1746
-0.0365 1.4841 -40.4821
51.4000 -38.7200 0.0510 -0.0696
0.3007 0.0841 -0.0639 -0.2178 0.1847
-0.0341 1.4858 -40.4819
51.4200 -39.0500 -0.2191 -0.1718
0.2614 0.0326 -0.0646 -0.2153 0.1948
-0.0317 1.4875 -40.4818
51.4400 -39.5000 -0.4485 -0.0906
0.1878 -0.0257 -0.0645 -0.2123 0.2051
-0.0294 1.4892 -40.4816
51.4600 -38.6900 0.2711 0.1276
0.0875 -0.0860 -0.0634 -0.2088 0.2156
-0.0271 1.4909 -40.4815
51.4800 -38.6800 0.3264 0.2197 -
0.0333 -0.1432 -0.0612 -0.2048 0.2261
-0.0247 1.4926 -40.4813
51.5000 -39.3900 -0.0717 0.0700 -
0.1636 -0.1923 -0.0578 -0.2004 0.2368
-0.0224 1.4943 -40.4812
51.5200 -39.9800 -0.4370 -0.0964 -
0.2831 -0.2286 -0.0531 -0.1955 0.2475
-0.0201 1.4959 -40.4810
51.5400 -39.4100 0.2859 -0.1019 -
0.3650 -0.2483 -0.0469 -0.1902 0.2584
-0.0179 1.4976 -40.4809
51.5600 -39.4700 0.0945 -0.0662 -
0.3920 -0.2488 -0.0394 -0.1845 0.2694
-0.0156 1.4992 -40.4807
51.5800 -39.9000 -0.3647 -0.0681 -
0.3615 -0.2296 -0.0304 -0.1784 0.2804
-0.0134 1.5008 -40.4806
51.6000 -39.1600 0.2625 -0.0416 -
0.2780 -0.1925 -0.0204 -0.1721 0.2916
-0.0112 1.5024 -40.4804
51.6200 -39.1600 -0.0965 0.0079 -
0.1551 -0.1412 -0.0095 -0.1654 0.3028
-0.0090 1.5039 -40.4802
51.6400 -38.7400 0.2022 0.0293 -
0.0154 -0.0805 0.0020 -0.1585 0.3141
-0.0068 1.5055 -40.4801
51.6600 -39.0900 -0.4118 0.0521
0.1173 -0.0153 0.0139 -0.1514 0.3255
-0.0047 1.5070 -40.4799
51.6800 -37.9200 0.4105 0.0582
0.2238 0.0495 0.0258 -0.1441 0.3369
-0.0026 1.5086 -40.4798
51.7000 -38.2800 -0.0323 -0.0365
0.2924 0.1096 0.0375 -0.1366 0.3484
-0.0004 1.5101 -40.4796

51.7200 -38.6100 -0.2244 -0.1025
0.3209 0.1609 0.0487 -0.1291 0.3600
0.0017 1.5116 -40.4794
51.7400 -38.3100 -0.2286 0.0374
0.3071 0.2004 0.0592 -0.1215 0.3716
0.0037 1.5131 -40.4793
51.7600 -37.4100 0.4967 0.1859
0.2502 0.2261 0.0687 -0.1138 0.3833
0.0058 1.5145 -40.4791
51.7800 -38.3400 -0.2226 0.1077
0.1580 0.2372 0.0770 -0.1061 0.3950
0.0078 1.5160 -40.4789
51.8000 -38.5300 -0.1264 -0.0480
0.0514 0.2340 0.0840 -0.0985 0.4067
0.0098 1.5174 -40.4787
51.8200 -38.5900 -0.1206 -0.0850 -
0.0470 0.2174 0.0897 -0.0909 0.4184
0.0118 1.5188 -40.4786
51.8400 -38.4900 0.0362 -0.0507 -
0.1183 0.1886 0.0940 -0.0835 0.4302
0.0138 1.5202 -40.4784
51.8600 -38.3500 0.2740 -0.0669 -
0.1472 0.1497 0.0970 -0.0761 0.4419
0.0158 1.5216 -40.4782
51.8800 -39.0200 -0.4518 -0.1251 -
0.1301 0.1032 0.0986 -0.0690 0.4537
0.0177 1.5230 -40.4780
51.9000 -38.1500 0.5040 -0.1732 -
0.0744 0.0524 0.0991 -0.0620 0.4655
0.0196 1.5243 -40.4778
51.9200 -38.6300 -0.0747 -0.1729
0.0001 0.0013 0.0983 -0.0552 0.4772
0.0215 1.5257 -40.4776
51.9400 -38.8200 -0.4025 0.0050
0.0632 -0.0464 0.0964 -0.0486 0.4890
0.0234 1.5270 -40.4775
51.9600 -37.8000 0.1955 0.3250
0.0898 -0.0871 0.0935 -0.0423 0.5007
0.0252 1.5283 -40.4773
51.9800 -37.5600 0.3142 0.4749
0.0651 -0.1182 0.0897 -0.0362 0.5123
0.0270 1.5296 -40.4771
52.0000 -38.2500 0.0154 0.2335 -
0.0089 -0.1381 0.0851 -0.0304 0.5239
0.0288 1.5308 -40.4769
52.0200 -38.9100 -0.1067 -0.2110 -
0.1024 -0.1462 0.0797 -0.0249 0.5355
0.0306 1.5321 -40.4767
52.0400 -39.2600 -0.0609 -0.4626 -
0.1747 -0.1429 0.0737 -0.0197 0.5470
0.0324 1.5333 -40.4765

52.0600 -39.3000 -0.3470 -0.2946 -
0.1928 -0.1288 0.0672 -0.0147 0.5585
0.0341 1.5345 -40.4763
52.0800 -38.0700 0.3874 0.0845 -
0.1521 -0.1061 0.0604 -0.0101 0.5698
0.0358 1.5357 -40.4761
52.1000 -38.0800 0.1214 0.2720 -
0.0686 -0.0777 0.0533 -0.0057 0.5811
0.0375 1.5369 -40.4759
52.1200 -37.8900 0.3505 0.0893
0.0321 -0.0474 0.0461 -0.0016 0.5923
0.0392 1.5381 -40.4757
52.1400 -38.9700 -0.7131 -0.1150
0.1219 -0.0186 0.0390 0.0022 0.6033
0.0408 1.5392 -40.4755
52.1600 -37.4800 0.6305 -0.0217
0.1778 0.0054 0.0320 0.0058 0.6143
0.0425 1.5403 -40.4753
52.1800 -38.2000 -0.3046 0.0849
0.1930 0.0225 0.0252 0.0092 0.6251
0.0441 1.5415 -40.4751
52.2000 -38.0400 -0.0392 0.0504
0.1709 0.0316 0.0187 0.0123 0.6357
0.0456 1.5426 -40.4749
52.2200 -38.0800 -0.1029 0.0498
0.1181 0.0328 0.0125 0.0152 0.6463
0.0472 1.5436 -40.4747
52.2400 -37.9900 0.0670 0.0717
0.0473 0.0277 0.0066 0.0179 0.6566
0.0487 1.5447 -40.4744
52.2600 -38.1500 0.1865 -0.0051 -
0.0228 0.0182 0.0008 0.0205 0.6669
0.0502 1.5457 -40.4742
52.2800 -38.5900 -0.0681 -0.1499 -
0.0743 0.0070 -0.0048 0.0229 0.6769
0.0517 1.5467 -40.4740
52.3000 -38.6300 -0.1514 -0.2028 -
0.0962 -0.0036 -0.0104 0.0252 0.6867
0.0532 1.5477 -40.4738
52.3200 -38.4900 -0.1573 -0.0603 -
0.0926 -0.0112 -0.0160 0.0274 0.6963
0.0546 1.5487 -40.4736
52.3400 -37.9100 0.2630 0.1614 -
0.0818 -0.0136 -0.0218 0.0295 0.7058
0.0560 1.5497 -40.4734
52.3600 -38.1600 -0.0740 0.2227 -
0.0780 -0.0094 -0.0278 0.0316 0.7150
0.0574 1.5506 -40.4731
52.3800 -37.9700 0.1473 0.0984 -
0.0840 0.0017 -0.0339 0.0337 0.7240
0.0588 1.5516 -40.4729

52.4000	-38.3500	-0.1374	-0.0590	-
0.0900	0.0189	-0.0403	0.0357	0.7327
	0.0601	1.5525	-40.4727	
52.4200	-38.2000	0.2730	-0.1799	-
0.0819	0.0405	-0.0467	0.0378	0.7412
	0.0614	1.5534	-40.4725	
52.4400	-38.9400	-0.6765	-0.1425	-
0.0509	0.0636	-0.0533	0.0400	0.7494
	0.0627	1.5542	-40.4722	
52.4600	-37.8000	0.0634	0.0356	
0.0035	0.0854	-0.0600	0.0422	0.7574
	0.0640	1.5551	-40.4720	
52.4800	-37.0300	0.7342	0.1164	
0.0746	0.1030	-0.0665	0.0446	0.7650
	0.0652	1.5559	-40.4718	
52.5000	-38.5900	-0.7664	0.0054	
0.1441	0.1139	-0.0730	0.0472	0.7724
	0.0664	1.5567	-40.4715	
52.5200	-37.5400	0.2406	0.0153	
0.1875	0.1162	-0.0794	0.0499	0.7795
	0.0676	1.5575	-40.4713	
52.5400	-37.7000	-0.0331	0.1886	
0.1908	0.1087	-0.0854	0.0529	0.7862
	0.0688	1.5583	-40.4711	
52.5600	-37.2300	0.4651	0.1450	
0.1529	0.0910	-0.0912	0.0561	0.7927
	0.0699	1.5591	-40.4708	
52.5800	-38.3400	-0.3883	-0.0843	
0.0893	0.0640	-0.0965	0.0596	0.7987
	0.0710	1.5598	-40.4706	
52.6000	-38.3000	-0.0652	-0.1969	
0.0238	0.0300	-0.1012	0.0634	0.8045
	0.0721	1.5605	-40.4703	
52.6200	-37.8800	0.3229	-0.1403	-
0.0271	-0.0081	-0.1054	0.0676	0.8099
	0.0731	1.5612	-40.4701	
52.6400	-38.2500	-0.1031	-0.0198	-
0.0581	-0.0469	-0.1088	0.0721	0.8149
	0.0742	1.5619	-40.4698	
52.6600	-38.3000	-0.1815	0.1031	-
0.0780	-0.0830	-0.1115	0.0770	0.8196
	0.0752	1.5625	-40.4696	
52.6800	-37.6500	0.3778	0.1717	-
0.0973	-0.1130	-0.1134	0.0823	0.8238
	0.0761	1.5632	-40.4694	
52.7000	-38.4600	-0.2452	0.1069	-
0.1185	-0.1342	-0.1145	0.0881	0.8277
	0.0771	1.5638	-40.4691	
52.7200	-38.1700	0.1282	-0.0213	-
0.1352	-0.1446	-0.1148	0.0943	0.8312
	0.0780	1.5644	-40.4688	

52.7400 -38.6500 -0.1955 -0.1210 -
0.1340 -0.1432 -0.1142 0.1010 0.8343
0.0789 1.5649 -40.4686
52.7600 -38.1900 0.1278 -0.1556 -
0.1052 -0.1302 -0.1129 0.1081 0.8370
0.0798 1.5655 -40.4683
52.7800 -38.1300 0.0685 -0.0856 -
0.0564 -0.1068 -0.1111 0.1157 0.8393
0.0806 1.5660 -40.4681
52.8000 -38.3000 -0.3872 0.0970 -
0.0049 -0.0753 -0.1090 0.1238 0.8411
0.0814 1.5665 -40.4678
52.8200 -37.2100 0.5747 0.2034
0.0314 -0.0388 -0.1067 0.1324 0.8425
0.0822 1.5670 -40.4676
52.8400 -38.3500 -0.5128 0.0399
0.0430 -0.0003 -0.1045 0.1415 0.8435
0.0829 1.5675 -40.4673
52.8600 -37.6500 0.3161 -0.1351
0.0383 0.0372 -0.1025 0.1510 0.8440
0.0836 1.5680 -40.4670
52.8800 -38.2000 -0.2881 -0.1294
0.0340 0.0709 -0.1010 0.1609 0.8441
0.0843 1.5684 -40.4668
52.9000 -37.6300 0.1699 -0.0457
0.0447 0.0985 -0.1001 0.1713 0.8438
0.0850 1.5688 -40.4665
52.9200 -37.7500 0.0197 0.0180
0.0721 0.1177 -0.0999 0.1821 0.8430
0.0857 1.5692 -40.4662
52.9400 -37.6000 -0.0084 0.0273
0.1060 0.1276 -0.1006 0.1932 0.8418
0.0863 1.5695 -40.4660
52.9600 -37.7000 -0.1480 0.0323
0.1301 0.1284 -0.1022 0.2046 0.8401
0.0869 1.5699 -40.4657
52.9800 -37.4600 0.1844 0.0493
0.1293 0.1204 -0.1046 0.2164 0.8379
0.0874 1.5702 -40.4654
53.0000 -37.4900 0.1063 0.0351
0.0955 0.1047 -0.1078 0.2283 0.8353
0.0880 1.5705 -40.4651
53.0200 -38.1200 -0.3617 0.0026
0.0317 0.0828 -0.1116 0.2405 0.8322
0.0885 1.5708 -40.4649
53.0400 -37.5700 0.2764 0.0058 -
0.0456 0.0570 -0.1158 0.2528 0.8286
0.0890 1.5710 -40.4646
53.0600 -37.8800 0.0495 -0.0254 -
0.1114 0.0295 -0.1202 0.2653 0.8246
0.0894 1.5713 -40.4643

53.0800 -38.1900 -0.1883 -0.1069 -
0.1429 0.0030 -0.1247 0.2778 0.8201
0.0898 1.5715 -40.4640
53.1000 -38.1000 0.0261 -0.1298 -
0.1304 -0.0206 -0.1289 0.2903 0.8151
0.0903 1.5717 -40.4637
53.1200 -37.9100 0.0446 -0.0534 -
0.0832 -0.0395 -0.1326 0.3028 0.8096
0.0907 1.5718 -40.4635
53.1400 -37.9000 -0.1031 0.0626 -
0.0215 -0.0527 -0.1354 0.3151 0.8037
0.0910 1.5720 -40.4632
53.1600 -37.7600 0.0745 0.1317
0.0334 -0.0601 -0.1373 0.3273 0.7972
0.0914 1.5721 -40.4629
53.1800 -37.5500 0.2008 0.0548
0.0635 -0.0620 -0.1378 0.3393 0.7903
0.0917 1.5722 -40.4626
53.2000 -38.2500 -0.3365 -0.0535
0.0648 -0.0594 -0.1368 0.3510 0.7828
0.0920 1.5723 -40.4623
53.2200 -37.5500 0.2465 0.0005
0.0452 -0.0533 -0.1341 0.3623 0.7749
0.0923 1.5723 -40.4620
53.2400 -37.7300 -0.0151 0.0937
0.0163 -0.0453 -0.1295 0.3733 0.7665
0.0925 1.5724 -40.4617
53.2600 -37.7000 0.0510 0.0346 -
0.0048 -0.0364 -0.1230 0.3838 0.7576
0.0928 1.5724 -40.4614
53.2800 -38.0900 -0.0968 -0.1420 -
0.0096 -0.0267 -0.1144 0.3938 0.7482
0.0930 1.5724 -40.4611
53.3000 -37.8200 0.2000 -0.2152 -
0.0033 -0.0166 -0.1036 0.4033 0.7383
0.0932 1.5723 -40.4608
53.3200 -38.3100 -0.5830 -0.0005
0.0058 -0.0060 -0.0906 0.4121 0.7279
0.0934 1.5723 -40.4605
53.3400 -36.8500 0.6756 0.2542
0.0061 0.0046 -0.0754 0.4204 0.7171
0.0936 1.5722 -40.4602
53.3600 -37.4800 0.0430 0.1695 -
0.0102 0.0150 -0.0580 0.4279 0.7057
0.0937 1.5721 -40.4599
53.3800 -38.3400 -0.5120 -0.0866 -
0.0352 0.0251 -0.0385 0.4347 0.6939
0.0938 1.5720 -40.4596
53.4000 -37.5100 0.4412 -0.1912 -
0.0508 0.0347 -0.0167 0.4407 0.6815
0.0940 1.5718 -40.4593

53.4200 -38.1400 -0.4253 -0.0556 -
0.0457 0.0438 0.0072 0.4459 0.6687
0.0941 1.5717 -40.4590
53.4400 -37.1300 0.3967 0.0766 -
0.0209 0.0516 0.0334 0.4502 0.6555
0.0941 1.5715 -40.4587
53.4600 -37.8100 -0.3347 0.0737
0.0107 0.0566 0.0617 0.4536 0.6417
0.0942 1.5713 -40.4584
53.4800 -37.0700 0.4916 0.0016
0.0336 0.0579 0.0921 0.4560 0.6275
0.0943 1.5710 -40.4581
53.5000 -37.8900 -0.3520 -0.0041
0.0382 0.0550 0.1245 0.4575 0.6128
0.0943 1.5708 -40.4577
53.5200 -37.5600 -0.1428 0.0567
0.0246 0.0479 0.1588 0.4579 0.5977
0.0944 1.5705 -40.4574
53.5400 -37.0000 0.5180 0.0451
0.0024 0.0371 0.1949 0.4573 0.5821
0.0944 1.5702 -40.4571
53.5600 -37.8000 -0.2766 -0.0669 -
0.0157 0.0232 0.2326 0.4556 0.5661
0.0944 1.5698 -40.4568
53.5800 -37.9500 -0.2979 -0.1100 -
0.0230 0.0072 0.2716 0.4527 0.5497
0.0944 1.5695 -40.4565
53.6000 -37.3400 0.2246 -0.0082 -
0.0229 -0.0100 0.3118 0.4487 0.5329
0.0944 1.5691 -40.4561
53.6200 -37.3500 0.0483 0.1218 -
0.0222 -0.0270 0.3528 0.4434 0.5156
0.0944 1.5687 -40.4558
53.6400 -37.4000 0.0836 0.1203 -
0.0282 -0.0426 0.3944 0.4369 0.4980
0.0943 1.5683 -40.4555
53.6600 -37.6400 -0.0276 -0.0171 -
0.0419 -0.0562 0.4360 0.4291 0.4800
0.0943 1.5678 -40.4552
53.6800 -37.8700 -0.0900 -0.1445 -
0.0560 -0.0676 0.4772 0.4200 0.4616
0.0942 1.5673 -40.4548
53.7000 -37.6000 0.0459 -0.1068 -
0.0581 -0.0769 0.5175 0.4097 0.4428
0.0942 1.5668 -40.4545
53.7200 -37.6800 -0.1822 0.0743 -
0.0329 -0.0841 0.5563 0.3980 0.4237
0.0941 1.5663 -40.4542
53.7400 -36.9100 0.3930 0.1543
0.0275 -0.0891 0.5929 0.3851 0.4043
0.0940 1.5658 -40.4538

53.7600 -37.5600 -0.0648 -0.0452
0.1183 -0.0913 0.6267 0.3708 0.3846
0.0939 1.5652 -40.4535
53.7800 -37.6700 -0.1422 -0.3266
0.2231 -0.0896 0.6569 0.3552 0.3645
0.0939 1.5646 -40.4532
53.8000 -37.8800 -0.2613 -0.3866
0.3204 -0.0828 0.6831 0.3384 0.3442
0.0938 1.5640 -40.4528
53.8200 -37.0400 0.2296 -0.1695
0.3872 -0.0690 0.7046 0.3203 0.3236
0.0937 1.5634 -40.4525
53.8400 -36.9000 0.0543 0.1839
0.4018 -0.0466 0.7210 0.3009 0.3027
0.0936 1.5627 -40.4521
53.8600 -36.7900 -0.0828 0.4958
0.3458 -0.0144 0.7318 0.2802 0.2816
0.0934 1.5620 -40.4518
53.8800 -36.4100 0.2120 0.6286
0.2044 0.0271 0.7364 0.2584 0.2603
0.0933 1.5613 -40.4515
53.9000 -36.9900 -0.0982 0.5514 -
0.0197 0.0768 0.7346 0.2353 0.2388
0.0932 1.5606 -40.4511
53.9200 -37.3600 0.0927 0.3113 -
0.2911 0.1326 0.7261 0.2112 0.2170
0.0931 1.5598 -40.4508
53.9400 -38.1700 -0.0345 -0.0860 -
0.5577 0.1928 0.7105 0.1860 0.1952
0.0930 1.5590 -40.4504
53.9600 -38.6100 0.2688 -0.6085 -
0.7646 0.2553 0.6876 0.1597 0.1731
0.0928 1.5582 -40.4501
53.9800 -39.1300 0.1846 -0.9688 -
0.8589 0.3179 0.6575 0.1326 0.1509
0.0927 1.5574 -40.4497
54.0000 -40.0900 -0.9050 -0.7794 -
0.8023 0.3783 0.6201 0.1046 0.1286
0.0926 1.5565 -40.4494
54.0200 -37.5800 0.5742 0.0159 -
0.6054 0.4325 0.5755 0.0758 0.1061
0.0925 1.5556 -40.4490
54.0400 -36.6500 0.6099 0.7263 -
0.3013 0.4764 0.5237 0.0464 0.0836
0.0923 1.5547 -40.4486
54.0600 -37.1600 -0.2699 0.7391
0.0716 0.5057 0.4652 0.0164 0.0610
0.0922 1.5538 -40.4483
54.0800 -36.9900 0.3532 0.1015
0.4609 0.5161 0.4002 -0.0139 0.0383
0.0921 1.5528 -40.4479

54.1000 -38.2800 -0.4955 -0.5299
0.7923 0.5032 0.3293 -0.0446 0.0156
0.0920 1.5518 -40.4476
54.1200 -37.8600 -0.0557 -0.6324
0.9911 0.4627 0.2529 -0.0755 -0.0072
0.0918 1.5508 -40.4472
54.1400 -37.6500 -0.2198 -0.1000
1.0180 0.3919 0.1721 -0.1064 -0.0299
0.0917 1.5498 -40.4468
54.1600 -37.4300 -0.3587 0.6757
0.8821 0.2926 0.0876 -0.1373 -0.0527
0.0916 1.5487 -40.4465
54.1800 -36.2200 0.9423 1.0847
0.6075 0.1709 0.0007 -0.1681 -0.0754
0.0915 1.5477 -40.4461
54.2000 -37.7000 0.4876 0.8259
0.2353 0.0345 -0.0878 -0.1985 -0.0982
0.0914 1.5466 -40.4457
54.2200 -40.7200 -1.2977 0.1440 -
0.1676 -0.1090 -0.1767 -0.2286 -0.1208
0.0913 1.5454 -40.4454
54.2400 -41.5900 -0.9053 -0.5665 -
0.5283 -0.2518 -0.2651 -0.2582 -0.1434
0.0912 1.5443 -40.4450
54.2600 -40.0700 1.6926 -0.9472 -
0.7750 -0.3861 -0.3520 -0.2872 -0.1659
0.0911 1.5431 -40.4446
54.2800 -42.2600 -0.3193 -0.7380 -
0.8515 -0.5044 -0.4362 -0.3154 -0.1884
0.0910 1.5419 -40.4442
54.3000 -43.0000 -1.6224 -0.1255 -
0.7615 -0.6007 -0.5169 -0.3428 -0.2107
0.0909 1.5407 -40.4439
54.3200 -39.3100 1.4878 0.5057 -
0.5477 -0.6730 -0.5930 -0.3692 -0.2328
0.0909 1.5394 -40.4435
54.3400 -41.1000 -0.5788 0.5498 -
0.2618 -0.7218 -0.6635 -0.3945 -0.2549
0.0908 1.5381 -40.4431
54.3600 -41.5400 -0.7279 0.1660
0.0339 -0.7480 -0.7277 -0.4186 -0.2767
0.0907 1.5368 -40.4427
54.3800 -41.2500 -0.3639 -0.0947
0.2757 -0.7529 -0.7848 -0.4414 -0.2984
0.0907 1.5355 -40.4423
54.4000 -40.7700 0.0229 -0.0425
0.4181 -0.7379 -0.8343 -0.4628 -0.3199
0.0906 1.5341 -40.4419
54.4200 -40.4300 0.3311 0.0946
0.4530 -0.7052 -0.8756 -0.4827 -0.3412
0.0906 1.5327 -40.4416

54.4400 -40.8300 -0.1845 0.1628
0.3904 -0.6578 -0.9085 -0.5008 -0.3622
0.0906 1.5313 -40.4412
54.4600 -40.8200 0.0206 0.1454
0.2545 -0.5998 -0.9329 -0.5173 -0.3830
0.0906 1.5299 -40.4408
54.4800 -41.0200 0.1246 0.0383
0.0840 -0.5357 -0.9488 -0.5320 -0.4036
0.0906 1.5284 -40.4404
54.5000 -41.1000 0.3797 -0.1594 -
0.0744 -0.4701 -0.9562 -0.5448 -0.4239
0.0906 1.5270 -40.4400
54.5200 -41.7900 -0.0602 -0.2938 -
0.1806 -0.4069 -0.9555 -0.5558 -0.4438
0.0906 1.5254 -40.4396
54.5400 -42.2900 -0.8134 -0.1397 -
0.2101 -0.3486 -0.9468 -0.5649 -0.4635
0.0907 1.5239 -40.4392
54.5600 -40.2100 0.8302 0.2092 -
0.1720 -0.2954 -0.9308 -0.5723 -0.4829
0.0907 1.5223 -40.4388
54.5800 -41.5500 -0.5273 0.1731 -
0.0945 -0.2449 -0.9083 -0.5778 -0.5019
0.0908 1.5208 -40.4384
54.6000 -40.9500 0.1929 -0.1046
0.0034 -0.1943 -0.8801 -0.5817 -0.5205
0.0908 1.5191 -40.4380
54.6200 -41.4100 -0.5092 -0.0830
0.1145 -0.1400 -0.8472 -0.5837 -0.5388
0.0909 1.5175 -40.4376
54.6400 -40.0800 0.6052 0.0098
0.2293 -0.0777 -0.8107 -0.5842 -0.5568
0.0910 1.5158 -40.4372
54.6600 -41.1000 -0.5600 -0.0747
0.3195 -0.0030 -0.7716 -0.5830 -0.5743
0.0912 1.5142 -40.4368
54.6800 -40.2400 0.0375 0.0663
0.3307 0.0875 -0.7308 -0.5802 -0.5915
0.0913 1.5124 -40.4364
54.7000 -40.0900 -0.3419 0.5596
0.2230 0.1950 -0.6888 -0.5759 -0.6083
0.0914 1.5107 -40.4360
54.7200 -38.7900 0.9753 0.6070 -
0.0037 0.3179 -0.6464 -0.5701 -0.6247
0.0916 1.5089 -40.4356
54.7400 -41.4100 -0.8404 -0.0307 -
0.3100 0.4530 -0.6036 -0.5629 -0.6406
0.0918 1.5071 -40.4352
54.7600 -41.7600 -0.4812 -0.5768 -
0.6125 0.5962 -0.5608 -0.5544 -0.6561
0.0920 1.5053 -40.4348

54.7800 -41.0500 0.2258 -0.6795 -
0.8184 0.7432 -0.5181 -0.5446 -0.6712
0.0922 1.5035 -40.4343
54.8000 -40.6100 0.4102 -0.5799 -
0.8503 0.8894 -0.4756 -0.5336 -0.6859
0.0925 1.5016 -40.4339
54.8200 -40.7900 -0.0727 -0.5314 -
0.6762 1.0288 -0.4333 -0.5214 -0.7001
0.0927 1.4997 -40.4335
54.8400 -40.1700 0.0401 -0.6011 -
0.3220 1.1526 -0.3913 -0.5082 -0.7139
0.0930 1.4978 -40.4331
54.8600 -39.7000 -0.1138 -0.5428
0.1302 1.2508 -0.3498 -0.4940 -0.7272
0.0933 1.4958 -40.4327
54.8800 -38.7600 -0.0963 -0.1459
0.5873 1.3133 -0.3089 -0.4788 -0.7401
0.0937 1.4938 -40.4323
54.9000 -37.3000 0.2263 0.4658
0.9627 1.3300 -0.2686 -0.4628 -0.7524
0.0940 1.4918 -40.4318
54.9200 -37.0900 -0.2994 1.0165
1.1747 1.2911 -0.2291 -0.4461 -0.7644
0.0944 1.4898 -40.4314
54.9400 -36.3600 0.3167 1.1961
1.1547 1.1891 -0.1903 -0.4286 -0.7758
0.0948 1.4878 -40.4310
54.9600 -37.3900 0.0415 0.7658
0.8903 1.0275 -0.1521 -0.4106 -0.7868
0.0952 1.4857 -40.4306
54.9800 -38.6900 0.3415 -0.1708
0.4590 0.8168 -0.1145 -0.3922 -0.7972
0.0956 1.4836 -40.4301
55.0000 -41.2400 -0.6878 -0.9538 -
0.0388 0.5686 -0.0772 -0.3734 -0.8072
0.0961 1.4814 -40.4297
55.0200 -40.6300 0.5021 -0.8726 -
0.5083 0.2947 -0.0401 -0.3543 -0.8167
0.0965 1.4793 -40.4293
55.0400 -41.5300 -0.5496 -0.1264 -
0.8773 0.0068 -0.0030 -0.3351 -0.8257
0.0970 1.4771 -40.4288
55.0600 -40.8100 0.1166 0.4234 -
1.0799 -0.2833 0.0341 -0.3159 -0.8342
0.0976 1.4749 -40.4284
55.0800 -40.2800 0.8518 0.4245 -
1.0726 -0.5644 0.0715 -0.2968 -0.8422
0.0981 1.4726 -40.4280
55.1000 -42.0700 -0.5055 -0.0032 -
0.8726 -0.8264 0.1092 -0.2779 -0.8496
0.0987 1.4703 -40.4275

55.1200 -42.5800 -0.8004 -0.3419 -
0.5430 -1.0611 0.1476 -0.2593 -0.8566
0.0993 1.4680 -40.4271
55.1400 -40.6000 0.8512 -0.2833 -
0.1564 -1.2611 0.1866 -0.2411 -0.8631
0.1000 1.4657 -40.4267
55.1600 -41.7000 -0.7021 -0.0325
0.2192 -1.4189 0.2264 -0.2234 -0.8690
0.1006 1.4634 -40.4262
55.1800 -40.1600 0.3502 0.1966
0.5162 -1.5275 0.2673 -0.2064 -0.8745
0.1013 1.4610 -40.4258
55.2000 -40.1300 0.0481 0.3144
0.6744 -1.5803 0.3092 -0.1901 -0.8794
0.1020 1.4586 -40.4253
55.2200 -40.2300 0.0147 0.2039
0.6611 -1.5735 0.3522 -0.1746 -0.8838
0.1028 1.4562 -40.4249
55.2400 -40.1500 0.4278 -0.0701
0.4934 -1.5083 0.3959 -0.1599 -0.8877
0.1035 1.4537 -40.4244
55.2600 -41.3100 -0.4498 -0.2091
0.2172 -1.3908 0.4399 -0.1462 -0.8911
0.1043 1.4512 -40.4240
55.2800 -41.5300 -0.6660 -0.0253 -
0.1072 -1.2294 0.4839 -0.1334 -0.8940
0.1051 1.4487 -40.4235
55.3000 -39.7200 0.8045 0.2736 -
0.4008 -1.0330 0.5274 -0.1217 -0.8963
0.1060 1.4462 -40.4231
55.3200 -40.6300 -0.1795 0.1861 -
0.5862 -0.8107 0.5699 -0.1109 -0.8981
0.1069 1.4436 -40.4226
55.3400 -41.1900 -0.5449 -0.2414 -
0.6194 -0.5720 0.6111 -0.1012 -0.8994
0.1078 1.4410 -40.4222
55.3600 -40.0800 0.4727 -0.5803 -
0.4914 -0.3255 0.6503 -0.0925 -0.9002
0.1087 1.4384 -40.4217
55.3800 -40.4600 -0.5747 -0.3635 -
0.2280 -0.0774 0.6872 -0.0850 -0.9004
0.1097 1.4357 -40.4213
55.4000 -38.5400 0.1474 0.1980
0.0992 0.1670 0.7214 -0.0785 -0.9001
0.1106 1.4331 -40.4208
55.4200 -37.3300 0.4189 0.5810
0.4026 0.4027 0.7523 -0.0731 -0.8993
0.1116 1.4304 -40.4203
55.4400 -37.6600 -0.3255 0.5476
0.5996 0.6246 0.7796 -0.0688 -0.8979
0.1127 1.4276 -40.4199

55.4600 -37.2100 0.0427 0.3048
0.6438 0.8275 0.8026 -0.0656 -0.8961
0.1137 1.4249 -40.4194
55.4800 -37.3300 0.1203 0.0325
0.5433 1.0070 0.8210 -0.0635 -0.8937
0.1148 1.4221 -40.4190
55.5000 -37.8300 -0.0666 -0.2246
0.3409 1.1593 0.8343 -0.0624 -0.8908
0.1159 1.4193 -40.4185
55.5200 -37.8700 0.0477 -0.3662
0.0958 1.2823 0.8420 -0.0625 -0.8873
0.1171 1.4164 -40.4180
55.5400 -38.3900 -0.3885 -0.2992 -
0.1320 1.3744 0.8437 -0.0635 -0.8834
0.1182 1.4136 -40.4176
55.5600 -37.3900 0.5534 -0.2182 -
0.2812 1.4342 0.8388 -0.0655 -0.8790
0.1194 1.4107 -40.4171
55.5800 -38.5200 -0.4126 -0.3215 -
0.3110 1.4611 0.8270 -0.0686 -0.8740
0.1206 1.4078 -40.4166
55.6000 -38.4900 -0.2477 -0.4383 -
0.2213 1.4553 0.8083 -0.0725 -0.8686
0.1218 1.4048 -40.4161
55.6200 -37.7800 0.3519 -0.3672 -
0.0366 1.4179 0.7826 -0.0772 -0.8626
0.1231 1.4019 -40.4157
55.6400 -37.7600 -0.0813 -0.1239
0.2011 1.3500 0.7502 -0.0828 -0.8562
0.1243 1.3989 -40.4152
55.6600 -37.2600 0.0853 0.1492
0.4374 1.2532 0.7115 -0.0891 -0.8493
0.1256 1.3958 -40.4147
55.6800 -37.0700 -0.0976 0.4247
0.6118 1.1288 0.6671 -0.0960 -0.8419
0.1269 1.3928 -40.4142
55.7000 -36.8900 -0.0177 0.6596
0.6700 0.9786 0.6176 -0.1035 -0.8340
0.1283 1.3897 -40.4138
55.7200 -36.8800 0.2233 0.7108
0.5830 0.8059 0.5633 -0.1115 -0.8257
0.1296 1.3866 -40.4133
55.7400 -37.9500 -0.1053 0.4987
0.3661 0.6165 0.5051 -0.1200 -0.8169
0.1310 1.3835 -40.4128
55.7600 -38.8600 -0.0009 0.1204
0.0720 0.4173 0.4433 -0.1288 -0.8077
0.1324 1.3803 -40.4123
55.7800 -39.8700 -0.0512 -0.3077 -
0.2356 0.2154 0.3786 -0.1380 -0.7981
0.1338 1.3771 -40.4118

55.8000 -40.5100 0.2105 -0.7041 -
0.4927 0.0180 0.3114 -0.1474 -0.7879
0.1352 1.3739 -40.4113
55.8200 -41.2500 0.0501 -0.8624 -
0.6469 -0.1678 0.2425 -0.1570 -0.7774
0.1366 1.3706 -40.4108
55.8400 -41.9200 -0.6003 -0.5336 -
0.6741 -0.3345 0.1722 -0.1667 -0.7665
0.1381 1.3674 -40.4104
55.8600 -40.1400 0.6387 0.1296 -
0.6012 -0.4753 0.1013 -0.1765 -0.7551
0.1395 1.3641 -40.4099
55.8800 -40.7000 -0.4438 0.5196 -
0.4834 -0.5839 0.0302 -0.1862 -0.7433
0.1410 1.3607 -40.4094
55.9000 -40.1000 0.2529 0.5593 -
0.3920 -0.6557 -0.0405 -0.1959 -0.7311
0.1425 1.3574 -40.4089
55.9200 -40.5700 -0.0292 0.5259 -
0.3769 -0.6882 -0.1102 -0.2054 -0.7186
0.1440 1.3540 -40.4084
55.9400 -41.3400 -0.5330 0.3816 -
0.4310 -0.6838 -0.1783 -0.2147 -0.7056
0.1455 1.3506 -40.4079
55.9600 -40.5200 0.7673 -0.0400 -
0.5043 -0.6497 -0.2441 -0.2237 -0.6923
0.1471 1.3472 -40.4074
55.9800 -42.5500 -0.7788 -0.5173 -
0.5267 -0.5938 -0.3070 -0.2324 -0.6787
0.1486 1.3437 -40.4069
56.0000 -41.4200 0.2133 -0.5002 -
0.4430 -0.5244 -0.3662 -0.2406 -0.6646
0.1502 1.3402 -40.4064
56.0200 -40.7600 0.3140 -0.1705 -
0.2456 -0.4490 -0.4212 -0.2484 -0.6503
0.1518 1.3367 -40.4059
56.0400 -40.6900 -0.1586 0.0597
0.0355 -0.3742 -0.4712 -0.2556 -0.6356
0.1533 1.3332 -40.4054
56.0600 -40.0800 0.1991 0.0395
0.3482 -0.3059 -0.5155 -0.2622 -0.6205
0.1549 1.3296 -40.4049
56.0800 -40.1000 -0.1866 0.0511
0.6226 -0.2496 -0.5535 -0.2682 -0.6052
0.1565 1.3260 -40.4044
56.1000 -39.8500 -0.3210 0.2473
0.8033 -0.2108 -0.5845 -0.2734 -0.5895
0.1582 1.3224 -40.4039
56.1200 -38.8400 0.5432 0.3679
0.8687 -0.1938 -0.6079 -0.2778 -0.5736
0.1598 1.3188 -40.4034

56.1400 -40.0700 -0.3487 0.1151
0.8204 -0.2003 -0.6233 -0.2813 -0.5574
0.1614 1.3151 -40.4028
56.1600 -40.1000 0.0539 -0.2246
0.6848 -0.2285 -0.6306 -0.2839 -0.5409
0.1631 1.3114 -40.4023
56.1800 -40.5900 -0.0935 -0.3451
0.4942 -0.2739 -0.6299 -0.2856 -0.5241
0.1647 1.3077 -40.4018
56.2000 -40.3100 0.3294 -0.2451
0.2745 -0.3308 -0.6218 -0.2863 -0.5071
0.1663 1.3039 -40.4013
56.2200 -41.0800 -0.6197 0.1008
0.0356 -0.3932 -0.6067 -0.2861 -0.4899
0.1680 1.3002 -40.4008
56.2400 -39.8300 0.6557 0.4933 -
0.2098 -0.4549 -0.5850 -0.2850 -0.4724
0.1697 1.2963 -40.4003
56.2600 -40.9400 -0.2286 0.4302 -
0.4349 -0.5097 -0.5573 -0.2829 -0.4548
0.1713 1.2925 -40.3997
56.2800 -41.5500 -0.0582 -0.0947 -
0.6070 -0.5520 -0.5242 -0.2800 -0.4369
0.1730 1.2887 -40.3992
56.3000 -41.7100 0.2949 -0.6356 -
0.6879 -0.5770 -0.4864 -0.2762 -0.4188
0.1747 1.2848 -40.3987
56.3200 -42.5800 -0.6928 -0.6395 -
0.6463 -0.5811 -0.4444 -0.2715 -0.4006
0.1764 1.2809 -40.3982
56.3400 -40.3800 0.6464 -0.0660 -
0.4925 -0.5638 -0.3989 -0.2660 -0.3822
0.1780 1.2770 -40.3977
56.3600 -40.6700 -0.4877 0.4617 -
0.2740 -0.5265 -0.3507 -0.2597 -0.3636
0.1797 1.2730 -40.3971
56.3800 -39.7400 0.0333 0.5647 -
0.0460 -0.4713 -0.3005 -0.2526 -0.3449
0.1814 1.2690 -40.3966
56.4000 -39.3400 0.4251 0.2773
0.1474 -0.4007 -0.2488 -0.2448 -0.3260
0.1831 1.2650 -40.3961
56.4200 -40.1900 -0.2649 -0.1646
0.2847 -0.3174 -0.1962 -0.2362 -0.3070
0.1848 1.2610 -40.3955
56.4400 -39.9700 -0.1701 -0.4161
0.3525 -0.2241 -0.1433 -0.2269 -0.2879
0.1865 1.2569 -40.3950
56.4600 -39.4400 0.0405 -0.2863
0.3509 -0.1242 -0.0906 -0.2169 -0.2687
0.1882 1.2528 -40.3945

56.4800 -38.9900 0.0836 0.0563
0.2958 -0.0217 -0.0388 -0.2063 -0.2494
0.1898 1.2487 -40.3940
56.5000 -38.9400 -0.1580 0.2915
0.2034 0.0791 0.0117 -0.1951 -0.2300
0.1915 1.2446 -40.3934
56.5200 -38.2400 0.4499 0.2186
0.0932 0.1736 0.0603 -0.1833 -0.2105
0.1932 1.2404 -40.3929
56.5400 -39.5500 -0.5815 -0.0309 -
0.0128 0.2573 0.1066 -0.1709 -0.1910
0.1949 1.2363 -40.3923
56.5600 -39.1600 -0.0122 -0.2143 -
0.0900 0.3260 0.1499 -0.1580 -0.1714
0.1965 1.2320 -40.3918
56.5800 -38.3700 0.6634 -0.2881 -
0.1146 0.3765 0.1897 -0.1445 -0.1517
0.1982 1.2278 -40.3913
56.6000 -39.6200 -0.7515 -0.2410 -
0.0787 0.4064 0.2257 -0.1306 -0.1320
0.1999 1.2236 -40.3907
56.6200 -37.9500 0.6147 0.0222 -
0.0032 0.4154 0.2574 -0.1163 -0.1123
0.2015 1.2193 -40.3902
56.6400 -38.4400 -0.3426 0.3362
0.0780 0.4039 0.2845 -0.1015 -0.0925
0.2032 1.2150 -40.3896
56.6600 -38.1000 -0.1214 0.3900
0.1350 0.3735 0.3070 -0.0864 -0.0727
0.2048 1.2106 -40.3891
56.6800 -37.5900 0.5353 0.1086
0.1549 0.3261 0.3249 -0.0709 -0.0529
0.2065 1.2063 -40.3885
56.7000 -38.9000 -0.3796 -0.3267
0.1456 0.2648 0.3381 -0.0550 -0.0331
0.2081 1.2019 -40.3880
56.7200 -39.0500 -0.2018 -0.4714
0.1182 0.1932 0.3469 -0.0389 -0.0134
0.2097 1.1975 -40.3874
56.7400 -38.5800 -0.0648 -0.1760
0.0828 0.1150 0.3514 -0.0226 0.0064
0.2113 1.1931 -40.3869
56.7600 -37.7200 0.5368 0.2515
0.0414 0.0347 0.3517 -0.0060 0.0261
0.2129 1.1886 -40.3863
56.7800 -38.6400 -0.4246 0.4373 -
0.0068 -0.0432 0.3480 0.0107 0.0458
0.2145 1.1842 -40.3858
56.8000 -38.2200 0.2195 0.2412 -
0.0601 -0.1143 0.3406 0.0275 0.0654
0.2161 1.1797 -40.3852

56.8200 -38.4900 0.5449 -0.1435 -
0.1090 -0.1740 0.3295 0.0444 0.0850
0.2177 1.1751 -40.3847
56.8400 -39.7500 -0.5716 -0.3721 -
0.1423 -0.2185 0.3150 0.0613 0.1045
0.2192 1.1706 -40.3841
56.8600 -39.6000 -0.5421 -0.2362 -
0.1535 -0.2451 0.2973 0.0781 0.1239
0.2208 1.1660 -40.3836
56.8800 -38.1700 0.6834 0.0972 -
0.1465 -0.2526 0.2766 0.0948 0.1433
0.2223 1.1614 -40.3830
56.9000 -38.9900 -0.3163 0.2083 -
0.1271 -0.2415 0.2533 0.1113 0.1625
0.2238 1.1568 -40.3824
56.9200 -38.6400 0.1234 0.0651 -
0.1025 -0.2134 0.2276 0.1276 0.1817
0.2253 1.1522 -40.3819
56.9400 -38.8700 -0.1877 -0.0785 -
0.0758 -0.1706 0.2000 0.1436 0.2008
0.2268 1.1475 -40.3813
56.9600 -38.5800 0.0842 -0.0917 -
0.0404 -0.1169 0.1707 0.1593 0.2197
0.2283 1.1428 -40.3807
56.9800 -38.7900 -0.2454 -0.0205
0.0112 -0.0564 0.1402 0.1746 0.2385
0.2298 1.1381 -40.3802
57.0000 -37.7800 0.5836 -0.0343
0.0790 0.0064 0.1089 0.1894 0.2572
0.2312 1.1334 -40.3796
57.0200 -39.0300 -0.6533 -0.0843
0.1472 0.0672 0.0774 0.2037 0.2757
0.2327 1.1287 -40.3790
57.0400 -37.6900 0.3497 0.0319
0.1974 0.1223 0.0460 0.2175 0.2941
0.2341 1.1239 -40.3785
57.0600 -37.5700 0.3326 0.1745
0.2130 0.1688 0.0152 0.2306 0.3124
0.2355 1.1191 -40.3779
57.0800 -38.3200 -0.3756 0.1872
0.1843 0.2046 -0.0145 0.2431 0.3304
0.2368 1.1143 -40.3773
57.1000 -37.9900 0.1377 0.0761
0.1182 0.2287 -0.0428 0.2550 0.3483
0.2382 1.1094 -40.3767
57.1200 -38.2300 0.0773 -0.0973
0.0355 0.2408 -0.0693 0.2660 0.3660
0.2395 1.1046 -40.3762
57.1400 -38.4800 0.0704 -0.2952 -
0.0387 0.2412 -0.0936 0.2763 0.3835
0.2408 1.0997 -40.3756

57.1600 -38.8500 -0.1876 -0.3239 -
0.0915 0.2303 -0.1154 0.2858 0.4008
0.2421 1.0948 -40.3750
57.1800 -38.5900 -0.2816 0.0046 -
0.1208 0.2092 -0.1347 0.2943 0.4179
0.2434 1.0898 -40.3744
57.2000 -37.5100 0.5664 0.3984 -
0.1342 0.1792 -0.1511 0.3020 0.4348
0.2447 1.0849 -40.3739
57.2200 -38.4100 -0.2593 0.3702 -
0.1401 0.1418 -0.1646 0.3088 0.4515
0.2459 1.0799 -40.3733
57.2400 -38.4600 -0.0113 0.0269 -
0.1360 0.0985 -0.1750 0.3146 0.4679
0.2471 1.0749 -40.3727
57.2600 -38.8200 -0.1528 -0.2977 -
0.1117 0.0510 -0.1823 0.3194 0.4841
0.2483 1.0699 -40.3721
57.2800 -38.8000 0.1658 -0.4464 -
0.0557 0.0009 -0.1863 0.3232 0.5000
0.2494 1.0649 -40.3715
57.3000 -38.9000 -0.1894 -0.2773
0.0252 -0.0503 -0.1869 0.3260 0.5156
0.2505 1.0598 -40.3709
57.3200 -38.6800 -0.4187 0.1390
0.1118 -0.1008 -0.1841 0.3279 0.5310
0.2517 1.0547 -40.3703
57.3400 -37.1600 0.7178 0.4367
0.1796 -0.1491 -0.1778 0.3289 0.5460
0.2527 1.0496 -40.3698
57.3600 -37.9400 0.0928 0.2639
0.2065 -0.1936 -0.1679 0.3289 0.5608
0.2538 1.0445 -40.3692
57.3800 -38.5900 -0.1619 -0.1464
0.1857 -0.2327 -0.1543 0.3281 0.5753
0.2548 1.0394 -40.3686
57.4000 -39.4200 -0.7658 -0.2749
0.1207 -0.2651 -0.1371 0.3265 0.5895
0.2558 1.0342 -40.3680
57.4200 -38.3400 0.2378 -0.0339
0.0234 -0.2891 -0.1164 0.3241 0.6034
0.2568 1.0290 -40.3674
57.4400 -37.8400 0.6280 0.2126 -
0.0807 -0.3026 -0.0923 0.3211 0.6169
0.2577 1.0238 -40.3668
57.4600 -38.8700 -0.3470 0.1714 -
0.1644 -0.3038 -0.0650 0.3174 0.6301
0.2586 1.0186 -40.3662
57.4800 -38.9800 -0.2005 -0.0454 -
0.2071 -0.2915 -0.0348 0.3132 0.6430
0.2595 1.0133 -40.3656

57.5000	-38.9700	-0.2395	-0.1581	-
0.1978	-0.2653	-0.0022	0.3085	0.6556
	0.2603	1.0081	-40.3650	
57.5200	-38.2400	0.4417	-0.1616	-
0.1416	-0.2262	0.0323	0.3033	0.6677
	0.2612	1.0028	-40.3644	
57.5400	-38.1800	0.1830	-0.0926	-
0.0570	-0.1760	0.0683	0.2977	0.6796
	0.2619	0.9975	-40.3638	
57.5600	-38.7200	-0.7047	0.0884	
0.0308	-0.1176	0.1050	0.2918	0.6911
	0.2627	0.9922	-40.3632	
57.5800	-37.0100	0.7866	0.2338	
0.0992	-0.0539	0.1419	0.2856	0.7022
	0.2634	0.9868	-40.3626	
57.6000	-38.2500	-0.6323	0.1127	
0.1321	0.0120	0.1784	0.2792	0.7130
	0.2641	0.9815	-40.3620	
57.6200	-38.1000	-0.2271	-0.1083	
0.1293	0.0772	0.2138	0.2727	0.7233
	0.2647	0.9761	-40.3614	
57.6400	-37.3500	0.5650	-0.2194	
0.1034	0.1393	0.2475	0.2660	0.7333
	0.2653	0.9707	-40.3607	
57.6600	-37.8700	-0.1162	-0.1398	
0.0694	0.1965	0.2789	0.2592	0.7430
	0.2659	0.9653	-40.3601	
57.6800	-37.8400	-0.4770	0.0547	
0.0376	0.2476	0.3074	0.2525	0.7522
	0.2665	0.9598	-40.3595	
57.7000	-36.8100	0.4271	0.2169	
0.0156	0.2917	0.3322	0.2457	0.7610
	0.2670	0.9544	-40.3589	
57.7200	-36.9300	0.3823	0.1695	
0.0053	0.3282	0.3528	0.2391	0.7695
	0.2674	0.9489	-40.3583	
57.7400	-37.6200	-0.2082	-0.0845	
0.0021	0.3567	0.3688	0.2325	0.7776
	0.2679	0.9434	-40.3577	
57.7600	-38.0500	-0.4572	-0.2457	-
0.0031	0.3769	0.3797	0.2262	0.7852
	0.2683	0.9379	-40.3571	
57.7800	-37.4100	0.1655	-0.0994	-
0.0193	0.3886	0.3850	0.2200	0.7925
	0.2686	0.9324	-40.3565	
57.8000	-37.0700	0.1802	0.1756	-
0.0473	0.3918	0.3847	0.2141	0.7993
	0.2689	0.9268	-40.3558	
57.8200	-37.0500	0.1353	0.3233	-
0.0852	0.3861	0.3784	0.2084	0.8057
	0.2692	0.9212	-40.3552	

57.8400 -37.2400 0.0595 0.2639 -
0.1251 0.3711 0.3662 0.2030 0.8117
0.2694 0.9156 -40.3546
57.8600 -37.5800 -0.0579 0.1019 -
0.1509 0.3463 0.3480 0.1979 0.8173
0.2696 0.9100 -40.3540
57.8800 -37.7500 0.0102 -0.1395 -
0.1417 0.3124 0.3241 0.1931 0.8225
0.2698 0.9044 -40.3533
57.9000 -37.5600 0.6153 -0.4803 -
0.0836 0.2707 0.2947 0.1886 0.8272
0.2699 0.8988 -40.3527
57.9200 -38.7500 -0.3895 -0.7169
0.0218 0.2241 0.2601 0.1844 0.8316
0.2699 0.8931 -40.3521
57.9400 -39.1400 -0.9867 -0.5120
0.1566 0.1761 0.2207 0.1806 0.8355
0.2699 0.8875 -40.3515
57.9600 -37.3900 -0.0925 0.2093
0.2763 0.1306 0.1767 0.1771 0.8390
0.2699 0.8818 -40.3508
57.9800 -35.8100 0.9913 0.8851
0.3179 0.0914 0.1286 0.1740 0.8420
0.2698 0.8761 -40.3502
58.0000 -36.9700 -0.0836 0.8688
0.2279 0.0612 0.0768 0.1712 0.8446
0.2697 0.8703 -40.3496
58.0200 -37.7300 0.2936 0.1551
0.0113 0.0407 0.0218 0.1688 0.8468
0.2696 0.8646 -40.3489
58.0400 -39.6800 -0.7110 -0.5207 -
0.2689 0.0294 -0.0360 0.1668 0.8486
0.2694 0.8588 -40.3483
58.0600 -39.1700 0.2686 -0.6255 -
0.5320 0.0265 -0.0958 0.1652 0.8499
0.2691 0.8531 -40.3477
58.0800 -39.7700 -0.3606 -0.3953 -
0.6826 0.0312 -0.1570 0.1639 0.8508
0.2688 0.8473 -40.3470
58.1000 -39.0200 0.3702 -0.3499 -
0.6427 0.0427 -0.2187 0.1631 0.8513
0.2685 0.8415 -40.3464
58.1200 -39.0600 0.3037 -0.5671 -
0.4029 0.0579 -0.2801 0.1626 0.8514
0.2681 0.8356 -40.3458
58.1400 -39.6900 -0.2793 -0.7217 -
0.0334 0.0717 -0.3403 0.1626 0.8510
0.2677 0.8298 -40.3451
58.1600 -39.1700 -0.6427 -0.3743
0.3698 0.0782 -0.3983 0.1629 0.8501
0.2672 0.8240 -40.3445

58.1800 -36.7700 0.7169 0.4075
0.7186 0.0716 -0.4534 0.1637 0.8489
0.2667 0.8181 -40.3438
58.2000 -37.1200 -0.3518 0.9947
0.9311 0.0460 -0.5047 0.1650 0.8472
0.2662 0.8122 -40.3432
58.2200 -36.6500 0.3338 0.9622
0.9417 -0.0031 -0.5514 0.1666 0.8451
0.2655 0.8063 -40.3426
58.2400 -37.3000 0.3952 0.4053
0.7555 -0.0755 -0.5931 0.1687 0.8425
0.2649 0.8004 -40.3419
58.2600 -39.2800 -0.3064 -0.3105
0.4417 -0.1663 -0.6292 0.1712 0.8395
0.2642 0.7945 -40.3413
58.2800 -40.4100 -0.5997 -0.7566
0.0815 -0.2693 -0.6595 0.1742 0.8361
0.2635 0.7885 -40.3406
58.3000 -39.8700 0.5014 -0.7386 -
0.2515 -0.3783 -0.6834 0.1776 0.8323
0.2627 0.7826 -40.3400
58.3200 -40.2900 0.1999 -0.4319 -
0.5095 -0.4869 -0.7008 0.1815 0.8281
0.2619 0.7766 -40.3393
58.3400 -40.9000 -0.6406 -0.0611 -
0.6733 -0.5886 -0.7112 0.1858 0.8234
0.2610 0.7706 -40.3387
58.3600 -39.6700 0.5380 0.2177 -
0.7494 -0.6764 -0.7143 0.1905 0.8183
0.2601 0.7646 -40.3380
58.3800 -40.1100 0.0882 0.3013 -
0.7595 -0.7434 -0.7097 0.1956 0.8128
0.2592 0.7586 -40.3374
58.4000 -40.9900 -0.5629 0.2148 -
0.7341 -0.7836 -0.6972 0.2011 0.8069
0.2582 0.7525 -40.3367
58.4200 -39.7000 0.6660 0.0951 -
0.6976 -0.7930 -0.6765 0.2070 0.8006
0.2571 0.7465 -40.3360
58.4400 -40.6800 -0.1802 0.0387 -
0.6569 -0.7703 -0.6478 0.2131 0.7940
0.2561 0.7404 -40.3354
58.4600 -41.1300 -0.7619 -0.0118 -
0.5981 -0.7174 -0.6117 0.2194 0.7869
0.2550 0.7344 -40.3347
58.4800 -39.3300 0.9411 -0.1358 -
0.4975 -0.6389 -0.5688 0.2259 0.7794
0.2538 0.7283 -40.3341
58.5000 -40.8000 -0.8984 -0.1800 -
0.3395 -0.5401 -0.5199 0.2326 0.7716
0.2526 0.7222 -40.3334

58.5200 -38.7200 0.7299 0.0544 -
0.1288 -0.4262 -0.4655 0.2393 0.7634
0.2514 0.7161 -40.3327
58.5400 -38.8700 -0.1197 0.1975
0.1147 -0.3023 -0.4064 0.2461 0.7548
0.2501 0.7099 -40.3321
58.5600 -38.6900 -0.2267 0.0285
0.3665 -0.1731 -0.3434 0.2529 0.7459
0.2488 0.7038 -40.3314
58.5800 -38.2300 0.1065 -0.2239
0.6006 -0.0429 -0.2771 0.2596 0.7366
0.2474 0.6976 -40.3307
58.6000 -38.1500 -0.2434 -0.1548
0.7903 0.0841 -0.2083 0.2662 0.7270
0.2461 0.6915 -40.3301
58.6200 -37.7500 -0.5556 0.2054
0.9110 0.2044 -0.1376 0.2727 0.7171
0.2446 0.6853 -40.3294
58.6400 -35.8600 1.0569 0.3542
0.9324 0.3153 -0.0658 0.2789 0.7068
0.2432 0.6791 -40.3287
58.6600 -38.0800 -0.8895 0.0226
0.8304 0.4145 0.0064 0.2849 0.6962
0.2417 0.6729 -40.3281
58.6800 -38.2500 -0.8039 -0.2521
0.6035 0.5007 0.0782 0.2906 0.6853
0.2401 0.6667 -40.3274
58.7000 -37.4200 0.1248 -0.1118
0.2687 0.5728 0.1490 0.2959 0.6742
0.2385 0.6605 -40.3267
58.7200 -36.7100 0.9224 0.1702 -
0.1262 0.6294 0.2181 0.3008 0.6627
0.2369 0.6543 -40.3261
58.7400 -37.9200 -0.1025 0.1879 -
0.5159 0.6693 0.2850 0.3053 0.6509
0.2353 0.6480 -40.3254
58.7600 -38.2700 0.3044 -0.1994 -
0.8369 0.6911 0.3492 0.3093 0.6389
0.2336 0.6417 -40.3247
58.7800 -39.6200 -0.7126 -0.5369 -
1.0310 0.6937 0.4103 0.3127 0.6266
0.2319 0.6355 -40.3240
58.8000 -39.1700 -0.1630 -0.4159 -
1.0545 0.6762 0.4678 0.3156 0.6141
0.2301 0.6292 -40.3233
58.8200 -38.1700 0.1527 0.0035 -
0.9110 0.6390 0.5217 0.3177 0.6013
0.2283 0.6229 -40.3227
58.8400 -37.3000 0.4893 0.3045 -
0.6366 0.5841 0.5717 0.3192 0.5883
0.2265 0.6166 -40.3220

58.8600	-37.1800	0.3020	0.2507	-
0.2761	0.5139	0.6177	0.3200	0.5750
	0.2247	0.6103	-40.3213	
58.8800	-37.6400	-0.3183	0.0266	
0.1233	0.4310	0.6596	0.3199	0.5616
	0.2228	0.6040	-40.3206	
58.9000	-37.5900	-0.4224	-0.0493	
0.5136	0.3379	0.6974	0.3191	0.5479
	0.2208	0.5976	-40.3199	
58.9200	-36.2500	0.4843	0.0659	
0.8586	0.2371	0.7310	0.3174	0.5340
	0.2189	0.5913	-40.3192	
58.9400	-36.4300	0.0842	0.0998	
1.1336	0.1313	0.7604	0.3148	0.5200
	0.2169	0.5849	-40.3186	
58.9600	-36.4600	0.0293	0.0028	
1.3128	0.0232	0.7855	0.3112	0.5058
	0.2149	0.5786	-40.3179	
58.9800	-37.1800	-0.6389	0.0772	
1.3653	-0.0846	0.8062	0.3067	0.4914
	0.2128	0.5722	-40.3172	
59.0000	-36.6000	-0.1068	0.3656	
1.2696	-0.1897	0.8226	0.3012	0.4768
	0.2107	0.5658	-40.3165	
59.0200	-36.0200	0.6954	0.5395	
1.0266	-0.2892	0.8345	0.2947	0.4621
	0.2086	0.5594	-40.3158	
59.0400	-36.7800	0.7240	0.2744	
0.6597	-0.3801	0.8420	0.2872	0.4472
	0.2064	0.5530	-40.3151	
59.0600	-38.3900	0.1297	-0.2552	
0.2156	-0.4593	0.8448	0.2787	0.4323
	0.2043	0.5466	-40.3144	
59.0800	-40.4300	-1.0480	-0.5742	-
0.2513	-0.5236	0.8430	0.2691	0.4172
	0.2020	0.5402	-40.3137	
59.1000	-39.9300	-0.1843	-0.3731	-
0.6893	-0.5698	0.8365	0.2585	0.4019
	0.1998	0.5338	-40.3130	
59.1200	-38.8200	0.9196	0.0976	-
1.0584	-0.5941	0.8252	0.2470	0.3866
	0.1975	0.5274	-40.3123	
59.1400	-40.4100	-0.7251	0.4584	-
1.3298	-0.5923	0.8091	0.2345	0.3712
	0.1952	0.5209	-40.3116	
59.1600	-39.7100	0.0458	0.5502	-
1.4883	-0.5609	0.7881	0.2211	0.3557
	0.1929	0.5145	-40.3109	
59.1800	-39.7000	0.2575	0.3984	-
1.5259	-0.4985	0.7623	0.2068	0.3401
	0.1905	0.5080	-40.3102	

59.2000	-40.1500	0.3018	-0.0948	-
1.4352	-0.4081	0.7318	0.1916	0.3245
	0.1881	0.5015	-40.3095	
59.2200	-40.9600	-0.2102	-0.7461	-
1.2153	-0.2950	0.6967	0.1757	0.3088
	0.1857	0.4951	-40.3088	
59.2400	-41.1900	-0.3853	-1.1247	-
0.8723	-0.1657	0.6571	0.1591	0.2931
	0.1833	0.4886	-40.3081	
59.2600	-39.9200	0.1284	-1.0674	-
0.4224	-0.0279	0.6132	0.1418	0.2773
	0.1808	0.4821	-40.3074	
59.2800	-38.6100	0.5782	-0.7488	
0.0917	0.1106	0.5652	0.1239	0.2614
	0.1783	0.4756	-40.3067	
59.3000	-38.8600	-0.7002	-0.2422	
0.6048	0.2421	0.5132	0.1055	0.2456
	0.1758	0.4691	-40.3060	
59.3200	-36.5800	0.3566	0.4770	
1.0419	0.3585	0.4575	0.0867	0.2297
	0.1732	0.4626	-40.3053	
59.3400	-36.0900	0.0282	1.0832	
1.3286	0.4520	0.3982	0.0675	0.2139
	0.1706	0.4561	-40.3046	
59.3600	-36.0200	0.0190	1.2274	
1.4021	0.5151	0.3357	0.0480	0.1980
	0.1680	0.4495	-40.3039	
59.3800	-36.5700	0.0443	0.8224	
1.2487	0.5441	0.2704	0.0282	0.1821
	0.1654	0.4430	-40.3032	
59.4000	-37.4700	0.3925	0.0117	
0.9247	0.5416	0.2027	0.0083	0.1663
	0.1627	0.4365	-40.3025	
59.4200	-39.8000	-0.6866	-0.7112	
0.5082	0.5125	0.1331	-0.0117	0.1505
	0.1600	0.4299	-40.3017	
59.4400	-39.9400	-0.0973	-0.8629	
0.0779	0.4617	0.0622	-0.0318	0.1347
	0.1573	0.4234	-40.3010	
59.4600	-39.7500	0.4715	-0.6217	-
0.2918	0.3943	-0.0095	-0.0518	0.1190
	0.1546	0.4168	-40.3003	
59.4800	-40.2200	0.2976	-0.4248	-
0.5398	0.3155	-0.0815	-0.0717	0.1033
	0.1518	0.4103	-40.2996	
59.5000	-40.6700	0.0628	-0.4017	-
0.6478	0.2301	-0.1531	-0.0915	0.0877
	0.1490	0.4037	-40.2989	
59.5200	-41.1800	-0.4240	-0.3004	-
0.6436	0.1429	-0.2238	-0.1110	0.0721
	0.1462	0.3971	-40.2982	

59.5400 -40.8600 -0.2580 0.0614 -
0.5718 0.0590 -0.2930 -0.1302 0.0566
0.1434 0.3905 -40.2974
59.5600 -39.7400 0.5626 0.4849 -
0.4672 -0.0165 -0.3602 -0.1490 0.0412
0.1405 0.3840 -40.2967
59.5800 -40.5600 -0.4036 0.6228 -
0.3534 -0.0789 -0.4249 -0.1673 0.0259
0.1377 0.3774 -40.2960
59.6000 -40.2800 0.0342 0.4016 -
0.2361 -0.1260 -0.4864 -0.1852 0.0107
0.1348 0.3708 -40.2953
59.6200 -40.6300 0.2813 -0.0671 -
0.1121 -0.1589 -0.5441 -0.2025 -0.0044
0.1318 0.3642 -40.2946
59.6400 -41.6000 -0.3849 -0.5138
0.0200 -0.1798 -0.5977 -0.2192 -0.0193
0.1289 0.3576 -40.2938
59.6600 -40.9300 0.3544 -0.5872
0.1570 -0.1912 -0.6464 -0.2351 -0.0342
0.1259 0.3510 -40.2931
59.6800 -41.4900 -0.5811 -0.1529
0.2823 -0.1944 -0.6897 -0.2503 -0.0489
0.1229 0.3444 -40.2924
59.7000 -39.5900 0.7384 0.3779
0.3639 -0.1897 -0.7271 -0.2647 -0.0635
0.1199 0.3377 -40.2916
59.7200 -40.8900 -0.6821 0.5715
0.3677 -0.1778 -0.7582 -0.2782 -0.0779
0.1169 0.3311 -40.2909
59.7400 -40.3600 0.0801 0.5110
0.2746 -0.1604 -0.7824 -0.2907 -0.0922
0.1139 0.3245 -40.2902
59.7600 -40.4100 0.4978 0.2726
0.1023 -0.1399 -0.7997 -0.3022 -0.1063
0.1108 0.3179 -40.2895
59.7800 -41.1700 0.3818 -0.2166 -
0.0932 -0.1184 -0.8098 -0.3126 -0.1202
0.1077 0.3112 -40.2887
59.8000 -42.8700 -0.7478 -0.6230 -
0.2476 -0.0982 -0.8126 -0.3219 -0.1340
0.1046 0.3046 -40.2880
59.8200 -41.5100 0.4489 -0.5005 -
0.3070 -0.0807 -0.8082 -0.3300 -0.1475
0.1015 0.2980 -40.2872
59.8400 -41.6300 0.0196 -0.0569 -
0.2626 -0.0677 -0.7966 -0.3368 -0.1609
0.0984 0.2913 -40.2865
59.8600 -41.0400 0.1021 0.2454 -
0.1344 -0.0599 -0.7781 -0.3425 -0.1740
0.0952 0.2847 -40.2858

59.8800 -40.6000 0.3076 0.1705
0.0440 -0.0581 -0.7526 -0.3469 -0.1870
0.0920 0.2781 -40.2850
59.9000 -40.8100 0.2494 -0.1162
0.2199 -0.0627 -0.7206 -0.3501 -0.1997
0.0888 0.2714 -40.2843
59.9200 -41.9000 -0.7796 -0.1568
0.3364 -0.0741 -0.6824 -0.3521 -0.2122
0.0856 0.2648 -40.2836
59.9400 -40.9800 -0.2487 0.1710
0.3600 -0.0916 -0.6384 -0.3530 -0.2245
0.0824 0.2581 -40.2828
59.9600 -39.7600 0.8900 0.4193
0.2888 -0.1134 -0.5891 -0.3527 -0.2365
0.0792 0.2514 -40.2821
59.9800 -41.6600 -0.7155 0.2075
0.1369 -0.1354 -0.5353 -0.3513 -0.2483
0.0759 0.2448 -40.2813
60.0000 -41.3400 -0.0646 -0.0821 -
0.0567 -0.1533 -0.4776 -0.3489 -0.2599
0.0726 0.2381 -40.2806
60.0200 -41.5200 0.0615 -0.1393 -
0.2427 -0.1627 -0.4170 -0.3454 -0.2713
0.0694 0.2315 -40.2798
60.0400 -41.3200 0.3542 -0.1654 -
0.3719 -0.1596 -0.3542 -0.3410 -0.2824
0.0661 0.2248 -40.2791
60.0600 -41.3700 0.4248 -0.2511 -
0.4072 -0.1410 -0.2899 -0.3356 -0.2933
0.0628 0.2182 -40.2783
60.0800 -42.4100 -0.9526 -0.1634 -
0.3446 -0.1064 -0.2249 -0.3293 -0.3039
0.0594 0.2115 -40.2776
60.1000 -39.9900 1.0148 0.1155 -
0.2043 -0.0583 -0.1600 -0.3220 -0.3144
0.0561 0.2048 -40.2768
60.1200 -41.1000 -0.4606 0.1487 -
0.0235 -0.0007 -0.0959 -0.3140 -0.3246
0.0528 0.1982 -40.2761
60.1400 -41.1100 -0.6167 0.0367
0.1473 0.0620 -0.0332 -0.3051 -0.3345
0.0494 0.1915 -40.2753
60.1600 -40.1600 0.1106 0.0680
0.2609 0.1251 0.0274 -0.2954 -0.3443
0.0461 0.1848 -40.2746
60.1800 -39.7400 0.1634 0.2536
0.2956 0.1843 0.0852 -0.2850 -0.3537
0.0427 0.1782 -40.2738
60.2000 -39.5300 0.3393 0.2898
0.2562 0.2359 0.1397 -0.2738 -0.3630
0.0393 0.1715 -40.2731

60.2200 -40.3200 -0.2409 0.0562
0.1689 0.2771 0.1902 -0.2620 -0.3720
0.0360 0.1648 -40.2723
60.2400 -40.4100 -0.0780 -0.2760
0.0684 0.3058 0.2361 -0.2495 -0.3808
0.0326 0.1582 -40.2716
60.2600 -40.2200 0.4667 -0.4499 -
0.0140 0.3206 0.2771 -0.2364 -0.3894
0.0292 0.1515 -40.2708
60.2800 -41.1400 -0.7027 -0.2620 -
0.0585 0.3209 0.3127 -0.2228 -0.3977
0.0258 0.1448 -40.2701
60.3000 -40.3100 -0.3251 0.1765 -
0.0675 0.3074 0.3428 -0.2085 -0.4059
0.0224 0.1382 -40.2693
60.3200 -38.6800 1.0078 0.4616 -
0.0566 0.2815 0.3674 -0.1938 -0.4137
0.0190 0.1315 -40.2685
60.3400 -40.1500 -0.1774 0.2721 -
0.0435 0.2456 0.3866 -0.1787 -0.4214
0.0156 0.1248 -40.2678
60.3600 -41.0300 -0.8417 -0.0897 -
0.0308 0.2017 0.4009 -0.1631 -0.4288
0.0121 0.1182 -40.2670
60.3800 -40.6300 -0.0415 -0.2334 -
0.0149 0.1519 0.4104 -0.1473 -0.4360
0.0087 0.1115 -40.2662
60.4000 -39.9700 0.3076 -0.1288
0.0058 0.0982 0.4158 -0.1311 -0.4430
0.0053 0.1048 -40.2655
60.4200 -40.0300 0.2429 -0.0080
0.0316 0.0426 0.4173 -0.1148 -0.4497
0.0019 0.0982 -40.2647
60.4400 -40.3800 -0.1931 -0.0090
0.0615 -0.0130 0.4157 -0.0984 -0.4562
-0.0015 0.0915 -40.2639
60.4600 -39.9900 0.3907 -0.0980
0.0863 -0.0667 0.4112 -0.0820 -0.4625
-0.0049 0.0848 -40.2632
60.4800 -40.9400 -0.4624 -0.1007
0.0876 -0.1164 0.4045 -0.0656 -0.4686
-0.0083 0.0782 -40.2624
60.5000 -40.2000 0.1567 0.1117
0.0502 -0.1602 0.3959 -0.0494 -0.4745
-0.0117 0.0715 -40.2616
60.5200 -40.2100 0.0247 0.3487 -
0.0248 -0.1969 0.3861 -0.0334 -0.4801
-0.0151 0.0649 -40.2609
60.5400 -39.9000 0.4197 0.3531 -
0.1217 -0.2256 0.3754 -0.0177 -0.4855
-0.0185 0.0582 -40.2601

60.5600 -41.2700 -0.5587 0.1687 -
0.2162 -0.2457 0.3643 -0.0024 -0.4907
-0.0219 0.0516 -40.2593
60.5800 -40.9500 -0.0499 -0.0708 -
0.2768 -0.2570 0.3533 0.0125 -0.4957
-0.0253 0.0449 -40.2585
60.6000 -40.5100 0.7129 -0.3304 -
0.2706 -0.2599 0.3428 0.0267 -0.5005
-0.0286 0.0383 -40.2578
60.6200 -42.0200 -0.6754 -0.5155 -
0.1811 -0.2550 0.3330 0.0404 -0.5050
-0.0320 0.0317 -40.2570
60.6400 -41.4300 -0.3758 -0.3403 -
0.0179 -0.2437 0.3244 0.0533 -0.5094
-0.0354 0.0250 -40.2562
60.6600 -40.5500 -0.2970 0.1755
0.1753 -0.2280 0.3172 0.0655 -0.5135
-0.0387 0.0184 -40.2554
60.6800 -38.7900 0.9685 0.4728
0.3360 -0.2096 0.3118 0.0768 -0.5174
-0.0420 0.0118 -40.2547
60.7000 -40.4900 -0.5820 0.3274
0.4063 -0.1903 0.3081 0.0872 -0.5211
-0.0454 0.0051 -40.2539
60.7200 -40.9200 -0.7444 0.0884
0.3668 -0.1710 0.3062 0.0966 -0.5246
-0.0487 -0.0015 -40.2531
60.7400 -39.6400 0.7631 -0.0381
0.2281 -0.1514 0.3060 0.1051 -0.5279
-0.0520 -0.0081 -40.2523
60.7600 -41.2800 -0.5526 -0.1257
0.0319 -0.1305 0.3071 0.1127 -0.5310
-0.0553 -0.0147 -40.2515
60.7800 -40.5600 0.3541 -0.1612 -
0.1676 -0.1070 0.3093 0.1193 -0.5338
-0.0585 -0.0214 -40.2507
60.8000 -41.2700 -0.3453 -0.0703 -
0.3213 -0.0797 0.3122 0.1249 -0.5365
-0.0618 -0.0280 -40.2500
60.8200 -40.4300 0.5368 -0.0692 -
0.4055 -0.0474 0.3153 0.1297 -0.5390
-0.0650 -0.0346 -40.2492
60.8400 -41.6000 -0.6541 -0.0609 -
0.4261 -0.0089 0.3185 0.1336 -0.5412
-0.0682 -0.0412 -40.2484
60.8600 -40.7600 -0.0031 0.1410 -
0.3970 0.0359 0.3213 0.1367 -0.5433
-0.0714 -0.0478 -40.2476
60.8800 -39.7500 0.6900 0.2770 -
0.3202 0.0858 0.3233 0.1390 -0.5451
-0.0746 -0.0544 -40.2468

60.9000 -41.2300 -0.7309 0.0666 -
0.1961 0.1382 0.3242 0.1405 -0.5468
-0.0778 -0.0609 -40.2460
60.9200 -40.2400 0.4488 -0.3022 -
0.0351 0.1896 0.3238 0.1412 -0.5482
-0.0809 -0.0675 -40.2452
60.9400 -40.2800 0.3122 -0.4558
0.1456 0.2358 0.3216 0.1413 -0.5494
-0.0840 -0.0741 -40.2444
60.9600 -41.0500 -0.7434 -0.2766
0.3172 0.2728 0.3173 0.1406 -0.5505
-0.0872 -0.0807 -40.2436
60.9800 -40.0400 -0.4304 0.1793
0.4440 0.2967 0.3107 0.1393 -0.5513
-0.0902 -0.0872 -40.2429
61.0000 -39.1200 -0.0606 0.6608
0.4904 0.3038 0.3014 0.1374 -0.5520
-0.0933 -0.0938 -40.2421
61.0200 -37.9900 1.2091 0.6471
0.4321 0.2922 0.2893 0.1350 -0.5524
-0.0963 -0.1004 -40.2413
61.0400 -40.3600 -0.3661 -0.0086
0.2895 0.2625 0.2743 0.1321 -0.5526
-0.0993 -0.1069 -40.2405
61.0600 -42.1300 -1.2144 -0.6015
0.1264 0.2172 0.2565 0.1286 -0.5527
-0.1023 -0.1134 -40.2397
61.0800 -40.2800 0.6874 -0.4903
0.0016 0.1596 0.2360 0.1248 -0.5525
-0.1053 -0.1200 -40.2389
61.1000 -40.2900 0.4055 -0.0350 -
0.0804 0.0948 0.2130 0.1205 -0.5522
-0.1082 -0.1265 -40.2381
61.1200 -40.1000 0.6847 0.1103 -
0.1366 0.0295 0.1877 0.1159 -0.5517
-0.1111 -0.1330 -40.2373
61.1400 -41.8600 -1.0463 0.1871 -
0.1998 -0.0292 0.1603 0.1109 -0.5510
-0.1140 -0.1396 -40.2365
61.1600 -40.8800 -0.0014 0.3286 -
0.2896 -0.0752 0.1310 0.1056 -0.5500
-0.1168 -0.1461 -40.2357
61.1800 -40.0500 1.0788 0.2298 -
0.3700 -0.1056 0.1000 0.1001 -0.5489
-0.1197 -0.1526 -40.2349
61.2000 -42.6700 -1.0264 -0.2506 -
0.3910 -0.1192 0.0677 0.0943 -0.5476
-0.1225 -0.1591 -40.2341
61.2200 -41.0100 0.8588 -0.5557 -
0.3159 -0.1156 0.0344 0.0882 -0.5462
-0.1253 -0.1656 -40.2332

61.2400	-42.1300	-0.5456	-0.4602	-
0.1364	-0.0961	0.0004	0.0820	-0.5445
	-0.1280	-0.1720	-40.2324	
61.2600	-41.0200	0.1743	-0.1247	
0.1052	-0.0645	-0.0337	0.0755	-0.5427
	-0.1307	-0.1785	-40.2316	
61.2800	-40.6700	-0.2974	0.4336	
0.3248	-0.0252	-0.0676	0.0689	-0.5407
	-0.1334	-0.1850	-40.2308	
61.3000	-39.4600	0.4753	0.7925	
0.4433	0.0168	-0.1006	0.0622	-0.5385
	-0.1361	-0.1914	-40.2300	
61.3200	-40.2900	-0.0847	0.5528	
0.4134	0.0566	-0.1324	0.0554	-0.5361
	-0.1387	-0.1979	-40.2292	
61.3400	-41.1300	-0.1852	-0.0639	
0.2651	0.0902	-0.1624	0.0485	-0.5335
	-0.1413	-0.2043	-40.2284	
61.3600	-41.4300	0.2958	-0.6107	
0.0653	0.1152	-0.1902	0.0416	-0.5308
	-0.1439	-0.2108	-40.2276	
61.3800	-42.4100	-0.3866	-0.7332	-
0.1174	0.1301	-0.2153	0.0347	-0.5279
	-0.1464	-0.2172	-40.2268	
61.4000	-42.1100	-0.2116	-0.3968	-
0.2357	0.1338	-0.2375	0.0277	-0.5249
	-0.1490	-0.2236	-40.2260	
61.4200	-41.0300	0.3844	0.0865	-
0.2751	0.1258	-0.2563	0.0208	-0.5216
	-0.1514	-0.2300	-40.2251	
61.4400	-41.1700	0.1094	0.3256	-
0.2363	0.1065	-0.2716	0.0140	-0.5183
	-0.1539	-0.2364	-40.2243	
61.4600	-41.1700	0.1001	0.2283	-
0.1380	0.0773	-0.2832	0.0073	-0.5147
	-0.1563	-0.2428	-40.2235	
61.4800	-41.4700	0.0379	0.0393	-
0.0229	0.0402	-0.2911	0.0007	-0.5110
	-0.1588	-0.2492	-40.2227	
61.5000	-42.0000	-0.6374	0.0707	
0.0682	-0.0031	-0.2950	-0.0059	-0.5071
	-0.1611	-0.2556	-40.2219	
61.5200	-40.4700	0.6432	0.2285	
0.1184	-0.0506	-0.2950	-0.0123	-0.5031
	-0.1635	-0.2619	-40.2211	
61.5400	-41.3800	-0.1349	0.1598	
0.1237	-0.1006	-0.2910	-0.0186	-0.4989
	-0.1658	-0.2683	-40.2202	
61.5600	-41.8200	-0.1678	-0.0759	
0.0856	-0.1507	-0.2829	-0.0249	-0.4945
	-0.1681	-0.2746	-40.2194	

61.5800 -42.0500 -0.4218 -0.1175
0.0183 -0.1984 -0.2707 -0.0310 -0.4900
-0.1704 -0.2809 -40.2186
61.6000 -41.4000 0.2355 0.0727 -
0.0577 -0.2414 -0.2545 -0.0370 -0.4853
-0.1726 -0.2873 -40.2178
61.6200 -41.6700 0.0316 0.1483 -
0.1218 -0.2775 -0.2343 -0.0430 -0.4804
-0.1748 -0.2936 -40.2169
61.6400 -41.3800 0.5261 -0.0915 -
0.1532 -0.3044 -0.2103 -0.0489 -0.4753
-0.1770 -0.2999 -40.2161
61.6600 -42.5100 -0.3177 -0.3349 -
0.1410 -0.3204 -0.1827 -0.0547 -0.4701
-0.1792 -0.3062 -40.2153
61.6800 -42.6200 -0.6134 -0.2427 -
0.0846 -0.3246 -0.1518 -0.0605 -0.4646
-0.1813 -0.3124 -40.2145
61.7000 -40.9400 0.6448 0.1365 -
0.0050 -0.3167 -0.1179 -0.0664 -0.4590
-0.1834 -0.3187 -40.2136
61.7200 -41.1200 0.1495 0.3255
0.0634 -0.2973 -0.0816 -0.0722 -0.4532
-0.1855 -0.3250 -40.2128
61.7400 -41.1300 0.1381 0.1556
0.0931 -0.2672 -0.0434 -0.0782 -0.4472
-0.1875 -0.3312 -40.2120
61.7600 -41.9800 -0.6205 -0.0123
0.0781 -0.2269 -0.0038 -0.0841 -0.4410
-0.1896 -0.3375 -40.2112
61.7800 -41.1100 0.2615 -0.0123
0.0317 -0.1775 0.0365 -0.0902 -0.4346
-0.1916 -0.3437 -40.2103
61.8000 -40.9400 0.4304 0.0149 -
0.0219 -0.1205 0.0769 -0.0964 -0.4280
-0.1935 -0.3499 -40.2095
61.8200 -41.9400 -0.5522 -0.0304 -
0.0633 -0.0573 0.1168 -0.1027 -0.4213
-0.1955 -0.3561 -40.2087
61.8400 -40.8800 0.3856 -0.1216 -
0.0834 0.0103 0.1556 -0.1092 -0.4143
-0.1974 -0.3623 -40.2078
61.8600 -41.1800 0.1631 -0.2071 -
0.0785 0.0795 0.1925 -0.1158 -0.4071
-0.1993 -0.3685 -40.2070
61.8800 -41.4600 -0.2819 -0.2124 -
0.0544 0.1473 0.2270 -0.1227 -0.3997
-0.2011 -0.3746 -40.2062
61.9000 -41.1700 -0.3034 0.0049 -
0.0231 0.2107 0.2585 -0.1298 -0.3921
-0.2030 -0.3808 -40.2053

61.9200 -40.1100 0.3655 0.3605
0.0060 0.2665 0.2863 -0.1371 -0.3843
-0.2048 -0.3869 -40.2045
61.9400 -40.2900 -0.0126 0.4623
0.0321 0.3108 0.3100 -0.1447 -0.3763
-0.2066 -0.3930 -40.2036
61.9600 -40.0000 0.4675 0.0750
0.0628 0.3401 0.3290 -0.1525 -0.3681
-0.2083 -0.3992 -40.2028
61.9800 -41.6800 -0.7293 -0.4117
0.1100 0.3516 0.3430 -0.1607 -0.3597
-0.2101 -0.4053 -40.2020
62.0000 -40.2600 0.8145 -0.5421
0.1801 0.3438 0.3516 -0.1691 -0.3511
-0.2118 -0.4114 -40.2011
62.0200 -41.6000 -0.8986 -0.2390
0.2592 0.3169 0.3550 -0.1779 -0.3423
-0.2135 -0.4174 -40.2003
62.0400 -39.9000 0.1660 0.3007
0.3123 0.2727 0.3532 -0.1870 -0.3332
-0.2151 -0.4235 -40.1994
62.0600 -39.2900 0.5677 0.7036
0.2990 0.2141 0.3466 -0.1963 -0.3240
-0.2168 -0.4295 -40.1986
62.0800 -39.4400 0.6817 0.5893
0.1954 0.1454 0.3354 -0.2060 -0.3146
-0.2184 -0.4356 -40.1978
62.1000 -41.7100 -0.8266 0.1161
0.0233 0.0724 0.3201 -0.2158 -0.3050
-0.2200 -0.4416 -40.1969
62.1200 -41.9100 -0.4423 -0.1819 -
0.1739 0.0011 0.3011 -0.2259 -0.2952
-0.2215 -0.4476 -40.1961
62.1400 -41.9000 -0.2920 -0.1571 -
0.3404 -0.0624 0.2787 -0.2362 -0.2852
-0.2231 -0.4536 -40.1952
62.1600 -40.9400 0.9888 -0.3031 -
0.4145 -0.1126 0.2534 -0.2466 -0.2750
-0.2246 -0.4596 -40.1944
62.1800 -42.3800 0.0346 -0.7076 -
0.3713 -0.1453 0.2256 -0.2572 -0.2647
-0.2261 -0.4656 -40.1935
62.2000 -43.2700 -0.9073 -0.8031 -
0.2380 -0.1606 0.1957 -0.2678 -0.2542
-0.2276 -0.4715 -40.1927
62.2200 -41.0200 0.6180 -0.1215 -
0.0630 -0.1607 0.1641 -0.2784 -0.2436
-0.2290 -0.4775 -40.1918
62.2400 -40.9300 -0.4233 0.8158
0.0973 -0.1482 0.1314 -0.2890 -0.2328
-0.2304 -0.4834 -40.1910

62.2600 -39.2000 0.9059 1.0365
0.1923 -0.1261 0.0979 -0.2996 -0.2219
-0.2318 -0.4893 -40.1901
62.2800 -41.5900 -0.8283 0.5259
0.2019 -0.0977 0.0641 -0.3101 -0.2109
-0.2332 -0.4952 -40.1893
62.3000 -42.1300 -0.6633 -0.1840
0.1495 -0.0672 0.0305 -0.3205 -0.1997
-0.2345 -0.5011 -40.1884
62.3200 -40.9100 1.2539 -0.7529
0.0732 -0.0386 -0.0025 -0.3307 -0.1884
-0.2359 -0.5069 -40.1876
62.3400 -42.7700 -0.3902 -0.9487
0.0130 -0.0160 -0.0345 -0.3407 -0.1769
-0.2372 -0.5128 -40.1867
62.3600 -43.4900 -1.2388 -0.6810 -
0.0056 -0.0026 -0.0651 -0.3504 -0.1654
-0.2384 -0.5186 -40.1858
62.3800 -41.9100 -0.3841 -0.0068
0.0160 -0.0005 -0.0939 -0.3599 -0.1538
-0.2397 -0.5244 -40.1850
62.4000 -39.6400 1.2684 0.6096
0.0621 -0.0092 -0.1207 -0.3690 -0.1420
-0.2409 -0.5302 -40.1841
62.4200 -41.0200 -0.1502 0.7013
0.1136 -0.0272 -0.1454 -0.3778 -0.1302
-0.2422 -0.5360 -40.1833
62.4400 -42.4200 -1.0953 0.2608
0.1488 -0.0517 -0.1679 -0.3861 -0.1183
-0.2434 -0.5418 -40.1824
62.4600 -40.8800 1.1249 -0.3929
0.1497 -0.0788 -0.1884 -0.3939 -0.1063
-0.2445 -0.5476 -40.1815
62.4800 -43.2100 -1.0969 -0.4260
0.1071 -0.1043 -0.2068 -0.4012 -0.0942
-0.2457 -0.5533 -40.1807
62.5000 -40.6200 0.9888 0.1151
0.0280 -0.1247 -0.2234 -0.4078 -0.0821
-0.2468 -0.5590 -40.1798
62.5200 -42.0500 -0.4498 0.3151 -
0.0664 -0.1367 -0.2382 -0.4138 -0.0699
-0.2479 -0.5647 -40.1790
62.5400 -41.9900 -0.1832 0.1559 -
0.1608 -0.1365 -0.2514 -0.4191 -0.0576
-0.2490 -0.5704 -40.1781
62.5600 -42.2300 -0.1569 0.0488 -
0.2417 -0.1216 -0.2632 -0.4236 -0.0452
-0.2501 -0.5761 -40.1772
62.5800 -41.7800 0.3529 -0.0447 -
0.2835 -0.0920 -0.2738 -0.4272 -0.0329
-0.2512 -0.5818 -40.1764

62.6000 -42.3900 -0.1553 -0.2379 -
0.2501 -0.0498 -0.2834 -0.4299 -0.0204
-0.2522 -0.5874 -40.1755
62.6200 -42.2600 0.0171 -0.3442 -
0.1273 0.0011 -0.2922 -0.4317 -0.0080
-0.2533 -0.5930 -40.1746
62.6400 -42.0300 -0.2787 -0.1195
0.0519 0.0556 -0.3005 -0.4324 0.0046
-0.2543 -0.5987 -40.1738
62.6600 -40.6300 0.4437 0.3117
0.2220 0.1082 -0.3084 -0.4321 0.0171
-0.2553 -0.6042 -40.1729
62.6800 -41.2100 -0.2762 0.4440
0.3131 0.1536 -0.3162 -0.4306 0.0297
-0.2562 -0.6098 -40.1720
62.7000 -40.6000 0.6891 0.0353
0.2873 0.1874 -0.3239 -0.4279 0.0422
-0.2572 -0.6154 -40.1712
62.7200 -42.4600 -0.7854 -0.3492
0.1750 0.2069 -0.3318 -0.4241 0.0548
-0.2581 -0.6209 -40.1703
62.7400 -41.1000 0.5811 -0.1849
0.0237 0.2113 -0.3400 -0.4190 0.0674
-0.2591 -0.6264 -40.1694
62.7600 -41.3800 0.1827 0.1253 -
0.1199 0.2007 -0.3485 -0.4127 0.0800
-0.2600 -0.6319 -40.1685
62.7800 -41.5700 0.1406 0.0807 -
0.2096 0.1758 -0.3574 -0.4050 0.0926
-0.2609 -0.6374 -40.1677
62.8000 -42.4100 -0.4360 -0.1812 -
0.2214 0.1379 -0.3666 -0.3960 0.1052
-0.2617 -0.6429 -40.1668
62.8200 -41.7700 0.2919 -0.3194 -
0.1552 0.0889 -0.3761 -0.3858 0.1178
-0.2626 -0.6484 -40.1659
62.8400 -41.9400 -0.0373 -0.2045 -
0.0317 0.0318 -0.3855 -0.3742 0.1304
-0.2635 -0.6538 -40.1650
62.8600 -41.8700 -0.4102 0.0792
0.1141 -0.0294 -0.3949 -0.3613 0.1429
-0.2643 -0.6592 -40.1642
62.8800 -40.7100 0.6118 0.3000
0.2356 -0.0898 -0.4039 -0.3471 0.1554
-0.2651 -0.6646 -40.1633
62.9000 -41.6900 -0.5033 0.3345
0.2840 -0.1450 -0.4125 -0.3316 0.1678
-0.2659 -0.6700 -40.1624
62.9200 -41.3000 -0.1246 0.4113
0.2279 -0.1914 -0.4205 -0.3149 0.1802
-0.2667 -0.6753 -40.1615

62.9400	-41.4200	-0.1029	0.5157	
0.0718	-0.2259	-0.4276	-0.2969	0.1925
	-0.2675	-0.6807	-40.1606	
62.9600	-41.0300	0.6988	0.1817	-
0.1286	-0.2460	-0.4338	-0.2779	0.2048
	-0.2683	-0.6860	-40.1598	
62.9800	-43.3400	-0.5784	-0.5285	-
0.3046	-0.2489	-0.4389	-0.2576	0.2170
	-0.2690	-0.6913	-40.1589	
63.0000	-43.1100	-0.0086	-0.9119	-
0.3931	-0.2327	-0.4428	-0.2364	0.2291
	-0.2698	-0.6966	-40.1580	
63.0200	-42.8200	-0.0846	-0.6842	-
0.3558	-0.1969	-0.4457	-0.2141	0.2411
	-0.2705	-0.7018	-40.1571	
63.0400	-41.8500	0.1830	-0.0991	-
0.2215	-0.1442	-0.4473	-0.1909	0.2530
	-0.2712	-0.7071	-40.1562	
63.0600	-41.1500	-0.0321	0.4815	-
0.0432	-0.0788	-0.4476	-0.1668	0.2648
	-0.2720	-0.7123	-40.1554	
63.0800	-40.2200	0.3584	0.7911	
0.1268	-0.0050	-0.4467	-0.1420	0.2765
	-0.2727	-0.7175	-40.1545	
63.1000	-40.7300	-0.4805	0.7968	
0.2461	0.0727	-0.4443	-0.1164	0.2881
	-0.2734	-0.7227	-40.1536	
63.1200	-40.1600	0.2547	0.5488	
0.2928	0.1503	-0.4404	-0.0902	0.2995
	-0.2740	-0.7279	-40.1527	
63.1400	-40.1300	0.6244	0.0881	
0.2751	0.2243	-0.4348	-0.0634	0.3108
	-0.2747	-0.7330	-40.1518	
63.1600	-42.0800	-0.8385	-0.4169	
0.2140	0.2918	-0.4276	-0.0361	0.3220
	-0.2754	-0.7381	-40.1509	
63.1800	-41.3200	0.2217	-0.6738	
0.1284	0.3504	-0.4186	-0.0084	0.3330
	-0.2760	-0.7432	-40.1500	
63.2000	-40.5600	0.7426	-0.5191	
0.0340	0.3979	-0.4077	0.0196	0.3438
	-0.2767	-0.7483	-40.1491	
63.2200	-41.5900	-0.8514	0.0020	-
0.0486	0.4329	-0.3948	0.0479	0.3545
	-0.2773	-0.7534	-40.1482	
63.2400	-39.5800	0.7312	0.4874	-
0.1031	0.4547	-0.3799	0.0763	0.3650
	-0.2780	-0.7584	-40.1474	
63.2600	-40.3000	-0.0086	0.4379	-
0.1215	0.4631	-0.3626	0.1049	0.3753
	-0.2786	-0.7635	-40.1465	

63.2800 -41.2300 -0.4525 -0.0189 -
0.1090 0.4581 -0.3430 0.1334 0.3854
-0.2792 -0.7685 -40.1456
63.3000 -40.8300 0.1454 -0.3652 -
0.0781 0.4402 -0.3208 0.1619 0.3953
-0.2798 -0.7734 -40.1447
63.3200 -41.0800 -0.3195 -0.2210 -
0.0401 0.4096 -0.2959 0.1902 0.4050
-0.2804 -0.7784 -40.1438
63.3400 -40.3200 -0.0182 0.1857 -
0.0003 0.3663 -0.2681 0.2183 0.4145
-0.2810 -0.7833 -40.1429
63.3600 -39.3300 0.7156 0.3346
0.0386 0.3102 -0.2374 0.2461 0.4238
-0.2816 -0.7882 -40.1420
63.3800 -40.7600 -0.3830 0.0106
0.0743 0.2412 -0.2036 0.2735 0.4328
-0.2822 -0.7931 -40.1411
63.4000 -40.9900 -0.2647 -0.2979
0.1139 0.1596 -0.1667 0.3004 0.4416
-0.2828 -0.7980 -40.1402
63.4200 -40.8200 -0.1981 -0.1926
0.1622 0.0662 -0.1267 0.3267 0.4502
-0.2834 -0.8029 -40.1393
63.4400 -40.3000 0.0062 0.1247
0.2065 -0.0373 -0.0838 0.3525 0.4585
-0.2840 -0.8077 -40.1384
63.4600 -39.3800 0.7246 0.2070
0.2261 -0.1484 -0.0383 0.3775 0.4665
-0.2846 -0.8125 -40.1375
63.4800 -41.1300 -0.7384 0.0343
0.2013 -0.2641 0.0097 0.4018 0.4743
-0.2851 -0.8173 -40.1366
63.5000 -40.7900 -0.1535 -0.0106
0.1221 -0.3808 0.0598 0.4253 0.4818
-0.2857 -0.8220 -40.1357
63.5200 -40.4100 0.1992 0.1249 -
0.0024 -0.4943 0.1116 0.4479 0.4890
-0.2863 -0.8268 -40.1348
63.5400 -40.1400 0.6084 0.1210 -
0.1424 -0.6000 0.1649 0.4696 0.4959
-0.2869 -0.8315 -40.1339
63.5600 -41.4000 -0.2324 -0.1014 -
0.2623 -0.6926 0.2193 0.4902 0.5025
-0.2874 -0.8362 -40.1330
63.5800 -41.7100 -0.3561 -0.2474 -
0.3316 -0.7669 0.2746 0.5098 0.5089
-0.2880 -0.8409 -40.1321
63.6000 -40.9400 0.1604 -0.1618 -
0.3298 -0.8179 0.3303 0.5283 0.5149
-0.2886 -0.8455 -40.1312

63.6200 -41.1500 -0.1433 0.0418 -
0.2672 -0.8417 0.3862 0.5456 0.5205
-0.2891 -0.8501 -40.1303
63.6400 -40.6500 -0.0687 0.1219 -
0.1744 -0.8355 0.4420 0.5617 0.5259
-0.2897 -0.8547 -40.1294
63.6600 -39.9000 0.6533 -0.0051 -
0.0942 -0.7970 0.4971 0.5765 0.5309
-0.2903 -0.8593 -40.1285
63.6800 -41.2100 -0.7908 -0.0863 -
0.0705 -0.7252 0.5510 0.5900 0.5355
-0.2908 -0.8639 -40.1275
63.7000 -39.5600 0.5384 0.1821 -
0.1241 -0.6208 0.6029 0.6021 0.5398
-0.2914 -0.8684 -40.1266
63.7200 -39.4500 0.3154 0.4475 -
0.2338 -0.4872 0.6523 0.6127 0.5438
-0.2920 -0.8729 -40.1257
63.7400 -39.9600 -0.0891 0.3086 -
0.3594 -0.3300 0.6983 0.6218 0.5474
-0.2926 -0.8774 -40.1248
63.7600 -40.0700 0.1998 -0.1940 -
0.4524 -0.1557 0.7404 0.6294 0.5506
-0.2931 -0.8819 -40.1239
63.7800 -40.6600 -0.3102 -0.6112 -
0.4664 0.0289 0.7777 0.6353 0.5534
-0.2937 -0.8863 -40.1230
63.8000 -40.4000 -0.3299 -0.5820 -
0.3690 0.2171 0.8096 0.6396 0.5559
-0.2943 -0.8907 -40.1221
63.8200 -39.0100 0.1688 -0.1191 -
0.1724 0.4018 0.8354 0.6422 0.5580
-0.2949 -0.8951 -40.1212
63.8400 -38.2400 -0.0567 0.3934
0.0773 0.5756 0.8543 0.6431 0.5597
-0.2955 -0.8995 -40.1203
63.8600 -37.3200 0.3352 0.6139
0.3256 0.7316 0.8655 0.6421 0.5610
-0.2961 -0.9038 -40.1193
63.8800 -37.5900 -0.1417 0.5220
0.5258 0.8626 0.8685 0.6394 0.5620
-0.2967 -0.9081 -40.1184
63.9000 -37.6400 -0.2550 0.2978
0.6569 0.9616 0.8623 0.6349 0.5626
-0.2973 -0.9124 -40.1175
63.9200 -37.2300 0.2518 0.0435
0.7219 1.0225 0.8463 0.6286 0.5628
-0.2979 -0.9167 -40.1166
63.9400 -37.7500 0.0594 -0.2535
0.7416 1.0412 0.8199 0.6206 0.5627
-0.2986 -0.9209 -40.1157

63.9600 −38.0400 −0.0091 −0.5297
0.7369 1.0158 0.7831 0.6109 0.5622
−0.2992 −0.9251 −40.1148
63.9800 −38.3400 −0.0347 −0.5589
0.7110 0.9474 0.7364 0.5997 0.5614
−0.2998 −0.9293 −40.1138
64.0000 −38.7200 −0.4890 −0.1871
0.6514 0.8407 0.6810 0.5870 0.5602
−0.3005 −0.9335 −40.1129
64.0200 −37.8900 0.1845 0.3498
0.5410 0.7028 0.6177 0.5729 0.5587
−0.3011 −0.9376 −40.1120
64.0400 −37.5800 0.6439 0.5621
0.3657 0.5421 0.5477 0.5575 0.5569
−0.3018 −0.9417 −40.1111
64.0600 −39.5600 −0.5479 0.2471
0.1260 0.3669 0.4718 0.5408 0.5548
−0.3025 −0.9458 −40.1102
64.0800 −39.7000 0.3462 −0.1903 −
0.1478 0.1849 0.3912 0.5230 0.5524
−0.3031 −0.9499 −40.1092
64.1000 −41.0400 −0.3561 −0.3589 −
0.4184 0.0036 0.3067 0.5041 0.5497
−0.3038 −0.9539 −40.1083
64.1200 −41.2400 −0.0191 −0.2185 −
0.6471 −0.1698 0.2195 0.4842 0.5467
−0.3045 −0.9580 −40.1074
64.1400 −41.2600 0.0144 0.0448 −
0.8005 −0.3283 0.1305 0.4634 0.5434
−0.3052 −0.9619 −40.1065
64.1600 −41.1300 0.3934 0.1401 −
0.8592 −0.4653 0.0408 0.4418 0.5398
−0.3059 −0.9659 −40.1055
64.1800 −41.7500 0.0326 0.0220 −
0.8199 −0.5762 −0.0488 0.4194 0.5360
−0.3066 −0.9698 −40.1046
64.2000 −42.7100 −0.7339 −0.0620 −
0.6938 −0.6593 −0.1371 0.3963 0.5319
−0.3073 −0.9738 −40.1037
64.2200 −41.5800 0.3352 0.0355 −
0.5004 −0.7146 −0.2234 0.3727 0.5276
−0.3080 −0.9776 −40.1028
64.2400 −41.0500 0.5952 0.0972 −
0.2697 −0.7438 −0.3066 0.3485 0.5231
−0.3088 −0.9815 −40.1018
64.2600 −41.2800 0.4062 −0.0513 −
0.0314 −0.7494 −0.3860 0.3240 0.5183
−0.3095 −0.9853 −40.1009
64.2800 −42.6700 −0.9025 −0.1397
0.1941 −0.7351 −0.4607 0.2990 0.5133
−0.3102 −0.9891 −40.1000

64.3000 -41.1200 0.2973 0.0299
0.3886 -0.7044 -0.5300 0.2739 0.5080
-0.3110 -0.9929 -40.0990
64.3200 -40.5800 0.6167 0.1983
0.5304 -0.6607 -0.5930 0.2485 0.5026
-0.3117 -0.9967 -40.0981
64.3400 -41.6100 -0.3094 0.1518
0.5990 -0.6067 -0.6492 0.2231 0.4970
-0.3125 -1.0004 -40.0972
64.3600 -41.4500 -0.1142 0.0345
0.5812 -0.5446 -0.6978 0.1976 0.4911
-0.3132 -1.0041 -40.0962
64.3800 -41.6500 -0.1571 0.0647
0.4763 -0.4767 -0.7384 0.1722 0.4851
-0.3140 -1.0078 -40.0953
64.4000 -41.3600 0.1732 0.1520
0.3005 -0.4048 -0.7709 0.1469 0.4790
-0.3148 -1.0114 -40.0944
64.4200 -41.1400 0.5614 0.1235
0.0865 -0.3307 -0.7950 0.1219 0.4726
-0.3155 -1.0151 -40.0934
64.4400 -42.9400 -0.8797 -0.0185 -
0.1255 -0.2561 -0.8108 0.0971 0.4661
-0.3163 -1.0187 -40.0925
64.4600 -41.7800 0.5508 -0.2018 -
0.2947 -0.1828 -0.8183 0.0727 0.4595
-0.3171 -1.0222 -40.0916
64.4800 -41.8300 0.7504 -0.3755 -
0.3857 -0.1126 -0.8176 0.0488 0.4527
-0.3179 -1.0258 -40.0906
64.5000 -43.4600 -0.9048 -0.3640 -
0.3824 -0.0474 -0.8089 0.0252 0.4457
-0.3187 -1.0293 -40.0897
64.5200 -41.5800 0.4622 -0.0275 -
0.2952 0.0109 -0.7921 0.0022 0.4387
-0.3195 -1.0328 -40.0888
64.5400 -41.2300 0.2802 0.3283 -
0.1652 0.0607 -0.7676 -0.0203 0.4315
-0.3203 -1.0363 -40.0878
64.5600 -41.2500 0.0950 0.3947 -
0.0389 0.1008 -0.7354 -0.0423 0.4242
-0.3211 -1.0397 -40.0869
64.5800 -41.7700 -0.3429 0.2723
0.0521 0.1303 -0.6960 -0.0637 0.4168
-0.3219 -1.0431 -40.0859
64.6000 -41.3600 0.1230 0.0804
0.1022 0.1479 -0.6495 -0.0846 0.4094
-0.3227 -1.0465 -40.0850
64.6200 -41.4900 0.1268 -0.1816
0.1290 0.1527 -0.5964 -0.1049 0.4018
-0.3236 -1.0498 -40.0841

64.6400 -41.9900 -0.1377 -0.4182
0.1496 0.1441 -0.5372 -0.1247 0.3941
-0.3244 -1.0532 -40.0831
64.6600 -41.8100 -0.0192 -0.3775
0.1650 0.1229 -0.4727 -0.1439 0.3864
-0.3252 -1.0565 -40.0822
64.6800 -41.9600 -0.5976 0.0699
0.1681 0.0912 -0.4035 -0.1625 0.3787
-0.3260 -1.0597 -40.0812
64.7000 -40.4000 0.6215 0.4961
0.1400 0.0514 -0.3307 -0.1805 0.3709
-0.3269 -1.0630 -40.0803
64.7200 -40.3300 0.6562 0.4384
0.0652 0.0068 -0.2552 -0.1980 0.3630
-0.3277 -1.0662 -40.0794
64.7400 -42.3600 -0.7139 -0.0199 -
0.0421 -0.0387 -0.1779 -0.2149 0.3551
-0.3285 -1.0694 -40.0784
64.7600 -42.4600 -0.4017 -0.3316 -
0.1462 -0.0809 -0.0997 -0.2313 0.3472
-0.3294 -1.0726 -40.0775
64.7800 -41.6500 0.3199 -0.2513 -
0.2085 -0.1161 -0.0217 -0.2471 0.3392
-0.3302 -1.0757 -40.0765
64.8000 -41.8900 -0.1648 -0.0195 -
0.2053 -0.1406 0.0551 -0.2623 0.3313
-0.3311 -1.0788 -40.0756
64.8200 -41.0700 0.5081 -0.0211 -
0.1349 -0.1516 0.1300 -0.2769 0.3233
-0.3319 -1.0819 -40.0746
64.8400 -41.7000 -0.1725 -0.1167 -
0.0236 -0.1481 0.2021 -0.2910 0.3154
-0.3328 -1.0849 -40.0737
64.8600 -41.9100 -0.5995 -0.0305
0.0861 -0.1295 0.2705 -0.3044 0.3074
-0.3337 -1.0880 -40.0727
64.8800 -40.7700 0.1010 0.2379
0.1569 -0.0960 0.3348 -0.3172 0.2995
-0.3345 -1.0910 -40.0718
64.9000 -39.8700 0.8311 0.3305
0.1626 -0.0489 0.3944 -0.3294 0.2917
-0.3354 -1.0939 -40.0708
64.9200 -41.5400 -0.5511 0.0496
0.0951 0.0101 0.4486 -0.3410 0.2838
-0.3362 -1.0969 -40.0699
64.9400 -41.4000 -0.2279 -0.1906 -
0.0211 0.0775 0.4970 -0.3520 0.2760
-0.3371 -1.0998 -40.0689
64.9600 -41.6400 -0.6981 0.0330 -
0.1485 0.1490 0.5393 -0.3624 0.2683
-0.3380 -1.1027 -40.0680

64.9800 -39.8500 0.9001 0.3120 -
0.2429 0.2203 0.5748 -0.3721 0.2606
-0.3389 -1.1056 -40.0670
65.0000 -41.0400 -0.0953 0.1487 -
0.2643 0.2872 0.6032 -0.3811 0.2530
-0.3397 -1.1084 -40.0661
65.0200 -41.9000 -0.7543 -0.2954 -
0.2016 0.3458 0.6241 -0.3896 0.2455
-0.3406 -1.1112 -40.0651
65.0400 -40.5900 0.6686 -0.6059 -
0.0687 0.3928 0.6371 -0.3974 0.2381
-0.3415 -1.1140 -40.0642
65.0600 -41.5100 -0.5302 -0.4688
0.1070 0.4255 0.6419 -0.4045 0.2307
-0.3423 -1.1167 -40.0632
65.0800 -40.2200 0.1010 0.0216
0.2842 0.4413 0.6386 -0.4111 0.2235
-0.3432 -1.1194 -40.0623
65.1000 -39.5700 0.1678 0.5201
0.4162 0.4376 0.6271 -0.4171 0.2164
-0.3441 -1.1221 -40.0613
65.1200 -39.5100 0.0751 0.7101
0.4581 0.4127 0.6078 -0.4225 0.2093
-0.3450 -1.1248 -40.0603
65.1400 -39.7300 0.1825 0.5125
0.3886 0.3670 0.5815 -0.4273 0.2024
-0.3458 -1.1275 -40.0594
65.1600 -41.1700 -0.5719 0.1194
0.2319 0.3042 0.5488 -0.4315 0.1957
-0.3467 -1.1301 -40.0584
65.1800 -40.7700 0.4321 -0.2503
0.0342 0.2292 0.5105 -0.4352 0.1890
-0.3476 -1.1326 -40.0575
65.2000 -41.5300 0.3386 -0.5163 -
0.1573 0.1470 0.4675 -0.4383 0.1825
-0.3485 -1.1352 -40.0565
65.2200 -42.9500 -0.6660 -0.6085 -
0.3015 0.0626 0.4204 -0.4408 0.1762
-0.3494 -1.1377 -40.0556
65.2400 -42.2700 0.0104 -0.4367 -
0.3722 -0.0192 0.3701 -0.4428 0.1699
-0.3502 -1.1402 -40.0546
65.2600 -41.4100 0.6365 -0.0692 -
0.3734 -0.0943 0.3174 -0.4442 0.1639
-0.3511 -1.1427 -40.0536
65.2800 -42.2900 -0.5277 0.3223 -
0.3220 -0.1594 0.2631 -0.4451 0.1579
-0.3520 -1.1452 -40.0527
65.3000 -41.5800 -0.0431 0.5337 -
0.2305 -0.2123 0.2079 -0.4455 0.1522
-0.3528 -1.1476 -40.0517

65.3200 -41.0800 0.6020 0.3903 –
0.1093 -0.2524 0.1526 -0.4453 0.1466
-0.3537 -1.1500 -40.0508
65.3400 -42.4400 -0.3211 -0.0833
0.0235 -0.2801 0.0980 -0.4446 0.1411
-0.3546 -1.1523 -40.0498
65.3600 -42.5700 -0.0611 -0.5154
0.1508 -0.2958 0.0447 -0.4435 0.1358
-0.3554 -1.1547 -40.0488
65.3800 -42.9500 -0.7816 -0.3687
0.2557 -0.3005 -0.0065 -0.4418 0.1307
-0.3563 -1.1570 -40.0479
65.4000 -40.8400 0.9107 0.1602
0.3168 -0.2947 -0.0552 -0.4396 0.1257
-0.3572 -1.1592 -40.0469
65.4200 -42.1000 -0.6376 0.4141
0.3143 -0.2792 -0.1009 -0.4369 0.1209
-0.3580 -1.1615 -40.0459
65.4400 -41.9300 -0.2173 0.3389
0.2408 -0.2549 -0.1433 -0.4337 0.1163
-0.3589 -1.1637 -40.0450
65.4600 -41.4300 0.5855 0.0911
0.1115 -0.2230 -0.1822 -0.4301 0.1118
-0.3597 -1.1659 -40.0440
65.4800 -42.8700 -0.3044 -0.2225 –
0.0395 -0.1849 -0.2175 -0.4260 0.1075
-0.3605 -1.1681 -40.0430
65.5000 -42.7100 0.1475 -0.3992 –
0.1742 -0.1416 -0.2490 -0.4214 0.1034
-0.3614 -1.1702 -40.0421
65.5200 -43.2100 -0.4984 -0.2150 –
0.2627 -0.0943 -0.2766 -0.4164 0.0994
-0.3622 -1.1723 -40.0411
65.5400 -42.5000 -0.1426 0.1288 –
0.2991 -0.0442 -0.3004 -0.4109 0.0956
-0.3630 -1.1744 -40.0401
65.5600 -41.2900 0.9937 0.2782 –
0.2932 0.0076 -0.3203 -0.4050 0.0920
-0.3638 -1.1765 -40.0392
65.5800 -43.1800 -0.8496 0.1217 –
0.2577 0.0596 -0.3364 -0.3986 0.0885
-0.3647 -1.1785 -40.0382
65.6000 -43.0700 -0.8106 -0.0056 –
0.2046 0.1098 -0.3486 -0.3918 0.0851
-0.3655 -1.1805 -40.0372
65.6200 -42.4300 -0.1897 0.0589 –
0.1366 0.1553 -0.3571 -0.3846 0.0820
-0.3662 -1.1825 -40.0362
65.6400 -41.0300 0.9565 0.0850 –
0.0404 0.1933 -0.3618 -0.3770 0.0789
-0.3670 -1.1844 -40.0353

65.6600 -42.0800 0.0078 -0.1514
0.0888 0.2206 -0.3628 -0.3689 0.0761
-0.3678 -1.1864 -40.0343
65.6800 -42.8800 -0.7251 -0.4089
0.2286 0.2350 -0.3604 -0.3603 0.0733
-0.3686 -1.1882 -40.0333
65.7000 -41.2600 0.6736 -0.2807
0.3478 0.2347 -0.3547 -0.3514 0.0708
-0.3693 -1.1901 -40.0324
65.7200 -42.0800 -0.8201 0.3083
0.4110 0.2189 -0.3459 -0.3420 0.0683
-0.3700 -1.1920 -40.0314
65.7400 -40.4500 0.5217 0.6065
0.3836 0.1879 -0.3342 -0.3322 0.0660
-0.3708 -1.1938 -40.0304
65.7600 -40.4700 1.0420 0.3235
0.2535 0.1441 -0.3199 -0.3220 0.0638
-0.3715 -1.1956 -40.0294
65.7800 -43.4100 -1.1928 -0.2740
0.0595 0.0917 -0.3030 -0.3114 0.0617
-0.3722 -1.1973 -40.0285
65.8000 -41.8200 1.0182 -0.5080 -
0.1415 0.0352 -0.2839 -0.3005 0.0598
-0.3729 -1.1990 -40.0275
65.8200 -42.6300 0.2752 -0.4465 -
0.2891 -0.0211 -0.2628 -0.2892 0.0579
-0.3735 -1.2007 -40.0265
65.8400 -43.3600 -0.5083 -0.3762 -
0.3413 -0.0726 -0.2400 -0.2775 0.0562
-0.3742 -1.2024 -40.0255
65.8600 -42.7400 -0.1253 -0.2972 -
0.2984 -0.1163 -0.2158 -0.2655 0.0546
-0.3748 -1.2041 -40.0246
65.8800 -42.1700 0.1444 -0.0654 -
0.1914 -0.1502 -0.1904 -0.2533 0.0530
-0.3754 -1.2057 -40.0236
65.9000 -42.0900 -0.1602 0.2711 -
0.0614 -0.1735 -0.1641 -0.2407 0.0516
-0.3760 -1.2073 -40.0226
65.9200 -41.3600 0.2046 0.5285
0.0499 -0.1856 -0.1372 -0.2279 0.0502
-0.3766 -1.2089 -40.0216
65.9400 -41.4600 -0.0677 0.5501
0.1061 -0.1867 -0.1100 -0.2149 0.0489
-0.3772 -1.2104 -40.0206
65.9600 -41.6300 -0.1399 0.3735
0.0920 -0.1770 -0.0828 -0.2017 0.0477
-0.3777 -1.2119 -40.0197
65.9800 -41.5400 0.2925 0.1045
0.0224 -0.1577 -0.0559 -0.1884 0.0466
-0.3782 -1.2134 -40.0187

66.0000 -42.3700 -0.1459 -0.2027 -
0.0644 -0.1302 -0.0294 -0.1749 0.0455
-0.3787 -1.2149 -40.0177
66.0200 -42.8200 -0.3415 -0.4354 -
0.1245 -0.0961 -0.0036 -0.1612 0.0445
-0.3792 -1.2163 -40.0167
66.0400 -41.5800 0.6360 -0.4509 -
0.1277 -0.0575 0.0211 -0.1475 0.0435
-0.3796 -1.2177 -40.0157
66.0600 -42.6100 -0.7232 -0.1323 -
0.0737 -0.0161 0.0446 -0.1336 0.0426
-0.3800 -1.2191 -40.0148
66.0800 -40.9100 0.4232 0.2704
0.0086 0.0258 0.0666 -0.1197 0.0417
-0.3804 -1.2204 -40.0138
66.1000 -40.6200 0.4931 0.3525
0.0817 0.0660 0.0867 -0.1058 0.0409
-0.3808 -1.2218 -40.0128
66.1200 -41.8600 -0.6766 0.0648
0.1171 0.1022 0.1047 -0.0919 0.0401
-0.3811 -1.2231 -40.0118
66.1400 -40.9700 0.5237 -0.1761
0.1078 0.1317 0.1204 -0.0780 0.0393
-0.3814 -1.2243 -40.0108
66.1600 -41.8200 -0.4017 -0.0916
0.0681 0.1524 0.1335 -0.0642 0.0385
-0.3817 -1.2256 -40.0098
66.1800 -41.3500 -0.1673 0.1006
0.0304 0.1621 0.1438 -0.0505 0.0377
-0.3819 -1.2268 -40.0088
66.2000 -40.6900 0.3631 0.1460
0.0235 0.1598 0.1515 -0.0368 0.0370
-0.3821 -1.2280 -40.0079
66.2200 -40.9200 0.4144 -0.0926
0.0539 0.1462 0.1563 -0.0233 0.0362
-0.3823 -1.2292 -40.0069
66.2400 -42.2500 -0.6773 -0.3307
0.1037 0.1237 0.1585 -0.0101 0.0354
-0.3824 -1.2303 -40.0059
66.2600 -40.9900 0.3996 -0.1688
0.1418 0.0959 0.1580 0.0030 0.0347
-0.3825 -1.2314 -40.0049
66.2800 -40.6700 0.3412 0.2100
0.1407 0.0663 0.1551 0.0158 0.0339
-0.3826 -1.2325 -40.0039
66.3000 -41.4100 -0.4274 0.4257
0.0817 0.0380 0.1499 0.0284 0.0331
-0.3827 -1.2336 -40.0029
66.3200 -41.2900 -0.0999 0.2175 -
0.0354 0.0133 0.1427 0.0406 0.0322
-0.3827 -1.2346 -40.0019

66.3400 -41.0800 0.7266 -0.2632 -
0.1707 -0.0069 0.1338 0.0525 0.0313
-0.3826 -1.2356 -40.0009
66.3600 -43.0200 -0.8612 -0.5043 -
0.2687 -0.0219 0.1234 0.0640 0.0304
-0.3826 -1.2366 -39.9999
66.3800 -41.4500 0.5149 -0.1661 -
0.2849 -0.0315 0.1119 0.0750 0.0295
-0.3824 -1.2376 -39.9990
66.4000 -41.0000 0.4979 0.2059 -
0.2090 -0.0362 0.0997 0.0857 0.0285
-0.3823 -1.2385 -39.9980
66.4200 -41.6300 -0.2527 0.1525 -
0.0505 -0.0376 0.0872 0.0958 0.0274
-0.3821 -1.2394 -39.9970
66.4400 -41.5500 -0.1091 -0.1222
0.1411 -0.0375 0.0747 0.1055 0.0263
-0.3819 -1.2403 -39.9960
66.4600 -41.9100 -0.7823 -0.0371
0.3003 -0.0374 0.0625 0.1147 0.0251
-0.3816 -1.2412 -39.9950
66.4800 -39.9200 0.8794 0.2776
0.3813 -0.0390 0.0508 0.1233 0.0238
-0.3813 -1.2420 -39.9940
66.5000 -40.0900 0.5513 0.4270
0.3578 -0.0429 0.0401 0.1313 0.0225
-0.3809 -1.2428 -39.9930
66.5200 -41.9800 -1.1483 0.3545
0.2288 -0.0486 0.0304 0.1388 0.0210
-0.3805 -1.2436 -39.9920
66.5400 -41.7100 -0.3798 0.1283
0.0298 -0.0544 0.0219 0.1457 0.0195
-0.3800 -1.2444 -39.9910
66.5600 -40.7900 1.1012 -0.2217 -
0.1847 -0.0585 0.0148 0.1521 0.0179
-0.3795 -1.2451 -39.9900
66.5800 -43.2200 -0.9232 -0.5524 -
0.3542 -0.0591 0.0089 0.1579 0.0162
-0.3790 -1.2458 -39.9890
66.6000 -41.8600 0.4184 -0.3912 -
0.4313 -0.0546 0.0044 0.1631 0.0143
-0.3784 -1.2465 -39.9880
66.6200 -42.0700 -0.4471 0.2509 -
0.4070 -0.0442 0.0012 0.1677 0.0124
-0.3777 -1.2471 -39.9870
66.6400 -40.6100 0.6066 0.5736 -
0.2976 -0.0285 -0.0007 0.1718 0.0103
-0.3770 -1.2478 -39.9860
66.6600 -41.1700 0.1210 0.2583 -
0.1273 -0.0090 -0.0013 0.1753 0.0082
-0.3763 -1.2484 -39.9850

66.6800 -42.0600 -0.3832 -0.3020
0.0665 0.0120 -0.0008 0.1783 0.0059
-0.3755 -1.2489 -39.9840
66.7000 -42.0800 -0.3537 -0.5785
0.2414 0.0317 0.0009 0.1807 0.0034
-0.3746 -1.2495 -39.9830
66.7200 -40.9300 0.5719 -0.4555
0.3683 0.0474 0.0034 0.1826 0.0008
-0.3737 -1.2500 -39.9820
66.7400 -41.5400 -0.5301 -0.0951
0.4254 0.0580 0.0067 0.1839 -0.0019
-0.3727 -1.2505 -39.9810
66.7600 -40.0200 0.4891 0.4487
0.3838 0.0634 0.0104 0.1848 -0.0048
-0.3717 -1.2510 -39.9800
66.7800 -40.8100 -0.4797 0.7602
0.2304 0.0638 0.0145 0.1851 -0.0078
-0.3707 -1.2515 -39.9790
66.8000 -40.1500 1.1455 0.0927 -
0.0083 0.0605 0.0187 0.1850 -0.0110
-0.3695 -1.2519 -39.9780
66.8200 -43.8000 -1.3727 -0.7837 -
0.2401 0.0556 0.0228 0.1843 -0.0143
-0.3683 -1.2523 -39.9770
66.8400 -42.2700 0.1131 -0.7185 -
0.3709 0.0510 0.0265 0.1832 -0.0178
-0.3671 -1.2527 -39.9760
66.8600 -40.5900 1.1972 -0.1256 -
0.3711 0.0483 0.0298 0.1816 -0.0215
-0.3658 -1.2530 -39.9750
66.8800 -40.7000 0.7297 0.2762 -
0.2594 0.0479 0.0326 0.1796 -0.0254
-0.3644 -1.2534 -39.9740
66.9000 -41.7600 -0.6757 0.3304 -
0.0771 0.0486 0.0347 0.1771 -0.0294
-0.3630 -1.2537 -39.9730
66.9200 -41.4100 -0.3515 0.2130
0.1032 0.0496 0.0361 0.1742 -0.0336
-0.3615 -1.2539 -39.9720
66.9400 -41.1400 -0.2331 0.2369
0.2115 0.0499 0.0368 0.1709 -0.0379
-0.3600 -1.2542 -39.9710
66.9600 -40.7100 0.1141 0.3292
0.2295 0.0486 0.0368 0.1672 -0.0424
-0.3584 -1.2544 -39.9700
66.9800 -40.7700 0.1980 0.2280
0.1746 0.0445 0.0361 0.1632 -0.0471
-0.3567 -1.2546 -39.9690
67.0000 -41.3000 0.1833 -0.1468
0.0820 0.0362 0.0348 0.1588 -0.0520
-0.3550 -1.2548 -39.9680

67.0200 -42.2800 -0.3773 -0.5253 -
0.0029 0.0220 0.0329 0.1540 -0.0571
-0.3532 -1.2550 -39.9670
67.0400 -42.2500 -0.2476 -0.5006 -
0.0414 0.0016 0.0304 0.1490 -0.0623
-0.3514 -1.2551 -39.9660
67.0600 -41.2600 0.3680 -0.1323 -
0.0228 -0.0243 0.0274 0.1436 -0.0677
-0.3495 -1.2552 -39.9650
67.0800 -41.0500 0.2555 0.1839
0.0318 -0.0528 0.0241 0.1380 -0.0733
-0.3475 -1.2553 -39.9640
67.1000 -41.1700 0.1200 0.2530
0.0881 -0.0805 0.0205 0.1322 -0.0790
-0.3455 -1.2554 -39.9630
67.1200 -41.7400 -0.4868 0.2303
0.1062 -0.1036 0.0167 0.1261 -0.0850
-0.3434 -1.2554 -39.9620
67.1400 -41.2500 0.0566 0.2945
0.0580 -0.1187 0.0127 0.1198 -0.0910
-0.3412 -1.2554 -39.9610
67.1600 -40.9000 0.5093 0.3644 -
0.0549 -0.1234 0.0087 0.1133 -0.0973
-0.3390 -1.2554 -39.9599
67.1800 -42.1900 -0.5370 0.2628 -
0.1996 -0.1165 0.0046 0.1067 -0.1037
-0.3367 -1.2554 -39.9589
67.2000 -42.4100 -0.2354 -0.0928 -
0.3272 -0.0976 0.0004 0.0999 -0.1103
-0.3344 -1.2554 -39.9579
67.2200 -41.6500 0.9130 -0.4945 -
0.3916 -0.0666 -0.0039 0.0931 -0.1170
-0.3320 -1.2553 -39.9569
67.2400 -43.4400 -0.9435 -0.4859 -
0.3593 -0.0245 -0.0083 0.0861 -0.1239
-0.3295 -1.2552 -39.9559
67.2600 -41.0800 0.7064 0.0854 -
0.2332 0.0254 -0.0129 0.0791 -0.1309
-0.3270 -1.2551 -39.9549
67.2800 -40.9400 0.2077 0.4818 -
0.0485 0.0786 -0.0175 0.0720 -0.1381
-0.3244 -1.2549 -39.9539
67.3000 -41.4200 -0.3799 0.2987
0.1508 0.1297 -0.0222 0.0650 -0.1455
-0.3217 -1.2547 -39.9529
67.3200 -41.1900 0.1645 -0.2182
0.3176 0.1739 -0.0268 0.0579 -0.1529
-0.3190 -1.2546 -39.9519
67.3400 -41.3300 0.1533 -0.4326
0.4100 0.2064 -0.0315 0.0508 -0.1605
-0.3162 -1.2543 -39.9508

67.3600 -41.9000 -0.7472 -0.0920
0.4096 0.2237 -0.0360 0.0438 -0.1683
-0.3134 -1.2541 -39.9498
67.3800 -40.0400 0.7070 0.3976
0.3274 0.2247 -0.0404 0.0369 -0.1761
-0.3105 -1.2538 -39.9488
67.4000 -40.2200 0.6417 0.4313
0.1867 0.2106 -0.0443 0.0300 -0.1841
-0.3075 -1.2536 -39.9478
67.4200 -41.8200 -0.3545 0.0096
0.0225 0.1836 -0.0479 0.0232 -0.1922
-0.3045 -1.2533 -39.9468
67.4400 -42.7500 -0.8016 -0.3537 -
0.1261 0.1464 -0.0508 0.0165 -0.2005
-0.3014 -1.2529 -39.9458
67.4600 -41.6400 0.4755 -0.2581 -
0.2269 0.1020 -0.0530 0.0099 -0.2088
-0.2982 -1.2526 -39.9448
67.4800 -42.0600 -0.2946 0.1487 -
0.2731 0.0537 -0.0543 0.0033 -0.2172
-0.2950 -1.2522 -39.9437
67.5000 -41.1600 0.5483 0.2795 -
0.2741 0.0052 -0.0548 -0.0031 -0.2257
-0.2917 -1.2518 -39.9427
67.5200 -41.9900 -0.0954 0.0551 -
0.2436 -0.0404 -0.0542 -0.0093 -0.2344
-0.2884 -1.2514 -39.9417
67.5400 -42.4900 -0.2846 -0.1633 -
0.1966 -0.0809 -0.0525 -0.0155 -0.2431
-0.2850 -1.2510 -39.9407
67.5600 -42.2100 0.0690 -0.1434 -
0.1396 -0.1155 -0.0494 -0.0215 -0.2519
-0.2815 -1.2505 -39.9397
67.5800 -41.8400 0.2056 -0.0194 -
0.0700 -0.1441 -0.0448 -0.0274 -0.2607
-0.2780 -1.2500 -39.9387
67.6000 -41.9200 0.0063 -0.0080
0.0138 -0.1678 -0.0385 -0.0331 -0.2697
-0.2744 -1.2495 -39.9376
67.6200 -41.9100 0.0366 -0.0809
0.1043 -0.1878 -0.0305 -0.0387 -0.2786
-0.2708 -1.2490 -39.9366
67.6400 -42.2500 -0.2979 -0.0458
0.1840 -0.2052 -0.0206 -0.0442 -0.2877
-0.2671 -1.2484 -39.9356
67.6600 -41.4000 0.2357 0.1279
0.2302 -0.2202 -0.0088 -0.0495 -0.2968
-0.2633 -1.2479 -39.9346
67.6800 -41.6100 -0.0241 0.2484
0.2285 -0.2323 0.0048 -0.0546 -0.3059
-0.2595 -1.2473 -39.9336

67.7000 -41.8800 -0.1892 0.1761
0.1770 -0.2404 0.0199 -0.0596 -0.3151
-0.2556 -1.2467 -39.9326
67.7200 -41.8100 0.1615 -0.0747
0.0940 -0.2432 0.0362 -0.0644 -0.3243
-0.2517 -1.2461 -39.9315
67.7400 -42.4100 -0.1696 -0.2687
0.0102 -0.2386 0.0532 -0.0691 -0.3336
-0.2477 -1.2454 -39.9305
67.7600 -42.0400 0.2316 -0.2254 -
0.0530 -0.2241 0.0707 -0.0736 -0.3428
-0.2436 -1.2447 -39.9295
67.7800 -42.3700 -0.2576 -0.0695 -
0.0919 -0.1967 0.0879 -0.0779 -0.3521
-0.2395 -1.2441 -39.9285
67.8000 -41.7400 0.2882 0.0392 -
0.1171 -0.1548 0.1045 -0.0820 -0.3614
-0.2353 -1.2433 -39.9274
67.8200 -42.0700 -0.3079 0.1617 -
0.1469 -0.0985 0.1200 -0.0860 -0.3706
-0.2311 -1.2426 -39.9264
67.8400 -41.3400 0.1832 0.3553 -
0.1808 -0.0296 0.1338 -0.0898 -0.3799
-0.2268 -1.2419 -39.9254
67.8600 -41.4300 0.0521 0.3102 -
0.1913 0.0476 0.1457 -0.0934 -0.3891
-0.2225 -1.2411 -39.9244
67.8800 -41.5000 0.3118 -0.1625 -
0.1523 0.1280 0.1553 -0.0968 -0.3984
-0.2181 -1.2403 -39.9234
67.9000 -42.1500 0.1001 -0.6631 -
0.0611 0.2055 0.1622 -0.1000 -0.4075
-0.2136 -1.2395 -39.9223
67.9200 -42.8200 -0.8909 -0.6134
0.0705 0.2739 0.1661 -0.1030 -0.4167
-0.2091 -1.2387 -39.9213
67.9400 -40.3600 0.7054 0.1247
0.2095 0.3277 0.1667 -0.1058 -0.4258
-0.2046 -1.2378 -39.9203
67.9600 -39.9300 0.3692 0.7848
0.3011 0.3616 0.1636 -0.1083 -0.4348
-0.2000 -1.2369 -39.9193
67.9800 -39.7600 0.7019 0.6803
0.2991 0.3718 0.1568 -0.1106 -0.4438
-0.1953 -1.2360 -39.9182
68.0000 -41.6800 -0.5278 0.0799
0.2137 0.3572 0.1463 -0.1126 -0.4527
-0.1906 -1.2351 -39.9172
68.0200 -42.2900 -0.5391 -0.4404
0.0839 0.3207 0.1322 -0.1142 -0.4615
-0.1858 -1.2342 -39.9162

68.0400 -41.5900 0.5893 -0.6214 -
0.0468 0.2661 0.1151 -0.1155 -0.4703
-0.1810 -1.2333 -39.9152
68.0600 -42.5200 -0.2717 -0.5045 -
0.1398 0.1975 0.0955 -0.1163 -0.4789
-0.1761 -1.2323 -39.9141
68.0800 -42.1700 -0.1722 -0.1692 -
0.1769 0.1195 0.0741 -0.1167 -0.4875
-0.1712 -1.2313 -39.9131
68.1000 -41.6200 0.1529 0.2371 -
0.1666 0.0370 0.0513 -0.1166 -0.4959
-0.1662 -1.2303 -39.9121
68.1200 -41.5500 0.0346 0.4156 -
0.1224 -0.0453 0.0279 -0.1160 -0.5042
-0.1612 -1.2293 -39.9111
68.1400 -41.8600 -0.0271 0.2548 -
0.0610 -0.1230 0.0043 -0.1149 -0.5124
-0.1562 -1.2282 -39.9100
68.1600 -41.9000 0.3814 -0.0554 -
0.0018 -0.1920 -0.0187 -0.1132 -0.5205
-0.1510 -1.2272 -39.9090
68.1800 -43.0000 -0.7157 -0.1819
0.0403 -0.2491 -0.0407 -0.1110 -0.5283
-0.1459 -1.2261 -39.9080
68.2000 -41.6000 0.6989 -0.0850
0.0612 -0.2916 -0.0611 -0.1082 -0.5361
-0.1407 -1.2250 -39.9069
68.2200 -41.9700 0.3121 -0.0194
0.0627 -0.3174 -0.0794 -0.1047 -0.5437
-0.1354 -1.2239 -39.9059
68.2400 -43.1100 -0.8476 0.0119
0.0421 -0.3258 -0.0952 -0.1006 -0.5511
-0.1301 -1.2227 -39.9049
68.2600 -42.0200 0.1307 0.0998 -
0.0031 -0.3170 -0.1081 -0.0959 -0.5583
-0.1248 -1.2216 -39.9039
68.2800 -41.5000 0.6073 0.1650 -
0.0696 -0.2922 -0.1180 -0.0904 -0.5653
-0.1194 -1.2204 -39.9028
68.3000 -42.1500 0.0747 0.1046 -
0.1471 -0.2533 -0.1250 -0.0844 -0.5721
-0.1139 -1.2192 -39.9018
68.3200 -43.1900 -0.7268 -0.0160 -
0.2146 -0.2028 -0.1291 -0.0776 -0.5787
-0.1085 -1.2180 -39.9008
68.3400 -42.2100 0.3242 -0.1569 -
0.2480 -0.1435 -0.1307 -0.0702 -0.5851
-0.1029 -1.2168 -39.8997
68.3600 -41.8600 0.7393 -0.2996 -
0.2273 -0.0790 -0.1299 -0.0621 -0.5913
-0.0974 -1.2155 -39.8987

68.3800 -43.2300 -0.7713 -0.3490 -
0.1470 -0.0126 -0.1270 -0.0533 -0.5972
-0.0918 -1.2143 -39.8977
68.4000 -42.4300 -0.3520 -0.1611 -
0.0203 0.0524 -0.1221 -0.0439 -0.6029
-0.0861 -1.2130 -39.8966
68.4200 -40.7100 0.7835 0.1758
0.1250 0.1127 -0.1156 -0.0339 -0.6083
-0.0804 -1.2117 -39.8956
68.4400 -40.7900 0.3058 0.4248
0.2576 0.1649 -0.1078 -0.0234 -0.6135
-0.0747 -1.2104 -39.8946
68.4600 -41.6500 -0.7090 0.4368
0.3476 0.2059 -0.0989 -0.0122 -0.6184
-0.0689 -1.2090 -39.8935
68.4800 -40.8900 0.1231 0.1840
0.3774 0.2329 -0.0893 -0.0006 -0.6230
-0.0631 -1.2077 -39.8925
68.5000 -40.7500 0.5719 -0.1955
0.3486 0.2448 -0.0793 0.0116 -0.6273
-0.0573 -1.2063 -39.8915
68.5200 -42.3900 -0.6461 -0.4652
0.2729 0.2420 -0.0693 0.0243 -0.6313
-0.0514 -1.2049 -39.8904
68.5400 -41.5700 0.1738 -0.3091
0.1663 0.2266 -0.0598 0.0375 -0.6350
-0.0455 -1.2035 -39.8894
68.5600 -41.3100 0.0909 0.1632
0.0435 0.2016 -0.0511 0.0511 -0.6384
-0.0396 -1.2021 -39.8884
68.5800 -41.2800 -0.0664 0.4781 -
0.0824 0.1705 -0.0438 0.0652 -0.6415
-0.0336 -1.2007 -39.8873
68.6000 -40.9000 0.5539 0.2870 -
0.1993 0.1371 -0.0384 0.0797 -0.6442
-0.0276 -1.1992 -39.8863
68.6200 -42.7700 -0.7333 -0.1556 -
0.2940 0.1046 -0.0351 0.0945 -0.6465
-0.0215 -1.1978 -39.8853
68.6400 -42.0700 0.1357 -0.2938 -
0.3516 0.0766 -0.0346 0.1097 -0.6485
-0.0155 -1.1963 -39.8842
68.6600 -41.7800 0.4104 -0.1953 -
0.3541 0.0562 -0.0373 0.1251 -0.6502
-0.0094 -1.1948 -39.8832
68.6800 -41.6800 0.5897 -0.2629 -
0.2894 0.0460 -0.0432 0.1408 -0.6514
-0.0032 -1.1933 -39.8822
68.7000 -42.9500 -0.8354 -0.3250 -
0.1706 0.0472 -0.0526 0.1566 -0.6523
0.0029 -1.1917 -39.8811

68.7200	-41.5500	0.0229	-0.0289	-
0.0264	0.0602	-0.0651	0.1723	-0.6527
	0.0091	-1.1902	-39.8801	
68.7400	-40.3900	0.6183	0.4041	
0.1044	0.0846	-0.0806	0.1880	-0.6528
	0.0153	-1.1886	-39.8790	
68.7600	-40.4100	0.3155	0.5492	
0.1831	0.1193	-0.0984	0.2035	-0.6524
	0.0216	-1.1870	-39.8780	
68.7800	-40.7900	0.2247	0.2734	
0.1851	0.1625	-0.1177	0.2187	-0.6516
	0.0278	-1.1854	-39.8770	
68.8000	-41.7600	-0.4561	-0.0925	
0.1220	0.2106	-0.1380	0.2334	-0.6504
	0.0341	-1.1838	-39.8759	
68.8200	-42.3000	-0.7434	-0.1964	
0.0234	0.2598	-0.1582	0.2476	-0.6487
	0.0404	-1.1822	-39.8749	
68.8400	-40.5500	0.9228	-0.0932	-
0.0720	0.3054	-0.1777	0.2611	-0.6466
	0.0467	-1.1805	-39.8739	
68.8600	-41.3800	0.0933	-0.1753	-
0.1228	0.3430	-0.1958	0.2739	-0.6441
	0.0531	-1.1789	-39.8728	
68.8800	-42.0100	-0.2807	-0.4039	-
0.1023	0.3684	-0.2115	0.2857	-0.6410
	0.0594	-1.1772	-39.8718	
68.9000	-42.1300	-0.5804	-0.3854	-
0.0110	0.3776	-0.2244	0.2966	-0.6375
	0.0658	-1.1755	-39.8707	
68.9200	-40.9000	0.2133	-0.0519	
0.1239	0.3673	-0.2338	0.3064	-0.6335
	0.0722	-1.1738	-39.8697	
68.9400	-40.4600	0.2057	0.2726	
0.2569	0.3345	-0.2391	0.3151	-0.6291
	0.0786	-1.1720	-39.8687	
68.9600	-40.3000	0.3186	0.3194	
0.3433	0.2770	-0.2400	0.3224	-0.6241
	0.0850	-1.1703	-39.8676	
68.9800	-41.0600	-0.3586	0.1808	
0.3528	0.1940	-0.2365	0.3285	-0.6186
	0.0914	-1.1685	-39.8666	
69.0000	-40.9700	-0.0014	0.1183	
0.2747	0.0878	-0.2285	0.3331	-0.6127
	0.0978	-1.1668	-39.8655	
69.0200	-41.1200	0.0669	0.1141	
0.1291	-0.0357	-0.2162	0.3362	-0.6063
	0.1043	-1.1650	-39.8645	
69.0400	-41.3900	0.3185	-0.0525	-
0.0385	-0.1698	-0.1995	0.3378	-0.5993
	0.1107	-1.1632	-39.8634	

69.0600 -42.4400 -0.4093 -0.2906 -
0.1848 -0.3075 -0.1788 0.3379 -0.5919
0.1172 -1.1614 -39.8624
69.0800 -42.1700 0.2317 -0.2987 -
0.2806 -0.4419 -0.1540 0.3364 -0.5839
0.1236 -1.1595 -39.8614
69.1000 -42.6700 -0.3065 -0.0444 -
0.3174 -0.5662 -0.1255 0.3333 -0.5755
0.1301 -1.1577 -39.8603
69.1200 -41.9700 -0.0702 0.2330 -
0.3014 -0.6743 -0.0932 0.3285 -0.5665
0.1366 -1.1558 -39.8593
69.1400 -41.3400 0.5041 0.2540 -
0.2447 -0.7609 -0.0574 0.3222 -0.5570
0.1430 -1.1539 -39.8582
69.1600 -42.4700 -0.2609 -0.0625 -
0.1602 -0.8219 -0.0182 0.3142 -0.5471
0.1495 -1.1521 -39.8572
69.1800 -42.4600 -0.1869 -0.3242 -
0.0599 -0.8540 0.0241 0.3045 -0.5366
0.1560 -1.1501 -39.8562
69.2000 -42.5300 -0.5922 -0.0985
0.0425 -0.8543 0.0693 0.2932 -0.5257
0.1624 -1.1482 -39.8551
69.2200 -40.5900 0.7851 0.2775
0.1190 -0.8201 0.1168 0.2803 -0.5142
0.1689 -1.1463 -39.8541
69.2400 -41.4900 -0.2492 0.2339
0.1412 -0.7502 0.1661 0.2657 -0.5023
0.1753 -1.1443 -39.8530
69.2600 -41.5200 -0.2027 -0.0248
0.0895 -0.6450 0.2165 0.2494 -0.4900
0.1818 -1.1424 -39.8520
69.2800 -41.9500 -0.6098 0.0077 -
0.0381 -0.5073 0.2671 0.2315 -0.4772
0.1882 -1.1404 -39.8509
69.3000 -40.4400 0.6857 0.1752 -
0.2122 -0.3431 0.3172 0.2120 -0.4640
0.1947 -1.1384 -39.8499
69.3200 -41.6300 -0.4806 0.0397 -
0.3901 -0.1596 0.3658 0.1908 -0.4503
0.2011 -1.1364 -39.8488
69.3400 -41.0900 0.2302 -0.2511 -
0.5311 0.0352 0.4122 0.1680 -0.4363
0.2075 -1.1344 -39.8478
69.3600 -41.3700 -0.1307 -0.3232 -
0.5966 0.2333 0.4556 0.1436 -0.4219
0.2139 -1.1323 -39.8468
69.3800 -41.1400 -0.2817 -0.1903 -
0.5620 0.4272 0.4950 0.1175 -0.4071
0.2203 -1.1303 -39.8457

69.4000 -39.9600 0.4173 -0.0708 -
0.4195 0.6099 0.5296 0.0899 -0.3919
0.2267 -1.1282 -39.8447
69.4200 -39.9900 -0.0270 -0.1401 -
0.1748 0.7749 0.5586 0.0606 -0.3763
0.2331 -1.1262 -39.8436
69.4400 -39.8800 -0.2129 -0.2524
0.1400 0.9155 0.5812 0.0299 -0.3605
0.2394 -1.1241 -39.8426
69.4600 -39.2800 -0.1171 -0.2082
0.4751 1.0253 0.5964 -0.0025 -0.3443
0.2458 -1.1220 -39.8415
69.4800 -38.2400 0.3880 -0.0291
0.7746 1.0979 0.6034 -0.0363 -0.3278
0.2521 -1.1198 -39.8405
69.5000 -38.4000 -0.3059 0.2312
0.9841 1.1267 0.6014 -0.0715 -0.3111
0.2584 -1.1177 -39.8394
69.5200 -38.0900 -0.2633 0.5537
1.0551 1.1056 0.5902 -0.1080 -0.2940
0.2647 -1.1156 -39.8384
69.5400 -37.3900 0.3859 0.7649
0.9535 1.0327 0.5700 -0.1458 -0.2767
0.2709 -1.1134 -39.8373
69.5600 -37.8200 0.4827 0.5687
0.6801 0.9150 0.5415 -0.1847 -0.2592
0.2772 -1.1113 -39.8363
69.5800 -39.8900 -0.3539 0.0256
0.2922 0.7620 0.5056 -0.2245 -0.2415
0.2834 -1.1091 -39.8352
69.6000 -41.1700 -0.4269 -0.4999 -
0.1356 0.5833 0.4631 -0.2650 -0.2235
0.2896 -1.1069 -39.8342
69.6200 -41.2400 0.2618 -0.7223 -
0.5275 0.3886 0.4146 -0.3062 -0.2053
0.2957 -1.1047 -39.8331
69.6400 -41.9900 0.1522 -0.6401 -
0.8147 0.1877 0.3611 -0.3478 -0.1870
0.3019 -1.1024 -39.8321
69.6600 -42.1700 0.0160 -0.4055 -
0.9530 -0.0099 0.3034 -0.3898 -0.1686
0.3080 -1.1002 -39.8310
69.6800 -42.5400 -0.3001 -0.0964 -
0.9329 -0.1962 0.2421 -0.4319 -0.1499
0.3141 -1.0980 -39.8300
69.7000 -41.7500 0.3492 0.1657 -
0.7703 -0.3655 0.1781 -0.4740 -0.1312
0.3201 -1.0957 -39.8289
69.7200 -42.1000 -0.2025 0.2162 -
0.5010 -0.5148 0.1123 -0.5160 -0.1124
0.3262 -1.0934 -39.8279

69.7400 -41.7300 0.0516 0.1048 -
0.1785 -0.6415 0.0453 -0.5577 -0.0935
0.3322 -1.0912 -39.8268
69.7600 -41.8300 -0.0476 0.0229
0.1355 -0.7431 -0.0221 -0.5989 -0.0745
0.3381 -1.0889 -39.8258
69.7800 -41.7000 0.0294 0.0604
0.3866 -0.8179 -0.0889 -0.6394 -0.0554
0.3441 -1.0865 -39.8247
69.8000 -41.7500 -0.2051 0.2002
0.5344 -0.8649 -0.1544 -0.6793 -0.0363
0.3500 -1.0842 -39.8237
69.8200 -41.1400 0.4583 0.2902
0.5581 -0.8848 -0.2178 -0.7182 -0.0172
0.3558 -1.0819 -39.8226
69.8400 -42.0600 -0.2926 0.2071
0.4637 -0.8806 -0.2783 -0.7560 0.0019
0.3617 -1.0796 -39.8216
69.8600 -42.2800 -0.1510 0.1210
0.2823 -0.8569 -0.3351 -0.7926 0.0210
0.3675 -1.0772 -39.8205
69.8800 -42.4100 0.0722 0.0835
0.0614 -0.8187 -0.3877 -0.8278 0.0401
0.3732 -1.0748 -39.8195
69.9000 -42.2000 0.4830 -0.0989 -
0.1435 -0.7711 -0.4353 -0.8615 0.0591
0.3789 -1.0724 -39.8184
69.9200 -43.4500 -0.2115 -0.4152 -
0.2873 -0.7192 -0.4774 -0.8935 0.0781
0.3846 -1.0701 -39.8174
69.9400 -44.2000 -0.7063 -0.5820 -
0.3401 -0.6676 -0.5136 -0.9237 0.0970
0.3902 -1.0677 -39.8163
69.9600 -42.7400 0.4256 -0.4435 -
0.2909 -0.6197 -0.5434 -0.9519 0.1158
0.3958 -1.0652 -39.8153
69.9800 -41.9400 0.9278 -0.2265 -
0.1599 -0.5769 -0.5666 -0.9779 0.1345
0.4014 -1.0628 -39.8142
70.0000 -42.6700 -0.0791 -0.1172
0.0163 -0.5386 -0.5831 -1.0016 0.1531
0.4069 -1.0604 -39.8132
70.0200 -43.2400 -1.1773 0.0460
0.1877 -0.5037 -0.5929 -1.0230 0.1716
0.4123 -1.0579 -39.8121
70.0400 -42.4000 -0.8473 0.4099
0.3074 -0.4710 -0.5963 -1.0419 0.1899
0.4177 -1.0555 -39.8111
70.0600 -40.9700 0.2192 0.8048
0.3490 -0.4389 -0.5937 -1.0582 0.2081
0.4231 -1.0530 -39.8100

70.0800 -40.0800 1.0780 0.8553
0.2998 -0.4059 -0.5855 -1.0719 0.2261
0.4284 -1.0505 -39.8090
70.1000 -40.7800 0.9442 0.2950
0.1712 -0.3700 -0.5724 -1.0831 0.2439
0.4337 -1.0480 -39.8079
70.1200 -43.7200 -1.0678 -0.4955
0.0044 -0.3297 -0.5548 -1.0916 0.2615
0.4389 -1.0455 -39.8069
70.1400 -42.8700 0.1030 -0.8365 -
0.1530 -0.2833 -0.5334 -1.0974 0.2789
0.4441 -1.0430 -39.8058
70.1600 -42.8000 -0.0172 -0.5834 -
0.2620 -0.2294 -0.5087 -1.1006 0.2962
0.4492 -1.0405 -39.8047
70.1800 -42.0500 0.2161 -0.0910 -
0.3120 -0.1671 -0.4813 -1.1011 0.3132
0.4542 -1.0379 -39.8037
70.2000 -41.7400 -0.1577 0.3797 -
0.3072 -0.0965 -0.4517 -1.0990 0.3299
0.4593 -1.0354 -39.8026
70.2200 -41.0600 0.2027 0.5642 -
0.2533 -0.0192 -0.4201 -1.0942 0.3465
0.4642 -1.0328 -39.8016
70.2400 -40.9700 0.2700 0.3322 -
0.1593 0.0625 -0.3870 -1.0868 0.3628
0.4691 -1.0303 -39.8005
70.2600 -41.3000 0.1586 -0.1252 -
0.0423 0.1455 -0.3527 -1.0768 0.3789
0.4739 -1.0277 -39.7995
70.2800 -42.0600 -0.7377 -0.3445
0.0793 0.2262 -0.3173 -1.0642 0.3947
0.4787 -1.0251 -39.7984
70.3000 -40.1800 0.7892 -0.1571
0.1913 0.3011 -0.2812 -1.0491 0.4103
0.4834 -1.0225 -39.7974
70.3200 -41.1300 -0.6498 0.0219
0.2830 0.3668 -0.2446 -1.0315 0.4256
0.4881 -1.0199 -39.7963
70.3400 -40.0200 0.3573 0.0564
0.3360 0.4204 -0.2078 -1.0115 0.4407
0.4927 -1.0173 -39.7953
70.3600 -40.2100 -0.1400 0.0789
0.3240 0.4595 -0.1709 -0.9892 0.4555
0.4972 -1.0146 -39.7942
70.3800 -40.0400 0.1033 0.0908
0.2355 0.4823 -0.1342 -0.9645 0.4701
0.5017 -1.0120 -39.7931
70.4000 -40.1500 0.1146 0.1015
0.0824 0.4881 -0.0978 -0.9376 0.4844
0.5061 -1.0094 -39.7921

70.4200 -40.6100 -0.4281 0.1509 -
0.1031 0.4772 -0.0617 -0.9085 0.4984
0.5104 -1.0067 -39.7910
70.4400 -40.0100 0.3667 0.1025 -
0.2717 0.4503 -0.0261 -0.8773 0.5121
0.5147 -1.0040 -39.7900
70.4600 -40.4400 0.3363 -0.1749 -
0.3763 0.4085 0.0090 -0.8440 0.5256
0.5189 -1.0013 -39.7889
70.4800 -41.4400 -0.5973 -0.4649 -
0.3869 0.3538 0.0435 -0.8088 0.5388
0.5230 -0.9987 -39.7879
70.5000 -40.9900 -0.1113 -0.4291 -
0.2936 0.2885 0.0774 -0.7716 0.5518
0.5271 -0.9960 -39.7868
70.5200 -40.1300 0.1953 -0.0923 -
0.1225 0.2156 0.1105 -0.7325 0.5644
0.5311 -0.9933 -39.7857
70.5400 -39.4000 0.3869 0.2400
0.0774 0.1382 0.1427 -0.6917 0.5767
0.5350 -0.9905 -39.7847
70.5600 -39.7000 -0.3040 0.3933
0.2530 0.0596 0.1740 -0.6492 0.5888
0.5388 -0.9878 -39.7836
70.5800 -39.3700 -0.1535 0.4214
0.3573 -0.0168 0.2043 -0.6051 0.6006
0.5426 -0.9851 -39.7826
70.6000 -39.0000 0.3004 0.3473
0.3667 -0.0874 0.2333 -0.5594 0.6121
0.5463 -0.9823 -39.7815
70.6200 -39.6500 -0.1595 0.1046
0.2899 -0.1489 0.2611 -0.5123 0.6232
0.5499 -0.9796 -39.7805
70.6400 -39.7800 0.3089 -0.2824
0.1572 -0.1988 0.2874 -0.4638 0.6341
0.5534 -0.9768 -39.7794
70.6600 -40.7500 -0.5018 -0.4832
0.0022 -0.2347 0.3122 -0.4140 0.6447
0.5569 -0.9740 -39.7783
70.6800 -39.6100 0.4234 -0.1842 -
0.1453 -0.2548 0.3352 -0.3629 0.6549
0.5603 -0.9713 -39.7773
70.7000 -40.0300 -0.4183 0.3254 -
0.2598 -0.2572 0.3563 -0.3107 0.6648
0.5636 -0.9685 -39.7762
70.7200 -39.0300 0.6299 0.4105 -
0.3209 -0.2411 0.3754 -0.2575 0.6745
0.5668 -0.9657 -39.7752
70.7400 -39.7600 0.0617 0.0065 -
0.3196 -0.2069 0.3921 -0.2032 0.6837
0.5700 -0.9629 -39.7741

70.7600 -40.6500 -0.6933 -0.4028 -
0.2591 -0.1571 0.4062 -0.1478 0.6927
0.5730 -0.9601 -39.7730
70.7800 -39.2500 0.5853 -0.3429 -
0.1491 -0.0958 0.4176 -0.0916 0.7013
0.5760 -0.9572 -39.7720
70.8000 -39.3800 -0.4471 0.0403 -
0.0151 -0.0276 0.4258 -0.0343 0.7096
0.5789 -0.9544 -39.7709
70.8200 -38.1800 0.3634 0.3185
0.1102 0.0424 0.4308 0.0238 0.7175
0.5817 -0.9516 -39.7699
70.8400 -38.3800 -0.0679 0.3242
0.1976 0.1091 0.4320 0.0828 0.7251
0.5844 -0.9487 -39.7688
70.8600 -38.3600 -0.1219 0.1176
0.2347 0.1677 0.4293 0.1427 0.7324
0.5870 -0.9459 -39.7678
70.8800 -38.3000 0.2357 -0.1674
0.2288 0.2138 0.4225 0.2034 0.7393
0.5896 -0.9430 -39.7667
70.9000 -38.7400 -0.1912 -0.3489
0.1975 0.2439 0.4111 0.2649 0.7458
0.5920 -0.9401 -39.7656
70.9200 -38.5200 -0.0631 -0.2900
0.1564 0.2560 0.3951 0.3271 0.7520
0.5944 -0.9372 -39.7646
70.9400 -38.0800 0.2290 -0.0343
0.1131 0.2498 0.3744 0.3901 0.7578
0.5967 -0.9344 -39.7635
70.9600 -38.1400 -0.1590 0.2425
0.0661 0.2273 0.3489 0.4536 0.7632
0.5988 -0.9315 -39.7625
70.9800 -37.9000 0.0654 0.3551
0.0114 0.1915 0.3191 0.5177 0.7682
0.6009 -0.9285 -39.7614
71.0000 -38.1300 0.1242 0.1910 -
0.0493 0.1462 0.2850 0.5822 0.7729
0.6029 -0.9256 -39.7603
71.0200 -38.3900 -0.0057 -0.1174 -
0.1062 0.0954 0.2473 0.6471 0.7772
0.6048 -0.9227 -39.7593
71.0400 -39.0400 -0.3379 -0.2938 -
0.1425 0.0432 0.2063 0.7123 0.7811
0.6066 -0.9198 -39.7582
71.0600 -38.4700 0.2233 -0.2298 -
0.1431 -0.0064 0.1626 0.7776 0.7846
0.6083 -0.9168 -39.7571
71.0800 -38.4100 0.1272 -0.0471 -
0.1109 -0.0501 0.1165 0.8430 0.7877
0.6099 -0.9139 -39.7561

71.1000 -38.4100 -0.1288 0.0914 -
0.0633 -0.0850 0.0687 0.9084 0.7903
0.6114 -0.9109 -39.7550
71.1200 -38.0700 0.1449 0.1373 -
0.0228 -0.1089 0.0197 0.9736 0.7926
0.6128 -0.9080 -39.7540
71.1400 -38.4500 -0.2442 0.1176 -
0.0058 -0.1205 -0.0301 1.0385 0.7945
0.6141 -0.9050 -39.7529
71.1600 -38.0400 0.2341 0.0476 -
0.0130 -0.1200 -0.0802 1.1031 0.7960
0.6153 -0.9020 -39.7518
71.1800 -38.4000 -0.0083 -0.0333 -
0.0326 -0.1088 -0.1299 1.1671 0.7970
0.6164 -0.8991 -39.7508
71.2000 -38.5800 -0.1121 -0.0810 -
0.0459 -0.0890 -0.1789 1.2305 0.7976
0.6174 -0.8961 -39.7497
71.2200 -38.4400 -0.0929 -0.0804 -
0.0371 -0.0632 -0.2266 1.2932 0.7978
0.6183 -0.8931 -39.7487
71.2400 -37.9200 0.3691 -0.0891 -
0.0012 -0.0341 -0.2726 1.3549 0.7975
0.6191 -0.8901 -39.7476
71.2600 -38.5700 -0.3832 -0.1280
0.0489 -0.0041 -0.3165 1.4157 0.7968
0.6198 -0.8870 -39.7465
71.2800 -37.9000 0.0760 -0.0155
0.0898 0.0247 -0.3579 1.4754 0.7956
0.6204 -0.8840 -39.7455
71.3000 -37.9200 -0.3280 0.2737
0.0964 0.0504 -0.3964 1.5339 0.7940
0.6209 -0.8810 -39.7444
71.3200 -37.1000 0.4830 0.3677
0.0582 0.0715 -0.4317 1.5911 0.7919
0.6213 -0.8779 -39.7433
71.3400 -37.9200 -0.0941 0.0598 -
0.0139 0.0870 -0.4634 1.6469 0.7894
0.6216 -0.8749 -39.7423
71.3600 -38.5000 -0.1650 -0.3523 -
0.0850 0.0960 -0.4912 1.7012 0.7864
0.6218 -0.8719 -39.7412
71.3800 -38.6800 -0.1876 -0.4734 -
0.1152 0.0979 -0.5147 1.7539 0.7829
0.6219 -0.8688 -39.7402
71.4000 -38.0500 0.0093 -0.1994 -
0.0845 0.0918 -0.5335 1.8048 0.7790
0.6219 -0.8657 -39.7391
71.4200 -37.8800 -0.2716 0.2534 -
0.0140 0.0768 -0.5474 1.8539 0.7746
0.6217 -0.8627 -39.7380

71.4400 -36.8800 0.5219 0.4427
0.0629 0.0520 -0.5562 1.9011 0.7696
0.6215 -0.8596 -39.7370
71.4600 -37.8200 -0.3789 0.1895
0.1170 0.0170 -0.5598 1.9462 0.7642
0.6212 -0.8565 -39.7359
71.4800 -37.5400 0.2316 -0.1580
0.1403 -0.0277 -0.5581 1.9890 0.7583
0.6207 -0.8534 -39.7348
71.5000 -38.3600 -0.4192 -0.2543
0.1328 -0.0810 -0.5509 2.0296 0.7519
0.6202 -0.8503 -39.7338
71.5200 -37.4600 0.4277 -0.1264
0.0955 -0.1410 -0.5384 2.0678 0.7449
0.6196 -0.8472 -39.7327
71.5400 -37.9400 -0.2192 0.0708
0.0367 -0.2052 -0.5205 2.1034 0.7375
0.6188 -0.8441 -39.7317
71.5600 -37.8200 -0.2293 0.2201 –
0.0305 -0.2705 -0.4973 2.1364 0.7295
0.6180 -0.8409 -39.7306
71.5800 -37.3900 0.4341 0.1893 –
0.0905 -0.3342 -0.4687 2.1665 0.7210
0.6171 -0.8378 -39.7295
71.6000 -38.3100 -0.3181 -0.0475 –
0.1271 -0.3940 -0.4349 2.1938 0.7119
0.6160 -0.8347 -39.7285
71.6200 -38.0000 0.2067 -0.2806 –
0.1273 -0.4473 -0.3959 2.2181 0.7024
0.6149 -0.8315 -39.7274
71.6400 -38.2900 0.0156 -0.3069 –
0.0908 -0.4919 -0.3518 2.2394 0.6922
0.6137 -0.8284 -39.7263
71.6600 -38.1200 -0.1702 -0.0938 –
0.0339 -0.5261 -0.3028 2.2574 0.6816
0.6124 -0.8252 -39.7253
71.6800 -37.6300 -0.0614 0.2350
0.0188 -0.5491 -0.2491 2.2721 0.6703
0.6110 -0.8220 -39.7242
71.7000 -37.1500 0.2399 0.4227
0.0458 -0.5608 -0.1908 2.2835 0.6585
0.6095 -0.8189 -39.7231
71.7200 -37.2200 0.2359 0.2685
0.0372 -0.5613 -0.1283 2.2913 0.6462
0.6079 -0.8157 -39.7221
71.7400 -38.1100 -0.4256 -0.0833
0.0084 -0.5512 -0.0618 2.2957 0.6333
0.6062 -0.8125 -39.7210
71.7600 -37.6900 0.1783 -0.3159 –
0.0150 -0.5318 0.0086 2.2963 0.6199
0.6044 -0.8093 -39.7200

71.7800 -37.6900 0.1121 -0.3294 –
0.0146 -0.5047 0.0824 2.2932 0.6059
0.6025 -0.8061 -39.7189
71.8000 -37.6600 -0.1935 -0.1887
0.0151 -0.4716 0.1593 2.2863 0.5914
0.6005 -0.8029 -39.7178
71.8200 -37.0700 0.1838 0.0114
0.0644 -0.4340 0.2389 2.2755 0.5764
0.5985 -0.7997 -39.7168
71.8400 -37.0400 -0.1759 0.1902
0.1115 -0.3937 0.3206 2.2605 0.5609
0.5964 -0.7965 -39.7157
71.8600 -36.5500 0.1920 0.2515
0.1344 -0.3521 0.4038 2.2415 0.5449
0.5941 -0.7933 -39.7146
71.8800 -36.6600 0.0729 0.1551
0.1208 -0.3105 0.4877 2.2183 0.5284
0.5918 -0.7900 -39.7136
71.9000 -37.0200 -0.2099 0.0028
0.0683 -0.2689 0.5716 2.1907 0.5114
0.5894 -0.7868 -39.7125
71.9200 -36.8800 -0.0055 -0.0669 –
0.0131 -0.2261 0.6542 2.1587 0.4940
0.5869 -0.7835 -39.7114
71.9400 -36.7200 0.2095 -0.0552 –
0.1045 -0.1804 0.7344 2.1223 0.4761
0.5844 -0.7803 -39.7104
71.9600 -36.9700 -0.0696 -0.0418 –
0.1846 -0.1297 0.8109 2.0813 0.4578
0.5817 -0.7770 -39.7093
71.9800 -37.0800 -0.1604 -0.0475 –
0.2382 -0.0710 0.8823 2.0357 0.4391
0.5790 -0.7738 -39.7082
72.0000 -36.8300 -0.0462 -0.0444 –
0.2596 -0.0012 0.9472 1.9855 0.4200
0.5762 -0.7705 -39.7072
72.0200 -36.6700 0.2804 -0.0483 –
0.2554 0.0830 1.0043 1.9307 0.4005
0.5733 -0.7672 -39.7061
72.0400 -36.9000 -0.1965 -0.0540 –
0.2417 0.1842 1.0526 1.8712 0.3806
0.5703 -0.7639 -39.7050
72.0600 -36.4000 0.0509 0.0182 –
0.2326 0.3031 1.0909 1.8071 0.3604
0.5673 -0.7607 -39.7040
72.0800 -36.5400 -0.0906 0.1318 –
0.2337 0.4375 1.1185 1.7384 0.3399
0.5642 -0.7574 -39.7029
72.1000 -36.2800 0.0329 0.1466 –
0.2440 0.5824 1.1344 1.6652 0.3191
0.5610 -0.7541 -39.7018

72.1200 -36.3100 0.0559 -0.0056 -
0.2522 0.7302 1.1379 1.5876 0.2980
0.5577 -0.7507 -39.7008
72.1400 -36.2000 0.3277 -0.1900 -
0.2334 0.8714 1.1282 1.5059 0.2766
0.5543 -0.7474 -39.6997
72.1600 -37.0600 -0.5501 -0.1660 -
0.1637 0.9959 1.1045 1.4202 0.2550
0.5509 -0.7441 -39.6986
72.1800 -35.5500 0.5225 0.0722 -
0.0294 1.0943 1.0663 1.3307 0.2332
0.5474 -0.7408 -39.6976
72.2000 -36.1600 -0.1570 0.1170
0.1704 1.1590 1.0133 1.2376 0.2111
0.5439 -0.7375 -39.6965
72.2200 -36.1900 -0.0748 -0.0711
0.4136 1.1835 0.9457 1.1413 0.1888
0.5402 -0.7341 -39.6955
72.2400 -36.3600 -0.1357 -0.2052
0.6524 1.1628 0.8642 1.0420 0.1664
0.5365 -0.7308 -39.6944
72.2600 -36.0900 0.1944 -0.1497
0.8335 1.0933 0.7698 0.9399 0.1438
0.5327 -0.7274 -39.6933
72.2800 -36.4100 -0.1566 0.1130
0.9081 0.9740 0.6643 0.8352 0.1211
0.5289 -0.7241 -39.6923
72.3000 -36.3500 -0.0902 0.4739
0.8453 0.8086 0.5494 0.7283 0.0982
0.5250 -0.7207 -39.6912
72.3200 -36.5500 0.3247 0.6602
0.6389 0.6056 0.4269 0.6193 0.0753
0.5210 -0.7173 -39.6901
72.3400 -37.8200 0.0848 0.4118
0.3150 0.3763 0.2986 0.5086 0.0523
0.5170 -0.7140 -39.6891
72.3600 -39.2800 0.1809 -0.1654 -
0.0668 0.1326 0.1664 0.3964 0.0292
0.5129 -0.7106 -39.6880
72.3800 -41.2300 -0.4366 -0.6506 -
0.4342 -0.1138 0.0321 0.2828 0.0060
0.5087 -0.7072 -39.6869
72.4000 -41.6700 -0.0759 -0.7535 -
0.7183 -0.3508 -0.1025 0.1683 -0.0172
0.5045 -0.7038 -39.6859
72.4200 -41.5600 0.5417 -0.5471 -
0.8734 -0.5669 -0.2357 0.0530 -0.0404
0.5002 -0.7004 -39.6848
72.4400 -42.8100 -0.4379 -0.2569 -
0.8913 -0.7519 -0.3655 -0.0628 -0.0636
0.4958 -0.6970 -39.6837

72.4600 -42.1800 0.1132 0.0479 -
0.7952 -0.8989 -0.4901 -0.1789 -0.0867
0.4914 -0.6936 -39.6827
72.4800 -42.0000 0.2229 0.3515 -
0.6284 -1.0043 -0.6079 -0.2950 -0.1098
0.4869 -0.6902 -39.6816
72.5000 -42.4600 -0.3966 0.5638 -
0.4346 -1.0670 -0.7171 -0.4109 -0.1329
0.4824 -0.6868 -39.6805
72.5200 -42.0900 0.0995 0.5804 -
0.2468 -1.0882 -0.8162 -0.5262 -0.1559
0.4778 -0.6834 -39.6795
72.5400 -41.9000 0.5212 0.3249 -
0.0819 -1.0703 -0.9039 -0.6408 -0.1787
0.4731 -0.6800 -39.6784
72.5600 -43.1400 -0.1689 -0.1554
0.0566 -1.0178 -0.9789 -0.7545 -0.2015
0.4684 -0.6765 -39.6773
72.5800 -43.8700 -0.6191 -0.5126
0.1726 -0.9361 -1.0408 -0.8669 -0.2241
0.4637 -0.6731 -39.6763
72.6000 -43.3800 -0.2227 -0.4280
0.2711 -0.8309 -1.0891 -0.9777 -0.2466
0.4589 -0.6697 -39.6752
72.6200 -42.7900 0.0028 -0.0414
0.3511 -0.7081 -1.1236 -1.0869 -0.2689
0.4540 -0.6662 -39.6741
72.6400 -41.7800 0.5987 0.2473
0.4064 -0.5725 -1.1445 -1.1941 -0.2910
0.4491 -0.6628 -39.6731
72.6600 -42.0200 0.5242 0.1963
0.4280 -0.4288 -1.1520 -1.2990 -0.3129
0.4441 -0.6593 -39.6720
72.6800 -43.4200 -0.7925 -0.0002
0.4035 -0.2810 -1.1464 -1.4016 -0.3346
0.4391 -0.6558 -39.6709
72.7000 -42.8200 -0.1628 0.0079
0.3245 -0.1330 -1.1283 -1.5016 -0.3560
0.4341 -0.6524 -39.6699
72.7200 -42.5900 -0.0071 0.2221
0.2001 0.0113 -1.0983 -1.5988 -0.3772
0.4290 -0.6489 -39.6688
72.7400 -41.9500 0.6806 0.2450
0.0580 0.1475 -1.0572 -1.6932 -0.3981
0.4238 -0.6454 -39.6677
72.7600 -42.9600 -0.0128 -0.0639 -
0.0714 0.2715 -1.0057 -1.7846 -0.4187
0.4186 -0.6419 -39.6667
72.7800 -43.7500 -0.4412 -0.3808 -
0.1604 0.3795 -0.9446 -1.8730 -0.4390
0.4133 -0.6385 -39.6656

72.8000 -43.6700 -0.3239 -0.3608 -
0.1872 0.4683 -0.8746 -1.9581 -0.4589
0.4081 -0.6350 -39.6645
72.8200 -43.0500 -0.1947 0.0203 -
0.1522 0.5352 -0.7966 -2.0401 -0.4785
0.4027 -0.6315 -39.6635
72.8400 -41.7000 0.6391 0.3765 -
0.0816 0.5787 -0.7114 -2.1187 -0.4977
0.3973 -0.6280 -39.6624
72.8600 -42.2600 0.1795 0.3969 -
0.0038 0.5987 -0.6195 -2.1939 -0.5165
0.3919 -0.6245 -39.6613
72.8800 -42.9500 -0.5149 0.1733
0.0649 0.5959 -0.5221 -2.2656 -0.5349
0.3864 -0.6210 -39.6602
72.9000 -42.5200 0.0498 -0.0592
0.1164 0.5716 -0.4199 -2.3339 -0.5529
0.3809 -0.6175 -39.6592
72.9200 -42.6200 0.1431 -0.2262
0.1531 0.5279 -0.3142 -2.3985 -0.5704
0.3754 -0.6139 -39.6581
72.9400 -42.7900 0.0891 -0.3797
0.1814 0.4678 -0.2061 -2.4594 -0.5875
0.3698 -0.6104 -39.6570
72.9600 -43.1800 -0.2289 -0.4224
0.2002 0.3950 -0.0969 -2.5167 -0.6041
0.3642 -0.6069 -39.6560
72.9800 -42.7200 0.0723 -0.2198
0.1978 0.3140 0.0120 -2.5702 -0.6202
0.3585 -0.6034 -39.6549
73.0000 -42.5900 -0.1153 0.1549
0.1601 0.2299 0.1193 -2.6199 -0.6358
0.3529 -0.5998 -39.6538
73.0200 -41.9900 0.3302 0.4957
0.0805 0.1471 0.2237 -2.6658 -0.6509
0.3471 -0.5963 -39.6528
73.0400 -42.5100 -0.0992 0.5522 -
0.0380 0.0700 0.3238 -2.7079 -0.6654
0.3414 -0.5927 -39.6517
73.0600 -42.7400 0.1853 0.1631 -
0.1765 0.0024 0.4184 -2.7461 -0.6793
0.3356 -0.5892 -39.6506
73.0800 -43.1400 0.5242 -0.4480 -
0.3013 -0.0525 0.5062 -2.7803 -0.6927
0.3298 -0.5857 -39.6496
73.1000 -45.0700 -1.0549 -0.7165 -
0.3760 -0.0917 0.5861 -2.8106 -0.7054
0.3239 -0.5821 -39.6485
73.1200 -43.3300 0.1736 -0.3104 -
0.3764 -0.1140 0.6571 -2.8369 -0.7175
0.3180 -0.5785 -39.6474

73.1400 -41.7500 1.1058 0.3141 -
0.3140 -0.1202 0.7185 -2.8591 -0.7290
0.3121 -0.5750 -39.6464
73.1600 -42.7600 -0.2560 0.6209 -
0.2089 -0.1125 0.7698 -2.8773 -0.7399
0.3062 -0.5714 -39.6453
73.1800 -43.1900 -0.8251 0.5225 -
0.0805 -0.0938 0.8109 -2.8914 -0.7500
0.3002 -0.5678 -39.6442
73.2000 -42.2700 0.2391 0.1853
0.0556 -0.0681 0.8419 -2.9013 -0.7595
0.2942 -0.5643 -39.6432
73.2200 -42.1100 0.5659 -0.2106
0.1899 -0.0392 0.8627 -2.9071 -0.7682
0.2882 -0.5607 -39.6421
73.2400 -43.3400 -0.4172 -0.5108
0.3157 -0.0112 0.8737 -2.9088 -0.7763
0.2822 -0.5571 -39.6410
73.2600 -42.7700 -0.0362 -0.4728
0.4216 0.0124 0.8752 -2.9064 -0.7836
0.2761 -0.5535 -39.6400
73.2800 -42.5800 -0.3356 0.0006
0.4896 0.0291 0.8674 -2.9000 -0.7902
0.2700 -0.5499 -39.6389
73.3000 -41.3700 0.3300 0.4708
0.4970 0.0382 0.8508 -2.8895 -0.7960
0.2639 -0.5463 -39.6378
73.3200 -41.2700 0.5855 0.4580
0.4258 0.0399 0.8259 -2.8751 -0.8011
0.2578 -0.5428 -39.6368
73.3400 -43.1600 -0.6704 -0.0139
0.2858 0.0357 0.7933 -2.8569 -0.8055
0.2516 -0.5392 -39.6357
73.3600 -42.7700 0.1155 -0.2977
0.1019 0.0276 0.7535 -2.8349 -0.8091
0.2454 -0.5356 -39.6346
73.3800 -43.0600 0.0192 -0.0975 -
0.0971 0.0180 0.7071 -2.8093 -0.8120
0.2392 -0.5319 -39.6336
73.4000 -43.0900 -0.1313 0.2048 -
0.2768 0.0091 0.6545 -2.7802 -0.8142
0.2330 -0.5283 -39.6325
73.4200 -42.4000 0.7176 0.1473 -
0.4058 0.0037 0.5963 -2.7477 -0.8157
0.2268 -0.5247 -39.6314
73.4400 -43.9700 -0.2903 -0.2339 -
0.4749 0.0041 0.5332 -2.7118 -0.8165
0.2206 -0.5211 -39.6304
73.4600 -44.5400 -0.6581 -0.4739 -
0.4901 0.0124 0.4657 -2.6727 -0.8166
0.2143 -0.5175 -39.6293

73.4800 -43.5200 0.1137 -0.2622 -
0.4600 0.0292 0.3946 -2.6304 -0.8161
0.2080 -0.5139 -39.6282
73.5000 -42.7000 0.4682 0.2147 -
0.3951 0.0542 0.3207 -2.5852 -0.8149
0.2017 -0.5102 -39.6272
73.5200 -42.5200 0.1631 0.5200 -
0.3058 0.0863 0.2447 -2.5370 -0.8130
0.1954 -0.5066 -39.6261
73.5400 -42.2700 0.5292 0.3045 -
0.1996 0.1234 0.1674 -2.4861 -0.8105
0.1891 -0.5030 -39.6250
73.5600 -43.3700 -0.1239 -0.2897 -
0.0773 0.1622 0.0898 -2.4325 -0.8074
0.1828 -0.4993 -39.6240
73.5800 -43.7300 -0.0736 -0.7093
0.0632 0.1992 0.0126 -2.3763 -0.8037
0.1764 -0.4957 -39.6229
73.6000 -44.0400 -0.8584 -0.4403
0.2194 0.2305 -0.0633 -2.3177 -0.7994
0.1701 -0.4921 -39.6218
73.6200 -41.4300 0.8020 0.2749
0.3639 0.2525 -0.1371 -2.2568 -0.7944
0.1637 -0.4884 -39.6208
73.6400 -41.2700 0.4724 0.6959
0.4599 0.2623 -0.2081 -2.1936 -0.7890
0.1573 -0.4848 -39.6197
73.6600 -41.9600 -0.1872 0.5834
0.4738 0.2572 -0.2754 -2.1284 -0.7829
0.1509 -0.4811 -39.6186
73.6800 -42.8000 -0.5869 0.3013
0.3900 0.2357 -0.3385 -2.0611 -0.7763
0.1445 -0.4775 -39.6175
73.7000 -42.3800 0.1044 0.1693
0.2185 0.1977 -0.3966 -1.9921 -0.7692
0.1381 -0.4738 -39.6165
73.7200 -42.7100 0.1606 0.0262
0.0161 0.1443 -0.4493 -1.9213 -0.7616
0.1317 -0.4702 -39.6154
73.7400 -42.8900 0.5594 -0.3907 -
0.1510 0.0774 -0.4959 -1.8489 -0.7534
0.1252 -0.4665 -39.6143
73.7600 -44.6300 -0.7310 -0.7076 -
0.2355 -0.0009 -0.5359 -1.7751 -0.7447
0.1188 -0.4628 -39.6133
73.7800 -43.6000 0.0569 -0.4327 -
0.2115 -0.0874 -0.5690 -1.7000 -0.7356
0.1124 -0.4592 -39.6122
73.8000 -42.6900 0.3936 0.1500 -
0.1054 -0.1784 -0.5946 -1.6238 -0.7260
0.1059 -0.4555 -39.6111

73.8200 -42.0700 0.5145 0.4708
0.0332 -0.2697 -0.6123 -1.5466 -0.7159
0.0994 -0.4518 -39.6101
73.8400 -43.0500 -0.4404 0.4081
0.1524 -0.3570 -0.6219 -1.4684 -0.7054
0.0930 -0.4482 -39.6090
73.8600 -42.9500 -0.2686 0.1714
0.2139 -0.4360 -0.6232 -1.3896 -0.6945
0.0865 -0.4445 -39.6079
73.8800 -42.5200 0.4420 -0.0479
0.1999 -0.5026 -0.6159 -1.3100 -0.6831
0.0800 -0.4408 -39.6069
73.9000 -42.9700 0.0348 -0.1561
0.1123 -0.5529 -0.6000 -1.2300 -0.6714
0.0735 -0.4371 -39.6058
73.9200 -43.6500 -0.5163 -0.1262 -
0.0239 -0.5844 -0.5758 -1.1494 -0.6592
0.0671 -0.4334 -39.6047
73.9400 -43.2500 -0.2940 -0.0045 -
0.1656 -0.5948 -0.5434 -1.0685 -0.6467
0.0606 -0.4297 -39.6037
73.9600 -42.1800 0.7889 0.0206 -
0.2689 -0.5826 -0.5035 -0.9873 -0.6338
0.0541 -0.4260 -39.6026
73.9800 -43.5300 -0.4757 -0.1456 -
0.3060 -0.5475 -0.4566 -0.9059 -0.6205
0.0476 -0.4224 -39.6015
74.0000 -43.3500 -0.5155 -0.2274 -
0.2696 -0.4903 -0.4035 -0.8244 -0.6069
0.0411 -0.4187 -39.6005
74.0200 -42.0300 0.2977 -0.0571 -
0.1768 -0.4131 -0.3451 -0.7428 -0.5929
0.0347 -0.4150 -39.5994
74.0400 -41.3500 0.2482 0.1553 -
0.0699 -0.3186 -0.2823 -0.6613 -0.5786
0.0282 -0.4113 -39.5983
74.0600 -41.0800 0.2555 0.1373
0.0092 -0.2097 -0.2162 -0.5800 -0.5640
0.0217 -0.4076 -39.5973
74.0800 -41.7000 -0.5267 0.0241
0.0405 -0.0900 -0.1475 -0.4990 -0.5491
0.0152 -0.4039 -39.5962
74.1000 -40.7500 0.2145 0.0274
0.0219 0.0363 -0.0774 -0.4182 -0.5339
0.0088 -0.4001 -39.5951
74.1200 -40.3600 0.2592 0.1179 -
0.0287 0.1642 -0.0067 -0.3379 -0.5184
0.0023 -0.3964 -39.5941
74.1400 -40.6800 -0.3765 0.1729 -
0.0837 0.2885 0.0636 -0.2581 -0.5027
-0.0041 -0.3927 -39.5930

74.1600 -40.1000 -0.0067 0.1344 -
0.1131 0.4033 0.1325 -0.1789 -0.4867
-0.0106 -0.3890 -39.5919
74.1800 -39.4400 0.4573 -0.0477 -
0.0912 0.5037 0.1991 -0.1004 -0.4705
-0.0170 -0.3853 -39.5909
74.2000 -40.2300 -0.2551 -0.3597 -
0.0090 0.5849 0.2625 -0.0227 -0.4540
-0.0235 -0.3816 -39.5898
74.2200 -40.1800 -0.2908 -0.5417
0.1183 0.6436 0.3216 0.0541 -0.4373
-0.0299 -0.3779 -39.5887
74.2400 -39.2300 0.0948 -0.3508
0.2599 0.6776 0.3755 0.1300 -0.4204
-0.0363 -0.3741 -39.5877
74.2600 -38.7200 -0.2372 0.1720
0.3738 0.6850 0.4236 0.2048 -0.4033
-0.0427 -0.3704 -39.5866
74.2800 -38.0300 -0.0361 0.6155
0.4128 0.6645 0.4650 0.2785 -0.3860
-0.0491 -0.3667 -39.5855
74.3000 -37.3800 0.6236 0.6024
0.3401 0.6164 0.4996 0.3509 -0.3685
-0.0554 -0.3630 -39.5845
74.3200 -39.0800 -0.5034 0.1007
0.1695 0.5444 0.5271 0.4221 -0.3509
-0.0618 -0.3592 -39.5834
74.3400 -39.3700 -0.0892 -0.3531 -
0.0428 0.4542 0.5477 0.4918 -0.3331
-0.0681 -0.3555 -39.5823
74.3600 -39.4200 0.1112 -0.4503 -
0.2335 0.3515 0.5615 0.5600 -0.3152
-0.0745 -0.3518 -39.5813
74.3800 -39.4400 0.1122 -0.3393 -
0.3455 0.2422 0.5686 0.6268 -0.2972
-0.0808 -0.3480 -39.5802
74.4000 -39.4000 -0.0145 -0.1693 -
0.3547 0.1316 0.5694 0.6919 -0.2790
-0.0871 -0.3443 -39.5791
74.4200 -39.2200 -0.0769 0.0292 -
0.2773 0.0242 0.5640 0.7553 -0.2607
-0.0934 -0.3405 -39.5781
74.4400 -38.8500 0.0653 0.1900 -
0.1472 -0.0763 0.5528 0.8171 -0.2424
-0.0996 -0.3368 -39.5770
74.4600 -38.8100 -0.0478 0.2028 -
0.0022 -0.1669 0.5361 0.8771 -0.2239
-0.1059 -0.3331 -39.5759
74.4800 -38.6800 0.1856 0.0702
0.1227 -0.2452 0.5142 0.9354 -0.2054
-0.1121 -0.3293 -39.5749

74.5000 -39.2200 -0.2792 -0.0765
0.2013 -0.3095 0.4877 0.9920 -0.1868
-0.1183 -0.3256 -39.5738
74.5200 -38.8000 0.1265 -0.0525
0.2174 -0.3585 0.4569 1.0467 -0.1682
-0.1245 -0.3218 -39.5727
74.5400 -39.1200 -0.3055 0.1521
0.1712 -0.3917 0.4226 1.0996 -0.1496
-0.1306 -0.3181 -39.5717
74.5600 -38.1400 0.5326 0.2447
0.0747 -0.4095 0.3853 1.1508 -0.1309
-0.1367 -0.3143 -39.5706
74.5800 -39.2800 -0.4741 0.0888 -
0.0464 -0.4132 0.3455 1.2002 -0.1123
-0.1429 -0.3106 -39.5695
74.6000 -39.1000 0.1631 -0.1049 -
0.1592 -0.4043 0.3040 1.2478 -0.0936
-0.1489 -0.3068 -39.5685
74.6200 -38.9600 0.3527 -0.2199 -
0.2268 -0.3852 0.2615 1.2936 -0.0750
-0.1550 -0.3030 -39.5674
74.6400 -39.6500 -0.3611 -0.2221 -
0.2234 -0.3583 0.2184 1.3377 -0.0564
-0.1610 -0.2993 -39.5663
74.6600 -39.1500 -0.1511 -0.0926 -
0.1517 -0.3261 0.1755 1.3801 -0.0378
-0.1671 -0.2955 -39.5653
74.6800 -38.4200 0.3591 0.0747 -
0.0376 -0.2905 0.1331 1.4208 -0.0194
-0.1730 -0.2918 -39.5642
74.7000 -38.5400 0.0048 0.1504
0.0797 -0.2529 0.0915 1.4598 -0.0010
-0.1790 -0.2880 -39.5631
74.7200 -38.6000 -0.2835 0.1496
0.1636 -0.2144 0.0513 1.4972 0.0173
-0.1849 -0.2842 -39.5621
74.7400 -38.1400 0.2386 0.1401
0.1900 -0.1761 0.0126 1.5331 0.0355
-0.1908 -0.2805 -39.5610
74.7600 -38.2800 0.0384 0.0935
0.1546 -0.1390 -0.0242 1.5674 0.0536
-0.1967 -0.2767 -39.5599
74.7800 -38.5000 -0.0467 0.0019
0.0723 -0.1035 -0.0591 1.6002 0.0715
-0.2025 -0.2729 -39.5589
74.8000 -38.6600 -0.1122 -0.0705 -
0.0243 -0.0699 -0.0919 1.6316 0.0893
-0.2083 -0.2692 -39.5578
74.8200 -38.4700 0.2697 -0.1426 -
0.0983 -0.0383 -0.1227 1.6617 0.1069
-0.2141 -0.2654 -39.5567

74.8400 -38.8800 -0.2063 -0.2496 -
0.1248 -0.0090 -0.1514 1.6905 0.1243
-0.2198 -0.2616 -39.5557
74.8600 -38.6300 0.1511 -0.2564 -
0.0985 0.0181 -0.1781 1.7181 0.1415
-0.2255 -0.2578 -39.5546
74.8800 -38.7200 -0.3719 -0.0350 -
0.0358 0.0428 -0.2030 1.7445 0.1585
-0.2312 -0.2541 -39.5535
74.9000 -37.6900 0.2814 0.2570
0.0322 0.0648 -0.2263 1.7698 0.1752
-0.2368 -0.2503 -39.5525
74.9200 -37.6600 0.2273 0.3525
0.0745 0.0835 -0.2482 1.7942 0.1917
-0.2424 -0.2465 -39.5514
74.9400 -37.8900 -0.0137 0.2025
0.0751 0.0984 -0.2689 1.8176 0.2080
-0.2480 -0.2427 -39.5503
74.9600 -38.6100 -0.5075 -0.0358
0.0431 0.1091 -0.2886 1.8402 0.2239
-0.2535 -0.2389 -39.5493
74.9800 -37.7300 0.6243 -0.2389
0.0029 0.1155 -0.3075 1.8620 0.2396
-0.2590 -0.2352 -39.5482
75.0000 -39.0400 -0.5299 -0.2810 -
0.0217 0.1178 -0.3256 1.8831 0.2549
-0.2644 -0.2314 -39.5471
75.0200 -38.5300 -0.3199 -0.1213 -
0.0193 0.1159 -0.3431 1.9036 0.2699
-0.2698 -0.2276 -39.5461
75.0400 -37.3500 0.6456 0.0995
0.0057 0.1099 -0.3601 1.9235 0.2845
-0.2752 -0.2238 -39.5450
75.0600 -38.1100 -0.2060 0.1923
0.0374 0.1001 -0.3767 1.9430 0.2988
-0.2805 -0.2200 -39.5439
75.0800 -38.1600 -0.3193 0.1586
0.0543 0.0868 -0.3930 1.9620 0.3127
-0.2858 -0.2162 -39.5429
75.1000 -37.7400 0.1891 0.0790
0.0427 0.0708 -0.4090 1.9806 0.3262
-0.2910 -0.2125 -39.5418
75.1200 -37.7400 0.4175 -0.0574
0.0074 0.0529 -0.4245 1.9989 0.3393
-0.2962 -0.2087 -39.5407
75.1400 -38.5600 -0.3000 -0.1835 -
0.0359 0.0340 -0.4396 2.0169 0.3520
-0.3014 -0.2049 -39.5397
75.1600 -38.6900 -0.3981 -0.1579 -
0.0719 0.0146 -0.4541 2.0347 0.3643
-0.3065 -0.2011 -39.5386

75.1800 -37.6500 0.5624 -0.0108 -
0.0892 -0.0043 -0.4679 2.0523 0.3761
-0.3115 -0.1973 -39.5375
75.2000 -38.0100 0.0841 0.0771 -
0.0832 -0.0219 -0.4809 2.0697 0.3875
-0.3165 -0.1935 -39.5365
75.2200 -38.6000 -0.5248 0.0877 -
0.0548 -0.0373 -0.4928 2.0868 0.3984
-0.3215 -0.1897 -39.5354
75.2400 -37.6700 0.3973 0.0575 -
0.0115 -0.0499 -0.5036 2.1038 0.4089
-0.3264 -0.1859 -39.5344
75.2600 -37.9000 0.1739 -0.0747
0.0386 -0.0586 -0.5131 2.1206 0.4188
-0.3313 -0.1821 -39.5333
75.2800 -38.4200 -0.2640 -0.2197
0.0847 -0.0626 -0.5213 2.1372 0.4283
-0.3361 -0.1783 -39.5322
75.3000 -38.3500 -0.3141 -0.1209
0.1142 -0.0609 -0.5280 2.1536 0.4372
-0.3409 -0.1745 -39.5312
75.3200 -37.5000 0.3495 0.1531
0.1146 -0.0525 -0.5331 2.1698 0.4457
-0.3456 -0.1707 -39.5301
75.3400 -37.5800 0.1120 0.2582
0.0779 -0.0371 -0.5366 2.1857 0.4536
-0.3503 -0.1669 -39.5290
75.3600 -37.7700 0.0074 0.1048
0.0048 -0.0149 -0.5383 2.2014 0.4611
-0.3549 -0.1631 -39.5280
75.3800 -38.3700 -0.3675 -0.0637 -
0.0883 0.0133 -0.5382 2.2169 0.4680
-0.3595 -0.1593 -39.5269
75.4000 -38.0300 -0.0141 -0.1051 -
0.1754 0.0461 -0.5364 2.2321 0.4744
-0.3640 -0.1555 -39.5258
75.4200 -37.7500 0.4045 -0.1046 -
0.2282 0.0824 -0.5328 2.2469 0.4802
-0.3684 -0.1517 -39.5248
75.4400 -38.5500 -0.4418 -0.1033 -
0.2247 0.1201 -0.5275 2.2614 0.4856
-0.3728 -0.1479 -39.5237
75.4600 -37.5700 0.3139 -0.0479 -
0.1572 0.1571 -0.5204 2.2755 0.4904
-0.3772 -0.1441 -39.5226
75.4800 -37.6000 0.0049 -0.0389 -
0.0370 0.1908 -0.5116 2.2891 0.4947
-0.3814 -0.1403 -39.5216
75.5000 -37.5100 0.0177 -0.0693
0.1070 0.2187 -0.5010 2.3021 0.4985
-0.3857 -0.1365 -39.5205

75.5200	-37.5900	-0.4121	0.0490	
0.2327	0.2377	-0.4885	2.3145	0.5018
	-0.3899	-0.1327	-39.5195	
75.5400	-36.4300	0.5411	0.2204	
0.3051	0.2453	-0.4740	2.3263	0.5046
	-0.3940	-0.1289	-39.5184	
75.5600	-37.3400	-0.4545	0.1868	
0.3030	0.2395	-0.4575	2.3372	0.5068
	-0.3980	-0.1251	-39.5173	
75.5800	-36.8400	0.3139	0.0345	
0.2283	0.2198	-0.4388	2.3473	0.5086
	-0.4020	-0.1213	-39.5163	
75.6000	-37.4500	-0.0896	-0.0713	
0.1055	0.1877	-0.4177	2.3564	0.5098
	-0.4059	-0.1175	-39.5152	
75.6200	-37.5600	-0.1044	-0.0941	-
0.0266	0.1454	-0.3941	2.3643	0.5106
	-0.4098	-0.1137	-39.5141	
75.6400	-37.6000	0.0021	-0.0609	-
0.1321	0.0956	-0.3678	2.3710	0.5108
	-0.4136	-0.1099	-39.5131	
75.6600	-37.4500	0.2710	-0.0636	-
0.1872	0.0412	-0.3386	2.3762	0.5105
	-0.4173	-0.1061	-39.5120	
75.6800	-37.9500	-0.1856	-0.1331	-
0.1861	-0.0153	-0.3063	2.3798	0.5097
	-0.4210	-0.1023	-39.5109	
75.7000	-37.9100	-0.1963	-0.1296	-
0.1399	-0.0716	-0.2709	2.3818	0.5084
	-0.4246	-0.0985	-39.5099	
75.7200	-37.3300	0.1925	0.0281	-
0.0728	-0.1263	-0.2321	2.3818	0.5066
	-0.4282	-0.0947	-39.5088	
75.7400	-37.4100	-0.1352	0.2204	-
0.0101	-0.1786	-0.1898	2.3798	0.5043
	-0.4316	-0.0909	-39.5078	
75.7600	-37.1000	0.2271	0.2490	
0.0324	-0.2285	-0.1438	2.3756	0.5016
	-0.4350	-0.0871	-39.5067	
75.7800	-37.5600	-0.1421	0.0352	
0.0520	-0.2761	-0.0938	2.3690	0.4983
	-0.4384	-0.0833	-39.5056	
75.8000	-37.5600	0.1929	-0.2319	
0.0547	-0.3213	-0.0399	2.3600	0.4945
	-0.4416	-0.0795	-39.5046	
75.8200	-38.1600	-0.4419	-0.2223	
0.0486	-0.3643	0.0182	2.3485	0.4903
	-0.4448	-0.0757	-39.5035	
75.8400	-36.8700	0.4801	0.0809	
0.0389	-0.4054	0.0802	2.3342	0.4856
	-0.4480	-0.0719	-39.5024	

75.8600 -37.4000 -0.1638 0.2754
0.0258 -0.4441 0.1461 2.3172 0.4804
-0.4510 -0.0681 -39.5014
75.8800 -37.0900 0.1332 0.1519
0.0110 -0.4798 0.2154 2.2972 0.4748
-0.4540 -0.0643 -39.5003
75.9000 -37.5600 0.0806 -0.1193 -
0.0012 -0.5117 0.2877 2.2743 0.4687
-0.4569 -0.0604 -39.4992
75.9200 -37.9300 -0.1669 -0.2637 -
0.0063 -0.5385 0.3622 2.2483 0.4622
-0.4597 -0.0566 -39.4982
75.9400 -37.6500 -0.0948 -0.1242 -
0.0050 -0.5588 0.4381 2.2191 0.4552
-0.4625 -0.0528 -39.4971
75.9600 -37.0400 0.1579 0.1663 -
0.0017 -0.5706 0.5145 2.1867 0.4477
-0.4652 -0.0490 -39.4961
75.9800 -36.8900 0.2238 0.2781
0.0031 -0.5716 0.5904 2.1509 0.4399
-0.4678 -0.0452 -39.4950
76.0000 -37.2900 -0.1095 0.0719
0.0128 -0.5593 0.6647 2.1118 0.4317
-0.4704 -0.0414 -39.4939
76.0200 -37.4400 0.1603 -0.2197
0.0315 -0.5312 0.7364 2.0691 0.4230
-0.4728 -0.0376 -39.4929
76.0400 -37.9700 -0.5010 -0.2433
0.0621 -0.4851 0.8045 2.0229 0.4139
-0.4752 -0.0338 -39.4918
76.0600 -36.7000 0.3839 0.0222
0.0981 -0.4193 0.8680 1.9731 0.4045
-0.4776 -0.0300 -39.4907
76.0800 -36.8300 0.0198 0.2140
0.1232 -0.3326 0.9259 1.9196 0.3947
-0.4798 -0.0262 -39.4897
76.1000 -36.5700 0.1120 0.1384
0.1170 -0.2245 0.9772 1.8623 0.3845
-0.4820 -0.0224 -39.4886
76.1200 -36.8500 -0.0086 -0.0475
0.0628 -0.0962 1.0212 1.8013 0.3740
-0.4841 -0.0187 -39.4876
76.1400 -37.1300 -0.2421 -0.1015 -
0.0467 0.0490 1.0569 1.7365 0.3632
-0.4861 -0.0149 -39.4865
76.1600 -36.8700 -0.0404 0.0427 -
0.1989 0.2055 1.0835 1.6680 0.3521
-0.4881 -0.0111 -39.4854
76.1800 -36.5800 0.2698 0.1972 -
0.3572 0.3654 1.1003 1.5958 0.3406
-0.4900 -0.0073 -39.4844

76.2000 -36.8500 0.0079 0.1298 -
0.4789 0.5204 1.1064 1.5200 0.3288
-0.4918 -0.0035 -39.4833
76.2200 -37.1100 0.0047 -0.1479 -
0.5280 0.6624 1.1010 1.4406 0.3168
-0.4935 0.0003 -39.4823
76.2400 -37.2100 0.1280 -0.4244 -
0.4763 0.7846 1.0834 1.3579 0.3045
-0.4951 0.0041 -39.4812
76.2600 -37.6200 -0.4518 -0.4568 -
0.3115 0.8811 1.0529 1.2720 0.2919
-0.4967 0.0079 -39.4801
76.2800 -36.6200 0.0954 -0.1942 -
0.0476 0.9471 1.0092 1.1831 0.2791
-0.4982 0.0117 -39.4791
76.3000 -36.1000 0.1534 0.1420
0.2727 0.9777 0.9522 1.0916 0.2660
-0.4997 0.0155 -39.4780
76.3200 -35.8600 0.1197 0.3072
0.5935 0.9684 0.8826 0.9976 0.2528
-0.5010 0.0193 -39.4770
76.3400 -36.0800 -0.0732 0.2690
0.8491 0.9143 0.8013 0.9015 0.2393
-0.5023 0.0230 -39.4759
76.3600 -36.1100 0.0369 0.1885
0.9730 0.8123 0.7098 0.8035 0.2256
-0.5035 0.0268 -39.4748
76.3800 -36.6300 -0.0994 0.2325
0.9238 0.6639 0.6097 0.7038 0.2118
-0.5046 0.0306 -39.4738
76.4000 -37.2100 -0.2577 0.3970
0.6994 0.4778 0.5027 0.6027 0.1977
-0.5057 0.0344 -39.4727
76.4200 -37.3100 0.5401 0.4079
0.3402 0.2665 0.3904 0.5005 0.1835
-0.5067 0.0382 -39.4716
76.4400 -39.3800 -0.2534 0.0790 -
0.0822 0.0429 0.2745 0.3974 0.1692
-0.5076 0.0419 -39.4706
76.4600 -40.7700 -0.3557 -0.3626 -
0.4856 -0.1797 0.1566 0.2937 0.1547
-0.5084 0.0457 -39.4695
76.4800 -41.1800 0.2113 -0.5883 -
0.7886 -0.3883 0.0384 0.1897 0.1401
-0.5092 0.0495 -39.4685
76.5000 -42.2800 -0.4622 -0.4156 -
0.9302 -0.5701 -0.0784 0.0856 0.1255
-0.5099 0.0532 -39.4674
76.5200 -41.3200 0.3070 0.0139 -
0.9043 -0.7138 -0.1923 -0.0184 0.1107
-0.5105 0.0570 -39.4663

76.5400 -41.4900 0.0355 0.2811 -
0.7453 -0.8127 -0.3015 -0.1219 0.0958
-0.5110 0.0608 -39.4653
76.5600 -41.5200 0.1547 0.2073 -
0.5053 -0.8647 -0.4045 -0.2247 0.0808
-0.5115 0.0645 -39.4642
76.5800 -42.0100 -0.2134 0.0666 -
0.2488 -0.8703 -0.5000 -0.3266 0.0658
-0.5119 0.0683 -39.4632
76.6000 -42.0100 -0.3779 0.0975 -
0.0332 -0.8319 -0.5868 -0.4273 0.0508
-0.5122 0.0721 -39.4621
76.6200 -41.0400 0.5136 0.1555
0.1261 -0.7550 -0.6646 -0.5265 0.0357
-0.5124 0.0758 -39.4610
76.6400 -41.5400 0.1252 0.0326
0.2354 -0.6470 -0.7329 -0.6239 0.0206
-0.5126 0.0796 -39.4600
76.6600 -42.3200 -0.5817 -0.1703
0.3036 -0.5170 -0.7916 -0.7194 0.0055
-0.5127 0.0833 -39.4589
76.6800 -41.7600 0.1133 -0.2317
0.3374 -0.3741 -0.8409 -0.8127 -0.0096
-0.5127 0.0871 -39.4579
76.7000 -41.2900 0.4668 -0.1009
0.3410 -0.2273 -0.8807 -0.9036 -0.0247
-0.5127 0.0908 -39.4568
76.7200 -41.9800 -0.5139 0.1518
0.3160 -0.0849 -0.9114 -0.9918 -0.0398
-0.5126 0.0946 -39.4558
76.7400 -41.3900 -0.1030 0.3284
0.2681 0.0453 -0.9331 -1.0771 -0.0548
-0.5124 0.0983 -39.4547
76.7600 -40.8900 0.5384 0.2147
0.2114 0.1566 -0.9461 -1.1593 -0.0697
-0.5121 0.1021 -39.4536
76.7800 -42.5600 -0.5670 -0.1503
0.1716 0.2442 -0.9510 -1.2382 -0.0846
-0.5118 0.1058 -39.4526
76.8000 -41.5400 0.4412 -0.3641
0.1646 0.3060 -0.9481 -1.3137 -0.0994
-0.5114 0.1095 -39.4515
76.8200 -42.3500 -0.2449 -0.3294
0.1929 0.3422 -0.9384 -1.3854 -0.1141
-0.5109 0.1133 -39.4505
76.8400 -41.6900 0.3579 -0.1788
0.2418 0.3544 -0.9225 -1.4534 -0.1288
-0.5104 0.1170 -39.4494
76.8600 -42.4700 -0.8027 0.1803
0.2724 0.3461 -0.9014 -1.5173 -0.1432
-0.5098 0.1207 -39.4483

76.8800 -40.8800 0.3954 0.5817
0.2510 0.3220 -0.8762 -1.5771 -0.1576
-0.5091 0.1245 -39.4473
76.9000 -40.4600 0.9123 0.6347
0.1629 0.2872 -0.8477 -1.6325 -0.1718
-0.5083 0.1282 -39.4462
76.9200 -42.1400 -0.0854 0.1551
0.0188 0.2468 -0.8171 -1.6836 -0.1859
-0.5075 0.1319 -39.4452
76.9400 -43.4600 -0.5763 -0.4814 -
0.1395 0.2059 -0.7854 -1.7301 -0.1998
-0.5066 0.1356 -39.4441
76.9600 -44.1200 -0.6717 -0.7455 -
0.2634 0.1690 -0.7534 -1.7721 -0.2135
-0.5057 0.1393 -39.4430
76.9800 -43.3500 -0.2518 -0.4252 -
0.3142 0.1401 -0.7222 -1.8093 -0.2270
-0.5046 0.1431 -39.4420
77.0000 -42.0500 0.5007 0.1490 -
0.2913 0.1212 -0.6924 -1.8417 -0.2403
-0.5035 0.1468 -39.4409
77.0200 -42.1900 -0.0481 0.5109 -
0.2082 0.1127 -0.6648 -1.8693 -0.2534
-0.5024 0.1505 -39.4399
77.0400 -42.0300 0.0659 0.4577 -
0.0838 0.1133 -0.6397 -1.8921 -0.2663
-0.5011 0.1542 -39.4388
77.0600 -42.3300 0.0233 0.0825
0.0545 0.1211 -0.6174 -1.9100 -0.2789
-0.4998 0.1579 -39.4378
77.0800 -42.5000 0.1787 -0.3596
0.1791 0.1333 -0.5976 -1.9230 -0.2913
-0.4984 0.1616 -39.4367
77.1000 -42.7400 -0.2044 -0.4759
0.2601 0.1473 -0.5802 -1.9313 -0.3035
-0.4970 0.1653 -39.4356
77.1200 -42.7400 -0.6177 -0.0617
0.2788 0.1614 -0.5647 -1.9349 -0.3154
-0.4955 0.1689 -39.4346
77.1400 -40.8300 0.8355 0.4814
0.2293 0.1741 -0.5509 -1.9339 -0.3271
-0.4939 0.1726 -39.4335
77.1600 -41.7900 -0.0574 0.4629
0.1149 0.1845 -0.5381 -1.9285 -0.3385
-0.4923 0.1763 -39.4325
77.1800 -42.6200 -0.3696 0.0620 -
0.0382 0.1916 -0.5259 -1.9189 -0.3497
-0.4906 0.1800 -39.4314
77.2000 -42.8900 -0.0489 -0.2183 -
0.1875 0.1943 -0.5137 -1.9051 -0.3606
-0.4888 0.1837 -39.4304

77.2200 -43.0900 -0.1990 -0.2498 -
0.2844 0.1914 -0.5011 -1.8874 -0.3713
-0.4869 0.1873 -39.4293
77.2400 -42.4400 0.4139 -0.2470 -
0.2929 0.1822 -0.4874 -1.8660 -0.3817
-0.4850 0.1910 -39.4282
77.2600 -43.0900 -0.3068 -0.2518 -
0.2070 0.1658 -0.4723 -1.8410 -0.3919
-0.4830 0.1947 -39.4272
77.2800 -42.4100 -0.1052 -0.1005 -
0.0593 0.1418 -0.4553 -1.8127 -0.4018
-0.4810 0.1983 -39.4261
77.3000 -41.4800 0.4702 0.1636
0.1001 0.1099 -0.4361 -1.7811 -0.4114
-0.4789 0.2020 -39.4251
77.3200 -42.1700 -0.5683 0.3947
0.2200 0.0702 -0.4143 -1.7466 -0.4209
-0.4767 0.2056 -39.4240
77.3400 -41.1600 0.2832 0.4186
0.2643 0.0229 -0.3898 -1.7093 -0.4300
-0.4745 0.2093 -39.4230
77.3600 -41.1600 0.5841 0.1541
0.2286 -0.0311 -0.3623 -1.6694 -0.4390
-0.4722 0.2129 -39.4219
77.3800 -42.3400 -0.0460 -0.2634
0.1369 -0.0900 -0.3319 -1.6271 -0.4476
-0.4698 0.2166 -39.4209
77.4000 -43.4000 -0.8580 -0.4429
0.0233 -0.1519 -0.2983 -1.5827 -0.4561
-0.4674 0.2202 -39.4198
77.4200 -41.8300 0.5215 -0.1995 -
0.0785 -0.2148 -0.2617 -1.5361 -0.4642
-0.4649 0.2238 -39.4187
77.4400 -41.5300 0.6364 0.0694 -
0.1445 -0.2765 -0.2219 -1.4877 -0.4722
-0.4624 0.2274 -39.4177
77.4600 -42.0200 0.2054 0.0316 -
0.1621 -0.3348 -0.1791 -1.4377 -0.4799
-0.4598 0.2311 -39.4166
77.4800 -42.6200 -0.4077 -0.1626 -
0.1389 -0.3873 -0.1332 -1.3860 -0.4873
-0.4571 0.2347 -39.4156
77.5000 -42.9500 -0.7434 -0.1784 -
0.0911 -0.4320 -0.0845 -1.3330 -0.4945
-0.4543 0.2383 -39.4145
77.5200 -42.0100 -0.1294 0.0758 -
0.0381 -0.4669 -0.0332 -1.2787 -0.5015
-0.4515 0.2419 -39.4135
77.5400 -40.5800 0.9594 0.3274
0.0021 -0.4903 0.0204 -1.2232 -0.5082
-0.4487 0.2455 -39.4124

77.5600 -41.7600 -0.4031 0.3285
0.0147 -0.5010 0.0760 -1.1668 -0.5147
-0.4458 0.2491 -39.4114
77.5800 -42.1600 -0.7645 0.1937 -
0.0055 -0.4979 0.1332 -1.1094 -0.5209
-0.4428 0.2527 -39.4103
77.6000 -40.8100 0.5771 0.0594 -
0.0516 -0.4807 0.1915 -1.0512 -0.5269
-0.4397 0.2563 -39.4093
77.6200 -41.3800 0.2092 -0.1246 -
0.0959 -0.4498 0.2505 -0.9922 -0.5327
-0.4366 0.2599 -39.4082
77.6400 -41.6700 -0.0100 -0.4056 -
0.1109 -0.4057 0.3096 -0.9327 -0.5382
-0.4335 0.2634 -39.4071
77.6600 -41.8700 -0.3565 -0.4899 -
0.0878 -0.3493 0.3682 -0.8728 -0.5435
-0.4302 0.2670 -39.4061
77.6800 -41.2300 -0.3151 -0.1479 -
0.0351 -0.2815 0.4259 -0.8124 -0.5485
-0.4270 0.2706 -39.4050
77.7000 -39.9500 0.3514 0.3704
0.0206 -0.2037 0.4819 -0.7518 -0.5533
-0.4236 0.2742 -39.4040
77.7200 -39.4100 0.2399 0.5879
0.0485 -0.1175 0.5358 -0.6911 -0.5579
-0.4202 0.2777 -39.4029
77.7400 -39.6300 0.1704 0.3133
0.0302 -0.0248 0.5868 -0.6303 -0.5622
-0.4168 0.2813 -39.4019
77.7600 -40.4500 -0.3610 -0.1658 -
0.0192 0.0725 0.6345 -0.5695 -0.5663
-0.4133 0.2848 -39.4008
77.7800 -40.3700 -0.1227 -0.4302 -
0.0737 0.1718 0.6781 -0.5090 -0.5702
-0.4097 0.2884 -39.3998
77.8000 -40.0000 -0.1247 -0.3163 -
0.1083 0.2705 0.7171 -0.4487 -0.5738
-0.4061 0.2919 -39.3987
77.8200 -39.4000 0.1022 -0.0253 -
0.1132 0.3659 0.7508 -0.3888 -0.5772
-0.4025 0.2954 -39.3977
77.8400 -38.8800 0.1603 0.1742 -
0.0880 0.4553 0.7788 -0.3295 -0.5803
-0.3987 0.2989 -39.3966
77.8600 -38.8600 0.0772 0.1706 -
0.0409 0.5359 0.8003 -0.2707 -0.5832
-0.3950 0.3025 -39.3956
77.8800 -39.0800 -0.3190 0.0519
0.0134 0.6051 0.8148 -0.2127 -0.5859
-0.3911 0.3060 -39.3945

77.9000 -38.6000 0.0545 0.0079
0.0652 0.6592 0.8218 -0.1556 -0.5883
-0.3873 0.3095 -39.3934
77.9200 -38.2900 0.2043 0.0088
0.1192 0.6946 0.8210 -0.0993 -0.5905
-0.3833 0.3130 -39.3924
77.9400 -38.8600 -0.3961 -0.0622
0.1879 0.7073 0.8119 -0.0440 -0.5925
-0.3793 0.3165 -39.3913
77.9600 -38.1700 0.2875 -0.1943
0.2704 0.6943 0.7946 0.0102 -0.5942
-0.3753 0.3200 -39.3903
77.9800 -38.3500 0.1111 -0.2073
0.3498 0.6536 0.7692 0.0632 -0.5957
-0.3712 0.3235 -39.3892
78.0000 -38.7600 -0.5203 -0.0160
0.4002 0.5850 0.7364 0.1151 -0.5970
-0.3671 0.3269 -39.3882
78.0200 -37.4900 0.4720 0.2834
0.3900 0.4896 0.6969 0.1657 -0.5980
-0.3629 0.3304 -39.3871
78.0400 -37.9600 0.1335 0.3596
0.2951 0.3714 0.6516 0.2150 -0.5988
-0.3587 0.3339 -39.3861
78.0600 -38.6500 -0.0458 0.1182
0.1231 0.2366 0.6013 0.2630 -0.5994
-0.3544 0.3373 -39.3850
78.0800 -39.4800 -0.1121 -0.1976 -
0.0833 0.0924 0.5469 0.3095 -0.5997
-0.3501 0.3408 -39.3840
78.1000 -40.0100 -0.1218 -0.3025 -
0.2719 -0.0538 0.4891 0.3545 -0.5999
-0.3457 0.3442 -39.3829
78.1200 -40.0700 -0.2890 -0.1679 -
0.3961 -0.1946 0.4289 0.3981 -0.5997
-0.3413 0.3477 -39.3819
78.1400 -39.4900 0.4364 -0.0102 -
0.4358 -0.3229 0.3670 0.4400 -0.5994
-0.3368 0.3511 -39.3808
78.1600 -40.1900 -0.1164 0.0067 -
0.3944 -0.4325 0.3043 0.4804 -0.5988
-0.3323 0.3545 -39.3798
78.1800 -40.0700 -0.1742 -0.0208 -
0.2933 -0.5196 0.2416 0.5192 -0.5980
-0.3277 0.3580 -39.3787
78.2000 -39.7900 0.1241 0.0188 -
0.1674 -0.5819 0.1798 0.5563 -0.5969
-0.3231 0.3614 -39.3777
78.2200 -39.8100 0.0273 0.0673 -
0.0565 -0.6181 0.1196 0.5917 -0.5957
-0.3184 0.3648 -39.3766

78.2400 -39.7300 0.0258 0.0893
0.0070 -0.6277 0.0619 0.6254 -0.5942
-0.3137 0.3682 -39.3756
78.2600 -39.6700 0.0947 0.1122
0.0093 -0.6123 0.0072 0.6573 -0.5924
-0.3090 0.3716 -39.3745
78.2800 -39.9400 -0.2125 0.1398 -
0.0403 -0.5755 -0.0438 0.6874 -0.5905
-0.3042 0.3750 -39.3735
78.3000 -39.9400 -0.0648 0.1143 -
0.1072 -0.5226 -0.0907 0.7156 -0.5883
-0.2994 0.3784 -39.3724
78.3200 -39.5500 0.3621 -0.0629 -
0.1493 -0.4594 -0.1330 0.7420 -0.5859
-0.2945 0.3817 -39.3714
78.3400 -40.1300 -0.0722 -0.3186 -
0.1372 -0.3915 -0.1702 0.7666 -0.5833
-0.2896 0.3851 -39.3703
78.3600 -40.3400 -0.3789 -0.3895 -
0.0601 -0.3242 -0.2019 0.7892 -0.5804
-0.2847 0.3885 -39.3693
78.3800 -39.6000 -0.0392 -0.1443
0.0674 -0.2612 -0.2278 0.8099 -0.5773
-0.2797 0.3918 -39.3682
78.4000 -38.6300 0.4409 0.2098
0.2066 -0.2052 -0.2475 0.8287 -0.5740
-0.2746 0.3951 -39.3672
78.4200 -38.8700 -0.2372 0.3923
0.3131 -0.1586 -0.2608 0.8456 -0.5705
-0.2696 0.3985 -39.3661
78.4400 -38.8200 -0.1760 0.3114
0.3519 -0.1233 -0.2674 0.8607 -0.5667
-0.2645 0.4018 -39.3651
78.4600 -38.4600 0.3664 0.0676
0.3159 -0.1003 -0.2673 0.8738 -0.5628
-0.2593 0.4051 -39.3640
78.4800 -39.5600 -0.3484 -0.1578
0.2244 -0.0888 -0.2606 0.8850 -0.5586
-0.2541 0.4085 -39.3630
78.5000 -39.2400 0.0742 -0.2119
0.1103 -0.0872 -0.2475 0.8944 -0.5542
-0.2489 0.4118 -39.3619
78.5200 -39.1800 0.0906 -0.1565
0.0065 -0.0931 -0.2286 0.9020 -0.5495
-0.2437 0.4151 -39.3609
78.5400 -39.4300 -0.0636 -0.1109 -
0.0657 -0.1036 -0.2043 0.9077 -0.5447
-0.2384 0.4184 -39.3598
78.5600 -39.0800 0.1724 -0.0858 -
0.1037 -0.1148 -0.1755 0.9118 -0.5397
-0.2330 0.4216 -39.3588

78.5800 -39.2900 -0.1313 0.0063 -
0.1206 -0.1228 -0.1428 0.9140 -0.5344
-0.2277 0.4249 -39.3577
78.6000 -39.1900 -0.1471 0.1862 -
0.1352 -0.1240 -0.1070 0.9146 -0.5289
-0.2223 0.4282 -39.3567
78.6200 -38.4900 0.4186 0.2847 -
0.1560 -0.1157 -0.0688 0.9136 -0.5233
-0.2168 0.4314 -39.3556
78.6400 -39.2000 -0.2949 0.1239 -
0.1775 -0.0960 -0.0289 0.9109 -0.5174
-0.2114 0.4347 -39.3546
78.6600 -38.9100 0.2173 -0.1722 -
0.1843 -0.0654 0.0119 0.9067 -0.5113
-0.2058 0.4379 -39.3535
78.6800 -39.0900 0.1037 -0.3523 -
0.1541 -0.0248 0.0530 0.9009 -0.5051
-0.2003 0.4412 -39.3525
78.7000 -39.2800 -0.3908 -0.2458 -
0.0743 0.0234 0.0938 0.8936 -0.4986
-0.1947 0.4444 -39.3514
78.7200 -38.7800 -0.4019 0.1247
0.0367 0.0752 0.1338 0.8849 -0.4919
-0.1891 0.4476 -39.3504
78.7400 -37.1300 0.8113 0.3836
0.1405 0.1266 0.1724 0.8748 -0.4851
-0.1835 0.4508 -39.3493
78.7600 -38.4200 -0.5155 0.2061
0.2021 0.1733 0.2091 0.8633 -0.4781
-0.1778 0.4540 -39.3483
78.7800 -38.5400 -0.4763 -0.0925
0.2178 0.2120 0.2435 0.8505 -0.4709
-0.1721 0.4572 -39.3473
78.8000 -37.9500 0.2280 -0.1992
0.1979 0.2399 0.2749 0.8365 -0.4636
-0.1664 0.4604 -39.3462
78.8200 -37.8600 0.2199 -0.1362
0.1585 0.2557 0.3029 0.8212 -0.4561
-0.1606 0.4636 -39.3452
78.8400 -37.9700 -0.0187 -0.0548
0.1192 0.2595 0.3270 0.8047 -0.4484
-0.1549 0.4667 -39.3441
78.8600 -38.2100 -0.3827 0.0405
0.0872 0.2526 0.3469 0.7870 -0.4406
-0.1490 0.4699 -39.3431
78.8800 -37.6500 0.1867 0.1140
0.0614 0.2374 0.3623 0.7683 -0.4326
-0.1432 0.4731 -39.3420
78.9000 -37.5800 0.3443 0.0602
0.0394 0.2172 0.3731 0.7485 -0.4245
-0.1373 0.4762 -39.3410

78.9200 -38.5900 -0.3315 -0.0935
0.0163 0.1957 0.3790 0.7277 -0.4163
-0.1314 0.4793 -39.3399
78.9400 -38.2500 -0.1019 -0.1502 -
0.0111 0.1767 0.3800 0.7059 -0.4079
-0.1255 0.4824 -39.3389
78.9600 -37.9900 0.3074 -0.0373 -
0.0490 0.1637 0.3759 0.6833 -0.3995
-0.1195 0.4856 -39.3378
78.9800 -38.2200 -0.1432 0.1414 -
0.1044 0.1592 0.3667 0.6598 -0.3909
-0.1135 0.4887 -39.3368
79.0000 -38.1600 -0.1210 0.2071 -
0.1799 0.1643 0.3524 0.6356 -0.3822
-0.1075 0.4918 -39.3357
79.0200 -37.9600 0.3427 0.1038 -
0.2671 0.1782 0.3331 0.6106 -0.3734
-0.1015 0.4948 -39.3347
79.0400 -38.7200 -0.1515 -0.0955 -
0.3438 0.1982 0.3092 0.5851 -0.3645
-0.0954 0.4979 -39.3337
79.0600 -39.2600 -0.4640 -0.1998 -
0.3765 0.2210 0.2810 0.5589 -0.3555
-0.0893 0.5010 -39.3326
79.0800 -38.7500 -0.1236 -0.1146 -
0.3334 0.2429 0.2490 0.5323 -0.3464
-0.0832 0.5040 -39.3316
79.1000 -37.8400 0.6881 -0.0671 -
0.1967 0.2600 0.2137 0.5052 -0.3372
-0.0771 0.5071 -39.3305
79.1200 -39.1600 -0.7175 -0.1954
0.0225 0.2688 0.1757 0.4777 -0.3280
-0.0709 0.5101 -39.3295
79.1400 -37.7400 0.5505 -0.2187
0.2749 0.2664 0.1356 0.4500 -0.3187
-0.0647 0.5132 -39.3284
79.1600 -38.3200 -0.4083 0.0082
0.4894 0.2501 0.0938 0.4220 -0.3094
-0.0585 0.5162 -39.3274
79.1800 -37.3200 0.2230 0.3488
0.6003 0.2176 0.0511 0.3938 -0.3000
-0.0523 0.5192 -39.3263
79.2000 -37.6400 -0.0905 0.5559
0.5596 0.1680 0.0081 0.3656 -0.2905
-0.0460 0.5222 -39.3253
79.2200 -37.6500 0.3636 0.3097
0.3645 0.1044 -0.0346 0.3374 -0.2811
-0.0397 0.5252 -39.3243
79.2400 -39.3700 -0.4039 -0.2129
0.0796 0.0326 -0.0764 0.3092 -0.2715
-0.0334 0.5281 -39.3232

79.2600 -39.6800 0.0294 -0.4677 -
0.2103 -0.0414 -0.1167 0.2811 -0.2620
-0.0271 0.5311 -39.3222
79.2800 -39.6100 0.1241 -0.3457 -
0.4307 -0.1114 -0.1547 0.2532 -0.2524
-0.0207 0.5341 -39.3211
79.3000 -39.9000 -0.0939 -0.1355 -
0.5353 -0.1716 -0.1901 0.2255 -0.2429
-0.0144 0.5370 -39.3201
79.3200 -39.7700 0.0276 -0.0577 -
0.5148 -0.2174 -0.2220 0.1982 -0.2333
-0.0080 0.5400 -39.3190
79.3400 -39.7700 -0.0151 -0.0420 -
0.3992 -0.2471 -0.2501 0.1713 -0.2237
-0.0016 0.5429 -39.3180
79.3600 -39.5400 0.1093 -0.0011 -
0.2309 -0.2614 -0.2739 0.1448 -0.2141
0.0049 0.5458 -39.3170
79.3800 -39.5300 -0.1905 0.0829 -
0.0516 -0.2617 -0.2930 0.1188 -0.2046
0.0113 0.5487 -39.3159
79.4000 -39.0900 0.1163 0.1574
0.1032 -0.2497 -0.3074 0.0934 -0.1950
0.0178 0.5516 -39.3149
79.4200 -39.0400 0.0155 0.1481
0.2133 -0.2278 -0.3170 0.0687 -0.1855
0.0243 0.5545 -39.3138
79.4400 -39.1000 0.0310 0.0520
0.2753 -0.1991 -0.3220 0.0447 -0.1761
0.0308 0.5573 -39.3128
79.4600 -39.1900 -0.0481 -0.0542
0.2926 -0.1668 -0.3223 0.0214 -0.1666
0.0373 0.5602 -39.3117
79.4800 -39.1600 -0.0538 -0.0803
0.2743 -0.1342 -0.3183 -0.0010 -0.1573
0.0438 0.5631 -39.3107
79.5000 -38.9500 0.1466 -0.0100
0.2318 -0.1036 -0.3101 -0.0227 -0.1479
0.0504 0.5659 -39.3097
79.5200 -39.0800 -0.1521 0.0877
0.1685 -0.0763 -0.2980 -0.0434 -0.1387
0.0570 0.5687 -39.3086
79.5400 -38.7100 0.2642 0.1088
0.0903 -0.0531 -0.2824 -0.0633 -0.1295
0.0636 0.5715 -39.3076
79.5600 -39.2900 -0.2056 0.0085
0.0062 -0.0344 -0.2636 -0.0824 -0.1203
0.0702 0.5744 -39.3065
79.5800 -39.1900 0.0379 -0.0932 -
0.0694 -0.0200 -0.2422 -0.1006 -0.1113
0.0768 0.5771 -39.3055

79.6000 -39.4300 -0.1521 -0.0861 -
0.1206 -0.0094 -0.2185 -0.1179 -0.1024
0.0834 0.5799 -39.3044
79.6200 -39.1100 0.0852 -0.0373 -
0.1350 -0.0019 -0.1931 -0.1343 -0.0935
0.0901 0.5827 -39.3034
79.6400 -38.9700 0.2273 -0.0345 -
0.1135 0.0038 -0.1665 -0.1499 -0.0847
0.0968 0.5855 -39.3024
79.6600 -39.2200 -0.1123 -0.0422 -
0.0685 0.0093 -0.1391 -0.1645 -0.0761
0.1035 0.5882 -39.3013
79.6800 -39.2300 -0.3477 0.0102 -
0.0175 0.0161 -0.1114 -0.1784 -0.0675
0.1101 0.5910 -39.3003
79.7000 -38.4300 0.4344 0.0685
0.0209 0.0257 -0.0838 -0.1913 -0.0591
0.1169 0.5937 -39.2992
79.7200 -39.0700 -0.1569 0.0442
0.0327 0.0390 -0.0569 -0.2034 -0.0508
0.1236 0.5964 -39.2982
79.7400 -38.9600 -0.1937 0.0071
0.0152 0.0564 -0.0310 -0.2147 -0.0426
0.1303 0.5991 -39.2972
79.7600 -38.7400 0.1632 0.0159 -
0.0211 0.0772 -0.0065 -0.2251 -0.0345
0.1371 0.6018 -39.2961
79.7800 -38.7400 -0.0497 0.0469 -
0.0581 0.1000 0.0164 -0.2346 -0.0266
0.1438 0.6045 -39.2951
79.8000 -38.7900 -0.0428 0.0251 -
0.0757 0.1233 0.0374 -0.2433 -0.0188
0.1506 0.6071 -39.2940
79.8200 -38.4300 0.2738 -0.0686 -
0.0612 0.1453 0.0562 -0.2512 -0.0111
0.1574 0.6098 -39.2930
79.8400 -38.9100 -0.2883 -0.1432 -
0.0164 0.1641 0.0727 -0.2582 -0.0036
0.1642 0.6124 -39.2920
79.8600 -38.4000 0.0405 -0.0788
0.0486 0.1778 0.0866 -0.2643 0.0038
0.1710 0.6151 -39.2909
79.8800 -38.3300 -0.0327 0.0631
0.1153 0.1852 0.0978 -0.2697 0.0110
0.1778 0.6177 -39.2899
79.9000 -37.7900 0.3552 0.1406
0.1609 0.1851 0.1061 -0.2742 0.0181
0.1846 0.6203 -39.2888
79.9200 -38.6400 -0.5076 0.1492
0.1657 0.1770 0.1116 -0.2779 0.0251
0.1915 0.6229 -39.2878

79.9400 -38.1000 0.0383 0.1067
0.1250 0.1616 0.1142 -0.2808 0.0319
0.1983 0.6255 -39.2868
79.9600 -37.8700 0.5824 -0.0305
0.0515 0.1406 0.1139 -0.2830 0.0385
0.2051 0.6280 -39.2857
79.9800 -39.1500 -0.4455 -0.2167 -
0.0325 0.1160 0.1109 -0.2843 0.0451
0.2120 0.6306 -39.2847
80.0000 -39.0100 -0.2437 -0.2215 -
0.1023 0.0898 0.1055 -0.2850 0.0514
0.2188 0.6331 -39.2837
80.0200 -38.9100 -0.2269 0.0369 -
0.1388 0.0639 0.0977 -0.2849 0.0577
0.2257 0.6357 -39.2826
80.0400 -38.0000 0.5206 0.2353 -
0.1363 0.0398 0.0878 -0.2842 0.0637
0.2326 0.6382 -39.2816
80.0600 -38.6700 -0.2333 0.1191 -
0.0985 0.0187 0.0762 -0.2829 0.0697
0.2394 0.6407 -39.2805
80.0800 -38.7400 -0.0716 -0.1281 -
0.0388 0.0011 0.0631 -0.2810 0.0755
0.2463 0.6432 -39.2795
80.1000 -38.7000 -0.0114 -0.2235
0.0236 -0.0131 0.0491 -0.2785 0.0811
0.2532 0.6457 -39.2785
80.1200 -38.6400 -0.0374 -0.0573
0.0725 -0.0241 0.0345 -0.2756 0.0866
0.2600 0.6481 -39.2774
80.1400 -38.3500 0.0086 0.1792
0.0960 -0.0325 0.0198 -0.2721 0.0920
0.2669 0.6506 -39.2764
80.1600 -37.9500 0.3390 0.2188
0.0852 -0.0393 0.0052 -0.2683 0.0973
0.2738 0.6530 -39.2754
80.1800 -38.6500 -0.1462 0.0394
0.0426 -0.0455 -0.0087 -0.2640 0.1024
0.2806 0.6555 -39.2743
80.2000 -38.8900 -0.2313 -0.1119 -
0.0120 -0.0522 -0.0216 -0.2595 0.1074
0.2875 0.6579 -39.2733
80.2200 -38.8300 -0.0991 -0.1094 -
0.0519 -0.0603 -0.0332 -0.2546 0.1123
0.2944 0.6603 -39.2722
80.2400 -38.3700 0.3607 -0.1087 -
0.0524 -0.0707 -0.0432 -0.2496 0.1171
0.3012 0.6627 -39.2712
80.2600 -38.9100 -0.1650 -0.2088 -
0.0068 -0.0835 -0.0512 -0.2443 0.1217
0.3081 0.6651 -39.2702

80.2800 -38.9300 -0.2428 -0.2397
0.0678 -0.0978 -0.0571 -0.2388 0.1262
0.3149 0.6674 -39.2691
80.3000 -38.1400 0.3718 -0.0442
0.1390 -0.1122 -0.0608 -0.2332 0.1307
0.3217 0.6698 -39.2681
80.3200 -38.4100 -0.3333 0.2821
0.1756 -0.1251 -0.0620 -0.2275 0.1350
0.3286 0.6721 -39.2671
80.3400 -37.8300 0.1672 0.4229
0.1560 -0.1349 -0.0609 -0.2217 0.1392
0.3354 0.6744 -39.2660
80.3600 -37.9000 0.3642 0.2217
0.0786 -0.1406 -0.0574 -0.2158 0.1433
0.3422 0.6767 -39.2650
80.3800 -39.0000 -0.4318 -0.1515 -
0.0322 -0.1408 -0.0518 -0.2099 0.1473
0.3490 0.6790 -39.2640
80.4000 -38.9100 -0.0158 -0.3439 -
0.1422 -0.1347 -0.0442 -0.2040 0.1513
0.3558 0.6813 -39.2629
80.4200 -38.5900 0.2683 -0.2588 -
0.2202 -0.1211 -0.0349 -0.1980 0.1551
0.3625 0.6836 -39.2619
80.4400 -38.7700 -0.1290 -0.0190 -
0.2489 -0.0997 -0.0243 -0.1920 0.1589
0.3693 0.6858 -39.2609
80.4600 -38.3400 0.0189 0.1567 -
0.2269 -0.0706 -0.0126 -0.1860 0.1626
0.3760 0.6881 -39.2598
80.4800 -38.2300 0.0701 0.1406 -
0.1596 -0.0352 -0.0002 -0.1799 0.1662
0.3827 0.6903 -39.2588
80.5000 -38.0300 0.2149 -0.0326 -
0.0600 0.0036 0.0126 -0.1739 0.1697
0.3895 0.6925 -39.2578
80.5200 -38.6600 -0.4730 -0.1459
0.0498 0.0431 0.0255 -0.1679 0.1732
0.3961 0.6947 -39.2567
80.5400 -37.6200 0.3110 -0.0240
0.1437 0.0804 0.0380 -0.1618 0.1766
0.4028 0.6969 -39.2557
80.5600 -37.5700 0.0857 0.1464
0.2034 0.1127 0.0499 -0.1558 0.1799
0.4095 0.6991 -39.2546
80.5800 -37.8200 -0.3084 0.1797
0.2186 0.1377 0.0608 -0.1497 0.1832
0.4161 0.7012 -39.2536
80.6000 -37.3400 0.3055 0.0700
0.1871 0.1541 0.0703 -0.1437 0.1864
0.4227 0.7034 -39.2526

80.6200 -37.9700 -0.1863 -0.0818
0.1251 0.1612 0.0783 -0.1376 0.1896
0.4293 0.7055 -39.2515
80.6400 -37.9200 0.0595 -0.1364
0.0561 0.1593 0.0843 -0.1316 0.1927
0.4359 0.7076 -39.2505
80.6600 -37.8900 -0.0040 -0.1028
0.0023 0.1494 0.0884 -0.1256 0.1957
0.4424 0.7097 -39.2495
80.6800 -37.6700 0.1101 -0.0306 -
0.0300 0.1330 0.0905 -0.1196 0.1987
0.4489 0.7118 -39.2484
80.7000 -37.9400 -0.1826 0.0770 -
0.0457 0.1116 0.0905 -0.1136 0.2017
0.4554 0.7138 -39.2474
80.7200 -37.9000 -0.2751 0.1821 -
0.0543 0.0873 0.0884 -0.1076 0.2046
0.4619 0.7159 -39.2464
80.7400 -37.1200 0.5721 0.1524 -
0.0654 0.0622 0.0844 -0.1016 0.2075
0.4683 0.7179 -39.2454
80.7600 -38.5600 -0.5152 -0.0616 -
0.0795 0.0382 0.0786 -0.0956 0.2103
0.4747 0.7200 -39.2443
80.7800 -38.0700 0.0054 -0.1851 -
0.0864 0.0163 0.0710 -0.0896 0.2131
0.4811 0.7220 -39.2433
80.8000 -37.8000 0.2219 -0.1171 -
0.0759 -0.0026 0.0620 -0.0837 0.2159
0.4874 0.7240 -39.2423
80.8200 -38.0000 -0.0911 0.0212 -
0.0446 -0.0185 0.0516 -0.0777 0.2186
0.4937 0.7259 -39.2412
80.8400 -37.8800 -0.0962 0.0809
0.0005 -0.0310 0.0402 -0.0718 0.2213
0.5000 0.7279 -39.2402
80.8600 -37.5700 0.1082 0.0785
0.0439 -0.0400 0.0279 -0.0659 0.2240
0.5063 0.7299 -39.2392
80.8800 -37.7300 -0.1382 0.0834
0.0690 -0.0457 0.0150 -0.0600 0.2267
0.5125 0.7318 -39.2381
80.9000 -37.6400 0.0164 0.0980
0.0663 -0.0487 0.0018 -0.0541 0.2293
0.5187 0.7337 -39.2371
80.9200 -37.6000 0.1596 0.0337
0.0408 -0.0499 -0.0114 -0.0483 0.2319
0.5248 0.7356 -39.2361
80.9400 -38.1000 -0.2205 -0.0938
0.0096 -0.0496 -0.0244 -0.0424 0.2346
0.5309 0.7375 -39.2350

80.9600 -37.9500 0.0544 -0.1704 -
0.0083 -0.0482 -0.0369 -0.0366 0.2372
0.5369 0.7394 -39.2340
80.9800 -37.8600 0.1136 -0.1821
0.0005 -0.0455 -0.0486 -0.0307 0.2398
0.5430 0.7412 -39.2330
81.0000 -37.7900 0.1037 -0.1497
0.0339 -0.0415 -0.0593 -0.0249 0.2424
0.5489 0.7431 -39.2319
81.0200 -37.9400 -0.2845 -0.0341
0.0771 -0.0363 -0.0688 -0.0192 0.2450
0.5549 0.7449 -39.2309
81.0400 -37.7300 -0.2305 0.1724
0.1068 -0.0302 -0.0770 -0.0134 0.2476
0.5608 0.7467 -39.2299
81.0600 -36.9800 0.4707 0.3132
0.1024 -0.0231 -0.0838 -0.0077 0.2503
0.5666 0.7485 -39.2289
81.0800 -37.5100 0.0380 0.1974
0.0563 -0.0154 -0.0891 -0.0020 0.2529
0.5724 0.7503 -39.2278
81.1000 -38.2000 -0.4438 -0.0728 -
0.0178 -0.0070 -0.0928 0.0036 0.2555
0.5782 0.7520 -39.2268
81.1200 -38.0000 0.1688 -0.2551 -
0.0960 0.0022 -0.0948 0.0092 0.2582
0.5839 0.7538 -39.2258
81.1400 -38.0100 0.1073 -0.2210 -
0.1539 0.0117 -0.0953 0.0147 0.2609
0.5895 0.7555 -39.2247
81.1600 -38.0100 -0.1674 -0.0437 -
0.1741 0.0210 -0.0941 0.0201 0.2635
0.5951 0.7572 -39.2237
81.1800 -37.4000 0.2534 0.1048 -
0.1495 0.0294 -0.0913 0.0254 0.2662
0.6007 0.7589 -39.2227
81.2000 -37.3200 0.2265 0.0838 -
0.0840 0.0360 -0.0871 0.0305 0.2690
0.6062 0.7606 -39.2217
81.2200 -37.8700 -0.3020 -0.0539
0.0055 0.0403 -0.0813 0.0356 0.2717
0.6117 0.7623 -39.2206
81.2400 -37.3100 0.2761 -0.1258
0.0963 0.0416 -0.0743 0.0405 0.2745
0.6171 0.7639 -39.2196
81.2600 -37.8900 -0.4009 -0.0237
0.1654 0.0393 -0.0659 0.0452 0.2774
0.6224 0.7656 -39.2186
81.2800 -37.2000 0.0357 0.1372
0.1956 0.0330 -0.0565 0.0497 0.2802
0.6277 0.7672 -39.2175

81.3000 -36.5500 0.5662 0.1973
0.1783 0.0226 -0.0460 0.0540 0.2831
0.6330 0.7688 -39.2165
81.3200 -37.1200 0.1293 0.1064
0.1158 0.0088 -0.0346 0.0580 0.2860
0.6381 0.7704 -39.2155
81.3400 -38.0200 -0.7346 0.0189
0.0239 -0.0071 -0.0225 0.0618 0.2890
0.6433 0.7719 -39.2145
81.3600 -36.9500 0.5350 0.0066 -
0.0743 -0.0235 -0.0097 0.0653 0.2920
0.6483 0.7735 -39.2134
81.3800 -37.4200 0.2081 -0.0848 -
0.1520 -0.0383 0.0035 0.0685 0.2951
0.6533 0.7750 -39.2124
81.4000 -38.3500 -0.4839 -0.2236 -
0.1875 -0.0498 0.0170 0.0713 0.2982
0.6583 0.7765 -39.2114
81.4200 -37.8000 -0.0259 -0.1825 -
0.1726 -0.0567 0.0306 0.0738 0.3014
0.6632 0.7780 -39.2104
81.4400 -37.5900 -0.1388 0.0525 -
0.1164 -0.0584 0.0443 0.0759 0.3046
0.6680 0.7795 -39.2093
81.4600 -36.8000 0.3733 0.2215 -
0.0408 -0.0549 0.0580 0.0776 0.3079
0.6727 0.7810 -39.2083
81.4800 -37.0100 0.1303 0.1394
0.0305 -0.0468 0.0713 0.0789 0.3112
0.6774 0.7824 -39.2073
81.5000 -37.4800 -0.3060 -0.0648
0.0803 -0.0346 0.0843 0.0798 0.3146
0.6821 0.7839 -39.2062
81.5200 -37.4000 -0.1328 -0.1263
0.0974 -0.0193 0.0968 0.0802 0.3181
0.6866 0.7853 -39.2052
81.5400 -37.3000 -0.1606 0.0263
0.0812 -0.0019 0.1085 0.0801 0.3216
0.6911 0.7867 -39.2042
81.5600 -36.7200 0.3223 0.1835
0.0453 0.0163 0.1195 0.0795 0.3252
0.6955 0.7881 -39.2032
81.5800 -37.2100 -0.1251 0.1212
0.0064 0.0341 0.1295 0.0784 0.3288
0.6999 0.7894 -39.2021
81.6000 -37.4300 -0.0978 -0.0901 -
0.0211 0.0506 0.1385 0.0768 0.3326
0.7042 0.7908 -39.2011
81.6200 -37.2800 0.0441 -0.2138 -
0.0297 0.0647 0.1462 0.0746 0.3364
0.7084 0.7921 -39.2001

81.6400	-37.4400	-0.2400	-0.1476	-
0.0169	0.0757	0.1526	0.0719	0.3402
	0.7126	0.7934	-39.1991	
81.6600	-36.8500	0.1939	0.0156	
0.0092	0.0829	0.1574	0.0687	0.3442
	0.7167	0.7947	-39.1980	
81.6800	-36.8500	0.0712	0.1652	
0.0357	0.0855	0.1607	0.0649	0.3482
	0.7207	0.7960	-39.1970	
81.7000	-37.1400	-0.3720	0.2271	
0.0499	0.0832	0.1624	0.0605	0.3523
	0.7246	0.7973	-39.1960	
81.7200	-36.1700	0.6386	0.1434	
0.0458	0.0760	0.1624	0.0556	0.3565
	0.7285	0.7985	-39.1950	
81.7400	-37.0800	-0.0373	-0.0766	
0.0320	0.0642	0.1609	0.0502	0.3607
	0.7322	0.7997	-39.1940	
81.7600	-37.8500	-0.5670	-0.2469	
0.0196	0.0483	0.1578	0.0442	0.3651
	0.7359	0.8009	-39.1929	
81.7800	-37.1500	0.2411	-0.1986	
0.0163	0.0293	0.1533	0.0377	0.3695
	0.7396	0.8021	-39.1919	
81.8000	-36.9100	0.1761	-0.0300	
0.0229	0.0084	0.1474	0.0308	0.3740
	0.7431	0.8033	-39.1909	
81.8200	-37.1400	-0.0988	0.0890	
0.0325	-0.0127	0.1403	0.0234	0.3785
	0.7466	0.8044	-39.1899	
81.8400	-36.8200	0.1436	0.1164	
0.0337	-0.0321	0.1319	0.0156	0.3832
	0.7500	0.8056	-39.1888	
81.8600	-37.0300	0.0254	0.0958	
0.0168	-0.0481	0.1224	0.0073	0.3879
	0.7533	0.8067	-39.1878	
81.8800	-37.3600	-0.2694	0.0664	-
0.0201	-0.0590	0.1120	-0.0013	0.3927
	0.7566	0.8078	-39.1868	
81.9000	-37.0100	0.2956	0.0174	-
0.0686	-0.0640	0.1006	-0.0103	0.3976
	0.7597	0.8089	-39.1858	
81.9200	-37.4000	0.0159	-0.0889	-
0.1134	-0.0626	0.0883	-0.0196	0.4026
	0.7628	0.8099	-39.1847	
81.9400	-37.8800	-0.3391	-0.1372	-
0.1371	-0.0551	0.0754	-0.0292	0.4076
	0.7658	0.8110	-39.1837	
81.9600	-37.4500	-0.0913	-0.0558	-
0.1284	-0.0418	0.0618	-0.0390	0.4127
	0.7688	0.8120	-39.1827	

81.9800 -36.7500 0.6229 -0.0178 -
0.0893 -0.0239 0.0478 -0.0490 0.4179
0.7716 0.8130 -39.1817
82.0000 -37.9100 -0.6447 -0.0624 -
0.0324 -0.0029 0.0335 -0.0593 0.4232
0.7743 0.8140 -39.1807
82.0200 -36.9700 0.1062 0.0250
0.0220 0.0194 0.0192 -0.0696 0.4286
0.7770 0.8150 -39.1796
82.0400 -36.6300 0.3580 0.1954
0.0535 0.0414 0.0048 -0.0801 0.4340
0.7796 0.8159 -39.1786
82.0600 -36.8800 0.0078 0.2287
0.0544 0.0613 -0.0094 -0.0906 0.4396
0.7821 0.8169 -39.1776
82.0800 -37.1400 -0.1439 0.0969
0.0311 0.0776 -0.0232 -0.1012 0.4452
0.7845 0.8178 -39.1766
82.1000 -37.2500 -0.0211 -0.0700
0.0019 0.0889 -0.0367 -0.1117 0.4508
0.7869 0.8187 -39.1756
82.1200 -37.3000 0.0190 -0.1785 -
0.0142 0.0937 -0.0495 -0.1222 0.4566
0.7891 0.8195 -39.1745
82.1400 -37.2000 0.2250 -0.2345 -
0.0036 0.0912 -0.0617 -0.1326 0.4624
0.7913 0.8204 -39.1735
82.1600 -37.5700 -0.2509 -0.1856
0.0368 0.0811 -0.0729 -0.1429 0.4683
0.7934 0.8212 -39.1725
82.1800 -37.2800 -0.1898 0.0217
0.0940 0.0638 -0.0832 -0.1531 0.4743
0.7954 0.8221 -39.1715
82.2000 -36.6400 0.3292 0.2388
0.1463 0.0401 -0.0923 -0.1630 0.4804
0.7973 0.8229 -39.1705
82.2200 -37.0100 -0.0300 0.2508
0.1715 0.0113 -0.1002 -0.1727 0.4865
0.7991 0.8236 -39.1694
82.2400 -37.0000 0.1489 0.0579
0.1602 -0.0209 -0.1068 -0.1821 0.4928
0.8008 0.8244 -39.1684
82.2600 -37.7000 -0.3390 -0.1309
0.1178 -0.0543 -0.1120 -0.1913 0.4990
0.8025 0.8251 -39.1674
82.2800 -37.3400 0.1885 -0.1555
0.0532 -0.0864 -0.1157 -0.2001 0.5054
0.8040 0.8259 -39.1664
82.3000 -37.4300 0.0817 -0.0879 -
0.0197 -0.1150 -0.1179 -0.2086 0.5119
0.8055 0.8266 -39.1654

82.3200	−37.8600	−0.1133	−0.0115	−
0.0891	−0.1377	−0.1185	−0.2166	0.5184
	0.8069	0.8273	−39.1643	
82.3400	−37.7300	−0.0752	0.0750	−
0.1463	−0.1524	−0.1175	−0.2243	0.5250
	0.8082	0.8279	−39.1633	
82.3600	−37.6400	−0.1292	0.1082	−
0.1834	−0.1571	−0.1149	−0.2316	0.5316
	0.8094	0.8286	−39.1623	
82.3800	−37.2600	0.3560	0.0102	−
0.1920	−0.1512	−0.1109	−0.2384	0.5383
	0.8105	0.8292	−39.1613	
82.4000	−37.9200	−0.1325	−0.1408	−
0.1651	−0.1350	−0.1053	−0.2448	0.5451
	0.8115	0.8298	−39.1603	
82.4200	−37.9400	−0.3127	−0.1569	−
0.1019	−0.1100	−0.0985	−0.2507	0.5520
	0.8124	0.8304	−39.1592	
82.4400	−37.1700	0.3010	−0.0441	−
0.0129	−0.0782	−0.0906	−0.2561	0.5590
	0.8133	0.8310	−39.1582	
82.4600	−37.1500	0.0786	0.0235	
0.0794	−0.0415	−0.0817	−0.2611	0.5660
	0.8140	0.8315	−39.1572	
82.4800	−37.1900	−0.1153	0.0459	
0.1490	−0.0025	−0.0719	−0.2656	0.5730
	0.8147	0.8320	−39.1562	
82.5000	−36.8700	0.0566	0.1169	
0.1751	0.0366	−0.0614	−0.2696	0.5802
	0.8153	0.8326	−39.1552	
82.5200	−36.8700	0.0344	0.1661	
0.1522	0.0738	−0.0504	−0.2730	0.5874
	0.8157	0.8330	−39.1542	
82.5400	−36.8100	0.1576	0.0808	
0.0923	0.1070	−0.0391	−0.2761	0.5946
	0.8161	0.8335	−39.1531	
82.5600	−37.2900	−0.1446	−0.0877	
0.0221	0.1346	−0.0276	−0.2786	0.6020
	0.8164	0.8340	−39.1521	
82.5800	−37.1100	0.0703	−0.1537	−
0.0339	0.1552	−0.0160	−0.2806	0.6093
	0.8167	0.8344	−39.1511	
82.6000	−37.4200	−0.3698	−0.0385	−
0.0637	0.1676	−0.0046	−0.2822	0.6168
	0.8168	0.8348	−39.1501	
82.6200	−36.4200	0.4643	0.0833	−
0.0658	0.1712	0.0065	−0.2833	0.6242
	0.8168	0.8352	−39.1491	
82.6400	−36.9700	−0.0475	0.0530	−
0.0439	0.1660	0.0173	−0.2839	0.6318
	0.8168	0.8356	−39.1481	

82.6600 -37.3900 -0.4030 -0.0357 -
0.0078 0.1519 0.0276 -0.2841 0.6393
0.8166 0.8359 -39.1470
82.6800 -36.5600 0.3599 -0.0639
0.0325 0.1292 0.0373 -0.2838 0.6470
0.8164 0.8362 -39.1460
82.7000 -36.9000 -0.0278 -0.0412
0.0695 0.0985 0.0463 -0.2831 0.6546
0.8160 0.8365 -39.1450
82.7200 -37.0500 -0.1473 0.0031
0.0977 0.0609 0.0546 -0.2819 0.6623
0.8156 0.8368 -39.1440
82.7400 -37.0400 -0.1355 0.0899
0.1140 0.0178 0.0622 -0.2803 0.6700
0.8151 0.8371 -39.1430
82.7600 -36.4900 0.3313 0.1142
0.1175 -0.0289 0.0688 -0.2784 0.6778
0.8145 0.8374 -39.1420
82.7800 -37.2300 -0.2740 0.0109
0.1089 -0.0768 0.0746 -0.2760 0.6855
0.8138 0.8376 -39.1410
82.8000 -36.9700 0.1974 -0.1062
0.0861 -0.1228 0.0795 -0.2733 0.6933
0.8130 0.8378 -39.1399
82.8200 -37.2300 -0.0971 -0.1263
0.0478 -0.1635 0.0833 -0.2702 0.7011
0.8121 0.8380 -39.1389
82.8400 -37.0400 0.1603 -0.0669 -
0.0015 -0.1960 0.0862 -0.2667 0.7090
0.8112 0.8381 -39.1379
82.8600 -37.2500 -0.0814 0.0067 -
0.0590 -0.2176 0.0880 -0.2629 0.7168
0.8101 0.8383 -39.1369
82.8800 -37.3000 -0.1104 0.1044 -
0.1247 -0.2257 0.0888 -0.2588 0.7246
0.8089 0.8384 -39.1359
82.9000 -37.2500 -0.0069 0.1875 -
0.1955 -0.2182 0.0887 -0.2543 0.7324
0.8077 0.8385 -39.1349
82.9200 -37.0600 0.1602 0.1649 -
0.2618 -0.1941 0.0876 -0.2496 0.7403
0.8063 0.8386 -39.1339
82.9400 -37.3900 -0.0662 0.0050 -
0.3050 -0.1539 0.0856 -0.2445 0.7481
0.8049 0.8387 -39.1329
82.9600 -37.5200 0.0109 -0.2084 -
0.3027 -0.0996 0.0827 -0.2392 0.7558
0.8034 0.8387 -39.1318
82.9800 -37.2600 0.1402 -0.3090 -
0.2381 -0.0351 0.0790 -0.2336 0.7636
0.8018 0.8388 -39.1308

83.0000 -37.4500 -0.4142 -0.1532 -
0.1127 0.0349 0.0744 -0.2277 0.7713
0.8001 0.8388 -39.1298
83.0200 -36.2300 0.3001 0.1428
0.0489 0.1053 0.0689 -0.2216 0.7790
0.7983 0.8388 -39.1288
83.0400 -36.0700 0.1698 0.2614
0.2090 0.1705 0.0626 -0.2152 0.7867
0.7964 0.8387 -39.1278
83.0600 -36.4500 -0.1807 0.1381
0.3300 0.2254 0.0553 -0.2085 0.7943
0.7944 0.8387 -39.1268
83.0800 -36.1500 0.0603 0.0057
0.3866 0.2649 0.0473 -0.2016 0.8018
0.7923 0.8386 -39.1258
83.1000 -36.2900 -0.1101 0.0061
0.3721 0.2855 0.0383 -0.1944 0.8093
0.7902 0.8385 -39.1248
83.1200 -36.0200 0.1973 0.0351
0.2965 0.2859 0.0286 -0.1869 0.8168
0.7879 0.8384 -39.1237
83.1400 -36.5200 -0.0648 0.0006
0.1816 0.2676 0.0183 -0.1792 0.8241
0.7856 0.8383 -39.1227
83.1600 -36.7300 -0.2278 -0.0082
0.0519 0.2332 0.0075 -0.1713 0.8314
0.7831 0.8382 -39.1217
83.1800 -36.4600 0.2473 0.0523 -
0.0688 0.1861 -0.0034 -0.1631 0.8386
0.7806 0.8380 -39.1207
83.2000 -36.7600 0.0153 0.0485 -
0.1579 0.1294 -0.0144 -0.1546 0.8456
0.7780 0.8378 -39.1197
83.2200 -37.1400 -0.0930 -0.0735 -
0.2008 0.0665 -0.0250 -0.1459 0.8526
0.7752 0.8376 -39.1187
83.2400 -37.1000 0.1019 -0.1940 -
0.1937 0.0014 -0.0351 -0.1370 0.8595
0.7724 0.8374 -39.1177
83.2600 -37.2100 0.0028 -0.1808 -
0.1440 -0.0621 -0.0445 -0.1278 0.8663
0.7695 0.8371 -39.1167
83.2800 -37.3100 -0.3128 -0.0163 -
0.0727 -0.1201 -0.0529 -0.1184 0.8729
0.7666 0.8369 -39.1157
83.3000 -36.5400 0.3851 0.1626 -
0.0089 -0.1693 -0.0600 -0.1088 0.8794
0.7635 0.8366 -39.1147
83.3200 -37.0300 -0.2411 0.2016
0.0221 -0.2069 -0.0657 -0.0989 0.8858
0.7603 0.8363 -39.1136

83.3400 -36.7400 0.1886 0.0906
0.0108 -0.2312 -0.0697 -0.0888 0.8920
0.7570 0.8360 -39.1126
83.3600 -37.2100 -0.1433 -0.0676 -
0.0324 -0.2417 -0.0720 -0.0784 0.8981
0.7537 0.8357 -39.1116
83.3800 -37.2800 -0.1781 -0.1365 -
0.0868 -0.2385 -0.0725 -0.0678 0.9040
0.7503 0.8353 -39.1106
83.4000 -36.9400 0.2018 -0.0730 -
0.1303 -0.2220 -0.0711 -0.0569 0.9097
0.7467 0.8349 -39.1096
83.4200 -36.8800 0.1491 -0.0042 -
0.1479 -0.1935 -0.0681 -0.0458 0.9152
0.7431 0.8346 -39.1086
83.4400 -37.1800 -0.2289 0.0179 -
0.1347 -0.1544 -0.0636 -0.0344 0.9206
0.7394 0.8341 -39.1076
83.4600 -36.8200 -0.0048 0.0446 -
0.0966 -0.1071 -0.0579 -0.0227 0.9257
0.7356 0.8337 -39.1066
83.4800 -36.6000 0.1639 0.0137 -
0.0413 -0.0542 -0.0512 -0.0106 0.9307
0.7317 0.8333 -39.1056
83.5000 -36.8400 -0.2091 -0.0536
0.0208 0.0015 -0.0437 0.0017 0.9354
0.7277 0.8328 -39.1046
83.5200 -36.3400 0.2403 -0.0448
0.0780 0.0570 -0.0357 0.0144 0.9400
0.7237 0.8323 -39.1036
83.5400 -36.5100 -0.1637 0.0372
0.1198 0.1092 -0.0276 0.0274 0.9443
0.7195 0.8318 -39.1026
83.5600 -36.1500 0.0932 0.1055
0.1367 0.1552 -0.0195 0.0407 0.9483
0.7153 0.8313 -39.1015
83.5800 -36.1100 0.0616 0.1163
0.1260 0.1924 -0.0119 0.0544 0.9521
0.7110 0.8308 -39.1005
83.6000 -36.1800 -0.0419 0.0725
0.0944 0.2192 -0.0048 0.0685 0.9557
0.7065 0.8302 -39.0995
83.6200 -36.2000 0.0232 -0.0273
0.0591 0.2344 0.0012 0.0830 0.9590
0.7020 0.8297 -39.0985
83.6400 -36.4500 0.0382 -0.1418
0.0376 0.2380 0.0061 0.0978 0.9620
0.6974 0.8291 -39.0975
83.6600 -36.4700 -0.0391 -0.2128
0.0423 0.2308 0.0096 0.1129 0.9648
0.6928 0.8285 -39.0965

83.6800 -36.2800 0.0710 -0.1538
0.0729 0.2141 0.0115 0.1285 0.9673
0.6880 0.8279 -39.0955
83.7000 -36.3800 -0.2819 0.0461
0.1132 0.1901 0.0117 0.1444 0.9695
0.6832 0.8272 -39.0945
83.7200 -35.4000 0.4665 0.2246
0.1402 0.1615 0.0099 0.1606 0.9713
0.6782 0.8266 -39.0935
83.7400 -36.1600 -0.2072 0.2015
0.1307 0.1308 0.0063 0.1771 0.9729
0.6732 0.8259 -39.0925
83.7600 -36.2400 0.0074 0.0480
0.0745 0.1002 0.0007 0.1939 0.9742
0.6681 0.8252 -39.0915
83.7800 -36.3400 0.1172 -0.0906 -
0.0186 0.0722 -0.0067 0.2109 0.9751
0.6629 0.8245 -39.0905
83.8000 -36.8700 -0.1684 -0.1467 -
0.1238 0.0488 -0.0160 0.2280 0.9758
0.6577 0.8238 -39.0895
83.8200 -36.6400 0.1183 -0.1063 -
0.2131 0.0316 -0.0269 0.2452 0.9760
0.6523 0.8230 -39.0885
83.8400 -36.8300 -0.1588 -0.0158 -
0.2640 0.0221 -0.0392 0.2624 0.9760
0.6469 0.8223 -39.0875
83.8600 -36.4800 0.2489 0.0137 -
0.2641 0.0206 -0.0526 0.2797 0.9756
0.6414 0.8215 -39.0865
83.8800 -36.9900 -0.2657 -0.0599 -
0.2151 0.0265 -0.0666 0.2968 0.9748
0.6358 0.8207 -39.0855
83.9000 -36.3500 0.2715 -0.1419 -
0.1299 0.0379 -0.0808 0.3137 0.9737
0.6301 0.8199 -39.0845
83.9200 -36.8900 -0.4121 -0.1002 -
0.0261 0.0523 -0.0945 0.3305 0.9722
0.6244 0.8191 -39.0835
83.9400 -36.3000 -0.0277 0.0527
0.0745 0.0671 -0.1073 0.3470 0.9703
0.6186 0.8182 -39.0825
83.9600 -35.4400 0.5892 0.1689
0.1502 0.0792 -0.1186 0.3631 0.9681
0.6126 0.8174 -39.0815
83.9800 -36.5900 -0.5683 0.1544
0.1855 0.0859 -0.1279 0.3788 0.9654
0.6067 0.8165 -39.0804
84.0000 -35.8700 0.1630 0.1113
0.1783 0.0847 -0.1347 0.3940 0.9623
0.6006 0.8156 -39.0794

84.0200 -35.9600 0.1532 0.0609
0.1399 0.0738 -0.1387 0.4087 0.9589
0.5945 0.8147 -39.0784
84.0400 -36.1200 0.1993 -0.0787
0.0874 0.0527 -0.1396 0.4228 0.9550
0.5883 0.8138 -39.0774
84.0600 -36.8900 -0.3732 -0.2055
0.0383 0.0215 -0.1372 0.4363 0.9507
0.5820 0.8128 -39.0764
84.0800 -36.5300 0.0196 -0.1555
0.0025 -0.0185 -0.1315 0.4491 0.9459
0.5757 0.8119 -39.0754
84.1000 -36.3300 0.1153 0.0318 -
0.0191 -0.0652 -0.1223 0.4611 0.9408
0.5693 0.8109 -39.0744
84.1200 -36.3500 0.0774 0.1803 -
0.0342 -0.1161 -0.1099 0.4723 0.9351
0.5628 0.8099 -39.0734
84.1400 -36.4300 0.1078 0.1443 -
0.0504 -0.1686 -0.0943 0.4826 0.9291
0.5563 0.8089 -39.0724
84.1600 -36.4300 0.1387 -0.0122 -
0.0683 -0.2200 -0.0757 0.4920 0.9226
0.5497 0.8079 -39.0714
84.1800 -37.2900 -0.4754 -0.0729 -
0.0837 -0.2675 -0.0544 0.5006 0.9156
0.5430 0.8068 -39.0704
84.2000 -36.3900 0.4313 -0.0175 -
0.0912 -0.3087 -0.0304 0.5081 0.9081
0.5363 0.8058 -39.0694
84.2200 -36.8100 -0.0190 -0.0100 -
0.0883 -0.3414 -0.0041 0.5146 0.9002
0.5295 0.8047 -39.0684
84.2400 -37.1400 -0.2626 -0.0457 -
0.0788 -0.3635 0.0242 0.5201 0.8918
0.5226 0.8036 -39.0674
84.2600 -36.5700 0.1907 -0.0301 -
0.0706 -0.3736 0.0544 0.5245 0.8830
0.5157 0.8026 -39.0664
84.2800 -36.7200 -0.0774 0.0761 -
0.0709 -0.3707 0.0859 0.5278 0.8736
0.5087 0.8014 -39.0654
84.3000 -36.6300 -0.0300 0.1682 -
0.0791 -0.3543 0.1185 0.5299 0.8638
0.5017 0.8003 -39.0644
84.3200 -36.2500 0.3345 0.1074 -
0.0873 -0.3245 0.1515 0.5309 0.8536
0.4946 0.7992 -39.0634
84.3400 -36.8100 -0.2180 -0.0881 -
0.0855 -0.2814 0.1846 0.5306 0.8428
0.4874 0.7980 -39.0624

84.3600 -36.9300 -0.1832 -0.2122 -
0.0660 -0.2256 0.2172 0.5291 0.8316
0.4802 0.7968 -39.0614
84.3800 -36.1900 0.2768 -0.1654 -
0.0283 -0.1580 0.2487 0.5263 0.8200
0.4730 0.7956 -39.0604
84.4000 -36.4600 -0.1853 -0.0097
0.0181 -0.0801 0.2787 0.5222 0.8079
0.4657 0.7944 -39.0594
84.4200 -36.0400 -0.0744 0.1437
0.0559 0.0064 0.3066 0.5168 0.7953
0.4583 0.7932 -39.0584
84.4400 -35.6500 0.2487 0.1991
0.0673 0.0994 0.3318 0.5099 0.7823
0.4509 0.7920 -39.0574
84.4600 -35.7200 0.0451 0.1333
0.0414 0.1958 0.3539 0.5017 0.7689
0.4435 0.7907 -39.0564
84.4800 -36.0100 -0.1384 0.0229 -
0.0189 0.2912 0.3723 0.4921 0.7551
0.4359 0.7895 -39.0554
84.5000 -36.3100 -0.2326 -0.0387 -
0.0964 0.3802 0.3865 0.4811 0.7408
0.4284 0.7882 -39.0544
84.5200 -35.9600 0.1720 -0.0598 -
0.1650 0.4569 0.3958 0.4687 0.7262
0.4208 0.7869 -39.0534
84.5400 -35.8200 0.1716 -0.1076 -
0.1955 0.5152 0.3999 0.4549 0.7111
0.4132 0.7856 -39.0525
84.5600 -36.0800 -0.0023 -0.1979 -
0.1623 0.5495 0.3983 0.4398 0.6956
0.4055 0.7843 -39.0515
84.5800 -36.3300 -0.3402 -0.1995 -
0.0528 0.5569 0.3908 0.4233 0.6798
0.3978 0.7830 -39.0505
84.6000 -35.6500 0.1581 -0.0434
0.1297 0.5372 0.3774 0.4055 0.6636
0.3900 0.7816 -39.0495
84.6200 -35.4000 0.2295 0.0705
0.3625 0.4918 0.3586 0.3866 0.6471
0.3822 0.7802 -39.0485
84.6400 -35.8700 -0.3436 0.0132
0.6101 0.4229 0.3345 0.3666 0.6302
0.3744 0.7789 -39.0475
84.6600 -35.2400 0.3770 -0.1111
0.8222 0.3331 0.3058 0.3455 0.6130
0.3665 0.7775 -39.0465
84.6800 -35.4900 0.1968 -0.0683
0.9422 0.2253 0.2730 0.3236 0.5954
0.3586 0.7761 -39.0455

84.7000	-36.4300	-0.8429	0.2007	
0.9230	0.1037	0.2368	0.3007	0.5775
	0.3506	0.7746	-39.0445	
84.7200	-35.2500	0.4091	0.5123	
0.7429	-0.0257	0.1977	0.2772	0.5593
	0.3426	0.7732	-39.0435	
84.7400	-35.4800	0.7750	0.4858	
0.4111	-0.1556	0.1563	0.2530	0.5408
	0.3346	0.7718	-39.0425	
84.7600	-37.4700	-0.2013	0.0312	-
0.0234	-0.2785	0.1134	0.2282	0.5221
	0.3266	0.7703	-39.0415	
84.7800	-39.1900	-0.6405	-0.4710	-
0.4825	-0.3869	0.0695	0.2029	0.5030
	0.3185	0.7688	-39.0405	
84.8000	-39.0500	0.2485	-0.6054	-
0.8837	-0.4733	0.0252	0.1772	0.4837
	0.3104	0.7673	-39.0395	
84.8200	-39.3800	-0.0641	-0.3066	-
1.1533	-0.5302	-0.0188	0.1513	0.4641
	0.3023	0.7658	-39.0385	
84.8400	-39.1900	0.0430	0.1197	-
1.2498	-0.5506	-0.0618	0.1251	0.4443
	0.2941	0.7643	-39.0375	
84.8600	-38.8000	0.0396	0.3514	-
1.1639	-0.5307	-0.1035	0.0989	0.4242
	0.2859	0.7628	-39.0365	
84.8800	-38.8100	0.0998	0.2069	-
0.9045	-0.4744	-0.1431	0.0726	0.4039
	0.2777	0.7613	-39.0355	
84.9000	-38.8800	0.0607	-0.2368	-
0.5150	-0.3896	-0.1805	0.0464	0.3834
	0.2695	0.7597	-39.0345	
84.9200	-39.0100	-0.2504	-0.6160	-
0.0621	-0.2855	-0.2153	0.0203	0.3627
	0.2612	0.7581	-39.0335	
84.9400	-38.5400	-0.2148	-0.5978	
0.3890	-0.1715	-0.2473	-0.0055	0.3417
	0.2529	0.7566	-39.0325	
84.9600	-37.1600	0.3079	-0.1829	
0.7812	-0.0568	-0.2762	-0.0309	0.3206
	0.2446	0.7550	-39.0316	
84.9800	-37.0400	-0.4330	0.4288	
1.0552	0.0495	-0.3017	-0.0560	0.2993
	0.2363	0.7534	-39.0306	
85.0000	-35.5400	0.4558	0.8778	
1.1540	0.1388	-0.3238	-0.0805	0.2779
	0.2280	0.7517	-39.0296	
85.0200	-35.9400	0.2543	0.7686	
1.0421	0.2049	-0.3421	-0.1043	0.2562
	0.2196	0.7501	-39.0286	

85.0400 -37.2200 -0.1540 0.1342
0.7601 0.2464 -0.3567 -0.1275 0.2344
0.2113 0.7485 -39.0276
85.0600 -38.2100 0.0572 -0.5498
0.3982 0.2645 -0.3675 -0.1498 0.2125
0.2029 0.7468 -39.0266
85.0800 -39.4300 -0.6578 -0.7077
0.0517 0.2608 -0.3744 -0.1713 0.1905
0.1945 0.7451 -39.0256
85.1000 -38.1500 0.7136 -0.3561 -
0.2078 0.2371 -0.3775 -0.1918 0.1683
0.1861 0.7434 -39.0246
85.1200 -38.8400 -0.1651 -0.0178 -
0.3513 0.1956 -0.3770 -0.2114 0.1460
0.1777 0.7418 -39.0236
85.1400 -38.9500 -0.2170 0.1279 -
0.3896 0.1408 -0.3728 -0.2298 0.1236
0.1693 0.7400 -39.0226
85.1600 -38.4700 0.2272 0.1575 -
0.3668 0.0798 -0.3652 -0.2472 0.1011
0.1609 0.7383 -39.0216
85.1800 -39.0000 -0.2780 0.2308 -
0.3368 0.0205 -0.3543 -0.2635 0.0786
0.1524 0.7366 -39.0206
85.2000 -38.5000 0.2589 0.2733 -
0.3304 -0.0298 -0.3402 -0.2786 0.0560
0.1440 0.7348 -39.0197
85.2200 -39.0200 -0.0443 0.1631 -
0.3488 -0.0662 -0.3232 -0.2925 0.0333
0.1355 0.7331 -39.0187
85.2400 -39.4700 -0.1746 -0.0141 -
0.3747 -0.0874 -0.3034 -0.3052 0.0105
0.1271 0.7313 -39.0177
85.2600 -39.3800 -0.0007 -0.1496 -
0.3841 -0.0940 -0.2811 -0.3168 -0.0123
0.1186 0.7295 -39.0167
85.2800 -39.4500 0.0216 -0.2312 -
0.3504 -0.0881 -0.2567 -0.3272 -0.0351
0.1102 0.7278 -39.0157
85.3000 -39.1900 0.2060 -0.2543 -
0.2584 -0.0730 -0.2303 -0.3365 -0.0579
0.1017 0.7259 -39.0147
85.3200 -39.5600 -0.4366 -0.1574 -
0.1129 -0.0521 -0.2023 -0.3445 -0.0808
0.0933 0.7241 -39.0137
85.3400 -38.6200 0.1246 0.0754
0.0589 -0.0290 -0.1732 -0.3515 -0.1036
0.0848 0.7223 -39.0127
85.3600 -38.0300 0.3902 0.2767
0.2195 -0.0067 -0.1431 -0.3573 -0.1265
0.0764 0.7205 -39.0117

85.3800 -38.5200 -0.3485 0.3256
0.3278 0.0117 -0.1125 -0.3620 -0.1493
0.0679 0.7186 -39.0108
85.4000 -38.0300 0.0278 0.3145
0.3516 0.0237 -0.0818 -0.3656 -0.1721
0.0595 0.7168 -39.0098
85.4200 -38.1400 0.1122 0.3126
0.2834 0.0289 -0.0511 -0.3682 -0.1948
0.0511 0.7149 -39.0088
85.4400 -38.3500 0.0100 0.2076
0.1492 0.0288 -0.0211 -0.3697 -0.2175
0.0427 0.7130 -39.0078
85.4600 -38.7900 0.0514 -0.0624 -
0.0051 0.0254 0.0081 -0.3703 -0.2401
0.0342 0.7111 -39.0068
85.4800 -39.4800 -0.1237 -0.3819 -
0.1324 0.0207 0.0360 -0.3698 -0.2627
0.0258 0.7092 -39.0058
85.5000 -39.5600 0.0686 -0.5637 -
0.1943 0.0167 0.0624 -0.3685 -0.2851
0.0174 0.7073 -39.0048
85.5200 -39.9300 -0.3850 -0.4241 -
0.1764 0.0144 0.0869 -0.3662 -0.3075
0.0091 0.7053 -39.0038
85.5400 -39.1000 -0.0931 0.0135 -
0.0992 0.0141 0.1093 -0.3632 -0.3297
0.0007 0.7034 -39.0028
85.5600 -38.3400 0.1058 0.4281 -
0.0024 0.0155 0.1295 -0.3592 -0.3518
-0.0076 0.7014 -39.0019
85.5800 -37.7800 0.5425 0.4559
0.0759 0.0183 0.1472 -0.3546 -0.3737
-0.0160 0.6995 -39.0009
85.6000 -39.0600 -0.4544 0.1250
0.1142 0.0226 0.1622 -0.3492 -0.3955
-0.0243 0.6975 -38.9999
85.6200 -39.2300 -0.2410 -0.1707
0.1082 0.0284 0.1745 -0.3431 -0.4171
-0.0326 0.6955 -38.9989
85.6400 -38.6200 0.4150 -0.2247
0.0641 0.0361 0.1841 -0.3364 -0.4386
-0.0409 0.6935 -38.9979
85.6600 -39.3500 -0.3813 -0.0833
0.0031 0.0453 0.1909 -0.3292 -0.4598
-0.0491 0.6915 -38.9969
85.6800 -38.5700 0.2726 0.0819 -
0.0524 0.0553 0.1950 -0.3215 -0.4809
-0.0574 0.6895 -38.9959
85.7000 -38.7900 0.0334 0.1046 -
0.0850 0.0649 0.1966 -0.3133 -0.5017
-0.0656 0.6875 -38.9950

85.7200	-39.0800	-0.0384	-0.0462	-
0.0857	0.0733	0.1957	-0.3048	-0.5223
	-0.0738	0.6855	-38.9940	
85.7400	-39.4100	-0.2484	-0.1615	-
0.0546	0.0788	0.1927	-0.2959	-0.5427
	-0.0819	0.6834	-38.9930	
85.7600	-38.8300	0.2681	-0.0663	-
0.0016	0.0800	0.1877	-0.2868	-0.5628
	-0.0901	0.6813	-38.9920	
85.7800	-39.1100	-0.2468	0.1040	
0.0565	0.0759	0.1811	-0.2776	-0.5826
	-0.0982	0.6793	-38.9910	
85.8000	-38.3200	0.4840	0.1164	
0.1032	0.0660	0.1732	-0.2683	-0.6021
	-0.1062	0.6772	-38.9900	
85.8200	-39.2800	-0.3666	0.0003	
0.1254	0.0508	0.1642	-0.2589	-0.6214
	-0.1143	0.6751	-38.9891	
85.8400	-39.1900	-0.1244	-0.0332	
0.1175	0.0309	0.1544	-0.2496	-0.6403
	-0.1223	0.6730	-38.9881	
85.8600	-38.8600	0.1628	0.0326	
0.0811	0.0079	0.1441	-0.2403	-0.6589
	-0.1303	0.6709	-38.9871	
85.8800	-39.0500	0.1669	0.0306	
0.0233	-0.0159	0.1336	-0.2312	-0.6772
	-0.1382	0.6688	-38.9861	
85.9000	-39.4900	-0.2031	-0.0333	-
0.0418	-0.0383	0.1231	-0.2224	-0.6951
	-0.1462	0.6667	-38.9851	
85.9200	-39.5800	-0.1183	-0.0467	-
0.0977	-0.0567	0.1131	-0.2138	-0.7127
	-0.1540	0.6645	-38.9841	
85.9400	-39.2500	0.2719	-0.0309	-
0.1319	-0.0690	0.1035	-0.2055	-0.7299
	-0.1619	0.6624	-38.9831	
85.9600	-39.6600	-0.1066	-0.0526	-
0.1381	-0.0740	0.0948	-0.1977	-0.7468
	-0.1697	0.6602	-38.9822	
85.9800	-39.7100	0.0672	-0.0704	-
0.1199	-0.0717	0.0871	-0.1903	-0.7632
	-0.1775	0.6581	-38.9812	
86.0000	-39.7300	-0.2080	-0.0105	-
0.0881	-0.0628	0.0804	-0.1834	-0.7792
	-0.1852	0.6559	-38.9802	
86.0200	-39.1600	0.2540	0.1063	-
0.0523	-0.0489	0.0750	-0.1771	-0.7948
	-0.1929	0.6537	-38.9792	
86.0400	-39.6300	-0.2811	0.1319	-
0.0192	-0.0320	0.0708	-0.1714	-0.8100
	-0.2005	0.6515	-38.9782	

86.0600 -39.0000 0.4235 -0.0082
0.0151 -0.0147 0.0681 -0.1664 -0.8248
-0.2081 0.6493 -38.9773
86.0800 -39.9500 -0.3984 -0.1653
0.0596 0.0006 0.0667 -0.1620 -0.8391
-0.2157 0.6471 -38.9763
86.1000 -39.6800 -0.1707 -0.1551
0.1155 0.0119 0.0667 -0.1584 -0.8529
-0.2232 0.6449 -38.9753
86.1200 -38.7800 0.4796 -0.0293
0.1710 0.0179 0.0680 -0.1555 -0.8663
-0.2306 0.6427 -38.9743
86.1400 -39.5000 -0.1878 0.0692
0.2092 0.0182 0.0707 -0.1533 -0.8792
-0.2381 0.6404 -38.9733
86.1600 -39.4400 -0.2473 0.1133
0.2158 0.0136 0.0746 -0.1520 -0.8917
-0.2454 0.6382 -38.9723
86.1800 -38.9400 0.3255 0.1405
0.1812 0.0052 0.0796 -0.1515 -0.9036
-0.2527 0.6359 -38.9714
86.2000 -39.6200 -0.1985 0.1172
0.1072 -0.0052 0.0857 -0.1517 -0.9151
-0.2600 0.6337 -38.9704
86.2200 -39.7400 -0.0392 -0.0175
0.0111 -0.0154 0.0928 -0.1528 -0.9261
-0.2672 0.6314 -38.9694
86.2400 -39.6800 0.2843 -0.1915 -
0.0874 -0.0240 0.1008 -0.1546 -0.9365
-0.2744 0.6291 -38.9684
86.2600 -40.5200 -0.3756 -0.2319 -
0.1690 -0.0296 0.1096 -0.1573 -0.9465
-0.2815 0.6268 -38.9674
86.2800 -39.7200 0.1876 -0.0266 -
0.2213 -0.0310 0.1189 -0.1607 -0.9560
-0.2885 0.6245 -38.9665
86.3000 -39.6500 -0.0267 0.2022 -
0.2386 -0.0276 0.1287 -0.1649 -0.9649
-0.2955 0.6222 -38.9655
86.3200 -39.5100 0.2100 0.1707 -
0.2194 -0.0191 0.1388 -0.1698 -0.9734
-0.3024 0.6199 -38.9645
86.3400 -40.0000 -0.0374 -0.0713 -
0.1672 -0.0059 0.1489 -0.1755 -0.9813
-0.3093 0.6176 -38.9635
86.3600 -40.2600 -0.2726 -0.2100 -
0.0865 0.0106 0.1589 -0.1819 -0.9888
-0.3161 0.6152 -38.9626
86.3800 -40.1700 -0.5156 -0.0968
0.0145 0.0291 0.1687 -0.1889 -0.9957
-0.3228 0.6129 -38.9616

86.4000 -38.9100 0.6849 0.0696
0.1225 0.0481 0.1781 -0.1966 -1.0021
-0.3295 0.6105 -38.9606
86.4200 -38.9700 0.3176 0.0485
0.2227 0.0660 0.1868 -0.2050 -1.0080
-0.3361 0.6082 -38.9596
86.4400 -40.1800 -0.8164 -0.0701
0.2970 0.0818 0.1949 -0.2139 -1.0134
-0.3427 0.6058 -38.9586
86.4600 -39.7900 -0.4681 -0.0232
0.3278 0.0945 0.2022 -0.2235 -1.0183
-0.3491 0.6035 -38.9577
86.4800 -38.8400 0.2733 0.1744
0.3043 0.1034 0.2086 -0.2335 -1.0227
-0.3556 0.6011 -38.9567
86.5000 -38.2800 0.8797 0.2636
0.2264 0.1081 0.2140 -0.2441 -1.0265
-0.3619 0.5987 -38.9557
86.5200 -40.2200 -0.8168 0.1432
0.1062 0.1086 0.2185 -0.2552 -1.0299
-0.3682 0.5963 -38.9547
86.5400 -40.0700 -0.1981 -0.0803 -
0.0364 0.1050 0.2220 -0.2667 -1.0328
-0.3744 0.5939 -38.9538
86.5600 -39.3800 0.7940 -0.2780 -
0.1770 0.0975 0.2244 -0.2786 -1.0351
-0.3805 0.5915 -38.9528
86.5800 -40.6200 -0.2160 -0.3563 -
0.2920 0.0859 0.2257 -0.2909 -1.0370
-0.3866 0.5891 -38.9518
86.6000 -40.9200 -0.5936 -0.2060 -
0.3637 0.0700 0.2258 -0.3035 -1.0383
-0.3926 0.5867 -38.9508
86.6200 -40.2400 -0.1789 0.1814 -
0.3817 0.0501 0.2249 -0.3164 -1.0391
-0.3985 0.5842 -38.9498
86.6400 -39.3200 0.4922 0.4981 -
0.3421 0.0267 0.2229 -0.3295 -1.0395
-0.4044 0.5818 -38.9489
86.6600 -39.5100 0.2959 0.3724 -
0.2443 0.0003 0.2198 -0.3427 -1.0393
-0.4101 0.5793 -38.9479
86.6800 -40.8100 -0.4865 -0.1000 -
0.1008 -0.0283 0.2156 -0.3562 -1.0387
-0.4158 0.5769 -38.9469
86.7000 -40.4600 0.0013 -0.4953
0.0668 -0.0581 0.2103 -0.3697 -1.0375
-0.4215 0.5744 -38.9459
86.7200 -40.2700 0.0974 -0.5224
0.2342 -0.0879 0.2039 -0.3832 -1.0358
-0.4270 0.5720 -38.9450

86.7400 -40.2400 -0.2941 -0.1940
0.3749 -0.1165 0.1965 -0.3968 -1.0337
-0.4325 0.5695 -38.9440
86.7600 -39.1100 0.3948 0.2471
0.4633 -0.1427 0.1879 -0.4104 -1.0310
-0.4379 0.5670 -38.9430
86.7800 -39.1400 -0.0116 0.5606
0.4738 -0.1655 0.1780 -0.4238 -1.0279
-0.4432 0.5645 -38.9420
86.8000 -39.8800 -0.5395 0.5916
0.3908 -0.1838 0.1669 -0.4372 -1.0242
-0.4485 0.5620 -38.9411
86.8200 -39.0800 0.7428 0.2535
0.2258 -0.1965 0.1543 -0.4504 -1.0201
-0.4537 0.5596 -38.9401
86.8400 -41.2300 -0.5489 -0.2730
0.0197 -0.2019 0.1404 -0.4633 -1.0155
-0.4588 0.5570 -38.9391
86.8600 -41.3900 -0.2849 -0.5140 -
0.1787 -0.1983 0.1250 -0.4760 -1.0103
-0.4638 0.5545 -38.9382
86.8800 -41.0100 0.0829 -0.3174 -
0.3296 -0.1842 0.1083 -0.4884 -1.0047
-0.4688 0.5520 -38.9372
86.9000 -40.2200 0.6081 -0.0407 -
0.4147 -0.1580 0.0902 -0.5005 -0.9986
-0.4737 0.5495 -38.9362
86.9200 -41.2100 -0.4276 0.0789 -
0.4315 -0.1192 0.0709 -0.5121 -0.9920
-0.4785 0.5470 -38.9352
86.9400 -40.9600 -0.1836 0.0788 -
0.3884 -0.0684 0.0504 -0.5232 -0.9850
-0.4833 0.5444 -38.9343
86.9600 -40.3700 0.2809 0.0171 -
0.2965 -0.0079 0.0289 -0.5339 -0.9774
-0.4879 0.5419 -38.9333
86.9800 -40.2400 0.2441 -0.0784 -
0.1661 0.0585 0.0066 -0.5440 -0.9694
-0.4925 0.5393 -38.9323
87.0000 -40.8200 -0.4345 -0.1401 -
0.0129 0.1266 -0.0165 -0.5535 -0.9608
-0.4971 0.5368 -38.9313
87.0200 -39.9200 0.1734 -0.0908
0.1428 0.1920 -0.0402 -0.5623 -0.9518
-0.5015 0.5342 -38.9304
87.0400 -39.6100 0.3111 0.0124
0.2762 0.2505 -0.0643 -0.5704 -0.9424
-0.5059 0.5317 -38.9294
87.0600 -39.9600 -0.2582 0.1213
0.3630 0.2979 -0.0886 -0.5777 -0.9324
-0.5102 0.5291 -38.9284

87.0800 -39.8500 -0.3241 0.2814
0.3852 0.3310 -0.1132 -0.5843 -0.9220
-0.5145 0.5265 -38.9275
87.1000 -39.1000 0.4353 0.3889
0.3403 0.3481 -0.1377 -0.5899 -0.9111
-0.5187 0.5240 -38.9265
87.1200 -39.3700 0.2808 0.2972
0.2391 0.3488 -0.1619 -0.5946 -0.8998
-0.5228 0.5214 -38.9255
87.1400 -40.7900 -0.5814 0.0373
0.1057 0.3345 -0.1858 -0.5984 -0.8879
-0.5268 0.5188 -38.9245
87.1600 -40.5700 0.1386 -0.2533 -
0.0260 0.3070 -0.2091 -0.6011 -0.8757
-0.5308 0.5162 -38.9236
87.1800 -40.4500 0.4709 -0.4736 -
0.1215 0.2681 -0.2315 -0.6028 -0.8630
-0.5347 0.5136 -38.9226
87.2000 -41.5400 -0.5165 -0.5211 -
0.1586 0.2199 -0.2528 -0.6033 -0.8498
-0.5385 0.5110 -38.9216
87.2200 -41.2700 -0.2366 -0.3091 -
0.1373 0.1642 -0.2728 -0.6027 -0.8362
-0.5423 0.5084 -38.9207
87.2400 -40.2300 0.4325 0.0392 -
0.0793 0.1033 -0.2912 -0.6010 -0.8222
-0.5460 0.5058 -38.9197
87.2600 -39.9700 0.3722 0.3173 -
0.0129 0.0396 -0.3078 -0.5979 -0.8077
-0.5496 0.5031 -38.9187
87.2800 -40.9600 -0.7336 0.4853
0.0329 -0.0245 -0.3223 -0.5936 -0.7928
-0.5532 0.5005 -38.9178
87.3000 -39.8700 0.3803 0.4931
0.0403 -0.0871 -0.3344 -0.5880 -0.7776
-0.5567 0.4979 -38.9168
87.3200 -40.0200 0.4869 0.2129
0.0164 -0.1470 -0.3440 -0.5811 -0.7619
-0.5601 0.4952 -38.9158
87.3400 -41.6400 -0.5279 -0.2631 -
0.0168 -0.2029 -0.3506 -0.5728 -0.7459
-0.5635 0.4926 -38.9148
87.3600 -41.4500 -0.0160 -0.5490 -
0.0353 -0.2540 -0.3541 -0.5632 -0.7294
-0.5668 0.4900 -38.9139
87.3800 -41.5300 -0.2182 -0.3686 -
0.0228 -0.2990 -0.3542 -0.5522 -0.7127
-0.5701 0.4873 -38.9129
87.4000 -40.8100 -0.0127 0.0919
0.0168 -0.3366 -0.3507 -0.5399 -0.6955
-0.5732 0.4847 -38.9119

87.4200 -40.1300 0.3386 0.4091
0.0662 -0.3652 -0.3432 -0.5263 -0.6781
-0.5764 0.4820 -38.9110
87.4400 -40.2100 0.2761 0.3008
0.1059 -0.3833 -0.3320 -0.5114 -0.6603
-0.5794 0.4794 -38.9100
87.4600 -41.1900 -0.5148 -0.0435
0.1177 -0.3892 -0.3168 -0.4954 -0.6422
-0.5824 0.4767 -38.9090
87.4800 -40.9000 0.0329 -0.2058
0.0883 -0.3813 -0.2979 -0.4782 -0.6238
-0.5853 0.4740 -38.9081
87.5000 -40.6800 0.0921 -0.0791
0.0136 -0.3588 -0.2755 -0.4600 -0.6051
-0.5882 0.4714 -38.9071
87.5200 -40.4000 0.1738 0.0986 -
0.0959 -0.3212 -0.2499 -0.4407 -0.5862
-0.5910 0.4687 -38.9061
87.5400 -40.8200 -0.1989 0.1271 -
0.2231 -0.2690 -0.2214 -0.4205 -0.5669
-0.5937 0.4660 -38.9052
87.5600 -40.4800 0.2738 -0.0025 -
0.3457 -0.2037 -0.1904 -0.3994 -0.5475
-0.5964 0.4633 -38.9042
87.5800 -41.1900 -0.3754 -0.1092 -
0.4362 -0.1276 -0.1573 -0.3775 -0.5278
-0.5991 0.4607 -38.9032
87.6000 -40.1900 0.3283 -0.0504 -
0.4676 -0.0439 -0.1226 -0.3548 -0.5078
-0.6016 0.4580 -38.9023
87.6200 -40.6300 -0.2502 0.0113 -
0.4194 0.0437 -0.0866 -0.3315 -0.4877
-0.6041 0.4553 -38.9013
87.6400 -39.7400 0.3161 -0.0411 -
0.2866 0.1312 -0.0499 -0.3076 -0.4673
-0.6066 0.4526 -38.9003
87.6600 -39.9100 -0.1183 -0.1279 -
0.0833 0.2146 -0.0128 -0.2831 -0.4467
-0.6089 0.4499 -38.8994
87.6800 -39.3600 0.0487 -0.1103
0.1605 0.2895 0.0240 -0.2582 -0.4260
-0.6113 0.4472 -38.8984
87.7000 -39.0600 -0.3002 0.0650
0.4025 0.3515 0.0603 -0.2329 -0.4051
-0.6135 0.4445 -38.8974
87.7200 -37.9400 0.4853 0.2067
0.5968 0.3964 0.0955 -0.2073 -0.3841
-0.6158 0.4418 -38.8965
87.7400 -38.6600 -0.3795 0.1351
0.7036 0.4201 0.1292 -0.1815 -0.3629
-0.6179 0.4391 -38.8955

87.7600 -38.2300 0.0468 0.0169
0.7062 0.4198 0.1610 -0.1554 -0.3415
-0.6200 0.4364 -38.8946
87.7800 -38.4100 -0.0727 -0.0124
0.6063 0.3955 0.1906 -0.1293 -0.3201
-0.6221 0.4337 -38.8936
87.8000 -38.3500 0.1809 -0.0132
0.4228 0.3506 0.2177 -0.1031 -0.2985
-0.6240 0.4310 -38.8926
87.8200 -38.7400 -0.0911 -0.0038
0.1833 0.2898 0.2424 -0.0769 -0.2769
-0.6260 0.4282 -38.8917
87.8400 -39.1100 -0.1617 0.0376 -
0.0812 0.2185 0.2644 -0.0509 -0.2552
-0.6278 0.4255 -38.8907
87.8600 -38.9500 0.2813 0.0755 -
0.3364 0.1417 0.2837 -0.0250 -0.2334
-0.6297 0.4228 -38.8897
87.8800 -39.3700 0.0527 0.0386 -
0.5476 0.0647 0.3004 0.0007 -0.2115
-0.6314 0.4201 -38.8888
87.9000 -40.2400 -0.5020 -0.0426 -
0.6844 -0.0074 0.3142 0.0260 -0.1896
-0.6331 0.4174 -38.8878
87.9200 -39.2600 0.5622 -0.1170 -
0.7226 -0.0702 0.3253 0.0510 -0.1677
-0.6348 0.4146 -38.8868
87.9400 -39.9900 -0.1920 -0.2116 -
0.6520 -0.1207 0.3336 0.0755 -0.1457
-0.6364 0.4119 -38.8859
87.9600 -40.1400 -0.3720 -0.2098 -
0.4845 -0.1583 0.3393 0.0996 -0.1237
-0.6380 0.4092 -38.8849
87.9800 -39.0900 0.2400 -0.0509 -
0.2503 -0.1839 0.3424 0.1232 -0.1018
-0.6395 0.4065 -38.8840
88.0000 -38.5200 0.2663 0.1244
0.0053 -0.1988 0.3432 0.1461 -0.0798
-0.6409 0.4037 -38.8830
88.0200 -38.7200 -0.2971 0.2119
0.2330 -0.2045 0.3418 0.1685 -0.0579
-0.6423 0.4010 -38.8820
88.0400 -38.1000 0.1160 0.2142
0.3929 -0.2024 0.3385 0.1901 -0.0360
-0.6436 0.3983 -38.8811
88.0600 -38.2300 -0.0768 0.1458
0.4659 -0.1939 0.3335 0.2110 -0.0141
-0.6449 0.3955 -38.8801
88.0800 -38.1300 0.1732 -0.0134
0.4546 -0.1809 0.3271 0.2312 0.0077
-0.6462 0.3928 -38.8792

88.1000 -38.4800 0.0521 -0.1615
0.3778 -0.1653 0.3195 0.2505 0.0294
-0.6474 0.3900 -38.8782
88.1200 -39.0500 -0.4810 -0.1231
0.2568 -0.1493 0.3112 0.2691 0.0510
-0.6485 0.3873 -38.8772
88.1400 -37.8600 0.5120 0.0697
0.1137 -0.1344 0.3023 0.2867 0.0725
-0.6496 0.3846 -38.8763
88.1600 -38.6700 -0.1887 0.1035 -
0.0251 -0.1211 0.2929 0.3034 0.0939
-0.6507 0.3818 -38.8753
88.1800 -38.7000 0.0819 -0.0803 -
0.1326 -0.1096 0.2834 0.3193 0.1152
-0.6517 0.3791 -38.8744
88.2000 -39.1400 -0.2691 -0.1892 -
0.1894 -0.1000 0.2737 0.3342 0.1364
-0.6526 0.3763 -38.8734
88.2200 -38.6300 0.2103 -0.0727 -
0.1937 -0.0918 0.2640 0.3483 0.1574
-0.6535 0.3736 -38.8724
88.2400 -38.5800 -0.0518 0.0828 -
0.1565 -0.0842 0.2543 0.3614 0.1783
-0.6544 0.3708 -38.8715
88.2600 -38.4000 0.0538 0.1001 -
0.0968 -0.0762 0.2445 0.3736 0.1990
-0.6552 0.3681 -38.8705
88.2800 -38.4500 -0.0042 0.0271 -
0.0320 -0.0669 0.2346 0.3851 0.2195
-0.6559 0.3654 -38.8696
88.3000 -38.4100 -0.0867 -0.0396
0.0272 -0.0561 0.2243 0.3956 0.2398
-0.6567 0.3626 -38.8686
88.3200 -38.3100 0.1002 -0.0784
0.0720 -0.0438 0.2137 0.4054 0.2600
-0.6573 0.3599 -38.8676
88.3400 -38.4400 -0.0116 -0.0865
0.0943 -0.0303 0.2024 0.4145 0.2799
-0.6580 0.3571 -38.8667
88.3600 -38.4600 -0.1590 -0.0020
0.0875 -0.0152 0.1904 0.4229 0.2997
-0.6585 0.3544 -38.8657
88.3800 -37.8600 0.2415 0.1410
0.0510 0.0018 0.1775 0.4306 0.3192
-0.6591 0.3516 -38.8648
88.4000 -38.1900 -0.1230 0.1969 -
0.0078 0.0211 0.1635 0.4377 0.3385
-0.6596 0.3489 -38.8638
88.4200 -38.0400 0.0546 0.1044 -
0.0731 0.0430 0.1482 0.4442 0.3575
-0.6600 0.3462 -38.8628

88.4400 -38.3000 0.0628 -0.0923 -
0.1241 0.0675 0.1313 0.4502 0.3763
-0.6604 0.3434 -38.8619
88.4600 -38.4300 0.1180 -0.2818 -
0.1414 0.0944 0.1130 0.4557 0.3948
-0.6608 0.3407 -38.8609
88.4800 -38.7500 -0.3895 -0.2509 -
0.1165 0.1228 0.0930 0.4607 0.4130
-0.6611 0.3379 -38.8600
88.5000 -37.8400 0.1885 0.0626 -
0.0613 0.1512 0.0714 0.4654 0.4310
-0.6614 0.3352 -38.8590
88.5200 -37.5200 0.1275 0.3265 -
0.0003 0.1776 0.0483 0.4697 0.4487
-0.6616 0.3324 -38.8581
88.5400 -37.3500 0.3940 0.2235
0.0445 0.1996 0.0236 0.4736 0.4660
-0.6618 0.3297 -38.8571
88.5600 -38.3400 -0.2960 -0.0857
0.0680 0.2148 -0.0025 0.4772 0.4831
-0.6620 0.3270 -38.8562
88.5800 -38.5300 -0.3776 -0.2425
0.0766 0.2213 -0.0298 0.4805 0.4998
-0.6621 0.3242 -38.8552
88.6000 -37.9600 0.1838 -0.1329
0.0779 0.2173 -0.0581 0.4836 0.5162
-0.6622 0.3215 -38.8542
88.6200 -37.9400 0.0454 0.0466
0.0795 0.2018 -0.0872 0.4864 0.5322
-0.6622 0.3187 -38.8533
88.6400 -37.8200 0.0028 0.1176
0.0814 0.1744 -0.1168 0.4890 0.5479
-0.6622 0.3160 -38.8523
88.6600 -37.9600 -0.0130 0.0688
0.0814 0.1361 -0.1463 0.4914 0.5633
-0.6622 0.3133 -38.8514
88.6800 -38.1800 0.0368 -0.0700
0.0809 0.0889 -0.1756 0.4936 0.5782
-0.6621 0.3105 -38.8504
88.7000 -38.2500 0.0723 -0.1918
0.0803 0.0355 -0.2041 0.4956 0.5928
-0.6620 0.3078 -38.8495
88.7200 -38.8000 -0.3892 -0.1306
0.0763 -0.0214 -0.2314 0.4975 0.6070
-0.6618 0.3051 -38.8485
88.7400 -38.2100 0.0380 0.1082
0.0641 -0.0789 -0.2572 0.4992 0.6208
-0.6616 0.3024 -38.8476
88.7600 -37.8300 0.3702 0.2549
0.0347 -0.1339 -0.2810 0.5007 0.6342
-0.6614 0.2996 -38.8466

88.7800 -38.2000 0.1321 0.1405 -
0.0152 -0.1828 -0.3025 0.5021 0.6471
-0.6611 0.2969 -38.8457
88.8000 -39.2400 -0.5100 -0.0485 -
0.0787 -0.2228 -0.3212 0.5034 0.6597
-0.6608 0.2942 -38.8447
88.8200 -38.5100 0.3107 -0.1177 -
0.1445 -0.2509 -0.3368 0.5047 0.6718
-0.6605 0.2915 -38.8437
88.8400 -38.9500 0.1364 -0.1213 -
0.1948 -0.2648 -0.3489 0.5058 0.6834
-0.6601 0.2887 -38.8428
88.8600 -39.2100 -0.2245 -0.1316 -
0.2145 -0.2634 -0.3574 0.5069 0.6947
-0.6597 0.2860 -38.8418
88.8800 -39.0600 -0.1735 -0.1039 -
0.2003 -0.2468 -0.3620 0.5079 0.7054
-0.6593 0.2833 -38.8409
88.9000 -38.3100 0.5056 -0.0340 -
0.1575 -0.2163 -0.3630 0.5089 0.7157
-0.6588 0.2806 -38.8399
88.9200 -38.8700 -0.3355 0.0418 -
0.1009 -0.1740 -0.3604 0.5098 0.7256
-0.6583 0.2779 -38.8390
88.9400 -38.7600 -0.4448 0.1387 -
0.0451 -0.1227 -0.3546 0.5107 0.7350
-0.6577 0.2752 -38.8380
88.9600 -38.0000 0.1538 0.2156 -
0.0002 -0.0655 -0.3456 0.5115 0.7439
-0.6572 0.2725 -38.8371
88.9800 -37.5500 0.6843 0.0980
0.0321 -0.0059 -0.3337 0.5123 0.7524
-0.6566 0.2698 -38.8361
89.0000 -38.9300 -0.6365 -0.1934
0.0578 0.0522 -0.3191 0.5130 0.7604
-0.6559 0.2671 -38.8352
89.0200 -38.1600 0.1031 -0.2815
0.0844 0.1051 -0.3021 0.5136 0.7679
-0.6553 0.2644 -38.8342
89.0400 -37.9900 0.0714 -0.0845
0.1166 0.1493 -0.2830 0.5140 0.7750
-0.6546 0.2617 -38.8333
89.0600 -37.6900 -0.0560 0.1657
0.1501 0.1820 -0.2618 0.5144 0.7816
-0.6538 0.2590 -38.8323
89.0800 -37.3600 0.1714 0.2387
0.1716 0.2013 -0.2389 0.5146 0.7877
-0.6531 0.2563 -38.8314
89.1000 -37.5800 -0.0088 0.1254
0.1665 0.2065 -0.2144 0.5146 0.7934
-0.6523 0.2536 -38.8304

89.1200 -37.7500 0.0746 -0.0546
0.1309 0.1981 -0.1886 0.5145 0.7986
-0.6514 0.2509 -38.8295
89.1400 -37.9800 -0.0677 -0.1639
0.0734 0.1778 -0.1617 0.5141 0.8034
-0.6506 0.2483 -38.8285
89.1600 -38.1000 -0.1085 -0.1140
0.0086 0.1480 -0.1339 0.5136 0.8077
-0.6497 0.2456 -38.8276
89.1800 -37.7600 0.1727 0.0296 -
0.0509 0.1115 -0.1055 0.5128 0.8116
-0.6488 0.2429 -38.8266
89.2000 -38.1100 -0.1804 0.1276 -
0.0977 0.0714 -0.0767 0.5117 0.8150
-0.6479 0.2402 -38.8257
89.2200 -37.8100 0.0969 0.1290 -
0.1287 0.0311 -0.0479 0.5103 0.8180
-0.6469 0.2376 -38.8247
89.2400 -37.9600 0.0078 0.0391 -
0.1423 -0.0065 -0.0192 0.5086 0.8205
-0.6459 0.2349 -38.8238
89.2600 -38.0400 0.2168 -0.1308 -
0.1356 -0.0388 0.0092 0.5066 0.8226
-0.6449 0.2322 -38.8228
89.2800 -38.6400 -0.3762 -0.2189 -
0.1079 -0.0636 0.0370 0.5043 0.8243
-0.6438 0.2296 -38.8219
89.3000 -38.0000 0.0145 -0.0942 -
0.0639 -0.0794 0.0641 0.5015 0.8255
-0.6428 0.2269 -38.8209
89.3200 -37.4300 0.4046 0.1130 -
0.0168 -0.0859 0.0903 0.4983 0.8264
-0.6417 0.2243 -38.8200
89.3400 -37.5900 -0.0042 0.1998
0.0182 -0.0836 0.1156 0.4947 0.8268
-0.6405 0.2217 -38.8190
89.3600 -37.9000 -0.2242 0.1402
0.0321 -0.0737 0.1397 0.4906 0.8268
-0.6394 0.2190 -38.8181
89.3800 -38.1100 -0.1673 0.0263
0.0259 -0.0577 0.1627 0.4860 0.8264
-0.6382 0.2164 -38.8171
89.4000 -37.6000 0.3175 -0.0588
0.0081 -0.0377 0.1845 0.4809 0.8256
-0.6370 0.2137 -38.8162
89.4200 -37.9100 -0.0482 -0.1070 -
0.0088 -0.0156 0.2048 0.4752 0.8245
-0.6358 0.2111 -38.8152
89.4400 -37.9900 -0.1912 -0.0900 -
0.0148 0.0065 0.2238 0.4690 0.8229
-0.6345 0.2085 -38.8143

89.4600 -37.7200 -0.0516 0.0152 -
0.0055 0.0271 0.2413 0.4622 0.8210
-0.6333 0.2059 -38.8133
89.4800 -37.2600 0.3080 0.0796
0.0196 0.0445 0.2570 0.4547 0.8187
-0.6320 0.2033 -38.8124
89.5000 -37.7200 -0.1276 -0.0032
0.0557 0.0575 0.2711 0.4467 0.8161
-0.6307 0.2007 -38.8115
89.5200 -37.6700 -0.0403 -0.1069
0.0924 0.0658 0.2832 0.4380 0.8131
-0.6293 0.1981 -38.8105
89.5400 -37.7400 -0.1411 -0.0595
0.1138 0.0691 0.2934 0.4287 0.8097
-0.6279 0.1955 -38.8096
89.5600 -37.3800 0.0554 0.1014
0.1047 0.0681 0.3015 0.4188 0.8060
-0.6266 0.1929 -38.8086
89.5800 -37.3100 0.1044 0.1855
0.0600 0.0633 0.3074 0.4083 0.8020
-0.6252 0.1903 -38.8077
89.6000 -37.5400 0.0165 0.0889 -
0.0101 0.0560 0.3110 0.3971 0.7976
-0.6237 0.1877 -38.8067
89.6200 -37.8000 0.0232 -0.0745 -
0.0807 0.0471 0.3123 0.3854 0.7929
-0.6223 0.1851 -38.8058
89.6400 -38.2200 -0.1857 -0.1683 -
0.1221 0.0379 0.3111 0.3730 0.7880
-0.6208 0.1825 -38.8048
89.6600 -37.8100 0.1952 -0.1764 -
0.1124 0.0296 0.3074 0.3602 0.7827
-0.6193 0.1800 -38.8039
89.6800 -38.0600 -0.0821 -0.1751 -
0.0551 0.0236 0.3011 0.3467 0.7771
-0.6178 0.1774 -38.8029
89.7000 -38.0000 -0.0315 -0.1180
0.0240 0.0213 0.2923 0.3328 0.7713
-0.6162 0.1748 -38.8020
89.7200 -37.8400 -0.1649 0.0869
0.0901 0.0238 0.2809 0.3183 0.7651
-0.6147 0.1723 -38.8011
89.7400 -37.1500 0.3026 0.3178
0.1144 0.0320 0.2671 0.3035 0.7587
-0.6131 0.1697 -38.8001
89.7600 -37.4200 -0.1271 0.3850
0.0823 0.0455 0.2510 0.2882 0.7521
-0.6115 0.1672 -38.7992
89.7800 -37.7900 -0.0539 0.2421 -
0.0015 0.0627 0.2327 0.2725 0.7452
-0.6099 0.1646 -38.7982

89.8000 -37.9500 0.2074 -0.0531 -
0.1094 0.0818 0.2125 0.2564 0.7380
-0.6083 0.1621 -38.7973
89.8200 -38.6200 -0.1123 -0.3283 -
0.2019 0.1006 0.1904 0.2400 0.7306
-0.6066 0.1596 -38.7963
89.8400 -38.8900 -0.1491 -0.4319 -
0.2395 0.1170 0.1668 0.2233 0.7230
-0.6050 0.1571 -38.7954
89.8600 -38.3300 0.2563 -0.3207 -
0.1990 0.1290 0.1419 0.2064 0.7151
-0.6033 0.1545 -38.7945
89.8800 -38.4900 -0.2985 -0.0677 -
0.0881 0.1349 0.1159 0.1892 0.7071
-0.6016 0.1520 -38.7935
89.9000 -37.7100 0.2511 0.1585
0.0626 0.1333 0.0892 0.1718 0.6988
-0.5998 0.1495 -38.7926
89.9200 -37.8500 -0.1407 0.2423
0.2075 0.1229 0.0621 0.1542 0.6903
-0.5981 0.1470 -38.7916
89.9400 -37.6200 0.0827 0.2737
0.3029 0.1027 0.0348 0.1365 0.6816
-0.5963 0.1445 -38.7907
89.9600 -37.9200 -0.1101 0.3200
0.3221 0.0723 0.0076 0.1188 0.6727
-0.5946 0.1421 -38.7897
89.9800 -37.7400 0.2362 0.3146
0.2590 0.0330 -0.0191 0.1009 0.6637
-0.5928 0.1396 -38.7888
90.0000 -38.5100 -0.3503 0.2283
0.1302 -0.0126 -0.0451 0.0830 0.6544
-0.5910 0.1371 -38.7879
90.0200 -38.4100 0.2062 0.0705 -
0.0315 -0.0609 -0.0701 0.0651 0.6450
-0.5891 0.1346 -38.7869
90.0400 -39.0600 0.0537 -0.1834 -
0.1864 -0.1085 -0.0937 0.0472 0.6354
-0.5873 0.1322 -38.7860
90.0600 -39.5300 0.0979 -0.4722 -
0.2967 -0.1521 -0.1158 0.0294 0.6257
-0.5855 0.1297 -38.7850
90.0800 -40.0500 -0.2403 -0.5946 -
0.3338 -0.1884 -0.1359 0.0117 0.6158
-0.5836 0.1273 -38.7841
90.1000 -39.8200 -0.1270 -0.3961 -
0.2915 -0.2146 -0.1538 -0.0059 0.6058
-0.5817 0.1248 -38.7832
90.1200 -39.1300 0.0834 0.0186 -
0.1916 -0.2298 -0.1694 -0.0233 0.5956
-0.5798 0.1224 -38.7822

90.1400 -38.3900 0.4152 0.3451 -
0.0684 -0.2336 -0.1824 -0.0405 0.5853
-0.5779 0.1199 -38.7813
90.1600 -38.8400 -0.2724 0.4562
0.0409 -0.2264 -0.1927 -0.0574 0.5749
-0.5760 0.1175 -38.7803
90.1800 -39.0800 -0.4316 0.4570
0.1029 -0.2084 -0.2004 -0.0741 0.5643
-0.5741 0.1151 -38.7794
90.2000 -38.2200 0.4534 0.3429
0.1072 -0.1806 -0.2054 -0.0905 0.5537
-0.5721 0.1127 -38.7785
90.2200 -38.8300 0.2819 0.0114
0.0708 -0.1445 -0.2080 -0.1066 0.5429
-0.5702 0.1103 -38.7775
90.2400 -39.9700 -0.3362 -0.3859
0.0230 -0.1026 -0.2082 -0.1222 0.5321
-0.5682 0.1079 -38.7766
90.2600 -39.9500 -0.3329 -0.5143 -
0.0113 -0.0573 -0.2062 -0.1375 0.5211
-0.5662 0.1055 -38.7756
90.2800 -39.4300 -0.0753 -0.2761 -
0.0169 -0.0114 -0.2024 -0.1524 0.5101
-0.5642 0.1031 -38.7747
90.3000 -38.6200 0.3088 0.0977
0.0037 0.0328 -0.1969 -0.1668 0.4990
-0.5622 0.1007 -38.7738
90.3200 -38.6100 0.1343 0.3067
0.0393 0.0733 -0.1899 -0.1807 0.4878
-0.5602 0.0984 -38.7728
90.3400 -38.9600 -0.2396 0.2620
0.0719 0.1086 -0.1817 -0.1941 0.4765
-0.5582 0.0960 -38.7719
90.3600 -38.6500 0.2408 0.0598
0.0888 0.1383 -0.1726 -0.2071 0.4652
-0.5561 0.0937 -38.7710
90.3800 -39.1100 -0.0921 -0.1356
0.0843 0.1620 -0.1628 -0.2194 0.4538
-0.5541 0.0913 -38.7700
90.4000 -39.3600 -0.2645 -0.1687
0.0577 0.1794 -0.1525 -0.2313 0.4424
-0.5520 0.0890 -38.7691
90.4200 -38.6600 0.3222 -0.0210
0.0177 0.1901 -0.1420 -0.2426 0.4310
-0.5500 0.0866 -38.7681
90.4400 -39.0900 -0.2196 0.1397 -
0.0250 0.1946 -0.1315 -0.2533 0.4195
-0.5479 0.0843 -38.7672
90.4600 -38.6400 0.3486 0.1393 -
0.0608 0.1931 -0.1211 -0.2635 0.4080
-0.5458 0.0820 -38.7663

90.4800 -39.4000 -0.3576 0.0021 -
0.0790 0.1856 -0.1111 -0.2730 0.3964
-0.5437 0.0797 -38.7653
90.5000 -39.1800 0.1020 -0.1392 -
0.0678 0.1719 -0.1014 -0.2820 0.3849
-0.5416 0.0774 -38.7644
90.5200 -39.1800 0.0826 -0.1958 -
0.0257 0.1519 -0.0921 -0.2904 0.3733
-0.5395 0.0751 -38.7635
90.5400 -39.4800 -0.1609 -0.1171
0.0358 0.1257 -0.0832 -0.2982 0.3617
-0.5374 0.0728 -38.7625
90.5600 -39.1900 -0.1458 0.0725
0.0982 0.0940 -0.0746 -0.3054 0.3501
-0.5353 0.0705 -38.7616
90.5800 -38.5200 0.3972 0.1876
0.1419 0.0575 -0.0663 -0.3121 0.3385
-0.5331 0.0682 -38.7607
90.6000 -39.0600 -0.0914 0.0992
0.1503 0.0175 -0.0582 -0.3182 0.3270
-0.5310 0.0660 -38.7597
90.6200 -39.3800 -0.1372 -0.0419
0.1196 -0.0241 -0.0501 -0.3237 0.3154
-0.5289 0.0637 -38.7588
90.6400 -39.4700 -0.1213 -0.0641
0.0590 -0.0649 -0.0420 -0.3287 0.3039
-0.5267 0.0614 -38.7579
90.6600 -39.0900 0.3446 -0.0329 -
0.0166 -0.1022 -0.0338 -0.3331 0.2924
-0.5246 0.0592 -38.7569
90.6800 -39.7100 -0.1502 -0.0251 -
0.0894 -0.1336 -0.0255 -0.3371 0.2809
-0.5224 0.0570 -38.7560
90.7000 -39.8100 -0.2081 -0.0185 -
0.1432 -0.1570 -0.0170 -0.3405 0.2694
-0.5203 0.0547 -38.7551
90.7200 -39.3300 0.3488 -0.0251 -
0.1669 -0.1711 -0.0082 -0.3434 0.2580
-0.5181 0.0525 -38.7541
90.7400 -39.7500 -0.0536 -0.0730 -
0.1567 -0.1750 0.0009 -0.3459 0.2466
-0.5159 0.0503 -38.7532
90.7600 -39.8700 -0.2388 -0.0792 -
0.1188 -0.1685 0.0104 -0.3479 0.2353
-0.5138 0.0481 -38.7523
90.7800 -39.5500 -0.0511 0.0402 -
0.0677 -0.1523 0.0203 -0.3495 0.2240
-0.5116 0.0459 -38.7513
90.8000 -39.2000 0.0590 0.1763 -
0.0221 -0.1277 0.0304 -0.3507 0.2128
-0.5094 0.0437 -38.7504

90.8200 -38.7600 0.4784 0.1286
0.0027 -0.0965 0.0409 -0.3514 0.2016
-0.5072 0.0415 -38.7495
90.8400 -39.7800 -0.3665 -0.0497
0.0039 -0.0610 0.0515 -0.3518 0.1905
-0.5050 0.0394 -38.7485
90.8600 -39.7600 -0.3279 -0.1182 -
0.0091 -0.0236 0.0622 -0.3519 0.1795
-0.5029 0.0372 -38.7476
90.8800 -38.9700 0.4468 -0.0419 -
0.0219 0.0129 0.0729 -0.3515 0.1685
-0.5007 0.0350 -38.7467
90.9000 -39.1600 0.1339 0.0068 -
0.0222 0.0461 0.0834 -0.3509 0.1576
-0.4985 0.0329 -38.7457
90.9200 -39.6600 -0.2759 -0.0423 -
0.0032 0.0737 0.0937 -0.3499 0.1468
-0.4963 0.0307 -38.7448
90.9400 -39.6000 -0.2841 -0.0509
0.0343 0.0942 0.1035 -0.3487 0.1360
-0.4941 0.0286 -38.7439
90.9600 -38.7000 0.3724 0.0106
0.0828 0.1069 0.1128 -0.3472 0.1254
-0.4919 0.0265 -38.7429
90.9800 -38.9000 0.0603 0.0134
0.1297 0.1115 0.1213 -0.3454 0.1148
-0.4897 0.0244 -38.7420
91.0000 -39.4100 -0.3331 -0.0180
0.1614 0.1082 0.1289 -0.3434 0.1044
-0.4876 0.0223 -38.7411
91.0200 -38.7100 0.2859 0.0217
0.1684 0.0976 0.1353 -0.3411 0.0940
-0.4854 0.0202 -38.7401
91.0400 -39.1600 -0.1333 0.0888
0.1440 0.0815 0.1404 -0.3387 0.0837
-0.4832 0.0181 -38.7392
91.0600 -39.0900 -0.0353 0.0692
0.0898 0.0617 0.1441 -0.3360 0.0735
-0.4810 0.0160 -38.7383
91.0800 -39.0200 0.1820 -0.0321
0.0168 0.0408 0.1460 -0.3332 0.0634
-0.4788 0.0139 -38.7373
91.1000 -39.6200 -0.1635 -0.1017 -
0.0606 0.0210 0.1463 -0.3302 0.0534
-0.4766 0.0119 -38.7364
91.1200 -39.5500 -0.0543 -0.0485 -
0.1268 0.0047 0.1448 -0.3270 0.0436
-0.4745 0.0098 -38.7355
91.1400 -39.5700 -0.0397 0.0670 -
0.1698 -0.0064 0.1415 -0.3237 0.0338
-0.4723 0.0077 -38.7346

91.1600 -39.2600 0.3424 0.0610 -
0.1829 -0.0111 0.1363 -0.3202 0.0241
-0.4701 0.0057 -38.7336
91.1800 -39.6900 -0.0401 -0.0775 -
0.1674 -0.0092 0.1293 -0.3166 0.0146
-0.4679 0.0037 -38.7327
91.2000 -40.0600 -0.4175 -0.1411 -
0.1312 -0.0012 0.1206 -0.3128 0.0052
-0.4658 0.0016 -38.7318
91.2200 -39.2800 0.1014 0.0158 -
0.0850 0.0118 0.1104 -0.3090 -0.0042
-0.4636 -0.0004 -38.7308
91.2400 -39.1800 0.0409 0.2062 -
0.0375 0.0280 0.0986 -0.3050 -0.0134
-0.4615 -0.0024 -38.7299
91.2600 -38.7900 0.5857 0.1101
0.0058 0.0450 0.0856 -0.3008 -0.0225
-0.4593 -0.0044 -38.7290
91.2800 -40.1700 -0.6524 -0.1511
0.0449 0.0602 0.0715 -0.2966 -0.0314
-0.4571 -0.0064 -38.7281
91.3000 -39.3600 0.2176 -0.1687
0.0832 0.0709 0.0566 -0.2923 -0.0403
-0.4550 -0.0083 -38.7271
91.3200 -39.1700 0.1127 0.0178
0.1177 0.0753 0.0410 -0.2878 -0.0490
-0.4529 -0.0103 -38.7262
91.3400 -38.9200 0.2179 0.0938
0.1407 0.0719 0.0249 -0.2832 -0.0577
-0.4507 -0.0123 -38.7253
91.3600 -39.6000 -0.3307 0.0657
0.1470 0.0602 0.0088 -0.2785 -0.0662
-0.4486 -0.0142 -38.7244
91.3800 -39.1000 0.1188 0.0659
0.1331 0.0408 -0.0073 -0.2737 -0.0745
-0.4465 -0.0162 -38.7234
91.4000 -39.2900 0.1391 0.0483
0.0990 0.0150 -0.0229 -0.2688 -0.0828
-0.4444 -0.0181 -38.7225
91.4200 -39.7500 -0.1646 -0.0302
0.0511 -0.0146 -0.0378 -0.2637 -0.0910
-0.4422 -0.0201 -38.7216
91.4400 -39.5100 0.0795 -0.0859 -
0.0010 -0.0454 -0.0518 -0.2586 -0.0990
-0.4401 -0.0220 -38.7206
91.4600 -40.1100 -0.2772 -0.0333 -
0.0482 -0.0746 -0.0646 -0.2533 -0.1069
-0.4380 -0.0239 -38.7197
91.4800 -39.4700 0.3993 0.0518 -
0.0846 -0.0992 -0.0760 -0.2479 -0.1147
-0.4359 -0.0258 -38.7188

91.5000 -40.1200 -0.2298 0.0112 -
0.1096 -0.1168 -0.0858 -0.2423 -0.1224
-0.4338 -0.0277 -38.7179
91.5200 -40.0200 -0.0518 -0.0489 -
0.1226 -0.1261 -0.0937 -0.2367 -0.1300
-0.4318 -0.0296 -38.7169
91.5400 -39.8500 -0.0693 0.0179 -
0.1216 -0.1266 -0.0997 -0.2309 -0.1374
-0.4297 -0.0314 -38.7160
91.5600 -39.7500 0.0967 0.0984 -
0.1069 -0.1190 -0.1036 -0.2250 -0.1448
-0.4276 -0.0333 -38.7151
91.5800 -39.2800 0.5986 -0.0018 -
0.0788 -0.1048 -0.1055 -0.2190 -0.1520
-0.4256 -0.0352 -38.7142
91.6000 -40.4700 -0.5337 -0.1640 -
0.0388 -0.0858 -0.1053 -0.2129 -0.1591
-0.4235 -0.0370 -38.7132
91.6200 -40.3800 -0.5266 -0.1169
0.0073 -0.0640 -0.1032 -0.2067 -0.1661
-0.4215 -0.0389 -38.7123
91.6400 -38.8300 0.7182 0.0856
0.0538 -0.0412 -0.0993 -0.2004 -0.1730
-0.4194 -0.0407 -38.7114
91.6600 -39.1500 0.2459 0.1469
0.0954 -0.0190 -0.0938 -0.1940 -0.1797
-0.4174 -0.0425 -38.7105
91.6800 -39.7900 -0.3091 -0.0181
0.1267 0.0012 -0.0870 -0.1876 -0.1864
-0.4154 -0.0443 -38.7095
91.7000 -40.0200 -0.4171 -0.1269
0.1440 0.0183 -0.0790 -0.1811 -0.1929
-0.4134 -0.0462 -38.7086
91.7200 -39.8300 -0.3672 0.0157
0.1436 0.0318 -0.0702 -0.1745 -0.1993
-0.4113 -0.0480 -38.7077
91.7400 -38.7600 0.6461 0.1604
0.1197 0.0421 -0.0607 -0.1679 -0.2056
-0.4093 -0.0497 -38.7068
91.7600 -39.6500 -0.2361 0.0526
0.0699 0.0496 -0.0508 -0.1612 -0.2118
-0.4074 -0.0515 -38.7059
91.7800 -39.9400 -0.2864 -0.1002
0.0009 0.0551 -0.0407 -0.1545 -0.2179
-0.4054 -0.0533 -38.7049
91.8000 -39.6800 -0.0073 -0.0710 -
0.0737 0.0600 -0.0307 -0.1478 -0.2238
-0.4034 -0.0551 -38.7040
91.8200 -39.7100 -0.0262 0.0514 -
0.1359 0.0652 -0.0209 -0.1412 -0.2297
-0.4014 -0.0568 -38.7031

91.8400 -39.1900 0.3613 0.0299 -
0.1713 0.0715 -0.0115 -0.1345 -0.2354
-0.3995 -0.0586 -38.7022
91.8600 -39.8500 -0.1330 -0.1115 -
0.1746 0.0793 -0.0026 -0.1279 -0.2410
-0.3975 -0.0603 -38.7012
91.8800 -39.8800 -0.2213 -0.1390 -
0.1467 0.0880 0.0057 -0.1214 -0.2465
-0.3956 -0.0620 -38.7003
91.9000 -39.4300 0.0710 0.0016 -
0.0962 0.0969 0.0133 -0.1149 -0.2519
-0.3936 -0.0637 -38.6994
91.9200 -39.2000 0.1355 0.1547 -
0.0361 0.1050 0.0201 -0.1085 -0.2572
-0.3917 -0.0654 -38.6985
91.9400 -39.3500 -0.1676 0.1473
0.0236 0.1107 0.0261 -0.1022 -0.2624
-0.3898 -0.0672 -38.6976
91.9600 -39.0400 0.3049 -0.0301
0.0760 0.1125 0.0313 -0.0961 -0.2674
-0.3879 -0.0688 -38.6966
91.9800 -39.6500 -0.2247 -0.1881
0.1174 0.1085 0.0357 -0.0901 -0.2724
-0.3859 -0.0705 -38.6957
92.0000 -39.6300 -0.2670 -0.1247
0.1484 0.0972 0.0394 -0.0843 -0.2772
-0.3840 -0.0722 -38.6948
92.0200 -38.8300 0.3735 0.0871
0.1686 0.0778 0.0425 -0.0787 -0.2819
-0.3822 -0.0739 -38.6939
92.0400 -39.1800 -0.1342 0.1536
0.1758 0.0506 0.0453 -0.0734 -0.2865
-0.3803 -0.0755 -38.6930
92.0600 -39.0300 0.2057 0.0313
0.1649 0.0172 0.0477 -0.0682 -0.2910
-0.3784 -0.0772 -38.6920
92.0800 -39.5500 -0.1953 -0.0848
0.1310 -0.0199 0.0500 -0.0633 -0.2953
-0.3765 -0.0788 -38.6911
92.1000 -39.7700 -0.3361 -0.0400
0.0730 -0.0583 0.0523 -0.0587 -0.2996
-0.3747 -0.0804 -38.6902
92.1200 -39.8200 -0.3846 0.1460 -
0.0048 -0.0949 0.0545 -0.0544 -0.3037
-0.3728 -0.0821 -38.6893
92.1400 -38.6700 0.7872 0.2310 -
0.0905 -0.1268 0.0568 -0.0504 -0.3077
-0.3709 -0.0837 -38.6884
92.1600 -39.0300 0.6656 0.0554 -
0.1681 -0.1512 0.0592 -0.0467 -0.3116
-0.3691 -0.0853 -38.6874

92.1800 -40.8200 -0.7655 -0.2360 -
0.2179 -0.1659 0.0618 -0.0434 -0.3154
-0.3673 -0.0869 -38.6865
92.2000 -40.9100 -0.7614 -0.3386 -
0.2221 -0.1690 0.0645 -0.0404 -0.3191
-0.3654 -0.0885 -38.6856
92.2200 -39.8600 0.0010 -0.1335 -
0.1734 -0.1604 0.0674 -0.0377 -0.3227
-0.3636 -0.0900 -38.6847
92.2400 -38.6600 0.8082 0.1316 -
0.0875 -0.1414 0.0703 -0.0353 -0.3261
-0.3618 -0.0916 -38.6838
92.2600 -39.4300 -0.0369 0.1919
0.0122 -0.1141 0.0733 -0.0333 -0.3294
-0.3600 -0.0932 -38.6828
92.2800 -39.7000 -0.3646 0.0937
0.0976 -0.0810 0.0761 -0.0317 -0.3327
-0.3582 -0.0947 -38.6819
92.3000 -39.3500 0.0007 0.0206
0.1444 -0.0445 0.0786 -0.0304 -0.3358
-0.3563 -0.0962 -38.6810
92.3200 -39.1600 0.1478 0.0177
0.1422 -0.0074 0.0808 -0.0294 -0.3388
-0.3545 -0.0978 -38.6801
92.3400 -39.4500 -0.0686 0.0137
0.1009 0.0281 0.0825 -0.0288 -0.3417
-0.3528 -0.0993 -38.6792
92.3600 -39.4200 -0.1176 0.0206
0.0437 0.0597 0.0836 -0.0286 -0.3445
-0.3510 -0.1008 -38.6783
92.3800 -39.3000 0.0202 0.0493 -
0.0041 0.0857 0.0840 -0.0286 -0.3472
-0.3492 -0.1023 -38.6773
92.4000 -39.2800 0.0669 -0.0312 -
0.0217 0.1053 0.0837 -0.0290 -0.3497
-0.3474 -0.1038 -38.6764
92.4200 -39.2300 0.4161 -0.2230 -
0.0064 0.1185 0.0825 -0.0298 -0.3522
-0.3456 -0.1053 -38.6755
92.4400 -40.2700 -0.8199 -0.2157
0.0276 0.1258 0.0802 -0.0308 -0.3546
-0.3438 -0.1068 -38.6746
92.4600 -39.0100 0.2136 0.0949
0.0590 0.1283 0.0770 -0.0321 -0.3568
-0.3420 -0.1082 -38.6737
92.4800 -38.1300 0.8946 0.3450
0.0633 0.1272 0.0727 -0.0337 -0.3590
-0.3403 -0.1097 -38.6728
92.5000 -39.0100 0.0577 0.2198
0.0256 0.1237 0.0674 -0.0355 -0.3611
-0.3385 -0.1111 -38.6718

92.5200 -40.3300 -0.7984 -0.0501 -
0.0401 0.1186 0.0611 -0.0375 -0.3631
-0.3367 -0.1126 -38.6709
92.5400 -39.2600 0.3578 -0.1639 -
0.1098 0.1120 0.0539 -0.0398 -0.3650
-0.3349 -0.1140 -38.6700
92.5600 -39.5500 0.0470 -0.1209 -
0.1586 0.1038 0.0459 -0.0423 -0.3668
-0.3332 -0.1154 -38.6691
92.5800 -39.5600 0.0788 -0.1113 -
0.1668 0.0939 0.0372 -0.0449 -0.3685
-0.3314 -0.1169 -38.6682
92.6000 -39.8000 -0.0862 -0.1250 -
0.1268 0.0818 0.0279 -0.0476 -0.3701
-0.3296 -0.1183 -38.6673
92.6200 -39.5100 0.0311 -0.0513 -
0.0490 0.0673 0.0182 -0.0505 -0.3717
-0.3279 -0.1197 -38.6664
92.6400 -39.6000 -0.1917 0.0825
0.0464 0.0505 0.0083 -0.0535 -0.3731
-0.3261 -0.1210 -38.6654
92.6600 -39.0600 0.2143 0.1613
0.1363 0.0315 -0.0017 -0.0565 -0.3745
-0.3243 -0.1224 -38.6645
92.6800 -39.0200 0.1573 0.0991
0.1945 0.0101 -0.0116 -0.0596 -0.3758
-0.3225 -0.1238 -38.6636
92.7000 -39.6000 -0.2375 -0.0359
0.2037 -0.0137 -0.0212 -0.0627 -0.3770
-0.3207 -0.1252 -38.6627
92.7200 -39.6600 -0.1136 -0.0482
0.1609 -0.0391 -0.0302 -0.0658 -0.3781
-0.3190 -0.1265 -38.6618
92.7400 -39.3900 0.1371 0.0586
0.0797 -0.0646 -0.0384 -0.0689 -0.3792
-0.3172 -0.1279 -38.6609
92.7600 -39.3700 0.3182 0.0807 -
0.0155 -0.0886 -0.0458 -0.0719 -0.3802
-0.3154 -0.1292 -38.6600
92.7800 -39.9300 -0.0826 -0.0410 -
0.0994 -0.1092 -0.0522 -0.0748 -0.3811
-0.3136 -0.1305 -38.6591
92.8000 -40.3400 -0.2488 -0.1373 -
0.1530 -0.1246 -0.0575 -0.0777 -0.3820
-0.3118 -0.1318 -38.6581
92.8200 -40.0500 -0.0801 -0.0649 -
0.1676 -0.1328 -0.0615 -0.0804 -0.3828
-0.3100 -0.1331 -38.6572
92.8400 -39.7000 0.2273 0.0613 -
0.1453 -0.1328 -0.0643 -0.0829 -0.3835
-0.3082 -0.1344 -38.6563

92.8600	-39.8000	0.0423	0.0271	-
0.0935	-0.1248	-0.0658	-0.0854	-0.3842
	-0.3064	-0.1357	-38.6554	
92.8800	-39.9400	-0.0605	-0.1182	-
0.0251	-0.1094	-0.0661	-0.0876	-0.3848
	-0.3046	-0.1370	-38.6545	
92.9000	-40.1100	-0.2677	-0.1210	
0.0427	-0.0875	-0.0652	-0.0896	-0.3854
	-0.3027	-0.1383	-38.6536	
92.9200	-39.5600	0.0202	0.0780	
0.0932	-0.0608	-0.0634	-0.0915	-0.3859
	-0.3009	-0.1395	-38.6527	
92.9400	-38.9600	0.3381	0.2402	
0.1178	-0.0313	-0.0606	-0.0931	-0.3863
	-0.2991	-0.1408	-38.6518	
92.9600	-39.5000	-0.0922	0.1287	
0.1129	-0.0014	-0.0571	-0.0945	-0.3867
	-0.2972	-0.1420	-38.6509	
92.9800	-39.6300	-0.0187	-0.1251	
0.0870	0.0262	-0.0528	-0.0957	-0.3871
	-0.2954	-0.1433	-38.6499	
93.0000	-39.8500	-0.1579	-0.2003	
0.0564	0.0494	-0.0480	-0.0966	-0.3874
	-0.2935	-0.1445	-38.6490	
93.0200	-39.4400	0.1675	-0.0741	
0.0353	0.0665	-0.0427	-0.0973	-0.3876
	-0.2916	-0.1457	-38.6481	
93.0400	-39.3700	0.1177	0.0342	
0.0285	0.0766	-0.0373	-0.0977	-0.3878
	-0.2898	-0.1469	-38.6472	
93.0600	-39.5800	-0.2320	0.0463	
0.0309	0.0804	-0.0317	-0.0978	-0.3880
	-0.2879	-0.1482	-38.6463	
93.0800	-39.3600	0.0347	0.0463	
0.0241	0.0791	-0.0261	-0.0977	-0.3881
	-0.2860	-0.1493	-38.6454	
93.1000	-39.5100	-0.0495	0.1192	-
0.0084	0.0747	-0.0207	-0.0974	-0.3882
	-0.2841	-0.1505	-38.6445	
93.1200	-39.2000	0.1855	0.1937	-
0.0612	0.0691	-0.0156	-0.0967	-0.3882
	-0.2821	-0.1517	-38.6436	
93.1400	-39.4300	0.0784	0.0977	-
0.1175	0.0636	-0.0109	-0.0958	-0.3882
	-0.2802	-0.1529	-38.6427	
93.1600	-39.9100	-0.1974	-0.1505	-
0.1514	0.0594	-0.0066	-0.0946	-0.3882
	-0.2783	-0.1540	-38.6418	
93.1800	-39.8600	0.0242	-0.3112	-
0.1377	0.0563	-0.0030	-0.0931	-0.3882
	-0.2763	-0.1552	-38.6409	

93.2000 -40.0500 -0.3732 -0.1557 -
0.0677 0.0539 0.0002 -0.0914 -0.3881
-0.2743 -0.1563 -38.6399
93.2200 -38.7900 0.5625 0.1201
0.0365 0.0508 0.0029 -0.0894 -0.3880
-0.2724 -0.1575 -38.6390
93.2400 -39.5700 -0.3377 0.1442
0.1368 0.0454 0.0051 -0.0872 -0.3878
-0.2704 -0.1586 -38.6381
93.2600 -39.1800 -0.0346 0.0196
0.2006 0.0361 0.0069 -0.0848 -0.3877
-0.2684 -0.1597 -38.6372
93.2800 -39.3200 0.0083 -0.0043
0.2119 0.0222 0.0083 -0.0821 -0.3875
-0.2664 -0.1608 -38.6363
93.3000 -39.1800 0.1068 0.0540
0.1721 0.0040 0.0094 -0.0793 -0.3872
-0.2643 -0.1619 -38.6354
93.3200 -39.5300 -0.1325 0.0668
0.0982 -0.0172 0.0102 -0.0762 -0.3870
-0.2623 -0.1630 -38.6345
93.3400 -39.5200 0.0636 -0.0207
0.0155 -0.0396 0.0109 -0.0730 -0.3867
-0.2602 -0.1641 -38.6336
93.3600 -39.7200 0.0247 -0.1373 -
0.0548 -0.0607 0.0113 -0.0696 -0.3865
-0.2582 -0.1652 -38.6327
93.3800 -40.0000 -0.1731 -0.1713 -
0.1032 -0.0781 0.0116 -0.0661 -0.3862
-0.2561 -0.1662 -38.6318
93.4000 -39.7000 0.1351 -0.0735 -
0.1308 -0.0889 0.0117 -0.0625 -0.3858
-0.2540 -0.1673 -38.6309
93.4200 -39.8600 -0.1869 0.0929 -
0.1492 -0.0909 0.0118 -0.0588 -0.3855
-0.2519 -0.1683 -38.6300
93.4400 -39.5300 0.0431 0.2090 -
0.1712 -0.0826 0.0119 -0.0551 -0.3852
-0.2497 -0.1694 -38.6291
93.4600 -39.4700 0.1300 0.1441 -
0.1970 -0.0637 0.0120 -0.0513 -0.3848
-0.2476 -0.1704 -38.6282
93.4800 -39.8500 0.0436 -0.0762 -
0.2144 -0.0360 0.0122 -0.0475 -0.3844
-0.2454 -0.1714 -38.6273
93.5000 -40.1300 -0.1225 -0.2416 -
0.2069 -0.0022 0.0124 -0.0438 -0.3840
-0.2432 -0.1725 -38.6263
93.5200 -40.0400 -0.4058 -0.1625 -
0.1614 0.0343 0.0126 -0.0400 -0.3836
-0.2411 -0.1735 -38.6254

93.5400 -38.8800 0.5632 0.0607 -
0.0766 0.0699 0.0129 -0.0364 -0.3832
-0.2388 -0.1745 -38.6245
93.5600 -39.3200 -0.1078 0.1193
0.0420 0.1007 0.0133 -0.0328 -0.3828
-0.2366 -0.1755 -38.6236
93.5800 -39.2800 -0.2469 0.0045
0.1789 0.1237 0.0136 -0.0293 -0.3824
-0.2344 -0.1764 -38.6227
93.6000 -38.8900 0.1269 -0.1005
0.3078 0.1363 0.0140 -0.0259 -0.3820
-0.2321 -0.1774 -38.6218
93.6200 -39.1600 -0.0971 -0.0691
0.3988 0.1366 0.0143 -0.0226 -0.3815
-0.2298 -0.1784 -38.6209
93.6400 -38.7400 0.0463 0.0771
0.4243 0.1238 0.0146 -0.0195 -0.3811
-0.2275 -0.1793 -38.6200
93.6600 -38.7000 -0.0327 0.2158
0.3698 0.0983 0.0149 -0.0166 -0.3807
-0.2252 -0.1803 -38.6191
93.6800 -38.8900 -0.0531 0.2473
0.2390 0.0629 0.0152 -0.0139 -0.3802
-0.2229 -0.1812 -38.6182
93.7000 -38.9800 0.1790 0.1406
0.0549 0.0216 0.0157 -0.0114 -0.3798
-0.2206 -0.1822 -38.6173
93.7200 -39.7500 -0.1153 -0.0407 -
0.1425 -0.0212 0.0163 -0.0090 -0.3793
-0.2182 -0.1831 -38.6164
93.7400 -39.8200 0.1486 -0.1867 -
0.3074 -0.0611 0.0171 -0.0069 -0.3789
-0.2158 -0.1840 -38.6155
93.7600 -40.2800 -0.1861 -0.2336 -
0.3987 -0.0938 0.0182 -0.0051 -0.3784
-0.2134 -0.1849 -38.6146
93.7800 -39.6300 0.4070 -0.2024 -
0.3943 -0.1160 0.0195 -0.0035 -0.3779
-0.2110 -0.1858 -38.6137
93.8000 -40.4800 -0.6659 -0.0173 -
0.3061 -0.1266 0.0213 -0.0021 -0.3775
-0.2086 -0.1867 -38.6128
93.8200 -38.7900 0.6510 0.2247 -
0.1692 -0.1260 0.0235 -0.0010 -0.3770
-0.2061 -0.1876 -38.6119
93.8400 -39.4000 -0.0922 0.1951 -
0.0234 -0.1154 0.0260 -0.0002 -0.3766
-0.2036 -0.1885 -38.6110
93.8600 -39.6500 -0.3484 -0.0283
0.1003 -0.0969 0.0288 0.0004 -0.3761
-0.2011 -0.1894 -38.6101

93.8800	-39.3300	-0.0580	-0.1334	
0.1858	-0.0730	0.0320	0.0006	-0.3757
	-0.1986	-0.1902	-38.6092	
93.9000	-38.9300	0.2447	-0.0733	
0.2302	-0.0461	0.0354	0.0007	-0.3752
	-0.1961	-0.1911	-38.6083	
93.9200	-39.1900	-0.1078	0.0028	
0.2374	-0.0185	0.0390	0.0004	-0.3747
	-0.1936	-0.1919	-38.6074	
93.9400	-39.1500	-0.0810	0.0526	
0.2110	0.0077	0.0426	-0.0001	-0.3743
	-0.1910	-0.1928	-38.6065	
93.9600	-38.9800	0.0647	0.0721	
0.1577	0.0308	0.0462	-0.0008	-0.3738
	-0.1884	-0.1936	-38.6056	
93.9800	-39.2800	-0.1643	0.0528	
0.0878	0.0496	0.0497	-0.0018	-0.3733
	-0.1858	-0.1945	-38.6047	
94.0000	-39.0300	0.1959	-0.0180	
0.0159	0.0638	0.0529	-0.0031	-0.3729
	-0.1832	-0.1953	-38.6038	
94.0200	-39.5500	-0.1145	-0.1197	-
0.0433	0.0733	0.0557	-0.0046	-0.3724
	-0.1806	-0.1961	-38.6029	
94.0400	-39.5300	-0.0736	-0.1148	-
0.0812	0.0782	0.0580	-0.0063	-0.3719
	-0.1779	-0.1969	-38.6020	
94.0600	-39.3000	-0.0426	0.0356	-
0.0957	0.0788	0.0598	-0.0082	-0.3715
	-0.1752	-0.1977	-38.6011	
94.0800	-39.3100	-0.1309	0.1994	-
0.0926	0.0757	0.0609	-0.0103	-0.3710
	-0.1725	-0.1985	-38.6002	
94.1000	-38.6800	0.4281	0.1705	-
0.0804	0.0695	0.0612	-0.0126	-0.3705
	-0.1698	-0.1993	-38.5993	
94.1200	-39.7700	-0.3969	-0.0542	-
0.0629	0.0611	0.0608	-0.0151	-0.3700
	-0.1671	-0.2000	-38.5984	
94.1400	-39.3700	0.1571	-0.1762	-
0.0381	0.0512	0.0596	-0.0177	-0.3695
	-0.1644	-0.2008	-38.5975	
94.1600	-39.3000	-0.0554	-0.0912	-
0.0016	0.0408	0.0576	-0.0205	-0.3690
	-0.1616	-0.2016	-38.5966	
94.1800	-39.2300	-0.0857	0.0472	
0.0458	0.0304	0.0548	-0.0234	-0.3685
	-0.1588	-0.2023	-38.5957	
94.2000	-38.8300	0.2701	0.0744	
0.0959	0.0200	0.0514	-0.0264	-0.3680
	-0.1560	-0.2031	-38.5948	

94.2200 -39.0600 0.0432 -0.0378
0.1343 0.0096 0.0473 -0.0294 -0.3675
-0.1532 -0.2038 -38.5939
94.2400 -39.5100 -0.2724 -0.1114
0.1431 -0.0004 0.0428 -0.0326 -0.3670
-0.1504 -0.2045 -38.5930
94.2600 -39.2500 -0.0684 0.0015
0.1110 -0.0094 0.0378 -0.0358 -0.3664
-0.1475 -0.2053 -38.5921
94.2800 -38.8800 0.2629 0.1890
0.0416 -0.0170 0.0326 -0.0390 -0.3659
-0.1447 -0.2060 -38.5912
94.3000 -39.1100 0.0163 0.1745 -
0.0487 -0.0227 0.0272 -0.0422 -0.3653
-0.1418 -0.2067 -38.5903
94.3200 -39.4200 0.2321 -0.0904 -
0.1332 -0.0262 0.0217 -0.0455 -0.3647
-0.1389 -0.2074 -38.5894
94.3400 -40.3000 -0.5243 -0.2571 -
0.1845 -0.0276 0.0164 -0.0487 -0.3641
-0.1360 -0.2081 -38.5885
94.3600 -39.3600 0.2806 -0.1136 -
0.1822 -0.0271 0.0112 -0.0519 -0.3635
-0.1331 -0.2088 -38.5876
94.3800 -39.2300 0.2945 0.0715 -
0.1279 -0.0259 0.0063 -0.0550 -0.3629
-0.1302 -0.2095 -38.5867
94.4000 -39.3700 -0.0907 0.0868 -
0.0406 -0.0250 0.0018 -0.0581 -0.3623
-0.1272 -0.2102 -38.5858
94.4200 -39.6000 -0.2362 0.0262
0.0569 -0.0255 -0.0023 -0.0610 -0.3616
-0.1242 -0.2108 -38.5849
94.4400 -39.1700 0.0350 0.0049
0.1400 -0.0283 -0.0058 -0.0638 -0.3610
-0.1213 -0.2115 -38.5840
94.4600 -38.9400 0.2132 0.0007
0.1918 -0.0345 -0.0088 -0.0665 -0.3603
-0.1183 -0.2122 -38.5831
94.4800 -39.4200 -0.2216 0.0363
0.2011 -0.0441 -0.0113 -0.0691 -0.3596
-0.1153 -0.2128 -38.5822
94.5000 -39.1900 -0.1107 0.1296
0.1608 -0.0558 -0.0132 -0.0714 -0.3589
-0.1122 -0.2134 -38.5814
94.5200 -38.9400 0.3038 0.1794
0.0757 -0.0680 -0.0147 -0.0736 -0.3582
-0.1092 -0.2141 -38.5805
94.5400 -39.7700 -0.2768 0.0803 -
0.0365 -0.0781 -0.0158 -0.0756 -0.3575
-0.1062 -0.2147 -38.5796

94.5600	-39.4900	0.1213	-0.1058	-
0.1434	-0.0841	-0.0166	-0.0774	-0.3567
	-0.1031	-0.2153	-38.5787	
94.5800	-39.6800	0.2503	-0.2489	-
0.2149	-0.0837	-0.0171	-0.0789	-0.3559
	-0.1000	-0.2160	-38.5778	
94.6000	-40.1400	-0.3007	-0.2892	-
0.2281	-0.0752	-0.0175	-0.0802	-0.3552
	-0.0970	-0.2166	-38.5769	
94.6200	-39.9100	-0.1371	-0.1680	-
0.1809	-0.0581	-0.0177	-0.0813	-0.3544
	-0.0939	-0.2172	-38.5760	
94.6400	-39.1200	0.2796	0.0693	-
0.0971	-0.0335	-0.0179	-0.0821	-0.3536
	-0.0908	-0.2178	-38.5751	
94.6600	-39.2300	-0.1188	0.2455	-
0.0092	-0.0030	-0.0182	-0.0827	-0.3527
	-0.0876	-0.2184	-38.5742	
94.6800	-39.0200	0.0442	0.2611	
0.0561	0.0315	-0.0186	-0.0830	-0.3519
	-0.0845	-0.2190	-38.5733	
94.7000	-38.9300	0.1005	0.1297	
0.0871	0.0676	-0.0193	-0.0830	-0.3511
	-0.0814	-0.2195	-38.5724	
94.7200	-39.2500	-0.1354	-0.0802	
0.0885	0.1025	-0.0202	-0.0828	-0.3502
	-0.0782	-0.2201	-38.5715	
94.7400	-39.1200	0.2859	-0.2445	
0.0747	0.1333	-0.0214	-0.0823	-0.3493
	-0.0751	-0.2207	-38.5706	
94.7600	-39.5600	-0.2630	-0.2286	
0.0612	0.1571	-0.0229	-0.0815	-0.3484
	-0.0719	-0.2212	-38.5697	
94.7800	-39.2400	-0.1471	-0.0113	
0.0590	0.1717	-0.0248	-0.0805	-0.3475
	-0.0688	-0.2218	-38.5688	
94.8000	-38.5300	0.3717	0.1886	
0.0727	0.1757	-0.0271	-0.0792	-0.3466
	-0.0656	-0.2223	-38.5680	
94.8200	-38.7100	0.1129	0.1457	
0.0999	0.1687	-0.0298	-0.0776	-0.3456
	-0.0624	-0.2229	-38.5671	
94.8400	-39.4700	-0.3831	-0.0586	
0.1285	0.1516	-0.0327	-0.0758	-0.3447
	-0.0592	-0.2234	-38.5662	
94.8600	-38.8400	0.3470	-0.1666	
0.1408	0.1263	-0.0358	-0.0737	-0.3437
	-0.0560	-0.2240	-38.5653	
94.8800	-39.3300	-0.2832	-0.0342	
0.1241	0.0948	-0.0390	-0.0714	-0.3428
	-0.0528	-0.2245	-38.5644	

94.9000	-39.0100	0.0225	0.2426	
0.0725	0.0597	-0.0420	-0.0689	-0.3418
	-0.0495	-0.2250	-38.5635	
94.9200	-38.8700	0.0528	0.4133	-
0.0108	0.0236	-0.0447	-0.0661	-0.3408
	-0.0463	-0.2255	-38.5626	
94.9400	-38.9400	0.1694	0.2774	-
0.1133	-0.0117	-0.0471	-0.0632	-0.3398
	-0.0431	-0.2260	-38.5617	
94.9600	-39.6500	-0.0005	-0.0910	-
0.2113	-0.0442	-0.0489	-0.0600	-0.3388
	-0.0398	-0.2265	-38.5608	
94.9800	-40.4500	-0.3468	-0.4425	-
0.2774	-0.0723	-0.0500	-0.0567	-0.3378
	-0.0366	-0.2270	-38.5599	
95.0000	-39.9600	0.1590	-0.5863	-
0.2866	-0.0941	-0.0503	-0.0533	-0.3367
	-0.0333	-0.2275	-38.5590	
95.0200	-39.9100	0.2506	-0.5494	-
0.2242	-0.1083	-0.0496	-0.0496	-0.3357
	-0.0300	-0.2280	-38.5582	
95.0400	-40.1000	-0.2776	-0.3735	-
0.0988	-0.1154	-0.0480	-0.0459	-0.3347
	-0.0267	-0.2285	-38.5573	
95.0600	-39.2100	0.0663	-0.0102	
0.0551	-0.1166	-0.0452	-0.0421	-0.3336
	-0.0235	-0.2289	-38.5564	
95.0800	-38.8100	-0.1593	0.4631	
0.1944	-0.1130	-0.0412	-0.0382	-0.3326
	-0.0202	-0.2294	-38.5555	
95.1000	-38.0500	0.2219	0.7630	
0.2812	-0.1063	-0.0361	-0.0342	-0.3315
	-0.0169	-0.2299	-38.5546	
95.1200	-38.0200	0.2401	0.6981	
0.2875	-0.0975	-0.0298	-0.0301	-0.3304
	-0.0136	-0.2303	-38.5537	
95.1400	-39.1200	-0.4013	0.3386	
0.2128	-0.0870	-0.0225	-0.0260	-0.3294
	-0.0103	-0.2308	-38.5528	
95.1600	-38.9900	0.2229	-0.0635	
0.0860	-0.0745	-0.0142	-0.0220	-0.3283
	-0.0069	-0.2312	-38.5519	
95.1800	-39.5600	0.0654	-0.3631	-
0.0525	-0.0596	-0.0051	-0.0179	-0.3273
	-0.0036	-0.2317	-38.5510	
95.2000	-40.0500	-0.2465	-0.5382	-
0.1638	-0.0421	0.0045	-0.0138	-0.3262
	-0.0003	-0.2321	-38.5501	
95.2200	-39.7400	0.2178	-0.5688	-
0.2208	-0.0219	0.0147	-0.0098	-0.3251
	0.0031	-0.2325	-38.5493	

95.2400 -39.9500 -0.2536 -0.4551 -
0.2107 0.0005 0.0250 -0.0058 -0.3241
0.0064 -0.2329 -38.5484
95.2600 -39.1800 0.1306 -0.2040 -
0.1431 0.0238 0.0354 -0.0019 -0.3230
0.0097 -0.2334 -38.5475
95.2800 -38.9800 0.0405 0.0821 -
0.0455 0.0465 0.0456 0.0019 -0.3220
0.0131 -0.2338 -38.5466
95.3000 -38.5500 0.0980 0.2835
0.0503 0.0672 0.0554 0.0057 -0.3210
0.0165 -0.2342 -38.5457
95.3200 -38.3900 -0.0801 0.3659
0.1176 0.0846 0.0647 0.0093 -0.3199
0.0198 -0.2346 -38.5448
95.3400 -38.2900 0.1052 0.3140
0.1434 0.0975 0.0733 0.0127 -0.3189
0.0232 -0.2350 -38.5439
95.3600 -38.5900 0.1533 0.1043
0.1328 0.1049 0.0811 0.0161 -0.3179
0.0266 -0.2354 -38.5430
95.3800 -38.8400 0.0336 -0.1814
0.1027 0.1060 0.0879 0.0192 -0.3169
0.0299 -0.2358 -38.5422
95.4000 -39.4600 -0.3855 -0.2997
0.0737 0.1002 0.0936 0.0222 -0.3159
0.0333 -0.2362 -38.5413
95.4200 -38.6600 0.2806 -0.1569
0.0607 0.0876 0.0981 0.0250 -0.3149
0.0367 -0.2365 -38.5404
95.4400 -38.7500 -0.0053 0.0193
0.0654 0.0690 0.1014 0.0276 -0.3139
0.0401 -0.2369 -38.5395
95.4600 -38.5200 0.1427 0.0751
0.0763 0.0461 0.1032 0.0300 -0.3129
0.0435 -0.2373 -38.5386
95.4800 -38.8800 -0.1168 0.0605
0.0754 0.0212 0.1037 0.0322 -0.3120
0.0469 -0.2376 -38.5377
95.5000 -38.8800 -0.1127 0.0554
0.0509 -0.0026 0.1028 0.0342 -0.3110
0.0503 -0.2380 -38.5368
95.5200 -38.6900 0.1551 0.0760 -
0.0005 -0.0230 0.1005 0.0359 -0.3101
0.0537 -0.2384 -38.5360
95.5400 -38.8400 0.0917 0.0808 -
0.0687 -0.0377 0.0969 0.0374 -0.3092
0.0571 -0.2387 -38.5351
95.5600 -39.1200 -0.0920 0.0562 -
0.1339 -0.0457 0.0920 0.0386 -0.3083
0.0605 -0.2390 -38.5342

95.5800 -38.9700 0.1207 -0.0144 -
0.1768 -0.0459 0.0860 0.0396 -0.3074
0.0639 -0.2394 -38.5333
95.6000 -39.2000 0.0164 -0.1445 -
0.1797 -0.0380 0.0788 0.0403 -0.3065
0.0673 -0.2397 -38.5324
95.6200 -39.3000 -0.0067 -0.2465 -
0.1365 -0.0223 0.0707 0.0408 -0.3056
0.0707 -0.2401 -38.5315
95.6400 -39.3500 -0.2280 -0.1960 -
0.0559 -0.0006 0.0617 0.0410 -0.3048
0.0742 -0.2404 -38.5306
95.6600 -38.7500 0.0585 0.0018
0.0407 0.0247 0.0520 0.0409 -0.3039
0.0776 -0.2407 -38.5298
95.6800 -38.2800 0.3286 0.1736
0.1270 0.0508 0.0417 0.0406 -0.3031
0.0810 -0.2410 -38.5289
95.7000 -38.5800 -0.1348 0.2117
0.1755 0.0749 0.0310 0.0400 -0.3023
0.0844 -0.2413 -38.5280
95.7200 -38.7000 -0.2338 0.1874
0.1718 0.0942 0.0201 0.0392 -0.3015
0.0879 -0.2416 -38.5271
95.7400 -38.4400 0.1300 0.1380
0.1241 0.1064 0.0092 0.0381 -0.3007
0.0913 -0.2420 -38.5262
95.7600 -38.5600 0.3083 0.0058
0.0549 0.1104 -0.0016 0.0368 -0.2999
0.0947 -0.2423 -38.5253
95.7800 -39.4700 -0.3984 -0.1675 -
0.0087 0.1056 -0.0120 0.0352 -0.2991
0.0982 -0.2426 -38.5245
95.8000 -38.8700 0.2596 -0.2311 -
0.0460 0.0925 -0.0219 0.0334 -0.2983
0.1016 -0.2428 -38.5236
95.8200 -39.1900 -0.2023 -0.1289 -
0.0485 0.0720 -0.0310 0.0314 -0.2976
0.1050 -0.2431 -38.5227
95.8400 -38.7200 0.1529 0.0552 -
0.0226 0.0458 -0.0391 0.0291 -0.2968
0.1085 -0.2434 -38.5218
95.8600 -38.8400 -0.0536 0.2020
0.0119 0.0162 -0.0462 0.0267 -0.2961
0.1119 -0.2437 -38.5209
95.8800 -38.5800 0.1300 0.1988
0.0358 -0.0145 -0.0520 0.0240 -0.2953
0.1153 -0.2440 -38.5200
95.9000 -39.0100 0.0027 0.0247
0.0383 -0.0437 -0.0566 0.0211 -0.2946
0.1188 -0.2443 -38.5192

95.9200 -39.2800 -0.0885 -0.1710
0.0184 -0.0696 -0.0596 0.0180 -0.2938
0.1222 -0.2445 -38.5183
95.9400 -39.5400 -0.2377 -0.1522 -
0.0172 -0.0908 -0.0612 0.0147 -0.2931
0.1257 -0.2448 -38.5174
95.9600 -39.0700 0.0133 0.0594 -
0.0569 -0.1063 -0.0611 0.0112 -0.2924
0.1291 -0.2451 -38.5165
95.9800 -38.5400 0.4963 0.1655 -
0.0920 -0.1158 -0.0593 0.0076 -0.2916
0.1325 -0.2453 -38.5156
96.0000 -39.4100 -0.2574 0.0119 -
0.1114 -0.1197 -0.0559 0.0038 -0.2909
0.1360 -0.2456 -38.5148
96.0200 -39.5300 -0.1648 -0.1652 -
0.1044 -0.1185 -0.0507 -0.0001 -0.2901
0.1394 -0.2458 -38.5139
96.0400 -39.1300 0.1411 -0.1519 -
0.0638 -0.1134 -0.0439 -0.0041 -0.2894
0.1429 -0.2461 -38.5130
96.0600 -39.1800 -0.1027 -0.0050
0.0047 -0.1050 -0.0357 -0.0083 -0.2886
0.1463 -0.2463 -38.5121
96.0800 -38.7000 0.1352 0.0886
0.0825 -0.0944 -0.0260 -0.0126 -0.2879
0.1497 -0.2465 -38.5112
96.1000 -38.7800 -0.0099 0.0563
0.1442 -0.0825 -0.0152 -0.0169 -0.2871
0.1532 -0.2468 -38.5103
96.1200 -38.8400 -0.1149 0.0470
0.1661 -0.0695 -0.0035 -0.0213 -0.2863
0.1566 -0.2470 -38.5095
96.1400 -38.6800 -0.0488 0.1652
0.1358 -0.0553 0.0088 -0.0258 -0.2855
0.1601 -0.2473 -38.5086
96.1600 -38.3900 0.3223 0.2269
0.0612 -0.0393 0.0216 -0.0302 -0.2847
0.1635 -0.2475 -38.5077
96.1800 -38.9600 -0.1090 0.0380 -
0.0371 -0.0209 0.0344 -0.0348 -0.2838
0.1670 -0.2477 -38.5068
96.2000 -39.2000 0.1055 -0.2665 -
0.1295 0.0000 0.0468 -0.0393 -0.2830
0.1704 -0.2479 -38.5059
96.2200 -39.7800 -0.3241 -0.3134 -
0.1879 0.0237 0.0587 -0.0438 -0.2821
0.1738 -0.2481 -38.5051
96.2400 -38.9400 0.0857 -0.0388 -
0.1986 0.0497 0.0697 -0.0483 -0.2812
0.1773 -0.2484 -38.5042

96.2600 -38.4600 0.3201 0.1989 -
0.1684 0.0775 0.0794 -0.0527 -0.2803
0.1807 -0.2486 -38.5033
96.2800 -38.8600 -0.2333 0.1508 -
0.1106 0.1059 0.0877 -0.0571 -0.2794
0.1841 -0.2488 -38.5024
96.3000 -38.6200 0.1124 -0.0384 -
0.0407 0.1330 0.0944 -0.0614 -0.2784
0.1876 -0.2490 -38.5015
96.3200 -38.9100 -0.2420 -0.0992
0.0283 0.1565 0.0993 -0.0657 -0.2774
0.1910 -0.2492 -38.5007
96.3400 -38.4000 0.1910 -0.0533
0.0919 0.1738 0.1022 -0.0699 -0.2764
0.1944 -0.2494 -38.4998
96.3600 -38.4100 0.1172 -0.0099
0.1480 0.1827 0.1031 -0.0739 -0.2754
0.1979 -0.2496 -38.4989
96.3800 -38.6600 -0.2514 0.0317
0.1936 0.1812 0.1019 -0.0778 -0.2743
0.2013 -0.2498 -38.4980
96.4000 -38.4100 -0.0533 0.1000
0.2278 0.1683 0.0986 -0.0817 -0.2732
0.2047 -0.2500 -38.4972
96.4200 -38.1000 0.3419 0.1232
0.2467 0.1439 0.0934 -0.0853 -0.2720
0.2082 -0.2502 -38.4963
96.4400 -38.4700 0.0128 -0.0032
0.2475 0.1089 0.0863 -0.0888 -0.2709
0.2116 -0.2503 -38.4954
96.4600 -38.7800 -0.1210 -0.1681
0.2327 0.0650 0.0777 -0.0922 -0.2697
0.2150 -0.2505 -38.4945
96.4800 -39.0900 -0.3459 -0.1449
0.2038 0.0152 0.0677 -0.0953 -0.2684
0.2184 -0.2507 -38.4936
96.5000 -38.2900 0.3920 0.0314
0.1573 -0.0368 0.0565 -0.0983 -0.2671
0.2218 -0.2509 -38.4928
96.5200 -38.7000 -0.0264 0.1426
0.0854 -0.0870 0.0444 -0.1010 -0.2658
0.2253 -0.2511 -38.4919
96.5400 -38.9700 -0.1367 0.1350 -
0.0177 -0.1313 0.0317 -0.1035 -0.2645
0.2287 -0.2512 -38.4910
96.5600 -39.2100 -0.2315 0.1102 -
0.1473 -0.1665 0.0187 -0.1058 -0.2631
0.2321 -0.2514 -38.4901
96.5800 -38.8300 0.5299 0.0513 -
0.2827 -0.1899 0.0055 -0.1079 -0.2616
0.2355 -0.2516 -38.4893

96.6000 -39.8700 -0.2923 -0.1056 -
0.3949 -0.1995 -0.0075 -0.1096 -0.2602
0.2389 -0.2517 -38.4884
96.6200 -39.9300 -0.2117 -0.1980 -
0.4553 -0.1939 -0.0201 -0.1111 -0.2587
0.2423 -0.2519 -38.4875
96.6400 -39.7900 -0.1508 -0.0980 -
0.4454 -0.1728 -0.0322 -0.1123 -0.2571
0.2457 -0.2521 -38.4866
96.6600 -38.8900 0.4929 0.0068 -
0.3602 -0.1373 -0.0437 -0.1132 -0.2555
0.2491 -0.2522 -38.4857
96.6800 -39.3000 -0.0794 -0.0560 -
0.2062 -0.0906 -0.0545 -0.1138 -0.2539
0.2525 -0.2524 -38.4849
96.7000 -39.3800 -0.3197 -0.1500 -
0.0082 -0.0370 -0.0645 -0.1141 -0.2523
0.2559 -0.2525 -38.4840
96.7200 -38.9400 -0.1866 -0.0425
0.1939 0.0188 -0.0738 -0.1140 -0.2506
0.2593 -0.2527 -38.4831
96.7400 -38.3200 -0.2087 0.2407
0.3592 0.0722 -0.0823 -0.1136 -0.2488
0.2626 -0.2528 -38.4822
96.7600 -37.5900 0.4026 0.4350
0.4541 0.1187 -0.0898 -0.1128 -0.2470
0.2660 -0.2530 -38.4814
96.7800 -37.4300 0.6077 0.3046
0.4584 0.1545 -0.0964 -0.1117 -0.2452
0.2694 -0.2531 -38.4805
96.8000 -38.7000 -0.1728 -0.0973
0.3810 0.1772 -0.1019 -0.1102 -0.2434
0.2728 -0.2533 -38.4796
96.8200 -39.4200 -0.6285 -0.3660
0.2506 0.1866 -0.1062 -0.1084 -0.2415
0.2761 -0.2534 -38.4787
96.8400 -38.5500 0.3159 -0.2445
0.0996 0.1833 -0.1093 -0.1062 -0.2395
0.2795 -0.2536 -38.4779
96.8600 -38.5600 0.1668 0.0518 -
0.0437 0.1687 -0.1109 -0.1037 -0.2375
0.2828 -0.2537 -38.4770
96.8800 -38.7500 0.0703 0.1970 -
0.1568 0.1444 -0.1111 -0.1008 -0.2355
0.2862 -0.2539 -38.4761
96.9000 -39.1900 -0.3228 0.1308 -
0.2259 0.1126 -0.1097 -0.0977 -0.2334
0.2895 -0.2540 -38.4752
96.9200 -38.9800 0.1214 -0.0346 -
0.2509 0.0759 -0.1069 -0.0942 -0.2313
0.2929 -0.2541 -38.4744

96. 9400 -39. 0400 0. 1487 -0. 1685 -
0. 2396 0. 0370 -0. 1026 -0. 0904 -0. 2292
0. 2962 -0. 2543 -38. 4735
96. 9600 -39. 5300 -0. 3759 -0. 1408 -
0. 1994 -0. 0012 -0. 0969 -0. 0864 -0. 2270
0. 2995 -0. 2544 -38. 4726
96. 9800 -38. 7000 0. 2468 0. 0439 -
0. 1405 -0. 0365 -0. 0898 -0. 0821 -0. 2248
0. 3028 -0. 2545 -38. 4717
97. 0000 -38. 5600 0. 2690 0. 1347 -
0. 0753 -0. 0675 -0. 0815 -0. 0776 -0. 2226
0. 3062 -0. 2547 -38. 4709
97. 0200 -38. 9400 -0. 0886 0. 0166 -
0. 0157 -0. 0932 -0. 0718 -0. 0729 -0. 2203
0. 3095 -0. 2548 -38. 4700
97. 0400 -39. 0600 -0. 2023 -0. 1203
0. 0313 -0. 1127 -0. 0609 -0. 0679 -0. 2179
0. 3128 -0. 2549 -38. 4691
97. 0600 -38. 8100 -0. 0053 -0. 0929
0. 0614 -0. 1257 -0. 0488 -0. 0628 -0. 2156
0. 3161 -0. 2550 -38. 4682
97. 0800 -38. 7000 0. 0935 0. 0303
0. 0716 -0. 1320 -0. 0356 -0. 0576 -0. 2132
0. 3193 -0. 2552 -38. 4674
97. 1000 -38. 6000 0. 0359 0. 1060
0. 0625 -0. 1319 -0. 0216 -0. 0522 -0. 2108
0. 3226 -0. 2553 -38. 4665
97. 1200 -38. 7200 -0. 1059 0. 1212
0. 0414 -0. 1254 -0. 0068 -0. 0468 -0. 2083
0. 3259 -0. 2554 -38. 4656
97. 1400 -38. 7900 -0. 1471 0. 0952
0. 0184 -0. 1128 0. 0085 -0. 0413 -0. 2058
0. 3292 -0. 2555 -38. 4647
97. 1600 -38. 4100 0. 3629 -0. 0175
0. 0037 -0. 0948 0. 0240 -0. 0358 -0. 2033
0. 3324 -0. 2556 -38. 4639
97. 1800 -39. 1300 -0. 3142 -0. 1893
0. 0049 -0. 0725 0. 0396 -0. 0303 -0. 2008
0. 3357 -0. 2558 -38. 4630
97. 2000 -38. 8800 -0. 0715 -0. 2418
0. 0201 -0. 0473 0. 0551 -0. 0248 -0. 1982
0. 3389 -0. 2559 -38. 4621
97. 2200 -38. 4600 0. 1634 -0. 1097
0. 0397 -0. 0200 0. 0702 -0. 0194 -0. 1956
0. 3421 -0. 2560 -38. 4612
97. 2400 -38. 4100 -0. 1024 0. 0939
0. 0476 0. 0086 0. 0848 -0. 0142 -0. 1930
0. 3454 -0. 2561 -38. 4604
97. 2600 -38. 0400 0. 1083 0. 2734
0. 0296 0. 0375 0. 0987 -0. 0090 -0. 1904
0. 3486 -0. 2562 -38. 4595

97.2800 -38.1300 -0.0150 0.3273 -
0.0163 0.0654 0.1115 -0.0041 -0.1877
0.3518 -0.2563 -38.4586
97.3000 -38.2100 0.0464 0.1797 -
0.0744 0.0905 0.1231 0.0007 -0.1850
0.3550 -0.2565 -38.4578
97.3200 -38.5300 -0.0891 -0.1017 -
0.1203 0.1113 0.1333 0.0052 -0.1823
0.3582 -0.2566 -38.4569
97.3400 -38.7900 -0.0256 -0.3448 -
0.1289 0.1266 0.1419 0.0095 -0.1795
0.3614 -0.2567 -38.4560
97.3600 -38.6600 0.0654 -0.4185 -
0.0797 0.1357 0.1488 0.0135 -0.1768
0.3645 -0.2568 -38.4551
97.3800 -38.4600 0.0250 -0.2782
0.0235 0.1380 0.1539 0.0172 -0.1740
0.3677 -0.2569 -38.4543
97.4000 -38.2100 -0.1995 0.0704
0.1450 0.1330 0.1571 0.0205 -0.1712
0.3708 -0.2570 -38.4534
97.4200 -37.7300 -0.1383 0.4562
0.2351 0.1202 0.1585 0.0235 -0.1684
0.3740 -0.2571 -38.4525
97.4400 -36.8300 0.6448 0.4862
0.2504 0.0997 0.1580 0.0262 -0.1656
0.3771 -0.2572 -38.4516
97.4600 -38.5600 -0.6147 0.0574
0.1774 0.0723 0.1556 0.0285 -0.1628
0.3802 -0.2574 -38.4508
97.4800 -38.4200 0.1136 -0.2856
0.0458 0.0405 0.1516 0.0305 -0.1599
0.3833 -0.2575 -38.4499
97.5000 -38.3700 0.2613 -0.2851 -
0.0990 0.0074 0.1461 0.0320 -0.1571
0.3864 -0.2576 -38.4490
97.5200 -38.6000 -0.0342 -0.1224 -
0.2089 -0.0240 0.1392 0.0332 -0.1542
0.3895 -0.2577 -38.4482
97.5400 -38.9600 -0.3588 0.0038 -
0.2537 -0.0508 0.1311 0.0341 -0.1513
0.3926 -0.2578 -38.4473
97.5600 -38.2000 0.5337 -0.0126 -
0.2268 -0.0705 0.1220 0.0345 -0.1485
0.3956 -0.2579 -38.4464
97.5800 -39.2300 -0.4972 -0.1175 -
0.1447 -0.0820 0.1120 0.0347 -0.1456
0.3987 -0.2580 -38.4455
97.6000 -38.6400 -0.1439 -0.0508 -
0.0374 -0.0853 0.1013 0.0344 -0.1427
0.4017 -0.2581 -38.4447

97.6200 -37.9800 0.2148 0.1438
0.0623 -0.0813 0.0900 0.0339 -0.1398
0.4048 -0.2582 -38.4438
97.6400 -37.9400 0.1889 0.1919
0.1291 -0.0713 0.0783 0.0330 -0.1369
0.4078 -0.2583 -38.4429
97.6600 -38.2300 -0.0562 0.0558
0.1511 -0.0571 0.0665 0.0318 -0.1340
0.4108 -0.2584 -38.4421
97.6800 -38.5100 -0.2918 -0.0373
0.1357 -0.0408 0.0546 0.0304 -0.1311
0.4138 -0.2586 -38.4412
97.7000 -38.1800 0.2295 -0.0051
0.0965 -0.0246 0.0428 0.0286 -0.1282
0.4167 -0.2587 -38.4403
97.7200 -38.3100 0.0276 0.0194
0.0487 -0.0104 0.0313 0.0266 -0.1253
0.4197 -0.2588 -38.4394
97.7400 -38.2900 -0.0317 -0.0083
0.0101 0.0007 0.0202 0.0244 -0.1224
0.4227 -0.2589 -38.4386
97.7600 -38.4400 -0.0999 -0.0245 -
0.0099 0.0079 0.0095 0.0221 -0.1195
0.4256 -0.2590 -38.4377
97.7800 -38.1800 0.3215 -0.0375 -
0.0121 0.0113 -0.0007 0.0195 -0.1166
0.4285 -0.2591 -38.4368
97.8000 -38.7500 -0.3646 -0.0563 -
0.0017 0.0112 -0.0105 0.0168 -0.1137
0.4314 -0.2592 -38.4360
97.8200 -38.2200 0.2046 -0.0269
0.0132 0.0084 -0.0197 0.0140 -0.1108
0.4343 -0.2593 -38.4351
97.8400 -38.1400 0.1936 0.0135
0.0220 0.0044 -0.0285 0.0112 -0.1080
0.4372 -0.2594 -38.4342
97.8600 -38.3600 -0.0593 0.0211
0.0162 0.0006 -0.0368 0.0083 -0.1051
0.4401 -0.2596 -38.4334
97.8800 -38.6200 -0.2265 0.0473 -
0.0043 -0.0019 -0.0447 0.0054 -0.1023
0.4429 -0.2597 -38.4325
97.9000 -37.8500 0.3409 0.0765 -
0.0323 -0.0022 -0.0521 0.0026 -0.0995
0.4457 -0.2598 -38.4316
97.9200 -38.3300 0.2126 -0.0053 -
0.0576 0.0002 -0.0590 -0.0001 -0.0967
0.4486 -0.2599 -38.4307
97.9400 -38.7500 -0.1160 -0.1467 -
0.0699 0.0051 -0.0656 -0.0028 -0.0939
0.4514 -0.2600 -38.4299

97.9600	-38.9900	-0.3513	-0.1387	-
0.0630	0.0119	-0.0719	-0.0053	-0.0911
	0.4542	-0.2601	-38.4290	
97.9800	-38.4000	0.0574	0.0348	-
0.0371	0.0199	-0.0778	-0.0076	-0.0883
	0.4569	-0.2603	-38.4281	
98.0000	-37.8500	0.4031	0.1401	-
0.0003	0.0284	-0.0834	-0.0097	-0.0856
	0.4597	-0.2604	-38.4273	
98.0200	-38.4900	-0.2588	0.0537	
0.0363	0.0368	-0.0887	-0.0116	-0.0829
	0.4624	-0.2605	-38.4264	
98.0400	-38.6500	-0.2325	-0.0550	
0.0635	0.0444	-0.0937	-0.0132	-0.0802
	0.4652	-0.2606	-38.4255	
98.0600	-38.2000	0.2587	-0.0534	
0.0746	0.0506	-0.0985	-0.0146	-0.0775
	0.4679	-0.2607	-38.4247	
98.0800	-38.2600	0.0076	0.0086	
0.0673	0.0547	-0.1030	-0.0157	-0.0749
	0.4706	-0.2609	-38.4238	
98.1000	-38.3200	-0.0315	0.0157	
0.0457	0.0565	-0.1072	-0.0164	-0.0723
	0.4732	-0.2610	-38.4229	
98.1200	-38.3800	-0.0090	-0.0262	
0.0187	0.0557	-0.1109	-0.0168	-0.0697
	0.4759	-0.2611	-38.4220	
98.1400	-38.3200	0.0195	-0.0353	-
0.0048	0.0518	-0.1142	-0.0169	-0.0671
	0.4785	-0.2612	-38.4212	
98.1600	-38.2900	-0.0171	0.0078	-
0.0202	0.0443	-0.1169	-0.0167	-0.0646
	0.4812	-0.2614	-38.4203	
98.1800	-38.3200	-0.0749	0.0903	-
0.0277	0.0331	-0.1189	-0.0161	-0.0621
	0.4838	-0.2615	-38.4194	
98.2000	-38.2000	0.0271	0.1225	-
0.0303	0.0185	-0.1202	-0.0151	-0.0597
	0.4864	-0.2616	-38.4186	
98.2200	-38.0300	0.3674	0.0167	-
0.0283	0.0009	-0.1205	-0.0139	-0.0572
	0.4889	-0.2618	-38.4177	
98.2400	-38.9300	-0.4447	-0.1386	-
0.0165	-0.0187	-0.1199	-0.0122	-0.0548
	0.4915	-0.2619	-38.4168	
98.2600	-38.3300	0.1046	-0.1234	
0.0127	-0.0392	-0.1183	-0.0103	-0.0525
	0.4940	-0.2620	-38.4160	
98.2800	-38.3100	0.0711	0.0010	
0.0549	-0.0588	-0.1156	-0.0080	-0.0502
	0.4965	-0.2622	-38.4151	

98.3000 -38.2000 0.1035 0.0577
0.0940 -0.0757 -0.1116 -0.0054 -0.0479
0.4990 -0.2623 -38.4142
98.3200 -38.2800 0.0000 0.0279
0.1079 -0.0879 -0.1064 -0.0025 -0.0456
0.5015 -0.2624 -38.4134
98.3400 -38.6100 -0.3601 0.0624
0.0762 -0.0936 -0.1000 0.0007 -0.0434
0.5040 -0.2626 -38.4125
98.3600 -38.2100 0.0976 0.1453
0.0002 -0.0915 -0.0923 0.0041 -0.0412
0.5064 -0.2627 -38.4116
98.3800 -37.8900 0.4320 0.0923 -
0.0982 -0.0816 -0.0835 0.0078 -0.0391
0.5088 -0.2629 -38.4108
98.4000 -38.6600 -0.0712 -0.1212 -
0.1907 -0.0646 -0.0736 0.0118 -0.0370
0.5112 -0.2630 -38.4099
98.4200 -39.1800 -0.4061 -0.2729 -
0.2458 -0.0416 -0.0627 0.0160 -0.0349
0.5136 -0.2632 -38.4090
98.4400 -38.7200 -0.0654 -0.1957 -
0.2414 -0.0143 -0.0511 0.0204 -0.0329
0.5160 -0.2633 -38.4081
98.4600 -38.1500 0.2931 -0.0098 -
0.1759 0.0148 -0.0387 0.0250 -0.0310
0.5183 -0.2635 -38.4073
98.4800 -38.0800 -0.0821 0.0972 -
0.0612 0.0432 -0.0259 0.0297 -0.0290
0.5207 -0.2636 -38.4064
98.5000 -37.9800 -0.0108 0.0784
0.0798 0.0684 -0.0127 0.0345 -0.0271
0.5230 -0.2638 -38.4055
98.5200 -37.4800 0.3172 0.0004
0.2135 0.0879 0.0008 0.0394 -0.0253
0.5253 -0.2639 -38.4047
98.5400 -38.1700 -0.4388 0.0223
0.3049 0.0998 0.0143 0.0444 -0.0235
0.5275 -0.2641 -38.4038
98.5600 -37.1200 0.2823 0.1611
0.3297 0.1025 0.0279 0.0494 -0.0217
0.5298 -0.2643 -38.4029
98.5800 -37.4800 -0.0222 0.2340
0.2794 0.0958 0.0413 0.0543 -0.0200
0.5320 -0.2644 -38.4021
98.6000 -37.7000 -0.0122 0.1833
0.1611 0.0810 0.0545 0.0593 -0.0183
0.5342 -0.2646 -38.4012
98.6200 -38.0900 -0.0949 0.0541
0.0013 0.0605 0.0672 0.0641 -0.0167
0.5364 -0.2647 -38.4003

98.6400 -38.0200 0.2060 -0.1183 -
0.1582 0.0366 0.0795 0.0689 -0.0151
0.5386 -0.2649 -38.3995
98.6600 -38.4900 0.0397 -0.2631 -
0.2755 0.0118 0.0911 0.0736 -0.0136
0.5407 -0.2651 -38.3986
98.6800 -38.9600 -0.4164 -0.2536 -
0.3189 -0.0115 0.1019 0.0781 -0.0121
0.5429 -0.2653 -38.3977
98.7000 -37.8900 0.4101 -0.0841 -
0.2769 -0.0313 0.1118 0.0824 -0.0107
0.5450 -0.2654 -38.3969
98.7200 -38.2300 -0.0679 0.0012 -
0.1638 -0.0464 0.1208 0.0865 -0.0093
0.5471 -0.2656 -38.3960
98.7400 -37.8700 0.0557 -0.0538 -
0.0157 -0.0563 0.1288 0.0904 -0.0079
0.5492 -0.2658 -38.3951
98.7600 -38.2300 -0.4178 0.0466
0.1199 -0.0606 0.1357 0.0941 -0.0066
0.5513 -0.2660 -38.3943
98.7800 -37.1600 0.2816 0.2992
0.2021 -0.0592 0.1414 0.0975 -0.0054
0.5533 -0.2662 -38.3934
98.8000 -37.0900 0.2319 0.3534
0.2124 -0.0520 0.1461 0.1006 -0.0042
0.5554 -0.2663 -38.3925
98.8200 -37.6700 -0.0987 0.1004
0.1578 -0.0401 0.1496 0.1035 -0.0030
0.5574 -0.2665 -38.3917
98.8400 -38.0600 -0.1431 -0.2160
0.0706 -0.0247 0.1520 0.1061 -0.0019
0.5594 -0.2667 -38.3908
98.8600 -38.2200 -0.1020 -0.3282 -
0.0134 -0.0076 0.1535 0.1084 -0.0009
0.5614 -0.2669 -38.3899
98.8800 -38.0900 -0.0278 -0.2243 -
0.0681 0.0095 0.1541 0.1104 0.0002
0.5634 -0.2671 -38.3890
98.9000 -37.7300 0.2005 -0.0762 -
0.0797 0.0252 0.1539 0.1121 0.0011
0.5654 -0.2673 -38.3882
98.9200 -37.8700 -0.0565 -0.0072 -
0.0485 0.0389 0.1532 0.1136 0.0021
0.5674 -0.2675 -38.3873
98.9400 -37.7500 -0.0899 0.0532
0.0056 0.0505 0.1520 0.1149 0.0029
0.5693 -0.2677 -38.3864
98.9600 -37.6000 -0.1210 0.2010
0.0552 0.0602 0.1505 0.1160 0.0038
0.5713 -0.2679 -38.3856

98.9800 -37.1900 0.1854 0.3059
0.0797 0.0682 0.1491 0.1168 0.0046
0.5732 -0.2680 -38.3847
99.0000 -37.2900 0.2022 0.2290
0.0738 0.0749 0.1477 0.1176 0.0055
0.5752 -0.2682 -38.3838

DATA:
Time-varying Shannon entropy (SE) and 95% significance level of DO modes extracted by EEMD
(shown in the 5-8 columns in the above).

Column 1: Time (kyr. BP)
Column 2: Shannon entropy of IMF3 (SE3)
Column 3: 95% significance level of SE of IMF3 (95% SL3)
Column 4: Shannon entropy of IMF4 (SE4)
Column 5: 95% significance level of SE of IMF4 (95% SL4)
Column 6: Shannon entropy of IMF5 (SE5)
Column 7: 95% significance level of SE of IMF5 (95% SL5)
Column 8: Shannon entropy of IMF6 (SE6)
Column 9: 95% significance level of SE of IMF6 (95% SL6)

Time SE3 95% SL3 SE4
95% SL4 SE5 95% SL5
SE6 95% SL6
4.9900 1.2629 1.2913 1.0796
1.4725 1.2717 1.7077 1.0979
1.8576
5.5900 0.8023 1.2891 0.9552
1.4648 0.9990 1.7057 1.2323
1.8519
6.1900 1.1527 1.2787 1.0085
1.4786 1.0531 1.6994 1.2401
1.8611
6.7900 1.1652 1.2966 1.0089
1.4822 1.0684 1.7029 1.3775
1.8583
7.3900 1.1782 1.2962 1.0154
1.4894 1.1407 1.7027 1.4287
1.8620
7.9900 1.1858 1.3159 1.0107
1.4964 1.5099 1.7030 1.5960
1.8677
8.5900 0.4997 1.3331 0.8943
1.5061 1.0231 1.7140 1.7165
1.8523
9.1900 0.7403 1.3327 1.0171
1.4953 1.1331 1.6977 1.3224
1.8448

9.7900	0.7860	1.3139	1.0960
1.4828	1.2343	1.7101	1.4055
1.8541			
10.3900	1.1822	1.3024	1.4437
1.4893	1.3615	1.6877	1.5784
1.8722			
10.9900	1.2276	1.3156	1.3116
1.4786	1.4527	1.7010	1.6868
1.8661			
11.5900	1.2810	1.3235	1.2281
1.4765	1.4294	1.6972	1.7041
1.8612			
12.1900	1.3140	1.3290	1.2151
1.4758	1.4489	1.6871	1.6750
1.8618			
12.7900	1.3297	1.3367	1.2508
1.4817	1.4821	1.7031	1.7035
1.8600			
13.3900	1.3617	1.3256	1.2765
1.4767	1.4932	1.6842	1.7611
1.8725			
13.9900	1.3845	1.3349	1.2778
1.4655	1.5010	1.6758	1.8376
1.8624			
14.5900	1.2557	1.3321	1.2621
1.4877	1.4503	1.6811	1.7367
1.8576			
15.1900	1.2821	1.3332	1.2388
1.4716	1.4336	1.6838	1.7752
1.8597			
15.7900	1.2801	1.3269	1.2196
1.4750	1.4275	1.6736	1.6218
1.8549			
16.3900	1.4909	1.3204	1.1771
1.4728	1.4269	1.6820	1.4816
1.8588			
16.9900	1.5296	1.3233	1.1509
1.4806	1.4472	1.7006	1.3839
1.8643			
17.5900	1.5109	1.3044	1.0360
1.4632	1.2652	1.6938	1.2561
1.8576			
18.1900	1.4346	1.3296	1.4843
1.4665	1.6227	1.6984	1.5405
1.8579			
18.7900	1.5471	1.3226	1.4502
1.4647	1.6162	1.6952	1.4941
1.8575			
19.3900	1.5433	1.3157	1.4343
1.4657	1.6549	1.6954	1.7111
1.8578			

19.9900	0.9794	1.3372	1.4356
1.4603	1.4113	1.6810	1.5849
1.8652			
20.5900	0.9986	1.3077	1.4263
1.4844	1.3340	1.6791	1.6065
1.8659			
21.1900	0.9958	1.3223	1.4331
1.4933	1.4152	1.6829	1.6243
1.8752			
21.7900	0.9820	1.3196	1.4502
1.5047	1.4522	1.7123	1.7552
1.8672			
22.3900	0.9933	1.3160	1.5696
1.4973	1.4571	1.6988	1.7245
1.8754			
22.9900	0.9835	1.3222	1.3585
1.4815	1.4892	1.6907	1.7402
1.8821			
23.5900	0.9701	1.3264	1.3488
1.4910	1.4878	1.7019	1.7332
1.8745			
24.1900	0.9801	1.3184	1.3145
1.4977	1.4933	1.6929	1.7220
1.8793			
24.7900	1.0341	1.3114	1.1356
1.4918	1.5248	1.6946	1.7077
1.8637			
25.3900	1.0374	1.2961	1.1668
1.4807	1.5848	1.7053	1.6308
1.8692			
25.9900	1.0606	1.3076	1.2813
1.4698	1.5865	1.6949	1.6180
1.8675			
26.5900	1.4475	1.3052	1.2721
1.4811	1.5582	1.6945	1.6344
1.8677			
27.1900	1.4539	1.3026	1.2860
1.4840	1.4291	1.7055	1.6539
1.8729			
27.7900	1.4836	1.3208	1.3099
1.4854	1.4280	1.7020	1.6214
1.8668			
28.3900	1.4588	1.3130	1.3081
1.4859	1.4805	1.6930	1.3784
1.8565			
28.9900	1.4434	1.3077	1.3083
1.4820	1.5275	1.6971	1.5525
1.8707			
29.5900	1.4580	1.3083	1.3367
1.4786	1.6499	1.6917	1.6313
1.8653			

30.1900 1.4548 1.3096 1.3418
1.4862 1.7735 1.6962 1.7315
1.8662
30.7900 1.3265 1.3281 1.4801
1.4813 1.7070 1.6876 1.7998
1.8722
31.3900 1.0356 1.3185 1.4854
1.4762 1.5754 1.6935 1.8568
1.8610
31.9900 1.6099 1.3177 1.4837
1.4667 1.5917 1.6827 1.5983
1.8597
32.5900 1.2524 1.3172 1.4190
1.4881 1.7239 1.6680 1.6253
1.8681
33.1900 1.2591 1.3117 1.4216
1.4842 1.7521 1.6752 1.4138
1.8651
33.7900 1.2322 1.3305 1.4135
1.4878 1.7551 1.6730 1.5904
1.8539
34.3900 1.2386 1.3128 1.3970
1.4795 1.7287 1.6839 1.5902
1.8475
34.9900 1.2419 1.3129 1.4017
1.4828 1.7439 1.6975 1.7061
1.8407
35.5900 1.3842 1.3218 1.2573
1.4873 1.7197 1.6974 1.7275
1.8404
36.1900 1.3799 1.2917 1.2457
1.4723 1.7357 1.6947 1.7589
1.8438
36.7900 1.3474 1.2914 1.3075
1.4819 1.6203 1.6854 1.7952
1.8551
37.3900 1.3717 1.3162 1.4509
1.5026 1.5636 1.6815 1.7895
1.8510
37.9900 1.1098 1.3188 1.6743
1.4955 1.4704 1.6741 1.8554
1.8548
38.5900 1.5079 1.2870 1.4461
1.4974 1.6856 1.6740 1.8347
1.8622
39.1900 1.4894 1.3087 1.4413
1.4944 1.5069 1.6792 1.7870
1.8593
39.7900 1.4903 1.3109 1.4113
1.4841 1.5208 1.6879 1.7098
1.8492

40.3900	1.4853	1.3023	1.6330
1.4823	1.5973	1.6845	1.5416
1.8630			
40.9900	1.4838	1.3212	1.4687
1.4850	1.6326	1.6878	1.5096
1.8577			
41.5900	1.4473	1.3173	1.4686
1.4796	1.6266	1.7035	1.6171
1.8522			
42.1900	1.4354	1.3277	1.4700
1.4720	1.6195	1.6969	1.6056
1.8579			
42.7900	1.3369	1.3134	1.4753
1.4748	1.6200	1.6822	1.7202
1.8580			
43.3900	1.1515	1.3160	1.4598
1.4651	1.6160	1.6835	1.6506
1.8686			
43.9900	0.8463	1.2907	1.3975
1.4606	1.6126	1.6850	1.7178
1.8679			
44.5900	0.7883	1.3082	1.4225
1.4720	1.5925	1.6862	1.6755
1.8602			
45.1900	0.7690	1.3214	1.4168
1.4742	1.5188	1.6969	1.7939
1.8585			
45.7900	0.7798	1.3196	1.3989
1.4844	1.2581	1.6987	1.8305
1.8626			
46.3900	1.0647	1.3198	1.3687
1.4820	1.6039	1.6928	1.8189
1.8685			
46.9900	1.0739	1.3096	1.1656
1.4755	1.4564	1.6934	1.8416
1.8522			
47.5900	1.0707	1.3203	1.1275
1.4987	1.4053	1.6973	1.7952
1.8519			
48.1900	1.0490	1.3299	1.1053
1.4761	1.3722	1.6954	1.7207
1.8430			
48.7900	1.0653	1.3137	1.1122
1.4843	1.3634	1.7062	1.6364
1.8462			
49.3900	1.0707	1.3182	1.0916
1.4796	1.3093	1.6900	1.4555
1.8611			
49.9900	0.7253	1.3096	0.9516
1.4892	1.1954	1.6933	1.4749
1.8560			

50.5900 0.7043 1.2976 1.1403
1.4901 1.3975 1.7029 1.4349
1.8502
51.1900 0.9589 1.3001 1.1491
1.4874 1.4550 1.6919 1.3519
1.8596
51.7900 0.9791 1.2893 1.0154
1.4853 1.2281 1.7111 1.5184
1.8671
52.3900 0.9966 1.2885 0.9049
1.4766 1.3510 1.7017 1.6623
1.8498
52.9900 1.0547 1.2927 0.8960
1.4881 1.3521 1.7048 1.6345
1.8454
53.5900 1.0971 1.2933 0.9728
1.4728 1.5013 1.7157 1.6664
1.8529
54.1900 1.1068 1.2814 1.0201
1.4754 1.5388 1.7207 1.6938
1.8623
54.7900 1.0787 1.2854 1.0324
1.4698 1.5651 1.7199 1.7155
1.8571
55.3900 1.1213 1.2930 1.0509
1.4860 1.6169 1.7102 1.6968
1.8788
55.9900 1.2287 1.3132 1.0942
1.4815 1.6711 1.6997 1.6583
1.8652
56.5900 1.2603 1.3004 1.1260
1.4748 1.6868 1.6912 1.6465
1.8762
57.1900 1.2475 1.2976 1.1159
1.4729 1.7574 1.6966 1.6471
1.8672
57.7900 1.2235 1.3125 1.0927
1.4771 1.6675 1.6932 1.6104
1.8647
58.3900 1.1791 1.3314 1.0489
1.4707 1.6047 1.6859 1.6574
1.8726
58.9900 1.1388 1.3228 1.4514
1.4809 1.5749 1.6813 1.6800
1.8658
59.5900 1.1082 1.3273 1.4122
1.4746 1.5255 1.6928 1.7717
1.8672
60.1900 1.1219 1.3196 1.4070
1.4823 1.5752 1.6889 1.7559
1.8544

60.7900 1.1302 1.3266 1.4857
1.4915 1.6546 1.6866 1.5831
1.8609
61.3900 1.1522 1.3100 1.2353
1.4901 1.7300 1.6969 1.7093
1.8563
61.9900 0.8806 1.3005 1.1014
1.4854 1.5564 1.6900 1.6630
1.8501
62.5900 0.9436 1.3143 1.0689
1.4890 1.5531 1.6908 1.6385
1.8567
63.1900 0.9346 1.2867 1.0791
1.4815 1.4214 1.7067 1.6423
1.8694
63.7900 0.9438 1.3201 1.0625
1.4815 1.4213 1.7105 1.6475
1.8679
64.3900 0.9317 1.3060 1.0556
1.4899 1.3957 1.6907 1.6489
1.8668
64.9900 0.9374 1.3008 1.0493
1.4845 1.3736 1.6859 1.6440
1.8596
65.5900 0.9368 1.3002 1.0693
1.4829 1.3498 1.7037 1.6267
1.8689
66.1900 0.9354 1.2832 1.0857
1.4830 1.3229 1.6995 1.7098
1.8773
66.7900 0.9327 1.2919 1.2060
1.4897 1.3263 1.6987 1.4369
1.8693
67.3900 0.8756 1.2916 0.9460
1.4823 1.4208 1.6977 1.3865
1.8645
67.9900 0.8800 1.3149 0.9610
1.4828 1.5722 1.7010 1.3876
1.8647
68.5900 0.8667 1.3072 0.9409
1.4684 1.6286 1.7072 1.4027
1.8684
69.1900 0.8617 1.3173 1.2372
1.4641 1.1899 1.7056 1.4425
1.8654
69.7900 1.1310 1.3147 1.2682
1.4809 1.1837 1.7095 1.3955
1.8679
70.3900 1.1357 1.3190 1.2939
1.4619 1.3421 1.6966 1.5387
1.8559

70.9900 1.1569 1.3230 1.2969
1.4597 1.3658 1.6856 1.7334
1.8513
71.5900 1.1584 1.3226 1.4945
1.4680 1.3782 1.7031 1.7682
1.8541
72.1900 1.1447 1.3353 1.4757
1.4693 1.3801 1.6985 1.8070
1.8579
72.7900 0.8902 1.3350 1.2787
1.4772 1.3600 1.6971 1.8008
1.8532
73.3900 0.7685 1.3331 1.3086
1.4678 1.3976 1.6933 1.7820
1.8652
73.9900 1.0199 1.3167 1.3829
1.4755 1.7103 1.7051 1.7834
1.8619
74.5900 1.0334 1.3105 1.3946
1.4817 1.7583 1.7040 1.7871
1.8759
75.1900 1.0418 1.2981 1.3566
1.4766 1.7693 1.7073 1.9053
1.8764
75.7900 0.8239 1.3135 1.3772
1.4678 1.6786 1.7166 1.8753
1.8643
76.3900 0.8351 1.3273 1.3647
1.4672 1.6263 1.6948 1.8262
1.8664
76.9900 0.8041 1.3342 1.3512
1.4701 1.6125 1.7029 1.8150
1.8786
77.5900 0.7899 1.3317 1.3103
1.4668 1.5841 1.6882 1.5911
1.8680
78.1900 0.7875 1.3229 1.2923
1.4717 1.5586 1.6836 1.5240
1.8569
78.7900 0.7767 1.3138 1.2860
1.4572 1.5305 1.6874 1.4575
1.8607
79.3900 0.7719 1.3119 1.2520
1.4624 1.4088 1.6957 1.4785
1.8564
79.9900 0.9979 1.3326 1.0075
1.4626 1.2942 1.6919 1.3234
1.8557
80.5900 0.9868 1.3334 1.0712
1.4692 1.2653 1.7006 1.2441
1.8648

81.1900 0.9053 1.3114 0.9669
1.4534 1.3934 1.6893 1.5227
1.8591
81.7900 0.8801 1.3086 1.1973
1.4649 1.3508 1.6884 1.4980
1.8639
82.3900 0.5100 1.3114 1.0684
1.4737 1.3917 1.6867 1.6079
1.8593
82.9900 0.5061 1.3088 1.0423
1.4780 1.3162 1.6846 1.6078
1.8558
83.5900 0.5184 1.3041 1.0250
1.4831 1.3363 1.6920 1.6199
1.8419
84.1900 0.5682 1.3102 1.0676
1.4709 1.4336 1.6916 1.7095
1.8531
84.7900 0.5999 1.3064 1.1317
1.4689 1.4710 1.6719 1.7189
1.8556
85.3900 0.7863 1.3089 1.1487
1.4762 1.6706 1.6831 1.8096
1.8471
85.9900 0.7852 1.2949 1.1539
1.4653 1.7342 1.6766 1.8231
1.8487
86.5900 0.7732 1.3003 1.1332
1.4880 1.7970 1.6888 1.5927
1.8442
87.1900 0.7833 1.3035 1.1323
1.4854 1.7591 1.6925 1.6441
1.8514
87.7900 0.7054 1.2914 1.0890
1.4786 1.8107 1.6840 1.7111
1.8648
88.3900 0.8143 1.2936 1.2057
1.4741 1.7690 1.6835 1.6564
1.8677
88.9900 0.8095 1.2996 1.2173
1.4833 1.7814 1.6858 1.6715
1.8575
89.5900 0.7951 1.2949 1.2230
1.4807 1.7709 1.6988 1.6829
1.8606
90.1900 0.6878 1.2991 1.0327
1.4863 1.7079 1.7026 1.5602
1.8616
90.7900 0.9679 1.2890 1.0019
1.4919 1.4915 1.6991 1.5941
1.8722

91.3900 1.0766 1.3051 1.4696
1.4900 1.4224 1.7259 1.4401
1.8580
91.9900 1.1888 1.3412 1.4805
1.4921 1.2626 1.7093 1.4002
1.8505
92.5900 1.2035 1.3331 1.4673
1.4880 1.2406 1.7178 1.3224
1.8622
93.1900 1.0549 1.3236 1.5894
1.4868 1.3974 1.7234 1.6243
1.8686
93.7900 1.1868 1.3062 1.3719
1.4953 1.4854 1.7047 1.5819
1.8621
94.3900 1.1979 1.3127 1.3829
1.4763 1.3972 1.7106 1.4831
1.8591
94.9900 1.2047 1.3078 1.3842
1.4842 1.4755 1.7030 1.7941
1.8720
95.5900 1.2129 1.3033 1.1741
1.4823 1.4651 1.7049 1.6499
1.8711
