# Supplementary material for: Data concerning statistical relation between obliquity and Dansgaard–Oeschger events
Source: Data Brief. 2019 Mar 7;23:103727. doi: 10.1016/j.dib.2019.103727 (PMC6660458; doi:10.1016/j.dib.2019.103727)
Supplement: Multimedia component 4 [file mmc4.pdf]

Processed oxygen isotope (d18O) data from NGRIP ice core on GICC05 modelext timescale
----

NAME OF DATA SET:
Processed oxygen isotope (d18O) data from NGRIP ice core on GICC05 modelext timescale

LAST UPDATE: 21/12/2018

ORIGINAL REFERENCE:
Jia Deng, Zhaohua Wu, Min Zhanga, Norden. E Huang, Shizhu Wang,Fangli Qiao. 2018.
Data concerning statistical relation between obliquity and Dansgaard-Oeschger events.
Data in Brief, in press.

MAIN REFERENCES:
Datafile of the original data accompanies the following two papers:

Rasmussen, S. O et al., 2014. A stratigraphic framework for abrupt climatic changes during the Last
Glacial period based on three synchronized Greenland ice-core records: refining and extending the
INTIMATE event stratigraphy. Quaternary. Sci. Rev. 106: 14-28.

Seierstad, I. K et al., 2014. Consistently dated records from the Greenland GRIP, GISP2 and NGRIP
ice cores for the past 104 ka reveal regional millennial-scale ﾇﾄ18O gradients with possible Heinrich
event imprint. Quaternary. Sci. Rev. 106: 29-46.

ABSTRACT:
Data presented are related to the research article entitled 。-Using Holo-Hilbert spectral analysis to
quantify the modulation of Dansgaard-Oeschger events by obliquity。ｱ [1]. The datasets in Deng et al (2018)
are analyzed on the foundation of ensemble empirical mode decomposition (EEMD) [2], and reveal more occurrences
of Dansgaard-Oeschger (DO) events in the decreasing phase of obliquity. Here, we report the number of significant
high Shannon entropy (SE) [3] of 95% significance level of DO events in the increasing and decreasing phases of
obliquity, respectively. First, the proxy time series are filtered by EEMD to obtain DO events. Then, the
time-varying SE of DO modes are calculated on the basis of principle of histogram. The 95% significance level
is evaluated through surrogate data [4]. Finally, a comparison between the numbers of SE values that are larger
than 95% significance level in the increasing and decreasing phases of obliquity, respectively, is reported.

GEOGRAPHIC REGION: Greenland

PERIOD OF RECORD: 99 kyr. BP - 2 kyr.BP

FUNDING SOURCES:
This work was jointly supported by the National Basic Research Program of China (Grant 2012CB957802);
the National Natural Science Foundation of China (NSFC) (Grant 41506067); the Basic Scientific Fund
for National Public Research Institutes of China (Grant 2015G04); the US National Science Foundation
(Grant AGS-1723300); the NSFC-Shandong Joint Fund for Marine Science Research Centers (Grant U1406404);
and the National Programme on Global Change and Air-Sea Interaction (Grant GASI-IPOVAI-05).

DATA:
Calibrated NGRIP oxygen isotope (d18O) data on GICC05modeext timescale (Rasmussen et al, 2014; Seierstad et al, 2014).
Processed data by ensemble empirical mode decomposition (EEMD).

Column 1: Time (kyr. BP)
Column 2: (Calibrated secondary) d18O data (years before b2k)
Column 3: 1st EEMD component of d18O records
Column 4: 2nd EEMD component of d18O records
Column 5: 3rd EEMD component of d18O records
Column 6: 4th EEMD component of d18O records
Column 7: 5th EEMD component of d18O records
Column 8: 6th EEMD component of d18O records
Column 9: 8th EEMD component of d18O records
Column 10: 9th EEMD component of d18O records
Column 11: 10th EEMD component of d18O records
Column 12: EEMD trend of d18O records

Time	d18O data	IMF1	IMF2	IMF3	IMF4	IMF5	IMF6	IMF7	IMF8	IMF9	trend
2.0000	-35.1100	-0.0289	-0.0747	0.1042	-0.0274	0.0401	-0.0410	-0.0730	-0.4262	2.6108	-37.2690
2.0200	-34.7900	0.2550	-0.1010	0.1627	-0.0310	0.0374	-0.0418	-0.0741	-0.4258	2.6148	-37.2718
2.0400	-35.3500	-0.2969	-0.0634	0.1873	-0.0375	0.0343	-0.0425	-0.0753	-0.4254	2.6188	-37.2747
2.0600	-34.9500	0.0162	0.0623	0.1600	-0.0458	0.0309	-0.0433	-0.0765	-0.4250	2.6228	-37.2775

2.0800 -34.8900 0.0755 0.1859
0.0814 -0.0528 0.0270 -0.0440 -0.0776
-0.4246 2.6267 -37.2804
2.1000 -35.0700 0.0213 0.1994 -
0.0307 -0.0557 0.0228 -0.0446 -0.0787
-0.4242 2.6307 -37.2832
2.1200 -35.4600 -0.2073 0.0856 -
0.1490 -0.0530 0.0184 -0.0451 -0.0798
-0.4238 2.6347 -37.2861
2.1400 -35.1800 0.3449 -0.1184 -
0.2397 -0.0436 0.0138 -0.0455 -0.0809
-0.4234 2.6386 -37.2889
2.1600 -36.0700 -0.2825 -0.2983 -
0.2711 -0.0272 0.0090 -0.0458 -0.0819
-0.4229 2.6426 -37.2918
2.1800 -35.7600 -0.0766 -0.2591 -
0.2311 -0.0041 0.0043 -0.0460 -0.0829
-0.4225 2.6466 -37.2946
2.2000 -35.2000 0.2428 -0.0469 -
0.1336 0.0242 -0.0005 -0.0461 -0.0838
-0.4221 2.6505 -37.2974
2.2200 -35.2600 -0.2434 0.1238 -
0.0095 0.0553 -0.0051 -0.0461 -0.0847
-0.4216 2.6545 -37.3003
2.2400 -34.8400 -0.0523 0.1975
0.1059 0.0861 -0.0097 -0.0459 -0.0856
-0.4211 2.6584 -37.3031
2.2600 -34.5700 0.1389 0.2059
0.1835 0.1135 -0.0143 -0.0455 -0.0864
-0.4206 2.6624 -37.3060
2.2800 -34.8100 0.0603 0.0960
0.2146 0.1345 -0.0187 -0.0450 -0.0872
-0.4201 2.6663 -37.3088
2.3000 -35.1100 -0.2865 -0.0869
0.2077 0.1468 -0.0231 -0.0443 -0.0880
-0.4196 2.6703 -37.3117
2.3200 -34.9400 0.1872 -0.1811
0.1761 0.1492 -0.0272 -0.0435 -0.0887
-0.4191 2.6742 -37.3145
2.3400 -35.0900 0.1298 -0.1609
0.1317 0.1418 -0.0311 -0.0425 -0.0894
-0.4185 2.6781 -37.3174
2.3600 -35.2600 -0.2081 -0.0664
0.0814 0.1255 -0.0348 -0.0413 -0.0900
-0.4179 2.6820 -37.3202
2.3800 -35.0800 -0.1582 0.1099
0.0269 0.1020 -0.0381 -0.0399 -0.0906
-0.4173 2.6859 -37.3231
2.4000 -34.6800 0.2558 0.2772 -
0.0316 0.0731 -0.0410 -0.0384 -0.0912
-0.4167 2.6898 -37.3259

2.4200 -34.8600 0.1628 0.2205 -
0.0893 0.0412 -0.0436 -0.0368 -0.0917
-0.4160 2.6937 -37.3288
2.4400 -35.3200 -0.0395 -0.0450 -
0.1365 0.0090 -0.0457 -0.0350 -0.0922
-0.4153 2.6975 -37.3316
2.4600 -35.8800 -0.1882 -0.2823 -
0.1563 -0.0212 -0.0473 -0.0331 -0.0927
-0.4146 2.7014 -37.3345
2.4800 -35.4800 0.2176 -0.2886 -
0.1369 -0.0474 -0.0484 -0.0310 -0.0931
-0.4139 2.7053 -37.3373
2.5000 -35.7100 -0.2887 -0.0564 -
0.0812 -0.0687 -0.0487 -0.0288 -0.0934
-0.4131 2.7091 -37.3402
2.5200 -34.8300 0.2471 0.1621 -
0.0053 -0.0846 -0.0483 -0.0265 -0.0938
-0.4124 2.7129 -37.3430
2.5400 -35.2400 -0.2175 0.1761
0.0636 -0.0952 -0.0470 -0.0241 -0.0941
-0.4115 2.7167 -37.3459
2.5600 -34.8400 0.1747 0.0579
0.1041 -0.1006 -0.0449 -0.0216 -0.0943
-0.4107 2.7205 -37.3487
2.5800 -35.4300 -0.1876 -0.0123
0.1060 -0.1010 -0.0419 -0.0190 -0.0946
-0.4098 2.7243 -37.3516
2.6000 -35.0600 0.2436 0.0215
0.0739 -0.0966 -0.0381 -0.0163 -0.0948
-0.4089 2.7281 -37.3544
2.6200 -35.4100 -0.1779 0.0707
0.0207 -0.0882 -0.0334 -0.0135 -0.0949
-0.4080 2.7319 -37.3573
2.6400 -35.1400 -0.0246 0.0737 -
0.0365 -0.0764 -0.0281 -0.0108 -0.0951
-0.4070 2.7356 -37.3601
2.6600 -35.1400 0.1864 -0.0141 -
0.0790 -0.0618 -0.0221 -0.0079 -0.0951
-0.4060 2.7393 -37.3630
2.6800 -35.4300 -0.1640 -0.1280 -
0.0933 -0.0453 -0.0157 -0.0051 -0.0952
-0.4050 2.7430 -37.3658
2.7000 -35.3200 0.0105 -0.1415 -
0.0730 -0.0276 -0.0089 -0.0023 -0.0953
-0.4039 2.7467 -37.3687
2.7200 -35.1200 0.0557 -0.0546 -
0.0258 -0.0091 -0.0018 0.0006 -0.0953
-0.4028 2.7504 -37.3716
2.7400 -35.1000 -0.0949 0.0424
0.0310 0.0095 0.0054 0.0035 -0.0953
-0.4016 2.7541 -37.3744

2.7600	-34.8300	0.1348	0.0857	
0.0778	0.0273	0.0126	0.0063	-0.0952
	-0.4004	2.7577	-37.3773	
2.7800	-34.9100	-0.0154	0.0812	
0.0962	0.0434	0.0197	0.0091	-0.0951
	-0.3992	2.7614	-37.3801	
2.8000	-34.9900	-0.0941	0.0930	
0.0767	0.0574	0.0264	0.0118	-0.0950
	-0.3980	2.7650	-37.3830	
2.8200	-34.9200	-0.0958	0.1311	
0.0281	0.0688	0.0327	0.0145	-0.0949
	-0.3967	2.7686	-37.3859	
2.8400	-34.6300	0.4435	0.0640	-
0.0279	0.0774	0.0384	0.0171	-0.0948
	-0.3953	2.7722	-37.3887	
2.8600	-35.4900	-0.4082	-0.1344	-
0.0676	0.0831	0.0435	0.0196	-0.0946
	-0.3939	2.7757	-37.3916	
2.8800	-35.2000	0.1097	-0.2270	-
0.0735	0.0859	0.0477	0.0221	-0.0944
	-0.3925	2.7793	-37.3945	
2.9000	-34.9800	0.0328	-0.0749	-
0.0434	0.0862	0.0510	0.0244	-0.0942
	-0.3911	2.7828	-37.3973	
2.9200	-34.8100	-0.1277	0.1623	
0.0066	0.0845	0.0534	0.0266	-0.0940
	-0.3896	2.7863	-37.4002	
2.9400	-34.3800	0.2040	0.2403	
0.0506	0.0812	0.0548	0.0287	-0.0938
	-0.3880	2.7898	-37.4031	
2.9600	-34.8100	-0.1631	0.1190	
0.0681	0.0761	0.0552	0.0306	-0.0935
	-0.3865	2.7933	-37.4059	
2.9800	-34.8500	-0.0058	-0.0253	
0.0507	0.0692	0.0547	0.0324	-0.0932
	-0.3848	2.7967	-37.4088	
3.0000	-34.7900	0.2877	-0.0877	
0.0046	0.0607	0.0535	0.0340	-0.0929
	-0.3832	2.8001	-37.4117	
3.0200	-35.4500	-0.2564	-0.1127	-
0.0474	0.0508	0.0516	0.0355	-0.0926
	-0.3815	2.8035	-37.4145	
3.0400	-35.1400	-0.0376	-0.0917	-
0.0809	0.0402	0.0491	0.0368	-0.0923
	-0.3797	2.8069	-37.4174	
3.0600	-34.9400	0.1927	-0.0050	-
0.0793	0.0293	0.0462	0.0379	-0.0919
	-0.3779	2.8103	-37.4203	
3.0800	-35.1400	-0.0891	0.0749	-
0.0446	0.0182	0.0429	0.0388	-0.0916
	-0.3761	2.8136	-37.4232	

3.1000	-34.9700	-0.0803	0.0885	
0.0067	0.0072	0.0395	0.0396	-0.0912
	-0.3742	2.8170	-37.4260	
3.1200	-34.8700	0.1382	0.0039	
0.0549	-0.0036	0.0359	0.0402	-0.0908
	-0.3722	2.8203	-37.4289	
3.1400	-35.0400	-0.0186	-0.0930	
0.0818	-0.0138	0.0325	0.0405	-0.0905
	-0.3703	2.8235	-37.4318	
3.1600	-35.2600	-0.2135	-0.0756	
0.0786	-0.0232	0.0292	0.0407	-0.0901
	-0.3682	2.8268	-37.4347	
3.1800	-34.5400	0.3663	0.0459	
0.0486	-0.0315	0.0262	0.0407	-0.0897
	-0.3662	2.8300	-37.4375	
3.2000	-35.2500	-0.2488	0.1296	-
0.0005	-0.0380	0.0237	0.0405	-0.0893
	-0.3641	2.8332	-37.4404	
3.2200	-35.2800	-0.1918	0.1059	-
0.0544	-0.0420	0.0217	0.0402	-0.0888
	-0.3619	2.8364	-37.4433	
3.2400	-34.7600	0.3702	-0.0130	-
0.0925	-0.0432	0.0203	0.0396	-0.0884
	-0.3597	2.8396	-37.4462	
3.2600	-35.5400	-0.2739	-0.1719	-
0.0983	-0.0416	0.0196	0.0389	-0.0880
	-0.3575	2.8427	-37.4491	
3.2800	-35.2500	0.1283	-0.1981	-
0.0684	-0.0376	0.0195	0.0380	-0.0875
	-0.3552	2.8458	-37.4520	
3.3000	-35.2400	-0.1652	-0.0359	-
0.0126	-0.0317	0.0200	0.0369	-0.0871
	-0.3528	2.8489	-37.4548	
3.3200	-34.8900	-0.1446	0.1895	
0.0493	-0.0249	0.0212	0.0357	-0.0866
	-0.3504	2.8520	-37.4577	
3.3400	-34.2900	0.3736	0.2130	
0.0970	-0.0182	0.0229	0.0344	-0.0862
	-0.3480	2.8550	-37.4606	
3.3600	-34.9800	-0.0144	-0.0044	
0.1193	-0.0128	0.0252	0.0329	-0.0857
	-0.3455	2.8580	-37.4635	
3.3800	-35.4200	-0.4418	-0.1459	
0.1175	-0.0094	0.0278	0.0313	-0.0853
	-0.3429	2.8610	-37.4664	
3.4000	-34.6600	0.3410	-0.0166	
0.0980	-0.0085	0.0306	0.0296	-0.0848
	-0.3404	2.8640	-37.4693	
3.4200	-34.8000	-0.0623	0.1201	
0.0687	-0.0098	0.0336	0.0278	-0.0843
	-0.3377	2.8669	-37.4722	

3.4400 -34.6700 0.1851 0.0834
0.0361 -0.0130 0.0365 0.0258 -0.0839
-0.3350 2.8699 -37.4751
3.4600 -35.1900 -0.1921 -0.0066
0.0013 -0.0167 0.0394 0.0238 -0.0834
-0.3323 2.8728 -37.4780
3.4800 -35.2800 -0.1431 -0.0544 -
0.0371 -0.0195 0.0419 0.0217 -0.0829
-0.3295 2.8756 -37.4809
3.5000 -34.7500 0.3286 -0.0870 -
0.0776 -0.0197 0.0441 0.0196 -0.0824
-0.3267 2.8785 -37.4838
3.5200 -35.1000 -0.0204 -0.1025 -
0.1151 -0.0163 0.0460 0.0174 -0.0819
-0.3238 2.8813 -37.4867
3.5400 -35.4200 -0.3238 -0.0307 -
0.1432 -0.0087 0.0473 0.0151 -0.0815
-0.3209 2.8841 -37.4896
3.5600 -34.7700 0.1455 0.1253 -
0.1547 0.0032 0.0482 0.0128 -0.0810
-0.3179 2.8868 -37.4925
3.5800 -34.6300 0.2472 0.1635 -
0.1396 0.0187 0.0484 0.0105 -0.0805
-0.3149 2.8896 -37.4954
3.6000 -35.0900 -0.1530 -0.0136 -
0.0906 0.0367 0.0478 0.0081 -0.0800
-0.3119 2.8923 -37.4983
3.6200 -35.1800 -0.1426 -0.1650 -
0.0140 0.0554 0.0465 0.0057 -0.0796
-0.3087 2.8950 -37.5012
3.6400 -34.9300 -0.0164 -0.0927
0.0763 0.0729 0.0442 0.0033 -0.0791
-0.3056 2.8976 -37.5041
3.6600 -34.2800 0.2201 0.0505
0.1601 0.0868 0.0411 0.0010 -0.0787
-0.3024 2.9003 -37.5070
3.6800 -34.7100 -0.1530 0.1268
0.2132 0.0952 0.0370 -0.0014 -0.0782
-0.2991 2.9029 -37.5099
3.7000 -34.6800 -0.0672 0.1458
0.2201 0.0961 0.0319 -0.0038 -0.0778
-0.2958 2.9054 -37.5128
3.7200 -34.4100 0.1097 0.0916
0.1798 0.0889 0.0261 -0.0061 -0.0774
-0.2924 2.9080 -37.5157
3.7400 -34.6500 0.2010 -0.0571
0.1045 0.0740 0.0195 -0.0083 -0.0770
-0.2890 2.9105 -37.5186
3.7600 -35.3600 -0.3064 -0.1959
0.0153 0.0532 0.0124 -0.0105 -0.0766
-0.2855 2.9130 -37.5215

3.7800	-35.2700	-0.1345	-0.1478	-
0.0634	0.0288	0.0048	-0.0127	-0.0762
	-0.2820	2.9155	-37.5245	
3.8000	-34.7800	0.2517	0.0246	-
0.1171	0.0034	-0.0031	-0.0147	-0.0758
	-0.2785	2.9179	-37.5274	
3.8200	-35.0100	0.0406	0.1165	-
0.1415	-0.0206	-0.0110	-0.0167	-0.0754
	-0.2749	2.9203	-37.5303	
3.8400	-35.1700	0.0436	0.0811	-
0.1403	-0.0413	-0.0188	-0.0185	-0.0751
	-0.2712	2.9227	-37.5332	
3.8600	-35.3600	-0.1679	0.0089	-
0.1205	-0.0572	-0.0264	-0.0203	-0.0748
	-0.2675	2.9250	-37.5361	
3.8800	-34.9000	0.2262	-0.0446	-
0.0899	-0.0674	-0.0335	-0.0219	-0.0745
	-0.2638	2.9274	-37.5391	
3.9000	-35.3400	-0.2052	-0.0734	-
0.0518	-0.0721	-0.0400	-0.0234	-0.0742
	-0.2600	2.9296	-37.5420	
3.9200	-35.1600	-0.0581	-0.0387	-
0.0137	-0.0714	-0.0457	-0.0247	-0.0739
	-0.2561	2.9319	-37.5449	
3.9400	-34.8500	0.1891	0.0241	
0.0177	-0.0659	-0.0506	-0.0259	-0.0736
	-0.2522	2.9342	-37.5478	
3.9600	-35.1200	-0.0805	0.0102	
0.0399	-0.0563	-0.0544	-0.0269	-0.0734
	-0.2483	2.9364	-37.5508	
3.9800	-34.9600	0.0938	-0.0800	
0.0554	-0.0440	-0.0572	-0.0278	-0.0731
	-0.2443	2.9385	-37.5537	
4.0000	-35.1400	-0.3008	-0.0417	
0.0655	-0.0303	-0.0589	-0.0286	-0.0729
	-0.2402	2.9407	-37.5566	
4.0200	-34.4100	0.4030	0.1269	
0.0618	-0.0166	-0.0595	-0.0292	-0.0727
	-0.2361	2.9428	-37.5595	
4.0400	-35.2000	-0.3375	0.1636	
0.0368	-0.0034	-0.0591	-0.0296	-0.0726
	-0.2320	2.9449	-37.5625	
4.0600	-34.7400	0.3456	0.0067	-
0.0040	0.0087	-0.0577	-0.0299	-0.0724
	-0.2278	2.9470	-37.5654	
4.0800	-35.5300	-0.3895	-0.1197	-
0.0475	0.0197	-0.0553	-0.0300	-0.0723
	-0.2236	2.9490	-37.5683	
4.1000	-35.1400	0.0867	-0.1176	-
0.0747	0.0295	-0.0520	-0.0299	-0.0722
	-0.2193	2.9510	-37.5713	

4.1200 -34.8700 0.1512 -0.0899 -
0.0706 0.0382 -0.0479 -0.0298 -0.0721
-0.2150 2.9529 -37.5742
4.1400 -35.2000 -0.2077 -0.0334 -
0.0366 0.0450 -0.0431 -0.0294 -0.0720
-0.2106 2.9549 -37.5771
4.1600 -34.7200 0.0318 0.0783
0.0112 0.0495 -0.0377 -0.0289 -0.0720
-0.2062 2.9568 -37.5801
4.1800 -34.7600 -0.0312 0.1699
0.0533 0.0513 -0.0319 -0.0283 -0.0720
-0.2017 2.9587 -37.5830
4.2000 -34.5000 0.2403 0.1376
0.0762 0.0501 -0.0257 -0.0275 -0.0720
-0.1972 2.9605 -37.5860
4.2200 -34.7800 -0.0783 -0.0353
0.0779 0.0455 -0.0193 -0.0266 -0.0720
-0.1926 2.9623 -37.5889
4.2400 -35.0100 0.0401 -0.1971
0.0684 0.0380 -0.0128 -0.0256 -0.0721
-0.1880 2.9641 -37.5918
4.2600 -35.2600 -0.1997 -0.2060
0.0584 0.0280 -0.0064 -0.0244 -0.0722
-0.1833 2.9659 -37.5948
4.2800 -34.9200 0.0042 -0.0621
0.0505 0.0166 -0.0001 -0.0230 -0.0723
-0.1786 2.9676 -37.5977
4.3000 -34.6300 0.0933 0.1434
0.0404 0.0044 0.0059 -0.0216 -0.0725
-0.1739 2.9693 -37.6007
4.3200 -34.7900 -0.0201 0.2593
0.0231 -0.0075 0.0116 -0.0200 -0.0726
-0.1691 2.9709 -37.6036
4.3400 -34.7500 0.1039 0.1666 -
0.0048 -0.0183 0.0169 -0.0183 -0.0729
-0.1642 2.9726 -37.6066
4.3600 -35.0000 0.0240 -0.0793 -
0.0373 -0.0265 0.0218 -0.0166 -0.0731
-0.1593 2.9742 -37.6095
4.3800 -35.4700 -0.2579 -0.2588 -
0.0641 -0.0310 0.0261 -0.0147 -0.0734
-0.1544 2.9757 -37.6125
4.4000 -35.2000 -0.0398 -0.1676 -
0.0765 -0.0309 0.0300 -0.0128 -0.0737
-0.1494 2.9773 -37.6154
4.4200 -34.7600 -0.0690 0.1219 -
0.0731 -0.0263 0.0332 -0.0107 -0.0740
-0.1444 2.9788 -37.6184
4.4400 -34.0600 0.4823 0.3030 -
0.0607 -0.0177 0.0360 -0.0087 -0.0744
-0.1393 2.9802 -37.6213

4.4600 -34.8000 -0.1111 0.2045 -
0.0417 -0.0065 0.0382 -0.0066 -0.0748
-0.1342 2.9817 -37.6243
4.4800 -35.1500 -0.2168 -0.0456 -
0.0160 0.0060 0.0398 -0.0044 -0.0753
-0.1290 2.9831 -37.6273
4.5000 -34.8300 0.1970 -0.2396
0.0191 0.0181 0.0410 -0.0023 -0.0758
-0.1238 2.9845 -37.6302
4.5200 -35.2700 -0.3445 -0.2680
0.0619 0.0283 0.0418 -0.0001 -0.0763
-0.1186 2.9858 -37.6332
4.5400 -34.8300 -0.0185 -0.1020
0.1045 0.0352 0.0423 0.0021 -0.0768
-0.1133 2.9871 -37.6361
4.5600 -34.2100 0.3316 0.1236
0.1309 0.0376 0.0426 0.0042 -0.0774
-0.1080 2.9884 -37.6391
4.5800 -34.5700 -0.1499 0.2127
0.1241 0.0347 0.0427 0.0063 -0.0781
-0.1026 2.9896 -37.6421
4.6000 -34.5100 0.1240 0.1388
0.0797 0.0265 0.0427 0.0084 -0.0788
-0.0972 2.9908 -37.6450
4.6200 -35.0500 -0.1232 -0.0021
0.0112 0.0136 0.0427 0.0104 -0.0795
-0.0917 2.9920 -37.6480
4.6400 -34.6900 0.1879 -0.1254 -
0.0571 -0.0027 0.0427 0.0123 -0.0803
-0.0862 2.9931 -37.6510
4.6600 -35.2100 -0.2233 -0.1583 -
0.1035 -0.0205 0.0427 0.0142 -0.0811
-0.0807 2.9942 -37.6539
4.6800 -35.0200 0.0288 -0.0767 -
0.1198 -0.0377 0.0428 0.0160 -0.0819
-0.0751 2.9953 -37.6569
4.7000 -34.7100 0.1876 0.0187 -
0.1091 -0.0523 0.0430 0.0177 -0.0828
-0.0695 2.9963 -37.6599
4.7200 -34.7900 -0.0368 0.0425 -
0.0805 -0.0627 0.0433 0.0193 -0.0838
-0.0638 2.9974 -37.6628
4.7400 -35.0900 -0.2608 0.0399 -
0.0461 -0.0674 0.0437 0.0208 -0.0847
-0.0581 2.9983 -37.6658
4.7600 -34.5400 0.3028 0.0476 -
0.0171 -0.0657 0.0441 0.0222 -0.0858
-0.0524 2.9993 -37.6688
4.7800 -34.8200 -0.1531 0.0376
0.0054 -0.0574 0.0446 0.0235 -0.0868
-0.0466 3.0002 -37.6718

4.8000 -34.6600 0.0427 0.0094
0.0215 -0.0431 0.0451 0.0247 -0.0879
-0.0408 3.0010 -37.6747
4.8200 -34.9300 -0.2059 -0.0150
0.0326 -0.0237 0.0455 0.0257 -0.0891
-0.0350 3.0019 -37.6777
4.8400 -34.4200 0.2938 -0.0276
0.0406 -0.0006 0.0458 0.0266 -0.0903
-0.0291 3.0027 -37.6807
4.8600 -34.8900 -0.2102 -0.0328
0.0458 0.0240 0.0459 0.0274 -0.0915
-0.0232 3.0034 -37.6837
4.8800 -34.7900 -0.0089 -0.0619
0.0504 0.0479 0.0458 0.0280 -0.0928
-0.0172 3.0042 -37.6867
4.9000 -34.6500 0.0816 -0.0673
0.0565 0.0689 0.0454 0.0285 -0.0941
-0.0112 3.0048 -37.6896
4.9200 -34.5500 -0.0616 0.0196
0.0645 0.0855 0.0446 0.0288 -0.0955
-0.0052 3.0055 -37.6926
4.9400 -34.5700 -0.1021 0.1053
0.0663 0.0962 0.0433 0.0289 -0.0969
0.0009 3.0061 -37.6956
4.9600 -34.0800 0.3922 0.0553
0.0545 0.1004 0.0415 0.0289 -0.0983
0.0070 3.0067 -37.6986
4.9800 -34.9200 -0.3166 -0.0730
0.0293 0.0985 0.0392 0.0288 -0.0998
0.0131 3.0073 -37.7016
5.0000 -34.7900 -0.0622 -0.0973 -
0.0068 0.0912 0.0364 0.0284 -0.1013
0.0193 3.0078 -37.7046
5.0200 -34.5300 0.1498 -0.0148 -
0.0480 0.0799 0.0330 0.0280 -0.1029
0.0255 3.0083 -37.7076
5.0400 -34.8000 0.0642 0.0489 -
0.0829 0.0659 0.0290 0.0274 -0.1045
0.0317 3.0087 -37.7105
5.0600 -34.6300 0.0872 0.0580 -
0.1066 0.0503 0.0246 0.0266 -0.1061
0.0380 3.0091 -37.7135
5.0800 -34.8400 -0.2032 0.0326 -
0.1146 0.0340 0.0198 0.0257 -0.1077
0.0443 3.0095 -37.7165
5.1000 -34.6400 0.1937 -0.0392 -
0.1013 0.0174 0.0146 0.0248 -0.1094
0.0506 3.0098 -37.7195
5.1200 -35.1100 -0.1869 -0.1133 -
0.0626 0.0009 0.0092 0.0237 -0.1111
0.0570 3.0101 -37.7225

5.1400 -34.7300 0.1280 -0.1213
0.0003 -0.0157 0.0037 0.0225 -0.1129
0.0634 3.0104 -37.7255
5.1600 -35.0500 -0.4134 0.0253
0.0766 -0.0322 -0.0016 0.0212 -0.1146
0.0698 3.0106 -37.7285
5.1800 -33.8500 0.6259 0.1988
0.1456 -0.0482 -0.0067 0.0199 -0.1164
0.0762 3.0108 -37.7315
5.2000 -34.6300 -0.2025 0.1732
0.1850 -0.0634 -0.0113 0.0185 -0.1182
0.0827 3.0109 -37.7345
5.2200 -34.9000 -0.3046 0.0441
0.1792 -0.0772 -0.0155 0.0170 -0.1200
0.0892 3.0110 -37.7375
5.2400 -34.7400 0.0645 -0.0395
0.1259 -0.0889 -0.0191 0.0156 -0.1219
0.0957 3.0111 -37.7405
5.2600 -34.5600 0.2983 -0.0780
0.0395 -0.0974 -0.0220 0.0140 -0.1237
0.1023 3.0112 -37.7435
5.2800 -35.2700 -0.3000 -0.0910 -
0.0571 -0.1019 -0.0242 0.0125 -0.1256
0.1089 3.0112 -37.7465
5.3000 -35.0300 0.0613 -0.0486 -
0.1419 -0.1017 -0.0256 0.0109 -0.1275
0.1155 3.0111 -37.7495
5.3200 -34.7700 0.1695 -0.0203 -
0.1977 -0.0962 -0.0262 0.0094 -0.1294
0.1221 3.0110 -37.7525
5.3400 -35.3900 -0.2914 -0.0402 -
0.2157 -0.0850 -0.0260 0.0078 -0.1313
0.1287 3.0109 -37.7555
5.3600 -34.9600 0.0795 -0.0369 -
0.1932 -0.0685 -0.0249 0.0063 -0.1332
0.1354 3.0108 -37.7585
5.3800 -34.7800 0.1377 0.0183 -
0.1364 -0.0477 -0.0232 0.0048 -0.1351
0.1421 3.0106 -37.7615
5.4000 -35.1000 -0.2549 0.0673 -
0.0600 -0.0241 -0.0208 0.0033 -0.1370
0.1488 3.0104 -37.7645
5.4200 -34.3500 0.2660 0.0665
0.0188 0.0006 -0.0178 0.0019 -0.1389
0.1556 3.0101 -37.7676
5.4400 -34.8100 -0.0032 0.0115
0.0873 0.0244 -0.0143 0.0006 -0.1408
0.1623 3.0098 -37.7706
5.4600 -34.9100 -0.1550 -0.0512
0.1365 0.0454 -0.0104 -0.0007 -0.1427
0.1691 3.0094 -37.7736

5.4800 -34.4700 0.1210 -0.0598
0.1616 0.0619 -0.0063 -0.0019 -0.1446
0.1759 3.0091 -37.7766
5.5000 -34.5300 -0.0012 -0.0234
0.1655 0.0724 -0.0020 -0.0031 -0.1465
0.1827 3.0086 -37.7796
5.5200 -34.7000 -0.2279 0.0514
0.1535 0.0764 0.0023 -0.0041 -0.1483
0.1896 3.0082 -37.7826
5.5400 -34.3600 0.0846 0.1392
0.1273 0.0740 0.0065 -0.0051 -0.1502
0.1964 3.0077 -37.7856
5.5600 -34.2200 0.3140 0.1166
0.0908 0.0663 0.0105 -0.0061 -0.1520
0.2033 3.0071 -37.7887
5.5800 -35.0600 -0.3546 -0.0801
0.0528 0.0541 0.0142 -0.0070 -0.1538
0.2102 3.0065 -37.7917
5.6000 -34.7800 0.0996 -0.2378
0.0191 0.0393 0.0175 -0.0078 -0.1556
0.2171 3.0059 -37.7947
5.6200 -35.1900 -0.5307 -0.0605 -
0.0074 0.0236 0.0205 -0.0086 -0.1574
0.2240 3.0053 -37.7977
5.6400 -33.8800 0.8073 0.1952 -
0.0286 0.0091 0.0229 -0.0093 -0.1592
0.2310 3.0046 -37.8007
5.6600 -35.2000 -0.5157 0.1483 -
0.0516 -0.0025 0.0248 -0.0100 -0.1609
0.2379 3.0038 -37.8038
5.6800 -35.1800 -0.3298 -0.0321 -
0.0757 -0.0096 0.0262 -0.0107 -0.1626
0.2449 3.0030 -37.8068
5.7000 -34.5100 0.4075 -0.1345 -
0.0966 -0.0108 0.0271 -0.0113 -0.1643
0.2519 3.0022 -37.8098
5.7200 -35.2500 -0.2614 -0.1269 -
0.1080 -0.0060 0.0274 -0.0119 -0.1659
0.2589 3.0014 -37.8128
5.7400 -34.6900 0.1342 -0.0187 -
0.1057 0.0041 0.0271 -0.0125 -0.1675
0.2659 3.0005 -37.8159
5.7600 -34.7100 -0.0990 0.1085 -
0.0891 0.0181 0.0264 -0.0131 -0.1691
0.2729 2.9995 -37.8189
5.7800 -34.5500 0.0654 0.1402 -
0.0590 0.0344 0.0252 -0.0136 -0.1707
0.2800 2.9985 -37.8219
5.8000 -34.5000 0.0863 0.0229 -
0.0183 0.0505 0.0237 -0.0142 -0.1722
0.2870 2.9975 -37.8249

5.8200 -34.7100 0.0326 -0.1772
0.0312 0.0645 0.0217 -0.0148 -0.1737
0.2941 2.9964 -37.8280
5.8400 -35.0200 -0.2679 -0.2276
0.0862 0.0743 0.0195 -0.0154 -0.1751
0.3011 2.9953 -37.8310
5.8600 -34.7100 -0.1850 -0.0067
0.1371 0.0783 0.0171 -0.0160 -0.1765
0.3082 2.9942 -37.8340
5.8800 -33.9500 0.3037 0.2631
0.1671 0.0758 0.0144 -0.0167 -0.1778
0.3153 2.9930 -37.8371
5.9000 -34.2000 0.1081 0.2690
0.1591 0.0669 0.0115 -0.0173 -0.1791
0.3224 2.9917 -37.8401
5.9200 -34.6100 -0.1215 0.0414
0.1099 0.0527 0.0084 -0.0180 -0.1804
0.3295 2.9905 -37.8431
5.9400 -34.9100 -0.1335 -0.1603
0.0331 0.0347 0.0052 -0.0187 -0.1816
0.3366 2.9892 -37.8462
5.9600 -34.7300 0.1383 -0.1597 -
0.0502 0.0148 0.0020 -0.0195 -0.1828
0.3437 2.9878 -37.8492
5.9800 -34.8500 -0.0062 -0.0311 -
0.1177 -0.0058 -0.0012 -0.0203 -0.1839
0.3508 2.9864 -37.8523
6.0000 -35.0300 -0.1037 0.0863 -
0.1531 -0.0256 -0.0043 -0.0211 -0.1850
0.3580 2.9849 -37.8553
6.0200 -34.7700 0.1607 0.0592 -
0.1483 -0.0435 -0.0071 -0.0220 -0.1861
0.3651 2.9835 -37.8583
6.0400 -34.7800 0.1313 -0.0683 -
0.1096 -0.0590 -0.0096 -0.0229 -0.1870
0.3722 2.9819 -37.8614
6.0600 -35.4900 -0.5765 -0.0891 -
0.0493 -0.0713 -0.0118 -0.0238 -0.1880
0.3793 2.9804 -37.8644
6.0800 -34.2700 0.6050 -0.0053
0.0206 -0.0805 -0.0137 -0.0248 -0.1889
0.3865 2.9787 -37.8675
6.1000 -35.1700 -0.4166 0.0065
0.0877 -0.0864 -0.0150 -0.0258 -0.1897
0.3936 2.9771 -37.8705
6.1200 -34.7100 -0.0840 0.0148
0.1340 -0.0887 -0.0159 -0.0269 -0.1905
0.4008 2.9754 -37.8736
6.1400 -34.3900 0.1667 0.0557
0.1458 -0.0872 -0.0163 -0.0280 -0.1912
0.4079 2.9736 -37.8766

6.1600 -34.4200 0.1772 0.0292
0.1212 -0.0828 -0.0160 -0.0291 -0.1918
0.4150 2.9719 -37.8796
6.1800 -34.8900 -0.0845 -0.0506
0.0680 -0.0762 -0.0151 -0.0303 -0.1925
0.4222 2.9700 -37.8827
6.2000 -34.8900 -0.1045 -0.0698
0.0036 -0.0677 -0.0135 -0.0314 -0.1930
0.4293 2.9682 -37.8857
6.2200 -34.5800 0.0041 -0.0152 -
0.0525 -0.0572 -0.0112 -0.0326 -0.1935
0.4364 2.9662 -37.8888
6.2400 -34.6000 0.1925 0.0397 -
0.0912 -0.0442 -0.0083 -0.0338 -0.1940
0.4436 2.9643 -37.8918
6.2600 -35.1300 -0.3228 0.0486 -
0.1074 -0.0286 -0.0048 -0.0350 -0.1944
0.4507 2.9623 -37.8949
6.2800 -34.3800 0.3630 0.0268 -
0.1011 -0.0109 -0.0008 -0.0362 -0.1947
0.4578 2.9602 -37.8980
6.3000 -35.2000 -0.3212 0.0112 -
0.0811 0.0075 0.0037 -0.0374 -0.1950
0.4649 2.9581 -37.9010
6.3200 -34.5500 0.1915 0.0120 -
0.0590 0.0252 0.0087 -0.0386 -0.1952
0.4720 2.9560 -37.9041
6.3400 -34.6000 0.1200 -0.0127 -
0.0379 0.0406 0.0141 -0.0398 -0.1954
0.4791 2.9538 -37.9071
6.3600 -34.8600 -0.1501 -0.0795 -
0.0126 0.0527 0.0199 -0.0409 -0.1955
0.4862 2.9516 -37.9102
6.3800 -34.9900 -0.2766 -0.0828
0.0242 0.0606 0.0261 -0.0420 -0.1955
0.4933 2.9493 -37.9132
6.4000 -34.2300 0.3510 0.0244
0.0703 0.0639 0.0325 -0.0431 -0.1955
0.5004 2.9470 -37.9163
6.4200 -34.6200 -0.3949 0.1004
0.1143 0.0620 0.0390 -0.0440 -0.1954
0.5075 2.9446 -37.9193
6.4400 -34.0700 0.4563 0.0500
0.1398 0.0552 0.0456 -0.0450 -0.1953
0.5145 2.9422 -37.9224
6.4600 -34.9700 -0.4466 -0.0217
0.1317 0.0439 0.0520 -0.0458 -0.1951
0.5216 2.9398 -37.9255
6.4800 -34.3200 0.2076 0.0240
0.0875 0.0299 0.0582 -0.0465 -0.1949
0.5286 2.9373 -37.9285

6.5000 -34.5000 0.0427 0.0685
0.0152 0.0146 0.0640 -0.0472 -0.1946
0.5356 2.9348 -37.9316
6.5200 -34.5300 0.1029 -0.0038 -
0.0611 -0.0003 0.0692 -0.0477 -0.1943
0.5426 2.9322 -37.9347
6.5400 -35.0100 -0.0896 -0.1272 -
0.1161 -0.0132 0.0738 -0.0480 -0.1939
0.5496 2.9295 -37.9377
6.5600 -34.9700 -0.0269 -0.1508 -
0.1339 -0.0227 0.0777 -0.0483 -0.1934
0.5566 2.9269 -37.9408
6.5800 -34.9800 -0.1350 -0.0434 -
0.1140 -0.0279 0.0808 -0.0484 -0.1929
0.5636 2.9241 -37.9439
6.6000 -34.4500 0.2112 0.0914 -
0.0675 -0.0285 0.0829 -0.0483 -0.1924
0.5705 2.9214 -37.9469
6.6200 -34.3700 0.1979 0.1327 -
0.0104 -0.0248 0.0841 -0.0480 -0.1918
0.5775 2.9186 -37.9500
6.6400 -34.6100 -0.1455 0.0619
0.0406 -0.0174 0.0843 -0.0476 -0.1911
0.5844 2.9157 -37.9531
6.6600 -34.5700 0.0492 -0.0337
0.0724 -0.0072 0.0835 -0.0470 -0.1904
0.5913 2.9128 -37.9561
6.6800 -34.7400 -0.2003 -0.0578
0.0816 0.0045 0.0815 -0.0463 -0.1896
0.5981 2.9098 -37.9592
6.7000 -34.3700 0.1312 0.0111
0.0735 0.0165 0.0784 -0.0453 -0.1888
0.6050 2.9068 -37.9623
6.7200 -34.4900 -0.0213 0.0699
0.0529 0.0276 0.0742 -0.0442 -0.1880
0.6118 2.9038 -37.9653
6.7400 -34.5200 0.1291 0.0188
0.0256 0.0372 0.0690 -0.0429 -0.1871
0.6186 2.9007 -37.9684
6.7600 -34.5800 0.0609 -0.0901 -
0.0023 0.0448 0.0627 -0.0414 -0.1861
0.6254 2.8976 -37.9715
6.7800 -35.1200 -0.3132 -0.1435 -
0.0227 0.0502 0.0554 -0.0398 -0.1851
0.6321 2.8944 -37.9746
6.8000 -34.6900 -0.0582 -0.0478 -
0.0287 0.0532 0.0471 -0.0380 -0.1841
0.6389 2.8912 -37.9776
6.8200 -34.3200 0.1751 0.1234 -
0.0185 0.0537 0.0380 -0.0360 -0.1830
0.6456 2.8879 -37.9807

6.8400 -34.3600 0.0919 0.1647
0.0022 0.0520 0.0282 -0.0338 -0.1819
0.6523 2.8846 -37.9838
6.8600 -34.7200 -0.1409 0.0253
0.0244 0.0487 0.0176 -0.0315 -0.1807
0.6589 2.8812 -37.9869
6.8800 -34.5600 0.0979 -0.0957
0.0367 0.0442 0.0064 -0.0290 -0.1795
0.6655 2.8778 -37.9900
6.9000 -34.9700 -0.3215 -0.0423
0.0317 0.0389 -0.0052 -0.0264 -0.1782
0.6721 2.8744 -37.9930
6.9200 -34.3200 0.1898 0.0546
0.0124 0.0329 -0.0172 -0.0236 -0.1769
0.6787 2.8708 -37.9961
6.9400 -34.2600 0.3311 0.0485 -
0.0123 0.0263 -0.0294 -0.0207 -0.1756
0.6852 2.8673 -37.9992
6.9600 -35.1100 -0.4904 -0.0205 -
0.0327 0.0191 -0.0418 -0.0177 -0.1742
0.6917 2.8637 -38.0023
6.9800 -34.7300 0.0037 -0.0205 -
0.0410 0.0112 -0.0541 -0.0145 -0.1728
0.6981 2.8601 -38.0054
7.0000 -34.4700 0.2405 -0.0137 -
0.0346 0.0028 -0.0663 -0.0111 -0.1713
0.7045 2.8564 -38.0084
7.0200 -34.8200 -0.0869 -0.0380 -
0.0160 -0.0064 -0.0780 -0.0077 -0.1698
0.7109 2.8526 -38.0115
7.0400 -34.9000 -0.1121 -0.0230
0.0093 -0.0165 -0.0891 -0.0041 -0.1682
0.7173 2.8488 -38.0146
7.0600 -34.7700 -0.0329 0.0604
0.0346 -0.0276 -0.0992 -0.0004 -0.1666
0.7236 2.8450 -38.0177
7.0800 -34.4000 0.2954 0.0808
0.0481 -0.0390 -0.1082 0.0033 -0.1650
0.7298 2.8411 -38.0208
7.1000 -34.9500 -0.2784 0.0321
0.0412 -0.0499 -0.1158 0.0071 -0.1633
0.7360 2.8372 -38.0239
7.1200 -34.9700 -0.2030 0.0218
0.0184 -0.0590 -0.1220 0.0110 -0.1615
0.7422 2.8333 -38.0270
7.1400 -34.3600 0.5072 0.0094 -
0.0084 -0.0655 -0.1264 0.0148 -0.1598
0.7483 2.8292 -38.0301
7.1600 -35.2800 -0.5293 -0.0531 -
0.0249 -0.0685 -0.1290 0.0187 -0.1579
0.7544 2.8252 -38.0331

7.1800 -34.7400 0.2128 -0.0893 -
0.0232 -0.0672 -0.1296 0.0226 -0.1561
0.7604 2.8211 -38.0362
7.2000 -34.6800 0.1887 -0.0889 -
0.0073 -0.0618 -0.1282 0.0264 -0.1542
0.7664 2.8169 -38.0393
7.2200 -34.9300 -0.1013 -0.0224
0.0084 -0.0527 -0.1247 0.0301 -0.1523
0.7724 2.8127 -38.0424
7.2400 -34.8700 -0.2172 0.1075
0.0111 -0.0404 -0.1192 0.0338 -0.1503
0.7783 2.8085 -38.0455
7.2600 -34.1200 0.4895 0.1368 -
0.0019 -0.0258 -0.1117 0.0373 -0.1483
0.7841 2.8042 -38.0486
7.2800 -34.8400 -0.2292 0.0019 -
0.0247 -0.0102 -0.1023 0.0407 -0.1462
0.7899 2.7998 -38.0517
7.3000 -34.9900 -0.1374 -0.1062 -
0.0454 0.0049 -0.0911 0.0439 -0.1441
0.7956 2.7954 -38.0548
7.3200 -34.7300 0.1537 -0.1088 -
0.0514 0.0176 -0.0782 0.0469 -0.1419
0.8012 2.7910 -38.0579
7.3400 -34.6400 0.0355 -0.0557 -
0.0363 0.0262 -0.0637 0.0497 -0.1398
0.8068 2.7865 -38.0610
7.3600 -34.7400 -0.2129 0.0146 -
0.0012 0.0300 -0.0478 0.0523 -0.1375
0.8124 2.7820 -38.0641
7.3800 -34.3100 0.2029 0.0552
0.0426 0.0285 -0.0305 0.0546 -0.1353
0.8179 2.7774 -38.0672
7.4000 -34.5500 -0.0448 0.0188
0.0790 0.0218 -0.0121 0.0567 -0.1330
0.8233 2.7728 -38.0703
7.4200 -34.6200 -0.0915 -0.0285
0.0950 0.0107 0.0072 0.0585 -0.1307
0.8286 2.7682 -38.0734
7.4400 -34.5000 0.0017 0.0363
0.0833 -0.0038 0.0273 0.0599 -0.1283
0.8339 2.7634 -38.0765
7.4600 -34.4400 -0.1828 0.1672
0.0429 -0.0203 0.0479 0.0611 -0.1259
0.8392 2.7587 -38.0796
7.4800 -33.8800 0.4518 0.1862 -
0.0172 -0.0369 0.0687 0.0619 -0.1234
0.8443 2.7539 -38.0827
7.5000 -34.7700 -0.2007 -0.0141 -
0.0804 -0.0515 0.0894 0.0624 -0.1209
0.8494 2.7490 -38.0858

7.5200 -34.7800 0.0031 -0.2306 -
0.1264 -0.0622 0.1098 0.0625 -0.1184
0.8544 2.7441 -38.0889
7.5400 -35.0400 -0.2485 -0.2230 -
0.1381 -0.0674 0.1297 0.0622 -0.1159
0.8593 2.7392 -38.0920
7.5600 -34.3400 0.3079 -0.0335 -
0.1107 -0.0664 0.1488 0.0615 -0.1133
0.8642 2.7342 -38.0951
7.5800 -34.5200 -0.2410 0.1324 -
0.0539 -0.0591 0.1668 0.0604 -0.1106
0.8690 2.7292 -38.0982
7.6000 -34.0800 0.1496 0.1180
0.0130 -0.0468 0.1837 0.0588 -0.1079
0.8737 2.7241 -38.1013
7.6200 -34.0100 0.3492 0.0078
0.0681 -0.0307 0.1991 0.0568 -0.1052
0.8783 2.7190 -38.1044
7.6400 -34.8800 -0.5136 -0.0392
0.0961 -0.0122 0.2128 0.0543 -0.1025
0.8829 2.7138 -38.1076
7.6600 -33.9000 0.4110 0.0392
0.0961 0.0073 0.2246 0.0514 -0.0997
0.8874 2.7086 -38.1107
7.6800 -34.0100 0.0768 0.0861
0.0740 0.0263 0.2343 0.0480 -0.0968
0.8917 2.7033 -38.1138
7.7000 -34.6500 -0.3342 0.0544
0.0416 0.0436 0.2416 0.0441 -0.0940
0.8960 2.6980 -38.1169
7.7200 -34.2600 0.0717 -0.0061
0.0151 0.0581 0.2464 0.0397 -0.0910
0.9003 2.6927 -38.1200
7.7400 -34.0900 0.2507 -0.1002
0.0078 0.0701 0.2484 0.0348 -0.0881
0.9044 2.6873 -38.1231
7.7600 -34.7500 -0.2258 -0.1959
0.0211 0.0806 0.2474 0.0295 -0.0851
0.9084 2.6818 -38.1262
7.7800 -34.3500 -0.0440 -0.1497
0.0442 0.0906 0.2432 0.0238 -0.0820
0.9124 2.6763 -38.1293
7.8000 -34.1300 0.0031 0.0890
0.0571 0.1014 0.2358 0.0177 -0.0789
0.9162 2.6708 -38.1324
7.8200 -33.7600 0.2486 0.2925
0.0360 0.1135 0.2251 0.0111 -0.0758
0.9200 2.6652 -38.1356
7.8400 -34.3500 -0.2334 0.2472 -
0.0300 0.1270 0.2112 0.0043 -0.0726
0.9236 2.6596 -38.1387

7.8600 -34.4000 0.0920 0.0521 -
0.1266 0.1416 0.1941 -0.0029 -0.0694
0.9272 2.6539 -38.1418
7.8800 -34.6200 0.0536 -0.1214 -
0.2211 0.1565 0.1740 -0.0104 -0.0661
0.9307 2.6482 -38.1449
7.9000 -34.9800 -0.2662 -0.2095 -
0.2782 0.1711 0.1512 -0.0180 -0.0627
0.9340 2.6425 -38.1480
7.9200 -34.5100 0.3778 -0.2354 -
0.2729 0.1839 0.1259 -0.0259 -0.0593
0.9373 2.6366 -38.1511
7.9400 -34.7800 -0.0341 -0.1685 -
0.2018 0.1929 0.0985 -0.0339 -0.0558
0.9405 2.6308 -38.1543
7.9600 -34.8300 -0.4365 0.0377 -
0.0861 0.1959 0.0695 -0.0420 -0.0523
0.9435 2.6249 -38.1574
7.9800 -33.6000 0.4850 0.2748
0.0464 0.1913 0.0392 -0.0502 -0.0487
0.9465 2.6190 -38.1605
8.0000 -34.0400 -0.0780 0.2875
0.1722 0.1772 0.0081 -0.0584 -0.0450
0.9494 2.6130 -38.1636
8.0200 -34.4000 -0.1779 0.0519
0.2696 0.1522 -0.0232 -0.0665 -0.0413
0.9521 2.6070 -38.1667
8.0400 -34.4300 0.1592 -0.2145
0.3280 0.1161 -0.0544 -0.0746 -0.0375
0.9547 2.6009 -38.1699
8.0600 -35.0500 -0.2477 -0.2856
0.3468 0.0692 -0.0848 -0.0826 -0.0336
0.9573 2.5948 -38.1730
8.0800 -34.5100 0.0367 -0.0823
0.3258 0.0127 -0.1139 -0.0904 -0.0297
0.9597 2.5886 -38.1761
8.1000 -34.5000 -0.0522 0.2555
0.2593 -0.0500 -0.1413 -0.0980 -0.0257
0.9620 2.5824 -38.1792
8.1200 -33.9600 0.5675 0.3402
0.1435 -0.1146 -0.1664 -0.1054 -0.0216
0.9642 2.5762 -38.1824
8.1400 -35.4400 -0.4785 0.0593 -
0.0124 -0.1762 -0.1887 -0.1124 -0.0174
0.9663 2.5699 -38.1855
8.1600 -35.6800 -0.0247 -0.2122 -
0.1813 -0.2298 -0.2079 -0.1192 -0.0131
0.9682 2.5636 -38.1886
8.1800 -35.5400 0.1744 -0.2177 -
0.3277 -0.2709 -0.2234 -0.1256 -0.0088
0.9701 2.5572 -38.1917

8.2000 -36.0600 -0.0648 -0.1134 -
0.4134 -0.2955 -0.2348 -0.1316 -0.0044
0.9718 2.5508 -38.1949
8.2200 -35.8800 0.1222 -0.0943 -
0.4133 -0.3011 -0.2418 -0.1371 0.0002
0.9734 2.5443 -38.1980
8.2400 -36.0300 -0.2565 -0.1257 -
0.3235 -0.2871 -0.2443 -0.1422 0.0048
0.9749 2.5378 -38.2011
8.2600 -35.2100 0.3195 -0.0986 -
0.1650 -0.2559 -0.2424 -0.1467 0.0095
0.9763 2.5313 -38.2043
8.2800 -35.6800 -0.4706 0.0399
0.0190 -0.2111 -0.2362 -0.1507 0.0142
0.9776 2.5247 -38.2074
8.3000 -34.5000 0.3750 0.2223
0.1792 -0.1570 -0.2261 -0.1542 0.0191
0.9787 2.5180 -38.2105
8.3200 -34.7000 0.0480 0.2550
0.2775 -0.0975 -0.2124 -0.1572 0.0241
0.9797 2.5114 -38.2137
8.3400 -34.8700 -0.2546 0.1105
0.2996 -0.0365 -0.1956 -0.1595 0.0292
0.9806 2.5046 -38.2168
8.3600 -34.9500 -0.1127 -0.0448
0.2534 0.0227 -0.1760 -0.1613 0.0343
0.9814 2.4979 -38.2199
8.3800 -34.4100 0.4456 -0.1161
0.1644 0.0767 -0.1542 -0.1626 0.0396
0.9820 2.4911 -38.2230
8.4000 -35.3600 -0.4566 -0.0911
0.0644 0.1227 -0.1305 -0.1632 0.0450
0.9825 2.4842 -38.2262
8.4200 -34.7900 0.0319 -0.0200 -
0.0175 0.1580 -0.1055 -0.1634 0.0504
0.9829 2.4774 -38.2293
8.4400 -34.4600 0.3479 0.0059 -
0.0666 0.1814 -0.0794 -0.1629 0.0560
0.9832 2.4704 -38.2325
8.4600 -35.0700 -0.2273 -0.0092 -
0.0786 0.1931 -0.0527 -0.1620 0.0617
0.9833 2.4635 -38.2356
8.4800 -34.7000 -0.1622 0.0288 -
0.0615 0.1938 -0.0258 -0.1605 0.0675
0.9833 2.4565 -38.2387
8.5000 -34.4300 0.3430 0.0541 -
0.0297 0.1850 0.0009 -0.1585 0.0734
0.9832 2.4494 -38.2419
8.5200 -34.8300 -0.0372 -0.0387
0.0014 0.1680 0.0271 -0.1560 0.0793
0.9830 2.4423 -38.2450

8.5400 -34.9900 -0.0702 -0.1300
0.0220 0.1445 0.0524 -0.1530 0.0854
0.9826 2.4352 -38.2481
8.5600 -34.5900 -0.0310 -0.0609
0.0269 0.1158 0.0765 -0.1495 0.0916
0.9821 2.4280 -38.2513
8.5800 -34.3500 0.1483 0.1038
0.0167 0.0835 0.0992 -0.1456 0.0979
0.9815 2.4208 -38.2544
8.6000 -34.8300 -0.3798 0.2135 -
0.0026 0.0494 0.1202 -0.1412 0.1043
0.9807 2.4136 -38.2576
8.6200 -34.1800 0.4935 0.1142 -
0.0200 0.0152 0.1393 -0.1365 0.1108
0.9798 2.4063 -38.2607
8.6400 -35.0200 0.0376 -0.1638 -
0.0269 -0.0172 0.1565 -0.1313 0.1174
0.9788 2.3989 -38.2638
8.6600 -35.4600 -0.4841 -0.3359 -
0.0157 -0.0461 0.1714 -0.1258 0.1240
0.9777 2.3916 -38.2670
8.6800 -35.2100 -0.2565 -0.1330
0.0113 -0.0702 0.1841 -0.1199 0.1308
0.9764 2.3841 -38.2701
8.7000 -34.0100 0.5821 0.2114
0.0387 -0.0883 0.1944 -0.1136 0.1376
0.9750 2.3767 -38.2733
8.7200 -34.9200 -0.3180 0.2920
0.0440 -0.0995 0.2022 -0.1070 0.1446
0.9734 2.3692 -38.2764
8.7400 -34.4900 0.2250 0.1119
0.0169 -0.1035 0.2075 -0.1000 0.1516
0.9718 2.3617 -38.2795
8.7600 -35.3400 -0.6107 -0.0519 -
0.0336 -0.0998 0.2104 -0.0928 0.1587
0.9700 2.3541 -38.2827
8.7800 -34.2100 0.7095 -0.1484 -
0.0821 -0.0890 0.2107 -0.0852 0.1658
0.9680 2.3465 -38.2858
8.8000 -35.5700 -0.5795 -0.1655 -
0.1044 -0.0716 0.2087 -0.0774 0.1730
0.9660 2.3389 -38.2890
8.8200 -35.2200 -0.2771 -0.0898 -
0.0854 -0.0493 0.2042 -0.0692 0.1803
0.9638 2.3312 -38.2921
8.8400 -34.0800 0.7377 -0.0117 -
0.0289 -0.0241 0.1975 -0.0608 0.1877
0.9615 2.3234 -38.2953
8.8600 -35.1500 -0.6308 -0.0122
0.0502 0.0017 0.1886 -0.0522 0.1951
0.9590 2.3157 -38.2984

8.8800 -34.5700 0.0381 0.0238
0.1303 0.0266 0.1775 -0.0433 0.2025
0.9564 2.3079 -38.3016
8.9000 -34.1700 0.1968 0.0858
0.1866 0.0494 0.1644 -0.0342 0.2100
0.9537 2.3001 -38.3047
8.9200 -34.3000 0.0985 0.0801
0.1969 0.0693 0.1494 -0.0249 0.2175
0.9509 2.2922 -38.3079
8.9400 -34.7100 -0.2103 0.0735
0.1545 0.0857 0.1327 -0.0155 0.2250
0.9479 2.2843 -38.3110
8.9600 -34.5700 -0.1016 0.1138
0.0658 0.0987 0.1145 -0.0059 0.2325
0.9448 2.2763 -38.3142
8.9800 -34.4700 0.2007 0.0986 -
0.0495 0.1082 0.0949 0.0039 0.2401
0.9416 2.2683 -38.3173
9.0000 -34.6800 0.1604 -0.0769 -
0.1596 0.1139 0.0743 0.0138 0.2477
0.9382 2.2603 -38.3205
9.0200 -35.5700 -0.3421 -0.2634 -
0.2302 0.1151 0.0529 0.0238 0.2552
0.9348 2.2523 -38.3236
9.0400 -35.1800 -0.0102 -0.2341 -
0.2359 0.1113 0.0311 0.0340 0.2628
0.9312 2.2442 -38.3268
9.0600 -34.9200 0.0169 -0.0322 -
0.1717 0.1022 0.0091 0.0442 0.2703
0.9274 2.2360 -38.3299
9.0800 -34.7300 0.1219 0.0957 -
0.0502 0.0879 -0.0128 0.0545 0.2778
0.9236 2.2279 -38.3331
9.1000 -34.6100 0.1030 0.0653
0.0962 0.0689 -0.0343 0.0649 0.2853
0.9196 2.2197 -38.3362
9.1200 -34.9800 -0.3258 0.0127
0.2270 0.0457 -0.0550 0.0753 0.2927
0.9155 2.2115 -38.3394
9.1400 -34.4700 0.0085 0.0722
0.3070 0.0188 -0.0747 0.0858 0.3001
0.9112 2.2032 -38.3425
9.1600 -34.3400 0.2690 0.1780
0.3116 -0.0103 -0.0930 0.0963 0.3074
0.9069 2.1949 -38.3457
9.1800 -34.7000 -0.1868 0.1978
0.2334 -0.0401 -0.1097 0.1068 0.3147
0.9024 2.1865 -38.3488
9.2000 -34.8300 -0.1508 0.1482
0.0902 -0.0683 -0.1244 0.1174 0.3219
0.8978 2.1782 -38.3520

9.2200 -34.9200 0.3045 0.0362 -
0.0815 -0.0931 -0.1369 0.1279 0.3290
0.8931 2.1698 -38.3551
9.2400 -35.6200 -0.1816 -0.1784 -
0.2333 -0.1122 -0.1469 0.1384 0.3360
0.8882 2.1613 -38.3583
9.2600 -35.6600 0.0796 -0.3574 -
0.3216 -0.1238 -0.1540 0.1488 0.3429
0.8833 2.1528 -38.3615
9.2800 -36.0500 -0.2842 -0.3120 -
0.3217 -0.1265 -0.1581 0.1591 0.3498
0.8782 2.1443 -38.3646
9.3000 -35.1900 0.2576 -0.0759 -
0.2396 -0.1209 -0.1590 0.1694 0.3565
0.8729 2.1358 -38.3678
9.3200 -35.0400 0.0211 0.1491 -
0.1101 -0.1087 -0.1568 0.1796 0.3631
0.8676 2.1272 -38.3709
9.3400 -34.9100 -0.0870 0.2610
0.0255 -0.0920 -0.1514 0.1896 0.3696
0.8622 2.1186 -38.3741
9.3600 -34.5600 0.2005 0.2580
0.1323 -0.0729 -0.1430 0.1995 0.3760
0.8566 2.1100 -38.3773
9.3800 -34.8700 -0.1903 0.1581
0.1859 -0.0534 -0.1317 0.2091 0.3822
0.8509 2.1013 -38.3804
9.4000 -34.8000 0.0358 0.0218
0.1843 -0.0358 -0.1178 0.2186 0.3882
0.8451 2.0926 -38.3836
9.4200 -34.8300 0.0996 -0.1189
0.1474 -0.0218 -0.1014 0.2279 0.3941
0.8392 2.0839 -38.3867
9.4400 -35.1200 -0.0346 -0.2097
0.0994 -0.0123 -0.0829 0.2369 0.3999
0.8331 2.0751 -38.3899
9.4600 -35.1200 -0.1134 -0.1803
0.0598 -0.0072 -0.0627 0.2456 0.4055
0.8270 2.0663 -38.3931
9.4800 -34.9200 -0.0013 -0.0534
0.0367 -0.0056 -0.0410 0.2540 0.4109
0.8207 2.0575 -38.3962
9.5000 -34.6600 0.1715 0.0522
0.0240 -0.0056 -0.0183 0.2622 0.4161
0.8144 2.0486 -38.3994
9.5200 -35.0100 -0.2513 0.1026
0.0064 -0.0054 0.0050 0.2699 0.4211
0.8079 2.0397 -38.4025
9.5400 -34.5600 0.2636 0.1138 -
0.0281 -0.0025 0.0285 0.2774 0.4259
0.8013 2.0308 -38.4057

9.5600 -35.0200 -0.1988 0.0948 -
0.0808 0.0050 0.0518 0.2844 0.4304
0.7946 2.0218 -38.4089
9.5800 -35.0200 -0.1728 0.0567 -
0.1360 0.0174 0.0744 0.2910 0.4348
0.7878 2.0128 -38.4120
9.6000 -34.5500 0.3991 -0.0425 -
0.1684 0.0341 0.0961 0.2973 0.4389
0.7808 2.0038 -38.4152
9.6200 -35.0800 0.0423 -0.1848 -
0.1571 0.0533 0.1163 0.3031 0.4428
0.7738 1.9948 -38.4184
9.6400 -35.3800 -0.4087 -0.1933 -
0.0971 0.0729 0.1349 0.3084 0.4465
0.7667 1.9857 -38.4215
9.6600 -34.5000 0.0956 0.0062 -
0.0018 0.0903 0.1517 0.3133 0.4499
0.7594 1.9766 -38.4247
9.6800 -34.0500 0.4020 0.1575
0.0991 0.1033 0.1663 0.3177 0.4530
0.7521 1.9675 -38.4279
9.7000 -34.6200 -0.1494 0.0890
0.1747 0.1098 0.1786 0.3217 0.4559
0.7447 1.9583 -38.4310
9.7200 -34.7500 -0.2747 -0.0301
0.2031 0.1081 0.1883 0.3251 0.4585
0.7371 1.9491 -38.4342
9.7400 -34.4100 0.1189 0.0123
0.1786 0.0976 0.1955 0.3281 0.4608
0.7295 1.9399 -38.4374
9.7600 -34.5400 -0.0454 0.1244
0.1165 0.0791 0.2001 0.3306 0.4629
0.7217 1.9307 -38.4405
9.7800 -34.4400 0.3080 0.0808
0.0370 0.0546 0.2021 0.3327 0.4646
0.7139 1.9214 -38.4437
9.8000 -34.7700 0.1655 -0.1041 -
0.0386 0.0267 0.2015 0.3343 0.4660
0.7060 1.9121 -38.4469
9.8200 -35.3700 -0.3821 -0.2059 -
0.0909 -0.0016 0.1984 0.3355 0.4671
0.6979 1.9028 -38.4500
9.8400 -35.2200 -0.0701 -0.0531 -
0.1083 -0.0269 0.1929 0.3363 0.4680
0.6898 1.8934 -38.4532
9.8600 -34.5400 0.3791 0.1320 -
0.0971 -0.0468 0.1851 0.3367 0.4684
0.6816 1.8841 -38.4564
9.8800 -35.0600 -0.0087 0.1104 -
0.0676 -0.0599 0.1750 0.3369 0.4686
0.6733 1.8746 -38.4595

9.9000	-35.2600	-0.2252	-0.0238	-
0.0306	-0.0649	0.1629	0.3366	0.4684
	0.6648	1.8652	-38.4627	
9.9200	-34.8500	0.2962	-0.1061	
0.0060	-0.0614	0.1490	0.3361	0.4679
	0.6563	1.8558	-38.4659	
9.9400	-35.4600	-0.3116	-0.0541	
0.0285	-0.0499	0.1335	0.3354	0.4671
	0.6478	1.8463	-38.4691	
9.9600	-34.6500	0.3069	0.0798	
0.0282	-0.0316	0.1165	0.3344	0.4659
	0.6391	1.8368	-38.4722	
9.9800	-35.1000	-0.1230	0.1436	
0.0062	-0.0081	0.0984	0.3332	0.4644
	0.6303	1.8272	-38.4754	
10.0000	-34.9400	0.0680	0.0838	-
0.0318	0.0184	0.0794	0.3319	0.4625
	0.6215	1.8177	-38.4786	
10.0200	-35.1100	0.1505	-0.0451	-
0.0706	0.0451	0.0599	0.3304	0.4603
	0.6126	1.8081	-38.4817	
10.0400	-35.7900	-0.2994	-0.1503	-
0.0913	0.0693	0.0402	0.3288	0.4577
	0.6035	1.7985	-38.4849	
10.0600	-35.2100	0.1571	-0.1232	-
0.0787	0.0884	0.0205	0.3272	0.4547
	0.5944	1.7889	-38.4881	
10.0800	-35.3200	-0.0640	-0.0653	-
0.0315	0.1002	0.0014	0.3255	0.4514
	0.5853	1.7792	-38.4913	
10.1000	-35.2900	0.1275	-0.0677	
0.0392	0.1029	-0.0170	0.3238	0.4478
	0.5760	1.7695	-38.4944	
10.1200	-35.4500	-0.2576	-0.0374	
0.1113	0.0952	-0.0344	0.3222	0.4437
	0.5667	1.7598	-38.4976	
10.1400	-34.9900	0.1307	0.0912	
0.1611	0.0771	-0.0505	0.3206	0.4393
	0.5573	1.7501	-38.5008	
10.1600	-35.0200	-0.0301	0.2487	
0.1698	0.0489	-0.0651	0.3192	0.4345
	0.5478	1.7403	-38.5040	
10.1800	-34.8900	0.3151	0.2644	
0.1277	0.0125	-0.0780	0.3179	0.4293
	0.5383	1.7306	-38.5071	
10.2000	-35.9300	-0.4590	0.0895	
0.0398	-0.0294	-0.0892	0.3169	0.4238
	0.5286	1.7208	-38.5103	
10.2200	-35.5400	0.4626	-0.1343	-
0.0616	-0.0737	-0.0984	0.3161	0.4178
	0.5189	1.7109	-38.5135	

10.2400 -36.3300 -0.1469 -0.2972 -
0.1343 -0.1169 -0.1057 0.3156 0.4115
0.5092 1.7011 -38.5167
10.2600 -36.3900 -0.1892 -0.2900 -
0.1498 -0.1555 -0.1108 0.3154 0.4048
0.4994 1.6912 -38.5198
10.2800 -36.0100 0.1162 -0.0533 -
0.1081 -0.1865 -0.1137 0.3156 0.3977
0.4895 1.6814 -38.5230
10.3000 -36.0100 -0.2350 0.2324 -
0.0366 -0.2080 -0.1144 0.3163 0.3902
0.4795 1.6715 -38.5262
10.3200 -35.2000 0.5122 0.2969
0.0272 -0.2188 -0.1130 0.3174 0.3823
0.4695 1.6615 -38.5294
10.3400 -36.1200 -0.2697 0.1083
0.0572 -0.2182 -0.1097 0.3192 0.3740
0.4594 1.6516 -38.5325
10.3600 -36.1900 -0.0182 -0.0782
0.0486 -0.2063 -0.1047 0.3215 0.3653
0.4493 1.6416 -38.5357
10.3800 -36.3600 -0.0807 -0.1434
0.0188 -0.1834 -0.0982 0.3244 0.3562
0.4391 1.6316 -38.5389
10.4000 -35.9600 0.0860 -0.1098 -
0.0101 -0.1512 -0.0906 0.3281 0.3467
0.4289 1.6216 -38.5421
10.4200 -36.1200 0.0019 -0.0363 -
0.0202 -0.1111 -0.0821 0.3325 0.3369
0.4186 1.6116 -38.5453
10.4400 -36.0400 -0.0502 0.0187 -
0.0048 -0.0655 -0.0732 0.3376 0.3266
0.4082 1.6015 -38.5484
10.4600 -35.5300 0.3116 0.0240
0.0264 -0.0167 -0.0643 0.3435 0.3159
0.3979 1.5915 -38.5516
10.4800 -36.3400 -0.4920 0.0276
0.0526 0.0329 -0.0556 0.3502 0.3049
0.3874 1.5814 -38.5548
10.5000 -35.4700 0.2798 0.0853
0.0569 0.0814 -0.0478 0.3578 0.2935
0.3769 1.5713 -38.5580
10.5200 -35.7300 0.0528 0.0976
0.0367 0.1267 -0.0412 0.3661 0.2817
0.3664 1.5612 -38.5612
10.5400 -35.8600 -0.0527 0.0235
0.0020 0.1672 -0.0364 0.3753 0.2696
0.3558 1.5510 -38.5643
10.5600 -36.0000 -0.0874 -0.0367 -
0.0322 0.2016 -0.0338 0.3853 0.2571
0.3452 1.5408 -38.5675

10.5800 -35.8800 0.1616 -0.0898 -
0.0534 0.2289 -0.0336 0.3960 0.2443
0.3346 1.5307 -38.5707
10.6000 -36.1100 -0.0239 -0.1520 -
0.0552 0.2487 -0.0363 0.4075 0.2312
0.3239 1.5205 -38.5739
10.6200 -36.1300 -0.0801 -0.1147 -
0.0422 0.2609 -0.0417 0.4197 0.2178
0.3132 1.5103 -38.5771
10.6400 -36.1600 -0.2856 0.0671 -
0.0250 0.2651 -0.0501 0.4326 0.2040
0.3024 1.5000 -38.5802
10.6600 -35.4900 0.4104 0.2165 -
0.0101 0.2610 -0.0612 0.4460 0.1900
0.2916 1.4898 -38.5834
10.6800 -35.7700 -0.1064 0.1839
0.0015 0.2480 -0.0748 0.4599 0.1758
0.2808 1.4795 -38.5866
10.7000 -36.0500 -0.1433 0.0245
0.0136 0.2252 -0.0906 0.4741 0.1613
0.2700 1.4692 -38.5898
10.7200 -36.4500 -0.3094 -0.1353
0.0371 0.1922 -0.1083 0.4886 0.1466
0.2591 1.4589 -38.5930
10.7400 -35.8800 0.3270 -0.2125
0.0748 0.1488 -0.1273 0.5033 0.1316
0.2482 1.4486 -38.5962
10.7600 -36.2100 0.2306 -0.1850
0.1180 0.0960 -0.1472 0.5181 0.1165
0.2373 1.4383 -38.5993
10.7800 -36.8400 -0.5391 -0.0380
0.1506 0.0356 -0.1674 0.5328 0.1012
0.2264 1.4279 -38.6025
10.8000 -35.8300 0.4771 0.2012
0.1559 -0.0299 -0.1875 0.5474 0.0857
0.2154 1.4176 -38.6057
10.8200 -36.5900 -0.4158 0.3112
0.1213 -0.0970 -0.2067 0.5616 0.0701
0.2044 1.4072 -38.6089
10.8400 -36.0900 0.3633 0.1294
0.0470 -0.1614 -0.2246 0.5755 0.0543
0.1934 1.3968 -38.6121
10.8600 -37.2300 -0.1303 -0.1732 -
0.0500 -0.2186 -0.2405 0.5888 0.0384
0.1824 1.3864 -38.6153
10.8800 -37.6000 -0.1715 -0.2712 -
0.1462 -0.2640 -0.2538 0.6016 0.0224
0.1714 1.3760 -38.6185
10.9000 -37.2500 0.1228 -0.0694 -
0.2180 -0.2932 -0.2639 0.6136 0.0064
0.1604 1.3655 -38.6216

10.9200 -37.1500 0.0814 0.1116 -
0.2451 -0.3034 -0.2704 0.6248 -0.0097
0.1493 1.3551 -38.6248
10.9400 -36.9900 0.2802 -0.0028 -
0.2150 -0.2937 -0.2728 0.6350 -0.0259
0.1383 1.3446 -38.6280
10.9600 -38.0200 -0.6278 -0.1839 -
0.1305 -0.2653 -0.2708 0.6442 -0.0421
0.1272 1.3341 -38.6312
10.9800 -36.5300 0.5385 -0.0698 -
0.0119 -0.2213 -0.2643 0.6522 -0.0583
0.1162 1.3236 -38.6344
11.0000 -37.0500 -0.3056 0.1897
0.1056 -0.1662 -0.2530 0.6589 -0.0745
0.1051 1.3131 -38.6376
11.0200 -36.4400 0.1291 0.2674
0.1884 -0.1050 -0.2371 0.6643 -0.0907
0.0941 1.3026 -38.6408
11.0400 -36.6500 0.0868 0.1185
0.2206 -0.0425 -0.2164 0.6682 -0.1069
0.0830 1.2921 -38.6439
11.0600 -36.8900 -0.0903 -0.1024
0.2039 0.0168 -0.1912 0.6705 -0.1230
0.0720 1.2816 -38.6471
11.0800 -37.1000 -0.1901 -0.1854
0.1482 0.0688 -0.1613 0.6712 -0.1391
0.0609 1.2710 -38.6503
11.1000 -36.7100 0.0455 -0.0692
0.0621 0.1096 -0.1270 0.6701 -0.1551
0.0499 1.2604 -38.6535
11.1200 -36.4300 0.3494 0.0869 -
0.0405 0.1366 -0.0884 0.6672 -0.1710
0.0388 1.2499 -38.6567
11.1400 -37.1400 -0.3371 0.1492 -
0.1360 0.1478 -0.0458 0.6624 -0.1869
0.0278 1.2393 -38.6599
11.1600 -36.6200 0.2568 0.0830 -
0.1966 0.1420 0.0001 0.6557 -0.2026
0.0168 1.2287 -38.6631
11.1800 -37.2400 -0.1375 -0.1130 -
0.1989 0.1189 0.0489 0.6470 -0.2182
0.0058 1.2181 -38.6663
11.2000 -37.3900 -0.0248 -0.2757 -
0.1319 0.0806 0.0996 0.6362 -0.2337
-0.0052 1.2074 -38.6694
11.2200 -37.6000 -0.4597 -0.1744 -
0.0045 0.0307 0.1514 0.6232 -0.2490
-0.0162 1.1968 -38.6726
11.2400 -36.5800 0.1998 0.1224
0.1533 -0.0253 0.2034 0.6081 -0.2642
-0.0271 1.1862 -38.6758

11.2600 -36.1500 0.3519 0.2667
0.2988 -0.0816 0.2545 0.5907 -0.2792
-0.0380 1.1755 -38.6790
11.2800 -36.5900 0.0835 0.0948
0.3918 -0.1329 0.3038 0.5710 -0.2940
-0.0490 1.1649 -38.6822
11.3000 -37.5400 -0.6388 -0.1097
0.4064 -0.1741 0.3505 0.5491 -0.3087
-0.0598 1.1542 -38.6854
11.3200 -36.6700 0.4553 -0.1002
0.3325 -0.2015 0.3934 0.5247 -0.3231
-0.0707 1.1435 -38.6886
11.3400 -36.9100 0.1342 0.0061
0.1805 -0.2126 0.4320 0.4980 -0.3374
-0.0815 1.1328 -38.6918
11.3600 -37.4500 -0.2562 0.0956 -
0.0313 -0.2060 0.4655 0.4688 -0.3515
-0.0923 1.1221 -38.6950
11.3800 -37.6300 -0.2243 0.1927 -
0.2769 -0.1808 0.4934 0.4371 -0.3654
-0.1031 1.1114 -38.6981
11.4000 -37.1200 0.4997 0.1334 -
0.5172 -0.1371 0.5151 0.4031 -0.3790
-0.1139 1.1007 -38.7013
11.4200 -38.4700 -0.3776 -0.1679 -
0.7057 -0.0757 0.5304 0.3666 -0.3924
-0.1246 1.0900 -38.7045
11.4400 -38.2500 0.1356 -0.3973 -
0.7917 0.0014 0.5391 0.3276 -0.4056
-0.1352 1.0792 -38.7077
11.4600 -38.7200 -0.3913 -0.2967 -
0.7364 0.0910 0.5409 0.2864 -0.4186
-0.1459 1.0685 -38.7109
11.4800 -37.4200 0.5132 -0.0380 -
0.5425 0.1878 0.5358 0.2429 -0.4313
-0.1565 1.0578 -38.7141
11.5000 -37.8400 -0.4777 0.1308 -
0.2466 0.2852 0.5238 0.1972 -0.4437
-0.1670 1.0470 -38.7173
11.5200 -36.9000 0.2259 0.1870
0.0974 0.3758 0.5049 0.1495 -0.4559
-0.1775 1.0363 -38.7205
11.5400 -36.5900 0.1280 0.1085
0.4335 0.4522 0.4791 0.0999 -0.4678
-0.1880 1.0255 -38.7237
11.5600 -36.8800 -0.1727 -0.0387
0.7100 0.5066 0.4464 0.0486 -0.4795
-0.1984 1.0147 -38.7269
11.5800 -36.7800 -0.1293 -0.0270
0.8835 0.5323 0.4072 -0.0043 -0.4908
-0.2088 1.0039 -38.7300

11.6000 -36.4100 0.0757 0.2023
0.9253 0.5238 0.3619 -0.0584 -0.5019
-0.2191 0.9932 -38.7332
11.6200 -36.5700 -0.1789 0.4406
0.8199 0.4803 0.3113 -0.1138 -0.5126
-0.2294 0.9824 -38.7364
11.6400 -36.6900 0.4643 0.3666
0.5769 0.4062 0.2562 -0.1702 -0.5231
-0.2396 0.9716 -38.7396
11.6600 -38.5000 -0.3613 -0.0283
0.2403 0.3085 0.1979 -0.2274 -0.5332
-0.2498 0.9608 -38.7428
11.6800 -39.0300 -0.0693 -0.2853 -
0.1242 0.1947 0.1372 -0.2852 -0.5430
-0.2599 0.9500 -38.7460
11.7000 -39.6000 -0.0187 -0.2800 -
0.4443 0.0722 0.0754 -0.3435 -0.5525
-0.2700 0.9392 -38.7492
11.7200 -39.7100 0.3762 -0.2462 -
0.6572 -0.0515 0.0134 -0.4020 -0.5617
-0.2800 0.9283 -38.7524
11.7400 -40.8600 -0.4498 -0.2576 -
0.7312 -0.1689 -0.0476 -0.4607 -0.5705
-0.2899 0.9175 -38.7556
11.7600 -40.5500 -0.0670 -0.2430 -
0.6676 -0.2737 -0.1067 -0.5193 -0.5789
-0.2998 0.9067 -38.7588
11.7800 -39.8600 0.6650 -0.1745 -
0.4966 -0.3608 -0.1627 -0.5776 -0.5870
-0.3096 0.8959 -38.7620
11.8000 -40.4600 -0.1530 -0.0143 -
0.2748 -0.4263 -0.2147 -0.6356 -0.5947
-0.3193 0.8850 -38.7652
11.8200 -40.9600 -1.0318 0.2498 -
0.0651 -0.4667 -0.2615 -0.6929 -0.6020
-0.3290 0.8742 -38.7683
11.8400 -39.1700 0.6164 0.4750
0.0817 -0.4791 -0.3024 -0.7494 -0.6090
-0.3386 0.8634 -38.7715
11.8600 -38.9300 1.0143 0.4102
0.1364 -0.4622 -0.3368 -0.8049 -0.6156
-0.3481 0.8525 -38.7747
11.8800 -41.5200 -1.1900 0.0795
0.1013 -0.4190 -0.3644 -0.8593 -0.6217
-0.3576 0.8417 -38.7779
11.9000 -39.7300 1.0656 -0.1454
0.0157 -0.3558 -0.3853 -0.9124 -0.6275
-0.3670 0.8309 -38.7811
11.9200 -41.9300 -0.8305 -0.2571 -
0.0541 -0.2803 -0.3995 -0.9639 -0.6328
-0.3763 0.8200 -38.7843

11.9400 -40.3300 0.6998 -0.4168 -
0.0518 -0.2003 -0.4074 -1.0139 -0.6377
-0.3855 0.8092 -38.7875
11.9600 -41.5400 -0.5655 -0.3870
0.0341 -0.1219 -0.4091 -1.0620 -0.6422
-0.3947 0.7983 -38.7907
11.9800 -40.5300 0.0471 -0.0577
0.1707 -0.0487 -0.4050 -1.1083 -0.6463
-0.4037 0.7875 -38.7939
12.0000 -39.6000 0.3768 0.3534
0.2969 0.0167 -0.3957 -1.1524 -0.6499
-0.4127 0.7766 -38.7971
12.0200 -40.1900 -0.5030 0.5697
0.3502 0.0715 -0.3816 -1.1945 -0.6531
-0.4216 0.7658 -38.8003
12.0400 -39.3100 0.5300 0.4610
0.2945 0.1137 -0.3634 -1.2342 -0.6558
-0.4304 0.7549 -38.8035
12.0600 -40.7700 -0.3990 0.0724
0.1428 0.1434 -0.3418 -1.2715 -0.6580
-0.4392 0.7441 -38.8067
12.0800 -40.7300 0.2261 -0.2621 -
0.0491 0.1629 -0.3174 -1.3064 -0.6598
-0.4478 0.7332 -38.8099
12.1000 -41.8900 -0.4855 -0.3925 -
0.2123 0.1750 -0.2910 -1.3387 -0.6610
-0.4564 0.7224 -38.8130
12.1200 -40.6000 0.9025 -0.4623 -
0.2887 0.1829 -0.2634 -1.3683 -0.6618
-0.4648 0.7115 -38.8162
12.1400 -41.7500 -0.5142 -0.3420 -
0.2588 0.1888 -0.2353 -1.3952 -0.6621
-0.4732 0.7007 -38.8194
12.1600 -42.0900 -1.2220 0.0248 -
0.1459 0.1934 -0.2076 -1.4192 -0.6619
-0.4815 0.6898 -38.8226
12.1800 -38.9100 1.4141 0.4020
0.0061 0.1966 -0.1810 -1.4404 -0.6611
-0.4897 0.6790 -38.8258
12.2000 -41.4000 -1.1715 0.3275
0.1489 0.1986 -0.1561 -1.4587 -0.6599
-0.4978 0.6681 -38.8290
12.2200 -40.9200 -0.4775 0.0279
0.2419 0.1991 -0.1333 -1.4740 -0.6581
-0.5058 0.6573 -38.8322
12.2400 -39.8500 0.8625 -0.1298
0.2598 0.1982 -0.1131 -1.4864 -0.6558
-0.5137 0.6464 -38.8354
12.2600 -40.7600 -0.0998 -0.0347
0.1970 0.1962 -0.0955 -1.4958 -0.6529
-0.5215 0.6356 -38.8386

12.2800 -40.9800 -0.3262 0.0792
0.0762 0.1929 -0.0807 -1.5023 -0.6495
-0.5292 0.6247 -38.8418
12.3000 -40.4400 0.3963 0.0405 -
0.0684 0.1885 -0.0686 -1.5059 -0.6455
-0.5368 0.6139 -38.8450
12.3200 -40.9500 0.2068 -0.1150 -
0.2045 0.1831 -0.0591 -1.5066 -0.6411
-0.5443 0.6031 -38.8482
12.3400 -41.7200 -0.4672 -0.1962 -
0.3038 0.1770 -0.0519 -1.5044 -0.6361
-0.5517 0.5922 -38.8514
12.3600 -41.1900 0.0370 -0.0523 -
0.3439 0.1698 -0.0469 -1.4995 -0.6305
-0.5590 0.5814 -38.8546
12.3800 -40.7200 0.3384 0.1221 -
0.3193 0.1605 -0.0435 -1.4920 -0.6245
-0.5662 0.5706 -38.8578
12.4000 -41.2000 -0.2006 0.1010 -
0.2347 0.1475 -0.0414 -1.4819 -0.6179
-0.5733 0.5597 -38.8610
12.4200 -41.0200 -0.0115 -0.0247 -
0.0978 0.1287 -0.0401 -1.4692 -0.6108
-0.5803 0.5489 -38.8641
12.4400 -41.4200 -0.5462 -0.0824
0.0737 0.1027 -0.0390 -1.4542 -0.6033
-0.5872 0.5381 -38.8673
12.4600 -39.9900 0.9545 -0.2533
0.2545 0.0686 -0.0378 -1.4368 -0.5952
-0.5940 0.5273 -38.8705
12.4800 -41.9200 -1.0358 -0.3497
0.4083 0.0260 -0.0361 -1.4173 -0.5867
-0.6007 0.5165 -38.8737
12.5000 -40.6200 -0.1933 0.0511
0.4959 -0.0247 -0.0337 -1.3957 -0.5777
-0.6073 0.5057 -38.8769
12.5200 -39.0900 0.9773 0.5467
0.4868 -0.0821 -0.0301 -1.3721 -0.5682
-0.6138 0.4949 -38.8801
12.5400 -40.9800 -0.7440 0.4952
0.3698 -0.1433 -0.0253 -1.3465 -0.5583
-0.6202 0.4841 -38.8833
12.5600 -40.5400 0.5084 -0.1319
0.1719 -0.2051 -0.0190 -1.3192 -0.5480
-0.6265 0.4733 -38.8865
12.5800 -41.4500 0.2571 -0.5911 -
0.0556 -0.2635 -0.0111 -1.2902 -0.5373
-0.6327 0.4626 -38.8897
12.6000 -42.7100 -0.9263 -0.4414 -
0.2636 -0.3144 -0.0012 -1.2597 -0.5261
-0.6388 0.4518 -38.8929

12.6200 -40.5900 0.8961 0.1817 -
0.4260 -0.3530 0.0105 -1.2277 -0.5145
-0.6448 0.4410 -38.8961
12.6400 -41.8100 -0.5664 0.4802 -
0.5353 -0.3746 0.0245 -1.1943 -0.5026
-0.6507 0.4303 -38.8993
12.6600 -40.9300 0.4976 0.2380 -
0.5941 -0.3749 0.0405 -1.1596 -0.4902
-0.6565 0.4195 -38.9025
12.6800 -42.2600 -0.3712 -0.1758 -
0.6048 -0.3516 0.0586 -1.1237 -0.4775
-0.6622 0.4088 -38.9057
12.7000 -41.8200 0.0469 -0.4405 -
0.5640 -0.3044 0.0784 -1.0867 -0.4645
-0.6678 0.3980 -38.9089
12.7200 -41.9500 -0.2877 -0.3328 -
0.4721 -0.2354 0.0997 -1.0486 -0.4511
-0.6733 0.3873 -38.9120
12.7400 -41.0400 -0.0746 0.0544 -
0.3409 -0.1485 0.1220 -1.0095 -0.4374
-0.6787 0.3766 -38.9152
12.7600 -39.8600 0.5562 0.3575 -
0.1901 -0.0488 0.1448 -0.9694 -0.4234
-0.6840 0.3658 -38.9184
12.7800 -40.1700 -0.1461 0.3441 -
0.0408 0.0580 0.1678 -0.9285 -0.4091
-0.6892 0.3551 -38.9216
12.8000 -40.3600 -0.5420 0.1660
0.0955 0.1656 0.1904 -0.8869 -0.3945
-0.6943 0.3444 -38.9248
12.8200 -39.8800 -0.0030 0.0393
0.2168 0.2673 0.2121 -0.8444 -0.3796
-0.6993 0.3338 -38.9280
12.8400 -38.9800 0.7685 -0.0996
0.3307 0.3560 0.2326 -0.8013 -0.3644
-0.7042 0.3231 -38.9312
12.8600 -39.8100 -0.2060 -0.3186
0.4470 0.4257 0.2512 -0.7576 -0.3490
-0.7090 0.3124 -38.9344
12.8800 -40.1100 -0.5001 -0.3910
0.5640 0.4715 0.2676 -0.7134 -0.3334
-0.7136 0.3017 -38.9376
12.9000 -39.4200 -0.4153 -0.0672
0.6653 0.4902 0.2813 -0.6686 -0.3175
-0.7182 0.2911 -38.9408
12.9200 -37.7700 0.6613 0.3603
0.7237 0.4800 0.2920 -0.6235 -0.3015
-0.7227 0.2804 -38.9440
12.9400 -38.2600 0.0543 0.4292
0.7140 0.4408 0.2996 -0.5780 -0.2852
-0.7271 0.2698 -38.9472

12.9600 -39.1200 -0.3823 0.1332
0.6329 0.3753 0.3044 -0.5321 -0.2687
-0.7314 0.2592 -38.9504
12.9800 -38.9600 0.3247 -0.1714
0.4950 0.2878 0.3064 -0.4861 -0.2521
-0.7356 0.2486 -38.9536
13.0000 -39.8700 -0.3634 -0.2133
0.3228 0.1840 0.3061 -0.4398 -0.2353
-0.7397 0.2380 -38.9567
13.0200 -39.5600 0.1664 -0.1119
0.1416 0.0697 0.3037 -0.3934 -0.2183
-0.7437 0.2274 -38.9599
13.0400 -39.4900 0.3047 -0.0313 -
0.0328 -0.0488 0.2995 -0.3469 -0.2012
-0.7476 0.2168 -38.9631
13.0600 -40.5200 -0.4525 0.0378 -
0.1957 -0.1651 0.2938 -0.3003 -0.1840
-0.7514 0.2062 -38.9663
13.0800 -39.7700 0.2074 0.1524 -
0.3479 -0.2723 0.2869 -0.2537 -0.1666
-0.7551 0.1957 -38.9695
13.1000 -40.5800 -0.1660 0.1352 -
0.4807 -0.3641 0.2791 -0.2072 -0.1492
-0.7587 0.1851 -38.9727
13.1200 -40.1500 0.5710 -0.0928 -
0.5780 -0.4356 0.2707 -0.1607 -0.1316
-0.7622 0.1746 -38.9759
13.1400 -41.5200 -0.7475 -0.2093 -
0.6185 -0.4831 0.2620 -0.1143 -0.1140
-0.7656 0.1640 -38.9791
13.1600 -40.0800 0.6087 -0.0375 -
0.5865 -0.5038 0.2533 -0.0680 -0.0963
-0.7689 0.1535 -38.9823
13.1800 -40.5300 -0.0972 0.0821 -
0.4900 -0.4976 0.2449 -0.0219 -0.0786
-0.7721 0.1430 -38.9855
13.2000 -40.1000 0.3389 -0.0472 -
0.3497 -0.4659 0.2366 0.0242 -0.0608
-0.7752 0.1326 -38.9887
13.2200 -40.5800 -0.5147 -0.1608 -
0.1894 -0.4114 0.2283 0.0701 -0.0430
-0.7782 0.1221 -38.9919
13.2400 -39.4600 0.2835 -0.0114 -
0.0324 -0.3374 0.2199 0.1160 -0.0252
-0.7811 0.1116 -38.9950
13.2600 -39.3000 -0.1279 0.2041
0.0978 -0.2476 0.2111 0.1618 -0.0074
-0.7840 0.1012 -38.9982
13.2800 -38.9700 0.1629 0.1890
0.1823 -0.1463 0.2017 0.2075 0.0104
-0.7867 0.0907 -39.0014

13.3000 -39.0200 0.0854 -0.0158
0.2147 -0.0383 0.1917 0.2532 0.0282
-0.7894 0.0803 -39.0046
13.3200 -39.3400 -0.2351 -0.1698
0.2058 0.0708 0.1807 0.2989 0.0460
-0.7919 0.0699 -39.0078
13.3400 -39.0800 -0.0722 -0.1119
0.1771 0.1747 0.1688 0.3446 0.0637
-0.7944 0.0595 -39.0110
13.3600 -38.5500 0.1187 0.0599
0.1505 0.2671 0.1557 0.3903 0.0813
-0.7967 0.0492 -39.0142
13.3800 -38.3600 0.1468 0.1364
0.1450 0.3428 0.1414 0.4361 0.0988
-0.7990 0.0388 -39.0174
13.4000 -38.7500 -0.3176 0.0999
0.1705 0.3981 0.1256 0.4819 0.1163
-0.8012 0.0284 -39.0206
13.4200 -38.1400 0.4561 -0.0274
0.2230 0.4311 0.1083 0.5279 0.1336
-0.8033 0.0181 -39.0238
13.4400 -38.5700 -0.1272 -0.2304
0.2893 0.4412 0.0890 0.5739 0.1508
-0.8053 0.0078 -39.0269
13.4600 -38.8000 -0.3422 -0.2974
0.3480 0.4292 0.0677 0.6200 0.1679
-0.8072 -0.0025 -39.0301
13.4800 -38.3300 -0.0139 -0.0285
0.3744 0.3974 0.0440 0.6662 0.1848
-0.8090 -0.0128 -39.0333
13.5000 -37.7400 0.2098 0.3603
0.3445 0.3492 0.0179 0.7125 0.2016
-0.8108 -0.0231 -39.0365
13.5200 -38.0800 -0.1024 0.4760
0.2443 0.2898 -0.0109 0.7590 0.2182
-0.8124 -0.0333 -39.0397
13.5400 -38.2900 0.1672 0.2533
0.0785 0.2254 -0.0423 0.8055 0.2346
-0.8140 -0.0436 -39.0429
13.5600 -38.9900 -0.0743 -0.0703 -
0.1229 0.1630 -0.0766 0.8522 0.2508
-0.8154 -0.0538 -39.0461
13.5800 -39.3600 -0.0108 -0.3392 -
0.3153 0.1090 -0.1139 0.8989 0.2668
-0.8168 -0.0640 -39.0493
13.6000 -39.6300 0.1025 -0.4831 -
0.4506 0.0701 -0.1541 0.9456 0.2825
-0.8181 -0.0742 -39.0525
13.6200 -40.0400 -0.2700 -0.4077 -
0.4966 0.0516 -0.1973 0.9922 0.2980
-0.8193 -0.0843 -39.0556

13.6400 -39.2000 0.1253 -0.1417 -
0.4521 0.0556 -0.2430 1.0387 0.3133
-0.8204 -0.0945 -39.0588
13.6600 -39.0900 0.0061 0.0842 -
0.3453 0.0806 -0.2907 1.0850 0.3282
-0.8215 -0.1046 -39.0620
13.6800 -38.6700 0.0320 0.2186 -
0.2206 0.1235 -0.3394 1.1310 0.3430
-0.8224 -0.1148 -39.0652
13.7000 -38.9300 -0.3941 0.3488 -
0.1227 0.1804 -0.3880 1.1766 0.3574
-0.8233 -0.1249 -39.0684
13.7200 -37.6600 0.6587 0.3340 -
0.0733 0.2455 -0.4357 1.2217 0.3715
-0.8241 -0.1350 -39.0716
13.7400 -38.4500 0.1076 0.0321 -
0.0698 0.3111 -0.4814 1.2662 0.3852
-0.8248 -0.1450 -39.0748
13.7600 -39.4100 -0.6399 -0.2859 -
0.0824 0.3672 -0.5241 1.3100 0.3987
-0.8254 -0.1551 -39.0780
13.7800 -38.8100 -0.0023 -0.2843 -
0.0733 0.4035 -0.5628 1.3529 0.4118
-0.8259 -0.1651 -39.0811
13.8000 -37.8500 0.7338 -0.0915 -
0.0156 0.4108 -0.5966 1.3950 0.4246
-0.8263 -0.1751 -39.0843
13.8200 -39.0700 -0.6661 0.0435
0.0988 0.3835 -0.6247 1.4359 0.4370
-0.8267 -0.1851 -39.0875
13.8400 -37.9600 0.2863 0.0815
0.2567 0.3199 -0.6465 1.4757 0.4490
-0.8270 -0.1951 -39.0907
13.8600 -38.0000 0.1535 0.0424
0.4259 0.2216 -0.6616 1.5143 0.4607
-0.8272 -0.2051 -39.0939
13.8800 -38.4400 -0.2519 -0.0544
0.5585 0.0927 -0.6696 1.5514 0.4719
-0.8273 -0.2150 -39.0971
13.9000 -38.2200 0.1709 -0.0241
0.6016 -0.0613 -0.6704 1.5871 0.4828
-0.8273 -0.2249 -39.1003
13.9200 -38.6100 -0.3621 0.1799
0.5199 -0.2325 -0.6637 1.6213 0.4932
-0.8273 -0.2348 -39.1034
13.9400 -38.0100 0.6421 0.1802
0.3097 -0.4102 -0.6491 1.6538 0.5032
-0.8272 -0.2447 -39.1066
13.9600 -39.9800 -0.7639 -0.0191
0.0088 -0.5824 -0.6264 1.6845 0.5128
-0.8270 -0.2546 -39.1098

13.9800 -39.0400 0.5809 0.0181 -
0.3124 -0.7371 -0.5954 1.7133 0.5219
-0.8267 -0.2644 -39.1130
14.0000 -39.7300 -0.0651 0.0764 -
0.5640 -0.8623 -0.5559 1.7401 0.5306
-0.8263 -0.2743 -39.1162
14.0200 -40.2100 -0.0644 -0.1651 -
0.6699 -0.9463 -0.5075 1.7648 0.5388
-0.8259 -0.2841 -39.1194
14.0400 -39.9000 0.4740 -0.5075 -
0.5947 -0.9821 -0.4503 1.7873 0.5465
-0.8254 -0.2939 -39.1225
14.0600 -40.9500 -0.8994 -0.3529 -
0.3546 -0.9726 -0.3847 1.8074 0.5537
-0.8248 -0.3036 -39.1257
14.0800 -38.0500 0.8428 0.3006 -
0.0342 -0.9252 -0.3113 1.8250 0.5604
-0.8241 -0.3134 -39.1289
14.1000 -38.3400 -0.1072 0.5670
0.2562 -0.8474 -0.2311 1.8401 0.5667
-0.8234 -0.3231 -39.1321
14.1200 -38.0100 0.0481 0.2589
0.4292 -0.7469 -0.1450 1.8524 0.5723
-0.8226 -0.3328 -39.1353
14.1400 -38.6800 -0.4835 -0.0752
0.4627 -0.6312 -0.0540 1.8619 0.5775
-0.8217 -0.3425 -39.1385
14.1600 -38.0300 0.0895 -0.1165
0.3805 -0.5078 0.0408 1.8684 0.5822
-0.8208 -0.3522 -39.1416
14.1800 -37.5900 0.1268 -0.0076
0.2364 -0.3836 0.1384 1.8718 0.5863
-0.8197 -0.3618 -39.1448
14.2000 -37.4600 0.3943 -0.0773
0.0934 -0.2646 0.2377 1.8719 0.5899
-0.8186 -0.3714 -39.1480
14.2200 -38.4300 -0.4577 -0.2412 -
0.0111 -0.1546 0.3375 1.8687 0.5929
-0.8175 -0.3810 -39.1512
14.2400 -37.7600 -0.0234 -0.1270 -
0.0704 -0.0553 0.4366 1.8619 0.5954
-0.8162 -0.3906 -39.1544
14.2600 -37.1900 0.2483 0.1405 -
0.0986 0.0342 0.5334 1.8516 0.5974
-0.8149 -0.4002 -39.1576
14.2800 -37.1400 -0.0118 0.2179 -
0.1164 0.1169 0.6265 1.8374 0.5989
-0.8136 -0.4097 -39.1607
14.3000 -37.0500 0.0811 0.0321 -
0.1420 0.1971 0.7142 1.8193 0.5998
-0.8121 -0.4193 -39.1639

14.3200 -37.5400 -0.2590 -0.1574 -
0.1807 0.2789 0.7950 1.7972 0.6003
-0.8106 -0.4288 -39.1671
14.3400 -36.8100 0.2440 -0.1315 -
0.2302 0.3660 0.8674 1.7710 0.6002
-0.8090 -0.4382 -39.1703
14.3600 -37.5000 -0.5816 0.0890 -
0.2889 0.4602 0.9298 1.7404 0.5997
-0.8074 -0.4477 -39.1735
14.3800 -36.0700 0.7075 0.2419 -
0.3518 0.5608 0.9810 1.7055 0.5987
-0.8057 -0.4571 -39.1766
14.4000 -37.1700 -0.2560 0.0693 -
0.4048 0.6648 1.0200 1.6661 0.5972
-0.8040 -0.4665 -39.1798
14.4200 -37.2900 -0.2066 -0.2363 -
0.4250 0.7669 1.0457 1.6223 0.5952
-0.8021 -0.4759 -39.1830
14.4400 -37.0400 -0.0293 -0.3320 -
0.3876 0.8612 1.0574 1.5741 0.5928
-0.8003 -0.4853 -39.1862
14.4600 -36.8600 0.0046 -0.1147 -
0.2800 0.9406 1.0545 1.5215 0.5899
-0.7983 -0.4947 -39.1893
14.4800 -36.2700 0.0898 0.1867 -
0.1063 0.9978 1.0366 1.4647 0.5866
-0.7963 -0.5040 -39.1925
14.5000 -36.1100 -0.0626 0.2906
0.1218 1.0263 1.0036 1.4039 0.5829
-0.7943 -0.5133 -39.1957
14.5200 -36.1300 0.0011 0.0904
0.3793 1.0201 0.9557 1.3394 0.5788
-0.7921 -0.5226 -39.1989
14.5400 -36.1400 0.1455 -0.2288
0.6192 0.9741 0.8938 1.2714 0.5743
-0.7900 -0.5319 -39.2021
14.5600 -36.9700 -0.5221 -0.3003
0.7895 0.8847 0.8190 1.2001 0.5694
-0.7877 -0.5411 -39.2052
14.5800 -35.8200 0.6386 0.0001
0.8480 0.7520 0.7331 1.1259 0.5641
-0.7854 -0.5503 -39.2084
14.6000 -36.7400 -0.1105 0.4050
0.7720 0.5809 0.6377 1.0489 0.5585
-0.7831 -0.5595 -39.2116
14.6200 -37.4800 -0.5373 0.7039
0.5579 0.3809 0.5348 0.9695 0.5524
-0.7807 -0.5687 -39.2148
14.6400 -37.5200 0.0372 0.7489
0.2274 0.1635 0.4261 0.8878 0.5461
-0.7782 -0.5778 -39.2179

14.6600	-37.9700	0.8354	0.3485	-
0.1610	-0.0588	0.3134	0.8042	0.5394
	-0.7757	-0.5870	-39.2211	
14.6800	-40.6500	-0.2949	-0.4168	-
0.5277	-0.2729	0.1986	0.7188	0.5324
	-0.7732	-0.5961	-39.2243	
14.7000	-41.9700	-0.4316	-0.9892	-
0.7888	-0.4662	0.0835	0.6320	0.5250
	-0.7705	-0.6052	-39.2275	
14.7200	-42.4800	-0.6077	-0.8154	-
0.8743	-0.6261	-0.0301	0.5440	0.5174
	-0.7679	-0.6142	-39.2306	
14.7400	-40.8200	0.6014	-0.0302	-
0.7804	-0.7433	-0.1403	0.4551	0.5094
	-0.7652	-0.6233	-39.2338	
14.7600	-41.0400	-0.3546	0.7035	-
0.5581	-0.8150	-0.2455	0.3655	0.5012
	-0.7624	-0.6323	-39.2370	
14.7800	-39.9100	0.6546	0.7403	-
0.2695	-0.8427	-0.3439	0.2754	0.4927
	-0.7596	-0.6413	-39.2402	
14.8000	-40.6900	0.4065	0.1517	
0.0277	-0.8300	-0.4342	0.1852	0.4839
	-0.7567	-0.6503	-39.2433	
14.8200	-42.4300	-0.8601	-0.4310	
0.2787	-0.7818	-0.5154	0.0950	0.4749
	-0.7538	-0.6592	-39.2465	
14.8400	-40.7900	0.5141	-0.3588	
0.4439	-0.7044	-0.5866	0.0052	0.4657
	-0.7508	-0.6682	-39.2497	
14.8600	-41.1600	-0.2522	0.0870	
0.5166	-0.6053	-0.6474	-0.0841	0.4562
	-0.7478	-0.6771	-39.2528	
14.8800	-40.1500	0.6215	0.2279	
0.5097	-0.4925	-0.6972	-0.1725	0.4464
	-0.7448	-0.6859	-39.2560	
14.9000	-41.4600	-0.2099	0.0176	
0.4321	-0.3747	-0.7356	-0.2598	0.4365
	-0.7417	-0.6948	-39.2592	
14.9200	-41.7700	-0.2597	-0.0929	
0.2900	-0.2589	-0.7626	-0.3458	0.4264
	-0.7385	-0.7036	-39.2624	
14.9400	-41.6700	-0.2172	0.1269	
0.1003	-0.1513	-0.7780	-0.4302	0.4161
	-0.7353	-0.7125	-39.2655	
14.9600	-40.8800	0.6659	0.2725	-
0.0977	-0.0575	-0.7821	-0.5128	0.4056
	-0.7321	-0.7212	-39.2687	
14.9800	-42.6200	-0.7478	0.0280	-
0.2583	0.0180	-0.7749	-0.5934	0.3949
	-0.7288	-0.7300	-39.2719	

15.0000 -42.3800 -0.0846 -0.2970 -
0.3398 0.0724 -0.7570 -0.6719 0.3840
-0.7255 -0.7388 -39.2750
15.0200 -41.5100 0.9311 -0.2977 -
0.3155 0.1047 -0.7287 -0.7480 0.3731
-0.7222 -0.7475 -39.2782
15.0400 -43.1700 -1.3188 0.1229 -
0.1985 0.1163 -0.6910 -0.8217 0.3619
-0.7188 -0.7562 -39.2814
15.0600 -39.9600 1.5896 0.3045 -
0.0357 0.1110 -0.6450 -0.8926 0.3507
-0.7153 -0.7649 -39.2845
15.0800 -43.5100 -1.5896 0.0222
0.1247 0.0939 -0.5923 -0.9609 0.3393
-0.7119 -0.7735 -39.2877
15.1000 -42.7400 -0.7844 -0.1439
0.2496 0.0696 -0.5344 -1.0262 0.3279
-0.7083 -0.7821 -39.2909
15.1200 -40.0500 1.5989 -0.0915
0.3172 0.0436 -0.4732 -1.0886 0.3163
-0.7048 -0.7907 -39.2940
15.1400 -42.3300 -0.6178 -0.0199
0.3136 0.0209 -0.4101 -1.1478 0.3046
-0.7012 -0.7993 -39.2972
15.1600 -42.9900 -1.1106 -0.0048
0.2357 0.0054 -0.3468 -1.2038 0.2929
-0.6976 -0.8079 -39.3004
15.1800 -42.0700 0.0521 0.0162
0.0848 0.0005 -0.2849 -1.2565 0.2811
-0.6939 -0.8164 -39.3035
15.2000 -40.5600 1.4126 0.1949 -
0.1270 0.0078 -0.2258 -1.3057 0.2693
-0.6902 -0.8249 -39.3067
15.2200 -43.5500 -1.6811 0.4607 -
0.3509 0.0284 -0.1705 -1.3514 0.2574
-0.6865 -0.8334 -39.3099
15.2400 -40.6200 1.8171 0.1287 -
0.5039 0.0628 -0.1198 -1.3935 0.2454
-0.6827 -0.8419 -39.3130
15.2600 -44.9000 -1.8021 -0.6572 -
0.5169 0.1102 -0.0741 -1.4320 0.2335
-0.6789 -0.8503 -39.3162
15.2800 -41.9800 1.1390 -0.7499 -
0.3590 0.1670 -0.0337 -1.4669 0.2215
-0.6751 -0.8588 -39.3194
15.3000 -41.3000 0.9388 -0.2671 -
0.0610 0.2265 0.0013 -1.4980 0.2096
-0.6712 -0.8671 -39.3225
15.3200 -42.0900 -0.8323 0.2977
0.2806 0.2806 0.0308 -1.5254 0.1976
-0.6674 -0.8755 -39.3257

15.3400	-40.7600	-0.0074	0.6184	
0.5459	0.3208	0.0546	-1.5491	0.1857
	-0.6634	-0.8839	-39.3288	
15.3600	-40.6900	-0.2914	0.8026	
0.6374	0.3394	0.0730	-1.5691	0.1738
	-0.6595	-0.8922	-39.3320	
15.3800	-39.9500	0.9791	0.3324	
0.5408	0.3327	0.0860	-1.5856	0.1619
	-0.6555	-0.9005	-39.3352	
15.4000	-43.3100	-0.9884	-0.6176	
0.3154	0.3023	0.0940	-1.5985	0.1501
	-0.6515	-0.9088	-39.3383	
15.4200	-42.4000	0.3933	-0.7776	
0.0385	0.2524	0.0976	-1.6080	0.1383
	-0.6475	-0.9170	-39.3415	
15.4400	-42.8800	-0.2461	-0.1439	-
0.2227	0.1878	0.0974	-1.6143	0.1266
	-0.6434	-0.9253	-39.3446	
15.4600	-41.6000	0.5468	0.3976	-
0.4178	0.1138	0.0939	-1.6173	0.1150
	-0.6393	-0.9335	-39.3478	
15.4800	-41.9700	0.3860	0.5611	-
0.5218	0.0360	0.0877	-1.6174	0.1034
	-0.6352	-0.9416	-39.3510	
15.5000	-43.5600	-0.9980	0.4342	-
0.5311	-0.0402	0.0795	-1.6145	0.0919
	-0.6311	-0.9498	-39.3541	
15.5200	-41.8600	0.9800	0.0873	-
0.4530	-0.1112	0.0700	-1.6089	0.0806
	-0.6269	-0.9579	-39.3573	
15.5400	-43.2400	0.0853	-0.4290	-
0.2962	-0.1754	0.0598	-1.6007	0.0693
	-0.6227	-0.9660	-39.3604	
15.5600	-44.5400	-1.0666	-0.7552	-
0.0790	-0.2311	0.0495	-1.5900	0.0581
	-0.6185	-0.9741	-39.3636	
15.5800	-42.3300	0.8306	-0.5711	
0.1655	-0.2763	0.0399	-1.5769	0.0471
	-0.6143	-0.9822	-39.3667	
15.6000	-42.1800	0.2015	-0.0851	
0.3950	-0.3088	0.0315	-1.5616	0.0362
	-0.6100	-0.9902	-39.3699	
15.6200	-42.1100	-0.2535	0.4203	
0.5531	-0.3265	0.0248	-1.5443	0.0254
	-0.6058	-0.9983	-39.3731	
15.6400	-42.2000	-0.7345	0.9097	
0.5732	-0.3278	0.0200	-1.5251	0.0147
	-0.6015	-1.0062	-39.3762	
15.6600	-40.4100	0.9913	0.8904	
0.4160	-0.3117	0.0171	-1.5040	0.0042
	-0.5972	-1.0142	-39.3794	

15.6800 -43.5300 -0.9248 -0.0001
0.1163 -0.2780 0.0161 -1.4812 -0.0062
-0.5929 -1.0222 -39.3825
15.7000 -43.0500 0.6097 -0.7559 -
0.2197 -0.2273 0.0168 -1.4568 -0.0164
-0.5885 -1.0301 -39.3857
15.7200 -44.6200 -0.8241 -0.7722 -
0.4740 -0.1603 0.0189 -1.4309 -0.0265
-0.5842 -1.0380 -39.3888
15.7400 -42.8000 0.9862 -0.7365 -
0.5552 -0.0786 0.0222 -1.4035 -0.0365
-0.5798 -1.0458 -39.3920
15.7600 -43.9700 -0.4325 -0.7390 -
0.4447 0.0138 0.0263 -1.3747 -0.0463
-0.5754 -1.0537 -39.3951
15.7800 -43.8800 -1.0912 -0.2922 -
0.2205 0.1108 0.0307 -1.3446 -0.0559
-0.5710 -1.0615 -39.3983
15.8000 -40.1900 1.2494 0.6216
0.0238 0.2064 0.0351 -1.3133 -0.0654
-0.5665 -1.0693 -39.4014
15.8200 -41.7000 -0.8943 1.1528
0.2163 0.2946 0.0389 -1.2807 -0.0747
-0.5621 -1.0771 -39.4046
15.8400 -41.1600 -0.2241 0.7825
0.3112 0.3695 0.0417 -1.2470 -0.0838
-0.5577 -1.0849 -39.4077
15.8600 -40.8100 0.9576 -0.1372
0.3204 0.4255 0.0432 -1.2122 -0.0928
-0.5532 -1.0926 -39.4109
15.8800 -43.1800 -0.7193 -0.8778
0.2822 0.4578 0.0430 -1.1764 -0.1016
-0.5487 -1.1003 -39.4140
15.9000 -42.9500 -0.5051 -0.8840
0.2328 0.4634 0.0408 -1.1395 -0.1102
-0.5442 -1.1080 -39.4172
15.9200 -41.5500 0.2659 -0.2229
0.1878 0.4411 0.0367 -1.1017 -0.1187
-0.5397 -1.1156 -39.4203
15.9400 -40.5400 0.6644 0.4129
0.1527 0.3920 0.0308 -1.0630 -0.1270
-0.5352 -1.1233 -39.4235
15.9600 -41.6000 -0.2533 0.5358
0.1260 0.3195 0.0233 -1.0235 -0.1351
-0.5307 -1.1309 -39.4266
15.9800 -41.9700 -0.5057 0.3863
0.0901 0.2294 0.0147 -0.9833 -0.1431
-0.5262 -1.1385 -39.4298
16.0000 -42.2700 -0.4868 0.3140
0.0248 0.1283 0.0052 -0.9423 -0.1509
-0.5217 -1.1460 -39.4329

16.0200 -41.4100 0.5309 0.1946 -
0.0637 0.0231 -0.0047 -0.9008 -0.1585
-0.5171 -1.1536 -39.4361
16.0400 -41.3000 1.1919 -0.1419 -
0.1550 -0.0796 -0.0146 -0.8586 -0.1660
-0.5126 -1.1611 -39.4392
16.0600 -42.9900 0.2043 -0.5650 -
0.2332 -0.1735 -0.0240 -0.8159 -0.1732
-0.5081 -1.1686 -39.4424
16.0800 -44.9600 -1.5783 -0.6081 -
0.2833 -0.2530 -0.0327 -0.7727 -0.1803
-0.5035 -1.1760 -39.4455
16.1000 -43.2500 -0.7139 0.0240 -
0.2901 -0.3135 -0.0401 -0.7290 -0.1873
-0.4990 -1.1835 -39.4486
16.1200 -40.3200 1.6523 0.6960 -
0.2575 -0.3527 -0.0459 -0.6850 -0.1940
-0.4944 -1.1909 -39.4518
16.1400 -43.5900 -1.6076 0.5851 -
0.1943 -0.3700 -0.0497 -0.6407 -0.2006
-0.4898 -1.1983 -39.4549
16.1600 -41.8500 0.8195 -0.2511 -
0.1084 -0.3670 -0.0511 -0.5962 -0.2069
-0.4853 -1.2056 -39.4581
16.1800 -41.7100 1.2688 -0.7493
0.0003 -0.3463 -0.0500 -0.5514 -0.2132
-0.4807 -1.2130 -39.4612
16.2000 -44.2300 -1.6153 -0.3519
0.1254 -0.3114 -0.0461 -0.5064 -0.2192
-0.4762 -1.2203 -39.4644
16.2200 -39.8900 1.5790 0.4322
0.2363 -0.2665 -0.0396 -0.4613 -0.2250
-0.4716 -1.2276 -39.4675
16.2400 -42.3900 -1.3009 0.5849
0.2987 -0.2154 -0.0303 -0.4161 -0.2307
-0.4670 -1.2349 -39.4706
16.2600 -41.9900 -0.5776 0.2669
0.2943 -0.1613 -0.0185 -0.3709 -0.2361
-0.4625 -1.2421 -39.4738
16.2800 -40.7700 0.9541 -0.1075
0.2299 -0.1063 -0.0045 -0.3256 -0.2414
-0.4579 -1.2494 -39.4769
16.3000 -42.0700 -0.0914 -0.3382
0.1274 -0.0520 0.0113 -0.2805 -0.2465
-0.4534 -1.2566 -39.4801
16.3200 -42.3000 -0.1438 -0.3117
0.0101 0.0006 0.0285 -0.2354 -0.2514
-0.4488 -1.2637 -39.4832
16.3400 -42.5200 -0.9837 0.1837 -
0.1042 0.0510 0.0466 -0.1905 -0.2562
-0.4443 -1.2709 -39.4863

16.3600 -39.7600 1.3476 0.5244 -
0.2061 0.0990 0.0652 -0.1459 -0.2607
-0.4397 -1.2780 -39.4895
16.3800 -42.7700 -1.3738 0.2369 -
0.2827 0.1439 0.0837 -0.1015 -0.2650
-0.4352 -1.2851 -39.4926
16.4000 -41.6500 0.1224 -0.0879 -
0.3168 0.1833 0.1017 -0.0575 -0.2691
-0.4307 -1.2922 -39.4957
16.4200 -40.7300 1.0522 -0.3455 -
0.2616 0.2141 0.1190 -0.0140 -0.2731
-0.4261 -1.2993 -39.4989
16.4400 -42.6000 -0.7579 -0.6167 -
0.0858 0.2339 0.1349 0.0290 -0.2768
-0.4216 -1.3063 -39.5020
16.4600 -41.2300 0.2755 -0.5778
0.1824 0.2407 0.1493 0.0715 -0.2803
-0.4171 -1.3133 -39.5051
16.4800 -41.1900 -0.8613 0.2674
0.4605 0.2329 0.1617 0.1133 -0.2837
-0.4126 -1.3203 -39.5083
16.5000 -38.3000 1.1697 0.9809
0.6311 0.2098 0.1719 0.1545 -0.2868
-0.4081 -1.3272 -39.5114
16.5200 -40.3700 -0.6560 0.7453
0.6004 0.1713 0.1796 0.1948 -0.2897
-0.4036 -1.3342 -39.5145
16.5400 -41.6900 -1.0691 0.1116
0.3921 0.1209 0.1848 0.2344 -0.2924
-0.3992 -1.3411 -39.5177
16.5600 -41.5000 -0.3129 -0.3389
0.0823 0.0641 0.1874 0.2731 -0.2950
-0.3947 -1.3480 -39.5208
16.5800 -40.6000 1.1843 -0.5907 -
0.2403 0.0073 0.1877 0.3108 -0.2972
-0.3903 -1.3548 -39.5239
16.6000 -42.7800 -0.6017 -0.6589 -
0.4969 -0.0432 0.1856 0.3475 -0.2993
-0.3858 -1.3617 -39.5270
16.6200 -42.7900 -0.5794 -0.3853 -
0.6434 -0.0810 0.1812 0.3832 -0.3012
-0.3814 -1.3685 -39.5302
16.6400 -40.8800 0.6208 0.1240 -
0.6660 -0.1009 0.1748 0.4179 -0.3029
-0.3770 -1.3753 -39.5333
16.6600 -41.5600 -0.3003 0.3605 -
0.5788 -0.1008 0.1667 0.4513 -0.3043
-0.3726 -1.3820 -39.5364
16.6800 -40.6500 0.8452 0.0176 -
0.4173 -0.0824 0.1570 0.4836 -0.3055
-0.3683 -1.3888 -39.5396

16.7000 -42.7000 -1.3130 -0.1122 -
0.2261 -0.0501 0.1462 0.5146 -0.3066
-0.3639 -1.3955 -39.5427
16.7200 -39.3800 1.4243 0.1701 -
0.0320 -0.0097 0.1346 0.5444 -0.3074
-0.3596 -1.4022 -39.5458
16.7400 -40.7300 -0.1801 0.1287
0.1590 0.0319 0.1225 0.5729 -0.3079
-0.3552 -1.4089 -39.5489
16.7600 -41.7700 -1.1606 -0.1374
0.3495 0.0681 0.1102 0.6000 -0.3083
-0.3509 -1.4155 -39.5521
16.7800 -40.9300 -0.3652 -0.2239
0.5315 0.0927 0.0980 0.6258 -0.3084
-0.3466 -1.4221 -39.5552
16.8000 -39.2700 0.9191 -0.1050
0.6715 0.1010 0.0862 0.6501 -0.3083
-0.3423 -1.4287 -39.5583
16.8200 -40.6300 -0.5726 0.0791
0.7232 0.0905 0.0751 0.6730 -0.3080
-0.3381 -1.4353 -39.5614
16.8400 -39.4600 0.4042 0.1963
0.6473 0.0614 0.0648 0.6945 -0.3075
-0.3338 -1.4419 -39.5645
16.8600 -40.3400 -0.1842 0.2318
0.4318 0.0170 0.0554 0.7146 -0.3067
-0.3296 -1.4484 -39.5677
16.8800 -40.3000 0.5186 0.0631
0.1186 -0.0363 0.0470 0.7332 -0.3057
-0.3254 -1.4549 -39.5708
16.9000 -42.1200 -0.6879 -0.2159 -
0.2086 -0.0913 0.0396 0.7503 -0.3045
-0.3212 -1.4614 -39.5739
16.9200 -41.2200 0.5981 -0.2902 -
0.4619 -0.1404 0.0333 0.7659 -0.3031
-0.3171 -1.4678 -39.5770
16.9400 -42.4200 -0.6981 -0.1324 -
0.5859 -0.1756 0.0280 0.7800 -0.3014
-0.3129 -1.4742 -39.5801
16.9600 -40.7000 0.7325 0.1229 -
0.5687 -0.1901 0.0239 0.7927 -0.2995
-0.3088 -1.4807 -39.5833
16.9800 -42.1100 -0.7163 0.2032 -
0.4331 -0.1804 0.0209 0.8038 -0.2973
-0.3047 -1.4870 -39.5864
17.0000 -40.4000 0.8955 -0.1183 -
0.2097 -0.1481 0.0189 0.8134 -0.2949
-0.3006 -1.4934 -39.5895
17.0200 -42.2800 -1.0548 -0.3398
0.0423 -0.0972 0.0178 0.8216 -0.2923
-0.2966 -1.4997 -39.5926

17.0400 -39.9900 0.4842 0.1126
0.2533 -0.0334 0.0176 0.8282 -0.2895
-0.2925 -1.5060 -39.5957
17.0600 -39.7600 0.0894 0.6142
0.3611 0.0363 0.0181 0.8333 -0.2864
-0.2885 -1.5123 -39.5988
17.0800 -38.9900 1.2575 0.1699
0.3353 0.1047 0.0194 0.8370 -0.2831
-0.2845 -1.5186 -39.6020
17.1000 -42.3800 -1.3418 -0.5753
0.2263 0.1650 0.0213 0.8392 -0.2795
-0.2805 -1.5248 -39.6051
17.1200 -39.8400 1.0351 -0.4496
0.0969 0.2122 0.0238 0.8399 -0.2758
-0.2766 -1.5310 -39.6082
17.1400 -40.2200 0.2564 0.1331 -
0.0162 0.2426 0.0269 0.8391 -0.2718
-0.2726 -1.5372 -39.6113
17.1600 -40.5000 -0.3398 0.4540 -
0.0946 0.2535 0.0306 0.8369 -0.2676
-0.2687 -1.5434 -39.6144
17.1800 -40.5500 -0.2002 0.3353 -
0.1321 0.2434 0.0348 0.8332 -0.2631
-0.2648 -1.5496 -39.6175
17.2000 -40.1800 0.6879 -0.0918 -
0.1296 0.2132 0.0396 0.8282 -0.2585
-0.2609 -1.5557 -39.6206
17.2200 -41.2400 -0.0237 -0.4458 -
0.0880 0.1651 0.0449 0.8217 -0.2536
-0.2571 -1.5618 -39.6237
17.2400 -42.2400 -1.1238 -0.3798 -
0.0127 0.1016 0.0510 0.8138 -0.2486
-0.2532 -1.5678 -39.6268
17.2600 -39.8200 0.9855 0.0558
0.0893 0.0265 0.0577 0.8045 -0.2433
-0.2494 -1.5739 -39.6299
17.2800 -39.7900 0.6159 0.2759
0.2034 -0.0554 0.0653 0.7939 -0.2378
-0.2456 -1.5799 -39.6331
17.3000 -41.5500 -1.0345 0.1863
0.2925 -0.1382 0.0738 0.7820 -0.2322
-0.2418 -1.5859 -39.6362
17.3200 -40.5700 0.0639 0.1446
0.3005 -0.2163 0.0832 0.7688 -0.2264
-0.2380 -1.5919 -39.6393
17.3400 -40.6600 -0.2258 0.3769
0.1985 -0.2841 0.0935 0.7543 -0.2204
-0.2343 -1.5979 -39.6424
17.3600 -39.9300 0.7652 0.3850
0.0094 -0.3368 0.1049 0.7387 -0.2143
-0.2306 -1.6038 -39.6455

17.3800 -41.2500 0.2229 -0.0997 -
0.2140 -0.3694 0.1172 0.7218 -0.2079
-0.2269 -1.6097 -39.6486
17.4000 -42.9600 -0.6795 -0.6119 -
0.3944 -0.3779 0.1306 0.7038 -0.2015
-0.2232 -1.6156 -39.6517
17.4200 -42.3700 -0.0449 -0.5864 -
0.4624 -0.3593 0.1448 0.6847 -0.1949
-0.2195 -1.6215 -39.6548
17.4400 -41.5300 0.2949 -0.1367 -
0.4062 -0.3140 0.1596 0.6645 -0.1882
-0.2159 -1.6273 -39.6579
17.4600 -40.7500 0.5097 0.1943 -
0.2604 -0.2454 0.1748 0.6434 -0.1813
-0.2122 -1.6331 -39.6610
17.4800 -40.7400 0.2482 0.1937 -
0.0716 -0.1595 0.1897 0.6213 -0.1743
-0.2086 -1.6389 -39.6641
17.5000 -41.4300 -0.7527 0.1511
0.1138 -0.0631 0.2040 0.5983 -0.1672
-0.2050 -1.6447 -39.6672
17.5200 -41.2300 -0.8637 0.2369
0.2628 0.0366 0.2173 0.5746 -0.1600
-0.2014 -1.6504 -39.6703
17.5400 -38.8800 1.3449 0.2184
0.3658 0.1323 0.2291 0.5500 -0.1527
-0.1979 -1.6561 -39.6734
17.5600 -40.4500 -0.1575 -0.1250
0.4212 0.2171 0.2389 0.5248 -0.1453
-0.1943 -1.6618 -39.6765
17.5800 -41.8700 -1.2637 -0.4081
0.4137 0.2863 0.2463 0.4989 -0.1379
-0.1908 -1.6675 -39.6796
17.6000 -39.5800 0.9002 -0.2312
0.3304 0.3379 0.2509 0.4725 -0.1303
-0.1873 -1.6732 -39.6827
17.6200 -39.7000 0.5250 0.2762
0.1788 0.3718 0.2521 0.4455 -0.1227
-0.1838 -1.6788 -39.6858
17.6400 -40.8900 -0.8642 0.6261 -
0.0165 0.3886 0.2497 0.4182 -0.1150
-0.1803 -1.6844 -39.6888
17.6600 -40.0500 0.3255 0.4148 -
0.2190 0.3890 0.2434 0.3904 -0.1073
-0.1768 -1.6900 -39.6919
17.6800 -40.7000 0.6564 -0.1932 -
0.3790 0.3742 0.2331 0.3624 -0.0995
-0.1733 -1.6956 -39.6950
17.7000 -42.5200 -0.7915 -0.7232 -
0.4426 0.3453 0.2188 0.3342 -0.0917
-0.1699 -1.7011 -39.6981

17.7200 -41.3800 0.5940 -0.7048 -
0.3662 0.3044 0.2008 0.3057 -0.0838
-0.1665 -1.7066 -39.7012
17.7400 -41.3100 0.0853 -0.3625 -
0.1566 0.2533 0.1793 0.2773 -0.0759
-0.1631 -1.7121 -39.7043
17.7600 -41.0400 -0.1193 0.0946
0.1123 0.1937 0.1548 0.2487 -0.0680
-0.1597 -1.7176 -39.7074
17.7800 -40.8900 -0.7820 0.7184
0.3321 0.1275 0.1276 0.2203 -0.0601
-0.1563 -1.7231 -39.7105
17.8000 -38.9500 1.0981 0.9217
0.4239 0.0567 0.0981 0.1920 -0.0522
-0.1529 -1.7285 -39.7136
17.8200 -41.4600 -0.6115 0.1527
0.3616 -0.0161 0.0666 0.1638 -0.0443
-0.1496 -1.7339 -39.7167
17.8400 -42.3800 -0.2008 -0.7552
0.2064 -0.0872 0.0334 0.1360 -0.0364
-0.1463 -1.7393 -39.7197
17.8600 -43.3900 -0.9844 -0.7138
0.0335 -0.1532 -0.0009 0.1084 -0.0286
-0.1429 -1.7446 -39.7228
17.8800 -41.1200 0.7983 0.0270 -
0.1154 -0.2106 -0.0361 0.0813 -0.0207
-0.1396 -1.7500 -39.7259
17.9000 -40.9300 0.6217 0.5760 -
0.2138 -0.2559 -0.0718 0.0547 -0.0130
-0.1363 -1.7553 -39.7290
17.9200 -42.5100 -0.7759 0.4690 -
0.2455 -0.2859 -0.1076 0.0286 -0.0052
-0.1331 -1.7606 -39.7321
17.9400 -41.7400 0.6400 -0.1159 -
0.2205 -0.2975 -0.1429 0.0032 0.0025
-0.1298 -1.7659 -39.7352
17.9600 -43.5200 -0.6565 -0.5241 -
0.1726 -0.2889 -0.1774 -0.0216 0.0102
-0.1265 -1.7711 -39.7382
17.9800 -42.2700 0.4837 -0.4496 -
0.1340 -0.2604 -0.2107 -0.0456 0.0177
-0.1233 -1.7764 -39.7413
18.0000 -42.6800 -0.4823 0.0470 -
0.1148 -0.2140 -0.2422 -0.0688 0.0252
-0.1201 -1.7816 -39.7444
18.0200 -41.8500 -0.2192 0.6187 -
0.1147 -0.1535 -0.2715 -0.0910 0.0327
-0.1168 -1.7867 -39.7475
18.0400 -40.5100 0.8122 0.7505 -
0.1286 -0.0836 -0.2982 -0.1124 0.0400
-0.1136 -1.7919 -39.7505

18.0600 -42.8300 -0.7005 0.1299 –
0.1425 -0.0104 -0.3219 -0.1327 0.0472
-0.1104 -1.7971 -39.7536
18.0800 -42.8400 0.1712 -0.6905 –
0.1277 0.0595 -0.3422 -0.1519 0.0544
-0.1073 -1.8022 -39.7567
18.1000 -42.8100 0.2493 -0.9607 –
0.0548 0.1201 -0.3587 -0.1699 0.0614
-0.1041 -1.8073 -39.7598
18.1200 -42.9700 -0.6883 -0.3929
0.0836 0.1662 -0.3711 -0.1868 0.0683
-0.1009 -1.8124 -39.7628
18.1400 -40.5900 0.6469 0.5204
0.2445 0.1943 -0.3791 -0.2024 0.0751
-0.0978 -1.8174 -39.7659
18.1600 -40.6800 0.1084 0.9088
0.3704 0.2016 -0.3824 -0.2167 0.0817
-0.0946 -1.8225 -39.7690
18.1800 -40.9800 -0.0980 0.6358
0.4140 0.1865 -0.3809 -0.2297 0.0882
-0.0915 -1.8275 -39.7721
18.2000 -42.2900 -0.7689 0.1361
0.3664 0.1500 -0.3746 -0.2414 0.0945
-0.0884 -1.8325 -39.7751
18.2200 -41.8300 0.3561 -0.3710
0.2461 0.0956 -0.3636 -0.2517 0.1007
-0.0852 -1.8374 -39.7782
18.2400 -41.6300 1.2312 -0.6671
0.0886 0.0288 -0.3480 -0.2607 0.1067
-0.0821 -1.8424 -39.7813
18.2600 -44.3700 -1.5903 -0.4605 –
0.0740 -0.0443 -0.3279 -0.2684 0.1125
-0.0790 -1.8473 -39.7843
18.2800 -42.1400 0.2128 0.1906 –
0.2139 -0.1168 -0.3036 -0.2748 0.1182
-0.0759 -1.8522 -39.7874
18.3000 -40.7300 1.4923 0.5531 –
0.3091 -0.1816 -0.2752 -0.2800 0.1237
-0.0729 -1.8571 -39.7905
18.3200 -43.7900 -1.2285 0.1234 –
0.3530 -0.2324 -0.2430 -0.2839 0.1289
-0.0698 -1.8620 -39.7935
18.3400 -42.4300 0.6339 -0.3204 –
0.3498 -0.2647 -0.2070 -0.2867 0.1340
-0.0667 -1.8668 -39.7966
18.3600 -43.0100 -0.2554 -0.1570 –
0.3075 -0.2761 -0.1678 -0.2883 0.1389
-0.0637 -1.8717 -39.7997
18.3800 -42.3400 0.0572 0.1424 –
0.2350 -0.2664 -0.1257 -0.2888 0.1436
-0.0606 -1.8765 -39.8027

18.4000 -41.9100 0.1845 0.1916 -
0.1401 -0.2372 -0.0812 -0.2882 0.1480
-0.0575 -1.8813 -39.8058
18.4200 -42.1000 0.0718 -0.0359 -
0.0356 -0.1915 -0.0351 -0.2867 0.1523
-0.0545 -1.8860 -39.8089
18.4400 -42.2600 -0.1316 -0.2783
0.0648 -0.1334 0.0120 -0.2842 0.1563
-0.0515 -1.8908 -39.8119
18.4600 -42.8400 -0.8984 -0.1764
0.1478 -0.0673 0.0596 -0.2807 0.1601
-0.0484 -1.8955 -39.8150
18.4800 -40.0400 1.3205 0.1759
0.1992 0.0020 0.1070 -0.2764 0.1638
-0.0454 -1.9002 -39.8180
18.5000 -42.2100 -1.2475 0.3716
0.2129 0.0694 0.1535 -0.2712 0.1671
-0.0424 -1.9049 -39.8211
18.5200 -41.7300 -0.7866 0.3169
0.1908 0.1297 0.1985 -0.2652 0.1703
-0.0394 -1.9096 -39.8241
18.5400 -39.9100 1.3328 0.0562
0.1464 0.1781 0.2413 -0.2585 0.1733
-0.0364 -1.9142 -39.8272
18.5600 -40.9400 0.6927 -0.3540
0.1033 0.2110 0.2814 -0.2512 0.1760
-0.0334 -1.9188 -39.8303
18.5800 -42.8700 -1.1560 -0.6090
0.0840 0.2260 0.3180 -0.2431 0.1786
-0.0304 -1.9234 -39.8333
18.6000 -41.4000 0.0772 -0.3613
0.1010 0.2225 0.3507 -0.2345 0.1809
-0.0274 -1.9280 -39.8364
18.6200 -40.3800 0.5332 0.1781
0.1375 0.2023 0.3789 -0.2253 0.1830
-0.0244 -1.9326 -39.8394
18.6400 -40.9600 -0.3980 0.5339
0.1638 0.1690 0.4022 -0.2157 0.1849
-0.0214 -1.9371 -39.8425
18.6600 -40.1100 0.5424 0.4059
0.1443 0.1266 0.4204 -0.2055 0.1866
-0.0184 -1.9417 -39.8455
18.6800 -41.7200 -0.6517 0.1517
0.0638 0.0797 0.4332 -0.1950 0.1882
-0.0155 -1.9462 -39.8486
18.7000 -41.0800 0.2280 -0.0126 -
0.0617 0.0335 0.4403 -0.1840 0.1895
-0.0125 -1.9507 -39.8516
18.7200 -41.0300 0.6670 -0.2028 -
0.1909 -0.0073 0.4417 -0.1728 0.1906
-0.0095 -1.9551 -39.8547

18.7400 -42.6700 -0.6522 -0.4088 -
0.2777 -0.0380 0.4372 -0.1613 0.1915
-0.0066 -1.9596 -39.8577
18.7600 -42.2300 -0.2713 -0.3943 -
0.2889 -0.0550 0.4268 -0.1496 0.1922
-0.0036 -1.9640 -39.8608
18.7800 -41.4400 0.3748 -0.1583 -
0.2226 -0.0565 0.4106 -0.1376 0.1927
-0.0006 -1.9684 -39.8638
18.8000 -41.2600 0.2483 0.0379 -
0.1024 -0.0435 0.3887 -0.1255 0.1931
0.0023 -1.9728 -39.8668
18.8200 -40.8700 0.3674 0.0981
0.0306 -0.0188 0.3616 -0.1134 0.1932
0.0053 -1.9772 -39.8699
18.8400 -41.9700 -0.9247 0.2489
0.1295 0.0143 0.3297 -0.1011 0.1932
0.0082 -1.9815 -39.8729
18.8600 -40.3800 0.6631 0.3797
0.1664 0.0520 0.2935 -0.0887 0.1930
0.0112 -1.9859 -39.8760
18.8800 -40.2700 0.7139 0.2404
0.1359 0.0899 0.2536 -0.0764 0.1926
0.0141 -1.9902 -39.8790
18.9000 -42.3500 -0.8307 -0.1009
0.0606 0.1237 0.2107 -0.0640 0.1920
0.0171 -1.9945 -39.8821
18.9200 -42.0900 -0.4868 -0.2668 -
0.0232 0.1497 0.1654 -0.0517 0.1912
0.0200 -1.9988 -39.8851
18.9400 -40.8600 0.8347 -0.1775 -
0.0743 0.1645 0.1186 -0.0395 0.1903
0.0229 -2.0030 -39.8881
18.9600 -42.1000 -0.4590 -0.0947 -
0.0627 0.1656 0.0708 -0.0274 0.1892
0.0259 -2.0073 -39.8912
18.9800 -41.3000 0.3025 -0.0826
0.0051 0.1517 0.0230 -0.0154 0.1879
0.0288 -2.0115 -39.8942
19.0000 -42.1600 -0.7803 0.2078
0.0899 0.1236 -0.0243 -0.0035 0.1865
0.0317 -2.0157 -39.8972
19.0200 -40.0600 0.9652 0.3582
0.1513 0.0837 -0.0704 0.0082 0.1849
0.0347 -2.0199 -39.9003
19.0400 -42.6000 -0.8314 -0.0334
0.1591 0.0353 -0.1146 0.0196 0.1831
0.0376 -2.0240 -39.9033
19.0600 -41.8200 0.1243 -0.3009
0.1194 -0.0165 -0.1563 0.0309 0.1812
0.0405 -2.0282 -39.9063

19.0800 -42.0400 0.0291 -0.1730
0.0511 -0.0667 -0.1950 0.0419 0.1791
0.0434 -2.0323 -39.9094
19.1000 -41.4800 0.5537 0.0439 -
0.0251 -0.1103 -0.2300 0.0526 0.1768
0.0463 -2.0364 -39.9124
19.1200 -42.4900 -0.5296 0.1564 -
0.0912 -0.1426 -0.2610 0.0630 0.1744
0.0492 -2.0405 -39.9154
19.1400 -42.7600 -0.5307 0.0635 -
0.1415 -0.1607 -0.2875 0.0731 0.1718
0.0521 -2.0446 -39.9185
19.1600 -41.3700 1.1898 -0.1177 -
0.1759 -0.1638 -0.3089 0.0828 0.1691
0.0550 -2.0487 -39.9215
19.1800 -43.7100 -1.3237 -0.0715 -
0.1917 -0.1523 -0.3250 0.0922 0.1662
0.0579 -2.0527 -39.9245
19.2000 -42.5500 -0.5588 0.1927 -
0.1777 -0.1288 -0.3357 0.1012 0.1631
0.0608 -2.0567 -39.9275
19.2200 -40.5000 1.5783 0.2421 -
0.1190 -0.0975 -0.3411 0.1097 0.1599
0.0637 -2.0607 -39.9306
19.2400 -43.7600 -1.4814 -0.2081 -
0.0104 -0.0635 -0.3410 0.1179 0.1566
0.0665 -2.0647 -39.9336
19.2600 -41.2400 1.1371 -0.4567
0.1212 -0.0315 -0.3358 0.1256 0.1531
0.0694 -2.0687 -39.9366
19.2800 -42.7100 -0.9076 -0.0512
0.2335 -0.0057 -0.3255 0.1328 0.1495
0.0723 -2.0726 -39.9396
19.3000 -40.4300 0.8134 0.4556
0.2834 0.0111 -0.3105 0.1396 0.1457
0.0751 -2.0766 -39.9426
19.3200 -41.2600 -0.1276 0.5817
0.2469 0.0176 -0.2911 0.1460 0.1418
0.0780 -2.0805 -39.9457
19.3400 -41.7000 -0.1624 0.2877
0.1325 0.0144 -0.2677 0.1518 0.1377
0.0809 -2.0844 -39.9487
19.3600 -41.7300 0.4539 -0.2273 -
0.0165 0.0035 -0.2407 0.1572 0.1335
0.0837 -2.0883 -39.9517
19.3800 -42.8800 -0.4023 -0.6625 -
0.1434 -0.0125 -0.2106 0.1622 0.1292
0.0865 -2.0921 -39.9547
19.4000 -43.0800 -0.3210 -0.6125 -
0.2064 -0.0302 -0.1779 0.1667 0.1247
0.0894 -2.0960 -39.9577

19.4200 -42.3400 -0.2776 -0.0305 -
0.2064 -0.0456 -0.1431 0.1707 0.1202
0.0922 -2.0998 -39.9607
19.4400 -40.8300 0.7094 0.5238 -
0.1710 -0.0547 -0.1067 0.1743 0.1154
0.0950 -2.1036 -39.9637
19.4600 -41.3700 0.0407 0.5369 -
0.1310 -0.0543 -0.0693 0.1774 0.1106
0.0978 -2.1074 -39.9668
19.4800 -42.1600 -0.5180 0.1792 -
0.1055 -0.0429 -0.0314 0.1801 0.1057
0.1006 -2.1112 -39.9698
19.5000 -41.3700 0.5776 -0.1448 -
0.0927 -0.0211 0.0065 0.1823 0.1006
0.1034 -2.1149 -39.9728
19.5200 -42.6400 -0.7804 -0.1796 -
0.0752 0.0088 0.0439 0.1841 0.0955
0.1062 -2.1187 -39.9758
19.5400 -40.9500 0.8824 -0.1797 -
0.0269 0.0435 0.0803 0.1855 0.0902
0.1090 -2.1224 -39.9788
19.5600 -42.5300 -0.9189 -0.2034
0.0637 0.0788 0.1151 0.1864 0.0849
0.1118 -2.1261 -39.9818
19.5800 -40.4600 0.7755 -0.0182
0.1773 0.1110 0.1479 0.1868 0.0794
0.1146 -2.1298 -39.9848
19.6000 -40.8500 0.2321 0.1200
0.2770 0.1369 0.1783 0.1868 0.0739
0.1173 -2.1335 -39.9878
19.6200 -40.8800 0.1290 0.0601
0.3321 0.1545 0.2060 0.1863 0.0682
0.1201 -2.1371 -39.9908
19.6400 -41.8200 -0.9293 0.1394
0.3215 0.1624 0.2305 0.1854 0.0625
0.1228 -2.1408 -39.9938
19.6600 -39.9300 0.9581 0.2819
0.2377 0.1612 0.2517 0.1841 0.0567
0.1256 -2.1444 -39.9968
19.6800 -41.5100 -0.4202 0.1589
0.0916 0.1527 0.2693 0.1823 0.0509
0.1283 -2.1480 -39.9998
19.7000 -42.0200 -0.6320 -0.0126 -
0.0903 0.1390 0.2832 0.1800 0.0449
0.1310 -2.1516 -40.0028
19.7200 -41.4700 0.2929 0.0108 -
0.2751 0.1221 0.2934 0.1774 0.0389
0.1337 -2.1552 -40.0058
19.7400 -41.3700 0.2723 0.0971 -
0.4144 0.1040 0.2999 0.1743 0.0329
0.1364 -2.1587 -40.0088

19.7600 -41.6300 0.5601 -0.1321 -
0.4598 0.0865 0.3026 0.1708 0.0268
0.1391 -2.1622 -40.0118
19.7800 -42.8100 -0.1936 -0.5404 -
0.3960 0.0705 0.3017 0.1669 0.0206
0.1418 -2.1658 -40.0148
19.8000 -43.0400 -0.7841 -0.5948 -
0.2334 0.0562 0.2975 0.1626 0.0144
0.1445 -2.1693 -40.0178
19.8200 -41.3800 0.2547 -0.1102 -
0.0084 0.0424 0.2899 0.1579 0.0081
0.1471 -2.1728 -40.0208
19.8400 -40.2000 0.7541 0.4334
0.2158 0.0277 0.2793 0.1528 0.0018
0.1498 -2.1762 -40.0238
19.8600 -40.7100 -0.0145 0.5985
0.3750 0.0107 0.2657 0.1474 -0.0045
0.1524 -2.1797 -40.0268
19.8800 -41.4100 -0.6390 0.4636
0.4304 -0.0096 0.2496 0.1416 -0.0109
0.1551 -2.1831 -40.0298
19.9000 -40.6600 0.5130 0.1848
0.3827 -0.0326 0.2309 0.1355 -0.0172
0.1577 -2.1865 -40.0327
19.9200 -41.6000 -0.0209 -0.1593
0.2652 -0.0562 0.2101 0.1291 -0.0236
0.1603 -2.1899 -40.0357
19.9400 -42.6000 -0.3745 -0.4284
0.1195 -0.0784 0.1872 0.1225 -0.0300
0.1629 -2.1933 -40.0387
19.9600 -42.1600 0.3206 -0.4370 -
0.0181 -0.0975 0.1625 0.1156 -0.0364
0.1654 -2.1967 -40.0417
19.9800 -42.6400 -0.4855 -0.2060 -
0.1210 -0.1112 0.1364 0.1084 -0.0428
0.1680 -2.2000 -40.0447
20.0000 -42.0100 0.2985 0.0245 -
0.1761 -0.1170 0.1090 0.1010 -0.0492
0.1705 -2.2034 -40.0477
20.0200 -41.6400 0.5705 0.0804 -
0.1894 -0.1121 0.0807 0.0935 -0.0556
0.1731 -2.2067 -40.0507
20.0400 -42.7700 -0.4615 0.0253 -
0.1839 -0.0947 0.0517 0.0858 -0.0619
0.1756 -2.2100 -40.0536
20.0600 -42.7100 -0.5631 0.0922 -
0.1845 -0.0637 0.0225 0.0779 -0.0682
0.1781 -2.2133 -40.0566
20.0800 -41.3300 0.7562 0.2256 -
0.1982 -0.0204 -0.0067 0.0699 -0.0745
0.1806 -2.2166 -40.0596

20.1000 -42.6700 -0.5480 0.1722 -
0.2126 0.0320 -0.0355 0.0619 -0.0808
0.1831 -2.2198 -40.0626
20.1200 -41.9900 0.2944 -0.0283 -
0.2028 0.0887 -0.0632 0.0537 -0.0870
0.1855 -2.2230 -40.0655
20.1400 -42.6200 -0.0644 -0.2710 -
0.1385 0.1439 -0.0895 0.0455 -0.0932
0.1880 -2.2263 -40.0685
20.1600 -42.1800 0.3443 -0.4247 -
0.0078 0.1914 -0.1139 0.0373 -0.0992
0.1904 -2.2295 -40.0715
20.1800 -42.7900 -0.8199 -0.0805
0.1628 0.2252 -0.1360 0.0290 -0.1053
0.1928 -2.2327 -40.0745
20.2000 -40.3500 0.9312 0.4525
0.3127 0.2402 -0.1552 0.0207 -0.1112
0.1952 -2.2358 -40.0774
20.2200 -41.4100 -0.0667 0.3669
0.3832 0.2320 -0.1714 0.0125 -0.1171
0.1976 -2.2390 -40.0804
20.2400 -42.4800 -0.5638 -0.1070
0.3608 0.1995 -0.1843 0.0043 -0.1229
0.2000 -2.2421 -40.0834
20.2600 -42.5200 -0.2595 -0.3055
0.2651 0.1444 -0.1938 -0.0038 -0.1286
0.2023 -2.2452 -40.0863
20.2800 -41.9000 0.5644 -0.1581
0.1255 0.0709 -0.1999 -0.0119 -0.1342
0.2047 -2.2483 -40.0893
20.3000 -42.4000 0.0290 0.1028 -
0.0205 -0.0161 -0.2026 -0.0199 -0.1397
0.2070 -2.2514 -40.0923
20.3200 -43.1500 -0.6167 0.2688 -
0.1366 -0.1113 -0.2019 -0.0277 -0.1451
0.2093 -2.2545 -40.0952
20.3400 -41.8900 0.7811 0.1857 -
0.1967 -0.2090 -0.1978 -0.0355 -0.1504
0.2116 -2.2576 -40.0982
20.3600 -43.0900 0.0080 -0.1295 -
0.1943 -0.3033 -0.1906 -0.0431 -0.1556
0.2138 -2.2606 -40.1012
20.3800 -44.5500 -0.9678 -0.4015 -
0.1425 -0.3871 -0.1801 -0.0506 -0.1606
0.2160 -2.2636 -40.1041
20.4000 -42.4500 0.9587 -0.3756 -
0.0659 -0.4540 -0.1667 -0.0579 -0.1655
0.2183 -2.2666 -40.1071
20.4200 -43.2000 -0.0152 -0.1226
0.0001 -0.4986 -0.1502 -0.0651 -0.1702
0.2205 -2.2696 -40.1100

20.4400 -43.5100 -0.8292 0.2592
0.0223 -0.5170 -0.1310 -0.0722 -0.1748
0.2227 -2.2726 -40.1130
20.4600 -42.0100 0.3317 0.6328 -
0.0210 -0.5068 -0.1092 -0.0790 -0.1792
0.2248 -2.2756 -40.1160
20.4800 -41.9300 0.4762 0.6998 -
0.1241 -0.4674 -0.0851 -0.0858 -0.1835
0.2270 -2.2785 -40.1189
20.5000 -43.2000 -0.1717 0.2255 -
0.2564 -0.3997 -0.0592 -0.0923 -0.1876
0.2291 -2.2814 -40.1219
20.5200 -43.3600 0.3832 -0.5670 -
0.3638 -0.3063 -0.0322 -0.0987 -0.1915
0.2312 -2.2843 -40.1248
20.5400 -44.9600 -0.9032 -0.8916 -
0.3878 -0.1911 -0.0046 -0.1050 -0.1953
0.2332 -2.2872 -40.1278
20.5600 -42.5400 0.7529 -0.4035 -
0.2946 -0.0598 0.0230 -0.1111 -0.1988
0.2353 -2.2901 -40.1307
20.5800 -42.0100 0.2946 0.2451 -
0.1186 0.0797 0.0498 -0.1171 -0.2022
0.2373 -2.2930 -40.1337
20.6000 -41.9200 -0.4550 0.6060
0.0839 0.2188 0.0751 -0.1229 -0.2053
0.2394 -2.2958 -40.1366
20.6200 -42.4200 -1.0718 0.6529
0.2541 0.3488 0.0984 -0.1286 -0.2083
0.2413 -2.2986 -40.1396
20.6400 -40.6200 0.7314 0.3111
0.3584 0.4607 0.1188 -0.1341 -0.2110
0.2433 -2.3015 -40.1425
20.6600 -40.0600 1.5958 -0.2210
0.3906 0.5458 0.1358 -0.1396 -0.2135
0.2453 -2.3043 -40.1455
20.6800 -43.6000 -1.7390 -0.5163
0.3485 0.5982 0.1487 -0.1448 -0.2158
0.2472 -2.3070 -40.1484
20.7000 -42.1100 -0.3184 -0.2026
0.2440 0.6148 0.1568 -0.1500 -0.2179
0.2491 -2.3098 -40.1513
20.7200 -39.9600 1.5665 0.1781
0.1164 0.5958 0.1601 -0.1550 -0.2197
0.2510 -2.3126 -40.1543
20.7400 -42.7300 -0.8573 0.0009
0.0101 0.5439 0.1587 -0.1599 -0.2213
0.2528 -2.3153 -40.1572
20.7600 -42.4000 -0.1140 -0.2930 -
0.0389 0.4636 0.1528 -0.1647 -0.2227
0.2546 -2.3180 -40.1602

20.7800 -42.9800 -0.9141 0.0825 -
0.0142 0.3607 0.1429 -0.1693 -0.2237
0.2564 -2.3207 -40.1631
20.8000 -40.6900 1.1955 0.3611
0.0498 0.2421 0.1297 -0.1738 -0.2246
0.2582 -2.3234 -40.1660
20.8200 -43.3700 -1.0982 0.0072
0.1039 0.1155 0.1136 -0.1781 -0.2252
0.2600 -2.3261 -40.1690
20.8400 -42.5300 0.1926 -0.2272
0.1204 -0.0116 0.0952 -0.1824 -0.2255
0.2617 -2.3287 -40.1719
20.8600 -42.1000 0.6707 -0.1106
0.0861 -0.1325 0.0752 -0.1864 -0.2256
0.2634 -2.3314 -40.1748
20.8800 -43.3000 -0.6382 0.1251
0.0072 -0.2419 0.0543 -0.1904 -0.2253
0.2651 -2.3340 -40.1778
20.9000 -42.2400 0.5668 0.1371 -
0.0945 -0.3353 0.0330 -0.1941 -0.2249
0.2667 -2.3366 -40.1807
20.9200 -43.9400 -0.7704 0.0562 -
0.2012 -0.4082 0.0120 -0.1977 -0.2241
0.2684 -2.3392 -40.1836
20.9400 -42.9400 0.5626 -0.0196 -
0.2903 -0.4567 -0.0082 -0.2012 -0.2231
0.2700 -2.3418 -40.1866
20.9600 -43.1100 0.5455 -0.1837 -
0.3354 -0.4786 -0.0272 -0.2044 -0.2218
0.2715 -2.3443 -40.1895
20.9800 -44.8200 -0.9919 -0.2874 -
0.3217 -0.4732 -0.0445 -0.2075 -0.2202
0.2731 -2.3469 -40.1924
21.0000 -43.4900 -0.0444 -0.0710 -
0.2473 -0.4423 -0.0597 -0.2104 -0.2183
0.2746 -2.3494 -40.1953
21.0200 -42.5300 0.5682 0.2306 -
0.1294 -0.3897 -0.0727 -0.2131 -0.2161
0.2761 -2.3519 -40.1983
21.0400 -42.2700 0.6602 0.1934
0.0084 -0.3205 -0.0833 -0.2156 -0.2137
0.2776 -2.3544 -40.2012
21.0600 -43.6600 -0.5319 -0.1138
0.1395 -0.2403 -0.0913 -0.2179 -0.2109
0.2790 -2.3569 -40.2041
21.0800 -43.2700 -0.3146 -0.2206
0.2313 -0.1549 -0.0968 -0.2201 -0.2079
0.2805 -2.3593 -40.2070
21.1000 -42.6900 -0.1918 0.1092
0.2605 -0.0694 -0.0997 -0.2220 -0.2046
0.2819 -2.3618 -40.2099

21.1200 -41.8400 0.2388 0.5619
0.2225 0.0109 -0.0999 -0.2237 -0.2010
0.2832 -2.3642 -40.2128
21.1400 -42.0700 -0.0384 0.6502
0.1249 0.0818 -0.0976 -0.2253 -0.1971
0.2846 -2.3667 -40.2158
21.1600 -41.9100 0.5668 0.1864 -
0.0083 0.1405 -0.0928 -0.2266 -0.1930
0.2859 -2.3691 -40.2187
21.1800 -43.8900 -0.7724 -0.4020 -
0.1308 0.1851 -0.0857 -0.2277 -0.1885
0.2872 -2.3714 -40.2216
21.2000 -42.8800 0.6025 -0.6414 -
0.1907 0.2141 -0.0765 -0.2287 -0.1838
0.2884 -2.3738 -40.2245
21.2200 -43.8900 -0.5189 -0.6103 -
0.1509 0.2272 -0.0656 -0.2294 -0.1788
0.2897 -2.3762 -40.2274
21.2400 -42.6000 0.3568 -0.2989 -
0.0264 0.2256 -0.0533 -0.2299 -0.1736
0.2909 -2.3785 -40.2303
21.2600 -42.6700 -0.5265 0.3591
0.1292 0.2116 -0.0401 -0.2303 -0.1681
0.2920 -2.3809 -40.2332
21.2800 -41.0300 0.5973 0.8162
0.2479 0.1876 -0.0267 -0.2304 -0.1623
0.2932 -2.3832 -40.2361
21.3000 -41.9500 -0.2600 0.5289
0.2769 0.1573 -0.0135 -0.2304 -0.1563
0.2943 -2.3855 -40.2390
21.3200 -42.4300 0.1443 -0.1658
0.2227 0.1247 -0.0011 -0.2301 -0.1500
0.2954 -2.3877 -40.2419
21.3400 -43.8400 -0.8664 -0.5347
0.1171 0.0934 0.0098 -0.2297 -0.1435
0.2964 -2.3900 -40.2448
21.3600 -42.0600 0.9690 -0.4660 -
0.0041 0.0665 0.0188 -0.2290 -0.1368
0.2975 -2.3923 -40.2477
21.3800 -42.5300 0.3164 -0.1924 -
0.1111 0.0467 0.0253 -0.2282 -0.1298
0.2985 -2.3945 -40.2506
21.4000 -43.7500 -1.1995 0.2116 -
0.2003 0.0361 0.0290 -0.2271 -0.1226
0.2994 -2.3967 -40.2535
21.4200 -41.2200 1.0688 0.5667 -
0.2697 0.0359 0.0299 -0.2259 -0.1152
0.3004 -2.3989 -40.2564
21.4400 -42.3900 0.1627 0.3867 -
0.3108 0.0469 0.0278 -0.2244 -0.1076
0.3013 -2.4011 -40.2593

21.4600 -43.0200 0.1729 -0.2847 -
0.3090 0.0669 0.0230 -0.2226 -0.0998
0.3021 -2.4033 -40.2622
21.4800 -44.5900 -1.2374 -0.5715 -
0.2455 0.0923 0.0158 -0.2207 -0.0919
0.3030 -2.4055 -40.2651
21.5000 -41.8400 1.1275 -0.2465 -
0.1173 0.1190 0.0067 -0.2185 -0.0837
0.3038 -2.4076 -40.2680
21.5200 -42.0300 0.2910 0.1027
0.0472 0.1427 -0.0041 -0.2162 -0.0753
0.3046 -2.4097 -40.2709
21.5400 -42.8800 -0.7910 0.2920
0.2113 0.1590 -0.0160 -0.2136 -0.0668
0.3053 -2.4119 -40.2738
21.5600 -42.0800 -0.2452 0.3790
0.3386 0.1634 -0.0286 -0.2108 -0.0582
0.3060 -2.4140 -40.2767
21.5800 -41.0400 0.6988 0.2859
0.4091 0.1525 -0.0414 -0.2078 -0.0494
0.3067 -2.4161 -40.2796
21.6000 -42.8400 -0.6886 -0.0339
0.4133 0.1242 -0.0542 -0.2047 -0.0405
0.3074 -2.4181 -40.2824
21.6200 -41.9300 0.6398 -0.3087
0.3594 0.0794 -0.0662 -0.2014 -0.0314
0.3080 -2.4202 -40.2853
21.6400 -43.3800 -0.9266 -0.1543
0.2630 0.0214 -0.0773 -0.1979 -0.0222
0.3086 -2.4222 -40.2882
21.6600 -42.0400 0.3393 0.1601
0.1378 -0.0449 -0.0868 -0.1942 -0.0129
0.3092 -2.4243 -40.2911
21.6800 -41.5700 1.1280 0.2034 -
0.0050 -0.1140 -0.0944 -0.1904 -0.0036
0.3097 -2.4263 -40.2940
21.7000 -44.1600 -0.9931 -0.0732 -
0.1492 -0.1803 -0.0997 -0.1865 0.0059
0.3102 -2.4283 -40.2968
21.7200 -43.9200 -0.6344 -0.2186 -
0.2727 -0.2382 -0.1023 -0.1824 0.0155
0.3106 -2.4303 -40.2997
21.7400 -42.4800 0.9311 -0.0942 -
0.3554 -0.2829 -0.1018 -0.1782 0.0251
0.3111 -2.4322 -40.3026
21.7600 -43.7000 -0.3229 0.0961 -
0.3821 -0.3106 -0.0979 -0.1738 0.0347
0.3115 -2.4342 -40.3055
21.7800 -43.5200 -0.3712 0.1512 -
0.3478 -0.3199 -0.0904 -0.1693 0.0445
0.3118 -2.4361 -40.3083

21.8000 -43.1100 0.0439 0.0438 -
0.2558 -0.3111 -0.0792 -0.1646 0.0542
0.3122 -2.4381 -40.3112
21.8200 -42.7500 0.5481 -0.2163 -
0.1175 -0.2864 -0.0647 -0.1597 0.0640
0.3124 -2.4400 -40.3141
21.8400 -43.6000 -0.4463 -0.3540
0.0393 -0.2489 -0.0471 -0.1547 0.0738
0.3127 -2.4419 -40.3169
21.8600 -43.1300 -0.4527 -0.0898
0.1781 -0.2020 -0.0269 -0.1494 0.0836
0.3129 -2.4437 -40.3198
21.8800 -41.3300 0.7236 0.3306
0.2666 -0.1494 -0.0044 -0.1440 0.0934
0.3131 -2.4456 -40.3227
21.9000 -41.9200 -0.1157 0.4311
0.2853 -0.0943 0.0198 -0.1383 0.1032
0.3133 -2.4475 -40.3255
21.9200 -42.2600 -0.1594 0.1810
0.2410 -0.0390 0.0451 -0.1323 0.1130
0.3134 -2.4493 -40.3284
21.9400 -42.4400 -0.0191 -0.1567
0.1619 0.0143 0.0709 -0.1261 0.1228
0.3135 -2.4511 -40.3313
21.9600 -42.6800 -0.0151 -0.3746
0.0823 0.0639 0.0965 -0.1195 0.1325
0.3135 -2.4529 -40.3341
21.9800 -42.6100 -0.0857 -0.3837
0.0261 0.1091 0.1212 -0.1127 0.1421
0.3135 -2.4547 -40.3370
22.0000 -42.5400 -0.4444 -0.1005 -
0.0060 0.1502 0.1442 -0.1055 0.1517
0.3135 -2.4565 -40.3398
22.0200 -41.8600 0.0423 0.3071 -
0.0286 0.1879 0.1650 -0.0980 0.1612
0.3134 -2.4583 -40.3427
22.0400 -40.5400 1.0719 0.4073 -
0.0612 0.2231 0.1829 -0.0902 0.1706
0.3134 -2.4601 -40.3456
22.0600 -43.2600 -1.2459 0.0556 -
0.1138 0.2559 0.1972 -0.0819 0.1800
0.3132 -2.4618 -40.3484
22.0800 -41.0200 1.1975 -0.2464 -
0.1671 0.2859 0.2074 -0.0733 0.1892
0.3131 -2.4635 -40.3513
22.1000 -43.2600 -0.9971 -0.2273 -
0.1874 0.3122 0.2130 -0.0643 0.1983
0.3129 -2.4652 -40.3541
22.1200 -42.0500 -0.0061 -0.0432 -
0.1511 0.3337 0.2137 -0.0549 0.2073
0.3126 -2.4669 -40.3570

22.1400 -40.9700 0.7646 0.0678 -
0.0597 0.3485 0.2093 -0.0451 0.2162
0.3123 -2.4686 -40.3598
22.1600 -42.1900 -0.4664 0.0235
0.0612 0.3538 0.1997 -0.0350 0.2249
0.3120 -2.4703 -40.3627
22.1800 -41.5200 0.0134 -0.0034
0.1721 0.3469 0.1851 -0.0244 0.2335
0.3117 -2.4719 -40.3655
22.2000 -41.5300 -0.0054 0.1503
0.2366 0.3257 0.1657 -0.0135 0.2419
0.3113 -2.4736 -40.3683
22.2200 -41.1700 0.1657 0.3007
0.2391 0.2890 0.1419 -0.0023 0.2501
0.3109 -2.4752 -40.3712
22.2400 -41.4200 0.2261 0.1567
0.1866 0.2372 0.1142 0.0093 0.2582
0.3104 -2.4768 -40.3740
22.2600 -42.3300 -0.1925 -0.2203
0.0976 0.1720 0.0833 0.0211 0.2661
0.3099 -2.4784 -40.3769
22.2800 -42.3800 0.1821 -0.4473 -
0.0003 0.0965 0.0498 0.0332 0.2738
0.3094 -2.4800 -40.3797
22.3000 -43.0600 -0.6264 -0.2033 -
0.0792 0.0148 0.0144 0.0456 0.2812
0.3088 -2.4816 -40.3825
22.3200 -42.6900 -0.5558 0.3062 -
0.1309 -0.0688 -0.0223 0.0582 0.2885
0.3082 -2.4832 -40.3854
22.3400 -41.0200 1.0951 0.4654 -
0.1564 -0.1497 -0.0594 0.0710 0.2955
0.3075 -2.4847 -40.3882
22.3600 -43.7100 -1.0031 -0.0595 -
0.1541 -0.2237 -0.0965 0.0839 0.3024
0.3068 -2.4862 -40.3910
22.3800 -42.4200 0.6823 -0.4575 -
0.1200 -0.2876 -0.1327 0.0970 0.3089
0.3061 -2.4878 -40.3939
22.4000 -43.4900 -0.4952 -0.2762 -
0.0580 -0.3392 -0.1673 0.1101 0.3153
0.3053 -2.4893 -40.3967
22.4200 -42.2900 0.3839 0.0913
0.0159 -0.3775 -0.1996 0.1233 0.3213
0.3045 -2.4908 -40.3995
22.4400 -42.5300 -0.4172 0.4380
0.0776 -0.4019 -0.2292 0.1366 0.3271
0.3037 -2.4922 -40.4023
22.4600 -41.8600 0.2219 0.4838
0.1142 -0.4124 -0.2553 0.1498 0.3327
0.3028 -2.4937 -40.4052

22.4800 -42.0200 0.5730 0.0686
0.1220 -0.4092 -0.2776 0.1630 0.3379
0.3019 -2.4951 -40.4080
22.5000 -43.9600 -0.7566 -0.5044
0.1099 -0.3931 -0.2957 0.1761 0.3429
0.3009 -2.4966 -40.4108
22.5200 -42.9900 0.3251 -0.5984
0.0858 -0.3646 -0.3096 0.1891 0.3475
0.2999 -2.4980 -40.4136
22.5400 -42.8300 -0.0976 -0.1482
0.0522 -0.3246 -0.3190 0.2020 0.3519
0.2989 -2.4994 -40.4165
22.5600 -42.0700 0.1827 0.3544
0.0055 -0.2742 -0.3240 0.2146 0.3559
0.2978 -2.5008 -40.4193
22.5800 -42.3200 -0.2969 0.5951 -
0.0587 -0.2146 -0.3247 0.2271 0.3597
0.2967 -2.5022 -40.4221
22.6000 -41.6700 0.4531 0.5187 -
0.1353 -0.1477 -0.3211 0.2392 0.3630
0.2955 -2.5036 -40.4249
22.6200 -42.7800 -0.2751 0.1482 -
0.2056 -0.0757 -0.3134 0.2510 0.3661
0.2943 -2.5049 -40.4277
22.6400 -42.9300 -0.0032 -0.3637 -
0.2392 -0.0013 -0.3017 0.2625 0.3688
0.2931 -2.5063 -40.4305
22.6600 -43.0100 0.2364 -0.7552 -
0.2059 0.0729 -0.2861 0.2735 0.3712
0.2918 -2.5076 -40.4333
22.6800 -43.3100 -0.2632 -0.7669 -
0.0900 0.1436 -0.2669 0.2841 0.3732
0.2905 -2.5089 -40.4361
22.7000 -42.7400 -0.4763 -0.2545
0.0895 0.2073 -0.2442 0.2942 0.3748
0.2891 -2.5102 -40.4389
22.7200 -40.5500 0.6134 0.5236
0.2708 0.2601 -0.2181 0.3037 0.3760
0.2877 -2.5115 -40.4418
22.7400 -40.8400 -0.1161 0.9108
0.3829 0.2983 -0.1890 0.3127 0.3769
0.2863 -2.5128 -40.4446
22.7600 -40.1900 0.6704 0.5094
0.3724 0.3186 -0.1570 0.3211 0.3774
0.2848 -2.5141 -40.4474
22.7800 -42.5600 -0.9200 -0.0707
0.2414 0.3188 -0.1225 0.3288 0.3775
0.2833 -2.5153 -40.4502
22.8000 -41.2800 0.6351 -0.1110
0.0289 0.2995 -0.0858 0.3358 0.3772
0.2818 -2.5165 -40.4530

22.8200 -42.2000 -0.1826 -0.0343 -
0.1833 0.2619 -0.0475 0.3421 0.3765
0.2802 -2.5178 -40.4558
22.8400 -41.8300 0.5629 -0.4158 -
0.3037 0.2076 -0.0079 0.3476 0.3754
0.2786 -2.5190 -40.4585
22.8600 -43.6700 -0.8474 -0.7317 -
0.2923 0.1393 0.0325 0.3523 0.3739
0.2769 -2.5202 -40.4613
22.8800 -42.3400 0.0890 -0.4939 -
0.1474 0.0605 0.0732 0.3561 0.3720
0.2752 -2.5214 -40.4641
22.9000 -41.0600 0.7386 0.0196
0.0771 -0.0242 0.1138 0.3590 0.3697
0.2735 -2.5225 -40.4669
22.9200 -42.0100 -0.8304 0.4080
0.3043 -0.1095 0.1539 0.3610 0.3670
0.2717 -2.5237 -40.4697
22.9400 -40.3400 0.6959 0.5036
0.4577 -0.1906 0.1930 0.3620 0.3639
0.2699 -2.5248 -40.4725
22.9600 -41.3300 -0.0887 0.2664
0.4911 -0.2625 0.2306 0.3620 0.3604
0.2680 -2.5260 -40.4753
22.9800 -41.8100 0.0267 -0.1538
0.3981 -0.3207 0.2662 0.3609 0.3564
0.2661 -2.5271 -40.4781
23.0000 -42.7900 -0.5117 -0.3086
0.2034 -0.3621 0.2994 0.3587 0.3521
0.2642 -2.5282 -40.4809
23.0200 -42.0500 0.1377 -0.0491 -
0.0504 -0.3846 0.3298 0.3553 0.3474
0.2623 -2.5293 -40.4836
23.0400 -41.9400 0.2855 0.2199 -
0.3077 -0.3864 0.3570 0.3507 0.3424
0.2603 -2.5304 -40.4864
23.0600 -42.1900 0.3851 0.0751 -
0.5183 -0.3658 0.3807 0.3449 0.3369
0.2582 -2.5314 -40.4892
23.0800 -43.6000 -0.6426 -0.2521 -
0.6496 -0.3215 0.4009 0.3378 0.3311
0.2562 -2.5325 -40.4920
23.1000 -43.0300 -0.1272 -0.2580 -
0.6889 -0.2532 0.4172 0.3295 0.3250
0.2541 -2.5335 -40.4948
23.1200 -42.0700 0.4422 -0.0080 -
0.6442 -0.1625 0.4296 0.3199 0.3185
0.2520 -2.5346 -40.4975
23.1400 -41.8300 0.3308 0.1066 -
0.5304 -0.0534 0.4379 0.3090 0.3117
0.2499 -2.5356 -40.5003

23.1600 -42.8100 -0.7552 0.0379 -
0.3578 0.0678 0.4419 0.2969 0.3045
0.2477 -2.5366 -40.5031
23.1800 -41.4500 0.2423 -0.1032 -
0.1401 0.1937 0.4413 0.2836 0.2971
0.2455 -2.5376 -40.5058
23.2000 -41.1800 0.5256 -0.2997
0.1057 0.3161 0.4359 0.2691 0.2893
0.2433 -2.5385 -40.5086
23.2200 -41.9200 -0.4525 -0.4366
0.3564 0.4273 0.4255 0.2535 0.2812
0.2411 -2.5395 -40.5114
23.2400 -41.2100 -0.1513 -0.2127
0.5783 0.5197 0.4098 0.2369 0.2729
0.2388 -2.5405 -40.5141
23.2600 -40.3200 -0.0626 0.3474
0.7356 0.5861 0.3887 0.2193 0.2643
0.2365 -2.5414 -40.5169
23.2800 -39.1200 0.6008 0.7821
0.7958 0.6200 0.3622 0.2008 0.2555
0.2342 -2.5423 -40.5197
23.3000 -40.3400 -0.5435 0.8309
0.7350 0.6176 0.3304 0.1815 0.2464
0.2319 -2.5432 -40.5224
23.3200 -40.0400 0.3802 0.6125
0.5637 0.5816 0.2939 0.1615 0.2371
0.2295 -2.5441 -40.5252
23.3400 -41.2200 0.0388 0.1522
0.3224 0.5172 0.2531 0.1409 0.2276
0.2271 -2.5450 -40.5279
23.3600 -42.4700 -0.0835 -0.5223
0.0648 0.4306 0.2090 0.1198 0.2178
0.2247 -2.5459 -40.5307
23.3800 -42.8100 0.3892 -1.0933 -
0.1556 0.3277 0.1621 0.0982 0.2079
0.2223 -2.5467 -40.5335
23.4000 -44.3600 -0.7389 -1.0722 -
0.2972 0.2146 0.1133 0.0763 0.1977
0.2199 -2.5476 -40.5362
23.4200 -42.7800 0.2469 -0.2794 -
0.3550 0.0973 0.0632 0.0542 0.1874
0.2175 -2.5484 -40.5390
23.4400 -42.4000 -0.0698 0.7292 -
0.3551 -0.0188 0.0126 0.0319 0.1770
0.2150 -2.5492 -40.5417
23.4600 -41.8900 0.1950 1.1620 -
0.3242 -0.1280 -0.0377 0.0096 0.1664
0.2125 -2.5500 -40.5445
23.4800 -42.0400 0.5844 0.6542 -
0.2859 -0.2255 -0.0870 -0.0128 0.1556
0.2100 -2.5508 -40.5472

23.5000 -44.6500 -0.6880 -0.3745 -
0.2521 -0.3074 -0.1346 -0.0350 0.1447
0.2075 -2.5516 -40.5499
23.5200 -44.0900 0.4563 -0.9816 -
0.2291 -0.3708 -0.1797 -0.0570 0.1337
0.2050 -2.5524 -40.5527
23.5400 -44.6400 -0.3604 -0.6208 -
0.2184 -0.4144 -0.2217 -0.0787 0.1226
0.2025 -2.5531 -40.5554
23.5600 -44.2500 -0.7374 0.2415 -
0.2121 -0.4388 -0.2600 -0.1000 0.1114
0.2000 -2.5539 -40.5582
23.5800 -41.7800 1.3685 0.7573 -
0.1959 -0.4459 -0.2939 -0.1208 0.1001
0.1974 -2.5546 -40.5609
23.6000 -44.7300 -1.4208 0.5589 -
0.1598 -0.4382 -0.3229 -0.1410 0.0887
0.1949 -2.5553 -40.5636
23.6200 -44.6500 -0.8464 0.0887 -
0.1064 -0.4184 -0.3468 -0.1606 0.0773
0.1923 -2.5560 -40.5664
23.6400 -42.8400 1.3686 -0.3315 -
0.0403 -0.3892 -0.3650 -0.1794 0.0658
0.1897 -2.5567 -40.5691
23.6600 -43.0900 1.3906 -0.5382
0.0380 -0.3530 -0.3775 -0.1973 0.0543
0.1872 -2.5574 -40.5718
23.6800 -44.5700 -0.4856 -0.4951
0.1240 -0.3118 -0.3840 -0.2144 0.0427
0.1846 -2.5580 -40.5746
23.7000 -45.3000 -1.5660 -0.1693
0.2071 -0.2665 -0.3846 -0.2304 0.0312
0.1820 -2.5587 -40.5773
23.7200 -43.4200 -0.2529 0.3569
0.2695 -0.2176 -0.3796 -0.2454 0.0196
0.1794 -2.5593 -40.5800
23.7400 -41.5300 1.3502 0.6250
0.2795 -0.1652 -0.3695 -0.2593 0.0080
0.1768 -2.5599 -40.5827
23.7600 -44.1100 -0.9871 0.3053
0.2152 -0.1091 -0.3546 -0.2721 -0.0035
0.1742 -2.5605 -40.5855
23.7800 -43.9600 -0.1281 -0.2023
0.0913 -0.0497 -0.3357 -0.2837 -0.0150
0.1717 -2.5611 -40.5882
23.8000 -43.8700 0.3055 -0.4492 -
0.0619 0.0121 -0.3132 -0.2941 -0.0265
0.1691 -2.5617 -40.5909
23.8200 -44.1100 0.0558 -0.3987 -
0.2134 0.0749 -0.2878 -0.3034 -0.0379
0.1665 -2.5623 -40.5936

23.8400 -44.1300 -0.1387 -0.1434 -
0.3317 0.1375 -0.2601 -0.3114 -0.0493
0.1639 -2.5628 -40.5963
23.8600 -43.6700 -0.1066 0.2320 -
0.3896 0.1986 -0.2306 -0.3182 -0.0606
0.1613 -2.5634 -40.5991
23.8800 -43.1000 0.1957 0.4680 -
0.3639 0.2563 -0.2000 -0.3238 -0.0718
0.1587 -2.5639 -40.6018
23.9000 -43.5300 -0.1064 0.2504 -
0.2412 0.3074 -0.1686 -0.3282 -0.0829
0.1561 -2.5644 -40.6045
23.9200 -42.9400 0.7248 -0.3568 -
0.0453 0.3482 -0.1370 -0.3314 -0.0939
0.1536 -2.5649 -40.6072
23.9400 -44.4600 -0.8039 -0.6681
0.1729 0.3747 -0.1056 -0.3335 -0.1048
0.1510 -2.5654 -40.6099
23.9600 -43.8500 -0.6545 -0.2283
0.3594 0.3831 -0.0747 -0.3343 -0.1155
0.1484 -2.5659 -40.6126
23.9800 -40.9400 1.1252 0.5406
0.4572 0.3700 -0.0448 -0.3340 -0.1262
0.1459 -2.5664 -40.6153
24.0000 -42.6600 -0.6109 0.7096
0.4181 0.3340 -0.0162 -0.3325 -0.1367
0.1433 -2.5668 -40.6180
24.0200 -42.5100 0.4155 0.1542
0.2493 0.2775 0.0108 -0.3300 -0.1471
0.1408 -2.5673 -40.6207
24.0400 -44.0500 -0.4315 -0.4168
0.0262 0.2058 0.0359 -0.3264 -0.1573
0.1383 -2.5677 -40.6234
24.0600 -44.0800 -0.1241 -0.5541 -
0.1626 0.1248 0.0591 -0.3218 -0.1673
0.1357 -2.5681 -40.6261
24.0800 -44.2400 -0.1823 -0.2231 -
0.2541 0.0409 0.0803 -0.3162 -0.1772
0.1332 -2.5685 -40.6288
24.1000 -42.9300 0.6926 0.1018 -
0.2360 -0.0396 0.0993 -0.3096 -0.1870
0.1308 -2.5689 -40.6315
24.1200 -44.3000 -0.6755 0.0523 -
0.1399 -0.1117 0.1161 -0.3022 -0.1965
0.1283 -2.5692 -40.6342
24.1400 -43.0700 0.6116 0.0071 -
0.0418 -0.1708 0.1308 -0.2940 -0.2059
0.1258 -2.5696 -40.6369
24.1600 -44.1600 -0.7868 0.3174 -
0.0095 -0.2131 0.1434 -0.2850 -0.2151
0.1234 -2.5699 -40.6396

24.1800 -42.3000 0.9240 0.4808 -
0.0402 -0.2372 0.1540 -0.2754 -0.2241
0.1209 -2.5703 -40.6423
24.2000 -44.3400 -0.5977 0.0643 -
0.0964 -0.2440 0.1628 -0.2651 -0.2330
0.1185 -2.5706 -40.6450
24.2200 -44.5100 -0.2935 -0.4732 -
0.1258 -0.2361 0.1700 -0.2542 -0.2416
0.1161 -2.5709 -40.6476
24.2400 -44.3400 -0.0233 -0.6284 -
0.0864 -0.2163 0.1757 -0.2429 -0.2501
0.1137 -2.5712 -40.6503
24.2600 -43.5800 0.4705 -0.3329
0.0241 -0.1878 0.1799 -0.2311 -0.2583
0.1114 -2.5714 -40.6530
24.2800 -43.9100 -0.7148 0.2784
0.1577 -0.1534 0.1828 -0.2189 -0.2664
0.1090 -2.5717 -40.6557
24.3000 -41.7900 0.7998 0.6825
0.2457 -0.1156 0.1844 -0.2064 -0.2742
0.1067 -2.5719 -40.6584
24.3200 -43.6000 -0.6932 0.3884
0.2342 -0.0766 0.1846 -0.1935 -0.2818
0.1044 -2.5722 -40.6610
24.3400 -43.1400 0.4249 -0.1799
0.1377 -0.0382 0.1835 -0.1805 -0.2892
0.1022 -2.5724 -40.6637
24.3600 -44.0700 -0.1887 -0.5132
0.0016 -0.0012 0.1810 -0.1672 -0.2964
0.0999 -2.5726 -40.6664
24.3800 -43.9000 0.0814 -0.4768 -
0.1234 0.0344 0.1769 -0.1538 -0.3034
0.0977 -2.5728 -40.6691
24.4000 -44.2800 -0.7420 -0.0077 -
0.2004 0.0682 0.1713 -0.1402 -0.3102
0.0955 -2.5729 -40.6717
24.4200 -42.2700 0.6909 0.4580 -
0.2170 0.0995 0.1640 -0.1266 -0.3167
0.0933 -2.5731 -40.6744
24.4400 -42.0400 0.9630 0.4295 -
0.1757 0.1275 0.1550 -0.1130 -0.3230
0.0912 -2.5733 -40.6771
24.4600 -44.6400 -1.2408 -0.0142 -
0.0890 0.1506 0.1443 -0.0994 -0.3291
0.0891 -2.5734 -40.6797
24.4800 -43.0000 0.5959 -0.3296
0.0201 0.1678 0.1318 -0.0859 -0.3350
0.0870 -2.5735 -40.6824
24.5000 -42.9200 0.6508 -0.4195
0.1318 0.1782 0.1178 -0.0725 -0.3407
0.0850 -2.5736 -40.6850

24.5200 -43.7000 -0.1321 -0.4136
0.2244 0.1810 0.1022 -0.0593 -0.3461
0.0830 -2.5737 -40.6877
24.5400 -43.7600 -0.6257 -0.1683
0.2690 0.1758 0.0854 -0.0462 -0.3513
0.0810 -2.5738 -40.6904
24.5600 -42.7300 0.1149 0.3702
0.2458 0.1622 0.0675 -0.0335 -0.3562
0.0790 -2.5738 -40.6930
24.5800 -42.0300 0.4005 0.8309
0.1478 0.1403 0.0489 -0.0211 -0.3610
0.0771 -2.5739 -40.6957
24.6000 -43.2800 -0.4665 0.7049 -
0.0124 0.1103 0.0297 -0.0090 -0.3655
0.0752 -2.5739 -40.6983
24.6200 -42.7900 0.8764 -0.0963 -
0.1810 0.0731 0.0104 0.0026 -0.3698
0.0734 -2.5739 -40.7010
24.6400 -45.8000 -1.0625 -0.8331 -
0.2873 0.0298 -0.0086 0.0138 -0.3738
0.0716 -2.5739 -40.7036
24.6600 -43.6500 1.0717 -0.7955 -
0.2728 -0.0178 -0.0269 0.0245 -0.3777
0.0698 -2.5739 -40.7063
24.6800 -44.8000 -0.9104 -0.1701 -
0.1400 -0.0670 -0.0440 0.0346 -0.3813
0.0681 -2.5739 -40.7089
24.7000 -42.5000 0.5838 0.5509
0.0477 -0.1145 -0.0595 0.0442 -0.3846
0.0664 -2.5739 -40.7116
24.7200 -42.7300 0.1559 0.7871
0.2120 -0.1572 -0.0728 0.0532 -0.3878
0.0648 -2.5738 -40.7142
24.7400 -43.0400 0.0546 0.4460
0.2818 -0.1920 -0.0836 0.0615 -0.3907
0.0632 -2.5738 -40.7168
24.7600 -44.3200 -0.7283 0.0530
0.2343 -0.2162 -0.0914 0.0692 -0.3934
0.0616 -2.5737 -40.7195
24.7800 -43.2400 0.9101 -0.2155
0.0874 -0.2281 -0.0959 0.0763 -0.3958
0.0601 -2.5736 -40.7221
24.8000 -45.2600 -0.8877 -0.3468 -
0.1126 -0.2268 -0.0970 0.0826 -0.3981
0.0586 -2.5735 -40.7247
24.8200 -44.3000 0.0247 -0.1883 -
0.3104 -0.2119 -0.0945 0.0883 -0.4001
0.0572 -2.5733 -40.7274
24.8400 -43.6800 0.6830 -0.0185 -
0.4411 -0.1829 -0.0885 0.0933 -0.4019
0.0558 -2.5732 -40.7300

24.8600 -44.1300 0.2624 -0.1873 -
0.4531 -0.1406 -0.0791 0.0976 -0.4036
0.0545 -2.5730 -40.7326
24.8800 -44.9300 -0.3711 -0.4794 -
0.3354 -0.0877 -0.0667 0.1013 -0.4050
0.0532 -2.5729 -40.7353
24.9000 -44.7400 -0.5573 -0.4206 -
0.1082 -0.0288 -0.0515 0.1043 -0.4061
0.0520 -2.5727 -40.7379
24.9200 -43.4000 -0.1281 0.0789
0.1705 0.0309 -0.0339 0.1066 -0.4071
0.0508 -2.5725 -40.7405
24.9400 -41.8800 0.8376 0.4668
0.4163 0.0862 -0.0144 0.1084 -0.4079
0.0497 -2.5723 -40.7431
24.9600 -43.2200 -0.6829 0.3470
0.5528 0.1321 0.0065 0.1095 -0.4086
0.0486 -2.5720 -40.7458
24.9800 -42.6800 0.0120 0.0830
0.5486 0.1640 0.0285 0.1100 -0.4090
0.0475 -2.5718 -40.7484
25.0000 -42.4100 0.4480 0.0405
0.4032 0.1805 0.0509 0.1100 -0.4092
0.0466 -2.5715 -40.7510
25.0200 -43.5300 -0.4815 0.1830
0.1570 0.1832 0.0733 0.1094 -0.4093
0.0456 -2.5712 -40.7536
25.0400 -42.7400 0.4129 0.2042 -
0.1191 0.1752 0.0954 0.1083 -0.4092
0.0448 -2.5709 -40.7562
25.0600 -43.6500 -0.0674 -0.0436 -
0.3542 0.1596 0.1167 0.1066 -0.4090
0.0439 -2.5706 -40.7588
25.0800 -44.3300 -0.2180 -0.3449 -
0.4934 0.1393 0.1366 0.1045 -0.4085
0.0432 -2.5703 -40.7614
25.1000 -44.1100 0.1949 -0.4076 -
0.4994 0.1172 0.1549 0.1019 -0.4080
0.0425 -2.5700 -40.7640
25.1200 -44.3800 -0.3980 -0.1862 -
0.3752 0.0949 0.1710 0.0988 -0.4073
0.0418 -2.5696 -40.7667
25.1400 -43.0400 0.3638 0.0842 -
0.1681 0.0739 0.1847 0.0953 -0.4064
0.0412 -2.5692 -40.7693
25.1600 -42.7600 0.3564 0.1976
0.0527 0.0554 0.1957 0.0915 -0.4054
0.0407 -2.5689 -40.7719
25.1800 -43.1000 -0.2620 0.2129
0.2218 0.0407 0.2038 0.0872 -0.4043
0.0402 -2.5684 -40.7745

25.2000 -43.4100 -0.5090 0.2457
0.2988 0.0307 0.2086 0.0826 -0.4031
0.0397 -2.5680 -40.7771
25.2200 -42.3400 0.8189 0.1485
0.2795 0.0250 0.2101 0.0777 -0.4017
0.0394 -2.5676 -40.7797
25.2400 -44.1200 -0.7595 -0.1466
0.1975 0.0224 0.2081 0.0725 -0.4003
0.0390 -2.5671 -40.7822
25.2600 -43.0800 0.5775 -0.2685
0.1063 0.0212 0.2024 0.0670 -0.3987
0.0388 -2.5667 -40.7848
25.2800 -43.9000 -0.3781 -0.2010
0.0528 0.0202 0.1931 0.0614 -0.3971
0.0386 -2.5662 -40.7874
25.3000 -43.1200 0.5574 -0.2121
0.0477 0.0196 0.1803 0.0556 -0.3953
0.0384 -2.5657 -40.7900
25.3200 -44.0200 -0.4690 -0.1094
0.0607 0.0207 0.1641 0.0496 -0.3935
0.0384 -2.5652 -40.7926
25.3400 -43.4200 -0.3226 0.3215
0.0491 0.0248 0.1447 0.0436 -0.3915
0.0383 -2.5646 -40.7952
25.3600 -42.1900 0.6478 0.7327 -
0.0113 0.0335 0.1222 0.0375 -0.3895
0.0384 -2.5641 -40.7978
25.3800 -43.4800 -0.3123 0.5697 -
0.1239 0.0469 0.0970 0.0315 -0.3874
0.0385 -2.5635 -40.8004
25.4000 -43.6800 0.1868 -0.0593 -
0.2552 0.0646 0.0695 0.0254 -0.3852
0.0386 -2.5629 -40.8029
25.4200 -45.1000 -0.5232 -0.6349 -
0.3529 0.0853 0.0400 0.0194 -0.3829
0.0388 -2.5623 -40.8055
25.4400 -44.3800 0.4131 -0.8421 -
0.3650 0.1075 0.0089 0.0135 -0.3806
0.0391 -2.5617 -40.8081
25.4600 -45.3000 -0.7778 -0.5601 -
0.2660 0.1296 -0.0234 0.0077 -0.3782
0.0394 -2.5611 -40.8107
25.4800 -44.0900 -0.4041 0.0678 -
0.0839 0.1484 -0.0562 0.0021 -0.3757
0.0398 -2.5605 -40.8133
25.5000 -42.3600 0.4430 0.7101
0.1296 0.1606 -0.0892 -0.0033 -0.3732
0.0402 -2.5598 -40.8158
25.5200 -41.3500 1.2439 0.8469
0.3179 0.1627 -0.1219 -0.0085 -0.3706
0.0407 -2.5591 -40.8184

25.5400 -42.6600 0.3002 0.3093
0.4286 0.1520 -0.1535 -0.0134 -0.3679
0.0412 -2.5584 -40.8210
25.5600 -44.9800 -1.3041 -0.3073
0.4385 0.1265 -0.1837 -0.0180 -0.3652
0.0418 -2.5577 -40.8235
25.5800 -43.5900 0.3088 -0.3181
0.3398 0.0872 -0.2119 -0.0222 -0.3623
0.0424 -2.5570 -40.8261
25.6000 -43.1900 0.3792 0.1158
0.1574 0.0375 -0.2377 -0.0261 -0.3595
0.0431 -2.5562 -40.8287
25.6200 -43.0000 0.6714 0.2946 -
0.0571 -0.0184 -0.2605 -0.0296 -0.3565
0.0438 -2.5554 -40.8312
25.6400 -44.9200 -0.5090 -0.0020 -
0.2453 -0.0759 -0.2801 -0.0327 -0.3535
0.0446 -2.5546 -40.8338
25.6600 -44.5000 0.4434 -0.4329 -
0.3594 -0.1307 -0.2959 -0.0353 -0.3504
0.0454 -2.5538 -40.8363
25.6800 -46.1200 -1.0779 -0.4178 -
0.3711 -0.1788 -0.3078 -0.0375 -0.3473
0.0463 -2.5530 -40.8389
25.7000 -43.7300 0.9951 -0.0626 -
0.2850 -0.2182 -0.3153 -0.0392 -0.3440
0.0472 -2.5522 -40.8414
25.7200 -43.9000 0.4454 0.1111 -
0.1358 -0.2487 -0.3182 -0.0405 -0.3407
0.0481 -2.5513 -40.8440
25.7400 -45.2100 -1.0219 0.1258
0.0226 -0.2706 -0.3162 -0.0413 -0.3373
0.0491 -2.5504 -40.8465
25.7600 -43.3400 0.5275 0.2869
0.1349 -0.2845 -0.3090 -0.0416 -0.3338
0.0501 -2.5496 -40.8491
25.7800 -43.4400 0.2345 0.4911
0.1725 -0.2914 -0.2966 -0.0415 -0.3302
0.0512 -2.5486 -40.8516
25.8000 -44.1700 -0.2576 0.3905
0.1408 -0.2929 -0.2787 -0.0410 -0.3264
0.0523 -2.5477 -40.8542
25.8200 -43.9200 0.2784 -0.0442
0.0718 -0.2911 -0.2557 -0.0400 -0.3226
0.0534 -2.5468 -40.8567
25.8400 -45.1400 -0.4074 -0.4707
0.0077 -0.2878 -0.2275 -0.0387 -0.3187
0.0545 -2.5458 -40.8593
25.8600 -44.3600 0.4038 -0.6080 -
0.0147 -0.2846 -0.1946 -0.0371 -0.3147
0.0557 -2.5448 -40.8618

25.8800 -44.5700 -0.0315 -0.4234
0.0143 -0.2821 -0.1575 -0.0352 -0.3106
0.0569 -2.5438 -40.8643
25.9000 -44.5200 -0.5994 -0.0073
0.0734 -0.2791 -0.1166 -0.0331 -0.3063
0.0582 -2.5428 -40.8669
25.9200 -42.5700 0.8639 0.4357
0.1255 -0.2737 -0.0726 -0.0308 -0.3020
0.0594 -2.5418 -40.8694
25.9400 -43.2000 -0.0105 0.6533
0.1339 -0.2636 -0.0262 -0.0285 -0.2975
0.0607 -2.5407 -40.8719
25.9600 -44.3700 -1.1125 0.5324
0.0810 -0.2470 0.0219 -0.0261 -0.2929
0.0620 -2.5396 -40.8745
25.9800 -42.5600 1.2226 0.0654 -
0.0214 -0.2224 0.0711 -0.0237 -0.2881
0.0633 -2.5385 -40.8770
26.0000 -44.1500 0.1173 -0.4729 -
0.1323 -0.1889 0.1208 -0.0214 -0.2832
0.0647 -2.5374 -40.8795
26.0200 -45.6600 -1.2418 -0.6632 -
0.2070 -0.1458 0.1701 -0.0192 -0.2782
0.0660 -2.5363 -40.8821
26.0400 -43.4000 0.6033 -0.2625 -
0.2114 -0.0937 0.2186 -0.0172 -0.2730
0.0674 -2.5351 -40.8846
26.0600 -42.3900 0.8366 0.3105 -
0.1448 -0.0344 0.2655 -0.0154 -0.2677
0.0688 -2.5340 -40.8871
26.0800 -43.7600 -0.9845 0.4913 -
0.0232 0.0293 0.3100 -0.0139 -0.2623
0.0702 -2.5328 -40.8896
26.1000 -41.9400 0.9295 0.0430
0.1214 0.0946 0.3516 -0.0127 -0.2567
0.0716 -2.5316 -40.8921
26.1200 -44.1000 -0.8354 -0.4075
0.2428 0.1590 0.3894 -0.0119 -0.2509
0.0730 -2.5303 -40.8947
26.1400 -42.5700 0.3002 -0.2761
0.3013 0.2207 0.4228 -0.0115 -0.2450
0.0744 -2.5291 -40.8972
26.1600 -42.4400 -0.0981 0.1666
0.2793 0.2775 0.4513 -0.0117 -0.2390
0.0759 -2.5278 -40.8997
26.1800 -41.6700 0.6365 0.3788
0.1695 0.3276 0.4742 -0.0123 -0.2327
0.0773 -2.5265 -40.9022
26.2000 -43.0900 -0.6935 0.2577 -
0.0186 0.3695 0.4911 -0.0135 -0.2264
0.0788 -2.5252 -40.9047

26.2200 -42.2500 0.6995 -0.0505 -
0.2397 0.4021 0.5016 -0.0153 -0.2198
0.0802 -2.5239 -40.9072
26.2400 -44.1100 -0.6160 -0.2985 -
0.4264 0.4233 0.5053 -0.0177 -0.2131
0.0816 -2.5226 -40.9097
26.2600 -43.2500 0.1075 -0.2646 -
0.5117 0.4311 0.5018 -0.0207 -0.2062
0.0831 -2.5212 -40.9122
26.2800 -42.8900 0.1940 -0.0577 -
0.4614 0.4242 0.4914 -0.0243 -0.1991
0.0845 -2.5198 -40.9147
26.3000 -43.2000 -0.3274 0.1292 -
0.2879 0.4023 0.4740 -0.0284 -0.1919
0.0859 -2.5184 -40.9172
26.3200 -42.2000 0.4941 0.1313 -
0.0345 0.3659 0.4502 -0.0330 -0.1845
0.0874 -2.5170 -40.9197
26.3400 -42.7800 -0.1680 0.0567
0.2395 0.3156 0.4205 -0.0380 -0.1769
0.0888 -2.5155 -40.9222
26.3600 -42.8500 -0.4161 0.0779
0.4780 0.2520 0.3857 -0.0433 -0.1691
0.0902 -2.5141 -40.9247
26.3800 -41.7700 0.6218 0.1503
0.6393 0.1757 0.3463 -0.0490 -0.1612
0.0916 -2.5126 -40.9272
26.4000 -42.7300 -0.2055 0.0385
0.6967 0.0878 0.3031 -0.0549 -0.1531
0.0930 -2.5111 -40.9297
26.4200 -42.8600 -0.0271 -0.1448
0.6362 -0.0091 0.2568 -0.0610 -0.1448
0.0944 -2.5096 -40.9322
26.4400 -43.3900 -0.2220 -0.0801
0.4524 -0.1107 0.2081 -0.0673 -0.1364
0.0957 -2.5080 -40.9346
26.4600 -43.2500 -0.3231 0.2760
0.1658 -0.2108 0.1579 -0.0736 -0.1277
0.0971 -2.5065 -40.9371
26.4800 -42.7200 0.6649 0.5547 -
0.1725 -0.3034 0.1067 -0.0800 -0.1189
0.0984 -2.5049 -40.9396
26.5000 -44.7400 -0.5321 0.2586 -
0.4974 -0.3820 0.0553 -0.0863 -0.1099
0.0997 -2.5033 -40.9421
26.5200 -44.4500 0.7667 -0.4913 -
0.7329 -0.4405 0.0044 -0.0926 -0.1008
0.1010 -2.5016 -40.9446
26.5400 -46.5000 -0.8859 -0.8250 -
0.8003 -0.4729 -0.0454 -0.0988 -0.0914
0.1023 -2.5000 -40.9470

26.5600 -45.5600 -0.3933 -0.4106 -
0.6605 -0.4765 -0.0936 -0.1048 -0.0819
0.1036 -2.4983 -40.9495
26.5800 -43.3000 0.9288 0.2528 -
0.3706 -0.4540 -0.1399 -0.1106 -0.0722
0.1048 -2.4966 -40.9520
26.6000 -43.2100 0.3544 0.5266 -
0.0141 -0.4105 -0.1839 -0.1161 -0.0623
0.1060 -2.4949 -40.9545
26.6200 -44.2100 -0.8180 0.3863
0.3226 -0.3514 -0.2255 -0.1212 -0.0523
0.1072 -2.4932 -40.9569
26.6400 -43.0500 0.3427 0.1545
0.5587 -0.2821 -0.2644 -0.1260 -0.0421
0.1084 -2.4914 -40.9594
26.6600 -43.1000 0.1390 0.0912
0.6450 -0.2076 -0.3007 -0.1303 -0.0317
0.1095 -2.4897 -40.9619
26.6800 -43.6100 -0.3324 0.1678
0.5783 -0.1322 -0.3343 -0.1342 -0.0211
0.1106 -2.4879 -40.9643
26.7000 -43.1100 0.2421 0.1889
0.3935 -0.0590 -0.3653 -0.1374 -0.0103
0.1117 -2.4861 -40.9668
26.7200 -43.5000 0.1628 0.0161
0.1519 0.0103 -0.3935 -0.1401 0.0006
0.1127 -2.4842 -40.9692
26.7400 -44.5200 -0.4925 -0.2506 -
0.0800 0.0737 -0.4191 -0.1422 0.0117
0.1138 -2.4824 -40.9717
26.7600 -43.8000 0.5994 -0.3693 -
0.2515 0.1301 -0.4421 -0.1435 0.0230
0.1148 -2.4805 -40.9742
26.7800 -44.3700 -0.0943 -0.2274 -
0.3380 0.1786 -0.4627 -0.1442 0.0344
0.1157 -2.4786 -40.9766
26.8000 -44.7300 -0.7491 0.1235 -
0.3401 0.2195 -0.4810 -0.1441 0.0460
0.1167 -2.4767 -40.9791
26.8200 -42.8800 0.6555 0.4533 -
0.2732 0.2535 -0.4973 -0.1432 0.0577
0.1176 -2.4747 -40.9815
26.8400 -42.8000 0.5702 0.3954 -
0.1577 0.2818 -0.5116 -0.1415 0.0696
0.1184 -2.4728 -40.9840
26.8600 -44.6600 -0.9577 -0.0591 -
0.0180 0.3060 -0.5243 -0.1389 0.0817
0.1192 -2.4708 -40.9864
26.8800 -43.4700 0.4017 -0.5078
0.1125 0.3279 -0.5356 -0.1354 0.0938
0.1200 -2.4688 -40.9888

26.9000	-43.1500	0.7348	-0.5849	
0.2012	0.3490	-0.5456	-0.1311	0.1061
	0.1208	-2.4667	-40.9913	
26.9200	-44.2200	-0.8676	-0.1958	
0.2270	0.3705	-0.5543	-0.1258	0.1185
	0.1215	-2.4647	-40.9937	
26.9400	-43.1000	-0.2407	0.3968	
0.1876	0.3928	-0.5617	-0.1198	0.1310
	0.1222	-2.4626	-40.9962	
26.9600	-41.7800	0.8915	0.7550	
0.0891	0.4156	-0.5676	-0.1129	0.1437
	0.1228	-2.4605	-40.9986	
26.9800	-43.7100	-0.7467	0.4995	-
0.0496	0.4379	-0.5715	-0.1053	0.1563
	0.1234	-2.4584	-41.0010	
27.0000	-43.4800	0.1986	-0.1147	-
0.1856	0.4584	-0.5732	-0.0971	0.1691
	0.1239	-2.4563	-41.0035	
27.0200	-43.9700	0.1535	-0.6091	-
0.2664	0.4756	-0.5721	-0.0883	0.1819
	0.1245	-2.4541	-41.0059	
27.0400	-44.5700	-0.2273	-0.7225	-
0.2489	0.4881	-0.5674	-0.0790	0.1947
	0.1249	-2.4519	-41.0083	
27.0600	-43.9800	-0.1877	-0.3789	-
0.1298	0.4936	-0.5587	-0.0693	0.2076
	0.1253	-2.4497	-41.0108	
27.0800	-42.8300	0.0705	0.2586	
0.0498	0.4882	-0.5452	-0.0594	0.2205
	0.1257	-2.4475	-41.0132	
27.1000	-42.4400	-0.1467	0.6505	
0.2316	0.4678	-0.5264	-0.0494	0.2334
	0.1260	-2.4452	-41.0156	
27.1200	-41.3000	1.2444	0.2933	
0.3618	0.4282	-0.5017	-0.0393	0.2462
	0.1263	-2.4429	-41.0180	
27.1400	-44.3800	-1.3933	-0.2738	
0.4136	0.3659	-0.4708	-0.0293	0.2591
	0.1266	-2.4406	-41.0204	
27.1600	-41.7900	1.2923	-0.1931	
0.3873	0.2793	-0.4335	-0.0195	0.2719
	0.1267	-2.4383	-41.0229	
27.1800	-43.2800	-0.3886	0.0115	
0.3203	0.1693	-0.3898	-0.0100	0.2846
	0.1269	-2.4360	-41.0253	
27.2000	-43.3800	-0.2899	-0.0085	
0.2563	0.0388	-0.3396	-0.0008	0.2973
	0.1270	-2.4336	-41.0277	
27.2200	-43.1800	-0.0425	-0.0196	
0.2147	-0.1085	-0.2834	0.0079	0.3099
	0.1270	-2.4312	-41.0301	

27.2400 -43.2100 -0.0508 0.0338
0.1954 -0.2665 -0.2214 0.0160 0.3224
0.1270 -2.4288 -41.0325
27.2600 -43.0500 0.1556 0.0916
0.1821 -0.4282 -0.1541 0.0236 0.3347
0.1269 -2.4264 -41.0349
27.2800 -43.4500 -0.1898 0.1151
0.1518 -0.5851 -0.0816 0.0305 0.3470
0.1268 -2.4239 -41.0373
27.3000 -43.1100 0.4796 0.0251
0.0840 -0.7286 -0.0045 0.0369 0.3591
0.1266 -2.4215 -41.0397
27.3200 -44.5900 -0.6792 -0.1153 -
0.0369 -0.8502 0.0770 0.0426 0.3711
0.1263 -2.4190 -41.0421
27.3400 -43.2500 0.5698 -0.0011 -
0.2182 -0.9425 0.1622 0.0476 0.3829
0.1261 -2.4164 -41.0445
27.3600 -44.3800 -0.4663 0.2071 -
0.4511 -0.9999 0.2508 0.0519 0.3945
0.1257 -2.4139 -41.0469
27.3800 -43.6800 0.4401 0.1268 -
0.7066 -1.0184 0.3421 0.0556 0.4059
0.1253 -2.4113 -41.0493
27.4000 -44.8500 -0.2949 -0.1406 -
0.9397 -0.9949 0.4351 0.0586 0.4171
0.1248 -2.4087 -41.0517
27.4200 -44.9100 -0.2761 -0.2847 -
1.1022 -0.9271 0.5288 0.0608 0.4281
0.1243 -2.4061 -41.0541
27.4400 -43.9400 0.5818 -0.2160 -
1.1620 -0.8150 0.6218 0.0624 0.4388
0.1237 -2.4035 -41.0565
27.4600 -44.4800 -0.6316 0.0115 -
1.1158 -0.6622 0.7127 0.0633 0.4493
0.1230 -2.4008 -41.0589
27.4800 -42.9400 0.3267 0.2115 -
0.9860 -0.4753 0.7999 0.0635 0.4596
0.1223 -2.3981 -41.0613
27.5000 -42.5100 0.3676 0.2030 -
0.7994 -0.2624 0.8820 0.0629 0.4695
0.1215 -2.3954 -41.0637
27.5200 -43.0200 -0.5629 0.0579 -
0.5779 -0.0324 0.9574 0.0617 0.4792
0.1207 -2.3927 -41.0661
27.5400 -41.7000 0.1633 0.0142 -
0.3400 0.2052 1.0247 0.0597 0.4886
0.1198 -2.3899 -41.0684
27.5600 -41.0700 0.3479 0.0330 -
0.1010 0.4404 1.0822 0.0569 0.4976
0.1188 -2.3871 -41.0708

27.5800	-40.9400	-0.0506	-0.0067	
0.1349	0.6628	1.1285	0.0535	0.5064
	0.1177	-2.3843	-41.0732	
27.6000	-40.9800	-0.6263	-0.0077	
0.3753	0.8617	1.1620	0.0492	0.5148
	0.1166	-2.3815	-41.0756	
27.6200	-39.3900	0.6022	-0.0263	
0.6337	1.0262	1.1813	0.0442	0.5229
	0.1154	-2.3787	-41.0779	
27.6400	-39.4200	0.5180	-0.2826	
0.9119	1.1463	1.1848	0.0385	0.5306
	0.1142	-2.3758	-41.0803	
27.6600	-40.2900	-0.5005	-0.6220	
1.1702	1.2131	1.1710	0.0320	0.5380
	0.1129	-2.3729	-41.0827	
27.6800	-40.4100	-0.7762	-0.5573	
1.3489	1.2187	1.1387	0.0249	0.5450
	0.1115	-2.3700	-41.0850	
27.7000	-38.8500	0.0739	0.1310	
1.3931	1.1583	1.0879	0.0172	0.5516
	0.1100	-2.3670	-41.0874	
27.7200	-38.2200	0.1478	0.9961	
1.2565	1.0339	1.0200	0.0090	0.5578
	0.1085	-2.3641	-41.0898	
27.7400	-38.3700	0.5409	1.2190	
0.9058	0.8556	0.9366	0.0005	0.5636
	0.1069	-2.3611	-41.0921	
27.7600	-39.4500	1.0232	0.4257	
0.3804	0.6374	0.8395	-0.0083	0.5691
	0.1052	-2.3581	-41.0945	
27.7800	-43.7800	-1.1472	-0.7903	-
0.1937	0.3937	0.7305	-0.0173	0.5741
	0.1034	-2.3550	-41.0969	
27.8000	-43.2500	0.6393	-1.2171	-
0.6737	0.1392	0.6114	-0.0264	0.5787
	0.1016	-2.3520	-41.0992	
27.8200	-44.2600	-0.5226	-0.5030	-
0.9457	-0.1118	0.4839	-0.0354	0.5829
	0.0996	-2.3489	-41.1016	
27.8400	-44.1500	-0.7532	0.4172	-
0.9840	-0.3456	0.3498	-0.0443	0.5867
	0.0976	-2.3458	-41.1039	
27.8600	-41.9600	1.3230	0.6535	-
0.8056	-0.5523	0.2109	-0.0530	0.5901
	0.0956	-2.3427	-41.1063	
27.8800	-44.8900	-1.1446	0.1586	-
0.4865	-0.7252	0.0690	-0.0613	0.5930
	0.0934	-2.3395	-41.1086	
27.9000	-44.5700	-0.4553	-0.1635	-
0.1500	-0.8575	-0.0741	-0.0692	0.5954
	0.0912	-2.3363	-41.1110	

27.9200 -43.2100 0.7602 -0.0154
0.0980 -0.9435 -0.2169 -0.0765 0.5975
0.0889 -2.3331 -41.1133
27.9400 -43.7000 0.0669 0.2650
0.2145 -0.9799 -0.3574 -0.0833 0.5990
0.0865 -2.3299 -41.1156
27.9600 -43.8400 -0.0644 0.2449
0.2015 -0.9693 -0.4941 -0.0893 0.6001
0.0840 -2.3266 -41.1180
27.9800 -44.0400 0.1680 -0.0353
0.1034 -0.9195 -0.6251 -0.0944 0.6008
0.0815 -2.3234 -41.1203
28.0000 -45.0000 -0.2648 -0.2135 -
0.0139 -0.8420 -0.7488 -0.0986 0.6009
0.0789 -2.3201 -41.1227
28.0200 -44.9300 -0.3324 -0.1177 -
0.0828 -0.7484 -0.8636 -0.1018 0.6006
0.0762 -2.3168 -41.1250
28.0400 -44.0300 0.5941 0.0170 -
0.0674 -0.6493 -0.9677 -0.1038 0.5999
0.0734 -2.3134 -41.1273
28.0600 -44.7600 -0.1493 -0.1234
0.0281 -0.5524 -1.0598 -0.1046 0.5986
0.0705 -2.3101 -41.1297
28.0800 -44.9600 -0.3865 -0.1988
0.1457 -0.4628 -1.1387 -0.1040 0.5969
0.0676 -2.3067 -41.1320
28.1000 -43.7500 0.3596 0.1537
0.2096 -0.3829 -1.2034 -0.1019 0.5947
0.0646 -2.3033 -41.1343
28.1200 -43.9500 -0.2397 0.5170
0.1758 -0.3144 -1.2535 -0.0982 0.5920
0.0615 -2.2998 -41.1366
28.1400 -43.1100 1.1860 0.0667
0.0399 -0.2578 -1.2886 -0.0929 0.5889
0.0583 -2.2964 -41.1390
28.1600 -46.3100 -1.3779 -0.5279 -
0.1204 -0.2132 -1.3086 -0.0858 0.5852
0.0551 -2.2929 -41.1413
28.1800 -43.6600 1.1496 -0.2406 -
0.2193 -0.1802 -1.3136 -0.0770 0.5810
0.0518 -2.2894 -41.1436
28.2000 -43.6200 0.6497 0.1740 -
0.2203 -0.1579 -1.3037 -0.0664 0.5764
0.0484 -2.2859 -41.1459
28.2200 -44.7900 -0.4323 0.1238 -
0.1257 -0.1474 -1.2789 -0.0541 0.5713
0.0450 -2.2823 -41.1482
28.2400 -44.6000 -0.0854 -0.1141
0.0165 -0.1500 -1.2392 -0.0402 0.5657
0.0415 -2.2787 -41.1505

28.2600 -44.8800 -0.9565 0.0807
0.1475 -0.1669 -1.1849 -0.0249 0.5596
0.0379 -2.2751 -41.1529
28.2800 -43.6800 -0.2128 0.3721
0.2370 -0.1996 -1.1164 -0.0082 0.5531
0.0342 -2.2715 -41.1552
28.3000 -42.0300 1.5089 0.2744
0.2744 -0.2484 -1.0348 0.0096 0.5461
0.0305 -2.2679 -41.1575
28.3200 -45.5900 -1.6017 -0.2433
0.2646 -0.3124 -0.9410 0.0284 0.5387
0.0268 -2.2642 -41.1598
28.3400 -44.6100 -0.3009 -0.4459
0.2218 -0.3882 -0.8365 0.0481 0.5308
0.0229 -2.2605 -41.1621
28.3600 -42.7200 1.3530 -0.2530
0.1576 -0.4708 -0.7226 0.0685 0.5224
0.0190 -2.2568 -41.1644
28.3800 -44.8800 -1.1057 0.1236
0.0687 -0.5531 -0.6009 0.0894 0.5136
0.0151 -2.2531 -41.1667
28.4000 -42.9100 0.5987 0.3481 -
0.0474 -0.6275 -0.4730 0.1107 0.5045
0.0111 -2.2493 -41.1690
28.4200 -43.3900 0.2407 0.3304 -
0.1899 -0.6859 -0.3405 0.1323 0.4948
0.0070 -2.2455 -41.1713
28.4400 -44.0700 -0.0569 0.0139 -
0.3480 -0.7212 -0.2049 0.1540 0.4848
0.0029 -2.2417 -41.1736
28.4600 -44.3800 0.0864 -0.3450 -
0.4994 -0.7266 -0.0677 0.1757 0.4744
-0.0013 -2.2379 -41.1759
28.4800 -45.0400 -0.8063 -0.3034 -
0.6143 -0.6970 0.0698 0.1971 0.4636
-0.0055 -2.2341 -41.1782
28.5000 -43.3200 0.3419 0.1396 -
0.6634 -0.6293 0.2061 0.2182 0.4524
-0.0098 -2.2302 -41.1804
28.5200 -42.3900 0.8350 0.3501 -
0.6264 -0.5241 0.3398 0.2388 0.4409
-0.0141 -2.2263 -41.1827
28.5400 -43.9400 -0.6805 -0.0093 -
0.4971 -0.3848 0.4696 0.2588 0.4290
-0.0184 -2.2224 -41.1850
28.5600 -43.1200 0.1363 -0.4332 -
0.2960 -0.2179 0.5940 0.2780 0.4167
-0.0229 -2.2185 -41.1873
28.5800 -42.9700 -0.5095 -0.2850 -
0.0639 -0.0308 0.7117 0.2963 0.4041
-0.0273 -2.2145 -41.1896

28.6000 -40.9000 0.5043 0.2097
0.1507 0.1691 0.8213 0.3134 0.3912
-0.0318 -2.2105 -41.1918
28.6200 -40.7400 -0.0047 0.5101
0.3020 0.3739 0.9216 0.3294 0.3779
-0.0363 -2.2065 -41.1941
28.6400 -40.5300 -0.3151 0.4925
0.3604 0.5756 1.0111 0.3439 0.3644
-0.0409 -2.2025 -41.1964
28.6600 -40.2200 0.0947 0.2713
0.3374 0.7647 1.0884 0.3570 0.3505
-0.0455 -2.1984 -41.1987
28.6800 -40.1600 0.3047 -0.0873
0.2803 0.9312 1.1523 0.3683 0.3364
-0.0502 -2.1944 -41.2009
28.7000 -41.1100 -0.5001 -0.4541
0.2500 1.0647 1.2015 0.3779 0.3219
-0.0549 -2.1903 -41.2032
28.7200 -40.1300 0.6098 -0.6707
0.2938 1.1567 1.2345 0.3855 0.3072
-0.0596 -2.1862 -41.2055
28.7400 -40.7900 -0.3740 -0.6227
0.4238 1.2021 1.2503 0.3910 0.2922
-0.0644 -2.1820 -41.2077
28.7600 -40.4800 -0.5452 -0.2208
0.6044 1.1992 1.2482 0.3944 0.2770
-0.0691 -2.1779 -41.2100
28.7800 -38.6100 0.7001 0.3513
0.7772 1.1475 1.2285 0.3956 0.2616
-0.0740 -2.1737 -41.2123
28.8000 -39.1000 -0.0912 0.6952
0.8810 1.0471 1.1927 0.3946 0.2459
-0.0788 -2.1695 -41.2145
28.8200 -39.6000 -0.5166 0.7495
0.8603 0.9011 1.1422 0.3915 0.2300
-0.0837 -2.1652 -41.2168
28.8400 -40.0500 -0.3021 0.6109
0.6929 0.7169 1.0788 0.3865 0.2140
-0.0886 -2.1610 -41.2190
28.8600 -40.0100 0.8118 0.2455
0.4126 0.5046 1.0039 0.3795 0.1977
-0.0935 -2.1567 -41.2213
28.8800 -42.0800 -0.1813 -0.2599
0.0775 0.2754 0.9192 0.3707 0.1813
-0.0984 -2.1524 -41.2235
28.9000 -43.8700 -0.9576 -0.5942 -
0.2511 0.0404 0.8264 0.3601 0.1647
-0.1034 -2.1481 -41.2258
28.9200 -43.5800 -0.0476 -0.5058 -
0.5166 -0.1891 0.7270 0.3479 0.1480
-0.1083 -2.1438 -41.2280

28.9400 -43.0900 0.5947 -0.2564 -
0.6847 -0.4021 0.6227 0.3341 0.1312
-0.1133 -2.1394 -41.2303
28.9600 -43.3200 0.7420 -0.2494 -
0.7418 -0.5890 0.5151 0.3187 0.1143
-0.1183 -2.1350 -41.2325
28.9800 -45.3300 -0.9279 -0.3531 -
0.6988 -0.7422 0.4057 0.3019 0.0973
-0.1233 -2.1306 -41.2348
29.0000 -44.6100 -0.2243 -0.1617 -
0.5799 -0.8572 0.2962 0.2838 0.0802
-0.1284 -2.1262 -41.2370
29.0200 -43.9800 0.1390 0.3094 -
0.4154 -0.9318 0.1880 0.2644 0.0631
-0.1334 -2.1218 -41.2392
29.0400 -43.3900 0.4402 0.5661 -
0.2299 -0.9658 0.0825 0.2437 0.0459
-0.1385 -2.1173 -41.2415
29.0600 -43.7800 0.2639 0.2423 -
0.0466 -0.9604 -0.0192 0.2220 0.0287
-0.1435 -2.1128 -41.2437
29.0800 -45.1400 -0.6594 -0.3221
0.1148 -0.9183 -0.1161 0.1993 0.0115
-0.1486 -2.1083 -41.2459
29.1000 -43.9800 0.5484 -0.4433
0.2411 -0.8433 -0.2076 0.1756 -0.0057
-0.1537 -2.1038 -41.2482
29.1200 -44.8100 -0.7069 -0.0407
0.3256 -0.7401 -0.2931 0.1510 -0.0229
-0.1588 -2.0992 -41.2504
29.1400 -42.9400 0.8648 0.2643
0.3555 -0.6134 -0.3722 0.1257 -0.0400
-0.1638 -2.0947 -41.2526
29.1600 -43.7900 0.1791 0.2508
0.3179 -0.4680 -0.4444 0.0997 -0.0571
-0.1689 -2.0901 -41.2548
29.1800 -45.2900 -1.2510 0.1508
0.2195 -0.3097 -0.5095 0.0730 -0.0742
-0.1740 -2.0855 -41.2570
29.2000 -43.8000 0.1749 0.1568
0.0780 -0.1452 -0.5673 0.0459 -0.0912
-0.1791 -2.0808 -41.2593
29.2200 -42.9000 1.3010 0.0299 -
0.0732 0.0185 -0.6174 0.0183 -0.1081
-0.1841 -2.0762 -41.2615
29.2400 -45.8500 -1.2552 -0.3155 -
0.1890 0.1743 -0.6599 -0.0095 -0.1248
-0.1892 -2.0715 -41.2637
29.2600 -44.0300 0.6505 -0.3825 -
0.2359 0.3157 -0.6945 -0.0377 -0.1415
-0.1943 -2.0668 -41.2659

29.2800 -43.6800 0.5260 -0.0128 -
0.1999 0.4370 -0.7209 -0.0659 -0.1581
-0.1993 -2.0621 -41.2681
29.3000 -44.5000 -0.8311 0.3751 -
0.0947 0.5334 -0.7392 -0.0943 -0.1745
-0.2044 -2.0574 -41.2703
29.3200 -42.8100 0.9642 0.1792
0.0466 0.6015 -0.7490 -0.1226 -0.1907
-0.2094 -2.0526 -41.2725
29.3400 -44.9300 -0.8859 -0.3017
0.1823 0.6388 -0.7506 -0.1507 -0.2067
-0.2144 -2.0478 -41.2747
29.3600 -43.8200 0.1376 -0.2323
0.2719 0.6434 -0.7438 -0.1787 -0.2226
-0.2194 -2.0430 -41.2769
29.3800 -43.2100 0.4340 0.1895
0.2920 0.6151 -0.7293 -0.2062 -0.2383
-0.2244 -2.0382 -41.2791
29.4000 -43.3400 0.2776 0.3458
0.2397 0.5571 -0.7075 -0.2333 -0.2537
-0.2294 -2.0333 -41.2813
29.4200 -43.9400 0.0504 0.1416
0.1337 0.4747 -0.6791 -0.2599 -0.2690
-0.2343 -2.0285 -41.2835
29.4400 -45.3500 -0.9033 -0.0741
0.0060 0.3743 -0.6450 -0.2858 -0.2839
-0.2393 -2.0236 -41.2857
29.4600 -44.6500 0.1673 -0.1404 -
0.1095 0.2628 -0.6059 -0.3109 -0.2987
-0.2442 -2.0187 -41.2879
29.4800 -43.9100 1.0918 -0.1846 -
0.1829 0.1473 -0.5627 -0.3351 -0.3131
-0.2491 -2.0138 -41.2901
29.5000 -46.3900 -1.2292 -0.2462 -
0.1991 0.0346 -0.5162 -0.3584 -0.3273
-0.2539 -2.0088 -41.2923
29.5200 -44.4400 0.6022 -0.0820 -
0.1669 -0.0696 -0.4671 -0.3807 -0.3411
-0.2587 -2.0039 -41.2945
29.5400 -44.0400 0.7597 0.1243 -
0.1074 -0.1615 -0.4162 -0.4018 -0.3547
-0.2636 -1.9989 -41.2967
29.5600 -44.6300 0.3455 0.0522 -
0.0440 -0.2383 -0.3641 -0.4217 -0.3679
-0.2683 -1.9939 -41.2989
29.5800 -45.8700 -0.6611 -0.1210
0.0096 -0.2980 -0.3116 -0.4403 -0.3808
-0.2731 -1.9888 -41.3010
29.6000 -46.1600 -1.1814 -0.0403
0.0427 -0.3389 -0.2594 -0.4576 -0.3933
-0.2778 -1.9838 -41.3032

29.6200 -44.5100 -0.0228 0.3375
0.0482 -0.3604 -0.2082 -0.4735 -0.4055
-0.2825 -1.9787 -41.3054
29.6400 -42.9600 1.4916 0.5816
0.0257 -0.3627 -0.1586 -0.4880 -0.4173
-0.2871 -1.9736 -41.3076
29.6600 -44.8900 -0.3074 0.3108 -
0.0160 -0.3465 -0.1110 -0.5009 -0.4287
-0.2917 -1.9685 -41.3097
29.6800 -46.4500 -1.1919 -0.1899 -
0.0559 -0.3135 -0.0661 -0.5121 -0.4397
-0.2963 -1.9634 -41.3119
29.7000 -44.2200 1.0368 -0.4940 -
0.0724 -0.2659 -0.0241 -0.5217 -0.4502
-0.3008 -1.9583 -41.3141
29.7200 -46.1400 -0.8039 -0.4508 -
0.0533 -0.2064 0.0145 -0.5296 -0.4604
-0.3053 -1.9531 -41.3162
29.7400 -44.5500 0.3154 -0.1240 -
0.0085 -0.1382 0.0495 -0.5357 -0.4701
-0.3098 -1.9479 -41.3184
29.7600 -44.4300 -0.2073 0.3534
0.0388 -0.0645 0.0807 -0.5398 -0.4794
-0.3142 -1.9427 -41.3206
29.7800 -43.5500 0.4280 0.5242
0.0629 0.0111 0.1077 -0.5421 -0.4882
-0.3185 -1.9375 -41.3227
29.8000 -44.0400 0.1380 0.2323
0.0507 0.0846 0.1302 -0.5424 -0.4965
-0.3228 -1.9322 -41.3249
29.8200 -45.2100 -0.6270 -0.1646
0.0119 0.1518 0.1479 -0.5408 -0.5044
-0.3271 -1.9270 -41.3270
29.8400 -44.5200 0.1108 -0.2628 -
0.0335 0.2087 0.1607 -0.5371 -0.5118
-0.3313 -1.9217 -41.3292
29.8600 -43.7300 0.5333 -0.1399 -
0.0593 0.2517 0.1684 -0.5316 -0.5187
-0.3355 -1.9164 -41.3313
29.8800 -44.7200 -0.3940 -0.0674 -
0.0457 0.2787 0.1709 -0.5241 -0.5252
-0.3396 -1.9110 -41.3335
29.9000 -44.4500 -0.1192 -0.1036
0.0113 0.2891 0.1683 -0.5147 -0.5311
-0.3436 -1.9057 -41.3356
29.9200 -43.7200 0.4817 -0.1148
0.0944 0.2829 0.1607 -0.5035 -0.5366
-0.3476 -1.9003 -41.3378
29.9400 -44.6200 -0.5752 0.0071
0.1736 0.2609 0.1487 -0.4906 -0.5417
-0.3516 -1.8949 -41.3399

29.9600 -44.0900 -0.2276 0.2230
0.2147 0.2241 0.1326 -0.4761 -0.5462
-0.3555 -1.8895 -41.3421
29.9800 -43.0000 0.7679 0.3371
0.1930 0.1749 0.1129 -0.4600 -0.5503
-0.3593 -1.8841 -41.3442
30.0000 -43.6600 0.4974 0.1762
0.1032 0.1168 0.0904 -0.4424 -0.5539
-0.3631 -1.8787 -41.3464
30.0200 -45.5100 -0.8892 -0.0925 -
0.0283 0.0546 0.0655 -0.4235 -0.5571
-0.3668 -1.8732 -41.3485
30.0400 -45.7000 -0.8673 -0.1711 -
0.1577 -0.0068 0.0391 -0.4032 -0.5598
-0.3704 -1.8678 -41.3506
30.0600 -43.8600 1.1372 -0.1077 -
0.2396 -0.0626 0.0117 -0.3818 -0.5621
-0.3740 -1.8623 -41.3528
30.0800 -44.9500 0.0267 -0.1489 -
0.2461 -0.1090 -0.0160 -0.3593 -0.5640
-0.3775 -1.8567 -41.3549
30.1000 -46.2400 -0.9389 -0.2635 -
0.1790 -0.1437 -0.0432 -0.3358 -0.5654
-0.3809 -1.8512 -41.3570
30.1200 -44.2000 0.8107 -0.2402 -
0.0678 -0.1655 -0.0692 -0.3115 -0.5663
-0.3843 -1.8457 -41.3591
30.1400 -45.0500 -0.4410 0.1103
0.0416 -0.1743 -0.0933 -0.2863 -0.5669
-0.3876 -1.8401 -41.3613
30.1600 -44.5800 -0.5121 0.4728
0.1047 -0.1711 -0.1146 -0.2604 -0.5670
-0.3909 -1.8345 -41.3634
30.1800 -43.0500 0.9895 0.4848
0.0985 -0.1582 -0.1325 -0.2340 -0.5667
-0.3941 -1.8289 -41.3655
30.2000 -45.4300 -0.9526 0.0761
0.0337 -0.1391 -0.1464 -0.2070 -0.5660
-0.3972 -1.8233 -41.3676
30.2200 -44.5600 0.3338 -0.2664 -
0.0533 -0.1176 -0.1557 -0.1797 -0.5649
-0.4002 -1.8176 -41.3697
30.2400 -44.6200 0.4987 -0.4091 -
0.1130 -0.0975 -0.1598 -0.1520 -0.5634
-0.4032 -1.8119 -41.3718
30.2600 -45.2800 -0.2778 -0.4812 -
0.1011 -0.0822 -0.1584 -0.1241 -0.5614
-0.4061 -1.8063 -41.3740
30.2800 -44.7500 0.1059 -0.4761 -
0.0070 -0.0745 -0.1513 -0.0961 -0.5591
-0.4089 -1.8006 -41.3761

30.3000	-44.9300	-0.3141	-0.2630	
0.1402	-0.0753	-0.1385	-0.0680	-0.5564
	-0.4116	-1.7948	-41.3782	
30.3200	-43.4300	0.6354	0.0906	
0.2835	-0.0852	-0.1202	-0.0400	-0.5534
	-0.4143	-1.7891	-41.3803	
30.3400	-44.3200	-0.8653	0.5373	
0.3608	-0.1038	-0.0967	-0.0122	-0.5499
	-0.4169	-1.7834	-41.3824	
30.3600	-42.2500	0.8403	0.8048	
0.3303	-0.1298	-0.0684	0.0153	-0.5461
	-0.4195	-1.7776	-41.3845	
30.3800	-43.9300	-0.3019	0.4651	
0.1954	-0.1596	-0.0361	0.0424	-0.5418
	-0.4219	-1.7718	-41.3866	
30.4000	-44.7500	-0.2742	-0.1918	
0.0052	-0.1887	-0.0005	0.0690	-0.5373
	-0.4243	-1.7660	-41.3887	
30.4200	-44.9300	0.1366	-0.6431	-
0.1739	-0.2126	0.0377	0.0949	-0.5323
	-0.4266	-1.7602	-41.3908	
30.4400	-45.2300	-0.2804	-0.6563	-
0.2844	-0.2270	0.0776	0.1200	-0.5270
	-0.4289	-1.7543	-41.3929	
30.4600	-44.7400	-0.0667	-0.2935	-
0.3089	-0.2275	0.1184	0.1442	-0.5214
	-0.4310	-1.7485	-41.3950	
30.4800	-43.9800	0.2042	0.1747	-
0.2655	-0.2115	0.1592	0.1673	-0.5154
	-0.4331	-1.7426	-41.3971	
30.5000	-43.4900	0.1023	0.3940	-
0.1871	-0.1776	0.1992	0.1893	-0.5091
	-0.4351	-1.7367	-41.3991	
30.5200	-43.4600	0.1912	0.3062	-
0.1172	-0.1256	0.2377	0.2099	-0.5024
	-0.4371	-1.7308	-41.4012	
30.5400	-44.2400	-0.6826	0.2078	-
0.0901	-0.0568	0.2738	0.2291	-0.4955
	-0.4389	-1.7249	-41.4033	
30.5600	-42.6800	0.8076	0.1310	-
0.0993	0.0257	0.3070	0.2468	-0.4881
	-0.4407	-1.7189	-41.4054	
30.5800	-44.2300	-0.6576	-0.1469	-
0.1034	0.1163	0.3364	0.2627	-0.4805
	-0.4424	-1.7129	-41.4075	
30.6000	-43.3000	0.2694	-0.3737	-
0.0588	0.2078	0.3614	0.2769	-0.4726
	-0.4441	-1.7070	-41.4095	
30.6200	-43.8000	-0.7798	-0.0911	
0.0520	0.2926	0.3813	0.2891	-0.4644
	-0.4456	-1.7010	-41.4116	

30.6400 -41.6000 0.8195 0.3176
0.1987 0.3633 0.3954 0.2993 -0.4558
-0.4471 -1.6950 -41.4137
30.6600 -42.4200 0.0696 0.2282
0.3324 0.4134 0.4032 0.3073 -0.4470
-0.4485 -1.6889 -41.4158
30.6800 -43.3800 -0.7802 -0.1361
0.4087 0.4376 0.4041 0.3131 -0.4379
-0.4499 -1.6829 -41.4178
30.7000 -42.2200 0.3653 -0.1849
0.3980 0.4322 0.3977 0.3166 -0.4285
-0.4511 -1.6768 -41.4199
30.7200 -42.7100 -0.2922 0.2033
0.3050 0.3968 0.3840 0.3177 -0.4189
-0.4523 -1.6707 -41.4220
30.7400 -42.0400 0.1475 0.4650
0.1706 0.3346 0.3637 0.3166 -0.4090
-0.4535 -1.6646 -41.4240
30.7600 -42.0100 0.8353 0.1746
0.0419 0.2506 0.3374 0.3133 -0.3989
-0.4545 -1.6585 -41.4261
30.7800 -44.0200 -0.4434 -0.4351 -
0.0452 0.1510 0.3062 0.3080 -0.3885
-0.4555 -1.6524 -41.4281
30.8000 -45.0600 -0.9586 -0.7236 -
0.0679 0.0429 0.2709 0.3006 -0.3779
-0.4564 -1.6463 -41.4302
30.8200 -42.8300 0.9106 -0.3484 -
0.0275 -0.0656 0.2326 0.2914 -0.3671
-0.4572 -1.6401 -41.4323
30.8400 -43.9000 -0.6182 0.2864
0.0298 -0.1657 0.1923 0.2804 -0.3561
-0.4579 -1.6339 -41.4343
30.8600 -42.4500 0.4851 0.6684
0.0381 -0.2489 0.1509 0.2677 -0.3448
-0.4586 -1.6277 -41.4364
30.8800 -43.2200 -0.0099 0.6184 -
0.0387 -0.3088 0.1094 0.2535 -0.3334
-0.4592 -1.6215 -41.4384
30.9000 -43.7200 0.2970 0.0642 -
0.1756 -0.3420 0.0687 0.2379 -0.3219
-0.4598 -1.6153 -41.4404
30.9200 -44.7600 -0.0254 -0.5410 -
0.3093 -0.3475 0.0296 0.2209 -0.3101
-0.4602 -1.6091 -41.4425
30.9400 -45.4000 -0.6883 -0.5518 -
0.3686 -0.3258 -0.0071 0.2028 -0.2982
-0.4606 -1.6028 -41.4445
30.9600 -43.4500 0.8053 0.0482 -
0.3087 -0.2792 -0.0411 0.1837 -0.2862
-0.4609 -1.5966 -41.4466

30.9800 -44.3800 -0.6164 0.2549 -
0.1399 -0.2136 -0.0720 0.1636 -0.2741
-0.4611 -1.5903 -41.4486
31.0000 -42.9800 0.9496 -0.2235
0.0730 -0.1375 -0.0995 0.1428 -0.2618
-0.4613 -1.5840 -41.4506
31.0200 -44.9200 -1.3068 -0.1052
0.2316 -0.0604 -0.1235 0.1213 -0.2495
-0.4614 -1.5777 -41.4527
31.0400 -41.7600 1.3450 0.4457
0.2880 0.0081 -0.1437 0.0994 -0.2371
-0.4614 -1.5714 -41.4547
31.0600 -44.2600 -1.0295 0.1710
0.2593 0.0606 -0.1602 0.0771 -0.2246
-0.4614 -1.5650 -41.4567
31.0800 -44.1900 -0.2747 -0.3398
0.1943 0.0939 -0.1730 0.0547 -0.2120
-0.4612 -1.5587 -41.4588
31.1000 -44.1300 -0.2719 -0.2880
0.1359 0.1081 -0.1824 0.0323 -0.1994
-0.4610 -1.5523 -41.4608
31.1200 -43.0500 0.4252 0.1135
0.0888 0.1058 -0.1888 0.0101 -0.1867
-0.4608 -1.5459 -41.4628
31.1400 -43.6000 -0.3642 0.4544
0.0277 0.0919 -0.1928 -0.0117 -0.1741
-0.4604 -1.5395 -41.4648
31.1600 -42.7000 0.8090 0.1945 -
0.0646 0.0717 -0.1950 -0.0328 -0.1614
-0.4600 -1.5331 -41.4669
31.1800 -45.1700 -0.9136 -0.3513 -
0.1653 0.0505 -0.1961 -0.0532 -0.1487
-0.4595 -1.5267 -41.4689
31.2000 -43.6900 0.6471 -0.3557 -
0.2295 0.0328 -0.1969 -0.0726 -0.1360
-0.4590 -1.5203 -41.4709
31.2200 -44.2600 -0.0832 -0.0885 -
0.2219 0.0216 -0.1980 -0.0908 -0.1234
-0.4583 -1.5138 -41.4729
31.2400 -44.1900 -0.1785 0.0159 -
0.1385 0.0175 -0.2002 -0.1077 -0.1108
-0.4576 -1.5073 -41.4749
31.2600 -43.6800 0.2036 0.0400 -
0.0177 0.0204 -0.2041 -0.1231 -0.0982
-0.4568 -1.5009 -41.4769
31.2800 -43.9700 -0.3951 0.2629
0.0753 0.0292 -0.2104 -0.1368 -0.0858
-0.4560 -1.4944 -41.4789
31.3000 -42.7900 0.3220 0.5366
0.1041 0.0427 -0.2195 -0.1487 -0.0733
-0.4551 -1.4879 -41.4809

31.3200 -43.6300 -0.1855 0.4320
0.0685 0.0596 -0.2320 -0.1586 -0.0610
-0.4541 -1.4814 -41.4829
31.3400 -43.6800 0.1535 -0.0634 -
0.0021 0.0782 -0.2480 -0.1664 -0.0487
-0.4530 -1.4748 -41.4849
31.3600 -44.3500 0.1427 -0.5883 -
0.0611 0.0963 -0.2675 -0.1721 -0.0366
-0.4519 -1.4683 -41.4869
31.3800 -45.2100 -0.6819 -0.7289 -
0.0704 0.1118 -0.2906 -0.1756 -0.0246
-0.4507 -1.4617 -41.4889
31.4000 -43.5600 0.7207 -0.4316 -
0.0187 0.1236 -0.3170 -0.1769 -0.0126
-0.4494 -1.4552 -41.4909
31.4200 -43.0800 0.5660 0.0795
0.0697 0.1314 -0.3466 -0.1760 -0.0009
-0.4481 -1.4486 -41.4929
31.4400 -44.5700 -1.3337 0.5597
0.1520 0.1354 -0.3788 -0.1730 0.0108
-0.4467 -1.4420 -41.4949
31.4600 -41.6300 1.4153 0.6465
0.1886 0.1360 -0.4132 -0.1679 0.0223
-0.4452 -1.4354 -41.4969
31.4800 -43.3000 0.0052 0.2054
0.1599 0.1344 -0.4494 -0.1608 0.0337
-0.4436 -1.4288 -41.4989
31.5000 -45.4100 -1.3063 -0.3150
0.0781 0.1322 -0.4867 -0.1518 0.0449
-0.4420 -1.4222 -41.5009
31.5200 -43.8600 0.5046 -0.4506 -
0.0321 0.1314 -0.5245 -0.1411 0.0560
-0.4403 -1.4155 -41.5028
31.5400 -43.3800 0.7829 -0.2236 -
0.1386 0.1342 -0.5623 -0.1287 0.0669
-0.4385 -1.4089 -41.5048
31.5600 -44.7500 -0.7937 0.0898 -
0.2122 0.1418 -0.5992 -0.1147 0.0776
-0.4367 -1.4022 -41.5068
31.5800 -43.3200 0.5505 0.2173 -
0.2361 0.1540 -0.6346 -0.0994 0.0882
-0.4348 -1.3956 -41.5088
31.6000 -44.0000 -0.0749 0.1432 -
0.2084 0.1694 -0.6674 -0.0828 0.0986
-0.4328 -1.3889 -41.5108
31.6200 -44.1800 -0.2660 -0.0431 -
0.1404 0.1855 -0.6967 -0.0651 0.1088
-0.4307 -1.3822 -41.5127
31.6400 -43.7000 0.3159 -0.1920 -
0.0464 0.1984 -0.7215 -0.0465 0.1188
-0.4286 -1.3755 -41.5147

31.6600 -44.2900 -0.3115 -0.2071
0.0599 0.2038 -0.7406 -0.0271 0.1287
-0.4264 -1.3688 -41.5167
31.6800 -43.6200 -0.0549 -0.0153
0.1597 0.1977 -0.7532 -0.0071 0.1383
-0.4242 -1.3621 -41.5186
31.7000 -43.2200 0.0300 0.2702
0.2281 0.1765 -0.7583 0.0135 0.1478
-0.4219 -1.3553 -41.5206
31.7200 -42.6900 0.5987 0.3181
0.2429 0.1370 -0.7554 0.0344 0.1571
-0.4195 -1.3486 -41.5225
31.7400 -43.1200 0.4481 0.0097
0.1967 0.0775 -0.7439 0.0556 0.1662
-0.4170 -1.3418 -41.5245
31.7600 -45.0200 -1.0515 -0.2931
0.1095 -0.0013 -0.7235 0.0769 0.1751
-0.4145 -1.3351 -41.5265
31.7800 -43.2400 0.7225 -0.1626
0.0099 -0.0971 -0.6942 0.0982 0.1838
-0.4119 -1.3283 -41.5284
31.8000 -43.4000 0.4094 0.0882 -
0.0721 -0.2058 -0.6559 0.1194 0.1923
-0.4092 -1.3215 -41.5304
31.8200 -43.5200 0.3905 0.0159 -
0.1123 -0.3224 -0.6086 0.1403 0.2006
-0.4065 -1.3147 -41.5323
31.8400 -45.2200 -1.1086 -0.0898 -
0.1000 -0.4406 -0.5522 0.1610 0.2087
-0.4036 -1.3079 -41.5343
31.8600 -42.8100 1.0966 -0.0290 -
0.0445 -0.5532 -0.4869 0.1812 0.2166
-0.4008 -1.3011 -41.5362
31.8800 -44.8900 -1.0294 0.0350
0.0192 -0.6525 -0.4127 0.2010 0.2243
-0.3978 -1.2943 -41.5382
31.9000 -42.7400 0.8410 0.1109
0.0475 -0.7312 -0.3297 0.2202 0.2317
-0.3948 -1.2875 -41.5401
31.9200 -44.2300 -0.4934 0.0883
0.0127 -0.7833 -0.2381 0.2386 0.2390
-0.3917 -1.2807 -41.5420
31.9400 -43.4900 0.1783 0.0620 -
0.0786 -0.8047 -0.1383 0.2564 0.2460
-0.3886 -1.2738 -41.5440
31.9600 -44.1400 -0.5733 0.1452 -
0.1836 -0.7935 -0.0308 0.2733 0.2529
-0.3854 -1.2670 -41.5459
31.9800 -42.5700 0.9757 -0.0444 -
0.2378 -0.7491 0.0834 0.2892 0.2595
-0.3821 -1.2601 -41.5478

32.0000 -44.4500 -0.7975 -0.3762 -
0.2004 -0.6742 0.2031 0.3042 0.2659
-0.3787 -1.2532 -41.5498
32.0200 -43.8600 -0.5933 -0.2537 -
0.0777 -0.5738 0.3267 0.3180 0.2720
-0.3753 -1.2464 -41.5517
32.0400 -41.4000 0.8553 0.2451
0.0852 -0.4548 0.4527 0.3307 0.2780
-0.3718 -1.2395 -41.5536
32.0600 -41.7300 0.0538 0.4447
0.2212 -0.3248 0.5796 0.3420 0.2837
-0.3683 -1.2326 -41.5556
32.0800 -41.2200 0.6338 -0.0270
0.2812 -0.1909 0.7058 0.3520 0.2892
-0.3646 -1.2257 -41.5575
32.1000 -42.7900 -0.9621 -0.3338
0.2704 -0.0591 0.8296 0.3606 0.2944
-0.3609 -1.2188 -41.5594
32.1200 -40.4700 0.8057 -0.0575
0.2199 0.0663 0.9494 0.3677 0.2994
-0.3572 -1.2119 -41.5613
32.1400 -41.0500 -0.2379 0.1088
0.1649 0.1828 1.0635 0.3731 0.3042
-0.3534 -1.2050 -41.5632
32.1600 -40.6900 0.2062 -0.0439
0.1212 0.2906 1.1703 0.3768 0.3088
-0.3495 -1.1980 -41.5652
32.1800 -40.8500 -0.2249 -0.1084
0.0792 0.3919 1.2679 0.3787 0.3131
-0.3455 -1.1911 -41.5671
32.2000 -40.6000 -0.0914 0.0585
0.0104 0.4904 1.3546 0.3788 0.3171
-0.3415 -1.1842 -41.5690
32.2200 -39.8600 0.3439 0.2052 -
0.1058 0.5893 1.4286 0.3769 0.3209
-0.3375 -1.1772 -41.5709
32.2400 -40.3300 -0.0698 0.1447 -
0.2641 0.6896 1.4884 0.3730 0.3245
-0.3333 -1.1703 -41.5728
32.2600 -40.7100 -0.1531 -0.0297 -
0.4281 0.7888 1.5323 0.3669 0.3278
-0.3291 -1.1633 -41.5747
32.2800 -40.5100 0.1059 -0.1743 -
0.5455 0.8824 1.5592 0.3587 0.3309
-0.3248 -1.1564 -41.5766
32.3000 -40.7000 -0.0487 -0.2277 -
0.5735 0.9650 1.5681 0.3482 0.3337
-0.3205 -1.1494 -41.5785
32.3200 -40.4300 0.1398 -0.1720 -
0.4870 1.0307 1.5580 0.3354 0.3362
-0.3161 -1.1424 -41.5804

32.3400 -40.4200 -0.3692 -0.0523 -
0.2826 1.0738 1.5285 0.3205 0.3385
-0.3117 -1.1354 -41.5823
32.3600 -39.8500 0.0973 -0.0331
0.0166 1.0883 1.4795 0.3035 0.3405
-0.3071 -1.1285 -41.5842
32.3800 -39.0500 0.7121 -0.1858
0.3547 1.0682 1.4114 0.2846 0.3423
-0.3026 -1.1215 -41.5861
32.4000 -40.3800 -0.7928 -0.2703
0.6523 1.0070 1.3255 0.2639 0.3439
-0.2979 -1.1145 -41.5880
32.4200 -39.6300 -0.2737 0.0568
0.8345 0.8997 1.2234 0.2416 0.3451
-0.2932 -1.1075 -41.5898
32.4400 -38.8000 0.3326 0.6196
0.8550 0.7447 1.1067 0.2178 0.3462
-0.2885 -1.1005 -41.5917
32.4600 -38.8900 0.4862 0.8534
0.6897 0.5477 0.9774 0.1927 0.3470
-0.2837 -1.0935 -41.5936
32.4800 -40.1400 0.3782 0.3992
0.3633 0.3202 0.8372 0.1664 0.3475
-0.2788 -1.0865 -41.5955
32.5000 -42.3800 -0.1820 -0.3831 -
0.0380 0.0753 0.6879 0.1390 0.3478
-0.2739 -1.0794 -41.5974
32.5200 -43.8300 -0.4749 -0.8536 -
0.4136 -0.1737 0.5313 0.1106 0.3478
-0.2689 -1.0724 -41.5993
32.5400 -43.5000 0.3832 -0.7391 -
0.6733 -0.4137 0.3693 0.0815 0.3477
-0.2639 -1.0654 -41.6011
32.5600 -44.1500 -0.1522 -0.2788 -
0.7737 -0.6319 0.2036 0.0517 0.3472
-0.2588 -1.0584 -41.6030
32.5800 -43.7600 0.0496 0.1921 -
0.7226 -0.8180 0.0361 0.0214 0.3466
-0.2536 -1.0513 -41.6049
32.6000 -43.7000 -0.0397 0.4617 -
0.5597 -0.9647 -0.1316 -0.0093 0.3457
-0.2484 -1.0443 -41.6067
32.6200 -43.3400 0.4365 0.5021 -
0.3490 -1.0672 -0.2977 -0.0402 0.3445
-0.2432 -1.0373 -41.6086
32.6400 -44.7900 -0.8279 0.4199 -
0.1499 -1.1233 -0.4608 -0.0712 0.3432
-0.2379 -1.0302 -41.6105
32.6600 -43.2700 0.9755 0.0511
0.0253 -1.1352 -0.6193 -0.1022 0.3416
-0.2325 -1.0232 -41.6123

32.6800 -45.8000 -0.9239 -0.5594
0.1812 -1.1067 -0.7718 -0.1330 0.3398
-0.2271 -1.0161 -41.6142
32.7000 -44.5800 0.2614 -0.6054
0.3134 -1.0424 -0.9170 -0.1634 0.3377
-0.2217 -1.0091 -41.6160
32.7200 -44.5800 -0.3047 0.0882
0.4037 -0.9461 -1.0537 -0.1934 0.3355
-0.2162 -1.0020 -41.6179
32.7400 -43.0800 0.6284 0.6433
0.4111 -0.8207 -1.1810 -0.2228 0.3330
-0.2107 -0.9950 -41.6197
32.7600 -43.4400 0.4035 0.5423
0.3048 -0.6705 -1.2979 -0.2514 0.3303
-0.2051 -0.9879 -41.6216
32.7800 -45.0300 -0.5536 0.0758
0.1103 -0.5007 -1.4040 -0.2792 0.3274
-0.1995 -0.9809 -41.6234
32.8000 -45.2500 -0.3519 -0.2351 -
0.1168 -0.3168 -1.4988 -0.3060 0.3243
-0.1938 -0.9738 -41.6253
32.8200 -44.8300 0.3039 -0.2996 -
0.3088 -0.1248 -1.5816 -0.3316 0.3209
-0.1881 -0.9667 -41.6271
32.8400 -44.8200 0.4056 -0.3596 -
0.3969 0.0695 -1.6521 -0.3560 0.3174
-0.1824 -0.9597 -41.6290
32.8600 -45.7700 -0.6162 -0.4097 -
0.3514 0.2596 -1.7095 -0.3790 0.3136
-0.1766 -0.9526 -41.6308
32.8800 -44.2600 0.3159 -0.3199 -
0.1880 0.4393 -1.7533 -0.4006 0.3097
-0.1708 -0.9455 -41.6327
32.9000 -44.0700 -0.0143 -0.0154
0.0371 0.6028 -1.7825 -0.4205 0.3056
-0.1649 -0.9385 -41.6345
32.9200 -43.8400 -0.6308 0.3997
0.2551 0.7440 -1.7964 -0.4387 0.3013
-0.1590 -0.9314 -41.6363
32.9400 -42.0900 0.7730 0.5527
0.4013 0.8572 -1.7943 -0.4551 0.2968
-0.1531 -0.9243 -41.6382
32.9600 -42.7500 0.2597 0.1833
0.4333 0.9368 -1.7755 -0.4696 0.2921
-0.1471 -0.9173 -41.6400
32.9800 -44.2900 -0.6842 -0.3315
0.3593 0.9783 -1.7395 -0.4822 0.2872
-0.1411 -0.9102 -41.6418
33.0000 -43.8100 0.0561 -0.3901
0.2037 0.9792 -1.6857 -0.4929 0.2822
-0.1351 -0.9031 -41.6436

33.0200 -43.8100 -0.2679 0.0699
0.0078 0.9385 -1.6142 -0.5017 0.2770
-0.1291 -0.8961 -41.6455
33.0400 -42.6800 0.6565 0.3888 -
0.1702 0.8563 -1.5253 -0.5086 0.2716
-0.1230 -0.8890 -41.6473
33.0600 -43.8900 -0.2058 0.1946 -
0.2775 0.7338 -1.4199 -0.5139 0.2661
-0.1169 -0.8819 -41.6491
33.0800 -44.2900 -0.2209 -0.2163 -
0.2887 0.5742 -1.2994 -0.5174 0.2605
-0.1108 -0.8748 -41.6509
33.1000 -44.1600 0.0417 -0.4424 -
0.1971 0.3835 -1.1653 -0.5193 0.2547
-0.1047 -0.8678 -41.6527
33.1200 -44.2300 -0.1994 -0.3153 -
0.0181 0.1703 -1.0193 -0.5197 0.2487
-0.0985 -0.8607 -41.6545
33.1400 -43.2800 0.0171 0.0833
0.1978 -0.0550 -0.8633 -0.5186 0.2427
-0.0923 -0.8536 -41.6564
33.1600 -42.9000 0.1431 0.4614
0.3807 -0.2816 -0.6990 -0.5161 0.2365
-0.0861 -0.8465 -41.6582
33.1800 -42.6600 0.3170 0.4346
0.4683 -0.4983 -0.5282 -0.5122 0.2301
-0.0799 -0.8395 -41.6600
33.2000 -43.1500 0.3006 0.0479
0.4195 -0.6955 -0.3527 -0.5070 0.2237
-0.0737 -0.8324 -41.6618
33.2200 -44.7300 -0.9639 -0.1388
0.2312 -0.8644 -0.1743 -0.5007 0.2172
-0.0675 -0.8253 -41.6636
33.2400 -42.9800 0.7194 0.1557 -
0.0623 -0.9963 0.0053 -0.4932 0.2106
-0.0612 -0.8183 -41.6654
33.2600 -43.4400 0.4035 0.2813 -
0.3766 -1.0827 0.1844 -0.4846 0.2038
-0.0549 -0.8112 -41.6672
33.2800 -43.6700 0.6751 -0.2564 -
0.6212 -1.1158 0.3612 -0.4751 0.1970
-0.0487 -0.8041 -41.6690
33.3000 -45.8600 -1.0402 -0.7531 -
0.7213 -1.0901 0.5343 -0.4646 0.1901
-0.0424 -0.7971 -41.6708
33.3200 -43.3500 0.7753 -0.4896 -
0.6344 -1.0060 0.7023 -0.4532 0.1832
-0.0361 -0.7900 -41.6726
33.3400 -43.3500 -0.4014 0.1479 -
0.4020 -0.8704 0.8639 -0.4410 0.1762
-0.0298 -0.7830 -41.6743

33.3600 -41.8900 0.1437 0.4653 -
0.0998 -0.6930 1.0180 -0.4282 0.1691
-0.0236 -0.7759 -41.6761
33.3800 -41.1500 0.2988 0.3265
0.1955 -0.4839 1.1633 -0.4146 0.1619
-0.0173 -0.7689 -41.6779
33.4000 -41.5600 -0.3184 0.0547
0.4206 -0.2534 1.2987 -0.4005 0.1548
-0.0110 -0.7618 -41.6797
33.4200 -40.9700 -0.3132 -0.0244
0.5349 -0.0119 1.4229 -0.3859 0.1475
-0.0047 -0.7548 -41.6815
33.4400 -39.7500 0.4202 0.1050
0.5367 0.2301 1.5348 -0.3708 0.1403
0.0016 -0.7477 -41.6832
33.4600 -40.0700 -0.1008 0.1567
0.4452 0.4626 1.6332 -0.3553 0.1330
0.0079 -0.7407 -41.6850
33.4800 -39.6100 0.4145 -0.0244
0.2856 0.6767 1.7168 -0.3395 0.1257
0.0141 -0.7336 -41.6868
33.5000 -40.4300 -0.4497 -0.1803
0.0896 0.8642 1.7845 -0.3235 0.1185
0.0204 -0.7266 -41.6886
33.5200 -40.3200 -0.3000 -0.0656 -
0.1038 1.0179 1.8351 -0.3073 0.1112
0.0266 -0.7196 -41.6903
33.5400 -38.9100 0.6213 0.1521 -
0.2517 1.1310 1.8675 -0.2910 0.1039
0.0329 -0.7125 -41.6921
33.5600 -39.9800 -0.3082 0.1426 -
0.3162 1.1987 1.8804 -0.2747 0.0966
0.0391 -0.7055 -41.6939
33.5800 -39.9600 -0.2037 -0.0997 -
0.2765 1.2187 1.8731 -0.2584 0.0894
0.0453 -0.6985 -41.6956
33.6000 -39.6900 0.3309 -0.3557 -
0.1424 1.1922 1.8454 -0.2422 0.0822
0.0515 -0.6915 -41.6974
33.6200 -39.9300 -0.0674 -0.4377
0.0577 1.1213 1.7984 -0.2260 0.0750
0.0577 -0.6844 -41.6991
33.6400 -40.0600 -0.4841 -0.1452
0.2714 1.0089 1.7334 -0.2100 0.0678
0.0638 -0.6774 -41.7009
33.6600 -38.5800 0.5017 0.3943
0.4334 0.8592 1.6523 -0.1939 0.0607
0.0700 -0.6704 -41.7027
33.6800 -39.0600 -0.0266 0.6376
0.4807 0.6786 1.5568 -0.1780 0.0537
0.0761 -0.6634 -41.7044

33.7000	-39.8400	-0.0495	0.3673	
0.3866	0.4754	1.4485	-0.1621	0.0467
	0.0822	-0.6564	-41.7062	
33.7200	-40.5400	0.0420	-0.1372	
0.1928	0.2590	1.3293	-0.1463	0.0398
	0.0882	-0.6494	-41.7079	
33.7400	-41.8200	-0.2459	-0.4751	-
0.0352	0.0386	1.2009	-0.1306	0.0329
	0.0943	-0.6425	-41.7097	
33.7600	-41.9700	0.1904	-0.4210	-
0.2284	-0.1767	1.0651	-0.1150	0.0262
	0.1003	-0.6355	-41.7114	
33.7800	-42.0600	0.0737	-0.0897	-
0.3314	-0.3774	0.9236	-0.0995	0.0195
	0.1063	-0.6285	-41.7131	
33.8000	-42.4200	-0.3232	0.1816	-
0.3164	-0.5550	0.7782	-0.0842	0.0128
	0.1122	-0.6215	-41.7149	
33.8200	-42.3800	0.1473	0.0815	-
0.1942	-0.7023	0.6305	-0.0689	0.0063
	0.1181	-0.6146	-41.7166	
33.8400	-42.1700	0.6343	-0.2522	-
0.0270	-0.8143	0.4824	-0.0538	-0.0001
	0.1240	-0.6076	-41.7183	
33.8600	-44.0900	-1.0716	-0.2679	
0.1027	-0.8875	0.3356	-0.0389	-0.0065
	0.1298	-0.6007	-41.7201	
33.8800	-41.3700	1.1267	0.2612	
0.1428	-0.9209	0.1915	-0.0241	-0.0127
	0.1357	-0.5937	-41.7218	
33.9000	-43.3800	-0.9780	0.5596	
0.0863	-0.9157	0.0516	-0.0095	-0.0188
	0.1414	-0.5868	-41.7235	
33.9200	-42.9900	-0.0511	0.2259	-
0.0429	-0.8746	-0.0831	0.0050	-0.0248
	0.1472	-0.5798	-41.7253	
33.9400	-42.8000	0.8625	-0.3621	-
0.1819	-0.8014	-0.2119	0.0193	-0.0307
	0.1529	-0.5729	-41.7270	
33.9600	-44.8900	-0.8786	-0.5944	-
0.2550	-0.7002	-0.3343	0.0333	-0.0364
	0.1585	-0.5660	-41.7287	
33.9800	-42.8400	0.6974	-0.1960	-
0.2214	-0.5763	-0.4500	0.0472	-0.0420
	0.1641	-0.5591	-41.7304	
34.0000	-43.3800	-0.4016	0.2556	-
0.1094	-0.4364	-0.5588	0.0609	-0.0475
	0.1697	-0.5522	-41.7321	
34.0200	-42.2700	0.3322	0.3441	
0.0281	-0.2878	-0.6604	0.0744	-0.0528
	0.1752	-0.5453	-41.7339	

34.0400 -42.7000 -0.0931 0.2223
0.1322 -0.1376 -0.7549 0.0878 -0.0580
0.1807 -0.5384 -41.7356
34.0600 -43.0400 -0.3541 0.0795
0.1678 0.0075 -0.8423 0.1012 -0.0630
0.1861 -0.5315 -41.7373
34.0800 -42.1900 0.5547 -0.0441
0.1357 0.1414 -0.9227 0.1147 -0.0678
0.1915 -0.5246 -41.7390
34.1000 -43.3200 -0.5051 -0.1262
0.0618 0.2589 -0.9964 0.1282 -0.0725
0.1968 -0.5177 -41.7407
34.1200 -43.1000 -0.3818 -0.0653 -
0.0154 0.3561 -1.0634 0.1419 -0.0770
0.2021 -0.5109 -41.7424
34.1400 -42.1400 0.6875 0.0036 -
0.0583 0.4296 -1.1242 0.1558 -0.0814
0.2073 -0.5040 -41.7441
34.1600 -42.3500 0.3775 -0.0810 -
0.0491 0.4780 -1.1790 0.1702 -0.0855
0.2124 -0.4972 -41.7458
34.1800 -43.3600 -0.4900 -0.1887
0.0038 0.5019 -1.2282 0.1850 -0.0895
0.2175 -0.4903 -41.7475
34.2000 -43.1400 -0.4847 -0.0551
0.0734 0.5033 -1.2724 0.2003 -0.0933
0.2226 -0.4835 -41.7492
34.2200 -41.8400 0.6232 0.2582
0.1222 0.4859 -1.3121 0.2162 -0.0969
0.2276 -0.4767 -41.7509
34.2400 -42.7000 -0.4052 0.3387
0.1179 0.4542 -1.3476 0.2328 -0.1002
0.2325 -0.4698 -41.7526
34.2600 -42.3000 0.5038 0.0257
0.0518 0.4133 -1.3795 0.2501 -0.1034
0.2374 -0.4630 -41.7543
34.2800 -43.4600 -0.2528 -0.3097 -
0.0528 0.3681 -1.4080 0.2683 -0.1064
0.2422 -0.4562 -41.7559
34.3000 -44.0600 -0.5119 -0.3043 -
0.1583 0.3224 -1.4335 0.2872 -0.1091
0.2470 -0.4495 -41.7576
34.3200 -43.3500 -0.1997 0.0601 -
0.2268 0.2787 -1.4560 0.3070 -0.1116
0.2517 -0.4427 -41.7593
34.3400 -42.3600 0.6322 0.3009 -
0.2354 0.2374 -1.4753 0.3275 -0.1139
0.2563 -0.4359 -41.7610
34.3600 -42.4100 0.6776 0.0594 -
0.1765 0.1975 -1.4911 0.3487 -0.1159
0.2608 -0.4291 -41.7627

34.3800 -44.3900 -0.9852 -0.3437 -
0.0624 0.1574 -1.5029 0.3705 -0.1178
0.2653 -0.4224 -41.7643
34.4000 -42.6400 0.6227 -0.3125
0.0828 0.1153 -1.5101 0.3928 -0.1194
0.2698 -0.4157 -41.7660
34.4200 -43.1700 -0.3779 0.1034
0.2245 0.0692 -1.5122 0.4155 -0.1207
0.2742 -0.4089 -41.7677
34.4400 -42.3200 0.1108 0.3715
0.3270 0.0176 -1.5085 0.4386 -0.1218
0.2785 -0.4022 -41.7694
34.4600 -42.0100 0.3850 0.2813
0.3578 -0.0402 -1.4985 0.4618 -0.1227
0.2827 -0.3955 -41.7710
34.4800 -43.2100 -0.4846 -0.0065
0.3093 -0.1028 -1.4818 0.4851 -0.1233
0.2869 -0.3888 -41.7727
34.5000 -42.7100 0.5180 -0.2382
0.2023 -0.1673 -1.4581 0.5085 -0.1237
0.2910 -0.3821 -41.7743
34.5200 -43.3900 -0.0242 -0.2884
0.0714 -0.2292 -1.4271 0.5317 -0.1239
0.2950 -0.3754 -41.7760
34.5400 -43.9400 -0.5851 -0.2012 -
0.0490 -0.2837 -1.3887 0.5546 -0.1238
0.2989 -0.3688 -41.7777
34.5600 -42.6100 0.7189 -0.1054 -
0.1368 -0.3257 -1.3429 0.5773 -0.1234
0.3028 -0.3621 -41.7793
34.5800 -43.0800 0.2682 -0.1017 -
0.1930 -0.3507 -1.2896 0.5995 -0.1228
0.3067 -0.3555 -41.7810
34.6000 -43.9200 -0.7368 -0.0741 -
0.2358 -0.3550 -1.2288 0.6212 -0.1220
0.3104 -0.3488 -41.7826
34.6200 -43.5100 -0.6244 0.2113 -
0.2847 -0.3362 -1.1607 0.6423 -0.1209
0.3141 -0.3422 -41.7843
34.6400 -41.7700 0.6154 0.6292 -
0.3379 -0.2937 -1.0854 0.6626 -0.1195
0.3177 -0.3356 -41.7859
34.6600 -41.3200 0.8504 0.7107 -
0.3820 -0.2300 -1.0035 0.6822 -0.1179
0.3212 -0.3290 -41.7876
34.6800 -42.2300 0.2393 0.1525 -
0.3944 -0.1498 -0.9153 0.7009 -0.1161
0.3247 -0.3224 -41.7892
34.7000 -43.1000 0.0680 -0.7139 -
0.3405 -0.0593 -0.8215 0.7187 -0.1140
0.3281 -0.3158 -41.7908

34.7200 -44.3400 -1.2887 -0.9717 -
0.1900 0.0341 -0.7225 0.7354 -0.1117
0.3314 -0.3093 -41.7925
34.7400 -40.9100 0.7612 -0.2379
0.0548 0.1229 -0.6187 0.7509 -0.1091
0.3346 -0.3027 -41.7941
34.7600 -40.2000 0.2148 0.6038
0.3231 0.1995 -0.5107 0.7653 -0.1063
0.3378 -0.2962 -41.7957
34.7800 -39.3600 0.6343 0.7044
0.5309 0.2567 -0.3991 0.7784 -0.1032
0.3409 -0.2897 -41.7974
34.8000 -40.4000 -0.3883 0.2858
0.6186 0.2875 -0.2842 0.7901 -0.1000
0.3439 -0.2831 -41.7990
34.8200 -40.9900 -0.4794 -0.0588
0.5674 0.2878 -0.1667 0.8004 -0.0964
0.3469 -0.2766 -41.8006
34.8400 -40.2200 0.3332 -0.0954
0.3941 0.2587 -0.0473 0.8091 -0.0927
0.3498 -0.2702 -41.8023
34.8600 -40.3500 0.2144 -0.0179
0.1516 0.2050 0.0732 0.8163 -0.0887
0.3526 -0.2637 -41.8039
34.8800 -41.1700 -0.4108 -0.0450 -
0.0963 0.1327 0.1940 0.8217 -0.0845
0.3553 -0.2572 -41.8055
34.9000 -40.4400 0.6497 -0.1790 -
0.3014 0.0482 0.3142 0.8254 -0.0800
0.3580 -0.2508 -41.8071
34.9200 -41.7700 -0.8330 -0.1027 -
0.4372 -0.0415 0.4330 0.8272 -0.0754
0.3606 -0.2443 -41.8087
34.9400 -40.0800 0.8268 0.1210 -
0.4867 -0.1293 0.5494 0.8271 -0.0705
0.3631 -0.2379 -41.8103
34.9600 -41.3000 -0.4379 0.0116 -
0.4493 -0.2089 0.6627 0.8249 -0.0654
0.3655 -0.2315 -41.8120
34.9800 -41.0500 -0.0476 -0.2980 -
0.3309 -0.2763 0.7719 0.8207 -0.0601
0.3679 -0.2251 -41.8136
35.0000 -41.1600 -0.3902 -0.2944 -
0.1546 -0.3294 0.8763 0.8143 -0.0546
0.3702 -0.2188 -41.8152
35.0200 -40.0400 0.2149 0.0607
0.0371 -0.3669 0.9753 0.8056 -0.0489
0.3724 -0.2124 -41.8168
35.0400 -39.7100 -0.0495 0.4010
0.1946 -0.3873 1.0682 0.7946 -0.0430
0.3746 -0.2060 -41.8184

35.0600 -39.0700 0.3742 0.4378
0.2763 -0.3896 1.1542 0.7812 -0.0369
0.3766 -0.1997 -41.8200
35.0800 -39.5300 0.0674 0.1877
0.2725 -0.3732 1.2329 0.7653 -0.0307
0.3786 -0.1934 -41.8216
35.1000 -40.0200 -0.2671 -0.1148
0.1994 -0.3374 1.3035 0.7469 -0.0242
0.3806 -0.1871 -41.8232
35.1200 -40.3600 -0.3985 -0.2306
0.0855 -0.2831 1.3654 0.7258 -0.0176
0.3824 -0.1808 -41.8248
35.1400 -39.7500 0.2475 -0.1844 -
0.0366 -0.2119 1.4178 0.7022 -0.0108
0.3842 -0.1745 -41.8264
35.1600 -39.3100 0.7097 -0.1692 -
0.1399 -0.1261 1.4602 0.6758 -0.0038
0.3859 -0.1683 -41.8279
35.1800 -40.1100 -0.3109 -0.2049 -
0.2141 -0.0276 1.4920 0.6468 0.0033
0.3875 -0.1620 -41.8295
35.2000 -40.4500 -0.7622 -0.1047 -
0.2606 0.0816 1.5125 0.6152 0.0106
0.3891 -0.1558 -41.8311
35.2200 -38.7500 0.7195 0.1285 -
0.2893 0.1981 1.5212 0.5810 0.0181
0.3906 -0.1496 -41.8327
35.2400 -39.6400 -0.3388 0.1882 -
0.3075 0.3172 1.5177 0.5443 0.0257
0.3920 -0.1434 -41.8343
35.2600 -39.3100 0.1730 -0.0121 -
0.3095 0.4326 1.5015 0.5053 0.0335
0.3934 -0.1372 -41.8359
35.2800 -39.8200 -0.2115 -0.2400 -
0.2730 0.5367 1.4723 0.4639 0.0414
0.3946 -0.1310 -41.8374
35.3000 -39.2100 0.2232 -0.2760 -
0.1742 0.6216 1.4297 0.4203 0.0494
0.3959 -0.1249 -41.8390
35.3200 -39.6100 -0.4769 -0.1051
0.0023 0.6804 1.3737 0.3748 0.0575
0.3970 -0.1188 -41.8406
35.3400 -38.4900 0.5247 0.0068
0.2474 0.7072 1.3046 0.3274 0.0658
0.3980 -0.1127 -41.8421
35.3600 -38.8000 0.0941 -0.1244
0.5219 0.6977 1.2229 0.2782 0.0743
0.3990 -0.1066 -41.8437
35.3800 -39.4600 -0.4901 -0.2069
0.7544 0.6484 1.1297 0.2275 0.0828
0.3999 -0.1005 -41.8453

35.4000 -38.8100 -0.0677 0.0688
0.8655 0.5580 1.0261 0.1754 0.0914
0.4008 -0.0944 -41.8468
35.4200 -38.2500 0.4166 0.5155
0.8052 0.4290 0.9136 0.1221 0.1002
0.4016 -0.0884 -41.8484
35.4400 -39.1800 -0.2261 0.7750
0.5594 0.2699 0.7938 0.0676 0.1091
0.4023 -0.0823 -41.8499
35.4600 -39.4600 0.2760 0.7046
0.1613 0.0935 0.6682 0.0121 0.1180
0.4029 -0.0763 -41.8515
35.4800 -40.9700 0.0097 0.2411 -
0.3018 -0.0856 0.5384 -0.0441 0.1271
0.4035 -0.0703 -41.8531
35.5000 -42.7400 -0.1398 -0.5123 -
0.7155 -0.2529 0.4059 -0.1010 0.1362
0.4040 -0.0644 -41.8546
35.5200 -42.9400 0.6576 -0.9914 -
0.9638 -0.3940 0.2722 -0.1584 0.1455
0.4044 -0.0584 -41.8562
35.5400 -44.6500 -1.3527 -0.4436 -
0.9681 -0.4962 0.1389 -0.2162 0.1548
0.4047 -0.0525 -41.8577
35.5600 -40.8600 1.4317 0.6549 -
0.7696 -0.5538 0.0075 -0.2742 0.1642
0.4050 -0.0465 -41.8592
35.5800 -43.1700 -1.1011 0.6967 -
0.4597 -0.5689 -0.1207 -0.3322 0.1736
0.4052 -0.0406 -41.8608
35.6000 -42.7800 -0.2134 0.0894 -
0.1338 -0.5460 -0.2444 -0.3901 0.1832
0.4053 -0.0347 -41.8623
35.6200 -42.8900 -0.4154 -0.0500
0.1354 -0.4927 -0.3626 -0.4478 0.1928
0.4054 -0.0289 -41.8639
35.6400 -41.5100 0.8813 -0.0844
0.3481 -0.4220 -0.4741 -0.5051 0.2024
0.4054 -0.0230 -41.8654
35.6600 -42.9600 -0.4030 -0.3289
0.5339 -0.3497 -0.5779 -0.5618 0.2121
0.4053 -0.0172 -41.8669
35.6800 -42.9100 -0.4980 -0.3626
0.6926 -0.2894 -0.6730 -0.6179 0.2219
0.4052 -0.0114 -41.8685
35.7000 -41.7500 0.2489 0.0336
0.7961 -0.2487 -0.7588 -0.6730 0.2317
0.4050 -0.0056 -41.8700
35.7200 -41.5600 0.2780 0.4348
0.8025 -0.2300 -0.8346 -0.7272 0.2416
0.4047 0.0002 -41.8715

35.7400 -41.3100 0.8850 0.2933
0.6794 -0.2327 -0.9004 -0.7802 0.2515
0.4044 0.0060 -41.8730
35.7600 -44.0000 -1.1176 -0.0958
0.4340 -0.2517 -0.9564 -0.8320 0.2614
0.4040 0.0117 -41.8746
35.7800 -43.1000 0.3326 -0.1525
0.1069 -0.2796 -1.0028 -0.8823 0.2714
0.4035 0.0174 -41.8761
35.8000 -43.1400 0.6575 -0.0421 -
0.2400 -0.3084 -1.0399 -0.9310 0.2814
0.4029 0.0231 -41.8776
35.8200 -44.0400 0.1187 -0.0599 -
0.5353 -0.3306 -1.0682 -0.9780 0.2914
0.4023 0.0288 -41.8791
35.8400 -44.9600 -0.4375 -0.1706 -
0.7082 -0.3388 -1.0882 -1.0232 0.3014
0.4016 0.0345 -41.8806
35.8600 -44.3700 0.3773 -0.2494 -
0.7089 -0.3277 -1.1003 -1.0663 0.3115
0.4008 0.0401 -41.8821
35.8800 -44.7500 -0.2462 -0.3194 -
0.5357 -0.2961 -1.1053 -1.1075 0.3215
0.4000 0.0458 -41.8836
35.9000 -43.8800 0.2516 -0.3180 -
0.2337 -0.2463 -1.1036 -1.1464 0.3316
0.3991 0.0514 -41.8851
35.9200 -44.0600 -0.6523 0.0487
0.0998 -0.1817 -1.0960 -1.1831 0.3417
0.3981 0.0570 -41.8866
35.9400 -41.7500 0.8120 0.5171
0.3624 -0.1055 -1.0830 -1.2174 0.3518
0.3971 0.0625 -41.8881
35.9600 -43.0700 -0.5826 0.5160
0.4760 -0.0211 -1.0653 -1.2493 0.3619
0.3960 0.0681 -41.8896
35.9800 -42.4200 0.3898 0.1568
0.4210 0.0678 -1.0434 -1.2786 0.3719
0.3948 0.0736 -41.8911
36.0000 -43.3600 -0.1398 -0.2122
0.2442 0.1555 -1.0179 -1.3053 0.3820
0.3936 0.0791 -41.8926
36.0200 -43.3300 -0.0652 -0.4085
0.0214 0.2365 -0.9895 -1.3294 0.3920
0.3923 0.0846 -41.8941
36.0400 -43.6200 -0.0985 -0.3103 -
0.1725 0.3053 -0.9588 -1.3508 0.4021
0.3909 0.0901 -41.8956
36.0600 -43.2100 0.1727 -0.0767 -
0.2769 0.3570 -0.9262 -1.3694 0.4121
0.3895 0.0956 -41.8971

36.0800 -43.1000 -0.0575 -0.0123 -
0.2586 0.3887 -0.8924 -1.3852 0.4221
0.3880 0.1010 -41.8986
36.1000 -43.1100 -0.0391 -0.1446 -
0.1248 0.4006 -0.8580 -1.3982 0.4320
0.3864 0.1064 -41.9001
36.1200 -42.5400 0.2581 -0.1906
0.0686 0.3944 -0.8232 -1.4084 0.4420
0.3848 0.1118 -41.9016
36.1400 -43.0600 -0.5872 0.0922
0.2449 0.3720 -0.7884 -1.4157 0.4519
0.3831 0.1172 -41.9030
36.1600 -42.4000 -0.4687 0.5315
0.3327 0.3351 -0.7541 -1.4203 0.4617
0.3813 0.1225 -41.9045
36.1800 -40.6600 1.2719 0.5942
0.2769 0.2860 -0.7203 -1.4220 0.4716
0.3795 0.1279 -41.9060
36.2000 -43.7700 -1.2210 0.0316
0.0843 0.2282 -0.6874 -1.4209 0.4813
0.3775 0.1332 -41.9075
36.2200 -42.7100 0.5164 -0.2320 -
0.1694 0.1659 -0.6554 -1.4170 0.4910
0.3756 0.1385 -41.9089
36.2400 -43.1200 0.2882 -0.1026 -
0.3836 0.1035 -0.6244 -1.4105 0.5007
0.3735 0.1437 -41.9104
36.2600 -43.1600 0.5247 -0.2627 -
0.4681 0.0454 -0.5944 -1.4013 0.5103
0.3714 0.1490 -41.9119
36.2800 -44.5800 -0.9116 -0.4367 -
0.3966 -0.0057 -0.5652 -1.3895 0.5199
0.3693 0.1542 -41.9133
36.3000 -42.3200 0.9127 -0.2352 -
0.1982 -0.0493 -0.5369 -1.3753 0.5294
0.3670 0.1595 -41.9148
36.3200 -43.3600 -0.8672 0.2031
0.0492 -0.0871 -0.5093 -1.3586 0.5388
0.3647 0.1646 -41.9162
36.3400 -41.1400 0.7948 0.5602
0.2490 -0.1213 -0.4822 -1.3395 0.5482
0.3623 0.1698 -41.9177
36.3600 -41.8900 -0.0664 0.4424
0.3375 -0.1536 -0.4554 -1.3182 0.5574
0.3599 0.1750 -41.9191
36.3800 -42.5000 -0.1084 -0.0182
0.3155 -0.1844 -0.4289 -1.2947 0.5666
0.3574 0.1801 -41.9206
36.4000 -43.5600 -0.6764 -0.3437
0.2334 -0.2129 -0.4027 -1.2692 0.5757
0.3548 0.1852 -41.9220

36.4200 -42.0400 0.9164 -0.4531
0.1509 -0.2376 -0.3766 -1.2417 0.5847
0.3522 0.1903 -41.9235
36.4400 -43.7400 -0.8898 -0.3449
0.0904 -0.2568 -0.3507 -1.2122 0.5936
0.3495 0.1954 -41.9249
36.4600 -41.8100 0.5255 0.1854
0.0353 -0.2676 -0.3253 -1.1809 0.6024
0.3467 0.2005 -41.9264
36.4800 -41.9100 -0.2204 0.7124 -
0.0382 -0.2658 -0.3005 -1.1479 0.6111
0.3439 0.2055 -41.9278
36.5000 -41.3900 0.6256 0.5272 -
0.1389 -0.2480 -0.2765 -1.1131 0.6197
0.3410 0.2105 -41.9293
36.5200 -43.4100 -0.5725 -0.1647 -
0.2488 -0.2126 -0.2536 -1.0767 0.6281
0.3380 0.2155 -41.9307
36.5400 -42.9900 0.0242 -0.5875 -
0.3295 -0.1599 -0.2322 -1.0386 0.6365
0.3350 0.2205 -41.9321
36.5600 -43.0500 -0.0167 -0.5661 -
0.3337 -0.0918 -0.2124 -0.9990 0.6447
0.3319 0.2254 -41.9335
36.5800 -42.3800 0.1782 -0.3765 -
0.2392 -0.0117 -0.1946 -0.9579 0.6527
0.3288 0.2304 -41.9350
36.6000 -41.5200 0.4547 -0.1024 -
0.0731 0.0746 -0.1792 -0.9154 0.6606
0.3256 0.2353 -41.9364
36.6200 -41.9100 -0.8922 0.3289
0.1092 0.1609 -0.1664 -0.8715 0.6684
0.3223 0.2402 -41.9378
36.6400 -39.8800 0.8772 0.6333
0.2467 0.2412 -0.1565 -0.8264 0.6760
0.3189 0.2451 -41.9392
36.6600 -40.2000 0.3854 0.4448
0.2964 0.3095 -0.1499 -0.7799 0.6835
0.3155 0.2499 -41.9407
36.6800 -41.8100 -0.8772 0.0110
0.2639 0.3609 -0.1467 -0.7324 0.6908
0.3121 0.2548 -41.9421
36.7000 -41.0900 0.1182 -0.2118
0.1783 0.3920 -0.1472 -0.6837 0.6980
0.3085 0.2596 -41.9435
36.7200 -40.7400 0.4012 -0.1941
0.0793 0.4011 -0.1514 -0.6340 0.7049
0.3049 0.2644 -41.9449
36.7400 -41.3400 -0.1577 -0.1791
0.0080 0.3875 -0.1594 -0.5833 0.7117
0.3013 0.2692 -41.9463

36.7600 -41.3300 0.0146 -0.2676 -
0.0102 0.3518 -0.1709 -0.5318 0.7183
0.2975 0.2739 -41.9477
36.7800 -41.2300 -0.0481 -0.2216
0.0162 0.2963 -0.1853 -0.4795 0.7247
0.2938 0.2787 -41.9491
36.8000 -41.3800 -0.5240 0.0893
0.0592 0.2250 -0.2023 -0.4264 0.7309
0.2899 0.2834 -41.9505
36.8200 -39.9200 0.7738 0.3650
0.0867 0.1424 -0.2210 -0.3726 0.7370
0.2860 0.2881 -41.9519
36.8400 -41.1500 -0.5153 0.2455
0.0716 0.0534 -0.2411 -0.3183 0.7428
0.2820 0.2928 -41.9533
36.8600 -41.2100 0.0129 0.0339
0.0135 -0.0369 -0.2617 -0.2634 0.7484
0.2780 0.2974 -41.9547
36.8800 -41.2100 0.0292 -0.0520 -
0.0652 -0.1234 -0.2822 -0.2081 0.7538
0.2739 0.3021 -41.9561
36.9000 -41.5000 -0.0094 -0.1040 -
0.1409 -0.2012 -0.3021 -0.1525 0.7589
0.2698 0.3067 -41.9575
36.9200 -41.4700 0.2438 -0.2071 -
0.1910 -0.2658 -0.3206 -0.0965 0.7639
0.2656 0.3113 -41.9589
36.9400 -42.1700 -0.4824 -0.2364 -
0.1999 -0.3135 -0.3372 -0.0404 0.7686
0.2613 0.3159 -41.9603
36.9600 -41.1800 0.2572 -0.0445 -
0.1701 -0.3419 -0.3513 0.0159 0.7730
0.2570 0.3204 -41.9617
36.9800 -41.0300 0.0932 0.1879 -
0.1184 -0.3499 -0.3626 0.0723 0.7772
0.2526 0.3250 -41.9631
37.0000 -40.8400 0.3005 0.2196 -
0.0680 -0.3376 -0.3710 0.1287 0.7812
0.2481 0.3295 -41.9644
37.0200 -41.2000 -0.2212 0.0720 -
0.0403 -0.3067 -0.3762 0.1850 0.7849
0.2436 0.3340 -41.9658
37.0400 -41.4700 -0.3253 -0.0542 -
0.0388 -0.2597 -0.3784 0.2412 0.7884
0.2391 0.3385 -41.9672
37.0600 -40.7000 0.4077 -0.0934 -
0.0538 -0.2005 -0.3775 0.2972 0.7916
0.2344 0.3430 -41.9686
37.0800 -40.8000 0.0701 -0.1127 -
0.0715 -0.1330 -0.3738 0.3529 0.7945
0.2298 0.3474 -41.9699

37.1000	-41.3100	-0.5839	-0.0822	-
0.0750	-0.0617	-0.3674	0.4083	0.7971
	0.2250	0.3518	-41.9713	
37.1200	-40.0400	0.4931	0.0162	-
0.0496	0.0085	-0.3584	0.4633	0.7995
	0.2202	0.3562	-41.9727	
37.1400	-40.5300	-0.2510	0.0199	
0.0057	0.0732	-0.3471	0.5178	0.8015
	0.2154	0.3606	-41.9740	
37.1600	-40.2500	-0.0833	-0.0297	
0.0819	0.1281	-0.3336	0.5718	0.8033
	0.2105	0.3650	-41.9754	
37.1800	-39.9200	-0.1559	0.0238	
0.1598	0.1699	-0.3181	0.6252	0.8047
	0.2056	0.3694	-41.9767	
37.2000	-39.4800	0.2443	0.1526	
0.2207	0.1961	-0.3007	0.6779	0.8059
	0.2006	0.3737	-41.9781	
37.2200	-39.6900	-0.0789	0.1495	
0.2511	0.2056	-0.2817	0.7300	0.8067
	0.1955	0.3780	-41.9795	
37.2400	-39.3500	0.4414	-0.1007	
0.2482	0.1988	-0.2613	0.7812	0.8073
	0.1904	0.3823	-41.9808	
37.2600	-40.4000	-0.5371	-0.3315	
0.2092	0.1780	-0.2398	0.8316	0.8074
	0.1853	0.3866	-41.9822	
37.2800	-39.9300	-0.0985	-0.1778	
0.1336	0.1466	-0.2172	0.8810	0.8073
	0.1801	0.3909	-41.9835	
37.3000	-39.4100	0.0142	0.2843	
0.0261	0.1090	-0.1939	0.9295	0.8068
	0.1748	0.3951	-41.9849	
37.3200	-38.8900	0.5545	0.5523	-
0.0968	0.0696	-0.1702	0.9768	0.8060
	0.1695	0.3993	-41.9862	
37.3400	-39.4300	0.2573	0.2976	-
0.2134	0.0328	-0.1462	1.0230	0.8048
	0.1642	0.4035	-41.9875	
37.3600	-40.8300	-0.6005	-0.2506	-
0.2956	0.0028	-0.1224	1.0679	0.8033
	0.1588	0.4077	-41.9889	
37.3800	-40.1900	0.3948	-0.6236	-
0.3154	-0.0166	-0.0988	1.1116	0.8014
	0.1534	0.4119	-41.9902	
37.4000	-40.7600	-0.6162	-0.4666	-
0.2586	-0.0231	-0.0755	1.1538	0.7992
	0.1479	0.4160	-41.9915	
37.4200	-39.0500	0.4026	0.0704	-
0.1420	-0.0176	-0.0526	1.1945	0.7966
	0.1424	0.4201	-41.9929	

37.4400 -39.1500 -0.0474 0.5060 -
0.0042 -0.0023 -0.0300 1.2337 0.7936
0.1369 0.4243 -41.9942
37.4600 -38.7400 -0.0895 0.5481
0.1147 0.0196 -0.0073 1.2711 0.7902
0.1313 0.4283 -41.9955
37.4800 -38.5700 0.3371 0.2173
0.1875 0.0448 0.0156 1.3067 0.7864
0.1256 0.4324 -41.9969
37.5000 -39.3100 0.1042 -0.2475
0.2052 0.0699 0.0392 1.3404 0.7822
0.1200 0.4365 -41.9982
37.5200 -39.9600 -0.6963 -0.3982
0.1776 0.0916 0.0639 1.3720 0.7776
0.1142 0.4405 -41.9995
37.5400 -38.5700 0.3865 -0.0293
0.1200 0.1070 0.0901 1.4015 0.7726
0.1085 0.4445 -42.0008
37.5600 -38.6600 -0.0097 0.3061
0.0480 0.1135 0.1182 1.4286 0.7672
0.1027 0.4485 -42.0021
37.5800 -38.4800 0.3729 0.1503 -
0.0191 0.1092 0.1484 1.4534 0.7614
0.0969 0.4525 -42.0034
37.6000 -39.6000 -0.4927 -0.1349 -
0.0627 0.0930 0.1812 1.4757 0.7551
0.0911 0.4565 -42.0048
37.6200 -39.0200 0.2014 -0.1662 -
0.0734 0.0645 0.2167 1.4953 0.7484
0.0852 0.4604 -42.0061
37.6400 -38.7100 0.2226 -0.0220 -
0.0508 0.0250 0.2550 1.5121 0.7413
0.0793 0.4643 -42.0074
37.6600 -39.1800 -0.3931 0.0933 -
0.0040 -0.0232 0.2960 1.5260 0.7338
0.0733 0.4683 -42.0087
37.6800 -38.5600 0.3099 0.1128
0.0492 -0.0779 0.3394 1.5370 0.7258
0.0674 0.4721 -42.0100
37.7000 -38.7200 0.0571 0.0427
0.0877 -0.1363 0.3849 1.5449 0.7174
0.0614 0.4760 -42.0113
37.7200 -39.3100 -0.2644 -0.0416
0.1002 -0.1952 0.4321 1.5496 0.7085
0.0554 0.4799 -42.0126
37.7400 -38.7400 0.1695 -0.0474
0.0825 -0.2508 0.4806 1.5510 0.6992
0.0493 0.4837 -42.0139
37.7600 -39.1100 -0.1709 0.0207
0.0406 -0.2996 0.5296 1.5490 0.6895
0.0433 0.4875 -42.0152

37.7800	-38.9100	0.1054	0.0704	-
0.0148	-0.3379	0.5786	1.5436	0.6793
	0.0372	0.4913	-42.0164	
37.8000	-39.0300	0.0372	0.0668	-
0.0750	-0.3624	0.6269	1.5347	0.6687
	0.0311	0.4951	-42.0177	
37.8200	-39.3700	-0.2319	0.0299	-
0.1313	-0.3699	0.6737	1.5222	0.6577
	0.0249	0.4989	-42.0190	
37.8400	-38.7600	0.4553	-0.0543	-
0.1766	-0.3582	0.7182	1.5060	0.6463
	0.0188	0.5026	-42.0203	
37.8600	-39.6600	-0.4259	-0.1230	-
0.2038	-0.3256	0.7596	1.4860	0.6345
	0.0126	0.5064	-42.0216	
37.8800	-39.1800	0.1518	-0.0950	-
0.2115	-0.2713	0.7969	1.4622	0.6223
	0.0065	0.5101	-42.0229	
37.9000	-38.9200	0.1477	-0.0300	-
0.2037	-0.1958	0.8294	1.4346	0.6097
	0.0003	0.5138	-42.0241	
37.9200	-39.1800	-0.2317	0.0498	-
0.1893	-0.1009	0.8561	1.4031	0.5967
	-0.0059	0.5174	-42.0254	
37.9400	-38.9600	-0.1500	0.1187	-
0.1803	0.0098	0.8764	1.3677	0.5834
	-0.0121	0.5211	-42.0267	
37.9600	-38.1200	0.5015	0.1069	-
0.1876	0.1311	0.8892	1.3285	0.5698
	-0.0184	0.5247	-42.0279	
37.9800	-39.0700	-0.3512	0.0533	-
0.2112	0.2565	0.8940	1.2854	0.5558
	-0.0246	0.5284	-42.0292	
38.0000	-38.8800	-0.2468	0.0505	-
0.2309	0.3776	0.8897	1.2387	0.5415
	-0.0308	0.5320	-42.0305	
38.0200	-38.1600	0.5075	0.0301	-
0.2110	0.4855	0.8757	1.1885	0.5268
	-0.0371	0.5356	-42.0317	
38.0400	-38.7500	-0.1688	-0.1481	-
0.1195	0.5722	0.8514	1.1348	0.5119
	-0.0433	0.5391	-42.0330	
38.0600	-39.0100	-0.1474	-0.3684	
0.0522	0.6319	0.8161	1.0780	0.4967
	-0.0496	0.5427	-42.0342	
38.0800	-38.6800	-0.0695	-0.3891	
0.2878	0.6605	0.7697	1.0182	0.4812
	-0.0559	0.5462	-42.0355	
38.1000	-38.2000	0.1213	-0.2015	
0.5468	0.6556	0.7125	0.9556	0.4655
	-0.0621	0.5498	-42.0368	

38.1200 -37.8600 0.3419 0.0584
0.7724 0.6151 0.6451 0.8904 0.4495
-0.0684 0.5533 -42.0380
38.1400 -38.2100 -0.3511 0.3875
0.8977 0.5385 0.5688 0.8229 0.4333
-0.0747 0.5567 -42.0392
38.1600 -38.5500 -0.6135 0.6856
0.8687 0.4283 0.4849 0.7533 0.4168
-0.0809 0.5602 -42.0405
38.1800 -37.5600 0.9237 0.6099
0.6661 0.2915 0.3948 0.6819 0.4001
-0.0872 0.5637 -42.0417
38.2000 -39.5700 0.2608 -0.0028
0.3276 0.1379 0.3001 0.6087 0.3833
-0.0934 0.5671 -42.0430
38.2200 -42.1000 -0.8860 -0.6392 -
0.0729 -0.0218 0.2023 0.5342 0.3662
-0.0997 0.5705 -42.0442
38.2400 -41.8500 0.2680 -0.6840 -
0.4566 -0.1769 0.1029 0.4585 0.3489
-0.1059 0.5739 -42.0454
38.2600 -42.1100 0.0885 -0.2015 -
0.7539 -0.3168 0.0033 0.3818 0.3315
-0.1122 0.5773 -42.0467
38.2800 -42.1200 0.0336 0.3038 -
0.9183 -0.4309 -0.0949 0.3044 0.3140
-0.1184 0.5807 -42.0479
38.3000 -42.0500 0.1405 0.4580 -
0.9275 -0.5107 -0.1903 0.2266 0.2963
-0.1246 0.5840 -42.0491
38.3200 -42.6100 0.0725 0.1894 -
0.7891 -0.5522 -0.2813 0.1484 0.2784
-0.1309 0.5873 -42.0504
38.3400 -43.2800 -0.3500 -0.2014 -
0.5344 -0.5571 -0.3665 0.0702 0.2605
-0.1371 0.5907 -42.0516
38.3600 -43.4600 -0.3902 -0.4139 -
0.2107 -0.5303 -0.4448 -0.0078 0.2424
-0.1432 0.5940 -42.0528
38.3800 -41.5000 1.1676 -0.3228
0.1263 -0.4774 -0.5149 -0.0854 0.2242
-0.1494 0.5972 -42.0540
38.4000 -43.5400 -1.4598 0.0789
0.4147 -0.4044 -0.5761 -0.1623 0.2060
-0.1556 0.6005 -42.0552
38.4200 -39.9300 1.6601 0.4493
0.5983 -0.3174 -0.6276 -0.2384 0.1877
-0.1617 0.6038 -42.0565
38.4400 -41.9000 -0.3117 0.4324
0.6407 -0.2228 -0.6688 -0.3133 0.1693
-0.1678 0.6070 -42.0577

38.4600 -43.5900 -1.6424 0.2210
0.5465 -0.1278 -0.6991 -0.3869 0.1509
-0.1739 0.6102 -42.0589
38.4800 -41.9000 0.4308 0.0704
0.3545 -0.0396 -0.7182 -0.4590 0.1324
-0.1800 0.6134 -42.0601
38.5000 -41.4400 1.2667 -0.1180
0.1270 0.0356 -0.7258 -0.5293 0.1139
-0.1861 0.6166 -42.0613
38.5200 -44.3000 -1.1178 -0.3687 -
0.0682 0.0918 -0.7218 -0.5977 0.0954
-0.1921 0.6198 -42.0625
38.5400 -42.9600 0.2565 -0.2525 -
0.1812 0.1250 -0.7065 -0.6640 0.0769
-0.1981 0.6229 -42.0637
38.5600 -43.0800 -0.3269 0.2631 -
0.1993 0.1334 -0.6801 -0.7281 0.0585
-0.2041 0.6260 -42.0649
38.5800 -41.7800 1.0871 0.1992 -
0.1434 0.1183 -0.6435 -0.7899 0.0400
-0.2100 0.6292 -42.0661
38.6000 -44.3100 -1.1205 -0.2661 -
0.0521 0.0840 -0.5977 -0.8493 0.0216
-0.2160 0.6323 -42.0673
38.6200 -42.1600 0.8974 -0.0895
0.0311 0.0372 -0.5438 -0.9062 0.0032
-0.2219 0.6353 -42.0685
38.6400 -42.7900 0.0584 0.2346
0.0792 -0.0140 -0.4834 -0.9605 -0.0151
-0.2278 0.6384 -42.0697
38.6600 -42.7000 0.3727 -0.0072
0.0835 -0.0615 -0.4180 -1.0122 -0.0334
-0.2336 0.6415 -42.0708
38.6800 -43.7600 -0.2908 -0.3641
0.0544 -0.0991 -0.3491 -1.0612 -0.0515
-0.2394 0.6445 -42.0720
38.7000 -44.4100 -0.8319 -0.2720
0.0033 -0.1216 -0.2783 -1.1074 -0.0696
-0.2452 0.6475 -42.0732
38.7200 -42.3400 0.8221 0.2451 -
0.0607 -0.1253 -0.2071 -1.1507 -0.0875
-0.2509 0.6505 -42.0744
38.7400 -43.4500 -0.3966 0.4186 -
0.1358 -0.1087 -0.1369 -1.1911 -0.1054
-0.2566 0.6535 -42.0756
38.7600 -42.7400 0.4331 0.0747 -
0.2187 -0.0720 -0.0689 -1.2285 -0.1231
-0.2623 0.6565 -42.0767
38.7800 -44.2100 -0.5698 -0.2614 -
0.2895 -0.0180 -0.0040 -1.2628 -0.1406
-0.2679 0.6594 -42.0779

38.8000 -43.2000 0.4350 -0.3279 -
0.3128 0.0482 0.0568 -1.2940 -0.1580
-0.2735 0.6624 -42.0791
38.8200 -43.3600 0.0738 -0.1773 -
0.2634 0.1201 0.1130 -1.3221 -0.1753
-0.2790 0.6653 -42.0802
38.8400 -43.5400 -0.5793 0.0688 -
0.1387 0.1902 0.1639 -1.3469 -0.1923
-0.2845 0.6682 -42.0814
38.8600 -42.4300 0.1317 0.2056
0.0439 0.2520 0.2088 -1.3684 -0.2092
-0.2900 0.6711 -42.0826
38.8800 -41.6600 0.8526 0.0993
0.2506 0.2993 0.2472 -1.3867 -0.2259
-0.2954 0.6740 -42.0837
38.9000 -42.8500 -0.3710 -0.1930
0.4340 0.3260 0.2786 -1.4016 -0.2423
-0.3008 0.6768 -42.0849
38.9200 -43.2400 -0.8649 -0.2487
0.5345 0.3271 0.3026 -1.4132 -0.2586
-0.3061 0.6797 -42.0860
38.9400 -41.3300 0.8043 0.1663
0.5052 0.2998 0.3189 -1.4216 -0.2745
-0.3114 0.6825 -42.0872
38.9600 -42.4700 -0.4364 0.5609
0.3407 0.2464 0.3280 -1.4267 -0.2903
-0.3166 0.6853 -42.0883
38.9800 -41.7100 0.8073 0.3425
0.0712 0.1738 0.3302 -1.4285 -0.3058
-0.3218 0.6881 -42.0895
39.0000 -43.8500 -0.3637 -0.3031 -
0.2260 0.0902 0.3261 -1.4272 -0.3210
-0.3269 0.6909 -42.0906
39.0200 -44.4300 -0.2014 -0.7295 -
0.4530 0.0037 0.3164 -1.4229 -0.3359
-0.3320 0.6937 -42.0918
39.0400 -45.1700 -1.0003 -0.3181 -
0.5335 -0.0776 0.3017 -1.4155 -0.3506
-0.3370 0.6964 -42.0929
39.0600 -42.0700 1.2761 0.4255 -
0.4682 -0.1475 0.2829 -1.4054 -0.3649
-0.3419 0.6991 -42.0941
39.0800 -43.1000 0.0441 0.5073 -
0.2906 -0.2021 0.2606 -1.3924 -0.3790
-0.3468 0.7019 -42.0952
39.1000 -44.6100 -0.9556 0.0437 -
0.0553 -0.2399 0.2355 -1.3769 -0.3927
-0.3517 0.7046 -42.0963
39.1200 -43.1700 0.5673 -0.3445
0.1667 -0.2600 0.2084 -1.3589 -0.4062
-0.3565 0.7073 -42.0975

39.1400 -43.6900 -0.0586 -0.2240
0.3113 -0.2615 0.1799 -1.3386 -0.4193
-0.3612 0.7099 -42.0986
39.1600 -43.4500 -0.4466 0.1679
0.3515 -0.2446 0.1504 -1.3160 -0.4320
-0.3658 0.7126 -42.0997
39.1800 -42.3000 0.5231 0.4247
0.2947 -0.2110 0.1205 -1.2915 -0.4445
-0.3704 0.7152 -42.1008
39.2000 -43.0100 0.2112 0.2507
0.1674 -0.1642 0.0904 -1.2650 -0.4566
-0.3750 0.7179 -42.1020
39.2200 -44.1600 -0.6379 -0.1594
0.0158 -0.1091 0.0604 -1.2367 -0.4684
-0.3794 0.7205 -42.1031
39.2400 -43.4400 0.5644 -0.3509 -
0.1110 -0.0508 0.0308 -1.2068 -0.4799
-0.3838 0.7231 -42.1042
39.2600 -44.2300 -0.5177 -0.1981 -
0.1767 0.0062 0.0017 -1.1753 -0.4910
-0.3882 0.7257 -42.1053
39.2800 -43.3900 0.0288 0.0413 -
0.1765 0.0591 -0.0267 -1.1425 -0.5018
-0.3924 0.7282 -42.1064
39.3000 -42.5800 0.8017 0.1446 -
0.1251 0.1061 -0.0542 -1.1083 -0.5122
-0.3966 0.7308 -42.1075
39.3200 -44.1900 -0.9235 0.0804 -
0.0453 0.1458 -0.0805 -1.0730 -0.5223
-0.4007 0.7333 -42.1086
39.3400 -42.3700 0.9247 -0.0128
0.0352 0.1775 -0.1056 -1.0366 -0.5321
-0.4048 0.7358 -42.1097
39.3600 -43.5200 -0.2414 -0.1282
0.0951 0.2009 -0.1294 -0.9993 -0.5415
-0.4088 0.7383 -42.1108
39.3800 -43.7600 -0.5477 -0.1276
0.1183 0.2158 -0.1518 -0.9611 -0.5506
-0.4127 0.7408 -42.1119
39.4000 -43.5900 -0.7792 0.2292
0.0956 0.2229 -0.1729 -0.9221 -0.5594
-0.4165 0.7433 -42.1130
39.4200 -41.9000 0.6060 0.5936
0.0352 0.2235 -0.1926 -0.8825 -0.5679
-0.4203 0.7458 -42.1141
39.4400 -41.6700 1.0600 0.4109 -
0.0436 0.2190 -0.2108 -0.8423 -0.5760
-0.4240 0.7482 -42.1152
39.4600 -44.6600 -0.9495 -0.3025 -
0.1129 0.2110 -0.2274 -0.8017 -0.5839
-0.4276 0.7507 -42.1163

39.4800 -44.1600 0.0237 -0.8133 -
0.1436 0.2010 -0.2422 -0.7606 -0.5914
-0.4311 0.7531 -42.1174
39.5000 -44.3700 -0.2509 -0.6723 -
0.1167 0.1891 -0.2547 -0.7193 -0.5986
-0.4345 0.7555 -42.1185
39.5200 -43.4000 -0.0889 -0.0592 -
0.0448 0.1743 -0.2645 -0.6778 -0.6055
-0.4379 0.7579 -42.1196
39.5400 -42.2300 0.3540 0.5870
0.0392 0.1547 -0.2710 -0.6363 -0.6122
-0.4412 0.7602 -42.1207
39.5600 -42.5600 -0.2678 0.8370
0.1003 0.1287 -0.2738 -0.5947 -0.6185
-0.4444 0.7626 -42.1217
39.5800 -41.7100 0.9694 0.3146
0.1199 0.0947 -0.2724 -0.5532 -0.6245
-0.4475 0.7649 -42.1228
39.6000 -44.6900 -1.0915 -0.5395
0.1072 0.0507 -0.2663 -0.5119 -0.6302
-0.4506 0.7673 -42.1239
39.6200 -43.4500 0.2568 -0.6677
0.0813 -0.0041 -0.2553 -0.4708 -0.6357
-0.4535 0.7696 -42.1250
39.6400 -43.0100 0.1618 -0.1237
0.0636 -0.0694 -0.2393 -0.4299 -0.6408
-0.4564 0.7719 -42.1260
39.6600 -42.3500 0.4806 0.3410
0.0720 -0.1430 -0.2182 -0.3894 -0.6457
-0.4592 0.7742 -42.1271
39.6800 -42.5000 0.3464 0.2560
0.1141 -0.2209 -0.1923 -0.3491 -0.6503
-0.4619 0.7765 -42.1282
39.7000 -43.1100 0.0962 -0.1972
0.1652 -0.2981 -0.1617 -0.3093 -0.6546
-0.4645 0.7787 -42.1292
39.7200 -44.2500 -0.8915 -0.3868
0.1910 -0.3687 -0.1268 -0.2698 -0.6586
-0.4671 0.7810 -42.1303
39.7400 -42.1500 0.9593 -0.0466
0.1633 -0.4263 -0.0878 -0.2309 -0.6624
-0.4695 0.7832 -42.1313
39.7600 -43.6600 -0.9037 0.3461
0.0690 -0.4652 -0.0451 -0.1923 -0.6659
-0.4719 0.7854 -42.1324
39.7800 -42.3100 0.4512 0.3887 -
0.0888 -0.4808 0.0008 -0.1544 -0.6691
-0.4742 0.7876 -42.1334
39.8000 -42.8600 0.3521 0.0878 -
0.2829 -0.4699 0.0496 -0.1170 -0.6721
-0.4764 0.7898 -42.1345

39.8200	-44.0800	-0.4613	-0.2869	-
0.4645	-0.4313	0.1007	-0.0802	-0.6748
	-0.4785	0.7920	-42.1355	
39.8400	-43.5300	0.1120	-0.3800	-
0.5866	-0.3645	0.1531	-0.0441	-0.6772
	-0.4806	0.7941	-42.1366	
39.8600	-43.5300	-0.1912	-0.1329	-
0.6197	-0.2712	0.2061	-0.0087	-0.6794
	-0.4825	0.7963	-42.1376	
39.8800	-42.6000	0.2152	0.1805	-
0.5656	-0.1552	0.2587	0.0259	-0.6813
	-0.4844	0.7984	-42.1387	
39.9000	-42.2100	0.1357	0.2489	-
0.4420	-0.0224	0.3099	0.0597	-0.6829
	-0.4861	0.8005	-42.1397	
39.9200	-42.2000	0.0031	0.0388	-
0.2732	0.1208	0.3588	0.0926	-0.6843
	-0.4878	0.8026	-42.1407	
39.9400	-42.1700	-0.2683	-0.1404	-
0.0864	0.2670	0.4042	0.1246	-0.6854
	-0.4894	0.8047	-42.1418	
39.9600	-41.6300	-0.1056	-0.0821	
0.0981	0.4085	0.4452	0.1556	-0.6863
	-0.4910	0.8068	-42.1428	
39.9800	-40.8400	0.0592	0.0538	
0.2727	0.5364	0.4808	0.1856	-0.6869
	-0.4924	0.8089	-42.1438	
40.0000	-40.3300	0.4382	0.0093	
0.4369	0.6418	0.5101	0.2145	-0.6873
	-0.4938	0.8109	-42.1449	
40.0200	-40.8400	-0.3406	-0.2171	
0.5856	0.7164	0.5320	0.2423	-0.6874
	-0.4950	0.8130	-42.1459	
40.0400	-40.7100	-0.0045	-0.3012	
0.7003	0.7534	0.5455	0.2690	-0.6873
	-0.4962	0.8150	-42.1469	
40.0600	-40.4900	-0.1402	-0.1222	
0.7536	0.7490	0.5499	0.2944	-0.6870
	-0.4973	0.8170	-42.1479	
40.0800	-39.6800	0.4862	0.1951	
0.7164	0.7024	0.5449	0.3186	-0.6864
	-0.4983	0.8190	-42.1489	
40.1000	-40.5800	-0.7540	0.5235	
0.5627	0.6175	0.5308	0.3416	-0.6855
	-0.4992	0.8210	-42.1499	
40.1200	-39.4300	0.7674	0.5657	
0.2977	0.5023	0.5079	0.3634	-0.6844
	-0.5001	0.8229	-42.1510	
40.1400	-40.8500	0.3476	0.1596	-
0.0356	0.3667	0.4768	0.3839	-0.6831
	-0.5008	0.8249	-42.1520	

40.1600 -42.5400 -0.2754 -0.4182 -
0.3607 0.2214 0.4380 0.4032 -0.6816
-0.5015 0.8269 -42.1530
40.1800 -43.7100 -0.8005 -0.6375 -
0.5968 0.0767 0.3918 0.4214 -0.6799
-0.5021 0.8288 -42.1540
40.2000 -42.5700 0.3215 -0.3443 -
0.6895 -0.0571 0.3389 0.4385 -0.6779
-0.5026 0.8307 -42.1550
40.2200 -42.1100 0.5138 0.0479 -
0.6429 -0.1713 0.2796 0.4545 -0.6757
-0.5030 0.8326 -42.1560
40.2400 -42.5100 -0.2374 0.2338 -
0.4952 -0.2595 0.2145 0.4694 -0.6733
-0.5033 0.8345 -42.1570
40.2600 -42.0700 0.1094 0.2184 -
0.3021 -0.3176 0.1442 0.4834 -0.6708
-0.5035 0.8364 -42.1580
40.2800 -42.5000 -0.2315 0.1713 -
0.1165 -0.3440 0.0691 0.4964 -0.6680
-0.5037 0.8382 -42.1589
40.3000 -42.1300 0.1027 0.1015
0.0260 -0.3389 -0.0097 0.5084 -0.6650
-0.5037 0.8401 -42.1599
40.3200 -41.9800 0.1662 0.0035
0.1127 -0.3051 -0.0914 0.5196 -0.6619
-0.5037 0.8419 -42.1609
40.3400 -42.4000 -0.1855 -0.0296
0.1426 -0.2482 -0.1746 0.5300 -0.6586
-0.5036 0.8438 -42.1619
40.3600 -42.3800 -0.1208 0.0816
0.1278 -0.1763 -0.2582 0.5396 -0.6551
-0.5034 0.8456 -42.1629
40.3800 -41.7700 0.3338 0.1799
0.0905 -0.0992 -0.3406 0.5485 -0.6514
-0.5032 0.8474 -42.1639
40.4000 -42.4300 -0.2308 0.0481
0.0592 -0.0270 -0.4206 0.5566 -0.6476
-0.5028 0.8492 -42.1648
40.4200 -42.2500 0.3037 -0.1957
0.0576 0.0304 -0.4968 0.5641 -0.6437
-0.5023 0.8510 -42.1658
40.4400 -43.0000 -0.4318 -0.2763
0.0981 0.0650 -0.5679 0.5710 -0.6396
-0.5018 0.8527 -42.1668
40.4600 -42.0700 0.2667 -0.1177
0.1723 0.0719 -0.6330 0.5773 -0.6353
-0.5012 0.8545 -42.1678
40.4800 -42.2900 -0.2028 0.1345
0.2539 0.0485 -0.6911 0.5831 -0.6310
-0.5005 0.8562 -42.1687

40.5000	-41.7900	0.1600	0.3169	
0.3061	-0.0056	-0.7417	0.5885	-0.6265
	-0.4997	0.8580	-42.1697	
40.5200	-41.9000	0.2020	0.3107	
0.2992	-0.0882	-0.7840	0.5934	-0.6218
	-0.4988	0.8597	-42.1707	
40.5400	-42.4400	-0.0484	0.2000	
0.2290	-0.1939	-0.8178	0.5979	-0.6171
	-0.4979	0.8614	-42.1716	
40.5600	-43.4100	-0.6449	0.1278	
0.1134	-0.3147	-0.8426	0.6022	-0.6122
	-0.4968	0.8631	-42.1726	
40.5800	-42.9800	0.1329	-0.0097	-
0.0045	-0.4414	-0.8582	0.6062	-0.6073
	-0.4957	0.8648	-42.1735	
40.6000	-42.5900	1.1076	-0.3537	-
0.0840	-0.5648	-0.8642	0.6099	-0.6022
	-0.4945	0.8665	-42.1745	
40.6200	-45.4100	-1.3387	-0.5814	-
0.1146	-0.6763	-0.8602	0.6134	-0.5971
	-0.4932	0.8681	-42.1754	
40.6400	-42.5700	1.0367	-0.1261	-
0.1075	-0.7683	-0.8461	0.6168	-0.5919
	-0.4918	0.8698	-42.1764	
40.6600	-42.4900	0.5668	0.4469	-
0.1020	-0.8351	-0.8214	0.6200	-0.5865
	-0.4904	0.8714	-42.1773	
40.6800	-42.8500	0.2397	0.4870	-
0.1306	-0.8728	-0.7862	0.6231	-0.5812
	-0.4888	0.8731	-42.1783	
40.7000	-44.2900	-0.9779	0.2692	-
0.1857	-0.8797	-0.7405	0.6262	-0.5757
	-0.4872	0.8747	-42.1792	
40.7200	-42.5300	0.9272	-0.0248	-
0.2279	-0.8572	-0.6846	0.6291	-0.5702
	-0.4855	0.8763	-42.1801	
40.7400	-44.5300	-0.6652	-0.4573	-
0.1991	-0.8086	-0.6191	0.6319	-0.5646
	-0.4837	0.8779	-42.1811	
40.7600	-43.8100	-0.1161	-0.5721	-
0.0782	-0.7394	-0.5449	0.6347	-0.5590
	-0.4818	0.8795	-42.1820	
40.7800	-43.2100	-0.4960	-0.0286	
0.1022	-0.6558	-0.4626	0.6373	-0.5533
	-0.4798	0.8811	-42.1829	
40.8000	-41.0500	0.9159	0.4483	
0.2593	-0.5638	-0.3733	0.6399	-0.5476
	-0.4778	0.8826	-42.1839	
40.8200	-42.6100	-0.8355	0.3034	
0.3109	-0.4690	-0.2778	0.6424	-0.5419
	-0.4757	0.8842	-42.1848	

40.8400 -41.4700 0.6723 0.0063
0.2271 -0.3760 -0.1769 0.6449 -0.5361
-0.4735 0.8857 -42.1857
40.8600 -42.6500 -0.4558 -0.1466
0.0433 -0.2867 -0.0717 0.6473 -0.5303
-0.4712 0.8873 -42.1866
40.8800 -41.9500 0.2467 -0.2226 -
0.1605 -0.2017 0.0369 0.6496 -0.5245
-0.4688 0.8888 -42.1876
40.9000 -42.3700 -0.3325 -0.1759 -
0.3063 -0.1214 0.1478 0.6518 -0.5187
-0.4663 0.8903 -42.1885
40.9200 -41.2900 0.4343 -0.0698 -
0.3481 -0.0456 0.2600 0.6540 -0.5128
-0.4638 0.8918 -42.1894
40.9400 -42.1700 -0.6191 -0.0020 -
0.2826 0.0252 0.3724 0.6561 -0.5070
-0.4612 0.8933 -42.1903
40.9600 -40.4200 0.7076 0.0086 -
0.1359 0.0906 0.4840 0.6582 -0.5012
-0.4585 0.8948 -42.1912
40.9800 -41.1600 -0.4451 -0.0144
0.0499 0.1502 0.5936 0.6602 -0.4954
-0.4557 0.8963 -42.1921
41.0000 -40.8100 -0.3784 0.0110
0.2283 0.2043 0.7001 0.6622 -0.4896
-0.4529 0.8978 -42.1930
41.0200 -39.6000 0.5944 0.0588
0.3589 0.2536 0.8024 0.6641 -0.4838
-0.4499 0.8992 -42.1939
41.0400 -39.8200 0.2010 0.0308
0.4119 0.2986 0.8994 0.6660 -0.4780
-0.4469 0.9007 -42.1948
41.0600 -40.3500 -0.5531 0.0217
0.3726 0.3397 0.9898 0.6678 -0.4722
-0.4438 0.9021 -42.1957
41.0800 -39.5900 0.1386 0.1135
0.2454 0.3780 1.0724 0.6695 -0.4665
-0.4406 0.9036 -42.1966
41.1000 -39.2400 0.5095 0.1489
0.0552 0.4153 1.1463 0.6712 -0.4607
-0.4374 0.9050 -42.1975
41.1200 -40.3500 -0.4136 -0.0219 -
0.1582 0.4533 1.2102 0.6729 -0.4551
-0.4340 0.9064 -42.1984
41.1400 -39.7100 0.6431 -0.3079 -
0.3448 0.4934 1.2632 0.6745 -0.4494
-0.4306 0.9078 -42.1993
41.1600 -41.2900 -0.9323 -0.2675 -
0.4620 0.5371 1.3044 0.6761 -0.4438
-0.4272 0.9092 -42.2001

41.1800 -39.2300 0.7085 0.1264 -
0.4943 0.5853 1.3329 0.6776 -0.4382
-0.4236 0.9106 -42.2010
41.2000 -39.3700 0.2618 0.2979 -
0.4577 0.6378 1.3480 0.6790 -0.4326
-0.4200 0.9120 -42.2019
41.2200 -40.1200 -0.3844 0.0766 -
0.3788 0.6932 1.3492 0.6804 -0.4271
-0.4162 0.9134 -42.2028
41.2400 -39.8400 0.0061 -0.1418 -
0.2834 0.7487 1.3360 0.6818 -0.4216
-0.4125 0.9147 -42.2037
41.2600 -39.9600 -0.5402 0.0574 -
0.1841 0.7999 1.3082 0.6831 -0.4161
-0.4086 0.9161 -42.2045
41.2800 -38.3600 0.7098 0.2919 -
0.0645 0.8405 1.2659 0.6843 -0.4107
-0.4047 0.9174 -42.2054
41.3000 -39.4700 -0.2511 0.0355
0.0957 0.8640 1.2093 0.6856 -0.4054
-0.4007 0.9188 -42.2063
41.3200 -39.4100 -0.0200 -0.4409
0.2893 0.8646 1.1389 0.6868 -0.4001
-0.3966 0.9201 -42.2071
41.3400 -39.9500 -0.3496 -0.5924
0.4851 0.8381 1.0555 0.6881 -0.3948
-0.3924 0.9215 -42.2080
41.3600 -38.9500 0.2704 -0.2585
0.6436 0.7816 0.9602 0.6893 -0.3896
-0.3882 0.9228 -42.2088
41.3800 -38.8300 -0.1954 0.3165
0.7243 0.6935 0.8541 0.6905 -0.3845
-0.3839 0.9241 -42.2097
41.4000 -38.5700 0.1286 0.7395
0.6882 0.5747 0.7385 0.6918 -0.3794
-0.3796 0.9254 -42.2106
41.4200 -38.3600 0.6539 0.7109
0.5105 0.4308 0.6148 0.6931 -0.3743
-0.3751 0.9267 -42.2114
41.4400 -39.8000 0.3609 0.1912
0.2213 0.2702 0.4845 0.6945 -0.3694
-0.3706 0.9280 -42.2123
41.4600 -41.7800 -0.4746 -0.4028 -
0.1106 0.1021 0.3491 0.6958 -0.3644
-0.3661 0.9293 -42.2131
41.4800 -43.2300 -1.0887 -0.5801 -
0.4095 -0.0643 0.2098 0.6973 -0.3596
-0.3614 0.9306 -42.2139
41.5000 -42.2600 0.0733 -0.2866 -
0.6104 -0.2200 0.0683 0.6988 -0.3548
-0.3567 0.9318 -42.2148

41.5200	-41.3000	1.1627	-0.0027	-
0.6752	-0.3565	-0.0740	0.7003	-0.3500
	-0.3519	0.9331	-42.2156	
41.5400	-43.5000	-0.7399	-0.0829	-
0.5947	-0.4674	-0.2158	0.7020	-0.3453
	-0.3471	0.9344	-42.2165	
41.5600	-42.7600	-0.0861	-0.1953	-
0.4030	-0.5497	-0.3555	0.7037	-0.3407
	-0.3422	0.9356	-42.2173	
41.5800	-42.7000	-0.1623	-0.0123	-
0.1599	-0.6026	-0.4919	0.7054	-0.3361
	-0.3372	0.9369	-42.2181	
41.6000	-41.7600	0.3786	0.2976	
0.0754	-0.6267	-0.6237	0.7073	-0.3316
	-0.3322	0.9381	-42.2190	
41.6200	-42.3100	-0.3311	0.3861	
0.2527	-0.6236	-0.7500	0.7093	-0.3272
	-0.3271	0.9394	-42.2198	
41.6400	-41.6600	0.6948	0.0614	
0.3410	-0.5966	-0.8699	0.7113	-0.3228
	-0.3220	0.9406	-42.2206	
41.6600	-43.6100	-0.9513	-0.2703	
0.3368	-0.5504	-0.9826	0.7134	-0.3185
	-0.3168	0.9418	-42.2214	
41.6800	-42.9200	-0.2691	-0.1229	
0.2531	-0.4909	-1.0875	0.7157	-0.3143
	-0.3115	0.9430	-42.2222	
41.7000	-41.9200	0.4860	0.2413	
0.1215	-0.4243	-1.1841	0.7180	-0.3101
	-0.3062	0.9443	-42.2231	
41.7200	-41.6900	0.8142	0.2763	-
0.0186	-0.3567	-1.2717	0.7205	-0.3060
	-0.3008	0.9455	-42.2239	
41.7400	-43.7500	-0.7384	-0.1113	-
0.1263	-0.2929	-1.3500	0.7231	-0.3019
	-0.2954	0.9467	-42.2247	
41.7600	-43.2900	0.1894	-0.4685	-
0.1748	-0.2370	-1.4184	0.7258	-0.2979
	-0.2899	0.9479	-42.2255	
41.7800	-43.5900	-0.2838	-0.3949	-
0.1533	-0.1919	-1.4768	0.7286	-0.2940
	-0.2843	0.9491	-42.2263	
41.8000	-42.8600	-0.1023	0.0090	-
0.0766	-0.1590	-1.5249	0.7316	-0.2901
	-0.2787	0.9503	-42.2271	
41.8200	-42.2500	0.1469	0.3995	
0.0217	-0.1383	-1.5627	0.7347	-0.2863
	-0.2731	0.9514	-42.2279	
41.8400	-41.8100	0.3985	0.4615	
0.1071	-0.1291	-1.5903	0.7379	-0.2826
	-0.2674	0.9526	-42.2287	

41.8600 -42.5400 -0.0159 0.1131
0.1552 -0.1303 -1.6077 0.7413 -0.2789
-0.2616 0.9538 -42.2295
41.8800 -43.0700 -0.2166 -0.3050
0.1601 -0.1410 -1.6154 0.7448 -0.2753
-0.2559 0.9550 -42.2303
41.9000 -43.6700 -0.5657 -0.3639
0.1242 -0.1597 -1.6138 0.7484 -0.2717
-0.2500 0.9562 -42.2311
41.9200 -42.5000 0.2211 -0.0365
0.0582 -0.1841 -1.6035 0.7522 -0.2682
-0.2441 0.9573 -42.2319
41.9400 -42.0300 0.6070 0.2670 -
0.0220 -0.2112 -1.5850 0.7561 -0.2648
-0.2382 0.9585 -42.2327
41.9600 -42.8400 0.0242 0.2140 -
0.0975 -0.2380 -1.5590 0.7601 -0.2613
-0.2322 0.9596 -42.2335
41.9800 -43.1800 -0.2027 -0.0621 -
0.1488 -0.2618 -1.5263 0.7642 -0.2580
-0.2262 0.9608 -42.2342
42.0000 -43.9500 -0.9568 -0.1378 -
0.1631 -0.2800 -1.4876 0.7683 -0.2547
-0.2201 0.9619 -42.2350
42.0200 -41.9800 0.7961 0.0331 -
0.1340 -0.2908 -1.4439 0.7724 -0.2514
-0.2140 0.9631 -42.2358
42.0400 -42.1100 0.5238 0.0597 -
0.0622 -0.2933 -1.3956 0.7765 -0.2482
-0.2079 0.9642 -42.2366
42.0600 -43.4900 -0.7553 -0.1428
0.0398 -0.2871 -1.3436 0.7806 -0.2450
-0.2017 0.9654 -42.2373
42.0800 -42.2400 0.2925 -0.2488
0.1458 -0.2726 -1.2883 0.7847 -0.2418
-0.1955 0.9665 -42.2381
42.1000 -42.6500 -0.2748 -0.0273
0.2227 -0.2503 -1.2303 0.7887 -0.2387
-0.1893 0.9676 -42.2389
42.1200 -41.5100 0.3284 0.2338
0.2351 -0.2208 -1.1702 0.7926 -0.2356
-0.1830 0.9688 -42.2396
42.1400 -41.7300 0.0366 0.2453
0.1648 -0.1849 -1.1084 0.7965 -0.2325
-0.1767 0.9699 -42.2404
42.1600 -42.1300 -0.0701 0.0612
0.0260 -0.1434 -1.0454 0.8001 -0.2294
-0.1703 0.9710 -42.2412
42.1800 -42.2700 -0.1332 -0.0597 -
0.1386 -0.0975 -0.9816 0.8037 -0.2264
-0.1640 0.9722 -42.2419

42.2000 -42.1700 -0.0258 -0.0676 -
0.2734 -0.0488 -0.9174 0.8070 -0.2234
-0.1576 0.9733 -42.2427
42.2200 -42.0300 0.1018 -0.1551 -
0.3263 0.0005 -0.8531 0.8102 -0.2204
-0.1511 0.9744 -42.2434
42.2400 -41.7300 0.4351 -0.3476 -
0.2733 0.0479 -0.7892 0.8132 -0.2174
-0.1447 0.9755 -42.2442
42.2600 -42.6700 -0.9625 -0.2453 -
0.1317 0.0905 -0.7256 0.8159 -0.2144
-0.1382 0.9766 -42.2449
42.2800 -40.5600 0.5052 0.2264
0.0516 0.1247 -0.6627 0.8183 -0.2115
-0.1317 0.9777 -42.2457
42.3000 -39.5700 0.9047 0.4853
0.2189 0.1472 -0.6006 0.8204 -0.2085
-0.1252 0.9789 -42.2464
42.3200 -41.4900 -0.8648 0.1725
0.3224 0.1546 -0.5392 0.8222 -0.2056
-0.1187 0.9800 -42.2471
42.3400 -40.6400 0.3501 -0.2576
0.3515 0.1447 -0.4787 0.8236 -0.2026
-0.1122 0.9811 -42.2479
42.3600 -41.2400 -0.2462 -0.2777
0.3127 0.1161 -0.4190 0.8245 -0.1996
-0.1056 0.9822 -42.2486
42.3800 -40.7600 0.0741 -0.0286
0.2204 0.0698 -0.3598 0.8250 -0.1967
-0.0990 0.9833 -42.2493
42.4000 -40.8000 -0.1673 0.2307
0.0935 0.0092 -0.3011 0.8250 -0.1937
-0.0924 0.9844 -42.2501
42.4200 -40.2800 0.5540 0.2381 -
0.0437 -0.0606 -0.2424 0.8245 -0.1907
-0.0858 0.9855 -42.2508
42.4400 -41.6300 -0.3215 -0.0762 -
0.1672 -0.1344 -0.1835 0.8233 -0.1877
-0.0792 0.9866 -42.2515
42.4600 -41.7500 -0.1162 -0.3377 -
0.2523 -0.2068 -0.1240 0.8216 -0.1847
-0.0726 0.9877 -42.2522
42.4800 -41.8600 -0.2802 -0.2443 -
0.2811 -0.2728 -0.0636 0.8191 -0.1817
-0.0660 0.9888 -42.2530
42.5000 -41.1200 0.1564 0.0526 -
0.2527 -0.3282 -0.0021 0.8159 -0.1787
-0.0594 0.9899 -42.2537
42.5200 -40.6100 0.3994 0.1961 -
0.1888 -0.3699 0.0610 0.8119 -0.1756
-0.0527 0.9910 -42.2544

42.5400 -41.0200 0.0232 0.0894 -
0.1161 -0.3958 0.1257 0.8071 -0.1726
-0.0461 0.9921 -42.2551
42.5600 -41.6500 -0.6654 -0.0072 -
0.0593 -0.4051 0.1925 0.8014 -0.1695
-0.0395 0.9932 -42.2558
42.5800 -40.9400 -0.1698 0.0640 -
0.0291 -0.3983 0.2615 0.7949 -0.1664
-0.0328 0.9943 -42.2565
42.6000 -39.9700 0.6416 0.1424 -
0.0129 -0.3776 0.3326 0.7873 -0.1633
-0.0262 0.9954 -42.2572
42.6200 -40.3900 0.0809 0.0322
0.0063 -0.3463 0.4059 0.7788 -0.1602
-0.0196 0.9965 -42.2579
42.6400 -41.0300 -0.5096 -0.1467
0.0388 -0.3072 0.4810 0.7693 -0.1570
-0.0130 0.9976 -42.2586
42.6600 -40.6400 -0.3187 -0.1087
0.0866 -0.2635 0.5579 0.7587 -0.1538
-0.0063 0.9987 -42.2593
42.6800 -39.6700 0.3126 0.1043
0.1351 -0.2179 0.6360 0.7471 -0.1506
0.0003 0.9998 -42.2600
42.7000 -39.2900 0.4250 0.1916
0.1614 -0.1729 0.7150 0.7343 -0.1474
0.0069 1.0009 -42.2607
42.7200 -40.4000 -0.6088 0.0910
0.1513 -0.1308 0.7943 0.7203 -0.1442
0.0134 1.0020 -42.2614
42.7400 -39.3900 0.4180 -0.0010
0.1064 -0.0930 0.8736 0.7051 -0.1409
0.0200 1.0031 -42.2621
42.7600 -39.6600 0.2601 -0.0818
0.0424 -0.0596 0.9521 0.6888 -0.1377
0.0266 1.0042 -42.2628
42.7800 -40.2200 -0.3667 -0.1328 -
0.0236 -0.0299 1.0294 0.6711 -0.1344
0.0331 1.0053 -42.2634
42.8000 -39.5400 0.0518 -0.0282 -
0.0777 -0.0025 1.1046 0.6522 -0.1310
0.0396 1.0064 -42.2641
42.8200 -39.4300 0.1202 0.0824 -
0.1107 0.0246 1.1771 0.6319 -0.1277
0.0461 1.0075 -42.2648
42.8400 -39.5300 -0.0012 0.0297 -
0.1248 0.0536 1.2461 0.6102 -0.1243
0.0526 1.0086 -42.2655
42.8600 -39.4800 0.0527 -0.1084 -
0.1265 0.0857 1.3109 0.5872 -0.1209
0.0591 1.0097 -42.2661

42.8800 -39.8300 -0.3861 -0.0616 -
0.1198 0.1218 1.3705 0.5627 -0.1175
0.0655 1.0109 -42.2668
42.9000 -38.6700 0.3669 0.1767 -
0.1065 0.1622 1.4242 0.5368 -0.1141
0.0719 1.0120 -42.2675
42.9200 -38.9900 -0.0208 0.2348 -
0.0854 0.2067 1.4712 0.5094 -0.1107
0.0783 1.0131 -42.2681
42.9400 -39.0500 0.1234 -0.0394 -
0.0516 0.2547 1.5107 0.4805 -0.1072
0.0847 1.0142 -42.2688
42.9600 -39.6400 -0.3582 -0.3091
0.0034 0.3047 1.5420 0.4500 -0.1037
0.0910 1.0153 -42.2695
42.9800 -39.3600 -0.2581 -0.2519
0.0811 0.3555 1.5645 0.4180 -0.1002
0.0973 1.0164 -42.2701
43.0000 -38.4800 0.2341 0.0331
0.1672 0.4060 1.5776 0.3845 -0.0966
0.1036 1.0176 -42.2708
43.0200 -38.0400 0.4353 0.2342
0.2352 0.4557 1.5807 0.3494 -0.0930
0.1098 1.0187 -42.2714
43.0400 -38.6300 -0.3540 0.2383
0.2587 0.5041 1.5734 0.3129 -0.0894
0.1160 1.0198 -42.2721
43.0600 -38.7200 -0.4147 0.1793
0.2230 0.5503 1.5557 0.2749 -0.0857
0.1221 1.0209 -42.2727
43.0800 -38.0500 0.5164 0.1239
0.1289 0.5936 1.5274 0.2355 -0.0820
0.1283 1.0221 -42.2734
43.1000 -38.8000 0.0902 0.0480 -
0.0103 0.6331 1.4886 0.1949 -0.0783
0.1343 1.0232 -42.2740
43.1200 -39.5500 -0.4559 0.0065 -
0.1701 0.6674 1.4395 0.1530 -0.0745
0.1404 1.0243 -42.2746
43.1400 -38.8400 0.3973 0.0208 -
0.3105 0.6952 1.3805 0.1100 -0.0707
0.1464 1.0255 -42.2753
43.1600 -39.5300 -0.0206 -0.1248 -
0.3881 0.7145 1.3120 0.0660 -0.0668
0.1523 1.0266 -42.2759
43.1800 -39.7500 0.1682 -0.4228 -
0.3751 0.7230 1.2346 0.0211 -0.0628
0.1583 1.0278 -42.2765
43.2000 -40.7500 -0.5833 -0.5010 -
0.2636 0.7181 1.1491 -0.0247 -0.0588
0.1641 1.0289 -42.2772

43.2200 -39.4600 0.2942 -0.2488 -
0.0706 0.6970 1.0564 -0.0712 -0.0547
0.1699 1.0301 -42.2778
43.2400 -38.9500 0.4894 0.0865
0.1561 0.6571 0.9573 -0.1184 -0.0506
0.1757 1.0313 -42.2784
43.2600 -39.6700 -0.6085 0.3575
0.3564 0.5962 0.8530 -0.1661 -0.0463
0.1814 1.0324 -42.2790
43.2800 -39.3200 -0.3466 0.5614
0.4739 0.5125 0.7444 -0.2144 -0.0420
0.1871 1.0336 -42.2797
43.3000 -38.4600 0.7480 0.5716
0.4795 0.4054 0.6327 -0.2629 -0.0377
0.1927 1.0348 -42.2803
43.3200 -39.9300 0.0887 0.2251
0.3815 0.2773 0.5188 -0.3118 -0.0332
0.1983 1.0359 -42.2809
43.3400 -41.0700 -0.1903 -0.3178
0.2197 0.1325 0.4038 -0.3609 -0.0287
0.2038 1.0371 -42.2815
43.3600 -42.4600 -0.8437 -0.5376
0.0430 -0.0240 0.2887 -0.4100 -0.0240
0.2093 1.0383 -42.2821
43.3800 -41.4600 0.3793 -0.2489 -
0.1035 -0.1870 0.1745 -0.4592 -0.0193
0.2147 1.0395 -42.2827
43.4000 -41.4900 0.3964 0.0788 -
0.1965 -0.3506 0.0624 -0.5083 -0.0145
0.2200 1.0407 -42.2833
43.4200 -42.0000 0.3318 0.0679 -
0.2280 -0.5079 -0.0468 -0.5572 -0.0096
0.2253 1.0419 -42.2839
43.4400 -42.9800 -0.2445 -0.1506 -
0.2108 -0.6514 -0.1520 -0.6058 -0.0046
0.2305 1.0431 -42.2845
43.4600 -43.8400 -0.6238 -0.2862 -
0.1746 -0.7731 -0.2522 -0.6540 0.0006
0.2357 1.0443 -42.2851
43.4800 -42.6200 0.6683 -0.1508 -
0.1461 -0.8653 -0.3464 -0.7019 0.0058
0.2408 1.0455 -42.2857
43.5000 -43.8300 -0.6954 0.2246 -
0.1451 -0.9216 -0.4335 -0.7491 0.0111
0.2458 1.0467 -42.2863
43.5200 -42.3300 0.6711 0.4764 -
0.1791 -0.9377 -0.5126 -0.7958 0.0166
0.2508 1.0479 -42.2869
43.5400 -43.6000 -0.3934 0.2621 -
0.2419 -0.9115 -0.5831 -0.8416 0.0221
0.2557 1.0492 -42.2875

43.5600	-43.7900	-0.0452	-0.1840	-
0.3078	-0.8429	-0.6446	-0.8867	0.0278
	0.2605	1.0504	-42.2880	
43.5800	-43.9900	0.1036	-0.4437	-
0.3372	-0.7343	-0.6973	-0.9308	0.0336
	0.2653	1.0516	-42.2886	
43.6000	-44.1600	-0.1541	-0.3892	-
0.2956	-0.5907	-0.7415	-0.9739	0.0396
	0.2700	1.0529	-42.2892	
43.6200	-43.3600	0.2405	-0.1392	-
0.1846	-0.4201	-0.7779	-1.0159	0.0456
	0.2746	1.0541	-42.2898	
43.6400	-43.2000	-0.2706	0.1046	-
0.0363	-0.2326	-0.8068	-1.0566	0.0518
	0.2792	1.0554	-42.2903	
43.6600	-42.0800	0.4091	0.2163	
0.1061	-0.0384	-0.8287	-1.0960	0.0582
	0.2836	1.0566	-42.2909	
43.6800	-42.8800	-0.6202	0.2675	
0.2009	0.1518	-0.8443	-1.1340	0.0646
	0.2881	1.0579	-42.2915	
43.7000	-41.5500	0.5365	0.2658	
0.2282	0.3275	-0.8540	-1.1704	0.0712
	0.2924	1.0592	-42.2920	
43.7200	-42.2100	-0.0136	0.0617	
0.2040	0.4791	-0.8584	-1.2052	0.0780
	0.2966	1.0605	-42.2926	
43.7400	-42.8600	-0.4925	-0.1891	
0.1660	0.5985	-0.8578	-1.2383	0.0849
	0.3008	1.0618	-42.2932	
43.7600	-41.8400	0.4749	-0.2286	
0.1524	0.6801	-0.8529	-1.2695	0.0919
	0.3049	1.0631	-42.2937	
43.7800	-42.6700	-0.2932	-0.1697	
0.1801	0.7221	-0.8442	-1.2988	0.0991
	0.3090	1.0644	-42.2943	
43.8000	-41.5100	0.7356	-0.2325	
0.2439	0.7261	-0.8321	-1.3261	0.1065
	0.3129	1.0657	-42.2948	
43.8200	-42.9200	-1.0458	0.0444	
0.2887	0.6967	-0.8171	-1.3513	0.1140
	0.3168	1.0670	-42.2954	
43.8400	-40.4100	1.0714	0.5435	
0.2446	0.6412	-0.7995	-1.3744	0.1217
	0.3206	1.0683	-42.2959	
43.8600	-42.6600	-0.9223	0.3776	
0.0781	0.5673	-0.7800	-1.3953	0.1295
	0.3243	1.0696	-42.2965	
43.8800	-42.0700	0.4121	-0.0652	-
0.1626	0.4820	-0.7590	-1.4140	0.1375
	0.3279	1.0710	-42.2970	

43.9000 -42.9300 0.1257 -0.1203 -
0.3856 0.3908 -0.7368 -1.4305 0.1457
0.3315 1.0723 -42.2975
43.9200 -44.0200 -0.7738 -0.0687 -
0.4869 0.2990 -0.7137 -1.4448 0.1540
0.3349 1.0737 -42.2981
43.9400 -42.2500 1.2065 -0.3717 -
0.4029 0.2101 -0.6896 -1.4571 0.1624
0.3383 1.0750 -42.2986
43.9600 -44.8400 -1.3679 -0.4813 -
0.1623 0.1245 -0.6645 -1.4674 0.1711
0.3416 1.0764 -42.2991
43.9800 -41.3000 1.2974 0.1299
0.1371 0.0407 -0.6378 -1.4757 0.1798
0.3448 1.0778 -42.2997
44.0000 -41.8200 -0.0998 0.5832
0.3826 -0.0430 -0.6091 -1.4822 0.1888
0.3479 1.0792 -42.3002
44.0200 -42.6900 -0.7443 0.4427
0.4854 -0.1282 -0.5779 -1.4868 0.1978
0.3509 1.0806 -42.3007
44.0400 -41.9200 0.6299 -0.0877
0.4303 -0.2147 -0.5438 -1.4897 0.2070
0.3539 1.0820 -42.3012
44.0600 -43.6100 -0.5689 -0.3051
0.2655 -0.3004 -0.5063 -1.4909 0.2164
0.3567 1.0834 -42.3018
44.0800 -43.0700 0.0205 -0.1722
0.0676 -0.3823 -0.4652 -1.4905 0.2258
0.3595 1.0848 -42.3023
44.1000 -42.6700 0.5508 -0.0844 -
0.0917 -0.4580 -0.4205 -1.4886 0.2354
0.3622 1.0862 -42.3028
44.1200 -43.8100 -0.3736 -0.2553 -
0.1731 -0.5239 -0.3722 -1.4852 0.2451
0.3647 1.0877 -42.3033
44.1400 -43.6700 -0.3152 -0.3388 -
0.1903 -0.5752 -0.3204 -1.4805 0.2549
0.3672 1.0891 -42.3038
44.1600 -43.5200 -0.4320 0.0175 -
0.1879 -0.6058 -0.2655 -1.4745 0.2649
0.3696 1.0906 -42.3043
44.1800 -41.8400 0.6283 0.5128 -
0.2065 -0.6107 -0.2079 -1.4673 0.2749
0.3720 1.0920 -42.3048
44.2000 -41.6700 0.7443 0.5823 -
0.2737 -0.5864 -0.1478 -1.4589 0.2851
0.3742 1.0935 -42.3053
44.2200 -43.3700 -0.3728 0.0748 -
0.3872 -0.5321 -0.0858 -1.4496 0.2953
0.3763 1.0950 -42.3058

44.2400 -43.1300 0.2051 -0.5174 -
0.4984 -0.4500 -0.0227 -1.4392 0.3056
0.3783 1.0965 -42.3063
44.2600 -44.0700 -0.8611 -0.4601 -
0.5507 -0.3439 0.0409 -1.4280 0.3161
0.3803 1.0980 -42.3068
44.2800 -41.5100 0.8210 0.0805 -
0.5102 -0.2190 0.1040 -1.4160 0.3266
0.3821 1.0995 -42.3073
44.3000 -41.9900 -0.0633 0.2916 -
0.3715 -0.0822 0.1658 -1.4032 0.3372
0.3839 1.1010 -42.3078
44.3200 -41.7500 0.0151 0.0226 -
0.1524 0.0591 0.2255 -1.3898 0.3478
0.3856 1.1025 -42.3083
44.3400 -41.7500 -0.4161 -0.1980
0.1034 0.1969 0.2822 -1.3759 0.3586
0.3871 1.1041 -42.3087
44.3600 -41.1300 -0.2552 -0.0391
0.3473 0.3238 0.3350 -1.3614 0.3694
0.3886 1.1056 -42.3092
44.3800 -39.8700 0.3220 0.2432
0.5445 0.4322 0.3831 -1.3465 0.3803
0.3900 1.1072 -42.3097
44.4000 -39.4600 0.4916 0.1835
0.6716 0.5151 0.4257 -1.3313 0.3913
0.3913 1.1087 -42.3102
44.4200 -40.8400 -0.7063 -0.1937
0.7189 0.5670 0.4619 -1.3158 0.4023
0.3925 1.1103 -42.3106
44.4400 -39.8100 0.4037 -0.2937
0.6860 0.5849 0.4911 -1.3002 0.4134
0.3936 1.1119 -42.3111
44.4600 -40.2600 -0.2868 0.0230
0.5764 0.5699 0.5128 -1.2844 0.4245
0.3946 1.1135 -42.3116
44.4800 -39.3100 0.5295 0.2834
0.3979 0.5260 0.5272 -1.2687 0.4357
0.3955 1.1151 -42.3120
44.5000 -40.5700 -0.3520 0.2985
0.1629 0.4593 0.5345 -1.2529 0.4469
0.3963 1.1167 -42.3125
44.5200 -41.0400 -0.3743 0.1797 -
0.1056 0.3760 0.5351 -1.2372 0.4582
0.3970 1.1183 -42.3130
44.5400 -40.4600 0.6256 0.0083 -
0.3719 0.2826 0.5295 -1.2216 0.4695
0.3976 1.1200 -42.3134
44.5600 -41.5100 0.0705 -0.2007 -
0.5962 0.1848 0.5182 -1.2061 0.4808
0.3981 1.1216 -42.3139

44.5800 -42.5700 -0.6713 -0.3040 -
0.7354 0.0885 0.5017 -1.1907 0.4922
0.3986 1.1233 -42.3143
44.6000 -41.5900 0.3660 -0.1700 -
0.7571 -0.0011 0.4805 -1.1754 0.5036
0.3989 1.1249 -42.3148
44.6200 -41.7800 0.0749 -0.0523 -
0.6546 -0.0806 0.4552 -1.1602 0.5150
0.3991 1.1266 -42.3152
44.6400 -41.3900 0.2846 -0.1666 -
0.4444 -0.1481 0.4264 -1.1451 0.5265
0.3992 1.1283 -42.3157
44.6600 -42.1700 -0.6552 -0.1591 -
0.1697 -0.2035 0.3948 -1.1300 0.5379
0.3993 1.1300 -42.3161
44.6800 -40.6000 0.3470 0.1253
0.1153 -0.2474 0.3609 -1.1149 0.5493
0.3992 1.1317 -42.3165
44.7000 -40.2300 0.3676 0.3416
0.3561 -0.2803 0.3254 -1.0998 0.5607
0.3990 1.1334 -42.3170
44.7200 -40.6600 0.0113 0.2874
0.5080 -0.3025 0.2889 -1.0847 0.5721
0.3988 1.1352 -42.3174
44.7400 -41.3900 -0.7468 0.1039
0.5467 -0.3145 0.2520 -1.0696 0.5834
0.3984 1.1369 -42.3178
44.7600 -40.1700 0.8951 -0.0652
0.4762 -0.3177 0.2153 -1.0544 0.5947
0.3979 1.1387 -42.3183
44.7800 -41.6900 -0.6680 -0.1387
0.3283 -0.3133 0.1794 -1.0391 0.6060
0.3974 1.1404 -42.3187
44.8000 -41.5300 -0.3546 0.0118
0.1364 -0.3019 0.1445 -1.0237 0.6172
0.3967 1.1422 -42.3191
44.8200 -40.8300 0.3132 0.2600 -
0.0651 -0.2833 0.1112 -1.0081 0.6283
0.3959 1.1440 -42.3195
44.8400 -40.5900 0.7664 0.1754 -
0.2382 -0.2576 0.0796 -0.9923 0.6393
0.3951 1.1458 -42.3200
44.8600 -42.5300 -0.7616 -0.1900 -
0.3499 -0.2248 0.0500 -0.9763 0.6502
0.3941 1.1476 -42.3204
44.8800 -42.0200 -0.0596 -0.3508 -
0.3742 -0.1854 0.0226 -0.9601 0.6610
0.3930 1.1494 -42.3208
44.9000 -41.2500 0.4639 -0.2402 -
0.3057 -0.1407 -0.0025 -0.9435 0.6718
0.3918 1.1512 -42.3212

44.9200 -41.1600 0.1422 -0.0833 -
0.1637 -0.0918 -0.0254 -0.9266 0.6823
0.3906 1.1530 -42.3216
44.9400 -41.5000 -0.5587 0.0361
0.0117 -0.0405 -0.0459 -0.9094 0.6928
0.3892 1.1549 -42.3220
44.9600 -40.0100 0.5707 0.1813
0.1681 0.0114 -0.0643 -0.8917 0.7031
0.3877 1.1567 -42.3224
44.9800 -41.0300 -0.5518 0.2971
0.2557 0.0616 -0.0805 -0.8736 0.7132
0.3861 1.1586 -42.3228
45.0000 -39.7300 0.7146 0.1580
0.2534 0.1076 -0.0947 -0.8550 0.7232
0.3845 1.1605 -42.3232
45.0200 -41.4700 -0.6018 -0.2017
0.1760 0.1468 -0.1068 -0.8359 0.7330
0.3827 1.1623 -42.3236
45.0400 -40.7900 0.2178 -0.3061
0.0592 0.1773 -0.1171 -0.8163 0.7426
0.3808 1.1642 -42.3240
45.0600 -40.9400 -0.0400 -0.0836 -
0.0609 0.1980 -0.1255 -0.7960 0.7520
0.3788 1.1661 -42.3244
45.0800 -40.8800 -0.1630 0.1894 -
0.1509 0.2083 -0.1322 -0.7751 0.7612
0.3767 1.1680 -42.3248
45.1000 -40.7400 -0.2176 0.2999 -
0.1899 0.2081 -0.1373 -0.7536 0.7702
0.3745 1.1700 -42.3251
45.1200 -39.9500 0.7500 0.0841 -
0.1712 0.1975 -0.1409 -0.7314 0.7790
0.3722 1.1719 -42.3255
45.1400 -41.6800 -0.7133 -0.3025 -
0.1018 0.1775 -0.1429 -0.7085 0.7875
0.3698 1.1739 -42.3259
45.1600 -41.0600 0.0411 -0.3395
0.0024 0.1493 -0.1435 -0.6848 0.7958
0.3673 1.1758 -42.3263
45.1800 -40.3300 0.1836 0.0062
0.1122 0.1144 -0.1425 -0.6603 0.8038
0.3647 1.1778 -42.3266
45.2000 -39.9200 0.3580 0.3006
0.1937 0.0745 -0.1399 -0.6350 0.8116
0.3620 1.1797 -42.3270
45.2200 -40.3700 -0.0897 0.2008
0.2174 0.0313 -0.1357 -0.6088 0.8191
0.3593 1.1817 -42.3274
45.2400 -40.1800 0.4686 -0.1509
0.1756 -0.0124 -0.1298 -0.5818 0.8263
0.3564 1.1837 -42.3278

45.2600 -41.7100 -0.9280 -0.1862
0.0811 -0.0540 -0.1223 -0.5538 0.8332
0.3534 1.1857 -42.3281
45.2800 -39.8100 0.9883 0.1142 -
0.0390 -0.0907 -0.1130 -0.5249 0.8399
0.3503 1.1878 -42.3285
45.3000 -41.2300 -0.5565 0.1932 -
0.1550 -0.1202 -0.1020 -0.4950 0.8462
0.3471 1.1898 -42.3288
45.3200 -41.3800 -0.3980 0.0480 -
0.2365 -0.1398 -0.0893 -0.4642 0.8523
0.3438 1.1918 -42.3292
45.3400 -40.4800 0.5277 -0.1287 -
0.2580 -0.1477 -0.0750 -0.4324 0.8580
0.3404 1.1939 -42.3295
45.3600 -41.1700 -0.3837 -0.2110 -
0.2057 -0.1435 -0.0592 -0.3995 0.8634
0.3369 1.1959 -42.3299
45.3800 -40.5300 0.3221 -0.2213 -
0.0909 -0.1282 -0.0421 -0.3657 0.8685
0.3334 1.1980 -42.3302
45.4000 -40.4900 -0.0526 -0.1381
0.0538 -0.1037 -0.0241 -0.3308 0.8732
0.3297 1.2000 -42.3306
45.4200 -40.3900 -0.3959 0.0912
0.1829 -0.0724 -0.0055 -0.2948 0.8776
0.3259 1.2021 -42.3309
45.4400 -39.1400 0.4530 0.3516
0.2551 -0.0370 0.0134 -0.2577 0.8817
0.3221 1.2042 -42.3313
45.4600 -39.6400 -0.0867 0.3653
0.2466 0.0002 0.0318 -0.2195 0.8854
0.3181 1.2063 -42.3316
45.4800 -39.4400 0.1817 0.1380
0.1613 0.0367 0.0495 -0.1801 0.8888
0.3141 1.2084 -42.3319
45.5000 -40.5600 -0.4003 -0.1152
0.0331 0.0704 0.0658 -0.1396 0.8918
0.3100 1.2106 -42.3323
45.5200 -40.0000 0.2532 -0.2755 -
0.0916 0.0997 0.0804 -0.0980 0.8944
0.3058 1.2127 -42.3326
45.5400 -40.0500 0.1226 -0.3168 -
0.1669 0.1228 0.0928 -0.0551 0.8967
0.3014 1.2148 -42.3329
45.5600 -40.5500 -0.5277 -0.1868 -
0.1717 0.1388 0.1025 -0.0110 0.8986
0.2970 1.2170 -42.3332
45.5800 -39.2000 0.4840 0.0850 -
0.1172 0.1471 0.1092 0.0342 0.9001
0.2926 1.2192 -42.3336

45.6000 -39.2400 0.1208 0.2066 -
0.0335 0.1478 0.1126 0.0806 0.9013
0.2880 1.2213 -42.3339
45.6200 -39.1700 0.1343 0.0577
0.0440 0.1409 0.1124 0.1282 0.9020
0.2833 1.2235 -42.3342
45.6400 -40.1600 -0.6735 -0.0575
0.0932 0.1271 0.1084 0.1769 0.9024
0.2786 1.2257 -42.3345
45.6600 -39.2100 0.3711 0.0248
0.1074 0.1073 0.1006 0.2267 0.9024
0.2737 1.2279 -42.3348
45.6800 -38.9400 0.3723 0.0815
0.0926 0.0823 0.0890 0.2775 0.9020
0.2688 1.2301 -42.3351
45.7000 -39.7600 -0.4092 0.0054
0.0597 0.0535 0.0736 0.3293 0.9012
0.2638 1.2323 -42.3354
45.7200 -39.7100 -0.1147 -0.0688
0.0231 0.0226 0.0547 0.3820 0.9000
0.2587 1.2345 -42.3357
45.7400 -39.2600 0.2142 -0.0136 -
0.0077 -0.0086 0.0326 0.4356 0.8983
0.2536 1.2368 -42.3360
45.7600 -39.9500 -0.4789 0.1062 -
0.0275 -0.0380 0.0077 0.4898 0.8963
0.2483 1.2390 -42.3363
45.7800 -38.8100 0.6101 0.1173 -
0.0305 -0.0636 -0.0197 0.5446 0.8938
0.2430 1.2413 -42.3366
45.8000 -39.4300 0.2071 -0.0771 -
0.0139 -0.0838 -0.0493 0.5999 0.8909
0.2376 1.2435 -42.3369
45.8200 -40.4500 -0.6644 -0.2393
0.0120 -0.0969 -0.0805 0.6556 0.8876
0.2321 1.2458 -42.3372
45.8400 -39.1700 0.3948 -0.1226
0.0291 -0.1020 -0.1130 0.7115 0.8838
0.2265 1.2481 -42.3375
45.8600 -39.7100 -0.2654 0.1493
0.0221 -0.0982 -0.1463 0.7675 0.8797
0.2209 1.2504 -42.3378
45.8800 -38.9000 0.2658 0.3347 -
0.0152 -0.0858 -0.1798 0.8235 0.8750
0.2152 1.2527 -42.3380
45.9000 -39.4700 -0.3160 0.3195 -
0.0750 -0.0651 -0.2129 0.8793 0.8700
0.2094 1.2550 -42.3383
45.9200 -39.0500 0.3565 0.0506 -
0.1329 -0.0376 -0.2450 0.9347 0.8644
0.2035 1.2573 -42.3386

45.9400 -39.5800 0.1765 -0.3328 -
0.1617 -0.0047 -0.2756 0.9897 0.8585
0.1976 1.2596 -42.3389
45.9600 -40.4000 -0.5916 -0.5154 -
0.1387 0.0316 -0.3041 1.0440 0.8520
0.1916 1.2619 -42.3391
45.9800 -39.0000 0.5260 -0.3272 -
0.0589 0.0689 -0.3299 1.0975 0.8451
0.1855 1.2643 -42.3394
46.0000 -39.4000 -0.3294 0.0402
0.0534 0.1044 -0.3523 1.1502 0.8378
0.1794 1.2666 -42.3397
46.0200 -38.7100 -0.1184 0.3465
0.1603 0.1352 -0.3708 1.2017 0.8299
0.1732 1.2690 -42.3399
46.0400 -38.0200 0.2926 0.4826
0.2192 0.1583 -0.3846 1.2520 0.8216
0.1669 1.2714 -42.3402
46.0600 -38.4100 0.0533 0.3463
0.2052 0.1717 -0.3932 1.3010 0.8128
0.1606 1.2737 -42.3405
46.0800 -38.8100 0.1173 -0.0045
0.1323 0.1747 -0.3963 1.3484 0.8035
0.1542 1.2761 -42.3407
46.1000 -39.3600 -0.2049 -0.3075
0.0329 0.1672 -0.3934 1.3942 0.7937
0.1478 1.2785 -42.3410
46.1200 -39.3700 -0.0324 -0.3410 -
0.0584 0.1498 -0.3844 1.4382 0.7834
0.1413 1.2809 -42.3412
46.1400 -38.9800 -0.0288 -0.1397 -
0.1139 0.1233 -0.3692 1.4802 0.7726
0.1347 1.2833 -42.3415
46.1600 -38.6300 0.3095 0.0396 -
0.1227 0.0889 -0.3478 1.5201 0.7614
0.1281 1.2858 -42.3417
46.1800 -39.0900 -0.3013 0.0823 -
0.0888 0.0473 -0.3202 1.5577 0.7495
0.1215 1.2882 -42.3419
46.2000 -38.8600 0.0171 0.0820 -
0.0345 -0.0008 -0.2863 1.5930 0.7372
0.1147 1.2906 -42.3422
46.2200 -38.6400 0.1826 0.1005
0.0116 -0.0547 -0.2462 1.6257 0.7244
0.1080 1.2931 -42.3424
46.2400 -38.7200 -0.0523 0.1094
0.0292 -0.1135 -0.2001 1.6557 0.7110
0.1012 1.2955 -42.3427
46.2600 -38.9300 -0.1663 0.0731
0.0114 -0.1762 -0.1484 1.6829 0.6971
0.0943 1.2980 -42.3429

46.2800 -38.8500 0.0737 -0.0062 -
0.0323 -0.2414 -0.0913 1.7072 0.6827
0.0874 1.3004 -42.3431
46.3000 -38.9100 0.1702 -0.1045 -
0.0835 -0.3078 -0.0294 1.7283 0.6677
0.0805 1.3029 -42.3433
46.3200 -39.5500 -0.4815 -0.0916 -
0.1251 -0.3733 0.0368 1.7462 0.6522
0.0735 1.3054 -42.3436
46.3400 -38.4700 0.4430 0.0994 -
0.1392 -0.4355 0.1064 1.7608 0.6362
0.0665 1.3079 -42.3438
46.3600 -39.2100 -0.3162 0.1586 -
0.1128 -0.4916 0.1785 1.7718 0.6196
0.0595 1.3104 -42.3440
46.3800 -38.5800 0.3910 -0.0876 -
0.0422 -0.5382 0.2523 1.7792 0.6025
0.0524 1.3129 -42.3442
46.4000 -39.3300 -0.1532 -0.3816
0.0638 -0.5721 0.3266 1.7828 0.5849
0.0453 1.3154 -42.3444
46.4200 -39.0900 -0.0995 -0.3997
0.1843 -0.5899 0.4006 1.7824 0.5668
0.0382 1.3179 -42.3446
46.4400 -38.6500 -0.0389 -0.0808
0.2904 -0.5886 0.4730 1.7781 0.5481
0.0310 1.3205 -42.3448
46.4600 -38.0000 0.0254 0.3237
0.3468 -0.5652 0.5430 1.7695 0.5290
0.0238 1.3230 -42.3450
46.4800 -37.4200 0.3536 0.5104
0.3230 -0.5180 0.6093 1.7567 0.5094
0.0166 1.3256 -42.3452
46.5000 -38.3000 -0.2836 0.3693
0.2082 -0.4461 0.6708 1.7393 0.4894
0.0094 1.3281 -42.3454
46.5200 -38.5600 -0.1739 0.0673
0.0227 -0.3507 0.7264 1.7175 0.4690
0.0021 1.3307 -42.3456
46.5400 -38.5500 0.2738 -0.2341 -
0.1919 -0.2343 0.7749 1.6910 0.4481
-0.0051 1.3332 -42.3458
46.5600 -39.0100 0.0603 -0.4495 -
0.3872 -0.1002 0.8150 1.6598 0.4269
-0.0124 1.3358 -42.3460
46.5800 -39.4100 -0.3978 -0.3947 -
0.5223 0.0477 0.8459 1.6239 0.4053
-0.0197 1.3384 -42.3462
46.6000 -38.6300 -0.0123 0.0148 -
0.5760 0.2052 0.8663 1.5833 0.3833
-0.0270 1.3410 -42.3464

46.6200 -37.7800 0.2356 0.4183 –
0.5507 0.3677 0.8753 1.5381 0.3611
-0.0344 1.3436 -42.3466
46.6400 -37.4500 0.4280 0.4419 –
0.4573 0.5302 0.8718 1.4884 0.3385
-0.0417 1.3462 -42.3468
46.6600 -38.3400 -0.4692 0.1463 –
0.3112 0.6851 0.8549 1.4343 0.3156
-0.0490 1.3488 -42.3469
46.6800 -38.2400 -0.2336 -0.1256 –
0.1266 0.8218 0.8238 1.3761 0.2924
-0.0564 1.3514 -42.3471
46.7000 -37.6300 0.3897 -0.2927
0.0940 0.9278 0.7778 1.3141 0.2690
-0.0637 1.3541 -42.3473
46.7200 -37.7200 0.3042 -0.4883
0.3504 0.9908 0.7167 1.2484 0.2454
-0.0710 1.3567 -42.3475
46.7400 -38.2500 -0.2221 -0.6039
0.6095 1.0001 0.6409 1.1793 0.2216
-0.0784 1.3594 -42.3476
46.7600 -38.4400 -0.6335 -0.3487
0.8106 0.9489 0.5516 1.1072 0.1975
-0.0857 1.3620 -42.3478
46.7800 -37.2100 0.0944 0.3469
0.8856 0.8344 0.4506 1.0322 0.1733
-0.0930 1.3647 -42.3480
46.8000 -37.2800 -0.1694 1.0380
0.7766 0.6604 0.3401 0.9548 0.1490
-0.1004 1.3673 -42.3481
46.8200 -37.0700 1.0558 0.9476
0.4616 0.4399 0.2224 0.8750 0.1245
-0.1077 1.3700 -42.3483
46.8400 -41.0400 -1.2014 -0.0115
0.0235 0.1911 0.0998 0.7932 0.0999
-0.1150 1.3727 -42.3484
46.8600 -40.5400 1.2134 -0.8898 –
0.3975 -0.0671 -0.0254 0.7097 0.0752
-0.1223 1.3754 -42.3486
46.8800 -43.6100 -1.1848 -0.7830 –
0.6703 -0.3155 -0.1509 0.6247 0.0504
-0.1296 1.3781 -42.3487
46.9000 -41.0000 1.0334 0.0607 –
0.7480 -0.5356 -0.2743 0.5385 0.0256
-0.1368 1.3808 -42.3489
46.9200 -42.6400 -0.7312 0.5496 –
0.6620 -0.7125 -0.3934 0.4514 0.0008
-0.1441 1.3835 -42.3490
46.9400 -41.6000 0.6161 0.3806 –
0.4910 -0.8350 -0.5059 0.3637 -0.0241
-0.1513 1.3862 -42.3492

46.9600 -43.0100 -0.4464 0.1383 -
0.3271 -0.8970 -0.6096 0.2755 -0.0489
-0.1585 1.3889 -42.3493
46.9800 -42.5400 0.1932 0.0645 -
0.2065 -0.8993 -0.7026 0.1872 -0.0738
-0.1657 1.3916 -42.3494
47.0000 -42.8200 0.0716 -0.0012 -
0.1080 -0.8485 -0.7832 0.0991 -0.0986
-0.1728 1.3944 -42.3496
47.0200 -43.3500 -0.2403 -0.1734
0.0049 -0.7553 -0.8505 0.0113 -0.1233
-0.1800 1.3971 -42.3497
47.0400 -42.8800 0.2488 -0.2996
0.1466 -0.6312 -0.9036 -0.0757 -0.1479
-0.1870 1.3999 -42.3498
47.0600 -43.0900 -0.3660 -0.1475
0.2980 -0.4874 -0.9424 -0.1618 -0.1725
-0.1941 1.4026 -42.3499
47.0800 -42.5300 -0.3103 0.2071
0.4106 -0.3323 -0.9667 -0.2467 -0.1969
-0.2011 1.4054 -42.3501
47.1000 -41.2500 0.7145 0.4290
0.4357 -0.1734 -0.9765 -0.3302 -0.2211
-0.2081 1.4082 -42.3502
47.1200 -41.9000 0.2972 0.2052
0.3479 -0.0184 -0.9722 -0.4119 -0.2452
-0.2151 1.4109 -42.3503
47.1400 -42.8400 0.0146 -0.2944
0.1753 0.1256 -0.9542 -0.4918 -0.2692
-0.2220 1.4137 -42.3504
47.1600 -44.2600 -1.1084 -0.4545 -
0.0312 0.2527 -0.9231 -0.5695 -0.2929
-0.2289 1.4165 -42.3505
47.1800 -42.8500 0.0993 -0.0596 -
0.2168 0.3574 -0.8797 -0.6450 -0.3164
-0.2357 1.4193 -42.3506
47.2000 -41.6600 0.9761 0.3621 -
0.3361 0.4353 -0.8248 -0.7182 -0.3396
-0.2425 1.4221 -42.3507
47.2200 -42.6000 0.1106 0.2777 -
0.3539 0.4832 -0.7592 -0.7888 -0.3626
-0.2493 1.4249 -42.3509
47.2400 -43.7200 -0.6736 -0.1656 -
0.2598 0.5004 -0.6841 -0.8569 -0.3854
-0.2559 1.4277 -42.3510
47.2600 -42.8700 0.2960 -0.4896 -
0.0742 0.4886 -0.6005 -0.9222 -0.4078
-0.2626 1.4305 -42.3511
47.2800 -43.2300 -0.4689 -0.3278
0.1638 0.4513 -0.5099 -0.9847 -0.4299
-0.2692 1.4334 -42.3511

47.3000 -41.8000 0.3785 0.0903
0.3993 0.3932 -0.4136 -1.0443 -0.4516
-0.2757 1.4362 -42.3512
47.3200 -41.9100 -0.0921 0.3516
0.5648 0.3195 -0.3132 -1.1009 -0.4730
-0.2822 1.4390 -42.3513
47.3400 -41.7300 0.0066 0.4169
0.5999 0.2361 -0.2104 -1.1544 -0.4940
-0.2886 1.4419 -42.3514
47.3600 -42.2600 -0.1083 0.3113
0.4806 0.1494 -0.1067 -1.2047 -0.5146
-0.2950 1.4447 -42.3515
47.3800 -42.5400 0.1849 -0.0273
0.2315 0.0654 -0.0038 -1.2518 -0.5348
-0.3013 1.4476 -42.3516
47.4000 -43.0600 0.4349 -0.3518 -
0.0975 -0.0108 0.0970 -1.2956 -0.5546
-0.3075 1.4505 -42.3517
47.4200 -44.6700 -1.0283 -0.1499 -
0.4398 -0.0743 0.1944 -1.3360 -0.5738
-0.3137 1.4533 -42.3517
47.4400 -42.1400 1.0819 0.3479 -
0.7100 -0.1207 0.2869 -1.3730 -0.5927
-0.3198 1.4562 -42.3518
47.4600 -44.6100 -0.8721 0.1655 -
0.8264 -0.1462 0.3736 -1.4065 -0.6110
-0.3258 1.4591 -42.3519
47.4800 -43.8900 0.1722 -0.3998 -
0.7513 -0.1499 0.4534 -1.4364 -0.6287
-0.3318 1.4620 -42.3520
47.5000 -43.6700 0.2133 -0.4609 -
0.4930 -0.1349 0.5259 -1.4627 -0.6460
-0.3377 1.4649 -42.3520
47.5200 -43.7300 -0.7191 0.0663 -
0.1123 -0.1069 0.5908 -1.4854 -0.6627
-0.3435 1.4678 -42.3521
47.5400 -41.1600 0.9393 0.4826
0.2908 -0.0721 0.6481 -1.5044 -0.6788
-0.3493 1.4707 -42.3522
47.5600 -42.8100 -0.8675 0.2261
0.6151 -0.0366 0.6977 -1.5197 -0.6943
-0.3549 1.4736 -42.3522
47.5800 -41.3700 0.6819 -0.0819
0.7982 -0.0064 0.7396 -1.5312 -0.7092
-0.3605 1.4765 -42.3523
47.6000 -42.4200 -0.6080 0.0051
0.8219 0.0134 0.7736 -1.5390 -0.7234
-0.3660 1.4794 -42.3523
47.6200 -41.3200 0.5522 0.1281
0.7010 0.0211 0.8000 -1.5430 -0.7370
-0.3714 1.4824 -42.3524

47.6400 -42.1000 -0.0075 0.0468
0.4709 0.0180 0.8184 -1.5433 -0.7500
-0.3767 1.4853 -42.3524
47.6600 -42.8600 -0.1881 -0.0800
0.1724 0.0075 0.8287 -1.5398 -0.7622
-0.3820 1.4882 -42.3525
47.6800 -43.3800 -0.4687 -0.0381 -
0.1515 -0.0067 0.8307 -1.5327 -0.7738
-0.3871 1.4912 -42.3525
47.7000 -42.4500 0.6624 0.0435 -
0.4381 -0.0208 0.8243 -1.5218 -0.7847
-0.3922 1.4941 -42.3525
47.7200 -43.4800 0.0124 -0.0786 -
0.6228 -0.0307 0.8095 -1.5074 -0.7949
-0.3971 1.4971 -42.3526
47.7400 -44.6000 -0.7031 -0.3305 -
0.6698 -0.0327 0.7861 -1.4895 -0.8045
-0.4020 1.5001 -42.3526
47.7600 -43.9700 0.0212 -0.5636 -
0.5711 -0.0242 0.7545 -1.4682 -0.8133
-0.4068 1.5030 -42.3527
47.7800 -42.9800 0.8715 -0.6438 -
0.3483 -0.0047 0.7148 -1.4435 -0.8215
-0.4114 1.5060 -42.3527
47.8000 -43.7800 -0.6040 -0.3450 -
0.0627 0.0251 0.6674 -1.4157 -0.8290
-0.4160 1.5090 -42.3527
47.8200 -43.0700 -0.7537 0.3164
0.2017 0.0638 0.6128 -1.3849 -0.8359
-0.4205 1.5120 -42.3527
47.8400 -40.5100 1.0355 0.8577
0.3586 0.1099 0.5516 -1.3512 -0.8421
-0.4248 1.5149 -42.3528
47.8600 -42.1900 -0.4221 0.6203
0.3500 0.1607 0.4844 -1.3147 -0.8477
-0.4291 1.5179 -42.3528
47.8800 -42.6400 0.0155 -0.1512
0.2146 0.2112 0.4120 -1.2758 -0.8527
-0.4332 1.5209 -42.3528
47.9000 -43.4100 -0.0236 -0.6240
0.0341 0.2544 0.3351 -1.2344 -0.8571
-0.4373 1.5239 -42.3528
47.9200 -43.9400 -0.7232 -0.3479 -
0.1127 0.2832 0.2544 -1.1907 -0.8609
-0.4412 1.5269 -42.3528
47.9400 -41.8500 0.9270 0.2063 -
0.1675 0.2915 0.1710 -1.1450 -0.8641
-0.4450 1.5300 -42.3528
47.9600 -43.6400 -0.9050 0.2288 -
0.1065 0.2756 0.0856 -1.0974 -0.8667
-0.4487 1.5330 -42.3528

47.9800 -42.3000 0.9394 -0.1688
0.0375 0.2355 -0.0007 -1.0481 -0.8688
-0.4523 1.5360 -42.3528
48.0000 -44.0900 -1.0557 -0.2210
0.1875 0.1739 -0.0869 -0.9972 -0.8702
-0.4557 1.5390 -42.3528
48.0200 -41.8000 0.9563 0.1126
0.2762 0.0953 -0.1717 -0.9449 -0.8712
-0.4591 1.5421 -42.3528
48.0400 -42.5100 0.0118 0.3083
0.2742 0.0049 -0.2541 -0.8913 -0.8716
-0.4623 1.5451 -42.3528
48.0600 -43.6700 -0.7328 0.2850
0.1840 -0.0914 -0.3327 -0.8367 -0.8715
-0.4655 1.5481 -42.3528
48.0800 -43.3900 -0.1369 0.1535
0.0391 -0.1871 -0.4066 -0.7811 -0.8709
-0.4685 1.5512 -42.3528
48.1000 -43.2700 0.6440 -0.0924 -
0.1134 -0.2754 -0.4745 -0.7249 -0.8697
-0.4714 1.5542 -42.3528
48.1200 -44.5200 -0.1019 -0.4157 -
0.2276 -0.3499 -0.5352 -0.6680 -0.8681
-0.4741 1.5573 -42.3528
48.1400 -45.0800 -0.5086 -0.5008 -
0.2715 -0.4049 -0.5879 -0.6108 -0.8660
-0.4768 1.5604 -42.3528
48.1600 -43.7800 0.3804 -0.1216 -
0.2381 -0.4371 -0.6314 -0.5532 -0.8634
-0.4793 1.5634 -42.3528
48.1800 -43.7400 -0.3679 0.4062 -
0.1566 -0.4450 -0.6652 -0.4957 -0.8603
-0.4818 1.5665 -42.3527
48.2000 -42.9400 0.2745 0.5843 -
0.0638 -0.4293 -0.6888 -0.4381 -0.8568
-0.4841 1.5696 -42.3527
48.2200 -42.8700 0.4582 0.2812
0.0170 -0.3921 -0.7023 -0.3808 -0.8528
-0.4863 1.5727 -42.3527
48.2400 -44.3300 -0.6988 -0.1952
0.0806 -0.3369 -0.7060 -0.3239 -0.8484
-0.4884 1.5757 -42.3526
48.2600 -43.6000 0.1164 -0.4212
0.1269 -0.2674 -0.7005 -0.2674 -0.8435
-0.4904 1.5788 -42.3526
48.2800 -42.7900 0.5809 -0.3491
0.1550 -0.1874 -0.6862 -0.2115 -0.8382
-0.4922 1.5819 -42.3526
48.3000 -43.3400 -0.3493 -0.0983
0.1625 -0.1006 -0.6640 -0.1562 -0.8325
-0.4940 1.5850 -42.3525

48.3200 -42.8600 -0.4037 0.2252
0.1421 -0.0108 -0.6345 -0.1015 -0.8264
-0.4956 1.5881 -42.3525
48.3400 -41.4700 0.6438 0.4026
0.0894 0.0782 -0.5984 -0.0477 -0.8199
-0.4972 1.5912 -42.3525
48.3600 -42.3500 -0.0782 0.2285
0.0055 0.1628 -0.5565 0.0052 -0.8130
-0.4986 1.5943 -42.3524
48.3800 -42.5400 -0.1278 -0.0982 -
0.0923 0.2400 -0.5094 0.0573 -0.8058
-0.4999 1.5974 -42.3524
48.4000 -42.9800 -0.6847 -0.1613 -
0.1729 0.3074 -0.4579 0.1084 -0.7981
-0.5011 1.6006 -42.3523
48.4200 -41.4000 0.8678 -0.0613 -
0.1974 0.3627 -0.4027 0.1584 -0.7901
-0.5021 1.6037 -42.3523
48.4400 -43.0000 -0.8172 -0.1450 -
0.1493 0.4037 -0.3446 0.2074 -0.7818
-0.5031 1.6068 -42.3522
48.4600 -41.3200 0.4880 -0.2483 -
0.0415 0.4281 -0.2841 0.2551 -0.7731
-0.5040 1.6099 -42.3521
48.4800 -41.6700 -0.1314 -0.1162
0.0912 0.4343 -0.2221 0.3016 -0.7640
-0.5047 1.6130 -42.3521
48.5000 -41.1700 -0.1688 0.1747
0.2027 0.4205 -0.1591 0.3468 -0.7547
-0.5054 1.6162 -42.3520
48.5200 -40.5000 0.2228 0.4019
0.2559 0.3857 -0.0958 0.3907 -0.7450
-0.5059 1.6193 -42.3520
48.5400 -40.5500 0.0335 0.3290
0.2329 0.3302 -0.0328 0.4331 -0.7350
-0.5063 1.6224 -42.3519
48.5600 -40.9100 0.0801 0.0082
0.1467 0.2570 0.0294 0.4740 -0.7247
-0.5067 1.6256 -42.3518
48.5800 -41.6800 -0.2735 -0.2830
0.0342 0.1706 0.0902 0.5134 -0.7142
-0.5069 1.6287 -42.3517
48.6000 -41.5300 0.0973 -0.3151 -
0.0662 0.0766 0.1491 0.5511 -0.7033
-0.5070 1.6319 -42.3517
48.6200 -41.4300 -0.0578 -0.0916 -
0.1290 -0.0190 0.2055 0.5871 -0.6922
-0.5069 1.6350 -42.3516
48.6400 -41.1100 -0.0159 0.1668 -
0.1491 -0.1101 0.2590 0.6214 -0.6808
-0.5068 1.6382 -42.3515

48.6600 -40.4200 0.6500 0.1390 -
0.1396 -0.1908 0.3091 0.6538 -0.6692
-0.5066 1.6413 -42.3514
48.6800 -42.0200 -0.7778 -0.0824 -
0.1238 -0.2561 0.3554 0.6842 -0.6573
-0.5063 1.6445 -42.3513
48.7000 -41.2200 -0.0098 -0.0825 -
0.1234 -0.3018 0.3975 0.7127 -0.6452
-0.5058 1.6476 -42.3512
48.7200 -40.4000 0.4999 0.1077 -
0.1474 -0.3254 0.4352 0.7392 -0.6329
-0.5053 1.6508 -42.3511
48.7400 -40.5700 0.4163 0.1799 -
0.1885 -0.3265 0.4683 0.7635 -0.6203
-0.5046 1.6539 -42.3510
48.7600 -41.6200 -0.6722 0.0632 -
0.2261 -0.3073 0.4968 0.7855 -0.6076
-0.5039 1.6571 -42.3510
48.7800 -40.6800 0.5701 -0.1426 -
0.2304 -0.2715 0.5205 0.8054 -0.5946
-0.5030 1.6602 -42.3509
48.8000 -41.4400 -0.4640 -0.2211 -
0.1758 -0.2230 0.5395 0.8229 -0.5815
-0.5021 1.6634 -42.3507
48.8200 -40.9900 -0.3580 -0.0748 -
0.0572 -0.1662 0.5537 0.8380 -0.5681
-0.5010 1.6666 -42.3506
48.8400 -39.5400 0.5826 0.1468
0.1077 -0.1050 0.5632 0.8508 -0.5546
-0.4998 1.6697 -42.3505
48.8600 -39.9200 -0.0670 0.1391
0.2878 -0.0426 0.5680 0.8611 -0.5410
-0.4985 1.6729 -42.3504
48.8800 -40.0500 -0.1416 -0.0593
0.4523 0.0172 0.5682 0.8691 -0.5272
-0.4971 1.6760 -42.3503
48.9000 -39.9200 -0.0965 -0.1625
0.5763 0.0712 0.5638 0.8746 -0.5132
-0.4956 1.6792 -42.3502
48.9200 -39.6000 -0.0366 -0.0717
0.6389 0.1168 0.5547 0.8778 -0.4991
-0.4941 1.6823 -42.3501
48.9400 -39.0600 0.3812 0.0565
0.6229 0.1534 0.5409 0.8786 -0.4849
-0.4924 1.6855 -42.3500
48.9600 -39.7400 -0.2495 0.1606
0.5148 0.1812 0.5226 0.8772 -0.4706
-0.4906 1.6887 -42.3498
48.9800 -39.6600 -0.2332 0.3314
0.3034 0.2018 0.4997 0.8735 -0.4562
-0.4887 1.6918 -42.3497

49.0000	-39.1900	0.3714	0.5173	-
0.0011	0.2174	0.4724	0.8677	-0.4417
	-0.4867	1.6950	-42.3496	
49.0200	-40.2500	-0.2737	0.4637	-
0.3595	0.2300	0.4408	0.8598	-0.4271
	-0.4845	1.6981	-42.3494	
49.0400	-40.3100	0.4269	0.0472	-
0.7100	0.2411	0.4049	0.8501	-0.4125
	-0.4823	1.7013	-42.3493	
49.0600	-41.9100	-0.2936	-0.5348	-
0.9684	0.2522	0.3650	0.8384	-0.3979
	-0.4800	1.7044	-42.3492	
49.0800	-41.9700	-0.0002	-0.8982	-
1.0512	0.2649	0.3214	0.8251	-0.3832
	-0.4776	1.7075	-42.3490	
49.1000	-42.2100	-0.4055	-0.7323	-
0.9090	0.2798	0.2744	0.8101	-0.3685
	-0.4751	1.7107	-42.3489	
49.1200	-40.6500	0.0549	-0.1628	-
0.5701	0.2943	0.2246	0.7936	-0.3538
	-0.4725	1.7138	-42.3488	
49.1400	-39.6100	0.5329	0.2967	-
0.1097	0.3045	0.1725	0.7758	-0.3392
	-0.4698	1.7170	-42.3486	
49.1600	-39.9400	-0.5347	0.2994	
0.3844	0.3065	0.1188	0.7567	-0.3245
	-0.4670	1.7201	-42.3485	
49.1800	-39.1800	0.1788	0.1266	
0.8094	0.2964	0.0643	0.7364	-0.3100
	-0.4641	1.7232	-42.3483	
49.2000	-39.0100	0.0630	0.2238	
1.0620	0.2702	0.0094	0.7152	-0.2955
	-0.4611	1.7263	-42.3482	
49.2200	-39.3800	-0.5280	0.5096	
1.0945	0.2247	-0.0450	0.6930	-0.2810
	-0.4580	1.7295	-42.3480	
49.2400	-38.4100	0.8138	0.5373	
0.9147	0.1604	-0.0983	0.6700	-0.2667
	-0.4548	1.7326	-42.3478	
49.2600	-40.4300	-0.3412	0.0614	
0.5793	0.0812	-0.1499	0.6464	-0.2525
	-0.4515	1.7357	-42.3477	
49.2800	-41.1900	-0.1649	-0.3929	
0.1757	-0.0077	-0.1990	0.6223	-0.2385
	-0.4481	1.7388	-42.3475	
49.3000	-41.9200	-0.4845	-0.3027	-
0.2116	-0.1009	-0.2450	0.5977	-0.2246
	-0.4446	1.7419	-42.3474	
49.3200	-40.9400	0.7713	0.0354	-
0.5210	-0.1930	-0.2872	0.5728	-0.2108
	-0.4410	1.7450	-42.3472	

49.3400 -42.3800 -0.4958 0.1376 -
0.7131 -0.2787 -0.3250 0.5477 -0.1973
-0.4373 1.7481 -42.3470
49.3600 -41.9900 0.1119 -0.0037 -
0.7808 -0.3527 -0.3578 0.5226 -0.1840
-0.4336 1.7512 -42.3468
49.3800 -42.4500 -0.0676 -0.1735 -
0.7397 -0.4104 -0.3850 0.4975 -0.1708
-0.4297 1.7543 -42.3467
49.4000 -42.6700 -0.2030 -0.2197 -
0.6144 -0.4483 -0.4059 0.4726 -0.1579
-0.4257 1.7574 -42.3465
49.4200 -41.7500 0.3050 -0.1457 -
0.4385 -0.4641 -0.4201 0.4480 -0.1453
-0.4217 1.7604 -42.3463
49.4400 -42.0300 -0.1431 -0.0269 -
0.2495 -0.4573 -0.4273 0.4238 -0.1329
-0.4175 1.7635 -42.3461
49.4600 -41.5200 0.0163 0.1071 -
0.0841 -0.4283 -0.4275 0.4000 -0.1209
-0.4133 1.7666 -42.3459
49.4800 -41.1300 0.1368 0.2025
0.0298 -0.3783 -0.4212 0.3768 -0.1091
-0.4089 1.7696 -42.3457
49.5000 -41.5100 -0.2635 0.2429
0.0791 -0.3101 -0.4089 0.3543 -0.0976
-0.4045 1.7727 -42.3455
49.5200 -41.2400 -0.1454 0.1949
0.0689 -0.2278 -0.3912 0.3323 -0.0865
-0.4000 1.7757 -42.3454
49.5400 -40.7000 0.5458 -0.0226
0.0308 -0.1368 -0.3687 0.3111 -0.0758
-0.3953 1.7787 -42.3452
49.5600 -41.7800 -0.4018 -0.3241
0.0056 -0.0434 -0.3421 0.2906 -0.0653
-0.3906 1.7818 -42.3450
49.5800 -41.1000 0.1221 -0.3499
0.0236 0.0466 -0.3120 0.2708 -0.0553
-0.3858 1.7848 -42.3448
49.6000 -40.9400 -0.1458 -0.0339
0.0887 0.1284 -0.2791 0.2518 -0.0457
-0.3810 1.7878 -42.3446
49.6200 -39.9300 0.4706 0.2472
0.1719 0.1982 -0.2440 0.2337 -0.0365
-0.3760 1.7908 -42.3443
49.6400 -40.7000 -0.4815 0.2291
0.2346 0.2531 -0.2074 0.2163 -0.0277
-0.3709 1.7938 -42.3441
49.6600 -39.9400 0.4237 0.0224
0.2496 0.2912 -0.1699 0.1998 -0.0193
-0.3658 1.7968 -42.3439

49.6800 -40.8000 -0.4201 -0.1014
0.2157 0.3115 -0.1321 0.1842 -0.0113
-0.3605 1.7998 -42.3437
49.7000 -40.3000 0.1273 -0.0694
0.1554 0.3139 -0.0949 0.1695 -0.0038
-0.3552 1.8027 -42.3435
49.7200 -40.0700 0.3930 -0.0352
0.0972 0.2998 -0.0587 0.1556 0.0032
-0.3498 1.8057 -42.3433
49.7400 -40.6000 -0.2317 -0.0791
0.0565 0.2717 -0.0241 0.1427 0.0098
-0.3443 1.8087 -42.3431
49.7600 -40.9000 -0.4267 -0.0964
0.0364 0.2329 0.0083 0.1307 0.0160
-0.3387 1.8116 -42.3428
49.7800 -40.0400 0.5249 -0.0272
0.0296 0.1871 0.0382 0.1196 0.0217
-0.3330 1.8145 -42.3426
49.8000 -40.8400 -0.3719 0.0528
0.0195 0.1383 0.0649 0.1095 0.0269
-0.3273 1.8175 -42.3424
49.8200 -40.5300 -0.0875 0.1363 -
0.0110 0.0905 0.0882 0.1003 0.0316
-0.3215 1.8204 -42.3421
49.8400 -40.3200 0.1360 0.2138 -
0.0675 0.0478 0.1077 0.0921 0.0359
-0.3155 1.8233 -42.3419
49.8600 -40.2700 0.3246 0.1372 -
0.1427 0.0134 0.1234 0.0848 0.0397
-0.3095 1.8262 -42.3417
49.8800 -41.3100 -0.4966 -0.0588 -
0.2133 -0.0104 0.1350 0.0784 0.0431
-0.3035 1.8291 -42.3414
49.9000 -40.9200 0.1425 -0.1558 -
0.2501 -0.0229 0.1427 0.0729 0.0461
-0.2973 1.8319 -42.3412
49.9200 -41.1400 -0.1101 -0.1510 -
0.2299 -0.0246 0.1466 0.0682 0.0486
-0.2911 1.8348 -42.3409
49.9400 -40.4100 0.5228 -0.1740 -
0.1446 -0.0172 0.1470 0.0644 0.0507
-0.2848 1.8377 -42.3407
49.9600 -41.1300 -0.3758 -0.1904 -
0.0136 -0.0036 0.1442 0.0613 0.0524
-0.2785 1.8405 -42.3405
49.9800 -40.8400 -0.3777 -0.0037
0.1226 0.0129 0.1387 0.0589 0.0537
-0.2721 1.8433 -42.3402
50.0000 -39.8100 0.1245 0.3071
0.2241 0.0287 0.1310 0.0572 0.0546
-0.2656 1.8461 -42.3399

50.0200 -39.0600 0.8765 0.3463
0.2602 0.0406 0.1215 0.0560 0.0552
-0.2590 1.8490 -42.3397
50.0400 -41.1700 -0.9686 0.0920
0.2206 0.0464 0.1109 0.0553 0.0554
-0.2524 1.8517 -42.3394
50.0600 -39.5900 0.7912 -0.0949
0.1265 0.0451 0.0995 0.0551 0.0552
-0.2457 1.8545 -42.3392
50.0800 -40.6800 0.0472 -0.2318
0.0156 0.0369 0.0878 0.0551 0.0548
-0.2390 1.8573 -42.3389
50.1000 -41.0900 -0.1736 -0.3308 -
0.0694 0.0226 0.0762 0.0555 0.0540
-0.2322 1.8601 -42.3386
50.1200 -41.4700 -0.5244 -0.2078 -
0.1014 0.0031 0.0652 0.0561 0.0529
-0.2254 1.8628 -42.3384
50.1400 -39.9600 0.6498 -0.0108 -
0.0781 -0.0204 0.0549 0.0568 0.0516
-0.2186 1.8655 -42.3381
50.1600 -40.5900 -0.1259 0.0290 -
0.0180 -0.0467 0.0458 0.0575 0.0500
-0.2116 1.8682 -42.3378
50.1800 -40.7700 -0.3151 0.0318
0.0483 -0.0740 0.0381 0.0582 0.0481
-0.2047 1.8710 -42.3376
50.2000 -40.3300 0.1059 0.1455
0.0886 -0.1000 0.0319 0.0588 0.0460
-0.1977 1.8736 -42.3373
50.2200 -40.2500 0.0613 0.1938
0.0864 -0.1228 0.0274 0.0592 0.0438
-0.1907 1.8763 -42.3370
50.2400 -40.1700 0.4196 0.0290
0.0402 -0.1405 0.0247 0.0594 0.0413
-0.1836 1.8790 -42.3367
50.2600 -41.2000 -0.3452 -0.1534 -
0.0319 -0.1514 0.0238 0.0593 0.0386
-0.1765 1.8816 -42.3364
50.2800 -41.4300 -0.5332 -0.1114 -
0.1009 -0.1542 0.0249 0.0589 0.0358
-0.1694 1.8843 -42.3361
50.3000 -39.9800 0.8558 0.0401 -
0.1409 -0.1481 0.0279 0.0581 0.0329
-0.1622 1.8869 -42.3358
50.3200 -41.3900 -0.6247 0.0092 -
0.1454 -0.1329 0.0328 0.0570 0.0298
-0.1551 1.8895 -42.3356
50.3400 -40.9500 -0.2170 -0.0473 -
0.1227 -0.1102 0.0394 0.0554 0.0266
-0.1479 1.8921 -42.3353

50.3600	-40.1900	0.3262	0.0475	-
0.0855	-0.0821	0.0475	0.0533	0.0234
	-0.1407	1.8947	-42.3350	
50.3800	-40.2200	0.0404	0.1617	-
0.0437	-0.0510	0.0568	0.0508	0.0200
	-0.1335	1.8972	-42.3347	
50.4000	-40.4000	0.0117	0.1135	-
0.0011	-0.0195	0.0670	0.0477	0.0166
	-0.1262	1.8998	-42.3344	
50.4200	-40.6500	-0.0444	-0.1066	
0.0429	0.0099	0.0779	0.0442	0.0132
	-0.1190	1.9023	-42.3341	
50.4400	-40.2400	0.3440	-0.3644	
0.0874	0.0352	0.0890	0.0402	0.0097
	-0.1118	1.9048	-42.3337	
50.4600	-41.3800	-0.8536	-0.3068	
0.1208	0.0551	0.1001	0.0356	0.0063
	-0.1045	1.9073	-42.3334	
50.4800	-39.8700	0.2263	0.1743	
0.1276	0.0694	0.1108	0.0305	0.0028
	-0.0973	1.9098	-42.3331	
50.5000	-38.8100	0.8445	0.5375	
0.0949	0.0786	0.1208	0.0249	-0.0006
	-0.0901	1.9122	-42.3328	
50.5200	-40.7000	-0.6965	0.3509	
0.0212	0.0832	0.1299	0.0187	-0.0040
	-0.0828	1.9147	-42.3325	
50.5400	-40.3700	0.1391	-0.1111	-
0.0687	0.0839	0.1377	0.0120	-0.0073
	-0.0756	1.9171	-42.3322	
50.5600	-40.6800	0.1294	-0.4377	-
0.1401	0.0816	0.1442	0.0048	-0.0106
	-0.0684	1.9195	-42.3319	
50.5800	-41.0900	-0.2554	-0.4747	-
0.1638	0.0769	0.1491	-0.0030	-0.0138
	-0.0613	1.9219	-42.3315	
50.6000	-40.3500	0.2582	-0.2565	-
0.1292	0.0700	0.1524	-0.0112	-0.0169
	-0.0541	1.9243	-42.3312	
50.6200	-40.5000	-0.2953	0.1055	-
0.0552	0.0614	0.1541	-0.0199	-0.0198
	-0.0470	1.9266	-42.3309	
50.6400	-39.9200	-0.0696	0.3812	
0.0307	0.0512	0.1541	-0.0291	-0.0227
	-0.0399	1.9290	-42.3305	
50.6600	-39.2200	0.6764	0.3229	
0.0992	0.0394	0.1526	-0.0387	-0.0254
	-0.0328	1.9313	-42.3302	
50.6800	-40.8600	-0.6836	-0.0220	
0.1329	0.0264	0.1496	-0.0486	-0.0279
	-0.0257	1.9336	-42.3299	

50.7000 -40.2000 0.1488 -0.2393
0.1300 0.0126 0.1452 -0.0589 -0.0303
-0.0187 1.9359 -42.3295
50.7200 -40.2300 0.2892 -0.1696
0.0952 -0.0015 0.1395 -0.0695 -0.0325
-0.0117 1.9382 -42.3292
50.7400 -40.7300 -0.3774 0.0632
0.0399 -0.0151 0.1328 -0.0804 -0.0345
-0.0048 1.9404 -42.3288
50.7600 -39.9900 0.3148 0.2245 -
0.0166 -0.0271 0.1251 -0.0915 -0.0363
0.0021 1.9426 -42.3285
50.7800 -40.6800 -0.2794 0.1309 -
0.0552 -0.0365 0.1167 -0.1028 -0.0379
0.0090 1.9448 -42.3282
50.8000 -40.2100 0.3869 -0.1614 -
0.0642 -0.0429 0.1077 -0.1143 -0.0392
0.0158 1.9470 -42.3278
50.8200 -41.1200 -0.3161 -0.3496 -
0.0404 -0.0457 0.0984 -0.1258 -0.0404
0.0225 1.9492 -42.3274
50.8400 -40.6400 -0.1484 -0.1488
0.0045 -0.0449 0.0888 -0.1375 -0.0412
0.0293 1.9513 -42.3271
50.8600 -39.8700 0.3448 0.2395
0.0412 -0.0407 0.0791 -0.1491 -0.0419
0.0359 1.9535 -42.3267
50.8800 -40.3400 -0.2225 0.4193
0.0419 -0.0339 0.0695 -0.1608 -0.0422
0.0425 1.9556 -42.3264
50.9000 -39.7000 0.4422 0.2206 -
0.0038 -0.0253 0.0602 -0.1724 -0.0423
0.0490 1.9577 -42.3260
50.9200 -40.3900 0.2467 -0.1608 -
0.0712 -0.0157 0.0512 -0.1838 -0.0421
0.0555 1.9597 -42.3256
50.9400 -41.8700 -0.8262 -0.3800 -
0.1235 -0.0059 0.0425 -0.1952 -0.0417
0.0619 1.9618 -42.3253
50.9600 -40.6200 0.2743 -0.2590 -
0.1267 0.0031 0.0344 -0.2062 -0.0409
0.0682 1.9638 -42.3249
50.9800 -39.6500 0.8041 -0.0456 -
0.0752 0.0105 0.0267 -0.2171 -0.0398
0.0745 1.9658 -42.3245
51.0000 -41.0100 -0.7365 0.0600
0.0138 0.0155 0.0194 -0.2276 -0.0383
0.0807 1.9678 -42.3242
51.0200 -39.9600 0.2392 0.1952
0.1032 0.0176 0.0127 -0.2377 -0.0366
0.0868 1.9697 -42.3238

51.0400 -39.8700 0.0413 0.3681
0.1557 0.0167 0.0063 -0.2475 -0.0345
0.0929 1.9717 -42.3234
51.0600 -40.2400 -0.1514 0.3179
0.1552 0.0134 0.0001 -0.2568 -0.0321
0.0988 1.9736 -42.3230
51.0800 -40.0300 0.3562 -0.0175
0.1098 0.0087 -0.0059 -0.2656 -0.0293
0.1047 1.9755 -42.3226
51.1000 -41.2900 -0.4541 -0.3303
0.0438 0.0041 -0.0118 -0.2738 -0.0261
0.1105 1.9773 -42.3223
51.1200 -40.8800 0.0766 -0.3526 -
0.0173 0.0009 -0.0179 -0.2814 -0.0226
0.1163 1.9792 -42.3219
51.1400 -40.1900 0.5541 -0.1534 -
0.0633 0.0005 -0.0242 -0.2885 -0.0187
0.1219 1.9810 -42.3215
51.1600 -41.3000 -0.7062 0.1133 -
0.0949 0.0045 -0.0309 -0.2948 -0.0145
0.1274 1.9828 -42.3211
51.1800 -40.0200 0.2888 0.3333 -
0.1231 0.0142 -0.0381 -0.3005 -0.0098
0.1329 1.9846 -42.3207
51.2000 -39.8200 0.4326 0.3748 -
0.1585 0.0301 -0.0458 -0.3054 -0.0048
0.1383 1.9863 -42.3203
51.2200 -41.1200 -0.5869 0.1796 -
0.1996 0.0513 -0.0541 -0.3095 0.0006
0.1436 1.9881 -42.3199
51.2400 -40.3200 0.5347 -0.1377 -
0.2285 0.0759 -0.0629 -0.3129 0.0064
0.1488 1.9898 -42.3195
51.2600 -41.0700 -0.1085 -0.3668 -
0.2230 0.1009 -0.0722 -0.3154 0.0125
0.1539 1.9914 -42.3191
51.2800 -41.2800 -0.4051 -0.3363 -
0.1654 0.1233 -0.0819 -0.3170 0.0191
0.1589 1.9931 -42.3187
51.3000 -40.5600 -0.0329 -0.0360 -
0.0552 0.1400 -0.0917 -0.3178 0.0260
0.1638 1.9947 -42.3183
51.3200 -39.8300 0.2455 0.2605
0.0901 0.1482 -0.1015 -0.3177 0.0333
0.1686 1.9963 -42.3178
51.3400 -39.4100 0.5221 0.2261
0.2424 0.1457 -0.1112 -0.3167 0.0410
0.1734 1.9979 -42.3174
51.3600 -40.7700 -0.7121 -0.0673
0.3717 0.1308 -0.1205 -0.3148 0.0490
0.1780 1.9995 -42.3170

51.3800 -39.7100 0.4174 -0.1846
0.4560 0.1023 -0.1292 -0.3121 0.0574
0.1826 2.0010 -42.3166
51.4000 -40.2400 -0.3270 0.0035
0.4828 0.0607 -0.1370 -0.3084 0.0661
0.1870 2.0025 -42.3162
51.4200 -39.7800 0.0841 0.2268
0.4402 0.0088 -0.1436 -0.3039 0.0752
0.1914 2.0040 -42.3158
51.4400 -39.9100 0.0753 0.2335
0.3296 -0.0497 -0.1487 -0.2985 0.0847
0.1957 2.0054 -42.3153
51.4600 -40.2500 0.2281 -0.0132
0.1657 -0.1102 -0.1521 -0.2924 0.0944
0.1999 2.0069 -42.3149
51.4800 -40.8700 -0.0326 -0.2929 -
0.0273 -0.1676 -0.1534 -0.2854 0.1045
0.2040 2.0083 -42.3145
51.5000 -41.8700 -0.6416 -0.2895 -
0.2220 -0.2168 -0.1523 -0.2777 0.1148
0.2080 2.0096 -42.3140
51.5200 -40.5100 0.4862 0.0333 -
0.3946 -0.2530 -0.1488 -0.2693 0.1255
0.2119 2.0110 -42.3136
51.5400 -40.6200 0.2977 0.2696 -
0.5233 -0.2720 -0.1424 -0.2602 0.1365
0.2157 2.0123 -42.3132
51.5600 -41.4200 -0.3623 0.1720 -
0.5886 -0.2709 -0.1332 -0.2506 0.1477
0.2195 2.0136 -42.3127
51.5800 -41.7400 -0.6478 -0.0617 -
0.5739 -0.2493 -0.1212 -0.2404 0.1592
0.2231 2.0149 -42.3123
51.6000 -40.3000 0.9162 -0.2863 -
0.4652 -0.2094 -0.1066 -0.2296 0.1710
0.2267 2.0161 -42.3118
51.6200 -41.8000 -0.8372 -0.3975 -
0.2641 -0.1554 -0.0898 -0.2185 0.1830
0.2302 2.0173 -42.3114
51.6400 -40.1700 0.2428 -0.1640 -
0.0043 -0.0923 -0.0712 -0.2069 0.1953
0.2335 2.0185 -42.3109
51.6600 -39.4600 0.0784 0.2400
0.2613 -0.0253 -0.0510 -0.1950 0.2077
0.2369 2.0196 -42.3105
51.6800 -38.9100 0.2028 0.4164
0.4711 0.0406 -0.0297 -0.1829 0.2205
0.2401 2.0208 -42.3100
51.7000 -39.0200 -0.0845 0.3002
0.5735 0.1001 -0.0077 -0.1707 0.2334
0.2432 2.0219 -42.3096

51.7200 -38.9800 0.1123 0.1370
0.5462 0.1488 0.0147 -0.1583 0.2465
0.2463 2.0229 -42.3091
51.7400 -39.4100 -0.1961 0.0759
0.4031 0.1842 0.0371 -0.1458 0.2598
0.2492 2.0240 -42.3087
51.7600 -39.3600 0.1180 0.0358
0.1864 0.2056 0.0592 -0.1334 0.2733
0.2521 2.0250 -42.3082
51.7800 -39.4900 0.2221 -0.1070 -
0.0465 0.2132 0.0807 -0.1211 0.2869
0.2549 2.0259 -42.3077
51.8000 -40.1900 -0.2554 -0.2886 -
0.2395 0.2074 0.1012 -0.1090 0.3008
0.2576 2.0269 -42.3073
51.8200 -40.3900 -0.2942 -0.2895 -
0.3501 0.1886 0.1204 -0.0971 0.3147
0.2603 2.0278 -42.3068
51.8400 -39.4500 0.4265 -0.1098 -
0.3619 0.1578 0.1382 -0.0855 0.3288
0.2628 2.0287 -42.3063
51.8600 -39.7800 0.1106 -0.0067 -
0.2841 0.1175 0.1543 -0.0742 0.3430
0.2653 2.0296 -42.3058
51.8800 -39.7000 -0.0249 -0.0590 -
0.1462 0.0714 0.1685 -0.0634 0.3574
0.2677 2.0304 -42.3054
51.9000 -39.9600 -0.6281 -0.0027
0.0128 0.0239 0.1806 -0.0531 0.3718
0.2700 2.0312 -42.3049
51.9200 -38.5700 0.7926 0.1422
0.1481 -0.0204 0.1905 -0.0433 0.3864
0.2722 2.0320 -42.3044
51.9400 -39.4500 -0.4223 0.1114
0.2211 -0.0573 0.1981 -0.0341 0.4010
0.2743 2.0327 -42.3039
51.9600 -39.3000 -0.0706 0.0373
0.2105 -0.0834 0.2033 -0.0255 0.4157
0.2764 2.0334 -42.3034
51.9800 -39.0900 0.2308 0.0664
0.1195 -0.0977 0.2061 -0.0177 0.4305
0.2784 2.0341 -42.3029
52.0000 -39.4100 -0.0102 0.0552 -
0.0201 -0.1005 0.2066 -0.0106 0.4453
0.2803 2.0347 -42.3024
52.0200 -39.5800 -0.0005 -0.1143 -
0.1606 -0.0929 0.2049 -0.0043 0.4602
0.2821 2.0353 -42.3020
52.0400 -39.8100 0.0272 -0.2715 -
0.2583 -0.0763 0.2012 0.0013 0.4751
0.2839 2.0359 -42.3015

52.0600 -39.8900 -0.2338 -0.2311 -
0.2842 -0.0525 0.1956 0.0060 0.4899
0.2856 2.0364 -42.3010
52.0800 -39.4600 0.1133 -0.0036 -
0.2345 -0.0234 0.1881 0.0099 0.5048
0.2872 2.0370 -42.3005
52.1000 -39.0400 0.1832 0.1834 -
0.1294 0.0076 0.1790 0.0130 0.5197
0.2887 2.0374 -42.3000
52.1200 -38.8100 0.3127 0.1411
0.0040 0.0370 0.1683 0.0154 0.5345
0.2902 2.0379 -42.2995
52.1400 -39.4200 -0.3127 -0.0517
0.1362 0.0615 0.1563 0.0169 0.5493
0.2915 2.0383 -42.2989
52.1600 -39.2900 -0.2399 -0.1178
0.2415 0.0787 0.1431 0.0176 0.5640
0.2928 2.0387 -42.2984
52.1800 -38.9000 -0.0444 0.0188
0.3027 0.0867 0.1288 0.0176 0.5786
0.2941 2.0390 -42.2979
52.2000 -38.3000 0.3544 0.1708
0.3101 0.0846 0.1134 0.0169 0.5931
0.2952 2.0393 -42.2974
52.2200 -39.0000 -0.3293 0.1666
0.2601 0.0727 0.0972 0.0155 0.6076
0.2963 2.0396 -42.2969
52.2400 -38.6500 0.4837 0.0060
0.1617 0.0526 0.0801 0.0135 0.6218
0.2973 2.0399 -42.2964
52.2600 -39.7500 -0.4985 -0.1543
0.0347 0.0275 0.0624 0.0109 0.6359
0.2982 2.0401 -42.2959
52.2800 -39.0200 0.3970 -0.1379 -
0.0972 0.0011 0.0440 0.0078 0.6499
0.2991 2.0402 -42.2953
52.3000 -39.9600 -0.4044 0.0262 -
0.2126 -0.0228 0.0250 0.0042 0.6636
0.2999 2.0404 -42.2948
52.3200 -39.0500 0.3271 0.1505 -
0.2942 -0.0408 0.0056 0.0003 0.6772
0.3006 2.0405 -42.2943
52.3400 -39.5000 0.0316 0.0502 -
0.3293 -0.0509 -0.0142 -0.0040 0.6905
0.3012 2.0406 -42.2937
52.3600 -39.9200 -0.0986 -0.1619 -
0.3105 -0.0519 -0.0343 -0.0086 0.7035
0.3018 2.0406 -42.2932
52.3800 -40.0700 -0.3838 -0.1921 -
0.2367 -0.0448 -0.0545 -0.0133 0.7163
0.3023 2.0406 -42.2927

52.4000 −38.9000 0.6319 −0.0573 −
0.1173 −0.0314 −0.0746 −0.0181 0.7289
0.3028 2.0406 −42.2921
52.4200 −39.6900 −0.4425 0.0378
0.0280 −0.0141 −0.0946 −0.0230 0.7411
0.3031 2.0405 −42.2916
52.4400 −39.1800 −0.1393 0.1436
0.1661 0.0046 −0.1142 −0.0279 0.7530
0.3034 2.0404 −42.2911
52.4600 −38.7900 0.0194 0.2750
0.2655 0.0216 −0.1333 −0.0327 0.7645
0.3037 2.0402 −42.2905
52.4800 −38.1900 0.6715 0.1922
0.3065 0.0341 −0.1518 −0.0373 0.7757
0.3038 2.0401 −42.2900
52.5000 −39.4500 −0.2873 −0.1264
0.2875 0.0403 −0.1695 −0.0416 0.7865
0.3039 2.0399 −42.2894
52.5200 −39.8900 −0.5250 −0.2970
0.2261 0.0395 −0.1863 −0.0457 0.7969
0.3039 2.0396 −42.2889
52.5400 −39.4600 −0.0264 −0.0965
0.1428 0.0320 −0.2020 −0.0494 0.8069
0.3039 2.0393 −42.2883
52.5600 −38.6800 0.4703 0.1836
0.0519 0.0189 −0.2164 −0.0527 0.8164
0.3038 2.0390 −42.2878
52.5800 −38.8300 0.3912 0.2007 −
0.0348 0.0020 −0.2296 −0.0554 0.8255
0.3036 2.0386 −42.2872
52.6000 −39.8800 −0.3632 −0.0205 −
0.1051 −0.0164 −0.2413 −0.0575 0.8341
0.3034 2.0382 −42.2867
52.6200 −40.0900 −0.2656 −0.1854 −
0.1530 −0.0336 −0.2514 −0.0590 0.8422
0.3031 2.0378 −42.2861
52.6400 −39.5500 0.1178 −0.1048 −
0.1784 −0.0470 −0.2600 −0.0597 0.8499
0.3027 2.0373 −42.2855
52.6600 −39.4500 0.1251 0.0765 −
0.1795 −0.0549 −0.2669 −0.0597 0.8569
0.3023 2.0368 −42.2850
52.6800 −39.4000 −0.0852 0.1464 −
0.1568 −0.0565 −0.2722 −0.0587 0.8635
0.3018 2.0363 −42.2844
52.7000 −39.7300 −0.1124 0.0401 −
0.1117 −0.0514 −0.2759 −0.0569 0.8695
0.3012 2.0357 −42.2838
52.7200 −39.3500 0.1478 −0.1499 −
0.0475 −0.0399 −0.2782 −0.0541 0.8749
0.3006 2.0351 −42.2833

52.7400 -39.8700 -0.3494 -0.2012
0.0287 -0.0234 -0.2792 -0.0503 0.8797
0.2999 2.0344 -42.2827
52.7600 -39.4300 -0.1449 0.0032
0.1044 -0.0038 -0.2788 -0.0454 0.8839
0.2992 2.0337 -42.2821
52.7800 -38.4300 0.6275 0.1916
0.1570 0.0169 -0.2774 -0.0395 0.8876
0.2984 2.0330 -42.2815
52.8000 -39.6000 -0.4638 0.1066
0.1625 0.0367 -0.2750 -0.0324 0.8906
0.2975 2.0322 -42.2809
52.8200 -39.0900 0.0107 0.0217
0.1174 0.0536 -0.2718 -0.0242 0.8930
0.2966 2.0314 -42.2804
52.8400 -39.3100 -0.0656 0.0800
0.0389 0.0663 -0.2681 -0.0149 0.8948
0.2956 2.0306 -42.2798
52.8600 -38.9600 0.3289 0.0264 -
0.0484 0.0738 -0.2641 -0.0045 0.8959
0.2945 2.0297 -42.2792
52.8800 -39.7000 -0.3864 -0.1166 -
0.1180 0.0759 -0.2599 0.0071 0.8965
0.2934 2.0288 -42.2786
52.9000 -39.3300 0.1515 -0.1444 -
0.1477 0.0728 -0.2556 0.0197 0.8964
0.2923 2.0278 -42.2780
52.9200 -39.4200 0.0343 -0.1085 -
0.1248 0.0651 -0.2514 0.0335 0.8957
0.2910 2.0268 -42.2774
52.9400 -39.3400 0.0296 -0.0868 -
0.0495 0.0534 -0.2473 0.0482 0.8944
0.2898 2.0258 -42.2768
52.9600 -39.1000 0.0968 -0.0458
0.0629 0.0383 -0.2434 0.0640 0.8924
0.2884 2.0247 -42.2762
52.9800 -39.4100 -0.5477 0.1092
0.1761 0.0209 -0.2397 0.0807 0.8898
0.2870 2.0236 -42.2756
53.0000 -38.2100 0.5649 0.2949
0.2463 0.0022 -0.2362 0.0982 0.8866
0.2856 2.0224 -42.2750
53.0200 -39.1200 -0.4392 0.2139
0.2416 -0.0162 -0.2328 0.1166 0.8828
0.2841 2.0212 -42.2744
53.0400 -38.8300 0.2803 -0.0675
0.1623 -0.0331 -0.2294 0.1358 0.8783
0.2826 2.0200 -42.2738
53.0600 -39.6000 -0.0605 -0.2610
0.0362 -0.0468 -0.2260 0.1555 0.8732
0.2810 2.0187 -42.2732

53.0800 -39.5900 -0.1381 -0.2232 -
0.1042 -0.0558 -0.2224 0.1759 0.8674
0.2793 2.0174 -42.2726
53.1000 -39.2900 0.1002 0.0535 -
0.2218 -0.0585 -0.2184 0.1967 0.8611
0.2776 2.0161 -42.2720
53.1200 -39.4300 -0.2282 0.2857 -
0.2817 -0.0545 -0.2140 0.2178 0.8541
0.2759 2.0147 -42.2714
53.1400 -38.5400 0.8824 0.0429 -
0.2671 -0.0439 -0.2089 0.2393 0.8464
0.2741 2.0133 -42.2707
53.1600 -40.5500 -0.9320 -0.3875 -
0.1831 -0.0275 -0.2032 0.2608 0.8382
0.2722 2.0118 -42.2701
53.1800 -39.0600 0.3637 -0.2790 -
0.0524 -0.0067 -0.1967 0.2824 0.8293
0.2704 2.0103 -42.2695
53.2000 -38.4900 0.3150 0.1498
0.0887 0.0159 -0.1893 0.3040 0.8197
0.2684 2.0088 -42.2689
53.2200 -38.2800 0.0487 0.3987
0.1997 0.0378 -0.1809 0.3253 0.8096
0.2665 2.0072 -42.2682
53.2400 -38.5900 -0.0962 0.3209
0.2487 0.0565 -0.1716 0.3464 0.7988
0.2645 2.0056 -42.2676
53.2600 -38.7200 0.0338 0.0245
0.2313 0.0701 -0.1612 0.3670 0.7874
0.2624 2.0040 -42.2670
53.2800 -38.8200 0.2136 -0.2931
0.1658 0.0782 -0.1496 0.3871 0.7754
0.2603 2.0023 -42.2663
53.3000 -39.6500 -0.4095 -0.3843
0.0788 0.0806 -0.1366 0.4066 0.7627
0.2582 2.0005 -42.2657
53.3200 -39.7600 -0.5557 -0.1443 -
0.0061 0.0782 -0.1221 0.4253 0.7495
0.2561 1.9988 -42.2651
53.3400 -37.8900 0.8343 0.1923 -
0.0735 0.0724 -0.1061 0.4431 0.7356
0.2539 1.9970 -42.2644
53.3600 -38.2100 0.4242 0.2344 -
0.1132 0.0644 -0.0884 0.4598 0.7211
0.2516 1.9951 -42.2638
53.3800 -39.5900 -0.7137 0.0212 -
0.1257 0.0548 -0.0689 0.4755 0.7060
0.2494 1.9932 -42.2631
53.4000 -39.3700 -0.1865 -0.1372 -
0.1207 0.0439 -0.0476 0.4900 0.6904
0.2471 1.9913 -42.2625

53.4200	-38.6800	0.2845	-0.0935	-
0.1065	0.0320	-0.0245	0.5032	0.6741
	0.2448	1.9893	-42.2618	
53.4400	-38.8500	0.0992	0.0298	-
0.0882	0.0194	0.0006	0.5151	0.6572
	0.2424	1.9873	-42.2612	
53.4600	-39.2500	-0.4478	0.1386	-
0.0644	0.0062	0.0277	0.5255	0.6398
	0.2400	1.9853	-42.2605	
53.4800	-38.1500	0.5163	0.1288	-
0.0293	-0.0070	0.0568	0.5344	0.6218
	0.2376	1.9832	-42.2599	
53.5000	-39.2400	-0.3617	-0.0465	
0.0216	-0.0204	0.0880	0.5416	0.6032
	0.2352	1.9811	-42.2592	
53.5200	-38.7200	0.1868	-0.2006	
0.0883	-0.0337	0.1214	0.5473	0.5841
	0.2327	1.9789	-42.2586	
53.5400	-39.1400	-0.3381	-0.1660	
0.1663	-0.0464	0.1568	0.5512	0.5644
	0.2303	1.9768	-42.2579	
53.5600	-38.3300	0.3880	-0.0266	
0.2370	-0.0575	0.1941	0.5533	0.5442
	0.2278	1.9745	-42.2572	
53.5800	-38.8800	-0.5233	0.1502	
0.2657	-0.0663	0.2333	0.5537	0.5234
	0.2252	1.9722	-42.2566	
53.6000	-38.0600	0.2738	0.2780	
0.2219	-0.0717	0.2742	0.5521	0.5022
	0.2227	1.9699	-42.2559	
53.6200	-37.9800	0.5259	0.1577	
0.0962	-0.0738	0.3165	0.5487	0.4804
	0.2201	1.9676	-42.2552	
53.6400	-39.6400	-0.6566	-0.1417	-
0.0870	-0.0735	0.3600	0.5433	0.4582
	0.2175	1.9652	-42.2546	
53.6600	-38.7700	0.4899	-0.2525	-
0.2807	-0.0729	0.4044	0.5360	0.4355
	0.2149	1.9627	-42.2539	
53.6800	-39.7800	-0.3831	-0.1049	-
0.4276	-0.0739	0.4493	0.5267	0.4124
	0.2123	1.9603	-42.2532	
53.7000	-39.1200	0.2437	-0.0275	-
0.4839	-0.0779	0.4940	0.5154	0.3888
	0.2097	1.9578	-42.2525	
53.7200	-39.0100	0.1581	-0.0675	-
0.4290	-0.0854	0.5382	0.5021	0.3648
	0.2070	1.9552	-42.2519	
53.7400	-39.7300	-0.5692	-0.0461	-
0.2722	-0.0963	0.5810	0.4869	0.3405
	0.2044	1.9526	-42.2512	

53.7600 -38.3800 0.4771 0.0786 -
0.0403 -0.1094 0.6219 0.4696 0.3157
0.2017 1.9500 -42.2505
53.7800 -38.2900 0.2771 0.0695
0.2327 -0.1226 0.6603 0.4504 0.2906
0.1990 1.9473 -42.2498
53.8000 -38.8700 -0.4592 -0.1132
0.5049 -0.1336 0.6954 0.4292 0.2652
0.1963 1.9446 -42.2491
53.8200 -38.2600 0.2183 -0.2014
0.7259 -0.1400 0.7267 0.4061 0.2395
0.1936 1.9419 -42.2484
53.8400 -38.3100 -0.2187 -0.0073
0.8438 -0.1393 0.7532 0.3810 0.2135
0.1909 1.9391 -42.2477
53.8600 -37.7000 0.0621 0.3582
0.8160 -0.1297 0.7745 0.3540 0.1872
0.1882 1.9363 -42.2470
53.8800 -37.5100 0.2418 0.6407
0.6194 -0.1102 0.7897 0.3252 0.1607
0.1855 1.9334 -42.2463
53.9000 -38.2100 -0.2251 0.7059
0.2601 -0.0804 0.7983 0.2946 0.1340
0.1827 1.9305 -42.2456
53.9200 -38.7900 -0.2980 0.5739 -
0.2093 -0.0400 0.7996 0.2622 0.1071
0.1800 1.9276 -42.2449
53.9400 -38.8500 0.6474 0.1550 -
0.6983 0.0113 0.7931 0.2282 0.0800
0.1773 1.9246 -42.2442
53.9600 -41.1900 -0.4663 -0.5835 -
1.0951 0.0738 0.7783 0.1927 0.0528
0.1746 1.9216 -42.2435
53.9800 -41.3500 0.2190 -1.1094 -
1.2933 0.1476 0.7548 0.1557 0.0254
0.1718 1.9185 -42.2428
54.0000 -41.6100 -0.5769 -0.7686 -
1.2227 0.2312 0.7224 0.1175 -0.0021
0.1691 1.9154 -42.2421
54.0200 -39.3100 0.4588 0.1774 -
0.9197 0.3189 0.6806 0.0782 -0.0297
0.1664 1.9123 -42.2414
54.0400 -38.6500 0.1950 0.8232 -
0.4648 0.4032 0.6295 0.0380 -0.0574
0.1637 1.9091 -42.2407
54.0600 -38.4400 -0.0420 0.7502
0.0579 0.4760 0.5689 -0.0030 -0.0851
0.1609 1.9059 -42.2400
54.0800 -38.9400 -0.1968 0.2314
0.5665 0.5292 0.4989 -0.0445 -0.1128
0.1582 1.9026 -42.2392

54.1000 -38.6200 0.1319 -0.3482
0.9733 0.5538 0.4197 -0.0864 -0.1405
0.1555 1.8993 -42.2385
54.1200 -39.1400 -0.1597 -0.6490
1.2024 0.5403 0.3321 -0.1284 -0.1683
0.1528 1.8960 -42.2378
54.1400 -39.1800 -0.1415 -0.3244
1.2004 0.4827 0.2369 -0.1704 -0.1959
0.1501 1.8926 -42.2371
54.1600 -39.6000 -0.8879 0.4938
0.9660 0.3841 0.1358 -0.2122 -0.2236
0.1475 1.8892 -42.2363
54.1800 -37.7700 1.3041 0.8514
0.5468 0.2544 0.0305 -0.2536 -0.2511
0.1448 1.8857 -42.2356
54.2000 -39.9700 0.5608 0.2413
0.0340 0.1049 -0.0773 -0.2944 -0.2785
0.1421 1.8822 -42.2349
54.2200 -43.6100 -1.4553 -0.5602 -
0.4450 -0.0529 -0.1857 -0.3343 -0.3058
0.1395 1.8787 -42.2341
54.2400 -41.9400 1.0038 -0.6443 -
0.7652 -0.2075 -0.2931 -0.3733 -0.3330
0.1369 1.8751 -42.2334
54.2600 -42.5900 0.2253 -0.2955 -
0.8555 -0.3480 -0.3976 -0.4110 -0.3600
0.1343 1.8715 -42.2327
54.2800 -42.8200 0.0724 -0.0659 -
0.7318 -0.4645 -0.4975 -0.4474 -0.3868
0.1317 1.8678 -42.2319
54.3000 -43.0800 -0.5497 0.1930 -
0.4777 -0.5517 -0.5911 -0.4822 -0.4134
0.1291 1.8641 -42.2312
54.3200 -41.8100 0.6303 0.3569 -
0.1892 -0.6074 -0.6768 -0.5153 -0.4398
0.1265 1.8604 -42.2304
54.3400 -42.9900 -0.5706 0.2330
0.0546 -0.6311 -0.7531 -0.5463 -0.4659
0.1240 1.8566 -42.2297
54.3600 -42.0900 0.4750 0.0560
0.2111 -0.6248 -0.8190 -0.5752 -0.4918
0.1215 1.8528 -42.2290
54.3800 -43.0000 -0.2658 -0.0940
0.2769 -0.5928 -0.8734 -0.6018 -0.5173
0.1190 1.8489 -42.2282
54.4000 -42.6700 0.1721 -0.1171
0.2683 -0.5420 -0.9158 -0.6259 -0.5425
0.1165 1.8451 -42.2274
54.4200 -43.2600 -0.5770 0.0847
0.2037 -0.4798 -0.9457 -0.6472 -0.5674
0.1141 1.8411 -42.2267

54.4400 -41.7800 0.9022 0.1645
0.1120 -0.4130 -0.9630 -0.6656 -0.5920
0.1117 1.8371 -42.2259
54.4600 -43.6800 -0.5904 -0.0352
0.0253 -0.3482 -0.9676 -0.6811 -0.6161
0.1093 1.8331 -42.2252
54.4800 -43.6400 -0.5671 -0.1667 -
0.0274 -0.2916 -0.9597 -0.6934 -0.6399
0.1069 1.8291 -42.2244
54.5000 -42.4300 0.6445 -0.1117 -
0.0296 -0.2475 -0.9395 -0.7027 -0.6632
0.1046 1.8250 -42.2237
54.5200 -42.6200 0.1336 -0.0209
0.0116 -0.2176 -0.9077 -0.7089 -0.6860
0.1023 1.8208 -42.2229
54.5400 -43.2300 -0.4577 0.0475
0.0785 -0.2007 -0.8654 -0.7121 -0.7084
0.1000 1.8167 -42.2221
54.5600 -42.6900 -0.1815 0.1417
0.1483 -0.1952 -0.8136 -0.7124 -0.7304
0.0977 1.8125 -42.2214
54.5800 -42.0100 0.3848 0.1744
0.2047 -0.1984 -0.7537 -0.7098 -0.7518
0.0955 1.8082 -42.2206
54.6000 -42.1700 0.2437 0.0560
0.2343 -0.2072 -0.6872 -0.7045 -0.7727
0.0934 1.8039 -42.2198
54.6200 -43.1500 -0.5042 -0.0991
0.2302 -0.2176 -0.6159 -0.6965 -0.7931
0.0912 1.7996 -42.2190
54.6400 -42.6200 -0.1323 -0.0622
0.1957 -0.2237 -0.5417 -0.6859 -0.8130
0.0891 1.7952 -42.2183
54.6600 -41.7600 0.5186 0.0686
0.1367 -0.2184 -0.4664 -0.6728 -0.8323
0.0871 1.7908 -42.2175
54.6800 -42.6900 -0.3525 -0.0064
0.0569 -0.1950 -0.3916 -0.6573 -0.8510
0.0850 1.7863 -42.2167
54.7000 -42.3400 0.3758 -0.2034 -
0.0459 -0.1466 -0.3189 -0.6395 -0.8692
0.0830 1.7818 -42.2159
54.7200 -43.0100 -0.5663 -0.0880 -
0.1749 -0.0669 -0.2494 -0.6195 -0.8868
0.0811 1.7773 -42.2151
54.7400 -42.6900 -0.5926 0.3229 -
0.3226 0.0483 -0.1841 -0.5974 -0.9039
0.0792 1.7727 -42.2143
54.7600 -40.8200 0.9778 0.5741 -
0.4817 0.2000 -0.1237 -0.5733 -0.9203
0.0773 1.7681 -42.2136

54.7800 -41.0600 0.6932 0.3242 -
0.6368 0.3852 -0.0684 -0.5473 -0.9362
0.0755 1.7635 -42.2128
54.8000 -43.4200 -1.2544 -0.1498 -
0.7390 0.5950 -0.0184 -0.5195 -0.9515
0.0737 1.7588 -42.2120
54.8200 -40.9500 1.1365 -0.4073 -
0.7185 0.8146 0.0259 -0.4900 -0.9662
0.0720 1.7540 -42.2112
54.8400 -42.6400 -0.7986 -0.6619 -
0.5003 1.0271 0.0643 -0.4589 -0.9803
0.0703 1.7493 -42.2104
54.8600 -41.0100 0.4668 -0.9553 -
0.0845 1.2152 0.0967 -0.4263 -0.9937
0.0687 1.7444 -42.2096
54.8800 -41.1400 -0.5768 -0.6585
0.4278 1.3609 0.1228 -0.3924 -1.0066
0.0671 1.7396 -42.2088
54.9000 -39.0700 -0.0329 0.2712
0.9050 1.4461 0.1424 -0.3572 -1.0188
0.0656 1.7347 -42.2080
54.9200 -37.6500 0.1015 1.2053
1.2126 1.4529 0.1556 -0.3210 -1.0304
0.0641 1.7298 -42.2072
54.9400 -37.0800 0.4813 1.4364
1.2239 1.3669 0.1624 -0.2840 -1.0414
0.0627 1.7248 -42.2064
54.9600 -38.8600 -0.1644 0.7310
0.8883 1.1891 0.1637 -0.2463 -1.0518
0.0613 1.7198 -42.2055
54.9800 -40.2400 0.4181 -0.4303
0.3351 0.9360 0.1603 -0.2081 -1.0616
0.0600 1.7147 -42.2047
55.0000 -43.1300 -0.7972 -1.1338 -
0.2639 0.6274 0.1533 -0.1697 -1.0707
0.0588 1.7096 -42.2039
55.0200 -42.2900 0.4800 -0.8569 -
0.7480 0.2831 0.1436 -0.1313 -1.0791
0.0576 1.7045 -42.2031
55.0400 -42.9900 -0.0190 -0.1928 -
1.0016 -0.0771 0.1322 -0.0932 -1.0870
0.0564 1.6993 -42.2023
55.0600 -42.8600 0.0858 0.0304 -
0.9608 -0.4345 0.1201 -0.0554 -1.0942
0.0553 1.6941 -42.2015
55.0800 -42.4600 0.6267 -0.1603 -
0.6728 -0.7742 0.1083 -0.0184 -1.1008
0.0543 1.6888 -42.2006
55.1000 -44.0200 -1.2094 -0.0599 -
0.2718 -1.0830 0.0978 0.0178 -1.1067
0.0533 1.6835 -42.1998

55.1200 -41.1000 1.1917 0.3787
0.1076 -1.3474 0.0895 0.0529 -1.1120
0.0524 1.6782 -42.1990
55.1400 -41.8100 0.3172 0.4136
0.3690 -1.5553 0.0846 0.0867 -1.1166
0.0516 1.6728 -42.1982
55.1600 -43.6100 -1.0592 0.0954
0.4618 -1.6970 0.0838 0.1190 -1.1206
0.0508 1.6674 -42.1973
55.1800 -42.1200 0.7352 -0.1390
0.4007 -1.7686 0.0880 0.1495 -1.1240
0.0501 1.6620 -42.1965
55.2000 -42.7500 -0.0088 0.0346
0.2355 -1.7717 0.0979 0.1781 -1.1267
0.0494 1.6565 -42.1957
55.2200 -43.5800 -0.6988 0.2759
0.0255 -1.7117 0.1132 0.2047 -1.1287
0.0488 1.6509 -42.1948
55.2400 -42.0500 0.9032 0.0856 -
0.1662 -1.5962 0.1336 0.2291 -1.1301
0.0483 1.6454 -42.1940
55.2600 -43.9800 -0.6909 -0.4314 -
0.2996 -1.4332 0.1584 0.2513 -1.1308
0.0478 1.6397 -42.1932
55.2800 -43.4200 -0.1661 -0.4909 -
0.3626 -1.2311 0.1871 0.2713 -1.1309
0.0474 1.6341 -42.1923
55.3000 -42.5700 -0.2264 0.1022 -
0.3680 -0.9980 0.2189 0.2890 -1.1304
0.0471 1.6284 -42.1915
55.3200 -40.9500 0.6680 0.6548 -
0.3294 -0.7418 0.2533 0.3044 -1.1291
0.0468 1.6226 -42.1906
55.3400 -41.5500 -0.3219 0.5464 -
0.2584 -0.4703 0.2895 0.3175 -1.1273
0.0466 1.6169 -42.1898
55.3600 -41.2700 0.1756 -0.1564 -
0.1525 -0.1911 0.3269 0.3284 -1.1247
0.0464 1.6111 -42.1889
55.3800 -41.3000 0.3407 -0.7696 -
0.0064 0.0886 0.3647 0.3371 -1.1215
0.0463 1.6052 -42.1881
55.4000 -42.1000 -1.0917 -0.6823
0.1764 0.3626 0.4024 0.3435 -1.1177
0.0463 1.5993 -42.1872
55.4200 -39.6800 0.0734 0.0504
0.3630 0.6249 0.4392 0.3478 -1.1132
0.0464 1.5934 -42.1864
55.4400 -37.8100 1.1054 0.6485
0.4962 0.8697 0.4744 0.3499 -1.1081
0.0465 1.5874 -42.1855

55.4600 -39.3400 -0.7587 0.4987
0.5216 1.0913 0.5073 0.3498 -1.1023
0.0466 1.5814 -42.1847
55.4800 -39.2000 -0.1709 0.0391
0.4227 1.2841 0.5372 0.3477 -1.0959
0.0469 1.5753 -42.1838
55.5000 -39.5500 -0.2948 -0.1094
0.2168 1.4445 0.5632 0.3436 -1.0889
0.0471 1.5692 -42.1829
55.5200 -38.6700 0.5030 -0.0670 -
0.0459 1.5705 0.5847 0.3374 -1.0812
0.0475 1.5631 -42.1821
55.5400 -39.9600 -0.4803 -0.0295 -
0.2975 1.6616 0.6009 0.3293 -1.0730
0.0479 1.5569 -42.1812
55.5600 -38.9800 0.4846 -0.0281 -
0.4737 1.7170 0.6111 0.3193 -1.0641
0.0484 1.5507 -42.1803
55.5800 -40.0000 -0.3358 -0.1209 -
0.5323 1.7363 0.6148 0.3075 -1.0546
0.0489 1.5445 -42.1795
55.6000 -39.5600 0.0884 -0.2497 -
0.4555 1.7194 0.6117 0.2939 -1.0446
0.0495 1.5382 -42.1786
55.6200 -39.5000 0.0829 -0.3089 -
0.2507 1.6669 0.6017 0.2786 -1.0340
0.0501 1.5319 -42.1777
55.6400 -39.3600 0.0618 -0.2035
0.0426 1.5794 0.5850 0.2619 -1.0228
0.0508 1.5255 -42.1768
55.6600 -39.3000 -0.3481 0.0854
0.3589 1.4573 0.5619 0.2437 -1.0110
0.0515 1.5191 -42.1760
55.6800 -38.2100 0.5257 0.3515
0.6219 1.3014 0.5327 0.2242 -0.9987
0.0523 1.5126 -42.1751
55.7000 -39.0700 -0.4489 0.3836
0.7606 1.1127 0.4979 0.2036 -0.9858
0.0532 1.5061 -42.1742
55.7200 -38.9700 0.1142 0.3107
0.7299 0.8939 0.4581 0.1820 -0.9724
0.0541 1.4996 -42.1733
55.7400 -39.7800 -0.2810 0.2751
0.5341 0.6516 0.4139 0.1594 -0.9585
0.0551 1.4930 -42.1724
55.7600 -39.8600 0.4572 0.2046
0.2240 0.3945 0.3658 0.1361 -0.9441
0.0561 1.4864 -42.1715
55.7800 -41.1000 0.1365 -0.1250 -
0.1188 0.1326 0.3143 0.1121 -0.9291
0.0571 1.4798 -42.1706

55.8000 -41.9700 0.4282 -0.6426 -
0.4136 -0.1239 0.2600 0.0876 -0.9137
0.0582 1.4731 -42.1698
55.8200 -44.0500 -1.0791 -0.7502 -
0.5943 -0.3651 0.2034 0.0627 -0.8978
0.0594 1.4664 -42.1689
55.8400 -43.9100 -1.1192 -0.1980 -
0.6326 -0.5811 0.1452 0.0375 -0.8815
0.0606 1.4597 -42.1680
55.8600 -40.6400 1.5844 0.4999 -
0.5667 -0.7639 0.0858 0.0122 -0.8646
0.0618 1.4529 -42.1671
55.8800 -43.1200 -0.8899 0.5605 -
0.4516 -0.9077 0.0258 -0.0132 -0.8474
0.0631 1.4460 -42.1662
55.9000 -43.5900 -0.9135 0.2300 -
0.3317 -1.0092 -0.0342 -0.0385 -0.8297
0.0645 1.4392 -42.1653
55.9200 -43.5400 -0.5965 0.0758 -
0.2352 -1.0671 -0.0938 -0.0636 -0.8115
0.0658 1.4322 -42.1644
55.9400 -42.4900 0.4802 0.0357 -
0.1668 -1.0835 -0.1524 -0.0884 -0.7930
0.0672 1.4253 -42.1635
55.9600 -42.0500 1.0344 -0.1354 -
0.1153 -1.0632 -0.2095 -0.1127 -0.7741
0.0687 1.4183 -42.1625
55.9800 -44.2500 -1.1424 -0.2817 -
0.0694 -1.0128 -0.2644 -0.1364 -0.7547
0.0702 1.4113 -42.1616
56.0000 -42.3400 0.7553 -0.1432 -
0.0190 -0.9391 -0.3165 -0.1594 -0.7350
0.0717 1.4042 -42.1607
56.0200 -42.2300 0.5269 0.0065
0.0457 -0.8494 -0.3651 -0.1815 -0.7150
0.0733 1.3971 -42.1598
56.0400 -42.9700 -0.2057 -0.0558
0.1193 -0.7506 -0.4098 -0.2027 -0.6946
0.0749 1.3900 -42.1589
56.0600 -43.2100 -0.5493 -0.1068
0.1899 -0.6494 -0.4499 -0.2229 -0.6738
0.0766 1.3828 -42.1580
56.0800 -42.2500 0.0130 0.0936
0.2465 -0.5521 -0.4848 -0.2418 -0.6527
0.0783 1.3756 -42.1571
56.1000 -41.8600 0.0699 0.3604
0.2746 -0.4645 -0.5140 -0.2594 -0.6313
0.0800 1.3684 -42.1561
56.1200 -41.4600 0.4892 0.3454
0.2659 -0.3915 -0.5369 -0.2756 -0.6096
0.0818 1.3611 -42.1552

56.1400 -41.9900 0.2241 -0.0409
0.2290 -0.3365 -0.5529 -0.2902 -0.5876
0.0835 1.3538 -42.1543
56.1600 -43.0700 -0.4267 -0.4815
0.1816 -0.3013 -0.5616 -0.3033 -0.5653
0.0854 1.3464 -42.1534
56.1800 -43.2700 -0.5362 -0.4921
0.1410 -0.2861 -0.5629 -0.3146 -0.5428
0.0872 1.3391 -42.1524
56.2000 -42.3200 0.0872 -0.0132
0.1133 -0.2891 -0.5569 -0.3242 -0.5200
0.0891 1.3316 -42.1515
56.2200 -41.8300 0.2064 0.4217
0.0873 -0.3071 -0.5438 -0.3322 -0.4970
0.0910 1.3242 -42.1506
56.2400 -40.9700 0.9496 0.3119
0.0496 -0.3361 -0.5241 -0.3385 -0.4737
0.0929 1.3167 -42.1496
56.2600 -43.5100 -1.0177 -0.0989 -
0.0034 -0.3713 -0.4985 -0.3431 -0.4503
0.0949 1.3092 -42.1487
56.2800 -42.8400 -0.2549 -0.1949 -
0.0677 -0.4077 -0.4677 -0.3462 -0.4266
0.0969 1.3016 -42.1478
56.3000 -41.9000 0.4828 0.0213 -
0.1333 -0.4410 -0.4322 -0.3478 -0.4028
0.0989 1.2940 -42.1468
56.3200 -41.7000 0.6140 0.1163 -
0.1836 -0.4677 -0.3927 -0.3479 -0.3787
0.1009 1.2864 -42.1459
56.3400 -42.3500 0.1524 -0.0438 -
0.2064 -0.4842 -0.3498 -0.3465 -0.3546
0.1030 1.2787 -42.1449
56.3600 -43.5200 -0.8836 -0.1487 -
0.1948 -0.4882 -0.3043 -0.3438 -0.3302
0.1051 1.2710 -42.1440
56.3800 -41.8400 0.4285 -0.0245 -
0.1456 -0.4787 -0.2567 -0.3397 -0.3058
0.1072 1.2633 -42.1430
56.4000 -41.5200 0.4041 0.0554 -
0.0607 -0.4558 -0.2078 -0.3343 -0.2812
0.1093 1.2555 -42.1421
56.4200 -42.1000 -0.1058 -0.0540
0.0462 -0.4207 -0.1582 -0.3277 -0.2565
0.1114 1.2477 -42.1411
56.4400 -42.3400 -0.4850 -0.1181
0.1485 -0.3746 -0.1086 -0.3199 -0.2317
0.1136 1.2399 -42.1402
56.4600 -41.2700 0.2232 0.0179
0.2161 -0.3189 -0.0595 -0.3109 -0.2068
0.1158 1.2320 -42.1392

56.4800 -41.0600 0.1314 0.1977
0.2263 -0.2550 -0.0117 -0.3008 -0.1818
0.1180 1.2241 -42.1383
56.5000 -41.3600 -0.2988 0.2082
0.1728 -0.1843 0.0345 -0.2896 -0.1568
0.1202 1.2162 -42.1373
56.5200 -40.7900 0.3491 0.0350
0.0720 -0.1085 0.0782 -0.2774 -0.1318
0.1224 1.2082 -42.1363
56.5400 -41.5000 -0.1842 -0.1980 -
0.0417 -0.0293 0.1190 -0.2643 -0.1067
0.1246 1.2002 -42.1354
56.5600 -41.2700 -0.0073 -0.2487 -
0.1321 0.0508 0.1563 -0.2502 -0.0815
0.1269 1.1922 -42.1344
56.5800 -41.0500 -0.0633 -0.0689 -
0.1735 0.1293 0.1895 -0.2352 -0.0564
0.1291 1.1842 -42.1335
56.6000 -40.6000 0.0935 0.1203 -
0.1585 0.2035 0.2180 -0.2194 -0.0312
0.1314 1.1761 -42.1325
56.6200 -40.2600 0.2295 0.1126 -
0.0941 0.2709 0.2415 -0.2028 -0.0061
0.1337 1.1680 -42.1315
56.6400 -40.5500 -0.0642 -0.0247 -
0.0038 0.3293 0.2592 -0.1855 0.0191
0.1360 1.1598 -42.1305
56.6600 -40.8200 -0.4499 -0.0499
0.0859 0.3764 0.2710 -0.1675 0.0442
0.1383 1.1516 -42.1296
56.6800 -39.7600 0.3437 0.0782
0.1571 0.4104 0.2765 -0.1488 0.0692
0.1406 1.1434 -42.1286
56.7000 -39.5400 0.1898 0.1139
0.1989 0.4298 0.2755 -0.1296 0.0942
0.1429 1.1352 -42.1276
56.7200 -40.3200 -0.2385 -0.0129
0.2074 0.4340 0.2681 -0.1098 0.1192
0.1452 1.1269 -42.1266
56.7400 -40.0000 0.0044 -0.1397
0.1815 0.4232 0.2545 -0.0895 0.1441
0.1475 1.1186 -42.1257
56.7600 -40.2200 0.0346 -0.0963
0.1214 0.3986 0.2352 -0.0688 0.1689
0.1498 1.1102 -42.1247
56.7800 -40.4900 -0.4931 0.1600
0.0346 0.3619 0.2104 -0.0478 0.1936
0.1521 1.1019 -42.1237
56.8000 -39.2900 0.6191 0.3234 -
0.0626 0.3157 0.1809 -0.0265 0.2182
0.1544 1.0935 -42.1227

56.8200 -40.3400 0.0057 0.0714 -
0.1491 0.2630 0.1472 -0.0051 0.2426
0.1567 1.0851 -42.1217
56.8400 -41.0500 -0.2213 -0.3236 -
0.2015 0.2070 0.1100 0.0164 0.2670
0.1590 1.0766 -42.1207
56.8600 -41.4100 -0.4954 -0.3544 -
0.2001 0.1504 0.0701 0.0379 0.2912
0.1613 1.0681 -42.1197
56.8800 -40.0300 0.3811 0.0006 -
0.1423 0.0954 0.0281 0.0593 0.3153
0.1636 1.0596 -42.1187
56.9000 -40.1600 0.0182 0.2702 -
0.0466 0.0433 -0.0150 0.0804 0.3393
0.1659 1.0511 -42.1177
56.9200 -40.1000 0.1868 0.1560
0.0603 -0.0050 -0.0586 0.1012 0.3630
0.1682 1.0425 -42.1167
56.9400 -40.5700 -0.0167 -0.1078
0.1454 -0.0490 -0.1016 0.1216 0.3866
0.1705 1.0339 -42.1157
56.9600 -41.0100 -0.4957 -0.1266
0.1769 -0.0884 -0.1434 0.1415 0.4100
0.1727 1.0253 -42.1147
56.9800 -40.6100 -0.1841 0.1707
0.1430 -0.1229 -0.1831 0.1607 0.4333
0.1750 1.0167 -42.1137
57.0000 -39.3800 0.7897 0.3582
0.0589 -0.1518 -0.2199 0.1793 0.4563
0.1773 1.0080 -42.1127
57.0200 -40.9500 -0.2561 0.0942 -
0.0480 -0.1748 -0.2531 0.1969 0.4791
0.1795 0.9993 -42.1117
57.0400 -41.4800 -0.4510 -0.2798 -
0.1387 -0.1918 -0.2819 0.2137 0.5017
0.1817 0.9906 -42.1107
57.0600 -41.6700 -0.5096 -0.3005 -
0.1773 -0.2025 -0.3059 0.2294 0.5240
0.1839 0.9818 -42.1097
57.0800 -40.1400 0.7095 -0.0449 -
0.1526 -0.2071 -0.3247 0.2441 0.5461
0.1861 0.9730 -42.1087
57.1000 -40.8400 -0.2362 0.0829 -
0.0777 -0.2059 -0.3378 0.2575 0.5680
0.1883 0.9642 -42.1077
57.1200 -40.4800 0.1875 -0.0135
0.0194 -0.1995 -0.3451 0.2697 0.5896
0.1905 0.9554 -42.1067
57.1400 -40.7900 -0.2267 -0.0375
0.1014 -0.1880 -0.3464 0.2807 0.6109
0.1926 0.9465 -42.1056

57.1600 -40.6700 -0.4149 0.1207
0.1394 -0.1723 -0.3417 0.2902 0.6320
0.1948 0.9377 -42.1046
57.1800 -39.5200 0.6799 0.2649
0.1235 -0.1529 -0.3309 0.2985 0.6527
0.1969 0.9288 -42.1036
57.2000 -40.7900 -0.4919 0.1506
0.0636 -0.1308 -0.3144 0.3053 0.6732
0.1990 0.9198 -42.1026
57.2200 -40.5300 -0.1100 -0.0875 -
0.0158 -0.1066 -0.2923 0.3108 0.6933
0.2010 0.9109 -42.1016
57.2400 -40.4200 0.3571 -0.2454 -
0.0846 -0.0809 -0.2650 0.3150 0.7131
0.2031 0.9019 -42.1005
57.2600 -40.6400 0.0121 -0.2761 -
0.1143 -0.0543 -0.2327 0.3180 0.7326
0.2051 0.8929 -42.0995
57.2800 -40.6900 -0.1955 -0.2028 -
0.0919 -0.0279 -0.1961 0.3197 0.7517
0.2071 0.8839 -42.0985
57.3000 -40.0000 0.0664 0.0046 -
0.0285 -0.0028 -0.1554 0.3202 0.7705
0.2090 0.8748 -42.0974
57.3200 -39.9100 -0.1561 0.2413
0.0467 0.0199 -0.1113 0.3195 0.7889
0.2110 0.8658 -42.0964
57.3400 -39.3200 0.2729 0.3268
0.1000 0.0397 -0.0641 0.3177 0.8070
0.2129 0.8567 -42.0954
57.3600 -39.7000 -0.0446 0.1575
0.1077 0.0556 -0.0146 0.3149 0.8246
0.2148 0.8476 -42.0943
57.3800 -39.7200 0.1479 -0.1239
0.0711 0.0671 0.0369 0.3111 0.8419
0.2166 0.8384 -42.0933
57.4000 -40.4700 -0.4832 -0.2258
0.0072 0.0744 0.0898 0.3063 0.8588
0.2185 0.8293 -42.0923
57.4200 -39.3700 0.3854 -0.0657 -
0.0617 0.0779 0.1437 0.3006 0.8753
0.2202 0.8201 -42.0912
57.4400 -39.6200 0.0879 0.0904 -
0.1152 0.0784 0.1980 0.2941 0.8913
0.2220 0.8109 -42.0902
57.4600 -39.2300 0.4520 0.0260 -
0.1384 0.0767 0.2525 0.2867 0.9070
0.2237 0.8017 -42.0891
57.4800 -40.6400 -0.8092 -0.1078 -
0.1256 0.0731 0.3067 0.2785 0.9222
0.2254 0.7924 -42.0881

57.5000 -39.1800 0.5325 -0.1100 -
0.0787 0.0678 0.3601 0.2696 0.9370
0.2270 0.7832 -42.0870
57.5200 -39.0400 0.4699 -0.0627 -
0.0040 0.0608 0.4123 0.2601 0.9514
0.2287 0.7739 -42.0860
57.5400 -39.4800 -0.2186 -0.0519
0.0844 0.0523 0.4627 0.2499 0.9654
0.2302 0.7646 -42.0849
57.5600 -40.0100 -0.7224 -0.0129
0.1601 0.0424 0.5108 0.2390 0.9789
0.2318 0.7552 -42.0839
57.5800 -38.1500 0.8671 0.1196
0.1938 0.0316 0.5562 0.2277 0.9920
0.2332 0.7459 -42.0828
57.6000 -39.1600 -0.2525 0.2553
0.1640 0.0205 0.5982 0.2159 1.0046
0.2347 0.7365 -42.0817
57.6200 -39.6500 -0.7291 0.2313
0.0697 0.0107 0.6364 0.2036 1.0168
0.2361 0.7272 -42.0807
57.6400 -38.4600 0.9346 -0.0068 -
0.0621 0.0048 0.6703 0.1909 1.0285
0.2375 0.7178 -42.0796
57.6600 -40.5900 -0.8691 -0.2746 -
0.1822 0.0056 0.6994 0.1778 1.0398
0.2388 0.7083 -42.0786
57.6800 -39.2100 0.5099 -0.2686 -
0.2426 0.0154 0.7231 0.1645 1.0507
0.2400 0.6989 -42.0775
57.7000 -39.5900 0.0000 -0.1071 -
0.2244 0.0354 0.7411 0.1509 1.0611
0.2413 0.6894 -42.0764
57.7200 -39.5100 -0.1912 0.0108 -
0.1342 0.0652 0.7529 0.1372 1.0710
0.2424 0.6800 -42.0754
57.7400 -39.0900 0.0099 0.0727 -
0.0023 0.1024 0.7582 0.1232 1.0805
0.2436 0.6705 -42.0743
57.7600 -38.8000 0.0078 0.0847
0.1303 0.1436 0.7567 0.1092 1.0895
0.2446 0.6610 -42.0732
57.7800 -38.5900 0.1490 0.0530
0.2272 0.1852 0.7481 0.0952 1.0980
0.2457 0.6514 -42.0721
57.8000 -38.7700 -0.0207 0.0255
0.2639 0.2229 0.7320 0.0811 1.1061
0.2466 0.6419 -42.0711
57.8200 -38.8700 -0.1610 0.1115
0.2353 0.2519 0.7084 0.0671 1.1137
0.2476 0.6323 -42.0700

57.8400 -38.7900 -0.2555 0.3060
0.1616 0.2672 0.6770 0.0532 1.1209
0.2484 0.6228 -42.0689
57.8600 -38.2700 0.5232 0.3290
0.0830 0.2644 0.6379 0.0395 1.1275
0.2492 0.6132 -42.0678
57.8800 -39.5900 -0.3198 -0.0362
0.0379 0.2405 0.5912 0.0259 1.1337
0.2500 0.6036 -42.0667
57.9000 -39.9400 0.0419 -0.5426
0.0477 0.1951 0.5374 0.0126 1.1395
0.2507 0.5939 -42.0657
57.9200 -40.2400 -0.1642 -0.7927
0.1196 0.1320 0.4771 -0.0004 1.1447
0.2513 0.5843 -42.0646
57.9400 -40.0100 0.0128 -0.6105
0.2367 0.0596 0.4108 -0.0131 1.1495
0.2519 0.5746 -42.0635
57.9600 -40.0000 -0.6605 0.0441
0.3436 -0.0127 0.3392 -0.0254 1.1538
0.2524 0.5650 -42.0624
57.9800 -38.1700 0.5661 0.8829
0.3709 -0.0750 0.2628 -0.0374 1.1577
0.2529 0.5553 -42.0613
58.0000 -38.2600 0.2834 1.2108
0.2535 -0.1195 0.1825 -0.0488 1.1610
0.2533 0.5456 -42.0602
58.0200 -38.9100 0.7426 0.5854 -
0.0344 -0.1419 0.0988 -0.0598 1.1639
0.2536 0.5359 -42.0591
58.0400 -42.2000 -0.9954 -0.3984 -
0.4201 -0.1399 0.0126 -0.0702 1.1663
0.2539 0.5262 -42.0580
58.0600 -42.1100 -0.2148 -0.8851 -
0.8016 -0.1119 -0.0752 -0.0800 1.1683
0.2541 0.5164 -42.0569
58.0800 -41.4400 0.9284 -0.7767 -
1.0642 -0.0571 -0.1639 -0.0891 1.1697
0.2542 0.5067 -42.0558
58.1000 -42.9200 -0.7853 -0.5454 -
1.0865 0.0243 -0.2523 -0.0976 1.1707
0.2543 0.4969 -42.0547
58.1200 -41.1800 0.9269 -0.7609 -
0.8024 0.1271 -0.3395 -0.1054 1.1712
0.2543 0.4871 -42.0536
58.1400 -42.5500 -0.9252 -0.9413 -
0.2901 0.2381 -0.4244 -0.1124 1.1712
0.2543 0.4773 -42.0525
58.1600 -40.5300 0.0789 -0.4194
0.3053 0.3422 -0.5059 -0.1185 1.1707
0.2541 0.4675 -42.0514

58.1800 -38.3600 0.7648 0.4988
0.8372 0.4244 -0.5830 -0.1238 1.1697
0.2540 0.4577 -42.0503
58.2000 -38.9900 -0.8021 1.1651
1.1680 0.4696 -0.6547 -0.1283 1.1683
0.2537 0.4479 -42.0492
58.2200 -37.8500 0.6120 1.0208
1.1943 0.4651 -0.7202 -0.1317 1.1664
0.2534 0.4381 -42.0481
58.2400 -38.7300 0.7801 0.2458
0.9432 0.4074 -0.7788 -0.1342 1.1640
0.2530 0.4282 -42.0470
58.2600 -42.0800 -1.1910 -0.5169
0.5207 0.3040 -0.8300 -0.1357 1.1611
0.2526 0.4184 -42.0458
58.2800 -41.8300 -0.2610 -0.6848
0.0445 0.1654 -0.8733 -0.1361 1.1577
0.2521 0.4085 -42.0447
58.3000 -41.0200 1.1080 -0.4159 -
0.3744 0.0019 -0.9083 -0.1354 1.1539
0.2515 0.3986 -42.0436
58.3200 -43.1300 -0.8184 -0.1773 -
0.6509 -0.1761 -0.9345 -0.1336 1.1495
0.2508 0.3887 -42.0425
58.3400 -42.1200 0.5540 -0.0612 -
0.7528 -0.3577 -0.9516 -0.1306 1.1447
0.2501 0.3788 -42.0414
58.3600 -43.1200 -0.5242 0.0624 -
0.6972 -0.5312 -0.9593 -0.1265 1.1394
0.2494 0.3689 -42.0402
58.3800 -41.8800 0.7058 -0.0105 -
0.5406 -0.6837 -0.9570 -0.1213 1.1337
0.2485 0.3590 -42.0391
58.4000 -43.5100 -0.8336 -0.0582 -
0.3701 -0.8030 -0.9445 -0.1149 1.1275
0.2476 0.3491 -42.0380
58.4200 -41.9100 0.5474 0.1747 -
0.2597 -0.8784 -0.9214 -0.1075 1.1208
0.2467 0.3391 -42.0369
58.4400 -41.9900 0.3125 0.2855 -
0.2466 -0.9026 -0.8876 -0.0990 1.1136
0.2456 0.3292 -42.0357
58.4600 -42.2800 0.2281 0.0491 -
0.3193 -0.8753 -0.8432 -0.0896 1.1060
0.2445 0.3192 -42.0346
58.4800 -43.3500 -0.7297 -0.2114 -
0.4175 -0.8019 -0.7892 -0.0793 1.0980
0.2434 0.3093 -42.0335
58.5000 -42.0400 0.6247 -0.2282 -
0.4691 -0.6909 -0.7265 -0.0682 1.0895
0.2422 0.2993 -42.0323

58.5200 -42.5800 -0.2919 -0.0874 -
0.4271 -0.5523 -0.6562 -0.0564 1.0806
0.2409 0.2893 -42.0312
58.5400 -41.9100 -0.2733 0.0393 -
0.2842 -0.3959 -0.5794 -0.0439 1.0712
0.2395 0.2794 -42.0300
58.5600 -40.6800 0.4974 0.0312 -
0.0604 -0.2299 -0.4970 -0.0309 1.0614
0.2382 0.2694 -42.0289
58.5800 -40.9600 -0.1486 -0.0671
0.2015 -0.0616 -0.4101 -0.0174 1.0512
0.2367 0.2594 -42.0278
58.6000 -40.9400 -0.5584 -0.0417
0.4532 0.1025 -0.3199 -0.0035 1.0406
0.2352 0.2494 -42.0266
58.6200 -39.0400 0.5617 0.1612
0.6529 0.2561 -0.2273 0.0108 1.0295
0.2336 0.2394 -42.0255
58.6400 -39.3500 0.0231 0.2078
0.7697 0.3939 -0.1334 0.0253 1.0181
0.2320 0.2294 -42.0243
58.6600 -39.0100 0.2156 0.0286
0.7735 0.5118 -0.0392 0.0400 1.0063
0.2303 0.2193 -42.0232
58.6800 -39.9300 -0.7402 0.0795
0.6376 0.6079 0.0542 0.0547 0.9941
0.2286 0.2093 -42.0220
58.7000 -38.2000 0.6608 0.4088
0.3639 0.6819 0.1457 0.0695 0.9816
0.2268 0.1993 -42.0209
58.7200 -39.3500 -0.1837 0.4630 -
0.0147 0.7346 0.2344 0.0842 0.9687
0.2249 0.1892 -42.0197
58.7400 -39.8400 0.0042 0.0884 -
0.4393 0.7671 0.3192 0.0988 0.9554
0.2230 0.1792 -42.0186
58.7600 -40.3600 0.2123 -0.3925 -
0.8269 0.7805 0.3995 0.1131 0.9418
0.2210 0.1692 -42.0174
58.7800 -41.5000 -0.5355 -0.6003 -
1.0901 0.7758 0.4744 0.1271 0.9278
0.2190 0.1591 -42.0162
58.8000 -40.6000 0.3632 -0.4709 -
1.1610 0.7540 0.5433 0.1408 0.9135
0.2170 0.1491 -42.0151
58.8200 -40.5500 -0.0767 -0.1959 -
1.0279 0.7163 0.6059 0.1539 0.8989
0.2149 0.1390 -42.0139
58.8400 -40.3300 -0.3175 0.0887 -
0.7272 0.6640 0.6620 0.1666 0.8840
0.2127 0.1290 -42.0128

58.8600 -38.8700 0.4455 0.2836 -
0.3176 0.5990 0.7113 0.1786 0.8688
0.2105 0.1189 -42.0116
58.8800 -39.3600 -0.3868 0.2856
0.1384 0.5229 0.7540 0.1899 0.8532
0.2082 0.1088 -42.0104
58.9000 -38.4500 0.2455 0.1266
0.5967 0.4366 0.7901 0.2004 0.8374
0.2059 0.0988 -42.0093
58.9200 -38.6400 -0.1256 -0.0572
1.0254 0.3399 0.8198 0.2102 0.8213
0.2036 0.0887 -42.0081
58.9400 -38.4200 -0.0945 -0.1766
1.4003 0.2323 0.8431 0.2190 0.8049
0.2011 0.0786 -42.0069
58.9600 -37.8900 0.3638 -0.1669
1.6915 0.1141 0.8603 0.2269 0.7883
0.1987 0.0685 -42.0057
58.9800 -38.3800 -0.4086 0.0153
1.8548 -0.0127 0.8714 0.2337 0.7714
0.1962 0.0585 -42.0046
59.0000 -38.1700 -0.3622 0.4040
1.8255 -0.1456 0.8768 0.2396 0.7543
0.1937 0.0484 -42.0034
59.0200 -37.5700 0.3113 0.7771
1.5584 -0.2814 0.8766 0.2443 0.7369
0.1911 0.0383 -42.0022
59.0400 -37.8200 0.8634 0.6511
1.0578 -0.4152 0.8709 0.2479 0.7194
0.1885 0.0282 -42.0010
59.0600 -40.8300 -0.6589 -0.0174
0.3947 -0.5409 0.8599 0.2503 0.7015
0.1858 0.0181 -41.9999
59.0800 -42.2000 -0.5650 -0.6142 -
0.3190 -0.6519 0.8437 0.2516 0.6835
0.1831 0.0081 -41.9987
59.1000 -42.6900 -0.2739 -0.7200 -
0.9730 -0.7417 0.8225 0.2517 0.6653
0.1803 -0.0020 -41.9975
59.1200 -41.9400 1.0912 -0.5250 -
1.5002 -0.8035 0.7965 0.2506 0.6469
0.1775 -0.0121 -41.9963
59.1400 -44.1900 -1.1393 -0.0734 -
1.8718 -0.8306 0.7660 0.2485 0.6283
0.1747 -0.0222 -41.9951
59.1600 -41.7400 0.9774 0.5236 -
2.0930 -0.8172 0.7311 0.2452 0.6096
0.1718 -0.0323 -41.9939
59.1800 -42.6800 -0.0473 0.6110 -
2.1574 -0.7607 0.6924 0.2408 0.5907
0.1689 -0.0424 -41.9927

59.2000	-43.0900	0.1345	0.0228	-
2.0508	-0.6638	0.6500	0.2353	0.5716
	0.1660	-0.0525	-41.9915	
59.2200	-44.3500	-1.0331	-0.5808	-
1.7617	-0.5342	0.6041	0.2289	0.5524
	0.1630	-0.0625	-41.9903	
59.2400	-42.7200	0.3924	-0.8616	-
1.2893	-0.3817	0.5551	0.2215	0.5331
	0.1600	-0.0726	-41.9892	
59.2600	-41.3100	1.0559	-0.8685	-
0.6565	-0.2162	0.5033	0.2133	0.5136
	0.1569	-0.0827	-41.9880	
59.2800	-42.4300	-1.0610	-0.5879	
0.0793	-0.0469	0.4489	0.2041	0.4940
	0.1538	-0.0928	-41.9868	
59.3000	-40.4900	-0.4845	-0.0087	
0.8286	0.1171	0.3923	0.1942	0.4744
	0.1507	-0.1029	-41.9856	
59.3200	-37.6100	1.0035	0.5989	
1.4916	0.2669	0.3339	0.1836	0.4546
	0.1476	-0.1129	-41.9844	
59.3400	-38.4600	-0.7209	0.8825	
1.9668	0.3934	0.2740	0.1723	0.4348
	0.1444	-0.1230	-41.9831	
59.3600	-37.7200	-0.0416	0.9093	
2.1686	0.4878	0.2130	0.1604	0.4148
	0.1412	-0.1331	-41.9819	
59.3800	-37.6800	0.1631	0.8276	
2.0673	0.5434	0.1512	0.1479	0.3948
	0.1379	-0.1431	-41.9807	
59.4000	-38.1800	0.4291	0.4215	
1.7132	0.5621	0.0890	0.1350	0.3748
	0.1346	-0.1532	-41.9795	
59.4200	-39.7700	0.2264	-0.2730	
1.1904	0.5488	0.0271	0.1216	0.3547
	0.1313	-0.1633	-41.9783	
59.4400	-42.3100	-0.9896	-0.7786	
0.5877	0.5092	-0.0341	0.1078	0.3346
	0.1280	-0.1733	-41.9771	
59.4600	-41.8400	0.3299	-0.7789	-
0.0091	0.4488	-0.0943	0.0937	0.3144
	0.1246	-0.1834	-41.9759	
59.4800	-41.7500	0.8930	-0.5846	-
0.5301	0.3732	-0.1529	0.0793	0.2942
	0.1213	-0.1934	-41.9747	
59.5000	-42.8000	0.2186	-0.5337	-
0.9249	0.2880	-0.2097	0.0648	0.2740
	0.1178	-0.2035	-41.9735	
59.5200	-44.2800	-0.8377	-0.5115	-
1.1719	0.1988	-0.2643	0.0500	0.2538
	0.1144	-0.2135	-41.9722	

59.5400 -44.1900 -0.4944 -0.3263 -
1.2704 0.1101 -0.3162 0.0352 0.2335
0.1109 -0.2236 -41.9710
59.5600 -42.5100 0.8872 -0.0540 -
1.2367 0.0268 -0.3653 0.0203 0.2133
0.1074 -0.2336 -41.9698
59.5800 -43.7200 -0.4197 0.1047 -
1.0927 -0.0472 -0.4111 0.0054 0.1931
0.1039 -0.2436 -41.9686
59.6000 -43.6900 -0.3227 0.1158 -
0.8632 -0.1092 -0.4532 -0.0094 0.1730
0.1004 -0.2537 -41.9674
59.6200 -42.7700 0.4288 0.0434 -
0.5773 -0.1580 -0.4914 -0.0242 0.1529
0.0968 -0.2637 -41.9661
59.6400 -43.3200 -0.2307 -0.0559 -
0.2684 -0.1936 -0.5252 -0.0387 0.1328
0.0932 -0.2737 -41.9649
59.6600 -42.6300 0.2338 -0.0854
0.0258 -0.2162 -0.5543 -0.0531 0.1127
0.0896 -0.2837 -41.9637
59.6800 -42.9100 -0.3848 0.1058
0.2647 -0.2255 -0.5785 -0.0672 0.0928
0.0860 -0.2937 -41.9624
59.7000 -42.1000 0.1092 0.4709
0.4087 -0.2207 -0.5974 -0.0810 0.0729
0.0824 -0.3037 -41.9612
59.7200 -41.6600 0.3494 0.6494
0.4302 -0.2017 -0.6109 -0.0945 0.0531
0.0787 -0.3137 -41.9600
59.7400 -41.9500 0.4507 0.3644
0.3280 -0.1701 -0.6188 -0.1076 0.0333
0.0750 -0.3237 -41.9587
59.7600 -43.7600 -0.8364 -0.0968
0.1441 -0.1288 -0.6214 -0.1203 0.0137
0.0713 -0.3337 -41.9575
59.7800 -42.7700 0.8183 -0.3458 -
0.0598 -0.0818 -0.6187 -0.1325 -0.0059
0.0676 -0.3436 -41.9563
59.8000 -44.0500 -0.4051 -0.4571 -
0.2169 -0.0326 -0.6109 -0.1442 -0.0253
0.0639 -0.3536 -41.9550
59.8200 -44.1500 -0.3456 -0.4240 -
0.2826 0.0148 -0.5983 -0.1554 -0.0446
0.0601 -0.3636 -41.9538
59.8400 -43.6700 -0.3625 -0.1333 -
0.2484 0.0571 -0.5811 -0.1661 -0.0638
0.0564 -0.3735 -41.9526
59.8600 -42.1100 1.0530 0.1071 -
0.1437 0.0917 -0.5598 -0.1762 -0.0829
0.0526 -0.3835 -41.9513

59.8800 -43.8900 -1.0016 0.1656 -
0.0135 0.1171 -0.5345 -0.1857 -0.1018
0.0488 -0.3934 -41.9501
59.9000 -42.7000 -0.0108 0.2401
0.0977 0.1324 -0.5058 -0.1946 -0.1206
0.0450 -0.4033 -41.9488
59.9200 -41.5700 0.9546 0.2673
0.1658 0.1369 -0.4739 -0.2029 -0.1392
0.0412 -0.4132 -41.9476
59.9400 -42.8700 -0.1888 0.1044
0.1847 0.1305 -0.4392 -0.2106 -0.1576
0.0374 -0.4232 -41.9463
59.9600 -43.5400 -0.5696 -0.1121
0.1645 0.1133 -0.4023 -0.2177 -0.1759
0.0336 -0.4331 -41.9451
59.9800 -43.3900 -0.3169 -0.1236
0.1218 0.0866 -0.3635 -0.2241 -0.1940
0.0298 -0.4430 -41.9438
60.0000 -42.3300 0.7726 -0.0365
0.0701 0.0532 -0.3233 -0.2298 -0.2119
0.0259 -0.4528 -41.9426
60.0200 -43.8000 -0.7525 -0.0271
0.0173 0.0168 -0.2821 -0.2348 -0.2297
0.0221 -0.4627 -41.9413
60.0400 -42.5800 0.5244 0.0188 -
0.0312 -0.0189 -0.2403 -0.2391 -0.2472
0.0182 -0.4726 -41.9400
60.0600 -43.1500 0.1269 0.0257 -
0.0729 -0.0504 -0.1982 -0.2428 -0.2645
0.0144 -0.4824 -41.9388
60.0800 -43.6500 -0.2259 -0.0860 -
0.1085 -0.0746 -0.1562 -0.2457 -0.2816
0.0105 -0.4923 -41.9375
60.1000 -43.4000 -0.0245 -0.1030 -
0.1379 -0.0893 -0.1146 -0.2479 -0.2986
0.0066 -0.5021 -41.9363
60.1200 -43.6800 -0.4568 0.1145 -
0.1567 -0.0933 -0.0736 -0.2495 -0.3152
0.0027 -0.5119 -41.9350
60.1400 -42.4500 0.6100 0.2587 -
0.1590 -0.0870 -0.0336 -0.2503 -0.3317
-0.0011 -0.5218 -41.9337
60.1600 -43.3100 0.0236 0.0372 -
0.1349 -0.0719 0.0053 -0.2504 -0.3479
-0.0050 -0.5316 -41.9325
60.1800 -43.9400 -0.5631 -0.2944 -
0.0733 -0.0506 0.0428 -0.2498 -0.3639
-0.0089 -0.5414 -41.9312
60.2000 -42.9600 0.3225 -0.2846
0.0274 -0.0263 0.0789 -0.2486 -0.3797
-0.0128 -0.5511 -41.9299

60.2200 -43.1900 -0.3485 0.0397
0.1475 -0.0022 0.1133 -0.2467 -0.3952
-0.0167 -0.5609 -41.9287
60.2400 -42.0500 0.4819 0.2773
0.2546 0.0181 0.1458 -0.2442 -0.4105
-0.0205 -0.5707 -41.9274
60.2600 -42.8700 -0.3619 0.2610
0.3101 0.0325 0.1763 -0.2411 -0.4255
-0.0244 -0.5804 -41.9261
60.2800 -42.4600 0.2506 0.0670
0.2956 0.0400 0.2047 -0.2373 -0.4402
-0.0283 -0.5901 -41.9248
60.3000 -42.4700 0.3719 -0.1318
0.2138 0.0404 0.2308 -0.2329 -0.4547
-0.0322 -0.5999 -41.9236
60.3200 -44.0300 -0.9788 -0.1710
0.0870 0.0350 0.2544 -0.2280 -0.4689
-0.0360 -0.6096 -41.9223
60.3400 -42.5600 0.5506 -0.0729 -
0.0537 0.0254 0.2754 -0.2224 -0.4829
-0.0399 -0.6193 -41.9210
60.3600 -42.1800 1.0726 0.0176 -
0.1766 0.0139 0.2938 -0.2163 -0.4966
-0.0438 -0.6290 -41.9197
60.3800 -44.3500 -1.2139 0.0484 -
0.2591 0.0028 0.3092 -0.2096 -0.5100
-0.0476 -0.6386 -41.9184
60.4000 -43.8200 -0.4794 0.0607 -
0.2872 -0.0063 0.3218 -0.2024 -0.5231
-0.0514 -0.6483 -41.9172
60.4200 -42.0100 1.2455 0.0033 -
0.2605 -0.0118 0.3313 -0.1947 -0.5360
-0.0553 -0.6579 -41.9159
60.4400 -44.4000 -1.0481 -0.1214 -
0.1927 -0.0130 0.3377 -0.1864 -0.5486
-0.0591 -0.6676 -41.9146
60.4600 -42.6100 0.6376 -0.1087 -
0.1043 -0.0098 0.3411 -0.1775 -0.5609
-0.0629 -0.6772 -41.9133
60.4800 -43.0200 -0.0126 0.0325 -
0.0172 -0.0022 0.3413 -0.1683 -0.5729
-0.0667 -0.6868 -41.9120
60.5000 -43.0100 -0.1588 0.1592
0.0509 0.0095 0.3383 -0.1585 -0.5847
-0.0705 -0.6964 -41.9107
60.5200 -42.6500 0.0565 0.2459
0.0866 0.0244 0.3322 -0.1483 -0.5961
-0.0743 -0.7060 -41.9094
60.5400 -42.6300 0.1362 0.2051
0.0851 0.0411 0.3231 -0.1377 -0.6073
-0.0780 -0.7155 -41.9081

60.5600 -43.1300 -0.1045 -0.0292
0.0588 0.0577 0.3111 -0.1267 -0.6182
-0.0818 -0.7251 -41.9068
60.5800 -43.4400 -0.1871 -0.2713
0.0322 0.0724 0.2961 -0.1154 -0.6288
-0.0855 -0.7346 -41.9055
60.6000 -43.3400 0.0145 -0.2412
0.0273 0.0832 0.2785 -0.1038 -0.6391
-0.0892 -0.7441 -41.9042
60.6200 -43.5700 -0.5823 0.0469
0.0520 0.0884 0.2585 -0.0919 -0.6491
-0.0929 -0.7536 -41.9029
60.6400 -42.0400 0.8785 0.1890
0.0942 0.0870 0.2362 -0.0798 -0.6589
-0.0966 -0.7631 -41.9016
60.6600 -43.8800 -0.8845 0.0645
0.1265 0.0786 0.2121 -0.0675 -0.6683
-0.1002 -0.7726 -41.9003
60.6800 -42.4800 0.6680 0.0507
0.1255 0.0638 0.1863 -0.0551 -0.6775
-0.1039 -0.7820 -41.8990
60.7000 -43.4800 -0.1229 0.0110
0.0872 0.0446 0.1594 -0.0425 -0.6864
-0.1075 -0.7915 -41.8977
60.7200 -43.3800 0.1135 -0.1343
0.0261 0.0230 0.1315 -0.0299 -0.6949
-0.1111 -0.8009 -41.8964
60.7400 -43.8800 -0.3230 -0.0543 -
0.0420 0.0022 0.1033 -0.0172 -0.7033
-0.1146 -0.8103 -41.8951
60.7600 -43.6700 -0.2824 0.1787 -
0.1056 -0.0144 0.0750 -0.0044 -0.7113
-0.1181 -0.8197 -41.8938
60.7800 -42.5200 0.9982 0.1347 -
0.1548 -0.0244 0.0472 0.0084 -0.7190
-0.1217 -0.8290 -41.8925
60.8000 -45.0500 -1.0571 -0.1983 -
0.1741 -0.0275 0.0204 0.0211 -0.7265
-0.1251 -0.8384 -41.8912
60.8200 -43.1400 0.7608 -0.2156 -
0.1472 -0.0247 -0.0051 0.0337 -0.7336
-0.1286 -0.8477 -41.8899
60.8400 -43.6000 0.1698 -0.1015 -
0.0686 -0.0184 -0.0289 0.0463 -0.7405
-0.1320 -0.8570 -41.8885
60.8600 -44.1200 -0.2759 -0.1979
0.0495 -0.0120 -0.0506 0.0588 -0.7471
-0.1354 -0.8663 -41.8872
60.8800 -43.5500 0.0150 -0.2452
0.1697 -0.0092 -0.0699 0.0711 -0.7535
-0.1388 -0.8756 -41.8859

60.9000 -43.8400 -0.5551 0.1450
0.2409 -0.0123 -0.0865 0.0832 -0.7595
-0.1421 -0.8849 -41.8846
60.9200 -42.2100 0.6954 0.6780
0.2335 -0.0232 -0.1002 0.0951 -0.7653
-0.1454 -0.8941 -41.8833
60.9400 -43.1200 -0.2105 0.7175
0.1492 -0.0415 -0.1108 0.1068 -0.7708
-0.1486 -0.9033 -41.8820
60.9600 -44.3700 -0.7731 0.2361
0.0194 -0.0646 -0.1180 0.1182 -0.7760
-0.1519 -0.9125 -41.8806
60.9800 -44.0300 0.3992 -0.5012 -
0.1102 -0.0891 -0.1218 0.1294 -0.7810
-0.1550 -0.9217 -41.8793
61.0000 -43.7600 1.1649 -0.9320 -
0.1952 -0.1116 -0.1223 0.1402 -0.7856
-0.1582 -0.9309 -41.8780
61.0200 -46.0100 -1.4870 -0.5112 -
0.2117 -0.1286 -0.1194 0.1508 -0.7900
-0.1613 -0.9400 -41.8766
61.0400 -42.1000 1.4210 0.5176 -
0.1750 -0.1378 -0.1136 0.1610 -0.7942
-0.1644 -0.9492 -41.8753
61.0600 -42.7500 0.2559 0.8995 -
0.1128 -0.1379 -0.1050 0.1708 -0.7980
-0.1674 -0.9583 -41.8740
61.0800 -44.6300 -1.0047 0.3665 -
0.0497 -0.1284 -0.0941 0.1802 -0.8016
-0.1704 -0.9674 -41.8727
61.1000 -43.8200 0.6571 -0.5317
0.0122 -0.1098 -0.0811 0.1893 -0.8049
-0.1734 -0.9764 -41.8713
61.1200 -45.1900 -0.8170 -0.6135
0.0759 -0.0831 -0.0666 0.1979 -0.8080
-0.1763 -0.9855 -41.8700
61.1400 -42.9600 0.5621 0.0069
0.1354 -0.0490 -0.0509 0.2061 -0.8108
-0.1791 -0.9945 -41.8686
61.1600 -42.8300 0.3943 0.3644
0.1737 -0.0086 -0.0345 0.2139 -0.8133
-0.1819 -1.0035 -41.8673
61.1800 -43.7800 -0.5257 0.1529
0.1689 0.0366 -0.0176 0.2212 -0.8156
-0.1847 -1.0125 -41.8660
61.2000 -43.1600 0.4913 -0.1201
0.0968 0.0844 -0.0009 0.2280 -0.8176
-0.1874 -1.0215 -41.8646
61.2200 -44.3900 -0.7447 0.0331 -
0.0461 0.1324 0.0153 0.2343 -0.8194
-0.1901 -1.0304 -41.8633

61.2400 -42.6700 0.7422 0.2429 -
0.2145 0.1769 0.0305 0.2401 -0.8209
-0.1927 -1.0393 -41.8619
61.2600 -44.2300 -0.5350 0.0458 -
0.3385 0.2137 0.0445 0.2454 -0.8221
-0.1953 -1.0482 -41.8606
61.2800 -43.9300 0.0085 -0.2674 -
0.3644 0.2392 0.0569 0.2500 -0.8231
-0.1978 -1.0571 -41.8593
61.3000 -43.7500 0.3896 -0.3881 -
0.2655 0.2511 0.0676 0.2542 -0.8238
-0.2003 -1.0659 -41.8579
61.3200 -43.3300 0.2620 -0.2527 -
0.0579 0.2486 0.0764 0.2577 -0.8242
-0.2028 -1.0748 -41.8566
61.3400 -43.9400 -0.8428 0.1355
0.2061 0.2318 0.0833 0.2606 -0.8245
-0.2051 -1.0836 -41.8552
61.3600 -41.6800 0.8742 0.4800
0.4548 0.2018 0.0881 0.2629 -0.8244
-0.2075 -1.0924 -41.8539
61.3800 -41.8700 0.5254 0.3863
0.6202 0.1595 0.0909 0.2645 -0.8241
-0.2097 -1.1011 -41.8525
61.4000 -42.9600 -0.0206 -0.0485
0.6618 0.1065 0.0918 0.2655 -0.8236
-0.2120 -1.1099 -41.8512
61.4200 -44.3400 -1.0483 -0.2946
0.5708 0.0454 0.0911 0.2659 -0.8228
-0.2141 -1.1186 -41.8498
61.4400 -43.5200 -0.1434 -0.0779
0.3565 -0.0199 0.0889 0.2656 -0.8217
-0.2162 -1.1273 -41.8484
61.4600 -42.6300 0.8525 0.2579
0.0607 -0.0848 0.0853 0.2646 -0.8205
-0.2183 -1.1359 -41.8471
61.4800 -43.5900 0.1490 0.3046 -
0.2574 -0.1448 0.0807 0.2630 -0.8189
-0.2203 -1.1446 -41.8457
61.5000 -44.9600 -0.5942 0.0737 -
0.5403 -0.1952 0.0752 0.2606 -0.8172
-0.2223 -1.1532 -41.8444
61.5200 -44.8400 0.1433 -0.2286 -
0.7374 -0.2315 0.0690 0.2577 -0.8151
-0.2242 -1.1618 -41.8430
61.5400 -44.9800 0.1115 -0.3991 -
0.8028 -0.2499 0.0623 0.2541 -0.8129
-0.2260 -1.1703 -41.8416
61.5600 -45.3900 -0.3314 -0.3562 -
0.7108 -0.2484 0.0554 0.2498 -0.8104
-0.2278 -1.1789 -41.8403

61.5800	-44.4000	0.2955	-0.1033	-
0.4807	-0.2289	0.0484	0.2450	-0.8076
	-0.2295	-1.1874	-41.8389	
61.6000	-44.2600	-0.4636	0.2492	-
0.1661	-0.1951	0.0415	0.2395	-0.8047
	-0.2312	-1.1959	-41.8375	
61.6200	-42.8000	0.5819	0.4085	
0.1729	-0.1511	0.0349	0.2335	-0.8014
	-0.2328	-1.2043	-41.8362	
61.6400	-43.3100	-0.1611	0.1561	
0.4756	-0.1009	0.0286	0.2268	-0.7980
	-0.2344	-1.2128	-41.8348	
61.6600	-43.6900	-0.2875	-0.1612	
0.6788	-0.0487	0.0228	0.2196	-0.7942
	-0.2359	-1.2212	-41.8334	
61.6800	-43.0900	0.0712	-0.1112	
0.7373	0.0016	0.0175	0.2119	-0.7903
	-0.2374	-1.2296	-41.8321	
61.7000	-42.9000	0.0660	0.2354	
0.6489	0.0466	0.0128	0.2036	-0.7861
	-0.2388	-1.2379	-41.8307	
61.7200	-43.0700	-0.1942	0.4962	
0.4373	0.0843	0.0088	0.1947	-0.7816
	-0.2402	-1.2463	-41.8293	
61.7400	-42.7800	0.4791	0.3418	
0.1436	0.1132	0.0056	0.1854	-0.7769
	-0.2415	-1.2546	-41.8279	
61.7600	-43.5800	0.5268	-0.1400	-
0.1643	0.1318	0.0032	0.1755	-0.7720
	-0.2428	-1.2629	-41.8266	
61.7800	-45.7600	-1.0698	-0.5121	-
0.4091	0.1389	0.0016	0.1652	-0.7667
	-0.2440	-1.2711	-41.8252	
61.8000	-43.8600	0.9583	-0.4640	-
0.5226	0.1333	0.0008	0.1543	-0.7613
	-0.2451	-1.2794	-41.8238	
61.8200	-44.7000	-0.0792	-0.3043	-
0.4824	0.1149	0.0008	0.1430	-0.7555
	-0.2462	-1.2876	-41.8224	
61.8400	-44.7200	-0.5051	-0.1495	-
0.3167	0.0853	0.0015	0.1311	-0.7496
	-0.2473	-1.2957	-41.8210	
61.8600	-44.3500	-0.3515	0.1510	-
0.0897	0.0481	0.0031	0.1188	-0.7433
	-0.2483	-1.3039	-41.8196	
61.8800	-42.9300	0.5201	0.4610	
0.1316	0.0075	0.0053	0.1060	-0.7368
	-0.2492	-1.3120	-41.8183	
61.9000	-42.9100	0.3457	0.5412	
0.2898	-0.0326	0.0082	0.0928	-0.7301
	-0.2501	-1.3201	-41.8169	

61.9200 -43.5300 -0.1024 0.3729
0.3483 -0.0684 0.0116 0.0791 -0.7230
-0.2510 -1.3281 -41.8155
61.9400 -44.6400 -1.1381 0.2077
0.3015 -0.0972 0.0154 0.0650 -0.7158
-0.2518 -1.3362 -41.8141
61.9600 -43.0500 0.8678 0.1244
0.1704 -0.1176 0.0197 0.0505 -0.7082
-0.2525 -1.3442 -41.8127
61.9800 -43.3600 0.9930 -0.0497 -
0.0024 -0.1287 0.0241 0.0355 -0.7004
-0.2532 -1.3522 -41.8113
62.0000 -44.6600 -0.0075 -0.3689 -
0.1670 -0.1300 0.0286 0.0202 -0.6924
-0.2539 -1.3601 -41.8099
62.0200 -46.3400 -1.3310 -0.5084 -
0.2778 -0.1212 0.0332 0.0044 -0.6840
-0.2545 -1.3680 -41.8085
62.0400 -43.7700 0.9394 -0.3092 -
0.3011 -0.1020 0.0376 -0.0118 -0.6754
-0.2550 -1.3759 -41.8071
62.0600 -44.0200 0.4848 -0.1394 -
0.2301 -0.0734 0.0417 -0.0284 -0.6666
-0.2555 -1.3838 -41.8057
62.0800 -45.0400 -0.6974 -0.1759 -
0.0839 -0.0377 0.0454 -0.0454 -0.6575
-0.2560 -1.3916 -41.8043
62.1000 -44.4200 0.0153 -0.2270
0.0899 0.0020 0.0486 -0.0627 -0.6481
-0.2564 -1.3994 -41.8029
62.1200 -43.6500 0.2527 -0.0356
0.2327 0.0425 0.0511 -0.0804 -0.6384
-0.2568 -1.4072 -41.8015
62.1400 -43.8300 -0.4445 0.3738
0.3000 0.0810 0.0528 -0.0985 -0.6285
-0.2571 -1.4149 -41.8001
62.1600 -42.5800 0.5267 0.6374
0.2721 0.1149 0.0535 -0.1168 -0.6184
-0.2574 -1.4226 -41.7987
62.1800 -42.8100 0.6443 0.3806
0.1554 0.1420 0.0533 -0.1354 -0.6079
-0.2576 -1.4303 -41.7973
62.2000 -45.2200 -0.8487 -0.2466 -
0.0047 0.1613 0.0521 -0.1543 -0.5972
-0.2578 -1.4380 -41.7959
62.2200 -44.9700 -0.2051 -0.5912 -
0.1466 0.1717 0.0500 -0.1734 -0.5863
-0.2580 -1.4456 -41.7945
62.2400 -44.9100 -0.3699 -0.3324 -
0.2169 0.1723 0.0468 -0.1927 -0.5751
-0.2581 -1.4532 -41.7931

62.2600 -43.2300 0.8310 0.0616 -
0.1973 0.1631 0.0427 -0.2121 -0.5636
-0.2582 -1.4608 -41.7917
62.2800 -43.8200 0.1873 0.0971 -
0.0903 0.1448 0.0377 -0.2317 -0.5519
-0.2582 -1.4683 -41.7903
62.3000 -44.3800 -0.2690 -0.1192
0.0696 0.1197 0.0320 -0.2513 -0.5399
-0.2582 -1.4758 -41.7889
62.3200 -44.3800 -0.1560 -0.2065
0.2206 0.0902 0.0256 -0.2709 -0.5276
-0.2581 -1.4833 -41.7874
62.3400 -44.0200 -0.0647 0.0251
0.3093 0.0585 0.0187 -0.2905 -0.5151
-0.2580 -1.4907 -41.7860
62.3600 -43.1600 0.4421 0.3725
0.3064 0.0269 0.0116 -0.3101 -0.5024
-0.2579 -1.4981 -41.7846
62.3800 -44.0600 -0.3593 0.5147
0.2025 -0.0031 0.0045 -0.3295 -0.4894
-0.2577 -1.5055 -41.7832
62.4000 -44.2400 -0.0847 0.2193
0.0182 -0.0299 -0.0023 -0.3488 -0.4762
-0.2575 -1.5129 -41.7818
62.4200 -44.2500 0.7758 -0.3654 -
0.1843 -0.0519 -0.0086 -0.3679 -0.4627
-0.2573 -1.5202 -41.7804
62.4400 -46.4200 -0.9944 -0.5826 -
0.3343 -0.0677 -0.0140 -0.3867 -0.4490
-0.2570 -1.5275 -41.7789
62.4600 -43.9800 0.8796 -0.0097 -
0.3812 -0.0765 -0.0183 -0.4052 -0.4350
-0.2567 -1.5347 -41.7775
62.4800 -44.8600 -0.5241 0.4947 -
0.3276 -0.0781 -0.0213 -0.4234 -0.4208
-0.2564 -1.5419 -41.7761
62.5000 -43.9600 0.3951 0.3055 -
0.1964 -0.0733 -0.0229 -0.4411 -0.4064
-0.2560 -1.5491 -41.7747
62.5200 -44.5500 0.1840 -0.1627 -
0.0223 -0.0638 -0.0230 -0.4583 -0.3917
-0.2556 -1.5563 -41.7732
62.5400 -45.4400 -0.6241 -0.3940
0.1517 -0.0516 -0.0217 -0.4749 -0.3768
-0.2551 -1.5634 -41.7718
62.5600 -43.9600 0.6383 -0.2679
0.2902 -0.0386 -0.0190 -0.4909 -0.3618
-0.2546 -1.5705 -41.7704
62.5800 -44.4200 -0.0685 -0.1171
0.3731 -0.0269 -0.0151 -0.5062 -0.3465
-0.2541 -1.5776 -41.7690

62.6000	-44.3000	-0.3966	0.0824	
0.3860	-0.0177	-0.0101	-0.5207	-0.3310
	-0.2536	-1.5846	-41.7675	
62.6200	-44.4300	-0.7249	0.5182	
0.3160	-0.0117	-0.0041	-0.5344	-0.3153
	-0.2530	-1.5916	-41.7661	
62.6400	-42.5700	0.8485	0.7679	
0.1702	-0.0086	0.0027	-0.5472	-0.2994
	-0.2524	-1.5986	-41.7647	
62.6600	-43.4200	0.7177	0.4010	-
0.0248	-0.0070	0.0101	-0.5589	-0.2833
	-0.2518	-1.6055	-41.7632	
62.6800	-45.6700	-0.6996	-0.3658	-
0.2170	-0.0051	0.0180	-0.5695	-0.2670
	-0.2511	-1.6124	-41.7618	
62.7000	-46.1100	-0.5849	-0.8877	-
0.3458	-0.0012	0.0260	-0.5789	-0.2506
	-0.2505	-1.6193	-41.7604	
62.7200	-45.3000	0.1775	-0.8047	-
0.3624	0.0065	0.0339	-0.5871	-0.2340
	-0.2497	-1.6261	-41.7589	
62.7400	-45.1000	-0.0917	-0.3198	-
0.2722	0.0178	0.0414	-0.5940	-0.2173
	-0.2490	-1.6329	-41.7575	
62.7600	-43.7500	0.4823	0.1627	-
0.1219	0.0323	0.0481	-0.5994	-0.2004
	-0.2483	-1.6397	-41.7560	
62.7800	-44.2600	-0.5043	0.5592	
0.0262	0.0495	0.0538	-0.6034	-0.1834
	-0.2475	-1.6464	-41.7546	
62.8000	-42.9800	0.3565	0.8595	
0.1118	0.0686	0.0580	-0.6058	-0.1663
	-0.2467	-1.6531	-41.7532	
62.8200	-43.1400	0.2316	0.8137	
0.1131	0.0879	0.0606	-0.6067	-0.1491
	-0.2458	-1.6597	-41.7517	
62.8400	-43.7300	0.1992	0.2627	
0.0513	0.1049	0.0614	-0.6058	-0.1317
	-0.2450	-1.6664	-41.7503	
62.8600	-44.7100	0.0417	-0.4639	-
0.0272	0.1166	0.0602	-0.6033	-0.1143
	-0.2441	-1.6730	-41.7488	
62.8800	-45.9300	-0.8203	-0.7821	-
0.0733	0.1201	0.0569	-0.5990	-0.0967
	-0.2432	-1.6795	-41.7474	
62.9000	-44.2200	0.6346	-0.5207	-
0.0556	0.1132	0.0515	-0.5929	-0.0791
	-0.2423	-1.6860	-41.7459	
62.9200	-44.1700	0.1298	-0.1600	
0.0217	0.0948	0.0438	-0.5850	-0.0615
	-0.2414	-1.6925	-41.7445	

62.9400 -44.3000 -0.2984 0.0482
0.1335 0.0651 0.0339 -0.5753 -0.0438
-0.2404 -1.6990 -41.7430
62.9600 -43.9300 -0.0816 0.2147
0.2342 0.0249 0.0219 -0.5638 -0.0260
-0.2395 -1.7054 -41.7416
62.9800 -43.5700 0.0216 0.4248
0.2807 -0.0248 0.0077 -0.5504 -0.0083
-0.2385 -1.7118 -41.7401
63.0000 -43.3100 0.2564 0.4902
0.2543 -0.0819 -0.0086 -0.5352 0.0095
-0.2375 -1.7181 -41.7387
63.0200 -44.1100 -0.1644 0.2335
0.1609 -0.1424 -0.0270 -0.5183 0.0273
-0.2365 -1.7244 -41.7372
63.0400 -44.4000 0.2429 -0.1922
0.0333 -0.2010 -0.0476 -0.4995 0.0451
-0.2355 -1.7307 -41.7357
63.0600 -45.0400 0.0026 -0.4842 -
0.0881 -0.2528 -0.0702 -0.4791 0.0629
-0.2344 -1.7370 -41.7343
63.0800 -45.3600 -0.3928 -0.4159 -
0.1707 -0.2926 -0.0948 -0.4569 0.0806
-0.2334 -1.7432 -41.7328
63.1000 -44.2700 0.4491 0.0068 -
0.1998 -0.3159 -0.1213 -0.4332 0.0982
-0.2323 -1.7493 -41.7314
63.1200 -44.5200 -0.3190 0.3993 -
0.1862 -0.3195 -0.1495 -0.4079 0.1159
-0.2312 -1.7555 -41.7299
63.1400 -43.8900 0.2605 0.4287 -
0.1489 -0.3016 -0.1791 -0.3812 0.1334
-0.2301 -1.7616 -41.7284
63.1600 -44.1300 0.1873 0.0523 -
0.1013 -0.2616 -0.2095 -0.3531 0.1508
-0.2291 -1.7676 -41.7270
63.1800 -45.1700 -0.4861 -0.3960 -
0.0542 -0.1998 -0.2405 -0.3237 0.1682
-0.2279 -1.7737 -41.7255
63.2000 -44.3500 0.0755 -0.3592 -
0.0145 -0.1175 -0.2714 -0.2932 0.1854
-0.2268 -1.7796 -41.7241
63.2200 -44.6300 -0.7329 0.2679
0.0177 -0.0186 -0.3019 -0.2616 0.2025
-0.2257 -1.7856 -41.7226
63.2400 -42.1900 1.2213 0.5478
0.0406 0.0909 -0.3313 -0.2290 0.2195
-0.2246 -1.7915 -41.7211
63.2600 -44.9800 -1.2750 0.0302
0.0544 0.2042 -0.3591 -0.1956 0.2363
-0.2234 -1.7974 -41.7197

63.2800 -43.5100 0.6460 -0.4444
0.0744 0.3150 -0.3846 -0.1614 0.2529
-0.2223 -1.8032 -41.7182
63.3000 -43.3900 0.7485 -0.4537
0.1153 0.4169 -0.4074 -0.1267 0.2694
-0.2212 -1.8090 -41.7167
63.3200 -44.3100 -0.8503 -0.0960
0.1734 0.5042 -0.4268 -0.0914 0.2856
-0.2200 -1.8148 -41.7152
63.3400 -42.2400 0.6348 0.2550
0.2271 0.5720 -0.4424 -0.0557 0.3017
-0.2188 -1.8205 -41.7138
63.3600 -42.8900 -0.2259 0.3683
0.2446 0.6164 -0.4536 -0.0196 0.3175
-0.2177 -1.8262 -41.7123
63.3800 -42.8200 0.0819 0.2249
0.2010 0.6346 -0.4601 0.0166 0.3331
-0.2165 -1.8318 -41.7108
63.4000 -43.2300 -0.2654 0.0473
0.0928 0.6250 -0.4615 0.0530 0.3484
-0.2154 -1.8375 -41.7093
63.4200 -43.2700 -0.0673 0.0287 -
0.0651 0.5872 -0.4575 0.0893 0.3635
-0.2142 -1.8430 -41.7079
63.4400 -43.3300 -0.0077 0.0808 -
0.2321 0.5218 -0.4480 0.1256 0.3783
-0.2130 -1.8486 -41.7064
63.4600 -43.0200 0.5331 -0.0431 -
0.3574 0.4302 -0.4330 0.1617 0.3928
-0.2119 -1.8541 -41.7049
63.4800 -44.5100 -0.6061 -0.2758 -
0.3931 0.3150 -0.4124 0.1974 0.4070
-0.2107 -1.8595 -41.7034
63.5000 -43.6600 0.2739 -0.3062 -
0.3108 0.1798 -0.3865 0.2327 0.4208
-0.2096 -1.8649 -41.7019
63.5200 -43.6400 0.0268 -0.0943 -
0.1219 0.0297 -0.3552 0.2675 0.4344
-0.2084 -1.8703 -41.7004
63.5400 -43.4900 -0.3809 0.1637
0.1253 -0.1297 -0.3188 0.3017 0.4476
-0.2073 -1.8757 -41.6990
63.5600 -42.4200 0.5664 0.2185
0.3665 -0.2921 -0.2773 0.3351 0.4605
-0.2061 -1.8810 -41.6975
63.5800 -43.2300 -0.1992 0.0487
0.5463 -0.4511 -0.2309 0.3676 0.4729
-0.2050 -1.8862 -41.6960
63.6000 -43.4200 -0.3113 -0.0855
0.6319 -0.6006 -0.1796 0.3992 0.4850
-0.2038 -1.8915 -41.6945

63.6200	-43.0200	0.2860	-0.0316	
0.6090	-0.7350	-0.1238	0.4298	0.4967
	-0.2027	-1.8966	-41.6930	
63.6400	-43.1200	-0.0678	0.0754	
0.4835	-0.8502	-0.0636	0.4591	0.5080
	-0.2016	-1.9018	-41.6915	
63.6600	-43.0600	0.2396	0.1031	
0.2749	-0.9420	0.0006	0.4872	0.5189
	-0.2005	-1.9069	-41.6900	
63.6800	-44.0600	-0.4320	0.0801	
0.0073	-1.0051	0.0685	0.5139	0.5293
	-0.1994	-1.9120	-41.6885	
63.7000	-43.5600	0.5278	-0.0439	-
0.2881	-1.0335	0.1394	0.5391	0.5393
	-0.1983	-1.9170	-41.6870	
63.7200	-44.9100	-0.4626	-0.2104	-
0.5755	-1.0223	0.2129	0.5627	0.5488
	-0.1972	-1.9220	-41.6855	
63.7400	-44.0900	0.1936	-0.1554	-
0.8109	-0.9674	0.2884	0.5846	0.5578
	-0.1961	-1.9269	-41.6841	
63.7600	-44.6200	-0.5006	0.0799	-
0.9509	-0.8665	0.3648	0.6048	0.5663
	-0.1951	-1.9318	-41.6826	
63.7800	-43.1000	0.6339	0.1270	-
0.9623	-0.7193	0.4412	0.6231	0.5744
	-0.1940	-1.9367	-41.6811	
63.8000	-43.5300	0.1307	-0.1878	-
0.8309	-0.5301	0.5162	0.6394	0.5819
	-0.1930	-1.9415	-41.6796	
63.8200	-43.9200	-0.4435	-0.5250	-
0.5734	-0.3075	0.5886	0.6536	0.5889
	-0.1920	-1.9463	-41.6781	
63.8400	-43.1600	-0.6347	-0.3371	-
0.2271	-0.0629	0.6569	0.6657	0.5953
	-0.1910	-1.9510	-41.6766	
63.8600	-40.9400	0.1607	0.3106	
0.1457	0.1914	0.7198	0.6754	0.6012
	-0.1900	-1.9557	-41.6751	
63.8800	-39.4300	0.6472	0.7670	
0.4682	0.4432	0.7761	0.6828	0.6065
	-0.1890	-1.9604	-41.6736	
63.9000	-39.5000	0.3168	0.5852	
0.6765	0.6801	0.8244	0.6878	0.6113
	-0.1880	-1.9650	-41.6720	
63.9200	-40.6500	-0.6815	0.0304	
0.7663	0.8895	0.8633	0.6901	0.6155
	-0.1871	-1.9696	-41.6705	
63.9400	-40.0900	-0.0067	-0.3548	
0.7731	1.0591	0.8916	0.6898	0.6191
	-0.1862	-1.9741	-41.6690	

63.9600 -39.8500 0.2477 -0.4481
0.7573 1.1774 0.9078 0.6867 0.6221
-0.1853 -1.9786 -41.6675
63.9800 -39.7800 0.2429 -0.4368
0.7718 1.2357 0.9106 0.6809 0.6246
-0.1844 -1.9830 -41.6660
64.0000 -40.1300 -0.2065 -0.2881
0.8184 1.2326 0.8991 0.6723 0.6265
-0.1835 -1.9875 -41.6645
64.0200 -39.9900 -0.4816 0.1233
0.8541 1.1731 0.8734 0.6610 0.6278
-0.1826 -1.9918 -41.6630
64.0400 -38.5400 0.6717 0.5250
0.8234 1.0653 0.8344 0.6472 0.6286
-0.1818 -1.9961 -41.6615
64.0600 -39.9500 -0.1273 0.4574
0.6845 0.9188 0.7832 0.6310 0.6289
-0.1810 -2.0004 -41.6600
64.0800 -40.8300 -0.1503 0.0492
0.4356 0.7442 0.7212 0.6125 0.6286
-0.1802 -2.0047 -41.6585
64.1000 -42.2300 -0.5236 -0.2473
0.1069 0.5518 0.6496 0.5919 0.6279
-0.1794 -2.0089 -41.6569
64.1200 -41.5600 0.6323 -0.2062 -
0.2577 0.3515 0.5696 0.5694 0.6266
-0.1787 -2.0130 -41.6554
64.1400 -43.4000 -0.8880 0.1348 -
0.6015 0.1533 0.4825 0.5450 0.6249
-0.1780 -2.0171 -41.6539
64.1600 -42.7300 0.2228 0.3205 -
0.8547 -0.0336 0.3895 0.5190 0.6227
-0.1773 -2.0212 -41.6524
64.1800 -42.6500 0.9682 0.0190 -
0.9616 -0.2007 0.2919 0.4915 0.6200
-0.1766 -2.0252 -41.6509
64.2000 -45.3600 -1.0856 -0.5646 -
0.9081 -0.3425 0.1910 0.4626 0.6169
-0.1759 -2.0292 -41.6494
64.2200 -44.2300 0.2565 -0.5984 -
0.7084 -0.4570 0.0880 0.4325 0.6133
-0.1752 -2.0331 -41.6478
64.2400 -43.9600 -0.2558 0.0386 -
0.4088 -0.5449 -0.0159 0.4013 0.6093
-0.1746 -2.0370 -41.6463
64.2600 -42.7700 0.1150 0.6629 -
0.0834 -0.6077 -0.1192 0.3692 0.6049
-0.1740 -2.0409 -41.6448
64.2800 -42.4500 0.3781 0.7340
0.1942 -0.6471 -0.2206 0.3364 0.6002
-0.1734 -2.0447 -41.6433

64.3000	-42.4800	0.9551	0.1033	
0.3845	-0.6659	-0.3186	0.3029	0.5950
	-0.1728	-2.0485	-41.6417	
64.3200	-45.4200	-1.1554	-0.6264	
0.4933	-0.6682	-0.4120	0.2690	0.5894
	-0.1723	-2.0522	-41.6402	
64.3400	-43.2700	1.0311	-0.6497	
0.5355	-0.6590	-0.4991	0.2347	0.5835
	-0.1718	-2.0559	-41.6387	
64.3600	-43.9000	-0.0094	-0.1848	
0.5257	-0.6426	-0.5787	0.2003	0.5773
	-0.1712	-2.0595	-41.6372	
64.3800	-44.2400	-0.6520	0.2569	
0.4773	-0.6231	-0.6494	0.1659	0.5707
	-0.1708	-2.0631	-41.6356	
64.4000	-43.9800	-0.3297	0.5013	
0.3867	-0.6026	-0.7100	0.1316	0.5638
	-0.1703	-2.0666	-41.6341	
64.4200	-43.2200	0.6049	0.4832	
0.2522	-0.5826	-0.7598	0.0975	0.5565
	-0.1698	-2.0702	-41.6326	
64.4400	-44.0300	0.3129	0.1203	
0.0892	-0.5633	-0.7981	0.0639	0.5490
	-0.1694	-2.0736	-41.6310	
64.4600	-45.7700	-0.8037	-0.2926	-
0.0722	-0.5440	-0.8249	0.0309	0.5412
	-0.1690	-2.0770	-41.6295	
64.4800	-44.9100	0.3619	-0.3908	-
0.1976	-0.5232	-0.8403	-0.0015	0.5331
	-0.1686	-2.0804	-41.6280	
64.5000	-44.7800	0.4439	-0.2826	-
0.2619	-0.4988	-0.8445	-0.0331	0.5248
	-0.1683	-2.0837	-41.6264	
64.5200	-45.8500	-0.6400	-0.1474	-
0.2590	-0.4690	-0.8382	-0.0638	0.5162
	-0.1679	-2.0870	-41.6249	
64.5400	-44.8200	0.1926	-0.0390	-
0.2047	-0.4319	-0.8219	-0.0935	0.5074
	-0.1676	-2.0902	-41.6234	
64.5600	-44.1200	0.5852	0.0902	-
0.1343	-0.3860	-0.7965	-0.1223	0.4983
	-0.1673	-2.0934	-41.6218	
64.5800	-44.7300	-0.2977	0.2664	-
0.0828	-0.3305	-0.7628	-0.1500	0.4890
	-0.1670	-2.0966	-41.6203	
64.6000	-45.2800	-1.0602	0.4955	-
0.0735	-0.2654	-0.7217	-0.1765	0.4796
	-0.1668	-2.0997	-41.6188	
64.6200	-42.8400	1.1862	0.5390	-
0.1009	-0.1922	-0.6741	-0.2020	0.4699
	-0.1665	-2.1028	-41.6172	

64.6400 -43.6100 0.8298 0.0982 -
0.1392 -0.1135 -0.6208 -0.2263 0.4601
-0.1663 -2.1058 -41.6157
64.6600 -46.2400 -1.3168 -0.5334 -
0.1558 -0.0334 -0.5628 -0.2494 0.4501
-0.1661 -2.1088 -41.6141
64.6800 -44.6300 0.4798 -0.7997 -
0.1208 0.0438 -0.5007 -0.2714 0.4400
-0.1660 -2.1117 -41.6126
64.7000 -43.8200 0.7404 -0.5300 -
0.0204 0.1139 -0.4353 -0.2921 0.4297
-0.1658 -2.1146 -41.6110
64.7200 -44.6200 -0.7518 0.1037
0.1184 0.1733 -0.3675 -0.3117 0.4194
-0.1657 -2.1174 -41.6095
64.7400 -43.1400 -0.0469 0.6358
0.2434 0.2191 -0.2980 -0.3300 0.4089
-0.1656 -2.1202 -41.6079
64.7600 -42.1200 0.6473 0.7355
0.3019 0.2485 -0.2274 -0.3471 0.3983
-0.1655 -2.1229 -41.6064
64.7800 -42.8200 0.3434 0.3128
0.2684 0.2595 -0.1566 -0.3630 0.3876
-0.1655 -2.1256 -41.6049
64.8000 -44.6000 -0.8073 -0.2501
0.1614 0.2529 -0.0860 -0.3777 0.3768
-0.1654 -2.1283 -41.6033
64.8200 -44.0100 0.1371 -0.4851
0.0155 0.2317 -0.0163 -0.3913 0.3660
-0.1654 -2.1309 -41.6018
64.8400 -43.5400 0.6568 -0.3734 -
0.1264 0.1992 0.0520 -0.4036 0.3551
-0.1655 -2.1335 -41.6002
64.8600 -44.7300 -0.6903 -0.1449 -
0.2172 0.1593 0.1183 -0.4147 0.3442
-0.1655 -2.1360 -41.5986
64.8800 -43.6200 0.3368 0.1099 -
0.2292 0.1157 0.1820 -0.4247 0.3333
-0.1656 -2.1385 -41.5971
64.9000 -43.5900 0.0801 0.1736 -
0.1558 0.0722 0.2426 -0.4335 0.3224
-0.1657 -2.1409 -41.5955
64.9200 -43.1900 0.7915 -0.2322 -
0.0192 0.0328 0.2995 -0.4411 0.3114
-0.1658 -2.1433 -41.5940
64.9400 -45.0500 -1.1865 -0.4119
0.1261 0.0007 0.3522 -0.4476 0.3005
-0.1659 -2.1457 -41.5924
64.9600 -42.2400 1.0048 0.1698
0.2259 -0.0208 0.4001 -0.4529 0.2896
-0.1661 -2.1480 -41.5909

64.9800 -42.4100 0.4156 0.6179
0.2326 -0.0292 0.4428 -0.4571 0.2787
-0.1663 -2.1502 -41.5893
65.0000 -42.8900 0.3070 0.3030
0.1235 -0.0230 0.4799 -0.4602 0.2679
-0.1665 -2.1525 -41.5878
65.0200 -44.8700 -0.9443 -0.2232 -
0.0515 -0.0025 0.5110 -0.4622 0.2571
-0.1667 -2.1546 -41.5862
65.0400 -43.7100 0.4554 -0.4038 -
0.2226 0.0309 0.5357 -0.4631 0.2464
-0.1670 -2.1568 -41.5846
65.0600 -43.7200 0.3350 -0.2850 -
0.3375 0.0754 0.5540 -0.4630 0.2358
-0.1673 -2.1588 -41.5831
65.0800 -44.4400 -0.5132 -0.0917 -
0.3691 0.1277 0.5658 -0.4618 0.2253
-0.1676 -2.1609 -41.5815
65.1000 -43.2600 0.4021 0.0382 -
0.3159 0.1836 0.5711 -0.4597 0.2149
-0.1679 -2.1629 -41.5800
65.1200 -43.4100 0.1357 0.0872 -
0.1931 0.2380 0.5701 -0.4567 0.2047
-0.1683 -2.1648 -41.5784
65.1400 -43.8200 -0.5789 0.1255 -
0.0279 0.2857 0.5631 -0.4528 0.1945
-0.1687 -2.1667 -41.5768
65.1600 -42.5500 0.3398 0.1650
0.1484 0.3216 0.5502 -0.4480 0.1845
-0.1691 -2.1686 -41.5753
65.1800 -42.4000 0.5167 0.0624
0.3079 0.3403 0.5317 -0.4425 0.1747
-0.1695 -2.1704 -41.5737
65.2000 -43.9000 -0.8653 -0.0960
0.4127 0.3373 0.5082 -0.4364 0.1650
-0.1700 -2.1722 -41.5721
65.2200 -43.1000 -0.1284 0.0394
0.4281 0.3102 0.4801 -0.4296 0.1555
-0.1705 -2.1739 -41.5706
65.2400 -42.7900 0.0160 0.4034
0.3402 0.2598 0.4481 -0.4223 0.1461
-0.1710 -2.1756 -41.5690
65.2600 -41.9300 1.1203 0.4025
0.1592 0.1903 0.4130 -0.4144 0.1370
-0.1715 -2.1772 -41.5674
65.2800 -44.3800 -0.4021 -0.0654 -
0.0720 0.1079 0.3754 -0.4062 0.1281
-0.1721 -2.1788 -41.5658
65.3000 -45.5000 -0.9567 -0.4377 -
0.2816 0.0195 0.3360 -0.3975 0.1194
-0.1727 -2.1804 -41.5643

65.3200 -43.8800 0.9210 -0.3107 -
0.3990 -0.0680 0.2953 -0.3885 0.1109
-0.1733 -2.1819 -41.5627
65.3400 -45.0000 -0.5157 0.0192 -
0.3937 -0.1474 0.2541 -0.3792 0.1026
-0.1739 -2.1833 -41.5611
65.3600 -44.2400 0.1609 0.1203 -
0.2768 -0.2128 0.2130 -0.3698 0.0945
-0.1746 -2.1848 -41.5595
65.3800 -44.1500 0.2778 0.0302 -
0.0955 -0.2594 0.1725 -0.3602 0.0867
-0.1753 -2.1861 -41.5580
65.4000 -44.9100 -0.5436 -0.0165
0.0867 -0.2838 0.1332 -0.3504 0.0792
-0.1760 -2.1875 -41.5564
65.4200 -44.2200 -0.0069 0.1054
0.2099 -0.2832 0.0955 -0.3407 0.0719
-0.1767 -2.1888 -41.5548
65.4400 -43.2400 0.6248 0.2144
0.2379 -0.2572 0.0600 -0.3309 0.0649
-0.1775 -2.1900 -41.5532
65.4600 -44.7000 -0.7094 0.1543
0.1628 -0.2079 0.0269 -0.3212 0.0581
-0.1783 -2.1912 -41.5517
65.4800 -44.4200 -0.1425 -0.0112
0.0154 -0.1394 -0.0038 -0.3116 0.0516
-0.1791 -2.1924 -41.5501
65.5000 -43.8000 0.8826 -0.1536 -
0.1510 -0.0573 -0.0321 -0.3021 0.0454
-0.1799 -2.1935 -41.5485
65.5200 -45.5600 -0.9700 -0.1133 -
0.2797 0.0320 -0.0580 -0.2928 0.0395
-0.1808 -2.1946 -41.5469
65.5400 -43.5800 0.9066 -0.0307 -
0.3177 0.1217 -0.0817 -0.2837 0.0339
-0.1817 -2.1956 -41.5453
65.5600 -45.1700 -0.6411 -0.2029 -
0.2388 0.2053 -0.1032 -0.2748 0.0285
-0.1826 -2.1966 -41.5438
65.5800 -44.2900 0.1942 -0.2424 -
0.0607 0.2768 -0.1226 -0.2661 0.0235
-0.1835 -2.1976 -41.5422
65.6000 -44.0800 -0.5074 0.2097
0.1531 0.3310 -0.1400 -0.2578 0.0187
-0.1845 -2.1985 -41.5406
65.6200 -42.4000 0.7234 0.5385
0.3211 0.3626 -0.1556 -0.2497 0.0143
-0.1854 -2.1994 -41.5390
65.6400 -44.0400 -0.6545 0.1936
0.3877 0.3681 -0.1693 -0.2420 0.0102
-0.1864 -2.2002 -41.5374

65.6600 -43.2900 0.6815 -0.3482
0.3632 0.3467 -0.1812 -0.2347 0.0063
-0.1875 -2.2010 -41.5358
65.6800 -45.0300 -0.9446 -0.3605
0.2855 0.3005 -0.1912 -0.2276 0.0028
-0.1885 -2.2017 -41.5342
65.7000 -42.9600 1.0554 -0.0604
0.1921 0.2334 -0.1994 -0.2210 -0.0004
-0.1896 -2.2024 -41.5326
65.7200 -44.9200 -0.9454 0.1147
0.0959 0.1510 -0.2056 -0.2147 -0.0033
-0.1907 -2.2031 -41.5311
65.7400 -44.3700 -0.2679 0.2569 -
0.0118 0.0601 -0.2098 -0.2088 -0.0059
-0.1918 -2.2037 -41.5295
65.7600 -43.4000 0.9352 0.3350 -
0.1368 -0.0324 -0.2119 -0.2032 -0.0082
-0.1929 -2.2043 -41.5279
65.7800 -44.8400 -0.2760 0.2373 -
0.2649 -0.1206 -0.2120 -0.1980 -0.0102
-0.1941 -2.2049 -41.5263
65.8000 -45.6000 -0.7438 -0.0359 -
0.3623 -0.1996 -0.2100 -0.1931 -0.0119
-0.1952 -2.2054 -41.5247
65.8200 -44.2700 0.9102 -0.3505 -
0.3954 -0.2647 -0.2058 -0.1885 -0.0133
-0.1964 -2.2058 -41.5231
65.8400 -46.1200 -0.9340 -0.3903 -
0.3464 -0.3125 -0.1996 -0.1843 -0.0144
-0.1976 -2.2062 -41.5215
65.8600 -44.4100 0.5845 -0.0832 -
0.2258 -0.3414 -0.1913 -0.1802 -0.0152
-0.1988 -2.2066 -41.5199
65.8800 -43.8700 0.6379 0.1910 -
0.0666 -0.3514 -0.1810 -0.1765 -0.0158
-0.2001 -2.2069 -41.5183
65.9000 -44.7800 -0.4369 0.2054
0.0914 -0.3436 -0.1689 -0.1729 -0.0161
-0.2013 -2.2072 -41.5167
65.9200 -44.8800 -0.6549 0.1263
0.2121 -0.3193 -0.1553 -0.1695 -0.0161
-0.2025 -2.2075 -41.5151
65.9400 -43.9000 0.1378 0.0934
0.2724 -0.2802 -0.1405 -0.1662 -0.0159
-0.2038 -2.2077 -41.5135
65.9600 -43.3500 0.8177 0.0360
0.2656 -0.2288 -0.1247 -0.1631 -0.0154
-0.2051 -2.2079 -41.5119
65.9800 -45.1400 -0.9367 -0.0759
0.2023 -0.1681 -0.1083 -0.1600 -0.0147
-0.2063 -2.2080 -41.5103

66.0000 -44.3000 -0.0034 -0.0861
0.1062 -0.1017 -0.0914 -0.1570 -0.0138
-0.2076 -2.2081 -41.5087
66.0200 -43.4100 0.8536 0.0064
0.0042 -0.0329 -0.0745 -0.1540 -0.0127
-0.2089 -2.2082 -41.5071
66.0400 -45.0000 -0.9240 0.0844 -
0.0807 0.0347 -0.0577 -0.1510 -0.0114
-0.2102 -2.2082 -41.5055
66.0600 -43.5000 0.4961 0.0268 -
0.1321 0.0981 -0.0413 -0.1480 -0.0099
-0.2115 -2.2082 -41.5039
66.0800 -43.1400 0.8313 -0.0891 -
0.1395 0.1548 -0.0255 -0.1449 -0.0082
-0.2127 -2.2081 -41.5023
66.1000 -45.5200 -1.3266 -0.1618 -
0.1024 0.2031 -0.0108 -0.1417 -0.0063
-0.2140 -2.2080 -41.5007
66.1200 -43.3100 0.6842 -0.1367 -
0.0281 0.2418 0.0026 -0.1384 -0.0043
-0.2153 -2.2079 -41.4991
66.1400 -42.4000 1.3889 -0.0732
0.0646 0.2700 0.0145 -0.1350 -0.0021
-0.2166 -2.2077 -41.4975
66.1600 -44.4000 -0.6801 0.0031
0.1470 0.2872 0.0247 -0.1315 0.0002
-0.2178 -2.2075 -41.4959
66.1800 -44.4800 -1.2964 0.1830
0.1913 0.2926 0.0330 -0.1278 0.0026
-0.2191 -2.2072 -41.4943
66.2000 -42.1100 0.9112 0.4619
0.1812 0.2859 0.0394 -0.1239 0.0052
-0.2203 -2.2069 -41.4926
66.2200 -42.5300 0.5526 0.4725
0.1129 0.2669 0.0438 -0.1198 0.0078
-0.2216 -2.2066 -41.4910
66.2400 -43.6400 0.0267 0.0391
0.0028 0.2360 0.0465 -0.1155 0.0105
-0.2228 -2.2062 -41.4894
66.2600 -44.9100 -0.5172 -0.4746 -
0.1009 0.1948 0.0475 -0.1110 0.0134
-0.2240 -2.2058 -41.4878
66.2800 -44.2600 0.2198 -0.6086 -
0.1459 0.1452 0.0471 -0.1063 0.0162
-0.2252 -2.2054 -41.4862
66.3000 -44.8700 -0.6219 -0.2617 -
0.1087 0.0894 0.0454 -0.1013 0.0192
-0.2264 -2.2049 -41.4846
66.3200 -42.8700 0.8083 0.2377 -
0.0128 0.0294 0.0426 -0.0960 0.0222
-0.2276 -2.2044 -41.4830

66.3400 -44.1400 -0.7231 0.4908
0.0916 -0.0325 0.0391 -0.0905 0.0252
-0.2287 -2.2038 -41.4814
66.3600 -42.9200 0.3972 0.4709
0.1499 -0.0937 0.0349 -0.0847 0.0282
-0.2298 -2.2032 -41.4797
66.3800 -43.1000 0.5265 0.2866
0.1304 -0.1515 0.0305 -0.0786 0.0313
-0.2309 -2.2026 -41.4781
66.4000 -44.8500 -0.7610 0.0506
0.0433 -0.2026 0.0260 -0.0723 0.0344
-0.2320 -2.2019 -41.4765
66.4200 -43.7500 0.7729 -0.1881 -
0.0650 -0.2442 0.0217 -0.0656 0.0374
-0.2331 -2.2012 -41.4749
66.4400 -45.3700 -0.5686 -0.4551 -
0.1444 -0.2738 0.0178 -0.0587 0.0405
-0.2341 -2.2004 -41.4733
66.4600 -45.2700 -0.2682 -0.6168 -
0.1679 -0.2895 0.0144 -0.0515 0.0435
-0.2351 -2.1996 -41.4717
66.4800 -43.8900 0.7802 -0.5001 -
0.1386 -0.2901 0.0115 -0.0440 0.0465
-0.2361 -2.1988 -41.4700
66.5000 -44.8100 -0.4190 -0.0540 -
0.0832 -0.2755 0.0092 -0.0363 0.0494
-0.2370 -2.1980 -41.4684
66.5200 -44.3800 -0.7260 0.5514 -
0.0326 -0.2464 0.0075 -0.0284 0.0523
-0.2379 -2.1971 -41.4668
66.5400 -42.3600 0.8840 0.9222 -
0.0074 -0.2041 0.0063 -0.0202 0.0551
-0.2388 -2.1961 -41.4652
66.5600 -43.4100 -0.0014 0.5847 -
0.0137 -0.1508 0.0055 -0.0118 0.0578
-0.2396 -2.1952 -41.4635
66.5800 -44.6800 -0.3977 -0.2430 -
0.0334 -0.0898 0.0050 -0.0032 0.0605
-0.2405 -2.1942 -41.4619
66.6000 -45.0500 -0.2159 -0.8846 -
0.0356 -0.0251 0.0046 0.0055 0.0631
-0.2412 -2.1931 -41.4603
66.6200 -44.5500 -0.0263 -0.8333
0.0057 0.0394 0.0043 0.0144 0.0655
-0.2420 -2.1921 -41.4587
66.6400 -44.3000 -0.4999 -0.1758
0.0860 0.1003 0.0039 0.0235 0.0679
-0.2426 -2.1909 -41.4570
66.6600 -42.1100 0.7447 0.5258
0.1792 0.1548 0.0033 0.0326 0.0701
-0.2433 -2.1898 -41.4554

66.6800 -42.4500 0.1929 0.7479
0.2523 0.2005 0.0023 0.0419 0.0722
-0.2439 -2.1886 -41.4538
66.7000 -43.6300 -0.8794 0.5363
0.2707 0.2354 0.0008 0.0512 0.0742
-0.2445 -2.1874 -41.4522
66.7200 -42.2100 0.8134 0.2165
0.2153 0.2587 -0.0013 0.0606 0.0760
-0.2450 -2.1861 -41.4505
66.7400 -43.5700 -0.1948 -0.0040
0.0980 0.2710 -0.0041 0.0699 0.0777
-0.2454 -2.1849 -41.4489
66.7600 -44.2600 -0.5600 -0.1681 -
0.0430 0.2738 -0.0077 0.0793 0.0792
-0.2459 -2.1835 -41.4473
66.7800 -43.2600 0.6507 -0.3696 -
0.1606 0.2689 -0.0120 0.0886 0.0805
-0.2462 -2.1822 -41.4456
66.8000 -44.1800 0.0772 -0.5425 -
0.2173 0.2582 -0.0169 0.0979 0.0817
-0.2466 -2.1808 -41.4440
66.8200 -44.9700 -0.8716 -0.5067 -
0.1977 0.2429 -0.0225 0.1071 0.0827
-0.2468 -2.1794 -41.4424
66.8400 -42.9000 0.8579 -0.2199 -
0.1182 0.2236 -0.0284 0.1163 0.0835
-0.2470 -2.1779 -41.4407
66.8600 -43.5800 -0.0942 0.0927 -
0.0173 0.2001 -0.0345 0.1252 0.0841
-0.2472 -2.1764 -41.4391
66.8800 -43.7400 -0.7325 0.3569
0.0646 0.1722 -0.0405 0.1341 0.0845
-0.2473 -2.1749 -41.4375
66.9000 -42.3500 0.5003 0.5969
0.0974 0.1398 -0.0461 0.1427 0.0847
-0.2473 -2.1733 -41.4358
66.9200 -42.8800 -0.0096 0.6254
0.0764 0.1026 -0.0511 0.1512 0.0846
-0.2473 -2.1717 -41.4342
66.9400 -43.1800 0.0561 0.2957
0.0209 0.0606 -0.0551 0.1594 0.0844
-0.2473 -2.1701 -41.4326
66.9600 -44.3300 -0.4028 -0.1791 -
0.0358 0.0135 -0.0580 0.1673 0.0839
-0.2471 -2.1684 -41.4309
66.9800 -44.4000 0.0302 -0.5559 -
0.0622 -0.0384 -0.0595 0.1749 0.0832
-0.2469 -2.1667 -41.4293
67.0000 -44.0900 0.4625 -0.8052 -
0.0319 -0.0949 -0.0593 0.1822 0.0822
-0.2467 -2.1650 -41.4277

67.0200 -44.7800 -0.1180 -0.8885
0.0579 -0.1547 -0.0573 0.1890 0.0810
-0.2464 -2.1632 -41.4260
67.0400 -44.7700 -0.5411 -0.5866
0.1774 -0.2149 -0.0534 0.1955 0.0796
-0.2460 -2.1614 -41.4244
67.0600 -43.8600 -0.3508 0.1487
0.2812 -0.2720 -0.0476 0.2015 0.0779
-0.2455 -2.1596 -41.4227
67.0800 -42.0900 0.6790 0.8903
0.3256 -0.3225 -0.0398 0.2070 0.0759
-0.2450 -2.1577 -41.4211
67.1000 -42.0800 0.4369 1.0971
0.2741 -0.3634 -0.0302 0.2120 0.0737
-0.2444 -2.1558 -41.4195
67.1200 -43.7700 -0.4871 0.6273
0.1167 -0.3917 -0.0187 0.2164 0.0711
-0.2438 -2.1539 -41.4178
67.1400 -44.0200 0.2109 -0.1394 -
0.1065 -0.4053 -0.0056 0.2203 0.0684
-0.2430 -2.1519 -41.4162
67.1600 -45.3200 -0.5529 -0.6678 -
0.3393 -0.4019 0.0090 0.2235 0.0653
-0.2422 -2.1499 -41.4145
67.1800 -44.9300 0.1836 -0.7629 -
0.5237 -0.3798 0.0248 0.2260 0.0620
-0.2414 -2.1479 -41.4129
67.2000 -44.7800 0.1451 -0.5539 -
0.6185 -0.3376 0.0416 0.2278 0.0583
-0.2405 -2.1459 -41.4112
67.2200 -44.7800 -0.2790 -0.2239 -
0.6158 -0.2751 0.0589 0.2289 0.0544
-0.2395 -2.1438 -41.4096
67.2400 -43.8500 0.1411 0.0849 -
0.5312 -0.1947 0.0764 0.2292 0.0502
-0.2384 -2.1416 -41.4079
67.2600 -43.5700 -0.0188 0.2708 -
0.3899 -0.1001 0.0935 0.2287 0.0457
-0.2372 -2.1395 -41.4063
67.2800 -43.0700 0.1414 0.3293 -
0.2212 0.0038 0.1100 0.2273 0.0410
-0.2360 -2.1373 -41.4047
67.3000 -43.5500 -0.3928 0.2627 -
0.0507 0.1119 0.1252 0.2251 0.0359
-0.2347 -2.1351 -41.4030
67.3200 -42.7300 0.1284 0.0879
0.1049 0.2183 0.1388 0.2219 0.0305
-0.2334 -2.1329 -41.4014
67.3400 -42.1700 0.9520 -0.1854
0.2397 0.3166 0.1505 0.2179 0.0249
-0.2320 -2.1306 -41.3997

67.3600 -42.4700 0.3930 -0.3847
0.3547 0.4004 0.1596 0.2129 0.0190
-0.2305 -2.1283 -41.3981
67.3800 -44.2400 -1.6044 -0.2030
0.4503 0.4633 0.1658 0.2069 0.0128
-0.2289 -2.1260 -41.3964
67.4000 -40.5100 1.5982 0.2715
0.5228 0.5004 0.1687 0.2000 0.0064
-0.2273 -2.1236 -41.3948
67.4200 -42.0700 0.0383 0.3431
0.5621 0.5088 0.1679 0.1921 -0.0003
-0.2256 -2.1212 -41.3931
67.4400 -43.2000 -0.8775 0.0447
0.5572 0.4884 0.1634 0.1834 -0.0072
-0.2238 -2.1188 -41.3914
67.4600 -43.5200 -0.8472 -0.1824
0.5011 0.4413 0.1553 0.1737 -0.0144
-0.2220 -2.1163 -41.3898
67.4800 -42.5000 0.3233 -0.1037
0.3931 0.3720 0.1441 0.1632 -0.0219
-0.2201 -2.1139 -41.3881
67.5000 -42.9000 -0.0762 0.1655
0.2390 0.2858 0.1302 0.1518 -0.0295
-0.2182 -2.1113 -41.3865
67.5200 -42.5900 0.5243 0.2709
0.0528 0.1888 0.1141 0.1397 -0.0374
-0.2161 -2.1088 -41.3848
67.5400 -43.4400 0.1937 0.0307 -
0.1446 0.0871 0.0960 0.1269 -0.0455
-0.2140 -2.1062 -41.3832
67.5600 -44.6000 -0.3637 -0.3335 -
0.3235 -0.0131 0.0765 0.1135 -0.0538
-0.2119 -2.1036 -41.3815
67.5800 -44.8000 -0.2822 -0.3790 -
0.4539 -0.1060 0.0561 0.0994 -0.0622
-0.2096 -2.1010 -41.3799
67.6000 -45.1500 -0.7993 0.0468 -
0.5133 -0.1862 0.0351 0.0849 -0.0709
-0.2074 -2.0984 -41.3782
67.6200 -43.0000 0.9530 0.4876 -
0.5005 -0.2496 0.0139 0.0698 -0.0797
-0.2050 -2.0957 -41.3765
67.6400 -43.1300 0.8782 0.4325 -
0.4203 -0.2941 -0.0070 0.0544 -0.0886
-0.2026 -2.0930 -41.3749
67.6600 -45.3800 -1.0839 -0.0300 -
0.2862 -0.3193 -0.0271 0.0387 -0.0977
-0.2001 -2.0903 -41.3732
67.6800 -45.1800 -0.3367 -0.4180 -
0.1256 -0.3259 -0.0462 0.0227 -0.1070
-0.1976 -2.0875 -41.3716

67.7000 -43.8200 0.8136 -0.5043
0.0343 -0.3154 -0.0639 0.0064 -0.1163
-0.1950 -2.0847 -41.3699
67.7200 -44.7100 -0.3932 -0.2671
0.1698 -0.2899 -0.0797 -0.0099 -0.1258
-0.1923 -2.0819 -41.3682
67.7400 -43.9700 -0.2052 0.1334
0.2592 -0.2520 -0.0935 -0.0263 -0.1354
-0.1896 -2.0791 -41.3666
67.7600 -43.1900 0.2712 0.5316
0.2808 -0.2051 -0.1049 -0.0427 -0.1451
-0.1868 -2.0762 -41.3649
67.7800 -43.2700 -0.1385 0.6286
0.2279 -0.1528 -0.1138 -0.0591 -0.1548
-0.1840 -2.0733 -41.3633
67.8000 -43.4300 0.2814 0.2584
0.1151 -0.0992 -0.1200 -0.0754 -0.1646
-0.1811 -2.0704 -41.3616
67.8200 -44.6800 -0.3393 -0.3348 -
0.0123 -0.0486 -0.1231 -0.0915 -0.1744
-0.1781 -2.0674 -41.3599
67.8400 -44.4900 0.1649 -0.6515 -
0.0938 -0.0053 -0.1233 -0.1073 -0.1843
-0.1751 -2.0645 -41.3583
67.8600 -45.0500 -0.5108 -0.4224 -
0.0885 0.0275 -0.1203 -0.1230 -0.1943
-0.1720 -2.0615 -41.3566
67.8800 -44.1300 -0.2944 0.1003 -
0.0092 0.0483 -0.1143 -0.1382 -0.2042
-0.1689 -2.0585 -41.3549
67.9000 -42.3600 1.1907 0.4312
0.1029 0.0572 -0.1053 -0.1532 -0.2142
-0.1657 -2.0554 -41.3533
67.9200 -44.4000 -0.8732 0.3093
0.1995 0.0553 -0.0936 -0.1677 -0.2241
-0.1625 -2.0523 -41.3516
67.9400 -44.6400 -0.9489 0.1188
0.2317 0.0441 -0.0794 -0.1818 -0.2340
-0.1592 -2.0492 -41.3499
67.9600 -42.6200 1.1455 0.1279
0.1702 0.0260 -0.0631 -0.1953 -0.2439
-0.1558 -2.0461 -41.3483
67.9800 -43.7700 0.0234 0.2801
0.0375 0.0045 -0.0449 -0.2083 -0.2538
-0.1524 -2.0430 -41.3466
68.0000 -44.9900 -1.0465 0.2249 -
0.1061 -0.0169 -0.0253 -0.2207 -0.2636
-0.1489 -2.0398 -41.3449
68.0200 -43.4400 1.1203 -0.2739 -
0.1994 -0.0344 -0.0047 -0.2325 -0.2733
-0.1454 -2.0366 -41.3433

68.0400 -46.0500 -1.0559 -0.6998 -
0.2131 -0.0449 0.0166 -0.2435 -0.2830
-0.1419 -2.0334 -41.3416
68.0600 -43.6100 0.8999 -0.3485 -
0.1512 -0.0465 0.0382 -0.2538 -0.2926
-0.1382 -2.0301 -41.3399
68.0800 -44.3400 -0.6231 0.3913 -
0.0564 -0.0389 0.0596 -0.2633 -0.3021
-0.1346 -2.0269 -41.3383
68.1000 -43.0400 0.1486 0.8322
0.0145 -0.0221 0.0805 -0.2720 -0.3114
-0.1309 -2.0236 -41.3366
68.1200 -43.0000 0.3342 0.7184
0.0173 0.0031 0.1005 -0.2798 -0.3207
-0.1271 -2.0202 -41.3349
68.1400 -43.4300 0.5325 0.0634 -
0.0404 0.0347 0.1192 -0.2866 -0.3298
-0.1233 -2.0169 -41.3332
68.1600 -45.7500 -1.0186 -0.6249 -
0.1193 0.0694 0.1363 -0.2925 -0.3388
-0.1194 -2.0135 -41.3316
68.1800 -45.0200 -0.0601 -0.8588 -
0.1747 0.1033 0.1514 -0.2974 -0.3477
-0.1155 -2.0101 -41.3299
68.2000 -43.3800 1.2352 -0.6578 -
0.1749 0.1326 0.1642 -0.3012 -0.3563
-0.1115 -2.0067 -41.3282
68.2200 -45.1600 -1.2707 -0.1659 -
0.1140 0.1541 0.1745 -0.3039 -0.3648
-0.1075 -2.0033 -41.3265
68.2400 -43.3300 0.0429 0.3867 -
0.0023 0.1656 0.1820 -0.3054 -0.3732
-0.1035 -1.9998 -41.3249
68.2600 -41.9500 1.0507 0.6573
0.1423 0.1661 0.1865 -0.3058 -0.3813
-0.0994 -1.9964 -41.3232
68.2800 -42.8700 0.1339 0.3632
0.2916 0.1553 0.1880 -0.3050 -0.3892
-0.0952 -1.9929 -41.3215
68.3000 -43.9200 -0.2070 -0.2043
0.4116 0.1332 0.1865 -0.3029 -0.3970
-0.0910 -1.9893 -41.3198
68.3200 -44.7400 -0.8436 -0.4006
0.4699 0.1001 0.1820 -0.2995 -0.4045
-0.0868 -1.9858 -41.3182
68.3400 -42.7500 0.7131 -0.0931
0.4453 0.0577 0.1747 -0.2949 -0.4118
-0.0825 -1.9822 -41.3165
68.3600 -43.3000 0.0153 0.2701
0.3418 0.0088 0.1646 -0.2889 -0.4188
-0.0782 -1.9786 -41.3148

68.3800 -43.9200 -0.5116 0.3386
0.1727 -0.0424 0.1519 -0.2817 -0.4256
-0.0738 -1.9750 -41.3131
68.4000 -43.3500 0.5747 0.1281 -
0.0401 -0.0915 0.1367 -0.2732 -0.4322
-0.0694 -1.9714 -41.3114
68.4200 -44.5300 -0.0231 -0.1343 -
0.2611 -0.1338 0.1193 -0.2635 -0.4385
-0.0650 -1.9677 -41.3098
68.4400 -45.3400 -0.6227 -0.2280 -
0.4476 -0.1651 0.0998 -0.2526 -0.4445
-0.0605 -1.9640 -41.3081
68.4600 -44.1500 0.6130 -0.0578 -
0.5597 -0.1811 0.0784 -0.2405 -0.4503
-0.0560 -1.9604 -41.3064
68.4800 -45.0400 -0.4217 0.0522 -
0.5726 -0.1790 0.0553 -0.2273 -0.4558
-0.0514 -1.9566 -41.3047
68.5000 -44.0000 0.6731 -0.1078 -
0.4800 -0.1583 0.0306 -0.2131 -0.4610
-0.0469 -1.9529 -41.3030
68.5200 -45.3100 -0.8139 -0.1724 -
0.2962 -0.1213 0.0045 -0.1979 -0.4659
-0.0422 -1.9491 -41.3014
68.5400 -44.1800 -0.0525 0.0111 -
0.0586 -0.0718 -0.0228 -0.1817 -0.4705
-0.0376 -1.9453 -41.2997
68.5600 -42.7200 0.8754 0.1752
0.1828 -0.0140 -0.0512 -0.1647 -0.4748
-0.0329 -1.9415 -41.2980
68.5800 -43.7800 -0.3534 0.1139
0.3798 0.0474 -0.0805 -0.1468 -0.4788
-0.0281 -1.9377 -41.2963
68.6000 -43.9100 -0.5186 -0.0044
0.4973 0.1076 -0.1104 -0.1281 -0.4824
-0.0234 -1.9339 -41.2946
68.6200 -42.7600 0.5948 0.0001
0.5166 0.1620 -0.1408 -0.1088 -0.4857
-0.0185 -1.9300 -41.2929
68.6400 -43.5400 -0.3453 0.0557
0.4407 0.2075 -0.1714 -0.0888 -0.4887
-0.0137 -1.9261 -41.2912
68.6600 -43.2100 0.2890 -0.0300
0.2931 0.2428 -0.2019 -0.0682 -0.4914
-0.0089 -1.9222 -41.2896
68.6800 -43.9900 -0.2907 -0.1321
0.1079 0.2679 -0.2319 -0.0471 -0.4936
-0.0040 -1.9183 -41.2879
68.7000 -43.7200 0.1563 -0.0292 -
0.0811 0.2835 -0.2613 -0.0256 -0.4956
0.0010 -1.9144 -41.2862

68.7200 -43.8800 -0.2364 0.1940 -
0.2367 0.2913 -0.2898 -0.0038 -0.4971
0.0059 -1.9104 -41.2845
68.7400 -43.1600 0.7085 0.1639 -
0.3277 0.2927 -0.3171 0.0182 -0.4983
0.0109 -1.9064 -41.2828
68.7600 -44.8500 -0.5827 -0.2242 -
0.3457 0.2896 -0.3430 0.0404 -0.4991
0.0159 -1.9024 -41.2811
68.7800 -44.4000 0.0301 -0.4277 -
0.3044 0.2841 -0.3673 0.0626 -0.4995
0.0209 -1.8984 -41.2794
68.8000 -44.4200 -0.4742 -0.0645 -
0.2281 0.2781 -0.3896 0.0848 -0.4995
0.0260 -1.8944 -41.2777
68.8200 -43.0700 0.2020 0.4812 -
0.1441 0.2731 -0.4097 0.1067 -0.4991
0.0311 -1.8903 -41.2761
68.8400 -42.3900 0.6401 0.6342 -
0.0796 0.2696 -0.4271 0.1284 -0.4983
0.0362 -1.8862 -41.2744
68.8600 -43.3100 0.0605 0.2327 -
0.0452 0.2667 -0.4412 0.1497 -0.4970
0.0413 -1.8821 -41.2727
68.8800 -44.6200 -0.7269 -0.3020 -
0.0198 0.2617 -0.4513 0.1705 -0.4954
0.0464 -1.8780 -41.2710
68.9000 -44.5800 -0.4029 -0.5362
0.0232 0.2517 -0.4569 0.1907 -0.4933
0.0516 -1.8739 -41.2693
68.9200 -42.8700 1.0934 -0.4380
0.1016 0.2341 -0.4573 0.2102 -0.4907
0.0568 -1.8698 -41.2676
68.9400 -43.4900 -0.0574 -0.1078
0.2153 0.2063 -0.4521 0.2289 -0.4877
0.0620 -1.8656 -41.2659
68.9600 -44.3800 -1.4018 0.2568
0.3396 0.1665 -0.4408 0.2467 -0.4843
0.0672 -1.8614 -41.2642
68.9800 -41.5400 1.2052 0.4047
0.4411 0.1133 -0.4232 0.2635 -0.4803
0.0724 -1.8572 -41.2625
69.0000 -41.7100 1.1652 0.2603
0.4916 0.0459 -0.3991 0.2792 -0.4759
0.0777 -1.8530 -41.2608
69.0200 -44.4800 -1.4423 0.0215
0.4770 -0.0361 -0.3686 0.2937 -0.4711
0.0830 -1.8488 -41.2591
69.0400 -41.9600 1.2485 -0.0068
0.3990 -0.1319 -0.3318 0.3069 -0.4657
0.0882 -1.8445 -41.2574

69.0600 -43.7700 -0.3694 -0.0405
0.2823 -0.2389 -0.2890 0.3188 -0.4599
0.0935 -1.8402 -41.2557
69.0800 -44.2900 -0.4988 -0.2277
0.1561 -0.3537 -0.2409 0.3292 -0.4536
0.0988 -1.8359 -41.2540
69.1000 -43.6800 0.3920 -0.3511
0.0330 -0.4720 -0.1878 0.3381 -0.4468
0.1041 -1.8316 -41.2524
69.1200 -44.4100 -0.6510 -0.0476 -
0.0867 -0.5887 -0.1303 0.3454 -0.4395
0.1095 -1.8273 -41.2507
69.1400 -43.0700 0.5390 0.4100 -
0.2101 -0.6981 -0.0691 0.3511 -0.4317
0.1148 -1.8230 -41.2490
69.1600 -43.3800 0.1368 0.4415 -
0.3465 -0.7935 -0.0048 0.3550 -0.4234
0.1201 -1.8186 -41.2473
69.1800 -44.5100 -0.2213 0.0388 -
0.4926 -0.8684 0.0620 0.3572 -0.4145
0.1254 -1.8143 -41.2456
69.2000 -44.7000 0.0807 -0.3898 -
0.6258 -0.9167 0.1308 0.3575 -0.4052
0.1308 -1.8099 -41.2439
69.2200 -45.3500 -0.5790 -0.4634 -
0.7192 -0.9325 0.2009 0.3558 -0.3955
0.1361 -1.8055 -41.2422
69.2400 -44.2800 0.2153 -0.1912 -
0.7584 -0.9109 0.2716 0.3522 -0.3852
0.1414 -1.8011 -41.2405
69.2600 -43.8700 0.2182 0.1234 -
0.7559 -0.8490 0.3422 0.3465 -0.3744
0.1468 -1.7967 -41.2388
69.2800 -43.5900 0.0878 0.2271 -
0.7377 -0.7458 0.4119 0.3388 -0.3632
0.1521 -1.7922 -41.2371
69.3000 -43.8900 -0.3512 0.1573 -
0.7336 -0.6020 0.4795 0.3289 -0.3515
0.1574 -1.7877 -41.2354
69.3200 -43.2000 0.2198 0.1302 -
0.7578 -0.4205 0.5442 0.3167 -0.3393
0.1627 -1.7833 -41.2337
69.3400 -43.3700 -0.0977 0.0870 -
0.7902 -0.2082 0.6048 0.3023 -0.3267
0.1681 -1.7788 -41.2320
69.3600 -42.6200 0.5111 -0.1632 -
0.7919 0.0247 0.6602 0.2855 -0.3137
0.1734 -1.7743 -41.2303
69.3800 -43.5200 -0.6356 -0.3897 -
0.7205 0.2668 0.7093 0.2664 -0.3003
0.1787 -1.7698 -41.2286

69.4000 -42.0500 0.2928 -0.2249 -
0.5494 0.5066 0.7512 0.2449 -0.2864
0.1840 -1.7652 -41.2269
69.4200 -41.7600 -0.2256 0.1182 -
0.2828 0.7335 0.7846 0.2209 -0.2722
0.1892 -1.7607 -41.2252
69.4400 -40.3400 0.5089 0.2090
0.0623 0.9371 0.8085 0.1945 -0.2577
0.1945 -1.7561 -41.2235
69.4600 -40.9700 -0.3937 -0.0018
0.4551 1.1073 0.8217 0.1656 -0.2428
0.1998 -1.7515 -41.2217
69.4800 -39.9700 0.2661 -0.2763
0.8440 1.2338 0.8232 0.1343 -0.2275
0.2050 -1.7470 -41.2200
69.5000 -40.0300 0.0597 -0.3634
1.1677 1.3069 0.8118 0.1007 -0.2120
0.2102 -1.7423 -41.2183
69.5200 -40.0400 -0.5250 -0.1137
1.3701 1.3170 0.7870 0.0649 -0.1961
0.2154 -1.7377 -41.2166
69.5400 -38.5400 0.5404 0.4336
1.4066 1.2572 0.7488 0.0270 -0.1800
0.2206 -1.7331 -41.2149
69.5600 -39.3300 -0.3816 0.8945
1.2427 1.1292 0.6982 -0.0127 -0.1636
0.2258 -1.7285 -41.2132
69.5800 -39.1900 0.4139 0.8660
0.8717 0.9447 0.6368 -0.0540 -0.1470
0.2309 -1.7238 -41.2115
69.6000 -40.9200 0.0590 0.3137
0.3534 0.7185 0.5661 -0.0968 -0.1301
0.2360 -1.7191 -41.2098
69.6200 -42.7400 -0.2710 -0.4298 -
0.2097 0.4658 0.4876 -0.1408 -0.1131
0.2411 -1.7144 -41.2081
69.6400 -43.7300 0.2390 -0.9003 -
0.7078 0.2020 0.4029 -0.1859 -0.0959
0.2462 -1.7098 -41.2064
69.6600 -45.0900 -0.4533 -0.8696 -
1.0424 -0.0578 0.3136 -0.2317 -0.0785
0.2512 -1.7050 -41.2047
69.6800 -44.3000 0.5383 -0.5157 -
1.1594 -0.2990 0.2211 -0.2782 -0.0609
0.2562 -1.7003 -41.2030
69.7000 -44.7200 -0.0757 -0.1750 -
1.0651 -0.5097 0.1270 -0.3250 -0.0433
0.2612 -1.6956 -41.2013
69.7200 -44.8000 -0.4167 0.1763 -
0.8281 -0.6827 0.0329 -0.3720 -0.0255
0.2661 -1.6908 -41.1996

69.7400 -44.2700 -0.4345 0.6311 -
0.5381 -0.8128 -0.0598 -0.4190 -0.0076
0.2711 -1.6861 -41.1979
69.7600 -42.6600 0.9377 0.7912 -
0.2662 -0.8966 -0.1495 -0.4657 0.0103
0.2759 -1.6813 -41.1961
69.7800 -44.3600 -0.3757 0.3370 -
0.0627 -0.9330 -0.2351 -0.5119 0.0283
0.2808 -1.6765 -41.1944
69.8000 -44.8800 -0.3467 -0.2743
0.0675 -0.9245 -0.3155 -0.5575 0.0464
0.2856 -1.6717 -41.1927
69.8200 -45.1700 -0.4373 -0.4717
0.1398 -0.8772 -0.3898 -0.6022 0.0645
0.2904 -1.6669 -41.1910
69.8400 -43.9900 0.4312 -0.2792
0.1843 -0.7999 -0.4571 -0.6458 0.0825
0.2951 -1.6621 -41.1893
69.8600 -44.1900 0.1546 -0.1089
0.2382 -0.7025 -0.5168 -0.6881 0.1006
0.2998 -1.6573 -41.1876
69.8800 -44.3500 -0.3166 -0.1567
0.3163 -0.5930 -0.5684 -0.7288 0.1186
0.3045 -1.6525 -41.1859
69.9000 -43.8700 0.2515 -0.1732
0.3815 -0.4764 -0.6115 -0.7679 0.1366
0.3091 -1.6476 -41.1842
69.9200 -44.1200 -0.4491 0.0714
0.3899 -0.3568 -0.6459 -0.8051 0.1545
0.3136 -1.6428 -41.1825
69.9400 -42.8100 0.4068 0.4824
0.3141 -0.2381 -0.6718 -0.8401 0.1723
0.3182 -1.6379 -41.1807
69.9600 -43.2200 -0.0814 0.6047
0.1495 -0.1239 -0.6894 -0.8729 0.1900
0.3226 -1.6330 -41.1790
69.9800 -43.4600 0.4651 0.1439 -
0.0705 -0.0176 -0.6993 -0.9033 0.2076
0.3271 -1.6281 -41.1773
70.0000 -45.0900 -0.5921 -0.4796 -
0.2710 0.0780 -0.7020 -0.9311 0.2250
0.3314 -1.6232 -41.1756
70.0200 -44.3600 0.3361 -0.6562 -
0.3760 0.1610 -0.6980 -0.9561 0.2423
0.3358 -1.6183 -41.1739
70.0400 -44.1000 0.2227 -0.3763 -
0.3460 0.2303 -0.6877 -0.9783 0.2594
0.3400 -1.6134 -41.1722
70.0600 -44.3300 -0.5184 0.0809 -
0.2018 0.2841 -0.6715 -0.9976 0.2764
0.3443 -1.6085 -41.1705

70.0800 -42.5400 0.4483 0.3830
0.0078 0.3199 -0.6498 -1.0139 0.2931
0.3484 -1.6035 -41.1687
70.1000 -42.6100 0.1461 0.3664
0.2235 0.3356 -0.6228 -1.0271 0.3096
0.3525 -1.5986 -41.1670
70.1200 -43.4800 -0.6098 0.1660
0.3829 0.3292 -0.5909 -1.0372 0.3259
0.3566 -1.5936 -41.1653
70.1400 -42.2600 0.5876 0.0578
0.4392 0.3001 -0.5547 -1.0443 0.3420
0.3606 -1.5886 -41.1636
70.1600 -42.5900 0.1135 0.0930
0.3824 0.2503 -0.5150 -1.0483 0.3578
0.3645 -1.5837 -41.1619
70.1800 -43.7900 -0.7670 0.1228
0.2410 0.1854 -0.4726 -1.0493 0.3733
0.3684 -1.5787 -41.1602
70.2000 -42.4900 0.8651 -0.0220
0.0641 0.1123 -0.4284 -1.0472 0.3886
0.3722 -1.5737 -41.1584
70.2200 -44.1900 -0.5323 -0.3201 -
0.0996 0.0384 -0.3832 -1.0423 0.4037
0.3760 -1.5687 -41.1567
70.2400 -44.2900 -0.3808 -0.3894 -
0.2163 -0.0291 -0.3380 -1.0344 0.4184
0.3796 -1.5637 -41.1550
70.2600 -43.2200 0.2313 -0.0275 -
0.2739 -0.0837 -0.2938 -1.0237 0.4329
0.3833 -1.5586 -41.1533
70.2800 -43.2300 -0.0941 0.4023 -
0.2900 -0.1200 -0.2512 -1.0102 0.4472
0.3868 -1.5536 -41.1516
70.3000 -42.5700 0.4944 0.3597 -
0.2877 -0.1346 -0.2111 -0.9940 0.4611
0.3903 -1.5486 -41.1498
70.3200 -44.0800 -0.5402 -0.1121 -
0.2734 -0.1260 -0.1739 -0.9752 0.4747
0.3937 -1.5435 -41.1481
70.3400 -43.3600 0.2082 -0.3539 -
0.2308 -0.0957 -0.1401 -0.9537 0.4881
0.3971 -1.5385 -41.1464
70.3600 -43.9600 -0.7843 -0.0243 -
0.1440 -0.0478 -0.1099 -0.9297 0.5012
0.4003 -1.5334 -41.1447
70.3800 -41.5100 1.0844 0.2558 -
0.0167 0.0121 -0.0833 -0.9033 0.5139
0.4035 -1.5284 -41.1430
70.4000 -42.1200 0.3209 0.0597
0.1289 0.0776 -0.0604 -0.8745 0.5264
0.4067 -1.5233 -41.1412

70.4200 -43.6200 -1.1620 -0.1828
0.2539 0.1421 -0.0413 -0.8434 0.5385
0.4097 -1.5182 -41.1395
70.4400 -41.2800 0.8266 -0.0047
0.3211 0.1991 -0.0258 -0.8101 0.5504
0.4127 -1.5131 -41.1378
70.4600 -41.6700 0.0499 0.2345
0.3129 0.2427 -0.0137 -0.7747 0.5619
0.4156 -1.5080 -41.1361
70.4800 -41.7800 -0.0044 0.1365
0.2340 0.2689 -0.0050 -0.7372 0.5731
0.4184 -1.5029 -41.1344
70.5000 -42.4100 -0.2099 -0.0972
0.1147 0.2760 0.0006 -0.6979 0.5841
0.4212 -1.4978 -41.1326
70.5200 -42.1700 -0.0046 -0.2224 -
0.0034 0.2633 0.0036 -0.6567 0.5947
0.4238 -1.4927 -41.1309
70.5400 -42.0800 0.1223 -0.2428 -
0.0832 0.2317 0.0042 -0.6138 0.6050
0.4264 -1.4876 -41.1292
70.5600 -42.2200 -0.0373 -0.1844 -
0.1070 0.1834 0.0030 -0.5692 0.6149
0.4289 -1.4824 -41.1275
70.5800 -42.2300 -0.1202 -0.0170 -
0.0806 0.1217 0.0003 -0.5232 0.6246
0.4314 -1.4773 -41.1257
70.6000 -41.5900 0.1620 0.2073 -
0.0321 0.0512 -0.0034 -0.4758 0.6339
0.4337 -1.4722 -41.1240
70.6200 -41.6200 -0.1713 0.3716
0.0046 -0.0231 -0.0074 -0.4271 0.6429
0.4359 -1.4670 -41.1223
70.6400 -41.6000 -0.0111 0.3625
0.0095 -0.0956 -0.0112 -0.3773 0.6516
0.4381 -1.4619 -41.1206
70.6600 -41.5000 0.5542 0.0740 -
0.0180 -0.1614 -0.0144 -0.3264 0.6600
0.4402 -1.4567 -41.1188
70.6800 -42.3600 -0.0066 -0.3691 -
0.0649 -0.2159 -0.0164 -0.2746 0.6680
0.4422 -1.4515 -41.1171
70.7000 -43.2200 -0.7423 -0.5280 -
0.1124 -0.2550 -0.0168 -0.2219 0.6757
0.4440 -1.4464 -41.1154
70.7200 -42.1600 -0.1421 -0.1164 -
0.1453 -0.2752 -0.0152 -0.1685 0.6831
0.4459 -1.4412 -41.1137
70.7400 -40.8500 0.7365 0.4114 -
0.1608 -0.2748 -0.0116 -0.1144 0.6901
0.4476 -1.4360 -41.1119

70.7600 -41.8200 -0.3428 0.4137 -
0.1592 -0.2540 -0.0058 -0.0598 0.6968
0.4492 -1.4308 -41.1102
70.7800 -41.4200 0.5269 -0.1668 -
0.1400 -0.2143 0.0018 -0.0045 0.7032
0.4507 -1.4256 -41.1085
70.8000 -42.7700 -0.7499 -0.5741 -
0.1026 -0.1586 0.0109 0.0512 0.7092
0.4521 -1.4204 -41.1068
70.8200 -41.4000 0.2057 -0.3142 -
0.0505 -0.0908 0.0211 0.1075 0.7149
0.4535 -1.4152 -41.1050
70.8400 -40.7300 0.1416 0.2014
0.0068 -0.0159 0.0321 0.1642 0.7203
0.4547 -1.4100 -41.1033
70.8600 -40.6100 -0.1196 0.4739
0.0585 0.0605 0.0433 0.2213 0.7253
0.4558 -1.4048 -41.1016
70.8800 -40.1900 0.2453 0.3554
0.0975 0.1322 0.0544 0.2790 0.7299
0.4569 -1.3996 -41.0998
70.9000 -40.5300 -0.0444 0.0112
0.1250 0.1931 0.0648 0.3370 0.7342
0.4578 -1.3944 -41.0981
70.9200 -40.8000 -0.0906 -0.3059
0.1494 0.2378 0.0741 0.3955 0.7382
0.4586 -1.3892 -41.0964
70.9400 -40.7100 -0.0452 -0.3418
0.1810 0.2619 0.0819 0.4544 0.7418
0.4594 -1.3839 -41.0947
70.9600 -40.1100 0.1918 -0.0767
0.2250 0.2635 0.0878 0.5138 0.7450
0.4600 -1.3787 -41.0929
70.9800 -40.3300 -0.3832 0.1610
0.2720 0.2433 0.0914 0.5735 0.7478
0.4605 -1.3735 -41.0912
71.0000 -39.1700 0.6553 0.1257
0.3012 0.2044 0.0926 0.6337 0.7503
0.4610 -1.3682 -41.0895
71.0200 -40.7200 -0.6622 -0.0093
0.2799 0.1513 0.0911 0.6942 0.7524
0.4613 -1.3630 -41.0877
71.0400 -39.6400 0.3558 0.0543
0.1842 0.0892 0.0871 0.7550 0.7542
0.4615 -1.3577 -41.0860
71.0600 -40.0700 0.1105 0.1731
0.0166 0.0243 0.0807 0.8160 0.7555
0.4616 -1.3525 -41.0843
71.0800 -40.2200 0.1187 0.1594 -
0.1858 -0.0378 0.0718 0.8772 0.7565
0.4616 -1.3472 -41.0825

71.1000 -41.1000 -0.5047 0.0315 -
0.3648 -0.0914 0.0607 0.9386 0.7571
0.4615 -1.3420 -41.0808
71.1200 -40.3900 0.6537 -0.2144 -
0.4587 -0.1318 0.0476 0.9999 0.7572
0.4613 -1.3367 -41.0791
71.1400 -41.6500 -0.7250 -0.3764 -
0.4325 -0.1558 0.0328 1.0613 0.7570
0.4609 -1.3314 -41.0773
71.1600 -40.5300 0.0155 -0.1425 -
0.2888 -0.1637 0.0164 1.1224 0.7564
0.4605 -1.3262 -41.0756
71.1800 -39.6400 0.3697 0.2417 -
0.0808 -0.1582 -0.0013 1.1834 0.7554
0.4600 -1.3209 -41.0739
71.2000 -39.2100 0.4077 0.3079
0.1209 -0.1431 -0.0199 1.2440 0.7539
0.4593 -1.3156 -41.0721
71.2200 -40.0100 -0.5029 0.1051
0.2636 -0.1228 -0.0392 1.3042 0.7521
0.4586 -1.3103 -41.0704
71.2400 -39.9000 -0.1756 -0.0095
0.3255 -0.1015 -0.0589 1.3640 0.7498
0.4577 -1.3051 -41.0687
71.2600 -38.9800 0.5790 0.0171
0.3115 -0.0825 -0.0788 1.4231 0.7471
0.4567 -1.2998 -41.0669
71.2800 -39.8400 -0.2947 0.0009
0.2440 -0.0687 -0.0984 1.4816 0.7439
0.4557 -1.2945 -41.0652
71.3000 -39.3200 0.3072 -0.0814
0.1480 -0.0614 -0.1175 1.5393 0.7403
0.4545 -1.2892 -41.0635
71.3200 -40.2500 -0.4908 -0.0529
0.0411 -0.0610 -0.1356 1.5960 0.7363
0.4532 -1.2839 -41.0617
71.3400 -39.2900 0.4094 0.0790 -
0.0619 -0.0665 -0.1524 1.6516 0.7318
0.4518 -1.2786 -41.0600
71.3600 -39.7100 -0.0424 0.1149 -
0.1488 -0.0767 -0.1675 1.7061 0.7269
0.4503 -1.2733 -41.0583
71.3800 -40.2300 -0.2228 0.0067 -
0.2045 -0.0906 -0.1804 1.7592 0.7215
0.4487 -1.2680 -41.0565
71.4000 -39.9000 0.1046 -0.1415 -
0.2171 -0.1065 -0.1907 1.8108 0.7157
0.4470 -1.2627 -41.0548
71.4200 -39.9100 0.1153 -0.1884 -
0.1792 -0.1237 -0.1978 1.8609 0.7093
0.4452 -1.2574 -41.0531

71.4400 -40.0800 -0.5052 -0.0578 -
0.0982 -0.1417 -0.2015 1.9093 0.7025
0.4433 -1.2521 -41.0513
71.4600 -38.9000 0.5734 0.1417
0.0028 -0.1607 -0.2012 1.9557 0.6952
0.4413 -1.2468 -41.0496
71.4800 -39.3400 -0.0651 0.1528
0.0919 -0.1810 -0.1965 2.0002 0.6875
0.4392 -1.2415 -41.0478
71.5000 -39.4900 -0.1674 0.0131
0.1414 -0.2031 -0.1870 2.0426 0.6792
0.4370 -1.2362 -41.0461
71.5200 -39.5500 -0.1034 -0.0499
0.1375 -0.2269 -0.1725 2.0826 0.6705
0.4347 -1.2309 -41.0444
71.5400 -39.3100 0.1046 0.0004
0.0866 -0.2521 -0.1527 2.1202 0.6612
0.4323 -1.2255 -41.0426
71.5600 -39.2300 0.1774 0.0190
0.0135 -0.2776 -0.1274 2.1552 0.6515
0.4298 -1.2202 -41.0409
71.5800 -39.5100 -0.0260 -0.0493 -
0.0511 -0.3021 -0.0964 2.1875 0.6412
0.4272 -1.2149 -41.0392
71.6000 -40.1100 -0.5470 -0.0893 -
0.0843 -0.3245 -0.0598 2.2170 0.6304
0.4245 -1.2096 -41.0374
71.6200 -38.8700 0.6800 -0.0835 -
0.0761 -0.3443 -0.0175 2.2435 0.6192
0.4217 -1.2043 -41.0357
71.6400 -40.0700 -0.5914 -0.0821 -
0.0347 -0.3613 0.0305 2.2668 0.6074
0.4189 -1.1989 -41.0339
71.6600 -38.9100 0.2756 0.0485
0.0180 -0.3754 0.0842 2.2869 0.5950
0.4159 -1.1936 -41.0322
71.6800 -39.0200 -0.0815 0.2108
0.0566 -0.3872 0.1435 2.3036 0.5822
0.4129 -1.1883 -41.0305
71.7000 -38.6200 0.1711 0.1604
0.0656 -0.3971 0.2083 2.3167 0.5688
0.4097 -1.1829 -41.0287
71.7200 -39.2700 -0.1831 -0.0511
0.0482 -0.4048 0.2782 2.3262 0.5549
0.4065 -1.1776 -41.0270
71.7400 -39.0700 0.0094 -0.1942
0.0223 -0.4100 0.3529 2.3319 0.5405
0.4032 -1.1723 -41.0252
71.7600 -39.2200 -0.1421 -0.1359
0.0047 -0.4116 0.4316 2.3336 0.5256
0.3998 -1.1669 -41.0235

71.7800 −39.0400 −0.1352 0.0119
0.0020 −0.4089 0.5138 2.3314 0.5101
0.3964 −1.1616 −41.0218
71.8000 −38.2600 0.4419 0.0752
0.0133 −0.4012 0.5985 2.3249 0.4941
0.3928 −1.1563 −41.0200
71.8200 −39.2300 −0.5433 0.0294
0.0303 −0.3874 0.6849 2.3141 0.4777
0.3892 −1.1509 −41.0183
71.8400 −38.2100 0.3476 0.0073
0.0412 −0.3664 0.7721 2.2989 0.4607
0.3855 −1.1456 −41.0165
71.8600 −38.2800 0.1531 −0.0036
0.0391 −0.3372 0.8589 2.2791 0.4433
0.3817 −1.1403 −41.0148
71.8800 −38.7200 −0.1982 −0.0408
0.0184 −0.2980 0.9441 2.2546 0.4253
0.3778 −1.1349 −41.0130
71.9000 −38.3200 0.1574 −0.0628 −
0.0231 −0.2475 1.0266 2.2254 0.4070
0.3739 −1.1296 −41.0113
71.9200 −38.4500 −0.0839 −0.0091 −
0.0858 −0.1846 1.1048 2.1912 0.3882
0.3699 −1.1242 −41.0096
71.9400 −38.3900 −0.0493 0.0667 −
0.1664 −0.1078 1.1775 2.1520 0.3690
0.3658 −1.1189 −41.0078
71.9600 −38.1000 0.1602 0.0932 −
0.2561 −0.0163 1.2432 2.1078 0.3493
0.3616 −1.1135 −41.0061
71.9800 −38.6100 −0.3597 0.0442 −
0.3343 0.0900 1.3005 2.0584 0.3293
0.3574 −1.1082 −41.0043
72.0000 −37.7500 0.5336 −0.0610 −
0.3812 0.2101 1.3479 2.0037 0.3089
0.3531 −1.1028 −41.0026
72.0200 −38.8700 −0.5460 −0.1591 −
0.3878 0.3420 1.3842 1.9439 0.2882
0.3488 −1.0975 −41.0008
72.0400 −37.9300 0.2118 −0.1151 −
0.3578 0.4832 1.4082 1.8789 0.2671
0.3443 −1.0922 −40.9991
72.0600 −37.8600 0.1325 0.0255 −
0.3045 0.6298 1.4187 1.8087 0.2457
0.3398 −1.0868 −40.9973
72.0800 −37.7700 −0.1635 0.1688 −
0.2420 0.7766 1.4147 1.7335 0.2240
0.3353 −1.0815 −40.9956
72.1000 −37.4400 0.1437 0.2103 −
0.1702 0.9163 1.3953 1.6535 0.2020
0.3306 −1.0761 −40.9939

72.1200 -37.1500 0.4202 0.0155 -
0.0753 1.0396 1.3596 1.5687 0.1798
0.3260 -1.0707 -40.9921
72.1400 -38.4200 -0.3984 -0.3093
0.0597 1.1356 1.3071 1.4796 0.1573
0.3212 -1.0654 -40.9904
72.1600 -38.3000 -0.1636 -0.4707
0.2468 1.1936 1.2377 1.3863 0.1346
0.3164 -1.0600 -40.9886
72.1800 -37.7800 0.0253 -0.3883
0.4832 1.2050 1.1517 1.2891 0.1117
0.3116 -1.0547 -40.9869
72.2000 -37.2600 0.4344 -0.2227
0.7368 1.1644 1.0497 1.1883 0.0887
0.3066 -1.0493 -40.9851
72.2200 -37.8100 -0.2560 -0.0279
0.9459 1.0686 0.9333 1.0842 0.0654
0.3017 -1.0440 -40.9834
72.2400 -38.1200 -0.4473 0.2920
1.0381 0.9180 0.8043 0.9771 0.0421
0.2966 -1.0386 -40.9816
72.2600 -37.5500 0.3669 0.6496
0.9619 0.7171 0.6649 0.8673 0.0186
0.2916 -1.0333 -40.9799
72.2800 -38.4800 0.0570 0.7707
0.6956 0.4759 0.5172 0.7550 -0.0049
0.2864 -1.0279 -40.9781
72.3000 -39.6100 0.2138 0.4296
0.2740 0.2089 0.3635 0.6407 -0.0286
0.2812 -1.0226 -40.9764
72.3200 -41.3000 0.2789 -0.2046 -
0.2150 -0.0689 0.2060 0.5245 -0.0523
0.2760 -1.0172 -40.9746
72.3400 -43.7800 -0.6993 -0.6293 -
0.6650 -0.3421 0.0470 0.4067 -0.0760
0.2707 -1.0118 -40.9729
72.3600 -43.2000 0.6584 -0.5297 -
0.9762 -0.5957 -0.1114 0.2877 -0.0998
0.2654 -1.0065 -40.9711
72.3800 -44.5000 -0.4222 -0.2802 -
1.0851 -0.8150 -0.2669 0.1677 -0.1235
0.2600 -1.0011 -40.9694
72.4000 -43.9500 0.4465 -0.1904 -
0.9827 -0.9887 -0.4172 0.0471 -0.1472
0.2546 -0.9958 -40.9677
72.4200 -44.9600 -0.3880 -0.0940 -
0.7161 -1.1122 -0.5603 -0.0740 -0.1709
0.2492 -0.9904 -40.9659
72.4400 -44.1300 0.0818 0.1821 -
0.3669 -1.1853 -0.6941 -0.1951 -0.1945
0.2437 -0.9851 -40.9642

72.4600 -44.0400 -0.3234 0.4279 -
0.0130 -1.2102 -0.8166 -0.3160 -0.2180
0.2381 -0.9797 -40.9624
72.4800 -43.0200 0.9118 0.2424
0.2839 -1.1921 -0.9264 -0.4364 -0.2414
0.2326 -0.9744 -40.9607
72.5000 -45.4100 -1.1204 -0.1087
0.4865 -1.1371 -1.0225 -0.5561 -0.2647
0.2270 -0.9690 -40.9589
72.5200 -43.4600 0.7578 -0.0067
0.5827 -1.0520 -1.1040 -0.6747 -0.2878
0.2213 -0.9636 -40.9572
72.5400 -43.4100 0.6058 0.2385
0.5699 -0.9437 -1.1705 -0.7919 -0.3108
0.2156 -0.9583 -40.9554
72.5600 -43.9200 0.5273 0.1103
0.4577 -0.8192 -1.2218 -0.9075 -0.3336
0.2099 -0.9529 -40.9537
72.5800 -45.6600 -0.7974 -0.1296
0.2887 -0.6852 -1.2579 -1.0212 -0.3562
0.2041 -0.9476 -40.9519
72.6000 -45.5600 -0.6153 -0.0796
0.1089 -0.5480 -1.2793 -1.1327 -0.3785
0.1984 -0.9422 -40.9502
72.6200 -44.4300 0.6029 0.1460 -
0.0408 -0.4139 -1.2863 -1.2419 -0.4006
0.1926 -0.9369 -40.9484
72.6400 -44.4300 0.5403 0.1186 -
0.1268 -0.2884 -1.2794 -1.3486 -0.4225
0.1867 -0.9315 -40.9466
72.6600 -46.0300 -0.7616 -0.1782 -
0.1418 -0.1748 -1.2593 -1.4525 -0.4440
0.1809 -0.9262 -40.9449
72.6800 -44.8600 0.4431 -0.2034 -
0.1012 -0.0744 -1.2269 -1.5535 -0.4653
0.1750 -0.9208 -40.9431
72.7000 -45.3700 -0.5811 0.2042 -
0.0323 0.0136 -1.1832 -1.6515 -0.4862
0.1690 -0.9155 -40.9414
72.7200 -43.8400 0.9914 0.1744
0.0294 0.0906 -1.1292 -1.7465 -0.5068
0.1631 -0.9101 -40.9396
72.7400 -46.2400 -1.0856 -0.2691
0.0642 0.1580 -1.0660 -1.8383 -0.5270
0.1571 -0.9048 -40.9379
72.7600 -44.7700 0.2078 -0.2034
0.0677 0.2169 -0.9949 -1.9268 -0.5468
0.1512 -0.8994 -40.9361
72.7800 -44.1400 0.3900 0.1866
0.0433 0.2682 -0.9170 -2.0120 -0.5662
0.1452 -0.8941 -40.9344

72.8000 -44.1300 0.5601 0.2952 -
0.0010 0.3123 -0.8336 -2.0938 -0.5852
0.1391 -0.8887 -40.9326
72.8200 -45.6000 -0.7977 0.0805 -
0.0452 0.3496 -0.7460 -2.1722 -0.6038
0.1331 -0.8834 -40.9309
72.8400 -44.2500 0.7524 -0.2244 -
0.0636 0.3804 -0.6552 -2.2470 -0.6219
0.1270 -0.8780 -40.9291
72.8600 -46.0000 -0.8259 -0.3189 -
0.0383 0.4051 -0.5624 -2.3182 -0.6395
0.1210 -0.8727 -40.9274
72.8800 -44.6900 0.0784 -0.1267
0.0297 0.4238 -0.4688 -2.3858 -0.6566
0.1149 -0.8674 -40.9256
72.9000 -43.7000 0.7385 0.1245
0.1136 0.4367 -0.3752 -2.4496 -0.6732
0.1088 -0.8620 -40.9239
72.9200 -44.0700 0.2182 0.1871
0.1778 0.4438 -0.2826 -2.5097 -0.6893
0.1027 -0.8567 -40.9221
72.9400 -45.0000 -0.6670 0.1690
0.1914 0.4453 -0.1919 -2.5660 -0.7048
0.0966 -0.8514 -40.9204
72.9600 -44.8900 -0.5157 0.2608
0.1380 0.4423 -0.1040 -2.6184 -0.7198
0.0905 -0.8460 -40.9186
72.9800 -43.3600 1.0015 0.2941
0.0281 0.4364 -0.0197 -2.6668 -0.7342
0.0843 -0.8407 -40.9168
73.0000 -45.4200 -0.7256 0.0479 -
0.1090 0.4293 0.0602 -2.7113 -0.7480
0.0782 -0.8354 -40.9151
73.0200 -45.3000 -0.2004 -0.2071 -
0.2315 0.4226 0.1351 -2.7517 -0.7612
0.0721 -0.8300 -40.9133
73.0400 -45.0900 0.0886 -0.2545 -
0.2979 0.4174 0.2044 -2.7881 -0.7737
0.0659 -0.8247 -40.9116
73.0600 -44.5300 0.7015 -0.3087 -
0.2802 0.4140 0.2679 -2.8205 -0.7856
0.0598 -0.8194 -40.9098
73.0800 -45.6000 -0.5536 -0.3633 -
0.1829 0.4119 0.3252 -2.8487 -0.7968
0.0536 -0.8140 -40.9081
73.1000 -44.9200 -0.0694 -0.1413 -
0.0387 0.4100 0.3759 -2.8729 -0.8073
0.0475 -0.8087 -40.9063
73.1200 -44.3000 -0.1105 0.3011
0.1087 0.4061 0.4199 -2.8930 -0.8171
0.0413 -0.8034 -40.9046

73.1400 -43.2600 0.4031 0.6082
0.2211 0.3981 0.4569 -2.9090 -0.8262
0.0352 -0.7981 -40.9028
73.1600 -43.5100 0.2039 0.5115
0.2714 0.3838 0.4870 -2.9210 -0.8346
0.0291 -0.7928 -40.9010
73.1800 -44.4700 -0.2756 0.0943
0.2586 0.3622 0.5100 -2.9289 -0.8422
0.0229 -0.7874 -40.8993
73.2000 -44.7900 0.1874 -0.3822
0.2075 0.3328 0.5260 -2.9329 -0.8491
0.0168 -0.7821 -40.8975
73.2200 -45.5100 -0.3703 -0.6494
0.1466 0.2961 0.5351 -2.9329 -0.8552
0.0107 -0.7768 -40.8958
73.2400 -44.8400 0.2235 -0.5518
0.0957 0.2535 0.5375 -2.9290 -0.8605
0.0046 -0.7715 -40.8940
73.2600 -44.9100 -0.0737 -0.1551
0.0556 0.2073 0.5335 -2.9213 -0.8651
-0.0015 -0.7662 -40.8923
73.2800 -44.7700 -0.3952 0.3694
0.0143 0.1608 0.5233 -2.9099 -0.8689
-0.0076 -0.7609 -40.8905
73.3000 -43.6800 0.5705 0.7276 -
0.0389 0.1172 0.5073 -2.8947 -0.8719
-0.0136 -0.7556 -40.8887
73.3200 -44.4400 -0.0383 0.5522 -
0.1041 0.0795 0.4858 -2.8760 -0.8741
-0.0197 -0.7503 -40.8870
73.3400 -45.0500 0.0120 -0.0727 -
0.1692 0.0504 0.4592 -2.8537 -0.8756
-0.0257 -0.7450 -40.8852
73.3600 -46.2600 -0.4440 -0.6542 -
0.2134 0.0321 0.4278 -2.8280 -0.8763
-0.0317 -0.7397 -40.8835
73.3800 -45.6500 0.3111 -0.7358 -
0.2153 0.0260 0.3920 -2.7989 -0.8762
-0.0377 -0.7344 -40.8817
73.4000 -46.0100 -0.7212 -0.1480 -
0.1703 0.0326 0.3522 -2.7665 -0.8754
-0.0437 -0.7291 -40.8800
73.4200 -43.7000 0.7050 0.5847 -
0.1033 0.0509 0.3088 -2.7310 -0.8738
-0.0497 -0.7238 -40.8782
73.4400 -44.2600 0.0363 0.7031 -
0.0431 0.0794 0.2623 -2.6925 -0.8715
-0.0556 -0.7185 -40.8764
73.4600 -44.7700 -0.1381 0.2727 -
0.0079 0.1155 0.2131 -2.6510 -0.8685
-0.0615 -0.7132 -40.8747

73.4800 -45.0600 -0.0720 -0.1764
0.0013 0.1565 0.1619 -2.6067 -0.8648
-0.0674 -0.7079 -40.8729
73.5000 -45.1900 0.0011 -0.4105
0.0031 0.1985 0.1092 -2.5597 -0.8604
-0.0733 -0.7026 -40.8712
73.5200 -45.2500 -0.2105 -0.4291
0.0237 0.2375 0.0555 -2.5101 -0.8553
-0.0792 -0.6974 -40.8694
73.5400 -44.4400 0.5091 -0.2743
0.0750 0.2697 0.0015 -2.4581 -0.8496
-0.0850 -0.6921 -40.8676
73.5600 -45.0600 -0.5572 0.0318
0.1487 0.2928 -0.0522 -2.4036 -0.8431
-0.0909 -0.6868 -40.8659
73.5800 -44.4000 -0.2991 0.3184
0.2247 0.3054 -0.1051 -2.3470 -0.8361
-0.0966 -0.6815 -40.8641
73.6000 -43.1900 0.8728 0.3690
0.2795 0.3070 -0.1566 -2.2883 -0.8284
-0.1024 -0.6763 -40.8624
73.6200 -44.5300 -0.3892 0.1269
0.2891 0.2969 -0.2062 -2.2275 -0.8201
-0.1082 -0.6710 -40.8606
73.6400 -45.0200 -0.6083 -0.0622
0.2342 0.2753 -0.2533 -2.1650 -0.8112
-0.1139 -0.6657 -40.8588
73.6600 -44.2200 0.2182 0.0470
0.1118 0.2430 -0.2975 -2.1007 -0.8018
-0.1196 -0.6605 -40.8571
73.6800 -44.4200 -0.0990 0.2951 -
0.0563 0.2014 -0.3383 -2.0348 -0.7917
-0.1252 -0.6552 -40.8553
73.7000 -44.3000 0.5238 0.2302 -
0.2307 0.1520 -0.3752 -1.9675 -0.7811
-0.1309 -0.6499 -40.8536
73.7200 -44.8900 0.3365 -0.1749 -
0.3632 0.0966 -0.4079 -1.8989 -0.7700
-0.1365 -0.6447 -40.8518
73.7400 -46.3100 -0.8766 -0.4654 -
0.4090 0.0369 -0.4359 -1.8291 -0.7583
-0.1421 -0.6394 -40.8500
73.7600 -44.8400 0.3825 -0.2476 -
0.3391 -0.0258 -0.4589 -1.7583 -0.7461
-0.1477 -0.6342 -40.8483
73.7800 -44.4200 0.2923 0.1060 -
0.1758 -0.0905 -0.4765 -1.6866 -0.7334
-0.1532 -0.6289 -40.8465
73.8000 -44.0600 0.4332 0.1221
0.0331 -0.1563 -0.4884 -1.6141 -0.7202
-0.1587 -0.6237 -40.8448

73.8200	-45.1400	-0.7791	0.0085	
0.2364	-0.2224	-0.4942	-1.5410	-0.7065
	-0.1642	-0.6184	-40.8430	
73.8400	-43.9700	0.2408	0.0665	
0.3862	-0.2881	-0.4938	-1.4674	-0.6924
	-0.1696	-0.6132	-40.8412	
73.8600	-43.6600	0.3123	0.1340	
0.4546	-0.3521	-0.4870	-1.3933	-0.6778
	-0.1750	-0.6079	-40.8395	
73.8800	-43.7700	0.3155	0.0251	
0.4303	-0.4126	-0.4736	-1.3188	-0.6627
	-0.1804	-0.6027	-40.8377	
73.9000	-44.6100	-0.4239	-0.0386	
0.3139	-0.4671	-0.4539	-1.2442	-0.6473
	-0.1858	-0.5974	-40.8359	
73.9200	-44.4800	-0.4228	0.1557	
0.1232	-0.5126	-0.4279	-1.1693	-0.6314
	-0.1911	-0.5922	-40.8342	
73.9400	-43.3100	0.6544	0.3599	-
0.1022	-0.5466	-0.3960	-1.0943	-0.6151
	-0.1964	-0.5870	-40.8324	
73.9600	-44.3300	-0.0530	0.1936	-
0.3170	-0.5660	-0.3584	-1.0192	-0.5984
	-0.2016	-0.5818	-40.8307	
73.9800	-44.7600	-0.0553	-0.2720	-
0.4817	-0.5677	-0.3155	-0.9442	-0.5814
	-0.2069	-0.5765	-40.8289	
74.0000	-45.3400	-0.2589	-0.5854	-
0.5651	-0.5490	-0.2679	-0.8692	-0.5639
	-0.2121	-0.5713	-40.8271	
74.0200	-44.5900	-0.0834	-0.4307	-
0.5504	-0.5080	-0.2161	-0.7943	-0.5462
	-0.2172	-0.5661	-40.8254	
74.0400	-43.6700	0.2050	0.0399	-
0.4621	-0.4451	-0.1608	-0.7195	-0.5280
	-0.2224	-0.5609	-40.8236	
74.0600	-43.1500	-0.0091	0.4406	-
0.3405	-0.3620	-0.1027	-0.6450	-0.5096
	-0.2275	-0.5556	-40.8218	
74.0800	-42.6300	0.0637	0.5566	-
0.2242	-0.2608	-0.0426	-0.5708	-0.4908
	-0.2325	-0.5504	-40.8201	
74.1000	-42.5100	-0.0089	0.4009	-
0.1370	-0.1449	0.0188	-0.4969	-0.4717
	-0.2375	-0.5452	-40.8183	
74.1200	-42.7500	-0.2758	0.0718	-
0.0848	-0.0194	0.0807	-0.4234	-0.4523
	-0.2425	-0.5400	-40.8166	
74.1400	-41.9700	0.5582	-0.2908	-
0.0495	0.1094	0.1422	-0.3504	-0.4326
	-0.2475	-0.5348	-40.8148	

74.1600 -42.9400 -0.6700 -0.4540
0.0012 0.2343 0.2027 -0.2778 -0.4127
-0.2524 -0.5296 -40.8130
74.1800 -41.2900 0.5266 -0.3880
0.0993 0.3484 0.2613 -0.2058 -0.3925
-0.2573 -0.5244 -40.8113
74.2000 -41.5300 -0.1357 -0.2937
0.2525 0.4459 0.3173 -0.1345 -0.3720
-0.2621 -0.5192 -40.8095
74.2200 -40.6900 0.1070 -0.1667
0.4331 0.5222 0.3698 -0.0638 -0.3513
-0.2670 -0.5140 -40.8077
74.2400 -40.3500 -0.1429 0.1047
0.5916 0.5731 0.4182 0.0062 -0.3304
-0.2717 -0.5088 -40.8060
74.2600 -39.4500 0.1719 0.4635
0.6763 0.5952 0.4618 0.0754 -0.3093
-0.2765 -0.5037 -40.8042
74.2800 -39.5300 -0.1537 0.6449
0.6507 0.5860 0.5000 0.1438 -0.2879
-0.2812 -0.4985 -40.8024
74.3000 -39.1600 0.3983 0.4600
0.5078 0.5462 0.5327 0.2113 -0.2664
-0.2858 -0.4933 -40.8007
74.3200 -40.5800 -0.3833 0.0434
0.2828 0.4807 0.5597 0.2779 -0.2447
-0.2905 -0.4881 -40.7989
74.3400 -40.7600 -0.0465 -0.2865
0.0282 0.3960 0.5811 0.3434 -0.2228
-0.2951 -0.4829 -40.7971
74.3600 -40.8100 0.3557 -0.4349 -
0.2037 0.2986 0.5971 0.4080 -0.2008
-0.2996 -0.4778 -40.7954
74.3800 -41.3100 0.0221 -0.4740 -
0.3716 0.1952 0.6079 0.4714 -0.1786
-0.3041 -0.4726 -40.7936
74.4000 -41.7600 -0.2493 -0.3734 -
0.4579 0.0923 0.6135 0.5337 -0.1563
-0.3086 -0.4674 -40.7918
74.4200 -41.4200 -0.3056 -0.0701 -
0.4662 -0.0047 0.6142 0.5949 -0.1338
-0.3130 -0.4623 -40.7901
74.4400 -40.4400 0.2092 0.2918 -
0.4170 -0.0909 0.6102 0.6548 -0.1113
-0.3174 -0.4571 -40.7883
74.4600 -39.9100 0.5676 0.4033 -
0.3317 -0.1627 0.6017 0.7135 -0.0887
-0.3218 -0.4520 -40.7866
74.4800 -40.7900 -0.2684 0.1535 -
0.2288 -0.2177 0.5891 0.7710 -0.0659
-0.3261 -0.4468 -40.7848

74.5000 -40.9900 -0.2959 -0.1616 -
0.1235 -0.2548 0.5727 0.8271 -0.0432
-0.3303 -0.4417 -40.7830
74.5200 -40.5600 0.1199 -0.2542 -
0.0271 -0.2741 0.5527 0.8820 -0.0203
-0.3346 -0.4365 -40.7813
74.5400 -40.4900 -0.0438 -0.1394
0.0513 -0.2762 0.5297 0.9356 0.0026
-0.3387 -0.4314 -40.7795
74.5600 -40.3500 -0.0568 0.0233
0.1052 -0.2627 0.5041 0.9879 0.0255
-0.3429 -0.4262 -40.7777
74.5800 -40.0300 -0.0062 0.1680
0.1260 -0.2362 0.4764 1.0389 0.0485
-0.3470 -0.4211 -40.7760
74.6000 -39.6900 0.1996 0.2260
0.1101 -0.1994 0.4470 1.0886 0.0714
-0.3510 -0.4159 -40.7742
74.6200 -40.0700 -0.2068 0.0937
0.0651 -0.1552 0.4161 1.1369 0.0944
-0.3551 -0.4108 -40.7724
74.6400 -39.8700 0.1505 -0.0979
0.0045 -0.1060 0.3841 1.1839 0.1173
-0.3590 -0.4057 -40.7707
74.6600 -40.2800 -0.2223 -0.1332 -
0.0493 -0.0548 0.3513 1.2296 0.1402
-0.3630 -0.4006 -40.7689
74.6800 -39.7300 0.1679 -0.0242 -
0.0774 -0.0045 0.3177 1.2741 0.1630
-0.3669 -0.3954 -40.7671
74.7000 -39.5400 0.1028 0.0393 -
0.0701 0.0416 0.2838 1.3172 0.1857
-0.3707 -0.3903 -40.7654
74.7200 -39.7600 -0.1256 -0.0147 -
0.0286 0.0807 0.2496 1.3592 0.2083
-0.3745 -0.3852 -40.7636
74.7400 -40.0600 -0.2974 -0.0453
0.0379 0.1105 0.2154 1.3998 0.2309
-0.3782 -0.3801 -40.7618
74.7600 -38.7700 0.6164 -0.0320
0.1078 0.1294 0.1811 1.4393 0.2532
-0.3820 -0.3750 -40.7601
74.7800 -40.0800 -0.6608 -0.0198
0.1593 0.1367 0.1470 1.4776 0.2755
-0.3856 -0.3699 -40.7583
74.8000 -38.7200 0.5412 0.0444
0.1721 0.1324 0.1129 1.5147 0.2976
-0.3892 -0.3648 -40.7565
74.8200 -39.1800 0.0035 0.1121
0.1385 0.1175 0.0788 1.5507 0.3194
-0.3928 -0.3597 -40.7547

74.8400 -39.6600 -0.4767 0.1339
0.0665 0.0939 0.0446 1.5856 0.3411
-0.3963 -0.3546 -40.7530
74.8600 -39.3000 -0.0184 0.0904 -
0.0232 0.0648 0.0104 1.6194 0.3625
-0.3998 -0.3495 -40.7512
74.8800 -39.0600 0.5791 -0.0309 -
0.1080 0.0336 -0.0240 1.6521 0.3837
-0.4033 -0.3444 -40.7494
74.9000 -40.2400 -0.5622 -0.1486 -
0.1640 0.0039 -0.0585 1.6838 0.4046
-0.4066 -0.3393 -40.7477
74.9200 -39.7000 0.1244 -0.1284 -
0.1696 -0.0214 -0.0931 1.7145 0.4253
-0.4100 -0.3342 -40.7459
74.9400 -39.2400 0.4073 -0.0469 -
0.1235 -0.0409 -0.1278 1.7443 0.4456
-0.4133 -0.3292 -40.7441
74.9600 -39.9600 -0.3955 0.0059 -
0.0420 -0.0545 -0.1624 1.7731 0.4656
-0.4165 -0.3241 -40.7424
74.9800 -39.1400 0.1663 0.0654
0.0468 -0.0625 -0.1969 1.8011 0.4853
-0.4197 -0.3190 -40.7406
75.0000 -39.4900 -0.2901 0.1574
0.1130 -0.0654 -0.2311 1.8283 0.5045
-0.4229 -0.3140 -40.7388
75.0200 -38.7700 0.4264 0.1336
0.1363 -0.0636 -0.2651 1.8547 0.5234
-0.4260 -0.3089 -40.7371
75.0400 -39.4500 -0.0705 -0.0590
0.1131 -0.0581 -0.2987 1.8804 0.5419
-0.4290 -0.3038 -40.7353
75.0600 -39.8000 -0.2609 -0.1710
0.0577 -0.0499 -0.3319 1.9055 0.5600
-0.4320 -0.2988 -40.7335
75.0800 -39.5300 -0.0813 -0.0382 -
0.0108 -0.0400 -0.3647 1.9298 0.5776
-0.4350 -0.2937 -40.7318
75.1000 -39.1500 0.1722 0.1190 -
0.0763 -0.0288 -0.3969 1.9536 0.5947
-0.4379 -0.2887 -40.7300
75.1200 -39.0400 0.3906 0.0586 -
0.1226 -0.0163 -0.4286 1.9769 0.6113
-0.4407 -0.2836 -40.7282
75.1400 -39.9900 -0.6328 -0.0969 -
0.1391 -0.0035 -0.4598 1.9996 0.6275
-0.4436 -0.2786 -40.7265
75.1600 -39.0000 0.3891 -0.1133 -
0.1194 0.0083 -0.4903 2.0219 0.6431
-0.4463 -0.2735 -40.7247

75.1800 -39.0000 0.2800 -0.0361 -
0.0693 0.0173 -0.5202 2.0437 0.6582
-0.4490 -0.2685 -40.7229
75.2000 -39.6600 -0.4074 0.0373 -
0.0047 0.0218 -0.5495 2.0650 0.6726
-0.4517 -0.2635 -40.7211
75.2200 -39.1300 0.0274 0.1229
0.0537 0.0205 -0.5781 2.0859 0.6866
-0.4543 -0.2584 -40.7194
75.2400 -38.6500 0.3477 0.1293
0.0949 0.0127 -0.6058 2.1065 0.6999
-0.4568 -0.2534 -40.7176
75.2600 -39.5900 -0.3041 -0.0186
0.1155 -0.0019 -0.6328 2.1266 0.7126
-0.4593 -0.2484 -40.7158
75.2800 -39.1100 0.0401 -0.1566
0.1158 -0.0227 -0.6588 2.1464 0.7247
-0.4618 -0.2434 -40.7141
75.3000 -39.3000 -0.0427 -0.1088
0.0955 -0.0480 -0.6838 2.1658 0.7362
-0.4642 -0.2384 -40.7123
75.3200 -39.5600 -0.4392 0.1051
0.0617 -0.0759 -0.7074 2.1848 0.7470
-0.4665 -0.2333 -40.7105
75.3400 -38.3800 0.7561 0.1863
0.0224 -0.1039 -0.7293 2.2035 0.7571
-0.4688 -0.2283 -40.7088
75.3600 -39.9900 -0.5122 -0.0413 -
0.0178 -0.1292 -0.7492 2.2218 0.7666
-0.4710 -0.2233 -40.7070
75.3800 -39.8900 -0.3782 -0.2331 -
0.0492 -0.1491 -0.7666 2.2396 0.7755
-0.4732 -0.2183 -40.7052
75.4000 -38.9700 0.4856 -0.1503 -
0.0655 -0.1612 -0.7812 2.2570 0.7836
-0.4753 -0.2133 -40.7034
75.4200 -39.4700 -0.2038 0.0787 -
0.0726 -0.1633 -0.7927 2.2738 0.7911
-0.4774 -0.2083 -40.7017
75.4400 -39.0900 0.1184 0.1714 -
0.0777 -0.1543 -0.8009 2.2901 0.7979
-0.4794 -0.2034 -40.6999
75.4600 -39.2800 -0.0860 0.1081 -
0.0853 -0.1338 -0.8056 2.3058 0.8040
-0.4814 -0.1984 -40.6981
75.4800 -39.2000 -0.0930 0.0653 -
0.0981 -0.1030 -0.8066 2.3208 0.8094
-0.4833 -0.1934 -40.6964
75.5000 -39.1000 -0.0068 0.0630 -
0.1074 -0.0643 -0.8039 2.3351 0.8142
-0.4851 -0.1884 -40.6946

75.5200 -38.7200 0.3720 -0.0544 -
0.0928 -0.0213 -0.7971 2.3486 0.8183
-0.4869 -0.1834 -40.6928
75.5400 -38.8800 0.3706 -0.2731 -
0.0422 0.0219 -0.7862 2.3613 0.8216
-0.4886 -0.1785 -40.6911
75.5600 -39.9400 -0.9202 -0.2571
0.0413 0.0609 -0.7709 2.3730 0.8244
-0.4903 -0.1735 -40.6893
75.5800 -37.9300 0.6725 0.1154
0.1392 0.0915 -0.7510 2.3837 0.8264
-0.4919 -0.1686 -40.6875
75.6000 -37.7400 0.4133 0.3432
0.2142 0.1102 -0.7264 2.3933 0.8278
-0.4935 -0.1636 -40.6857
75.6200 -38.4800 -0.1103 0.1608
0.2327 0.1144 -0.6967 2.4017 0.8285
-0.4950 -0.1587 -40.6840
75.6400 -39.0800 -0.4350 -0.1472
0.1882 0.1035 -0.6620 2.4088 0.8285
-0.4965 -0.1537 -40.6822
75.6600 -38.4600 0.2332 -0.2516
0.0969 0.0790 -0.6220 2.4145 0.8279
-0.4978 -0.1488 -40.6804
75.6800 -38.8400 0.0163 -0.1156 -
0.0134 0.0433 -0.5767 2.4187 0.8266
-0.4992 -0.1438 -40.6787
75.7000 -38.8200 -0.2353 0.1125 -
0.1142 -0.0002 -0.5263 2.4211 0.8246
-0.5004 -0.1389 -40.6769
75.7200 -38.4600 0.2593 0.2267 -
0.1813 -0.0481 -0.4708 2.4218 0.8219
-0.5016 -0.1340 -40.6751
75.7400 -38.9300 -0.2530 0.1099 -
0.2042 -0.0974 -0.4105 2.4205 0.8187
-0.5027 -0.1290 -40.6733
75.7600 -38.6100 0.3133 -0.0876 -
0.1863 -0.1451 -0.3455 2.4172 0.8147
-0.5038 -0.1241 -40.6716
75.7800 -39.0800 -0.2224 -0.1922 -
0.1316 -0.1892 -0.2759 2.4116 0.8101
-0.5048 -0.1192 -40.6698
75.8000 -38.6500 -0.0726 -0.1121 -
0.0481 -0.2293 -0.2022 2.4038 0.8049
-0.5058 -0.1143 -40.6680
75.8200 -38.4500 -0.0195 0.0510
0.0496 -0.2656 -0.1243 2.3934 0.7991
-0.5066 -0.1094 -40.6663
75.8400 -37.6900 0.4681 0.0661
0.1391 -0.2987 -0.0426 2.3804 0.7926
-0.5074 -0.1045 -40.6645

75.8600	-38.8300	-0.6291	-0.0112	
0.1983	-0.3293	0.0426	2.3648	0.7855
	-0.5082	-0.0996	-40.6627	
75.8800	-37.6900	0.4255	0.0423	
0.2153	-0.3581	0.1309	2.3463	0.7778
	-0.5089	-0.0947	-40.6609	
75.9000	-37.9700	0.0586	0.1169	
0.1867	-0.3854	0.2219	2.3248	0.7695
	-0.5095	-0.0898	-40.6592	
75.9200	-38.5500	-0.3486	0.0737	
0.1227	-0.4112	0.3149	2.3003	0.7606
	-0.5100	-0.0849	-40.6574	
75.9400	-38.0700	0.2934	-0.0311	
0.0420	-0.4347	0.4090	2.2727	0.7512
	-0.5105	-0.0801	-40.6556	
75.9600	-38.5600	-0.1618	-0.0986	-
0.0355	-0.4540	0.5032	2.2419	0.7411
	-0.5109	-0.0752	-40.6539	
75.9800	-38.3400	0.0795	-0.1209	-
0.0932	-0.4660	0.5963	2.2077	0.7305
	-0.5112	-0.0703	-40.6521	
76.0000	-38.4600	-0.0771	-0.0908	-
0.1244	-0.4668	0.6869	2.1702	0.7194
	-0.5115	-0.0655	-40.6503	
76.0200	-37.8800	0.1549	-0.0010	-
0.1340	-0.4530	0.7740	2.1291	0.7077
	-0.5117	-0.0606	-40.6485	
76.0400	-38.2700	-0.2080	0.0880	-
0.1359	-0.4214	0.8561	2.0844	0.6955
	-0.5118	-0.0558	-40.6468	
76.0600	-37.9200	0.1204	0.1406	-
0.1465	-0.3691	0.9322	2.0361	0.6828
	-0.5119	-0.0509	-40.6450	
76.0800	-37.9800	-0.0962	0.1026	-
0.1740	-0.2941	1.0012	1.9841	0.6697
	-0.5119	-0.0461	-40.6432	
76.1000	-37.6300	0.3720	-0.0592	-
0.2182	-0.1955	1.0621	1.9282	0.6560
	-0.5118	-0.0413	-40.6415	
76.1200	-38.5800	-0.5476	-0.2091	-
0.2692	-0.0742	1.1140	1.8685	0.6419
	-0.5117	-0.0364	-40.6397	
76.1400	-37.5800	0.3715	-0.1561	-
0.3193	0.0670	1.1560	1.8050	0.6273
	-0.5115	-0.0316	-40.6379	
76.1600	-37.7800	0.1525	-0.0007	-
0.3623	0.2228	1.1871	1.7376	0.6123
	-0.5112	-0.0268	-40.6361	
76.1800	-37.8100	-0.3518	0.1572	-
0.3919	0.3858	1.2064	1.6664	0.5969
	-0.5109	-0.0220	-40.6344	

76.2000 -37.2700 0.0462 0.2791 -
0.3988 0.5471 1.2131 1.5915 0.5810
-0.5105 -0.0172 -40.6326
76.2200 -36.9400 0.3550 0.2314 -
0.3676 0.6970 1.2063 1.5130 0.5648
-0.5100 -0.0124 -40.6308
76.2400 -37.6300 -0.0250 -0.0841 -
0.2791 0.8265 1.1852 1.4310 0.5483
-0.5094 -0.0076 -40.6291
76.2600 -38.0400 -0.4514 -0.4261 -
0.1164 0.9275 1.1491 1.3458 0.5314
-0.5088 -0.0028 -40.6273
76.2800 -37.2100 0.4704 -0.5648
0.1226 0.9929 1.0979 1.2576 0.5141
-0.5081 0.0020 -40.6255
76.3000 -37.9000 -0.3428 -0.4919
0.4191 1.0171 1.0315 1.1666 0.4965
-0.5074 0.0067 -40.6237
76.3200 -37.1300 -0.0429 -0.2195
0.7277 0.9953 0.9506 1.0731 0.4787
-0.5066 0.0115 -40.6220
76.3400 -36.5600 0.2351 0.1528
0.9854 0.9241 0.8565 0.9773 0.4605
-0.5057 0.0163 -40.6202
76.3600 -36.5500 0.0955 0.4874
1.1236 0.8022 0.7510 0.8795 0.4421
-0.5047 0.0210 -40.6184
76.3800 -36.8900 -0.1383 0.7548
1.0819 0.6329 0.6360 0.7800 0.4234
-0.5037 0.0258 -40.6166
76.4000 -37.9800 -0.6365 0.8251
0.8317 0.4261 0.5135 0.6790 0.4045
-0.5026 0.0305 -40.6149
76.4200 -37.6400 1.0848 0.3930
0.4095 0.1950 0.3855 0.5768 0.3854
-0.5014 0.0353 -40.6131
76.4400 -41.6900 -1.1490 -0.3674 -
0.0929 -0.0463 0.2539 0.4737 0.3660
-0.5002 0.0400 -40.6113
76.4600 -41.7500 0.1141 -0.7205 -
0.5699 -0.2837 0.1207 0.3698 0.3465
-0.4989 0.0447 -40.6096
76.4800 -41.8200 0.5946 -0.5994 -
0.9243 -0.5032 -0.0121 0.2655 0.3268
-0.4976 0.0494 -40.6078
76.5000 -42.5300 0.3985 -0.3859 -
1.0960 -0.6910 -0.1425 0.1610 0.3070
-0.4962 0.0542 -40.6060
76.5200 -43.7500 -0.6639 -0.2138 -
1.0726 -0.8355 -0.2686 0.0566 0.2870
-0.4947 0.0589 -40.6042

76.5400 -42.9900 0.1188 -0.0193 -
0.8840 -0.9305 -0.3885 -0.0475 0.2668
-0.4931 0.0636 -40.6025
76.5600 -42.5400 0.1854 0.1918 -
0.5826 -0.9763 -0.5003 -0.1509 0.2466
-0.4915 0.0682 -40.6007
76.5800 -42.6400 -0.2046 0.3301 -
0.2330 -0.9771 -0.6024 -0.2535 0.2263
-0.4898 0.0729 -40.5989
76.6000 -42.1200 0.1531 0.3676
0.0996 -0.9380 -0.6937 -0.3550 0.2059
-0.4881 0.0776 -40.5972
76.6200 -42.2200 0.0419 0.2600
0.3701 -0.8653 -0.7730 -0.4551 0.1854
-0.4863 0.0823 -40.5954
76.6400 -42.3800 -0.0692 0.0639
0.5541 -0.7665 -0.8399 -0.5536 0.1650
-0.4844 0.0869 -40.5936
76.6600 -42.6400 -0.0757 -0.1281
0.6447 -0.6504 -0.8939 -0.6502 0.1444
-0.4825 0.0916 -40.5918
76.6800 -42.2400 0.3502 -0.1712
0.6409 -0.5257 -0.9349 -0.7446 0.1239
-0.4805 0.0962 -40.5901
76.7000 -43.1300 -0.6726 0.0137
0.5483 -0.3996 -0.9629 -0.8367 0.1034
-0.4784 0.1009 -40.5883
76.7200 -41.7000 0.5432 0.3335
0.3867 -0.2769 -0.9782 -0.9261 0.0829
-0.4763 0.1055 -40.5865
76.7400 -42.4900 0.0928 0.3363
0.1904 -0.1618 -0.9813 -1.0128 0.0624
-0.4741 0.1102 -40.5847
76.7600 -42.7400 0.3846 -0.1224
0.0029 -0.0575 -0.9728 -1.0964 0.0420
-0.4718 0.1148 -40.5830
76.7800 -44.5700 -1.0150 -0.4972 -
0.1305 0.0334 -0.9536 -1.1769 0.0217
-0.4695 0.1194 -40.5812
76.8000 -42.6600 0.9561 -0.4990 -
0.1782 0.1097 -0.9245 -1.2540 0.0014
-0.4671 0.1240 -40.5794
76.8200 -42.9700 0.3489 -0.2992 -
0.1455 0.1730 -0.8869 -1.3276 -0.0187
-0.4647 0.1286 -40.5777
76.8400 -44.0300 -1.0405 0.0419 -
0.0715 0.2266 -0.8421 -1.3975 -0.0388
-0.4622 0.1332 -40.5759
76.8600 -42.3100 0.2655 0.4815 -
0.0085 0.2737 -0.7913 -1.4635 -0.0586
-0.4597 0.1378 -40.5741

76.8800 -41.3500 0.7939 0.6803
0.0080 0.3168 -0.7361 -1.5255 -0.0784
-0.4570 0.1423 -40.5723
76.9000 -42.8700 -0.3197 0.3441 -
0.0295 0.3568 -0.6778 -1.5834 -0.0979
-0.4544 0.1469 -40.5706
76.9200 -42.8200 0.4258 -0.3140 -
0.0905 0.3930 -0.6176 -1.6369 -0.1173
-0.4516 0.1514 -40.5688
76.9400 -44.2200 -0.7632 -0.6200 -
0.1288 0.4246 -0.5568 -1.6860 -0.1365
-0.4488 0.1560 -40.5670
76.9600 -42.5900 0.7384 -0.3945 -
0.1066 0.4513 -0.4967 -1.7305 -0.1555
-0.4460 0.1605 -40.5652
76.9800 -43.3800 -0.5729 -0.0263 -
0.0252 0.4726 -0.4382 -1.7704 -0.1742
-0.4431 0.1651 -40.5635
77.0000 -41.9800 0.3502 0.2556
0.0851 0.4879 -0.3825 -1.8055 -0.1927
-0.4401 0.1696 -40.5617
77.0200 -42.2100 0.0004 0.3652
0.1798 0.4966 -0.3306 -1.8356 -0.2109
-0.4371 0.1741 -40.5599
77.0400 -42.4100 -0.1754 0.2832
0.2171 0.4987 -0.2832 -1.8608 -0.2288
-0.4340 0.1786 -40.5582
77.0600 -42.0000 0.2739 0.1335
0.1768 0.4942 -0.2413 -1.8810 -0.2465
-0.4309 0.1831 -40.5564
77.0800 -42.6500 -0.1030 0.0393
0.0703 0.4841 -0.2054 -1.8961 -0.2639
-0.4277 0.1876 -40.5546
77.1000 -43.2500 -0.3697 0.0069 -
0.0702 0.4698 -0.1760 -1.9060 -0.2810
-0.4244 0.1921 -40.5528
77.1200 -42.3300 0.5063 -0.0330 -
0.2045 0.4525 -0.1533 -1.9108 -0.2978
-0.4211 0.1966 -40.5511
77.1400 -43.5100 -0.4088 -0.1259 -
0.2915 0.4335 -0.1373 -1.9107 -0.3142
-0.4178 0.2010 -40.5493
77.1600 -43.3400 -0.1606 -0.1572 -
0.3000 0.4134 -0.1279 -1.9055 -0.3304
-0.4144 0.2055 -40.5475
77.1800 -42.4900 0.4481 -0.1568 -
0.2198 0.3912 -0.1246 -1.8957 -0.3463
-0.4109 0.2099 -40.5457
77.2000 -43.2900 -0.3040 -0.1714 -
0.0675 0.3647 -0.1267 -1.8811 -0.3618
-0.4074 0.2144 -40.5440

77.2200	-42.9600	-0.1238	-0.1365	
0.1178	0.3319	-0.1333	-1.8622	-0.3771
	-0.4038	0.2188	-40.5422	
77.2400	-42.0400	0.4673	0.0040	
0.2862	0.2907	-0.1438	-1.8391	-0.3920
	-0.4002	0.2232	-40.5404	
77.2600	-42.8400	-0.5858	0.2578	
0.3875	0.2397	-0.1572	-1.8120	-0.4066
	-0.3965	0.2276	-40.5387	
77.2800	-41.7000	0.4502	0.4863	
0.3863	0.1789	-0.1727	-1.7810	-0.4210
	-0.3928	0.2320	-40.5369	
77.3000	-42.4000	-0.0050	0.3723	
0.2784	0.1095	-0.1895	-1.7466	-0.4350
	-0.3890	0.2364	-40.5351	
77.3200	-42.5800	0.5583	-0.2117	
0.1021	0.0344	-0.2069	-1.7088	-0.4487
	-0.3852	0.2408	-40.5333	
77.3400	-44.5900	-0.7838	-0.6453	-
0.0788	-0.0431	-0.2239	-1.6678	-0.4621
	-0.3813	0.2451	-40.5316	
77.3600	-43.1800	0.4616	-0.3475	-
0.2120	-0.1192	-0.2399	-1.6240	-0.4752
	-0.3774	0.2495	-40.5298	
77.3800	-43.0300	0.1952	0.2402	-
0.2856	-0.1905	-0.2539	-1.5775	-0.4880
	-0.3734	0.2539	-40.5280	
77.4000	-43.2100	-0.1772	0.4473	-
0.3055	-0.2539	-0.2651	-1.5286	-0.5005
	-0.3694	0.2582	-40.5263	
77.4200	-42.6700	0.7070	0.0739	-
0.2837	-0.3073	-0.2726	-1.4775	-0.5126
	-0.3653	0.2625	-40.5245	
77.4400	-44.6100	-1.0495	-0.2897	-
0.2266	-0.3498	-0.2758	-1.4244	-0.5245
	-0.3612	0.2668	-40.5227	
77.4600	-42.8600	0.7611	-0.2305	-
0.1308	-0.3828	-0.2739	-1.3696	-0.5361
	-0.3571	0.2712	-40.5209	
77.4800	-42.7000	0.5893	-0.0850	
0.0023	-0.4089	-0.2662	-1.3133	-0.5473
	-0.3529	0.2755	-40.5192	
77.5000	-43.7500	-0.7821	-0.0300	
0.1573	-0.4311	-0.2522	-1.2556	-0.5583
	-0.3486	0.2797	-40.5174	
77.5200	-42.4100	0.2949	0.0503	
0.2959	-0.4515	-0.2314	-1.1969	-0.5690
	-0.3443	0.2840	-40.5156	
77.5400	-42.3500	-0.0489	0.2178	
0.3729	-0.4709	-0.2038	-1.1373	-0.5793
	-0.3400	0.2883	-40.5138	

77.5600 -42.3800 -0.1872 0.2638
0.3634 -0.4892 -0.1693 -1.0769 -0.5894
-0.3356 0.2926 -40.5121
77.5800 -41.9700 0.4402 0.0860
0.2737 -0.5050 -0.1286 -1.0159 -0.5991
-0.3312 0.2968 -40.5103
77.6000 -43.1500 -0.4730 -0.1799
0.1352 -0.5159 -0.0822 -0.9545 -0.6086
-0.3267 0.3010 -40.5085
77.6200 -42.5700 0.2869 -0.2502 -
0.0140 -0.5189 -0.0309 -0.8926 -0.6177
-0.3222 0.3053 -40.5068
77.6400 -42.9200 -0.1223 -0.1327 -
0.1428 -0.5109 0.0243 -0.8305 -0.6265
-0.3177 0.3095 -40.5050
77.6600 -42.4500 0.0496 0.0065 -
0.2349 -0.4887 0.0827 -0.7683 -0.6351
-0.3131 0.3137 -40.5032
77.6800 -42.3600 0.2148 0.0265 -
0.2842 -0.4492 0.1433 -0.7060 -0.6433
-0.3084 0.3179 -40.5014
77.7000 -42.7100 -0.3672 -0.0070 -
0.3019 -0.3893 0.2052 -0.6438 -0.6512
-0.3038 0.3221 -40.4997
77.7200 -41.6800 0.3542 0.0476 -
0.3063 -0.3074 0.2674 -0.5818 -0.6589
-0.2991 0.3263 -40.4979
77.7400 -42.0400 -0.3975 0.2244 -
0.3087 -0.2042 0.3291 -0.5201 -0.6662
-0.2943 0.3304 -40.4961
77.7600 -41.0300 0.1275 0.3328 -
0.3081 -0.0829 0.3892 -0.4588 -0.6732
-0.2896 0.3346 -40.4944
77.7800 -40.7300 0.4099 0.1281 -
0.2903 0.0517 0.4469 -0.3980 -0.6800
-0.2848 0.3387 -40.4926
77.8000 -41.2600 -0.0026 -0.3342 -
0.2375 0.1939 0.5013 -0.3378 -0.6864
-0.2799 0.3428 -40.4908
77.8200 -41.8600 -0.7252 -0.5048 -
0.1357 0.3374 0.5515 -0.2784 -0.6925
-0.2750 0.3470 -40.4890
77.8400 -39.7900 0.5171 -0.0923
0.0140 0.4758 0.5966 -0.2198 -0.6983
-0.2701 0.3511 -40.4873
77.8600 -39.5500 -0.0488 0.3465
0.1804 0.6020 0.6356 -0.1622 -0.7038
-0.2652 0.3552 -40.4855
77.8800 -39.0100 0.1367 0.3628
0.3233 0.7093 0.6678 -0.1056 -0.7091
-0.2602 0.3592 -40.4837

77.9000 −39.2400 −0.2284 0.1239
0.4142 0.7909 0.6921 −0.0502 −0.7140
−0.2552 0.3633 −40.4820
77.9200 −38.8300 0.2296 −0.0964
0.4499 0.8414 0.7076 0.0039 −0.7186
−0.2501 0.3674 −40.4802
77.9400 −39.6400 −0.4625 −0.1831
0.4448 0.8576 0.7138 0.0566 −0.7229
−0.2451 0.3714 −40.4784
77.9600 −38.5800 0.5612 −0.1329
0.4193 0.8378 0.7104 0.1079 −0.7269
−0.2400 0.3754 −40.4766
77.9800 −39.5400 −0.4780 −0.0154
0.3821 0.7825 0.6975 0.1576 −0.7307
−0.2348 0.3795 −40.4749
78.0000 −38.9400 0.0641 0.1205
0.3336 0.6947 0.6759 0.2056 −0.7341
−0.2297 0.3835 −40.4731
78.0200 −38.6400 0.5400 0.1404
0.2693 0.5800 0.6462 0.2521 −0.7372
−0.2245 0.3875 −40.4713
78.0400 −39.7000 −0.1922 −0.0058
0.1838 0.4447 0.6094 0.2968 −0.7400
−0.2193 0.3915 −40.4696
78.0600 −39.9700 −0.1347 −0.1522
0.0783 0.2952 0.5663 0.3398 −0.7426
−0.2140 0.3954 −40.4678
78.0800 −40.2600 −0.0382 −0.1645 −
0.0398 0.1381 0.5179 0.3811 −0.7448
−0.2088 0.3994 −40.4660
78.1000 −40.4100 0.0156 −0.0752 −
0.1570 −0.0197 0.4650 0.4206 −0.7467
−0.2035 0.4034 −40.4642
78.1200 −40.2600 0.3157 0.0560 −
0.2587 −0.1716 0.4085 0.4584 −0.7483
−0.1982 0.4073 −40.4625
78.1400 −41.0700 −0.4260 0.1840 −
0.3342 −0.3110 0.3492 0.4944 −0.7497
−0.1928 0.4112 −40.4607
78.1600 −40.8300 0.0145 0.1999 −
0.3746 −0.4319 0.2880 0.5287 −0.7507
−0.1875 0.4151 −40.4589
78.1800 −40.4700 0.6325 0.0175 −
0.3737 −0.5294 0.2258 0.5612 −0.7515
−0.1821 0.4190 −40.4572
78.2000 −42.0700 −0.7691 −0.2341 −
0.3280 −0.6000 0.1634 0.5919 −0.7520
−0.1767 0.4229 −40.4554
78.2200 −40.6500 0.6992 −0.2085 −
0.2484 −0.6430 0.1017 0.6209 −0.7522
−0.1712 0.4268 −40.4536

78.2400 -41.5900 -0.4685 -0.0215 -
0.1588 -0.6587 0.0413 0.6482 -0.7521
-0.1658 0.4306 -40.4518
78.2600 -40.6200 0.2358 0.1135 -
0.0887 -0.6488 -0.0169 0.6737 -0.7517
-0.1603 0.4345 -40.4501
78.2800 -40.6900 0.0875 0.2206 -
0.0596 -0.6161 -0.0725 0.6975 -0.7510
-0.1548 0.4383 -40.4483
78.3000 -41.0400 -0.2577 0.2310 -
0.0709 -0.5647 -0.1246 0.7196 -0.7501
-0.1493 0.4421 -40.4465
78.3200 -40.4400 0.4379 0.0539 -
0.0956 -0.5003 -0.1727 0.7399 -0.7489
-0.1437 0.4460 -40.4448
78.3400 -41.5100 -0.4058 -0.2079 -
0.0990 -0.4289 -0.2163 0.7587 -0.7474
-0.1382 0.4498 -40.4430
78.3600 -41.1500 -0.0634 -0.2820 -
0.0548 -0.3558 -0.2548 0.7757 -0.7457
-0.1326 0.4535 -40.4412
78.3800 -40.3400 0.3723 -0.1613
0.0339 -0.2860 -0.2876 0.7911 -0.7436
-0.1270 0.4573 -40.4395
78.4000 -40.9300 -0.4404 0.0024
0.1426 -0.2233 -0.3142 0.8049 -0.7414
-0.1214 0.4611 -40.4377
78.4200 -39.6700 0.5110 0.1159
0.2411 -0.1709 -0.3342 0.8171 -0.7388
-0.1158 0.4648 -40.4359
78.4400 -40.3500 -0.4260 0.2232
0.3022 -0.1320 -0.3470 0.8277 -0.7360
-0.1101 0.4685 -40.4341
78.4600 -39.7100 0.1591 0.2689
0.3120 -0.1089 -0.3523 0.8368 -0.7330
-0.1045 0.4722 -40.4324
78.4800 -39.6600 0.4109 0.1141
0.2751 -0.1024 -0.3501 0.8444 -0.7297
-0.0988 0.4759 -40.4306
78.5000 -40.8300 -0.4231 -0.1593
0.2075 -0.1116 -0.3403 0.8505 -0.7261
-0.0931 0.4796 -40.4288
78.5200 -40.7700 -0.1946 -0.2740
0.1315 -0.1339 -0.3235 0.8552 -0.7223
-0.0874 0.4833 -40.4271
78.5400 -39.9600 0.4805 -0.1604
0.0626 -0.1657 -0.3002 0.8584 -0.7183
-0.0817 0.4869 -40.4253
78.5600 -40.7100 -0.3505 -0.0051
0.0041 -0.2026 -0.2712 0.8602 -0.7140
-0.0759 0.4906 -40.4235

78.5800 -40.3000 0.0408 0.1442 -
0.0502 -0.2397 -0.2372 0.8606 -0.7094
-0.0702 0.4942 -40.4218
78.6000 -40.1000 0.1144 0.2335 -
0.1068 -0.2722 -0.1990 0.8596 -0.7047
-0.0644 0.4978 -40.4200
78.6200 -40.2700 0.0575 0.1922 -
0.1673 -0.2957 -0.1574 0.8574 -0.6997
-0.0586 0.5014 -40.4182
78.6400 -40.7400 -0.3171 0.0596 -
0.2246 -0.3067 -0.1132 0.8538 -0.6945
-0.0529 0.5050 -40.4165
78.6600 -40.1400 0.2484 -0.0948 -
0.2587 -0.3029 -0.0672 0.8489 -0.6890
-0.0471 0.5086 -40.4147
78.6800 -40.4300 0.1025 -0.2457 -
0.2512 -0.2838 -0.0200 0.8428 -0.6834
-0.0412 0.5121 -40.4129
78.7000 -40.6300 -0.2241 -0.2954 -
0.1948 -0.2504 0.0277 0.8355 -0.6775
-0.0354 0.5157 -40.4111
78.7200 -40.3200 -0.2683 -0.0903 -
0.0982 -0.2052 0.0753 0.8270 -0.6714
-0.0296 0.5192 -40.4094
78.7400 -39.0400 0.4061 0.2164
0.0118 -0.1514 0.1224 0.8174 -0.6650
-0.0237 0.5227 -40.4076
78.7600 -39.3200 -0.1412 0.3254
0.1070 -0.0927 0.1685 0.8066 -0.6585
-0.0179 0.5262 -40.4058
78.7800 -39.1100 0.0690 0.1591
0.1697 -0.0321 0.2130 0.7947 -0.6518
-0.0120 0.5297 -40.4041
78.8000 -39.5400 -0.1258 -0.1043
0.1966 0.0279 0.2557 0.7818 -0.6449
-0.0061 0.5331 -40.4023
78.8200 -39.2200 0.1740 -0.2530
0.1934 0.0851 0.2958 0.7679 -0.6378
-0.0003 0.5366 -40.4005
78.8400 -39.4700 -0.2881 -0.1607
0.1660 0.1380 0.3331 0.7530 -0.6305
0.0056 0.5400 -40.3988
78.8600 -38.7900 0.1919 0.0838
0.1213 0.1854 0.3668 0.7371 -0.6231
0.0115 0.5434 -40.3970
78.8800 -38.4900 0.3004 0.2178
0.0678 0.2268 0.3966 0.7203 -0.6155
0.0175 0.5468 -40.3952
78.9000 -39.0000 -0.0534 0.0663
0.0142 0.2619 0.4220 0.7027 -0.6077
0.0234 0.5502 -40.3935

78.9200 -39.6300 -0.3564 -0.2090 -
0.0310 0.2914 0.4424 0.6842 -0.5997
0.0293 0.5536 -40.3917
78.9400 -39.3600 -0.0926 -0.2668 -
0.0666 0.3165 0.4575 0.6649 -0.5916
0.0352 0.5569 -40.3899
78.9600 -38.6500 0.2260 -0.0358 -
0.0981 0.3383 0.4671 0.6449 -0.5834
0.0412 0.5603 -40.3882
78.9800 -38.9100 -0.2178 0.2746 -
0.1340 0.3578 0.4708 0.6242 -0.5750
0.0471 0.5636 -40.3864
79.0000 -38.4700 0.3247 0.4032 -
0.1791 0.3750 0.4686 0.6028 -0.5665
0.0531 0.5669 -40.3846
79.0200 -39.1000 -0.1922 0.2301 -
0.2245 0.3888 0.4606 0.5808 -0.5579
0.0591 0.5702 -40.3829
79.0400 -39.0800 0.1011 -0.1215 -
0.2463 0.3974 0.4470 0.5582 -0.5491
0.0650 0.5735 -40.3811
79.0600 -39.6900 -0.2019 -0.3742 -
0.2197 0.3989 0.4279 0.5351 -0.5403
0.0710 0.5767 -40.3793
79.0800 -39.5000 0.1331 -0.4316 -
0.1276 0.3915 0.4038 0.5115 -0.5313
0.0770 0.5800 -40.3776
79.1000 -39.2300 0.1230 -0.3702
0.0262 0.3739 0.3751 0.4876 -0.5222
0.0830 0.5832 -40.3758
79.1200 -39.4700 -0.2986 -0.2160
0.2144 0.3452 0.3422 0.4633 -0.5131
0.0890 0.5864 -40.3740
79.1400 -38.4200 0.2681 0.0275
0.3924 0.3049 0.3056 0.4387 -0.5038
0.0950 0.5896 -40.3723
79.1600 -38.5100 -0.0401 0.2986
0.5113 0.2521 0.2660 0.4139 -0.4945
0.1010 0.5928 -40.3705
79.1800 -38.6600 -0.4378 0.5100
0.5322 0.1870 0.2240 0.3889 -0.4851
0.1070 0.5959 -40.3687
79.2000 -37.8800 0.5098 0.5405
0.4372 0.1111 0.1802 0.3637 -0.4756
0.1130 0.5991 -40.3670
79.2200 -38.9800 0.0340 0.2627
0.2425 0.0288 0.1353 0.3385 -0.4660
0.1190 0.6022 -40.3652
79.2400 -40.2400 -0.3132 -0.1815 -
0.0028 -0.0549 0.0899 0.3133 -0.4564
0.1250 0.6053 -40.3634

79.2600 -40.6800 -0.0365 -0.4760 -
0.2409 -0.1346 0.0446 0.2880 -0.4468
0.1310 0.6084 -40.3617
79.2800 -41.0100 -0.0850 -0.3958 -
0.4181 -0.2049 0.0002 0.2629 -0.4371
0.1371 0.6115 -40.3599
79.3000 -41.0400 -0.4327 -0.0803 -
0.4976 -0.2607 -0.0427 0.2379 -0.4273
0.1431 0.6146 -40.3581
79.3200 -39.6000 0.9184 0.0823 -
0.4738 -0.2985 -0.0834 0.2130 -0.4175
0.1491 0.6176 -40.3564
79.3400 -41.5500 -1.0028 0.0024 -
0.3673 -0.3169 -0.1216 0.1884 -0.4077
0.1551 0.6206 -40.3546
79.3600 -40.0000 0.4464 0.0122 -
0.2130 -0.3166 -0.1566 0.1640 -0.3978
0.1612 0.6236 -40.3528
79.3800 -39.9900 0.4404 0.0268 -
0.0409 -0.2999 -0.1883 0.1399 -0.3879
0.1672 0.6266 -40.3511
79.4000 -40.6800 -0.3491 -0.0818
0.1233 -0.2698 -0.2163 0.1162 -0.3780
0.1732 0.6296 -40.3493
79.4200 -39.9400 0.0289 -0.0961
0.2518 -0.2298 -0.2405 0.0929 -0.3681
0.1792 0.6325 -40.3475
79.4400 -39.9300 -0.1359 0.1197
0.3224 -0.1836 -0.2609 0.0700 -0.3582
0.1853 0.6355 -40.3458
79.4600 -39.4200 0.2557 0.2751
0.3240 -0.1350 -0.2774 0.0476 -0.3483
0.1913 0.6384 -40.3440
79.4800 -39.8900 0.0050 0.1795
0.2638 -0.0870 -0.2900 0.0257 -0.3383
0.1973 0.6413 -40.3422
79.5000 -40.2800 -0.3070 -0.0372
0.1663 -0.0426 -0.2987 0.0044 -0.3284
0.2033 0.6442 -40.3405
79.5200 -40.0700 0.1857 -0.2028
0.0636 -0.0042 -0.3035 -0.0164 -0.3185
0.2094 0.6471 -40.3387
79.5400 -39.9700 0.3809 -0.2770 -
0.0168 0.0263 -0.3046 -0.0366 -0.3086
0.2154 0.6499 -40.3369
79.5600 -40.7800 -0.4489 -0.2428 -
0.0607 0.0478 -0.3021 -0.0562 -0.2987
0.2214 0.6527 -40.3352
79.5800 -40.3400 -0.2562 -0.0291 -
0.0732 0.0603 -0.2962 -0.0751 -0.2888
0.2274 0.6556 -40.3334

79.6000 -39.5900 0.2356 0.2651 -
0.0670 0.0651 -0.2871 -0.0933 -0.2790
0.2334 0.6584 -40.3316
79.6200 -39.0000 0.6747 0.2942 -
0.0616 0.0641 -0.2752 -0.1108 -0.2691
0.2394 0.6611 -40.3299
79.6400 -39.8500 0.2172 -0.0512 -
0.0694 0.0595 -0.2607 -0.1276 -0.2594
0.2454 0.6639 -40.3281
79.6600 -40.7600 -0.2942 -0.3510 -
0.0831 0.0530 -0.2438 -0.1437 -0.2496
0.2514 0.6666 -40.3264
79.6800 -41.1900 -0.9761 -0.1639 -
0.0908 0.0464 -0.2249 -0.1591 -0.2399
0.2574 0.6694 -40.3246
79.7000 -38.8200 1.0985 0.2391 -
0.0858 0.0410 -0.2042 -0.1738 -0.2303
0.2634 0.6721 -40.3228
79.7200 -40.2800 -0.5319 0.2525 -
0.0658 0.0371 -0.1819 -0.1877 -0.2207
0.2693 0.6747 -40.3211
79.7400 -40.3400 -0.4016 0.0091 -
0.0372 0.0346 -0.1585 -0.2010 -0.2111
0.2753 0.6774 -40.3193
79.7600 -40.0100 0.0088 -0.0809 -
0.0099 0.0331 -0.1340 -0.2135 -0.2016
0.2813 0.6801 -40.3175
79.7800 -39.8700 0.0254 -0.0057
0.0140 0.0324 -0.1087 -0.2252 -0.1922
0.2872 0.6827 -40.3158
79.8000 -39.3500 0.4814 -0.0149
0.0411 0.0320 -0.0829 -0.2363 -0.1828
0.2932 0.6853 -40.3140
79.8200 -40.0900 -0.2202 -0.1492
0.0738 0.0309 -0.0569 -0.2467 -0.1735
0.2991 0.6879 -40.3122
79.8400 -40.1800 -0.3741 -0.1665
0.1077 0.0287 -0.0308 -0.2564 -0.1642
0.3050 0.6905 -40.3105
79.8600 -39.0000 0.3481 0.0184
0.1315 0.0256 -0.0049 -0.2655 -0.1550
0.3110 0.6930 -40.3087
79.8800 -39.1000 0.2317 0.2084
0.1303 0.0218 0.0203 -0.2738 -0.1459
0.3169 0.6956 -40.3070
79.9000 -39.7600 -0.4202 0.2326
0.0954 0.0183 0.0447 -0.2816 -0.1369
0.3228 0.6981 -40.3052
79.9200 -39.1400 0.3395 0.0785
0.0341 0.0160 0.0680 -0.2887 -0.1279
0.3287 0.7006 -40.3034

79.9400	-39.8400	-0.1767	-0.1272	-
0.0333	0.0160	0.0897	-0.2951	-0.1190
	0.3345	0.7031	-40.3017	
79.9600	-39.9200	-0.0804	-0.2506	-
0.0869	0.0192	0.1095	-0.3009	-0.1102
	0.3404	0.7055	-40.2999	
79.9800	-39.7200	0.1603	-0.1823	-
0.1151	0.0262	0.1272	-0.3061	-0.1014
	0.3463	0.7080	-40.2981	
80.0000	-39.7100	-0.2338	0.0349	-
0.1161	0.0372	0.1424	-0.3107	-0.0928
	0.3521	0.7104	-40.2964	
80.0200	-39.2100	0.1931	0.2179	-
0.0989	0.0518	0.1549	-0.3146	-0.0842
	0.3579	0.7128	-40.2946	
80.0400	-39.1300	0.0510	0.2040	-
0.0733	0.0692	0.1647	-0.3179	-0.0757
	0.3638	0.7152	-40.2929	
80.0600	-39.3200	0.0786	0.0090	-
0.0450	0.0878	0.1714	-0.3206	-0.0672
	0.3696	0.7176	-40.2911	
80.0800	-40.0300	-0.4827	-0.1263	-
0.0105	0.1055	0.1751	-0.3228	-0.0589
	0.3754	0.7199	-40.2893	
80.1000	-39.1600	0.2253	-0.0933	
0.0382	0.1203	0.1757	-0.3243	-0.0506
	0.3812	0.7222	-40.2876	
80.1200	-38.8600	0.3674	-0.0669	
0.1017	0.1297	0.1732	-0.3253	-0.0424
	0.3869	0.7246	-40.2858	
80.1400	-39.3600	-0.0911	-0.1495	
0.1677	0.1317	0.1676	-0.3258	-0.0342
	0.3927	0.7268	-40.2841	
80.1600	-39.5900	-0.5318	-0.0954	
0.2097	0.1253	0.1592	-0.3257	-0.0262
	0.3984	0.7291	-40.2823	
80.1800	-39.0400	-0.3539	0.2268	
0.2079	0.1096	0.1482	-0.3251	-0.0182
	0.4042	0.7314	-40.2805	
80.2000	-37.7900	0.9128	0.4224	
0.1553	0.0851	0.1349	-0.3241	-0.0103
	0.4099	0.7336	-40.2788	
80.2200	-39.9100	-0.8026	0.1796	
0.0606	0.0532	0.1198	-0.3225	-0.0024
	0.4156	0.7358	-40.2770	
80.2400	-39.5400	0.0461	-0.1729	-
0.0459	0.0165	0.1032	-0.3206	0.0053
	0.4212	0.7380	-40.2753	
80.2600	-39.1600	0.5412	-0.3428	-
0.1317	-0.0213	0.0857	-0.3182	0.0131
	0.4269	0.7402	-40.2735	

80.2800	-40.3500	-0.5100	-0.2659	-
0.1747	-0.0562	0.0677	-0.3154	0.0207
	0.4325	0.7423	-40.2717	
80.3000	-39.5500	-0.0449	-0.0091	-
0.1772	-0.0847	0.0497	-0.3122	0.0283
	0.4381	0.7445	-40.2700	
80.3200	-39.1000	0.1836	0.2599	-
0.1562	-0.1040	0.0321	-0.3087	0.0358
	0.4437	0.7466	-40.2682	
80.3400	-38.7300	0.5138	0.2416	-
0.1343	-0.1119	0.0153	-0.3048	0.0433
	0.4493	0.7487	-40.2665	
80.3600	-40.0300	-0.5523	-0.0135	-
0.1311	-0.1079	-0.0004	-0.3005	0.0507
	0.4549	0.7507	-40.2647	
80.3800	-39.5100	0.0593	-0.0972	-
0.1417	-0.0931	-0.0147	-0.2960	0.0581
	0.4604	0.7528	-40.2629	
80.4000	-39.4000	0.0661	0.0145	-
0.1475	-0.0698	-0.0275	-0.2912	0.0654
	0.4659	0.7548	-40.2612	
80.4200	-39.3200	0.1202	0.0046	-
0.1273	-0.0414	-0.0385	-0.2861	0.0726
	0.4714	0.7568	-40.2594	
80.4400	-39.4600	-0.0496	-0.1574	-
0.0683	-0.0120	-0.0477	-0.2807	0.0799
	0.4769	0.7588	-40.2577	
80.4600	-39.4400	-0.1966	-0.1779	
0.0260	0.0140	-0.0550	-0.2751	0.0870
	0.4823	0.7608	-40.2559	
80.4800	-38.7900	0.1872	0.0307	
0.1333	0.0331	-0.0604	-0.2693	0.0942
	0.4877	0.7627	-40.2542	
80.5000	-38.7300	0.0074	0.2195	
0.2221	0.0433	-0.0638	-0.2633	0.1012
	0.4931	0.7647	-40.2524	
80.5200	-38.4300	0.0294	0.1963	
0.2684	0.0435	-0.0653	-0.2570	0.1083
	0.4985	0.7666	-40.2506	
80.5400	-38.9100	0.1042	-0.0536	
0.2642	0.0346	-0.0650	-0.2505	0.1153
	0.5038	0.7685	-40.2489	
80.5600	-38.8800	0.3599	-0.3246	
0.2127	0.0186	-0.0629	-0.2439	0.1223
	0.5091	0.7703	-40.2471	
80.5800	-40.1100	-0.9027	-0.2158	
0.1194	-0.0018	-0.0593	-0.2370	0.1293
	0.5144	0.7722	-40.2454	
80.6000	-38.0400	0.7835	0.2192	-
0.0017	-0.0239	-0.0544	-0.2299	0.1362
	0.5196	0.7740	-40.2436	

80.6200 -38.4200 0.2940 0.3700 -
0.1287 -0.0443 -0.0484 -0.2227 0.1431
0.5248 0.7758 -40.2418
80.6400 -39.9900 -0.6742 0.0905 -
0.2384 -0.0601 -0.0415 -0.2153 0.1500
0.5300 0.7776 -40.2401
80.6600 -39.3300 0.4421 -0.2528 -
0.3038 -0.0691 -0.0340 -0.2078 0.1568
0.5351 0.7794 -40.2383
80.6800 -39.7500 -0.1644 -0.2984 -
0.3028 -0.0697 -0.0262 -0.2001 0.1637
0.5402 0.7811 -40.2366
80.7000 -39.2900 0.0104 -0.1064 -
0.2317 -0.0619 -0.0182 -0.1922 0.1705
0.5453 0.7828 -40.2348
80.7200 -39.0400 0.0456 0.1458 -
0.1153 -0.0469 -0.0105 -0.1842 0.1773
0.5503 0.7846 -40.2331
80.7400 -38.6600 0.0148 0.2336
0.0143 -0.0271 -0.0030 -0.1761 0.1841
0.5553 0.7862 -40.2313
80.7600 -38.5700 0.0078 0.0907
0.1280 -0.0050 0.0040 -0.1678 0.1909
0.5603 0.7879 -40.2296
80.7800 -38.7100 0.1095 -0.1113
0.2034 0.0168 0.0104 -0.1594 0.1976
0.5652 0.7895 -40.2278
80.8000 -39.2600 -0.4552 -0.1310
0.2322 0.0361 0.0160 -0.1508 0.2044
0.5701 0.7912 -40.2260
80.8200 -38.0100 0.4788 -0.0269
0.2167 0.0512 0.0207 -0.1422 0.2111
0.5749 0.7928 -40.2243
80.8400 -38.3600 0.0973 0.0052
0.1690 0.0609 0.0245 -0.1335 0.2179
0.5797 0.7943 -40.2225
80.8600 -38.9100 -0.3862 0.0002
0.1033 0.0645 0.0271 -0.1246 0.2246
0.5844 0.7959 -40.2208
80.8800 -38.6400 -0.0608 0.0945
0.0288 0.0618 0.0285 -0.1156 0.2314
0.5892 0.7974 -40.2190
80.9000 -38.1500 0.2860 0.1852 -
0.0448 0.0538 0.0288 -0.1066 0.2381
0.5938 0.7989 -40.2173
80.9200 -38.5700 -0.0682 0.1124 -
0.1043 0.0416 0.0279 -0.0975 0.2448
0.5984 0.8004 -40.2155
80.9400 -38.8400 -0.0483 -0.0781 -
0.1376 0.0269 0.0258 -0.0883 0.2515
0.6030 0.8019 -40.2138

80.9600 -38.6600 0.1787 -0.2579 -
0.1359 0.0114 0.0226 -0.0790 0.2583
0.6075 0.8033 -40.2120
80.9800 -39.1100 -0.1804 -0.2870 -
0.1000 -0.0031 0.0185 -0.0697 0.2650
0.6120 0.8048 -40.2103
81.0000 -38.7500 -0.1234 -0.0782 -
0.0422 -0.0154 0.0135 -0.0604 0.2717
0.6164 0.8062 -40.2085
81.0200 -38.3300 0.0165 0.2345
0.0144 -0.0244 0.0078 -0.0511 0.2785
0.6208 0.8076 -40.2067
81.0400 -38.0800 0.0580 0.3448
0.0434 -0.0294 0.0015 -0.0418 0.2852
0.6251 0.8089 -40.2050
81.0600 -37.6600 0.7065 0.1139
0.0304 -0.0304 -0.0052 -0.0325 0.2920
0.6294 0.8103 -40.2032
81.0800 -39.7400 -0.9455 -0.1830 -
0.0161 -0.0274 -0.0122 -0.0233 0.2987
0.6336 0.8116 -40.2015
81.1000 -38.3200 0.4398 -0.1798 -
0.0743 -0.0205 -0.0193 -0.0141 0.3055
0.6378 0.8129 -40.1997
81.1200 -38.0900 0.6257 -0.0393 -
0.1179 -0.0104 -0.0263 -0.0050 0.3122
0.6419 0.8142 -40.1980
81.1400 -38.7600 -0.0925 -0.0030 -
0.1274 0.0021 -0.0331 0.0040 0.3190
0.6460 0.8154 -40.1962
81.1600 -39.0200 -0.6683 -0.0020 -
0.0959 0.0154 -0.0395 0.0129 0.3257
0.6500 0.8166 -40.1945
81.1800 -38.0300 0.4129 0.0299 -
0.0316 0.0283 -0.0453 0.0216 0.3325
0.6539 0.8178 -40.1927
81.2000 -38.1700 0.1866 0.0094
0.0451 0.0396 -0.0503 0.0302 0.3393
0.6578 0.8190 -40.1910
81.2200 -38.3100 -0.0145 -0.0809
0.1116 0.0478 -0.0544 0.0386 0.3461
0.6616 0.8202 -40.1892
81.2400 -38.4000 -0.2247 -0.0681
0.1505 0.0520 -0.0575 0.0467 0.3529
0.6654 0.8213 -40.1875
81.2600 -38.2800 -0.0635 0.0741
0.1541 0.0511 -0.0594 0.0546 0.3597
0.6691 0.8225 -40.1857
81.2800 -37.8000 0.2408 0.1894
0.1205 0.0450 -0.0601 0.0622 0.3665
0.6727 0.8236 -40.1840

81.3000 -38.1300 -0.0236 0.1369
0.0592 0.0342 -0.0593 0.0696 0.3733
0.6763 0.8246 -40.1822
81.3200 -38.3200 -0.0002 -0.0596 -
0.0081 0.0198 -0.0572 0.0765 0.3801
0.6798 0.8257 -40.1805
81.3400 -38.5800 -0.0480 -0.2184 -
0.0575 0.0028 -0.0536 0.0832 0.3869
0.6832 0.8267 -40.1787
81.3600 -38.6000 -0.1168 -0.1762 -
0.0714 -0.0158 -0.0486 0.0895 0.3938
0.6866 0.8277 -40.1770
81.3800 -38.2300 -0.0354 -0.0047 -
0.0501 -0.0349 -0.0421 0.0953 0.4006
0.6899 0.8287 -40.1752
81.4000 -37.8800 0.3227 0.0700 -
0.0058 -0.0532 -0.0341 0.1008 0.4075
0.6932 0.8297 -40.1735
81.4200 -38.3300 -0.1461 0.0185
0.0369 -0.0693 -0.0248 0.1058 0.4143
0.6963 0.8306 -40.1717
81.4400 -38.7000 -0.5408 0.0596
0.0519 -0.0821 -0.0142 0.1103 0.4212
0.6994 0.8316 -40.1700
81.4600 -37.3700 0.5322 0.2066
0.0292 -0.0906 -0.0025 0.1143 0.4281
0.7024 0.8325 -40.1682
81.4800 -38.0400 0.1151 0.1865 -
0.0217 -0.0938 0.0102 0.1178 0.4350
0.7054 0.8333 -40.1665
81.5000 -38.2400 -0.1292 -0.0358 -
0.0800 -0.0914 0.0236 0.1208 0.4418
0.7083 0.8342 -40.1647
81.5200 -38.5100 -0.1211 -0.2131 -
0.1212 -0.0835 0.0375 0.1232 0.4487
0.7111 0.8350 -40.1630
81.5400 -38.4400 -0.0552 -0.1786 -
0.1255 -0.0701 0.0517 0.1250 0.4557
0.7138 0.8358 -40.1612
81.5600 -38.0200 0.2063 -0.0155 -
0.0923 -0.0521 0.0659 0.1262 0.4626
0.7165 0.8366 -40.1595
81.5800 -37.9600 -0.0910 0.1091 -
0.0356 -0.0304 0.0799 0.1268 0.4695
0.7190 0.8374 -40.1577
81.6000 -37.4400 0.2759 0.1075
0.0240 -0.0059 0.0935 0.1267 0.4764
0.7215 0.8381 -40.1560
81.6200 -37.9600 -0.3531 0.0204
0.0637 0.0206 0.1064 0.1259 0.4834
0.7239 0.8388 -40.1542

81.6400 −37.6500 0.3228 −0.0229
0.0690 0.0484 0.1185 0.1245 0.4903
0.7263 0.8395 −40.1525
81.6600 −37.9700 −0.1935 0.0074
0.0388 0.0764 0.1294 0.1224 0.4973
0.7285 0.8402 −40.1507
81.6800 −37.6900 −0.0360 0.0986 −
0.0149 0.1034 0.1389 0.1195 0.5042
0.7307 0.8409 −40.1490
81.7000 −37.6400 −0.0690 0.1663 −
0.0658 0.1279 0.1469 0.1159 0.5112
0.7327 0.8415 −40.1472
81.7200 −37.1800 0.5480 0.0285 −
0.0871 0.1480 0.1531 0.1117 0.5182
0.7347 0.8421 −40.1455
81.7400 −38.3100 −0.4379 −0.2434 −
0.0641 0.1620 0.1573 0.1067 0.5251
0.7366 0.8427 −40.1437
81.7600 −38.0600 −0.1605 −0.3100
0.0061 0.1686 0.1594 0.1009 0.5321
0.7385 0.8432 −40.1420
81.7800 −37.6400 0.0749 −0.0784
0.1069 0.1666 0.1593 0.0945 0.5391
0.7402 0.8438 −40.1403
81.8000 −36.9700 0.1306 0.2272
0.2022 0.1556 0.1571 0.0874 0.5461
0.7418 0.8443 −40.1385
81.8200 −36.9100 0.0965 0.3266
0.2532 0.1353 0.1527 0.0795 0.5531
0.7434 0.8448 −40.1368
81.8400 −37.2300 −0.0211 0.1624
0.2376 0.1064 0.1462 0.0711 0.5602
0.7449 0.8452 −40.1350
81.8600 −37.8300 −0.0881 −0.0505
0.1613 0.0707 0.1378 0.0620 0.5672
0.7462 0.8457 −40.1333
81.8800 −37.7600 0.1148 −0.1301
0.0491 0.0305 0.1278 0.0523 0.5742
0.7475 0.8461 −40.1315
81.9000 −38.0200 −0.1219 −0.1031 −
0.0661 −0.0110 0.1163 0.0421 0.5813
0.7487 0.8465 −40.1298
81.9200 −38.1600 0.0507 −0.1021 −
0.1583 −0.0504 0.1036 0.0314 0.5883
0.7498 0.8469 −40.1280
81.9400 −38.0800 0.1394 −0.1176 −
0.2134 −0.0841 0.0899 0.0201 0.5954
0.7508 0.8472 −40.1263
81.9600 −38.7900 −0.5072 −0.0243 −
0.2328 −0.1088 0.0756 0.0085 0.6024
0.7517 0.8476 −40.1245

81.9800	-37.6100	0.5795	0.1569	-
0.2318	-0.1222	0.0608	-0.0035	0.6095
	0.7525	0.8479	-40.1228	
82.0000	-38.2400	-0.1714	0.1630	-
0.2251	-0.1229	0.0459	-0.0159	0.6166
	0.7532	0.8481	-40.1211	
82.0200	-38.4400	-0.2692	-0.0127	-
0.2136	-0.1110	0.0311	-0.0285	0.6237
	0.7538	0.8484	-40.1193	
82.0400	-37.9000	0.3336	-0.1627	-
0.1878	-0.0888	0.0166	-0.0414	0.6309
	0.7543	0.8486	-40.1176	
82.0600	-38.5700	-0.2040	-0.1594	-
0.1344	-0.0598	0.0026	-0.0544	0.6380
	0.7547	0.8489	-40.1158	
82.0800	-38.0000	-0.0393	-0.0233	-
0.0421	-0.0279	-0.0108	-0.0677	0.6452
	0.7550	0.8491	-40.1141	
82.1000	-37.4800	0.2671	0.0707	
0.0832	0.0029	-0.0234	-0.0810	0.6523
	0.7552	0.8492	-40.1123	
82.1200	-37.7600	0.0081	0.0294	
0.2168	0.0288	-0.0351	-0.0943	0.6595
	0.7553	0.8494	-40.1106	
82.1400	-38.1000	-0.3795	-0.0193	
0.3240	0.0469	-0.0458	-0.1076	0.6667
	0.7553	0.8495	-40.1089	
82.1600	-37.2000	0.4436	0.0399	
0.3723	0.0546	-0.0553	-0.1209	0.6739
	0.7552	0.8496	-40.1071	
82.1800	-37.7000	-0.1164	0.1588	
0.3443	0.0505	-0.0636	-0.1341	0.6812
	0.7550	0.8497	-40.1054	
82.2000	-37.8500	-0.3375	0.2313	
0.2398	0.0355	-0.0708	-0.1471	0.6884
	0.7547	0.8497	-40.1036	
82.2200	-37.2400	0.5393	0.1311	
0.0802	0.0124	-0.0766	-0.1599	0.6957
	0.7543	0.8498	-40.1019	
82.2400	-38.3300	-0.0762	-0.1411	-
0.0922	-0.0152	-0.0812	-0.1725	0.7030
	0.7538	0.8498	-40.1002	
82.2600	-39.1800	-0.4334	-0.3341	-
0.2280	-0.0433	-0.0846	-0.1848	0.7103
	0.7532	0.8497	-40.0984	
82.2800	-38.5600	0.0380	-0.2438	-
0.2890	-0.0681	-0.0867	-0.1967	0.7177
	0.7524	0.8497	-40.0967	
82.3000	-38.2300	0.2753	-0.0587	-
0.2707	-0.0862	-0.0878	-0.2083	0.7251
	0.7516	0.8496	-40.0949	

82.3200 -38.2700 0.0794 0.0114 -
0.1936 -0.0957 -0.0878 -0.2195 0.7325
0.7507 0.8496 -40.0932
82.3400 -38.6100 -0.3609 0.0807 -
0.0943 -0.0956 -0.0869 -0.2303 0.7399
0.7496 0.8495 -40.0915
82.3600 -37.9400 0.0899 0.2504 -
0.0103 -0.0857 -0.0853 -0.2406 0.7473
0.7485 0.8493 -40.0897
82.3800 -37.5500 0.2696 0.3216
0.0407 -0.0665 -0.0830 -0.2503 0.7548
0.7472 0.8492 -40.0880
82.4000 -37.8900 0.0488 0.1237
0.0587 -0.0397 -0.0803 -0.2596 0.7623
0.7458 0.8490 -40.0862
82.4200 -38.2600 -0.0525 -0.2012
0.0583 -0.0082 -0.0773 -0.2682 0.7698
0.7443 0.8488 -40.0845
82.4400 -38.7600 -0.4784 -0.3174
0.0545 0.0252 -0.0741 -0.2763 0.7773
0.7427 0.8486 -40.0828
82.4600 -37.8400 0.2658 -0.1431
0.0561 0.0581 -0.0707 -0.2838 0.7849
0.7410 0.8483 -40.0810
82.4800 -37.6400 0.2759 0.0552
0.0658 0.0879 -0.0671 -0.2906 0.7925
0.7392 0.8481 -40.0793
82.5000 -38.1100 -0.3379 0.1116
0.0780 0.1133 -0.0635 -0.2967 0.8001
0.7373 0.8478 -40.0776
82.5200 -37.8200 -0.0324 0.0911
0.0791 0.1329 -0.0598 -0.3023 0.8077
0.7352 0.8475 -40.0758
82.5400 -37.3700 0.3733 0.0584
0.0556 0.1459 -0.0561 -0.3071 0.8154
0.7331 0.8471 -40.0741
82.5600 -38.3700 -0.5402 0.0504
0.0061 0.1520 -0.0524 -0.3113 0.8231
0.7308 0.8468 -40.0723
82.5800 -37.4300 0.4736 0.0685 -
0.0562 0.1511 -0.0488 -0.3148 0.8308
0.7284 0.8464 -40.0706
82.6000 -38.1600 -0.1886 0.0069 -
0.1089 0.1432 -0.0451 -0.3177 0.8386
0.7259 0.8460 -40.0689
82.6200 -38.2200 -0.0378 -0.1059 -
0.1292 0.1281 -0.0415 -0.3198 0.8463
0.7233 0.8456 -40.0671
82.6400 -38.0600 0.1799 -0.1691 -
0.1007 0.1057 -0.0378 -0.3214 0.8541
0.7206 0.8451 -40.0654

82.6600	-38.3400	-0.2791	-0.1115	-
0.0233	0.0764	-0.0340	-0.3222	0.8619
	0.7178	0.8447	-40.0637	
82.6800	-37.8900	-0.0591	0.0334	
0.0828	0.0408	-0.0301	-0.3225	0.8697
	0.7148	0.8442	-40.0619	
82.7000	-37.2900	0.4509	0.1171	
0.1835	0.0001	-0.0261	-0.3221	0.8776
	0.7118	0.8437	-40.0602	
82.7200	-37.4100	0.1407	0.0990	
0.2452	-0.0446	-0.0219	-0.3211	0.8854
	0.7086	0.8432	-40.0585	
82.7400	-38.4100	-0.8015	0.0996	
0.2474	-0.0913	-0.0175	-0.3195	0.8932
	0.7053	0.8426	-40.0567	
82.7600	-36.9900	0.6395	0.1863	
0.1853	-0.1377	-0.0129	-0.3174	0.9011
	0.7019	0.8420	-40.0550	
82.7800	-37.8300	0.0547	0.1147	
0.0787	-0.1806	-0.0081	-0.3148	0.9089
	0.6984	0.8414	-40.0533	
82.8000	-38.7200	-0.3364	-0.1496	-
0.0416	-0.2165	-0.0031	-0.3116	0.9168
	0.6948	0.8408	-40.0515	
82.8200	-38.5600	-0.0480	-0.3293	-
0.1457	-0.2424	0.0021	-0.3080	0.9246
	0.6911	0.8402	-40.0498	
82.8400	-38.5700	0.0472	-0.2440	-
0.2131	-0.2557	0.0076	-0.3039	0.9324
	0.6872	0.8395	-40.0481	
82.8600	-38.2300	0.1407	0.0135	-
0.2414	-0.2545	0.0134	-0.2994	0.9402
	0.6833	0.8388	-40.0463	
82.8800	-38.0700	-0.0110	0.2569	-
0.2430	-0.2375	0.0194	-0.2946	0.9480
	0.6792	0.8381	-40.0446	
82.9000	-38.0500	-0.1508	0.3508	-
0.2333	-0.2047	0.0257	-0.2893	0.9558
	0.6750	0.8374	-40.0429	
82.9200	-37.8500	0.2068	0.2255	-
0.2185	-0.1570	0.0320	-0.2838	0.9635
	0.6707	0.8367	-40.0411	
82.9400	-37.9700	0.1565	-0.0930	-
0.1930	-0.0967	0.0384	-0.2778	0.9712
	0.6663	0.8359	-40.0394	
82.9600	-38.6400	-0.3707	-0.3605	-
0.1485	-0.0275	0.0448	-0.2716	0.9789
	0.6618	0.8351	-40.0377	
82.9800	-38.1000	-0.0326	-0.3307	-
0.0795	0.0463	0.0509	-0.2651	0.9865
	0.6572	0.8343	-40.0359	

83.0000	-37.5600	0.1922	-0.0666	
0.0109	0.1203	0.0568	-0.2584	0.9940
	0.6525	0.8335	-40.0342	
83.0200	-37.3800	-0.0446	0.1922	
0.1110	0.1900	0.0622	-0.2513	1.0015
	0.6477	0.8326	-40.0325	
83.0400	-37.0100	-0.0273	0.2629	
0.2055	0.2511	0.0671	-0.2441	1.0089
	0.6428	0.8318	-40.0307	
83.0600	-36.7800	0.3592	0.1367	
0.2800	0.2992	0.0714	-0.2366	1.0163
	0.6378	0.8309	-40.0290	
83.0800	-37.2400	-0.1708	-0.0904	
0.3221	0.3308	0.0749	-0.2289	1.0235
	0.6326	0.8300	-40.0273	
83.1000	-37.3500	-0.3215	-0.1687	
0.3213	0.3436	0.0775	-0.2211	1.0307
	0.6274	0.8291	-40.0256	
83.1200	-36.7900	0.2968	-0.0056	
0.2756	0.3372	0.0793	-0.2130	1.0377
	0.6220	0.8281	-40.0238	
83.1400	-36.9000	-0.0134	0.1704	
0.1899	0.3126	0.0802	-0.2048	1.0447
	0.6166	0.8272	-40.0221	
83.1600	-37.0900	0.1276	0.1233	
0.0747	0.2714	0.0804	-0.1964	1.0515
	0.6110	0.8262	-40.0204	
83.1800	-37.4200	0.1429	-0.0966	-
0.0495	0.2164	0.0799	-0.1878	1.0582
	0.6054	0.8252	-40.0186	
83.2000	-38.3900	-0.5078	-0.2015	-
0.1542	0.1506	0.0790	-0.1791	1.0647
	0.5996	0.8241	-40.0169	
83.2200	-37.3000	0.3756	-0.0657	-
0.2115	0.0773	0.0777	-0.1702	1.0711
	0.5938	0.8231	-40.0152	
83.2400	-38.0400	-0.1637	0.0485	-
0.2082	0.0001	0.0763	-0.1611	1.0773
	0.5878	0.8220	-40.0135	
83.2600	-37.8700	-0.0969	0.0590	-
0.1503	-0.0771	0.0749	-0.1518	1.0834
	0.5818	0.8210	-40.0117	
83.2800	-37.6300	0.1493	0.0187	-
0.0627	-0.1504	0.0737	-0.1423	1.0892
	0.5756	0.8199	-40.0100	
83.3000	-37.7300	0.1682	-0.0641	
0.0267	-0.2157	0.0730	-0.1326	1.0949
	0.5694	0.8188	-40.0083	
83.3200	-38.1700	-0.3055	-0.0581	
0.0889	-0.2698	0.0728	-0.1227	1.1003
	0.5630	0.8176	-40.0066	

83.3400 -37.8500 -0.2504 0.1015
0.1029 -0.3097 0.0733 -0.1125 1.1056
0.5566 0.8165 -40.0048
83.3600 -36.9700 0.5922 0.2028
0.0627 -0.3336 0.0746 -0.1021 1.1105
0.5500 0.8153 -40.0031
83.3800 -38.4400 -0.5535 0.0947 -
0.0218 -0.3400 0.0767 -0.0914 1.1153
0.5434 0.8141 -40.0014
83.4000 -37.8000 0.2116 -0.0530 -
0.1246 -0.3282 0.0796 -0.0803 1.1197
0.5367 0.8129 -39.9997
83.4200 -38.0300 0.0707 -0.1165 -
0.2121 -0.2979 0.0830 -0.0689 1.1239
0.5299 0.8117 -39.9979
83.4400 -38.3000 -0.2118 -0.1080 -
0.2569 -0.2498 0.0867 -0.0570 1.1278
0.5229 0.8104 -39.9962
83.4600 -37.8700 0.1768 -0.0770 -
0.2426 -0.1858 0.0904 -0.0447 1.1314
0.5159 0.8092 -39.9945
83.4800 -37.9200 -0.2107 -0.0481 -
0.1706 -0.1091 0.0938 -0.0319 1.1347
0.5088 0.8079 -39.9928
83.5000 -37.4300 0.1751 -0.0160 -
0.0613 -0.0246 0.0965 -0.0186 1.1376
0.5016 0.8066 -39.9910
83.5200 -37.4900 -0.1641 0.0268
0.0516 0.0624 0.0981 -0.0047 1.1402
0.4944 0.8053 -39.9893
83.5400 -36.8600 0.1893 0.1069
0.1348 0.1458 0.0984 0.0097 1.1424
0.4870 0.8040 -39.9876
83.5600 -37.1900 -0.2119 0.1732
0.1726 0.2199 0.0969 0.0247 1.1442
0.4795 0.8026 -39.9859
83.5800 -36.6400 0.3117 0.1442
0.1689 0.2792 0.0933 0.0404 1.1457
0.4720 0.8013 -39.9841
83.6000 -37.2400 -0.0136 -0.0312
0.1443 0.3194 0.0874 0.0567 1.1468
0.4644 0.7999 -39.9824
83.6200 -37.3100 0.0431 -0.2494
0.1249 0.3374 0.0788 0.0736 1.1474
0.4567 0.7985 -39.9807
83.6400 -37.5600 -0.4397 -0.2387
0.1250 0.3314 0.0675 0.0911 1.1477
0.4489 0.7971 -39.9790
83.6600 -36.4300 0.5148 0.0549
0.1416 0.3013 0.0534 0.1092 1.1475
0.4410 0.7956 -39.9773

83.6800 -37.1500 -0.3389 0.2835
0.1571 0.2501 0.0367 0.1279 1.1468
0.4331 0.7942 -39.9755
83.7000 -36.4500 0.5400 0.1947
0.1513 0.1822 0.0176 0.1471 1.1457
0.4250 0.7927 -39.9738
83.7200 -37.5400 -0.1912 -0.0618
0.1180 0.1032 -0.0035 0.1668 1.1442
0.4169 0.7913 -39.9721
83.7400 -37.9600 -0.4392 -0.1869
0.0626 0.0192 -0.0264 0.1868 1.1421
0.4087 0.7898 -39.9704
83.7600 -37.1300 0.3879 -0.0715 -
0.0054 -0.0631 -0.0507 0.2072 1.1396
0.4005 0.7883 -39.9687
83.7800 -37.8100 -0.1904 0.0916 -
0.0732 -0.1374 -0.0759 0.2278 1.1366
0.3921 0.7867 -39.9669
83.8000 -37.3700 0.2696 0.1016 -
0.1330 -0.1979 -0.1019 0.2486 1.1331
0.3837 0.7852 -39.9652
83.8200 -38.1800 -0.1316 0.0018 -
0.1797 -0.2402 -0.1281 0.2696 1.1291
0.3753 0.7836 -39.9635
83.8400 -38.3100 -0.1981 -0.0429 -
0.2115 -0.2611 -0.1541 0.2906 1.1246
0.3667 0.7821 -39.9618
83.8600 -37.7800 0.2802 0.0233 -
0.2275 -0.2593 -0.1795 0.3115 1.1195
0.3581 0.7805 -39.9601
83.8800 -38.2400 -0.1959 0.0644 -
0.2220 -0.2354 -0.2038 0.3324 1.1139
0.3494 0.7789 -39.9584
83.9000 -37.8300 0.0475 0.0065 -
0.1883 -0.1920 -0.2264 0.3531 1.1078
0.3407 0.7772 -39.9566
83.9200 -37.8300 0.1331 -0.1232 -
0.1204 -0.1332 -0.2466 0.3735 1.1011
0.3318 0.7756 -39.9549
83.9400 -37.9700 -0.0590 -0.1724 -
0.0217 -0.0643 -0.2638 0.3936 1.0939
0.3230 0.7740 -39.9532
83.9600 -37.9900 -0.3593 -0.0244
0.0896 0.0085 -0.2775 0.4133 1.0861
0.3140 0.7723 -39.9515
83.9800 -36.7200 0.6101 0.1546
0.1895 0.0790 -0.2868 0.4325 1.0778
0.3050 0.7706 -39.9498
84.0000 -37.5300 -0.4644 0.1189
0.2568 0.1409 -0.2914 0.4512 1.0689
0.2960 0.7689 -39.9481

84.0200 -37.2100 0.0720 -0.0035
0.2803 0.1891 -0.2905 0.4692 1.0594
0.2869 0.7672 -39.9463
84.0400 -37.2500 -0.0872 -0.0221
0.2581 0.2192 -0.2837 0.4866 1.0493
0.2777 0.7655 -39.9446
84.0600 -37.0800 0.1528 -0.0034
0.1943 0.2280 -0.2707 0.5031 1.0387
0.2685 0.7638 -39.9429
84.0800 -37.5300 -0.1547 -0.0157
0.1034 0.2148 -0.2515 0.5189 1.0274
0.2592 0.7620 -39.9412
84.1000 -37.5400 -0.1426 0.0128
0.0043 0.1807 -0.2262 0.5338 1.0155
0.2499 0.7603 -39.9395
84.1200 -37.3100 0.1629 0.0630 -
0.0832 0.1280 -0.1953 0.5477 1.0031
0.2405 0.7585 -39.9378
84.1400 -37.1900 0.3881 -0.0343 -
0.1381 0.0604 -0.1595 0.5606 0.9900
0.2311 0.7567 -39.9361
84.1600 -38.3900 -0.5163 -0.2150 -
0.1481 -0.0176 -0.1194 0.5724 0.9764
0.2216 0.7549 -39.9344
84.1800 -37.6600 0.2013 -0.1737 -
0.1111 -0.1013 -0.0759 0.5830 0.9621
0.2121 0.7531 -39.9326
84.2000 -37.8300 -0.2270 0.0758 -
0.0449 -0.1859 -0.0299 0.5925 0.9472
0.2025 0.7512 -39.9309
84.2200 -37.0500 0.5298 0.2050
0.0184 -0.2667 0.0179 0.6006 0.9317
0.1929 0.7494 -39.9292
84.2400 -37.9900 -0.3100 0.0831
0.0482 -0.3387 0.0666 0.6075 0.9157
0.1833 0.7475 -39.9275
84.2600 -37.9300 -0.1123 -0.0706
0.0277 -0.3975 0.1154 0.6129 0.8990
0.1736 0.7457 -39.9258
84.2800 -37.9100 0.0193 -0.0392 -
0.0436 -0.4387 0.1636 0.6168 0.8817
0.1639 0.7438 -39.9241
84.3000 -37.9700 -0.0318 0.1285 -
0.1462 -0.4587 0.2105 0.6192 0.8639
0.1542 0.7419 -39.9224
84.3200 -37.8400 0.0971 0.1694 -
0.2463 -0.4547 0.2554 0.6200 0.8455
0.1444 0.7400 -39.9207
84.3400 -37.9700 0.1758 0.0034 -
0.3139 -0.4250 0.2978 0.6192 0.8265
0.1346 0.7380 -39.9190

84.3600 -38.5400 -0.2378 -0.1917 -
0.3287 -0.3694 0.3373 0.6166 0.8069
0.1247 0.7361 -39.9172
84.3800 -38.1000 -0.0556 -0.2278 -
0.2841 -0.2899 0.3734 0.6122 0.7868
0.1149 0.7342 -39.9155
84.4000 -37.8400 0.0122 -0.0833 -
0.1929 -0.1894 0.4056 0.6060 0.7662
0.1050 0.7322 -39.9138
84.4200 -37.2500 0.0560 0.1151 -
0.0857 -0.0715 0.4337 0.5979 0.7450
0.0951 0.7302 -39.9121
84.4400 -37.1300 0.0573 0.2106
0.0017 0.0600 0.4571 0.5879 0.7233
0.0851 0.7282 -39.9104
84.4600 -37.0200 0.0087 0.1552
0.0422 0.2000 0.4754 0.5760 0.7011
0.0752 0.7262 -39.9087
84.4800 -37.0000 -0.0347 0.0172
0.0315 0.3425 0.4883 0.5621 0.6784
0.0652 0.7242 -39.9070
84.5000 -36.8800 0.2040 -0.0881 -
0.0119 0.4798 0.4954 0.5462 0.6553
0.0552 0.7222 -39.9053
84.5200 -37.4600 -0.4276 -0.0755 -
0.0587 0.6035 0.4962 0.5285 0.6317
0.0452 0.7202 -39.9036
84.5400 -36.5400 0.5121 -0.0237 -
0.0797 0.7053 0.4903 0.5089 0.6076
0.0351 0.7181 -39.9019
84.5600 -37.3000 -0.3783 -0.0492 -
0.0483 0.7780 0.4774 0.4875 0.5832
0.0251 0.7161 -39.9002
84.5800 -36.8300 0.0418 -0.0500
0.0518 0.8159 0.4573 0.4644 0.5583
0.0151 0.7140 -39.8985
84.6000 -36.8700 -0.0576 0.0063
0.2216 0.8162 0.4300 0.4397 0.5331
0.0050 0.7119 -39.8968
84.6200 -36.7100 -0.0943 0.0323
0.4496 0.7785 0.3961 0.4136 0.5075
-0.0051 0.7098 -39.8951
84.6400 -36.4400 0.1670 -0.0657
0.7016 0.7041 0.3561 0.3861 0.4815
-0.0152 0.7077 -39.8934
84.6600 -36.6200 0.2856 -0.2297
0.9249 0.5959 0.3110 0.3573 0.4552
-0.0252 0.7056 -39.8917
84.6800 -37.1700 -0.3167 -0.1631
1.0518 0.4577 0.2618 0.3274 0.4286
-0.0353 0.7035 -39.8900

84.7000 -37.3900 -0.6034 0.2478
1.0220 0.2952 0.2093 0.2965 0.4017
-0.0454 0.7014 -39.8883
84.7200 -36.0900 0.8359 0.6223
0.8036 0.1162 0.1544 0.2647 0.3745
-0.0555 0.6992 -39.8865
84.7400 -37.7400 0.1200 0.4266
0.4042 -0.0703 0.0982 0.2321 0.3471
-0.0656 0.6971 -39.8848
84.7600 -39.7600 -0.5598 -0.1611 -
0.0989 -0.2548 0.0415 0.1987 0.3194
-0.0757 0.6949 -39.8831
84.7800 -40.3200 0.1318 -0.6024 -
0.6032 -0.4280 -0.0148 0.1648 0.2915
-0.0857 0.6928 -39.8814
84.8000 -41.4500 -0.3446 -0.5343 -
1.0126 -0.5804 -0.0696 0.1305 0.2633
-0.0958 0.6906 -39.8797
84.8200 -41.0900 0.1254 -0.1108 -
1.2622 -0.7026 -0.1221 0.0957 0.2350
-0.1059 0.6884 -39.8780
84.8400 -40.7900 0.2384 0.2768 -
1.3216 -0.7861 -0.1714 0.0608 0.2065
-0.1159 0.6862 -39.8763
84.8600 -41.0500 -0.0609 0.3608 -
1.1847 -0.8258 -0.2165 0.0257 0.1778
-0.1260 0.6840 -39.8746
84.8800 -41.0600 -0.1696 0.1341 -
0.8737 -0.8230 -0.2568 -0.0094 0.1489
-0.1360 0.6817 -39.8729
84.9000 -40.8000 0.2675 -0.2346 -
0.4412 -0.7837 -0.2920 -0.0445 0.1200
-0.1460 0.6795 -39.8712
84.9200 -41.1700 -0.2539 -0.5191
0.0400 -0.7144 -0.3219 -0.0793 0.0909
-0.1560 0.6773 -39.8695
84.9400 -40.4500 -0.1323 -0.4763
0.4952 -0.6219 -0.3466 -0.1138 0.0618
-0.1660 0.6750 -39.8678
84.9600 -39.2000 0.3401 -0.0406
0.8665 -0.5127 -0.3663 -0.1478 0.0325
-0.1759 0.6728 -39.8662
84.9800 -39.1400 -0.5997 0.5907
1.1086 -0.3934 -0.3810 -0.1813 0.0032
-0.1859 0.6705 -39.8645
85.0000 -37.5900 0.4888 0.9323
1.1867 -0.2707 -0.3910 -0.2142 -0.0261
-0.1958 0.6682 -39.8628
85.0200 -37.7300 0.7148 0.6604
1.0903 -0.1504 -0.3965 -0.2463 -0.0555
-0.2057 0.6659 -39.8611

85.0400 -39.8400 -0.4354 -0.1146
0.8698 -0.0370 -0.3979 -0.2775 -0.0849
-0.2155 0.6636 -39.8594
85.0600 -40.8900 -0.5494 -0.7850
0.5975 0.0657 -0.3954 -0.3078 -0.1142
-0.2254 0.6613 -39.8577
85.0800 -40.6700 0.0161 -0.8836
0.3409 0.1542 -0.3893 -0.3370 -0.1436
-0.2352 0.6590 -39.8560
85.1000 -40.1100 0.3667 -0.4981
0.1400 0.2261 -0.3800 -0.3651 -0.1729
-0.2449 0.6567 -39.8543
85.1200 -40.4100 -0.4515 0.0852 -
0.0052 0.2810 -0.3680 -0.3918 -0.2021
-0.2547 0.6544 -39.8526
85.1400 -39.4400 0.2967 0.5838 -
0.1244 0.3215 -0.3538 -0.4173 -0.2313
-0.2644 0.6520 -39.8509
85.1600 -39.8400 -0.0799 0.6886 -
0.2452 0.3508 -0.3377 -0.4413 -0.2603
-0.2740 0.6497 -39.8492
85.1800 -39.9800 0.2029 0.3009 -
0.3768 0.3719 -0.3203 -0.4639 -0.2893
-0.2837 0.6473 -39.8475
85.2000 -41.2800 -0.3179 -0.2168 -
0.5033 0.3865 -0.3023 -0.4848 -0.3181
-0.2932 0.6450 -39.8458
85.2200 -41.2100 0.0607 -0.4247 -
0.5935 0.3955 -0.2840 -0.5041 -0.3468
-0.3028 0.6426 -39.8441
85.2400 -41.2700 -0.0893 -0.2806 -
0.6110 0.3996 -0.2660 -0.5217 -0.3753
-0.3123 0.6402 -39.8424
85.2600 -40.6800 0.2944 -0.0991 -
0.5347 0.3997 -0.2488 -0.5375 -0.4037
-0.3217 0.6379 -39.8407
85.2800 -40.7900 0.0552 -0.0557 -
0.3707 0.3962 -0.2328 -0.5516 -0.4318
-0.3312 0.6355 -39.8390
85.3000 -41.1700 -0.6795 0.0116 -
0.1550 0.3892 -0.2181 -0.5638 -0.4598
-0.3405 0.6331 -39.8373
85.3200 -39.6200 0.6062 0.1682
0.0621 0.3778 -0.2049 -0.5742 -0.4875
-0.3498 0.6307 -39.8357
85.3400 -39.6600 0.4363 0.1825
0.2289 0.3611 -0.1933 -0.5827 -0.5149
-0.3591 0.6283 -39.8340
85.3600 -40.9600 -0.7732 0.0828
0.3083 0.3388 -0.1833 -0.5894 -0.5422
-0.3683 0.6258 -39.8323

85.3800 -39.6800 0.6535 0.0837
0.2923 0.3103 -0.1749 -0.5943 -0.5691
-0.3775 0.6234 -39.8306
85.4000 -40.8200 -0.3862 0.1269
0.2009 0.2760 -0.1680 -0.5974 -0.5957
-0.3866 0.6210 -39.8289
85.4200 -40.3900 0.1133 0.0485
0.0781 0.2363 -0.1623 -0.5988 -0.6221
-0.3956 0.6185 -39.8272
85.4400 -40.9500 0.0762 -0.1526 -
0.0282 0.1921 -0.1578 -0.5984 -0.6481
-0.4046 0.6161 -39.8255
85.4600 -41.2600 0.0763 -0.4004 -
0.0856 0.1446 -0.1541 -0.5965 -0.6739
-0.4135 0.6137 -39.8238
85.4800 -41.9100 -0.2958 -0.4723 -
0.0826 0.0951 -0.1509 -0.5929 -0.6992
-0.4224 0.6112 -39.8221
85.5000 -41.3900 -0.2013 -0.1515 -
0.0321 0.0450 -0.1482 -0.5878 -0.7243
-0.4311 0.6087 -39.8205
85.5200 -40.4700 0.3592 0.3790
0.0282 -0.0042 -0.1455 -0.5812 -0.7489
-0.4399 0.6063 -39.8188
85.5400 -40.4300 0.0037 0.6564
0.0633 -0.0506 -0.1427 -0.5733 -0.7732
-0.4485 0.6038 -39.8171
85.5600 -40.4700 0.3347 0.3517
0.0524 -0.0923 -0.1396 -0.5640 -0.7971
-0.4571 0.6013 -39.8154
85.5800 -41.2200 0.3213 -0.2932
0.0075 -0.1279 -0.1359 -0.5535 -0.8205
-0.4656 0.5988 -39.8137
85.6000 -42.8600 -0.8170 -0.6325 -
0.0441 -0.1566 -0.1313 -0.5418 -0.8436
-0.4741 0.5963 -39.8120
85.6200 -41.1000 0.5295 -0.3125 -
0.0772 -0.1786 -0.1254 -0.5291 -0.8662
-0.4824 0.5938 -39.8103
85.6400 -41.1600 0.2088 0.1582 -
0.0861 -0.1941 -0.1182 -0.5153 -0.8884
-0.4907 0.5913 -39.8087
85.6600 -41.4400 -0.2277 0.3389 -
0.0736 -0.2037 -0.1092 -0.5006 -0.9101
-0.4990 0.5888 -39.8070
85.6800 -41.2000 0.0184 0.2648 -
0.0533 -0.2083 -0.0983 -0.4851 -0.9313
-0.5071 0.5863 -39.8053
85.7000 -41.2100 0.1195 0.1353 -
0.0381 -0.2090 -0.0853 -0.4688 -0.9521
-0.5151 0.5838 -39.8036

85.7200 -41.7200 -0.2335 -0.0012 -
0.0258 -0.2070 -0.0699 -0.4518 -0.9723
-0.5231 0.5813 -39.8019
85.7400 -41.1300 0.3844 -0.1822 -
0.0048 -0.2031 -0.0523 -0.4343 -0.9921
-0.5310 0.5787 -39.8002
85.7600 -41.8600 -0.2305 -0.2964
0.0360 -0.1982 -0.0324 -0.4162 -1.0114
-0.5388 0.5762 -39.7986
85.7800 -41.9000 -0.5396 -0.1692
0.0941 -0.1926 -0.0104 -0.3977 -1.0301
-0.5465 0.5737 -39.7969
85.8000 -40.4500 0.6329 0.1236
0.1506 -0.1868 0.0136 -0.3788 -1.0483
-0.5541 0.5711 -39.7952
85.8200 -41.0900 -0.1658 0.2099
0.1814 -0.1803 0.0393 -0.3597 -1.0659
-0.5617 0.5686 -39.7935
85.8400 -41.0400 0.1844 0.0067
0.1726 -0.1722 0.0663 -0.3404 -1.0830
-0.5691 0.5660 -39.7918
85.8600 -41.8900 -0.5024 -0.1275
0.1219 -0.1609 0.0943 -0.3210 -1.0996
-0.5765 0.5635 -39.7902
85.8800 -41.2700 -0.0786 0.0201
0.0342 -0.1449 0.1230 -0.3016 -1.1155
-0.5837 0.5609 -39.7885
85.9000 -41.2900 -0.2435 0.2521 -
0.0774 -0.1226 0.1518 -0.2823 -1.1309
-0.5909 0.5584 -39.7868
85.9200 -40.2800 0.8877 0.1545 -
0.1927 -0.0926 0.1803 -0.2631 -1.1457
-0.5979 0.5558 -39.7851
85.9400 -42.3400 -0.8032 -0.2158 -
0.2843 -0.0544 0.2081 -0.2441 -1.1599
-0.6049 0.5532 -39.7834
85.9600 -41.8900 -0.2541 -0.3159 -
0.3230 -0.0082 0.2348 -0.2254 -1.1734
-0.6117 0.5507 -39.7818
85.9800 -40.9600 0.3519 -0.0855 -
0.2929 0.0446 0.2598 -0.2071 -1.1864
-0.6185 0.5481 -39.7801
86.0000 -40.8600 0.1083 0.1287 -
0.1997 0.1010 0.2828 -0.1893 -1.1987
-0.6251 0.5455 -39.7784
86.0200 -40.7100 0.0318 0.1025 -
0.0626 0.1573 0.3033 -0.1720 -1.2104
-0.6316 0.5429 -39.7767
86.0400 -40.8600 -0.2147 -0.0130
0.0902 0.2089 0.3212 -0.1553 -1.2214
-0.6381 0.5403 -39.7751

86.0600	-40.4100	0.1900	-0.0526	
0.2302	0.2514	0.3360	-0.1394	-1.2318
	-0.6444	0.5377	-39.7734	
86.0800	-40.3300	0.0679	-0.0653	
0.3336	0.2801	0.3474	-0.1242	-1.2415
	-0.6506	0.5352	-39.7717	
86.1000	-40.3800	-0.1113	-0.0300	
0.3796	0.2916	0.3551	-0.1099	-1.2505
	-0.6567	0.5326	-39.7700	
86.1200	-40.6000	-0.5880	0.1555	
0.3590	0.2838	0.3591	-0.0965	-1.2589
	-0.6627	0.5300	-39.7684	
86.1400	-39.2200	0.9174	0.2735	
0.2799	0.2573	0.3594	-0.0841	-1.2666
	-0.6685	0.5274	-39.7667	
86.1600	-40.7300	-0.3410	0.0704	
0.1602	0.2147	0.3563	-0.0727	-1.2736
	-0.6743	0.5248	-39.7650	
86.1800	-41.3100	-0.4182	-0.2072	
0.0277	0.1596	0.3501	-0.0623	-1.2798
	-0.6799	0.5222	-39.7633	
86.2000	-41.4700	-0.2562	-0.2690	-
0.0867	0.0960	0.3410	-0.0531	-1.2854
	-0.6854	0.5196	-39.7617	
86.2200	-40.8400	0.4530	-0.1260	-
0.1594	0.0280	0.3295	-0.0449	-1.2903
	-0.6908	0.5169	-39.7600	
86.2400	-41.1200	0.0614	0.0080	-
0.1868	-0.0398	0.3159	-0.0379	-1.2945
	-0.6960	0.5143	-39.7583	
86.2600	-41.2300	-0.0336	0.0006	-
0.1769	-0.1025	0.3005	-0.0321	-1.2979
	-0.7011	0.5117	-39.7567	
86.2800	-41.3300	-0.0274	-0.0204	-
0.1487	-0.1556	0.2837	-0.0274	-1.3006
	-0.7062	0.5091	-39.7550	
86.3000	-41.3400	-0.1141	0.0967	-
0.1245	-0.1951	0.2657	-0.0239	-1.3027
	-0.7110	0.5065	-39.7533	
86.3200	-41.0800	0.1074	0.2634	-
0.1235	-0.2178	0.2470	-0.0215	-1.3039
	-0.7158	0.5039	-39.7516	
86.3400	-41.0400	0.1972	0.2438	-
0.1531	-0.2227	0.2279	-0.0204	-1.3045
	-0.7204	0.5013	-39.7500	
86.3600	-41.5700	-0.0850	0.0433	-
0.2011	-0.2106	0.2087	-0.0203	-1.3044
	-0.7249	0.4986	-39.7483	
86.3800	-42.0800	-0.4068	-0.1321	-
0.2380	-0.1837	0.1897	-0.0215	-1.3035
	-0.7293	0.4960	-39.7466	

86.4000 -41.0700 0.5838 -0.1764 -
0.2297 -0.1451 0.1712 -0.0238 -1.3020
-0.7336 0.4934 -39.7450
86.4200 -42.0800 -0.6488 -0.1496 -
0.1579 -0.0989 0.1534 -0.0272 -1.2997
-0.7377 0.4908 -39.7433
86.4400 -40.7100 0.5356 -0.0753 -
0.0341 -0.0495 0.1365 -0.0317 -1.2968
-0.7417 0.4882 -39.7416
86.4600 -41.4300 -0.4713 0.0472
0.1084 -0.0012 0.1209 -0.0373 -1.2932
-0.7455 0.4855 -39.7400
86.4800 -40.6100 0.1898 0.1731
0.2332 0.0416 0.1068 -0.0439 -1.2888
-0.7493 0.4829 -39.7383
86.5000 -40.4900 0.0951 0.1958
0.3136 0.0750 0.0944 -0.0516 -1.2838
-0.7529 0.4803 -39.7366
86.5200 -40.7300 0.0257 0.0582
0.3394 0.0955 0.0839 -0.0603 -1.2782
-0.7563 0.4777 -39.7350
86.5400 -41.0900 -0.0952 -0.1044
0.3118 0.1013 0.0753 -0.0701 -1.2719
-0.7597 0.4750 -39.7333
86.5600 -41.1800 -0.0920 -0.1324
0.2422 0.0921 0.0688 -0.0807 -1.2649
-0.7629 0.4724 -39.7316
86.5800 -41.0900 0.0319 -0.0357
0.1483 0.0692 0.0643 -0.0923 -1.2573
-0.7660 0.4698 -39.7300
86.6000 -41.2900 0.1065 0.0118
0.0501 0.0357 0.0617 -0.1048 -1.2491
-0.7690 0.4672 -39.7283
86.6200 -41.4900 -0.1075 -0.0705 -
0.0384 -0.0048 0.0606 -0.1181 -1.2402
-0.7718 0.4645 -39.7267
86.6400 -41.3900 0.1646 -0.1065 -
0.1067 -0.0477 0.0610 -0.1322 -1.2307
-0.7745 0.4619 -39.7250
86.6600 -41.9600 -0.4140 0.0192 -
0.1542 -0.0880 0.0626 -0.1469 -1.2207
-0.7771 0.4593 -39.7233
86.6800 -40.8200 0.6751 0.1525 -
0.1869 -0.1205 0.0651 -0.1623 -1.2100
-0.7796 0.4566 -39.7217
86.7000 -42.0800 -0.6030 0.1488 -
0.2105 -0.1409 0.0683 -0.1782 -1.1988
-0.7820 0.4540 -39.7200
86.7200 -42.0200 -0.4097 0.0400 -
0.2257 -0.1459 0.0719 -0.1946 -1.1870
-0.7842 0.4514 -39.7184

86.7400 -40.9700 1.0013 -0.1332 -
0.2254 -0.1344 0.0756 -0.2114 -1.1746
-0.7863 0.4488 -39.7167
86.7600 -42.7400 -0.9287 -0.2350 -
0.2046 -0.1069 0.0792 -0.2285 -1.1617
-0.7883 0.4462 -39.7150
86.7800 -41.8700 -0.0522 -0.0956 -
0.1683 -0.0657 0.0823 -0.2458 -1.1482
-0.7901 0.4435 -39.7134
86.8000 -40.7700 0.5301 0.1278 -
0.1221 -0.0141 0.0847 -0.2633 -1.1343
-0.7919 0.4409 -39.7117
86.8200 -41.0100 0.1386 0.1820 -
0.0715 0.0439 0.0861 -0.2807 -1.1198
-0.7935 0.4383 -39.7101
86.8400 -41.7700 -0.3664 0.0507 -
0.0199 0.1037 0.0861 -0.2982 -1.1048
-0.7951 0.4357 -39.7084
86.8600 -41.4500 -0.2067 -0.0702
0.0321 0.1607 0.0846 -0.3155 -1.0893
-0.7965 0.4331 -39.7067
86.8800 -40.7600 0.5233 -0.0995
0.0850 0.2106 0.0812 -0.3326 -1.0733
-0.7978 0.4304 -39.7051
86.9000 -41.3400 -0.2860 -0.0568
0.1355 0.2496 0.0757 -0.3494 -1.0568
-0.7989 0.4278 -39.7034
86.9200 -41.3200 -0.4137 0.0811
0.1792 0.2744 0.0680 -0.3659 -1.0399
-0.8000 0.4252 -39.7018
86.9400 -40.2000 0.5522 0.1914
0.2101 0.2828 0.0577 -0.3819 -1.0226
-0.8010 0.4226 -39.7001
86.9600 -40.8300 0.0768 0.0290
0.2236 0.2737 0.0448 -0.3973 -1.0048
-0.8018 0.4200 -39.6985
86.9800 -41.7300 -0.5011 -0.2066
0.2172 0.2474 0.0293 -0.4123 -0.9865
-0.8026 0.4174 -39.6968
87.0000 -41.0600 0.1685 -0.1624
0.1876 0.2061 0.0114 -0.4265 -0.9679
-0.8032 0.4148 -39.6952
87.0200 -41.1300 0.0350 0.1159
0.1300 0.1536 -0.0087 -0.4401 -0.9488
-0.8037 0.4122 -39.6935
87.0400 -41.2400 -0.1983 0.3057
0.0438 0.0944 -0.0308 -0.4530 -0.9293
-0.8042 0.4096 -39.6919
87.0600 -40.9700 0.4845 0.1583 -
0.0609 0.0334 -0.0546 -0.4650 -0.9095
-0.8045 0.4070 -39.6902

87.0800 -42.4000 -0.4507 -0.1969 -
0.1678 -0.0249 -0.0798 -0.4762 -0.8893
-0.8047 0.4044 -39.6886
87.1000 -42.0700 0.1172 -0.3211 -
0.2542 -0.0759 -0.1061 -0.4866 -0.8687
-0.8048 0.4018 -39.6869
87.1200 -42.3600 -0.2299 -0.1171 -
0.3010 -0.1155 -0.1332 -0.4960 -0.8477
-0.8048 0.3992 -39.6852
87.1400 -41.5900 0.2107 0.1462 -
0.2999 -0.1409 -0.1606 -0.5045 -0.8265
-0.8048 0.3966 -39.6836
87.1600 -41.4400 0.4163 0.2104 -
0.2522 -0.1504 -0.1881 -0.5120 -0.8048
-0.8046 0.3940 -39.6819
87.1800 -41.9500 -0.0916 0.0202 -
0.1685 -0.1447 -0.2151 -0.5185 -0.7829
-0.8043 0.3915 -39.6803
87.2000 -42.5000 -0.5436 -0.1490 -
0.0624 -0.1262 -0.2412 -0.5240 -0.7607
-0.8039 0.3889 -39.6787
87.2200 -41.2600 0.4695 -0.0639
0.0488 -0.0984 -0.2660 -0.5284 -0.7381
-0.8034 0.3863 -39.6770
87.2400 -41.7600 -0.3037 0.1072
0.1461 -0.0658 -0.2890 -0.5316 -0.7153
-0.8029 0.3837 -39.6754
87.2600 -41.3600 0.0651 0.1577
0.2123 -0.0327 -0.3098 -0.5338 -0.6922
-0.8022 0.3812 -39.6737
87.2800 -41.0900 0.1559 0.0582
0.2406 -0.0032 -0.3280 -0.5348 -0.6688
-0.8015 0.3786 -39.6721
87.3000 -41.6100 -0.0786 -0.0994
0.2382 0.0190 -0.3432 -0.5346 -0.6452
-0.8006 0.3760 -39.6704
87.3200 -41.7400 -0.1886 -0.1480
0.2146 0.0313 -0.3551 -0.5332 -0.6214
-0.7997 0.3735 -39.6688
87.3400 -41.1900 0.0925 -0.0204
0.1794 0.0313 -0.3633 -0.5307 -0.5973
-0.7987 0.3709 -39.6671
87.3600 -41.3700 -0.0960 0.1212
0.1444 0.0180 -0.3676 -0.5270 -0.5730
-0.7975 0.3684 -39.6655
87.3800 -41.1300 0.2895 0.0403
0.1153 -0.0081 -0.3679 -0.5221 -0.5485
-0.7963 0.3658 -39.6638
87.4000 -41.9500 -0.2382 -0.1606
0.0884 -0.0441 -0.3641 -0.5161 -0.5238
-0.7951 0.3633 -39.6622

87.4200 -41.5600 -0.0064 -0.1664
0.0542 -0.0856 -0.3563 -0.5090 -0.4990
-0.7937 0.3607 -39.6605
87.4400 -41.7500 -0.2200 0.0494
0.0043 -0.1281 -0.3446 -0.5008 -0.4739
-0.7922 0.3582 -39.6589
87.4600 -40.8000 0.4661 0.2009 -
0.0670 -0.1665 -0.3291 -0.4916 -0.4487
-0.7907 0.3557 -39.6573
87.4800 -41.6800 -0.1598 0.0836 -
0.1567 -0.1968 -0.3100 -0.4814 -0.4234
-0.7891 0.3532 -39.6556
87.5000 -41.9100 -0.0743 -0.1133 -
0.2468 -0.2154 -0.2876 -0.4702 -0.3979
-0.7873 0.3506 -39.6540
87.5200 -42.1700 -0.4486 -0.0964 -
0.3138 -0.2197 -0.2621 -0.4582 -0.3723
-0.7856 0.3481 -39.6523
87.5400 -40.9200 0.6129 0.0280 -
0.3452 -0.2074 -0.2339 -0.4454 -0.3466
-0.7837 0.3456 -39.6507
87.5600 -42.0100 -0.4149 0.0607 -
0.3447 -0.1778 -0.2033 -0.4317 -0.3207
-0.7817 0.3431 -39.6491
87.5800 -41.2600 0.2068 0.0524 -
0.3188 -0.1316 -0.1708 -0.4174 -0.2948
-0.7797 0.3406 -39.6474
87.6000 -41.2600 -0.0153 0.0290 -
0.2685 -0.0717 -0.1368 -0.4024 -0.2688
-0.7776 0.3381 -39.6458
87.6200 -41.0900 0.0013 -0.0275 -
0.1881 -0.0026 -0.1017 -0.3867 -0.2427
-0.7754 0.3356 -39.6441
87.6400 -41.0000 -0.2166 -0.0494 -
0.0708 0.0709 -0.0660 -0.3705 -0.2166
-0.7732 0.3331 -39.6425
87.6600 -40.1100 0.3493 -0.0822
0.0810 0.1438 -0.0301 -0.3538 -0.1904
-0.7709 0.3306 -39.6409
87.6800 -40.4900 -0.1616 -0.1830
0.2535 0.2112 0.0056 -0.3366 -0.1642
-0.7685 0.3281 -39.6392
87.7000 -40.1300 -0.1057 -0.1591
0.4217 0.2682 0.0407 -0.3190 -0.1379
-0.7660 0.3257 -39.6376
87.7200 -39.5300 -0.0204 0.0739
0.5495 0.3107 0.0748 -0.3011 -0.1116
-0.7635 0.3232 -39.6360
87.7400 -38.9100 0.1617 0.3213
0.6018 0.3348 0.1074 -0.2829 -0.0854
-0.7608 0.3207 -39.6343

87.7600 -39.0200 0.0445 0.3690
0.5551 0.3380 0.1383 -0.2644 -0.0591
-0.7582 0.3183 -39.6327
87.7800 -39.1800 0.0566 0.2174
0.4107 0.3209 0.1671 -0.2458 -0.0328
-0.7554 0.3158 -39.6311
87.8000 -39.8000 -0.1849 0.0194
0.1982 0.2868 0.1938 -0.2270 -0.0065
-0.7526 0.3134 -39.6294
87.8200 -40.0000 -0.1947 -0.0995 -
0.0379 0.2400 0.2183 -0.2081 0.0197
-0.7497 0.3109 -39.6278
87.8400 -39.7700 0.3163 -0.1750 -
0.2503 0.1846 0.2405 -0.1891 0.0459
-0.7468 0.3085 -39.6262
87.8600 -40.2600 0.0500 -0.2452 -
0.4048 0.1248 0.2606 -0.1702 0.0720
-0.7438 0.3061 -39.6245
87.8800 -40.7700 -0.2682 -0.2199 -
0.4895 0.0651 0.2784 -0.1513 0.0981
-0.7407 0.3036 -39.6229
87.9000 -40.4100 -0.1046 -0.0317 -
0.5101 0.0091 0.2941 -0.1326 0.1241
-0.7376 0.3012 -39.6213
87.9200 -39.9400 0.0706 0.1852 -
0.4787 -0.0397 0.3077 -0.1140 0.1500
-0.7344 0.2988 -39.6196
87.9400 -39.5300 0.3765 0.1983 -
0.4102 -0.0796 0.3193 -0.0956 0.1759
-0.7311 0.2964 -39.6180
87.9600 -40.3600 -0.3405 0.0100 -
0.3142 -0.1098 0.3290 -0.0775 0.2016
-0.7278 0.2940 -39.6164
87.9800 -40.0700 -0.1321 -0.0967 -
0.1949 -0.1312 0.3369 -0.0596 0.2272
-0.7244 0.2916 -39.6147
88.0000 -39.4500 0.1699 -0.0668 -
0.0532 -0.1452 0.3431 -0.0420 0.2527
-0.7210 0.2892 -39.6131
88.0200 -39.3700 0.1358 -0.0830
0.1045 -0.1536 0.3477 -0.0249 0.2780
-0.7175 0.2868 -39.6115
88.0400 -39.7300 -0.2512 -0.1231
0.2581 -0.1577 0.3508 -0.0081 0.3032
-0.7140 0.2844 -39.6099
88.0600 -39.1100 0.0463 -0.0636
0.3798 -0.1585 0.3525 0.0083 0.3283
-0.7104 0.2821 -39.6082
88.0800 -38.8900 0.1923 0.0584
0.4436 -0.1567 0.3529 0.0242 0.3532
-0.7068 0.2797 -39.6066

88.1000	-38.9600	-0.1224	0.1568	
0.4265	-0.1526	0.3522	0.0396	0.3779
	-0.7031	0.2773	-39.6050	
88.1200	-38.8800	-0.0857	0.2698	
0.3201	-0.1468	0.3503	0.0545	0.4024
	-0.6993	0.2750	-39.6034	
88.1400	-38.5000	0.3553	0.3039	
0.1377	-0.1392	0.3473	0.0690	0.4267
	-0.6955	0.2726	-39.6017	
88.1600	-39.2400	-0.0284	0.1250	-
0.0836	-0.1293	0.3432	0.0829	0.4508
	-0.6917	0.2703	-39.6001	
88.1800	-39.9300	-0.1792	-0.1507	-
0.2894	-0.1169	0.3381	0.0964	0.4747
	-0.6878	0.2680	-39.5985	
88.2000	-39.9300	-0.1863	-0.3581	-
0.4195	-0.1013	0.3318	0.1094	0.4984
	-0.6838	0.2656	-39.5969	
88.2200	-39.4600	0.4705	-0.4112	-
0.4289	-0.0823	0.3244	0.1219	0.5218
	-0.6798	0.2633	-39.5952	
88.2400	-40.3000	-0.9119	-0.0796	-
0.3188	-0.0600	0.3159	0.1340	0.5450
	-0.6758	0.2610	-39.5936	
88.2600	-37.9100	1.0216	0.3522	-
0.1304	-0.0349	0.3061	0.1456	0.5678
	-0.6717	0.2587	-39.5920	
88.2800	-39.2100	-0.5006	0.2535	
0.0837	-0.0085	0.2949	0.1568	0.5905
	-0.6676	0.2564	-39.5904	
88.3000	-39.0800	-0.4174	-0.0999	
0.2674	0.0179	0.2824	0.1675	0.6128
	-0.6635	0.2541	-39.5888	
88.3200	-38.3000	0.3935	-0.2007	
0.3763	0.0427	0.2683	0.1779	0.6348
	-0.6593	0.2518	-39.5871	
88.3400	-38.7200	-0.3187	0.0763	
0.3941	0.0645	0.2527	0.1879	0.6565
	-0.6550	0.2495	-39.5855	
88.3600	-37.9800	0.2745	0.2896	
0.3267	0.0822	0.2355	0.1975	0.6779
	-0.6507	0.2473	-39.5839	
88.3800	-38.3700	0.0518	0.1551	
0.1901	0.0961	0.2165	0.2068	0.6989
	-0.6464	0.2450	-39.5823	
88.4000	-39.0200	-0.2216	-0.1214	
0.0170	0.1069	0.1956	0.2158	0.7196
	-0.6420	0.2428	-39.5807	
88.4200	-39.0600	-0.0200	-0.2003	-
0.1547	0.1158	0.1729	0.2245	0.7399
	-0.6377	0.2405	-39.5790	

88.4400 -38.6700 0.3685 -0.1102 -
0.2911 0.1236 0.1482 0.2330 0.7598
-0.6332 0.2383 -39.5774
88.4600 -39.3100 -0.2997 -0.0158 -
0.3695 0.1316 0.1216 0.2412 0.7794
-0.6288 0.2360 -39.5758
88.4800 -38.8500 0.1170 0.0822 -
0.3822 0.1411 0.0929 0.2491 0.7986
-0.6243 0.2338 -39.5742
88.5000 -39.0300 -0.1885 0.1193 -
0.3339 0.1533 0.0624 0.2569 0.8173
-0.6197 0.2316 -39.5726
88.5200 -38.6300 0.3588 0.0404 -
0.2383 0.1681 0.0301 0.2645 0.8357
-0.6152 0.2294 -39.5710
88.5400 -38.8500 -0.0526 -0.0757 -
0.1119 0.1841 -0.0036 0.2720 0.8536
-0.6106 0.2272 -39.5694
88.5600 -38.8600 -0.2102 -0.1112
0.0257 0.1987 -0.0385 0.2793 0.8711
-0.6060 0.2250 -39.5677
88.5800 -38.4600 0.0569 -0.0462
0.1570 0.2092 -0.0744 0.2864 0.8881
-0.6013 0.2228 -39.5661
88.6000 -38.1400 0.1231 0.0442
0.2668 0.2128 -0.1108 0.2934 0.9047
-0.5966 0.2206 -39.5645
88.6200 -38.3400 -0.1320 0.1209
0.3396 0.2070 -0.1474 0.3003 0.9208
-0.5919 0.2185 -39.5629
88.6400 -38.2900 -0.0211 0.1589
0.3687 0.1898 -0.1840 0.3070 0.9364
-0.5872 0.2163 -39.5613
88.6600 -38.1700 0.1274 0.1094
0.3564 0.1600 -0.2201 0.3137 0.9515
-0.5824 0.2141 -39.5597
88.6800 -38.5900 0.0100 -0.0526
0.3117 0.1174 -0.2554 0.3202 0.9661
-0.5777 0.2120 -39.5581
88.7000 -38.6400 0.1476 -0.2280
0.2485 0.0628 -0.2895 0.3266 0.9802
-0.5729 0.2098 -39.5565
88.7200 -39.2900 -0.3007 -0.2407
0.1769 -0.0006 -0.3218 0.3330 0.9938
-0.5680 0.2077 -39.5549
88.7400 -38.7600 0.2001 -0.0545
0.0998 -0.0688 -0.3521 0.3392 1.0069
-0.5632 0.2056 -39.5533
88.7600 -38.9400 -0.0186 0.1289
0.0136 -0.1367 -0.3797 0.3453 1.0194
-0.5583 0.2035 -39.5516

88.7800 -38.9500 0.0726 0.2003 -
0.0872 -0.1994 -0.4044 0.3514 1.0314
-0.5535 0.2014 -39.5500
88.8000 -39.3200 -0.2180 0.1937 -
0.2027 -0.2521 -0.4256 0.3573 1.0429
-0.5486 0.1993 -39.5484
88.8200 -39.0500 0.3768 0.0820 -
0.3184 -0.2907 -0.4431 0.3632 1.0537
-0.5436 0.1972 -39.5468
88.8400 -39.3600 0.2629 -0.1289 -
0.4094 -0.3118 -0.4563 0.3689 1.0641
-0.5387 0.1951 -39.5452
88.8600 -40.5300 -0.6574 -0.2427 -
0.4488 -0.3129 -0.4652 0.3746 1.0739
-0.5338 0.1930 -39.5436
88.8800 -39.9300 -0.2347 -0.1512 -
0.4191 -0.2934 -0.4694 0.3802 1.0831
-0.5288 0.1910 -39.5420
88.9000 -38.7100 0.8162 0.0064 -
0.3229 -0.2552 -0.4691 0.3857 1.0917
-0.5238 0.1889 -39.5404
88.9200 -39.6200 -0.4994 0.0660 -
0.1753 -0.2021 -0.4646 0.3911 1.0998
-0.5188 0.1869 -39.5388
88.9400 -39.1600 -0.3807 0.0847 -
0.0014 -0.1387 -0.4560 0.3964 1.1073
-0.5139 0.1848 -39.5372
88.9600 -38.3400 0.2610 0.1165
0.1682 -0.0701 -0.4436 0.4016 1.1143
-0.5088 0.1828 -39.5356
88.9800 -38.2500 0.1758 0.0688
0.3027 -0.0013 -0.4278 0.4065 1.1206
-0.5038 0.1808 -39.5340
89.0000 -38.6300 -0.2813 -0.0148
0.3757 0.0629 -0.4089 0.4113 1.1264
-0.4988 0.1788 -39.5324
89.0200 -38.2800 -0.1700 0.0328
0.3739 0.1183 -0.3871 0.4159 1.1316
-0.4938 0.1768 -39.5308
89.0400 -37.8900 0.1939 0.1745
0.3045 0.1618 -0.3628 0.4203 1.1362
-0.4887 0.1748 -39.5292
89.0600 -38.0300 -0.0422 0.2060
0.1920 0.1912 -0.3363 0.4244 1.1402
-0.4837 0.1728 -39.5276
89.0800 -38.3600 -0.0222 0.0128
0.0706 0.2050 -0.3078 0.4283 1.1437
-0.4787 0.1709 -39.5260
89.1000 -38.4700 0.1062 -0.2504 -
0.0233 0.2030 -0.2778 0.4318 1.1465
-0.4736 0.1689 -39.5244

89.1200 -38.8000 -0.1702 -0.3102 -
0.0675 0.1862 -0.2464 0.4351 1.1488
-0.4686 0.1669 -39.5228
89.1400 -38.4300 0.0448 -0.1420 -
0.0611 0.1571 -0.2139 0.4380 1.1505
-0.4635 0.1650 -39.5212
89.1600 -38.2400 -0.0510 0.0650 -
0.0280 0.1196 -0.1806 0.4405 1.1517
-0.4585 0.1631 -39.5196
89.1800 -37.9500 0.1082 0.2108 -
0.0021 0.0784 -0.1465 0.4426 1.1522
-0.4534 0.1611 -39.5180
89.2000 -38.4000 -0.2427 0.2717 -
0.0119 0.0384 -0.1121 0.4443 1.1522
-0.4483 0.1592 -39.5164
89.2200 -37.7900 0.1740 0.2276 -
0.0639 0.0034 -0.0773 0.4456 1.1516
-0.4433 0.1573 -39.5148
89.2400 -38.1700 0.1204 0.0461 -
0.1387 -0.0245 -0.0425 0.4463 1.1505
-0.4382 0.1554 -39.5132
89.2600 -38.6700 -0.1583 -0.1949 -
0.2006 -0.0435 -0.0078 0.4465 1.1488
-0.4332 0.1535 -39.5117
89.2800 -38.9900 -0.2153 -0.3098 -
0.2169 -0.0527 0.0267 0.4461 1.1465
-0.4281 0.1517 -39.5101
89.3000 -38.5500 -0.0362 -0.2087 -
0.1718 -0.0523 0.0608 0.4451 1.1437
-0.4231 0.1498 -39.5085
89.3200 -38.0400 0.2036 -0.0084 -
0.0750 -0.0434 0.0945 0.4435 1.1403
-0.4180 0.1479 -39.5069
89.3400 -38.1300 -0.1517 0.1121
0.0456 -0.0285 0.1276 0.4412 1.1365
-0.4130 0.1461 -39.5053
89.3600 -37.5700 0.1745 0.1406
0.1530 -0.0102 0.1600 0.4382 1.1320
-0.4079 0.1443 -39.5037
89.3800 -37.7800 -0.1436 0.1287
0.2174 0.0089 0.1916 0.4344 1.1271
-0.4029 0.1424 -39.5021
89.4000 -37.7400 0.0969 0.0856
0.2236 0.0263 0.2223 0.4298 1.1216
-0.3979 0.1406 -39.5005
89.4200 -37.9400 -0.1273 0.0135
0.1721 0.0403 0.2520 0.4245 1.1157
-0.3928 0.1388 -39.4989
89.4400 -37.7500 0.1730 -0.0732
0.0805 0.0502 0.2806 0.4182 1.1092
-0.3878 0.1370 -39.4974

89.4600 -38.1200 -0.0621 -0.1538 -
0.0262 0.0555 0.3080 0.4111 1.1023
-0.3828 0.1352 -39.4958
89.4800 -38.0200 0.0942 -0.1425 -
0.1225 0.0561 0.3339 0.4030 1.0948
-0.3778 0.1335 -39.4942
89.5000 -38.4000 -0.3743 0.0486 -
0.1843 0.0522 0.3582 0.3940 1.0869
-0.3728 0.1317 -39.4926
89.5200 -37.2600 0.5839 0.1932 -
0.1998 0.0439 0.3806 0.3841 1.0786
-0.3678 0.1299 -39.4910
89.5400 -38.0700 -0.2055 0.0739 -
0.1698 0.0316 0.4008 0.3732 1.0697
-0.3628 0.1282 -39.4894
89.5600 -38.4000 -0.3737 -0.1125 -
0.1032 0.0163 0.4186 0.3613 1.0605
-0.3578 0.1265 -39.4878
89.5800 -37.9200 0.1648 -0.1611 -
0.0130 -0.0009 0.4337 0.3485 1.0508
-0.3529 0.1247 -39.4863
89.6000 -37.7000 0.0653 -0.0717
0.0877 -0.0187 0.4458 0.3347 1.0407
-0.3479 0.1230 -39.4847
89.6200 -37.6600 -0.0239 0.0791
0.1838 -0.0359 0.4548 0.3201 1.0302
-0.3429 0.1213 -39.4831
89.6400 -37.5100 -0.1313 0.2068
0.2595 -0.0511 0.4603 0.3045 1.0193
-0.3380 0.1196 -39.4815
89.6600 -37.3600 0.2849 0.1562
0.3045 -0.0633 0.4622 0.2881 1.0080
-0.3331 0.1180 -39.4799
89.6800 -38.0100 -0.1543 -0.0892
0.3113 -0.0713 0.4602 0.2708 0.9963
-0.3281 0.1163 -39.4784
89.7000 -38.1200 -0.1405 -0.2448
0.2685 -0.0737 0.4542 0.2528 0.9842
-0.3232 0.1146 -39.4768
89.7200 -38.0200 0.0651 -0.0644
0.1676 -0.0695 0.4441 0.2341 0.9719
-0.3183 0.1130 -39.4752
89.7400 -38.1600 -0.3967 0.2916
0.0133 -0.0581 0.4299 0.2147 0.9591
-0.3134 0.1114 -39.4736
89.7600 -37.2000 0.7684 0.3661 -
0.1740 -0.0395 0.4117 0.1947 0.9461
-0.3085 0.1097 -39.4720
89.7800 -38.7900 -0.3136 0.0218 -
0.3573 -0.0140 0.3896 0.1741 0.9327
-0.3037 0.1081 -39.4705

89.8000 -39.4600 -0.4540 -0.3431 -
0.4921 0.0181 0.3638 0.1531 0.9190
-0.2988 0.1065 -39.4689
89.8200 -39.1700 -0.0359 -0.3705 -
0.5368 0.0558 0.3344 0.1316 0.9050
-0.2940 0.1049 -39.4673
89.8400 -38.4000 0.4218 -0.1474 -
0.4751 0.0976 0.3017 0.1098 0.8908
-0.2892 0.1034 -39.4657
89.8600 -38.6700 -0.1930 0.0647 -
0.3200 0.1405 0.2661 0.0876 0.8762
-0.2844 0.1018 -39.4642
89.8800 -38.0600 0.1238 0.1541 -
0.1016 0.1810 0.2279 0.0652 0.8614
-0.2796 0.1002 -39.4626
89.9000 -38.3100 -0.1545 0.1309
0.1399 0.2149 0.1873 0.0427 0.8464
-0.2748 0.0987 -39.4610
89.9200 -37.8500 0.2008 0.0013
0.3647 0.2382 0.1448 0.0200 0.8311
-0.2700 0.0972 -39.4594
89.9400 -38.2600 -0.3701 -0.0729
0.5348 0.2467 0.1007 -0.0026 0.8156
-0.2653 0.0956 -39.4579
89.9600 -37.6300 0.1914 0.0418
0.6181 0.2366 0.0554 -0.0253 0.7999
-0.2605 0.0941 -39.4563
89.9800 -37.8300 0.1005 0.2153
0.5991 0.2065 0.0095 -0.0478 0.7840
-0.2558 0.0926 -39.4547
90.0000 -38.2600 -0.2243 0.2802
0.4776 0.1586 -0.0364 -0.0702 0.7678
-0.2511 0.0911 -39.4532
90.0200 -38.1400 0.2448 0.1886
0.2702 0.0975 -0.0819 -0.0923 0.7515
-0.2465 0.0897 -39.4516
90.0400 -39.1500 -0.0653 0.0189
0.0135 0.0284 -0.1263 -0.1142 0.7351
-0.2418 0.0882 -39.4500
90.0600 -39.9800 -0.2582 -0.1387 -
0.2408 -0.0434 -0.1691 -0.1356 0.7184
-0.2372 0.0868 -39.4485
90.0800 -39.8800 0.2035 -0.2566 -
0.4375 -0.1128 -0.2096 -0.1566 0.7017
-0.2325 0.0853 -39.4469
90.1000 -40.2100 0.1521 -0.3245 -
0.5380 -0.1746 -0.2473 -0.1772 0.6847
-0.2279 0.0839 -39.4453
90.1200 -40.8400 -0.4474 -0.2441 -
0.5258 -0.2245 -0.2816 -0.1971 0.6677
-0.2234 0.0825 -39.4438

90.1400 -39.7200 0.2777 0.0352 -
0.4139 -0.2600 -0.3119 -0.2164 0.6506
-0.2188 0.0811 -39.4422
90.1600 -39.7500 0.1567 0.2913 -
0.2443 -0.2809 -0.3377 -0.2350 0.6333
-0.2143 0.0797 -39.4406
90.1800 -39.8900 -0.3490 0.2893 -
0.0614 -0.2877 -0.3587 -0.2528 0.6160
-0.2098 0.0783 -39.4391
90.2000 -39.2200 0.4291 0.0797
0.0998 -0.2811 -0.3745 -0.2698 0.5986
-0.2053 0.0769 -39.4375
90.2200 -40.0900 -0.3822 -0.1079
0.2126 -0.2622 -0.3850 -0.2859 0.5811
-0.2008 0.0756 -39.4359
90.2400 -39.4900 0.3680 -0.1301
0.2641 -0.2323 -0.3902 -0.3010 0.5635
-0.1963 0.0742 -39.4344
90.2600 -40.0900 -0.4079 -0.0425
0.2569 -0.1930 -0.3901 -0.3152 0.5459
-0.1919 0.0729 -39.4328
90.2800 -39.6400 0.0945 0.0784
0.2016 -0.1467 -0.3852 -0.3283 0.5283
-0.1875 0.0716 -39.4313
90.3000 -39.6500 0.0251 0.1048
0.1155 -0.0962 -0.3756 -0.3403 0.5107
-0.1832 0.0703 -39.4297
90.3200 -39.6400 0.1866 -0.0131
0.0176 -0.0438 -0.3619 -0.3513 0.4930
-0.1788 0.0690 -39.4281
90.3400 -40.2200 -0.3429 -0.1403 -
0.0682 0.0080 -0.3444 -0.3612 0.4754
-0.1745 0.0677 -39.4266
90.3600 -39.8400 0.2763 -0.1084 -
0.1182 0.0569 -0.3237 -0.3700 0.4577
-0.1702 0.0664 -39.4250
90.3800 -40.0000 -0.3964 0.0370 -
0.1263 0.1009 -0.3002 -0.3777 0.4401
-0.1659 0.0652 -39.4235
90.4000 -39.2500 0.2966 0.1381 -
0.0983 0.1383 -0.2744 -0.3843 0.4225
-0.1617 0.0639 -39.4219
90.4200 -39.4900 0.1184 0.0931 -
0.0477 0.1676 -0.2468 -0.3898 0.4050
-0.1574 0.0627 -39.4203
90.4400 -39.5300 -0.0240 -0.0465
0.0123 0.1879 -0.2178 -0.3942 0.3875
-0.1533 0.0615 -39.4188
90.4600 -39.8100 -0.2821 -0.0979
0.0657 0.1988 -0.1881 -0.3976 0.3700
-0.1491 0.0603 -39.4172

90.4800 -39.0700 0.2851 -0.0042
0.0947 0.2003 -0.1579 -0.4000 0.3527
-0.1450 0.0591 -39.4157
90.5000 -39.2800 -0.0899 0.1395
0.0902 0.1932 -0.1277 -0.4013 0.3354
-0.1408 0.0579 -39.4141
90.5200 -39.3000 -0.0951 0.2000
0.0579 0.1784 -0.0980 -0.4016 0.3181
-0.1368 0.0567 -39.4126
90.5400 -39.2000 0.1541 0.0846
0.0104 0.1573 -0.0691 -0.4010 0.3010
-0.1327 0.0556 -39.4110
90.5600 -39.6700 0.0926 -0.1145 -
0.0337 0.1320 -0.0411 -0.3994 0.2840
-0.1287 0.0544 -39.4095
90.5800 -39.8900 -0.0657 -0.2222 -
0.0576 0.1043 -0.0145 -0.3969 0.2671
-0.1247 0.0533 -39.4079
90.6000 -40.0100 -0.3049 -0.1354 -
0.0552 0.0755 0.0109 -0.3936 0.2503
-0.1208 0.0522 -39.4064
90.6200 -38.9500 0.5478 0.0531 -
0.0314 0.0467 0.0347 -0.3894 0.2336
-0.1169 0.0510 -39.4048
90.6400 -39.8800 -0.4543 0.1276 -
0.0004 0.0190 0.0571 -0.3845 0.2170
-0.1130 0.0499 -39.4033
90.6600 -39.0700 0.4375 0.0725
0.0199 -0.0068 0.0780 -0.3788 0.2006
-0.1091 0.0489 -39.4017
90.6800 -39.9900 -0.4719 0.0175
0.0227 -0.0304 0.0973 -0.3725 0.1843
-0.1053 0.0478 -39.4002
90.7000 -39.3500 0.2652 0.0296
0.0134 -0.0514 0.1151 -0.3654 0.1681
-0.1015 0.0467 -39.3986
90.7200 -39.3100 0.2107 0.0386
0.0029 -0.0695 0.1314 -0.3578 0.1521
-0.0977 0.0457 -39.3971
90.7400 -40.0400 -0.4061 -0.0172 -
0.0056 -0.0842 0.1464 -0.3497 0.1362
-0.0940 0.0446 -39.3955
90.7600 -39.5200 0.2577 -0.0538 -
0.0114 -0.0949 0.1601 -0.3410 0.1205
-0.0903 0.0436 -39.3940
90.7800 -39.6200 0.0426 -0.0597 -
0.0197 -0.1013 0.1726 -0.3318 0.1049
-0.0867 0.0426 -39.3925
90.8000 -39.7700 -0.0226 -0.0168 -
0.0338 -0.1033 0.1840 -0.3223 0.0895
-0.0831 0.0416 -39.3909

90.8200 -39.6600 -0.1705 0.0831 -
0.0508 -0.1013 0.1942 -0.3124 0.0742
-0.0795 0.0406 -39.3894
90.8400 -39.3400 0.1635 0.1516 -
0.0638 -0.0958 0.2034 -0.3021 0.0592
-0.0759 0.0396 -39.3878
90.8600 -39.3300 0.2898 0.0381 -
0.0624 -0.0872 0.2115 -0.2916 0.0443
-0.0724 0.0387 -39.3863
90.8800 -40.0700 -0.2828 -0.1880 -
0.0376 -0.0759 0.2183 -0.2809 0.0296
-0.0689 0.0377 -39.3847
90.9000 -39.8800 -0.2422 -0.2563
0.0115 -0.0625 0.2238 -0.2699 0.0150
-0.0655 0.0368 -39.3832
90.9200 -39.0400 0.4219 -0.0949
0.0724 -0.0476 0.2280 -0.2589 0.0007
-0.0621 0.0358 -39.3817
90.9400 -39.5300 -0.3262 0.1274
0.1226 -0.0319 0.2307 -0.2476 -0.0135
-0.0588 0.0349 -39.3801
90.9600 -39.1500 -0.0108 0.2311
0.1420 -0.0158 0.2318 -0.2364 -0.0275
-0.0554 0.0340 -39.3786
90.9800 -38.7800 0.3819 0.1478
0.1205 -0.0002 0.2314 -0.2250 -0.0412
-0.0522 0.0331 -39.3770
91.0000 -39.6200 -0.3069 -0.0272
0.0642 0.0142 0.2294 -0.2137 -0.0548
-0.0489 0.0322 -39.3755
91.0200 -39.5900 -0.0621 -0.1208 -
0.0052 0.0273 0.2256 -0.2024 -0.0682
-0.0457 0.0314 -39.3740
91.0400 -39.3000 0.2325 -0.0995 -
0.0678 0.0397 0.2201 -0.1912 -0.0813
-0.0426 0.0305 -39.3724
91.0600 -39.7000 -0.0810 -0.0478 -
0.1090 0.0518 0.2127 -0.1800 -0.0943
-0.0394 0.0297 -39.3709
91.0800 -39.4200 0.0787 0.0059 -
0.1234 0.0640 0.2034 -0.1690 -0.1070
-0.0364 0.0288 -39.3694
91.1000 -39.4300 -0.0541 0.0494 -
0.1139 0.0766 0.1922 -0.1581 -0.1196
-0.0333 0.0280 -39.3678
91.1200 -39.3200 0.1214 0.0263 -
0.0874 0.0890 0.1793 -0.1474 -0.1319
-0.0303 0.0272 -39.3663
91.1400 -39.5400 -0.1119 -0.0760 -
0.0455 0.1005 0.1646 -0.1369 -0.1440
-0.0274 0.0264 -39.3648

91.1600	-39.5700	-0.0590	-0.1550	
0.0085	0.1100	0.1483	-0.1265	-0.1559
	-0.0244	0.0256	-39.3632	
91.1800	-39.6800	-0.1989	-0.1035	
0.0674	0.1163	0.1306	-0.1164	-0.1676
	-0.0216	0.0248	-39.3617	
91.2000	-38.9800	0.2157	0.0832	
0.1136	0.1186	0.1116	-0.1064	-0.1791
	-0.0187	0.0241	-39.3602	
91.2200	-39.3900	-0.3863	0.2499	
0.1292	0.1161	0.0916	-0.0967	-0.1904
	-0.0159	0.0233	-39.3587	
91.2400	-38.7600	0.4055	0.2386	
0.1086	0.1084	0.0710	-0.0873	-0.2015
	-0.0132	0.0226	-39.3571	
91.2600	-39.3900	0.0083	-0.0361	
0.0607	0.0960	0.0499	-0.0780	-0.2123
	-0.0105	0.0218	-39.3556	
91.2800	-39.8700	-0.0566	-0.3650	
0.0078	0.0799	0.0286	-0.0690	-0.2230
	-0.0078	0.0211	-39.3541	
91.3000	-40.4000	-0.5374	-0.3070	-
0.0278	0.0610	0.0075	-0.0602	-0.2334
	-0.0052	0.0204	-39.3525	
91.3200	-39.0100	0.5323	0.0940	-
0.0362	0.0406	-0.0131	-0.0517	-0.2437
	-0.0026	0.0197	-39.3510	
91.3400	-39.4100	0.0085	0.3428	-
0.0270	0.0195	-0.0329	-0.0434	-0.2537
	0.0000	0.0191	-39.3495	
91.3600	-39.5300	-0.0939	0.2408	-
0.0137	-0.0012	-0.0517	-0.0354	-0.2636
	0.0025	0.0184	-39.3480	
91.3800	-39.6300	0.0897	-0.0358	-
0.0027	-0.0214	-0.0690	-0.0276	-0.2733
	0.0050	0.0177	-39.3465	
91.4000	-39.9700	-0.0635	-0.2207	
0.0035	-0.0410	-0.0847	-0.0201	-0.2827
	0.0075	0.0171	-39.3449	
91.4200	-40.0000	-0.0600	-0.1948	
0.0103	-0.0604	-0.0985	-0.0128	-0.2920
	0.0099	0.0164	-39.3434	
91.4400	-39.7800	0.0257	-0.0058	
0.0183	-0.0799	-0.1101	-0.0058	-0.3011
	0.0122	0.0158	-39.3419	
91.4600	-39.7000	-0.0294	0.1537	
0.0264	-0.0995	-0.1193	0.0010	-0.3100
	0.0146	0.0152	-39.3404	
91.4800	-39.7200	0.0092	0.1360	
0.0305	-0.1192	-0.1259	0.0075	-0.3187
	0.0169	0.0146	-39.3388	

91.5000 -39.4600 0.4037 -0.0278
0.0264 -0.1385 -0.1299 0.0138 -0.3273
0.0191 0.0140 -39.3373
91.5200 -40.6300 -0.5748 -0.1229
0.0165 -0.1561 -0.1313 0.0198 -0.3356
0.0213 0.0134 -39.3358
91.5400 -39.5000 0.3083 -0.0196
0.0081 -0.1701 -0.1301 0.0257 -0.3438
0.0235 0.0129 -39.3343
91.5600 -39.4300 0.3303 0.1054
0.0010 -0.1783 -0.1264 0.0313 -0.3518
0.0257 0.0123 -39.3328
91.5800 -40.1400 -0.2522 0.0845 -
0.0100 -0.1785 -0.1205 0.0367 -0.3596
0.0278 0.0117 -39.3313
91.6000 -39.7800 0.2329 -0.0152 -
0.0312 -0.1686 -0.1125 0.0420 -0.3673
0.0299 0.0112 -39.3297
91.6200 -40.2700 -0.2894 -0.0509 -
0.0683 -0.1467 -0.1028 0.0470 -0.3748
0.0319 0.0107 -39.3282
91.6400 -39.8700 0.2045 -0.0123 -
0.1165 -0.1124 -0.0917 0.0518 -0.3821
0.0339 0.0102 -39.3267
91.6600 -39.8900 -0.0314 0.0021 -
0.1569 -0.0671 -0.0793 0.0565 -0.3893
0.0359 0.0097 -39.3252
91.6800 -39.7400 0.1383 -0.0588 -
0.1657 -0.0138 -0.0662 0.0609 -0.3963
0.0379 0.0092 -39.3237
91.7000 -40.1200 -0.2804 -0.0984 -
0.1300 0.0441 -0.0526 0.0652 -0.4032
0.0398 0.0087 -39.3222
91.7200 -39.5000 0.1177 -0.0549 -
0.0502 0.1027 -0.0389 0.0692 -0.4099
0.0417 0.0082 -39.3207
91.7400 -39.2400 0.1074 -0.0227
0.0571 0.1575 -0.0254 0.0731 -0.4165
0.0435 0.0078 -39.3192
91.7600 -39.4600 -0.1216 -0.0310
0.1647 0.2042 -0.0124 0.0767 -0.4229
0.0454 0.0073 -39.3176
91.7800 -39.3100 -0.1995 0.0438
0.2423 0.2389 -0.0004 0.0802 -0.4292
0.0472 0.0069 -39.3161
91.8000 -38.4200 0.3756 0.1691
0.2685 0.2584 0.0104 0.0834 -0.4353
0.0489 0.0064 -39.3146
91.8200 -39.0500 -0.1069 0.1678
0.2324 0.2611 0.0197 0.0864 -0.4413
0.0507 0.0060 -39.3131

91.8400 -39.0100 0.0351 0.0587
0.1440 0.2474 0.0274 0.0892 -0.4472
0.0524 0.0056 -39.3116
91.8600 -39.6000 -0.2118 -0.0429
0.0294 0.2198 0.0336 0.0917 -0.4529
0.0541 0.0052 -39.3101
91.8800 -39.2500 0.4159 -0.1122 -
0.0827 0.1818 0.0381 0.0940 -0.4585
0.0557 0.0048 -39.3086
91.9000 -40.0800 -0.3420 -0.0915 -
0.1666 0.1368 0.0411 0.0961 -0.4639
0.0574 0.0045 -39.3071
91.9200 -39.8100 -0.0904 -0.0030 -
0.2078 0.0889 0.0428 0.0979 -0.4692
0.0590 0.0041 -39.3056
91.9400 -39.2600 0.3737 0.0395 -
0.2084 0.0411 0.0431 0.0994 -0.4744
0.0605 0.0037 -39.3041
91.9600 -40.1200 -0.1939 -0.0226 -
0.1760 -0.0038 0.0424 0.1006 -0.4795
0.0621 0.0034 -39.3026
91.9800 -39.7700 0.0104 -0.0864 -
0.1221 -0.0436 0.0406 0.1016 -0.4844
0.0636 0.0031 -39.3011
92.0000 -39.7000 0.0079 -0.0513 -
0.0571 -0.0766 0.0381 0.1022 -0.4893
0.0652 0.0027 -39.2996
92.0200 -39.5500 0.1071 0.0201
0.0092 -0.1023 0.0351 0.1026 -0.4940
0.0667 0.0024 -39.2981
92.0400 -39.6300 -0.1434 0.0578
0.0690 -0.1212 0.0318 0.1027 -0.4986
0.0681 0.0021 -39.2966
92.0600 -39.3600 0.2201 0.0403
0.1174 -0.1341 0.0285 0.1024 -0.5031
0.0696 0.0018 -39.2951
92.0800 -39.7600 -0.1799 -0.0143
0.1483 -0.1422 0.0255 0.1019 -0.5075
0.0710 0.0016 -39.2936
92.1000 -39.3500 0.2356 -0.0806
0.1597 -0.1464 0.0229 0.1011 -0.5118
0.0725 0.0013 -39.2921
92.1200 -40.0500 -0.3534 -0.0498
0.1475 -0.1479 0.0209 0.0999 -0.5160
0.0739 0.0010 -39.2906
92.1400 -39.5200 -0.0008 0.1494
0.1064 -0.1470 0.0197 0.0985 -0.5200
0.0752 0.0008 -39.2891
92.1600 -39.2100 0.2182 0.2824
0.0354 -0.1439 0.0193 0.0967 -0.5240
0.0766 0.0005 -39.2876

92.1800 -39.4300 0.2506 0.1552 -
0.0560 -0.1384 0.0198 0.0946 -0.5279
0.0780 0.0003 -39.2861
92.2000 -40.1200 -0.0433 -0.1157 -
0.1474 -0.1301 0.0213 0.0922 -0.5317
0.0793 0.0001 -39.2846
92.2200 -40.8100 -0.5523 -0.2866 -
0.2121 -0.1182 0.0237 0.0894 -0.5354
0.0807 -0.0001 -39.2831
92.2400 -39.7800 0.3760 -0.2943 -
0.2253 -0.1020 0.0269 0.0864 -0.5390
0.0820 -0.0004 -39.2816
92.2600 -39.6300 0.3981 -0.2653 -
0.1766 -0.0811 0.0310 0.0831 -0.5425
0.0833 -0.0005 -39.2801
92.2800 -40.6000 -0.6224 -0.1639 -
0.0803 -0.0560 0.0358 0.0795 -0.5459
0.0846 -0.0007 -39.2787
92.3000 -39.6700 -0.1631 0.1173
0.0327 -0.0283 0.0411 0.0756 -0.5493
0.0859 -0.0009 -39.2772
92.3200 -38.9800 0.2158 0.3877
0.1294 0.0005 0.0468 0.0714 -0.5525
0.0872 -0.0011 -39.2757
92.3400 -38.8200 0.1729 0.3866
0.1789 0.0286 0.0527 0.0669 -0.5557
0.0884 -0.0012 -39.2742
92.3600 -39.3200 -0.0221 0.1404
0.1668 0.0545 0.0586 0.0622 -0.5588
0.0897 -0.0014 -39.2727
92.3800 -39.8600 -0.3092 -0.0961
0.1078 0.0764 0.0643 0.0573 -0.5618
0.0910 -0.0015 -39.2712
92.4000 -39.5800 0.1666 -0.2526
0.0354 0.0936 0.0695 0.0521 -0.5647
0.0922 -0.0016 -39.2697
92.4200 -39.5300 0.3768 -0.3517 -
0.0159 0.1056 0.0741 0.0468 -0.5676
0.0934 -0.0018 -39.2682
92.4400 -40.7000 -0.8942 -0.2245 -
0.0281 0.1124 0.0778 0.0413 -0.5703
0.0947 -0.0019 -39.2668
92.4600 -38.8200 0.5566 0.1492 -
0.0012 0.1145 0.0804 0.0356 -0.5730
0.0959 -0.0020 -39.2653
92.4800 -38.5700 0.6102 0.3458
0.0405 0.1127 0.0819 0.0297 -0.5756
0.0972 -0.0021 -39.2638
92.5000 -39.4500 -0.1293 0.1747
0.0675 0.1084 0.0820 0.0237 -0.5781
0.0984 -0.0021 -39.2623

92.5200 -40.1300 -0.6292 -0.0803
0.0641 0.1030 0.0807 0.0177 -0.5806
0.0996 -0.0022 -39.2608
92.5400 -39.2000 0.4457 -0.1262
0.0248 0.0985 0.0780 0.0115 -0.5830
0.1008 -0.0023 -39.2594
92.5600 -39.8900 -0.2644 0.0163 -
0.0441 0.0961 0.0739 0.0052 -0.5853
0.1021 -0.0023 -39.2579
92.5800 -39.5100 0.0282 0.1351 -
0.1214 0.0963 0.0683 -0.0010 -0.5875
0.1033 -0.0024 -39.2564
92.6000 -39.3800 0.3461 0.0696 -
0.1831 0.0988 0.0613 -0.0073 -0.5896
0.1045 -0.0024 -39.2549
92.6200 -40.1400 -0.2525 -0.1225 -
0.2096 0.1028 0.0531 -0.0136 -0.5917
0.1057 -0.0024 -39.2534
92.6400 -40.2400 -0.2789 -0.1876 -
0.1872 0.1072 0.0436 -0.0198 -0.5937
0.1070 -0.0024 -39.2520
92.6600 -39.4800 0.3219 -0.0510 -
0.1155 0.1103 0.0332 -0.0260 -0.5957
0.1082 -0.0025 -39.2505
92.6800 -39.5500 -0.0603 0.0912 -
0.0105 0.1107 0.0219 -0.0320 -0.5975
0.1094 -0.0025 -39.2490
92.7000 -39.5800 -0.1086 0.1230
0.1009 0.1073 0.0099 -0.0379 -0.5993
0.1107 -0.0024 -39.2475
92.7200 -39.1900 0.2851 0.0416
0.1904 0.0989 -0.0025 -0.0437 -0.6011
0.1119 -0.0024 -39.2461
92.7400 -39.6300 -0.1043 -0.0496
0.2377 0.0847 -0.0152 -0.0493 -0.6028
0.1131 -0.0024 -39.2446
92.7600 -39.9400 -0.2954 -0.0529
0.2305 0.0646 -0.0279 -0.0547 -0.6044
0.1144 -0.0024 -39.2431
92.7800 -39.2000 0.4435 0.0218
0.1706 0.0392 -0.0402 -0.0598 -0.6059
0.1156 -0.0023 -39.2416
92.8000 -40.0600 -0.4536 0.1280
0.0709 0.0096 -0.0520 -0.0647 -0.6074
0.1169 -0.0023 -39.2402
92.8200 -39.5500 0.2647 0.1855 -
0.0482 -0.0221 -0.0631 -0.0693 -0.6088
0.1182 -0.0022 -39.2387
92.8400 -39.9900 0.0049 0.0785 -
0.1584 -0.0539 -0.0730 -0.0737 -0.6101
0.1194 -0.0021 -39.2372

92.8600 -40.4700 -0.0608 -0.1392 -
0.2306 -0.0836 -0.0818 -0.0776 -0.6114
0.1207 -0.0021 -39.2358
92.8800 -40.6900 -0.2265 -0.2817 -
0.2429 -0.1088 -0.0891 -0.0813 -0.6126
0.1220 -0.0020 -39.2343
92.9000 -40.1300 0.3472 -0.2478 -
0.1876 -0.1279 -0.0949 -0.0845 -0.6138
0.1233 -0.0019 -39.2328
92.9200 -40.5800 -0.3017 -0.0569 -
0.0825 -0.1406 -0.0989 -0.0874 -0.6149
0.1246 -0.0018 -39.2314
92.9400 -39.9200 -0.0676 0.1704
0.0392 -0.1467 -0.1012 -0.0898 -0.6160
0.1259 -0.0017 -39.2299
92.9600 -39.1800 0.4286 0.2374
0.1414 -0.1468 -0.1017 -0.0918 -0.6169
0.1273 -0.0015 -39.2284
92.9800 -40.0200 -0.3556 0.1205
0.1999 -0.1414 -0.1005 -0.0934 -0.6179
0.1286 -0.0014 -39.2270
93.0000 -39.8000 0.0810 -0.0103
0.2081 -0.1308 -0.0976 -0.0945 -0.6187
0.1300 -0.0013 -39.2255
93.0200 -39.9700 0.0122 -0.0677
0.1705 -0.1159 -0.0932 -0.0951 -0.6196
0.1313 -0.0011 -39.2241
93.0400 -39.9800 -0.0469 -0.0667
0.1020 -0.0971 -0.0873 -0.0953 -0.6203
0.1327 -0.0010 -39.2226
93.0600 -40.0800 -0.0323 -0.0310
0.0226 -0.0752 -0.0801 -0.0950 -0.6210
0.1341 -0.0008 -39.2211
93.0800 -39.9300 0.0728 -0.0138 -
0.0484 -0.0510 -0.0718 -0.0943 -0.6217
0.1355 -0.0007 -39.2197
93.1000 -39.7400 0.2841 -0.0596 -
0.0987 -0.0253 -0.0627 -0.0932 -0.6223
0.1370 -0.0005 -39.2182
93.1200 -40.5100 -0.4967 -0.0555 -
0.1267 0.0011 -0.0530 -0.0916 -0.6228
0.1384 -0.0003 -39.2168
93.1400 -39.7100 0.1053 0.1128 -
0.1358 0.0275 -0.0430 -0.0897 -0.6233
0.1399 -0.0001 -39.2153
93.1600 -39.4100 0.1562 0.2156 -
0.1251 0.0530 -0.0329 -0.0873 -0.6238
0.1413 0.0001 -39.2138
93.1800 -39.4400 0.4383 -0.0110 -
0.0941 0.0765 -0.0230 -0.0846 -0.6242
0.1428 0.0003 -39.2124

93.2000 -40.7200 -0.5634 -0.3016 -
0.0385 0.0965 -0.0135 -0.0815 -0.6245
0.1444 0.0005 -39.2109
93.2200 -40.1800 -0.3028 -0.2869
0.0387 0.1116 -0.0046 -0.0781 -0.6248
0.1459 0.0007 -39.2095
93.2400 -39.0000 0.6598 -0.0319
0.1234 0.1206 0.0034 -0.0744 -0.6251
0.1474 0.0009 -39.2080
93.2600 -39.2000 -0.0251 0.2068
0.1940 0.1222 0.0105 -0.0704 -0.6253
0.1490 0.0012 -39.2066
93.2800 -39.5700 -0.4841 0.3035
0.2267 0.1164 0.0163 -0.0661 -0.6255
0.1506 0.0014 -39.2051
93.3000 -38.7100 0.4734 0.2084
0.2070 0.1040 0.0210 -0.0616 -0.6256
0.1522 0.0017 -39.2037
93.3200 -39.6300 -0.0739 0.0239
0.1351 0.0868 0.0243 -0.0569 -0.6257
0.1538 0.0019 -39.2022
93.3400 -40.1400 -0.4201 -0.1055
0.0282 0.0672 0.0264 -0.0520 -0.6257
0.1554 0.0022 -39.2008
93.3600 -39.3300 0.5416 -0.1118 -
0.0837 0.0475 0.0272 -0.0470 -0.6257
0.1571 0.0025 -39.1993
93.3800 -40.3200 -0.3174 -0.0874 -
0.1708 0.0300 0.0268 -0.0418 -0.6257
0.1588 0.0028 -39.1979
93.4000 -40.0300 -0.1035 -0.0935 -
0.2131 0.0169 0.0251 -0.0365 -0.6257
0.1605 0.0030 -39.1964
93.4200 -39.4700 0.4360 -0.0856 -
0.2098 0.0092 0.0222 -0.0312 -0.6256
0.1622 0.0033 -39.1950
93.4400 -40.3700 -0.5223 -0.0208 -
0.1740 0.0078 0.0183 -0.0258 -0.6254
0.1639 0.0036 -39.1935
93.4600 -39.3500 0.2615 0.1000 -
0.1239 0.0129 0.0135 -0.0204 -0.6253
0.1657 0.0039 -39.1921
93.4800 -39.5900 -0.0167 0.1914 -
0.0782 0.0236 0.0081 -0.0151 -0.6251
0.1674 0.0043 -39.1906
93.5000 -39.5500 -0.0716 0.1778 -
0.0452 0.0376 0.0023 -0.0097 -0.6248
0.1692 0.0046 -39.1892
93.5200 -39.2800 0.1843 0.0475 -
0.0159 0.0519 -0.0037 -0.0045 -0.6246
0.1710 0.0049 -39.1878

93.5400 −39.7200 −0.0222 −0.1628
0.0249 0.0634 −0.0096 0.0007 −0.6243
0.1729 0.0053 −39.1863
93.5600 −40.0000 −0.1265 −0.2881
0.0859 0.0696 −0.0152 0.0057 −0.6240
0.1747 0.0056 −39.1849
93.5800 −39.8300 −0.2683 −0.1419
0.1588 0.0684 −0.0202 0.0106 −0.6237
0.1766 0.0059 −39.1834
93.6000 −39.0300 0.1551 0.1673
0.2171 0.0591 −0.0245 0.0154 −0.6233
0.1785 0.0063 −39.1820
93.6200 −38.5800 0.4663 0.3247
0.2334 0.0416 −0.0279 0.0200 −0.6229
0.1804 0.0067 −39.1806
93.6400 −39.4500 −0.1563 0.1693
0.1950 0.0171 −0.0301 0.0243 −0.6225
0.1823 0.0070 −39.1791
93.6600 −39.5800 −0.0854 −0.0582
0.1120 −0.0126 −0.0309 0.0285 −0.6220
0.1842 0.0074 −39.1777
93.6800 −40.2400 −0.6136 −0.0297
0.0062 −0.0446 −0.0304 0.0324 −0.6216
0.1862 0.0078 −39.1763
93.7000 −39.0100 0.7118 0.0834 −
0.0947 −0.0759 −0.0282 0.0361 −0.6211
0.1882 0.0082 −39.1748
93.7200 −39.7600 0.0730 −0.0608 −
0.1620 −0.1039 −0.0245 0.0394 −0.6206
0.1902 0.0086 −39.1734
93.7400 −40.5600 −0.4856 −0.3143 −
0.1814 −0.1257 −0.0191 0.0425 −0.6200
0.1922 0.0090 −39.1720
93.7600 −40.1100 0.1412 −0.3065 −
0.1569 −0.1400 −0.0121 0.0453 −0.6195
0.1942 0.0094 −39.1705
93.7800 −39.7900 −0.1308 0.0334 −
0.1069 −0.1458 −0.0035 0.0478 −0.6189
0.1962 0.0098 −39.1691
93.8000 −39.3900 −0.0615 0.3560 −
0.0564 −0.1426 0.0064 0.0499 −0.6183
0.1983 0.0102 −39.1677
93.8200 −38.9500 0.4203 0.3486 −
0.0249 −0.1309 0.0176 0.0517 −0.6177
0.2004 0.0107 −39.1662
93.8400 −40.0600 −0.4048 0.0152 −
0.0128 −0.1117 0.0297 0.0531 −0.6170
0.2024 0.0111 −39.1648
93.8600 −39.6900 0.1881 −0.2528 −
0.0053 −0.0872 0.0425 0.0541 −0.6164
0.2045 0.0115 −39.1634

93.8800 -40.0600 -0.3205 -0.2232
0.0141 -0.0600 0.0557 0.0547 -0.6157
0.2067 0.0120 -39.1619
93.9000 -39.1500 0.3145 -0.0588
0.0542 -0.0323 0.0690 0.0548 -0.6149
0.2088 0.0124 -39.1605
93.9200 -39.4400 -0.0438 0.0156
0.1094 -0.0057 0.0822 0.0546 -0.6142
0.2109 0.0129 -39.1591
93.9400 -39.3400 -0.1749 0.0433
0.1579 0.0186 0.0950 0.0540 -0.6134
0.2131 0.0134 -39.1577
93.9600 -39.0400 -0.1004 0.1513
0.1734 0.0399 0.1073 0.0530 -0.6126
0.2153 0.0138 -39.1562
93.9800 -38.7700 0.1713 0.2162
0.1419 0.0576 0.1187 0.0515 -0.6118
0.2175 0.0143 -39.1548
94.0000 -39.0600 0.1441 0.0907
0.0636 0.0715 0.1290 0.0496 -0.6109
0.2197 0.0148 -39.1534
94.0200 -39.5000 -0.2284 -0.1054 -
0.0419 0.0823 0.1381 0.0474 -0.6100
0.2219 0.0153 -39.1520
94.0400 -39.2400 0.2703 -0.1454 -
0.1411 0.0908 0.1456 0.0447 -0.6091
0.2241 0.0158 -39.1506
94.0600 -39.8400 -0.3929 -0.0363 -
0.1953 0.0976 0.1513 0.0417 -0.6081
0.2263 0.0163 -39.1491
94.0800 -38.9400 0.5672 -0.0307 -
0.1829 0.1029 0.1551 0.0383 -0.6071
0.2286 0.0168 -39.1477
94.1000 -40.0000 -0.6530 -0.1194 -
0.1067 0.1063 0.1568 0.0345 -0.6061
0.2309 0.0173 -39.1463
94.1200 -38.7700 0.4297 -0.0243
0.0085 0.1074 0.1563 0.0305 -0.6050
0.2331 0.0178 -39.1449
94.1400 -39.0000 -0.0926 0.1870
0.1244 0.1055 0.1534 0.0261 -0.6038
0.2354 0.0183 -39.1435
94.1600 -39.1300 -0.3389 0.2612
0.2070 0.0994 0.1483 0.0215 -0.6027
0.2377 0.0188 -39.1421
94.1800 -38.2600 0.7276 0.0851
0.2377 0.0883 0.1409 0.0166 -0.6014
0.2400 0.0194 -39.1406
94.2000 -39.7700 -0.6578 -0.1740
0.2112 0.0725 0.1314 0.0115 -0.6001
0.2423 0.0199 -39.1392

94.2200 -39.0500 0.2183 -0.1480
0.1360 0.0534 0.1198 0.0062 -0.5988
0.2446 0.0205 -39.1378
94.2400 -39.0800 0.0770 0.0921
0.0306 0.0327 0.1065 0.0008 -0.5974
0.2470 0.0210 -39.1364
94.2600 -39.0700 0.1224 0.1779 -
0.0812 0.0123 0.0916 -0.0048 -0.5960
0.2493 0.0216 -39.1350
94.2800 -39.5500 0.0584 0.0098 -
0.1723 -0.0059 0.0753 -0.0104 -0.5945
0.2517 0.0221 -39.1336
94.3000 -40.2400 -0.3587 -0.1912 -
0.2218 -0.0199 0.0580 -0.0161 -0.5929
0.2540 0.0227 -39.1322
94.3200 -39.5300 0.2774 -0.2261 -
0.2222 -0.0288 0.0400 -0.0218 -0.5913
0.2564 0.0232 -39.1308
94.3400 -39.8800 -0.1154 -0.1112 -
0.1811 -0.0321 0.0214 -0.0275 -0.5896
0.2587 0.0238 -39.1293
94.3600 -39.6200 -0.1785 0.0840 -
0.1125 -0.0308 0.0027 -0.0332 -0.5879
0.2611 0.0244 -39.1279
94.3800 -39.0800 0.1609 0.2206 -
0.0331 -0.0265 -0.0159 -0.0388 -0.5861
0.2635 0.0250 -39.1265
94.4000 -39.4200 0.0094 0.1587
0.0436 -0.0209 -0.0341 -0.0442 -0.5842
0.2659 0.0255 -39.1251
94.4200 -39.7100 -0.1478 -0.0411
0.1126 -0.0157 -0.0515 -0.0496 -0.5822
0.2683 0.0261 -39.1237
94.4400 -39.6300 0.0003 -0.1684
0.1698 -0.0126 -0.0677 -0.0548 -0.5802
0.2707 0.0267 -39.1223
94.4600 -39.6300 -0.1533 -0.1282
0.2097 -0.0129 -0.0825 -0.0598 -0.5781
0.2731 0.0273 -39.1209
94.4800 -39.4000 -0.0319 0.0046
0.2258 -0.0175 -0.0956 -0.0645 -0.5760
0.2755 0.0279 -39.1195
94.5000 -39.1800 0.1758 0.1043
0.2119 -0.0263 -0.1067 -0.0690 -0.5737
0.2779 0.0285 -39.1181
94.5200 -39.3600 0.1331 0.1153
0.1615 -0.0385 -0.1158 -0.0733 -0.5714
0.2803 0.0291 -39.1167
94.5400 -39.7100 -0.3139 0.0856
0.0783 -0.0524 -0.1228 -0.0773 -0.5690
0.2827 0.0297 -39.1153

94.5600 -39.5000 0.1866 0.0844 -
0.0241 -0.0657 -0.1277 -0.0809 -0.5666
0.2851 0.0304 -39.1139
94.5800 -39.6100 0.1187 0.0155 -
0.1250 -0.0764 -0.1305 -0.0842 -0.5640
0.2875 0.0310 -39.1125
94.6000 -40.1300 -0.1367 -0.1256 -
0.2011 -0.0829 -0.1311 -0.0872 -0.5614
0.2899 0.0316 -39.1111
94.6200 -40.1300 -0.1401 -0.1608 -
0.2355 -0.0840 -0.1296 -0.0899 -0.5587
0.2924 0.0322 -39.1097
94.6400 -39.7700 0.1202 -0.0477 -
0.2230 -0.0791 -0.1260 -0.0922 -0.5559
0.2948 0.0329 -39.1083
94.6600 -39.5900 0.0397 0.0691 -
0.1687 -0.0678 -0.1204 -0.0941 -0.5531
0.2972 0.0335 -39.1069
94.6800 -39.7200 -0.0150 0.0611 -
0.0828 -0.0511 -0.1129 -0.0957 -0.5502
0.2996 0.0342 -39.1055
94.7000 -39.3700 0.0534 -0.0555
0.0151 -0.0308 -0.1035 -0.0970 -0.5472
0.3020 0.0348 -39.1041
94.7200 -39.5500 -0.1135 -0.1172
0.1033 -0.0090 -0.0925 -0.0979 -0.5441
0.3044 0.0355 -39.1027
94.7400 -39.5800 -0.3374 -0.0227
0.1655 0.0122 -0.0801 -0.0985 -0.5409
0.3069 0.0361 -39.1014
94.7600 -38.6200 0.6214 0.1222
0.1915 0.0307 -0.0665 -0.0988 -0.5377
0.3093 0.0368 -39.1000
94.7800 -39.0700 -0.1544 0.1703
0.1782 0.0449 -0.0519 -0.0988 -0.5344
0.3117 0.0374 -39.0986
94.8000 -39.5200 -0.4803 0.1337
0.1323 0.0539 -0.0365 -0.0985 -0.5310
0.3141 0.0381 -39.0972
94.8200 -38.7800 0.4509 0.0123
0.0711 0.0574 -0.0206 -0.0978 -0.5275
0.3165 0.0387 -39.0958
94.8400 -39.0800 0.5057 -0.1849
0.0125 0.0555 -0.0045 -0.0969 -0.5240
0.3189 0.0394 -39.0944
94.8600 -40.4100 -0.7550 -0.3009 -
0.0278 0.0494 0.0117 -0.0957 -0.5203
0.3213 0.0401 -39.0930
94.8800 -39.5300 -0.0346 -0.1389 -
0.0441 0.0405 0.0276 -0.0942 -0.5166
0.3237 0.0408 -39.0916

94.9000	-38.4600	0.6974	0.1296	-
0.0454	0.0307	0.0431	-0.0925	-0.5129
	0.3260	0.0414	-39.0902	
94.9200	-39.3600	-0.3210	0.2330	-
0.0448	0.0216	0.0579	-0.0905	-0.5091
	0.3284	0.0421	-39.0889	
94.9400	-39.4600	-0.3126	0.1694	-
0.0567	0.0149	0.0718	-0.0882	-0.5052
	0.3308	0.0428	-39.0875	
94.9600	-38.7000	0.5280	0.0666	-
0.0869	0.0117	0.0845	-0.0857	-0.5012
	0.3332	0.0435	-39.0861	
94.9800	-39.4100	-0.1435	-0.0278	-
0.1257	0.0123	0.0958	-0.0830	-0.4972
	0.3355	0.0442	-39.0847	
95.0000	-39.5800	-0.1857	-0.0788	-
0.1510	0.0162	0.1057	-0.0801	-0.4931
	0.3379	0.0449	-39.0833	
95.0200	-39.1900	0.1079	-0.0709	-
0.1391	0.0227	0.1139	-0.0769	-0.4889
	0.3402	0.0456	-39.0819	
95.0400	-39.4200	-0.0830	-0.0804	-
0.0751	0.0307	0.1203	-0.0736	-0.4847
	0.3425	0.0463	-39.0806	
95.0600	-39.0400	0.1733	-0.1732	
0.0363	0.0388	0.1248	-0.0701	-0.4805
	0.3449	0.0470	-39.0792	
95.0800	-39.2000	-0.1185	-0.2239	
0.1632	0.0458	0.1273	-0.0663	-0.4762
	0.3472	0.0477	-39.0778	
95.1000	-38.8400	-0.1647	-0.0415	
0.2612	0.0504	0.1279	-0.0625	-0.4718
	0.3495	0.0484	-39.0764	
95.1200	-38.7800	-0.3851	0.3484	
0.2933	0.0518	0.1266	-0.0584	-0.4674
	0.3518	0.0491	-39.0751	
95.1400	-37.5700	0.8364	0.4971	
0.2416	0.0498	0.1233	-0.0542	-0.4630
	0.3541	0.0498	-39.0737	
95.1600	-39.5100	-0.6788	0.0821	
0.1135	0.0450	0.1183	-0.0499	-0.4585
	0.3564	0.0505	-39.0723	
95.1800	-39.0100	0.4068	-0.3559	-
0.0461	0.0386	0.1116	-0.0455	-0.4540
	0.3586	0.0512	-39.0709	
95.2000	-39.8300	-0.4688	-0.3170	-
0.1841	0.0316	0.1033	-0.0410	-0.4494
	0.3609	0.0520	-39.0696	
95.2200	-39.5400	-0.2883	0.0076	-
0.2663	0.0248	0.0936	-0.0364	-0.4448
	0.3631	0.0527	-39.0682	

95.2400 -38.3900 0.6340 0.2348 -
0.2795 0.0187 0.0827 -0.0317 -0.4402
0.3654 0.0534 -39.0668
95.2600 -39.2200 -0.2224 0.1552 -
0.2259 0.0129 0.0708 -0.0269 -0.4356
0.3676 0.0541 -39.0654
95.2800 -39.6400 -0.4471 -0.0278 -
0.1223 0.0071 0.0581 -0.0221 -0.4309
0.3698 0.0549 -39.0641
95.3000 -39.2000 0.0151 -0.1040
0.0043 0.0005 0.0449 -0.0173 -0.4263
0.3720 0.0556 -39.0627
95.3200 -38.1700 0.6629 -0.0688
0.1236 -0.0077 0.0315 -0.0124 -0.4216
0.3742 0.0563 -39.0613
95.3400 -39.1300 -0.3885 0.0350
0.2097 -0.0177 0.0180 -0.0075 -0.4169
0.3764 0.0570 -39.0600
95.3600 -38.8800 -0.2356 0.1537
0.2461 -0.0296 0.0047 -0.0026 -0.4122
0.3785 0.0578 -39.0586
95.3800 -38.0600 0.6701 0.1790
0.2242 -0.0430 -0.0080 0.0022 -0.4075
0.3807 0.0585 -39.0572
95.4000 -39.4200 -0.5541 0.0030
0.1515 -0.0573 -0.0200 0.0071 -0.4028
0.3828 0.0592 -39.0559
95.4200 -38.8300 0.3419 -0.1772
0.0582 -0.0712 -0.0311 0.0119 -0.3981
0.3849 0.0600 -39.0545
95.4400 -39.5700 -0.3987 -0.1408 -
0.0263 -0.0834 -0.0409 0.0166 -0.3933
0.3870 0.0607 -39.0531
95.4600 -38.7600 0.4201 -0.0064 -
0.0846 -0.0926 -0.0494 0.0213 -0.3887
0.3891 0.0615 -39.0518
95.4800 -39.5100 -0.3785 0.0451 -
0.1161 -0.0973 -0.0565 0.0260 -0.3840
0.3912 0.0622 -39.0504
95.5000 -39.1200 0.1097 0.0424 -
0.1268 -0.0961 -0.0621 0.0305 -0.3793
0.3932 0.0630 -39.0490
95.5200 -39.2700 -0.1028 0.0667 -
0.1231 -0.0884 -0.0662 0.0350 -0.3746
0.3953 0.0637 -39.0477
95.5400 -39.0700 0.0084 0.0435 -
0.1047 -0.0737 -0.0688 0.0394 -0.3700
0.3973 0.0644 -39.0463
95.5600 -38.7400 0.4365 -0.0974 -
0.0725 -0.0527 -0.0702 0.0437 -0.3654
0.3993 0.0652 -39.0450

95.5800	-39.7300	-0.5813	-0.1834	-
0.0270	-0.0268	-0.0703	0.0479	-0.3608
	0.4013	0.0659	-39.0436	
95.6000	-39.1100	-0.1568	-0.0393	
0.0247	0.0019	-0.0694	0.0519	-0.3563
	0.4033	0.0667	-39.0423	
95.6200	-37.9800	0.6875	0.1548	
0.0707	0.0313	-0.0677	0.0559	-0.3517
	0.4053	0.0674	-39.0409	
95.6400	-39.0000	-0.4869	0.2027	
0.0952	0.0598	-0.0651	0.0598	-0.3472
	0.4072	0.0682	-39.0395	
95.6600	-38.4700	0.2026	0.1095	
0.0901	0.0854	-0.0620	0.0635	-0.3428
	0.4091	0.0689	-39.0382	
95.6800	-38.8100	-0.0281	-0.0491	
0.0594	0.1064	-0.0583	0.0672	-0.3383
	0.4110	0.0697	-39.0368	
95.7000	-38.8500	0.0397	-0.1807	
0.0189	0.1214	-0.0543	0.0707	-0.3340
	0.4129	0.0705	-39.0355	
95.7200	-38.9800	-0.1461	-0.1760	-
0.0118	0.1293	-0.0500	0.0740	-0.3296
	0.4148	0.0712	-39.0341	
95.7400	-38.6400	0.1357	-0.0329	-
0.0187	0.1296	-0.0457	0.0773	-0.3253
	0.4166	0.0720	-39.0328	
95.7600	-38.6000	-0.0500	0.1179	-
0.0009	0.1226	-0.0413	0.0803	-0.3211
	0.4185	0.0727	-39.0314	
95.7800	-38.5900	-0.0759	0.1500	
0.0301	0.1090	-0.0370	0.0833	-0.3168
	0.4203	0.0735	-39.0301	
95.8000	-38.3800	0.2103	0.0214	
0.0592	0.0903	-0.0327	0.0860	-0.3127
	0.4221	0.0742	-39.0287	
95.8200	-38.8100	0.0375	-0.1624	
0.0763	0.0679	-0.0284	0.0886	-0.3086
	0.4238	0.0750	-39.0274	
95.8400	-39.0000	-0.2152	-0.1830	
0.0771	0.0434	-0.0242	0.0909	-0.3045
	0.4256	0.0757	-39.0260	
95.8600	-38.6800	-0.0149	0.0259	
0.0580	0.0184	-0.0199	0.0930	-0.3005
	0.4273	0.0765	-39.0247	
95.8800	-38.1600	0.2767	0.2123	
0.0195	-0.0061	-0.0154	0.0950	-0.2966
	0.4290	0.0773	-39.0233	
95.9000	-38.6200	0.0529	0.1400	-
0.0360	-0.0289	-0.0106	0.0966	-0.2927
	0.4307	0.0780	-39.0220	

95.9200 -39.0600 -0.2249 -0.0639 -
0.0992 -0.0490 -0.0054 0.0980 -0.2889
0.4324 0.0788 -39.0206
95.9400 -39.2600 -0.2437 -0.1155 -
0.1514 -0.0658 0.0002 0.0992 -0.2852
0.4340 0.0795 -39.0193
95.9600 -38.6500 0.4228 -0.0175 -
0.1729 -0.0790 0.0065 0.1001 -0.2815
0.4357 0.0803 -39.0179
95.9800 -39.1100 -0.1852 0.0076 -
0.1500 -0.0887 0.0133 0.1007 -0.2779
0.4373 0.0810 -39.0166
96.0000 -38.8300 -0.0097 -0.0498 -
0.0814 -0.0951 0.0207 0.1009 -0.2743
0.4388 0.0818 -39.0152
96.0200 -38.9200 -0.1159 -0.0628
0.0176 -0.0986 0.0286 0.1009 -0.2708
0.4404 0.0825 -39.0139
96.0400 -38.3000 0.2359 -0.0021
0.1176 -0.1000 0.0372 0.1006 -0.2674
0.4419 0.0833 -39.0126
96.0600 -38.6800 -0.2271 0.0786
0.1882 -0.0999 0.0462 0.1000 -0.2640
0.4435 0.0841 -39.0112
96.0800 -38.2900 0.1008 0.1464
0.2052 -0.0986 0.0555 0.0991 -0.2608
0.4450 0.0848 -39.0099
96.1000 -38.3400 -0.0913 0.1982
0.1618 -0.0963 0.0651 0.0979 -0.2576
0.4464 0.0856 -39.0085
96.1200 -38.2700 0.2147 0.1322
0.0732 -0.0924 0.0747 0.0964 -0.2544
0.4479 0.0863 -39.0072
96.1400 -38.8900 -0.1188 -0.0868 -
0.0285 -0.0860 0.0842 0.0946 -0.2513
0.4493 0.0871 -39.0059
96.1600 -39.0100 -0.0468 -0.2891 -
0.1086 -0.0760 0.0932 0.0925 -0.2483
0.4507 0.0878 -39.0045
96.1800 -39.0400 0.0461 -0.2954 -
0.1444 -0.0617 0.1017 0.0901 -0.2454
0.4521 0.0886 -39.0032
96.2000 -39.0600 -0.2410 -0.0697 -
0.1333 -0.0427 0.1092 0.0874 -0.2426
0.4535 0.0893 -39.0019
96.2200 -38.3600 0.1279 0.2160 -
0.0909 -0.0195 0.1157 0.0845 -0.2398
0.4548 0.0901 -39.0005
96.2400 -37.9300 0.3128 0.3324 -
0.0414 0.0075 0.1210 0.0813 -0.2371
0.4561 0.0908 -38.9992

96.2600 -38.6700 -0.3212 0.1725 -
0.0057 0.0376 0.1248 0.0778 -0.2344
0.4574 0.0916 -38.9978
96.2800 -38.2700 0.3187 -0.1187
0.0070 0.0699 0.1270 0.0741 -0.2318
0.4587 0.0923 -38.9965
96.3000 -38.7900 -0.1072 -0.2967 -
0.0016 0.1027 0.1274 0.0702 -0.2293
0.4599 0.0930 -38.9952
96.3200 -38.6100 -0.0989 -0.1983 -
0.0215 0.1340 0.1259 0.0660 -0.2269
0.4611 0.0938 -38.9938
96.3400 -38.4000 0.0533 0.0530 -
0.0395 0.1614 0.1224 0.0617 -0.2245
0.4623 0.0945 -38.9925
96.3600 -37.7800 0.3744 0.1623 -
0.0416 0.1826 0.1169 0.0571 -0.2222
0.4635 0.0953 -38.9912
96.3800 -38.1100 0.0825 0.0710 -
0.0209 0.1952 0.1093 0.0524 -0.2200
0.4647 0.0960 -38.9899
96.4000 -38.7700 -0.5725 -0.0363
0.0251 0.1976 0.0998 0.0476 -0.2178
0.4658 0.0967 -38.9885
96.4200 -37.7100 0.5091 -0.0745
0.0923 0.1887 0.0883 0.0426 -0.2157
0.4669 0.0975 -38.9872
96.4400 -38.2800 -0.0724 -0.1042
0.1682 0.1687 0.0751 0.0375 -0.2137
0.4680 0.0982 -38.9859
96.4600 -38.3100 -0.2141 -0.0631
0.2349 0.1381 0.0604 0.0324 -0.2117
0.4691 0.0989 -38.9845
96.4800 -38.1400 -0.0834 0.0865
0.2738 0.0982 0.0444 0.0272 -0.2097
0.4701 0.0997 -38.9832
96.5000 -37.9500 0.1626 0.2109
0.2712 0.0511 0.0274 0.0220 -0.2079
0.4711 0.1004 -38.9819
96.5200 -38.2000 -0.0304 0.1591
0.2210 -0.0001 0.0097 0.0168 -0.2060
0.4721 0.1011 -38.9806
96.5400 -38.7000 -0.1518 -0.0245
0.1261 -0.0519 -0.0082 0.0117 -0.2043
0.4731 0.1018 -38.9792
96.5600 -38.7000 0.1612 -0.1637
0.0007 -0.1007 -0.0262 0.0065 -0.2025
0.4741 0.1025 -38.9779
96.5800 -39.1200 -0.0671 -0.1731 -
0.1324 -0.1430 -0.0438 0.0015 -0.2009
0.4750 0.1033 -38.9766

96.6000 -39.0800 -0.0192 -0.0399 -
0.2505 -0.1757 -0.0607 -0.0034 -0.1993
0.4759 0.1040 -38.9753
96.6200 -39.2500 -0.2067 0.1417 -
0.3356 -0.1956 -0.0766 -0.0083 -0.1977
0.4768 0.1047 -38.9739
96.6400 -38.7700 0.3972 0.1613 -
0.3769 -0.2005 -0.0912 -0.0130 -0.1962
0.4776 0.1054 -38.9726
96.6600 -39.5600 -0.3884 -0.0125 -
0.3688 -0.1894 -0.1043 -0.0175 -0.1947
0.4785 0.1061 -38.9713
96.6800 -39.2400 0.1392 -0.1968 -
0.3065 -0.1634 -0.1157 -0.0218 -0.1933
0.4793 0.1068 -38.9700
96.7000 -39.2200 0.2240 -0.3180 -
0.1901 -0.1255 -0.1254 -0.0259 -0.1919
0.4801 0.1075 -38.9687
96.7200 -39.2800 -0.1696 -0.3115 -
0.0327 -0.0798 -0.1334 -0.0299 -0.1905
0.4808 0.1082 -38.9673
96.7400 -38.8500 -0.2644 -0.0755
0.1377 -0.0307 -0.1395 -0.0336 -0.1892
0.4816 0.1089 -38.9660
96.7600 -38.2600 -0.0183 0.2916
0.2834 0.0170 -0.1439 -0.0370 -0.1879
0.4823 0.1096 -38.9647
96.7800 -37.3900 0.5614 0.4363
0.3646 0.0591 -0.1464 -0.0403 -0.1866
0.4830 0.1103 -38.9634
96.8000 -38.4000 -0.3283 0.1508
0.3586 0.0917 -0.1470 -0.0433 -0.1854
0.4837 0.1110 -38.9621
96.8200 -38.4200 0.0598 -0.1933
0.2755 0.1125 -0.1458 -0.0460 -0.1842
0.4843 0.1117 -38.9607
96.8400 -39.0900 -0.3973 -0.1925
0.1448 0.1209 -0.1427 -0.0485 -0.1831
0.4849 0.1124 -38.9594
96.8600 -38.5700 0.1215 -0.0029
0.0067 0.1177 -0.1377 -0.0507 -0.1819
0.4855 0.1130 -38.9581
96.8800 -38.2500 0.4622 0.0371 -
0.1005 0.1044 -0.1309 -0.0527 -0.1808
0.4861 0.1137 -38.9568
96.9000 -39.2700 -0.3347 -0.1049 -
0.1573 0.0829 -0.1223 -0.0545 -0.1797
0.4867 0.1144 -38.9555
96.9200 -39.0700 -0.2466 -0.1328 -
0.1676 0.0558 -0.1121 -0.0559 -0.1786
0.4872 0.1151 -38.9542

96.9400 -38.6700 0.1395 0.0684 -
0.1471 0.0259 -0.1005 -0.0572 -0.1775
0.4877 0.1157 -38.9529
96.9600 -38.6100 -0.0482 0.2803 -
0.1133 -0.0038 -0.0876 -0.0582 -0.1764
0.4882 0.1164 -38.9515
96.9800 -38.0000 0.5480 0.1982 -
0.0814 -0.0307 -0.0736 -0.0590 -0.1754
0.4887 0.1170 -38.9502
97.0000 -39.4100 -0.5720 -0.1210 -
0.0557 -0.0530 -0.0586 -0.0597 -0.1743
0.4891 0.1177 -38.9489
97.0200 -39.0600 0.0197 -0.2614 -
0.0359 -0.0691 -0.0429 -0.0601 -0.1733
0.4895 0.1183 -38.9476
97.0400 -38.6000 0.1108 -0.0805 -
0.0199 -0.0781 -0.0265 -0.0604 -0.1722
0.4899 0.1190 -38.9463
97.0600 -38.6600 -0.1423 0.1777 -
0.0061 -0.0801 -0.0097 -0.0605 -0.1711
0.4903 0.1196 -38.9450
97.0800 -38.0500 0.5061 0.1728
0.0051 -0.0758 0.0075 -0.0605 -0.1701
0.4906 0.1203 -38.9437
97.1000 -39.0600 -0.3523 -0.0504
0.0153 -0.0664 0.0250 -0.0604 -0.1690
0.4910 0.1209 -38.9424
97.1200 -39.1600 -0.4251 -0.1417
0.0230 -0.0533 0.0426 -0.0602 -0.1679
0.4913 0.1215 -38.9411
97.1400 -38.0200 0.5117 -0.0121
0.0261 -0.0384 0.0602 -0.0599 -0.1668
0.4916 0.1222 -38.9397
97.1600 -38.5600 -0.1509 0.0917
0.0241 -0.0232 0.0776 -0.0596 -0.1656
0.4918 0.1228 -38.9384
97.1800 -38.4000 0.0028 0.0617
0.0186 -0.0088 0.0946 -0.0592 -0.1645
0.4921 0.1234 -38.9371
97.2000 -38.5100 0.0372 -0.0279
0.0117 0.0045 0.1109 -0.0588 -0.1633
0.4923 0.1240 -38.9358
97.2200 -38.6900 -0.1713 -0.0883
0.0042 0.0168 0.1262 -0.0584 -0.1621
0.4925 0.1246 -38.9345
97.2400 -38.2800 0.1869 -0.0656 -
0.0061 0.0285 0.1402 -0.0580 -0.1609
0.4926 0.1252 -38.9332
97.2600 -38.3100 0.0456 0.0421 -
0.0224 0.0402 0.1527 -0.0577 -0.1596
0.4928 0.1258 -38.9319

97.2800 -38.5000 -0.4420 0.1675 -
0.0462 0.0523 0.1633 -0.0573 -0.1584
0.4929 0.1264 -38.9306
97.3000 -37.5800 0.6240 0.1645 -
0.0768 0.0648 0.1719 -0.0570 -0.1571
0.4930 0.1270 -38.9293
97.3200 -38.7100 -0.2720 -0.0279 -
0.1034 0.0774 0.1784 -0.0568 -0.1557
0.4931 0.1276 -38.9280
97.3400 -38.8900 -0.5116 -0.1967 -
0.1110 0.0895 0.1825 -0.0566 -0.1543
0.4931 0.1281 -38.9267
97.3600 -38.2000 0.3868 -0.1818 -
0.0878 0.1003 0.1841 -0.0564 -0.1529
0.4932 0.1287 -38.9254
97.3800 -38.1900 0.1860 -0.0985 -
0.0257 0.1088 0.1834 -0.0564 -0.1514
0.4932 0.1293 -38.9241
97.4000 -38.7500 -0.5459 -0.0516
0.0712 0.1134 0.1801 -0.0564 -0.1499
0.4932 0.1299 -38.9228
97.4200 -37.6400 0.4257 -0.0318
0.1823 0.1129 0.1744 -0.0565 -0.1484
0.4932 0.1304 -38.9215
97.4400 -37.7500 0.2072 -0.0025
0.2748 0.1063 0.1664 -0.0567 -0.1468
0.4931 0.1310 -38.9202
97.4600 -38.2100 -0.3126 0.0817
0.3157 0.0931 0.1562 -0.0570 -0.1451
0.4931 0.1315 -38.9189
97.4800 -37.9600 -0.2014 0.2790
0.2846 0.0734 0.1440 -0.0573 -0.1434
0.4930 0.1320 -38.9176
97.5000 -37.3000 0.4661 0.4130
0.1815 0.0487 0.1300 -0.0577 -0.1417
0.4929 0.1326 -38.9163
97.5200 -37.6400 0.2472 0.2176
0.0268 0.0216 0.1148 -0.0582 -0.1399
0.4928 0.1331 -38.9150
97.5400 -38.9000 -0.2688 -0.1788 -
0.1347 -0.0051 0.0985 -0.0587 -0.1380
0.4926 0.1336 -38.9137
97.5600 -39.3200 -0.3156 -0.4423 -
0.2540 -0.0290 0.0816 -0.0593 -0.1361
0.4925 0.1341 -38.9124
97.5800 -39.1700 -0.0257 -0.4273 -
0.2949 -0.0478 0.0645 -0.0600 -0.1342
0.4923 0.1347 -38.9111
97.6000 -38.8100 0.1475 -0.2135 -
0.2495 -0.0606 0.0476 -0.0607 -0.1322
0.4921 0.1352 -38.9098

97.6200 -38.6200 -0.0857 0.0524 -
0.1412 -0.0678 0.0311 -0.0614 -0.1302
0.4919 0.1357 -38.9085
97.6400 -38.5200 -0.0880 0.2432 -
0.0110 -0.0700 0.0154 -0.0622 -0.1281
0.4916 0.1362 -38.9072
97.6600 -37.8000 0.4096 0.2736
0.0993 -0.0678 0.0007 -0.0630 -0.1260
0.4914 0.1366 -38.9059
97.6800 -38.5400 -0.2553 0.1514
0.1616 -0.0622 -0.0126 -0.0638 -0.1238
0.4911 0.1371 -38.9046
97.7000 -38.6200 -0.2023 0.0274
0.1642 -0.0547 -0.0246 -0.0646 -0.1215
0.4908 0.1376 -38.9033
97.7200 -38.0600 0.3852 -0.0401
0.1151 -0.0465 -0.0352 -0.0654 -0.1192
0.4905 0.1381 -38.9020
97.7400 -38.8200 -0.1783 -0.1024
0.0393 -0.0385 -0.0443 -0.0661 -0.1169
0.4902 0.1385 -38.9007
97.7600 -38.5600 0.1620 -0.1121 -
0.0338 -0.0312 -0.0520 -0.0668 -0.1145
0.4898 0.1390 -38.8994
97.7800 -38.9500 -0.3330 -0.0179 -
0.0812 -0.0250 -0.0585 -0.0675 -0.1121
0.4895 0.1394 -38.8981
97.8000 -38.3000 0.2317 0.0747 -
0.0948 -0.0197 -0.0637 -0.0681 -0.1096
0.4891 0.1399 -38.8968
97.8200 -38.5900 -0.0694 0.0360 -
0.0806 -0.0156 -0.0679 -0.0686 -0.1071
0.4887 0.1403 -38.8956
97.8400 -38.3300 0.1225 -0.0868 -
0.0512 -0.0121 -0.0712 -0.0691 -0.1045
0.4883 0.1407 -38.8943
97.8600 -38.8200 -0.1795 -0.1571 -
0.0192 -0.0082 -0.0738 -0.0694 -0.1019
0.4879 0.1412 -38.8930
97.8800 -38.5500 0.1043 -0.0785
0.0052 -0.0030 -0.0759 -0.0696 -0.0993
0.4874 0.1416 -38.8917
97.9000 -38.2600 0.1668 0.0894
0.0148 0.0040 -0.0777 -0.0696 -0.0966
0.4870 0.1420 -38.8904
97.9200 -38.5800 -0.3215 0.2123
0.0104 0.0130 -0.0795 -0.0694 -0.0939
0.4865 0.1424 -38.8891
97.9400 -38.3800 -0.0058 0.1401 -
0.0045 0.0242 -0.0813 -0.0691 -0.0911
0.4860 0.1428 -38.8878

97.9600 -38.1900 0.4154 -0.1110 -
0.0200 0.0371 -0.0835 -0.0685 -0.0883
0.4855 0.1432 -38.8865
97.9800 -39.4600 -0.6414 -0.2738 -
0.0237 0.0509 -0.0859 -0.0677 -0.0855
0.4850 0.1436 -38.8852
98.0000 -38.2300 0.2046 -0.1090 -
0.0097 0.0642 -0.0887 -0.0666 -0.0826
0.4844 0.1439 -38.8839
98.0200 -38.0200 0.2733 0.1449
0.0154 0.0756 -0.0920 -0.0653 -0.0797
0.4839 0.1443 -38.8826
98.0400 -38.2500 -0.1679 0.2094
0.0381 0.0836 -0.0956 -0.0637 -0.0767
0.4833 0.1447 -38.8814
98.0600 -38.3100 0.0392 0.0932
0.0459 0.0865 -0.0995 -0.0617 -0.0738
0.4827 0.1450 -38.8801
98.0800 -38.4200 0.0224 -0.0351
0.0351 0.0838 -0.1036 -0.0595 -0.0708
0.4822 0.1454 -38.8788
98.1000 -38.6800 -0.2718 -0.0605
0.0117 0.0756 -0.1077 -0.0569 -0.0677
0.4815 0.1457 -38.8775
98.1200 -37.9200 0.4642 -0.0080 -
0.0145 0.0623 -0.1117 -0.0540 -0.0647
0.4809 0.1461 -38.8762
98.1400 -38.9400 -0.4698 -0.0385 -
0.0282 0.0445 -0.1154 -0.0508 -0.0616
0.4803 0.1464 -38.8749
98.1600 -38.2200 0.5467 -0.1423 -
0.0183 0.0227 -0.1185 -0.0473 -0.0585
0.4797 0.1467 -38.8736
98.1800 -38.8300 -0.3965 -0.0929
0.0090 -0.0019 -0.1208 -0.0435 -0.0554
0.4790 0.1470 -38.8723
98.2000 -38.6700 -0.4102 0.1141
0.0374 -0.0275 -0.1221 -0.0393 -0.0523
0.4783 0.1473 -38.8710
98.2200 -37.4700 0.7214 0.2517
0.0524 -0.0524 -0.1223 -0.0350 -0.0491
0.4776 0.1476 -38.8698
98.2400 -38.6800 -0.3395 0.1096
0.0444 -0.0746 -0.1211 -0.0303 -0.0460
0.4769 0.1479 -38.8685
98.2600 -38.8100 -0.1241 -0.1591
0.0182 -0.0927 -0.1186 -0.0254 -0.0428
0.4762 0.1482 -38.8672
98.2800 -38.8800 -0.2262 -0.2370 -
0.0134 -0.1055 -0.1145 -0.0202 -0.0397
0.4755 0.1485 -38.8659

98.3000 -38.5200 0.1607 -0.0429 -
0.0403 -0.1120 -0.1089 -0.0148 -0.0365
0.4748 0.1488 -38.8646
98.3200 -38.2400 0.0788 0.1959 -
0.0620 -0.1115 -0.1018 -0.0092 -0.0333
0.4741 0.1490 -38.8633
98.3400 -38.4500 0.0095 0.2133 -
0.0795 -0.1038 -0.0932 -0.0034 -0.0302
0.4733 0.1493 -38.8620
98.3600 -38.1600 0.2478 0.0354 -
0.0917 -0.0898 -0.0831 0.0026 -0.0270
0.4725 0.1495 -38.8608
98.3800 -38.8600 -0.3183 -0.1396 -
0.0916 -0.0712 -0.0716 0.0088 -0.0239
0.4718 0.1498 -38.8595
98.4000 -38.6100 -0.1265 -0.1463 -
0.0731 -0.0500 -0.0588 0.0152 -0.0207
0.4710 0.1500 -38.8582
98.4200 -38.2000 0.1880 -0.0634 -
0.0336 -0.0280 -0.0447 0.0217 -0.0176
0.4702 0.1503 -38.8569
98.4400 -37.9000 0.2668 -0.0494
0.0252 -0.0073 -0.0296 0.0283 -0.0145
0.4694 0.1505 -38.8556
98.4600 -38.7500 -0.5389 -0.0143
0.0906 0.0104 -0.0135 0.0350 -0.0114
0.4686 0.1507 -38.8543
98.4800 -38.0100 -0.0394 0.1324
0.1429 0.0233 0.0034 0.0418 -0.0084
0.4678 0.1509 -38.8531
98.5000 -37.1700 0.5173 0.1958
0.1692 0.0305 0.0210 0.0486 -0.0053
0.4669 0.1511 -38.8518
98.5200 -37.9300 0.0193 0.0064
0.1632 0.0320 0.0391 0.0554 -0.0023
0.4661 0.1513 -38.8505
98.5400 -38.5400 -0.3825 -0.2116
0.1244 0.0286 0.0576 0.0621 0.0006
0.4652 0.1515 -38.8492
98.5600 -38.0500 0.0137 -0.1809
0.0605 0.0217 0.0763 0.0687 0.0036
0.4644 0.1517 -38.8479
98.5800 -38.1800 -0.1340 0.0422 -
0.0166 0.0137 0.0948 0.0752 0.0065
0.4635 0.1519 -38.8466
98.6000 -37.5400 0.4253 0.1594 -
0.0943 0.0067 0.1131 0.0815 0.0093
0.4626 0.1521 -38.8454
98.6200 -38.4200 -0.4252 0.0807 -
0.1571 0.0031 0.1307 0.0876 0.0121
0.4617 0.1523 -38.8441

98.6400	-38.0000	0.2000	-0.0405	-
0.1944	0.0047	0.1476	0.0935	0.0149
	0.4608	0.1524	-38.8428	
98.6600	-38.4100	-0.2479	-0.0718	-
0.2036	0.0125	0.1633	0.0990	0.0176
	0.4599	0.1526	-38.8415	
98.6800	-38.2100	-0.0966	-0.0016	-
0.1810	0.0265	0.1778	0.1043	0.0202
	0.4590	0.1528	-38.8402	
98.7000	-37.5200	0.3687	0.0486	-
0.1250	0.0459	0.1907	0.1093	0.0228
	0.4581	0.1529	-38.8389	
98.7200	-38.1300	-0.2419	-0.0327	-
0.0392	0.0685	0.2020	0.1138	0.0254
	0.4572	0.1531	-38.8377	
98.7400	-37.6100	0.2614	-0.1275	
0.0679	0.0912	0.2113	0.1180	0.0278
	0.4562	0.1532	-38.8364	
98.7600	-38.2000	-0.4730	-0.0676	
0.1775	0.1108	0.2186	0.1218	0.0302
	0.4553	0.1534	-38.8351	
98.7800	-37.0000	0.4122	0.1083	
0.2634	0.1241	0.2237	0.1251	0.0326
	0.4543	0.1535	-38.8338	
98.8000	-37.4000	-0.2047	0.2102	
0.2965	0.1290	0.2265	0.1279	0.0348
	0.4534	0.1537	-38.8325	
98.8200	-37.0000	0.3437	0.1377	
0.2564	0.1240	0.2271	0.1304	0.0370
	0.4524	0.1538	-38.8313	
98.8400	-37.8100	-0.2559	-0.0249	
0.1493	0.1094	0.2255	0.1323	0.0392
	0.4514	0.1539	-38.8300	
98.8600	-37.7400	0.1217	-0.1342	
0.0126	0.0867	0.2218	0.1338	0.0412
	0.4504	0.1541	-38.8287	
98.8800	-38.1200	-0.0707	-0.1643	-
0.1067	0.0577	0.2163	0.1349	0.0432
	0.4495	0.1542	-38.8274	
98.9000	-38.2100	0.0385	-0.1438	-
0.1736	0.0248	0.2093	0.1355	0.0451
	0.4485	0.1543	-38.8261	
98.9200	-38.2700	-0.1088	-0.0625	-
0.1862	-0.0092	0.2010	0.1358	0.0469
	0.4475	0.1544	-38.8248	
98.9400	-37.9900	-0.0214	0.1065	-
0.1693	-0.0403	0.1917	0.1357	0.0487
	0.4465	0.1545	-38.8236	
98.9600	-37.7300	0.1258	0.2331	-
0.1525	-0.0647	0.1818	0.1354	0.0504
	0.4455	0.1546	-38.8223	

98.9800	-37.7100	0.1581	0.1498	-0.1536	-0.0808	0.1716	0.1349	0.0521	0.4445	0.1548	-38.8210
99.0000	-38.6200	-0.3524	-0.1166	-0.1721	-0.0911	0.1613	0.1342	0.0537	0.4434	0.1549	-38.8197

DATA:
Time-varying Shannon entropy (SE) and 95% significance level of DO modes extracted by EEMD
(shown in the 5-8 columns in the above).

Column 1: Time (kyr. BP)
Column 2: Shannon entropy of IMF3 (SE3)
Column 3: 95% significance level of SE of IMF3 (95% SL3)
Column 4: Shannon entropy of IMF4 (SE4)
Column 5: 95% significance level of SE of IMF4 (95% SL4)
Column 6: Shannon entropy of IMF5 (SE5)
Column 7: 95% significance level of SE of IMF5 (95% SL5)
Column 8: Shannon entropy of IMF6 (SE6)
Column 9: 95% significance level of SE of IMF6 (95% SL6)

Time	SE3	95% SL3	SE4	95% SL4	SE5	95% SL5	SE6	95% SL6
4.9900	1.1822	1.3164	1.3305	1.4630	1.1702	1.6624	1.6524	1.8559
5.5900	0.9198	1.3019	1.0051	1.4676	1.2668	1.6660	1.2641	1.8509
6.1900	1.0077	1.3085	1.0189	1.4685	1.4370	1.6623	1.3098	1.8557
6.7900	1.0265	1.3026	1.0635	1.4697	1.5421	1.6664	1.3943	1.8536
7.3900	1.0345	1.2941	1.2086	1.4537	1.5593	1.6630	1.4546	1.8441
7.9900	1.0402	1.2871	1.2334	1.4492	1.6209	1.6610	1.4744	1.8461
8.5900	0.4590	1.2893	0.8694	1.4461	1.1332	1.6664	1.5633	1.8473
9.1900	0.7520	1.2928	1.0964	1.4658	1.2194	1.6655	1.2015	1.8458

9.7900 0.9596 1.2781 1.2267
1.4650 1.2156 1.6582 1.3163
1.8362
10.3900 1.1595 1.2703 1.4411
1.4591 1.4897 1.6604 1.5166
1.8377
10.9900 1.1956 1.2626 1.0417
1.4683 1.3256 1.6664 1.6054
1.8398
11.5900 1.3564 1.2727 1.0763
1.4609 1.1702 1.6587 1.6764
1.8503
12.1900 1.4780 1.2795 1.2230
1.4649 1.2954 1.6617 1.7398
1.8542
12.7900 1.5045 1.2975 1.2376
1.4687 1.2974 1.6695 1.7722
1.8460
13.3900 1.5136 1.2920 1.2509
1.4726 1.2779 1.6722 1.8402
1.8637
13.9900 1.5414 1.3009 1.2472
1.4560 1.2636 1.6676 1.8772
1.8641
14.5900 1.4404 1.3163 1.2445
1.4554 1.1081 1.6604 1.8617
1.8456
15.1900 1.1192 1.3123 1.2344
1.4667 1.0939 1.6655 1.8669
1.8373
15.7900 1.1115 1.3185 1.2335
1.4613 1.1371 1.6594 1.8404
1.8455
16.3900 1.0880 1.3063 1.1993
1.4620 1.1435 1.6606 1.7658
1.8380
16.9900 1.0809 1.3275 1.0995
1.4716 1.1248 1.6488 1.5148
1.8364
17.5900 1.2167 1.3114 1.0832
1.4577 1.1245 1.6633 1.5253
1.8504
18.1900 1.2961 1.3169 1.2329
1.4502 1.6439 1.6597 1.5227
1.8485
18.7900 1.1786 1.3262 1.2153
1.4514 1.6439 1.6682 1.3804
1.8578
19.3900 1.1722 1.3211 1.1692
1.4529 1.6524 1.6607 1.5102
1.8488

19.9900 1.5142 1.3089 1.1930
1.4509 1.6869 1.6832 1.4388
1.8475
20.5900 0.9332 1.3154 1.3683
1.4569 1.7147 1.6816 1.4499
1.8410
21.1900 0.9368 1.2910 1.3576
1.4624 1.6975 1.6803 1.7490
1.8406
21.7900 0.9307 1.2997 1.3588
1.4665 1.5378 1.6675 1.7514
1.8486
22.3900 0.9556 1.2831 1.3565
1.4666 1.5042 1.6641 1.7377
1.8407
22.9900 0.9584 1.2974 1.3606
1.4433 1.4597 1.6721 1.6259
1.8555
23.5900 1.0747 1.2921 1.3730
1.4530 1.5042 1.6964 1.6155
1.8630
24.1900 1.0877 1.3008 1.2655
1.4583 1.5924 1.6820 1.6122
1.8607
24.7900 0.9257 1.2973 0.9663
1.4577 1.1566 1.6956 1.5949
1.8624
25.3900 0.9437 1.3097 1.0099
1.4527 1.3305 1.6725 1.5854
1.8301
25.9900 0.9762 1.2930 1.4271
1.4532 1.3051 1.6760 1.4873
1.8392
26.5900 0.9487 1.2958 1.4342
1.4468 1.3595 1.6721 1.4191
1.8468
27.1900 0.9372 1.3022 1.4349
1.4498 1.3482 1.6776 1.4591
1.8392
27.7900 0.9389 1.3014 1.4418
1.4464 1.3667 1.6671 1.4068
1.8474
28.3900 0.9163 1.3036 1.4435
1.4382 1.3642 1.6655 1.4525
1.8436
28.9900 0.9018 1.2919 1.4577
1.4445 1.4501 1.6568 1.4912
1.8600
29.5900 0.9008 1.3013 1.6010
1.4514 1.5320 1.6439 1.5160
1.8602

30.1900 0.9042 1.3141 1.5961
1.4622 1.5804 1.6561 1.6770
1.8567
30.7900 1.1085 1.3144 1.6023
1.4581 1.5397 1.6549 1.7414
1.8547
31.3900 1.2381 1.3220 1.6118
1.4589 1.6810 1.6639 1.7600
1.8479
31.9900 1.0960 1.3032 1.5692
1.4694 1.6708 1.6449 1.6778
1.8549
32.5900 1.0634 1.2924 1.5698
1.4598 1.7023 1.6480 1.8024
1.8587
33.1900 1.1011 1.2830 1.5803
1.4684 1.8812 1.6636 1.5094
1.8634
33.7900 1.1030 1.3090 1.5779
1.4725 1.8648 1.6780 1.6369
1.8545
34.3900 1.1010 1.3145 1.5739
1.4649 1.6953 1.6768 1.6295
1.8579
34.9900 1.1005 1.3213 1.4576
1.4774 1.8875 1.6707 1.7374
1.8529
35.5900 1.1472 1.3169 1.3245
1.4710 1.7106 1.6731 1.7844
1.8428
36.1900 1.1457 1.3112 1.0714
1.4570 1.6629 1.6691 1.7772
1.8437
36.7900 0.8922 1.3126 1.1277
1.4538 1.6383 1.6597 1.7720
1.8425
37.3900 1.1514 1.2993 1.4250
1.4811 1.6185 1.6660 1.7876
1.8496
37.9900 1.1270 1.2955 1.2892
1.4607 1.5188 1.6713 1.7705
1.8612
38.5900 1.2543 1.3141 1.4050
1.4699 1.5147 1.6637 1.7191
1.8551
39.1900 1.2153 1.3035 1.4074
1.4675 1.3772 1.6668 1.7020
1.8453
39.7900 1.2084 1.3070 1.4024
1.4572 1.5951 1.6665 1.6498
1.8386

40.3900 1.2237 1.3111 1.4341
1.4742 1.6560 1.6692 1.5180
1.8305
40.9900 1.2382 1.3060 1.6042
1.4686 1.6514 1.6802 1.4716
1.8470
41.5900 1.2925 1.2834 1.6049
1.4610 1.6416 1.6630 1.6415
1.8355
42.1900 1.2898 1.2829 1.5961
1.4524 1.6365 1.6645 1.6677
1.8579
42.7900 1.2958 1.2866 1.5910
1.4505 1.6286 1.6614 1.6688
1.8632
43.3900 1.3127 1.2814 1.5735
1.4559 1.6315 1.6634 1.5773
1.8499
43.9900 1.4389 1.2797 1.4164
1.4445 1.6461 1.6547 1.6844
1.8532
44.5900 1.4328 1.2918 1.3170
1.4422 1.6300 1.6556 1.7204
1.8617
45.1900 1.4591 1.3076 1.3073
1.4333 1.4823 1.6707 1.8005
1.8650
45.7900 1.4452 1.3048 1.3208
1.4459 1.4145 1.6677 1.8236
1.8558
46.3900 1.1750 1.2961 1.3014
1.4520 1.8426 1.6655 1.8126
1.8507
46.9900 1.1736 1.3023 1.1560
1.4414 1.5873 1.6632 1.8284
1.8439
47.5900 1.1318 1.2828 1.0229
1.4572 1.5551 1.6489 1.7852
1.8419
48.1900 1.0980 1.2870 0.9852
1.4684 1.5289 1.6588 1.7013
1.8460
48.7900 1.1356 1.2951 1.0002
1.4589 1.5436 1.6670 1.6548
1.8571
49.3900 1.1587 1.3145 0.9789
1.4607 1.5230 1.6691 1.4928
1.8542
49.9900 1.1111 1.3150 0.7974
1.4472 1.5386 1.6532 1.4553
1.8468

50.5900 1.0150 1.3140 1.3301
1.4385 1.3628 1.6592 1.3824
1.8375
51.1900 1.0555 1.3102 1.2241
1.4446 1.3793 1.6480 1.4019
1.8391
51.7900 1.1022 1.2979 1.1004
1.4514 1.3647 1.6525 1.4991
1.8381
52.3900 0.9961 1.3089 0.8265
1.4514 1.3120 1.6592 1.6312
1.8358
52.9900 1.0427 1.3188 0.9858
1.4605 1.3427 1.6528 1.6176
1.8455
53.5900 1.0474 1.3140 1.0565
1.4608 1.4070 1.6579 1.6439
1.8424
54.1900 1.0487 1.3086 1.1249
1.4652 1.4297 1.6630 1.6568
1.8375
54.7900 1.0126 1.3073 1.1074
1.4677 1.4506 1.6675 1.6526
1.8481
55.3900 1.0560 1.3101 1.1475
1.4557 1.6256 1.6798 1.6700
1.8418
55.9900 0.8679 1.3004 1.2338
1.4568 1.7618 1.6789 1.6787
1.8343
56.5900 1.1176 1.2982 1.2798
1.4786 1.7464 1.6833 1.6245
1.8500
57.1900 1.0613 1.3210 1.2693
1.4720 1.7470 1.6728 1.5794
1.8382
57.7900 1.0426 1.3137 1.2278
1.4557 1.4866 1.6705 1.3286
1.8277
58.3900 1.0004 1.3077 1.0908
1.4461 1.5005 1.6633 1.8342
1.8394
58.9900 0.9991 1.3096 1.2277
1.4627 1.4630 1.6718 1.8185
1.8430
59.5900 1.0121 1.3031 0.9817
1.4422 1.4054 1.6600 1.5389
1.8538
60.1900 1.0212 1.3068 0.9587
1.4568 1.3598 1.6745 1.6123
1.8547

60.7900 1.0516 1.2938 1.3332
1.4619 1.3594 1.6759 1.4573
1.8450
61.3900 1.0613 1.3105 1.0752
1.4478 1.3804 1.6810 1.5381
1.8370
61.9900 0.8579 1.3059 1.0359
1.4644 1.4533 1.6781 1.5609
1.8436
62.5900 1.2430 1.2933 0.9963
1.4554 1.4005 1.6781 1.5795
1.8439
63.1900 1.2404 1.2940 1.0228
1.4653 1.2926 1.6751 1.5745
1.8543
63.7900 1.2355 1.2730 1.0527
1.4646 1.2094 1.6785 1.5841
1.8611
64.3900 1.2575 1.2757 1.0774
1.4529 1.2149 1.6686 1.5830
1.8422
64.9900 1.1489 1.2678 1.0938
1.4532 1.2563 1.6762 1.5506
1.8451
65.5900 1.1582 1.2914 1.0927
1.4579 1.2843 1.6706 1.5411
1.8537
66.1900 1.1704 1.3062 1.1112
1.4626 1.3301 1.6655 1.5789
1.8419
66.7900 0.9236 1.2876 1.1611
1.4605 1.3702 1.6621 1.5488
1.8369
67.3900 0.8086 1.2746 0.8840
1.4442 1.4628 1.6708 1.3780
1.8341
67.9900 0.8060 1.2997 0.8600
1.4586 1.4330 1.6743 1.4198
1.8545
68.5900 0.7966 1.2808 0.8566
1.4609 1.1724 1.6771 1.4299
1.8471
69.1900 0.7918 1.2920 1.0583
1.4775 1.1111 1.6622 1.5192
1.8491
69.7900 1.1120 1.3018 1.2300
1.4709 1.3016 1.6734 1.4067
1.8440
70.3900 1.0971 1.2986 1.2494
1.4835 1.3749 1.6646 1.5224
1.8409

70.9900 1.0856 1.3051 1.2504
1.4984 1.4008 1.6610 1.7367
1.8410
71.5900 1.0984 1.2985 1.3300
1.4701 1.4245 1.6623 1.7666
1.8504
72.1900 1.0706 1.3100 1.3167
1.4657 1.4303 1.6551 1.8036
1.8520
72.7900 0.7372 1.3306 1.1051
1.4669 1.3967 1.6524 1.8428
1.8474
73.3900 0.7220 1.3317 1.1478
1.4689 1.4855 1.6535 1.8233
1.8465
73.9900 0.9543 1.3551 1.3804
1.4600 1.6446 1.6486 1.8137
1.8408
74.5900 0.9630 1.3318 1.4020
1.4596 1.6435 1.6502 1.8214
1.8448
75.1900 0.9795 1.3326 1.3958
1.4541 1.5963 1.6731 1.9098
1.8472
75.7900 0.6272 1.3015 1.3701
1.4556 1.4528 1.6700 1.8795
1.8401
76.3900 0.6367 1.2982 1.3743
1.4533 1.4520 1.6662 1.8472
1.8404
76.9900 0.6242 1.3074 1.3448
1.4469 1.4332 1.6661 1.7797
1.8421
77.5900 0.5828 1.3089 1.3029
1.4434 1.4140 1.6631 1.6252
1.8425
78.1900 0.5865 1.2932 1.2938
1.4616 1.3796 1.6505 1.4670
1.8468
78.7900 0.5870 1.3003 1.2893
1.4744 1.3005 1.6551 1.4643
1.8443
79.3900 0.5574 1.3102 1.1766
1.4468 1.3485 1.6580 1.4722
1.8441
79.9900 1.2620 1.3215 0.9848
1.4646 1.4079 1.6687 1.3637
1.8314
80.5900 1.2473 1.3199 0.9198
1.4459 1.4118 1.6740 1.2273
1.8366

81.1900	1.1320	1.3199	1.0349
1.4460	1.3293	1.6766	1.6526
1.8347			
81.7900	0.7592	1.3082	0.9437
1.4622	1.4092	1.6698	1.6341
1.8528			
82.3900	0.7112	1.3052	0.9995
1.4524	1.1435	1.6669	1.5783
1.8478			
82.9900	0.7133	1.2810	1.0244
1.4538	1.2244	1.6629	1.7252
1.8468			
83.5900	0.7276	1.2858	1.0404
1.4570	1.5883	1.6722	1.7353
1.8436			
84.1900	0.7355	1.2889	1.0496
1.4654	1.5870	1.6700	1.7583
1.8462			
84.7900	0.7737	1.3095	1.0532
1.4538	1.6226	1.6750	1.7612
1.8547			
85.3900	0.8053	1.3257	1.0521
1.4533	1.6423	1.6879	1.7762
1.8543			
85.9900	0.8284	1.3088	1.0582
1.4650	1.6128	1.6804	1.8432
1.8346			
86.5900	0.8280	1.2987	1.0381
1.4599	1.7247	1.6703	1.8270
1.8377			
87.1900	0.8611	1.3014	1.0314
1.4684	1.8151	1.6734	1.7291
1.8474			
87.7900	0.7928	1.3043	0.9297
1.4841	1.8085	1.6590	1.8836
1.8466			
88.3900	1.2682	1.2923	1.4839
1.4575	1.7491	1.6535	1.8796
1.8464			
88.9900	1.2658	1.3032	1.4979
1.4567	1.7505	1.6524	1.9352
1.8468			
89.5900	1.2506	1.2977	1.4823
1.4454	1.7269	1.6677	1.9103
1.8489			
90.1900	1.2431	1.3028	1.4542
1.4484	1.7310	1.6588	1.7522
1.8371			
90.7900	1.2152	1.2939	1.4289
1.4594	1.6209	1.6746	1.7914
1.8440			

91.3900 1.0731 1.2949 1.3831
1.4545 1.5445 1.6615 1.4644
1.8415
91.9900 1.0272 1.3012 1.3107
1.4593 1.3123 1.6535 1.3772
1.8329
92.5900 0.9993 1.3076 1.2991
1.4487 1.2462 1.6560 1.3307
1.8510
93.1900 1.4131 1.2826 1.0890
1.4470 1.1794 1.6613 1.4543
1.8499
93.7900 1.1685 1.2773 1.3844
1.4690 1.5167 1.6716 1.3445
1.8517
94.3900 1.1676 1.2828 1.3848
1.4625 1.6120 1.6719 1.8455
1.8462
94.9900 1.3550 1.2963 1.3001
1.4700 1.7634 1.6711 1.7753
1.8518
95.5900 1.3467 1.2941 1.2952
1.4594 1.7702 1.6634 1.7781
1.8410
