# Supplementary material for: Data concerning statistical relation between obliquity and Dansgaard–Oeschger events
Source: Data Brief. 2019 Mar 7;23:103727. doi: 10.1016/j.dib.2019.103727 (PMC6660458; doi:10.1016/j.dib.2019.103727)
Supplement: Multimedia component 5 [file mmc5.pdf]

Processed deuterium isotope (dD) data from EPICA DOME C ice core on EDC3 age scale
----

NAME OF DATA SET:
Processed deuterium isotope (dD) data from EPICA DOME C ice core on EDC3 age scale

LAST UPDATE: 21/12/2018

ORIGINAL REFERENCE:
Jia Deng, Zhaohua Wu, Min Zhanga, Norden. E Huang, Shizhu Wang,Fangli Qiao. 2018.
Data concerning statistical relation between obliquity and Dansgaard-Oeschger events.
Data in Brief, in press.

MAIN REFERENCES:
Datafile of the original data accompanies the following paper:

Jouzel et al., 2007. Orbital and Millennial Antarctic Climate Variability over the Past 800,000 Years.
Science. 317(5839), 793-796.

ABSTRACT:
Data presented are related to the research article entitled 。-Using Holo-Hilbert spectral analysis to
quantify the modulation of Dansgaard-Oeschger events by obliquity。7 [1]. The datasets in Deng et al (2018)
are analyzed on the foundation of ensemble empirical mode decomposition (EEMD) [2], and reveal more occurrences
of Dansgaard-Oeschger (DO) events in the decreasing phase of obliquity. Here, we report the number of significant
high Shannon entropy (SE) [3] of 95% significance level of DO events in the increasing and decreasing phases of
obliquity, respectively. First, the proxy time series are filtered by EEMD to obtain DO events. Then, the
time-varying SE of DO modes are calculated on the basis of principle of histogram. The 95% significance level
is evaluated through surrogate data [4]. Finally, a comparison between the numbers of SE values that are larger
than 95% significance level in the increasing and decreasing phases of obliquity, respectively, is reported.

GEOGRAPHIC REGION: Antarctica
PERIOD OF RECORD: 99 kyr. BP - 2 kyr.BP

FUNDING SOURCES:
This work was jointly supported by the National Basic Research Program of China (Grant 2012CB957802);
the National Natural Science Foundation of China (NSFC) (Grant 41506067); the Basic Scientific Fund

for National Public Research Institutes of China (Grant 2015G04); the US National Science Foundation
(Grant AGS-1723300); the NSFC-Shandong Joint Fund for Marine Science Research Centers (Grant U1406404);
and the National Programme on Global Change and Air-Sea Interaction (Grant GASI-IPOVAI-05).

DATA:
Interpolated EPICA DOME C deuterium isotope (dD) data on EDC3 age scale (Jouzel et al, 2007).
Processed data by ensemble empirical mode decomposition (EEMD).

Column 1: Time (kyr. BP)
Column 2: (Interpolated) dD data (years before year 1950)
Column 3: 1st EEMD component of dD records
Column 4: 2nd EEMD component of dD records
Column 5: 3rd EEMD component of dD records
Column 6: 4th EEMD component of dD records
Column 7: 5th EEMD component of dD records
Column 8: 6th EEMD component of dD records
Column 9: 8th EEMD component of dD records
Column 10: 9th EEMD component of dD records
Column 11: 10th EEMD component of dD records
Column 12: EEMD trend of dD records

Time	dD data	IMF1	IMF2	IMF3	IMF4	IMF5	IMF6	IMF7	IMF8	IMF9	trend
2.0000	-401.8294	-2.9633	-1.8033	-0.3089	1.0036	-0.4145	-0.3709	1.1987	-3.1378	12.1941	-407.2984
2.0200	-397.3256	1.6606	-2.5053	-0.3465	1.1345	-0.4931	-0.3658	1.2110	-3.1492	12.2239	-407.3174
2.0400	-400.8785	-5.0190	1.3157	-0.0856	1.2342	-0.5760	-0.3604	1.2235	-3.1606	12.2538	-407.3363
2.0600	-386.2407	6.1373	4.0575	0.2044	1.3029	-0.6641	-0.3545	1.2361	-3.1720	12.2836	-407.3553
2.0800	-400.1415	-5.4505	1.9839	0.2106	1.3458	-0.7559	-0.3480	1.2488	-3.1835	12.3135	-407.3742
2.1000	-398.9728	-1.1665	-0.6870	-0.0333	1.3567	-0.8494	-0.3408	1.2618	-3.1949	12.3433	-407.3932
2.1200	-395.8092	2.8930	-1.6483	-0.3553	1.3241	-0.9424	-0.3329	1.2750	-3.2063	12.3731	-407.4121

2.1400 -398.5788 0.5717 -1.8680 -
0.4700 1.2393 -1.0328 -0.3243 1.2884
-3.2177 12.4029 -407.4311
2.1600 -401.1352 -1.2472 -2.2367 -
0.1965 1.0986 -1.1185 -0.3151 1.3021
-3.2291 12.4327 -407.4500
2.1800 -399.1146 1.0592 -2.2455
0.4419 0.9007 -1.1972 -0.3051 1.3161
-3.2405 12.4625 -407.4690
2.2000 -398.6187 -1.3619 -0.9756
1.2207 0.6473 -1.2671 -0.2946 1.3303
-3.2519 12.4922 -407.4880
2.2200 -394.3598 0.0964 1.4376
1.8457 0.3418 -1.3263 -0.2834 1.3448
-3.2633 12.5220 -407.5069
2.2400 -390.8490 2.0422 3.2679
2.0661 -0.0103 -1.3731 -0.2716 1.3596
-3.2747 12.5517 -407.5259
2.2600 -395.7930 -1.2362 2.6598
1.7345 -0.3983 -1.4057 -0.2591 1.3746
-3.2860 12.5814 -407.5448
2.2800 -396.3045 2.4019 -0.0711
0.9360 -0.8033 -1.4223 -0.2462 1.3899
-3.2973 12.6111 -407.5638
2.3000 -404.2333 -1.8204 -2.3952 -
0.0793 -1.2028 -1.4216 -0.2327 1.4054
-3.3086 12.6407 -407.5827
2.3200 -405.9029 -2.2361 -2.3474 -
1.0519 -1.5716 -1.4021 -0.2187 1.4212
-3.3199 12.6704 -407.6017
2.3400 -400.5355 2.0283 -0.4404 -
1.7924 -1.8837 -1.3628 -0.2043 1.4372
-3.3312 12.7000 -407.6207
2.3600 -400.3499 1.4121 0.8436 -
2.1776 -2.1140 -1.3029 -0.1895 1.4535
-3.3424 12.7295 -407.6396
2.3800 -401.2283 1.0792 0.1671 -
2.1593 -2.2443 -1.2223 -0.1743 1.4699
-3.3536 12.7591 -407.6586
2.4000 -406.8755 -4.0161 -0.7225 -
1.8048 -2.2683 -1.1215 -0.1589 1.4866
-3.3647 12.7886 -407.6775
2.4200 -401.9526 -0.3344 -0.2092 -
1.2351 -2.1895 -1.0017 -0.1432 1.5035
-3.3758 12.8181 -407.6965
2.4400 -395.9995 3.3496 0.5788 -
0.5555 -2.0178 -0.8646 -0.1275 1.5207
-3.3869 12.8476 -407.7155
2.4600 -399.5210 -1.4296 0.2766
0.1529 -1.7683 -0.7124 -0.1118 1.5380
-3.3979 12.8770 -407.7344

2.4800 -399.6093 -1.4147 -0.3167
0.8235 -1.4598 -0.5479 -0.0960 1.5555
-3.4089 12.9064 -407.7534
2.5000 -394.8041 1.9642 -0.0353
1.3873 -1.1113 -0.3736 -0.0805 1.5732
-3.4198 12.9358 -407.7724
2.5200 -398.1329 -2.7990 0.7152
1.7806 -0.7404 -0.1921 -0.0651 1.5911
-3.4307 12.9651 -407.7913
2.5400 -391.3546 3.1898 0.6934
1.9396 -0.3623 -0.0062 -0.0500 1.6091
-3.4416 12.9944 -407.8103
2.5600 -395.1029 -0.4652 -0.3359
1.7964 0.0100 0.1814 -0.0353 1.6273
-3.4524 13.0237 -407.8293
2.5800 -396.5953 -1.0109 -0.9400
1.3301 0.3646 0.3680 -0.0211 1.6457
-3.4631 13.0529 -407.8482
2.6000 -396.4981 -1.8290 0.1172
0.5732 0.6929 0.5506 -0.0074 1.6642
-3.4738 13.0821 -407.8672
2.6200 -390.8892 3.0461 1.7019 -
0.3588 0.9876 0.7264 0.0057 1.6828
-3.4845 13.1112 -407.8862
2.6400 -394.8519 -0.9090 1.5125 -
1.2880 1.2416 0.8925 0.0182 1.7015
-3.4951 13.1403 -407.9051
2.6600 -397.0009 -0.8181 -0.3379 -
2.0216 1.4487 1.0458 0.0298 1.7203
-3.5056 13.1694 -407.9241
2.6800 -399.0318 0.0748 -2.1741 -
2.3656 1.6048 1.1837 0.0407 1.7393
-3.5160 13.1984 -407.9431
2.7000 -397.2715 0.7030 -2.4520 -
2.1680 1.7074 1.3036 0.0506 1.7583
-3.5264 13.2274 -407.9621
2.7200 -398.3635 -2.9784 -0.6786 -
1.4267 1.7543 1.4034 0.0595 1.7774
-3.5367 13.2563 -407.9811
2.7400 -388.7137 3.2323 1.7090 -
0.3358 1.7468 1.4814 0.0674 1.7966
-3.5469 13.2851 -408.0000
2.7600 -390.6874 0.1999 2.0790
0.8349 1.6881 1.5365 0.0742 1.8158
-3.5571 13.3140 -408.0190
2.7800 -390.2967 1.4488 0.2951
1.8008 1.5821 1.5679 0.0799 1.8350
-3.5672 13.3427 -408.0380
2.8000 -395.4914 -4.4299 -0.6696
2.3332 1.4338 1.5753 0.0844 1.8543
-3.5772 13.3714 -408.0570

2.8200 -387.7314 3.5400 0.5529
2.3325 1.2519 1.5591 0.0879 1.8736
-3.5871 13.4001 -408.0760
2.8400 -388.9000 0.9107 1.5634
1.8614 1.0488 1.5196 0.0903 1.8929
-3.5969 13.4287 -408.0950
2.8600 -391.1448 1.8260 0.3527
1.0533 0.8392 1.4576 0.0917 1.9121
-3.6067 13.4573 -408.1140
2.8800 -397.9685 -1.5284 -1.4879
0.0788 0.6384 1.3742 0.0922 1.9314
-3.6164 13.4858 -408.1330
2.9000 -401.3259 -4.0420 -1.6457 -
0.8654 0.4605 1.2706 0.0918 1.9506
-3.6259 13.5142 -408.1519
2.9200 -393.5220 2.9934 -0.0861 -
1.6001 0.3174 1.1486 0.0907 1.9697
-3.6354 13.5426 -408.1709
2.9400 -393.5280 2.4448 0.8479 -
2.0440 0.2164 1.0102 0.0890 1.9888
-3.6448 13.5709 -408.1899
2.9600 -397.4594 -0.4952 0.2361 -
2.1701 0.1587 0.8576 0.0867 2.0078
-3.6541 13.5992 -408.2089
2.9800 -401.0965 -3.1524 -0.6447 -
1.9674 0.1398 0.6934 0.0839 2.0267
-3.6633 13.6274 -408.2279
3.0000 -395.7023 2.6257 -0.8691 -
1.4365 0.1480 0.5204 0.0809 2.0455
-3.6724 13.6555 -408.2469
3.0200 -398.6521 -1.6513 -0.8341 -
0.6019 0.1665 0.3416 0.0776 2.0641
-3.6814 13.6836 -408.2660
3.0400 -394.9255 1.2120 -0.9112
0.4182 0.1769 0.1601 0.0742 2.0826
-3.6902 13.7116 -408.2850
3.0600 -394.8373 -0.5183 -0.4024
1.4163 0.1609 -0.0208 0.0707 2.1010
-3.6990 13.7396 -408.3040
3.0800 -394.9879 -2.1994 1.1827
2.1464 0.1030 -0.1979 0.0673 2.1192
-3.7077 13.7674 -408.3230
3.1000 -390.5901 0.5143 2.8044
2.3890 -0.0065 -0.3684 0.0641 2.1373
-3.7162 13.7952 -408.3420
3.1200 -388.2084 4.3689 2.3693
2.0274 -0.1691 -0.5292 0.0611 2.1551
-3.7247 13.8230 -408.3610
3.1400 -399.3165 -2.7571 -0.4427
1.1920 -0.3755 -0.6777 0.0585 2.1728
-3.7330 13.8507 -408.3800

3.1600	-401.7488	-2.3764	-2.3562	
0.1899	-0.6111	-0.8114	0.0564	2.1902
	-3.7412	13.8783	-408.3991	
3.1800	-402.9603	-3.4931	-1.2595	-
0.6644	-0.8601	-0.9281	0.0548	2.2075
	-3.7493	13.9058	-408.4181	
3.2000	-394.5284	4.2829	0.3605	-
1.1766	-1.1057	-1.0261	0.0538	2.2245
	-3.7572	13.9333	-408.4371	
3.2200	-397.2010	1.9784	0.3243	-
1.2604	-1.3317	-1.1036	0.0535	2.2412
	-3.7651	13.9606	-408.4561	
3.2400	-405.1873	-4.8978	-0.6190	-
1.0137	-1.5245	-1.1595	0.0541	2.2578
	-3.7728	13.9879	-408.4752	
3.2600	-396.5845	3.3428	-0.7358	-
0.6303	-1.6718	-1.1931	0.0554	2.2740
	-3.7803	14.0152	-408.4942	
3.2800	-397.5622	1.1014	0.3031	-
0.2796	-1.7627	-1.2040	0.0577	2.2900
	-3.7878	14.0423	-408.5132	
3.3000	-401.6354	-3.6833	1.4677	-
0.0632	-1.7935	-1.1922	0.0608	2.3057
	-3.7951	14.0694	-408.5323	
3.3200	-393.6639	3.5527	1.3393	-
0.0197	-1.7651	-1.1584	0.0649	2.3211
	-3.8022	14.0964	-408.5513	
3.3400	-397.3251	1.4477	-0.0316	-
0.1112	-1.6815	-1.1039	0.0699	2.3362
	-3.8092	14.1233	-408.5704	
3.3600	-403.7460	-3.9364	-1.1120	-
0.2316	-1.5484	-1.0309	0.0757	2.3511
	-3.8161	14.1502	-408.5894	
3.3800	-397.0020	2.1831	-0.5668	-
0.2709	-1.3724	-0.9415	0.0824	2.3656
	-3.8229	14.1769	-408.6085	
3.4000	-396.7378	1.1903	0.3514	-
0.1828	-1.1591	-0.8383	0.0897	2.3798
	-3.8294	14.2036	-408.6275	
3.4200	-398.0836	-1.4014	0.4480	
0.0245	-0.9143	-0.7240	0.0978	2.3936
	-3.8359	14.2302	-408.6466	
3.4400	-397.0472	-0.4039	0.1133	
0.2970	-0.6451	-0.6013	0.1065	2.4071
	-3.8422	14.2567	-408.6656	
3.4600	-395.9077	0.1849	-0.2380	
0.5364	-0.3589	-0.4733	0.1157	2.4203
	-3.8483	14.2831	-408.6847	
3.4800	-392.9564	3.7356	-0.9277	
0.6641	-0.0625	-0.3431	0.1255	2.4332
	-3.8543	14.3094	-408.7037	

3.5000	-401.1803	-5.2382	-0.5589	
0.6044	0.2362	-0.2137	0.1357	2.4457
	-3.8601	14.3357	-408.7228	
3.5200	-387.7714	5.0532	1.3287	
0.3358	0.5276	-0.0882	0.1462	2.4578
	-3.8657	14.3618	-408.7419	
3.5400	-394.4707	-0.9864	1.6333	-
0.0968	0.8012	0.0305	0.1572	2.4695
	-3.8712	14.3879	-408.7610	
3.5600	-397.1819	-2.5746	-0.1611	-
0.5643	1.0471	0.1395	0.1684	2.4809
	-3.8765	14.4138	-408.7800	
3.5800	-394.5993	2.7923	-2.1320	-
0.8457	1.2574	0.2364	0.1799	2.4919
	-3.8817	14.4397	-408.7991	
3.6000	-399.2911	-3.5859	-1.4663	-
0.7694	1.4248	0.3194	0.1915	2.5024
	-3.8867	14.4655	-408.8182	
3.6200	-389.9193	2.7370	0.8084	-
0.3783	1.5404	0.3872	0.2033	2.5126
	-3.8914	14.4911	-408.8373	
3.6400	-391.4934	0.4834	1.5494	
0.1801	1.5958	0.4390	0.2151	2.5224
	-3.8961	14.5167	-408.8564	
3.6600	-393.8750	-2.3563	0.5617	
0.7019	1.5838	0.4747	0.2269	2.5317
	-3.9005	14.5422	-408.8754	
3.6800	-390.3465	2.2666	-0.2832	
0.9991	1.4997	0.4944	0.2387	2.5406
	-3.9048	14.5676	-408.8945	
3.7000	-395.6071	-3.2561	0.4539	
0.9742	1.3429	0.4993	0.2502	2.5490
	-3.9088	14.5929	-408.9136	
3.7200	-392.0853	-0.7551	1.0380	
0.7023	1.1203	0.4907	0.2616	2.5570
	-3.9127	14.6181	-408.9327	
3.7400	-387.5002	6.0641	-0.0551	
0.3114	0.8454	0.4704	0.2726	2.5646
	-3.9164	14.6432	-408.9518	
3.7600	-403.2010	-6.5027	-1.5945	-
0.0952	0.5346	0.4401	0.2833	2.5717
	-3.9199	14.6681	-408.9709	
3.7800	-389.9181	5.2375	-0.2689	-
0.4171	0.2049	0.4018	0.2935	2.5782
	-3.9232	14.6930	-408.9900	
3.8000	-394.6736	0.3602	1.2261	-
0.5967	-0.1243	0.3573	0.3032	2.5844
	-3.9262	14.7178	-409.0092	
3.8200	-393.8214	1.9607	0.0484	-
0.6277	-0.4319	0.3086	0.3122	2.5900
	-3.9291	14.7424	-409.0283	

3.8400 -399.3448 -1.8948 -1.3726 -
0.5256 -0.6990 0.2576 0.3205 2.5951
-3.9318 14.7670 -409.0474
3.8600 -400.8927 -3.6892 -0.7029 -
0.3363 -0.9103 0.2065 0.3281 2.5997
-3.9343 14.7914 -409.0665
3.8800 -391.0666 3.8976 1.0986 -
0.1628 -1.0552 0.1572 0.3347 2.6039
-3.9365 14.8157 -409.0856
3.9000 -396.0141 -0.7445 1.2212 -
0.0843 -1.1309 0.1116 0.3404 2.6074
-3.9386 14.8400 -409.1048
3.9200 -398.4989 -1.9849 -0.0242 -
0.0891 -1.1408 0.0710 0.3451 2.6105
-3.9404 14.8641 -409.1239
3.9400 -396.5001 0.4801 -0.6971 -
0.1161 -1.0926 0.0368 0.3486 2.6131
-3.9420 14.8881 -409.1430
3.9600 -397.8000 -1.0528 -0.2671 -
0.1100 -0.9979 0.0099 0.3510 2.6151
-3.9433 14.9119 -409.1622
3.9800 -392.9055 3.5976 -0.1598 -
0.0259 -0.8711 -0.0089 0.3521 2.6165
-3.9445 14.9357 -409.1813
4.0000 -398.7113 -2.8445 -0.3476
0.1419 -0.7246 -0.0193 0.3520 2.6174
-3.9454 14.9593 -409.2005
4.0200 -398.8519 -2.8383 0.0156
0.3282 -0.5690 -0.0215 0.3506 2.6178
-3.9460 14.9829 -409.2196
4.0400 -390.5583 4.0133 0.5569
0.4483 -0.4114 -0.0161 0.3480 2.6176
-3.9465 15.0063 -409.2388
4.0600 -391.2240 3.6083 0.2645
0.4449 -0.2568 -0.0039 0.3441 2.6169
-3.9467 15.0296 -409.2579
4.0800 -400.9849 -5.7940 -0.1525
0.2942 -0.1105 0.0143 0.3389 2.6156
-3.9466 15.0527 -409.2771
4.1000 -389.9239 4.3463 0.3927 -
0.0006 0.0232 0.0378 0.3325 2.6137
-3.9463 15.0758 -409.2962
4.1200 -391.9555 2.1696 1.0399 -
0.3706 0.1415 0.0655 0.3250 2.6113
-3.9458 15.0987 -409.3154
4.1400 -398.6741 -3.5067 0.8466 -
0.7027 0.2423 0.0963 0.3163 2.6083
-3.9450 15.1215 -409.3346
4.1600 -397.0690 -0.6225 -0.2213 -
0.8614 0.3230 0.1288 0.3065 2.6047
-3.9440 15.1442 -409.3537

4.1800 -393.7543 2.9734 -1.4366 -
0.7593 0.3818 0.1618 0.2957 2.6005
-3.9427 15.1667 -409.3729
4.2000 -396.0120 1.7212 -2.4102 -
0.3561 0.4175 0.1937 0.2839 2.5958
-3.9412 15.1891 -409.3921
4.2200 -400.5281 -4.3341 -1.7925
0.2436 0.4311 0.2235 0.2711 2.5905
-3.9394 15.2114 -409.4113
4.2400 -388.7038 3.8694 1.1276
0.8428 0.4260 0.2501 0.2574 2.5846
-3.9373 15.2336 -409.4304
4.2600 -392.0393 -1.7696 3.1537
1.1833 0.4067 0.2725 0.2429 2.5782
-3.9350 15.2556 -409.4496
4.2800 -390.6715 1.0172 2.0763
1.0673 0.3767 0.2901 0.2277 2.5711
-3.9324 15.2775 -409.4688
4.3000 -393.9745 1.3741 -0.8114
0.5443 0.3394 0.3021 0.2118 2.5635
-3.9296 15.2993 -409.4880
4.3200 -400.9405 -3.1950 -2.6778 -
0.1741 0.3000 0.3082 0.1954 2.5553
-3.9265 15.3209 -409.5072
4.3400 -397.1984 0.4495 -1.7008 -
0.8380 0.2632 0.3079 0.1785 2.5466
-3.9231 15.3424 -409.5264
4.3600 -393.6835 2.4737 0.2254 -
1.2091 0.2340 0.3011 0.1611 2.5373
-3.9195 15.3638 -409.5456
4.3800 -394.2350 1.0387 0.5674 -
1.1168 0.2170 0.2876 0.1434 2.5274
-3.9156 15.3851 -409.5648
4.4000 -396.3178 0.0314 -0.7351 -
0.5629 0.2128 0.2674 0.1255 2.5170
-3.9115 15.4062 -409.5840
4.4200 -400.0748 -4.1811 -1.4049
0.2130 0.2185 0.2409 0.1072 2.5060
-3.9071 15.4272 -409.6032
4.4400 -395.2793 -0.4058 -0.2036
0.9079 0.2288 0.2082 0.0888 2.4944
-3.9024 15.4480 -409.6225
4.4600 -387.7234 4.7563 1.4722
1.2830 0.2366 0.1700 0.0703 2.4823
-3.8974 15.4687 -409.6417
4.4800 -396.4077 -4.2293 1.7729
1.1791 0.2342 0.1270 0.0516 2.4697
-3.8922 15.4892 -409.6609
4.5000 -390.7811 3.5277 0.6062
0.6163 0.2150 0.0802 0.0330 2.4565
-3.8867 15.5097 -409.6801

4.5200 -399.1596 -2.4978 -0.9927 -
0.1724 0.1763 0.0310 0.0143 2.4428
-3.8810 15.5300 -409.6994
4.5400 -397.5406 1.0035 -1.9206 -
0.8620 0.1173 -0.0193 -0.0042 2.4286
-3.8749 15.5501 -409.7186
4.5600 -399.8185 -1.7331 -1.2626 -
1.1877 0.0381 -0.0690 -0.0226 2.4138
-3.8686 15.5701 -409.7378
4.5800 -395.3025 1.0948 0.5612 -
1.0709 -0.0590 -0.1164 -0.0408 2.3985
-3.8621 15.5899 -409.7571
4.6000 -394.1732 0.1225 1.7770 -
0.5941 -0.1692 -0.1601 -0.0587 2.3827
-3.8552 15.6097 -409.7763
4.6200 -393.4123 1.2462 1.0884
0.0967 -0.2862 -0.1984 -0.0763 2.3664
-3.8481 15.6292 -409.7956
4.6400 -398.3048 -2.5924 -0.6247
0.7940 -0.4011 -0.2300 -0.0934 2.3496
-3.8407 15.6487 -409.8148
4.6600 -395.1368 1.9217 -1.3475
1.2782 -0.5042 -0.2539 -0.1100 2.3322
-3.8331 15.6679 -409.8341
4.6800 -395.9482 0.0476 -0.3774
1.3966 -0.5849 -0.2687 -0.1260 2.3144
-3.8251 15.6871 -409.8533
4.7000 -397.2918 -2.9181 1.2312
1.1059 -0.6361 -0.2736 -0.1413 2.2961
-3.8169 15.7061 -409.8726
4.7200 -389.3454 4.6705 1.8642
0.4583 -0.6564 -0.2682 -0.1558 2.2773
-3.8085 15.7249 -409.8918
4.7400 -399.4661 -2.4110 0.7146 -
0.3794 -0.6462 -0.2520 -0.1695 2.2581
-3.7997 15.7436 -409.9111
4.7600 -402.2996 -3.7359 -0.8089 -
1.1792 -0.6064 -0.2254 -0.1822 2.2384
-3.7907 15.7621 -409.9304
4.7800 -396.7179 3.3283 -1.5897 -
1.7168 -0.5378 -0.1886 -0.1940 2.2182
-3.7814 15.7805 -409.9496
4.8000 -397.1531 1.9538 -1.6621 -
1.8226 -0.4424 -0.1426 -0.2046 2.1975
-3.7718 15.7988 -409.9689
4.8200 -401.9323 -3.3333 -1.1346 -
1.4592 -0.3269 -0.0887 -0.2142 2.1764
-3.7620 15.8169 -409.9882
4.8400 -397.5574 -1.1175 0.1137 -
0.7374 -0.2011 -0.0285 -0.2225 2.1548
-3.7518 15.8348 -410.0075

4.8600 -391.1078 3.5138 1.1388
0.1567 -0.0758 0.0361 -0.2295 2.1328
-3.7414 15.8526 -410.0267
4.8800 -393.1014 0.8811 0.7716
1.0136 0.0382 0.1035 -0.2353 2.1104
-3.7308 15.8702 -410.0460
4.9000 -395.8746 -1.8389 0.0748
1.6312 0.1311 0.1714 -0.2398 2.0875
-3.7198 15.8877 -410.0653
4.9200 -395.1207 -2.1316 0.5199
1.8600 0.1953 0.2379 -0.2429 2.0642
-3.7086 15.9050 -410.0846
4.9400 -392.5524 0.4325 1.3967
1.6483 0.2275 0.3008 -0.2447 2.0405
-3.6971 15.9222 -410.1039
4.9600 -389.1946 3.8302 1.0709
1.0365 0.2321 0.3580 -0.2451 2.0163
-3.6853 15.9392 -410.1232
4.9800 -398.1128 -2.4442 -0.5513
0.1815 0.2189 0.4072 -0.2442 1.9918
-3.6732 15.9560 -410.1425
5.0000 -398.8109 -1.5439 -1.5266 -
0.6815 0.1998 0.4463 -0.2420 1.9668
-3.6609 15.9727 -410.1618
5.0200 -396.7630 0.0840 -0.5717 -
1.3241 0.1874 0.4733 -0.2385 1.9415
-3.6483 15.9892 -410.1811
5.0400 -396.3569 -0.1350 0.8759 -
1.6005 0.1950 0.4868 -0.2338 1.9158
-3.6354 16.0056 -410.2004
5.0600 -393.1931 3.5285 0.5531 -
1.4175 0.2320 0.4858 -0.2278 1.8897
-3.6222 16.0218 -410.2197
5.0800 -400.3863 -3.1497 -1.3180 -
0.8337 0.3022 0.4696 -0.2207 1.8632
-3.6087 16.0379 -410.2391
5.1000 -396.1603 1.1573 -2.0022 -
0.0632 0.4047 0.4384 -0.2124 1.8363
-3.5950 16.0538 -410.2584
5.1200 -395.4342 -0.8126 -0.5302
0.6455 0.5363 0.3927 -0.2029 1.8091
-3.5810 16.0695 -410.2777
5.1400 -392.9061 0.2166 1.5857
1.1018 0.6919 0.3338 -0.1924 1.7815
-3.5667 16.0851 -410.2970
5.1600 -391.3635 0.6456 2.6993
1.1653 0.8618 0.2628 -0.1807 1.7536
-3.5521 16.1005 -410.3163
5.1800 -391.5130 0.4917 2.3630
0.8106 1.0275 0.1816 -0.1680 1.7253
-3.5372 16.1157 -410.3357

5.2000 -394.8293 -0.7648 0.8335
0.1836 1.1654 0.0920 -0.1543 1.6967
-3.5221 16.1308 -410.3550
5.2200 -397.0097 -0.1960 -1.3709 -
0.4659 1.2518 -0.0040 -0.1396 1.6677
-3.5067 16.1457 -410.3743
5.2400 -398.0466 1.3917 -3.1832 -
0.8604 1.2652 -0.1039 -0.1240 1.6383
-3.4910 16.1604 -410.3937
5.2600 -401.7043 -3.1130 -2.8661 -
0.7977 1.1894 -0.2055 -0.1074 1.6087
-3.4750 16.1750 -410.4130
5.2800 -393.7579 2.4459 -0.4172 -
0.2714 1.0200 -0.3063 -0.0900 1.5786
-3.4587 16.1894 -410.4323
5.3000 -392.3030 1.3478 1.7168
0.5054 0.7653 -0.4039 -0.0718 1.5483
-3.4422 16.2036 -410.4517
5.3200 -394.0482 -1.3571 2.1196
1.2483 0.4388 -0.4960 -0.0529 1.5176
-3.4254 16.2177 -410.4710
5.3400 -392.9953 -0.3226 1.6677
1.6869 0.0571 -0.5805 -0.0332 1.4866
-3.4083 16.2316 -410.4904
5.3600 -395.7108 -1.6867 1.4842
1.6465 -0.3593 -0.6555 -0.0128 1.4552
-3.3909 16.2453 -410.5097
5.3800 -392.5741 3.5204 0.7472
1.1351 -0.7849 -0.7190 0.0081 1.4235
-3.3732 16.2588 -410.5291
5.4000 -400.7747 -2.1708 -0.9636
0.3020 -1.1906 -0.7690 0.0297 1.3916
-3.3553 16.2722 -410.5485
5.4200 -404.1724 -2.7945 -1.8680 -
0.6786 -1.5477 -0.8036 0.0517 1.3593
-3.3370 16.2854 -410.5678
5.4400 -401.6813 -0.6900 -0.8596 -
1.6005 -1.8277 -0.8211 0.0742 1.3266
-3.3185 16.2985 -410.5872
5.4600 -400.4269 0.6468 0.6442 -
2.2112 -2.0038 -0.8201 0.0970 1.2937
-3.2997 16.3113 -410.6065
5.4800 -396.0800 4.9398 0.2407 -
2.3135 -2.0540 -0.7996 0.1202 1.2604
-3.2806 16.3240 -410.6259
5.5000 -404.0947 -1.1621 -1.9869 -
1.8686 -1.9699 -0.7597 0.1435 1.2269
-3.2613 16.3365 -410.6453
5.5200 -407.3873 -4.6669 -3.2905 -
0.9614 -1.7609 -0.7015 0.1668 1.1930
-3.2417 16.3489 -410.6647

5.5400 -399.2122 0.4870 -1.5142
0.2434 -1.4482 -0.6273 0.1901 1.1589
-3.2217 16.3610 -410.6840
5.5600 -394.7826 -0.9972 2.1721
1.3912 -1.0561 -0.5391 0.2133 1.1244
-3.2015 16.3730 -410.7034
5.5800 -387.2177 4.5816 3.7446
2.0722 -0.6094 -0.4396 0.2360 1.0896
-3.1811 16.3848 -410.7228
5.6000 -396.1291 -2.8039 1.6903
2.0174 -0.1334 -0.3314 0.2584 1.0546
-3.1603 16.3964 -410.7422
5.6200 -394.2902 1.3527 -0.7114
1.3300 0.3464 -0.2171 0.2801 1.0192
-3.1393 16.4079 -410.7616
5.6400 -398.8407 -2.5595 -1.0521
0.2712 0.8017 -0.0997 0.3010 0.9836
-3.1180 16.4191 -410.7809
5.6600 -395.2249 2.2717 -0.7562 -
0.7771 1.2047 0.0183 0.3211 0.9476
-3.0964 16.4302 -410.8003
5.6800 -396.4774 0.0875 -0.9421 -
1.4409 1.5293 0.1343 0.3402 0.9114
-3.0745 16.4411 -410.8197
5.7000 -399.7710 -3.0916 -1.1002 -
1.5376 1.7562 0.2456 0.3581 0.8749
-3.0524 16.4518 -410.8391
5.7200 -391.6740 4.0888 -0.5594 -
1.0897 1.8786 0.3501 0.3747 0.8381
-3.0299 16.4623 -410.8585
5.7400 -398.0045 -4.5216 0.5677 -
0.2872 1.8988 0.4458 0.3899 0.8011
-3.0072 16.4727 -410.8779
5.7600 -387.6718 4.8751 1.1222
0.5997 1.8234 0.5310 0.4036 0.7637
-2.9843 16.4829 -410.8973
5.7800 -396.8534 -4.6531 0.3496
1.3177 1.6609 0.6047 0.4158 0.7261
-2.9610 16.4929 -410.9167
5.8000 -388.6417 3.7016 -0.6780
1.7092 1.4210 0.6666 0.4262 0.6882
-2.9375 16.5027 -410.9361
5.8200 -393.4789 0.7234 -0.8989
1.7110 1.1165 0.7163 0.4349 0.6501
-2.9137 16.5123 -410.9555
5.8400 -396.9706 -3.8632 -0.0835
1.3323 0.7661 0.7538 0.4417 0.6117
-2.8897 16.5217 -410.9749
5.8600 -391.3986 1.4068 1.3070
0.6579 0.3932 0.7789 0.4466 0.5730
-2.8653 16.5309 -410.9943

5.8800 -391.7106 2.5078 1.9398 -
0.1714 0.0234 0.7917 0.4496 0.5340
-2.8407 16.5400 -411.0138
5.9000 -396.4379 -1.1303 1.0492 -
0.9881 -0.3178 0.7922 0.4506 0.4948
-2.8158 16.5488 -411.0332
5.9200 -399.5717 -0.8372 -0.7017 -
1.6345 -0.6070 0.7803 0.4496 0.4553
-2.7907 16.5575 -411.0526
5.9400 -398.9401 1.4075 -2.1237 -
1.9710 -0.8250 0.7564 0.4466 0.4155
-2.7653 16.5660 -411.0720
5.9600 -402.3335 -1.5050 -2.1782 -
1.8880 -0.9584 0.7208 0.4416 0.3755
-2.7396 16.5743 -411.0914
5.9800 -400.5299 -1.6844 -0.5620 -
1.3917 -1.0041 0.6741 0.4347 0.3352
-2.7137 16.5824 -411.1109
6.0000 -393.3654 2.3693 1.3634 -
0.6363 -0.9720 0.6170 0.4259 0.2946
-2.6875 16.5903 -411.1303
6.0200 -393.1029 1.4835 1.6705
0.1688 -0.8786 0.5506 0.4153 0.2538
-2.6610 16.5980 -411.1497
6.0400 -395.9639 -0.6220 0.5867
0.8348 -0.7423 0.4762 0.4030 0.2127
-2.6343 16.6055 -411.1692
6.0600 -398.7273 -3.1292 -0.0970
1.2420 -0.5836 0.3952 0.3890 0.1713
-2.6073 16.6129 -411.1886
6.0800 -395.9396 -0.5929 0.1221
1.3506 -0.4226 0.3091 0.3734 0.1297
-2.5800 16.6200 -411.2080
6.1000 -391.8301 4.1213 0.2206
1.2123 -0.2747 0.2197 0.3562 0.0878
-2.5525 16.6270 -411.2275
6.1200 -400.3353 -4.3028 -0.2325
0.9375 -0.1515 0.1286 0.3376 0.0457
-2.5248 16.6337 -411.2469
6.1400 -393.1977 3.4078 -0.2350
0.6344 -0.0603 0.0375 0.3176 0.0033
-2.4967 16.6403 -411.2663
6.1600 -397.5002 -1.0770 -0.0608
0.3878 -0.0052 -0.0522 0.2963 -0.0394
-2.4685 16.6466 -411.2858
6.1800 -397.8914 -0.2224 -0.1811
0.2377 0.0132 -0.1389 0.2738 -0.0823
-2.4399 16.6528 -411.3052
6.2000 -398.3856 -1.1789 -0.2598
0.1670 -0.0003 -0.2214 0.2502 -0.1254
-2.4111 16.6588 -411.3247

6.2200 -394.8090 2.1418 -0.2560
0.1348 -0.0369 -0.2986 0.2255 -0.1688
-2.3821 16.6645 -411.3441
6.2400 -397.2331 0.4003 -0.1930
0.0646 -0.0840 -0.3696 0.1998 -0.2124
-2.3528 16.6701 -411.3636
6.2600 -401.3399 -3.6456 0.3739 -
0.1122 -0.1272 -0.4336 0.1733 -0.2562
-2.3233 16.6755 -411.3830
6.2800 -394.4293 2.4982 1.2764 -
0.4131 -0.1552 -0.4897 0.1459 -0.3003
-2.2935 16.6806 -411.4025
6.3000 -394.1866 3.0858 1.2602 -
0.8001 -0.1630 -0.5371 0.1178 -0.3446
-2.2635 16.6856 -411.4219
6.3200 -400.5821 -1.4864 -0.0797 -
1.1820 -0.1501 -0.5748 0.0891 -0.3891
-2.2332 16.6904 -411.4414
6.3400 -403.4334 -2.6226 -1.3684 -
1.4212 -0.1165 -0.6021 0.0598 -0.4338
-2.2027 16.6949 -411.4608
6.3600 -398.4156 2.3763 -1.5865 -
1.3861 -0.0657 -0.6182 0.0300 -0.4787
-2.1720 16.6993 -411.4803
6.3800 -400.5181 -0.3779 -1.0771 -
1.0464 -0.0059 -0.6227 -0.0002 -0.5238
-2.1410 16.7035 -411.4998
6.4000 -400.6021 -1.5959 0.0484 -
0.4667 0.0508 -0.6154 -0.0306 -0.5691
-2.1098 16.7074 -411.5192
6.4200 -397.9435 -1.2521 1.5675
0.2199 0.0911 -0.5964 -0.0612 -0.6145
-2.0783 16.7112 -411.5387
6.4400 -392.7319 2.9210 1.9689
0.8717 0.1015 -0.5657 -0.0919 -0.6602
-2.0466 16.7148 -411.5582
6.4600 -391.7195 4.1523 0.4479
1.3600 0.0708 -0.5237 -0.1225 -0.7059
-2.0147 16.7181 -411.5776
6.4800 -402.2523 -3.7642 -1.5568
1.5847 -0.0061 -0.4714 -0.1530 -0.7519
-1.9825 16.7213 -411.5971
6.5000 -403.8268 -5.1549 -1.5675
1.4877 -0.1285 -0.4094 -0.1832 -0.7980
-1.9501 16.7242 -411.6166
6.5200 -394.8442 2.6063 0.4197
1.0814 -0.2873 -0.3393 -0.2130 -0.8442
-1.9175 16.7269 -411.6360
6.5400 -392.0838 4.4187 1.9143
0.4351 -0.4679 -0.2625 -0.2422 -0.8905
-1.8847 16.7295 -411.6555

6.5600 -400.1100 -2.8034 1.2166 -
0.3188 -0.6511 -0.1805 -0.2707 -0.9370
-1.8516 16.7318 -411.6750
6.5800 -400.0712 0.1901 -0.6029 -
0.9731 -0.8155 -0.0951 -0.2985 -0.9835
-1.8183 16.7339 -411.6945
6.6000 -402.4289 -0.7271 -1.4942 -
1.3417 -0.9400 -0.0076 -0.3253 -1.0302
-1.7848 16.7358 -411.7139
6.6200 -403.8600 -1.8623 -1.5013 -
1.3303 -1.0050 0.0801 -0.3510 -1.0770
-1.7511 16.7375 -411.7334
6.6400 -396.9357 4.6106 -1.7548 -
0.9917 -0.9961 0.1664 -0.3756 -1.1238
-1.7171 16.7390 -411.7529
6.6600 -404.2416 -3.3787 -1.5941 -
0.5121 -0.9086 0.2495 -0.3989 -1.1707
-1.6830 16.7403 -411.7724
6.6800 -403.9789 -4.8714 0.3136 -
0.1330 -0.7439 0.3275 -0.4208 -1.2176
-1.6486 16.7414 -411.7919
6.7000 -390.6190 4.9998 3.2338
0.0079 -0.5081 0.3991 -0.4411 -1.2646
-1.6140 16.7422 -411.8114
6.7200 -393.2534 1.1041 4.0452 -
0.1286 -0.2121 0.4626 -0.4599 -1.3115
-1.5792 16.7429 -411.8309
6.7400 -397.4654 -0.7232 1.7461 -
0.4822 0.1270 0.5164 -0.4770 -1.3585
-1.5442 16.7433 -411.8503
6.7600 -402.7785 -2.3416 -1.6066 -
0.8723 0.4871 0.5592 -0.4924 -1.4055
-1.5089 16.7436 -411.8698
6.7800 -401.7262 0.0117 -3.7064 -
1.0725 0.8452 0.5894 -0.5059 -1.4525
-1.4735 16.7436 -411.8893
6.8000 -401.0901 0.1863 -3.7910 -
0.8989 1.1786 0.6058 -0.5175 -1.4994
-1.4379 16.7434 -411.9088
6.8200 -400.2383 -0.4953 -2.2386 -
0.3481 1.4673 0.6071 -0.5273 -1.5463
-1.4020 16.7430 -411.9283
6.8400 -396.8526 -0.8991 0.4230
0.4043 1.6964 0.5923 -0.5353 -1.5931
-1.3660 16.7424 -411.9478
6.8600 -389.6655 2.8168 2.8732
1.0757 1.8533 0.5607 -0.5416 -1.6398
-1.3297 16.7415 -411.9673
6.8800 -394.7543 -2.7058 3.5109
1.4001 1.9263 0.5122 -0.5462 -1.6864
-1.2933 16.7405 -411.9868

6.9000 -389.9480 3.1001 2.1831
1.2615 1.9072 0.4472 -0.5492 -1.7328
-1.2567 16.7392 -412.0063
6.9200 -398.4723 -1.9069 -0.1858
0.7723 1.7972 0.3671 -0.5508 -1.7791
-1.2198 16.7378 -412.0258
6.9400 -399.4867 -0.7102 -1.8537
0.1551 1.6027 0.2739 -0.5511 -1.8252
-1.1828 16.7361 -412.0453
6.9600 -400.3300 -1.8069 -1.3881 -
0.3547 1.3319 0.1698 -0.5501 -1.8711
-1.1456 16.7342 -412.0648
6.9800 -396.2425 2.2911 0.0033 -
0.5585 0.9963 0.0575 -0.5482 -1.9168
-1.1082 16.7321 -412.0843
7.0000 -397.2635 1.0080 0.1927 -
0.3693 0.6116 -0.0605 -0.5453 -1.9623
-1.0706 16.7297 -412.1038
7.0200 -401.9450 -2.7299 -0.6358
0.1004 0.1976 -0.1816 -0.5416 -2.0074
-1.0328 16.7272 -412.1233
7.0400 -398.8989 1.4940 -0.9469
0.5988 -0.2230 -0.3033 -0.5373 -2.0523
-0.9948 16.7244 -412.1428
7.0600 -398.1010 0.6345 -0.1910
0.8793 -0.6280 -0.4229 -0.5325 -2.0968
-0.9566 16.7214 -412.1623
7.0800 -400.5905 -2.0820 1.2697
0.7807 -0.9976 -0.5377 -0.5273 -2.1410
-0.9183 16.7182 -412.1819
7.1000 -400.4873 -1.1386 2.1559
0.2637 -1.3167 -0.6449 -0.5219 -2.1848
-0.8798 16.7148 -412.2014
7.1200 -396.3497 5.3676 1.1635 -
0.5652 -1.5746 -0.7418 -0.5164 -2.2282
-0.8411 16.7112 -412.2209
7.1400 -405.8501 -1.7354 -1.3267 -
1.4632 -1.7643 -0.8259 -0.5110 -2.2712
-0.8022 16.7073 -412.2404
7.1600 -411.8937 -5.8174 -2.7110 -
2.1365 -1.8808 -0.8946 -0.5057 -2.3137
-0.7632 16.7032 -412.2599
7.1800 -402.5566 2.7629 -1.4018 -
2.3188 -1.9239 -0.9458 -0.5008 -2.3557
-0.7239 16.6990 -412.2794
7.2000 -399.5181 4.1275 0.4391 -
1.9282 -1.8987 -0.9778 -0.4963 -2.3972
-0.6846 16.6944 -412.2989
7.2200 -403.1257 -0.7821 0.4360 -
1.0031 -1.8165 -0.9891 -0.4924 -2.4381
-0.6450 16.6897 -412.3185

7.2400 -403.8545 -0.7206 -1.0380
0.2643 -1.6930 -0.9790 -0.4891 -2.4785
-0.6053 16.6848 -412.3380
7.2600 -402.7315 -2.2065 -1.1116
1.5335 -1.5447 -0.9468 -0.4865 -2.5183
-0.5654 16.6796 -412.3575
7.2800 -400.2707 -2.9152 1.2858
2.4562 -1.3865 -0.8926 -0.4848 -2.5575
-0.5253 16.6742 -412.3770
7.3000 -392.1305 2.4932 3.6695
2.7948 -1.2319 -0.8168 -0.4841 -2.5961
-0.4851 16.6686 -412.3965
7.3200 -392.0310 3.5774 2.9546
2.4110 -1.0895 -0.7203 -0.4844 -2.6340
-0.4447 16.6628 -412.4161
7.3400 -403.7584 -4.0524 -0.5674
1.4310 -0.9583 -0.6048 -0.4859 -2.6712
-0.4042 16.6567 -412.4356
7.3600 -403.1391 0.5430 -3.3018
0.1662 -0.8322 -0.4728 -0.4886 -2.7076
-0.3635 16.6505 -412.4551
7.3800 -401.7498 2.6573 -3.0906 -
1.0478 -0.7029 -0.3271 -0.4927 -2.7434
-0.3227 16.6440 -412.4746
7.4000 -404.5778 -1.8989 -0.4287 -
1.9670 -0.5587 -0.1707 -0.4982 -2.7783
-0.2817 16.6373 -412.4941
7.4200 -402.1418 -1.8327 2.2326 -
2.4638 -0.3861 -0.0066 -0.5052 -2.8125
-0.2405 16.6303 -412.5137
7.4400 -395.5437 4.1789 2.6136 -
2.4569 -0.1751 0.1623 -0.5138 -2.8459
-0.1992 16.6232 -412.5332
7.4600 -403.0825 -2.9009 0.4198 -
1.9423 0.0722 0.3330 -0.5241 -2.8784
-0.1578 16.6158 -412.5527
7.4800 -404.1058 -2.9845 -1.5267 -
1.0314 0.3454 0.5028 -0.5362 -2.9100
-0.1162 16.6082 -412.5722
7.5000 -398.6560 0.7737 -1.5697
0.0951 0.6292 0.6692 -0.5500 -2.9408
-0.0745 16.6004 -412.5918
7.5200 -393.3670 3.8416 -0.7964
1.2189 0.9051 0.8295 -0.5658 -2.9706
-0.0326 16.5923 -412.6113
7.5400 -398.9580 -4.1556 -0.0800
2.0835 1.1543 0.9811 -0.5836 -2.9995
0.0094 16.5841 -412.6308
7.5600 -389.6756 3.8910 1.1013
2.3997 1.3584 1.1214 -0.6034 -3.0274
0.0515 16.5756 -412.6504

7.5800 -396.7416 -4.2948 2.2990
2.0485 1.5037 1.2478 -0.6253 -3.0543
0.0938 16.5669 -412.6699
7.6000 -390.0291 4.6131 1.2725
1.1676 1.5883 1.3579 -0.6494 -3.0803
0.1362 16.5579 -412.6894
7.6200 -401.8113 -3.6780 -1.5012
0.1069 1.6179 1.4497 -0.6758 -3.1052
0.1788 16.5488 -412.7090
7.6400 -402.6817 -2.2810 -2.6710 -
0.7514 1.6007 1.5214 -0.7044 -3.1290
0.2214 16.5394 -412.7285
7.6600 -397.6523 1.9129 -1.5068 -
1.1626 1.5480 1.5718 -0.7355 -3.1517
0.2642 16.5298 -412.7480
7.6800 -394.9761 3.4277 -0.3090 -
1.1412 1.4746 1.5999 -0.7689 -3.1733
0.3071 16.5199 -412.7676
7.7000 -393.2996 4.2592 -0.2687 -
0.8337 1.3954 1.6051 -0.8046 -3.1938
0.3502 16.5099 -412.7871
7.7200 -404.0515 -6.5879 0.2339 -
0.4880 1.3233 1.5871 -0.8425 -3.2132
0.3933 16.4996 -412.8066
7.7400 -396.1289 -0.5678 2.0512 -
0.3138 1.2697 1.5459 -0.8826 -3.2313
0.4366 16.4891 -412.8262
7.7600 -388.7551 6.3346 3.2811 -
0.3214 1.2409 1.4820 -0.9247 -3.2482
0.4800 16.4784 -412.8457
7.7800 -395.3136 0.8049 1.9820 -
0.4279 1.2340 1.3961 -0.9684 -3.2639
0.5235 16.4674 -412.8652
7.8000 -403.5415 -4.3352 -0.9393 -
0.4914 1.2383 1.2896 -1.0137 -3.2784
0.5671 16.4563 -412.8848
7.8200 -400.2957 0.7389 -3.3987 -
0.3798 1.2404 1.1644 -1.0602 -3.2915
0.6109 16.4448 -412.9043
7.8400 -402.1533 -0.2825 -3.2608 -
0.0062 1.2259 1.0226 -1.1078 -3.3033
0.6547 16.4332 -412.9238
7.8600 -401.0766 -2.4239 -0.6181
0.5464 1.1805 0.8668 -1.1562 -3.3138
0.6986 16.4214 -412.9434
7.8800 -393.7416 1.4920 2.2993
1.0758 1.0924 0.6997 -1.2051 -3.3229
0.7426 16.4093 -412.9629
7.9000 -392.5651 1.6178 2.9996
1.3676 0.9510 0.5243 -1.2544 -3.3306
0.7868 16.3970 -412.9824

7.9200 -397.7608 -1.1403 1.2791
1.2887 0.7501 0.3433 -1.3038 -3.3369
0.8310 16.3845 -413.0020
7.9400 -399.9733 -0.5655 -0.5980
0.8772 0.4946 0.1596 -1.3530 -3.3418
0.8753 16.3717 -413.0215
7.9600 -401.0342 0.1565 -0.9930
0.2482 0.1983 -0.0242 -1.4018 -3.3453
0.9197 16.3587 -413.0411
7.9800 -401.3867 0.3580 -0.3833 -
0.4063 -0.1215 -0.2054 -1.4500 -3.3472
0.9642 16.3455 -413.0606
8.0000 -401.4455 0.5505 0.3747 -
0.8810 -0.4459 -0.3813 -1.4973 -3.3476
1.0088 16.3321 -413.0801
8.0200 -405.6645 -3.0637 0.4607 -
1.0467 -0.7552 -0.5494 -1.5436 -3.3466
1.0535 16.3184 -413.0997
8.0400 -398.6399 5.4695 -1.0850 -
0.8975 -1.0315 -0.7072 -1.5885 -3.3439
1.0982 16.3045 -413.1192
8.0600 -411.7000 -6.1940 -2.1170 -
0.5557 -1.2606 -0.8520 -1.6319 -3.3397
1.1430 16.2904 -413.1388
8.0800 -400.6399 2.9401 0.0135 -
0.1919 -1.4321 -0.9816 -1.6735 -3.3340
1.1879 16.2761 -413.1583
8.1000 -397.0398 4.0218 2.5630
0.0221 -1.5387 -1.0940 -1.7130 -3.3266
1.2329 16.2615 -413.1778
8.1200 -400.2912 0.9278 2.4281 -
0.0153 -1.5788 -1.1876 -1.7503 -3.3176
1.2779 16.2467 -413.1974
8.1400 -404.4212 -0.5741 0.0511 -
0.2211 -1.5579 -1.2612 -1.7851 -3.3069
1.3230 16.2317 -413.2169
8.1600 -407.7117 -1.2460 -2.2423 -
0.4046 -1.4874 -1.3142 -1.8172 -3.2946
1.3682 16.2164 -413.2365
8.1800 -407.7127 -1.5172 -2.7463 -
0.3740 -1.3803 -1.3464 -1.8464 -3.2806
1.4134 16.2010 -413.2560
8.2000 -404.0598 0.7244 -1.3743 -
0.0445 -1.2479 -1.3580 -1.8727 -3.2649
1.4586 16.1852 -413.2755
8.2200 -401.3574 1.0233 0.4099
0.4810 -1.1008 -1.3499 -1.8957 -3.2474
1.5039 16.1693 -413.2951
8.2400 -402.4138 -1.9445 1.3223
1.0282 -0.9480 -1.3230 -1.9155 -3.2283
1.5493 16.1532 -413.3146

8.2600 -397.8206 2.3392 1.3321
1.3958 -0.7972 -1.2788 -1.9321 -3.2074
1.5947 16.1368 -413.3342
8.2800 -401.1299 -0.8497 1.1175
1.4091 -0.6535 -1.2189 -1.9453 -3.1848
1.6401 16.1202 -413.3537
8.3000 -401.4657 -1.0879 1.1121
1.0261 -0.5187 -1.1451 -1.9552 -3.1605
1.6856 16.1033 -413.3732
8.3200 -400.3893 0.7180 0.7672
0.3430 -0.3905 -1.0592 -1.9618 -3.1344
1.7311 16.0862 -413.3928
8.3400 -400.3000 2.1815 -0.3506 -
0.4396 -0.2635 -0.9632 -1.9650 -3.1066
1.7766 16.0689 -413.4123
8.3600 -407.2122 -2.7233 -1.5241 -
1.0880 -0.1323 -0.8592 -1.9649 -3.0770
1.8222 16.0514 -413.4319
8.3800 -403.2425 1.4925 -1.7589 -
1.4155 0.0083 -0.7493 -1.9615 -3.0456
1.8677 16.0337 -413.4514
8.4000 -404.0283 -0.8656 -1.0009 -
1.3733 0.1606 -0.6356 -1.9548 -3.0126
1.9133 16.0157 -413.4709
8.4200 -402.4451 -0.3215 0.4030 -
1.0612 0.3218 -0.5202 -1.9448 -2.9778
1.9589 15.9975 -413.4905
8.4400 -398.2174 1.1465 1.7555 -
0.6395 0.4868 -0.4052 -1.9315 -2.9413
2.0045 15.9790 -413.5100
8.4600 -396.9509 1.2188 1.6796 -
0.2383 0.6474 -0.2922 -1.9150 -2.9030
2.0501 15.9603 -413.5296
8.4800 -397.2313 2.1727 -0.3017
0.0973 0.7930 -0.1829 -1.8953 -2.8631
2.0956 15.9414 -413.5491
8.5000 -405.2792 -3.7577 -2.0425
0.4106 0.9107 -0.0787 -1.8725 -2.8215
2.1412 15.9223 -413.5686
8.5200 -403.2791 -3.6835 -1.5403
0.7410 0.9867 0.0193 -1.8466 -2.7783
2.1867 15.9030 -413.5882
8.5400 -392.2946 4.6121 0.4779
1.0642 1.0104 0.1100 -1.8176 -2.7334
2.2323 15.8834 -413.6077
8.5600 -393.4127 2.7748 1.5585
1.3056 0.9768 0.1925 -1.7858 -2.6869
2.2778 15.8636 -413.6273
8.5800 -400.5157 -4.0388 1.0441
1.3940 0.8872 0.2661 -1.7510 -2.6388
2.3233 15.8435 -413.6468

8.6000	-396.5348	0.7573	-0.0724	
1.2902	0.7484	0.3301	-1.7134	-2.5891
	2.3687	15.8232	-413.6663	
8.6200	-396.8578	0.8097	-0.4664	
0.9964	0.5717	0.3839	-1.6730	-2.5379
	2.4141	15.8027	-413.6859	
8.6400	-399.6609	-1.3544	-0.0727	
0.5625	0.3724	0.4275	-1.6300	-2.4851
	2.4595	15.7820	-413.7054	
8.6600	-397.5433	1.2049	0.1740	
0.0749	0.1670	0.4609	-1.5844	-2.4308
	2.5048	15.7610	-413.7249	
8.6800	-400.1018	0.0141	-0.3981	-
0.3809	-0.0266	0.4841	-1.5363	-2.3751
	2.5501	15.7399	-413.7445	
8.7000	-401.9972	-1.5298	-0.8371	-
0.7772	-0.1914	0.4975	-1.4859	-2.3179
	2.5954	15.7184	-413.7640	
8.7200	-399.3618	0.4289	0.0207	-
1.1141	-0.3137	0.5012	-1.4331	-2.2592
	2.6405	15.6968	-413.7836	
8.7400	-398.8656	0.0246	1.2534	-
1.3742	-0.3831	0.4958	-1.3782	-2.1992
	2.6856	15.6749	-413.8031	
8.7600	-397.6181	1.2645	1.1192	-
1.5376	-0.3953	0.4817	-1.3212	-2.1379
	2.7307	15.6528	-413.8226	
8.7800	-399.7667	0.7849	-0.4334	-
1.5518	-0.3521	0.4598	-1.2624	-2.0752
	2.7756	15.6305	-413.8422	
8.8000	-403.7029	-2.8409	-1.5030	-
1.3468	-0.2606	0.4310	-1.2018	-2.0112
	2.8205	15.6079	-413.8617	
8.8200	-400.6492	-0.7899	-0.7866	-
0.9122	-0.1330	0.3963	-1.1395	-1.9459
	2.8654	15.5851	-413.8812	
8.8400	-395.9731	1.8602	0.5413	-
0.3538	0.0150	0.3567	-1.0757	-1.8795
	2.9101	15.5621	-413.9008	
8.8600	-394.8574	1.6139	1.0166	
0.1963	0.1658	0.3130	-1.0106	-1.8119
	2.9547	15.5388	-413.9203	
8.8800	-397.5840	-1.2767	0.3933	
0.6439	0.3014	0.2661	-0.9441	-1.7432
	2.9993	15.5153	-413.9398	
8.9000	-397.4000	-0.0919	-0.5434	
0.9687	0.4066	0.2168	-0.8764	-1.6734
	3.0437	15.4916	-413.9594	
8.9200	-398.1055	-1.5084	-0.4517	
1.1786	0.4693	0.1661	-0.8075	-1.6025
	3.0881	15.4677	-413.9789	

8.9400 -395.4534 -0.2739 0.5456
1.2768 0.4805 0.1146 -0.7377 -1.5307
3.1323 15.4435 -413.9984
8.9600 -392.0908 2.6449 0.9262
1.2842 0.4355 0.0630 -0.6668 -1.4580
3.1765 15.4191 -414.0179
8.9800 -398.0596 -1.8797 -0.1003
1.1860 0.3347 0.0119 -0.5950 -1.3843
3.2205 15.3945 -414.0375
9.0000 -397.8459 0.1982 -1.1771
0.9555 0.1851 -0.0380 -0.5224 -1.3098
3.2644 15.3696 -414.0570
9.0200 -397.1948 0.1204 -0.9978
0.5885 -0.0014 -0.0859 -0.4491 -1.2346
3.3081 15.3445 -414.0765
9.0400 -396.1842 0.2631 0.4039
0.0853 -0.2069 -0.1307 -0.3751 -1.1586
3.3518 15.3192 -414.0961
9.0600 -397.8793 -1.6943 2.0158 -
0.5104 -0.4099 -0.1717 -0.3006 -1.0818
3.3953 15.2937 -414.1156
9.0800 -395.6518 0.3399 2.3669 -
1.0998 -0.5897 -0.2078 -0.2255 -1.0045
3.4387 15.2679 -414.1351
9.1000 -394.9000 3.6982 0.4761 -
1.5494 -0.7278 -0.2382 -0.1501 -0.9265
3.4819 15.2419 -414.1546
9.1200 -404.8957 -3.3697 -2.4810 -
1.7168 -0.8086 -0.2621 -0.0744 -0.8480
3.5250 15.2157 -414.1742
9.1400 -405.3802 -3.0831 -3.5725 -
1.4913 -0.8213 -0.2788 0.0016 -0.7690
3.5679 15.1892 -414.1937
9.1600 -395.2400 4.1526 -1.7945 -
0.8844 -0.7622 -0.2877 0.0777 -0.6896
3.6107 15.1625 -414.2132
9.1800 -397.8034 -2.0191 1.3133 -
0.1146 -0.6346 -0.2887 0.1539 -0.6097
3.6534 15.1356 -414.2327
9.2000 -394.2393 -1.1728 3.2494
0.5253 -0.4459 -0.2817 0.2299 -0.5295
3.6958 15.1085 -414.2523
9.2200 -388.7150 3.3024 2.8781
0.7987 -0.2073 -0.2673 0.3058 -0.4489
3.7381 15.0811 -414.2718
9.2400 -394.1279 0.4903 0.4467
0.6543 0.0637 -0.2456 0.3814 -0.3681
3.7802 15.0535 -414.2913
9.2600 -397.4178 -0.6321 -2.2377
0.2438 0.3399 -0.2171 0.4565 -0.2871
3.8222 15.0256 -414.3108

9.2800	-401.1595	-3.7222	-2.3950	-
0.2042	0.5898	-0.1822	0.5310	-0.2059
	3.8639	14.9976	-414.3303	
9.3000	-391.9335	3.2968	-0.3712	-
0.4546	0.7817	-0.1414	0.6049	-0.1246
	3.9055	14.9693	-414.3499	
9.3200	-393.3314	0.6282	0.6834	-
0.3285	0.8911	-0.0952	0.6778	-0.0432
	3.9469	14.9408	-414.3694	
9.3400	-394.3781	-0.4016	-0.1500	
0.1964	0.9088	-0.0442	0.7497	0.0383
	3.9881	14.9120	-414.3889	
9.3600	-394.8241	-1.0823	-1.2992	
0.8802	0.8386	0.0108	0.8205	0.1197
	4.0291	14.8830	-414.4084	
9.3800	-392.5521	0.4893	-1.0912	
1.4155	0.6882	0.0689	0.8900	0.2011
	4.0699	14.8538	-414.4279	
9.4000	-392.6862	-1.0864	0.7183	
1.5675	0.4685	0.1290	0.9581	0.2824
	4.1105	14.8244	-414.4474	
9.4200	-389.4440	-0.2113	2.6966	
1.2391	0.1964	0.1901	1.0246	0.3635
	4.1508	14.7948	-414.4669	
9.4400	-388.5742	2.5811	2.5971	
0.4476	-0.0998	0.2509	1.0894	0.4445
	4.1910	14.7649	-414.4864	
9.4600	-392.3670	2.3865	-0.2709	-
0.5915	-0.3853	0.3101	1.1523	0.5252
	4.2309	14.7348	-414.5060	
9.4800	-402.9535	-4.4641	-2.9420	-
1.4773	-0.6249	0.3667	1.2133	0.6056
	4.2706	14.7044	-414.5255	
9.5000	-394.1729	3.8496	-2.1080	-
1.8561	-0.7852	0.4196	1.2720	0.6857
	4.3101	14.6739	-414.5450	
9.5200	-397.6872	-2.3779	0.1444	-
1.6893	-0.8416	0.4678	1.3285	0.7654
	4.3494	14.6431	-414.5645	
9.5400	-391.1827	2.3319	1.0239	-
1.1845	-0.7876	0.5108	1.3825	0.8447
	4.3884	14.6120	-414.5840	
9.5600	-395.7887	-3.6606	1.8296	-
0.6642	-0.6285	0.5476	1.4340	0.9236
	4.4271	14.5808	-414.6035	
9.5800	-386.4889	4.6232	1.9257	-
0.3084	-0.3809	0.5775	1.4829	1.0019
	4.4656	14.5493	-414.6230	
9.6000	-393.3340	-0.9058	-0.0100	-
0.1301	-0.0738	0.6000	1.5290	1.0797
	4.5039	14.5176	-414.6425	

9.6200 -396.7699 -3.4498 -1.8243
0.0128 0.2536 0.6145 1.5723 1.1569
4.5419 14.4857 -414.6620
9.6400 -392.1537 0.6709 -1.5671
0.2678 0.5614 0.6207 1.6127 1.2334
4.5796 14.4536 -414.6815
9.6600 -389.2085 2.2056 -0.3417
0.6839 0.8154 0.6182 1.6503 1.3093
4.6171 14.4212 -414.7010
9.6800 -389.2285 0.3929 0.0826
1.2268 0.9901 0.6068 1.6848 1.3844
4.6543 14.3886 -414.7205
9.7000 -391.1039 -1.8202 -0.1925
1.7242 1.0699 0.5868 1.7163 1.4587
4.6912 14.3558 -414.7400
9.7200 -388.3252 0.7271 0.1678
1.9213 1.0466 0.5587 1.7449 1.5322
4.7279 14.3227 -414.7595
9.7400 -391.1877 -3.4936 1.6415
1.6421 0.9222 0.5233 1.7705 1.6048
4.7642 14.2895 -414.7790
9.7600 -383.3921 5.0145 1.8964
0.9117 0.7124 0.4817 1.7931 1.6766
4.8003 14.2560 -414.7985
9.7800 -396.8339 -4.0284 -0.2009 -
0.0536 0.4413 0.4350 1.8129 1.7473
4.8361 14.2223 -414.8180
9.8000 -397.3462 -2.3157 -1.9994 -
0.9220 0.1364 0.3843 1.8299 1.8170
4.8716 14.1883 -414.8375
9.8200 -391.8372 3.7455 -1.9311 -
1.4111 -0.1727 0.3312 1.8442 1.8857
4.9067 14.1542 -414.8569
9.8400 -397.7578 -3.1111 -0.4489 -
1.4392 -0.4566 0.2767 1.8560 1.9532
4.9416 14.1198 -414.8764
9.8600 -390.0059 2.4511 1.2348 -
1.1449 -0.6900 0.2221 1.8652 2.0196
4.9761 14.0852 -414.8959
9.8800 -393.4298 -2.0103 1.9727 -
0.7562 -0.8528 0.1683 1.8720 2.0848
5.0104 14.0504 -414.9154
9.9000 -390.0585 2.6575 0.8666 -
0.4329 -0.9316 0.1160 1.8765 2.1488
5.0443 14.0153 -414.9349
9.9200 -394.7390 -0.9536 -0.7936 -
0.2397 -0.9236 0.0657 1.8789 2.2114
5.0779 13.9801 -414.9544
9.9400 -396.4636 -3.2812 -1.3116 -
0.1712 -0.8345 0.0181 1.8792 2.2727
5.1111 13.9446 -414.9738

9.9600 -388.7880 4.0296 -0.5040 -
0.1609 -0.6752 -0.0263 1.8776 2.3327
5.1441 13.9089 -414.9933
9.9800 -393.6249 -1.7491 0.1137 -
0.1157 -0.4618 -0.0668 1.8744 2.3911
5.1766 13.8729 -415.0128
10.0000 -393.6600 -1.9798 0.5452 -
0.0078 -0.2131 -0.1030 1.8696 2.4482
5.2089 13.8368 -415.0323
10.0200 -388.8231 2.3922 0.8797
0.1394 0.0495 -0.1342 1.8636 2.5036
5.2408 13.8004 -415.0518
10.0400 -390.7692 -0.2128 0.4173
0.3094 0.3029 -0.1600 1.8564 2.5576
5.2723 13.7639 -415.0712
10.0600 -389.2941 2.7970 -1.4369
0.5092 0.5223 -0.1802 1.8484 2.6099
5.3035 13.7271 -415.0907
10.0800 -397.1300 -4.7899 -2.1560
0.7312 0.6864 -0.1944 1.8398 2.6606
5.3343 13.6900 -415.1102
10.1000 -390.7297 -0.4624 -0.2408
0.9423 0.7785 -0.2024 1.8307 2.7096
5.3647 13.6528 -415.1296
10.1200 -382.4086 5.1906 2.2882
1.0453 0.7890 -0.2039 1.8215 2.7568
5.3948 13.6154 -415.1491
10.1400 -388.9866 -2.0185 2.9115
0.9523 0.7137 -0.1990 1.8124 2.8023
5.4245 13.5777 -415.1686
10.1600 -392.2561 -3.5226 1.6853
0.6556 0.5553 -0.1882 1.8037 2.8460
5.4538 13.5398 -415.1880
10.1800 -389.8210 1.1787 -0.4982
0.2526 0.3267 -0.1718 1.7956 2.8878
5.4827 13.5017 -415.2075
10.2000 -390.4074 4.0250 -2.5199 -
0.0923 0.0479 -0.1506 1.7884 2.9276
5.5112 13.4634 -415.2270
10.2200 -396.4231 -1.4020 -3.0786 -
0.2434 -0.2578 -0.1255 1.7823 2.9656
5.5394 13.4249 -415.2464
10.2400 -398.6962 -4.1834 -1.5237 -
0.1901 -0.5600 -0.0972 1.7778 3.0015
5.5671 13.3861 -415.2659
10.2600 -386.6151 4.4145 1.2633 -
0.0534 -0.8262 -0.0667 1.7750 3.0354
5.5944 13.3472 -415.2853
10.2800 -390.8048 -1.2786 2.5235 -
0.0064 -1.0248 -0.0348 1.7741 3.0672
5.6214 13.3080 -415.3048

10.3000 -390.9158 0.2019 1.8035 -
0.1915 -1.1285 -0.0024 1.7755 3.0969
5.6479 13.2686 -415.3242
10.3200 -393.2116 -0.1694 0.6339 -
0.6391 -1.1225 0.0295 1.7795 3.1244
5.6740 13.2290 -415.3437
10.3400 -395.3669 -1.6432 -0.0230 -
1.2454 -1.0088 0.0599 1.7861 3.1498
5.6996 13.1892 -415.3631
10.3600 -393.7485 0.9265 -0.2778 -
1.7821 -0.8002 0.0877 1.7957 3.1729
5.7249 13.1492 -415.3826
10.3800 -394.7496 0.3102 -0.8767 -
1.9472 -0.5143 0.1115 1.8085 3.1938
5.7497 13.1090 -415.4020
10.4000 -393.6856 1.9965 -2.2303 -
1.5428 -0.1730 0.1299 1.8246 3.2123
5.7741 13.0686 -415.4215
10.4200 -398.4736 -3.1156 -2.9331 -
0.6008 0.1947 0.1414 1.8443 3.2286
5.7980 13.0279 -415.4409
10.4400 -390.3033 0.9224 -1.2748
0.6585 0.5558 0.1448 1.8676 3.2426
5.8215 12.9871 -415.4604
10.4600 -387.0503 0.4633 1.2659
1.8708 0.8768 0.1387 1.8948 3.2542
5.8446 12.9460 -415.4798
10.4800 -383.0614 1.8421 2.6807
2.6433 1.1243 0.1219 1.9259 3.2635
5.8672 12.9047 -415.4992
10.5000 -388.5936 -3.4153 2.7945
2.6874 1.2701 0.0934 1.9609 3.2705
5.8894 12.8632 -415.5187
10.5200 -382.3989 4.6743 1.0875
2.0224 1.3051 0.0527 2.0000 3.2751
5.9111 12.8216 -415.5381
10.5400 -395.4485 -4.6775 -1.5877
0.9292 1.2408 -0.0000 2.0431 3.2774
5.9324 12.7797 -415.5575
10.5600 -390.7618 2.6837 -2.6355 -
0.2155 1.0958 -0.0642 2.0901 3.2774
5.9532 12.7376 -415.5770
10.5800 -393.1567 1.3099 -2.2234 -
1.0879 0.8903 -0.1387 2.1409 3.2751
5.9735 12.6953 -415.5964
10.6000 -395.1922 -0.7699 -1.4358 -
1.5199 0.6449 -0.2224 2.1954 3.2704
5.9934 12.6527 -415.6158
10.6200 -393.5918 0.0692 -0.2524 -
1.5395 0.3801 -0.3137 2.2534 3.2636
6.0129 12.6100 -415.6353

10.6400 -392.6983 -1.1792 1.5734 -
1.3362 0.1162 -0.4112 2.3146 3.2545
6.0319 12.5671 -415.6547
10.6600 -389.5099 0.5263 3.0857 -
1.0728 -0.1267 -0.5136 2.3789 3.2432
6.0504 12.5240 -415.6741
10.6800 -388.1292 2.7856 2.7502 -
0.8455 -0.3319 -0.6192 2.4459 3.2297
6.0685 12.4807 -415.6935
10.7000 -394.6329 -2.5173 0.4801 -
0.6626 -0.4887 -0.7268 2.5156 3.2141
6.0861 12.4371 -415.7129
10.7200 -395.4558 -0.0492 -1.9054 -
0.4859 -0.5934 -0.8347 2.5876 3.1963
6.1032 12.3934 -415.7323
10.7400 -396.1432 0.5168 -2.7997 -
0.2715 -0.6457 -0.9415 2.6616 3.1765
6.1199 12.3495 -415.7518
10.7600 -395.7563 -0.9855 -1.7481 -
0.0008 -0.6491 -1.0456 2.7373 3.1547
6.1361 12.3053 -415.7712
10.7800 -392.2938 0.4706 0.3630
0.2832 -0.6112 -1.1452 2.8144 3.1309
6.1519 12.2610 -415.7906
10.8000 -391.0535 -0.2760 2.1621
0.4973 -0.5402 -1.2385 2.8926 3.1051
6.1672 12.2164 -415.8100
10.8200 -388.0092 2.6165 2.1906
0.5648 -0.4462 -1.3240 2.9715 3.0773
6.1821 12.1717 -415.8294
10.8400 -393.1037 -0.8614 0.4929
0.4722 -0.3404 -1.4000 3.0509 3.0477
6.1965 12.1268 -415.8488
10.8600 -397.4467 -3.5610 -0.6646
0.2561 -0.2353 -1.4648 3.1303 3.0163
6.2104 12.0816 -415.8682
10.8800 -393.5967 -0.4061 -0.0100 -
0.0130 -0.1433 -1.5170 3.2096 2.9830
6.2239 12.0363 -415.8876
10.9000 -390.1743 2.5860 0.7071 -
0.2172 -0.0737 -1.5549 3.2883 2.9480
6.2370 11.9908 -415.9070
10.9200 -389.7448 3.4194 -0.2510 -
0.2358 -0.0318 -1.5772 3.3661 2.9113
6.2496 11.9451 -415.9264
10.9400 -398.5192 -3.0435 -1.9322 -
0.0427 -0.0193 -1.5826 3.4427 2.8729
6.2617 11.8991 -415.9458
10.9600 -396.9100 -2.2566 -1.9794
0.2955 -0.0350 -1.5702 3.5178 2.8329
6.2734 11.8530 -415.9652

10.9800 -390.8554 2.1600 0.0981
0.6366 -0.0770 -1.5393 3.5911 2.7913
6.2846 11.8067 -415.9845

11.0000 -390.6459 -0.8109 2.5136
0.8205 -0.1425 -1.4897 3.6622 2.7481
6.2954 11.7602 -416.0039

11.0200 -387.8067 1.4801 2.8858
0.7141 -0.2266 -1.4213 3.7308 2.7035
6.3057 11.7135 -416.0233

11.0400 -389.3633 2.2747 1.2189
0.3057 -0.3215 -1.3343 3.7966 2.6575
6.3155 11.6666 -416.0427

11.0600 -397.9995 -3.9397 -0.7669 -
0.2659 -0.4165 -1.2289 3.8592 2.6100
6.3250 11.6195 -416.0621

11.0800 -395.8543 -0.4091 -1.7365 -
0.8139 -0.5002 -1.1057 3.9185 2.5612
6.3339 11.5723 -416.0814

11.1000 -392.2230 4.4062 -2.1815 -
1.1231 -0.5610 -0.9654 3.9740 2.5111
6.3424 11.5248 -416.1008

11.1200 -398.0236 -1.5893 -2.6493 -
1.0533 -0.5902 -0.8091 4.0255 2.4598
6.3505 11.4771 -416.1202

11.1400 -398.7005 -3.4507 -1.9711 -
0.6763 -0.5859 -0.6381 4.0728 2.4073
6.3581 11.4293 -416.1396

11.1600 -393.3960 -0.8240 0.8023 -
0.1522 -0.5500 -0.4542 4.1156 2.3536
6.3653 11.3812 -416.1589

11.1800 -386.3542 2.0264 3.8753
0.3570 -0.4860 -0.2593 4.1536 2.2988
6.3720 11.3330 -416.1783

11.2000 -386.4365 1.6481 4.0894
0.7037 -0.3991 -0.0553 4.1865 2.2430
6.3783 11.2846 -416.1976

11.2200 -389.2617 1.6592 0.6601
0.8046 -0.2963 0.1558 4.2140 2.1861
6.3841 11.2360 -416.2170

11.2400 -399.5310 -4.5469 -3.4368
0.6896 -0.1850 0.3720 4.2360 2.1283
6.3895 11.1872 -416.2364

11.2600 -394.4286 1.8967 -4.9189
0.4169 -0.0727 0.5913 4.2522 2.0697
6.3944 11.1382 -416.2557

11.2800 -390.8757 3.1391 -2.4796
0.0522 0.0341 0.8115 4.2622 2.0101
6.3989 11.0891 -416.2751

11.3000 -394.4626 -5.5725 2.5595 -
0.3155 0.1302 1.0302 4.2659 1.9498
6.4029 11.0397 -416.2944

11.3200 -381.0049 5.6808 5.6736 -
0.5855 0.2110 1.2453 4.2631 1.8887
6.4065 10.9902 -416.3138
11.3400 -384.8862 2.5935 3.5050 -
0.6738 0.2738 1.4543 4.2534 1.8269
6.4097 10.9405 -416.3331
11.3600 -397.9081 -4.7743 -1.6056 -
0.5639 0.3182 1.6550 4.2367 1.7644
6.4124 10.8906 -416.3524
11.3800 -395.1770 1.1721 -5.0373 -
0.2701 0.3466 1.8455 4.2126 1.7013
6.4146 10.8405 -416.3718
11.4000 -396.7391 -1.1181 -4.6660
0.1737 0.3629 2.0238 4.1811 1.6377
6.4164 10.7903 -416.3911
11.4200 -392.2366 -0.1609 -2.0325
0.6718 0.3727 2.1880 4.1420 1.5735
6.4178 10.7398 -416.4105
11.4400 -387.8497 1.0089 1.0783
1.0939 0.3816 2.3363 4.0949 1.5088
6.4188 10.6892 -416.4298
11.4600 -387.4120 -0.7863 3.7475
1.2887 0.3955 2.4670 4.0399 1.4437
6.4192 10.6384 -416.4491
11.4800 -384.0287 1.1140 4.6231
1.1155 0.4190 2.5789 3.9767 1.3782
6.4193 10.5875 -416.4684
11.5000 -387.0703 1.2244 2.5638
0.5526 0.4564 2.6705 3.9052 1.3123
6.4189 10.5363 -416.4878
11.5200 -394.0101 -1.0687 -0.9237 -
0.2375 0.5097 2.7409 3.8255 1.2460
6.4181 10.4850 -416.5071
11.5400 -396.1318 -1.2585 -3.0217 -
1.0315 0.5794 2.7893 3.7375 1.1795
6.4168 10.4335 -416.5264
11.5600 -394.4177 1.3877 -2.7868 -
1.6138 0.6654 2.8153 3.6411 1.1127
6.4151 10.3818 -416.5457
11.5800 -395.4884 -0.3534 -1.4720 -
1.8252 0.7658 2.8185 3.5366 1.0457
6.4129 10.3300 -416.5650
11.6000 -393.6398 -0.8801 -0.1455 -
1.6061 0.8745 2.7988 3.4239 0.9785
6.4103 10.2779 -416.5844
11.6200 -391.5206 0.9060 0.8132 -
1.0213 0.9821 2.7566 3.3033 0.9111
6.4073 10.2257 -416.6037
11.6400 -391.5272 -0.0133 1.0922 -
0.2148 1.0782 2.6924 3.1750 0.8437
6.4038 10.1734 -416.6230

11.6600	-392.4766	-0.9917	0.6782	
0.6541	1.1506	2.6071	3.0392	0.7761
	6.3999	10.1208	-416.6423	
11.6800	-389.4245	2.4192	-0.1834	
1.4130	1.1841	2.5022	2.8963	0.7086
	6.3956	10.0681	-416.6616	
11.7000	-395.2020	-2.9436	-0.9211	
1.9095	1.1661	2.3790	2.7465	0.6410
	6.3908	10.0153	-416.6809	
11.7200	-391.5716	1.0735	-0.3296	
2.0475	1.0889	2.2393	2.5901	0.5735
	6.3856	9.9622	-416.7002	
11.7400	-393.5453	-2.4004	1.3930	
1.7562	0.9532	2.0849	2.4275	0.5060
	6.3800	9.9090	-416.7195	
11.7600	-387.8082	4.2578	1.7726	
1.0400	0.7660	1.9176	2.2591	0.4387
	6.3739	9.8556	-416.7387	
11.7800	-396.5358	-0.8738	-0.0995	
0.0425	0.5400	1.7394	2.0850	0.3716
	6.3673	9.8021	-416.7580	
11.8000	-403.1445	-3.3261	-1.9291	-
0.9785	0.2906	1.5520	1.9057	0.3046
	6.3604	9.7484	-416.7773	
11.8200	-399.4000	2.3624	-1.6897	-
1.7692	0.0343	1.3572	1.7216	0.2379
	6.3530	9.6945	-416.7966	
11.8400	-400.7830	-0.0979	-0.0435	-
2.1710	-0.2127	1.1567	1.5329	0.1715
	6.3452	9.6405	-416.8159	
11.8600	-399.4609	-0.0582	1.3064	-
2.1446	-0.4369	0.9521	1.3400	0.1053
	6.3369	9.5863	-416.8351	
11.8800	-400.7637	-0.9831	1.5920	-
1.7256	-0.6296	0.7451	1.1432	0.0396
	6.3282	9.5319	-416.8544	
11.9000	-399.4606	1.8320	0.4146	-
0.9965	-0.7876	0.5371	0.9430	-0.0258
	6.3191	9.4774	-416.8737	
11.9200	-403.6006	-0.2534	-1.4116	-
0.0934	-0.9100	0.3297	0.7396	-0.0908
	6.3096	9.4228	-416.8929	
11.9400	-404.5549	-1.6530	-1.9814	
0.8169	-0.9970	0.1242	0.5333	-0.1552
	6.2996	9.3679	-416.9122	
11.9600	-401.2139	0.4110	-0.5020	
1.5677	-1.0498	-0.0780	0.3247	-0.2192
	6.2892	9.3129	-416.9315	
11.9800	-398.7455	1.3016	1.4287	
1.9901	-1.0703	-0.2755	0.1139	-0.2826
	6.2783	9.2578	-416.9507	

12.0000 -399.9532 -0.0362 1.9700
1.9584 -1.0621 -0.4673 -0.0986 -0.3454
6.2670 9.2025 -416.9700
12.0200 -400.7980 0.4097 1.1790
1.4721 -1.0288 -0.6523 -0.3126 -0.4076
6.2553 9.1470 -416.9892
12.0400 -406.0461 -3.0180 0.3387
0.6480 -0.9726 -0.8300 -0.5276 -0.4692
6.2432 9.0914 -417.0085
12.0600 -403.5024 1.5841 -0.3357 -
0.3093 -0.8934 -0.9999 -0.7432 -0.5300
6.2306 9.0357 -417.0277
12.0800 -405.1546 3.3365 -1.2732 -
1.1480 -0.7907 -1.1620 -0.9592 -0.5901
6.2176 8.9798 -417.0470
12.1000 -413.5030 -4.4828 -2.0737 -
1.6582 -0.6639 -1.3163 -1.1752 -0.6494
6.2042 8.9237 -417.0662
12.1200 -409.7582 -0.0985 -1.7864 -
1.7287 -0.5122 -1.4632 -1.3907 -0.7079
6.1903 8.8675 -417.0854
12.1400 -405.9681 2.5728 -0.9799 -
1.3783 -0.3366 -1.6031 -1.6055 -0.7656
6.1761 8.8112 -417.1047
12.1600 -406.1414 1.9725 -0.6431 -
0.7226 -0.1421 -1.7364 -1.8192 -0.8223
6.1613 8.7547 -417.1239
12.1800 -410.8914 -3.6770 -0.1603
0.0546 0.0649 -1.8635 -2.0313 -0.8781
6.1462 8.6980 -417.1431
12.2000 -404.9857 0.4347 1.1287
0.7459 0.2775 -1.9849 -2.2416 -0.9329
6.1306 8.6412 -417.1624
12.2200 -401.0588 2.8950 2.5376
1.1810 0.4882 -2.1009 -2.4496 -0.9868
6.1147 8.5843 -417.1816
12.2400 -405.9340 -2.0721 3.2347
1.2441 0.6885 -2.2118 -2.6551 -1.0395
6.0982 8.5272 -417.2008
12.2600 -406.2897 -1.8383 2.8227
0.9265 0.8682 -2.3178 -2.8576 -1.0912
6.0814 8.4700 -417.2200
12.2800 -403.3377 3.8453 1.0728
0.3348 1.0159 -2.4189 -3.0569 -1.1418
6.0641 8.4126 -417.2392
12.3000 -413.4754 -3.0907 -1.3375 -
0.3269 1.1202 -2.5152 -3.2525 -1.1912
6.0465 8.3551 -417.2584
12.3200 -414.2781 -1.6693 -2.8070 -
0.8314 1.1701 -2.6067 -3.4441 -1.2394
6.0284 8.2975 -417.2776

12.3400	-410.5529	2.9979	-3.0976	-
0.9806	1.1566	-2.6933	-3.6314	-1.2863
	6.0098	8.2397	-417.2968	
12.3600	-411.7022	1.5231	-2.6034	-
0.6844	1.0754	-2.7748	-3.8142	-1.3320
	5.9909	8.1818	-417.3160	
12.3800	-416.7649	-4.3211	-1.0323	-
0.0418	0.9313	-2.8507	-3.9920	-1.3763
	5.9715	8.1237	-417.3352	
12.4000	-407.1622	1.9520	1.3844	
0.7321	0.7338	-2.9206	-4.1646	-1.4193
	5.9517	8.0655	-417.3544	
12.4200	-404.5629	3.1200	2.7868	
1.3929	0.4942	-2.9837	-4.3317	-1.4609
	5.9315	8.0072	-417.3736	
12.4400	-408.4037	-0.3759	2.2409	
1.7164	0.2241	-3.0392	-4.4929	-1.5010
	5.9108	7.9487	-417.3928	
12.4600	-412.5046	-2.1692	1.1951	
1.5760	-0.0639	-3.0861	-4.6480	-1.5397
	5.8898	7.8901	-417.4120	
12.4800	-412.5387	-0.9872	0.9089	
0.9746	-0.3557	-3.1236	-4.7968	-1.5768
	5.8683	7.8314	-417.4312	
12.5000	-410.9704	2.3812	0.5594	
0.0846	-0.6351	-3.1505	-4.9389	-1.6124
	5.8464	7.7726	-417.4503	
12.5200	-417.5911	-0.9949	-0.6139	-
0.8470	-0.8850	-3.1660	-5.0741	-1.6465
	5.8241	7.7136	-417.4695	
12.5400	-419.8666	-1.7256	-1.6549	-
1.5926	-1.0882	-3.1691	-5.2021	-1.6789
	5.8014	7.6545	-417.4887	
12.5600	-417.2022	1.1751	-1.3614	-
1.9733	-1.2286	-3.1592	-5.3228	-1.7096
	5.7782	7.5953	-417.5079	
12.5800	-418.4759	0.1239	-0.3516	-
1.9047	-1.2941	-3.1353	-5.4359	-1.7386
	5.7547	7.5359	-417.5270	
12.6000	-417.6681	0.1889	-0.1834	-
1.4085	-1.2835	-3.0969	-5.5412	-1.7659
	5.7307	7.4764	-417.5462	
12.6200	-415.6748	1.0004	-0.8540	-
0.6296	-1.2049	-3.0436	-5.6385	-1.7914
	5.7063	7.4168	-417.5653	
12.6400	-418.2824	-1.7189	-0.9552	
0.2117	-1.0710	-2.9751	-5.7277	-1.8151
	5.6815	7.3571	-417.5845	
12.6600	-414.5769	-0.4989	0.4841	
0.8942	-0.8956	-2.8915	-5.8086	-1.8369
	5.6563	7.2972	-417.6036	

12.6800 -411.3167 1.8117 2.3475
1.2683 -0.6930 -2.7932 -5.8811 -1.8568
5.6307 7.2372 -417.6228
12.7000 -413.6980 -1.1526 2.8857
1.2579 -0.4764 -2.6808 -5.9450 -1.8749
5.6046 7.1771 -417.6419
12.7200 -412.8811 0.3641 1.5750
0.9046 -0.2592 -2.5550 -6.0003 -1.8910
5.5782 7.1169 -417.6611
12.7400 -415.4218 0.8438 -0.8006
0.3684 -0.0532 -2.4167 -6.0470 -1.9051
5.5513 7.0566 -417.6802
12.7600 -419.7505 -1.5799 -2.7749 -
0.1522 0.1313 -2.2669 -6.0850 -1.9172
5.5241 6.9962 -417.6993
12.7800 -420.7629 -1.5790 -3.1554 -
0.4892 0.2859 -2.1067 -6.1144 -1.9273
5.4964 6.9356 -417.7185
12.8000 -415.7630 1.3431 -1.8082 -
0.5677 0.4040 -1.9374 -6.1351 -1.9354
5.4683 6.8749 -417.7376
12.8200 -412.7432 2.0500 0.2783 -
0.4268 0.4817 -1.7604 -6.1471 -1.9415
5.4399 6.8141 -417.7567
12.8400 -415.9939 -3.2886 2.2937 -
0.1779 0.5193 -1.5774 -6.1505 -1.9455
5.4110 6.7532 -417.7758
12.8600 -410.1470 1.3718 3.4325
0.0685 0.5205 -1.3901 -6.1454 -1.9475
5.3817 6.6922 -417.7949
12.8800 -410.7417 1.4145 2.6753
0.2477 0.4906 -1.2006 -6.1318 -1.9474
5.3520 6.6311 -417.8140
12.9000 -414.5802 -1.3169 0.3134
0.3437 0.4362 -1.0107 -6.1097 -1.9453
5.3220 6.5699 -417.8331
12.9200 -416.3008 -0.3857 -2.0252
0.3767 0.3644 -0.8222 -6.0791 -1.9412
5.2915 6.5085 -417.8523
12.9400 -417.4796 0.0699 -3.0068
0.3744 0.2846 -0.6372 -6.0402 -1.9351
5.2606 6.4471 -417.8714
12.9600 -417.1621 -0.5632 -2.3534
0.3532 0.2057 -0.4575 -5.9930 -1.9271
5.2294 6.3855 -417.8904
12.9800 -414.6709 -0.2088 -0.3471
0.2823 0.1372 -0.2849 -5.9375 -1.9171
5.1977 6.3239 -417.9095
13.0000 -410.7594 0.8473 1.9133
0.1195 0.0889 -0.1214 -5.8737 -1.9051
5.1657 6.2621 -417.9286

13.0200	-410.8418	0.8686	2.9283	-
0.1709	0.0689	0.0314	-5.8018	-1.8914
	5.1333	6.2003	-417.9477	
13.0400	-412.3591	0.3589	1.9389	-
0.5721	0.0804	0.1722	-5.7218	-1.8757
	5.1005	6.1383	-417.9668	
13.0600	-415.1337	-0.4554	-0.2504	-
0.9812	0.1216	0.2995	-5.6337	-1.8582
	5.0673	6.0762	-417.9859	
13.0800	-418.0585	-1.0189	-1.9956	-
1.2567	0.1880	0.4124	-5.5376	-1.8390
	5.0337	6.0141	-418.0049	
13.1000	-417.4318	-0.2012	-2.3266	-
1.2689	0.2735	0.5099	-5.4335	-1.8180
	4.9998	5.9518	-418.0240	
13.1200	-415.0855	0.1085	-1.3376	-
0.9580	0.3708	0.5911	-5.3216	-1.7953
	4.9654	5.8895	-418.0431	
13.1400	-413.2786	-0.2986	0.4568	-
0.3925	0.4720	0.6555	-5.2020	-1.7709
	4.9307	5.8270	-418.0622	
13.1600	-409.3146	1.2921	1.9271	
0.3053	0.5692	0.7026	-5.0746	-1.7449
	4.8957	5.7645	-418.0812	
13.1800	-408.5927	1.3856	1.4901	
1.0033	0.6530	0.7324	-4.9398	-1.7173
	4.8603	5.7018	-418.1003	
13.2000	-411.4248	0.2343	-0.4549	
1.5703	0.7140	0.7449	-4.7977	-1.6882
	4.8245	5.6391	-418.1193	
13.2200	-414.7531	-2.8407	-1.6019	
1.9071	0.7442	0.7404	-4.6484	-1.6575
	4.7883	5.5763	-418.1384	
13.2400	-410.3025	0.4413	-0.5403	
1.9421	0.7407	0.7194	-4.4923	-1.6254
	4.7518	5.5134	-418.1574	
13.2600	-408.5672	1.1757	1.4551	
1.6269	0.7073	0.6825	-4.3295	-1.5919
	4.7150	5.4504	-418.1765	
13.2800	-408.8081	0.6069	2.3459	
0.9593	0.6511	0.6306	-4.1603	-1.5570
	4.6777	5.3873	-418.1955	
13.3000	-411.7893	-1.1758	1.6092	
0.0003	0.5795	0.5647	-3.9852	-1.5207
	4.6402	5.3241	-418.2145	
13.3200	-412.4736	1.2112	0.1581	-
1.0747	0.4991	0.4860	-3.8042	-1.4831
	4.6023	5.2608	-418.2335	
13.3400	-416.0603	0.1460	-1.2124	-
2.0529	0.4161	0.3955	-3.6179	-1.4442
	4.5640	5.1975	-418.2526	

13.3600 -418.8990 -1.5889 -1.9397 -
2.7084 0.3365 0.2948 -3.4266 -1.4042
4.5254 5.1341 -418.2716
13.3800 -417.7304 0.1627 -1.6203 -
2.8325 0.2648 0.1854 -3.2307 -1.3629
4.4865 5.0705 -418.2906
13.4000 -415.1097 1.5969 -1.1789 -
2.3167 0.2028 0.0689 -3.0305 -1.3205
4.4472 5.0070 -418.3096
13.4200 -414.5052 0.9545 -1.3700 -
1.2346 0.1459 -0.0527 -2.8264 -1.2770
4.4077 4.9433 -418.3286
13.4400 -416.5996 -2.5359 -1.2228
0.1621 0.0854 -0.1773 -2.6189 -1.2324
4.3678 4.8795 -418.3476
13.4600 -412.8795 -2.0412 0.4135
1.5431 0.0115 -0.3025 -2.4083 -1.1868
4.3275 4.8157 -418.3666
13.4800 -406.7685 1.1378 2.6082
2.5877 -0.0853 -0.4260 -2.1951 -1.1402
4.2870 4.7518 -418.3856
13.5000 -404.5164 3.6335 2.9683
3.0256 -0.2132 -0.5456 -1.9796 -1.0927
4.2461 4.6878 -418.4046
13.5200 -413.6999 -3.8550 1.1226
2.7380 -0.3749 -0.6590 -1.7622 -1.0443
4.2049 4.6238 -418.4236
13.5400 -410.6202 1.4917 -0.5867
1.8645 -0.5637 -0.7638 -1.5434 -0.9950
4.1635 4.5596 -418.4426
13.5600 -413.1740 0.6808 -1.1495
0.6661 -0.7680 -0.8581 -1.3234 -0.9449
4.1217 4.4954 -418.4616
13.5800 -416.2548 -1.0579 -1.1072 -
0.5543 -0.9752 -0.9397 -1.1026 -0.8940
4.0796 4.4311 -418.4806
13.6000 -416.7903 0.1943 -1.0780 -
1.5183 -1.1727 -1.0067 -0.8815 -0.8423
4.0372 4.3668 -418.4995
13.6200 -417.2241 0.8279 -1.1822 -
2.0607 -1.3487 -1.0573 -0.6602 -0.7900
3.9946 4.3024 -418.5185
13.6400 -417.2716 -0.1771 -0.9841 -
2.1373 -1.4916 -1.0898 -0.4392 -0.7370
3.9516 4.2379 -418.5375
13.6600 -417.0337 -1.3264 -0.2040 -
1.7894 -1.5951 -1.1030 -0.2187 -0.6834
3.9084 4.1734 -418.5564
13.6800 -412.6612 2.5780 0.5455 -
1.1486 -1.6569 -1.0958 0.0009 -0.6292
3.8648 4.1088 -418.5754

13.7000 -413.5470 0.3698 0.3719 -
0.3886 -1.6764 -1.0675 0.2193 -0.5745
3.8211 4.0441 -418.5943
13.7200 -415.3948 -2.4042 -0.0637
0.3227 -1.6537 -1.0176 0.4363 -0.5193
3.7770 3.9793 -418.6133
13.7400 -413.6851 -1.6489 0.2840
0.8219 -1.5890 -0.9462 0.6515 -0.4637
3.7327 3.9145 -418.6322
13.7600 -407.2789 4.0971 0.8303
0.9989 -1.4862 -0.8536 0.8647 -0.4076
3.6881 3.8497 -418.6512
13.7800 -408.8498 1.8640 0.7067
0.8253 -1.3534 -0.7405 1.0756 -0.3511
3.6432 3.7847 -418.6701
13.8000 -416.5759 -5.3027 0.6526
0.3699 -1.2041 -0.6080 1.2838 -0.2943
3.5981 3.7198 -418.6891
13.8200 -405.7879 4.7300 0.9644 -
0.2177 -1.0531 -0.4575 1.4892 -0.2373
3.5528 3.6547 -418.7080
13.8400 -411.9818 0.2383 0.0595 -
0.6867 -0.9147 -0.2903 1.6915 -0.1799
3.5071 3.5896 -418.7269
13.8600 -414.6300 -1.3153 -1.9176 -
0.8232 -0.8008 -0.1083 1.8903 -0.1224
3.4613 3.5245 -418.7458
13.8800 -414.6794 -0.8408 -3.0504 -
0.5381 -0.7159 0.0865 2.0855 -0.0647
3.4152 3.4593 -418.7647
13.9000 -412.4490 -0.0372 -2.1444
0.1260 -0.6572 0.2915 2.2768 -0.0069
3.3689 3.3940 -418.7837
13.9200 -409.2419 -0.4483 0.1964
0.9814 -0.6181 0.5041 2.4638 0.0511
3.3223 3.3287 -418.8026
13.9400 -405.6408 0.7552 2.6207
1.7533 -0.5906 0.7212 2.6463 0.1090
3.2756 3.2633 -418.8215
13.9600 -403.0908 -0.3373 3.8264
2.1640 -0.5667 0.9395 2.8241 0.1670
3.2286 3.1979 -418.8404
13.9800 -402.3127 0.7362 3.2106
2.0374 -0.5372 1.1558 2.9969 0.2249
3.1814 3.1324 -418.8593
14.0000 -405.4682 0.3556 1.0730
1.4019 -0.4895 1.3670 3.1644 0.2828
3.1339 3.0669 -418.8781
14.0200 -409.5629 -0.8197 -1.4078
0.4441 -0.4103 1.5697 3.3263 0.3406
3.0863 3.0014 -418.8970

14.0400 -410.6018 -0.0689 -3.0999 -
0.5943 -0.2880 1.7611 3.4824 0.3981
3.0384 2.9358 -418.9159
14.0600 -411.3061 0.8440 -3.5811 -
1.4822 -0.1127 1.9384 3.6324 0.4555
2.9904 2.8701 -418.9348
14.0800 -413.3998 -2.3648 -2.5473 -
2.0855 0.1223 2.0991 3.7761 0.5127
2.9422 2.8044 -418.9537
14.1000 -407.7084 0.6636 -0.0829 -
2.3574 0.4182 2.2414 3.9131 0.5696
2.8937 2.7387 -418.9725
14.1200 -403.5308 1.9318 2.1617 -
2.2968 0.7714 2.3636 4.0432 0.6261
2.8451 2.6729 -418.9914
14.1400 -402.9271 1.3618 2.3215 -
1.9164 1.1712 2.4646 4.1662 0.6823
2.7963 2.6071 -419.0103
14.1600 -404.6887 1.0403 0.0641 -
1.2515 1.5982 2.5434 4.2817 0.7381
2.7473 2.5413 -419.0291
14.1800 -406.9596 0.5749 -2.6149 -
0.3655 2.0241 2.5993 4.3897 0.7935
2.6982 2.4754 -419.0480
14.2000 -410.9169 -5.4192 -2.7814
0.6438 2.4181 2.6311 4.4899 0.8483
2.6488 2.4095 -419.0668
14.2200 -398.6495 2.6003 -0.0512
1.6380 2.7507 2.6381 4.5821 0.9027
2.5993 2.3435 -419.0856
14.2400 -394.3776 4.1349 2.5077
2.4469 2.9952 2.6193 4.6665 0.9564
2.5497 2.2775 -419.1045
14.2600 -398.3235 -1.6873 2.4966
2.9005 3.1286 2.5739 4.7429 1.0096
2.4999 2.2115 -419.1233
14.2800 -400.2164 -1.0887 0.8689
2.8849 3.1332 2.5020 4.8116 1.0621
2.4499 2.1454 -419.1421
14.3000 -402.1452 -0.7236 -0.0492
2.3575 3.0024 2.4045 4.8726 1.1140
2.3998 2.0793 -419.1610
14.3200 -401.1770 1.0104 0.5913
1.3947 2.7425 2.2836 4.9262 1.1652
2.3495 2.0132 -419.1798
14.3400 -403.8877 -1.5834 1.3554
0.2349 2.3693 2.1421 4.9727 1.2156
2.2991 1.9470 -419.1986
14.3600 -401.4409 4.4625 -0.0641 -
0.8228 1.9005 1.9829 5.0122 1.2652
2.2486 1.8809 -419.2174

14.3800 -412.4379 -3.0278 -2.4529 -
1.5286 1.3535 1.8091 5.0450 1.3140
2.1979 1.8147 -419.2362
14.4000 -416.0183 -5.0450 -2.8691 -
1.7028 0.7467 1.6237 5.0713 1.3619
2.1471 1.7484 -419.2550
14.4200 -407.8893 2.3182 -0.7397 -
1.3114 0.1019 1.4298 5.0914 1.4090
2.0961 1.6822 -419.2738
14.4400 -402.3716 4.9364 1.3108 -
0.5548 -0.5551 1.2303 5.1055 1.4551
2.0451 1.6159 -419.2926
14.4600 -409.5595 -1.4263 1.2577
0.2899 -1.1948 1.0282 5.1139 1.5003
1.9939 1.5496 -419.3114
14.4800 -411.1000 -2.0583 0.3122
0.9507 -1.7868 0.8267 5.1168 1.5446
1.9427 1.4833 -419.3302
14.5000 -410.5256 -1.2545 0.2752
1.2240 -2.3044 0.6286 5.1145 1.5878
1.8913 1.4169 -419.3490
14.5200 -407.3005 2.6560 0.7295
1.0646 -2.7280 0.4370 5.1071 1.6300
1.8398 1.3506 -419.3677
14.5400 -412.6343 -1.8315 0.9221
0.5414 -3.0453 0.2549 5.0949 1.6711
1.7882 1.2842 -419.3865
14.5600 -412.4031 -0.3039 0.6296 -
0.2057 -3.2473 0.0849 5.0781 1.7112
1.7365 1.2178 -419.4053
14.5800 -412.2239 2.7166 -0.2560 -
0.9830 -3.3277 -0.0706 5.0571 1.7502
1.6848 1.1514 -419.4240
14.6000 -418.5592 -2.1938 -1.2342 -
1.5952 -3.2828 -0.2099 5.0320 1.7881
1.6329 1.0850 -419.4428
14.6200 -417.0723 -0.5448 -0.9575 -
1.8997 -3.1113 -0.3323 5.0030 1.8249
1.5810 1.0185 -419.4615
14.6400 -414.1097 0.5906 0.2758 -
1.8500 -2.8203 -0.4380 4.9704 1.8605
1.5290 0.9521 -419.4803
14.6600 -411.3002 2.5357 0.7078 -
1.4929 -2.4248 -0.5274 4.9345 1.8949
1.4769 0.8856 -419.4990
14.6800 -415.2623 -1.3560 0.0289 -
0.9162 -1.9475 -0.6013 4.8955 1.9282
1.4247 0.8191 -419.5177
14.7000 -415.1973 -1.7911 -0.4076 -
0.2288 -1.4138 -0.6605 4.8536 1.9603
1.3725 0.7527 -419.5365

14.7200 −411.8514 −0.0447 0.1889
0.4597 −0.8492 −0.7056 4.8090 1.9911
1.3202 0.6862 −419.5552
14.7400 −407.7941 2.4669 0.7905
1.0430 −0.2798 −0.7376 4.7620 2.0207
1.2679 0.6197 −419.5739
14.7600 −408.9838 0.0604 0.3711
1.4260 0.2688 −0.7572 4.7129 2.0491
1.2155 0.5532 −419.5926
14.7800 −410.8419 −0.8327 −0.3421
1.5396 0.7732 −0.7655 4.6619 2.0762
1.1631 0.4867 −419.6113
14.8000 −411.4004 −1.0080 −0.0214
1.3683 1.2150 −0.7638 4.6091 2.1020
1.1106 0.4202 −419.6301
14.8200 −407.6500 1.4447 0.9745
0.9894 1.5825 −0.7536 4.5550 2.1265
1.0580 0.3537 −419.6488
14.8400 −408.9205 0.6527 0.9358
0.5343 1.8699 −0.7368 4.4996 2.1498
1.0055 0.2872 −419.6675
14.8600 −410.4122 0.8872 −0.8639
0.1044 2.0778 −0.7153 4.4433 2.1717
0.9529 0.2207 −419.6861
14.8800 −411.9317 1.8515 −2.5398 −
0.2503 2.2120 −0.6911 4.3863 2.1923
0.9002 0.1541 −419.7048
14.9000 −418.5709 −6.0373 −1.7229 −
0.5109 2.2826 −0.6660 4.3287 2.2116
0.8476 0.0876 −419.7235
14.9200 −407.9982 1.8312 1.4442 −
0.7116 2.3006 −0.6421 4.2710 2.2295
0.7949 0.0211 −419.7422
14.9400 −401.4304 6.3641 3.5032 −
0.9039 2.2755 −0.6208 4.2131 2.2460
0.7422 −0.0454 −419.7609
14.9600 −413.2688 −3.3883 1.8908 −
1.0987 2.2132 −0.6032 4.1554 2.2612
0.6895 −0.1118 −419.7795
14.9800 −416.2753 −2.1933 −1.3066 −
1.1735 2.1158 −0.5901 4.0980 2.2750
0.6367 −0.1783 −419.7982
15.0000 −416.3711 −0.7179 −2.8453 −
0.9908 1.9840 −0.5819 4.0411 2.2874
0.5840 −0.2448 −419.8169
15.0200 −415.4889 −1.2756 −1.8303 −
0.5014 1.8189 −0.5785 3.9848 2.2985
0.5313 −0.3113 −419.8355
15.0400 −410.1101 1.8315 0.1942
0.2057 1.6209 −0.5797 3.9293 2.3081
0.4785 −0.3777 −419.8542

15.0600 -410.3434 0.2558 1.3701
0.9735 1.3886 -0.5848 3.8745 2.3163
0.4258 -0.4442 -419.8728
15.0800 -411.4687 -0.2128 1.0550
1.5893 1.1210 -0.5932 3.8207 2.3231
0.3730 -0.5106 -419.8914
15.1000 -412.6771 -0.6422 0.2916
1.8718 0.8193 -0.6041 3.7678 2.3285
0.3203 -0.5770 -419.9101
15.1200 -411.8765 0.3500 0.2741
1.7421 0.4874 -0.6167 3.7161 2.3325
0.2676 -0.6434 -419.9287
15.1400 -412.8200 0.2955 0.5905
1.2387 0.1351 -0.6300 3.6654 2.3350
0.2149 -0.7098 -419.9473
15.1600 -414.7802 0.0411 0.2363
0.5159 -0.2241 -0.6433 3.6159 2.3361
0.1622 -0.7762 -419.9659
15.1800 -416.4499 0.7068 -0.4971 -
0.2550 -0.5752 -0.6556 3.5676 2.3358
0.1095 -0.8425 -419.9845
15.2000 -419.1603 -0.6759 -0.8672 -
0.9323 -0.9020 -0.6661 3.5205 2.3340
0.0569 -0.9089 -420.0031
15.2200 -419.7060 -0.3884 -0.5651 -
1.4149 -1.1891 -0.6740 3.4748 2.3308
0.0043 -0.9752 -420.0218
15.2400 -418.5194 0.5432 0.1720 -
1.6662 -1.4232 -0.6783 3.4304 2.3262
-0.0482 -1.0415 -420.0403
15.2600 -419.1756 0.6074 0.5805 -
1.6943 -1.5937 -0.6782 3.3873 2.3202
-0.1008 -1.1077 -420.0589
15.2800 -419.4884 -0.8311 0.5843 -
1.5204 -1.6946 -0.6726 3.3455 2.3127
-0.1532 -1.1740 -420.0775
15.3000 -418.7457 0.5744 0.2557 -
1.1585 -1.7265 -0.6610 3.3051 2.3038
-0.2056 -1.2402 -420.0961
15.3200 -419.4124 0.5263 -0.4743 -
0.6303 -1.6948 -0.6429 3.2661 2.2935
-0.2580 -1.3064 -420.1147
15.3400 -420.0929 -0.4260 -1.1517
0.0224 -1.6076 -0.6183 3.2284 2.2818
-0.3103 -1.3726 -420.1333
15.3600 -419.9084 -0.8862 -0.9516
0.6932 -1.4730 -0.5875 3.1920 2.2687
-0.3626 -1.4387 -420.1518
15.3800 -417.3712 -0.6392 0.3635
1.2417 -1.2993 -0.5509 3.1569 2.2541
-0.4147 -1.5048 -420.1704

15.4000 -414.0347 1.5609 1.7361
1.5180 -1.0952 -0.5088 3.1231 2.2382
-0.4668 -1.5709 -420.1889
15.4200 -415.6418 -0.0671 1.7978
1.4095 -0.8702 -0.4618 3.0904 2.2210
-0.5189 -1.6370 -420.2075
15.4400 -416.7852 -0.0245 0.6947
0.9376 -0.6336 -0.4107 3.0588 2.2023
-0.5708 -1.7030 -420.2260
15.4600 -417.9256 -0.1842 -0.3793
0.2580 -0.3921 -0.3563 3.0283 2.1824
-0.6227 -1.7690 -420.2446
15.4800 -421.1120 -1.7281 -0.6954 -
0.4215 -0.1515 -0.2998 2.9986 2.1611
-0.6745 -1.8349 -420.2631
15.5000 -419.7821 0.1971 -0.6102 -
0.9131 0.0826 -0.2422 2.9696 2.1385
-0.7261 -1.9008 -420.2816
15.5200 -416.6654 3.2519 -0.7269 -
1.1054 0.3051 -0.1848 2.9414 2.1147
-0.7777 -1.9667 -420.3002
15.5400 -422.2688 -2.3865 -0.9988 -
0.9907 0.5117 -0.1288 2.9138 2.0896
-0.8292 -2.0325 -420.3187
15.5600 -421.2569 -2.2530 -0.5287 -
0.6484 0.6989 -0.0753 2.8866 2.0633
-0.8806 -2.0983 -420.3372
15.5800 -414.2394 3.3255 0.6568 -
0.1979 0.8618 -0.0252 2.8597 2.0358
-0.9319 -2.1641 -420.3557
15.6000 -416.5859 -0.6649 1.0812
0.2417 0.9946 0.0206 2.8330 2.0071
-0.9831 -2.2298 -420.3742
15.6200 -417.6418 -0.6796 0.3511
0.5662 1.0920 0.0617 2.8063 1.9772
-1.0341 -2.2955 -420.3927
15.6400 -416.9109 0.3035 -0.4115
0.7252 1.1498 0.0975 2.7795 1.9463
-1.0851 -2.3611 -420.4112
15.6600 -418.2980 -0.2313 -0.4857
0.7228 1.1643 0.1278 2.7524 1.9142
-1.1359 -2.4267 -420.4297
15.6800 -418.8269 -0.5874 -0.0791
0.5868 1.1336 0.1527 2.7249 1.8810
-1.1866 -2.4922 -420.4481
15.7000 -417.3505 0.5925 0.3748
0.3454 1.0565 0.1720 2.6967 1.8468
-1.2372 -2.5577 -420.4666
15.7200 -418.1459 -0.1194 0.4798
0.0474 0.9349 0.1861 2.6679 1.8116
-1.2876 -2.6231 -420.4851

15.7400 -419.3605 0.0329 0.1307 -
0.2353 0.7737 0.1953 2.6381 1.7753
-1.3379 -2.6885 -420.5036
15.7600 -420.0693 0.0487 -0.3733 -
0.4304 0.5796 0.2003 2.6072 1.7381
-1.3881 -2.7539 -420.5220
15.7800 -421.4920 -0.1220 -0.3713 -
0.4924 0.3615 0.2019 2.5752 1.7000
-1.4381 -2.8192 -420.5405
15.8000 -422.3932 -1.9998 0.2500 -
0.4106 0.1295 0.2008 2.5418 1.6609
-1.4880 -2.8844 -420.5589
15.8200 -418.7222 1.9402 0.5186 -
0.2130 -0.1052 0.1980 2.5071 1.6210
-1.5377 -2.9496 -420.5774
15.8400 -419.5787 1.5645 -0.2762
0.0400 -0.3307 0.1944 2.4707 1.5802
-1.5873 -3.0147 -420.5958
15.8600 -425.3363 -2.4114 -1.1716
0.2686 -0.5366 0.1909 2.4327 1.5386
-1.6367 -3.0798 -420.6142
15.8800 -422.6621 -0.1218 -0.6906
0.3984 -0.7150 0.1884 2.3930 1.4961
-1.6859 -3.1448 -420.6326
15.9000 -420.8109 0.8832 0.6823
0.3754 -0.8604 0.1878 2.3514 1.4530
-1.7350 -3.2098 -420.6511
15.9200 -420.9918 0.5381 1.4817
0.1616 -0.9674 0.1898 2.3079 1.4090
-1.7839 -3.2747 -420.6695
15.9400 -421.9443 0.1796 1.2340 -
0.2313 -1.0298 0.1950 2.2624 1.3644
-1.8326 -3.3395 -420.6879
15.9600 -424.1091 -0.5649 0.1790 -
0.6811 -1.0437 0.2039 2.2148 1.3191
-1.8812 -3.4043 -420.7063
15.9800 -426.1033 -0.2914 -1.1127 -
1.0026 -1.0074 0.2169 2.1650 1.2731
-1.9296 -3.4690 -420.7247
16.0000 -426.7648 0.3517 -1.8312 -
1.0372 -0.9194 0.2340 2.1132 1.2265
-1.9778 -3.5337 -420.7431
16.0200 -426.2520 -0.6034 -1.3983 -
0.7316 -0.7827 0.2550 2.0591 1.1794
-2.0258 -3.5982 -420.7615
16.0400 -424.4104 -0.5218 -0.0131 -
0.1892 -0.6064 0.2792 2.0029 1.1316
-2.0736 -3.6628 -420.7798
16.0600 -421.0149 1.3192 1.2123
0.4086 -0.4025 0.3055 1.9444 1.0833
-2.1213 -3.7272 -420.7982

16.0800 -421.4119 -0.1117 1.4916
0.8612 -0.1839 0.3331 1.8838 1.0345
-2.1687 -3.7916 -420.8166
16.1000 -421.8924 -0.8888 1.3550
1.0285 0.0362 0.3607 1.8211 0.9852
-2.2160 -3.8559 -420.8349
16.1200 -421.7359 0.0602 1.3254
0.8754 0.2451 0.3873 1.7563 0.9354
-2.2630 -3.9202 -420.8533
16.1400 -421.8211 0.2819 0.9872
0.4873 0.4335 0.4119 1.6895 0.8852
-2.3098 -3.9843 -420.8717
16.1600 -422.5225 1.2666 -0.3181
0.0282 0.5932 0.4333 1.6207 0.8346
-2.3564 -4.0484 -420.8900
16.1800 -426.7667 -0.3714 -2.0400 -
0.3300 0.7176 0.4505 1.5500 0.7836
-2.4028 -4.1125 -420.9083
16.2000 -429.6184 -2.1230 -2.6500 -
0.4676 0.8014 0.4623 1.4774 0.7323
-2.4490 -4.1764 -420.9267
16.2200 -425.7071 0.4597 -1.3065 -
0.3497 0.8433 0.4678 1.4031 0.6806
-2.4950 -4.2403 -420.9450
16.2400 -423.1373 0.4684 0.9317 -
0.0497 0.8450 0.4661 1.3270 0.6286
-2.5407 -4.3041 -420.9633
16.2600 -421.9785 0.8456 2.1202
0.3236 0.8093 0.4571 1.2493 0.5764
-2.5862 -4.3678 -420.9816
16.2800 -423.3786 -0.0666 1.3412
0.6596 0.7419 0.4406 1.1700 0.5238
-2.6315 -4.4314 -421.0000
16.3000 -425.8089 -0.7904 -0.2397
0.8665 0.6512 0.4167 1.0893 0.4711
-2.6765 -4.4950 -421.0183
16.3200 -425.9663 -0.2774 -0.9806
0.8815 0.5467 0.3853 1.0071 0.4181
-2.7213 -4.5585 -421.0366
16.3400 -425.9761 -0.2967 -0.5004
0.6855 0.4369 0.3468 0.9236 0.3650
-2.7659 -4.6219 -421.0548
16.3600 -425.7490 0.3352 0.6915
0.2965 0.3286 0.3016 0.8389 0.3117
-2.8102 -4.6852 -421.0731
16.3800 -425.7771 0.1019 1.4794 -
0.2216 0.2275 0.2503 0.7530 0.2582
-2.8542 -4.7484 -421.0914
16.4000 -426.2565 1.4965 0.8301 -
0.7570 0.1386 0.1937 0.6662 0.2047
-2.8980 -4.8116 -421.1097

16.4200 -429.7714 0.0519 -0.9956 -
1.1662 0.0657 0.1327 0.5784 0.1510
-2.9416 -4.8746 -421.1280
16.4400 -434.1987 -2.3388 -2.0902 -
1.3087 0.0105 0.0684 0.4899 0.0973
-2.9849 -4.9376 -421.1462
16.4600 -430.6468 0.3232 -1.2797 -
1.1051 -0.0294 0.0021 0.4006 0.0436
-3.0279 -5.0004 -421.1645
16.4800 -427.5162 2.3718 -0.0083 -
0.5731 -0.0598 -0.0646 0.3108 -0.0102
-3.0706 -5.0632 -421.1827
16.5000 -428.9849 -0.4132 0.3115
0.1544 -0.0890 -0.1301 0.2206 -0.0640
-3.1131 -5.1259 -421.2010
16.5200 -429.8571 -1.0702 0.1831
0.8582 -0.1249 -0.1927 0.1301 -0.1177
-3.1553 -5.1885 -421.2192
16.5400 -428.8705 -0.6839 0.7344
1.3119 -0.1751 -0.2506 0.0393 -0.1714
-3.1973 -5.2510 -421.2375
16.5600 -427.6443 -0.3269 1.7192
1.4048 -0.2461 -0.3024 -0.0515 -0.2250
-3.2389 -5.3134 -421.2557
16.5800 -426.3243 2.5276 1.6953
1.1563 -0.3407 -0.3469 -0.1423 -0.2785
-3.2803 -5.3758 -421.2739
16.6000 -428.6176 2.2953 -0.2094
0.6890 -0.4566 -0.3832 -0.2330 -0.3318
-3.3214 -5.4380 -421.2921
16.6200 -437.3685 -3.9445 -2.1484
0.2061 -0.5885 -0.4104 -0.3234 -0.3851
-3.3621 -5.5001 -421.3103
16.6400 -435.7877 -1.3990 -1.6970 -
0.1103 -0.7284 -0.4278 -0.4134 -0.4381
-3.4026 -5.5621 -421.3285
16.6600 -429.8639 2.8972 0.2798 -
0.2233 -0.8651 -0.4349 -0.5031 -0.4910
-3.4428 -5.6240 -421.3467
16.6800 -430.9620 1.2212 1.2516 -
0.2003 -0.9864 -0.4313 -0.5921 -0.5436
-3.4827 -5.6859 -421.3649
16.7000 -434.2706 -0.8135 0.6864 -
0.1551 -1.0807 -0.4169 -0.6806 -0.5960
-3.5223 -5.7476 -421.3831
16.7200 -434.3065 -0.4929 0.0625 -
0.2089 -1.1369 -0.3919 -0.7682 -0.6481
-3.5616 -5.8092 -421.4013
16.7400 -433.9210 0.0623 0.2018 -
0.4309 -1.1455 -0.3566 -0.8551 -0.6999
-3.6005 -5.8707 -421.4195

16.7600 -434.0230 -0.0326 0.5483 -
0.7523 -1.0986 -0.3120 -0.9410 -0.7514
-3.6392 -5.9321 -421.4376
16.7800 -434.4682 0.9517 0.1502 -
1.0491 -0.9917 -0.2595 -1.0260 -0.8025
-3.6775 -5.9934 -421.4558
16.8000 -436.3061 0.3447 -1.0368 -
1.2079 -0.8255 -0.2004 -1.1100 -0.8533
-3.7155 -6.0546 -421.4739
16.8200 -439.1070 -1.5947 -1.6977 -
1.1418 -0.6066 -0.1368 -1.1929 -0.9037
-3.7532 -6.1156 -421.4921
16.8400 -437.1214 -1.1153 -0.7023 -
0.8451 -0.3461 -0.0705 -1.2747 -0.9537
-3.7905 -6.1766 -421.5102
16.8600 -432.2246 1.0835 1.0231 -
0.3958 -0.0599 -0.0036 -1.3554 -1.0032
-3.8275 -6.2374 -421.5284
16.8800 -431.6461 0.5165 1.7376
0.0950 0.2345 0.0620 -1.4347 -1.0523
-3.8642 -6.2982 -421.5465
16.9000 -431.8801 0.3779 0.8941
0.5299 0.5189 0.1243 -1.5128 -1.1009
-3.9006 -6.3588 -421.5646
16.9200 -432.9891 -0.0237 -0.6160
0.8635 0.7774 0.1816 -1.5895 -1.1490
-3.9365 -6.4193 -421.5827
16.9400 -435.2621 -1.7875 -1.2911
1.0875 0.9961 0.2322 -1.6648 -1.1966
-3.9722 -6.4797 -421.6008
16.9600 -433.8524 -0.7374 -0.6355
1.2057 1.1638 0.2748 -1.7386 -1.2436
-4.0075 -6.5400 -421.6189
16.9800 -429.7826 3.0490 0.2177
1.2141 1.2723 0.3082 -1.8108 -1.2900
-4.0424 -6.6001 -421.6370
17.0000 -431.7246 0.9654 0.1858
1.0940 1.3185 0.3313 -1.8813 -1.3358
-4.0770 -6.6602 -421.6551
17.0200 -436.8891 -3.5401 0.0668
0.8275 1.3055 0.3437 -1.9501 -1.3810
-4.1112 -6.7201 -421.6732
17.0400 -434.8220 -1.7949 0.8197
0.4011 1.2417 0.3448 -2.0171 -1.4256
-4.1451 -6.7799 -421.6913
17.0600 -429.9627 3.3525 1.4439 -
0.1293 1.1386 0.3346 -2.0822 -1.4695
-4.1785 -6.8396 -421.7094
17.0800 -432.9025 2.6023 0.5622 -
0.6685 1.0080 0.3134 -2.1452 -1.5127
-4.2116 -6.8991 -421.7274

17.1000 -440.8634 -3.5287 -1.0803 -
1.1024 0.8579 0.2814 -2.2062 -1.5552
-4.2444 -6.9585 -421.7455
17.1200 -440.1570 -1.2675 -1.6984 -
1.3293 0.6942 0.2393 -2.2650 -1.5969
-4.2767 -7.0178 -421.7636
17.1400 -436.8364 0.8465 -0.7476 -
1.2803 0.5224 0.1881 -2.3214 -1.6379
-4.3087 -7.0770 -421.7816
17.1600 -435.9283 0.6789 0.7276 -
0.9720 0.3455 0.1292 -2.3753 -1.6782
-4.3403 -7.1360 -421.7996
17.1800 -435.6424 0.3590 1.2865 -
0.4590 0.1645 0.0642 -2.4267 -1.7176
-4.3715 -7.1949 -421.8177
17.2000 -436.1587 0.6230 0.3834
0.1630 -0.0196 -0.0050 -2.4755 -1.7562
-4.4023 -7.2537 -421.8357
17.2200 -438.9737 -0.5371 -0.9376
0.7878 -0.2055 -0.0762 -2.5214 -1.7940
-4.4327 -7.3124 -421.8537
17.2400 -439.5498 -1.1103 -1.0873
1.2996 -0.3913 -0.1476 -2.5645 -1.8309
-4.4627 -7.3709 -421.8717
17.2600 -436.7844 0.6817 0.1477
1.5954 -0.5742 -0.2172 -2.6047 -1.8670
-4.4923 -7.4293 -421.8897
17.2800 -435.6102 1.0981 1.4172
1.6003 -0.7487 -0.2833 -2.6417 -1.9022
-4.5215 -7.4875 -421.9078
17.3000 -437.6532 -0.7839 1.3625
1.2826 -0.9074 -0.3444 -2.6756 -1.9364
-4.5503 -7.5456 -421.9257
17.3200 -439.8850 0.0151 0.1263
0.6909 -1.0399 -0.3994 -2.7061 -1.9697
-4.5787 -7.6036 -421.9437
17.3400 -441.5599 -0.4974 -1.0737 -
0.0526 -1.1356 -0.4476 -2.7334 -2.0021
-4.6066 -7.6614 -421.9617
17.3600 -442.4948 0.0555 -1.3147 -
0.8004 -1.1838 -0.4881 -2.7571 -2.0336
-4.6342 -7.7191 -421.9797
17.3800 -442.3557 0.5327 -0.5152 -
1.4214 -1.1762 -0.5205 -2.7774 -2.0641
-4.6613 -7.7767 -421.9977
17.4000 -442.1264 -0.1673 0.6520 -
1.8089 -1.1062 -0.5442 -2.7940 -2.0936
-4.6879 -7.8341 -422.0156
17.4200 -442.1000 -0.6244 1.3885 -
1.9018 -0.9711 -0.5590 -2.8070 -2.1222
-4.7142 -7.8914 -422.0336

17.4400 -442.1000 -0.2995 0.9010 -
1.6678 -0.7762 -0.5649 -2.8163 -2.1497
-4.7400 -7.9485 -422.0515
17.4600 -442.1895 1.0264 -0.8600 -
1.1291 -0.5347 -0.5623 -2.8218 -2.1763
-4.7654 -8.0055 -422.0695
17.4800 -442.9894 -0.1552 -2.3230 -
0.3803 -0.2653 -0.5517 -2.8235 -2.2018
-4.7903 -8.0623 -422.0874
17.5000 -443.4781 -1.6438 -1.8269
0.4225 0.0130 -0.5338 -2.8214 -2.2264
-4.8148 -8.1190 -422.1053
17.5200 -439.3035 -0.3033 0.5018
1.0962 0.2818 -0.5091 -2.8154 -2.2499
-4.8388 -8.1755 -422.1233
17.5400 -434.2335 1.3692 2.7928
1.4947 0.5226 -0.4786 -2.8056 -2.2724
-4.8624 -8.2319 -422.1412
17.5600 -435.0103 0.7645 3.1013
1.5319 0.7179 -0.4430 -2.7920 -2.2939
-4.8855 -8.2881 -422.1591
17.5800 -438.1458 -0.5520 1.0707
1.2437 0.8541 -0.4030 -2.7746 -2.3144
-4.9082 -8.3442 -422.1770
17.6000 -440.8240 -0.4879 -1.7685
0.7992 0.9227 -0.3599 -2.7535 -2.3338
-4.9304 -8.4002 -422.1949
17.6200 -443.2647 -0.0599 -3.3121
0.4020 0.9186 -0.3147 -2.7287 -2.3523
-4.9522 -8.4559 -422.2128
17.6400 -443.1051 -0.5187 -2.7149
0.2125 0.8411 -0.2685 -2.7004 -2.3697
-4.9735 -8.5116 -422.2307
17.6600 -440.0585 0.2916 -0.4669
0.2375 0.6974 -0.2221 -2.6685 -2.3861
-4.9943 -8.5670 -422.2485
17.6800 -438.5642 0.3415 1.6922
0.3759 0.5021 -0.1763 -2.6331 -2.4015
-5.0146 -8.6223 -422.2664
17.7000 -438.4425 -0.4129 2.5027
0.4864 0.2741 -0.1319 -2.5945 -2.4159
-5.0345 -8.6775 -422.2843
17.7200 -438.4000 0.3608 1.9820
0.4397 0.0341 -0.0894 -2.5527 -2.4293
-5.0539 -8.7325 -422.3021
17.7400 -439.9102 0.0424 0.8490
0.1808 -0.1982 -0.0497 -2.5078 -2.4416
-5.0728 -8.7873 -422.3200
17.7600 -442.2258 -0.2776 -0.2047 -
0.2447 -0.4034 -0.0133 -2.4599 -2.4530
-5.0912 -8.8420 -422.3378

17.7800 -443.3363 -0.1028 -0.8317 -
0.7303 -0.5637 0.0194 -2.4093 -2.4634
-5.1092 -8.8965 -422.3556
17.8000 -444.0872 -0.0393 -0.9113 -
1.1485 -0.6659 0.0479 -2.3561 -2.4728
-5.1267 -8.9508 -422.3735
17.8200 -444.1242 0.2562 -0.6654 -
1.3890 -0.7018 0.0720 -2.3004 -2.4812
-5.1437 -9.0050 -422.3913
17.8400 -443.6491 0.1851 -0.4873 -
1.3799 -0.6686 0.0917 -2.2425 -2.4886
-5.1602 -9.0590 -422.4091
17.8600 -442.9701 -0.4600 -0.1345 -
1.1279 -0.5700 0.1073 -2.1823 -2.4951
-5.1762 -9.1129 -422.4269
17.8800 -441.6040 0.5033 0.4705 -
0.6931 -0.4176 0.1189 -2.1202 -2.5006
-5.1917 -9.1666 -422.4447
17.9000 -440.5277 0.2263 0.7332 -
0.1642 -0.2272 0.1269 -2.0562 -2.5051
-5.2067 -9.2201 -422.4625
17.9200 -440.5622 -0.2728 0.2295
0.3732 -0.0166 0.1316 -1.9906 -2.5087
-5.2213 -9.2734 -422.4803
17.9400 -440.6815 -0.0438 -0.5607
0.8341 0.1945 0.1332 -1.9233 -2.5113
-5.2353 -9.3266 -422.4981
17.9600 -440.3886 0.0019 -0.5753
1.1477 0.3855 0.1321 -1.8547 -2.5130
-5.2489 -9.3796 -422.5158
17.9800 -439.2003 0.2043 0.1381
1.2629 0.5393 0.1285 -1.7848 -2.5138
-5.2619 -9.4324 -422.5336
18.0000 -438.8643 0.1861 0.6946
1.1520 0.6426 0.1231 -1.7138 -2.5136
-5.2745 -9.4850 -422.5514
18.0200 -439.5608 -0.1930 0.6473
0.8347 0.6871 0.1163 -1.6419 -2.5125
-5.2866 -9.5375 -422.5691
18.0400 -440.3933 -0.0862 0.2091
0.4019 0.6696 0.1091 -1.5692 -2.5105
-5.2981 -9.5898 -422.5869
18.0600 -441.3015 0.1261 -0.3845 -
0.0175 0.5909 0.1018 -1.4958 -2.5076
-5.3092 -9.6419 -422.6046
18.0800 -441.8922 -0.4568 -0.8105 -
0.3120 0.4557 0.0947 -1.4220 -2.5039
-5.3197 -9.6939 -422.6223
18.1000 -441.8551 0.0555 -0.7408 -
0.4166 0.2741 0.0884 -1.3478 -2.4992
-5.3298 -9.7456 -422.6401

18.1200 -441.7448 0.3708 -0.3606 -
0.3287 0.0627 0.0831 -1.2735 -2.4936
-5.3394 -9.7972 -422.6578
18.1400 -441.6148 0.0023 0.0953 -
0.1071 -0.1583 0.0793 -1.1992 -2.4872
-5.3484 -9.8486 -422.6755
18.1600 -441.4502 0.0839 0.5263
0.1387 -0.3665 0.0773 -1.1250 -2.4800
-5.3570 -9.8998 -422.6932
18.1800 -441.4448 0.1829 0.6191
0.2784 -0.5406 0.0775 -1.0511 -2.4719
-5.3650 -9.9508 -422.7109
18.2000 -441.9482 -0.2338 0.4487
0.2157 -0.6641 0.0803 -0.9776 -2.4630
-5.3726 -10.0017 -422.7286
18.2200 -442.2997 -0.0293 0.5540 -
0.0978 -0.7265 0.0858 -0.9047 -2.4532
-5.3796 -10.0523 -422.7462
18.2400 -442.0090 -0.4297 0.8294 -
0.6098 -0.7226 0.0942 -0.8325 -2.4426
-5.3861 -10.1028 -422.7639
18.2600 -441.6297 1.7499 0.5365 -
1.1491 -0.6546 0.1050 -0.7611 -2.4313
-5.3922 -10.1531 -422.7816
18.2800 -442.9243 0.6376 -0.6715 -
1.5058 -0.5286 0.1177 -0.6906 -2.4191
-5.3977 -10.2032 -422.7992
18.3000 -446.4550 -1.1951 -1.9467 -
1.5033 -0.3544 0.1318 -0.6211 -2.4062
-5.4027 -10.2531 -422.8169
18.3200 -446.6556 -1.9671 -1.7992 -
1.0678 -0.1469 0.1465 -0.5526 -2.3925
-5.4072 -10.3028 -422.8345
18.3400 -441.2702 0.3903 -0.1303 -
0.2975 0.0740 0.1611 -0.4853 -2.3781
-5.4112 -10.3524 -422.8522
18.3600 -437.2268 2.0853 1.1879
0.5836 0.2878 0.1749 -0.4193 -2.3630
-5.4147 -10.4017 -422.8698
18.3800 -438.6115 0.3965 0.8671
1.3292 0.4751 0.1873 -0.3545 -2.3471
-5.4177 -10.4509 -422.8874
18.4000 -440.6776 -2.1091 0.0781
1.7459 0.6176 0.1978 -0.2911 -2.3306
-5.4202 -10.4998 -422.9050
18.4200 -439.6380 -0.3768 0.3179
1.7471 0.7028 0.2056 -0.2291 -2.3133
-5.4222 -10.5486 -422.9227
18.4400 -436.9868 0.9909 0.9936
1.3707 0.7286 0.2102 -0.1686 -2.2954
-5.4237 -10.5972 -422.9403

18.4600 -437.7227 1.3272 0.4390
0.7409 0.7009 0.2113 -0.1096 -2.2769
-5.4247 -10.6455 -422.9579
18.4800 -442.9594 -1.3484 -0.9934
0.0245 0.6303 0.2083 -0.0522 -2.2576
-5.4252 -10.6937 -422.9754
18.5000 -444.5648 -1.4847 -1.5074 -
0.6120 0.5297 0.2010 0.0036 -2.2378
-5.4251 -10.7417 -422.9930
18.5200 -442.1764 0.4732 -0.5103 -
1.0453 0.4125 0.1892 0.0576 -2.2173
-5.4246 -10.7895 -423.0106
18.5400 -440.5003 0.8974 0.6688 -
1.2354 0.2899 0.1727 0.1098 -2.1963
-5.4236 -10.8371 -423.0282
18.5600 -441.4800 0.3230 0.7349 -
1.1853 0.1709 0.1516 0.1603 -2.1746
-5.4221 -10.8845 -423.0457
18.5800 -442.7907 -0.7451 -0.0290 -
0.9217 0.0622 0.1266 0.2088 -2.1524
-5.4201 -10.9317 -423.0633
18.6000 -442.6818 -0.1845 -0.6651 -
0.5138 -0.0317 0.0985 0.2553 -2.1296
-5.4176 -10.9786 -423.0808
18.6200 -441.9090 -0.1555 -0.5996 -
0.0421 -0.1088 0.0682 0.2998 -2.1063
-5.4146 -11.0254 -423.0983
18.6400 -441.0325 -0.1556 0.1149
0.4047 -0.1679 0.0369 0.3421 -2.0824
-5.4111 -11.0720 -423.1159
18.6600 -439.8518 0.3184 0.8850
0.7211 -0.2103 0.0058 0.3823 -2.0581
-5.4072 -11.1184 -423.1334
18.6800 -439.3251 1.4299 0.6657
0.8184 -0.2394 -0.0238 0.4202 -2.0332
-5.4027 -11.1646 -423.1509
18.7000 -441.2582 -0.1026 -0.5389
0.6714 -0.2612 -0.0507 0.4558 -2.0079
-5.3977 -11.2106 -423.1684
18.7200 -443.3330 -1.7188 -0.9643
0.3473 -0.2836 -0.0736 0.4891 -1.9821
-5.3923 -11.2564 -423.1859
18.7400 -441.8400 -0.5032 0.2835 -
0.0330 -0.3153 -0.0913 0.5200 -1.9558
-5.3864 -11.3020 -423.2034
18.7600 -438.7298 1.5828 1.6249 -
0.3159 -0.3643 -0.1028 0.5486 -1.9291
-5.3800 -11.3474 -423.2209
18.7800 -439.3228 1.4387 1.0934 -
0.3801 -0.4351 -0.1070 0.5748 -1.9020
-5.3731 -11.3925 -423.2384

18.8000 -444.0507 -1.0273 -0.9736 -
0.2175 -0.5284 -0.1034 0.5986 -1.8745
-5.3657 -11.4375 -423.2558
18.8200 -445.9040 -0.9836 -2.4761
0.0936 -0.6405 -0.0920 0.6199 -1.8465
-5.3579 -11.4823 -423.2733
18.8400 -444.1071 0.3958 -1.9794
0.4487 -0.7627 -0.0729 0.6389 -1.8182
-5.3496 -11.5268 -423.2907
18.8600 -441.5278 -0.5673 0.2425
0.7305 -0.8819 -0.0468 0.6555 -1.7896
-5.3408 -11.5712 -423.3082
18.8800 -438.6994 -0.3114 2.8065
0.8274 -0.9842 -0.0143 0.6696 -1.7606
-5.3316 -11.6153 -423.3256
18.9000 -436.1059 1.5627 3.8923
0.6497 -1.0575 0.0236 0.6815 -1.7312
-5.3218 -11.6593 -423.3430
18.9200 -437.9999 1.7706 2.1412
0.1936 -1.0925 0.0662 0.6909 -1.7016
-5.3117 -11.7030 -423.3605
18.9400 -445.5720 -1.2627 -1.4726 -
0.4062 -1.0807 0.1123 0.6981 -1.6716
-5.3010 -11.7465 -423.3779
18.9600 -447.7863 -0.6744 -3.9445 -
0.9586 -1.0149 0.1606 0.7030 -1.6414
-5.2899 -11.7899 -423.3953
18.9800 -446.5924 -0.1242 -3.5702 -
1.2940 -0.8896 0.2097 0.7057 -1.6110
-5.2784 -11.8330 -423.4127
19.0000 -444.7138 -0.4853 -0.9465 -
1.3521 -0.7031 0.2581 0.7062 -1.5802
-5.2663 -11.8759 -423.4301
19.0200 -441.0572 -0.1652 2.1422 -
1.1727 -0.4590 0.3044 0.7046 -1.5493
-5.2539 -11.9186 -423.4475
19.0400 -436.9135 1.4063 3.6809 -
0.8260 -0.1678 0.3472 0.7008 -1.5182
-5.2410 -11.9610 -423.4648
19.0600 -437.2890 0.7771 2.7424 -
0.3796 0.1545 0.3852 0.6951 -1.4869
-5.2276 -12.0033 -423.4822
19.0800 -441.1905 -1.1486 0.1609
0.0834 0.4885 0.4173 0.6874 -1.4554
-5.2138 -12.0454 -423.4996
19.1000 -442.4683 -0.8984 -2.2044
0.5148 0.8133 0.4424 0.6777 -1.4238
-5.1995 -12.0872 -423.5169
19.1200 -442.0541 0.3388 -2.8518
0.8903 1.1084 0.4594 0.6662 -1.3920
-5.1848 -12.1289 -423.5343

19.1400	-441.2573	-1.6631	-1.3500	
1.1891	1.3552	0.4672	0.6530	-1.3602
	-5.1697	-12.1703	-423.5516	
19.1600	-436.9900	0.0929	1.1015	
1.3711	1.5386	0.4650	0.6381	-1.3282
	-5.1542	-12.2115	-423.5689	
19.1800	-432.2746	3.0646	2.5290	
1.3854	1.6464	0.4519	0.6216	-1.2961
	-5.1382	-12.2525	-423.5863	
19.2000	-435.2795	0.9867	1.7448	
1.1957	1.6717	0.4276	0.6036	-1.2640
	-5.1217	-12.2933	-423.6036	
19.2200	-442.0305	-3.0634	-0.0830	
0.8237	1.6149	0.3924	0.5842	-1.2319
	-5.1049	-12.3339	-423.6209	
19.2400	-441.7135	-1.4914	-0.9370	
0.3468	1.4816	0.3472	0.5637	-1.1997
	-5.0877	-12.3742	-423.6382	
19.2600	-439.3437	1.7161	-0.3466	-
0.1306	1.2811	0.2933	0.5420	-1.1675
	-5.0700	-12.4144	-423.6555	
19.2800	-439.2417	1.7444	0.1221	-
0.4939	1.0253	0.2322	0.5195	-1.1353
	-5.0519	-12.4543	-423.6728	
19.3000	-443.0356	-0.7434	-0.5682	-
0.6588	0.7278	0.1655	0.4961	-1.1032
	-5.0334	-12.4941	-423.6900	
19.3200	-445.0503	-1.7583	-1.1836	-
0.6248	0.4043	0.0946	0.4722	-1.0711
	-5.0145	-12.5336	-423.7073	
19.3400	-442.7957	-0.3138	-0.4809	-
0.4373	0.0695	0.0213	0.4477	-1.0391
	-4.9952	-12.5729	-423.7246	
19.3600	-440.3346	1.0973	0.6946	-
0.1914	-0.2633	-0.0527	0.4230	-1.0071
	-4.9755	-12.6119	-423.7418	
19.3800	-441.1159	1.0622	0.9973	
0.0066	-0.5816	-0.1258	0.3981	-0.9753
	-4.9554	-12.6508	-423.7591	
19.4000	-443.2806	-1.0339	0.5309	
0.1186	-0.8731	-0.1963	0.3731	-0.9436
	-4.9350	-12.6895	-423.7763	
19.4200	-443.7257	-0.7196	0.1387	
0.1570	-1.1249	-0.2627	0.3483	-0.9120
	-4.9141	-12.7279	-423.7935	
19.4400	-443.2692	-0.4040	0.0891	
0.1389	-1.3255	-0.3234	0.3237	-0.8805
	-4.8929	-12.7661	-423.8107	
19.4600	-443.0112	0.7671	-0.0628	
0.0953	-1.4648	-0.3771	0.2996	-0.8492
	-4.8712	-12.8041	-423.8280	

19.4800 -444.4867 0.3443 -0.6054
0.0285 -1.5342 -0.4228 0.2759 -0.8181
-4.8493 -12.8419 -423.8452
19.5000 -446.3290 -1.3047 -1.0050 -
0.0972 -1.5266 -0.4597 0.2529 -0.7872
-4.8269 -12.8795 -423.8624
19.5200 -445.5521 -0.2145 -0.3306 -
0.3224 -1.4383 -0.4870 0.2307 -0.7564
-4.8042 -12.9168 -423.8795
19.5400 -442.6513 0.4562 1.0406 -
0.6561 -1.2689 -0.5044 0.2094 -0.7259
-4.7811 -12.9539 -423.8967
19.5600 -441.6430 0.7684 1.5374 -
1.0918 -1.0257 -0.5121 0.1891 -0.6956
-4.7577 -12.9908 -423.9139
19.5800 -444.1493 -0.3753 0.6037 -
1.5534 -0.7238 -0.5102 0.1699 -0.6655
-4.7339 -13.0275 -423.9311
19.6000 -446.4460 -0.4692 -0.9007 -
1.8969 -0.3823 -0.4992 0.1519 -0.6356
-4.7098 -13.0640 -423.9482
19.6200 -446.2542 -0.0907 -1.7725 -
1.9665 -0.0228 -0.4794 0.1351 -0.6060
-4.6853 -13.1003 -423.9654
19.6400 -445.4948 0.2588 -1.6514 -
1.6515 0.3320 -0.4516 0.1196 -0.5767
-4.6605 -13.1363 -423.9825
19.6600 -444.1977 -1.1872 -0.7361 -
0.9293 0.6595 -0.4160 0.1053 -0.5476
-4.6353 -13.1721 -423.9996
19.6800 -441.0870 0.8945 0.3659
0.1001 0.9408 -0.3736 0.0923 -0.5188
-4.6099 -13.2077 -424.0168
19.7000 -438.9054 0.4925 0.9464
1.2593 1.1600 -0.3252 0.0805 -0.4903
-4.5841 -13.2431 -424.0339
19.7200 -438.6068 -0.5078 0.6887
2.3146 1.3029 -0.2719 0.0700 -0.4621
-4.5580 -13.2783 -424.0510
19.7400 -438.3978 -0.2062 0.3979
3.0073 1.3562 -0.2150 0.0607 -0.4342
-4.5316 -13.3132 -424.0681
19.7600 -437.6882 -0.4717 0.8436
3.1424 1.3100 -0.1557 0.0525 -0.4065
-4.5049 -13.3479 -424.0852
19.7800 -436.5964 0.4923 1.7948
2.6608 1.1667 -0.0952 0.0454 -0.3792
-4.4779 -13.3824 -424.1022
19.8000 -437.0046 0.3094 2.1880
1.6409 0.9456 -0.0348 0.0393 -0.3521
-4.4505 -13.4167 -424.1193

19.8200 -440.1684 0.0867 1.2339
0.2717 0.6733 0.0241 0.0342 -0.3254
-4.4229 -13.4508 -424.1364
19.8400 -443.2402 0.1656 -0.5929 -
1.1721 0.3773 0.0802 0.0301 -0.2989
-4.3951 -13.4846 -424.1534
19.8600 -446.3124 -0.6404 -1.9641 -
2.3933 0.0851 0.1322 0.0268 -0.2728
-4.3669 -13.5182 -424.1705
19.8800 -447.9957 -0.0260 -2.3059 -
3.1383 -0.1759 0.1790 0.0244 -0.2470
-4.3385 -13.5516 -424.1875
19.9000 -447.0241 -0.2309 -1.6852 -
3.2725 -0.3805 0.2193 0.0227 -0.2215
-4.3098 -13.5848 -424.2046
19.9200 -445.0417 0.4373 -0.4382 -
2.8311 -0.5141 0.2526 0.0216 -0.1964
-4.2808 -13.6177 -424.2216
19.9400 -443.6477 0.4166 0.6045 -
1.9692 -0.5755 0.2784 0.0212 -0.1715
-4.2516 -13.6504 -424.2386
19.9600 -442.6243 -0.2994 0.9813 -
0.8945 -0.5711 0.2967 0.0214 -0.1470
-4.2221 -13.6830 -424.2556
19.9800 -441.6610 -0.2957 1.1807
0.1696 -0.5093 0.3075 0.0221 -0.1228
-4.1924 -13.7152 -424.2726
20.0000 -440.4670 -0.1340 1.3954
1.0409 -0.3999 0.3112 0.0231 -0.0990
-4.1625 -13.7473 -424.2896
20.0200 -439.5654 0.8936 0.9862
1.6138 -0.2554 0.3084 0.0246 -0.0755
-4.1323 -13.7791 -424.3066
20.0400 -440.0790 0.2223 -0.2378
1.8715 -0.0907 0.2998 0.0265 -0.0523
-4.1019 -13.8108 -424.3236
20.0600 -441.6635 -1.1414 -1.2100
1.8662 0.0771 0.2861 0.0285 -0.0294
-4.0712 -13.8422 -424.3405
20.0800 -441.7251 -0.7146 -0.7672
1.6701 0.2294 0.2682 0.0309 -0.0068
-4.0404 -13.8733 -424.3575
20.1000 -439.0359 0.5887 0.5303
1.3591 0.3486 0.2469 0.0334 0.0154
-4.0094 -13.9043 -424.3744
20.1200 -437.5478 2.1589 0.9549
0.9938 0.4199 0.2230 0.0360 0.0374
-3.9781 -13.9350 -424.3914
20.1400 -440.6839 0.0123 -0.0118
0.6279 0.4329 0.1975 0.0388 0.0590
-3.9467 -13.9655 -424.4083

20.1600 -443.9984 -1.5294 -1.0744
0.3365 0.3829 0.1712 0.0416 0.0803
-3.9150 -13.9958 -424.4252
20.1800 -443.0924 -0.3629 -0.9326
0.1696 0.2754 0.1450 0.0444 0.1014
-3.8832 -14.0259 -424.4422
20.2000 -441.0326 0.1170 0.2160
0.1360 0.1234 0.1198 0.0472 0.1221
-3.8512 -14.0557 -424.4591
20.2200 -440.7662 0.7166 0.9276
0.1771 -0.0564 0.0963 0.0499 0.1426
-3.8191 -14.0853 -424.4760
20.2400 -441.7326 -0.1892 0.6661
0.1903 -0.2460 0.0753 0.0525 0.1627
-3.7868 -14.1147 -424.4929
20.2600 -442.5809 -0.2457 0.0558
0.1032 -0.4274 0.0574 0.0550 0.1827
-3.7543 -14.1439 -424.5097
20.2800 -443.1064 -0.1255 -0.2384 -
0.1392 -0.5836 0.0433 0.0573 0.2023
-3.7217 -14.1728 -424.5266
20.3000 -443.4611 0.1783 -0.1236 -
0.5274 -0.6996 0.0336 0.0594 0.2217
-3.6889 -14.2016 -424.5435
20.3200 -443.5727 -0.2303 0.3875 -
0.9704 -0.7643 0.0283 0.0612 0.2408
-3.6560 -14.2301 -424.5603
20.3400 -443.6305 0.2206 0.7158 -
1.3449 -0.7701 0.0276 0.0629 0.2597
-3.6230 -14.2583 -424.5772
20.3600 -443.9247 0.8584 0.1163 -
1.5294 -0.7146 0.0308 0.0643 0.2784
-3.5899 -14.2864 -424.5940
20.3800 -445.4209 -0.2858 -1.0950 -
1.4307 -0.6005 0.0374 0.0655 0.2968
-3.5566 -14.3142 -424.6109
20.4000 -446.4964 -0.9461 -1.6062 -
1.0160 -0.4385 0.0466 0.0666 0.3150
-3.5233 -14.3419 -424.6277
20.4200 -444.0383 -0.7246 -0.7664 -
0.3434 -0.2461 0.0576 0.0674 0.3330
-3.4898 -14.3692 -424.6445
20.4400 -439.7874 1.0402 0.6640
0.4445 -0.0423 0.0695 0.0680 0.3508
-3.4562 -14.3964 -424.6613
20.4600 -438.6188 0.0851 1.5326
1.1549 0.1546 0.0814 0.0684 0.3684
-3.4226 -14.4234 -424.6781
20.4800 -438.3668 0.3564 1.5133
1.6098 0.3271 0.0927 0.0687 0.3858
-3.3889 -14.4501 -424.6949

20.5000 -438.3549 0.2998 0.9709
1.7110 0.4602 0.1023 0.0689 0.4030
-3.3551 -14.4766 -424.7117
20.5200 -439.7034 -0.1001 0.3095
1.4591 0.5455 0.1095 0.0690 0.4201
-3.3212 -14.5029 -424.7284
20.5400 -441.1740 -0.6921 -0.2183
0.9165 0.5851 0.1137 0.0690 0.4370
-3.2873 -14.5289 -424.7452
20.5600 -441.7365 0.2160 -0.3255
0.2126 0.5851 0.1144 0.0690 0.4537
-3.2533 -14.5547 -424.7619
20.5800 -442.1478 -0.2026 -0.1525 -
0.4895 0.5528 0.1116 0.0691 0.4702
-3.2192 -14.5804 -424.7787
20.6000 -442.6327 0.1751 -0.1049 -
1.0506 0.4967 0.1052 0.0692 0.4866
-3.1852 -14.6057 -424.7954
20.6200 -443.1440 -0.0371 -0.3584 -
1.3826 0.4258 0.0955 0.0695 0.5029
-3.1511 -14.6309 -424.8122
20.6400 -443.2987 0.2257 -0.6360 -
1.4360 0.3485 0.0823 0.0699 0.5190
-3.1169 -14.6558 -424.8289
20.6600 -443.2714 0.3040 -0.7387 -
1.2094 0.2712 0.0659 0.0705 0.5349
-3.0827 -14.6806 -424.8456
20.6800 -443.2107 -0.5730 -0.4706 -
0.7605 0.1982 0.0463 0.0714 0.5508
-3.0486 -14.7051 -424.8623
20.7000 -442.0784 -0.1384 0.1477 -
0.2117 0.1310 0.0239 0.0725 0.5665
-3.0144 -14.7293 -424.8790
20.7200 -439.9649 0.4674 0.8217
0.3027 0.0680 -0.0010 0.0738 0.5821
-2.9802 -14.7534 -424.8957
20.7400 -439.6965 0.7097 0.9227
0.6866 0.0069 -0.0280 0.0754 0.5976
-2.9460 -14.7772 -424.9123
20.7600 -440.8352 -0.5282 0.4150
0.8994 -0.0530 -0.0563 0.0773 0.6130
-2.9118 -14.8008 -424.9290
20.7800 -441.5375 0.0756 -0.1589
0.9715 -0.1121 -0.0854 0.0793 0.6283
-2.8776 -14.8242 -424.9456
20.8000 -441.6738 -0.3894 -0.5273
0.9591 -0.1707 -0.1146 0.0816 0.6434
-2.8434 -14.8474 -424.9623
20.8200 -441.7778 0.4020 -0.6522
0.8832 -0.2289 -0.1433 0.0840 0.6585
-2.8093 -14.8703 -424.9789

20.8400 -441.9666 0.4871 -0.5146
0.7467 -0.2858 -0.1708 0.0866 0.6735
-2.7752 -14.8930 -424.9956
20.8600 -442.1633 -0.2438 -0.0667
0.5113 -0.3391 -0.1966 0.0893 0.6884
-2.7412 -14.9155 -425.0122
20.8800 -442.1466 -0.4043 0.6062
0.1402 -0.3842 -0.2201 0.0920 0.7032
-2.7072 -14.9378 -425.0288
20.9000 -441.8581 -0.1224 1.0089 -
0.3552 -0.4156 -0.2406 0.0947 0.7179
-2.6732 -14.9598 -425.0454
20.9200 -441.7296 1.0303 0.6494 -
0.9001 -0.4288 -0.2575 0.0973 0.7326
-2.6393 -14.9817 -425.0620
20.9400 -443.7676 0.4660 -0.3221 -
1.3689 -0.4215 -0.2705 0.0999 0.7471
-2.6055 -15.0033 -425.0786
20.9600 -446.1328 -1.1757 -0.9942 -
1.6323 -0.3938 -0.2791 0.1023 0.7616
-2.5717 -15.0247 -425.0952
20.9800 -445.1724 -0.7405 -0.6583 -
1.5883 -0.3478 -0.2832 0.1045 0.7760
-2.5380 -15.0458 -425.1117
21.0000 -442.0721 0.7356 0.2821 -
1.2117 -0.2867 -0.2827 0.1065 0.7904
-2.5044 -15.0668 -425.1283
21.0200 -440.9524 1.0567 0.5867 -
0.5525 -0.2150 -0.2778 0.1083 0.8047
-2.4708 -15.0875 -425.1448
21.0400 -441.7729 -0.2853 -0.1416
0.2900 -0.1365 -0.2686 0.1097 0.8189
-2.4374 -15.1080 -425.1614
21.0600 -442.4895 -0.8258 -0.8282
1.1413 -0.0555 -0.2555 0.1109 0.8330
-2.4040 -15.1283 -425.1779
21.0800 -440.9909 -0.4779 -0.3975
1.8034 0.0235 -0.2386 0.1117 0.8471
-2.3708 -15.1483 -425.1944
21.1000 -438.2673 0.7110 0.8759
2.1182 0.0967 -0.2184 0.1122 0.8611
-2.3377 -15.1681 -425.2109
21.1200 -437.9492 0.4222 1.9127
1.9791 0.1619 -0.1953 0.1122 0.8750
-2.3046 -15.1877 -425.2274
21.1400 -438.8150 -0.3832 1.8815
1.3709 0.2189 -0.1698 0.1119 0.8889
-2.2717 -15.2071 -425.2439
21.1600 -440.1810 0.5006 0.7058
0.4050 0.2685 -0.1423 0.1112 0.9027
-2.2390 -15.2263 -425.2604

21.1800	-443.3563	-0.5877	-0.9637	-
0.6960	0.3132	-0.1134	0.1101	0.9165
	-2.2063	-15.2452	-425.2769	
21.2000	-445.5869	-0.6962	-2.0801	-
1.6677	0.3559	-0.0835	0.1086	0.9302
	-2.1738	-15.2640	-425.2934	
21.2200	-445.6618	0.7817	-2.4565	-
2.2766	0.3998	-0.0531	0.1067	0.9439
	-2.1414	-15.2825	-425.3098	
21.2400	-445.6956	0.1593	-2.5026	-
2.3694	0.4478	-0.0228	0.1042	0.9575
	-2.1092	-15.3007	-425.3263	
21.2600	-445.3213	-0.2831	-2.2122	-
1.9287	0.4992	0.0072	0.1013	0.9710
	-2.0771	-15.3188	-425.3427	
21.2800	-443.0148	0.2183	-1.4607	-
1.0733	0.5476	0.0369	0.0978	0.9845
	-2.0452	-15.3366	-425.3591	
21.3000	-440.5656	0.0474	-0.4588	
0.0067	0.5841	0.0663	0.0937	0.9980
	-2.0134	-15.3543	-425.3756	
21.3200	-438.9511	0.1105	0.5888	
1.0829	0.5996	0.0954	0.0890	1.0114
	-1.9818	-15.3717	-425.3920	
21.3400	-437.4241	-0.4571	1.9219	
1.9239	0.5854	0.1242	0.0836	1.0247
	-1.9504	-15.3888	-425.4084	
21.3600	-435.3965	-0.6232	3.4788	
2.3597	0.5338	0.1527	0.0776	1.0380
	-1.9191	-15.4058	-425.4248	
21.3800	-433.2049	1.0359	4.0477	
2.3354	0.4404	0.1806	0.0709	1.0512
	-1.8880	-15.4225	-425.4412	
21.4000	-434.4614	2.4501	2.3074	
1.9231	0.3070	0.2079	0.0634	1.0644
	-1.8571	-15.4390	-425.4575	
21.4200	-442.7384	-1.8321	-1.1915	
1.3033	0.1391	0.2346	0.0553	1.0776
	-1.8264	-15.4553	-425.4739	
21.4400	-446.0508	-1.1201	-3.9293	
0.6797	-0.0567	0.2606	0.0465	1.0907
	-1.7958	-15.4714	-425.4903	
21.4600	-445.2076	0.3908	-4.0391	
0.2263	-0.2733	0.2856	0.0370	1.1037
	-1.7655	-15.4872	-425.5066	
21.4800	-443.8565	-0.0878	-1.8105	-
0.0038	-0.5012	0.3097	0.0268	1.1167
	-1.7353	-15.5029	-425.5229	
21.5000	-441.7365	0.0025	0.9891	-
0.0851	-0.7237	0.3326	0.0158	1.1297
	-1.7053	-15.5183	-425.5393	

21.5200 -439.2899 0.2811 2.8384 -
0.1511 -0.9192 0.3542 0.0041 1.1427
-1.6756 -15.5335 -425.5556
21.5400 -439.1493 0.7938 3.1116 -
0.3484 -1.0658 0.3742 -0.0083 1.1556
-1.6460 -15.5484 -425.5719
21.5600 -441.3592 -0.8105 1.9590 -
0.7283 -1.1445 0.3925 -0.0214 1.1685
-1.6167 -15.5632 -425.5882
21.5800 -443.4750 0.6027 0.1938 -
1.2382 -1.1446 0.4088 -0.0353 1.1813
-1.5876 -15.5777 -425.6045
21.6000 -445.0626 -0.3534 -1.4018 -
1.7431 -1.0617 0.4230 -0.0498 1.1941
-1.5586 -15.5920 -425.6208
21.6200 -446.1254 -0.2784 -2.0824 -
2.0886 -0.8961 0.4349 -0.0650 1.2069
-1.5300 -15.6061 -425.6370
21.6400 -445.7644 -0.0539 -1.6028 -
2.1356 -0.6529 0.4439 -0.0808 1.2197
-1.5015 -15.6200 -425.6533
21.6600 -444.2071 0.3199 -0.6584 -
1.8180 -0.3443 0.4499 -0.0972 1.2325
-1.4732 -15.6336 -425.6696
21.6800 -442.5848 -0.2205 0.0619 -
1.1744 0.0101 0.4523 -0.1141 1.2452
-1.4452 -15.6470 -425.6858
21.7000 -440.8883 0.0833 0.3130 -
0.3123 0.3866 0.4508 -0.1315 1.2579
-1.4174 -15.6603 -425.7020
21.7200 -439.4068 -0.1906 0.3979
0.6174 0.7592 0.4451 -0.1494 1.2706
-1.3899 -15.6732 -425.7183
21.7400 -438.5944 -0.0061 0.4560
1.4436 1.1015 0.4346 -0.1677 1.2833
-1.3625 -15.6860 -425.7345
21.7600 -438.0109 -0.7239 0.5042
2.0090 1.3869 0.4190 -0.1862 1.2959
-1.3355 -15.6986 -425.7507
21.7800 -437.3167 -0.0369 0.7138
2.1998 1.5916 0.3979 -0.2050 1.3085
-1.3086 -15.7109 -425.7669
21.8000 -436.4795 0.5382 0.8083
1.9813 1.6995 0.3712 -0.2239 1.3211
-1.2820 -15.7230 -425.7831
21.8200 -436.4112 1.0441 0.3142
1.4268 1.7073 0.3389 -0.2428 1.3337
-1.2557 -15.7349 -425.7992
21.8400 -439.6753 -0.2542 -0.6094
0.7067 1.6201 0.3016 -0.2618 1.3463
-1.2295 -15.7466 -425.8154

21.8600 -441.9985 -1.0814 -1.0437
0.0006 1.4469 0.2599 -0.2806 1.3588
-1.2037 -15.7581 -425.8316
21.8800 -441.3031 0.3020 -0.5017 -
0.5462 1.1987 0.2144 -0.2992 1.3713
-1.1781 -15.7693 -425.8477
21.9000 -440.3880 0.3588 0.3874 -
0.8703 0.8882 0.1662 -0.3175 1.3838
-1.1527 -15.7803 -425.8639
21.9200 -440.8654 0.9308 0.5388 -
0.9808 0.5321 0.1159 -0.3355 1.3962
-1.1276 -15.7912 -425.8800
21.9400 -442.5549 -0.8393 0.0196 -
0.9208 0.1495 0.0644 -0.3529 1.4086
-1.1027 -15.8017 -425.8961
21.9600 -443.0521 -0.5629 -0.2712 -
0.7543 -0.2385 0.0127 -0.3697 1.4209
-1.0781 -15.8121 -425.9122
21.9800 -442.5811 0.0884 -0.0203 -
0.5540 -0.6106 -0.0384 -0.3859 1.4332
-1.0538 -15.8223 -425.9283
22.0000 -442.2037 0.3297 0.3665 -
0.3972 -0.9475 -0.0878 -0.4012 1.4454
-1.0297 -15.8322 -425.9444
22.0200 -442.7873 0.1841 0.4897 -
0.3285 -1.2331 -0.1346 -0.4158 1.4575
-1.0059 -15.8419 -425.9605
22.0400 -443.9862 -0.2805 0.3156 -
0.3468 -1.4574 -0.1779 -0.4294 1.4696
-0.9823 -15.8515 -425.9766
22.0600 -444.6888 -0.1936 -0.1112 -
0.3848 -1.6144 -0.2170 -0.4419 1.4816
-0.9590 -15.8608 -425.9926
22.0800 -445.1893 0.1381 -0.8413 -
0.3440 -1.7002 -0.2515 -0.4534 1.4935
-0.9360 -15.8698 -426.0087
22.1000 -445.3997 0.0429 -1.6478 -
0.1581 -1.7144 -0.2811 -0.4637 1.5053
-0.9132 -15.8787 -426.0247
22.1200 -444.9869 0.0397 -1.7107
0.1517 -1.6601 -0.3057 -0.4727 1.5170
-0.8907 -15.8874 -426.0408
22.1400 -444.0450 -0.2975 -0.5788
0.4764 -1.5427 -0.3253 -0.4804 1.5286
-0.8685 -15.8958 -426.0568
22.1600 -442.5669 -0.4710 1.2438
0.6974 -1.3700 -0.3401 -0.4867 1.5401
-0.8465 -15.9040 -426.0728
22.1800 -439.7666 0.1405 2.8218
0.7161 -1.1510 -0.3504 -0.4916 1.5515
-0.8248 -15.9120 -426.0888

22.2000 -438.3144 1.3514 2.9974
0.4811 -0.8948 -0.3568 -0.4950 1.5627
-0.8033 -15.9198 -426.1048
22.2200 -441.6409 -0.4624 1.3105
0.0441 -0.6101 -0.3598 -0.4969 1.5738
-0.7821 -15.9274 -426.1208
22.2400 -445.5966 -1.0538 -1.3608 -
0.4365 -0.3050 -0.3601 -0.4971 1.5847
-0.7612 -15.9348 -426.1367
22.2600 -445.7168 0.6561 -3.3134 -
0.7887 0.0128 -0.3587 -0.4958 1.5955
-0.7406 -15.9419 -426.1527
22.2800 -445.3879 0.6740 -3.3905 -
0.8891 0.3343 -0.3564 -0.4927 1.6061
-0.7202 -15.9489 -426.1687
22.3000 -444.6296 -1.6915 -1.4788 -
0.7169 0.6477 -0.3540 -0.4880 1.6165
-0.7001 -15.9556 -426.1846
22.3200 -440.1582 -0.2283 1.4774 -
0.3608 0.9422 -0.3523 -0.4816 1.6267
-0.6802 -15.9622 -426.2005
22.3400 -435.8701 1.8615 3.4966
0.0528 1.2078 -0.3520 -0.4733 1.6367
-0.6606 -15.9685 -426.2165
22.3600 -436.6573 0.0482 3.0937
0.4076 1.4340 -0.3536 -0.4633 1.6465
-0.6413 -15.9746 -426.2324
22.3800 -439.4586 -0.9920 0.9260
0.6398 1.6111 -0.3578 -0.4514 1.6560
-0.6223 -15.9805 -426.2483
22.4000 -440.8811 0.0899 -1.3632
0.7493 1.7315 -0.3651 -0.4377 1.6653
-0.6035 -15.9862 -426.2642
22.4200 -441.7394 0.4884 -2.4768
0.7609 1.7886 -0.3758 -0.4222 1.6744
-0.5850 -15.9916 -426.2801
22.4400 -441.9111 -0.7012 -2.0027
0.7068 1.7781 -0.3899 -0.4049 1.6831
-0.5667 -15.9969 -426.2959
22.4600 -439.8929 -0.2652 -0.2927
0.6035 1.6999 -0.4073 -0.3857 1.6916
-0.5487 -16.0020 -426.3118
22.4800 -437.9559 0.6476 1.4827
0.4418 1.5586 -0.4277 -0.3649 1.6998
-0.5310 -16.0069 -426.3277
22.5000 -438.4833 0.0842 2.0785
0.2210 1.3631 -0.4507 -0.3424 1.7076
-0.5136 -16.0115 -426.3435
22.5200 -440.0528 -0.8312 1.0876 -
0.0471 1.1254 -0.4756 -0.3183 1.7152
-0.4964 -16.0160 -426.3593

22.5400 -441.8331 0.6655 -0.7675 -
0.3069 0.8580 -0.5017 -0.2928 1.7224
-0.4794 -16.0202 -426.3752
22.5600 -444.1529 -0.0171 -2.0814 -
0.4900 0.5717 -0.5284 -0.2659 1.7292
-0.4627 -16.0242 -426.3910
22.5800 -445.0304 -1.0530 -1.6360 -
0.5576 0.2781 -0.5547 -0.2377 1.7356
-0.4463 -16.0281 -426.4068
22.6000 -442.5123 0.0318 0.3168 -
0.5237 -0.0115 -0.5800 -0.2083 1.7417
-0.4301 -16.0317 -426.4226
22.6200 -439.7100 0.9595 1.7948 -
0.4411 -0.2882 -0.6033 -0.1779 1.7474
-0.4142 -16.0351 -426.4384
22.6400 -440.7767 0.8002 1.3586 -
0.3420 -0.5482 -0.6234 -0.1465 1.7527
-0.3986 -16.0384 -426.4541
22.6600 -444.3028 -0.7790 -0.2853 -
0.2279 -0.7894 -0.6392 -0.1142 1.7575
-0.3832 -16.0414 -426.4699
22.6800 -445.3340 -1.2489 -1.3433 -
0.0761 -1.0103 -0.6498 -0.0812 1.7619
-0.3680 -16.0442 -426.4857
22.7000 -443.4156 -0.0936 -0.9844
0.1346 -1.2091 -0.6538 -0.0475 1.7659
-0.3531 -16.0469 -426.5014
22.7200 -441.7411 0.9575 0.0517
0.3819 -1.3815 -0.6506 -0.0133 1.7693
-0.3385 -16.0493 -426.5171
22.7400 -442.1463 0.0964 0.5855
0.5994 -1.5222 -0.6395 0.0213 1.7723
-0.3241 -16.0515 -426.5329
22.7600 -443.0638 -0.5308 0.2758
0.7085 -1.6245 -0.6201 0.0562 1.7748
-0.3099 -16.0535 -426.5486
22.7800 -443.1948 -0.0326 -0.1616
0.6454 -1.6813 -0.5920 0.0912 1.7768
-0.2960 -16.0554 -426.5643
22.8000 -442.4359 0.1760 0.0024
0.3818 -1.6841 -0.5551 0.1264 1.7782
-0.2823 -16.0570 -426.5800
22.8200 -441.9005 0.3666 0.4253 -
0.0517 -1.6251 -0.5097 0.1614 1.7791
-0.2689 -16.0584 -426.5956
22.8400 -442.6390 -0.4348 0.4794 -
0.5694 -1.4993 -0.4559 0.1963 1.7795
-0.2557 -16.0597 -426.6113
22.8600 -443.8018 0.2269 0.1856 -
1.0646 -1.3038 -0.3943 0.2308 1.7793
-0.2428 -16.0607 -426.6270

22.8800 -443.9240 -0.2860 -0.1628 -
1.4182 -1.0388 -0.3254 0.2650 1.7784
-0.2300 -16.0616 -426.6426
22.9000 -443.4783 -0.3992 -0.3394 -
1.5266 -0.7104 -0.2500 0.2986 1.7770
-0.2176 -16.0623 -426.6583
22.9200 -442.8495 0.6570 -0.3046 -
1.3445 -0.3312 -0.1689 0.3315 1.7750
-0.2053 -16.0627 -426.6739
22.9400 -441.9307 -0.2946 -0.2525 -
0.8887 0.0809 -0.0830 0.3636 1.7724
-0.1933 -16.0630 -426.6895
22.9600 -440.6835 0.0947 -0.0222 -
0.2528 0.5044 0.0069 0.3948 1.7691
-0.1815 -16.0631 -426.7051
22.9800 -438.9815 -0.7552 0.4937
0.4199 0.9156 0.0998 0.4250 1.7651
-0.1699 -16.0630 -426.7207
23.0000 -436.7742 0.8394 0.8228
0.9976 1.2890 0.1950 0.4541 1.7605
-0.1585 -16.0627 -426.7363
23.0200 -436.2234 0.5293 0.5030
1.3882 1.6003 0.2914 0.4820 1.7553
-0.1474 -16.0622 -426.7519
23.0400 -437.6292 0.0523 -0.3780
1.5574 1.8256 0.3883 0.5086 1.7493
-0.1364 -16.0615 -426.7675
23.0600 -438.5972 -0.8777 -0.7913
1.5336 1.9445 0.4843 0.5338 1.7426
-0.1257 -16.0607 -426.7830
23.0800 -436.9568 -0.2886 -0.0607
1.3697 1.9444 0.5784 0.5574 1.7352
-0.1152 -16.0596 -426.7986
23.1000 -434.9854 0.6257 0.8923
1.1322 1.8222 0.6696 0.5794 1.7271
-0.1049 -16.0584 -426.8141
23.1200 -436.0777 0.5371 0.7268
0.8777 1.5850 0.7569 0.5998 1.7183
-0.0948 -16.0570 -426.8297
23.1400 -438.7873 -0.6299 -0.4154
0.6307 1.2509 0.8393 0.6184 1.7087
-0.0849 -16.0554 -426.8452
23.1600 -439.9411 0.0463 -1.3417
0.4062 0.8425 0.9162 0.6352 1.6984
-0.0752 -16.0536 -426.8607
23.1800 -440.5392 -0.3555 -1.1877
0.1970 0.3846 0.9865 0.6502 1.6873
-0.0657 -16.0516 -426.8762
23.2000 -440.6224 -0.3005 0.1603 -
0.0356 -0.0949 1.0497 0.6631 1.6755
-0.0563 -16.0495 -426.8917

23.2200 -439.3793 -0.9289 1.7976 -
0.3252 -0.5660 1.1049 0.6741 1.6629
-0.0472 -16.0472 -426.9071
23.2400 -438.3115 1.5230 2.2096 -
0.6999 -0.9981 1.1515 0.6831 1.6495
-0.0383 -16.0447 -426.9226
23.2600 -441.2356 0.8959 0.6351 -
1.1235 -1.3632 1.1886 0.6899 1.6354
-0.0295 -16.0420 -426.9381
23.2800 -446.3544 -1.7087 -1.5767 -
1.4854 -1.6381 1.2155 0.6946 1.6206
-0.0209 -16.0391 -426.9535
23.3000 -446.6749 -0.9263 -2.4286 -
1.6554 -1.8045 1.2317 0.6970 1.6049
-0.0125 -16.0361 -426.9689
23.3200 -444.1218 0.3218 -1.4531 -
1.5568 -1.8519 1.2364 0.6973 1.5885
-0.0043 -16.0328 -426.9844
23.3400 -441.7809 0.4792 0.3315 -
1.2299 -1.7829 1.2294 0.6954 1.5714
0.0037 -16.0294 -426.9998
23.3600 -440.3835 -0.1152 1.6332 -
0.7919 -1.6084 1.2105 0.6912 1.5535
0.0116 -16.0259 -427.0152
23.3800 -439.4152 0.1009 1.6854 -
0.3614 -1.3445 1.1800 0.6849 1.5348
0.0193 -16.0221 -427.0306
23.4000 -439.6040 0.3754 0.5660 -
0.0069 -1.0094 1.1382 0.6766 1.5154
0.0269 -16.0182 -427.0459
23.4200 -440.7942 -0.4655 -0.6696
0.2335 -0.6222 1.0857 0.6662 1.4953
0.0343 -16.0141 -427.0613
23.4400 -441.2076 -0.8857 -1.0580
0.3611 -0.2030 1.0230 0.6538 1.4744
0.0415 -16.0098 -427.0767
23.4600 -439.4135 0.3020 -0.4860
0.4023 0.2276 0.9507 0.6397 1.4528
0.0486 -16.0054 -427.0920
23.4800 -437.7418 0.3034 0.5360
0.3840 0.6499 0.8694 0.6237 1.4306
0.0555 -16.0008 -427.1074
23.5000 -437.9787 0.6963 1.0564
0.3117 1.0461 0.7795 0.6061 1.4076
0.0623 -15.9960 -427.1227
23.5200 -438.6475 -0.4892 0.6696
0.1861 1.4007 0.6818 0.5870 1.3839
0.0689 -15.9910 -427.1380
23.5400 -439.1990 0.2496 -0.1930
0.0538 1.6989 0.5767 0.5664 1.3596
0.0754 -15.9859 -427.1533

23.5600 -439.7280 -0.2767 -0.8618 -
0.0050 1.9272 0.4649 0.5445 1.3346
0.0817 -15.9806 -427.1686
23.5800 -439.8255 -0.1676 -0.8824
0.0841 2.0743 0.3471 0.5214 1.3089
0.0880 -15.9752 -427.1839
23.6000 -438.5385 0.5489 -0.2641
0.3151 2.1326 0.2242 0.4971 1.2826
0.0940 -15.9696 -427.1992
23.6200 -437.4139 0.1939 0.3679
0.6103 2.0975 0.0970 0.4718 1.2557
0.1000 -15.9638 -427.2144
23.6400 -437.8640 -0.0275 0.5320
0.8625 1.9678 -0.0331 0.4456 1.2282
0.1058 -15.9578 -427.2297
23.6600 -438.9235 -0.0565 0.2032
0.9899 1.7475 -0.1648 0.4187 1.2001
0.1115 -15.9517 -427.2449
23.6800 -439.9108 -0.2881 -0.2463
0.9608 1.4469 -0.2964 0.3911 1.1714
0.1170 -15.9455 -427.2601
23.7000 -440.9546 0.2334 -0.4473
0.7699 1.0805 -0.4264 0.3630 1.1421
0.1225 -15.9390 -427.2754
23.7200 -441.4663 -0.7732 -0.0327
0.4301 0.6658 -0.5531 0.3346 1.1123
0.1278 -15.9324 -427.2906
23.7400 -441.5986 -0.0577 0.7245 -
0.0305 0.2236 -0.6749 0.3058 1.0820
0.1330 -15.9257 -427.3058
23.7600 -441.7631 0.6918 1.0724 -
0.5505 -0.2238 -0.7900 0.2769 1.0511
0.1381 -15.9188 -427.3210
23.7800 -443.7211 0.2812 0.3725 -
1.0173 -0.6553 -0.8969 0.2479 1.0197
0.1431 -15.9117 -427.3361
23.8000 -447.2120 -0.6245 -0.9697 -
1.3022 -1.0514 -0.9940 0.2190 0.9878
0.1479 -15.9045 -427.3513
23.8200 -447.9723 -0.1661 -1.7816 -
1.2903 -1.3938 -1.0795 0.1903 0.9555
0.1527 -15.8971 -427.3665
23.8400 -446.9049 -0.0563 -1.4946 -
0.9422 -1.6683 -1.1520 0.1619 0.9227
0.1574 -15.8896 -427.3816
23.8600 -445.6016 0.0839 -0.4652 -
0.3542 -1.8674 -1.2099 0.1340 0.8894
0.1619 -15.8819 -427.3967
23.8800 -444.6695 0.0961 0.4513
0.2940 -1.9905 -1.2520 0.1065 0.8557
0.1664 -15.8740 -427.4119

23.9000 -443.8294 -0.2688 0.8527
0.8010 -2.0405 -1.2774 0.0796 0.8216
0.1708 -15.8660 -427.4270
23.9200 -443.3180 -0.0730 1.1521
1.0000 -2.0204 -1.2855 0.0534 0.7871
0.1751 -15.8579 -427.4421
23.9400 -442.9920 -0.4862 1.5874
0.8192 -1.9344 -1.2764 0.0280 0.7522
0.1793 -15.8495 -427.4572
23.9600 -442.8101 0.7138 1.4696
0.3212 -1.7905 -1.2504 0.0033 0.7169
0.1834 -15.8411 -427.4722
23.9800 -444.1349 1.1286 0.2328 -
0.3327 -1.5993 -1.2082 -0.0205 0.6813
0.1874 -15.8325 -427.4873
24.0000 -447.8281 -0.5933 -1.4456 -
0.9431 -1.3718 -1.1507 -0.0435 0.6454
0.1914 -15.8237 -427.5024
24.0200 -449.2043 -0.8444 -2.1501 -
1.3222 -1.1185 -1.0789 -0.0656 0.6092
0.1952 -15.8148 -427.5174
24.0400 -447.2080 0.4322 -1.3894 -
1.3432 -0.8500 -0.9943 -0.0869 0.5727
0.1990 -15.8058 -427.5324
24.0600 -444.5438 0.2200 -0.0110 -
1.0087 -0.5767 -0.8982 -0.1074 0.5359
0.2028 -15.7966 -427.5475
24.0800 -443.3299 0.0544 0.8242 -
0.4084 -0.3076 -0.7923 -0.1271 0.4989
0.2064 -15.7872 -427.5625
24.1000 -442.7108 -0.2214 0.8346
0.3307 -0.0512 -0.6783 -0.1460 0.4616
0.2100 -15.7777 -427.5775
24.1200 -442.0806 0.0232 0.3617
1.0591 0.1839 -0.5583 -0.1641 0.4241
0.2135 -15.7681 -427.5925
24.1400 -441.0949 -0.0606 0.0234
1.6368 0.3895 -0.4341 -0.1815 0.3864
0.2170 -15.7583 -427.6074
24.1600 -440.1823 0.0231 0.2523
1.9690 0.5583 -0.3078 -0.1982 0.3486
0.2204 -15.7484 -427.6224
24.1800 -439.8822 -0.2962 0.7261
2.0052 0.6871 -0.1815 -0.2142 0.3106
0.2237 -15.7384 -427.6374
24.2000 -439.7394 0.4182 0.7324
1.7447 0.7763 -0.0573 -0.2296 0.2724
0.2270 -15.7282 -427.6523
24.2200 -439.7981 0.9785 0.0741
1.2415 0.8297 0.0629 -0.2442 0.2341
0.2303 -15.7178 -427.6672

24.2400 -442.0465 -0.0284 -0.8672
0.5765 0.8554 0.1773 -0.2582 0.1957
0.2335 -15.7073 -427.6822

24.2600 -444.2293 -0.9842 -1.1447 -
0.1790 0.8645 0.2843 -0.2716 0.1572
0.2366 -15.6967 -427.6971

24.2800 -443.6391 -0.0868 -0.1680 -
0.9755 0.8698 0.3822 -0.2843 0.1186
0.2397 -15.6860 -427.7120

24.3000 -442.1699 0.4262 1.2604 -
1.7300 0.8815 0.4696 -0.2964 0.0800
0.2428 -15.6751 -427.7269

24.3200 -441.9036 0.5885 1.7337 -
2.3272 0.9063 0.5454 -0.3080 0.0413
0.2458 -15.6641 -427.7417

24.3400 -444.1436 -0.0301 0.6852 -
2.6576 0.9482 0.6087 -0.3190 0.0026
0.2488 -15.6529 -427.7566

24.3600 -446.1370 -0.3465 -1.2800 -
2.6125 1.0055 0.6589 -0.3295 -0.0361
0.2518 -15.6416 -427.7715

24.3800 -446.3084 0.5853 -3.0343 -
2.1199 1.0710 0.6959 -0.3395 -0.0747
0.2547 -15.6302 -427.7863

24.4000 -446.3831 -0.1434 -3.5212 -
1.1758 1.1326 0.7199 -0.3491 -0.1134
0.2576 -15.6186 -427.8011

24.4200 -446.0286 -2.1543 -2.2213
0.1025 1.1748 0.7313 -0.3582 -0.1520
0.2605 -15.6069 -427.8160

24.4400 -440.2350 0.0746 0.4911
1.4453 1.1817 0.7308 -0.3669 -0.1905
0.2634 -15.5951 -427.8308

24.4600 -434.1054 1.0990 3.0849
2.5306 1.1377 0.7191 -0.3753 -0.2289
0.2662 -15.5832 -427.8456

24.4800 -434.4856 1.0941 3.7912
3.0547 1.0282 0.6972 -0.3833 -0.2672
0.2690 -15.5711 -427.8604

24.5000 -437.9836 -0.3978 2.3915
2.8672 0.8465 0.6664 -0.3910 -0.3054
0.2718 -15.5589 -427.8751

24.5200 -440.9248 -0.9778 0.1746
2.0922 0.6018 0.6280 -0.3983 -0.3434
0.2746 -15.5465 -427.8899

24.5400 -443.5477 0.1162 -1.4479
0.9638 0.3116 0.5839 -0.4053 -0.3813
0.2774 -15.5341 -427.9047

24.5600 -445.3011 -0.2093 -1.9642 -
0.2295 -0.0037 0.5355 -0.4121 -0.4190
0.2802 -15.5215 -427.9194

24.5800 -446.3484 0.5313 -1.6000 -
1.2060 -0.3236 0.4844 -0.4187 -0.4565
0.2830 -15.5088 -427.9341
24.6000 -447.0128 -0.3234 -0.9347 -
1.8013 -0.6275 0.4321 -0.4250 -0.4937
0.2858 -15.4959 -427.9488
24.6200 -447.0274 0.0217 -0.3183 -
1.9753 -0.8960 0.3801 -0.4312 -0.5308
0.2886 -15.4830 -427.9636
24.6400 -446.6424 -0.6481 0.2217 -
1.7969 -1.1120 0.3299 -0.4372 -0.5676
0.2914 -15.4699 -427.9783
24.6600 -446.2298 0.2818 0.6164 -
1.4329 -1.2629 0.2828 -0.4431 -0.6042
0.2942 -15.4567 -427.9929
24.6800 -445.9678 -0.1922 0.6751 -
1.0354 -1.3398 0.2399 -0.4488 -0.6404
0.2970 -15.4434 -428.0076
24.7000 -445.7417 0.1787 0.4229 -
0.6532 -1.3416 0.2017 -0.4545 -0.6764
0.2998 -15.4299 -428.0223
24.7200 -445.5531 0.3503 0.0573 -
0.2792 -1.2756 0.1685 -0.4600 -0.7121
0.3027 -15.4164 -428.0369
24.7400 -445.4265 -0.2822 -0.2638
0.0900 -1.1551 0.1404 -0.4653 -0.7474
0.3055 -15.4027 -428.0516
24.7600 -445.2730 0.1021 -0.3083
0.4320 -0.9944 0.1175 -0.4705 -0.7825
0.3084 -15.3889 -428.0662
24.7800 -444.7370 -0.0740 -0.1194
0.6979 -0.8063 0.0995 -0.4754 -0.8171
0.3113 -15.3750 -428.0808
24.8000 -443.6271 -0.1349 0.1137
0.8579 -0.6017 0.0863 -0.4801 -0.8514
0.3143 -15.3610 -428.0954
24.8200 -443.1100 0.3552 0.3296
0.9128 -0.3901 0.0775 -0.4844 -0.8853
0.3173 -15.3468 -428.1100
24.8400 -443.8459 0.1671 0.0944
0.8911 -0.1804 0.0724 -0.4884 -0.9188
0.3203 -15.3326 -428.1246
24.8600 -444.7791 -0.3467 -0.4167
0.8240 0.0201 0.0703 -0.4919 -0.9520
0.3233 -15.3182 -428.1392
24.8800 -444.5205 -0.7898 -0.4998
0.7240 0.2066 0.0701 -0.4950 -0.9846
0.3264 -15.3037 -428.1537
24.9000 -443.0489 0.0637 0.0939
0.5646 0.3762 0.0705 -0.4975 -1.0169
0.3295 -15.2892 -428.1683

24.9200 -442.2022 0.5509 0.8031
0.3026 0.5276 0.0706 -0.4995 -1.0487
0.3326 -15.2745 -428.1828
24.9400 -443.1182 0.4104 1.0453 -
0.0843 0.6607 0.0691 -0.5007 -1.0800
0.3358 -15.2596 -428.1973
24.9600 -444.5451 -0.7517 0.5645 -
0.5566 0.7750 0.0650 -0.5013 -1.1109
0.3391 -15.2447 -428.2118
24.9800 -445.1349 0.0145 -0.3006 -
1.0130 0.8685 0.0575 -0.5011 -1.1413
0.3424 -15.2297 -428.2263
25.0000 -445.4661 0.4183 -0.7277 -
1.3162 0.9390 0.0460 -0.5000 -1.1712
0.3458 -15.2146 -428.2408
25.0200 -445.6000 0.0257 -0.6647 -
1.3568 0.9836 0.0302 -0.4980 -1.2005
0.3492 -15.1993 -428.2553
25.0400 -445.4735 -0.0836 -0.5078 -
1.0874 0.9997 0.0100 -0.4950 -1.2293
0.3527 -15.1840 -428.2698
25.0600 -445.1506 0.0251 -0.4550 -
0.5316 0.9844 -0.0142 -0.4911 -1.2576
0.3562 -15.1686 -428.2842
25.0800 -444.5878 -0.2667 -0.3925
0.1961 0.9335 -0.0421 -0.4861 -1.2854
0.3598 -15.1530 -428.2987
25.1000 -443.1252 -0.2218 -0.0109
0.9476 0.8426 -0.0729 -0.4800 -1.3125
0.3635 -15.1373 -428.3131
25.1200 -442.0295 0.9723 0.4311
1.5560 0.7069 -0.1058 -0.4729 -1.3391
0.3672 -15.1216 -428.3275
25.1400 -442.0000 -0.5928 0.7769
1.8694 0.5239 -0.1401 -0.4647 -1.3651
0.3710 -15.1057 -428.3419
25.1600 -442.0000 -0.2500 1.0890
1.8042 0.2957 -0.1747 -0.4555 -1.3905
0.3749 -15.0898 -428.3563
25.1800 -442.2422 0.7513 1.0720
1.3651 0.0320 -0.2087 -0.4452 -1.4153
0.3789 -15.0737 -428.3707
25.2000 -444.5996 -0.0661 0.4209
0.6351 -0.2505 -0.2411 -0.4339 -1.4394
0.3829 -15.0576 -428.3851
25.2200 -447.0268 -0.8244 -0.3677 -
0.2292 -0.5324 -0.2708 -0.4217 -1.4629
0.3871 -15.0413 -428.3994
25.2400 -448.0059 -0.0174 -0.7715 -
1.0405 -0.7939 -0.2967 -0.4084 -1.4858
0.3913 -15.0250 -428.4138

25.2600 -448.6986 0.2397 -0.9263 -
1.6314 -1.0140 -0.3178 -0.3943 -1.5080
0.3956 -15.0085 -428.4281
25.2800 -448.8924 0.0743 -0.8666 -
1.8760 -1.1726 -0.3331 -0.3793 -1.5295
0.4000 -14.9920 -428.4424
25.3000 -448.6656 0.4836 -0.7685 -
1.7266 -1.2552 -0.3417 -0.3635 -1.5503
0.4045 -14.9754 -428.4567
25.3200 -448.1990 -0.4074 -0.7007 -
1.2311 -1.2570 -0.3431 -0.3469 -1.5704
0.4090 -14.9586 -428.4710
25.3400 -447.2871 -0.2177 -0.1582 -
0.5269 -1.1803 -0.3372 -0.3296 -1.5898
0.4137 -14.9418 -428.4853
25.3600 -444.9639 -0.2751 0.9078
0.1861 -1.0317 -0.3238 -0.3117 -1.6085
0.4185 -14.9249 -428.4996
25.3800 -443.2527 1.0152 1.5810
0.7277 -0.8215 -0.3037 -0.2933 -1.6265
0.4234 -14.9079 -428.5139
25.4000 -443.6816 0.0953 1.1370
0.9699 -0.5621 -0.2774 -0.2745 -1.6438
0.4283 -14.8908 -428.5281
25.4200 -444.7863 -0.5623 0.1312
0.8914 -0.2695 -0.2457 -0.2554 -1.6603
0.4334 -14.8736 -428.5424
25.4400 -445.1976 -0.4473 -0.5542
0.5742 0.0388 -0.2097 -0.2361 -1.6760
0.4386 -14.8564 -428.5566
25.4600 -445.1451 0.6097 -0.6913
0.1717 0.3420 -0.1702 -0.2168 -1.6910
0.4439 -14.8390 -428.5708
25.4800 -445.0414 -0.3447 -0.4243 -
0.1595 0.6167 -0.1284 -0.1975 -1.7052
0.4492 -14.8216 -428.5850
25.5000 -444.8296 0.0016 -0.2372 -
0.3026 0.8411 -0.0853 -0.1785 -1.7187
0.4547 -14.8040 -428.5992
25.5200 -444.3904 0.4171 -0.2302 -
0.2052 0.9992 -0.0421 -0.1599 -1.7314
0.4603 -14.7864 -428.6134
25.5400 -443.9691 -0.2408 -0.2359
0.1024 1.0823 0.0003 -0.1418 -1.7433
0.4660 -14.7687 -428.6276
25.5600 -443.6697 0.1755 -0.1357
0.5263 1.0864 0.0409 -0.1244 -1.7543
0.4719 -14.7509 -428.6417
25.5800 -443.3695 -0.2868 0.2125
0.9245 1.0143 0.0794 -0.1078 -1.7646
0.4778 -14.7331 -428.6559

25.6000 -442.9016 -0.2325 0.7878
1.1367 0.8735 0.1152 -0.0921 -1.7741
0.4838 -14.7151 -428.6700
25.6200 -442.1768 0.0267 1.1724
1.0645 0.6773 0.1483 -0.0775 -1.7828
0.4900 -14.6971 -428.6841
25.6400 -441.8012 0.4568 0.8823
0.7153 0.4428 0.1785 -0.0641 -1.7907
0.4962 -14.6790 -428.6982
25.6600 -443.7283 -0.1995 -0.0664
0.1756 0.1892 0.2057 -0.0520 -1.7977
0.5026 -14.6608 -428.7123
25.6800 -446.6306 -0.0112 -1.0970 -
0.4054 -0.0621 0.2299 -0.0414 -1.8039
0.5091 -14.6425 -428.7264
25.7000 -447.1508 -0.5773 -1.5505 -
0.8696 -0.2903 0.2511 -0.0323 -1.8093
0.5157 -14.6241 -428.7405
25.7200 -446.8068 0.5593 -1.0934 -
1.1267 -0.4770 0.2692 -0.0249 -1.8139
0.5224 -14.6057 -428.7545
25.7400 -446.2744 -0.1560 -0.0174 -
1.1721 -0.6092 0.2843 -0.0192 -1.8176
0.5292 -14.5872 -428.7686
25.7600 -445.2079 -0.5362 1.1697 -
1.0588 -0.6798 0.2967 -0.0154 -1.8205
0.5361 -14.5686 -428.7826
25.7800 -443.7140 0.2298 1.8671 -
0.8580 -0.6885 0.3065 -0.0134 -1.8226
0.5431 -14.5500 -428.7966
25.8000 -443.3419 1.4887 1.3364 -
0.6061 -0.6412 0.3142 -0.0134 -1.8238
0.5503 -14.5312 -428.8107
25.8200 -445.8750 0.2585 -0.6286 -
0.3088 -0.5492 0.3201 -0.0154 -1.8241
0.5575 -14.5124 -428.8247
25.8400 -448.6162 -0.8852 -2.3886
0.0409 -0.4253 0.3247 -0.0195 -1.8237
0.5648 -14.4935 -428.8386
25.8600 -447.4928 -1.8541 -2.0058
0.4226 -0.2831 0.3283 -0.0255 -1.8224
0.5723 -14.4746 -428.8526
25.8800 -442.5603 0.9417 0.3514
0.7580 -0.1364 0.3311 -0.0337 -1.8202
0.5798 -14.4555 -428.8666
25.9000 -439.6000 0.9588 2.3582
0.9302 0.0018 0.3335 -0.0439 -1.8173
0.5875 -14.4364 -428.8805
25.9200 -440.7014 -0.2918 2.4882
0.8538 0.1211 0.3354 -0.0561 -1.8135
0.5952 -14.4173 -428.8945

25.9400 -442.8268 -0.2868 1.0389
0.5533 0.2156 0.3370 -0.0703 -1.8088
0.6031 -14.3980 -428.9084
25.9600 -444.5947 -0.0900 -0.8875
0.1693 0.2817 0.3384 -0.0865 -1.8034
0.6110 -14.3787 -428.9223
25.9800 -446.3979 0.4089 -2.2381 -
0.1320 0.3166 0.3397 -0.1047 -1.7971
0.6190 -14.3593 -428.9362
26.0000 -447.2995 -0.8709 -2.2356 -
0.2402 0.3206 0.3412 -0.1246 -1.7900
0.6271 -14.3398 -428.9501
26.0200 -444.9927 -0.5025 -0.5789 -
0.1580 0.2949 0.3429 -0.1464 -1.7820
0.6354 -14.3203 -428.9640
26.0400 -441.0408 0.7565 1.5978
0.0242 0.2435 0.3445 -0.1698 -1.7733
0.6437 -14.3007 -428.9778
26.0600 -440.1633 1.0162 2.5013
0.2116 0.1732 0.3460 -0.1948 -1.7638
0.6520 -14.2810 -428.9917
26.0800 -442.7387 -0.3801 1.4403
0.3368 0.0913 0.3471 -0.2211 -1.7534
0.6605 -14.2613 -429.0055
26.1000 -445.1028 -0.5942 -0.5936
0.3819 0.0054 0.3477 -0.2488 -1.7423
0.6691 -14.2415 -429.0194
26.1200 -445.5702 0.3560 -2.0619
0.3575 -0.0775 0.3473 -0.2775 -1.7304
0.6777 -14.2216 -429.0332
26.1400 -445.8384 -0.0096 -2.0490
0.2710 -0.1518 0.3458 -0.3072 -1.7177
0.6864 -14.2016 -429.0470
26.1600 -445.7404 -0.7454 -0.6234
0.1267 -0.2126 0.3428 -0.3376 -1.7043
0.6952 -14.1816 -429.0608
26.1800 -443.5657 0.1542 1.3243 -
0.0868 -0.2544 0.3380 -0.3686 -1.6901
0.7040 -14.1615 -429.0746
26.2000 -441.3144 0.8346 2.5385 -
0.3727 -0.2729 0.3313 -0.3999 -1.6751
0.7129 -14.1414 -429.0883
26.2200 -442.4172 1.1592 1.9435 -
0.7026 -0.2651 0.3223 -0.4314 -1.6595
0.7219 -14.1211 -429.1021
26.2400 -446.6665 -0.7581 -0.1811 -
1.0060 -0.2309 0.3106 -0.4629 -1.6430
0.7309 -14.1008 -429.1158
26.2600 -448.5831 -0.9198 -2.0514 -
1.1798 -0.1723 0.2958 -0.4942 -1.6259
0.7400 -14.0805 -429.1295

26.2800 -447.9247 -0.1433 -2.3480 -
1.1397 -0.0934 0.2774 -0.5249 -1.6080
0.7492 -14.0601 -429.1433
26.3000 -446.6710 -0.1543 -1.2327 -
0.8702 -0.0004 0.2551 -0.5550 -1.5895
0.7584 -14.0396 -429.1570
26.3200 -445.0500 -0.9199 0.3651 -
0.4342 0.0979 0.2286 -0.5842 -1.5702
0.7677 -14.0190 -429.1706
26.3400 -442.2334 0.4654 1.6897
0.0654 0.1925 0.1976 -0.6123 -1.5503
0.7770 -13.9984 -429.1843
26.3600 -440.2654 1.2973 2.0258
0.5232 0.2740 0.1619 -0.6391 -1.5297
0.7864 -13.9777 -429.1980
26.3800 -441.6464 0.4910 0.9753
0.8732 0.3327 0.1215 -0.6643 -1.5085
0.7958 -13.9569 -429.2116
26.4000 -444.8719 -0.4797 -0.6494
1.0921 0.3599 0.0763 -0.6878 -1.4866
0.8052 -13.9361 -429.2253
26.4200 -445.6072 -0.8500 -1.4755
1.2139 0.3496 0.0261 -0.7093 -1.4640
0.8147 -13.9152 -429.2389
26.4400 -443.8858 0.3785 -1.1433
1.2906 0.2989 -0.0290 -0.7287 -1.4408
0.8243 -13.8942 -429.2525
26.4600 -442.2969 -0.0327 -0.2331
1.3331 0.2110 -0.0888 -0.7457 -1.4170
0.8338 -13.8732 -429.2661
26.4800 -442.4040 0.0766 0.5305
1.2904 0.0952 -0.1534 -0.7601 -1.3927
0.8434 -13.8521 -429.2797
26.5000 -443.0953 -0.0015 0.7638
1.0935 -0.0355 -0.2226 -0.7719 -1.3677
0.8531 -13.8310 -429.2933
26.5200 -443.8718 -0.0806 0.6234
0.6826 -0.1657 -0.2964 -0.7808 -1.3421
0.8627 -13.8097 -429.3069
26.5400 -444.8084 0.1462 0.2837
0.0731 -0.2794 -0.3747 -0.7868 -1.3160
0.8724 -13.7885 -429.3204
26.5600 -445.7472 -0.3799 -0.0248 -
0.6478 -0.3610 -0.4572 -0.7897 -1.2893
0.8821 -13.7671 -429.3340
26.5800 -446.4648 0.0282 -0.1873 -
1.3443 -0.3966 -0.5434 -0.7894 -1.2621
0.8919 -13.7457 -429.3475
26.6000 -447.1134 0.3028 -0.2446 -
1.8634 -0.3747 -0.6327 -0.7859 -1.2343
0.9016 -13.7242 -429.3610

26.6200 -447.4000 0.1129 -0.3847 -
2.0570 -0.2892 -0.7243 -0.7793 -1.2061
0.9114 -13.7027 -429.3745
26.6400 -447.3244 -0.2829 -0.7889 -
1.8356 -0.1436 -0.8168 -0.7695 -1.1774
0.9212 -13.6811 -429.3880
26.6600 -447.1139 0.3521 -1.0934 -
1.2234 0.0466 -0.9085 -0.7565 -1.1482
0.9309 -13.6594 -429.4015
26.6800 -446.5777 -1.1637 -0.7192 -
0.3440 0.2590 -0.9978 -0.7405 -1.1185
0.9407 -13.6377 -429.4149
26.7000 -443.6366 -0.3100 0.4342
0.5878 0.4696 -1.0829 -0.7213 -1.0884
0.9505 -13.6159 -429.4284
26.7200 -440.7709 0.9750 1.6345
1.3496 0.6538 -1.1619 -0.6991 -1.0579
0.9603 -13.5940 -429.4418
26.7400 -440.8509 0.9500 1.9122
1.7490 0.7885 -1.2330 -0.6740 -1.0269
0.9701 -13.5721 -429.4552
26.7600 -442.1279 -0.6266 1.1528
1.7054 0.8556 -1.2946 -0.6461 -0.9956
0.9799 -13.5501 -429.4686
26.7800 -443.2082 -0.6845 0.2310
1.2770 0.8479 -1.3450 -0.6154 -0.9639
0.9897 -13.5280 -429.4820
26.8000 -443.7955 -0.1039 -0.2504
0.6244 0.7672 -1.3827 -0.5820 -0.9319
0.9995 -13.5059 -429.4954
26.8200 -444.3407 -0.0572 -0.4590 -
0.0452 0.6191 -1.4064 -0.5460 -0.8995
1.0093 -13.4838 -429.5088
26.8400 -445.2109 0.6591 -0.7465 -
0.5648 0.4110 -1.4152 -0.5077 -0.8668
1.0190 -13.4615 -429.5222
26.8600 -446.4550 0.0958 -1.1654 -
0.8233 0.1539 -1.4081 -0.4671 -0.8338
1.0287 -13.4392 -429.5355
26.8800 -447.1000 -0.3910 -1.1446 -
0.7999 -0.1365 -1.3846 -0.4244 -0.8004
1.0385 -13.4169 -429.5489
26.9000 -446.0453 -0.4199 -0.3804 -
0.5530 -0.4401 -1.3442 -0.3799 -0.7669
1.0482 -13.3944 -429.5622
26.9200 -444.3000 0.6791 0.5144 -
0.1926 -0.7366 -1.2863 -0.3337 -0.7331
1.0579 -13.3720 -429.5755
26.9400 -443.8485 -0.0196 0.8461
0.1531 -1.0049 -1.2108 -0.2859 -0.6990
1.0675 -13.3494 -429.5888

26.9600 -444.3236 -0.1414 0.6085
0.3674 -1.2250 -1.1174 -0.2369 -0.6647
1.0771 -13.3268 -429.6021
26.9800 -444.7711 -0.1396 0.2172
0.3599 -1.3805 -1.0062 -0.1869 -0.6302
1.0867 -13.3041 -429.6153
27.0000 -444.7419 -0.0776 0.0721
0.1090 -1.4598 -0.8776 -0.1360 -0.5956
1.0963 -13.2814 -429.6286
27.0200 -444.5779 -0.1945 0.3366 -
0.3329 -1.4597 -0.7326 -0.0845 -0.5607
1.1058 -13.2586 -429.6418
27.0400 -444.5102 0.5712 0.5576 -
0.8711 -1.3802 -0.5727 -0.0326 -0.5257
1.1153 -13.2358 -429.6551
27.0600 -445.0575 0.0543 0.1721 -
1.3704 -1.2237 -0.4000 0.0193 -0.4906
1.1247 -13.2129 -429.6683
27.0800 -445.7719 -0.3891 -0.4359 -
1.6993 -0.9951 -0.2170 0.0711 -0.4554
1.1341 -13.1899 -429.6815
27.1000 -445.5718 -0.3715 -0.7068 -
1.7696 -0.7028 -0.0261 0.1226 -0.4201
1.1435 -13.1669 -429.6947
27.1200 -443.7100 0.4201 -0.4676 -
1.5541 -0.3591 0.1699 0.1734 -0.3847
1.1528 -13.1438 -429.7079
27.1400 -441.7314 0.0908 0.0293 -
1.0915 0.0188 0.3684 0.2234 -0.3492
1.1620 -13.1206 -429.7210
27.1600 -440.6021 0.6452 0.3194 -
0.4701 0.4111 0.5669 0.2722 -0.3137
1.1712 -13.0974 -429.7342
27.1800 -439.6440 -0.2746 0.4285
0.2119 0.7963 0.7627 0.3197 -0.2781
1.1804 -13.0742 -429.7473
27.2000 -438.4636 -0.8803 0.6340
0.8602 1.1518 0.9532 0.3657 -0.2426
1.1895 -13.0508 -429.7605
27.2200 -436.7976 0.6095 0.6997
1.4241 1.4533 1.1359 0.4098 -0.2071
1.1985 -13.0274 -429.7736
27.2400 -435.9000 1.3531 -0.1053
1.9214 1.6778 1.3083 0.4518 -0.1716
1.2074 -13.0040 -429.7867
27.2600 -437.2042 0.4920 -1.5381
2.3624 1.8053 1.4678 0.4915 -0.1361
1.2163 -12.9805 -429.7998
27.2800 -439.0833 -2.2743 -2.0051
2.7406 1.8232 1.6121 0.5287 -0.1007
1.2252 -12.9569 -429.8129

27.3000 -437.0674 -2.1973 -0.1973
3.0206 1.7287 1.7392 0.5630 -0.0654
1.2339 -12.9333 -429.8259
27.3200 -429.8636 2.2426 2.4851
3.0958 1.5283 1.8478 0.5944 -0.0302
1.2426 -12.9096 -429.8390
27.3400 -428.5885 3.0317 3.0247
2.8733 1.2366 1.9371 0.6224 0.0049
1.2512 -12.8859 -429.8520
27.3600 -437.0506 -1.6037 0.3616
2.3303 0.8751 2.0066 0.6470 0.0399
1.2597 -12.8621 -429.8650
27.3800 -441.7924 -1.8916 -2.7390
1.5194 0.4685 2.0562 0.6679 0.0746
1.2681 -12.8382 -429.8780
27.4000 -441.3893 -0.2728 -3.2715
0.5098 0.0430 2.0856 0.6850 0.1093
1.2765 -12.8143 -429.8910
27.4200 -440.6841 0.3023 -1.1027 -
0.6164 -0.3736 2.0945 0.6982 0.1437
1.2847 -12.7904 -429.9040
27.4400 -439.8133 -0.4151 1.9738 -
1.7529 -0.7535 2.0828 0.7075 0.1779
1.2929 -12.7663 -429.9170
27.4600 -438.7421 0.0772 3.8267 -
2.7968 -1.0702 2.0502 0.7128 0.2119
1.3010 -12.7423 -429.9300
27.4800 -438.7844 2.3414 3.0050 -
3.6386 -1.2996 1.9966 0.7142 0.2456
1.3090 -12.7181 -429.9429
27.5000 -444.3062 -0.2465 -0.2498 -
4.1715 -1.4214 1.9220 0.7119 0.2791
1.3169 -12.6939 -429.9558
27.5200 -449.3493 -1.6300 -3.4204 -
4.2781 -1.4237 1.8268 0.7059 0.3123
1.3247 -12.6697 -429.9688
27.5400 -448.1944 -0.1241 -4.3016 -
3.8366 -1.3067 1.7119 0.6964 0.3452
1.3323 -12.6454 -429.9817
27.5600 -444.5309 0.8585 -2.9263 -
2.8190 -1.0837 1.5787 0.6836 0.3777
1.3399 -12.6210 -429.9946
27.5800 -440.8538 -0.4259 -0.4909 -
1.3673 -0.7776 1.4290 0.6677 0.4100
1.3474 -12.5966 -430.0074
27.6000 -437.0845 -0.1030 1.6927
0.2538 -0.4146 1.2646 0.6489 0.4418
1.3548 -12.5721 -430.0203
27.6200 -433.9413 0.4791 2.8899
1.7546 -0.0217 1.0876 0.6273 0.4733
1.3620 -12.5476 -430.0332

27.6400 -433.0108 -0.1847 3.1267
2.8723 0.3740 0.9004 0.6033 0.5045
1.3691 -12.5230 -430.0460
27.6600 -432.5875 -0.0043 2.6526
3.4190 0.7457 0.7054 0.5769 0.5352
1.3762 -12.4983 -430.0588
27.6800 -432.8118 0.7988 1.5877
3.3606 1.0710 0.5049 0.5484 0.5655
1.3831 -12.4736 -430.0716
27.7000 -435.7556 -0.1840 0.1974
2.8199 1.3353 0.3014 0.5179 0.5954
1.3898 -12.4489 -430.0844
27.7200 -438.1762 -0.6403 -0.8872
1.9837 1.5279 0.0973 0.4857 0.6248
1.3965 -12.4241 -430.0972
27.7400 -439.0589 0.0588 -1.3413
1.0613 1.6388 -0.1051 0.4519 0.6538
1.4030 -12.3992 -430.1100
27.7600 -439.7907 0.5580 -1.6438
0.2686 1.6594 -0.3037 0.4169 0.6823
1.4094 -12.3743 -430.1228
27.7800 -441.0027 0.5149 -2.2815 -
0.2101 1.5825 -0.4966 0.3806 0.7103
1.4157 -12.3493 -430.1355
27.8000 -442.3989 -0.5673 -2.7151 -
0.2964 1.4071 -0.6819 0.3435 0.7379
1.4218 -12.3243 -430.1482
27.8200 -442.5368 -0.8271 -1.8581 -
0.0597 1.1433 -0.8582 0.3057 0.7649
1.4278 -12.2992 -430.1610
27.8400 -439.4970 0.1161 0.2817
0.3368 0.8083 -1.0243 0.2674 0.7913
1.4336 -12.2741 -430.1737
27.8600 -436.7142 0.7347 2.3285
0.7097 0.4224 -1.1792 0.2289 0.8173
1.4393 -12.2489 -430.1864
27.8800 -437.0069 0.2215 3.1340
0.8832 0.0072 -1.3218 0.1903 0.8426
1.4449 -12.2236 -430.1990
27.9000 -438.2996 -0.5194 2.6976
0.7368 -0.4164 -1.4514 0.1518 0.8674
1.4503 -12.1983 -430.2117
27.9200 -440.0792 0.5571 1.4173
0.2826 -0.8316 -1.5671 0.1138 0.8917
1.4555 -12.1730 -430.2244
27.9400 -443.1749 -0.2710 -0.1949 -
0.3411 -1.2255 -1.6681 0.0763 0.9153
1.4606 -12.1476 -430.2370
27.9600 -445.6568 -0.2349 -1.6096 -
0.9295 -1.5879 -1.7536 0.0395 0.9383
1.4656 -12.1221 -430.2496

27.9800 -446.8159 0.5740 -2.4079 -
1.2831 -1.9097 -1.8227 0.0038 0.9608
1.4704 -12.0966 -430.2623
28.0000 -447.6166 -0.0931 -2.3973 -
1.2641 -2.1826 -1.8745 -0.0308 0.9825
1.4750 -12.0710 -430.2749
28.0200 -447.5451 -0.9019 -1.5082 -
0.8792 -2.4002 -1.9084 -0.0639 1.0037
1.4795 -12.0454 -430.2875
28.0400 -444.6843 -0.0238 0.1332 -
0.2865 -2.5563 -1.9237 -0.0955 1.0242
1.4838 -12.0197 -430.3000
28.0600 -441.6787 0.9492 1.7000
0.3031 -2.6456 -1.9201 -0.1253 1.0440
1.4880 -11.9940 -430.3126
28.0800 -441.4475 -0.2071 2.1941
0.6784 -2.6629 -1.8978 -0.1532 1.0631
1.4920 -11.9683 -430.3251
28.1000 -442.1567 -0.0454 1.7428
0.7033 -2.6043 -1.8578 -0.1790 1.0816
1.4958 -11.9424 -430.3377
28.1200 -443.0597 0.4808 0.7342
0.3904 -2.4674 -1.8013 -0.2025 1.0994
1.4994 -11.9166 -430.3502
28.1400 -444.4685 0.2132 -0.5513 -
0.1335 -2.2516 -1.7298 -0.2237 1.1164
1.5029 -11.8906 -430.3627
28.1600 -445.8536 -0.5498 -1.5244 -
0.7019 -1.9588 -1.6454 -0.2424 1.1328
1.5062 -11.8646 -430.3752
28.1800 -445.9272 -0.2778 -1.6367 -
1.1562 -1.5927 -1.5499 -0.2586 1.1484
1.5093 -11.8386 -430.3877
28.2000 -444.7955 -0.1185 -1.0427 -
1.3945 -1.1595 -1.4456 -0.2722 1.1633
1.5122 -11.8125 -430.4002
28.2200 -443.2717 -0.0921 -0.1853 -
1.4140 -0.6700 -1.3345 -0.2830 1.1775
1.5149 -11.7864 -430.4126
28.2400 -441.4758 -0.1292 0.8554 -
1.2714 -0.1379 -1.2189 -0.2911 1.1909
1.5175 -11.7602 -430.4251
28.2600 -439.3990 0.4403 1.5853 -
1.0152 0.4194 -1.1009 -0.2962 1.2035
1.5199 -11.7340 -430.4375
28.2800 -438.7284 0.3308 1.2526 -
0.6754 0.9817 -0.9827 -0.2983 1.2154
1.5221 -11.7077 -430.4499
28.3000 -439.2654 0.0200 -0.1043 -
0.2622 1.5259 -0.8665 -0.2974 1.2265
1.5241 -11.6814 -430.4623

28.3200	-439.7813	-0.1591	-1.4714	
0.2189	2.0277	-0.7544	-0.2933	1.2368
	1.5259	-11.6550	-430.4747	
28.3400	-438.9337	-0.1444	-1.7235	
0.7348	2.4629	-0.6487	-0.2861	1.2464
	1.5275	-11.6285	-430.4871	
28.3600	-436.7156	-0.2460	-0.6624	
1.2033	2.8089	-0.5514	-0.2755	1.2552
	1.5289	-11.6020	-430.4994	
28.3800	-435.1279	-0.0173	0.8417	
1.5252	3.0459	-0.4645	-0.2617	1.2632
	1.5302	-11.5755	-430.5118	
28.4000	-433.9176	-0.1024	1.7727	
1.6131	3.1598	-0.3896	-0.2445	1.2705
	1.5312	-11.5489	-430.5241	
28.4200	-433.4110	1.1872	1.4735	
1.4183	3.1427	-0.3277	-0.2240	1.2769
	1.5321	-11.5223	-430.5364	
28.4400	-435.7199	0.1866	0.0688	
1.0116	2.9976	-0.2791	-0.2001	1.2826
	1.5327	-11.4956	-430.5487	
28.4600	-438.4578	-0.9833	-1.2823	
0.5545	2.7355	-0.2428	-0.1731	1.2875
	1.5331	-11.4689	-430.5610	
28.4800	-438.4869	-0.4467	-1.4245	
0.1980	2.3718	-0.2178	-0.1430	1.2917
	1.5334	-11.4421	-430.5733	
28.5000	-438.2028	0.2659	-0.6601	
0.0209	1.9254	-0.2027	-0.1100	1.2951
	1.5334	-11.4153	-430.5856	
28.5200	-438.0732	0.1288	0.0609	-
0.0034	1.4179	-0.1961	-0.0743	1.2977
	1.5333	-11.3884	-430.5978	
28.5400	-438.0207	-0.3681	0.4982	
0.0251	0.8738	-0.1967	-0.0361	1.2995
	1.5329	-11.3615	-430.6101	
28.5600	-438.0000	0.0574	0.7931	-
0.0157	0.3181	-0.2031	0.0046	1.3006
	1.5323	-11.3345	-430.6223	
28.5800	-438.4018	0.1001	0.9588	-
0.1998	-0.2258	-0.2140	0.0474	1.3009
	1.5315	-11.3075	-430.6345	
28.6000	-439.2953	0.2149	0.7383	-
0.4915	-0.7382	-0.2279	0.0922	1.3004
	1.5306	-11.2804	-430.6467	
28.6200	-440.5279	0.0335	-0.0585	-
0.8039	-1.2024	-0.2435	0.1388	1.2992
	1.5294	-11.2533	-430.6589	
28.6400	-442.4276	0.2171	-0.9995	-
1.0286	-1.6034	-0.2596	0.1869	1.2972
	1.5280	-11.2261	-430.6711	

28.6600 -443.2930 -0.2868 -1.3875 -
1.0820 -1.9284 -0.2746 0.2364 1.2945
1.5263 -11.1989 -430.6832
28.6800 -442.7069 -0.2831 -1.0625 -
0.9487 -2.1696 -0.2872 0.2870 1.2910
1.5245 -11.1716 -430.6954
28.7000 -441.7393 0.6953 -0.1623 -
0.6970 -2.3238 -0.2960 0.3386 1.2868
1.5225 -11.1443 -430.7075
28.7200 -441.0062 -0.4142 0.9833 -
0.4236 -2.3928 -0.2993 0.3908 1.2818
1.5203 -11.1170 -430.7196
28.7400 -440.3116 0.5252 1.5322 -
0.1972 -2.3840 -0.2959 0.4435 1.2761
1.5178 -11.0896 -430.7317
28.7600 -440.0012 0.1120 0.8985 -
0.0485 -2.3090 -0.2844 0.4963 1.2697
1.5152 -11.0621 -430.7438
28.7800 -440.8138 0.2280 -0.5218
0.0458 -2.1812 -0.2637 0.5492 1.2625
1.5123 -11.0346 -430.7559
28.8000 -441.9944 -0.1684 -1.4618
0.1438 -2.0145 -0.2329 0.6018 1.2545
1.5093 -11.0071 -430.7680
28.8200 -441.5506 -0.7085 -1.0399
0.2877 -1.8206 -0.1915 0.6538 1.2459
1.5060 -10.9795 -430.7800
28.8400 -438.4504 0.6145 0.3324
0.4781 -1.6079 -0.1395 0.7051 1.2365
1.5025 -10.9519 -430.7921
28.8600 -436.6032 0.7415 1.1447
0.6593 -1.3824 -0.0772 0.7555 1.2264
1.4988 -10.9242 -430.8041
28.8800 -437.8612 0.0676 0.5096
0.7685 -1.1482 -0.0049 0.8046 1.2155
1.4949 -10.8965 -430.8161
28.9000 -439.5793 -0.5688 -0.4403
0.7432 -0.9081 0.0767 0.8524 1.2039
1.4908 -10.8687 -430.8281
28.9200 -439.2248 -0.1066 -0.4348
0.5494 -0.6646 0.1666 0.8985 1.1917
1.4865 -10.8409 -430.8401
28.9400 -437.1241 0.4988 0.3325
0.2237 -0.4200 0.2640 0.9428 1.1787
1.4820 -10.8131 -430.8521
28.9600 -436.2545 0.4277 0.6481 -
0.1607 -0.1751 0.3675 0.9850 1.1650
1.4773 -10.7852 -430.8640
28.9800 -437.5568 -0.0505 0.0267 -
0.5349 0.0691 0.4758 1.0251 1.1506
1.4724 -10.7572 -430.8760

29.0000 -438.8497 -0.2763 -0.7205 -
0.8255 0.3102 0.5877 1.0627 1.1355
1.4673 -10.7293 -430.8879
29.0200 -438.2741 -0.7208 -0.6693 -
0.9769 0.5455 0.7016 1.0978 1.1197
1.4620 -10.7012 -430.8998
29.0400 -436.7329 0.6454 0.1367 -
0.9677 0.7735 0.8162 1.1301 1.1032
1.4564 -10.6732 -430.9117
29.0600 -435.8449 0.3025 0.7896 -
0.8172 0.9933 0.9300 1.1594 1.0860
1.4507 -10.6451 -430.9236
29.0800 -435.3123 0.1938 0.6070 -
0.5634 1.2026 1.0416 1.1856 1.0682
1.4448 -10.6169 -430.9355
29.1000 -435.1013 0.3822 -0.1010 -
0.2533 1.3960 1.1496 1.2085 1.0497
1.4387 -10.5887 -430.9473
29.1200 -435.4469 -0.0471 -0.5705
0.0666 1.5630 1.2529 1.2281 1.0305
1.4324 -10.5605 -430.9592
29.1400 -435.8719 -0.6384 -0.3436
0.3504 1.6902 1.3507 1.2440 1.0107
1.4259 -10.5322 -430.9710
29.1600 -434.6861 -0.7750 0.6334
0.5710 1.7631 1.4417 1.2563 0.9903
1.4192 -10.5039 -430.9828
29.1800 -431.6186 1.1030 1.4308
0.7427 1.7679 1.5249 1.2648 0.9692
1.4124 -10.4755 -430.9946
29.2000 -431.4706 1.5146 0.7859
0.9124 1.6934 1.5993 1.2694 0.9475
1.4053 -10.4471 -431.0064
29.2200 -436.0157 -0.7764 -1.1916
1.1035 1.5346 1.6639 1.2701 0.9252
1.3981 -10.4187 -431.0182
29.2400 -438.0576 -1.4591 -2.5272
1.2865 1.2935 1.7180 1.2668 0.9023
1.3906 -10.3902 -431.0300
29.2600 -434.8593 0.1866 -1.4860
1.3942 0.9779 1.7612 1.2595 0.8788
1.3830 -10.3617 -431.0417
29.2800 -431.8652 1.0816 0.9532
1.3297 0.6026 1.7933 1.2482 0.8548
1.3752 -10.3331 -431.0535
29.3000 -432.2205 0.2057 2.5617
0.9876 0.1889 1.8141 1.2329 0.8303
1.3672 -10.3045 -431.0652
29.3200 -433.4757 0.2028 2.3441
0.3141 -0.2369 1.8232 1.2136 0.8052
1.3591 -10.2759 -431.0769

29.3400 -436.1267 1.1672 0.4254 -
0.5877 -0.6459 1.8206 1.1904 0.7796
1.3507 -10.2472 -431.0886
29.3600 -441.3050 -1.1579 -1.8475 -
1.4935 -1.0095 1.8060 1.1633 0.7535
1.3422 -10.2185 -431.1003
29.3800 -443.1292 -0.7011 -2.6697 -
2.1481 -1.2994 1.7794 1.1324 0.7270
1.3335 -10.1897 -431.1120
29.4000 -441.9050 0.6297 -1.7937 -
2.3612 -1.4889 1.7409 1.0978 0.7000
1.3247 -10.1609 -431.1236
29.4200 -440.2122 -0.0034 -0.1902 -
2.1156 -1.5585 1.6905 1.0597 0.6727
1.3156 -10.1321 -431.1353
29.4400 -438.8650 -0.2222 1.1880 -
1.5514 -1.5054 1.6284 1.0181 0.6449
1.3064 -10.1033 -431.1469
29.4600 -437.5930 0.5638 1.6269 -
0.8623 -1.3362 1.5546 0.9732 0.6168
1.2971 -10.0744 -431.1585
29.4800 -437.0222 -0.5940 1.1757 -
0.2519 -1.0615 1.4692 0.9251 0.5883
1.2875 -10.0454 -431.1701
29.5000 -436.8001 0.2443 0.5969
0.1285 -0.6966 1.3723 0.8742 0.5595
1.2778 -10.0165 -431.1817
29.5200 -436.7014 -0.4543 0.3184
0.2293 -0.2651 1.2642 0.8204 0.5304
1.2679 -9.9875 -431.1933
29.5400 -436.8220 0.2082 0.0710
0.1429 0.1999 1.1450 0.7642 0.5011
1.2579 -9.9584 -431.2048
29.5600 -437.0534 0.1163 -0.3377
0.0709 0.6588 1.0151 0.7057 0.4715
1.2477 -9.9294 -431.2164
29.5800 -437.0738 0.0082 -0.7642
0.2036 1.0723 0.8749 0.6452 0.4417
1.2374 -9.9003 -431.2279
29.6000 -436.8827 0.0920 -0.9816
0.6401 1.4073 0.7246 0.5830 0.4116
1.2269 -9.8711 -431.2394
29.6200 -436.5404 -0.3604 -0.5743
1.3164 1.6402 0.5647 0.5192 0.3814
1.2162 -9.8420 -431.2509
29.6400 -435.0572 0.1530 0.3282
2.0477 1.7583 0.3957 0.4541 0.3511
1.2054 -9.8128 -431.2624
29.6600 -433.5797 0.4752 0.8843
2.5853 1.7550 0.2186 0.3880 0.3206
1.1944 -9.7835 -431.2739

29.6800 -434.1078 -0.5472 0.7647
2.6995 1.6286 0.0350 0.3210 0.2900
1.1833 -9.7543 -431.2854
29.7000 -435.8913 -0.5319 0.4701
2.2891 1.3896 -0.1530 0.2534 0.2593
1.1720 -9.7250 -431.2968
29.7200 -437.4201 -0.2122 0.4025
1.4127 1.0615 -0.3433 0.1855 0.2285
1.1605 -9.6956 -431.3083
29.7400 -438.7215 -0.2049 0.3896
0.2737 0.6731 -0.5336 0.1173 0.1977
1.1490 -9.6663 -431.3197
29.7600 -440.2558 0.7149 0.0662 -
0.8583 0.2544 -0.7216 0.0492 0.1669
1.1373 -9.6369 -431.3311
29.7800 -442.9420 0.1904 -0.6222 -
1.7405 -0.1647 -0.9051 -0.0186 0.1361
1.1254 -9.6075 -431.3425
29.8000 -445.3837 -0.6327 -1.2061 -
2.2164 -0.5549 -1.0817 -0.0860 0.1053
1.1134 -9.5780 -431.3539
29.8200 -445.4487 0.2450 -1.1921 -
2.2655 -0.8918 -1.2493 -0.1527 0.0745
1.1012 -9.5486 -431.3653
29.8400 -444.3473 -0.2154 -0.4564 -
1.9730 -1.1595 -1.4056 -0.2186 0.0438
1.0889 -9.5191 -431.3766
29.8600 -443.4363 0.3655 0.6303 -
1.4842 -1.3504 -1.5483 -0.2834 0.0133
1.0765 -9.4896 -431.3879
29.8800 -443.1930 -0.1263 1.4187 -
0.9201 -1.4636 -1.6752 -0.3470 -0.0172
1.0640 -9.4600 -431.3993
29.9000 -443.0387 -0.2485 1.2824 -
0.3594 -1.5021 -1.7845 -0.4091 -0.0475
1.0513 -9.4304 -431.4106
29.9200 -443.0785 0.3026 0.0296
0.1488 -1.4720 -1.8745 -0.4695 -0.0777
1.0384 -9.4008 -431.4219
29.9400 -444.3558 0.4014 -1.5990
0.5775 -1.3818 -1.9440 -0.5282 -0.1077
1.0255 -9.3712 -431.4332
29.9600 -445.7421 -0.6330 -2.0705
0.8995 -1.2411 -1.9925 -0.5849 -0.1374
1.0124 -9.3415 -431.4444
29.9800 -444.6516 -1.6117 -0.6048
1.0753 -1.0587 -2.0196 -0.6394 -0.1670
0.9992 -9.3118 -431.4557
30.0000 -440.0135 0.9623 1.6546
1.0602 -0.8429 -2.0257 -0.6917 -0.1963
0.9858 -9.2821 -431.4670

30.0200 -437.1016 1.9647 2.6814
0.8261 -0.6043 -2.0113 -0.7417 -0.2253
0.9724 -9.2524 -431.4782
30.0400 -440.4238 0.7733 1.2676
0.3982 -0.3543 -1.9772 -0.7892 -0.2540
0.9588 -9.2227 -431.4894
30.0600 -445.8284 -1.7567 -1.1505 -
0.0999 -0.1027 -1.9243 -0.8342 -0.2825
0.9450 -9.1929 -431.5006
30.0800 -446.6496 -0.9654 -2.2411 -
0.5114 0.1410 -1.8536 -0.8766 -0.3105
0.9312 -9.1631 -431.5118
30.1000 -444.4279 0.5132 -1.5100 -
0.6960 0.3675 -1.7665 -0.9165 -0.3382
0.9173 -9.1333 -431.5230
30.1200 -442.4357 1.1923 -0.3038 -
0.5931 0.5683 -1.6646 -0.9538 -0.3656
0.9032 -9.1034 -431.5341
30.1400 -442.1528 0.1135 -0.0311 -
0.2309 0.7353 -1.5497 -0.9885 -0.3925
0.8890 -9.0736 -431.5453
30.1600 -442.0559 -0.5442 -0.2880
0.2350 0.8627 -1.4234 -1.0207 -0.4190
0.8747 -9.0437 -431.5564
30.1800 -441.7939 -1.2078 0.1985
0.5939 0.9468 -1.2875 -1.0502 -0.4451
0.8603 -9.0138 -431.5675
30.2000 -439.4742 -0.1350 1.5292
0.7105 0.9842 -1.1434 -1.0773 -0.4707
0.8458 -8.9838 -431.5786
30.2200 -436.8940 1.8894 2.1633
0.5596 0.9740 -0.9926 -1.1018 -0.4958
0.8311 -8.9539 -431.5897
30.2400 -438.1689 2.0593 0.8997
0.2009 0.9182 -0.8364 -1.1239 -0.5205
0.8164 -8.9239 -431.6008
30.2600 -443.7470 -1.2503 -1.3565 -
0.2002 0.8210 -0.6762 -1.1436 -0.5447
0.8016 -8.8940 -431.6119
30.2800 -446.4391 -2.2738 -2.4607 -
0.4477 0.6882 -0.5131 -1.1611 -0.5683
0.7866 -8.8640 -431.6229
30.3000 -443.2589 -0.4770 -1.2414 -
0.4267 0.5263 -0.3486 -1.1763 -0.5914
0.7716 -8.8339 -431.6340
30.3200 -439.1283 1.3260 0.7809 -
0.2030 0.3427 -0.1836 -1.1895 -0.6140
0.7564 -8.8039 -431.6450
30.3400 -438.6602 1.2636 1.7194
0.0841 0.1456 -0.0191 -1.2007 -0.6360
0.7411 -8.7739 -431.6560

30.3600 -440.0294 -0.1162 1.2927
0.2892 -0.0569 0.1441 -1.2101 -0.6574
0.7258 -8.7438 -431.6670
30.3800 -441.1913 -0.4900 0.3037
0.3416 -0.2563 0.3053 -1.2177 -0.6782
0.7103 -8.7137 -431.6780
30.4000 -441.6482 -0.1694 -0.3287
0.2272 -0.4448 0.4643 -1.2237 -0.6984
0.6948 -8.6836 -431.6889
30.4200 -441.9486 -0.3766 -0.3449
0.0037 -0.6154 0.6207 -1.2282 -0.7180
0.6792 -8.6535 -431.6999
30.4400 -442.0000 -0.1349 0.0472 -
0.2373 -0.7614 0.7743 -1.2312 -0.7370
0.6634 -8.6234 -431.7108
30.4600 -442.0000 0.0377 0.4283 -
0.4220 -0.8768 0.9246 -1.2329 -0.7554
0.6476 -8.5932 -431.7218
30.4800 -442.0000 0.1297 0.3755 -
0.4970 -0.9564 1.0717 -1.2335 -0.7730
0.6317 -8.5631 -431.7327
30.5000 -442.1571 -0.1155 -0.1629 -
0.4323 -0.9980 1.2152 -1.2328 -0.7901
0.6157 -8.5329 -431.7436
30.5200 -442.4862 -0.0581 -0.6004 -
0.2434 -1.0033 1.3547 -1.2311 -0.8064
0.5997 -8.5027 -431.7545
30.5400 -442.5193 -0.6599 -0.5152
0.0113 -0.9745 1.4894 -1.2284 -0.8221
0.5835 -8.4725 -431.7653
30.5600 -441.3701 -0.0625 -0.0649
0.2563 -0.9140 1.6185 -1.2247 -0.8370
0.5673 -8.4423 -431.7762
30.5800 -440.1968 0.8063 0.3511
0.4120 -0.8241 1.7408 -1.2200 -0.8513
0.5509 -8.4121 -431.7870
30.6000 -440.1000 -0.0406 0.5671
0.4252 -0.7060 1.8552 -1.2143 -0.8649
0.5346 -8.3819 -431.7979
30.6200 -440.1000 -0.2970 0.3807
0.2766 -0.5617 1.9601 -1.2077 -0.8777
0.5181 -8.3516 -431.8087
30.6400 -440.1000 -0.3363 0.0978 -
0.0131 -0.3922 2.0539 -1.2001 -0.8898
0.5015 -8.3214 -431.8195
30.6600 -440.1000 0.0264 -0.0316 -
0.3851 -0.1979 2.1351 -1.1914 -0.9012
0.4849 -8.2911 -431.8303
30.6800 -440.1000 0.1281 0.0167 -
0.7616 0.0208 2.2022 -1.1818 -0.9119
0.4682 -8.2609 -431.8410

30.7000 -440.1000 -0.3106 0.1061 -
1.0550 0.2622 2.2535 -1.1712 -0.9218
0.4515 -8.2306 -431.8518
30.7200 -440.1000 0.2921 0.0632 -
1.1870 0.5210 2.2873 -1.1597 -0.9310
0.4347 -8.2003 -431.8625
30.7400 -440.1000 0.3672 -0.4297 -
1.1045 0.7881 2.3021 -1.1472 -0.9395
0.4178 -8.1700 -431.8733
30.7600 -440.1000 0.4207 -1.0858 -
0.7652 1.0505 2.2961 -1.1338 -0.9473
0.4009 -8.1397 -431.8840
30.7800 -440.1000 -0.8639 -1.0774 -
0.2044 1.2940 2.2677 -1.1195 -0.9543
0.3839 -8.1094 -431.8947
30.8000 -438.9152 -1.5253 0.0256
0.4705 1.5023 2.2155 -1.1044 -0.9606
0.3668 -8.0791 -431.9054
30.8200 -434.4341 1.1838 1.5538
1.0722 1.6568 2.1385 -1.0886 -0.9662
0.3497 -8.0488 -431.9161
30.8400 -432.2623 1.5818 2.0428
1.4312 1.7405 2.0357 -1.0720 -0.9710
0.3326 -8.0185 -431.9267
30.8600 -435.3752 0.2412 0.9185
1.4927 1.7440 1.9070 -1.0547 -0.9752
0.3154 -7.9882 -431.9374
30.8800 -438.8148 -1.2325 -0.8681
1.3383 1.6656 1.7531 -1.0367 -0.9787
0.2982 -7.9579 -431.9480
30.9000 -439.2678 -0.1016 -1.9018
1.1016 1.5085 1.5755 -1.0181 -0.9815
0.2809 -7.9275 -431.9586
30.9200 -439.4628 0.2167 -1.6229
0.8661 1.2794 1.3767 -0.9988 -0.9836
0.2636 -7.8972 -431.9692
30.9400 -439.4173 -0.3783 -0.3623
0.6504 0.9896 1.1595 -0.9789 -0.9850
0.2462 -7.8669 -431.9798
30.9600 -438.6536 -0.3404 1.0895
0.3963 0.6554 0.9268 -0.9582 -0.9858
0.2288 -7.8365 -431.9904
30.9800 -438.0139 0.5991 1.8025
0.0452 0.2960 0.6817 -0.9368 -0.9860
0.2114 -7.8062 -432.0010
31.0000 -439.4190 0.3049 1.4318 -
0.4052 -0.0694 0.4273 -0.9147 -0.9855
0.1940 -7.7758 -432.0115
31.0200 -442.6399 -0.3010 0.3898 -
0.8962 -0.4229 0.1666 -0.8918 -0.9844
0.1765 -7.7455 -432.0221

31.0400 -444.5711 -0.1614 -0.7323 -
1.3110 -0.7487 -0.0973 -0.8682 -0.9828
0.1590 -7.7151 -432.0326
31.0600 -445.8415 0.2634 -1.4948 -
1.5214 -1.0320 -0.3613 -0.8437 -0.9805
0.1415 -7.6848 -432.0431
31.0800 -446.5596 -0.2273 -1.6295 -
1.4410 -1.2600 -0.6224 -0.8185 -0.9777
0.1240 -7.6545 -432.0536
31.1000 -445.9308 -0.5342 -0.9186 -
1.0893 -1.4235 -0.8777 -0.7923 -0.9743
0.1065 -7.6241 -432.0641
31.1200 -443.9310 0.2522 0.4469 -
0.5971 -1.5173 -1.1241 -0.7654 -0.9703
0.0890 -7.5938 -432.0745
31.1400 -442.7018 0.4216 1.6540 -
0.1515 -1.5398 -1.3591 -0.7375 -0.9659
0.0715 -7.5634 -432.0850
31.1600 -442.8914 -0.2673 1.9292
0.0585 -1.4905 -1.5802 -0.7088 -0.9609
0.0540 -7.5331 -432.0954
31.1800 -443.3817 0.2104 1.2087 -
0.0613 -1.3709 -1.7859 -0.6791 -0.9555
0.0365 -7.5028 -432.1059
31.2000 -444.0896 -0.2692 0.1779 -
0.4576 -1.1878 -1.9745 -0.6485 -0.9495
0.0190 -7.4724 -432.1163
31.2200 -445.7127 0.3108 -0.5831 -
0.9400 -0.9533 -2.1446 -0.6169 -0.9431
0.0015 -7.4421 -432.1267
31.2400 -447.1462 -0.2076 -1.1111 -
1.2666 -0.6830 -2.2951 -0.5845 -0.9362
-0.0160 -7.4118 -432.1371
31.2600 -446.9375 0.0254 -1.3278 -
1.2506 -0.3937 -2.4245 -0.5511 -0.9290
-0.0335 -7.3814 -432.1474
31.2800 -445.3889 -0.3779 -1.0085 -
0.8610 -0.1043 -2.5316 -0.5169 -0.9212
-0.0509 -7.3511 -432.1578
31.3000 -443.6760 0.4613 -0.2802 -
0.1922 0.1660 -2.6151 -0.4818 -0.9131
-0.0683 -7.3208 -432.1681
31.3200 -442.2311 -0.1883 0.4467
0.5812 0.3991 -2.6738 -0.4458 -0.9046
-0.0857 -7.2905 -432.1785
31.3400 -440.8566 0.2008 0.7966
1.3012 0.5795 -2.7068 -0.4089 -0.8958
-0.1030 -7.2602 -432.1888
31.3600 -440.4173 0.1054 0.6728
1.8396 0.6930 -2.7132 -0.3713 -0.8866
-0.1203 -7.2299 -432.1991

31.3800 -440.8488 -0.0110 0.1679
2.1161 0.7279 -2.6926 -0.3327 -0.8770
-0.1376 -7.1996 -432.2094
31.4000 -441.3420 0.0662 -0.2014
2.1262 0.6772 -2.6449 -0.2934 -0.8671
-0.1548 -7.1694 -432.2196
31.4200 -441.2520 -1.1864 -0.0348
1.9149 0.5404 -2.5708 -0.2534 -0.8569
-0.1720 -7.1391 -432.2299
31.4400 -440.7061 0.3345 0.3568
1.5558 0.3238 -2.4710 -0.2126 -0.8465
-0.1891 -7.1088 -432.2401
31.4600 -440.4083 1.0636 0.2520
1.1052 0.0410 -2.3470 -0.1713 -0.8357
-0.2061 -7.0786 -432.2504
31.4800 -442.5246 0.7130 -0.6241
0.5841 -0.2854 -2.2001 -0.1293 -0.8247
-0.2231 -7.0483 -432.2606
31.5000 -445.6255 -1.5619 -1.3220 -
0.0097 -0.6292 -2.0319 -0.0868 -0.8134
-0.2401 -7.0181 -432.2708
31.5200 -445.8598 -1.0877 -0.6110 -
0.6710 -0.9635 -1.8439 -0.0439 -0.8019
-0.2569 -6.9879 -432.2810
31.5400 -443.0284 0.2094 1.2680 -
1.3535 -1.2637 -1.6378 -0.0005 -0.7901
-0.2737 -6.9577 -432.2911
31.5600 -440.6138 1.7594 2.5480 -
1.9459 -1.5087 -1.4153 0.0433 -0.7782
-0.2904 -6.9275 -432.3013
31.5800 -442.2888 0.9920 1.5492 -
2.3323 -1.6813 -1.1784 0.0873 -0.7660
-0.3071 -6.8973 -432.3115
31.6000 -446.9697 -0.6622 -1.1711 -
2.4343 -1.7685 -0.9290 0.1316 -0.7536
-0.3236 -6.8671 -432.3216
31.6200 -449.2853 -1.0469 -3.5426 -
2.1977 -1.7634 -0.6693 0.1761 -0.7411
-0.3401 -6.8370 -432.3317
31.6400 -447.6511 -0.0543 -4.0459 -
1.5914 -1.6677 -0.4016 0.2206 -0.7284
-0.3565 -6.8068 -432.3418
31.6600 -444.4277 0.5899 -2.5417 -
0.6925 -1.4909 -0.1280 0.2652 -0.7156
-0.3728 -6.7767 -432.3519
31.6800 -440.9164 -0.7932 0.3115
0.3255 -1.2442 0.1493 0.3098 -0.7026
-0.3890 -6.7465 -432.3620
31.7000 -435.8117 0.2355 3.0669
1.2435 -0.9390 0.4279 0.3542 -0.6894
-0.4051 -6.7164 -432.3720

31.7200 -432.1038 1.0745 4.3824
1.8342 -0.5870 0.7057 0.3985 -0.6762
-0.4211 -6.6863 -432.3821
31.7400 -432.7650 -0.0353 3.7399
1.9212 -0.2008 0.9803 0.4425 -0.6628
-0.4369 -6.6563 -432.3921
31.7600 -435.3417 -0.3161 1.8339
1.4922 0.2058 1.2495 0.4862 -0.6493
-0.4527 -6.6262 -432.4021
31.7800 -437.4476 -0.1860 -0.2112
0.6866 0.6175 1.5110 0.5295 -0.6357
-0.4684 -6.5962 -432.4121
31.8000 -439.3214 -0.2696 -1.8995 -
0.2550 1.0177 1.7623 0.5723 -0.6220
-0.4839 -6.5661 -432.4221
31.8200 -440.2972 0.1012 -3.0177 -
1.0676 1.3886 2.0010 0.6146 -0.6083
-0.4993 -6.5361 -432.4321
31.8400 -439.8848 0.0509 -3.2692 -
1.5295 1.7131 2.2250 0.6563 -0.5945
-0.5146 -6.5061 -432.4421
31.8600 -438.7755 -0.2882 -2.3277 -
1.5161 1.9769 2.4318 0.6974 -0.5806
-0.5297 -6.4761 -432.4520
31.8800 -436.7649 -1.2072 -0.2844 -
1.0664 2.1695 2.6192 0.7377 -0.5667
-0.5448 -6.4462 -432.4620
31.9000 -432.0201 0.8103 1.6813 -
0.3130 2.2846 2.7850 0.7771 -0.5528
-0.5596 -6.4162 -432.4719
31.9200 -429.3039 2.2831 1.9458
0.5746 2.3189 2.9271 0.8157 -0.5388
-0.5744 -6.3863 -432.4818
31.9400 -432.0602 0.0418 0.4041
1.4017 2.2709 3.0437 0.8534 -0.5248
-0.5890 -6.3564 -432.4917
31.9600 -435.4985 -2.4921 -0.9536
1.9913 2.1396 3.1334 0.8900 -0.5108
-0.6034 -6.3265 -432.5016
31.9800 -433.8659 -1.7685 -0.4400
2.2186 1.9250 3.1952 0.9255 -0.4969
-0.6177 -6.2966 -432.5114
32.0000 -429.4319 1.7321 1.0864
2.0554 1.6334 3.2288 0.9598 -0.4829
-0.6319 -6.2668 -432.5213
32.0200 -429.1180 1.9511 1.7009
1.5472 1.2795 3.2340 0.9928 -0.4690
-0.6458 -6.2370 -432.5311
32.0400 -434.4927 -0.5891 0.6496
0.8002 0.8837 3.2114 1.0244 -0.4551
-0.6597 -6.2071 -432.5410

32.0600 -437.6493 -0.8929 -0.9758 -
0.0159 0.4687 3.1611 1.0547 -0.4412
-0.6733 -6.1774 -432.5508
32.0800 -438.4943 0.0053 -1.6524 -
0.7380 0.0578 3.0838 1.0834 -0.4274
-0.6868 -6.1476 -432.5606
32.1000 -438.8883 -0.3127 -0.9211 -
1.2555 -0.3246 2.9798 1.1105 -0.4137
-0.7001 -6.1179 -432.5703
32.1200 -438.4067 -0.5294 0.4851 -
1.5450 -0.6543 2.8498 1.1360 -0.4000
-0.7132 -6.0881 -432.5801
32.1400 -437.2682 -0.2132 1.3786 -
1.6237 -0.9089 2.6942 1.1599 -0.3865
-0.7262 -6.0584 -432.5899
32.1600 -437.0376 0.5635 0.9854 -
1.5197 -1.0692 2.5136 1.1822 -0.3730
-0.7389 -6.0288 -432.5996
32.1800 -438.5577 0.4159 -0.2456 -
1.2701 -1.1224 2.3088 1.2027 -0.3597
-0.7515 -5.9991 -432.6093
32.2000 -439.8698 -0.9177 -1.0360 -
0.9206 -1.0640 2.0813 1.2215 -0.3464
-0.7639 -5.9695 -432.6191
32.2200 -438.8971 -0.3924 -0.7021 -
0.5097 -0.8980 1.8326 1.2386 -0.3333
-0.7761 -5.9399 -432.6287
32.2400 -436.8093 0.7532 0.1245 -
0.0838 -0.6375 1.5651 1.2539 -0.3204
-0.7881 -5.9103 -432.6384
32.2600 -436.2635 0.3758 0.2551
0.2899 -0.3021 1.2810 1.2675 -0.3076
-0.7999 -5.8808 -432.6481
32.2800 -436.8399 0.0779 -0.1978
0.5318 0.0856 0.9830 1.2793 -0.2949
-0.8114 -5.8512 -432.6578
32.3000 -437.2957 -0.5645 -0.0803
0.5787 0.4998 0.6738 1.2892 -0.2825
-0.8228 -5.8217 -432.6674
32.3200 -436.1468 -0.6359 0.8996
0.4391 0.9120 0.3563 1.2974 -0.2702
-0.8340 -5.7922 -432.6770
32.3400 -434.0997 1.3668 1.6405
0.1991 1.2937 0.0333 1.3037 -0.2581
-0.8449 -5.7628 -432.6866
32.3600 -434.5261 1.4492 0.7632 -
0.0163 1.6181 -0.2922 1.3081 -0.2462
-0.8557 -5.7334 -432.6962
32.3800 -438.7573 -0.8861 -1.3417 -
0.1011 1.8616 -0.6176 1.3107 -0.2346
-0.8662 -5.7040 -432.7058

32.4000 -441.1827 -1.4930 -2.6302
0.0221 2.0067 -0.9401 1.3114 -0.2232
-0.8765 -5.6746 -432.7154
32.4200 -438.5708 -0.2520 -1.7685
0.3739 2.0417 -1.2575 1.3101 -0.2120
-0.8865 -5.6453 -432.7250
32.4400 -435.1360 1.5003 0.3338
0.8487 1.9623 -1.5674 1.3070 -0.2010
-0.8963 -5.6160 -432.7345
32.4600 -434.7671 0.0107 2.1034
1.2656 1.7701 -1.8677 1.3018 -0.1904
-0.9059 -5.5867 -432.7440
32.4800 -435.1274 -0.3149 2.6545
1.4489 1.4735 -2.1561 1.2948 -0.1799
-0.9153 -5.5574 -432.7536
32.5000 -435.7760 0.3410 2.0547
1.2977 1.0894 -2.4300 1.2857 -0.1698
-0.9244 -5.5282 -432.7631
32.5200 -438.3350 0.1161 0.7093
0.8525 0.6402 -2.6871 1.2747 -0.1600
-0.9333 -5.4990 -432.7725
32.5400 -441.5109 -0.2175 -0.8541
0.2593 0.1507 -2.9250 1.2617 -0.1505
-0.9419 -5.4698 -432.7820
32.5600 -443.5164 0.0569 -1.8426 -
0.3129 -0.3534 -3.1413 1.2466 -0.1412
-0.9503 -5.4407 -432.7915
32.5800 -445.1906 0.0981 -1.9909 -
0.7229 -0.8469 -3.3335 1.2296 -0.1323
-0.9584 -5.4116 -432.8009
32.6000 -445.7912 -0.1519 -1.5593 -
0.8932 -1.3061 -3.4992 1.2104 -0.1238
-0.9663 -5.3825 -432.8104
32.6200 -445.2237 -0.5247 -0.8714 -
0.8177 -1.7119 -3.6360 1.1893 -0.1155
-0.9739 -5.3534 -432.8198
32.6400 -444.3073 0.4165 0.0023 -
0.5799 -2.0517 -3.7413 1.1661 -0.1077
-0.9812 -5.3244 -432.8292
32.6600 -443.7218 0.0257 1.0452 -
0.3187 -2.3173 -3.8129 1.1408 -0.1001
-0.9883 -5.2954 -432.8386
32.6800 -443.2862 -0.3727 1.8422 -
0.1470 -2.5038 -3.8485 1.1136 -0.0930
-0.9951 -5.2665 -432.8479
32.7000 -443.0207 0.0404 1.7216 -
0.1200 -2.6111 -3.8464 1.0844 -0.0862
-1.0017 -5.2375 -432.8573
32.7200 -443.5345 0.4478 0.4781 -
0.2101 -2.6438 -3.8053 1.0533 -0.0797
-1.0079 -5.2086 -432.8666

32.7400 -445.3433 -0.1125 -1.0925 -
0.3291 -2.6094 -3.7247 1.0203 -0.0737
-1.0139 -5.1798 -432.8760
32.7600 -446.4971 -0.1444 -1.9468 -
0.3453 -2.5182 -3.6049 0.9855 -0.0680
-1.0197 -5.1510 -432.8853
32.7800 -445.7293 -0.1800 -1.7017 -
0.1643 -2.3812 -3.4469 0.9491 -0.0628
-1.0251 -5.1222 -432.8946
32.8000 -443.9718 0.5263 -0.6122
0.1907 -2.2086 -3.2524 0.9110 -0.0579
-1.0303 -5.0934 -432.9039
32.8200 -442.2864 -0.4173 0.7814
0.5972 -2.0070 -3.0240 0.8713 -0.0534
-1.0351 -5.0647 -432.9132
32.8400 -440.4112 -0.1850 1.7908
0.8905 -1.7796 -2.7644 0.8301 -0.0494
-1.0397 -5.0360 -432.9224
32.8600 -438.9146 0.3779 1.8571
0.9218 -1.5282 -2.4767 0.7874 -0.0457
-1.0440 -5.0073 -432.9317
32.8800 -439.1524 0.6906 0.8847
0.6567 -1.2535 -2.1644 0.7433 -0.0425
-1.0480 -4.9787 -432.9409
32.9000 -441.2767 -0.3254 -0.4032
0.1854 -0.9562 -1.8311 0.6979 -0.0396
-1.0518 -4.9501 -432.9501
32.9200 -442.6975 -0.5786 -1.2311 -
0.3137 -0.6376 -1.4802 0.6512 -0.0372
-1.0552 -4.9215 -432.9593
32.9400 -442.0280 -0.2870 -1.3559 -
0.6623 -0.3008 -1.1156 0.6032 -0.0353
-1.0583 -4.8930 -432.9685
32.9600 -440.5379 0.2579 -0.8312 -
0.7521 0.0492 -0.7409 0.5541 -0.0337
-1.0612 -4.8645 -432.9777
32.9800 -439.1173 -0.0721 0.0321 -
0.5994 0.4050 -0.3598 0.5039 -0.0326
-1.0637 -4.8361 -432.9869
33.0000 -437.5603 0.2428 0.6840 -
0.3073 0.7579 0.0240 0.4526 -0.0320
-1.0659 -4.8077 -432.9960
33.0200 -436.3335 -0.1295 0.8877 -
0.0167 1.0985 0.4070 0.4003 -0.0317
-1.0679 -4.7793 -433.0052
33.0400 -435.8157 0.2642 0.6478
0.1966 1.4162 0.7853 0.3471 -0.0319
-1.0695 -4.7510 -433.0143
33.0600 -435.4852 -0.2217 0.0787
0.3135 1.6997 1.1555 0.2931 -0.0326
-1.0708 -4.7227 -433.0234

33.0800 -435.2045 -0.1625 -0.4123
0.3465 1.9367 1.5141 0.2382 -0.0337
-1.0718 -4.6944 -433.0325
33.1000 -434.9984 0.6855 -0.5874
0.3234 2.1171 1.8578 0.1825 -0.0353
-1.0725 -4.6662 -433.0416
33.1200 -434.7153 -0.7860 -0.3279
0.2486 2.2339 2.1834 0.1262 -0.0373
-1.0730 -4.6380 -433.0506
33.1400 -433.6932 0.1800 0.4566
0.1263 2.2819 2.4880 0.0693 -0.0398
-1.0731 -4.6099 -433.0597
33.1600 -432.4592 0.7131 1.1479 -
0.0168 2.2585 2.7690 0.0117 -0.0428
-1.0728 -4.5818 -433.0687
33.1800 -432.8246 0.1010 0.7956 -
0.1292 2.1658 3.0243 -0.0463 -0.0462
-1.0723 -4.5537 -433.0777
33.2000 -434.7396 -0.6885 -0.4240 -
0.1601 2.0106 3.2520 -0.1048 -0.0501
-1.0715 -4.5257 -433.0868
33.2200 -435.4594 -0.6418 -1.3149 -
0.0822 1.8017 3.4508 -0.1637 -0.0545
-1.0704 -4.4977 -433.0957
33.2400 -434.7471 0.3560 -1.0671
0.0951 1.5512 3.6197 -0.2230 -0.0593
-1.0689 -4.4697 -433.1047
33.2600 -433.7834 -0.1247 -0.0118
0.3217 1.2725 3.7580 -0.2825 -0.0646
-1.0672 -4.4418 -433.1137
33.2800 -432.8956 -0.2157 1.0934
0.5233 0.9789 3.8654 -0.3423 -0.0704
-1.0651 -4.4139 -433.1227
33.3000 -432.1146 0.0481 1.4045
0.6090 0.6842 3.9417 -0.4023 -0.0767
-1.0627 -4.3861 -433.1316
33.3200 -432.7012 0.8379 0.5574
0.5294 0.4012 3.9867 -0.4623 -0.0835
-1.0600 -4.3583 -433.1405
33.3400 -435.7174 -0.2887 -0.6572
0.2855 0.1429 4.0005 -0.5225 -0.0908
-1.0571 -4.3306 -433.1494
33.3600 -436.9612 -0.7747 -0.9610 -
0.0659 -0.0783 3.9829 -0.5825 -0.0985
-1.0538 -4.3029 -433.1583
33.3800 -436.2409 0.1000 -0.2426 -
0.4394 -0.2512 3.9338 -0.6425 -0.1068
-1.0502 -4.2752 -433.1672
33.4000 -435.5327 0.3899 0.5109 -
0.7533 -0.3656 3.8533 -0.7021 -0.1155
-1.0462 -4.2476 -433.1761

33.4200 -436.1199 0.3392 0.4192 -
0.9411 -0.4151 3.7417 -0.7614 -0.1247
-1.0420 -4.2200 -433.1849
33.4400 -437.5773 -0.4106 -0.1997 -
0.9570 -0.3975 3.5995 -0.8201 -0.1344
-1.0375 -4.1924 -433.1938
33.4600 -437.8847 -0.6465 -0.5330 -
0.7785 -0.3157 3.4279 -0.8780 -0.1446
-1.0327 -4.1649 -433.2026
33.4800 -436.8756 0.2281 -0.4488 -
0.4267 -0.1783 3.2282 -0.9350 -0.1553
-1.0275 -4.1375 -433.2114
33.5000 -436.0215 0.4487 -0.3079
0.0335 0.0033 3.0022 -0.9910 -0.1664
-1.0221 -4.1101 -433.2202
33.5200 -435.9583 0.1491 -0.2438
0.4869 0.2157 2.7523 -1.0456 -0.1781
-1.0164 -4.0827 -433.2290
33.5400 -435.9317 -0.1079 -0.0355
0.7976 0.4425 2.4807 -1.0988 -0.1902
-1.0103 -4.0554 -433.2378
33.5600 -435.6611 -0.9232 0.7285
0.8637 0.6643 2.1902 -1.1503 -0.2027
-1.0040 -4.0281 -433.2465
33.5800 -434.3280 -0.0184 1.8180
0.6770 0.8610 1.8837 -1.2001 -0.2158
-0.9974 -4.0009 -433.2553
33.6000 -433.5058 1.5730 1.7905
0.3354 1.0138 1.5641 -1.2478 -0.2293
-0.9905 -3.9737 -433.2640
33.6200 -437.2643 1.1967 -0.1706 -
0.0020 1.1076 1.2343 -1.2933 -0.2432
-0.9832 -3.9465 -433.2727
33.6400 -442.4984 -2.2921 -2.3946 -
0.1925 1.1318 0.8973 -1.3366 -0.2576
-0.9757 -3.9194 -433.2814
33.6600 -442.1747 -1.5173 -2.6101 -
0.1486 1.0817 0.5559 -1.3773 -0.2725
-0.9680 -3.8923 -433.2901
33.6800 -438.1489 1.3638 -0.6831
0.0900 0.9560 0.2132 -1.4153 -0.2878
-0.9599 -3.8653 -433.2987
33.7000 -435.8018 1.2398 1.5056
0.3861 0.7585 -0.1282 -1.4504 -0.3035
-0.9515 -3.8383 -433.3074
33.7200 -436.8858 0.3688 2.1236
0.5752 0.4970 -0.4655 -1.4826 -0.3196
-0.9429 -3.8114 -433.3160
33.7400 -438.9714 -0.1964 1.1913
0.5410 0.1848 -0.7960 -1.5115 -0.3362
-0.9340 -3.7845 -433.3247

33.7600 -440.9446 -0.1772 -0.1398
0.2798 -0.1597 -1.1171 -1.5370 -0.3532
-0.9248 -3.7577 -433.3333
33.7800 -443.2397 0.1793 -1.0089 -
0.0977 -0.5150 -1.4265 -1.5591 -0.3706
-0.9153 -3.7309 -433.3419
33.8000 -444.4944 -0.3215 -1.0829 -
0.4350 -0.8575 -1.7215 -1.5774 -0.3884
-0.9055 -3.7041 -433.3505
33.8200 -444.5000 0.2093 -0.6257 -
0.6221 -1.1644 -1.9998 -1.5918 -0.4066
-0.8955 -3.6774 -433.3590
33.8400 -444.5000 0.1939 -0.0474 -
0.6113 -1.4151 -2.2588 -1.6022 -0.4251
-0.8852 -3.6508 -433.3676
33.8600 -444.5000 0.1603 0.2553 -
0.4455 -1.5926 -2.4961 -1.6084 -0.4441
-0.8747 -3.6242 -433.3761
33.8800 -444.5000 -0.4179 0.2247 -
0.2361 -1.6843 -2.7095 -1.6103 -0.4634
-0.8639 -3.5976 -433.3847
33.9000 -444.5000 0.0507 0.0318 -
0.0907 -1.6842 -2.8970 -1.6076 -0.4830
-0.8528 -3.5711 -433.3932
33.9200 -444.5000 0.3589 -0.0085 -
0.0865 -1.5931 -3.0568 -1.6002 -0.5030
-0.8414 -3.5446 -433.4017
33.9400 -444.5000 -0.0288 0.2034 -
0.2370 -1.4193 -3.1881 -1.5880 -0.5233
-0.8298 -3.5182 -433.4102
33.9600 -444.5000 0.5282 0.3062 -
0.4835 -1.1776 -3.2909 -1.5708 -0.5439
-0.8180 -3.4918 -433.4186
33.9800 -444.5000 -0.3067 0.1638 -
0.7257 -0.8877 -3.3656 -1.5485 -0.5648
-0.8059 -3.4654 -433.4271
34.0000 -444.5000 -0.1935 -0.0267 -
0.8727 -0.5727 -3.4128 -1.5210 -0.5859
-0.7936 -3.4391 -433.4356
34.0200 -444.5000 0.2629 -0.2876 -
0.8385 -0.2578 -3.4332 -1.4884 -0.6074
-0.7810 -3.4129 -433.4440
34.0400 -444.1287 -0.0652 -0.6360 -
0.5655 0.0324 -3.4273 -1.4506 -0.6290
-0.7681 -3.3867 -433.4524
34.0600 -443.1888 0.0341 -0.5718 -
0.0690 0.2757 -3.3960 -1.4078 -0.6509
-0.7551 -3.3606 -433.4608
34.0800 -442.0998 -0.1403 0.1119
0.5716 0.4531 -3.3399 -1.3600 -0.6730
-0.7418 -3.3345 -433.4692

34.1000 -440.3701 -0.1169 0.7806
1.2153 0.5485 -3.2598 -1.3075 -0.6953
-0.7282 -3.3084 -433.4776
34.1200 -438.7722 0.1883 0.9508
1.7052 0.5521 -3.1568 -1.2503 -0.7177
-0.7145 -3.2824 -433.4859
34.1400 -438.8630 0.8913 0.4036
1.9136 0.4608 -3.0321 -1.1887 -0.7403
-0.7005 -3.2564 -433.4943
34.1600 -440.7849 -0.0053 -0.5009
1.7780 0.2838 -2.8871 -1.1229 -0.7630
-0.6863 -3.2305 -433.5026
34.1800 -442.0976 -0.8841 -0.7448
1.3206 0.0410 -2.7236 -1.0531 -0.7858
-0.6718 -3.2047 -433.5109
34.2000 -441.8336 0.3332 -0.0588
0.6248 -0.2417 -2.5435 -0.9796 -0.8087
-0.6572 -3.1789 -433.5192
34.2200 -441.3560 0.0858 0.7245 -
0.1771 -0.5351 -2.3488 -0.9026 -0.8317
-0.6423 -3.1531 -433.5275
34.2400 -441.3261 0.8129 0.7594 -
0.9386 -0.8093 -2.1418 -0.8224 -0.8548
-0.6272 -3.1274 -433.5358
34.2600 -442.9879 -0.3749 0.0849 -
1.5478 -1.0355 -1.9245 -0.7390 -0.8778
-0.6120 -3.1017 -433.5440
34.2800 -444.7207 -0.1822 -0.6203 -
1.9025 -1.1881 -1.6991 -0.6529 -0.9009
-0.5965 -3.0761 -433.5523
34.3000 -444.6481 -0.2113 -0.9384 -
1.9147 -1.2486 -1.4675 -0.5641 -0.9240
-0.5808 -3.0505 -433.5605
34.3200 -443.5370 -0.0036 -0.9011 -
1.5589 -1.2137 -1.2319 -0.4731 -0.9471
-0.5649 -3.0250 -433.5687
34.3400 -442.0692 0.4462 -0.6108 -
0.9044 -1.0927 -0.9941 -0.3799 -0.9701
-0.5488 -2.9995 -433.5769
34.3600 -440.0794 -0.2243 -0.1047 -
0.1118 -0.9017 -0.7561 -0.2849 -0.9930
-0.5326 -2.9741 -433.5851
34.3800 -437.6365 0.2182 0.5830
0.6071 -0.6587 -0.5199 -0.1882 -1.0159
-0.5161 -2.9487 -433.5933
34.4000 -436.3504 0.2096 1.2162
1.0752 -0.3817 -0.2874 -0.0902 -1.0387
-0.4995 -2.9234 -433.6015
34.4200 -435.7993 -0.4612 1.3264
1.2123 -0.0910 -0.0605 0.0090 -1.0613
-0.4827 -2.8981 -433.6096

34.4400 -435.5141 0.0774 0.5971
1.0705 0.1914 0.1589 0.1090 -1.0838
-0.4657 -2.8729 -433.6177
34.4600 -436.2076 0.6807 -0.8622
0.7880 0.4434 0.3689 0.2097 -1.1061
-0.4485 -2.8478 -433.6259
34.4800 -438.0656 -0.3599 -1.8977
0.5127 0.6477 0.5677 0.3109 -1.1283
-0.4312 -2.8226 -433.6340
34.5000 -438.4194 -0.9921 -1.3460
0.3478 0.7954 0.7536 0.4121 -1.1503
-0.4137 -2.7976 -433.6421
34.5200 -435.0020 -0.6796 0.4183
0.2961 0.8854 0.9253 0.5133 -1.1720
-0.3960 -2.7725 -433.6501
34.5400 -431.7687 1.6010 1.6478
0.2909 0.9222 1.0818 0.6142 -1.1935
-0.3782 -2.7476 -433.6582
34.5600 -433.0820 0.5934 1.0414
0.2580 0.9136 1.2225 0.7145 -1.2148
-0.3602 -2.7227 -433.6662
34.5800 -436.5674 -1.2803 -0.6193
0.1696 0.8694 1.3468 0.8139 -1.2357
-0.3421 -2.6978 -433.6743
34.6000 -437.1081 -0.4294 -1.5304
0.0235 0.7992 1.4547 0.9123 -1.2564
-0.3238 -2.6730 -433.6823
34.6200 -435.2397 0.3505 -1.0038 -
0.1704 0.7123 1.5465 1.0093 -1.2768
-0.3053 -2.6482 -433.6903
34.6400 -433.8057 -0.0662 0.4102 -
0.3698 0.6157 1.6223 1.1047 -1.2968
-0.2868 -2.6235 -433.6983
34.6600 -433.5507 0.2544 1.6780 -
0.5459 0.5140 1.6830 1.1981 -1.3165
-0.2680 -2.5988 -433.7063
34.6800 -433.4136 0.6362 1.6802 -
0.6667 0.4098 1.7293 1.2893 -1.3358
-0.2492 -2.5742 -433.7142
34.7000 -434.1521 0.7755 0.1538 -
0.6839 0.3048 1.7625 1.3781 -1.3548
-0.2302 -2.5497 -433.7222
34.7200 -437.0106 -0.4865 -1.7281 -
0.5357 0.2002 1.7840 1.4639 -1.3733
-0.2110 -2.5252 -433.7301
34.7400 -438.1776 -1.4800 -2.2796 -
0.1951 0.0957 1.7956 1.5467 -1.3914
-0.1918 -2.5007 -433.7380
34.7600 -434.9522 -0.1763 -0.9973
0.2864 -0.0090 1.7990 1.6260 -1.4091
-0.1724 -2.4763 -433.7459

34.7800 -431.5886 1.7300 0.9136
0.7504 -0.1134 1.7960 1.7016 -1.4263
-0.1529 -2.4520 -433.7538
34.8000 -431.4332 -0.0471 2.1779
1.0012 -0.2149 1.7882 1.7731 -1.4430
-0.1333 -2.4277 -433.7617
34.8200 -431.5711 -0.3593 2.0920
0.9043 -0.3095 1.7768 1.8404 -1.4593
-0.1135 -2.4035 -433.7696
34.8400 -432.2226 0.7756 0.7394
0.4731 -0.3910 1.7630 1.9030 -1.4750
-0.0937 -2.3793 -433.7774
34.8600 -435.5349 -0.1022 -0.8766 -
0.1442 -0.4531 1.7476 1.9608 -1.4903
-0.0737 -2.3551 -433.7853
34.8800 -437.5986 -0.6409 -1.6740 -
0.7376 -0.4903 1.7313 2.0136 -1.5049
-0.0536 -2.3311 -433.7931
34.9000 -437.0281 0.2155 -1.4861 -
1.0863 -0.4980 1.7143 2.0611 -1.5191
-0.0335 -2.3070 -433.8009
34.9200 -435.9728 0.0974 -0.9005 -
1.0603 -0.4739 1.6968 2.1033 -1.5326
-0.0132 -2.2831 -433.8087
34.9400 -435.1186 0.5263 -0.6628 -
0.6715 -0.4206 1.6788 2.1401 -1.5456
0.0072 -2.2591 -433.8165
34.9600 -434.3261 -0.2204 -0.6848 -
0.0447 -0.3445 1.6602 2.1712 -1.5579
0.0276 -2.2353 -433.8242
34.9800 -433.2848 -0.2806 -0.1909
0.6118 -0.2519 1.6408 2.1966 -1.5697
0.0481 -2.2115 -433.8320
35.0000 -431.2044 -0.2047 1.0553
1.0655 -0.1489 1.6204 2.2163 -1.5808
0.0688 -2.1877 -433.8397
35.0200 -429.5631 0.3789 2.2461
1.1622 -0.0417 1.5987 2.2301 -1.5913
0.0895 -2.1640 -433.8474
35.0400 -430.3115 0.3853 2.3849
0.8587 0.0649 1.5755 2.2380 -1.6011
0.1102 -2.1404 -433.8551
35.0600 -432.5246 -0.3575 1.2923
0.2455 0.1677 1.5504 2.2401 -1.6103
0.1311 -2.1168 -433.8628
35.0800 -434.6437 0.2891 -0.4546 -
0.4799 0.2632 1.5233 2.2362 -1.6188
0.1520 -2.0932 -433.8705
35.1000 -436.9876 -0.1108 -2.0884 -
1.0653 0.3472 1.4937 2.2264 -1.6266
0.1730 -2.0698 -433.8782

35.1200 -437.8648 -0.9493 -2.9616 -
1.2946 0.4158 1.4612 2.2108 -1.6337
0.1940 -2.0463 -433.8858
35.1400 -436.5032 0.0623 -2.6448 -
1.1033 0.4672 1.4255 2.1893 -1.6401
0.2151 -2.0230 -433.8934
35.1600 -434.3417 0.6041 -1.2256 -
0.5616 0.4995 1.3862 2.1622 -1.6458
0.2363 -1.9997 -433.9011
35.1800 -432.4345 -0.6107 0.6813
0.1500 0.5124 1.3431 2.1295 -1.6507
0.2575 -1.9764 -433.9087
35.2000 -430.4868 0.2720 2.2541
0.8069 0.5054 1.2962 2.0913 -1.6550
0.2787 -1.9532 -433.9163
35.2200 -429.9671 0.0295 2.8099
1.2087 0.4781 1.2453 2.0477 -1.6585
0.3000 -1.9301 -433.9238
35.2400 -431.0012 -0.5844 2.1882
1.2306 0.4307 1.1908 1.9990 -1.6613
0.3214 -1.9070 -433.9314
35.2600 -432.4867 0.2588 0.8177
0.8663 0.3654 1.1329 1.9452 -1.6633
0.3427 -1.8839 -433.9390
35.2800 -434.4059 0.2682 -0.6911
0.2570 0.2874 1.0721 1.8865 -1.6646
0.3641 -1.8610 -433.9465
35.3000 -436.2278 -0.3072 -1.7436 -
0.3926 0.2027 1.0088 1.8232 -1.6651
0.3856 -1.8380 -433.9540
35.3200 -436.6710 -0.0438 -2.0167 -
0.8740 0.1176 0.9433 1.7555 -1.6648
0.4070 -1.8152 -433.9615
35.3400 -436.8509 -0.1033 -1.7593 -
1.0081 0.0384 0.8763 1.6835 -1.6638
0.4285 -1.7924 -433.9690
35.3600 -436.8925 0.4769 -1.6128 -
0.7173 -0.0286 0.8083 1.6074 -1.6621
0.4500 -1.7696 -433.9765
35.3800 -436.6176 -0.3345 -1.7283 -
0.1146 -0.0793 0.7396 1.5275 -1.6595
0.4715 -1.7469 -433.9840
35.4000 -436.0222 -0.4680 -1.0505
0.5537 -0.1106 0.6707 1.4440 -1.6562
0.4930 -1.7243 -433.9914
35.4200 -433.9421 -1.0749 1.0152
1.0476 -0.1207 0.6020 1.3571 -1.6521
0.5146 -1.7017 -433.9989
35.4400 -429.6255 0.7506 3.2357
1.1873 -0.1089 0.5337 1.2670 -1.6473
0.5361 -1.6792 -434.0063

35.4600 -428.6628 1.5652 3.5692
0.8701 -0.0758 0.4659 1.1739 -1.6416
0.5576 -1.6568 -434.0137
35.4800 -434.0487 -0.0808 1.5772
0.1715 -0.0263 0.3990 1.0781 -1.6352
0.5792 -1.6344 -434.0211
35.5000 -438.5596 -1.5537 -1.0891 -
0.6591 0.0331 0.3332 0.9797 -1.6280
0.6007 -1.6120 -434.0285
35.5200 -439.1415 -0.0080 -2.7842 -
1.3396 0.0956 0.2686 0.8790 -1.6200
0.6222 -1.5897 -434.0358
35.5400 -439.4552 0.0937 -2.9378 -
1.6274 0.1538 0.2054 0.7761 -1.6112
0.6437 -1.5675 -434.0432
35.5600 -439.0854 -0.4231 -1.7259 -
1.4064 0.2001 0.1437 0.6713 -1.6017
0.6652 -1.5453 -434.0505
35.5800 -436.5858 0.0708 0.0334 -
0.7497 0.2282 0.0834 0.5648 -1.5913
0.6867 -1.5232 -434.0578
35.6000 -434.3735 0.6291 1.0435
0.1629 0.2342 0.0245 0.4567 -1.5802
0.7081 -1.5012 -434.0651
35.6200 -433.6343 -0.3737 1.1905
1.0886 0.2155 -0.0330 0.3473 -1.5682
0.7295 -1.4792 -434.0724
35.6400 -433.1707 -0.1326 1.2049
1.7604 0.1713 -0.0891 0.2368 -1.5555
0.7509 -1.4573 -434.0797
35.6600 -433.2001 -0.5496 1.3721
2.0031 0.1048 -0.1437 0.1253 -1.5420
0.7722 -1.4354 -434.0870
35.6800 -433.8942 0.0371 1.2101
1.7777 0.0228 -0.1966 0.0130 -1.5277
0.7935 -1.4136 -434.0942
35.7000 -434.9277 1.0794 0.2270
1.1759 -0.0652 -0.2475 -0.0998 -1.5126
0.8148 -1.3918 -434.1015
35.7200 -437.2279 -0.6644 -1.1426
0.3638 -0.1495 -0.2964 -0.2131 -1.4967
0.8360 -1.3701 -434.1087
35.7400 -439.4116 -0.5242 -1.5523 -
0.4843 -0.2197 -0.3428 -0.3265 -1.4800
0.8571 -1.3485 -434.1159
35.7600 -439.0896 -0.5361 -0.3917 -
1.2215 -0.2658 -0.3862 -0.4399 -1.4626
0.8782 -1.3269 -434.1231
35.7800 -437.4591 0.2611 1.0632 -
1.7700 -0.2792 -0.4264 -0.5532 -1.4443
0.8993 -1.3054 -434.1303

35.8000 -436.6196 2.0570 1.2971 -
2.0873 -0.2548 -0.4627 -0.6662 -1.4253
0.9202 -1.2840 -434.1374
35.8200 -439.1522 0.1698 0.0583 -
2.1577 -0.1931 -0.4949 -0.7787 -1.4054
0.9411 -1.2626 -434.1446
35.8400 -442.5182 -1.1090 -1.3734 -
1.9664 -0.1004 -0.5224 -0.8904 -1.3848
0.9620 -1.2412 -434.1517
35.8600 -442.1043 -1.5365 -1.5011 -
1.5060 0.0130 -0.5450 -1.0014 -1.3634
0.9828 -1.2200 -434.1589
35.8800 -438.2413 0.7529 -0.2214 -
0.8173 0.1331 -0.5624 -1.1113 -1.3412
1.0035 -1.1988 -434.1660
35.9000 -435.3646 0.9303 0.9341
0.0144 0.2446 -0.5744 -1.2201 -1.3182
1.0241 -1.1776 -434.1731
35.9200 -435.3566 -0.0765 1.0346
0.8653 0.3318 -0.5806 -1.3275 -1.2944
1.0446 -1.1565 -434.1801
35.9400 -435.5070 -0.5436 0.3874
1.5982 0.3805 -0.5810 -1.4335 -1.2699
1.0650 -1.1355 -434.1872
35.9600 -435.6672 0.6739 -0.2306
2.1154 0.3775 -0.5753 -1.5378 -1.2445
1.0854 -1.1145 -434.1943
35.9800 -435.8772 -0.1855 -0.4261
2.3528 0.3127 -0.5634 -1.6404 -1.2183
1.1056 -1.0936 -434.2013
36.0000 -435.9995 -0.1080 -0.2566
2.2632 0.1856 -0.5453 -1.7411 -1.1914
1.1258 -1.0728 -434.2083
36.0200 -435.9355 -0.2907 0.3263
1.8437 0.0058 -0.5209 -1.8397 -1.1636
1.1458 -1.0520 -434.2153
36.0400 -435.8239 0.1383 0.9675
1.1503 -0.2099 -0.4902 -1.9361 -1.1351
1.1658 -1.0313 -434.2223
36.0600 -436.3642 0.9234 0.9027
0.2923 -0.4417 -0.4533 -2.0301 -1.1058
1.1856 -1.0107 -434.2293
36.0800 -439.6834 -0.0348 -0.1327 -
0.5832 -0.6680 -0.4106 -2.1217 -1.0756
1.2053 -0.9901 -434.2363
36.1000 -441.9953 -1.4230 -1.1744 -
1.3188 -0.8665 -0.3621 -2.2106 -1.0447
1.2249 -0.9696 -434.2432
36.1200 -441.4229 0.4308 -1.1940 -
1.7737 -1.0157 -0.3083 -2.2968 -1.0130
1.2444 -0.9491 -434.2502

36.1400 -440.2735 0.3608 -0.3732 -
1.8803 -1.0971 -0.2496 -2.3801 -0.9804
1.2637 -0.9287 -434.2571
36.1600 -439.5606 0.3002 0.3552 -
1.6830 -1.0999 -0.1868 -2.4604 -0.9470
1.2829 -0.9084 -434.2640
36.1800 -439.2236 -0.2671 0.6013 -
1.2829 -1.0240 -0.1205 -2.5376 -0.9129
1.3020 -0.8881 -434.2709
36.2000 -438.9187 -0.1811 0.5517 -
0.7990 -0.8748 -0.0519 -2.6116 -0.8779
1.3210 -0.8679 -434.2778
36.2200 -438.5158 -0.0204 0.2228 -
0.3191 -0.6658 0.0176 -2.6823 -0.8421
1.3398 -0.8477 -434.2846
36.2400 -438.0445 -0.2752 -0.4400
0.1151 -0.4143 0.0866 -2.7496 -0.8055
1.3585 -0.8276 -434.2915
36.2600 -437.4150 -0.0558 -0.7457
0.4938 -0.1403 0.1537 -2.8133 -0.7680
1.3770 -0.8076 -434.2983
36.2800 -436.0679 -0.4647 -0.3028
0.7989 0.1365 0.2174 -2.8734 -0.7298
1.3953 -0.7877 -434.3051
36.3000 -434.7687 1.0769 0.3358
1.0002 0.3975 0.2765 -2.9297 -0.6907
1.4135 -0.7678 -434.3119
36.3200 -434.6000 -0.3822 0.5285
1.0478 0.6283 0.3296 -2.9821 -0.6508
1.4316 -0.7479 -434.3187
36.3400 -434.6000 -0.1638 0.3563
0.9158 0.8223 0.3751 -3.0306 -0.6100
1.4495 -0.7282 -434.3255
36.3600 -434.6111 -0.0687 0.1862
0.6196 0.9767 0.4116 -3.0750 -0.5685
1.4672 -0.7085 -434.3323
36.3800 -435.1642 0.2444 0.0745
0.2252 1.0920 0.4378 -3.1152 -0.5261
1.4847 -0.6889 -434.3390
36.4000 -435.9456 -0.0728 -0.0347 -
0.1641 1.1701 0.4527 -3.1513 -0.4829
1.5021 -0.6693 -434.3458
36.4200 -436.3739 -0.0893 -0.2392 -
0.4431 1.2139 0.4555 -3.1830 -0.4389
1.5193 -0.6498 -434.3525
36.4400 -436.7002 0.0011 -0.5206 -
0.5443 1.2269 0.4456 -3.2104 -0.3940
1.5363 -0.6303 -434.3592
36.4600 -436.7889 -0.3833 -0.7440 -
0.4723 1.2111 0.4225 -3.2333 -0.3484
1.5532 -0.6110 -434.3659

36.4800 -436.5104 0.7054 -0.6701 -
0.2868 1.1678 0.3863 -3.2518 -0.3020
1.5698 -0.5917 -434.3726
36.5000 -436.0351 -0.5800 -0.0884 -
0.0736 1.0971 0.3370 -3.2657 -0.2548
1.5863 -0.5724 -434.3792
36.5200 -435.3785 0.2077 0.7868
0.0914 0.9986 0.2759 -3.2749 -0.2068
1.6025 -0.5532 -434.3859
36.5400 -434.4994 -0.3383 1.2363
0.1577 0.8703 0.2044 -3.2794 -0.1581
1.6186 -0.5341 -434.3925
36.5600 -434.3175 0.8927 0.7671
0.1316 0.7074 0.1241 -3.2790 -0.1088
1.6345 -0.5151 -434.3992
36.5800 -436.2835 -0.2710 -0.3160
0.0923 0.5041 0.0369 -3.2737 -0.0587
1.6501 -0.4961 -434.4058
36.6000 -438.2057 -0.5475 -1.1963
0.1406 0.2566 -0.0555 -3.2635 -0.0080
1.6655 -0.4772 -434.4124
36.6200 -437.8763 0.0828 -1.2392
0.3431 -0.0356 -0.1514 -3.2482 0.0434
1.6808 -0.4584 -434.4189
36.6400 -436.7260 0.3647 -0.4869
0.6882 -0.3646 -0.2489 -3.2279 0.0954
1.6958 -0.4396 -434.4255
36.6600 -435.9751 -0.3068 0.4832
1.0752 -0.7138 -0.3465 -3.2024 0.1479
1.7106 -0.4209 -434.4321
36.6800 -435.5479 -0.1033 1.1652
1.3438 -1.0627 -0.4425 -3.1719 0.2010
1.7251 -0.4022 -434.4386
36.7000 -435.3105 0.5084 1.3228
1.3542 -1.3906 -0.5356 -3.1362 0.2545
1.7395 -0.3836 -434.4451
36.7200 -436.1484 0.9110 0.7533
1.0238 -1.6769 -0.6244 -3.0953 0.3086
1.7536 -0.3651 -434.4516
36.7400 -438.4488 -0.4291 -0.2852
0.3792 -1.9020 -0.7076 -3.0493 0.3631
1.7675 -0.3467 -434.4581
36.7600 -439.7761 -0.5387 -1.0196 -
0.4448 -2.0486 -0.7838 -2.9982 0.4179
1.7811 -0.3283 -434.4646
36.7800 -440.3550 0.1329 -0.9754 -
1.2952 -2.1006 -0.8519 -2.9419 0.4732
1.7945 -0.3100 -434.4711
36.8000 -440.6759 -0.3473 -0.4723 -
2.0140 -2.0451 -0.9111 -2.8806 0.5288
1.8076 -0.2918 -434.4775

36.8200 -440.5081 -0.0831 0.0905 -
2.4662 -1.8729 -0.9610 -2.8142 0.5846
1.8205 -0.2736 -434.4840
36.8400 -439.7457 0.1326 0.4530 -
2.5704 -1.5842 -1.0016 -2.7429 0.6408
1.8332 -0.2555 -434.4904
36.8600 -438.8788 0.0645 0.4644 -
2.2899 -1.1941 -1.0336 -2.6668 0.6971
1.8456 -0.2374 -434.4968
36.8800 -438.1767 0.5253 -0.0698 -
1.6193 -0.7271 -1.0577 -2.5858 0.7536
1.8577 -0.2195 -434.5032
36.9000 -437.3487 -0.1021 -0.9248 -
0.6077 -0.2110 -1.0743 -2.5001 0.8103
1.8696 -0.2016 -434.5096
36.9200 -435.8236 -0.5585 -1.1950
0.6135 0.3242 -1.0841 -2.4098 0.8670
1.8812 -0.1837 -434.5159
36.9400 -432.4970 -0.2134 -0.3368
1.8242 0.8472 -1.0877 -2.3150 0.9238
1.8925 -0.1660 -434.5223
36.9600 -429.6691 0.3884 1.1237
2.7446 1.3264 -1.0859 -2.2159 0.9807
1.9036 -0.1483 -434.5286
36.9800 -428.2633 -0.1617 2.1737
3.1223 1.7313 -1.0791 -2.1125 1.0375
1.9143 -0.1306 -434.5349
37.0000 -427.3789 0.0519 2.0823
2.8197 2.0385 -1.0682 -2.0051 1.0943
1.9248 -0.1131 -434.5412
37.0200 -428.8975 1.2055 0.7840
1.9009 2.2406 -1.0535 -1.8937 1.1511
1.9350 -0.0956 -434.5475
37.0400 -433.8205 -1.1880 -0.9030
0.6262 2.3408 -1.0353 -1.7786 1.2076
1.9449 -0.0782 -434.5538
37.0600 -435.0836 -0.9038 -1.7635 -
0.6784 2.3457 -1.0140 -1.6599 1.2641
1.9546 -0.0608 -434.5601
37.0800 -434.9750 0.3069 -1.5107 -
1.7236 2.2631 -0.9898 -1.5379 1.3203
1.9639 -0.0435 -434.5663
37.1000 -434.9000 0.0375 -1.1143 -
2.2831 2.1026 -0.9630 -1.4126 1.3763
1.9729 -0.0263 -434.5726
37.1200 -434.9352 0.6652 -1.1539 -
2.2510 1.8752 -0.9338 -1.2843 1.4320
1.9817 -0.0092 -434.5788
37.1400 -434.9903 0.0912 -0.9936 -
1.7308 1.5926 -0.9021 -1.1533 1.4874
1.9901 0.0079 -434.5850

37.1600 -434.0510 -1.6096 0.1231 -
0.9484 1.2656 -0.8672 -1.0199 1.5424
1.9982 0.0249 -434.5912
37.1800 -429.6234 0.6273 1.6321 -
0.1380 0.9053 -0.8286 -0.8842 1.5970
2.0060 0.0418 -434.5974
37.2000 -427.3592 2.5665 1.9008
0.5053 0.5225 -0.7852 -0.7466 1.6512
2.0134 0.0587 -434.6035
37.2200 -430.7078 -0.1492 0.3231
0.8786 0.1263 -0.7356 -0.6074 1.7050
2.0206 0.0755 -434.6097
37.2400 -434.2696 -1.9792 -1.2296
1.0109 -0.2766 -0.6784 -0.4670 1.7582
2.0274 0.0922 -434.6158
37.2600 -432.7533 -1.2170 -0.8968
0.9735 -0.6813 -0.6124 -0.3254 1.8109
2.0339 0.1089 -434.6219
37.2800 -429.2987 1.7841 0.4865
0.8517 -1.0850 -0.5363 -0.1832 1.8630
2.0401 0.1255 -434.6281
37.3000 -429.3509 1.6433 0.7168
0.7258 -1.4832 -0.4494 -0.0406 1.9144
2.0459 0.1420 -434.6342
37.3200 -433.1501 -0.9975 -0.2890
0.6133 -1.8635 -0.3515 0.1021 1.9652
2.0514 0.1584 -434.6402
37.3400 -435.0283 -2.2496 -0.6495
0.4502 -2.2077 -0.2428 0.2447 2.0153
2.0566 0.1748 -434.6463
37.3600 -431.9664 0.1639 0.3308
0.1849 -2.4967 -0.1236 0.3867 2.0647
2.0614 0.1911 -434.6523
37.3800 -428.7354 2.6223 1.0405 -
0.1764 -2.7119 0.0054 0.5281 2.1133
2.0658 0.2074 -434.6584
37.4000 -430.7316 1.4068 0.2432 -
0.5873 -2.8368 0.1438 0.6683 2.1610
2.0699 0.2235 -434.6644
37.4200 -435.4254 -1.5172 -1.0816 -
0.9580 -2.8588 0.2907 0.8073 2.2079
2.0736 0.2396 -434.6704
37.4400 -435.7748 -2.1091 -1.2445 -
1.1854 -2.7684 0.4450 0.9446 2.2539
2.0770 0.2556 -434.6764
37.4600 -431.4539 1.3972 -0.1611 -
1.2034 -2.5617 0.6051 1.0800 2.2990
2.0800 0.2716 -434.6824
37.4800 -428.7007 1.4110 0.6085 -
1.0193 -2.2454 0.7686 1.2133 2.3431
2.0827 0.2875 -434.6883

37.5000 -429.5496 0.5699 0.0818 -
0.6780 -1.8344 0.9328 1.3440 2.3862
2.0849 0.3033 -434.6943
37.5200 -430.6309 -1.3680 -0.5966 -
0.2608 -1.3465 1.0949 1.4720 2.4282
2.0868 0.3190 -434.7002
37.5400 -428.9666 -0.7871 -0.2064
0.1356 -0.8017 1.2520 1.5970 2.4691
2.0883 0.3347 -434.7062
37.5600 -424.7555 1.3257 0.7404
0.4221 -0.2208 1.4013 1.7187 2.5089
2.0895 0.3503 -434.7121
37.5800 -423.8725 0.2054 1.1952
0.5475 0.3745 1.5398 1.8367 2.5476
2.0902 0.3658 -434.7180
37.6000 -424.3127 -0.0842 0.6010
0.5080 0.9617 1.6646 1.9510 2.5850
2.0906 0.3813 -434.7238
37.6200 -424.7162 0.4077 -0.6476
0.3376 1.5166 1.7728 2.0611 2.6212
2.0905 0.3966 -434.7297
37.6400 -425.0730 -0.8906 -1.2133
0.0877 2.0147 1.8614 2.1668 2.6560
2.0901 0.4120 -434.7355
37.6600 -424.9946 -1.5028 -0.2866 -
0.1525 2.4323 1.9277 2.2678 2.6896
2.0892 0.4272 -434.7414
37.6800 -420.9003 0.4668 1.1358 -
0.2560 2.7470 1.9688 2.3639 2.7217
2.0880 0.4424 -434.7472
37.7000 -418.3950 2.9821 1.1570 -
0.0937 2.9408 1.9822 2.4550 2.7525
2.0863 0.4575 -434.7530
37.7200 -422.9416 -0.6716 -0.7261
0.3740 3.0056 1.9659 2.5406 2.7818
2.0842 0.4725 -434.7588
37.7400 -425.9252 -2.8172 -2.4112
1.0810 2.9428 1.9190 2.6209 2.8096
2.0817 0.4875 -434.7646
37.7600 -422.5964 -0.7308 -1.9168
1.9014 2.7587 1.8421 2.6955 2.8358
2.0788 0.5024 -434.7703
37.7800 -418.7228 1.1057 0.3340
2.6357 2.4631 1.7372 2.7645 2.8605
2.0755 0.5172 -434.7761
37.8000 -416.4373 0.4372 2.6064
3.0391 2.0692 1.6070 2.8279 2.8835
2.0717 0.5319 -434.7818
37.8200 -415.4237 1.6021 3.3648
2.8815 1.5951 1.4545 2.8858 2.9049
2.0675 0.5466 -434.7875

37.8400 -419.6222 -0.4993 2.1441
2.0806 1.0657 1.2823 2.9382 2.9246
2.0628 0.5612 -434.7932
37.8600 -424.9216 -0.5076 -0.2530
0.7930 0.5101 1.0935 2.9852 2.9425
2.0578 0.5758 -434.7989
37.8800 -428.6867 -0.6843 -2.0408 -
0.7189 -0.0426 0.8908 3.0270 2.9587
2.0523 0.5903 -434.8046
37.9000 -430.6182 -0.3004 -2.1185 -
2.1704 -0.5631 0.6771 3.0636 2.9730
2.0463 0.6047 -434.8103
37.9200 -430.7961 -0.1793 -1.1657 -
3.2588 -1.0226 0.4552 3.0952 2.9855
2.0399 0.6190 -434.8159
37.9400 -430.9317 0.8926 -0.7211 -
3.7067 -1.3937 0.2281 3.1219 2.9961
2.0330 0.6333 -434.8216
37.9600 -432.0100 1.1250 -1.7300 -
3.3779 -1.6576 -0.0013 3.1438 3.0048
2.0257 0.6475 -434.8272
37.9800 -434.2084 -0.7428 -3.1039 -
2.3740 -1.8153 -0.2301 3.1611 3.0116
2.0180 0.6617 -434.8328
38.0000 -434.0547 -1.9814 -3.1029 -
0.9308 -1.8792 -0.4552 3.1738 3.0163
2.0098 0.6757 -434.8384
38.0200 -428.1849 0.5978 -0.9836
0.6481 -1.8644 -0.6734 3.1820 3.0191
2.0011 0.6897 -434.8440
38.0400 -423.2865 0.9423 2.1139
2.0295 -1.7863 -0.8817 3.1859 3.0199
1.9920 0.7037 -434.8495
38.0600 -421.4201 -0.0964 4.3209
2.8937 -1.6608 -1.0772 3.1856 3.0186
1.9825 0.7176 -434.8551
38.0800 -420.5087 1.2388 4.2089
3.0122 -1.5039 -1.2570 3.1812 3.0153
1.9725 0.7314 -434.8606
38.1000 -424.2581 0.5651 1.9220
2.4157 -1.3286 -1.4186 3.1727 3.0099
1.9620 0.7451 -434.8661
38.1200 -430.0408 -1.7966 -0.7785
1.3414 -1.1418 -1.5601 3.1603 3.0024
1.9511 0.7588 -434.8716
38.1400 -430.7122 -0.2706 -2.2264
0.0822 -0.9495 -1.6799 3.1440 2.9928
1.9398 0.7724 -434.8771
38.1600 -430.9363 0.5862 -2.4242 -
1.0364 -0.7572 -1.7768 3.1240 2.9812
1.9281 0.7860 -434.8826

38.1800 -431.5878 1.2640 -2.4053 -
1.7293 -0.5710 -1.8497 3.1002 2.9675
1.9159 0.7995 -434.8880
38.2000 -432.8918 -0.5433 -2.2397 -
1.8997 -0.3970 -1.8983 3.0728 2.9517
1.9032 0.8129 -434.8935
38.2200 -433.1199 -2.5069 -0.9011 -
1.6117 -0.2398 -1.9225 3.0417 2.9339
1.8902 0.8263 -434.8989
38.2400 -427.6995 0.2900 1.4602 -
1.0380 -0.0996 -1.9229 3.0070 2.9140
1.8767 0.8396 -434.9043
38.2600 -422.6322 3.5401 2.7540 -
0.3737 0.0280 -1.9006 2.9688 2.8920
1.8628 0.8529 -434.9097
38.2800 -425.4958 0.3956 1.5178
0.2104 0.1487 -1.8567 2.9271 2.8681
1.8485 0.8661 -434.9151
38.3000 -430.8032 -2.4901 -0.7442
0.6315 0.2668 -1.7929 2.8820 2.8422
1.8338 0.8792 -434.9205
38.3200 -429.9232 -1.2479 -1.4983
0.8514 0.3840 -1.7107 2.8335 2.8143
1.8187 0.8923 -434.9259
38.3400 -426.1614 1.1356 -0.3642
0.8721 0.5001 -1.6119 2.7818 2.7845
1.8033 0.9053 -434.9312
38.3600 -425.4219 0.5236 0.9897
0.6965 0.6130 -1.4983 2.7268 2.7529
1.7874 0.9182 -434.9366
38.3800 -426.2360 0.0637 1.2138
0.3362 0.7189 -1.3715 2.6688 2.7193
1.7711 0.9311 -434.9419
38.4000 -427.2890 0.1824 0.2576 -
0.1468 0.8135 -1.2331 2.6077 2.6840
1.7545 0.9440 -434.9472
38.4200 -428.9037 -0.4400 -0.7641 -
0.6402 0.8909 -1.0846 2.5436 2.6469
1.7375 0.9567 -434.9525
38.4400 -429.7978 -0.2425 -1.1607 -
1.0041 0.9451 -0.9273 2.4765 2.6081
1.7201 0.9695 -434.9577
38.4600 -428.9041 -0.0559 -0.8818 -
1.1229 0.9705 -0.7623 2.4066 2.5676
1.7024 0.9821 -434.9630
38.4800 -427.5111 0.1878 -0.1458 -
0.9401 0.9617 -0.5906 2.3339 2.5255
1.6843 0.9947 -434.9682
38.5000 -426.6408 -0.0921 0.5851 -
0.4938 0.9148 -0.4132 2.2583 2.4818
1.6659 1.0073 -434.9735

38.5200 -425.9877 0.4753 0.7406
0.1057 0.8264 -0.2314 2.1799 2.4366
1.6471 1.0198 -434.9787
38.5400 -425.9164 0.1837 0.2922
0.7197 0.6941 -0.0465 2.0987 2.3898
1.6280 1.0322 -434.9839
38.5600 -425.9805 -0.2475 -0.1938
1.2090 0.5176 0.1402 2.0148 2.3417
1.6085 1.0446 -434.9891
38.5800 -425.9667 -0.0270 -0.1897
1.4649 0.2988 0.3275 1.9280 2.2921
1.5887 1.0569 -434.9943
38.6000 -425.7191 -0.2092 0.1403
1.4170 0.0460 0.5141 1.8386 2.2411
1.5686 1.0692 -434.9994
38.6200 -425.6570 0.7682 0.4057
1.0683 -0.2265 0.6987 1.7463 2.1889
1.5482 1.0814 -435.0046
38.6400 -426.9484 0.0649 0.5241
0.4803 -0.4986 0.8801 1.6514 2.1354
1.5275 1.0936 -435.0097
38.6600 -428.5270 -0.6018 0.4911 -
0.2395 -0.7489 1.0571 1.5537 2.0807
1.5064 1.1057 -435.0148
38.6800 -429.5584 0.4053 0.2027 -
0.9488 -0.9562 1.2286 1.4534 2.0248
1.4851 1.1178 -435.0199
38.7000 -430.4943 0.1961 -0.4536 -
1.4818 -1.1009 1.3935 1.3506 1.9678
1.4635 1.1298 -435.0250
38.7200 -431.5754 0.5840 -1.2663 -
1.6792 -1.1670 1.5505 1.2452 1.9097
1.4415 1.1417 -435.0301
38.7400 -432.6013 -0.4593 -1.4582 -
1.4732 -1.1491 1.6985 1.1374 1.8506
1.4193 1.1536 -435.0351
38.7600 -431.9861 -1.7384 -0.4112 -
0.9176 -1.0504 1.8361 1.0274 1.7905
1.3968 1.1655 -435.0402
38.7800 -427.1989 0.2645 1.2109 -
0.1685 -0.8818 1.9619 0.9152 1.7294
1.3741 1.1773 -435.0452
38.8000 -424.4182 2.0931 1.8264
0.5774 -0.6570 2.0742 0.8011 1.6675
1.3511 1.1891 -435.0502
38.8200 -426.6521 -0.6474 0.7374
1.1416 -0.3903 2.1714 0.6852 1.6047
1.3278 1.2008 -435.0552
38.8400 -428.8824 -1.0849 -0.8400
1.3859 -0.0968 2.2519 0.5676 1.5411
1.3042 1.2124 -435.0602

38.8600 -428.3470 -0.0418 -1.1497
1.2322 0.2061 2.3143 0.4485 1.4768
1.2804 1.2240 -435.0652
38.8800 -427.3138 0.5292 0.0317
0.7282 0.4993 2.3572 0.3281 1.4117
1.2564 1.2356 -435.0701
38.9000 -426.7559 0.0315 1.4627
0.0540 0.7658 2.3791 0.2065 1.3460
1.2321 1.2471 -435.0751
38.9200 -426.3682 -0.1605 1.8631 -
0.5981 0.9901 2.3788 0.0840 1.2796
1.2075 1.2586 -435.0800
38.9400 -427.1745 1.4342 0.6539 -
1.0526 1.1578 2.3554 -0.0393 1.2127
1.1828 1.2700 -435.0849
38.9600 -432.0087 -0.6372 -1.7320 -
1.1933 1.2569 2.3078 -0.1632 1.1453
1.1578 1.2814 -435.0898
38.9800 -434.2245 -1.5125 -3.4087 -
0.9455 1.2784 2.2361 -0.2874 1.0773
1.1326 1.2927 -435.0947
39.0000 -432.1616 -0.3986 -2.7079 -
0.3299 1.2214 2.1406 -0.4117 1.0090
1.1072 1.3040 -435.0996
39.0200 -429.1487 -0.7854 0.0060
0.4670 1.0915 2.0222 -0.5359 0.9402
1.0815 1.3153 -435.1044
39.0400 -426.2106 0.0012 2.7750
1.1825 0.8976 1.8821 -0.6596 0.8710
1.0557 1.3265 -435.1093
39.0600 -423.5547 1.8292 3.6949
1.5582 0.6518 1.7216 -0.7826 0.8016
1.0297 1.3376 -435.1141
39.0800 -425.4789 1.6112 1.9029
1.4554 0.3705 1.5425 -0.9047 0.7319
1.0035 1.3487 -435.1189
39.1000 -433.8102 -2.3036 -1.0981
0.9728 0.0749 1.3464 -1.0256 0.6619
0.9771 1.3598 -435.1237
39.1200 -435.8367 -0.7659 -2.6190
0.2788 -0.2124 1.1351 -1.1449 0.5918
0.9505 1.3708 -435.1285
39.1400 -434.7731 0.1517 -1.7227 -
0.4499 -0.4697 0.9105 -1.2626 0.5216
0.9237 1.3818 -435.1333
39.1600 -434.0032 1.6489 -0.1176 -
1.0410 -0.6766 0.6743 -1.3782 0.4513
0.8968 1.3927 -435.1380
39.1800 -434.9448 -0.0257 0.5863 -
1.3699 -0.8149 0.4287 -1.4915 0.3809
0.8697 1.4036 -435.1428

39.2000 -436.5605 -0.3997 0.3128 -
1.4317 -0.8713 0.1760 -1.6023 0.3106
0.8424 1.4145 -435.1475
39.2200 -436.8360 -0.1795 -0.1877 -
1.3005 -0.8407 -0.0815 -1.7102 0.2402
0.8150 1.4253 -435.1522
39.2400 -436.4334 0.1100 -0.2066 -
1.0623 -0.7267 -0.3410 -1.8151 0.1700
0.7874 1.4361 -435.1569
39.2600 -436.0173 0.5699 0.1906 -
0.7828 -0.5415 -0.5998 -1.9166 0.0999
0.7597 1.4468 -435.1616
39.2800 -435.7509 -0.0915 0.4694 -
0.5087 -0.3032 -0.8549 -2.0144 0.0300
0.7319 1.4575 -435.1663
39.3000 -435.5423 -0.3554 0.3998 -
0.2599 -0.0365 -1.1032 -2.1084 -0.0397
0.7039 1.4682 -435.1709
39.3200 -435.5991 -0.1096 0.1662 -
0.0119 0.2289 -1.3417 -2.1983 -0.1091
0.6758 1.4788 -435.1756
39.3400 -436.3249 -0.1383 -0.2026
0.2864 0.4614 -1.5674 -2.2837 -0.1782
0.6476 1.4894 -435.1802
39.3600 -436.8924 0.2638 -0.5910
0.6698 0.6333 -1.7776 -2.3645 -0.2469
0.6192 1.4999 -435.1848
39.3800 -436.5030 0.0539 -0.6851
1.1106 0.7253 -1.9696 -2.4403 -0.3153
0.5908 1.5104 -435.1894
39.4000 -435.7259 -0.2093 -0.3459
1.5144 0.7274 -2.1413 -2.5109 -0.3832
0.5622 1.5209 -435.1940
39.4200 -435.5088 0.1260 0.3442
1.7448 0.6375 -2.2909 -2.5760 -0.4506
0.5335 1.5313 -435.1986
39.4400 -435.6224 -0.4098 1.0662
1.6833 0.4606 -2.4167 -2.6354 -0.5175
0.5048 1.5417 -435.2031
39.4600 -435.8517 0.1846 1.3331
1.2924 0.2107 -2.5174 -2.6888 -0.5839
0.4759 1.5521 -435.2077
39.4800 -437.3668 0.4831 0.6953
0.6370 -0.0907 -2.5919 -2.7360 -0.6496
0.4470 1.5624 -435.2122
39.5000 -440.4849 -0.6517 -0.4240 -
0.1521 -0.4170 -2.6392 -2.7769 -0.7146
0.4180 1.5727 -435.2167
39.5200 -442.0777 0.0508 -1.0241 -
0.9123 -0.7408 -2.6581 -2.8112 -0.7790
0.3889 1.5830 -435.2212

39.5400 -442.6439 0.5152 -0.7466 -
1.4794 -1.0354 -2.6477 -2.8389 -0.8426
0.3597 1.5932 -435.2257
39.5600 -442.9625 0.0707 -0.3445 -
1.7262 -1.2774 -2.6072 -2.8599 -0.9054
0.3305 1.6034 -435.2301
39.5800 -442.9732 0.1757 -0.3939 -
1.6044 -1.4490 -2.5358 -2.8741 -0.9674
0.3012 1.6136 -435.2346
39.6000 -442.7994 0.3228 -0.6663 -
1.1764 -1.5432 -2.4334 -2.8818 -1.0286
0.2719 1.6237 -435.2390
39.6200 -442.4906 -0.7055 -0.5757 -
0.5955 -1.5611 -2.3007 -2.8829 -1.0888
0.2426 1.6338 -435.2435
39.6400 -441.1983 -0.8451 0.4347 -
0.0438 -1.5063 -2.1393 -2.8777 -1.1482
0.2131 1.6438 -435.2479
39.6600 -438.3667 0.8472 1.8743
0.3396 -1.3845 -1.9512 -2.8663 -1.2066
0.1837 1.6539 -435.2523
39.6800 -436.8002 0.6662 2.0490
0.5021 -1.2052 -1.7395 -2.8490 -1.2641
0.1542 1.6639 -435.2567
39.7000 -438.9457 0.3678 0.1698
0.4810 -0.9822 -1.5070 -2.8260 -1.3206
0.1247 1.6739 -435.2610
39.7200 -442.3975 -1.0814 -2.1320
0.3822 -0.7292 -1.2570 -2.7975 -1.3761
0.0952 1.6838 -435.2654
39.7400 -442.8392 -1.4626 -2.3285
0.3084 -0.4591 -0.9926 -2.7637 -1.4306
0.0657 1.6937 -435.2697
39.7600 -438.9422 0.0752 -0.1723
0.3009 -0.1823 -0.7173 -2.7248 -1.4840
0.0361 1.7036 -435.2740
39.7800 -435.1066 1.8032 1.8725
0.3279 0.0921 -0.4347 -2.6810 -1.5365
0.0066 1.7135 -435.2783
39.8000 -435.3345 0.6967 1.7012
0.3444 0.3558 -0.1483 -2.6326 -1.5879
-0.0230 1.7233 -435.2826
39.8200 -437.5543 -0.6158 -0.1787
0.3317 0.6010 0.1382 -2.5797 -1.6382
-0.0525 1.7331 -435.2869
39.8400 -438.6788 -1.0781 -1.4320
0.2779 0.8210 0.4209 -2.5226 -1.6875
-0.0820 1.7429 -435.2912
39.8600 -436.9448 -0.4885 -0.7878
0.1937 1.0099 0.6965 -2.4614 -1.7358
-0.1115 1.7526 -435.2954

39.8800 -434.6168 1.2913 0.4513
0.1119 1.1625 0.9615 -2.3965 -1.7829
-0.1410 1.7623 -435.2997
39.9000 -434.4506 0.4905 0.4980
0.0710 1.2743 1.2130 -2.3279 -1.8290
-0.1705 1.7720 -435.3039
39.9200 -435.7061 -0.4879 -0.4896
0.0993 1.3406 1.4480 -2.2559 -1.8741
-0.1999 1.7817 -435.3081
39.9400 -436.4997 -1.1379 -0.7542
0.1847 1.3541 1.6643 -2.1807 -1.9180
-0.2293 1.7914 -435.3123
39.9600 -434.4873 -0.3290 0.3821
0.2897 1.3065 1.8597 -2.1024 -1.9608
-0.2586 1.8010 -435.3165
39.9800 -431.4511 0.7688 1.6739
0.3569 1.1925 2.0326 -2.0214 -2.0026
-0.2879 1.8106 -435.3207
40.0000 -431.6349 1.3418 1.4073
0.3491 1.0122 2.1816 -1.9378 -2.0433
-0.3171 1.8202 -435.3248
40.0200 -435.2331 -0.3374 -0.3524
0.3084 0.7687 2.3058 -1.8518 -2.0828
-0.3463 1.8297 -435.3289
40.0400 -437.2935 -1.1008 -1.7547
0.3276 0.4706 2.4047 -1.7636 -2.1213
-0.3754 1.8393 -435.3331
40.0600 -436.2247 0.1315 -1.5390
0.4815 0.1315 2.4778 -1.6735 -2.1587
-0.4045 1.8488 -435.3372
40.0800 -434.6048 0.8266 -0.2679
0.7239 -0.2282 2.5248 -1.5815 -2.1950
-0.4334 1.8583 -435.3413
40.1000 -433.8887 -0.4308 0.9347
0.9175 -0.5827 2.5457 -1.4881 -2.2302
-0.4623 1.8677 -435.3453
40.1200 -433.4565 -0.3115 1.4292
0.8917 -0.9033 2.5404 -1.3933 -2.2642
-0.4911 1.8772 -435.3494
40.1400 -433.3215 0.5899 1.1940
0.5215 -1.1621 2.5090 -1.2975 -2.2972
-0.5198 1.8866 -435.3535
40.1600 -435.8246 0.1575 0.3861 -
0.1813 -1.3382 2.4518 -1.2007 -2.3291
-0.5484 1.8960 -435.3575
40.1800 -439.1304 -0.7183 -0.4784 -
1.0746 -1.4179 2.3696 -1.1033 -2.3598
-0.5769 1.9054 -435.3615
40.2000 -439.5486 0.2028 -0.7196 -
1.9540 -1.3937 2.2633 -1.0054 -2.3895
-0.6054 1.9147 -435.3655

40.2200	-439.2983	0.0843	-0.4785	-
2.6117	-1.2611	2.1342	-0.9073	-2.4180
	-0.6337	1.9241	-435.3695	
40.2400	-439.0063	0.1662	-0.3928	-
2.8750	-1.0212	1.9837	-0.8091	-2.4454
	-0.6618	1.9334	-435.3735	
40.2600	-438.8000	0.7370	-0.7368	-
2.6663	-0.6850	1.8133	-0.7111	-2.4717
	-0.6899	1.9427	-435.3775	
40.2800	-438.5639	-0.3483	-0.9809	-
2.0002	-0.2781	1.6249	-0.6135	-2.4969
	-0.7179	1.9520	-435.3814	
40.3000	-437.8507	-0.4357	-0.5004	-
0.9685	0.1674	1.4201	-0.5165	-2.5209
	-0.7457	1.9613	-435.3853	
40.3200	-434.2495	-0.3148	0.5269	
0.2711	0.6165	1.2007	-0.4203	-2.5438
	-0.7734	1.9706	-435.3893	
40.3400	-431.0633	1.3384	1.1179	
1.5085	1.0344	0.9687	-0.3251	-2.5656
	-0.8010	1.9798	-435.3932	
40.3600	-431.3908	0.2907	0.6182	
2.5336	1.3869	0.7258	-0.2311	-2.5863
	-0.8284	1.9890	-435.3971	
40.3800	-432.6058	-0.4790	-0.1517	
3.1665	1.6425	0.4740	-0.1386	-2.6058
	-0.8557	1.9982	-435.4009	
40.4000	-432.9155	-0.4700	-0.0922	
3.2968	1.7755	0.2152	-0.0478	-2.6242
	-0.8828	2.0074	-435.4048	
40.4200	-432.0161	-0.0716	0.7181	
2.9026	1.7761	-0.0482	0.0411	-2.6415
	-0.9098	2.0166	-435.4086	
40.4400	-431.3043	0.6670	1.1617	
2.0632	1.6542	-0.3134	0.1279	-2.6577
	-0.9366	2.0258	-435.4125	
40.4600	-433.5374	0.5367	0.5846	
0.9264	1.4308	-0.5772	0.2124	-2.6727
	-0.9632	2.0349	-435.4163	
40.4800	-437.7836	-1.1716	-0.4490	-
0.2917	1.1306	-0.8366	0.2944	-2.6866
	-0.9897	2.0441	-435.4201	
40.5000	-439.7027	-0.1080	-0.9684	-
1.3704	0.7789	-1.0888	0.3737	-2.6994
	-1.0160	2.0532	-435.4239	
40.5200	-440.7201	0.3483	-0.7987	-
2.1427	0.4017	-1.3308	0.4500	-2.7110
	-1.0422	2.0623	-435.4277	
40.5400	-441.1930	-0.4227	-0.3666	-
2.5096	0.0249	-1.5599	0.5231	-2.7215
	-1.0681	2.0714	-435.4314	

40.5600 -440.9700 0.1876 0.1017 -
2.4743 -0.3275 -1.7734 0.5930 -2.7309
-1.0939 2.0805 -435.4352
40.5800 -440.4570 0.3425 0.5296 -
2.1314 -0.6368 -1.9688 0.6593 -2.7391
-1.1195 2.0896 -435.4389
40.6000 -440.1898 0.0595 0.6551 -
1.6162 -0.8893 -2.1431 0.7219 -2.7463
-1.1449 2.0987 -435.4426
40.6200 -440.1355 -0.1994 0.5244 -
1.0600 -1.0776 -2.2936 0.7806 -2.7522
-1.1701 2.1078 -435.4463
40.6400 -440.1048 -0.1137 0.2305 -
0.5420 -1.2024 -2.4178 0.8352 -2.7571
-1.1951 2.1168 -435.4500
40.6600 -440.1506 0.1338 -0.0401 -
0.1000 -1.2715 -2.5132 0.8857 -2.7608
-1.2199 2.1259 -435.4537
40.6800 -440.4856 0.2753 -0.3134
0.2874 -1.2984 -2.5777 0.9317 -2.7634
-1.2445 2.1349 -435.4574
40.7000 -440.7817 -0.2477 -0.7752
0.6519 -1.2985 -2.6096 0.9733 -2.7648
-1.2689 2.1439 -435.4610
40.7200 -440.4686 0.3666 -1.0270
1.0036 -1.2848 -2.6074 1.0104 -2.7652
-1.2930 2.1530 -435.4646
40.7400 -439.3923 0.1012 -0.6779
1.2766 -1.2663 -2.5708 1.0429 -2.7644
-1.3170 2.1620 -435.4683
40.7600 -438.3697 -0.0932 0.2465
1.3837 -1.2476 -2.4998 1.0710 -2.7625
-1.3407 2.1710 -435.4719
40.7800 -437.5311 -0.2792 1.3918
1.2333 -1.2291 -2.3952 1.0946 -2.7595
-1.3642 2.1800 -435.4755
40.8000 -436.8449 -0.0840 1.9575
0.7915 -1.2067 -2.2585 1.1139 -2.7554
-1.3875 2.1890 -435.4790
40.8200 -437.1644 0.9244 1.1558
0.1258 -1.1756 -2.0917 1.1289 -2.7503
-1.4105 2.1980 -435.4826
40.8400 -440.8051 0.1028 -0.7193 -
0.5934 -1.1314 -1.8975 1.1398 -2.7441
-1.4333 2.2069 -435.4861
40.8600 -444.0998 -1.2216 -2.1407 -
1.1193 -1.0700 -1.6786 1.1468 -2.7369
-1.4558 2.2159 -435.4897
40.8800 -443.0142 -0.8878 -1.6957 -
1.2569 -0.9881 -1.4381 1.1498 -2.7287
-1.4781 2.2249 -435.4932

40.9000 -439.2440 0.3543 0.0021 -
0.9812 -0.8860 -1.1794 1.1492 -2.7195
-1.5002 2.2339 -435.4967
40.9200 -436.7913 0.7914 1.1824 -
0.4107 -0.7644 -0.9061 1.1449 -2.7093
-1.5220 2.2428 -435.5002
40.9400 -436.4231 -0.3544 1.1373
0.2829 -0.6243 -0.6216 1.1372 -2.6982
-1.5436 2.2518 -435.5036
40.9600 -436.1972 -0.2894 0.3166
0.8976 -0.4673 -0.3294 1.1261 -2.6862
-1.5648 2.2607 -435.5071
40.9800 -435.8250 -0.1669 -0.2651
1.2446 -0.2942 -0.0327 1.1119 -2.6733
-1.5859 2.2697 -435.5105
41.0000 -435.2186 0.0501 -0.0739
1.2263 -0.1061 0.2650 1.0947 -2.6595
-1.6066 2.2787 -435.5140
41.0200 -434.5871 -0.0347 0.5982
0.8534 0.0948 0.5604 1.0746 -2.6450
-1.6271 2.2876 -435.5174
41.0400 -433.9863 -0.3886 1.3361
0.1976 0.3057 0.8503 1.0518 -2.6297
-1.6474 2.2966 -435.5208
41.0600 -433.4363 0.3266 1.6525 -
0.6061 0.5228 1.1315 1.0264 -2.6136
-1.6673 2.3055 -435.5242
41.0800 -433.6194 0.8902 0.8717 -
1.3627 0.7409 1.4007 0.9986 -2.5968
-1.6870 2.3145 -435.5276
41.1000 -436.6828 0.2533 -0.9730 -
1.8677 0.9534 1.6549 0.9685 -2.5794
-1.7064 2.3234 -435.5309
41.1200 -439.3242 -1.4958 -2.6755 -
1.9408 1.1535 1.8911 0.9362 -2.5613
-1.7255 2.3324 -435.5343
41.1400 -437.9213 -0.6918 -2.7259 -
1.4792 1.3355 2.1065 0.9020 -2.5426
-1.7444 2.3413 -435.5376
41.1600 -434.0933 1.0545 -0.9900 -
0.5709 1.4921 2.2986 0.8659 -2.5234
-1.7629 2.3503 -435.5409
41.1800 -430.9324 -0.0254 1.2798
0.5256 1.6143 2.4653 0.8282 -2.5036
-1.7812 2.3593 -435.5442
41.2000 -428.1369 -0.6271 2.7068
1.5061 1.6924 2.6047 0.7890 -2.4833
-1.7991 2.3682 -435.5475
41.2200 -426.5265 1.4325 2.6222
2.1014 1.7173 2.7154 0.7484 -2.4626
-1.8168 2.3772 -435.5508

41.2400	-428.6253	0.6252	0.9157	
2.1908	1.6823	2.7966	0.7066	-2.4415
	-1.8341	2.3862	-435.5540	
41.2600	-432.5473	-1.6529	-1.0884	
1.8143	1.5889	2.8481	0.6638	-2.4200
	-1.8512	2.3951	-435.5573	
41.2800	-433.2677	-0.0908	-1.6760	
1.0745	1.4456	2.8704	0.6201	-2.3981
	-1.8679	2.4041	-435.5605	
41.3000	-433.3234	1.0561	-0.8418	
0.1397	1.2637	2.8643	0.5757	-2.3760
	-1.8844	2.4131	-435.5637	
41.3200	-433.4316	-0.1717	0.3392	-
0.7607	1.0562	2.8310	0.5307	-2.3536
	-1.9005	2.4221	-435.5669	
41.3400	-433.8772	0.2362	0.8188	-
1.4153	0.8366	2.7718	0.4853	-2.3309
	-1.9163	2.4311	-435.5701	
41.3600	-434.6442	-0.0577	0.2460	-
1.6841	0.6173	2.6882	0.4396	-2.3081
	-1.9319	2.4401	-435.5733	
41.3800	-436.1189	0.9081	-1.1039	-
1.5467	0.4088	2.5820	0.3937	-2.2850
	-1.9470	2.4491	-435.5764	
41.4000	-438.4904	-0.4620	-2.1408	-
1.0677	0.2187	2.4546	0.3479	-2.2619
	-1.9619	2.4581	-435.5796	
41.4200	-439.2014	-2.7195	-1.5014	-
0.3764	0.0520	2.3075	0.3022	-2.2387
	-1.9764	2.4671	-435.5827	
41.4400	-434.4400	-0.7876	0.8746	
0.3306	-0.0878	2.1424	0.2568	-2.2154
	-1.9907	2.4761	-435.5858	
41.4600	-428.7357	3.1834	2.7865	
0.8434	-0.1974	1.9608	0.2118	-2.1921
	-2.0045	2.4852	-435.5889	
41.4800	-429.5833	3.4689	2.1963	
0.9997	-0.2755	1.7643	0.1673	-2.1689
	-2.0181	2.4942	-435.5920	
41.5000	-437.5132	-2.4459	-0.2360	
0.8292	-0.3241	1.5548	0.1235	-2.1457
	-2.0313	2.5033	-435.5950	
41.5200	-440.8791	-2.9640	-1.8216	
0.4684	-0.3475	1.3339	0.0806	-2.1226
	-2.0442	2.5123	-435.5981	
41.5400	-438.2713	-0.3966	-1.1772	
0.0663	-0.3517	1.1036	0.0386	-2.0996
	-2.0567	2.5214	-435.6011	
41.5600	-435.1932	1.1066	0.3561	-
0.2714	-0.3427	0.8656	-0.0022	-2.0768
	-2.0689	2.5305	-435.6042	

41.5800 -435.3626 1.7819 0.7129 -
0.4441 -0.3253 0.6217 -0.0418 -2.0542
-2.0808 2.5396 -435.6072
41.6000 -438.4431 0.3260 -0.4187 -
0.3921 -0.3035 0.3737 -0.0800 -2.0319
-2.0923 2.5487 -435.6102
41.6200 -440.4000 -1.2679 -1.5112 -
0.1641 -0.2790 0.1233 -0.1167 -2.0098
-2.1034 2.5578 -435.6132
41.6400 -438.9345 -0.5570 -1.1011
0.1335 -0.2529 -0.1276 -0.1516 -1.9879
-2.1142 2.5669 -435.6161
41.6600 -436.4673 0.2679 0.3385
0.3918 -0.2270 -0.3770 -0.1848 -1.9664
-2.1247 2.5761 -435.6191
41.6800 -435.7347 0.6370 1.4822
0.5332 -0.2039 -0.6234 -0.2160 -1.9453
-2.1348 2.5852 -435.6220
41.7000 -436.2283 0.1586 1.6032
0.5064 -0.1854 -0.8651 -0.2450 -1.9245
-2.1445 2.5944 -435.6250
41.7200 -437.0723 0.1111 0.9452
0.3086 -0.1726 -1.1002 -0.2718 -1.9041
-2.1539 2.6036 -435.6279
41.7400 -438.5777 0.2860 -0.1158
0.0077 -0.1664 -1.3272 -0.2962 -1.8842
-2.1629 2.6128 -435.6308
41.7600 -440.9091 -0.6618 -1.1089 -
0.2872 -0.1680 -1.5445 -0.3180 -1.8647
-2.1715 2.6220 -435.6336
41.7800 -441.8722 -0.0346 -1.3978 -
0.4759 -0.1785 -1.7508 -0.3370 -1.8457
-2.1798 2.6312 -435.6365
41.8000 -440.5291 -0.2718 -0.6461 -
0.5029 -0.2008 -1.9448 -0.3532 -1.8273
-2.1877 2.6404 -435.6394
41.8200 -438.6791 0.2488 0.6064 -
0.3783 -0.2374 -2.1251 -0.3664 -1.8093
-2.1952 2.6497 -435.6422
41.8400 -438.3490 0.4140 1.2533 -
0.1531 -0.2908 -2.2904 -0.3763 -1.7919
-2.2024 2.6590 -435.6450
41.8600 -439.6335 -0.3967 0.8114
0.0986 -0.3633 -2.4396 -0.3830 -1.7751
-2.2091 2.6683 -435.6478
41.8800 -440.8225 -0.6772 -0.1567
0.3134 -0.4559 -2.5716 -0.3862 -1.7588
-2.2155 2.6776 -435.6506
41.9000 -440.8297 0.3951 -0.7140
0.4557 -0.5676 -2.6856 -0.3859 -1.7432
-2.2215 2.6869 -435.6534

41.9200 -440.6228 -0.1420 -0.4510
0.5116 -0.6951 -2.7809 -0.3819 -1.7281
-2.2272 2.6962 -435.6562
41.9400 -440.5000 -0.0635 0.1444
0.4717 -0.8322 -2.8571 -0.3742 -1.7136
-2.2324 2.7056 -435.6589
41.9600 -440.5140 0.3165 0.4326
0.3383 -0.9705 -2.9142 -0.3626 -1.6997
-2.2372 2.7150 -435.6617
41.9800 -440.5546 -0.6300 0.3723
0.1333 -1.1015 -2.9522 -0.3471 -1.6864
-2.2417 2.7243 -435.6644
42.0000 -440.6623 0.3076 0.2108 -
0.1163 -1.2150 -2.9712 -0.3278 -1.6737
-2.2458 2.7338 -435.6671
42.0200 -441.4584 0.2702 0.0953 -
0.3789 -1.3007 -2.9714 -0.3046 -1.6616
-2.2495 2.7432 -435.6698
42.0400 -442.3478 -0.5591 -0.0887 -
0.5986 -1.3490 -2.9532 -0.2776 -1.6501
-2.2528 2.7526 -435.6725
42.0600 -442.4965 -0.1895 -0.4603 -
0.7228 -1.3512 -2.9172 -0.2469 -1.6392
-2.2557 2.7621 -435.6751
42.0800 -442.4703 0.3504 -0.8500 -
0.7235 -1.3009 -2.8645 -0.2125 -1.6288
-2.2582 2.7716 -435.6778
42.1000 -442.4192 -0.7252 -0.7527 -
0.6102 -1.1967 -2.7963 -0.1745 -1.6190
-2.2603 2.7811 -435.6804
42.1200 -441.5519 -0.3013 0.1108 -
0.4245 -1.0408 -2.7140 -0.1332 -1.6097
-2.2621 2.7906 -435.6831
42.1400 -439.0145 -0.1263 1.2389 -
0.2115 -0.8390 -2.6193 -0.0885 -1.6010
-2.2634 2.8001 -435.6857
42.1600 -437.4065 1.1546 1.4907
0.0061 -0.5991 -2.5138 -0.0406 -1.5928
-2.2644 2.8097 -435.6883
42.1800 -438.5615 0.4479 0.1558
0.2233 -0.3318 -2.3992 0.0104 -1.5850
-2.2650 2.8193 -435.6908
42.2000 -440.7356 -0.6742 -1.6075
0.4481 -0.0491 -2.2769 0.0643 -1.5778
-2.2651 2.8289 -435.6934
42.2200 -441.2789 -1.4912 -1.8050
0.6670 0.2384 -2.1487 0.1209 -1.5710
-2.2650 2.8385 -435.6960
42.2400 -437.6942 -0.0354 -0.2039
0.8306 0.5213 -2.0161 0.1803 -1.5647
-2.2644 2.8481 -435.6985

42.2600 -433.7222 1.5847 1.6882
0.8654 0.7903 -1.8807 0.2421 -1.5588
-2.2634 2.8577 -435.7010
42.2800 -433.6743 0.0517 2.2458
0.7006 1.0367 -1.7440 0.3063 -1.5533
-2.2621 2.8674 -435.7035
42.3000 -435.6724 -0.6304 1.2482
0.3303 1.2530 -1.6074 0.3727 -1.5482
-2.2604 2.8771 -435.7060
42.3200 -437.2245 -0.6146 -0.3712 -
0.1555 1.4330 -1.4722 0.4412 -1.5435
-2.2583 2.8868 -435.7085
42.3400 -437.8389 0.1441 -1.5205 -
0.6077 1.5725 -1.3397 0.5115 -1.5391
-2.2559 2.8965 -435.7110
42.3600 -438.2348 -0.2503 -1.8217 -
0.8580 1.6681 -1.2108 0.5836 -1.5351
-2.2531 2.9063 -435.7134
42.3800 -437.8900 0.0393 -1.2820 -
0.7996 1.7174 -1.0863 0.6572 -1.5314
-2.2499 2.9160 -435.7158
42.4000 -435.3449 -0.0313 -0.0731 -
0.4581 1.7178 -0.9663 0.7321 -1.5280
-2.2464 2.9258 -435.7183
42.4200 -433.3422 0.6441 0.9655
0.0389 1.6662 -0.8509 0.8082 -1.5249
-2.2426 2.9356 -435.7207
42.4400 -433.3129 -0.5331 1.1972
0.5120 1.5599 -0.7399 0.8852 -1.5221
-2.2383 2.9454 -435.7231
42.4600 -433.3573 -0.1793 1.0352
0.8029 1.3971 -0.6329 0.9629 -1.5195
-2.2338 2.9553 -435.7254
42.4800 -433.4588 0.0567 0.8703
0.8523 1.1782 -0.5289 1.0411 -1.5172
-2.2289 2.9651 -435.7278
42.5000 -433.9006 0.4741 0.5900
0.6903 0.9071 -0.4272 1.1195 -1.5151
-2.2236 2.9750 -435.7302
42.5200 -434.6132 -0.0272 -0.1999
0.4045 0.5922 -0.3267 1.1979 -1.5131
-2.2180 2.9849 -435.7325
42.5400 -436.1972 0.5624 -1.2485
0.1349 0.2451 -0.2264 1.2760 -1.5114
-2.2121 2.9948 -435.7348
42.5600 -438.3338 -0.5315 -1.7573 -
0.0032 -0.1210 -0.1255 1.3537 -1.5098
-2.2059 3.0047 -435.7371
42.5800 -438.4658 -1.9694 -1.0092
0.0104 -0.4909 -0.0230 1.4306 -1.5084
-2.1994 3.0147 -435.7394

42.6000 -434.5609 -0.2782 0.7121
0.1087 -0.8464 0.0818 1.5064 -1.5071
-2.1925 3.0247 -435.7417
42.6200 -430.9962 2.5191 1.9107
0.1819 -1.1682 0.1897 1.5810 -1.5059
-2.1853 3.0346 -435.7440
42.6400 -432.7540 1.2555 1.2814
0.1257 -1.4375 0.3011 1.6541 -1.5048
-2.1778 3.0446 -435.7462
42.6600 -437.6849 -1.8670 -0.4387 -
0.0873 -1.6380 0.4166 1.7254 -1.5038
-2.1700 3.0547 -435.7484
42.6800 -439.0780 -1.8538 -1.3210 -
0.4231 -1.7565 0.5365 1.7946 -1.5028
-2.1619 3.0647 -435.7507
42.7000 -436.5570 0.1805 -0.5787 -
0.8115 -1.7851 0.6610 1.8615 -1.5019
-2.1536 3.0748 -435.7529
42.7200 -434.0280 1.4576 0.6785 -
1.1544 -1.7216 0.7903 1.9259 -1.5010
-2.1449 3.0849 -435.7551
42.7400 -434.1583 0.4526 0.9827 -
1.3328 -1.5674 0.9238 1.9875 -1.5001
-2.1359 3.0950 -435.7572
42.7600 -435.2883 -0.3954 0.1311 -
1.2685 -1.3287 1.0607 2.0460 -1.4992
-2.1267 3.1051 -435.7594
42.7800 -435.7737 -0.0904 -0.9211 -
0.9472 -1.0191 1.1997 2.1014 -1.4983
-2.1172 3.1152 -435.7615
42.8000 -434.8056 0.3736 -1.3084 -
0.4159 -0.6596 1.3393 2.1532 -1.4973
-2.1074 3.1254 -435.7637
42.8200 -433.1758 -0.5681 -0.8726
0.2169 -0.2731 1.4781 2.2014 -1.4963
-2.0973 3.1355 -435.7658
42.8400 -431.4519 0.0219 0.0865
0.8071 0.1177 1.6146 2.2457 -1.4952
-2.0870 3.1457 -435.7679
42.8600 -429.3770 0.6002 0.9855
1.2070 0.4908 1.7473 2.2860 -1.4940
-2.0764 3.1559 -435.7700
42.8800 -428.6363 -0.1402 1.1060
1.3066 0.8246 1.8748 2.3219 -1.4927
-2.0656 3.1662 -435.7721
42.9000 -429.5911 -0.2728 0.5020
1.0730 1.1004 1.9954 2.3533 -1.4913
-2.0545 3.1764 -435.7741
42.9200 -430.4844 -0.3442 -0.0398
0.5976 1.3067 2.1080 2.3801 -1.4898
-2.0432 3.1867 -435.7762

42.9400	-430.4059	-0.0879	-0.1369	
0.0394	1.4386	2.2109	2.4019	-1.4881
	-2.0316	3.1970	-435.7782	
42.9600	-430.2317	0.1548	-0.0835	-
0.4187	1.4948	2.3033	2.4186	-1.4863
	-2.0199	3.2073	-435.7802	
42.9800	-430.3387	0.4708	-0.3290	-
0.6358	1.4778	2.3840	2.4301	-1.4842
	-2.0078	3.2176	-435.7822	
43.0000	-430.9823	-0.1104	-0.8578	-
0.5483	1.3920	2.4525	2.4361	-1.4820
	-1.9956	3.2279	-435.7842	
43.0200	-431.2536	-0.7055	-0.8644	-
0.1967	1.2446	2.5083	2.4364	-1.4796
	-1.9831	3.2383	-435.7862	
43.0400	-429.5823	0.2357	0.0853	
0.2863	1.0471	2.5517	2.4310	-1.4769
	-1.9705	3.2487	-435.7882	
43.0600	-427.8641	0.3162	1.1738	
0.7430	0.8129	2.5828	2.4196	-1.4740
	-1.9576	3.2591	-435.7901	
43.0800	-428.0097	-0.2638	1.4943	
1.0451	0.5561	2.6021	2.4022	-1.4709
	-1.9445	3.2695	-435.7920	
43.1000	-428.6786	-0.3594	0.7128	
1.1186	0.2900	2.6102	2.3786	-1.4675
	-1.9312	3.2799	-435.7940	
43.1200	-429.8331	0.7345	-0.7061	
0.9672	0.0279	2.6078	2.3488	-1.4639
	-1.9177	3.2904	-435.7959	
43.1400	-432.1484	-0.4749	-1.6752	
0.6304	-0.2176	2.5954	2.3127	-1.4600
	-1.9040	3.3008	-435.7977	
43.1600	-433.1502	-1.0050	-1.2825	
0.1492	-0.4344	2.5735	2.2705	-1.4558
	-1.8902	3.3113	-435.7996	
43.1800	-431.2614	0.0865	0.5140	-
0.4316	-0.6124	2.5423	2.2220	-1.4513
	-1.8761	3.3218	-435.8015	
43.2000	-429.2176	1.2851	2.2454	-
1.0272	-0.7433	2.5024	2.1674	-1.4465
	-1.8619	3.3324	-435.8033	
43.2200	-430.8485	0.8757	2.1543	-
1.5458	-0.8200	2.4544	2.1067	-1.4414
	-1.8475	3.3429	-435.8052	
43.2400	-435.1668	-1.1396	0.2302	-
1.8983	-0.8383	2.3989	2.0401	-1.4360
	-1.8330	3.3535	-435.8070	
43.2600	-436.3853	-0.7038	-1.8855	-
1.9787	-0.7984	2.3367	1.9677	-1.4303
	-1.8182	3.3640	-435.8088	

43.2800 -436.2140 0.0992 -2.8150 -
1.6828 -0.7067 2.2686 1.8896 -1.4242
-1.8034 3.3746 -435.8106
43.3000 -435.8721 -0.0101 -2.1840 -
0.9900 -0.5756 2.1955 1.8060 -1.4177
-1.7883 3.3853 -435.8123
43.3200 -433.3835 -0.8840 -0.3394 -
0.0209 -0.4220 2.1184 1.7170 -1.4109
-1.7731 3.3959 -435.8141
43.3400 -428.5062 0.7365 1.6577
1.0049 -0.2644 2.0380 1.6229 -1.4036
-1.7578 3.4065 -435.8158
43.3600 -426.9924 0.3754 2.6027
1.8267 -0.1204 1.9551 1.5238 -1.3960
-1.7423 3.4172 -435.8176
43.3800 -428.2246 -0.0939 1.9884
2.2380 -0.0062 1.8699 1.4199 -1.3880
-1.7268 3.4279 -435.8193
43.4000 -429.7275 -0.6872 0.7173
2.1677 0.0687 1.7826 1.3116 -1.3796
-1.7110 3.4386 -435.8210
43.4200 -430.8246 -0.0502 -0.1290
1.6567 0.1040 1.6931 1.1990 -1.3708
-1.6952 3.4493 -435.8227
43.4400 -431.9156 0.6067 -0.3800
0.8241 0.1071 1.6010 1.0824 -1.3615
-1.6792 3.4601 -435.8244
43.4600 -433.2642 -0.2060 -0.5111 -
0.1347 0.0902 1.5061 0.9621 -1.3518
-1.6631 3.4708 -435.8260
43.4800 -434.8090 -0.0417 -0.6327 -
1.0233 0.0676 1.4078 0.8384 -1.3417
-1.6469 3.4816 -435.8277
43.5000 -435.7423 -0.0047 -0.7768 -
1.6732 0.0527 1.3058 0.7115 -1.3310
-1.6306 3.4924 -435.8293
43.5200 -436.2790 0.5073 -1.1006 -
1.9468 0.0547 1.1998 0.5819 -1.3200
-1.6142 3.5032 -435.8309
43.5400 -436.5801 -0.5634 -1.2269 -
1.8000 0.0798 1.0894 0.4497 -1.3084
-1.5976 3.5141 -435.8325
43.5600 -435.8314 -0.3155 -0.5006 -
1.3231 0.1299 0.9743 0.3153 -1.2963
-1.5810 3.5249 -435.8341
43.5800 -433.4979 0.5777 0.7115 -
0.6885 0.2031 0.8543 0.1790 -1.2838
-1.5643 3.5358 -435.8357
43.6000 -432.4076 0.1887 1.4402 -
0.0593 0.2938 0.7295 0.0411 -1.2707
-1.5475 3.5467 -435.8372

43.6200 -432.7791 -0.2779 1.1983
0.4491 0.3916 0.5999 -0.0981 -1.2571
-1.5307 3.5576 -435.8388
43.6400 -433.4069 -0.1665 0.3138
0.8084 0.4837 0.4654 -0.2382 -1.2430
-1.5137 3.5685 -435.8403
43.6600 -434.0586 0.3703 -0.6790
1.0539 0.5562 0.3259 -0.3791 -1.2283
-1.4967 3.5794 -435.8418
43.6800 -434.8173 0.0286 -1.2371
1.2191 0.5966 0.1816 -0.5202 -1.2131
-1.4796 3.5904 -435.8433
43.7000 -435.0440 -0.4483 -0.7994
1.2793 0.5989 0.0330 -0.6615 -1.1973
-1.4624 3.6014 -435.8448
43.7200 -433.4313 -0.6147 0.5663
1.1804 0.5610 -0.1194 -0.8025 -1.1809
-1.4452 3.6123 -435.8463
43.7400 -431.6489 0.6616 1.8786
0.8585 0.4861 -0.2744 -0.9430 -1.1640
-1.4279 3.6234 -435.8477
43.7600 -432.7315 0.7755 1.8955
0.3117 0.3839 -0.4308 -1.0827 -1.1465
-1.4106 3.6344 -435.8492
43.7800 -436.6235 -0.5092 0.6202 -
0.3607 0.2658 -0.5870 -1.2213 -1.1283
-1.3932 3.6454 -435.8506
43.8000 -438.9879 -0.8250 -0.9234 -
0.9954 0.1444 -0.7417 -1.3585 -1.1096
-1.3758 3.6565 -435.8520
43.8200 -439.8473 0.7622 -1.9045 -
1.4166 0.0323 -0.8933 -1.4941 -1.0902
-1.3583 3.6676 -435.8534
43.8400 -440.3432 0.0910 -1.9880 -
1.4899 -0.0600 -1.0401 -1.6277 -1.0702
-1.3408 3.6787 -435.8548
43.8600 -439.9741 -0.6325 -1.1444 -
1.1791 -0.1291 -1.1803 -1.7593 -1.0496
-1.3233 3.6898 -435.8562
43.8800 -438.0254 0.3489 0.1109 -
0.5864 -0.1780 -1.3123 -1.8883 -1.0283
-1.3057 3.7009 -435.8576
43.9000 -436.4539 -0.1086 1.0741
0.0991 -0.2128 -1.4342 -2.0147 -1.0064
-1.2881 3.7121 -435.8589
43.9200 -436.1311 -0.2081 1.3489
0.6661 -0.2404 -1.5445 -2.1382 -0.9838
-1.2705 3.7232 -435.8602
43.9400 -435.9411 0.1303 1.2409
0.9494 -0.2679 -1.6420 -2.2584 -0.9605
-1.2529 3.7344 -435.8615

43.9600 -436.1105 1.1560 0.7328
0.8926 -0.3040 -1.7253 -2.3752 -0.9366
-1.2352 3.7456 -435.8628
43.9800 -438.4582 0.2297 -0.3152
0.5748 -0.3547 -1.7935 -2.4882 -0.9119
-1.2175 3.7568 -435.8641
44.0000 -440.6744 -1.2123 -1.1266
0.1826 -0.4238 -1.8458 -2.5973 -0.8865
-1.1999 3.7681 -435.8654
44.0200 -440.7490 0.5025 -1.0576 -
0.0839 -0.5118 -1.8818 -2.7021 -0.8604
-1.1822 3.7793 -435.8667
44.0400 -440.5556 0.0350 -0.6506 -
0.0994 -0.6147 -1.9013 -2.8026 -0.8336
-1.1645 3.7906 -435.8679
44.0600 -440.2710 -0.5087 -0.2852
0.1058 -0.7235 -1.9044 -2.8984 -0.8061
-1.1468 3.8019 -435.8691
44.0800 -439.4423 -0.1885 0.3973
0.3546 -0.8255 -1.8911 -2.9895 -0.7778
-1.1291 3.8132 -435.8704
44.1000 -438.4481 0.2521 1.0738
0.4558 -0.9064 -1.8620 -3.0757 -0.7487
-1.1115 3.8245 -435.8716
44.1200 -438.3694 0.2604 1.0772
0.3006 -0.9517 -1.8178 -3.1569 -0.7189
-1.0938 3.8358 -435.8727
44.1400 -439.8212 0.1784 0.3077 -
0.0982 -0.9492 -1.7595 -3.2331 -0.6883
-1.0762 3.8472 -435.8739
44.1600 -441.1936 -0.7314 -0.4844 -
0.6279 -0.8920 -1.6883 -3.3041 -0.6570
-1.0585 3.8585 -435.8751
44.1800 -441.2764 0.5244 -0.6520 -
1.1792 -0.7774 -1.6057 -3.3699 -0.6248
-1.0409 3.8699 -435.8762
44.2000 -441.1725 -0.6434 -0.3744 -
1.6592 -0.6053 -1.5138 -3.4304 -0.5919
-1.0233 3.8813 -435.8773
44.2200 -441.0070 0.3247 0.0008 -
1.9758 -0.3814 -1.4146 -3.4856 -0.5581
-1.0057 3.8927 -435.8785
44.2400 -440.5376 0.1463 0.2748 -
2.0251 -0.1182 -1.3101 -3.5353 -0.5236
-0.9882 3.9042 -435.8796
44.2600 -439.6974 -0.1328 0.0732 -
1.7270 0.1690 -1.2028 -3.5797 -0.4882
-0.9707 3.9156 -435.8807
44.2800 -438.8436 -0.0198 -0.5827 -
1.0590 0.4611 -1.0949 -3.6187 -0.4520
-0.9532 3.9271 -435.8817

44.3000	−438.0865	0.3318	−1.1764	−
0.0883	0.7377	−0.9884	−3.6522	−0.4150
	−0.9357	3.9386	−435.8828	
44.3200	−437.1842	−0.2435	−1.1421	
1.0393	0.9794	−0.8855	−3.6801	−0.3772
	−0.9183	3.9501	−435.8838	
44.3400	−435.5815	−0.4614	−0.3164	
2.1089	1.1670	−0.7882	−3.7026	−0.3385
	−0.9009	3.9616	−435.8849	
44.3600	−432.4912	−0.2772	1.0357	
2.8902	1.2811	−0.6988	−3.7195	−0.2990
	−0.8836	3.9731	−435.8859	
44.3800	−430.5190	0.4334	2.1124	
3.1709	1.3063	−0.6188	−3.7309	−0.2587
	−0.8662	3.9847	−435.8869	
44.4000	−431.2103	0.4782	2.0511	
2.8408	1.2402	−0.5494	−3.7367	−0.2175
	−0.8490	3.9963	−435.8879	
44.4200	−433.0121	−0.4757	0.9920	
1.9739	1.0961	−0.4912	−3.7370	−0.1756
	−0.8317	4.0078	−435.8888	
44.4400	−435.2229	0.6795	−0.3490	
0.7784	0.8946	−0.4444	−3.7317	−0.1328
	−0.8146	4.0194	−435.8898	
44.4600	−438.6551	−0.3853	−1.5273	−
0.4835	0.6580	−0.4092	−3.7208	−0.0893
	−0.7974	4.0310	−435.8907	
44.4800	−440.7809	−0.5611	−1.9438	−
1.5605	0.4088	−0.3855	−3.7045	−0.0450
	−0.7804	4.0427	−435.8917	
44.5000	−440.0950	0.1539	−1.1902	−
2.2976	0.1697	−0.3732	−3.6828	0.0000
	−0.7633	4.0543	−435.8926	
44.5200	−438.7023	0.3261	0.1207	−
2.6422	−0.0382	−0.3720	−3.6558	0.0458
	−0.7464	4.0660	−435.8935	
44.5400	−438.2253	0.4513	0.7023	−
2.5888	−0.2009	−0.3813	−3.6234	0.0923
	−0.7295	4.0777	−435.8944	
44.5600	−438.6580	0.1461	0.1698	−
2.1984	−0.3143	−0.4002	−3.5859	0.1395
	−0.7126	4.0894	−435.8952	
44.5800	−439.1385	−0.1872	−0.5734	−
1.5613	−0.3810	−0.4273	−3.5434	0.1874
	−0.6958	4.1011	−435.8961	
44.6000	−438.8579	−0.5732	−0.8721	−
0.7907	−0.4078	−0.4612	−3.4959	0.2359
	−0.6791	4.1128	−435.8969	
44.6200	−437.3482	−0.1372	−0.5303	−
0.0129	−0.4023	−0.5000	−3.4437	0.2850
	−0.6624	4.1245	−435.8978	

44.6400	-435.8490	0.2207	0.2473	
0.6635	-0.3722	-0.5420	-3.3869	0.3347
	-0.6458	4.1363	-435.8986	
44.6600	-434.8135	-0.2332	0.8034	
1.1515	-0.3258	-0.5854	-3.3256	0.3850
	-0.6293	4.1481	-435.8994	
44.6800	-433.9798	-0.3444	0.7594	
1.3837	-0.2723	-0.6284	-3.2600	0.4358
	-0.6128	4.1598	-435.9002	
44.7000	-433.9338	0.5808	0.4127	
1.3423	-0.2208	-0.6692	-3.1903	0.4872
	-0.5964	4.1717	-435.9010	
44.7200	-435.0084	0.0045	0.0318	
1.0685	-0.1793	-0.7064	-3.1166	0.5391
	-0.5801	4.1835	-435.9017	
44.7400	-435.8830	-0.2565	-0.3260	
0.6562	-0.1550	-0.7383	-3.0392	0.5914
	-0.5639	4.1953	-435.9025	
44.7600	-435.7263	0.1027	-0.3711	
0.2315	-0.1534	-0.7637	-2.9582	0.6442
	-0.5477	4.2072	-435.9032	
44.7800	-435.3501	0.0178	-0.2084	–
0.1038	-0.1764	-0.7814	-2.8738	0.6974
	-0.5316	4.2190	-435.9039	
44.8000	-435.1818	0.3455	-0.1551	–
0.2909	-0.2231	-0.7908	-2.7862	0.7510
	-0.5156	4.2309	-435.9046	
44.8200	-435.1505	0.4640	-0.1533	–
0.3221	-0.2889	-0.7914	-2.6955	0.8050
	-0.4997	4.2428	-435.9053	
44.8400	-435.1155	-0.5052	-0.0635	–
0.2349	-0.3646	-0.7830	-2.6018	0.8594
	-0.4839	4.2547	-435.9060	
44.8600	-434.7933	0.0184	0.0973	–
0.1003	-0.4396	-0.7657	-2.5055	0.9140
	-0.4682	4.2666	-435.9066	
44.8800	-434.0793	0.2991	0.2544	–
0.0012	-0.5018	-0.7398	-2.4066	0.9689
	-0.4525	4.2786	-435.9073	
44.9000	-433.8072	0.1413	0.3464	–
0.0048	-0.5394	-0.7056	-2.3052	1.0241
	-0.4370	4.2905	-435.9079	
44.9200	-433.9739	0.4013	0.4441	–
0.1347	-0.5434	-0.6640	-2.2016	1.0795
	-0.4215	4.3025	-435.9085	
44.9400	-434.2326	0.1206	0.4098	–
0.3561	-0.5096	-0.6158	-2.0958	1.1351
	-0.4062	4.3145	-435.9091	
44.9600	-434.4959	-0.3018	0.0063	–
0.5758	-0.4383	-0.5622	-1.9879	1.1909
	-0.3909	4.3265	-435.9097	

44.9800 -434.7928 0.3063 -0.7037 -
0.6946 -0.3330 -0.5048 -1.8781 1.2469
-0.3758 4.3385 -435.9103
45.0000 -434.8435 -0.4553 -0.9373 -
0.6427 -0.2000 -0.4451 -1.7665 1.3030
-0.3607 4.3505 -435.9108
45.0200 -433.3374 0.3492 -0.4067 -
0.4183 -0.0476 -0.3845 -1.6532 1.3591
-0.3457 4.3626 -435.9114
45.0400 -431.5998 0.4918 0.2812 -
0.0784 0.1144 -0.3245 -1.5382 1.4153
-0.3309 4.3746 -435.9119
45.0600 -431.2930 0.2235 0.5231
0.3029 0.2746 -0.2665 -1.4217 1.4716
-0.3162 4.3867 -435.9124
45.0800 -431.2126 -0.4508 0.2264
0.6396 0.4204 -0.2118 -1.3037 1.5279
-0.3015 4.3988 -435.9129
45.1000 -430.9634 -0.1951 -0.0326
0.8475 0.5408 -0.1615 -1.1844 1.5841
-0.2870 4.4109 -435.9134
45.1200 -429.4217 -0.0691 0.1740
0.8768 0.6293 -0.1165 -1.0639 1.6403
-0.2726 4.4230 -435.9139
45.1400 -428.1234 0.3627 0.5436
0.7248 0.6840 -0.0774 -0.9422 1.6964
-0.2583 4.4351 -435.9144
45.1600 -428.8816 0.2721 0.5161
0.4289 0.7062 -0.0443 -0.8196 1.7524
-0.2442 4.4473 -435.9148
45.1800 -430.3373 -0.0646 0.0502
0.0568 0.6989 -0.0174 -0.6960 1.8082
-0.2301 4.4594 -435.9152
45.2000 -430.5855 -0.6773 -0.3771 -
0.2895 0.6663 0.0035 -0.5716 1.8639
-0.2162 4.4716 -435.9157
45.2200 -430.4993 0.0577 -0.4939 -
0.5281 0.6116 0.0187 -0.4465 1.9194
-0.2024 4.4838 -435.9161
45.2400 -430.3793 -0.0544 -0.4947 -
0.6190 0.5380 0.0288 -0.3208 1.9747
-0.1887 4.4960 -435.9165
45.2600 -430.2186 0.1676 -0.4114 -
0.5657 0.4492 0.0341 -0.1946 2.0297
-0.1752 4.5082 -435.9168
45.2800 -429.9674 0.4199 -0.1148 -
0.4021 0.3494 0.0353 -0.0680 2.0845
-0.1617 4.5204 -435.9172
45.3000 -429.2577 -0.3476 0.2707 -
0.1675 0.2414 0.0331 0.0589 2.1389
-0.1485 4.5326 -435.9175

45.3200	-428.1567	0.3644	0.4655	
0.0836	0.1264	0.0285	0.1860	2.1930
	-0.1353	4.5449	-435.9179	
45.3400	-428.0115	0.1927	0.2802	
0.2888	0.0060	0.0225	0.3133	2.2467
	-0.1223	4.5572	-435.9182	
45.3600	-428.7177	0.1122	0.0274	
0.3914	-0.1177	0.0160	0.4406	2.3001
	-0.1094	4.5694	-435.9185	
45.3800	-429.0834	-0.6270	0.1221	
0.3423	-0.2418	0.0100	0.5678	2.3530
	-0.0967	4.5817	-435.9188	
45.4000	-428.2770	-0.1957	0.5777	
0.1457	-0.3645	0.0051	0.6949	2.4055
	-0.0841	4.5940	-435.9191	
45.4200	-427.4304	0.1512	0.9171	-
0.1362	-0.4835	0.0021	0.8217	2.4574
	-0.0716	4.6064	-435.9193	
45.4400	-428.2007	0.5696	0.4992	-
0.3823	-0.5965	0.0014	0.9481	2.5089
	-0.0593	4.6187	-435.9196	
45.4600	-429.8695	-0.1967	-0.7295	-
0.4708	-0.7006	0.0036	1.0740	2.5599
	-0.0472	4.6310	-435.9198	
45.4800	-430.1805	0.1672	-1.9188	-
0.3364	-0.7920	0.0089	1.1994	2.6102
	-0.0352	4.6434	-435.9200	
45.5000	-430.0317	0.0534	-2.0044	
0.0223	-0.8672	0.0174	1.3240	2.6600
	-0.0233	4.6558	-435.9202	
45.5200	-429.6172	-1.6061	-0.5544	
0.5140	-0.9225	0.0290	1.4479	2.7092
	-0.0116	4.6682	-435.9204	
45.5400	-424.5961	0.5497	1.5306	
0.9597	-0.9537	0.0432	1.5707	2.7577
	-0.0001	4.6806	-435.9206	
45.5600	-419.9641	3.0551	2.5042	
1.1604	-0.9568	0.0597	1.6925	2.8055
	0.0113	4.6930	-435.9208	
45.5800	-423.7503	1.0882	1.3503	
1.0057	-0.9285	0.0780	1.8131	2.8526
	0.0225	4.7054	-435.9209	
45.6000	-430.0868	-2.9147	-0.5140	
0.5604	-0.8640	0.0973	1.9324	2.8990
	0.0336	4.7178	-435.9211	
45.6200	-429.5816	-1.0120	-1.1099	-
0.0363	-0.7598	0.1169	2.0503	2.9446
	0.0445	4.7303	-435.9212	
45.6400	-426.3752	1.7503	-0.6090	-
0.6091	-0.6139	0.1363	2.1665	2.9894
	0.0552	4.7427	-435.9213	

45.6600 -425.7617 1.1904 -0.4596 -
0.9933 -0.4249 0.1546 2.2811 3.0334
0.0658 4.7552 -435.9214
45.6800 -427.5623 -0.0527 -0.8177 -
1.1054 -0.1936 0.1715 2.3938 3.0765
0.0762 4.7677 -435.9215
45.7000 -428.3867 -1.8424 -0.6094 -
0.9756 0.0758 0.1865 2.5045 3.1188
0.0864 4.7802 -435.9215
45.7200 -424.5855 -0.1060 0.7153 -
0.7024 0.3773 0.1993 2.6130 3.1602
0.0965 4.7927 -435.9216
45.7400 -420.9599 1.8545 1.8095 -
0.3927 0.7026 0.2098 2.7192 3.2006
0.1063 4.8052 -435.9216
45.7600 -422.2074 -0.1393 1.3677 -
0.1258 1.0395 0.2179 2.8230 3.2401
0.1160 4.8178 -435.9216
45.7800 -424.2736 -1.2258 0.1796
0.0676 1.3708 0.2238 2.9242 3.2786
0.1256 4.8303 -435.9216
45.8000 -423.2232 -0.6027 -0.5104
0.2147 1.6774 0.2276 3.0227 3.3161
0.1349 4.8429 -435.9216
45.8200 -420.6974 1.8886 -1.0052
0.3818 1.9395 0.2294 3.1183 3.3525
0.1440 4.8555 -435.9216
45.8400 -421.6302 1.7560 -1.9947
0.6201 2.1394 0.2294 3.2109 3.3878
0.1530 4.8681 -435.9216
45.8600 -425.2512 -1.7146 -2.5241
0.9082 2.2630 0.2277 3.3003 3.4221
0.1618 4.8806 -435.9215
45.8800 -424.2206 -3.2609 -1.1806
1.1730 2.3006 0.2245 3.3864 3.4552
0.1704 4.8933 -435.9215
45.9000 -416.2084 1.6128 1.7015
1.3267 2.2451 0.2201 3.4692 3.4872
0.1788 4.9059 -435.9214
45.9200 -414.0869 2.4231 3.8352
1.2825 2.0926 0.2149 3.5485 3.5180
0.1870 4.9185 -435.9213
45.9400 -417.2787 -0.2016 3.5531
0.9753 1.8457 0.2095 3.6241 3.5476
0.1950 4.9312 -435.9212
45.9600 -420.3285 -1.4125 1.5597
0.4363 1.5138 0.2046 3.6961 3.5759
0.2028 4.9438 -435.9211
45.9800 -422.9954 0.1369 -0.7658 -
0.1977 1.1110 0.2009 3.7643 3.6030
0.2104 4.9565 -435.9209

46.0000 -425.6119 0.2024 -2.6408 -
0.7658 0.6533 0.1990 3.8287 3.6288
0.2179 4.9692 -435.9208
46.0200 -428.4855 -0.3949 -3.7760 -
1.1057 0.1584 0.1997 3.8891 3.6533
0.2251 4.9818 -435.9206
46.0400 -429.3643 -1.0771 -3.6058 -
1.1054 -0.3550 0.2036 3.9455 3.6764
0.2321 4.9945 -435.9205
46.0600 -426.4842 -0.3186 -1.7092 -
0.7869 -0.8671 0.2112 3.9979 3.6981
0.2389 5.0072 -435.9203
46.0800 -423.1455 0.5785 0.9634 -
0.2781 -1.3600 0.2231 4.0463 3.7185
0.2455 5.0200 -435.9201
46.1000 -421.0599 -0.2090 2.9940
0.2477 -1.8154 0.2400 4.0905 3.7374
0.2519 5.0327 -435.9198
46.1200 -419.7466 0.9521 3.2538
0.6257 -2.2157 0.2624 4.1306 3.7548
0.2580 5.0454 -435.9196
46.1400 -422.0195 0.5887 1.6667
0.7530 -2.5449 0.2908 4.1665 3.7708
0.2640 5.0582 -435.9194
46.1600 -426.3003 -1.1577 -0.5771
0.6202 -2.7908 0.3257 4.1982 3.7852
0.2697 5.0710 -435.9191
46.1800 -426.5412 -0.2198 -1.8342
0.2745 -2.9450 0.3672 4.2257 3.7982
0.2753 5.0837 -435.9188
46.2000 -426.3296 0.3423 -1.4631 -
0.1913 -3.0020 0.4150 4.2489 3.8095
0.2806 5.0965 -435.9185
46.2200 -425.9541 -0.4325 -0.3168 -
0.6525 -2.9589 0.4686 4.2678 3.8193
0.2856 5.1093 -435.9182
46.2400 -425.2397 0.2673 0.5540 -
1.0200 -2.8142 0.5272 4.2824 3.8275
0.2905 5.1221 -435.9179
46.2600 -425.0215 0.4453 0.5710 -
1.2737 -2.5673 0.5896 4.2926 3.8341
0.2952 5.1349 -435.9176
46.2800 -425.2564 -0.3741 0.0968 -
1.4368 -2.2208 0.6542 4.2984 3.8391
0.2996 5.1478 -435.9173
46.3000 -425.3789 -0.6905 0.0913 -
1.5365 -1.7832 0.7196 4.2999 3.8424
0.3038 5.1606 -435.9169
46.3200 -423.9152 0.2510 0.5135 -
1.5436 -1.2690 0.7842 4.2969 3.8440
0.3077 5.1735 -435.9165

46.3400 -422.5099 0.2147 0.4033 -
1.3703 -0.6983 0.8464 4.2894 3.8441
0.3115 5.1863 -435.9161
46.3600 -422.5398 0.0087 -0.4281 -
0.9658 -0.0936 0.9045 4.2775 3.8424
0.3150 5.1992 -435.9157
46.3800 -422.5971 -0.4095 -1.2373 -
0.3318 0.5219 0.9571 4.2610 3.8391
0.3183 5.2121 -435.9153
46.4000 -421.1936 -0.6031 -1.2325
0.4597 1.1232 1.0024 4.2401 3.8341
0.3213 5.2249 -435.9149
46.4200 -417.9533 0.6097 -0.5284
1.2611 1.6843 1.0389 4.2145 3.8274
0.3242 5.2378 -435.9145
46.4400 -416.6443 0.2406 0.3523
1.8948 2.1798 1.0650 4.1844 3.8191
0.3268 5.2507 -435.9140
46.4600 -415.8993 -0.9650 1.3859
2.1827 2.5855 1.0790 4.1497 3.8091
0.3292 5.2636 -435.9135
46.4800 -414.2405 -0.1535 2.3126
2.0239 2.8824 1.0795 4.1104 3.7975
0.3313 5.2766 -435.9130
46.5000 -412.3719 2.1238 2.0012
1.4531 3.0604 1.0649 4.0667 3.7843
0.3333 5.2895 -435.9125
46.5200 -416.1377 0.8668 0.0142
0.6353 3.1199 1.0342 4.0185 3.7696
0.3350 5.3024 -435.9120
46.5400 -422.0395 -2.0832 -2.0847 -
0.1882 3.0671 0.9874 3.9660 3.7532
0.3365 5.3154 -435.9115
46.5600 -421.9052 -0.3006 -2.5594 -
0.7736 2.9094 0.9246 3.9092 3.7353
0.3378 5.3283 -435.9110
46.5800 -421.3915 0.2921 -1.5122 -
0.9760 2.6561 0.8470 3.8483 3.7159
0.3388 5.3413 -435.9104
46.6000 -420.0939 0.3679 0.0271 -
0.8052 2.3200 0.7561 3.7834 3.6950
0.3397 5.3543 -435.9098
46.6200 -418.5732 1.0708 0.9873 -
0.3866 1.9167 0.6535 3.7145 3.6727
0.3403 5.3672 -435.9093
46.6400 -419.7304 0.7440 0.8663
0.0989 1.4649 0.5412 3.6419 3.6488
0.3407 5.3802 -435.9087
46.6600 -422.1109 -2.0221 0.4378
0.4625 0.9850 0.4210 3.5655 3.6236
0.3410 5.3932 -435.9081

46.6800 -421.5672 -0.3078 0.6241
0.5591 0.4972 0.2949 3.4855 3.5969
0.3410 5.4062 -435.9074
46.7000 -419.8884 1.6914 1.0873
0.3533 0.0199 0.1650 3.4021 3.5689
0.3408 5.4192 -435.9068
46.7200 -421.6348 0.7504 0.8020 -
0.1012 -0.4325 0.0330 3.3152 3.5396
0.3404 5.4322 -435.9061
46.7400 -426.9503 -1.6961 -0.2265 -
0.6871 -0.8477 -0.0989 3.2250 3.5089
0.3397 5.4452 -435.9055
46.7600 -427.7020 -0.1428 -0.9450 -
1.2367 -1.2156 -0.2288 3.1316 3.4769
0.3389 5.4582 -435.9048
46.7800 -427.3325 0.6228 -0.8308 -
1.5885 -1.5274 -0.3546 3.0350 3.4436
0.3379 5.4712 -435.9041
46.8000 -427.3148 0.4017 -0.6543 -
1.6289 -1.7773 -0.4743 2.9355 3.4091
0.3367 5.4843 -435.9034
46.8200 -428.5976 -0.4557 -0.8276 -
1.3391 -1.9627 -0.5858 2.8330 3.3734
0.3353 5.4973 -435.9027
46.8400 -429.4985 -1.0835 -0.5925 -
0.7824 -2.0838 -0.6873 2.7277 3.3365
0.3337 5.5103 -435.9019
46.8600 -426.9901 -0.1441 0.3599 -
0.0934 -2.1439 -0.7770 2.6196 3.2984
0.3319 5.5234 -435.9012
46.8800 -423.7333 0.7885 1.2476
0.5825 -2.1481 -0.8534 2.5089 3.2592
0.3299 5.5364 -435.9004
46.9000 -424.3487 0.4930 1.2493
1.1268 -2.1014 -0.9153 2.3957 3.2188
0.3278 5.5495 -435.8997
46.9200 -426.4576 -1.0068 0.3346
1.4638 -2.0080 -0.9615 2.2800 3.1774
0.3254 5.5625 -435.8989
46.9400 -426.9000 -0.2290 -0.6157
1.5752 -1.8718 -0.9914 2.1620 3.1349
0.3229 5.5756 -435.8981
46.9600 -426.9000 -0.3448 -0.9356
1.4713 -1.6964 -1.0048 2.0417 3.0913
0.3201 5.5886 -435.8972
46.9800 -426.8404 0.2685 -0.5479
1.1863 -1.4864 -1.0018 1.9193 3.0468
0.3172 5.6017 -435.8964
47.0000 -425.9576 -0.3546 0.4346
0.7550 -1.2461 -0.9829 1.7948 3.0012
0.3142 5.6148 -435.8956

47.0200 -425.3001 0.5251 1.0824
0.1967 -0.9787 -0.9487 1.6683 2.9547
0.3109 5.6278 -435.8947
47.0400 -426.7230 0.6683 0.6639 -
0.4417 -0.6871 -0.9001 1.5399 2.9073
0.3075 5.6409 -435.8938
47.0600 -428.6462 -0.9088 -0.2525 -
1.0761 -0.3760 -0.8379 1.4099 2.8589
0.3039 5.6540 -435.8929
47.0800 -428.9588 -0.1330 -0.8219 -
1.5993 -0.0527 -0.7634 1.2782 2.8097
0.3001 5.6670 -435.8920
47.1000 -429.0811 0.1173 -0.6807 -
1.8820 0.2727 -0.6776 1.1450 2.7596
0.2962 5.6801 -435.8911
47.1200 -428.7716 -0.6781 -0.1511 -
1.8229 0.5895 -0.5816 1.0106 2.7087
0.2921 5.6932 -435.8902
47.1400 -426.7981 0.5668 0.0980 -
1.3895 0.8854 -0.4764 0.8749 2.6570
0.2878 5.7062 -435.8892
47.1600 -425.7089 1.1092 -0.3225 -
0.6262 1.1460 -0.3631 0.7382 2.6044
0.2834 5.7193 -435.8883
47.1800 -426.2934 -0.0537 -0.9573
0.3337 1.3547 -0.2425 0.6007 2.5512
0.2788 5.7324 -435.8873
47.2000 -426.8884 -1.7871 -0.6457
1.2870 1.4939 -0.1161 0.4626 2.4972
0.2741 5.7454 -435.8863
47.2200 -423.7746 -1.3828 1.0230
1.9954 1.5471 0.0146 0.3239 2.4425
0.2691 5.7585 -435.8853
47.2400 -418.4850 2.4370 2.4230
2.2622 1.5015 0.1480 0.1850 2.3872
0.2641 5.7715 -435.8843
47.2600 -420.4936 2.1522 1.8600
1.9857 1.3534 0.2822 0.0460 2.3311
0.2589 5.7846 -435.8833
47.2800 -427.0622 -2.9465 -0.0377
1.2750 1.1130 0.4152 -0.0929 2.2745
0.2535 5.7977 -435.8822
47.3000 -428.2708 -0.5689 -1.1445
0.3470 0.8001 0.5452 -0.2315 2.2173
0.2480 5.8107 -435.8812
47.3200 -428.1451 1.0983 -0.7800 -
0.5515 0.4379 0.6702 -0.3695 2.1595
0.2424 5.8238 -435.8801
47.3400 -428.2590 0.5264 -0.2965 -
1.2037 0.0503 0.7883 -0.5067 2.1011
0.2366 5.8368 -435.8790

47.3600 -429.9553 0.0705 -0.5342 -
1.4765 -0.3385 0.8975 -0.6430 2.0423
0.2307 5.8499 -435.8779

47.3800 -431.1998 -0.9389 -0.6752 -
1.3653 -0.7041 0.9960 -0.7782 1.9829
0.2246 5.8629 -435.8768

47.4000 -429.9318 -0.4060 -0.0017 -
0.9652 -1.0237 1.0818 -0.9119 1.9231
0.2184 5.8759 -435.8757

47.4200 -428.0500 1.3248 0.7219 -
0.4365 -1.2758 1.1531 -1.0442 1.8628
0.2120 5.8890 -435.8746

47.4400 -428.1875 -0.0122 0.5743
0.0634 -1.4430 1.2084 -1.1748 1.8021
0.2055 5.9020 -435.8734

47.4600 -429.5803 -0.2060 -0.0575
0.4046 -1.5126 1.2462 -1.3035 1.7411
0.1989 5.9150 -435.8723

47.4800 -430.0438 -0.2159 -0.2707
0.5104 -1.4771 1.2656 -1.4302 1.6796
0.1922 5.9280 -435.8711

47.5000 -429.3133 0.1733 0.0889
0.3718 -1.3365 1.2658 -1.5547 1.6179
0.1853 5.9411 -435.8699

47.5200 -428.7059 0.1467 0.5431
0.0453 -1.0995 1.2466 -1.6770 1.5558
0.1783 5.9541 -435.8687

47.5400 -429.1116 -0.0861 0.7554 -
0.3697 -0.7814 1.2084 -1.7968 1.4934
0.1712 5.9671 -435.8675

47.5600 -429.9898 0.0844 0.3556 -
0.7382 -0.4022 1.1521 -1.9140 1.4308
0.1640 5.9800 -435.8662

47.5800 -430.6617 0.1863 -0.5922 -
0.9236 0.0164 1.0786 -2.0286 1.3679
0.1566 5.9930 -435.8650

47.6000 -431.2789 -0.0050 -1.4111 -
0.8236 0.4526 0.9890 -2.1404 1.3049
0.1492 6.0060 -435.8637

47.6200 -431.4076 -0.7801 -1.3605 -
0.4341 0.8850 0.8849 -2.2493 1.2416
0.1416 6.0190 -435.8624

47.6400 -428.6354 0.1745 -0.1695
0.1231 1.2919 0.7677 -2.3552 1.1782
0.1339 6.0319 -435.8611

47.6600 -425.8130 1.2022 1.3177
0.6516 1.6514 0.6387 -2.4579 1.1147
0.1261 6.0449 -435.8598

47.6800 -425.7260 -0.5546 2.0958
0.9659 1.9427 0.4996 -2.5574 1.0510
0.1182 6.0578 -435.8585

47.7000 -425.8269 -0.0777 1.9250
0.9913 2.1486 0.3518 -2.6536 0.9873
0.1102 6.0708 -435.8572
47.7200 -426.4864 0.1119 0.9322
0.7800 2.2578 0.1968 -2.7463 0.9235
0.1020 6.0837 -435.8558
47.7400 -429.2029 0.3213 -0.3939
0.4536 2.2664 0.0365 -2.8355 0.8597
0.0938 6.0966 -435.8545
47.7600 -431.5097 0.1398 -1.6562
0.1717 2.1742 -0.1274 -2.9210 0.7959
0.0855 6.1095 -435.8531
47.7800 -433.2624 -0.0141 -2.6776
0.0719 1.9843 -0.2928 -3.0027 0.7322
0.0771 6.1224 -435.8517
47.8000 -434.2751 -0.6473 -2.6612
0.1774 1.7034 -0.4578 -3.0806 0.6684
0.0685 6.1353 -435.8503
47.8200 -432.6596 -0.3638 -1.0623
0.4076 1.3417 -0.6202 -3.1546 0.6048
0.0599 6.1481 -435.8489
47.8400 -429.1715 0.5643 1.3326
0.6242 0.9129 -0.7780 -3.2244 0.5413
0.0512 6.1610 -435.8475
47.8600 -428.3311 0.3779 3.1886
0.6833 0.4336 -0.9289 -3.2901 0.4779
0.0424 6.1738 -435.8460
47.8800 -429.6679 -0.4341 3.5559
0.4783 -0.0774 -1.0709 -3.3515 0.4146
0.0335 6.1867 -435.8446
47.9000 -431.7703 0.8614 2.0232
0.0413 -0.5996 -1.2019 -3.4086 0.3516
0.0246 6.1995 -435.8431
47.9200 -436.1732 0.1678 -0.8565 -
0.4839 -1.1123 -1.3197 -3.4612 0.2887
0.0155 6.2123 -435.8416
47.9400 -441.2021 -0.9385 -3.1842 -
0.9112 -1.5954 -1.4223 -3.5092 0.2261
0.0064 6.2251 -435.8401
47.9600 -441.5535 -0.5152 -3.0789 -
1.0820 -2.0289 -1.5075 -3.5526 0.1637
-0.0028 6.2378 -435.8386
47.9800 -438.5660 0.2354 -0.9208 -
0.9543 -2.3938 -1.5733 -3.5913 0.1016
-0.0121 6.2506 -435.8371
48.0000 -436.0396 0.3053 1.3163 -
0.6392 -2.6756 -1.6176 -3.6252 0.0398
-0.0215 6.2633 -435.8356
48.0200 -435.9594 0.2508 2.2022 -
0.3095 -2.8617 -1.6387 -3.6543 -0.0217
-0.0309 6.2761 -435.8340

48.0400 -436.1901 0.1588 1.7681 -
0.1073 -2.9436 -1.6351 -3.6785 -0.0828
-0.0404 6.2888 -435.8325
48.0600 -436.5487 -0.4149 0.7339 -
0.0865 -2.9175 -1.6060 -3.6980 -0.1435
-0.0500 6.3015 -435.8309
48.0800 -437.8403 0.1745 -0.3715 -
0.1909 -2.7837 -1.5518 -3.7126 -0.2038
-0.0597 6.3142 -435.8293
48.1000 -439.3787 0.0525 -1.3248 -
0.3081 -2.5462 -1.4740 -3.7226 -0.2638
-0.0694 6.3268 -435.8277
48.1200 -439.5133 -0.3456 -1.8146 -
0.3455 -2.2114 -1.3746 -3.7280 -0.3232
-0.0791 6.3395 -435.8261
48.1400 -438.1501 -0.1916 -1.3131 -
0.2780 -1.7902 -1.2561 -3.7290 -0.3823
-0.0890 6.3521 -435.8244
48.1600 -436.3747 -0.0256 0.0788 -
0.1709 -1.2978 -1.1214 -3.7257 -0.4408
-0.0989 6.3647 -435.8228
48.1800 -434.6927 -0.1296 1.4681 -
0.1173 -0.7517 -0.9730 -3.7184 -0.4989
-0.1088 6.3773 -435.8211
48.2000 -432.8895 -0.0047 1.8777 -
0.1861 -0.1717 -0.8138 -3.7071 -0.5564
-0.1188 6.3899 -435.8194
48.2200 -432.2395 0.9290 1.1282 -
0.3752 0.4195 -0.6463 -3.6922 -0.6134
-0.1289 6.4025 -435.8178
48.2400 -433.9580 0.5247 -0.5089 -
0.5763 0.9979 -0.4731 -3.6737 -0.6698
-0.1390 6.4150 -435.8161
48.2600 -436.0413 -0.5396 -1.9985 -
0.6067 1.5393 -0.2967 -3.6517 -0.7257
-0.1492 6.4275 -435.8143
48.2800 -435.3141 -1.0302 -1.9566 -
0.3356 2.0208 -0.1195 -3.6266 -0.7810
-0.1594 6.4400 -435.8126
48.3000 -431.5018 0.6264 -0.3770
0.2228 2.4222 0.0558 -3.5985 -0.8356
-0.1696 6.4525 -435.8109
48.3200 -429.0357 0.6185 1.1045
0.9176 2.7257 0.2268 -3.5674 -0.8896
-0.1799 6.4650 -435.8091
48.3400 -428.4368 -0.2371 1.3404
1.5443 2.9161 0.3912 -3.5338 -0.9430
-0.1902 6.4774 -435.8073
48.3600 -428.1224 -0.5781 0.7033
1.9429 2.9812 0.5467 -3.4976 -0.9958
-0.2006 6.4898 -435.8056

48.3800 -428.2101 0.0454 0.1501
2.0333 2.9145 0.6915 -3.4592 -1.0478
-0.2110 6.5022 -435.8038
48.4000 -428.6751 0.1275 -0.1875
1.8261 2.7191 0.8242 -3.4187 -1.0991
-0.2215 6.5146 -435.8020
48.4200 -429.4023 0.5794 -0.5382
1.3857 2.4071 0.9442 -3.3763 -1.1497
-0.2319 6.5269 -435.8001
48.4400 -431.0762 -0.1927 -0.7001
0.7835 1.9986 1.0509 -3.3322 -1.1996
-0.2424 6.5392 -435.7983
48.4600 -432.1966 -1.1386 -0.1176
0.0937 1.5178 1.1441 -3.2867 -1.2487
-0.2530 6.5515 -435.7964
48.4800 -431.5669 -0.5802 1.1901 -
0.5833 0.9894 1.2238 -3.2398 -1.2971
-0.2635 6.5638 -435.7946
48.5000 -430.5089 1.1714 1.9686 -
1.1329 0.4379 1.2897 -3.1920 -1.3446
-0.2741 6.5761 -435.7927
48.5200 -431.2976 1.8807 0.9949 -
1.4612 -0.1123 1.3417 -3.1432 -1.3914
-0.2847 6.5883 -435.7908
48.5400 -436.5124 -0.9889 -1.3540 -
1.5243 -0.6374 1.3796 -3.0938 -1.4373
-0.2953 6.6005 -435.7889
48.5600 -439.4891 -1.7181 -3.1538 -
1.2952 -1.1146 1.4033 -3.0438 -1.4823
-0.3060 6.6127 -435.7870
48.5800 -437.9539 0.3190 -2.8893 -
0.7895 -1.5232 1.4127 -2.9935 -1.5266
-0.3166 6.6248 -435.7851
48.6000 -435.2060 0.7332 -0.9813 -
0.1438 -1.8448 1.4076 -2.9430 -1.5699
-0.3273 6.6370 -435.7831
48.6200 -433.0588 -0.3193 1.3194
0.4467 -2.0634 1.3883 -2.8925 -1.6124
-0.3380 6.6491 -435.7812
48.6400 -431.1051 -0.1170 3.1140
0.7762 -2.1657 1.3546 -2.8421 -1.6540
-0.3486 6.6611 -435.7792
48.6600 -429.9366 0.7626 3.4677
0.6919 -2.1451 1.3071 -2.7918 -1.6946
-0.3593 6.6732 -435.7772
48.6800 -432.1329 0.5244 1.8627
0.1955 -2.0071 1.2463 -2.7418 -1.7344
-0.3700 6.6852 -435.7752
48.7000 -437.0253 -1.0541 -0.6492 -
0.5379 -1.7665 1.1734 -2.6920 -1.7732
-0.3807 6.6972 -435.7732

48.7200 -438.2750 -0.6105 -2.4242 -
1.2653 -1.4411 1.0895 -2.6425 -1.8111
-0.3915 6.7091 -435.7712
48.7400 -438.0249 0.3318 -2.8448 -
1.7319 -1.0500 0.9960 -2.5934 -1.8480
-0.4022 6.7211 -435.7691
48.7600 -437.5593 0.3516 -2.2931 -
1.7642 -0.6139 0.8941 -2.5446 -1.8840
-0.4129 6.7330 -435.7671
48.7800 -436.0319 -0.9656 -0.9646 -
1.3456 -0.1555 0.7853 -2.4962 -1.9190
-0.4236 6.7448 -435.7650
48.8000 -432.6990 0.3180 0.5311 -
0.5986 0.3024 0.6708 -2.4481 -1.9530
-0.4342 6.7567 -435.7629
48.8200 -430.7846 0.2742 1.2234
0.2972 0.7370 0.5520 -2.4004 -1.9861
-0.4449 6.7685 -435.7608
48.8400 -430.6116 0.2550 0.6873
1.1470 1.1263 0.4304 -2.3530 -2.0183
-0.4556 6.7803 -435.7587
48.8600 -430.4877 -0.6191 -0.1552
1.7672 1.4484 0.3074 -2.3060 -2.0494
-0.4662 6.7920 -435.7566
48.8800 -429.8026 -0.3744 0.0754
2.0265 1.6839 0.1844 -2.2594 -2.0796
-0.4769 6.8037 -435.7545
48.9000 -428.1482 0.2919 1.0969
1.9039 1.8208 0.0626 -2.2132 -2.1088
-0.4875 6.8154 -435.7523
48.9200 -427.6685 0.4445 1.5338
1.4429 1.8578 -0.0566 -2.1673 -2.1370
-0.4981 6.8271 -435.7501
48.9400 -430.7476 0.1485 0.6699
0.7646 1.8022 -0.1721 -2.1218 -2.1643
-0.5087 6.8387 -435.7480
48.9600 -433.7051 -0.9855 -0.6585
0.0317 1.6676 -0.2830 -2.0766 -2.1906
-0.5193 6.8503 -435.7458
48.9800 -434.1354 -0.0013 -1.2166 -
0.6019 1.4700 -0.3888 -2.0319 -2.2160
-0.5298 6.8618 -435.7436
49.0000 -434.3594 0.0077 -0.7225 -
1.0353 1.2267 -0.4890 -1.9875 -2.2403
-0.5403 6.8733 -435.7414
49.0200 -434.3692 -0.0850 0.2107 -
1.2537 0.9559 -0.5834 -1.9434 -2.2637
-0.5508 6.8848 -435.7391
49.0400 -434.1175 0.2701 0.9715 -
1.2894 0.6752 -0.6718 -1.8998 -2.2862
-0.5613 6.8963 -435.7369

49.0600 -433.9059 0.4130 0.9828 -
1.1830 0.3990 -0.7541 -1.8564 -2.3077
-0.5717 6.9077 -435.7346
49.0800 -434.7728 0.1796 -0.0647 -
0.9467 0.1349 -0.8296 -1.8133 -2.3283
-0.5821 6.9190 -435.7324
49.1000 -436.7111 -0.6704 -1.1708 -
0.5857 -0.1144 -0.8976 -1.7705 -2.3479
-0.5925 6.9304 -435.7301
49.1200 -437.2389 -0.8103 -1.1388 -
0.1276 -0.3498 -0.9572 -1.7280 -2.3666
-0.6028 6.9417 -435.7278
49.1400 -434.8070 0.3382 0.1198
0.3522 -0.5722 -1.0074 -1.6856 -2.3844
-0.6131 6.9529 -435.7255
49.1600 -432.2493 0.9937 1.3026
0.7559 -0.7824 -1.0474 -1.6434 -2.4012
-0.6233 6.9641 -435.7232
49.1800 -432.7893 0.1100 1.1163
0.9870 -0.9808 -1.0764 -1.6014 -2.4172
-0.6336 6.9753 -435.7208
49.2000 -435.3993 -0.3719 -0.1332
0.9964 -1.1675 -1.0940 -1.5594 -2.4322
-0.6437 6.9865 -435.7185
49.2200 -436.6845 -1.0481 -0.9516
0.8046 -1.3398 -1.0995 -1.5175 -2.4463
-0.6538 6.9976 -435.7161
49.2400 -435.7048 0.2344 -0.3983
0.4715 -1.4930 -1.0929 -1.4756 -2.4596
-0.6639 7.0086 -435.7137
49.2600 -434.4203 0.3098 0.5889
0.0891 -1.6194 -1.0739 -1.4336 -2.4720
-0.6740 7.0197 -435.7113
49.2800 -434.6870 1.1104 0.5389 -
0.2612 -1.7091 -1.0425 -1.3916 -2.4835
-0.6840 7.0307 -435.7089
49.3000 -437.0885 -0.6686 -0.6364 -
0.5189 -1.7522 -0.9989 -1.3494 -2.4941
-0.6939 7.0416 -435.7065
49.3200 -438.7881 -0.8313 -1.3791 -
0.6570 -1.7386 -0.9433 -1.3071 -2.5039
-0.7038 7.0525 -435.7041
49.3400 -437.0482 -0.5712 -0.4543 -
0.6766 -1.6606 -0.8765 -1.2647 -2.5128
-0.7136 7.0634 -435.7017
49.3600 -433.6776 1.0010 1.1514 -
0.6112 -1.5162 -0.7997 -1.2221 -2.5209
-0.7234 7.0742 -435.6992
49.3800 -432.6118 1.1675 1.6720 -
0.4985 -1.3082 -0.7144 -1.1794 -2.5282
-0.7331 7.0850 -435.6967

49.4000 -434.3258 -0.0002 0.5518 -
0.3484 -1.0439 -0.6223 -1.1365 -2.5346
-0.7428 7.0957 -435.6943
49.4200 -436.0695 -0.8011 -1.0340 -
0.1449 -0.7340 -0.5251 -1.0935 -2.5402
-0.7524 7.1064 -435.6918
49.4400 -435.4689 -0.3182 -1.6639
0.1173 -0.3922 -0.4247 -1.0504 -2.5449
-0.7619 7.1170 -435.6892
49.4600 -433.0783 0.3885 -0.8596
0.3962 -0.0342 -0.3228 -1.0073 -2.5489
-0.7714 7.1276 -435.6867
49.4800 -431.1097 0.4091 0.8116
0.6260 0.3229 -0.2213 -0.9640 -2.5520
-0.7809 7.1382 -435.6842
49.5000 -429.7501 0.0336 2.1832
0.7386 0.6627 -0.1220 -0.9207 -2.5544
-0.7902 7.1487 -435.6816
49.5200 -428.8669 0.1119 2.2289
0.6840 0.9701 -0.0265 -0.8773 -2.5559
-0.7995 7.1592 -435.6791
49.5400 -429.7353 0.3647 0.8683
0.4656 1.2329 0.0635 -0.8339 -2.5567
-0.8087 7.1696 -435.6765
49.5600 -432.5460 -0.4092 -0.9506
0.1582 1.4410 0.1465 -0.7905 -2.5566
-0.8179 7.1800 -435.6739
49.5800 -433.5936 -0.0961 -2.4325 -
0.1298 1.5882 0.2211 -0.7471 -2.5558
-0.8270 7.1903 -435.6713
49.6000 -433.4626 0.3483 -2.9921 -
0.3100 1.6719 0.2861 -0.7038 -2.5542
-0.8360 7.2006 -435.6687
49.6200 -433.1788 -1.0170 -1.8546 -
0.3851 1.6926 0.3407 -0.6607 -2.5518
-0.8449 7.2108 -435.6661
49.6400 -430.9565 -1.2156 1.0017 -
0.3986 1.6531 0.3842 -0.6178 -2.5486
-0.8538 7.2210 -435.6634
49.6600 -426.2965 1.4184 3.6812 -
0.4072 1.5564 0.4166 -0.5752 -2.5447
-0.8626 7.2311 -435.6608
49.6800 -425.4599 1.6366 3.7660 -
0.4456 1.4070 0.4383 -0.5331 -2.5400
-0.8713 7.2412 -435.6581
49.7000 -430.6907 -0.8010 1.1255 -
0.4937 1.2105 0.4499 -0.4915 -2.5345
-0.8799 7.2512 -435.6554
49.7200 -434.5927 -1.3367 -1.9867 -
0.4831 0.9738 0.4523 -0.4506 -2.5283
-0.8885 7.2612 -435.6527

49.7400 -434.5688 0.3199 -3.6944 -
0.3261 0.7037 0.4467 -0.4105 -2.5213
-0.8969 7.2711 -435.6500
49.7600 -434.4728 0.7868 -3.3422
0.0400 0.4092 0.4341 -0.3712 -2.5135
-0.9053 7.2810 -435.6473
49.7800 -433.5183 -0.9952 -1.1184
0.5457 0.1015 0.4159 -0.3330 -2.5050
-0.9136 7.2908 -435.6446
49.8000 -429.0207 -0.1438 1.7281
1.0261 -0.2077 0.3932 -0.2960 -2.4957
-0.9218 7.3006 -435.6418
49.8200 -426.1001 1.3398 3.2113
1.2950 -0.5065 0.3675 -0.2603 -2.4857
-0.9300 7.3103 -435.6391
49.8400 -429.1353 0.3701 2.1801
1.2033 -0.7830 0.3397 -0.2260 -2.4749
-0.9380 7.3200 -435.6363
49.8600 -433.6994 -1.5357 -0.0398
0.7346 -1.0254 0.3110 -0.1932 -2.4634
-0.9459 7.3296 -435.6335
49.8800 -434.2551 -0.2771 -1.0722 -
0.0329 -1.2216 0.2823 -0.1620 -2.4512
-0.9538 7.3391 -435.6307
49.9000 -433.6412 0.1227 -0.3347 -
0.9296 -1.3605 0.2548 -0.1326 -2.4381
-0.9616 7.3486 -435.6279
49.9200 -433.3114 1.1602 0.5114 -
1.7048 -1.4319 0.2295 -0.1051 -2.4244
-0.9693 7.3581 -435.6251
49.9400 -434.9005 0.1596 0.0151 -
2.1270 -1.4271 0.2071 -0.0795 -2.4099
-0.9768 7.3675 -435.6222
49.9600 -437.1013 -1.0523 -1.1479 -
2.0904 -1.3424 0.1884 -0.0560 -2.3946
-0.9843 7.3768 -435.6194
49.9800 -436.9288 -0.7523 -1.3834 -
1.5937 -1.1862 0.1736 -0.0346 -2.3787
-0.9917 7.3861 -435.6165
50.0000 -433.7973 0.4019 -0.4392 -
0.7489 -0.9754 0.1630 -0.0155 -2.3620
-0.9990 7.3953 -435.6136
50.0200 -430.8307 0.2290 0.8184
0.2422 -0.7288 0.1566 0.0013 -2.3445
-1.0062 7.4045 -435.6107
50.0400 -429.3899 -0.2192 1.4392
1.1698 -0.4652 0.1544 0.0158 -2.3264
-1.0133 7.4136 -435.6078
50.0600 -428.3763 0.1331 1.0998
1.8685 -0.2036 0.1567 0.0277 -2.3075
-1.0203 7.4226 -435.6049

50.0800 -428.3829 0.1114 0.0033
2.2758 0.0370 0.1634 0.0372 -2.2879
-1.0273 7.4316 -435.6020
50.1000 -429.4694 0.0590 -1.1748
2.4156 0.2389 0.1744 0.0441 -2.2676
-1.0341 7.4405 -435.5990
50.1200 -430.0893 -0.8871 -1.4716
2.3427 0.3887 0.1896 0.0484 -2.2466
-1.0408 7.4494 -435.5961
50.1400 -428.8832 0.0295 -0.4134
2.0997 0.4787 0.2085 0.0501 -2.2250
-1.0474 7.4582 -435.5931
50.1600 -427.4177 0.3872 1.0287
1.6909 0.5105 0.2307 0.0492 -2.2026
-1.0539 7.4669 -435.5901
50.1800 -427.8089 0.5956 1.4528
1.0783 0.4978 0.2556 0.0456 -2.1795
-1.0603 7.4756 -435.5871
50.2000 -429.5015 0.1478 0.5687
0.2795 0.4577 0.2824 0.0394 -2.1558
-1.0666 7.4842 -435.5841
50.2200 -431.0691 -0.3873 -0.5021 -
0.6258 0.4065 0.3105 0.0305 -2.1314
-1.0728 7.4928 -435.5811
50.2400 -432.7168 0.3526 -0.8146 -
1.5187 0.3553 0.3390 0.0190 -2.1064
-1.0789 7.5013 -435.5781
50.2600 -433.5985 -0.3039 -0.5863 -
2.2673 0.3122 0.3672 0.0049 -2.0806
-1.0849 7.5097 -435.5750
50.2800 -433.5687 0.0092 -0.2241 -
2.7574 0.2842 0.3941 -0.0118 -2.0543
-1.0908 7.5180 -435.5720
50.3000 -433.4763 -0.4983 0.0318 -
2.9124 0.2771 0.4190 -0.0311 -2.0273
-1.0966 7.5263 -435.5689
50.3200 -433.1482 -0.0788 0.3521 -
2.6750 0.2917 0.4412 -0.0528 -1.9996
-1.1023 7.5346 -435.5658
50.3400 -431.7421 0.0138 0.5544 -
2.0340 0.3236 0.4603 -0.0769 -1.9714
-1.1078 7.5427 -435.5627
50.3600 -430.7103 0.4265 -0.0421 -
1.0500 0.3630 0.4760 -0.1033 -1.9425
-1.1133 7.5508 -435.5596
50.3800 -430.7000 -0.0486 -1.1766
0.1527 0.3978 0.4880 -0.1318 -1.9130
-1.1187 7.5588 -435.5565
50.4000 -430.7000 -0.2258 -1.5992
1.3835 0.4169 0.4961 -0.1625 -1.8829
-1.1239 7.5668 -435.5533

50.4200 -429.8190 -2.1482 -0.3391
2.4192 0.4103 0.5001 -0.1951 -1.8522
-1.1290 7.5747 -435.5502
50.4400 -424.8862 0.8497 1.9599
3.0274 0.3692 0.4999 -0.2294 -1.8209
-1.1341 7.5825 -435.5470
50.4600 -422.1452 1.9857 3.1807
3.0010 0.2887 0.4955 -0.2655 -1.7891
-1.1390 7.5903 -435.5438
50.4800 -426.2195 -0.1283 2.1683
2.2650 0.1710 0.4869 -0.3030 -1.7566
-1.1438 7.5980 -435.5406
50.5000 -431.0915 -1.7126 0.2438
1.0144 0.0285 0.4743 -0.3419 -1.7236
-1.1485 7.6056 -435.5374
50.5200 -432.0795 -0.3123 -0.8010 -
0.4345 -0.1236 0.4579 -0.3819 -1.6901
-1.1531 7.6131 -435.5342
50.5400 -432.5867 0.7302 -0.9665 -
1.7061 -0.2697 0.4378 -0.4230 -1.6560
-1.1576 7.6206 -435.5310
50.5600 -433.6162 0.5959 -1.4762 -
2.4506 -0.3944 0.4142 -0.4649 -1.6214
-1.1620 7.6280 -435.5277
50.5800 -435.7213 0.1710 -2.5569 -
2.4888 -0.4845 0.3869 -0.5074 -1.5862
-1.1662 7.6353 -435.5245
50.6000 -437.0861 -0.9911 -2.7605 -
1.8711 -0.5359 0.3560 -0.5504 -1.5505
-1.1704 7.6426 -435.5212
50.6200 -433.7988 -0.9091 -0.8304 -
0.8384 -0.5499 0.3215 -0.5936 -1.5144
-1.1744 7.6498 -435.5180
50.6400 -427.5246 1.1318 1.9746
0.2702 -0.5286 0.2835 -0.6369 -1.4777
-1.1784 7.6569 -435.5147
50.6600 -426.0366 1.0995 3.1697
1.1351 -0.4744 0.2420 -0.6801 -1.4406
-1.1822 7.6639 -435.5114
50.6800 -428.1631 -1.1522 1.8631
1.5618 -0.3903 0.1972 -0.7228 -1.4030
-1.1859 7.6709 -435.5080
50.7000 -430.4559 0.2858 -0.5021
1.5547 -0.2824 0.1491 -0.7650 -1.3649
-1.1895 7.6778 -435.5047
50.7200 -432.0036 0.2918 -2.0862
1.2317 -0.1594 0.0979 -0.8065 -1.3264
-1.1930 7.6846 -435.5014
50.7400 -433.1526 -0.3117 -2.2007
0.7357 -0.0324 0.0437 -0.8470 -1.2874
-1.1963 7.6913 -435.4980

50.7600 -432.5704 -0.9993 -0.9362
0.1866 0.0911 -0.0132 -0.8863 -1.2480
-1.1996 7.6980 -435.4946
50.7800 -429.5054 0.6012 1.0250 -
0.3349 0.2062 -0.0727 -0.9242 -1.2082
-1.2027 7.7046 -435.4913
50.8000 -427.9355 1.3891 2.1683 -
0.7635 0.3090 -0.1346 -0.9606 -1.1681
-1.2057 7.7111 -435.4879
50.8200 -430.3132 -0.0055 1.5517 -
1.0413 0.3976 -0.1986 -0.9952 -1.1275
-1.2086 7.7175 -435.4845
50.8400 -433.1491 -1.2654 -0.1169 -
1.1210 0.4700 -0.2643 -1.0280 -1.0865
-1.2114 7.7238 -435.4810
50.8600 -433.3241 0.4464 -1.4612 -
0.9854 0.5229 -0.3311 -1.0586 -1.0452
-1.2141 7.7301 -435.4776
50.8800 -432.9910 -0.0918 -1.7847 -
0.6239 0.5527 -0.3982 -1.0870 -1.0035
-1.2167 7.7363 -435.4742
50.9000 -432.4241 -0.3043 -1.2479 -
0.0849 0.5554 -0.4648 -1.1130 -0.9614
-1.2191 7.7424 -435.4707
50.9200 -430.4561 -0.1358 -0.0649
0.5173 0.5266 -0.5303 -1.1366 -0.9191
-1.2214 7.7485 -435.4672
50.9400 -428.6215 0.8066 1.1992
1.0340 0.4620 -0.5938 -1.1575 -0.8764
-1.2236 7.7544 -435.4638
50.9600 -428.5095 -0.2952 1.9756
1.2980 0.3589 -0.6547 -1.1756 -0.8334
-1.2257 7.7603 -435.4603
50.9800 -428.5530 -0.2805 1.9353
1.2004 0.2187 -0.7124 -1.1910 -0.7901
-1.2277 7.7661 -435.4568
51.0000 -429.0106 0.7622 0.8523
0.7727 0.0497 -0.7664 -1.2034 -0.7465
-1.2295 7.7718 -435.4532
51.0200 -432.7276 -0.2083 -0.8528
0.1623 -0.1348 -0.8160 -1.2128 -0.7026
-1.2313 7.7775 -435.4497
51.0400 -435.4943 -0.8030 -2.0334 -
0.4459 -0.3200 -0.8608 -1.2192 -0.6584
-1.2329 7.7830 -435.4462
51.0600 -434.6239 -0.3306 -1.5701 -
0.8849 -0.4906 -0.9005 -1.2225 -0.6141
-1.2344 7.7885 -435.4426
51.0800 -433.1049 1.0361 -0.2154 -
1.0667 -0.6324 -0.9347 -1.2226 -0.5694
-1.2357 7.7939 -435.4390

51.1000 -432.7000 0.3431 0.5849 -
0.9807 -0.7348 -0.9629 -1.2195 -0.5246
-1.2370 7.7992 -435.4355
51.1200 -432.7000 -0.6234 0.6519 -
0.6856 -0.7916 -0.9848 -1.2133 -0.4795
-1.2381 7.8044 -435.4319
51.1400 -432.7000 -0.1187 0.4528 -
0.2997 -0.7998 -1.0002 -1.2039 -0.4343
-1.2391 7.8095 -435.4283
51.1600 -432.5225 0.5186 0.1762
0.0702 -0.7582 -1.0089 -1.1913 -0.3888
-1.2400 7.8146 -435.4246
51.1800 -432.0399 -0.1749 -0.1929
0.3585 -0.6682 -1.0109 -1.1756 -0.3432
-1.2408 7.8195 -435.4210
51.2000 -431.4815 -0.6216 -0.4193
0.5289 -0.5369 -1.0061 -1.1568 -0.2975
-1.2414 7.8244 -435.4174
51.2200 -430.6481 0.5430 -0.2387
0.5956 -0.3749 -0.9942 -1.1350 -0.2516
-1.2419 7.8292 -435.4137
51.2400 -430.1079 0.9623 0.0785
0.5988 -0.1935 -0.9754 -1.1103 -0.2055
-1.2423 7.8339 -435.4100
51.2600 -430.2211 -0.6606 0.0890
0.5377 -0.0028 -0.9494 -1.0827 -0.1594
-1.2426 7.8386 -435.4064
51.2800 -430.4501 -0.4684 -0.0994
0.4022 0.1887 -0.9164 -1.0524 -0.1131
-1.2427 7.8431 -435.4027
51.3000 -430.3548 0.0523 0.1299
0.1824 0.3730 -0.8762 -1.0196 -0.0668
-1.2427 7.8475 -435.3990
51.3200 -429.5173 -0.3916 0.7583 -
0.1342 0.5442 -0.8288 -0.9842 -0.0203
-1.2426 7.8519 -435.3952
51.3400 -429.0025 0.4016 0.8294 -
0.5013 0.6979 -0.7742 -0.9465 0.0262
-1.2424 7.8562 -435.3915
51.3600 -430.0920 0.7348 -0.1980 -
0.8035 0.8276 -0.7123 -0.9067 0.0727
-1.2420 7.8604 -435.3878
51.3800 -431.5445 -0.6182 -1.3336 -
0.9042 0.9278 -0.6432 -0.8649 0.1193
-1.2415 7.8645 -435.3840
51.4000 -431.1390 -0.1864 -1.1818 -
0.6960 0.9927 -0.5670 -0.8213 0.1659
-1.2409 7.8685 -435.3802
51.4200 -429.1620 0.5336 -0.3078 -
0.2156 1.0152 -0.4839 -0.7760 0.2126
-1.2401 7.8724 -435.3765

51.4400 -427.8597 0.1813 -0.0178
0.4231 0.9882 -0.3943 -0.7292 0.2592
-1.2392 7.8762 -435.3727
51.4600 -427.4245 0.6747 -0.1271
1.0442 0.9051 -0.2983 -0.6811 0.3058
-1.2382 7.8800 -435.3689
51.4800 -426.9883 -0.7621 0.2670
1.4569 0.7612 -0.1965 -0.6318 0.3524
-1.2370 7.8837 -435.3651
51.5000 -425.8145 -0.5408 1.2191
1.5256 0.5560 -0.0891 -0.5815 0.3990
-1.2357 7.8872 -435.3612
51.5200 -424.4012 1.1614 1.6232
1.2243 0.2957 0.0233 -0.5304 0.4455
-1.2343 7.8907 -435.3574
51.5400 -425.5478 1.6278 0.6488
0.6200 -0.0052 0.1400 -0.4786 0.4919
-1.2327 7.8941 -435.3535
51.5600 -430.8035 -1.1670 -1.0426 -
0.1335 -0.3279 0.2602 -0.4263 0.5383
-1.2310 7.8974 -435.3497
51.5800 -432.7311 -1.8927 -1.5993 -
0.8524 -0.6514 0.3828 -0.3737 0.5846
-1.2292 7.9006 -435.3458
51.6000 -430.1301 0.4997 -0.4701 -
1.3829 -0.9542 0.5070 -0.3208 0.6307
-1.2272 7.9037 -435.3419
51.6200 -427.8393 1.9644 0.6567 -
1.6374 -1.2154 0.6316 -0.2679 0.6767
-1.2251 7.9067 -435.3380
51.6400 -429.2028 0.9951 0.3117 -
1.5818 -1.4178 0.7556 -0.2151 0.7226
-1.2229 7.9096 -435.3341
51.6600 -431.8934 -1.6074 -0.8424 -
1.2306 -1.5501 0.8778 -0.1626 0.7683
-1.2205 7.9125 -435.3302
51.6800 -431.7922 -1.8552 -1.0809 -
0.6479 -1.6084 0.9969 -0.1104 0.8138
-1.2180 7.9152 -435.3262
51.7000 -427.5670 0.9377 -0.1183
0.0573 -1.5930 1.1118 -0.0587 0.8592
-1.2153 7.9179 -435.3223
51.7200 -425.0057 1.9008 0.6359
0.7532 -1.5053 1.2211 -0.0075 0.9043
-1.2125 7.9204 -435.3183
51.7400 -426.3885 -0.0209 0.2096
1.2877 -1.3469 1.3235 0.0429 0.9492
-1.2096 7.9229 -435.3143
51.7600 -428.0868 -1.8647 -0.3348
1.5145 -1.1207 1.4177 0.0925 0.9938
-1.2065 7.9253 -435.3104

51.7800 -426.5040 -1.8330 0.2838
1.3392 -0.8319 1.5020 0.1413 1.0382
-1.2033 7.9275 -435.3064
51.8000 -422.2573 1.2943 1.5214
0.7656 -0.4905 1.5750 0.1892 1.0823
-1.1999 7.9297 -435.3023
51.8200 -422.0477 2.5533 1.6783 -
0.0995 -0.1118 1.6351 0.2360 1.1261
-1.1964 7.9318 -435.2983
51.8400 -427.1243 -1.0707 0.2577 -
1.0417 0.2844 1.6808 0.2817 1.1696
-1.1928 7.9338 -435.2943
51.8600 -429.0990 -1.5793 -1.3160 -
1.8040 0.6776 1.7107 0.3262 1.2127
-1.1890 7.9357 -435.2902
51.8800 -427.6478 0.4672 -1.7742 -
2.1532 1.0489 1.7236 0.3696 1.2555
-1.1850 7.9375 -435.2862
51.9000 -425.9740 0.8149 -1.3488 -
1.9552 1.3816 1.7181 0.4117 1.2978
-1.1809 7.9392 -435.2821
51.9200 -425.2104 0.2310 -0.6671 -
1.2484 1.6609 1.6931 0.4527 1.3398
-1.1767 7.9408 -435.2780
51.9400 -424.4906 -1.0371 0.2625 -
0.2292 1.8741 1.6476 0.4923 1.3814
-1.1723 7.9424 -435.2739
51.9600 -421.8301 -1.4277 1.5288
0.8570 2.0094 1.5809 0.5308 1.4225
-1.1678 7.9438 -435.2698
51.9800 -416.8505 2.4080 2.1170
1.7919 2.0556 1.4925 0.5682 1.4632
-1.1631 7.9451 -435.2657
52.0000 -417.4849 2.3698 0.9672
2.4089 2.0020 1.3827 0.6045 1.5034
-1.1583 7.9464 -435.2616
52.0200 -424.4166 -2.5382 -1.0065
2.6262 1.8417 1.2527 0.6398 1.5430
-1.1533 7.9475 -435.2575
52.0400 -425.8855 -3.1461 -1.7145
2.4383 1.5767 1.1047 0.6742 1.5821
-1.1482 7.9485 -435.2533
52.0600 -421.5966 0.4103 -0.3693
1.8819 1.2197 0.9414 0.7078 1.6207
-1.1429 7.9495 -435.2491
52.0800 -419.8628 2.4745 1.4493
1.0504 0.7939 0.7654 0.7406 1.6586
-1.1375 7.9503 -435.2450
52.1000 -422.6607 0.7764 1.6276
0.0635 0.3276 0.5798 0.7728 1.6960
-1.1319 7.9511 -435.2408

52.1200 -426.0888 -0.0845 0.0025 -
0.9271 -0.1497 0.3872 0.8045 1.7327
-1.1262 7.9517 -435.2366
52.1400 -429.6327 -0.2098 -1.7764 -
1.7224 -0.6094 0.1907 0.8357 1.7688
-1.1203 7.9523 -435.2324
52.1600 -431.9545 -1.6828 -1.9775 -
2.1424 -1.0244 -0.0069 0.8665 1.8041
-1.1143 7.9527 -435.2281
52.1800 -430.3521 -0.4902 -0.5092 -
2.1201 -1.3700 -0.2028 0.8970 1.8388
-1.1081 7.9531 -435.2239
52.2000 -427.0407 1.0708 1.2201 -
1.7421 -1.6286 -0.3943 0.9274 1.8727
-1.1017 7.9534 -435.2197
52.2200 -426.4750 0.3065 1.6442 -
1.1607 -1.7906 -0.5786 0.9576 1.9059
-1.0953 7.9535 -435.2154
52.2400 -428.3732 0.3670 0.5250 -
0.5509 -1.8516 -0.7531 0.9878 1.9383
-1.0886 7.9536 -435.2111
52.2600 -429.4869 -1.3007 -0.5867 -
0.0649 -1.8120 -0.9157 1.0180 1.9700
-1.0818 7.9536 -435.2068
52.2800 -428.0345 -0.1718 -0.4294
0.2299 -1.6805 -1.0647 1.0484 2.0008
-1.0749 7.9535 -435.2026
52.3000 -426.3140 0.3578 0.3582
0.3702 -1.4753 -1.1991 1.0790 2.0307
-1.0677 7.9532 -435.1982
52.3200 -426.8163 1.1493 0.3827
0.4771 -1.2195 -1.3183 1.1098 2.0598
-1.0605 7.9529 -435.1939
52.3400 -428.3796 -0.5540 -0.5435
0.6368 -0.9367 -1.4217 1.1411 2.0880
-1.0530 7.9525 -435.1896
52.3600 -428.5450 -0.7828 -1.2955
0.8599 -0.6485 -1.5089 1.1727 2.1153
-1.0455 7.9520 -435.1853
52.3800 -426.5487 -0.1348 -0.8194
1.0841 -0.3720 -1.5799 1.2049 2.1417
-1.0377 7.9514 -435.1809
52.4000 -424.5653 -0.1835 0.6484
1.2039 -0.1193 -1.6350 1.2377 2.1671
-1.0298 7.9506 -435.1765
52.4200 -423.1022 0.0557 1.9140
1.1089 0.1012 -1.6746 1.2711 2.1915
-1.0218 7.9498 -435.1722
52.4400 -422.2194 1.4732 1.7596
0.7487 0.2853 -1.6995 1.3052 2.2150
-1.0136 7.9489 -435.1678

52.4600 -424.9239 0.1741 -0.1046
0.1971 0.4349 -1.7106 1.3402 2.2374
-1.0052 7.9479 -435.1634
52.4800 -429.9460 -1.7704 -2.1055 -
0.3824 0.5560 -1.7088 1.3760 2.2588
-0.9966 7.9468 -435.1590
52.5000 -429.4825 -0.8971 -2.0749 -
0.8049 0.6569 -1.6956 1.4127 2.2792
-0.9880 7.9456 -435.1546
52.5200 -425.5846 0.9375 -0.3904 -
0.9664 0.7469 -1.6721 1.4503 2.2985
-0.9791 7.9443 -435.1501
52.5400 -424.1940 1.2150 0.7057 -
0.8868 0.8328 -1.6393 1.4889 2.3167
-0.9701 7.9429 -435.1457
52.5600 -425.4779 -0.0785 0.5679 -
0.6764 0.9167 -1.5980 1.5284 2.3338
-0.9609 7.9414 -435.1412
52.5800 -426.3976 -2.1234 0.6000 -
0.5222 0.9985 -1.5487 1.5688 2.3497
-0.9516 7.9398 -435.1368
52.6000 -423.6175 -0.2780 1.4708 -
0.5455 1.0761 -1.4912 1.6100 2.3646
-0.9421 7.9381 -435.1323
52.6200 -420.1081 2.4058 2.0580 -
0.6915 1.1423 -1.4256 1.6519 2.3783
-0.9324 7.9363 -435.1278
52.6400 -422.3627 1.7553 1.0376 -
0.8252 1.1836 -1.3517 1.6945 2.3908
-0.9226 7.9343 -435.1233
52.6600 -427.8145 -2.0261 -1.1974 -
0.7949 1.1839 -1.2694 1.7376 2.4021
-0.9126 7.9323 -435.1188
52.6800 -428.2349 -0.7438 -2.9388 -
0.4608 1.1289 -1.1786 1.7811 2.4122
-0.9025 7.9302 -435.1143
52.7000 -426.4579 -0.3635 -2.6371
0.2333 1.0092 -1.0792 1.8248 2.4212
-0.8922 7.9280 -435.1097
52.7200 -423.7785 -0.6362 -0.1761
1.1519 0.8240 -0.9716 1.8686 2.4289
-0.8817 7.9257 -435.1052
52.7400 -418.4051 0.3967 2.5962
2.0114 0.5775 -0.8558 1.9124 2.4353
-0.8711 7.9233 -435.1006
52.7600 -415.9225 2.6505 3.3710
2.5184 0.2759 -0.7322 1.9559 2.4405
-0.8603 7.9208 -435.0961
52.7800 -420.8412 -0.9699 1.7111
2.4775 -0.0716 -0.6012 1.9991 2.4445
-0.8493 7.9182 -435.0915

52.8000 -424.9128 -1.3785 -0.5419
1.9061 -0.4481 -0.4634 2.0417 2.4471
-0.8382 7.9155 -435.0869
52.8200 -425.1051 -0.4638 -1.3045
0.9069 -0.8318 -0.3197 2.0836 2.4485
-0.8269 7.9127 -435.0823
52.8400 -425.2622 0.6387 -0.5785 -
0.3344 -1.1995 -0.1707 2.1247 2.4486
-0.8154 7.9098 -435.0777
52.8600 -426.1542 0.1648 0.2349 -
1.5299 -1.5282 -0.0175 2.1648 2.4474
-0.8038 7.9068 -435.0730
52.8800 -427.6201 -0.3085 0.2988 -
2.3897 -1.7948 0.1394 2.2037 2.4448
-0.7920 7.9037 -435.0684
52.9000 -428.4457 0.1571 -0.4458 -
2.7362 -1.9781 0.2990 2.2414 2.4410
-0.7801 7.9005 -435.0638
52.9200 -429.0359 0.0519 -1.8025 -
2.5207 -2.0666 0.4603 2.2776 2.4357
-0.7680 7.8972 -435.0591
52.9400 -429.1500 0.2119 -3.0501 -
1.7880 -2.0611 0.6221 2.3121 2.4292
-0.7557 7.8938 -435.0544
52.9600 -428.2070 -0.1611 -3.0032 -
0.6777 -1.9715 0.7831 2.3450 2.4213
-0.7433 7.8903 -435.0497
52.9800 -426.4237 -1.6065 -0.8866
0.5672 -1.8102 0.9422 2.3759 2.4120
-0.7307 7.8867 -435.0450
53.0000 -421.0078 -1.2256 2.4710
1.6610 -1.5901 1.0981 2.4049 2.4014
-0.7179 7.8830 -435.0403
53.0200 -414.1933 2.5537 4.4138
2.3181 -1.3237 1.2495 2.4317 2.3894
-0.7050 7.8793 -435.0356
53.0400 -415.6830 0.8854 3.0914
2.3456 -1.0227 1.3951 2.4563 2.3760
-0.6920 7.8754 -435.0309
53.0600 -421.4306 -0.8243 -0.2211
1.8430 -0.6972 1.5334 2.4785 2.3612
-0.6787 7.8714 -435.0262
53.0800 -423.8524 -0.8045 -2.9641
1.0489 -0.3558 1.6629 2.4982 2.3450
-0.6653 7.8673 -435.0214
53.1000 -425.1219 -0.1370 -3.5531
0.2105 -0.0071 1.7822 2.5153 2.3274
-0.6518 7.8631 -435.0167
53.1200 -424.3306 -0.9940 -1.9990 -
0.4901 0.3413 1.8894 2.5297 2.3084
-0.6381 7.8588 -435.0119

53.1400 -420.0760 1.1925 0.2535 -
0.9633 0.6844 1.9830 2.5412 2.2880
-0.6242 7.8544 -435.0071
53.1600 -418.7396 1.1582 1.4754 -
1.1735 1.0187 2.0614 2.5498 2.2662
-0.6102 7.8499 -435.0023
53.1800 -420.0138 -0.6459 1.1888 -
1.1482 1.3409 2.1230 2.5553 2.2430
-0.5960 7.8453 -434.9975
53.2000 -420.5060 -1.0140 0.5676 -
1.0053 1.6465 2.1664 2.5576 2.2185
-0.5817 7.8405 -434.9927
53.2200 -419.1061 0.7461 0.3509 -
0.8372 1.9293 2.1903 2.5567 2.1925
-0.5672 7.8357 -434.9879
53.2400 -418.3171 0.5768 0.0809 -
0.6538 2.1821 2.1937 2.5526 2.1652
-0.5526 7.8308 -434.9830
53.2600 -418.8434 0.2218 -0.3171 -
0.4340 2.3952 2.1758 2.5453 2.1365
-0.5378 7.8258 -434.9782
53.2800 -419.1876 0.0246 -0.4858 -
0.1423 2.5559 2.1362 2.5348 2.1064
-0.5229 7.8207 -434.9733
53.3000 -418.4449 -0.2684 -0.3780
0.2361 2.6492 2.0749 2.5210 2.0750
-0.5078 7.8155 -434.9685
53.3200 -417.7860 -0.1490 -0.1992
0.6577 2.6604 1.9926 2.5042 2.0423
-0.4926 7.8102 -434.9636
53.3400 -417.6493 0.2051 0.0625
1.0480 2.5783 1.8899 2.4843 2.0084
-0.4772 7.8048 -434.9587
53.3600 -417.6004 -0.1895 0.3665
1.3351 2.3973 1.7681 2.4614 1.9731
-0.4617 7.7993 -434.9538
53.3800 -417.7298 0.0413 0.5673
1.4691 2.1161 1.6295 2.4357 1.9366
-0.4461 7.7937 -434.9489
53.4000 -418.0158 0.0828 0.6455
1.4166 1.7384 1.4762 2.4071 1.8988
-0.4303 7.7880 -434.9439
53.4200 -418.6771 0.1752 0.7001
1.1677 1.2744 1.3111 2.3759 1.8598
-0.4143 7.7822 -434.9390
53.4400 -419.8178 0.5785 0.3229
0.7816 0.7412 1.1367 2.3420 1.8197
-0.3982 7.7763 -434.9341
53.4600 -422.5468 -0.3123 -0.5674
0.3478 0.1600 0.9556 2.3056 1.7784
-0.3820 7.7703 -434.9291

53.4800 -425.1170 -0.7304 -1.3755 -
0.0434 -0.4459 0.7707 2.2667 1.7359
-0.3656 7.7642 -434.9241
53.5000 -425.6321 0.3078 -1.4855 -
0.3388 -1.0496 0.5844 2.2255 1.6923
-0.3491 7.7580 -434.9192
53.5200 -425.8835 -0.1670 -0.5802 -
0.5483 -1.6231 0.3994 2.1821 1.6476
-0.3325 7.7517 -434.9142
53.5400 -425.6123 -0.7005 0.9300 -
0.7405 -2.1379 0.2183 2.1364 1.6018
-0.3157 7.7453 -434.9092
53.5600 -424.8083 0.7220 1.9516 -
0.9917 -2.5669 0.0439 2.0887 1.5550
-0.2988 7.7388 -434.9042
53.5800 -425.2102 1.1020 1.5131 -
1.3407 -2.8885 -0.1214 2.0390 1.5072
-0.2818 7.7322 -434.8991
53.6000 -429.1555 -0.5206 0.1757 -
1.7403 -3.0882 -0.2749 1.9874 1.4583
-0.2646 7.7255 -434.8941
53.6200 -431.2876 -0.6602 -0.7769 -
2.0753 -3.1587 -0.4146 1.9340 1.4085
-0.2473 7.7187 -434.8891
53.6400 -430.6182 0.0117 -0.8092 -
2.1907 -3.0993 -0.5392 1.8789 1.3577
-0.2299 7.7118 -434.8840
53.6600 -429.6131 0.4511 -0.6656 -
1.9481 -2.9171 -0.6478 1.8222 1.3060
-0.2123 7.7048 -434.8790
53.6800 -429.0336 0.0625 -0.9152 -
1.3072 -2.6282 -0.7399 1.7640 1.2533
-0.1946 7.6977 -434.8739
53.7000 -428.5908 0.0161 -1.0278 -
0.3306 -2.2593 -0.8151 1.7045 1.1999
-0.1768 7.6905 -434.8688
53.7200 -427.5731 -1.0850 -0.2379
0.7986 -1.8403 -0.8735 1.6438 1.1455
-0.1589 7.6832 -434.8637
53.7400 -423.9036 0.4384 1.0284
1.8534 -1.4006 -0.9148 1.5820 1.0904
-0.1408 7.6758 -434.8586
53.7600 -421.3070 1.4757 1.3880
2.6315 -0.9689 -0.9391 1.5192 1.0344
-0.1226 7.6683 -434.8535
53.7800 -423.0840 0.0476 0.3696
3.0053 -0.5718 -0.9466 1.4556 0.9777
-0.1043 7.6607 -434.8483
53.8000 -425.4967 -1.5662 -0.6947
2.9724 -0.2302 -0.9375 1.3914 0.9202
-0.0859 7.6530 -434.8432

53.8200 -424.3269 -0.7356 -0.5004
2.6094 0.0439 -0.9125 1.3268 0.8620
-0.0674 7.6452 -434.8381
53.8400 -421.7055 1.5183 0.3470
2.0444 0.2465 -0.8728 1.2619 0.8031
-0.0487 7.6373 -434.8329
53.8600 -422.2999 0.8347 0.5137
1.4244 0.3787 -0.8204 1.1969 0.7435
-0.0300 7.6293 -434.8277
53.8800 -426.0273 -0.6671 -0.2266
0.8402 0.4504 -0.7578 1.1319 0.6833
-0.0111 7.6213 -434.8226
53.9000 -427.0865 -1.3313 -0.7514
0.3045 0.4782 -0.6883 1.0672 0.6224
0.0079 7.6131 -434.8174
53.9200 -426.1492 0.8454 -0.3396 -
0.2120 0.4838 -0.6149 1.0030 0.5610
0.0270 7.6048 -434.8122
53.9400 -425.6168 0.7512 0.2762 -
0.7329 0.4897 -0.5410 0.9394 0.4990
0.0462 7.5964 -434.8069
53.9600 -426.7784 -0.5661 0.4465 -
1.2719 0.5161 -0.4699 0.8766 0.4365
0.0656 7.5879 -434.8017
53.9800 -428.0473 -0.9766 0.3702 -
1.8142 0.5806 -0.4047 0.8147 0.3734
0.0850 7.5793 -434.7965
54.0000 -427.8268 0.1272 0.3705 -
2.2999 0.6954 -0.3486 0.7541 0.3098
0.1045 7.5706 -434.7912
54.0200 -427.2310 0.4553 0.2628 -
2.6427 0.8661 -0.3038 0.6947 0.2458
0.1242 7.5619 -434.7860
54.0400 -427.4033 1.1235 -0.3289 -
2.7551 1.0906 -0.2722 0.6368 0.1813
0.1439 7.5530 -434.7807
54.0600 -429.0855 -0.5537 -1.0313 -
2.5660 1.3597 -0.2547 0.5806 0.1164
0.1638 7.5440 -434.7755
54.0800 -429.9407 -1.6826 -0.8977 -
2.0554 1.6589 -0.2520 0.5261 0.0512
0.1837 7.5349 -434.7702
54.1000 -426.3253 -0.3911 0.2946 -
1.2915 1.9694 -0.2643 0.4736 -0.0145
0.2038 7.5258 -434.7649
54.1200 -422.2453 2.0808 1.3145 -
0.3858 2.2704 -0.2918 0.4230 -0.0804
0.2239 7.5165 -434.7596
54.1400 -422.4889 -0.5970 1.0589
0.5613 2.5406 -0.3344 0.3744 -0.1467
0.2442 7.5071 -434.7543

54.1600 -423.6096 0.3094 -0.2412
1.4356 2.7587 -0.3923 0.3279 -0.2133
0.2645 7.4977 -434.7489
54.1800 -423.7310 -0.1582 -1.3262
2.1231 2.9043 -0.4655 0.2835 -0.2801
0.2850 7.4881 -434.7436
54.2000 -423.3464 -0.2613 -1.2294
2.5187 2.9614 -0.5535 0.2411 -0.3471
0.3055 7.4784 -434.7382
54.2200 -422.6912 -0.7941 0.1890
2.5486 2.9210 -0.6554 0.2006 -0.4144
0.3261 7.4687 -434.7329
54.2400 -421.1957 0.2733 1.8482
2.1876 2.7827 -0.7693 0.1621 -0.4818
0.3468 7.4588 -434.7275
54.2600 -420.6832 2.1114 2.0061
1.4691 2.5561 -0.8930 0.1255 -0.5493
0.3676 7.4489 -434.7221
54.2800 -425.8831 -0.1916 0.2352
0.5296 2.2555 -1.0238 0.0908 -0.6170
0.3885 7.4388 -434.7168
54.3000 -430.2931 -1.0886 -1.7761 -
0.4137 1.8968 -1.1593 0.0579 -0.6848
0.4095 7.4287 -434.7114
54.3200 -430.0464 -0.6443 -2.1609 -
1.1374 1.4970 -1.2969 0.0268 -0.7526
0.4306 7.4185 -434.7059
54.3400 -429.6031 0.6525 -0.8534 -
1.5212 1.0736 -1.4341 -0.0026 -0.8205
0.4517 7.4081 -434.7005
54.3600 -429.3875 0.5083 0.7131 -
1.5746 0.6431 -1.5682 -0.0303 -0.8883
0.4729 7.3977 -434.6951
54.3800 -429.2438 -0.4067 1.6075 -
1.4013 0.2210 -1.6966 -0.0563 -0.9562
0.4942 7.3872 -434.6897
54.4000 -429.2669 0.4528 1.5442 -
1.1241 -0.1808 -1.8166 -0.0807 -1.0239
0.5156 7.3765 -434.6842
54.4200 -430.8252 -0.0243 0.5571 -
0.8218 -0.5550 -1.9252 -0.1036 -1.0916
0.5371 7.3658 -434.6788
54.4400 -432.4169 -0.6442 -0.6786 -
0.4962 -0.9005 -2.0197 -0.1250 -1.1592
0.5586 7.3550 -434.6733
54.4600 -432.5000 0.6835 -1.4401 -
0.1083 -1.2186 -2.0971 -0.1449 -1.2266
0.5803 7.3441 -434.6678
54.4800 -432.5000 0.0562 -1.4799
0.3737 -1.5109 -2.1545 -0.1634 -1.2938
0.6020 7.3331 -434.6623

54.5000 -432.3773 -0.7159 -0.6638
0.8839 -1.7773 -2.1890 -0.1805 -1.3609
0.6237 7.3220 -434.6568
54.5200 -430.5953 0.0213 0.7445
1.2918 -2.0150 -2.1983 -0.1963 -1.4276
0.6456 7.3107 -434.6513
54.5400 -428.8835 0.9465 1.7415
1.4467 -2.2198 -2.1808 -0.2108 -1.4942
0.6675 7.2995 -434.6458
54.5600 -429.8078 0.3625 1.5146
1.2391 -2.3852 -2.1356 -0.2241 -1.5604
0.6895 7.2881 -434.6403
54.5800 -432.4883 -0.7815 0.3682
0.6896 -2.5011 -2.0632 -0.2363 -1.6262
0.7115 7.2766 -434.6347
54.6000 -434.1113 -0.2818 -0.6340 -
0.0661 -2.5548 -1.9641 -0.2476 -1.6917
0.7336 7.2650 -434.6292
54.6200 -434.9970 -0.1544 -0.9579 -
0.8507 -2.5340 -1.8397 -0.2581 -1.7568
0.7558 7.2533 -434.6236
54.6400 -435.4781 0.2036 -0.7104 -
1.4952 -2.4288 -1.6915 -0.2679 -1.8214
0.7780 7.2415 -434.6180
54.6600 -434.8438 -0.3979 -0.0970 -
1.8768 -2.2321 -1.5218 -0.2772 -1.8856
0.8003 7.2297 -434.6125
54.6800 -433.0945 -0.1543 0.4353 -
1.9230 -1.9429 -1.3331 -0.2862 -1.9492
0.8227 7.2177 -434.6069
54.7000 -432.3010 0.6093 0.2947 -
1.6184 -1.5694 -1.1281 -0.2948 -2.0124
0.8451 7.2056 -434.6013
54.7200 -432.3428 -0.0862 -0.5031 -
1.0308 -1.1290 -0.9096 -0.3033 -2.0749
0.8676 7.1935 -434.5957
54.7400 -432.3934 -0.3757 -1.1211 -
0.2854 -0.6445 -0.6804 -0.3119 -2.1369
0.8902 7.1812 -434.5900
54.7600 -431.0752 -0.8817 -0.5532
0.4532 -0.1401 -0.4433 -0.3205 -2.1982
0.9128 7.1689 -434.5844
54.7800 -426.0427 0.9367 0.9574
1.0258 0.3593 -0.2013 -0.3294 -2.2588
0.9354 7.1564 -434.5788
54.8000 -423.6422 0.8940 1.9704
1.3043 0.8283 0.0430 -0.3387 -2.3187
0.9581 7.1439 -434.5731
54.8200 -425.3902 0.0046 1.5443
1.2500 1.2439 0.2866 -0.3485 -2.3779
0.9809 7.1313 -434.5675

54.8400 -427.4507 -0.9841 0.0172
0.9413 1.5883 0.5268 -0.3589 -2.4363
1.0037 7.1186 -434.5618
54.8600 -428.1338 -0.0599 -1.5367
0.5346 1.8496 0.7610 -0.3701 -2.4940
1.0265 7.1057 -434.5561
54.8800 -428.5427 -0.1740 -2.3231
0.1981 2.0211 0.9867 -0.3822 -2.5507
1.0494 7.0928 -434.5504
54.9000 -428.2445 0.0701 -1.9384
0.0377 2.1017 1.2019 -0.3953 -2.6066
1.0724 7.0798 -434.5447
54.9200 -426.3417 -0.3401 -0.4174
0.0433 2.0966 1.4047 -0.4095 -2.6617
1.0953 7.0667 -434.5390
54.9400 -424.6289 0.1541 1.3674
0.1170 2.0159 1.5936 -0.4251 -2.7157
1.1184 7.0535 -434.5333
54.9600 -423.4030 -0.2165 2.5481
0.1613 1.8722 1.7672 -0.4422 -2.7689
1.1414 7.0403 -434.5276
54.9800 -422.7092 1.1969 2.4168
0.1016 1.6792 1.9244 -0.4608 -2.8210
1.1646 7.0269 -434.5218
55.0000 -425.1105 0.5446 0.7330 -
0.0781 1.4518 2.0644 -0.4812 -2.8721
1.1877 7.0134 -434.5161
55.0200 -429.1685 -1.3877 -1.4948 -
0.3170 1.2054 2.1864 -0.5034 -2.9222
1.2109 6.9998 -434.5103
55.0400 -429.5359 0.4430 -2.5227 -
0.5289 0.9535 2.2898 -0.5277 -2.9713
1.2341 6.9862 -434.5046
55.0600 -429.2324 -0.1038 -1.8303 -
0.6388 0.7081 2.3741 -0.5542 -3.0192
1.2574 6.9724 -434.4988
55.0800 -428.6033 -0.5022 -0.0106 -
0.6336 0.4775 2.4391 -0.5829 -3.0661
1.2807 6.9586 -434.4930
55.1000 -426.1141 0.3979 1.9679 -
0.5454 0.2672 2.4847 -0.6139 -3.1118
1.3040 6.9446 -434.4872
55.1200 -424.2150 0.7983 2.6534 -
0.4174 0.0810 2.5109 -0.6473 -3.1563
1.3274 6.9306 -434.4814
55.1400 -426.3003 0.3500 1.2889 -
0.2809 -0.0795 2.5179 -0.6832 -3.1997
1.3508 6.9165 -434.4756
55.1600 -429.8012 -0.9568 -0.9021 -
0.1216 -0.2142 2.5058 -0.7215 -3.2418
1.3742 6.9023 -434.4697

55.1800 -430.1950 0.3135 -2.1386
0.0958 -0.3243 2.4751 -0.7622 -3.2828
1.3976 6.8880 -434.4639
55.2000 -429.6651 -0.0179 -1.9293
0.3737 -0.4103 2.4263 -0.8052 -3.3225
1.4211 6.8736 -434.4581
55.2200 -428.9067 -0.3268 -0.8481
0.6495 -0.4710 2.3599 -0.8505 -3.3610
1.4446 6.8591 -434.4522
55.2400 -427.3082 0.5580 0.4994
0.8258 -0.5047 2.2766 -0.8979 -3.3982
1.4681 6.8445 -434.4464
55.2600 -426.0412 -0.1974 1.6145
0.8096 -0.5096 2.1768 -0.9473 -3.4342
1.4916 6.8298 -434.4405
55.2800 -426.2257 -0.5388 2.2302
0.5339 -0.4850 2.0610 -0.9985 -3.4688
1.5152 6.8150 -434.4346
55.3000 -426.9775 0.5269 1.9282
0.0060 -0.4309 1.9298 -1.0515 -3.5022
1.5387 6.8002 -434.4287
55.3200 -428.5950 0.9876 0.4251 -
0.6317 -0.3480 1.7838 -1.1060 -3.5342
1.5623 6.7852 -434.4228
55.3400 -432.7036 -0.4594 -1.5407 -
1.1875 -0.2381 1.6239 -1.1618 -3.5649
1.5859 6.7702 -434.4169
55.3600 -434.8814 -1.7957 -2.4349 -
1.4881 -0.1042 1.4512 -1.2188 -3.5942
1.6095 6.7550 -434.4110
55.3800 -432.4708 0.1162 -1.5734 -
1.4210 0.0480 1.2670 -1.2766 -3.6221
1.6332 6.7398 -434.4050
55.4000 -429.2150 1.1724 0.1136 -
1.0278 0.2090 1.0730 -1.3353 -3.6487
1.6568 6.7245 -434.3991
55.4200 -428.6597 0.3254 1.1183 -
0.4341 0.3671 0.8706 -1.3944 -3.6739
1.6805 6.7091 -434.3932
55.4400 -428.6133 -0.2563 1.0026
0.2103 0.5105 0.6616 -1.4540 -3.6976
1.7041 6.6936 -434.3872
55.4600 -428.6006 -0.0093 0.5628
0.7754 0.6274 0.4476 -1.5136 -3.7199
1.7278 6.6780 -434.3812
55.4800 -428.6476 0.1812 0.4123
1.1815 0.7070 0.2303 -1.5732 -3.7408
1.7514 6.6623 -434.3753
55.5000 -428.7599 -0.1948 0.3695
1.3974 0.7416 0.0114 -1.6326 -3.7602
1.7751 6.6465 -434.3693

55.5200 -429.2637 0.2356 0.1588
1.4294 0.7269 -0.2075 -1.6915 -3.7781
1.7988 6.6307 -434.3633
55.5400 -430.4601 0.5763 -0.1718
1.2900 0.6637 -0.4245 -1.7498 -3.7945
1.8225 6.6147 -434.3573
55.5600 -431.2729 -0.9026 -0.3172
0.9828 0.5572 -0.6380 -1.8072 -3.8095
1.8461 6.5987 -434.3513
55.5800 -431.6611 0.5081 -0.0103
0.5152 0.4178 -0.8463 -1.8635 -3.8229
1.8698 6.5825 -434.3452
55.6000 -432.1000 0.5143 0.4809 -
0.0836 0.2586 -1.0477 -1.9186 -3.8348
1.8935 6.5663 -434.3392
55.6200 -433.0896 -0.4860 0.7021 -
0.7424 0.0926 -1.2407 -1.9722 -3.8452
1.9171 6.5500 -434.3332
55.6400 -434.5784 -0.3819 0.4950 -
1.3510 -0.0683 -1.4236 -2.0242 -3.8540
1.9408 6.5336 -434.3271
55.6600 -435.8473 0.0943 -0.1355 -
1.7577 -0.2141 -1.5949 -2.0742 -3.8612
1.9644 6.5171 -434.3210
55.6800 -437.0796 0.3686 -1.1004 -
1.8256 -0.3360 -1.7530 -2.1222 -3.8669
1.9880 6.5005 -434.3150
55.7000 -437.9211 -0.8709 -1.7766 -
1.4945 -0.4292 -1.8962 -2.1680 -3.8710
2.0117 6.4838 -434.3089
55.7200 -437.1380 -0.6149 -1.1732 -
0.8168 -0.4955 -2.0231 -2.2112 -3.8735
2.0352 6.4671 -434.3028
55.7400 -433.9952 0.7666 0.3711
0.0327 -0.5413 -2.1325 -2.2518 -3.8744
2.0588 6.4502 -434.2967
55.7600 -431.9109 0.6086 1.6321
0.8283 -0.5733 -2.2233 -2.2894 -3.8737
2.0824 6.4333 -434.2906
55.7800 -431.9279 -0.5263 1.9489
1.3553 -0.5984 -2.2946 -2.3240 -3.8713
2.1059 6.4162 -434.2845
55.8000 -432.0159 0.0744 1.5807
1.4994 -0.6229 -2.3456 -2.3554 -3.8673
2.1294 6.3991 -434.2784
55.8200 -432.3661 0.3431 0.7580
1.2826 -0.6504 -2.3758 -2.3833 -3.8617
2.1529 6.3819 -434.2722
55.8400 -434.4462 0.2193 -0.4729
0.8341 -0.6809 -2.3849 -2.4077 -3.8544
2.1764 6.3646 -434.2661

55.8600 -436.5865 -0.5931 -1.7070
0.3420 -0.7137 -2.3728 -2.4286 -3.8454
2.1998 6.3472 -434.2599
55.8800 -437.4640 0.2278 -2.4428 -
0.0297 -0.7471 -2.3400 -2.4458 -3.8348
2.2232 6.3297 -434.2538
55.9000 -438.0877 0.1512 -2.3485 -
0.1981 -0.7752 -2.2874 -2.4593 -3.8226
2.2466 6.3122 -434.2476
55.9200 -438.2483 -1.6612 -1.1345 -
0.1710 -0.7882 -2.2160 -2.4692 -3.8086
2.2699 6.2945 -434.2414
55.9400 -434.5853 -1.3668 1.1510 -
0.0773 -0.7763 -2.1274 -2.4753 -3.7931
2.2932 6.2768 -434.2352
55.9600 -429.5291 1.7822 3.0614 -
0.0701 -0.7305 -2.0233 -2.4777 -3.7758
2.3164 6.2589 -434.2290
55.9800 -429.2948 3.2881 2.8768 -
0.2609 -0.6438 -1.9055 -2.4764 -3.7570
2.3396 6.2410 -434.2228
56.0000 -435.2496 -0.4381 0.3224 -
0.6271 -0.5142 -1.7763 -2.4714 -3.7365
2.3628 6.2230 -434.2166
56.0200 -439.9973 -2.9067 -2.3198 -
1.0101 -0.3453 -1.6380 -2.4626 -3.7144
2.3859 6.2049 -434.2104
56.0400 -438.2445 -1.4764 -2.6599 -
1.2377 -0.1446 -1.4932 -2.4501 -3.6907
2.4090 6.1867 -434.2041
56.0600 -434.0888 1.5298 -1.0468 -
1.1948 0.0767 -1.3442 -2.4340 -3.6654
2.4320 6.1685 -434.1979
56.0800 -432.1388 1.3462 0.4584 -
0.8298 0.3039 -1.1936 -2.4142 -3.6385
2.4550 6.1501 -434.1917
56.1000 -431.9600 -0.7720 0.4550 -
0.1500 0.5207 -1.0437 -2.3907 -3.6102
2.4779 6.1317 -434.1854
56.1200 -431.8173 -0.4426 -0.3781
0.6752 0.7111 -0.8971 -2.3636 -3.5803
2.5007 6.1131 -434.1791
56.1400 -430.9187 -0.3066 -0.4410
1.3842 0.8599 -0.7559 -2.3329 -3.5489
2.5235 6.0945 -434.1728
56.1600 -428.3157 0.2301 0.5682
1.7512 0.9530 -0.6224 -2.2987 -3.5161
2.5462 6.0758 -434.1666
56.1800 -427.0067 0.2790 1.8260
1.6674 0.9801 -0.4983 -2.2609 -3.4819
2.5689 6.0570 -434.1603

56.2000 -427.5886 -0.3427 2.3299
1.1447 0.9404 -0.3852 -2.2197 -3.4462
2.5915 6.0381 -434.1540
56.2200 -428.7925 0.4715 1.3676
0.3243 0.8441 -0.2842 -2.1750 -3.4092
2.6140 6.0192 -434.1476
56.2400 -431.1516 0.6873 -0.8362 -
0.5344 0.7074 -0.1959 -2.1270 -3.3708
2.6365 6.0001 -434.1413
56.2600 -435.0717 -1.0443 -2.6704 -
1.1698 0.5478 -0.1210 -2.0758 -3.3311
2.6589 5.9810 -434.1350
56.2800 -436.2829 -1.7982 -2.5668 -
1.4036 0.3831 -0.0594 -2.0214 -3.2901
2.6812 5.9618 -434.1286
56.3000 -432.3059 0.2942 -0.5618 -
1.2346 0.2284 -0.0110 -1.9641 -3.2479
2.7034 5.9425 -434.1223
56.3200 -428.0773 2.1053 1.6070 -
0.8109 0.0930 0.0247 -1.9040 -3.2044
2.7256 5.9231 -434.1159
56.3400 -428.0579 0.2513 2.2618 -
0.3257 -0.0164 0.0485 -1.8414 -3.1597
2.7477 5.9036 -434.1096
56.3600 -429.2324 -0.5769 1.4208
0.0324 -0.0946 0.0614 -1.7764 -3.1138
2.7696 5.8841 -434.1032
56.3800 -429.9777 -0.6994 0.3743
0.1660 -0.1395 0.0652 -1.7095 -3.0667
2.7916 5.8644 -434.0968
56.4000 -430.2368 -0.2602 -0.1718
0.1024 -0.1559 0.0616 -1.6407 -3.0185
2.8134 5.8447 -434.0904
56.4200 -430.4455 0.4721 -0.5175 -
0.0423 -0.1534 0.0529 -1.5705 -2.9692
2.8351 5.8249 -434.0840
56.4400 -430.7619 -0.2048 -0.8871 -
0.1209 -0.1441 0.0414 -1.4992 -2.9189
2.8567 5.8050 -434.0776
56.4600 -431.1033 -0.3722 -1.2505 -
0.0409 -0.1415 0.0294 -1.4270 -2.8675
2.8783 5.7850 -434.0712
56.4800 -431.0721 -0.3538 -1.1860
0.2037 -0.1554 0.0193 -1.3543 -2.8150
2.8997 5.7650 -434.0647
56.5000 -429.5154 0.1926 -0.3956
0.5469 -0.1909 0.0131 -1.2813 -2.7616
2.9211 5.7448 -434.0583
56.5200 -427.8374 0.2384 0.8022
0.8601 -0.2483 0.0130 -1.2084 -2.7072
2.9423 5.7246 -434.0519

56.5400 -427.1414 0.0152 1.7549
1.0030 -0.3249 0.0205 -1.1359 -2.6519
2.9635 5.7043 -434.0454
56.5600 -426.6793 0.2706 1.6222
0.8777 -0.4152 0.0369 -1.0640 -2.5957
2.9845 5.6839 -434.0389
56.5800 -427.2788 1.0822 0.1852
0.4935 -0.5104 0.0637 -0.9929 -2.5386
3.0054 5.6634 -434.0325
56.6000 -431.2971 -0.0596 -1.5008 -
0.0306 -0.6004 0.1018 -0.9230 -2.4806
3.0263 5.6429 -434.0260
56.6200 -433.5738 -1.9590 -1.8827 -
0.5504 -0.6752 0.1521 -0.8543 -2.4218
3.0470 5.6223 -434.0195
56.6400 -430.9843 -0.0603 -0.5136 -
0.9521 -0.7262 0.2152 -0.7871 -2.3622
3.0676 5.6015 -434.0130
56.6600 -427.5412 1.5028 1.1598 -
1.1758 -0.7479 0.2911 -0.7216 -2.3019
3.0881 5.5807 -434.0065
56.6800 -427.5487 1.1197 1.2918 -
1.2058 -0.7379 0.3793 -0.6578 -2.2408
3.1084 5.5599 -434.0000
56.7000 -429.8914 -0.4414 -0.1001 -
1.0671 -0.6973 0.4790 -0.5958 -2.1790
3.1287 5.5389 -433.9935
56.7200 -430.9388 -1.0609 -1.2845 -
0.8001 -0.6301 0.5888 -0.5358 -2.1165
3.1488 5.5178 -433.9869
56.7400 -428.9690 -0.0578 -0.9502 -
0.4374 -0.5435 0.7075 -0.4777 -2.0533
3.1688 5.4967 -433.9804
56.7600 -426.4288 0.4272 0.4098 -
0.0065 -0.4457 0.8336 -0.4218 -1.9896
3.1887 5.4755 -433.9738
56.7800 -425.3831 0.3746 1.3363
0.4640 -0.3456 0.9651 -0.3680 -1.9252
3.2084 5.4542 -433.9673
56.8000 -424.7342 -0.3649 0.9901
0.9307 -0.2516 1.1004 -0.3165 -1.8603
3.2280 5.4329 -433.9607
56.8200 -424.7965 0.7629 -0.3450
1.3251 -0.1701 1.2372 -0.2673 -1.7948
3.2475 5.4114 -433.9542
56.8400 -426.3117 -0.2911 -1.5115
1.5771 -0.1036 1.3737 -0.2204 -1.7288
3.2669 5.3899 -433.9476
56.8600 -427.1539 -1.4505 -1.0933
1.6218 -0.0515 1.5077 -0.1760 -1.6624
3.2861 5.3683 -433.9410

56.8800 -423.6197 -0.2383 0.7724
1.4183 -0.0091 1.6372 -0.1340 -1.5955
3.3051 5.3466 -433.9344
56.9000 -419.8238 2.5504 2.0703
0.9420 0.0303 1.7599 -0.0945 -1.5281
3.3241 5.3248 -433.9278
56.9200 -422.5121 1.0828 1.2108
0.2082 0.0735 1.8739 -0.0575 -1.4604
3.3429 5.3030 -433.9212
56.9400 -427.9977 -1.8869 -0.7141 -
0.6625 0.1274 1.9773 -0.0231 -1.3922
3.3615 5.2811 -433.9145
56.9600 -428.2211 -1.1938 -1.4548 -
1.4964 0.1985 2.0682 0.0087 -1.3238
3.3800 5.2591 -433.9079
56.9800 -426.0798 0.4875 -0.7076 -
2.1154 0.2937 2.1451 0.0378 -1.2550
3.3983 5.2370 -433.9013
57.0000 -425.3320 0.9763 0.0593 -
2.3982 0.4135 2.2066 0.0644 -1.1859
3.4165 5.2148 -433.8946
57.0200 -425.7117 0.2320 -0.0383 -
2.2886 0.5556 2.2517 0.0882 -1.1165
3.4346 5.1926 -433.8880
57.0400 -425.9997 -0.3430 -0.5435 -
1.8240 0.7142 2.2796 0.1095 -1.0470
3.4525 5.1703 -433.8813
57.0600 -424.8404 -0.3499 -0.5137 -
1.1266 0.8820 2.2898 0.1281 -0.9772
3.4702 5.1479 -433.8746
57.0800 -422.6382 -0.2306 0.3802 -
0.3400 1.0501 2.2818 0.1441 -0.9072
3.4877 5.1254 -433.8679
57.1000 -420.8135 -0.5066 1.3515
0.4345 1.2058 2.2552 0.1576 -0.8370
3.5051 5.1028 -433.8612
57.1200 -419.1735 1.2596 1.1945
1.1450 1.3328 2.2099 0.1688 -0.7668
3.5224 5.0802 -433.8545
57.1400 -419.6182 1.0932 -0.2394
1.7683 1.4140 2.1461 0.1777 -0.6964
3.5394 5.0575 -433.8478
57.1600 -422.7931 -1.7795 -1.6503
2.2884 1.4339 2.0642 0.1845 -0.6259
3.5563 5.0347 -433.8411
57.1800 -423.1146 -1.4696 -1.6813
2.6704 1.3812 1.9648 0.1893 -0.5553
3.5731 5.0119 -433.8344
57.2000 -419.2998 0.8515 -0.4904
2.8585 1.2499 1.8491 0.1924 -0.4847
3.5896 4.9890 -433.8277

57.2200 -417.6299 1.6456 0.7413
2.7711 1.0432 1.7183 0.1938 -0.4141
3.6060 4.9660 -433.8209
57.2400 -420.2254 -0.7632 1.3334
2.3315 0.7742 1.5737 0.1939 -0.3436
3.6222 4.9429 -433.8142
57.2600 -421.8935 -1.6728 1.8073
1.4931 0.4627 1.4169 0.1927 -0.2730
3.6382 4.9197 -433.8074
57.2800 -421.5218 -0.1749 2.2621
0.3035 0.1318 1.2492 0.1904 -0.2026
3.6540 4.8965 -433.8007
57.3000 -421.2000 2.2321 1.5790 -
1.0429 -0.1938 1.0721 0.1873 -0.1322
3.6697 4.8732 -433.7939
57.3200 -426.6131 1.1552 -0.7694 -
2.2818 -0.4889 0.8872 0.1834 -0.0620
3.6851 4.8498 -433.7871
57.3400 -434.0175 -2.6531 -3.1384 -
3.1500 -0.7294 0.6961 0.1791 0.0080
3.7004 4.8263 -433.7803
57.3600 -433.4980 -1.3825 -3.3980 -
3.4277 -0.8936 0.5003 0.1744 0.0779
3.7155 4.8028 -433.7735
57.3800 -429.9566 0.9914 -1.6971 -
3.0675 -0.9691 0.3015 0.1696 0.1476
3.7304 4.7792 -433.7667
57.4000 -427.1191 0.7745 0.4031 -
2.2281 -0.9580 0.1014 0.1649 0.2170
3.7451 4.7555 -433.7599
57.4200 -425.0866 0.2170 1.8267 -
1.1659 -0.8692 -0.0986 0.1605 0.2861
3.7596 4.7318 -433.7531
57.4400 -423.6261 -0.0359 2.4779 -
0.1522 -0.7132 -0.2970 0.1566 0.3549
3.7739 4.7079 -433.7463
57.4600 -422.7892 -0.5918 2.6409
0.5760 -0.5017 -0.4925 0.1533 0.4234
3.7880 4.6840 -433.7394
57.4800 -422.2550 0.4347 2.2943
0.9075 -0.2502 -0.6836 0.1510 0.4916
3.8019 4.6601 -433.7326
57.5000 -422.8743 1.1458 1.1665
0.9212 0.0186 -0.8690 0.1499 0.5594
3.8155 4.6360 -433.7257
57.5200 -425.3219 -1.1357 -0.4445
0.7718 0.2782 -1.0472 0.1500 0.6267
3.8290 4.6119 -433.7189
57.5400 -426.5981 -0.1009 -1.9425
0.6262 0.5006 -1.2170 0.1517 0.6937
3.8423 4.5877 -433.7120

57.5600 -427.1887 0.7425 -2.9681
0.6443 0.6588 -1.3770 0.1550 0.7601
3.8553 4.5635 -433.7051
57.5800 -427.3853 -0.6292 -3.0892
0.9186 0.7329 -1.5262 0.1603 0.8261
3.8682 4.5391 -433.6982
57.6000 -425.7573 0.2568 -1.9151
1.3920 0.7183 -1.6631 0.1676 0.8916
3.8808 4.5147 -433.6913
57.6200 -423.1453 -0.2130 0.2412
1.8739 0.6205 -1.7867 0.1770 0.9565
3.8932 4.4902 -433.6844
57.6400 -421.0617 -0.3210 2.4846
2.1476 0.4500 -1.8958 0.1887 1.0209
3.9054 4.4657 -433.6775
57.6600 -419.2622 0.9132 3.6490
2.0288 0.2201 -1.9894 0.2028 1.0847
3.9173 4.4411 -433.6706
57.6800 -420.5266 1.0506 2.8242
1.4337 -0.0517 -2.0663 0.2194 1.1479
3.9290 4.4164 -433.6637
57.7000 -426.9231 -1.0286 0.4571
0.4906 -0.3431 -2.1258 0.2385 1.2105
3.9405 4.3916 -433.6568
57.7200 -429.7371 -0.5848 -1.6545 -
0.5692 -0.6312 -2.1669 0.2602 1.2724
3.9518 4.3668 -433.6498
57.7400 -430.4393 -0.1543 -2.3568 -
1.4924 -0.8934 -2.1889 0.2846 1.3337
3.9628 4.3419 -433.6429
57.7600 -430.7000 -0.2042 -2.0724 -
2.0463 -1.1071 -2.1911 0.3115 1.3942
3.9736 4.3169 -433.6359
57.7800 -430.2078 0.3247 -1.2638 -
2.1385 -1.2539 -2.1731 0.3411 1.4541
3.9842 4.2919 -433.6290
57.8000 -429.1290 -0.3902 -0.0851 -
1.8327 -1.3270 -2.1350 0.3732 1.5133
3.9945 4.2668 -433.6220
57.8200 -427.8039 -0.5204 0.9406 -
1.2610 -1.3276 -2.0772 0.4077 1.5717
4.0046 4.2416 -433.6150
57.8400 -425.9024 0.2266 1.3559 -
0.5823 -1.2630 -2.0009 0.4444 1.6294
4.0144 4.2164 -433.6080
57.8600 -424.9793 -0.1222 1.1134
0.0501 -1.1427 -1.9070 0.4833 1.6863
4.0240 4.1911 -433.6010
57.8800 -424.8980 0.3602 0.4907
0.5348 -0.9788 -1.7970 0.5243 1.7425
4.0333 4.1657 -433.5940

57.9000 -424.8245 -0.6871 0.0189
0.8396 -0.7861 -1.6723 0.5670 1.7978
4.0424 4.1403 -433.5870
57.9200 -424.3315 0.4049 -0.1958
0.9778 -0.5797 -1.5344 0.6115 1.8524
4.0512 4.1147 -433.5800
57.9400 -423.5307 0.0279 -0.4194
0.9862 -0.3724 -1.3850 0.6574 1.9061
4.0598 4.0892 -433.5730
57.9600 -423.5218 0.4193 -0.6525
0.8991 -0.1745 -1.2256 0.7047 1.9590
4.0681 4.0635 -433.5660
57.9800 -424.0187 -0.2078 -0.4846
0.7394 0.0073 -1.0579 0.7532 2.0111
4.0762 4.0378 -433.5589
58.0000 -423.9329 -1.5284 0.3339
0.5032 0.1689 -0.8838 0.8026 2.0623
4.0840 4.0120 -433.5519
58.0200 -420.9496 0.4230 1.3937
0.1848 0.3080 -0.7049 0.8528 2.1127
4.0915 3.9862 -433.5448
58.0400 -419.0253 2.4785 1.3904 -
0.2037 0.4247 -0.5232 0.9036 2.1622
4.0988 3.9603 -433.5378
58.0600 -423.0575 0.1659 -0.2111 -
0.6052 0.5200 -0.3405 0.9549 2.2109
4.1058 3.9343 -433.5307
58.0800 -426.9872 -3.3531 -1.6976 -
0.9197 0.5955 -0.1585 1.0064 2.2586
4.1125 3.9083 -433.5236
58.1000 -424.0813 -0.2137 -1.5372 -
1.0517 0.6529 0.0209 1.0579 2.3055
4.1189 3.8822 -433.5165
58.1200 -419.6611 3.3141 -0.5272 -
0.9831 0.6934 0.1961 1.1094 2.3515
4.1251 3.8560 -433.5094
58.1400 -420.1322 1.2686 0.0006 -
0.7430 0.7174 0.3654 1.1606 2.3965
4.1310 3.8298 -433.5023
58.1600 -422.8145 -2.1876 0.3966 -
0.4094 0.7254 0.5272 1.2114 2.4406
4.1366 3.8035 -433.4952
58.1800 -422.0865 -3.1847 1.3946 -
0.0631 0.7172 0.6805 1.2615 2.4838
4.1419 3.7771 -433.4881
58.2000 -415.8328 2.3290 2.0451
0.2494 0.6906 0.8242 1.3108 2.5261
4.1470 3.7507 -433.4810
58.2200 -414.7952 3.2726 1.0278
0.5123 0.6408 0.9574 1.3591 2.5673
4.1517 3.7242 -433.4739

58.2400 -421.1097 -1.6732 -1.1861
0.7337 0.5633 1.0795 1.4062 2.6077
4.1562 3.6977 -433.4667
58.2600 -423.4052 -2.3484 -2.4708
0.9306 0.4564 1.1896 1.4520 2.6470
4.1604 3.6711 -433.4596
58.2800 -419.8515 0.2798 -1.4346
1.0934 0.3233 1.2872 1.4963 2.6854
4.1643 3.6444 -433.4524
58.3000 -417.4998 0.4741 0.8200
1.1432 0.1711 1.3719 1.5388 2.7228
4.1678 3.6177 -433.4453
58.3200 -417.0491 -0.4049 2.1408
0.9936 0.0085 1.4430 1.5795 2.7592
4.1711 3.5909 -433.4381
58.3400 -416.9356 1.1792 1.6921
0.5957 -0.1551 1.5003 1.6181 2.7946
4.1741 3.5641 -433.4309
58.3600 -420.0303 -0.0825 0.3037 -
0.0198 -0.3073 1.5436 1.6545 2.8290
4.1768 3.5372 -433.4238
58.3800 -422.5988 -1.3843 -0.4501 -
0.7660 -0.4344 1.5728 1.6886 2.8623
4.1792 3.5102 -433.4166
58.4000 -422.0336 0.0976 -0.2659 -
1.4850 -0.5221 1.5878 1.7201 2.8947
4.1812 3.4832 -433.4094
58.4200 -421.4108 0.7847 -0.3598 -
1.9651 -0.5570 1.5891 1.7491 2.9260
4.1830 3.4561 -433.4022
58.4400 -422.9934 0.3160 -1.5581 -
2.0347 -0.5288 1.5769 1.7753 2.9562
4.1844 3.4290 -433.3950
58.4600 -425.7076 -0.5449 -2.7288 -
1.6595 -0.4367 1.5518 1.7987 2.9855
4.1856 3.4018 -433.3877
58.4800 -424.0490 -1.1686 -2.1287 -
0.9168 -0.2891 1.5145 1.8193 3.0137
4.1864 3.3745 -433.3805
58.5000 -418.0726 1.0324 0.1340
0.0013 -0.1016 1.4661 1.8371 3.0408
4.1869 3.3472 -433.3733
58.5200 -415.7883 0.6907 2.3134
0.8506 0.1075 1.4072 1.8520 3.0669
4.1871 3.3198 -433.3661
58.5400 -415.0432 -0.3961 3.0057
1.4002 0.3191 1.3389 1.8639 3.0920
4.1869 3.2924 -433.3588
58.5600 -415.2036 0.3496 2.1676
1.5425 0.5131 1.2621 1.8730 3.1160
4.1865 3.2649 -433.3516

58.5800 -417.4480 0.0946 0.7183
1.3334 0.6711 1.1781 1.8792 3.1390
4.1857 3.2374 -433.3443
58.6000 -418.8934 -0.4127 -0.7313
0.9276 0.7792 1.0877 1.8825 3.1609
4.1846 3.2098 -433.3370
58.6200 -419.5957 0.1619 -1.8540
0.5249 0.8262 0.9921 1.8829 3.1818
4.1832 3.1821 -433.3298
58.6400 -420.2336 0.7717 -2.2266
0.2618 0.8048 0.8922 1.8805 3.2017
4.1814 3.1544 -433.3225
58.6600 -420.9454 -0.4534 -1.6466
0.1914 0.7141 0.7893 1.8752 3.2205
4.1793 3.1267 -433.3152
58.6800 -421.0656 -1.0099 -0.1635
0.2554 0.5605 0.6846 1.8671 3.2383
4.1769 3.0988 -433.3079
58.7000 -418.3713 0.4845 1.4119
0.3511 0.3555 0.5793 1.8562 3.2550
4.1741 3.0710 -433.3006
58.7200 -416.6496 1.5941 1.7759
0.3726 0.1146 0.4750 1.8425 3.2708
4.1710 3.0431 -433.2933
58.7400 -419.9965 0.1744 0.6645
0.2537 -0.1450 0.3730 1.8261 3.2855
4.1676 3.0151 -433.2860
58.7600 -422.9000 -1.5812 -0.5936
0.0075 -0.4046 0.2746 1.8070 3.2992
4.1638 2.9871 -433.2786
58.7800 -422.5448 -0.3364 -0.7901 -
0.2963 -0.6432 0.1813 1.7852 3.3119
4.1597 2.9590 -433.2713
58.8000 -422.1128 0.9218 -0.3774 -
0.5692 -0.8409 0.0943 1.7609 3.3236
4.1553 2.9309 -433.2640
58.8200 -422.6521 -0.1989 -0.3350 -
0.7412 -0.9813 0.0147 1.7339 3.3343
4.1505 2.9027 -433.2566
58.8400 -423.6772 0.1098 -0.3356 -
0.7948 -1.0530 -0.0565 1.7045 3.3441
4.1454 2.8745 -433.2493
58.8600 -423.4462 -0.3536 -0.0229 -
0.7496 -1.0520 -0.1185 1.6726 3.3528
4.1400 2.8462 -433.2419
58.8800 -422.3233 0.2398 0.3950 -
0.6249 -0.9801 -0.1713 1.6383 3.3606
4.1342 2.8179 -433.2345
58.9000 -422.0591 0.2724 0.4886 -
0.4624 -0.8445 -0.2146 1.6017 3.3674
4.1281 2.7895 -433.2272

58.9200 -422.4867 -0.1270 0.1618 -
0.2994 -0.6565 -0.2490 1.5628 3.3733
4.1217 2.7611 -433.2198
58.9400 -422.6912 0.0825 -0.2761 -
0.1624 -0.4305 -0.2748 1.5219 3.3782
4.1149 2.7327 -433.2124
58.9600 -422.4438 -0.5013 -0.4632 -
0.0431 -0.1810 -0.2931 1.4791 3.3822
4.1078 2.7042 -433.2050
58.9800 -422.1032 0.3312 -0.3566
0.0834 0.0762 -0.3048 1.4345 3.3853
4.1003 2.6756 -433.1976
59.0000 -421.8639 0.0933 -0.3075
0.2254 0.3236 -0.3110 1.3882 3.3875
4.0925 2.6470 -433.1902
59.0200 -421.5798 -0.0022 -0.2170
0.3618 0.5443 -0.3128 1.3405 3.3887
4.0844 2.6183 -433.1828
59.0400 -420.8642 0.0317 0.2350
0.4504 0.7236 -0.3114 1.2914 3.3890
4.0759 2.5897 -433.1754
59.0600 -419.8796 0.0856 1.0204
0.4663 0.8498 -0.3076 1.2411 3.3884
4.0671 2.5609 -433.1679
59.0800 -419.5617 -0.4625 1.5889
0.4130 0.9148 -0.3021 1.1898 3.3870
4.0580 2.5321 -433.1605
59.1000 -419.4203 1.4164 0.7982
0.3369 0.9151 -0.2956 1.1376 3.3846
4.0486 2.5033 -433.1530
59.1200 -420.8439 2.2432 -1.3843
0.2938 0.8526 -0.2882 1.0846 3.3814
4.0388 2.4744 -433.1456
59.1400 -427.5939 -2.6598 -3.2658
0.3178 0.7330 -0.2805 1.0309 3.3773
4.0286 2.4455 -433.1381
59.1600 -428.8554 -4.1057 -2.7475
0.4088 0.5667 -0.2724 0.9767 3.3723
4.0182 2.4166 -433.1307
59.1800 -420.6643 0.9690 0.2490
0.4966 0.3690 -0.2640 0.9220 3.3665
4.0074 2.3876 -433.1232
59.2000 -414.3423 4.8526 3.2002
0.4678 0.1576 -0.2554 0.8670 3.3598
3.9963 2.3586 -433.1157
59.2200 -418.5549 0.4726 3.8402
0.2206 -0.0492 -0.2466 0.8117 3.3523
3.9849 2.3295 -433.1083
59.2400 -424.1134 -3.1565 2.6045 -
0.2632 -0.2358 -0.2374 0.7561 3.3440
3.9731 2.3004 -433.1008

59.2600 -424.4787 -0.9696 1.0316 -
0.9146 -0.3915 -0.2277 0.7003 3.3348
3.9610 2.2712 -433.0933
59.2800 -424.6781 1.0196 -0.3637 -
1.5800 -0.5093 -0.2171 0.6444 3.3249
3.9486 2.2420 -433.0858
59.3000 -426.3090 1.8808 -2.0806 -
2.0272 -0.5843 -0.2053 0.5884 3.3141
3.9359 2.2128 -433.0783
59.3200 -429.9891 -0.4346 -3.8221 -
2.0741 -0.6133 -0.1916 0.5324 3.3026
3.9229 2.1836 -433.0707
59.3400 -430.8148 -0.8280 -4.4060 -
1.6396 -0.5997 -0.1752 0.4764 3.2902
3.9095 2.1543 -433.0632
59.3600 -427.8240 -0.1096 -2.9710 -
0.7834 -0.5567 -0.1557 0.4204 3.2771
3.8958 2.1249 -433.0557
59.3800 -423.9414 -0.1392 0.1806
0.2840 -0.4999 -0.1324 0.3645 3.2632
3.8818 2.0955 -433.0482
59.4000 -419.7444 -0.2715 3.4369
1.3065 -0.4444 -0.1051 0.3087 3.2485
3.8675 2.0661 -433.0406
59.4200 -416.2571 0.7768 4.8048
2.0363 -0.4049 -0.0736 0.2532 3.2331
3.8529 2.0367 -433.0331
59.4400 -418.0564 1.2486 3.2032
2.3221 -0.3922 -0.0378 0.1980 3.2169
3.8380 2.0072 -433.0255
59.4600 -423.5768 -1.5249 -0.1833
2.2256 -0.4084 0.0019 0.1433 3.2000
3.8228 1.9777 -433.0179
59.4800 -425.5338 0.1015 -2.8549
1.8741 -0.4507 0.0451 0.0890 3.1823
3.8072 1.9482 -433.0104
59.5000 -426.3239 -0.3928 -3.4562
1.4002 -0.5137 0.0908 0.0354 3.1639
3.7914 1.9186 -433.0028
59.5200 -426.4208 0.1274 -2.4388
0.8956 -0.5894 0.1382 -0.0176 3.1448
3.7752 1.8890 -432.9952
59.5400 -425.4829 0.1965 -0.9609
0.4132 -0.6662 0.1864 -0.0697 3.1249
3.7588 1.8593 -432.9876
59.5600 -424.3106 -0.0651 0.6982 -
0.0838 -0.7269 0.2342 -0.1210 3.1044
3.7420 1.8297 -432.9800
59.5800 -423.1648 -0.2149 2.3249 -
0.6506 -0.7520 0.2808 -0.1713 3.0831
3.7250 1.8000 -432.9724

59.6000 -422.2657 0.5994 2.9932 -
1.3042 -0.7236 0.3251 -0.2205 3.0611
3.7077 1.7702 -432.9648
59.6200 -424.0648 0.5662 1.9335 -
1.9701 -0.6330 0.3662 -0.2686 3.0385
3.6900 1.7405 -432.9572
59.6400 -428.9912 -0.9312 -0.2502 -
2.4845 -0.4806 0.4029 -0.3155 3.0151
3.6721 1.7107 -432.9496
59.6600 -430.0326 -0.1081 -2.1917 -
2.6715 -0.2701 0.4344 -0.3612 2.9911
3.6539 1.6809 -432.9420
59.6800 -429.5064 0.1118 -2.8896 -
2.3925 -0.0097 0.4592 -0.4055 2.9664
3.6354 1.6510 -432.9343
59.7000 -428.5858 -0.5671 -1.7043 -
1.6311 0.2862 0.4763 -0.4484 2.9409
3.6166 1.6211 -432.9267
59.7200 -424.5261 -0.6014 0.6638 -
0.5614 0.5978 0.4846 -0.4899 2.9149
3.5975 1.5912 -432.9190
59.7400 -419.5818 1.7242 2.3462
0.5775 0.9042 0.4831 -0.5297 2.8881
3.5781 1.5613 -432.9114
59.7600 -419.6877 0.3404 2.0583
1.5503 1.1840 0.4709 -0.5680 2.8606
3.5585 1.5314 -432.9037
59.7800 -421.9082 -0.5463 0.5299
2.1783 1.4158 0.4473 -0.6045 2.8325
3.5386 1.5014 -432.8961
59.8000 -422.5358 -0.4918 -0.7055
2.3857 1.5811 0.4118 -0.6393 2.8037
3.5184 1.4714 -432.8884
59.8200 -422.5880 -0.1319 -0.9665
2.1622 1.6692 0.3641 -0.6721 2.7742
3.4979 1.4414 -432.8807
59.8400 -422.5553 -0.5998 -0.0971
1.5644 1.6781 0.3044 -0.7030 2.7441
3.4772 1.4113 -432.8730
59.8600 -421.8769 0.2125 1.1808
0.7313 1.6120 0.2335 -0.7317 2.7133
3.4562 1.3812 -432.8653
59.8800 -421.4122 1.8835 1.3450 -
0.1592 1.4781 0.1525 -0.7581 2.6818
3.4349 1.3511 -432.8576
59.9000 -425.5510 0.6207 -0.1727 -
0.9150 1.2850 0.0628 -0.7822 2.6496
3.4134 1.3210 -432.8499
59.9200 -430.5418 -2.0258 -1.7708 -
1.3673 1.0416 -0.0337 -0.8038 2.6168
3.3916 1.2909 -432.8422

59.9400 -429.4205 -0.8016 -1.6281 -
1.4047 0.7585 -0.1352 -0.8226 2.5833
3.3695 1.2607 -432.8345
59.9600 -426.1318 1.3971 -0.0264 -
1.0467 0.4485 -0.2399 -0.8387 2.5492
3.3472 1.2305 -432.8268
59.9800 -425.3780 1.0987 1.1188 -
0.4426 0.1257 -0.3458 -0.8517 2.5144
3.3246 1.2003 -432.8191
60.0000 -426.8113 -0.0656 0.7041
0.2141 -0.1957 -0.4507 -0.8616 2.4789
3.3017 1.1701 -432.8113
60.0200 -427.8000 -0.9942 -0.0538
0.7288 -0.5021 -0.5526 -0.8683 2.4428
3.2786 1.1399 -432.8036
60.0400 -427.5332 0.1711 0.0367
0.9508 -0.7806 -0.6495 -0.8715 2.4061
3.2553 1.1096 -432.7958
60.0600 -427.1486 0.6012 0.5292
0.8254 -1.0207 -0.7392 -0.8712 2.3687
3.2317 1.0794 -432.7881
60.0800 -427.6298 -0.0977 0.4807
0.4204 -1.2167 -0.8196 -0.8672 2.3307
3.2079 1.0491 -432.7803
60.1000 -429.5926 -0.3839 -0.1181 -
0.0959 -1.3660 -0.8888 -0.8595 2.2920
3.1838 1.0188 -432.7726
60.1200 -430.6729 -0.0240 -0.5877 -
0.5291 -1.4673 -0.9451 -0.8479 2.2528
3.1594 0.9885 -432.7648
60.1400 -430.9520 -0.1047 -0.7531 -
0.7384 -1.5197 -0.9870 -0.8324 2.2129
3.1349 0.9581 -432.7570
60.1600 -431.0918 0.2882 -0.8088 -
0.6637 -1.5237 -1.0134 -0.8129 2.1724
3.1100 0.9278 -432.7492
60.1800 -430.7331 -0.3538 -0.6095 -
0.3282 -1.4817 -1.0237 -0.7894 2.1314
3.0850 0.8974 -432.7414
60.2000 -429.5103 -0.0219 0.0531
0.1322 -1.3991 -1.0179 -0.7619 2.0898
3.0597 0.8670 -432.7337
60.2200 -428.4516 0.1697 0.9866
0.5319 -1.2828 -0.9964 -0.7304 2.0476
3.0342 0.8366 -432.7259
60.2400 -427.5918 -0.1582 1.6828
0.7343 -1.1389 -0.9601 -0.6951 2.0048
3.0084 0.8062 -432.7180
60.2600 -427.0320 0.1119 1.5511
0.6974 -0.9731 -0.9099 -0.6561 1.9615
2.9825 0.7758 -432.7102

60.2800 -428.1244 1.0073 0.2887
0.4580 -0.7889 -0.8471 -0.6134 1.9177
2.9563 0.7454 -432.7024
60.3000 -431.0705 -0.8079 -1.5366
0.1169 -0.5881 -0.7733 -0.5674 1.8734
2.9298 0.7149 -432.6946
60.3200 -431.9071 0.6338 -2.5654 -
0.2049 -0.3716 -0.6902 -0.5182 1.8286
2.9032 0.6845 -432.6868
60.3400 -430.8249 -0.2045 -2.0918 -
0.4295 -0.1410 -0.5997 -0.4659 1.7833
2.8763 0.6540 -432.6789
60.3600 -429.2311 -0.5451 -0.3125 -
0.5487 0.1003 -0.5036 -0.4109 1.7375
2.8492 0.6236 -432.6711
60.3800 -427.3267 -0.1540 1.8772 -
0.6170 0.3453 -0.4038 -0.3532 1.6913
2.8219 0.5931 -432.6632
60.4000 -425.1238 0.2796 3.1019 -
0.6856 0.5852 -0.3022 -0.2932 1.6447
2.7944 0.5626 -432.6554
60.4200 -424.8229 0.9935 2.3497 -
0.7679 0.8110 -0.2005 -0.2309 1.5976
2.7667 0.5321 -432.6475
60.4400 -428.2094 -0.4461 0.0144 -
0.8337 1.0142 -0.1005 -0.1667 1.5502
2.7387 0.5016 -432.6396
60.4600 -430.5998 -0.9747 -2.2294 -
0.8136 1.1862 -0.0038 -0.1006 1.5023
2.7106 0.4711 -432.6318
60.4800 -429.8068 0.5726 -3.0259 -
0.6241 1.3187 0.0883 -0.0330 1.4542
2.6822 0.4406 -432.6239
60.5000 -428.1819 0.1228 -2.3243 -
0.2165 1.4028 0.1744 0.0360 1.4057
2.6537 0.4100 -432.6160
60.5200 -426.5656 -0.2693 -0.9775
0.3734 1.4304 0.2532 0.1061 1.3569
2.6249 0.3795 -432.6081
60.5400 -424.6990 0.0520 0.4552
1.0130 1.3961 0.3239 0.1771 1.3078
2.5960 0.3490 -432.6002
60.5600 -423.2787 0.1606 1.8487
1.5354 1.2977 0.3858 0.2489 1.2584
2.5668 0.3184 -432.5923
60.5800 -422.2701 -0.3177 2.7043
1.7975 1.1357 0.4386 0.3211 1.2088
2.5375 0.2879 -432.5844
60.6000 -421.7110 0.7344 2.1549
1.7316 0.9162 0.4821 0.3934 1.1590
2.5079 0.2573 -432.5765

60.6200 -424.4539 0.9236 0.2527
1.3579 0.6519 0.5166 0.4658 1.1090
2.4782 0.2268 -432.5686
60.6400 -429.6056 -1.3649 -1.6461
0.7760 0.3598 0.5421 0.5379 1.0588
2.4483 0.1962 -432.5607
60.6600 -430.1305 -1.1207 -2.0021
0.1065 0.0585 0.5594 0.6094 1.0084
2.4182 0.1657 -432.5527
60.6800 -429.0373 0.3101 -0.7160 -
0.5558 -0.2334 0.5687 0.6802 0.9579
2.3879 0.1351 -432.5448
60.7000 -428.5147 0.3659 0.7193 -
1.1291 -0.4979 0.5707 0.7500 0.9073
2.3574 0.1046 -432.5369
60.7200 -429.1814 0.6750 1.1137 -
1.5420 -0.7180 0.5658 0.8186 0.8566
2.3267 0.0740 -432.5289
60.7400 -430.2219 -0.5714 0.5458 -
1.7516 -0.8807 0.5547 0.8857 0.8059
2.2959 0.0435 -432.5210
60.7600 -431.0635 0.3112 -0.3475 -
1.6998 -0.9805 0.5378 0.9513 0.7551
2.2649 0.0129 -432.5130
60.7800 -431.8913 -0.3074 -1.3383 -
1.3464 -1.0185 0.5155 1.0150 0.7043
2.2337 -0.0176 -432.5050
60.8000 -432.1542 -0.7027 -1.8135 -
0.7076 -1.0006 0.4885 1.0767 0.6535
2.2024 -0.0482 -432.4971
60.8200 -430.2412 0.3606 -1.2114
0.1116 -0.9354 0.4574 1.1362 0.6027
2.1708 -0.0787 -432.4891
60.8400 -427.6463 0.6601 0.0963
0.9289 -0.8321 0.4227 1.1933 0.5519
2.1391 -0.1092 -432.4811
60.8600 -426.5609 -0.4571 1.3494
1.5323 -0.7004 0.3851 1.2480 0.5012
2.1073 -0.1398 -432.4731
60.8800 -425.8985 -0.3872 2.1276
1.7421 -0.5501 0.3453 1.3000 0.4506
2.0753 -0.1703 -432.4651
60.9000 -425.7557 0.1142 1.9674
1.4783 -0.3904 0.3040 1.3493 0.4001
2.0431 -0.2008 -432.4571
60.9200 -427.2650 -0.1019 0.8739
0.8376 -0.2291 0.2620 1.3958 0.3497
2.0107 -0.2314 -432.4491
60.9400 -429.4530 0.5660 -0.6239
0.0263 -0.0715 0.2202 1.4394 0.2995
1.9782 -0.2619 -432.4411

60.9600 -431.3581 0.3917 -2.0804 -
0.7071 0.0793 0.1793 1.4799 0.2494
1.9456 -0.2924 -432.4331
60.9800 -433.1941 -0.9149 -2.7051 -
1.1393 0.2208 0.1399 1.5173 0.1995
1.9128 -0.3229 -432.4251
61.0000 -433.3653 -1.5282 -1.7156 -
1.1745 0.3499 0.1027 1.5515 0.1498
1.8798 -0.3534 -432.4171
61.0200 -429.4001 0.0120 0.4697 -
0.8874 0.4627 0.0680 1.5825 0.1003
1.8467 -0.3839 -432.4090
61.0400 -425.6839 1.4704 2.0646 -
0.4254 0.5557 0.0364 1.6101 0.0511
1.8135 -0.4143 -432.4010
61.0600 -426.5119 0.4477 1.7812
0.0497 0.6262 0.0084 1.6344 0.0021
1.7801 -0.4448 -432.3930
61.0800 -428.9891 -0.8905 0.3062
0.4086 0.6714 -0.0156 1.6552 -0.0465
1.7465 -0.4753 -432.3849
61.1000 -429.7210 -0.4872 -0.7512
0.5913 0.6867 -0.0354 1.6726 -0.0949
1.7128 -0.5057 -432.3769
61.1200 -428.8483 -0.3352 -0.7506
0.5950 0.6673 -0.0506 1.6866 -0.1430
1.6790 -0.5362 -432.3688
61.1400 -428.1125 0.2974 -0.1282
0.4625 0.6116 -0.0611 1.6972 -0.1907
1.6451 -0.5666 -432.3607
61.1600 -428.3921 0.1682 0.4360
0.2656 0.5212 -0.0668 1.7043 -0.2380
1.6110 -0.5970 -432.3527
61.1800 -429.1066 0.1999 0.6097
0.0582 0.3998 -0.0678 1.7080 -0.2850
1.5768 -0.6274 -432.3446
61.2000 -429.7506 -0.2750 0.2675 -
0.1095 0.2515 -0.0640 1.7083 -0.3317
1.5424 -0.6578 -432.3365
61.2200 -430.4042 -0.4989 -0.2904 -
0.1933 0.0827 -0.0555 1.7053 -0.3779
1.5079 -0.6882 -432.3284
61.2400 -430.8794 0.7839 -0.6909 -
0.1553 -0.0980 -0.0426 1.6990 -0.4237
1.4733 -0.7185 -432.3203
61.2600 -431.1125 -0.0294 -0.8582
0.0075 -0.2803 -0.0254 1.6894 -0.4690
1.4386 -0.7489 -432.3123
61.2800 -431.2690 0.1784 -0.5214
0.2260 -0.4525 -0.0043 1.6767 -0.5140
1.4038 -0.7792 -432.3042

61.3000 -431.1546 -0.6655 0.4227
0.3667 -0.6055 0.0203 1.6609 -0.5584
1.3688 -0.8095 -432.2960
61.3200 -429.9859 0.0914 1.4581
0.3152 -0.7323 0.0479 1.6420 -0.6024
1.3337 -0.8398 -432.2879
61.3400 -429.0222 0.9967 1.6432
0.0474 -0.8264 0.0781 1.6202 -0.6459
1.2985 -0.8701 -432.2798
61.3600 -430.7400 0.2806 0.5172 -
0.3484 -0.8821 0.1103 1.5956 -0.6889
1.2632 -0.9004 -432.2717
61.3800 -434.4554 -0.9474 -1.1676 -
0.7135 -0.8953 0.1439 1.5681 -0.7314
1.2278 -0.9307 -432.2636
61.4000 -435.6313 -0.2655 -2.2740 -
0.8849 -0.8633 0.1781 1.5379 -0.7734
1.1923 -0.9609 -432.2554
61.4200 -434.6251 0.0221 -2.0358 -
0.7697 -0.7853 0.2120 1.5051 -0.8148
1.1566 -0.9911 -432.2473
61.4400 -433.0979 0.4157 -0.6930 -
0.4029 -0.6639 0.2446 1.4696 -0.8557
1.1209 -1.0213 -432.2392
61.4600 -431.5176 -0.2622 0.8067
0.0780 -0.5062 0.2751 1.4317 -0.8960
1.0850 -1.0515 -432.2310
61.4800 -429.6045 0.4529 1.7163
0.5038 -0.3207 0.3025 1.3913 -0.9358
1.0491 -1.0816 -432.2229
61.5000 -428.7015 0.1763 1.8570
0.7329 -0.1163 0.3258 1.3486 -0.9750
1.0131 -1.1118 -432.2147
61.5200 -429.5341 -0.3307 1.3447
0.6846 0.0980 0.3438 1.3034 -1.0135
0.9769 -1.1419 -432.2065
61.5400 -431.0227 0.0446 0.5334
0.3807 0.3142 0.3555 1.2561 -1.0515
0.9407 -1.1720 -432.1984
61.5600 -432.1117 -0.0803 -0.3209 -
0.0565 0.5250 0.3597 1.2065 -1.0889
0.9044 -1.2020 -432.1902
61.5800 -433.1008 0.1947 -1.0722 -
0.4723 0.7230 0.3555 1.1547 -1.1256
0.8680 -1.2321 -432.1820
61.6000 -433.5983 -0.1829 -1.3258 -
0.7220 0.9002 0.3421 1.1009 -1.1617
0.8315 -1.2621 -432.1738
61.6200 -433.2875 0.4280 -1.0075 -
0.7165 1.0475 0.3189 1.0450 -1.1971
0.7949 -1.2921 -432.1657

61.6400 -432.5742 -0.6820 -0.3829 -
0.4598 1.1551 0.2859 0.9872 -1.2319
0.7582 -1.3221 -432.1575
61.6600 -431.8909 0.1727 0.2594 -
0.0406 1.2155 0.2434 0.9275 -1.2660
0.7215 -1.3520 -432.1493
61.6800 -431.0953 -0.0347 0.6981
0.4017 1.2218 0.1920 0.8660 -1.2994
0.6847 -1.3819 -432.1411
61.7000 -430.5366 0.2410 0.8095
0.7496 1.1689 0.1328 0.8027 -1.3322
0.6478 -1.4118 -432.1329
61.7200 -430.8475 0.4843 0.4110
0.9471 1.0543 0.0668 0.7376 -1.3642
0.6108 -1.4417 -432.1246
61.7400 -431.9482 -0.4150 -0.1474
0.9907 0.8794 -0.0043 0.6708 -1.3955
0.5737 -1.4715 -432.1164
61.7600 -432.7692 -0.2838 -0.3901
0.9123 0.6522 -0.0789 0.6023 -1.4260
0.5366 -1.5013 -432.1082
61.7800 -433.0914 0.6129 -0.1834
0.7474 0.3837 -0.1552 0.5322 -1.4558
0.4994 -1.5311 -432.1000
61.8000 -433.3860 -0.4676 0.0564
0.5362 0.0871 -0.2311 0.4604 -1.4849
0.4622 -1.5609 -432.0918
61.8200 -434.1598 0.8643 -0.0666
0.3161 -0.2215 -0.3049 0.3871 -1.5132
0.4249 -1.5906 -432.0835
61.8400 -435.9439 -0.4506 -0.4725
0.0962 -0.5228 -0.3747 0.3122 -1.5407
0.3875 -1.6203 -432.0753
61.8600 -437.1740 0.1038 -0.7102 -
0.1435 -0.7958 -0.4386 0.2359 -1.5674
0.3501 -1.6500 -432.0670
61.8800 -436.9982 -0.3389 -0.1229 -
0.4391 -1.0204 -0.4948 0.1581 -1.5932
0.3126 -1.6796 -432.0588
61.9000 -436.5360 0.2768 1.0278 -
0.7987 -1.1823 -0.5414 0.0789 -1.6183
0.2750 -1.7092 -432.0505
61.9200 -436.3065 0.4245 1.5903 -
1.1878 -1.2707 -0.5770 -0.0017 -1.6425
0.2374 -1.7388 -432.0423
61.9400 -437.7306 -0.1572 0.8256 -
1.5332 -1.2802 -0.6002 -0.0834 -1.6659
0.1998 -1.7683 -432.0340
61.9600 -439.9622 -0.1053 -0.7258 -
1.7267 -1.2107 -0.6103 -0.1664 -1.6884
0.1621 -1.7978 -432.0257

61.9800 -440.6019 -0.6061 -1.6676 -
1.6553 -1.0681 -0.6072 -0.2503 -1.7100
0.1243 -1.8273 -432.0174
62.0000 -439.0526 0.0851 -1.2019 -
1.2560 -0.8653 -0.5909 -0.3352 -1.7308
0.0865 -1.8567 -432.0092
62.0200 -436.9150 0.5653 -0.0132 -
0.5762 -0.6222 -0.5621 -0.4209 -1.7506
0.0487 -1.8861 -432.0009
62.0400 -435.7466 -0.2595 0.8288
0.2531 -0.3618 -0.5213 -0.5073 -1.7695
0.0108 -1.9155 -431.9926
62.0600 -435.0680 -0.0618 0.9871
1.0437 -0.1062 -0.4693 -0.5941 -1.7875
-0.0271 -1.9448 -431.9843
62.0800 -434.6516 -0.1685 0.6529
1.6335 0.1239 -0.4066 -0.6812 -1.8046
-0.0651 -1.9741 -431.9760
62.1000 -434.6495 0.2637 0.2019
1.9309 0.3114 -0.3343 -0.7685 -1.8207
-0.1030 -2.0034 -431.9677
62.1200 -434.9171 -0.2569 -0.1762
1.9334 0.4449 -0.2534 -0.8557 -1.8358
-0.1411 -2.0326 -431.9594
62.1400 -435.2489 -0.0773 -0.2927
1.7087 0.5192 -0.1652 -0.9428 -1.8500
-0.1791 -2.0618 -431.9511
62.1600 -435.5068 -0.1765 -0.0311
1.3219 0.5356 -0.0712 -1.0293 -1.8631
-0.2172 -2.0909 -431.9428
62.1800 -435.7679 0.7619 0.2819
0.8005 0.5032 0.0271 -1.1153 -1.8753
-0.2553 -2.1201 -431.9344
62.2000 -436.1380 -0.4036 0.2793
0.1667 0.4357 0.1280 -1.2004 -1.8864
-0.2934 -2.1491 -431.9261
62.2200 -436.9362 0.4748 0.2210 -
0.5459 0.3480 0.2301 -1.2844 -1.8965
-0.3316 -2.1781 -431.9178
62.2400 -437.9842 0.0327 0.2504 -
1.2668 0.2549 0.3316 -1.3672 -1.9055
-0.3697 -2.2071 -431.9094
62.2600 -438.8061 0.0581 0.2584 -
1.8776 0.1690 0.4309 -1.4485 -1.9135
-0.4079 -2.2361 -431.9011
62.2800 -439.5462 -0.1657 0.0495 -
2.2275 0.1014 0.5264 -1.5281 -1.9204
-0.4461 -2.2650 -431.8927
62.3000 -440.0931 0.0934 -0.4363 -
2.1980 0.0578 0.6165 -1.6058 -1.9262
-0.4843 -2.2938 -431.8844

62.3200 -440.1535 -0.2791 -1.0639 -
1.7347 0.0399 0.7001 -1.6815 -1.9309
-0.5225 -2.3227 -431.8760
62.3400 -439.7078 0.6664 -1.6097 -
0.8737 0.0457 0.7760 -1.7550 -1.9345
-0.5608 -2.3514 -431.8677
62.3600 -438.9117 -0.2390 -1.6128
0.2292 0.0697 0.8431 -1.8260 -1.9369
-0.5990 -2.3802 -431.8593
62.3800 -437.4932 -1.2456 -0.5269
1.3405 0.1050 0.9006 -1.8944 -1.9382
-0.6373 -2.4088 -431.8510
62.4000 -433.8959 0.1212 1.5897
2.1920 0.1441 0.9476 -1.9600 -1.9384
-0.6755 -2.4375 -431.8426
62.4200 -430.7711 1.1768 3.1242
2.5381 0.1792 0.9832 -2.0226 -1.9375
-0.7137 -2.4661 -431.8342
62.4400 -431.7831 1.3947 2.3063
2.2349 0.2040 1.0065 -2.0821 -1.9353
-0.7520 -2.4946 -431.8258
62.4600 -437.2102 -0.8585 -0.1608
1.3935 0.2184 1.0172 -2.1383 -1.9320
-0.7902 -2.5231 -431.8174
62.4800 -440.5000 -1.4692 -1.9996
0.2715 0.2255 1.0146 -2.1911 -1.9275
-0.8285 -2.5516 -431.8090
62.5000 -440.4843 0.3076 -1.9837 -
0.8374 0.2298 0.9987 -2.2405 -1.9218
-0.8667 -2.5800 -431.8006
62.5200 -440.4460 0.5407 -0.7920 -
1.7022 0.2356 0.9692 -2.2862 -1.9149
-0.9049 -2.6083 -431.7922
62.5400 -440.3968 -0.3532 0.1629 -
2.1672 0.2475 0.9261 -2.3283 -1.9068
-0.9431 -2.6367 -431.7838
62.5600 -440.3327 0.2109 0.3743 -
2.1744 0.2703 0.8698 -2.3666 -1.8975
-0.9813 -2.6649 -431.7754
62.5800 -440.2336 -0.1682 0.2920 -
1.7804 0.3055 0.8002 -2.4011 -1.8869
-1.0195 -2.6931 -431.7670
62.6000 -440.0811 -0.1199 0.1033 -
1.0846 0.3509 0.7181 -2.4317 -1.8751
-1.0576 -2.7213 -431.7586
62.6200 -439.5961 -0.1961 -0.1054 -
0.2096 0.4030 0.6238 -2.4583 -1.8621
-1.0958 -2.7494 -431.7502
62.6400 -438.8286 0.4145 -0.1770
0.6841 0.4580 0.5182 -2.4810 -1.8479
-1.1339 -2.7775 -431.7418

62.6600 -438.1028 -0.2355 -0.0429
1.4239 0.5105 0.4020 -2.4996 -1.8324
-1.1720 -2.8055 -431.7333
62.6800 -437.3082 -0.2760 0.5745
1.8382 0.5551 0.2762 -2.5142 -1.8157
-1.2100 -2.8334 -431.7249
62.7000 -436.6031 0.0131 1.3924
1.8042 0.5896 0.1419 -2.5247 -1.7978
-1.2480 -2.8613 -431.7165
62.7200 -436.5195 0.6206 1.5006
1.3236 0.6118 0.0001 -2.5309 -1.7787
-1.2860 -2.8892 -431.7080
62.7400 -438.5092 -0.1209 0.7067
0.5177 0.6199 -0.1481 -2.5330 -1.7584
-1.3240 -2.9170 -431.6996
62.7600 -441.1744 -0.7263 -0.4118 -
0.4053 0.6128 -0.3017 -2.5307 -1.7369
-1.3619 -2.9447 -431.6911
62.7800 -442.4931 0.2047 -1.2676 -
1.1916 0.5904 -0.4595 -2.5240 -1.7142
-1.3998 -2.9724 -431.6827
62.8000 -443.2911 -0.0646 -1.6653 -
1.6244 0.5529 -0.6203 -2.5130 -1.6904
-1.4376 -3.0000 -431.6742
62.8200 -443.8020 0.3276 -1.6694 -
1.5888 0.5016 -0.7827 -2.4974 -1.6654
-1.4754 -3.0276 -431.6657
62.8400 -443.6954 -0.9164 -1.3609 -
1.0951 0.4380 -0.9457 -2.4772 -1.6394
-1.5131 -3.0551 -431.6573
62.8600 -441.8636 -0.0329 -0.6559 -
0.2860 0.3608 -1.1080 -2.4524 -1.6123
-1.5508 -3.0826 -431.6488
62.8800 -439.5563 0.4885 0.5621
0.6205 0.2694 -1.2683 -2.4228 -1.5842
-1.5885 -3.1100 -431.6403
62.9000 -438.4226 0.0782 1.8816
1.3644 0.1632 -1.4256 -2.3885 -1.5550
-1.6260 -3.1373 -431.6318
62.9200 -437.8988 -0.9501 2.7603
1.7180 0.0419 -1.5784 -2.3492 -1.5249
-1.6636 -3.1646 -431.6234
62.9400 -437.5671 0.1065 2.9987
1.5762 -0.0935 -1.7257 -2.3050 -1.4938
-1.7010 -3.1918 -431.6149
62.9600 -437.9437 1.2759 2.1358
0.9996 -0.2393 -1.8658 -2.2558 -1.4617
-1.7384 -3.2190 -431.6064
62.9800 -442.2478 0.1228 -0.0540
0.1911 -0.3888 -1.9975 -2.2016 -1.4288
-1.7758 -3.2461 -431.5979

63.0000 -447.1477 -0.8796 -2.5008 -
0.6113 -0.5346 -2.1193 -2.1423 -1.3951
-1.8130 -3.2732 -431.5894
63.0200 -448.0887 -0.7889 -3.3265 -
1.1814 -0.6696 -2.2295 -2.0780 -1.3605
-1.8502 -3.3001 -431.5809
63.0400 -446.2530 0.2152 -2.0353 -
1.3640 -0.7879 -2.3266 -2.0087 -1.3251
-1.8874 -3.3271 -431.5724
63.0600 -443.8946 0.2902 0.0982 -
1.1940 -0.8864 -2.4089 -1.9343 -1.2890
-1.9244 -3.3539 -431.5639
63.0800 -442.6922 0.9723 1.5757 -
0.7982 -0.9657 -2.4748 -1.8550 -1.2521
-1.9614 -3.3807 -431.5553
63.1000 -442.5378 -0.3706 1.8838 -
0.3341 -1.0285 -2.5224 -1.7709 -1.2146
-1.9983 -3.4074 -431.5468
63.1200 -442.4377 -0.2872 1.4071
0.0486 -1.0777 -2.5498 -1.6821 -1.1765
-2.0351 -3.4341 -431.5383
63.1400 -442.4001 0.1231 0.7532
0.2682 -1.1178 -2.5549 -1.5888 -1.1377
-2.0718 -3.4607 -431.5298
63.1600 -442.6685 0.4327 -0.0290
0.3471 -1.1549 -2.5358 -1.4913 -1.0984
-2.1084 -3.4872 -431.5212
63.1800 -443.2275 0.1571 -0.7688
0.3705 -1.1959 -2.4905 -1.3897 -1.0586
-2.1450 -3.5137 -431.5127
63.2000 -443.5921 -0.1552 -1.0197
0.4202 -1.2466 -2.4176 -1.2844 -1.0183
-2.1814 -3.5401 -431.5042
63.2200 -443.0821 -0.4388 -0.5582
0.5443 -1.3115 -2.3165 -1.1758 -0.9775
-2.2178 -3.5665 -431.4956
63.2400 -441.7983 0.3617 0.3597
0.7118 -1.3914 -2.1872 -1.0641 -0.9363
-2.2540 -3.5927 -431.4871
63.2600 -440.8522 0.0416 0.9364
0.8276 -1.4804 -2.0309 -0.9497 -0.8948
-2.2902 -3.6189 -431.4785
63.2800 -441.1209 -0.1895 0.7004
0.7994 -1.5667 -1.8492 -0.8330 -0.8529
-2.3262 -3.6451 -431.4700
63.3000 -442.0900 0.4045 -0.0808
0.5732 -1.6371 -1.6440 -0.7144 -0.8106
-2.3621 -3.6711 -431.4614
63.3200 -442.8486 -0.7358 -0.6931
0.1819 -1.6769 -1.4173 -0.5943 -0.7682
-2.3979 -3.6971 -431.4528

63.3400	-442.7537	-0.0738	-0.7513	-
0.3028	-1.6715	-1.1713	-0.4729	-0.7255
	-2.4336	-3.7231	-431.4443	
63.3600	-442.2198	0.4847	-0.3387	-
0.8000	-1.6081	-0.9082	-0.3507	-0.6826
	-2.4692	-3.7489	-431.4357	
63.3800	-441.5938	-0.2888	0.2182	-
1.2551	-1.4772	-0.6307	-0.2280	-0.6395
	-2.5047	-3.7747	-431.4271	
63.4000	-441.1336	-0.2898	0.6531	-
1.6220	-1.2732	-0.3416	-0.1053	-0.5964
	-2.5400	-3.8004	-431.4185	
63.4200	-440.7381	0.2172	0.6840	-
1.8365	-0.9962	-0.0441	0.0171	-0.5532
	-2.5752	-3.8260	-431.4100	
63.4400	-440.3072	-0.1846	0.1574	-
1.8296	-0.6521	0.2584	0.1388	-0.5099
	-2.6103	-3.8516	-431.4014	
63.4600	-439.7953	0.6062	-0.6165	-
1.5381	-0.2523	0.5624	0.2595	-0.4666
	-2.6452	-3.8771	-431.3928	
63.4800	-439.2315	0.0163	-1.5696	-
0.9228	0.1849	0.8644	0.3788	-0.4234
	-2.6800	-3.9025	-431.3842	
63.5000	-438.4183	-0.4920	-2.1782	-
0.0358	0.6375	1.1610	0.4962	-0.3802
	-2.7147	-3.9279	-431.3756	
63.5200	-436.1751	-0.9330	-1.2355	
0.9918	1.0821	1.4486	0.6115	-0.3371
	-2.7492	-3.9532	-431.3670	
63.5400	-430.9124	0.7373	1.0767	
1.9895	1.4966	1.7239	0.7242	-0.2942
	-2.7836	-3.9783	-431.3584	
63.5600	-427.1376	1.4598	2.9914	
2.7663	1.8598	1.9834	0.8340	-0.2515
	-2.8178	-4.0035	-431.3498	
63.5800	-427.0998	-0.5195	3.2270	
3.1614	2.1515	2.2237	0.9405	-0.2089
	-2.8519	-4.0285	-431.3412	
63.6000	-427.4721	-0.1120	1.9887	
3.1258	2.3559	2.4415	1.0433	-0.1667
	-2.8858	-4.0535	-431.3325	
63.6200	-428.2811	1.6027	-0.1920	
2.7366	2.4703	2.6336	1.1420	-0.1247
	-2.9196	-4.0784	-431.3239	
63.6400	-432.6064	0.4686	-2.6303	
2.1035	2.5010	2.7978	1.2363	-0.0831
	-2.9532	-4.1032	-431.3153	
63.6600	-436.7921	-2.6622	-3.6319	
1.3205	2.4581	2.9324	1.3258	-0.0418
	-2.9866	-4.1279	-431.3067	

63.6800 -435.3020 -1.9695 -1.8256
0.4628 2.3554 3.0362 1.4102 -0.0010
-3.0199 -4.1526 -431.2980
63.7000 -429.8388 0.4785 1.5395 -
0.4249 2.2109 3.1089 1.4890 0.0394
-3.0530 -4.1771 -431.2894
63.7200 -426.7018 3.2972 3.2796 -
1.3063 2.0434 3.1505 1.5621 0.0793
-3.0859 -4.2016 -431.2808
63.7400 -431.4223 0.6474 1.8616 -
2.1383 1.8691 3.1615 1.6291 0.1187
-3.1187 -4.2261 -431.2721
63.7600 -438.7071 -2.3984 -0.9536 -
2.8296 1.6990 3.1427 1.6899 0.1576
-3.1513 -4.2504 -431.2635
63.7800 -439.9101 -2.3208 -2.4604 -
3.2771 1.5405 3.0950 1.7443 0.1958
-3.1837 -4.2746 -431.2548
63.8000 -437.3119 0.0639 -1.7671 -
3.3879 1.3982 3.0196 1.7925 0.2334
-3.2159 -4.2988 -431.2462
63.8200 -434.7022 1.3747 -0.2212 -
3.0906 1.2730 2.9181 1.8345 0.2704
-3.2479 -4.3229 -431.2375
63.8400 -434.2706 0.7300 0.3214 -
2.3581 1.1617 2.7923 1.8705 0.3066
-3.2798 -4.3469 -431.2289
63.8600 -434.2465 0.1507 -0.2047 -
1.2596 1.0573 2.6444 1.9006 0.3422
-3.3114 -4.3708 -431.2202
63.8800 -434.2117 -0.4552 -0.7318
0.0211 0.9517 2.4768 1.9251 0.3769
-3.3428 -4.3947 -431.2116
63.9000 -433.1813 -0.2529 -0.5234
1.2661 0.8373 2.2919 1.9441 0.4108
-3.3741 -4.4184 -431.2029
63.9200 -430.8139 -0.1748 0.3951
2.2737 0.7054 2.0923 1.9578 0.4440
-3.4051 -4.4421 -431.1942
63.9400 -429.7060 0.2505 1.5330
2.8782 0.5468 1.8804 1.9665 0.4762
-3.4360 -4.4656 -431.1855
63.9600 -430.1800 0.2457 2.1175
2.9702 0.3554 1.6588 1.9704 0.5076
-3.4666 -4.4891 -431.1769
63.9800 -431.1708 -0.7513 1.7063
2.5441 0.1315 1.4298 1.9695 0.5381
-3.4970 -4.5125 -431.1682
64.0000 -432.6867 0.3759 0.6688
1.7067 -0.1185 1.1955 1.9642 0.5676
-3.5273 -4.5359 -431.1595

64.0200 -435.5263 0.3434 -0.3515
0.6554 -0.3858 0.9580 1.9547 0.5963
-3.5573 -4.5591 -431.1508
64.0400 -438.1071 -0.2401 -1.1276 -
0.3649 -0.6608 0.7191 1.9410 0.6239
-3.5871 -4.5822 -431.1421
64.0600 -439.7096 0.1487 -1.6326 -
1.1268 -0.9337 0.4806 1.9235 0.6506
-3.6166 -4.6053 -431.1334
64.0800 -441.0381 0.1882 -1.8938 -
1.4847 -1.1940 0.2443 1.9023 0.6763
-3.6460 -4.6282 -431.1247
64.1000 -441.6000 -0.5450 -1.6431 -
1.4006 -1.4295 0.0117 1.8776 0.7009
-3.6751 -4.6511 -431.1160
64.1200 -440.5168 -0.2034 -0.6388 -
0.9848 -1.6269 -0.2156 1.8495 0.7246
-3.7040 -4.6739 -431.1073
64.1400 -438.4872 0.4781 0.7100 -
0.4510 -1.7738 -0.4359 1.8184 0.7472
-3.7327 -4.6966 -431.0986
64.1600 -437.4000 0.5112 1.7200 -
0.0056 -1.8598 -0.6477 1.7843 0.7688
-3.7612 -4.7192 -431.0899
64.1800 -437.8413 0.0076 1.9127
0.2042 -1.8766 -0.8496 1.7475 0.7894
-3.7894 -4.7417 -431.0812
64.2000 -438.8290 -0.2567 1.3476
0.1138 -1.8216 -1.0403 1.7081 0.8089
-3.8174 -4.7641 -431.0725
64.2200 -439.8784 -0.3744 0.5306 -
0.2200 -1.6998 -1.2187 1.6664 0.8274
-3.8452 -4.7864 -431.0637
64.2400 -441.2048 0.3484 -0.3053 -
0.6522 -1.5202 -1.3838 1.6226 0.8448
-3.8727 -4.8086 -431.0550
64.2600 -442.5656 0.1124 -1.1657 -
1.0002 -1.2932 -1.5350 1.5768 0.8612
-3.9001 -4.8308 -431.0463
64.2800 -443.0902 -0.2072 -1.6666 -
1.1135 -1.0308 -1.6718 1.5293 0.8765
-3.9271 -4.8528 -431.0375
64.3000 -442.2872 0.1440 -1.4261 -
0.9242 -0.7453 -1.7935 1.4802 0.8908
-3.9540 -4.8747 -431.0288
64.3200 -440.7796 0.2102 -0.6956 -
0.4605 -0.4506 -1.8999 1.4297 0.9040
-3.9806 -4.8966 -431.0201
64.3400 -439.3938 -0.2172 0.0944
0.1469 -0.1608 -1.9906 1.3781 0.9162
-4.0069 -4.9183 -431.0113

64.3600 -438.1854 -0.1598 0.7838
0.7207 0.1105 -2.0652 1.3255 0.9274
-4.0330 -4.9400 -431.0026
64.3800 -437.0618 0.3017 1.2844
1.0941 0.3507 -2.1234 1.2721 0.9376
-4.0589 -4.9615 -430.9938
64.4000 -436.6027 0.3178 1.4721
1.1645 0.5479 -2.1651 1.2182 0.9467
-4.0845 -4.9830 -430.9851
64.4200 -437.4566 -0.0409 1.0710
0.9180 0.6948 -2.1900 1.1638 0.9548
-4.1099 -5.0043 -430.9763
64.4400 -439.0451 -0.1784 0.2433
0.4485 0.7902 -2.1982 1.1092 0.9618
-4.1351 -5.0256 -430.9676
64.4600 -440.2314 0.0059 -0.6430 -
0.0750 0.8359 -2.1898 1.0545 0.9679
-4.1599 -5.0468 -430.9588
64.4800 -441.0570 0.2476 -1.2953 -
0.4897 0.8343 -2.1651 0.9998 0.9730
-4.1846 -5.0678 -430.9501
64.5000 -441.7001 -0.0091 -1.4650 -
0.6923 0.7889 -2.1246 0.9454 0.9770
-4.2090 -5.0888 -430.9413
64.5200 -441.8540 -0.2433 -0.9801 -
0.6645 0.7045 -2.0688 0.8913 0.9801
-4.2331 -5.1096 -430.9325
64.5400 -440.4535 -0.5423 0.1992 -
0.4656 0.5871 -1.9985 0.8376 0.9822
-4.2570 -5.1304 -430.9238
64.5600 -438.4368 0.3699 1.4644 -
0.1974 0.4440 -1.9147 0.7844 0.9833
-4.2806 -5.1510 -430.9150
64.5800 -437.6336 0.9431 1.6320
0.0548 0.2817 -1.8182 0.7319 0.9834
-4.3039 -5.1716 -430.9062
64.6000 -439.3337 -0.0383 0.3177
0.2717 0.1058 -1.7098 0.6800 0.9826
-4.3271 -5.1920 -430.8974
64.6200 -441.8188 -0.3305 -1.3960
0.4666 -0.0792 -1.5900 0.6290 0.9809
-4.3499 -5.2124 -430.8886
64.6400 -442.6078 -1.0325 -1.9267
0.6441 -0.2680 -1.4595 0.5786 0.9782
-4.3725 -5.2326 -430.8799
64.6600 -440.6844 -0.1920 -0.8099
0.7721 -0.4532 -1.3188 0.5291 0.9747
-4.3948 -5.2527 -430.8711
64.6800 -438.0432 0.5470 0.9642
0.7806 -0.6275 -1.1687 0.4805 0.9703
-4.4169 -5.2727 -430.8623

64.7000 -437.0544 1.0807 2.0427
0.6026 -0.7836 -1.0100 0.4327 0.9650
-4.4386 -5.2927 -430.8535

64.7200 -439.1498 0.4144 1.4064
0.2394 -0.9128 -0.8440 0.3857 0.9588
-4.4602 -5.3125 -430.8447

64.7400 -442.1729 -1.1429 -0.3082 -
0.2153 -1.0063 -0.6719 0.3398 0.9518
-4.4814 -5.3322 -430.8359

64.7600 -443.2810 0.1154 -1.4576 -
0.6424 -1.0555 -0.4953 0.2947 0.9440
-4.5024 -5.3518 -430.8271

64.7800 -442.7542 -0.3064 -1.1999 -
0.9477 -1.0542 -0.3160 0.2507 0.9354
-4.5231 -5.3713 -430.8183

64.8000 -441.8985 0.7206 -0.0943 -
1.1020 -0.9967 -0.1358 0.2076 0.9261
-4.5436 -5.3906 -430.8095

64.8200 -441.2403 -0.1167 0.7647 -
1.1498 -0.8785 0.0435 0.1654 0.9159
-4.5637 -5.4099 -430.8007

64.8400 -440.7554 -0.1888 0.9016 -
1.1497 -0.7010 0.2201 0.1243 0.9051
-4.5836 -5.4291 -430.7918

64.8600 -440.3549 0.2818 0.5254 -
1.1099 -0.4706 0.3922 0.0842 0.8935
-4.6033 -5.4481 -430.7830

64.8800 -440.2004 -0.0922 -0.0917 -
0.9955 -0.2000 0.5577 0.0451 0.8812
-4.6226 -5.4670 -430.7742

64.9000 -440.3132 0.0115 -0.8410 -
0.7347 0.0947 0.7148 0.0070 0.8683
-4.6417 -5.4859 -430.7654

64.9200 -440.5049 0.1735 -1.6024 -
0.2668 0.3947 0.8612 -0.0302 0.8548
-4.6605 -5.5046 -430.7566

64.9400 -440.5950 -0.4026 -1.8082
0.3975 0.6801 0.9949 -0.0663 0.8406
-4.6790 -5.5232 -430.7477

64.9600 -438.5747 -0.8033 -1.0753
1.1566 0.9317 1.1140 -0.1016 0.8258
-4.6972 -5.5417 -430.7389

64.9800 -435.1265 0.7353 0.2868
1.8493 1.1316 1.2167 -0.1359 0.8105
-4.7151 -5.5600 -430.7301

65.0000 -433.5000 0.5969 1.7262
2.2813 1.2642 1.3015 -0.1692 0.7946
-4.7328 -5.5783 -430.7212

65.0200 -433.5000 -0.7534 2.9604
2.2766 1.3179 1.3672 -0.2018 0.7782
-4.7502 -5.5965 -430.7124

65.0400 -433.5000 -0.5042 3.4843
1.7629 1.2892 1.4134 -0.2335 0.7614
-4.7673 -5.6145 -430.7036
65.0600 -433.9053 1.5558 2.3535
0.8217 1.1882 1.4399 -0.2644 0.7440
-4.7841 -5.6324 -430.6947
65.0800 -438.9863 0.4307 -0.6045 -
0.2968 1.0315 1.4474 -0.2946 0.7262
-4.8006 -5.6502 -430.6859
65.1000 -444.8228 -1.3576 -3.5131 -
1.3001 0.8360 1.4370 -0.3242 0.7081
-4.8168 -5.6679 -430.6770
65.1200 -445.4918 -1.4423 -3.8928 -
1.9220 0.6186 1.4101 -0.3530 0.6895
-4.8327 -5.6854 -430.6682
65.1400 -442.1004 0.0614 -1.5516 -
2.0392 0.3937 1.3684 -0.3813 0.6706
-4.8484 -5.7029 -430.6593
65.1600 -438.3782 1.4516 1.4066 -
1.7228 0.1730 1.3137 -0.4090 0.6513
-4.8637 -5.7202 -430.6505
65.1800 -437.5561 0.6440 2.6354 -
1.1018 -0.0347 1.2480 -0.4360 0.6317
-4.8788 -5.7374 -430.6416
65.2000 -439.1825 0.0307 1.7186 -
0.3327 -0.2234 1.1736 -0.4626 0.6118
-4.8936 -5.7545 -430.6327
65.2200 -441.0555 -0.4202 -0.0081
0.4236 -0.3904 1.0926 -0.4887 0.5917
-4.9081 -5.7715 -430.6239
65.2400 -441.3983 -0.4445 -1.0749
1.0215 -0.5356 1.0072 -0.5143 0.5713
-4.9222 -5.7883 -430.6150
65.2600 -440.5404 -0.0094 -0.9600
1.3606 -0.6605 0.9195 -0.5395 0.5508
-4.9361 -5.8051 -430.6061
65.2800 -439.5618 0.6342 -0.1952
1.4093 -0.7649 0.8314 -0.5644 0.5300
-4.9497 -5.8217 -430.5973
65.3000 -439.3801 0.2708 0.4667
1.1662 -0.8458 0.7447 -0.5890 0.5090
-4.9630 -5.8381 -430.5884
65.3200 -440.2843 -0.2740 0.5614
0.6814 -0.9011 0.6609 -0.6133 0.4880
-4.9760 -5.8545 -430.5795
65.3400 -441.5528 -0.2394 0.2716
0.0744 -0.9293 0.5810 -0.6373 0.4667
-4.9887 -5.8707 -430.5706
65.3600 -442.4402 0.2496 -0.0932 -
0.4969 -0.9274 0.5061 -0.6610 0.4454
-5.0011 -5.8869 -430.5618

65.3800 -443.1757 0.1997 -0.4867 -
0.8844 -0.8912 0.4371 -0.6844 0.4241
-5.0132 -5.9029 -430.5529
65.4000 -443.7392 0.0449 -0.8354 -
1.0260 -0.8162 0.3745 -0.7074 0.4026
-5.0250 -5.9187 -430.5440
65.4200 -443.8412 -0.6669 -0.6504 -
0.9472 -0.7022 0.3188 -0.7300 0.3811
-5.0365 -5.9345 -430.5351
65.4400 -442.6168 0.4598 0.3322 -
0.7482 -0.5510 0.2702 -0.7521 0.3597
-5.0477 -5.9501 -430.5262
65.4600 -440.8905 -0.0805 1.4728 -
0.5584 -0.3672 0.2285 -0.7735 0.3382
-5.0586 -5.9656 -430.5173
65.4800 -440.1077 0.9031 1.5949 -
0.4568 -0.1609 0.1933 -0.7942 0.3167
-5.0692 -5.9809 -430.5084
65.5000 -441.4389 -0.1401 0.3657 -
0.4214 0.0547 0.1637 -0.8139 0.2953
-5.0795 -5.9962 -430.4995
65.5200 -443.6701 0.1883 -1.3528 -
0.3458 0.2659 0.1386 -0.8326 0.2740
-5.0895 -6.0113 -430.4906
65.5400 -444.7983 -1.0558 -2.1893 -
0.1302 0.4604 0.1167 -0.8500 0.2528
-5.0991 -6.0263 -430.4817
65.5600 -443.1406 -0.4315 -1.4296
0.2500 0.6272 0.0969 -0.8660 0.2317
-5.1085 -6.0411 -430.4728
65.5800 -440.0804 0.4256 0.3331
0.7181 0.7578 0.0776 -0.8805 0.2107
-5.1176 -6.0558 -430.4639
65.6000 -438.1390 0.3451 1.6498
1.1135 0.8459 0.0577 -0.8932 0.1898
-5.1263 -6.0704 -430.4550
65.6200 -438.5994 -0.2257 1.5731
1.2838 0.8867 0.0356 -0.9041 0.1692
-5.1348 -6.0849 -430.4461
65.6400 -439.9376 0.0762 0.4894
1.1500 0.8778 0.0103 -0.9130 0.1487
-5.1429 -6.0992 -430.4372
65.6600 -441.2555 0.0907 -0.4562
0.7092 0.8242 -0.0196 -0.9198 0.1284
-5.1507 -6.1134 -430.4283
65.6800 -442.1039 -0.0355 -0.6644
0.0591 0.7372 -0.0546 -0.9243 0.1083
-5.1582 -6.1275 -430.4193
65.7000 -442.8462 -0.1347 -0.3548 -
0.6366 0.6308 -0.0955 -0.9265 0.0885
-5.1654 -6.1414 -430.4104

65.7200 -443.4793 -0.3653 0.0292 -
1.2098 0.5190 -0.1426 -0.9263 0.0689
-5.1723 -6.1552 -430.4015
65.7400 -444.0577 0.6639 0.1024 -
1.5094 0.4139 -0.1960 -0.9237 0.0496
-5.1788 -6.1689 -430.3926
65.7600 -444.6171 0.0215 -0.3497 -
1.4391 0.3247 -0.2559 -0.9185 0.0306
-5.1851 -6.1824 -430.3836
65.7800 -444.9648 -0.1131 -0.8780 -
1.0066 0.2553 -0.3217 -0.9108 0.0119
-5.1910 -6.1958 -430.3747
65.8000 -444.5375 -0.4831 -0.7820 -
0.3306 0.2054 -0.3927 -0.9005 -0.0064
-5.1966 -6.2091 -430.3658
65.8200 -442.5424 0.2330 -0.0019
0.4100 0.1715 -0.4677 -0.8877 -0.0245
-5.2019 -6.2222 -430.3569
65.8400 -440.5532 0.4276 0.8516
1.0191 0.1487 -0.5455 -0.8724 -0.0422
-5.2068 -6.2352 -430.3479
65.8600 -440.2078 0.2354 1.1877
1.3313 0.1320 -0.6243 -0.8547 -0.0596
-5.2115 -6.2481 -430.3390
65.8800 -441.1748 -0.1240 0.8483
1.2752 0.1154 -0.7023 -0.8346 -0.0766
-5.2158 -6.2608 -430.3300
65.9000 -442.4968 -0.0837 0.1286
0.8966 0.0927 -0.7774 -0.8122 -0.0932
-5.2198 -6.2734 -430.3211
65.9200 -443.4289 0.2984 -0.5540
0.3387 0.0586 -0.8475 -0.7875 -0.1095
-5.2234 -6.2859 -430.3122
65.9400 -444.1969 0.0080 -0.7749 -
0.2282 0.0089 -0.9107 -0.7607 -0.1254
-5.2268 -6.2982 -430.3032
65.9600 -444.8014 0.4798 -0.6372 -
0.6515 -0.0600 -0.9648 -0.7318 -0.1409
-5.2298 -6.3104 -430.2943
65.9800 -444.9981 -0.5117 -0.4654 -
0.8389 -0.1498 -1.0082 -0.7009 -0.1560
-5.2325 -6.3224 -430.2853
66.0000 -444.9012 -0.1161 -0.2474 -
0.7620 -0.2570 -1.0393 -0.6681 -0.1707
-5.2348 -6.3343 -430.2764
66.0200 -444.6943 0.2589 -0.1762 -
0.4515 -0.3755 -1.0569 -0.6335 -0.1850
-5.2368 -6.3461 -430.2674
66.0400 -444.4270 -0.4263 -0.2556 -
0.0150 -0.4985 -1.0602 -0.5973 -0.1989
-5.2385 -6.3577 -430.2585

66.0600 -443.9030 0.0174 0.0544
0.3929 -0.6200 -1.0484 -0.5595 -0.2125
-5.2398 -6.3692 -430.2495
66.0800 -443.1027 -0.1387 0.7645
0.6354 -0.7344 -1.0211 -0.5202 -0.2256
-5.2408 -6.3806 -430.2405
66.1000 -442.5432 0.2907 1.2365
0.6349 -0.8342 -0.9780 -0.4797 -0.2384
-5.2415 -6.3918 -430.2316
66.1200 -442.9620 0.6967 0.9874
0.4063 -0.9107 -0.9189 -0.4381 -0.2507
-5.2418 -6.4028 -430.2226
66.1400 -444.6134 -0.4089 -0.0256
0.0503 -0.9560 -0.8442 -0.3956 -0.2627
-5.2418 -6.4138 -430.2137
66.1600 -446.1364 -0.6333 -1.0814 -
0.2967 -0.9628 -0.7544 -0.3524 -0.2744
-5.2414 -6.4246 -430.2047
66.1800 -446.1944 0.0670 -1.3155 -
0.5134 -0.9250 -0.6507 -0.3087 -0.2856
-5.2407 -6.4352 -430.1957
66.2000 -444.9172 -0.2160 -0.5508 -
0.5447 -0.8390 -0.5344 -0.2646 -0.2965
-5.2397 -6.4457 -430.1868
66.2200 -443.3856 0.1401 0.3818 -
0.4218 -0.7067 -0.4075 -0.2205 -0.3070
-5.2383 -6.4561 -430.1778
66.2400 -442.6510 -0.1084 0.7510 -
0.2240 -0.5343 -0.2723 -0.1765 -0.3172
-5.2365 -6.4663 -430.1688
66.2600 -442.4814 0.5060 0.5559 -
0.0571 -0.3295 -0.1309 -0.1327 -0.3271
-5.2344 -6.4764 -430.1598
66.2800 -442.3691 -0.2673 0.1510
0.0159 -0.1031 0.0142 -0.0895 -0.3366
-5.2320 -6.4863 -430.1509
66.3000 -442.1897 -0.0958 -0.0423 -
0.0077 0.1321 0.1607 -0.0470 -0.3458
-5.2292 -6.4961 -430.1419
66.3200 -441.6451 0.3629 -0.0114 -
0.0957 0.3617 0.3063 -0.0054 -0.3546
-5.2260 -6.5058 -430.1329
66.3400 -440.9086 -0.0897 0.2454 -
0.1860 0.5709 0.4490 0.0351 -0.3632
-5.2225 -6.5153 -430.1239
66.3600 -440.5026 -0.2160 0.3731 -
0.1943 0.7445 0.5865 0.0743 -0.3714
-5.2186 -6.5247 -430.1149
66.3800 -440.7812 0.2836 -0.1950 -
0.0529 0.8704 0.7168 0.1120 -0.3794
-5.2144 -6.5340 -430.1059

66.4000 -441.3893 -0.1252 -1.2584
0.2342 0.9415 0.8381 0.1481 -0.3871
-5.2098 -6.5431 -430.0970
66.4200 -441.7907 -0.3493 -1.6508
0.5887 0.9555 0.9486 0.1822 -0.3945
-5.2049 -6.5520 -430.0880
66.4400 -440.4708 -0.4917 -0.3581
0.8742 0.9153 1.0470 0.2143 -0.4016
-5.1996 -6.5608 -430.0790
66.4600 -437.1530 0.1604 1.9152
0.9647 0.8272 1.1322 0.2442 -0.4084
-5.1939 -6.5695 -430.0700
66.4800 -434.7247 1.1796 3.1777
0.7763 0.7007 1.2034 0.2717 -0.4150
-5.1878 -6.5780 -430.0610
66.5000 -436.6057 1.2273 2.0229
0.3081 0.5479 1.2599 0.2967 -0.4214
-5.1814 -6.5864 -430.0520
66.5200 -442.2790 -0.6356 -0.8340 -
0.2796 0.3814 1.3015 0.3191 -0.4275
-5.1747 -6.5947 -430.0430
66.5400 -445.8805 -1.8229 -3.1297 -
0.7792 0.2125 1.3281 0.3389 -0.4334
-5.1675 -6.6028 -430.0340
66.5600 -444.7636 -0.0951 -3.2033 -
1.0120 0.0521 1.3397 0.3559 -0.4391
-5.1600 -6.6108 -430.0250
66.5800 -442.1600 0.5617 -1.4182 -
0.9115 -0.0897 1.3366 0.3702 -0.4446
-5.1521 -6.6186 -430.0160
66.6000 -439.9237 0.7034 0.7730 -
0.5588 -0.2061 1.3190 0.3818 -0.4499
-5.1439 -6.6263 -430.0070
66.6200 -439.1375 -0.3856 1.9932 -
0.0966 -0.2935 1.2876 0.3907 -0.4550
-5.1352 -6.6339 -429.9980
66.6400 -438.6081 -0.2332 2.1863
0.2958 -0.3501 1.2430 0.3969 -0.4599
-5.1262 -6.6413 -429.9890
66.6600 -438.3233 0.0881 1.9601
0.4574 -0.3760 1.1860 0.4007 -0.4647
-5.1169 -6.6486 -429.9800
66.6800 -438.8413 0.7902 1.3482
0.3413 -0.3729 1.1177 0.4019 -0.4693
-5.1071 -6.6557 -429.9710
66.7000 -440.9146 -0.0812 0.3232
0.0098 -0.3443 1.0389 0.4008 -0.4739
-5.0970 -6.6627 -429.9620
66.7200 -442.7104 -1.1187 -0.5909 -
0.3960 -0.2945 0.9506 0.3975 -0.4782
-5.0865 -6.6696 -429.9529

66.7400 -442.9368 0.0158 -1.0694 -
0.7068 -0.2285 0.8534 0.3922 -0.4825
-5.0756 -6.6763 -429.9439
66.7600 -442.9772 0.5714 -1.3541 -
0.7894 -0.1525 0.7483 0.3849 -0.4868
-5.0643 -6.6829 -429.9349
66.7800 -442.9975 0.4812 -1.5338 -
0.5716 -0.0724 0.6360 0.3758 -0.4909
-5.0527 -6.6894 -429.9259
66.8000 -442.6925 -1.0672 -1.1176 -
0.1048 0.0060 0.5176 0.3652 -0.4950
-5.0406 -6.6957 -429.9169
66.8200 -440.9317 0.1100 0.0076
0.4660 0.0769 0.3941 0.3532 -0.4991
-5.0282 -6.7019 -429.9079
66.8400 -439.0640 0.2109 1.1930
0.9583 0.1336 0.2668 0.3399 -0.5031
-5.0154 -6.7079 -429.8988
66.8600 -438.6822 0.2631 1.7062
1.1961 0.1699 0.1369 0.3256 -0.5071
-5.0022 -6.7138 -429.8898
66.8800 -439.5482 -0.0663 1.3821
1.0691 0.1816 0.0056 0.3105 -0.5112
-4.9886 -6.7196 -429.8808
66.9000 -440.8685 -0.2867 0.5825
0.6077 0.1688 -0.1259 0.2947 -0.5152
-4.9747 -6.7252 -429.8718
66.9200 -442.1073 0.4233 -0.2376 -
0.0356 0.1349 -0.2561 0.2785 -0.5193
-4.9603 -6.7307 -429.8627
66.9400 -443.6304 0.1236 -0.9965 -
0.6305 0.0850 -0.3837 0.2620 -0.5234
-4.9456 -6.7361 -429.8537
66.9600 -444.9790 -0.0520 -1.6298 -
0.9682 0.0255 -0.5073 0.2455 -0.5275
-4.9305 -6.7413 -429.8447
66.9800 -445.3383 -0.6501 -1.5647 -
0.9582 -0.0374 -0.6254 0.2291 -0.5317
-4.9150 -6.7464 -429.8357
67.0000 -443.8936 0.1488 -0.4751 -
0.6570 -0.1003 -0.7368 0.2131 -0.5360
-4.8991 -6.7513 -429.8266
67.0200 -441.7641 0.1070 0.9685 -
0.2131 -0.1613 -0.8401 0.1976 -0.5404
-4.8828 -6.7562 -429.8176
67.0400 -440.6030 0.7043 1.7343
0.1942 -0.2182 -0.9340 0.1829 -0.5449
-4.8661 -6.7609 -429.8086
67.0600 -441.3861 0.0872 1.2946
0.4299 -0.2695 -1.0172 0.1691 -0.5495
-4.8490 -6.7654 -429.7995

67.0800 -443.0113 -0.6286 0.1716
0.4767 -0.3133 -1.0885 0.1564 -0.5542
-4.8316 -6.7698 -429.7905
67.1000 -444.0754 -0.2325 -0.6907
0.3720 -0.3478 -1.1470 0.1450 -0.5591
-4.8137 -6.7741 -429.7814
67.1200 -443.9617 0.0584 -0.8277
0.1644 -0.3713 -1.1918 0.1350 -0.5641
-4.7955 -6.7783 -429.7724
67.1400 -443.6087 0.2669 -0.2956 -
0.0840 -0.3829 -1.2220 0.1266 -0.5692
-4.7768 -6.7823 -429.7634
67.1600 -443.3274 0.0032 0.4220 -
0.3235 -0.3827 -1.2372 0.1199 -0.5745
-4.7578 -6.7862 -429.7543
67.1800 -443.4150 -0.1552 0.5835 -
0.5234 -0.3719 -1.2374 0.1149 -0.5799
-4.7384 -6.7899 -429.7453
67.2000 -443.8697 0.1005 0.0226 -
0.6240 -0.3521 -1.2225 0.1118 -0.5855
-4.7186 -6.7936 -429.7362
67.2200 -444.3091 -0.0764 -0.6005 -
0.5540 -0.3247 -1.1929 0.1106 -0.5913
-4.6984 -6.7971 -429.7272
67.2400 -444.2289 -0.4606 -0.7354 -
0.2811 -0.2901 -1.1491 0.1113 -0.5973
-4.6779 -6.8004 -429.7181
67.2600 -442.9478 -0.0516 -0.3369
0.1582 -0.2459 -1.0922 0.1139 -0.6034
-4.6569 -6.8036 -429.7091
67.2800 -441.4377 0.6511 0.2642
0.6377 -0.1916 -1.0231 0.1185 -0.6097
-4.6356 -6.8068 -429.7000
67.3000 -440.9014 -0.1167 0.7020
0.9731 -0.1286 -0.9429 0.1250 -0.6161
-4.6139 -6.8097 -429.6910
67.3200 -440.9498 -0.2475 0.9557
0.9894 -0.0571 -0.8530 0.1333 -0.6228
-4.5918 -6.8126 -429.6819
67.3400 -441.0585 0.0566 0.9616
0.6221 0.0219 -0.7551 0.1434 -0.6295
-4.5693 -6.8153 -429.6729
67.3600 -441.2232 -0.0603 0.5824 -
0.0561 0.1054 -0.6507 0.1553 -0.6364
-4.5465 -6.8179 -429.6638
67.3800 -442.1259 0.5197 -0.0315 -
0.8503 0.1881 -0.5416 0.1689 -0.6435
-4.5232 -6.8203 -429.6548
67.4000 -443.4758 -0.1156 -0.5994 -
1.5107 0.2644 -0.4295 0.1842 -0.6506
-4.4996 -6.8226 -429.6457

67.4200 -444.1992 -0.3507 -0.9999 -
1.8019 0.3297 -0.3163 0.2011 -0.6579
-4.4757 -6.8248 -429.6367
67.4400 -443.6888 0.0193 -1.1269 -
1.5687 0.3807 -0.2039 0.2195 -0.6653
-4.4513 -6.8269 -429.6276
67.4600 -442.4115 0.4086 -0.9732 -
0.8228 0.4152 -0.0942 0.2393 -0.6727
-4.4266 -6.8289 -429.6186
67.4800 -440.8637 -0.0685 -0.5428
0.2564 0.4324 0.0112 0.2606 -0.6802
-4.4015 -6.8307 -429.6095
67.5000 -438.9738 0.0021 0.4817
1.3677 0.4315 0.1106 0.2830 -0.6877
-4.3761 -6.8324 -429.6004
67.5200 -436.4243 -0.5067 1.8014
2.2038 0.4119 0.2023 0.3067 -0.6953
-4.3503 -6.8339 -429.5914
67.5400 -434.7146 0.9636 2.3336
2.5004 0.3736 0.2847 0.3314 -0.7028
-4.3241 -6.8354 -429.5823
67.5600 -435.7810 0.5231 1.4418
2.1409 0.3209 0.3559 0.3570 -0.7103
-4.2976 -6.8367 -429.5733
67.5800 -439.5801 0.0213 -0.2713
1.2471 0.2656 0.4145 0.3834 -0.7178
-4.2707 -6.8379 -429.5642
67.6000 -442.3948 -0.5047 -1.4208
0.0457 0.2216 0.4591 0.4104 -0.7253
-4.2435 -6.8389 -429.5551
67.6200 -442.5362 -0.3408 -1.3012 -
1.2094 0.2028 0.4886 0.4379 -0.7326
-4.2159 -6.8399 -429.5461
67.6400 -442.5566 0.4738 -0.3522 -
2.2491 0.2223 0.5024 0.4657 -0.7398
-4.1880 -6.8407 -429.5370
67.6600 -442.5850 -0.0011 0.4028 -
2.8378 0.2917 0.5003 0.4935 -0.7469
-4.1598 -6.8413 -429.5279
67.6800 -442.7776 0.2842 0.0421 -
2.8260 0.4171 0.4828 0.5210 -0.7539
-4.1312 -6.8419 -429.5189
67.7000 -443.2887 0.2363 -1.3700 -
2.2374 0.5940 0.4510 0.5481 -0.7607
-4.1022 -6.8423 -429.5098
67.7200 -443.7322 -0.7528 -2.5299 -
1.2351 0.8073 0.4066 0.5745 -0.7673
-4.0730 -6.8427 -429.5007
67.7400 -442.8182 -1.6525 -1.8710 -
0.0490 1.0391 0.3511 0.5998 -0.7737
-4.0434 -6.8428 -429.4917

67.7600 -437.5264 -0.0264 0.6788
1.0700 1.2719 0.2866 0.6239 -0.7798
-4.0135 -6.8429 -429.4826
67.7800 -431.9360 1.6514 3.2278
1.8729 1.4883 0.2149 0.6465 -0.7857
-3.9832 -6.8429 -429.4735
67.8000 -430.9779 1.6379 3.5624
2.1605 1.6699 0.1381 0.6673 -0.7914
-3.9527 -6.8427 -429.4644
67.8200 -435.6856 -0.3591 1.4143
1.9170 1.7954 0.0581 0.6860 -0.7967
-3.9218 -6.8424 -429.4554
67.8400 -440.5150 -1.7248 -1.3891
1.3624 1.8444 -0.0233 0.7025 -0.8017
-3.8906 -6.8420 -429.4463
67.8600 -440.9894 0.0970 -2.8563
0.7739 1.7994 -0.1043 0.7164 -0.8064
-3.8591 -6.8414 -429.4372
67.8800 -440.4605 0.1136 -2.5162
0.3932 1.6449 -0.1832 0.7277 -0.8107
-3.8273 -6.8408 -429.4281
67.9000 -439.7667 0.0215 -1.3506
0.3471 1.3714 -0.2583 0.7360 -0.8147
-3.7952 -6.8400 -429.4191
67.9200 -439.0852 0.0613 -0.1806
0.6170 0.9834 -0.3281 0.7411 -0.8183
-3.7628 -6.8391 -429.4100
67.9400 -438.2218 -0.2226 0.7248
1.0488 0.4979 -0.3909 0.7429 -0.8215
-3.7301 -6.8380 -429.4009
67.9600 -437.5846 -0.1072 1.3510
1.4190 -0.0621 -0.4453 0.7412 -0.8242
-3.6971 -6.8369 -429.3918
67.9800 -437.8245 0.5837 1.4764
1.5408 -0.6703 -0.4898 0.7358 -0.8265
-3.6638 -6.8356 -429.3827
68.0000 -439.5387 0.0937 0.8928
1.3367 -1.2975 -0.5229 0.7264 -0.8284
-3.6302 -6.8343 -429.3737
68.0200 -441.6151 -0.6415 0.0105
0.8435 -1.9131 -0.5431 0.7131 -0.8298
-3.5963 -6.8328 -429.3646
68.0400 -443.1073 0.5871 -0.6930
0.1754 -2.4876 -0.5491 0.6956 -0.8307
-3.5622 -6.8311 -429.3555
68.0600 -444.5654 0.0415 -0.9407 -
0.5308 -2.9919 -0.5394 0.6739 -0.8311
-3.5278 -6.8294 -429.3464
68.0800 -445.6054 -0.6508 -0.6965 -
1.1637 -3.3979 -0.5128 0.6479 -0.8310
-3.4931 -6.8275 -429.3373

68.1000 -445.7813 0.1292 -0.0653 -
1.6650 -3.6778 -0.4679 0.6175 -0.8304
-3.4582 -6.8256 -429.3282
68.1200 -445.6181 0.1155 0.6491 -
2.0150 -3.8072 -0.4039 0.5827 -0.8293
-3.4230 -6.8235 -429.3192
68.1400 -445.3550 -0.0688 0.9832 -
2.2029 -3.7690 -0.3206 0.5436 -0.8276
-3.3875 -6.8213 -429.3101
68.1600 -445.0752 -0.5429 0.7268 -
2.2027 -3.5590 -0.2187 0.5002 -0.8254
-3.3518 -6.8189 -429.3010
68.1800 -444.8185 0.3991 -0.1144 -
1.9845 -3.1874 -0.1002 0.4527 -0.8226
-3.3159 -6.8165 -429.2919
68.2000 -444.5041 0.6542 -1.0618 -
1.5260 -2.6740 0.0323 0.4010 -0.8193
-3.2796 -6.8139 -429.2828
68.2200 -444.0307 -0.8283 -1.5055 -
0.8343 -2.0479 0.1761 0.3453 -0.8154
-3.2432 -6.8113 -429.2737
68.2400 -442.2568 -0.4321 -0.8272
0.0071 -1.3417 0.3282 0.2857 -0.8110
-3.2065 -6.8085 -429.2646
68.2600 -438.3999 0.3975 0.6446
0.8403 -0.5889 0.4858 0.2222 -0.8060
-3.1695 -6.8056 -429.2556
68.2800 -435.1857 0.3182 1.7034
1.4851 0.1765 0.6459 0.1551 -0.8004
-3.1324 -6.8026 -429.2465
68.3000 -434.8376 0.3693 1.5278
1.7887 0.9215 0.8058 0.0845 -0.7943
-3.0949 -6.7994 -429.2374
68.3200 -435.5421 -0.3118 0.3516
1.7238 1.6164 0.9626 0.0103 -0.7877
-3.0573 -6.7962 -429.2283
68.3400 -436.1540 -0.3885 -0.8480
1.3795 2.2372 1.1132 -0.0671 -0.7805
-3.0195 -6.7928 -429.2192
68.3600 -436.1417 0.4703 -1.2792
0.8976 2.7652 1.2550 -0.1478 -0.7727
-2.9814 -6.7893 -429.2101
68.3800 -435.9022 0.2556 -0.9181
0.4185 3.1870 1.3849 -0.2316 -0.7645
-2.9431 -6.7858 -429.2010
68.4000 -435.5609 -0.6590 -0.2939
0.0603 3.4963 1.5000 -0.3183 -0.7557
-2.9046 -6.7821 -429.1919
68.4200 -434.9349 0.1649 0.3119 -
0.1510 3.6930 1.5978 -0.4079 -0.7464
-2.8659 -6.7782 -429.1828

68.4400 -433.9934 -0.3018 0.8178 -
0.2505 3.7830 1.6758 -0.5001 -0.7367
-2.8270 -6.7743 -429.1737
68.4600 -433.5000 0.8135 0.7709 -
0.2939 3.7754 1.7320 -0.5947 -0.7264
-2.7879 -6.7703 -429.1646
68.4800 -434.4670 0.3808 0.0043 -
0.3188 3.6799 1.7647 -0.6917 -0.7157
-2.7486 -6.7661 -429.1556
68.5000 -436.1946 -0.4062 -0.9041 -
0.3181 3.5061 1.7733 -0.7908 -0.7045
-2.7091 -6.7619 -429.1465
68.5200 -436.8757 -0.5638 -1.1484 -
0.2709 3.2640 1.7580 -0.8918 -0.6928
-2.6694 -6.7575 -429.1374
68.5400 -436.0595 -0.0912 -0.3907 -
0.1717 2.9636 1.7199 -0.9943 -0.6807
-2.6295 -6.7530 -429.1283
68.5600 -434.9390 -0.0641 0.7934 -
0.0275 2.6154 1.6600 -1.0981 -0.6681
-2.5894 -6.7484 -429.1192
68.5800 -434.7624 0.6394 1.2168
0.1313 2.2291 1.5800 -1.2029 -0.6552
-2.5492 -6.7437 -429.1101
68.6000 -436.8172 0.2864 0.2854
0.2680 1.8139 1.4814 -1.3083 -0.6417
-2.5088 -6.7389 -429.1010
68.6200 -439.1980 -0.4606 -1.0404
0.3588 1.3805 1.3659 -1.4140 -0.6279
-2.4682 -6.7340 -429.0919
68.6400 -439.5360 -0.4938 -1.2540
0.3851 0.9403 1.2354 -1.5197 -0.6137
-2.4275 -6.7290 -429.0828
68.6600 -438.3920 -0.4489 0.0797
0.3328 0.5042 1.0915 -1.6251 -0.5991
-2.3866 -6.7238 -429.0737
68.6800 -437.1673 0.1254 1.7274
0.1862 0.0829 0.9361 -1.7298 -0.5841
-2.3455 -6.7186 -429.0646
68.7000 -437.3264 1.1479 1.9250 -
0.0680 -0.3132 0.7709 -1.8336 -0.5687
-2.3043 -6.7132 -429.0555
68.7200 -440.5335 -0.0783 0.2370 -
0.3837 -0.6747 0.5977 -1.9361 -0.5530
-2.2629 -6.7078 -429.0464
68.7400 -443.9805 -0.8910 -1.6248 -
0.6455 -0.9943 0.4185 -2.0370 -0.5369
-2.2214 -6.7022 -429.0373
68.7600 -444.5628 -0.9200 -1.9263 -
0.7332 -1.2681 0.2353 -2.1359 -0.5204
-2.1798 -6.6965 -429.0282

68.7800 -443.5605 0.1827 -0.8485 -
0.5902 -1.4952 0.0501 -2.2327 -0.5036
-2.1380 -6.6907 -429.0191
68.8000 -442.3637 0.3756 0.3394 -
0.2582 -1.6785 -0.1347 -2.3269 -0.4865
-2.0961 -6.6848 -429.0100
68.8200 -441.8747 0.1401 0.8144
0.1587 -1.8224 -0.3171 -2.4183 -0.4690
-2.0540 -6.6788 -429.0009
68.8400 -441.7801 0.0675 0.9104
0.5049 -1.9319 -0.4947 -2.5065 -0.4512
-2.0119 -6.6727 -428.9918
68.8600 -441.7201 -0.3529 1.1337
0.6464 -2.0121 -0.6656 -2.5912 -0.4331
-1.9696 -6.6665 -428.9827
68.8800 -441.7016 0.1204 1.0047
0.5646 -2.0672 -0.8274 -2.6722 -0.4147
-1.9272 -6.6602 -428.9736
68.9000 -442.8984 0.7983 -0.0325
0.3292 -2.1017 -0.9782 -2.7491 -0.3960
-1.8847 -6.6538 -428.9645
68.9200 -445.0931 -0.3252 -1.4684
0.0542 -2.1202 -1.1161 -2.8216 -0.3771
-1.8421 -6.6473 -428.9554
68.9400 -446.4745 -0.6773 -2.1317 -
0.1445 -2.1262 -1.2395 -2.8894 -0.3578
-1.7993 -6.6406 -428.9463
68.9600 -445.6338 -0.4128 -1.2857 -
0.2143 -2.1212 -1.3469 -2.9522 -0.3383
-1.7565 -6.6339 -428.9372
68.9800 -443.4448 0.4000 0.5544 -
0.1893 -2.1059 -1.4372 -3.0097 -0.3185
-1.7136 -6.6271 -428.9281
69.0000 -441.5841 0.7191 2.0465 -
0.1440 -2.0813 -1.5097 -3.0616 -0.2984
-1.6706 -6.6201 -428.9190
69.0200 -441.6559 0.7859 1.9946 -
0.1311 -2.0472 -1.5634 -3.1077 -0.2781
-1.6275 -6.6131 -428.9099
69.0400 -443.5982 0.2106 0.2979 -
0.1412 -2.0023 -1.5982 -3.1477 -0.2576
-1.5844 -6.6059 -428.9008
69.0600 -445.6956 -0.6680 -1.6005 -
0.1134 -1.9449 -1.6137 -3.1814 -0.2368
-1.5411 -6.5987 -428.8917
69.0800 -446.1732 -0.6523 -2.1681
0.0175 -1.8736 -1.6103 -3.2087 -0.2158
-1.4978 -6.5913 -428.8826
69.1000 -444.4197 -0.2757 -1.0700
0.2652 -1.7882 -1.5883 -3.2295 -0.1946
-1.4544 -6.5839 -428.8735

69.1200 -441.9971 0.5355 0.6388
0.5425 -1.6897 -1.5487 -3.2438 -0.1731
-1.4109 -6.5763 -428.8644
69.1400 -440.7029 0.4592 1.6113
0.7128 -1.5786 -1.4924 -3.2515 -0.1515
-1.3674 -6.5687 -428.8553
69.1600 -441.3425 -0.2536 1.2740
0.6658 -1.4552 -1.4207 -3.2526 -0.1297
-1.3239 -6.5609 -428.8462
69.1800 -442.6736 -0.0149 0.0776
0.3557 -1.3198 -1.3353 -3.2472 -0.1078
-1.2803 -6.5530 -428.8371
69.2000 -443.5712 -0.7782 -0.7498 -
0.1513 -1.1738 -1.2380 -3.2353 -0.0857
-1.2366 -6.5451 -428.8280
69.2200 -443.4163 0.0521 -0.5258 -
0.7356 -1.0193 -1.1306 -3.2169 -0.0634
-1.1929 -6.5370 -428.8189
69.2400 -442.8830 -0.2001 0.2077 -
1.2340 -0.8615 -1.0149 -3.1919 -0.0410
-1.1491 -6.5289 -428.8098
69.2600 -442.3411 -0.1668 0.5379 -
1.4966 -0.7056 -0.8929 -3.1606 -0.0185
-1.1054 -6.5206 -428.8007
69.2800 -442.0738 0.8588 0.0331 -
1.4175 -0.5571 -0.7666 -3.1228 0.0041
-1.0616 -6.5123 -428.7916
69.3000 -441.9376 -0.2118 -0.8890 -
0.9641 -0.4196 -0.6377 -3.0787 0.0267
-1.0177 -6.5038 -428.7825
69.3200 -441.7723 -0.1468 -1.6327 -
0.1684 -0.2937 -0.5083 -3.0282 0.0495
-0.9739 -6.4952 -428.7734
69.3400 -441.0456 -1.1747 -1.3545
0.8208 -0.1789 -0.3799 -2.9715 0.0723
-0.9300 -6.4866 -428.7643
69.3600 -437.2590 -0.4357 0.3547
1.7708 -0.0743 -0.2545 -2.9086 0.0952
-0.8861 -6.4778 -428.7552
69.3800 -432.9946 0.7340 2.3613
2.4051 0.0210 -0.1338 -2.8395 0.1181
-0.8422 -6.4690 -428.7461
69.4000 -431.8835 0.9643 2.9876
2.4877 0.1072 -0.0194 -2.7642 0.1410
-0.7984 -6.4600 -428.7370
69.4200 -434.6439 -0.1656 1.7928
1.9303 0.1844 0.0869 -2.6829 0.1639
-0.7545 -6.4510 -428.7279
69.4400 -438.1690 -0.6644 -0.0749
0.8606 0.2564 0.1838 -2.5956 0.1869
-0.7106 -6.4419 -428.7188

69.4600	-439.6798	-0.1766	-1.3253	-
0.4736	0.3290	0.2699	-2.5023	0.2098
	-0.6667	-6.4326	-428.7097	
69.4800	-440.5201	0.0250	-1.6787	-
1.7719	0.4081	0.3439	-2.4031	0.2327
	-0.6229	-6.4233	-428.7006	
69.5000	-441.0228	0.0438	-1.7274	-
2.7292	0.4994	0.4049	-2.2981	0.2556
	-0.5791	-6.4139	-428.6915	
69.5200	-441.0104	0.1594	-1.8491	-
3.1176	0.6081	0.4521	-2.1873	0.2784
	-0.5352	-6.4044	-428.6824	
69.5400	-440.3144	-0.1780	-1.8108	-
2.8619	0.7346	0.4848	-2.0709	0.3011
	-0.4915	-6.3947	-428.6733	
69.5600	-439.1495	0.0358	-1.3217	-
2.0420	0.8725	0.5026	-1.9489	0.3238
	-0.4477	-6.3850	-428.6642	
69.5800	-437.5286	-0.8889	-0.2056	-
0.8771	1.0113	0.5056	-1.8213	0.3464
	-0.4040	-6.3752	-428.6551	
69.6000	-433.9142	-0.5054	1.2750	
0.3727	1.1396	0.4937	-1.6884	0.3689
	-0.3604	-6.3653	-428.6460	
69.6200	-430.4080	0.5956	2.3316	
1.4757	1.2458	0.4671	-1.5503	0.3913
	-0.3168	-6.3553	-428.6369	
69.6400	-429.8339	0.5977	2.0108	
2.2602	1.3186	0.4265	-1.4071	0.4135
	-0.2732	-6.3452	-428.6278	
69.6600	-431.7658	0.1417	0.3896	
2.6481	1.3477	0.3724	-1.2590	0.4357
	-0.2297	-6.3351	-428.6187	
69.6800	-433.5682	-0.6383	-1.1965	
2.6358	1.3263	0.3057	-1.1063	0.4576
	-0.1862	-6.3248	-428.6096	
69.7000	-433.3908	-0.3237	-1.3979	
2.2645	1.2522	0.2274	-0.9492	0.4795
	-0.1429	-6.3144	-428.6005	
69.7200	-432.4291	0.0914	-0.2473	
1.6212	1.1274	0.1386	-0.7879	0.5011
	-0.0995	-6.3040	-428.5914	
69.7400	-431.8039	0.1284	0.8261	
0.8189	0.9573	0.0408	-0.6228	0.5226
	-0.0563	-6.2934	-428.5823	
69.7600	-432.7909	0.5341	0.7962	-
0.0273	0.7495	-0.0642	-0.4540	0.5439
	-0.0132	-6.2828	-428.5732	
69.7800	-434.8400	-0.2072	-0.0215	-
0.8043	0.5128	-0.1749	-0.2819	0.5650
	0.0299	-6.2720	-428.5641	

69.8000 -435.9858 0.1909 -0.6774 -
1.4084 0.2572 -0.2892 -0.1068 0.5858
0.0729 -6.2612 -428.5550
69.8200 -436.3869 0.0703 -0.8205 -
1.7275 -0.0062 -0.4053 0.0708 0.6065
0.1158 -6.2503 -428.5459
69.8400 -436.6412 0.3468 -0.5934 -
1.7038 -0.2649 -0.5212 0.2508 0.6269
0.1586 -6.2393 -428.5369
69.8600 -436.6019 -0.5824 -0.1345 -
1.3724 -0.5062 -0.6349 0.4328 0.6470
0.2013 -6.2282 -428.5278
69.8800 -435.2509 0.1126 0.5509 -
0.8410 -0.7178 -0.7445 0.6164 0.6669
0.2439 -6.2170 -428.5187
69.9000 -433.6165 -0.1867 0.9485 -
0.2674 -0.8883 -0.8479 0.8012 0.6866
0.2863 -6.2058 -428.5096
69.9200 -433.2565 0.4595 0.4933
0.2072 -1.0088 -0.9434 0.9871 0.7059
0.3287 -6.1944 -428.5005
69.9400 -433.7713 0.1254 -0.4886
0.4856 -1.0753 -1.0290 1.1736 0.7249
0.3710 -6.1829 -428.4914
69.9600 -434.3148 -0.3731 -0.8716
0.5407 -1.0892 -1.1031 1.3604 0.7437
0.4131 -6.1714 -428.4823
69.9800 -434.1978 -0.1884 -0.2698
0.4091 -1.0562 -1.1642 1.5472 0.7621
0.4551 -6.1598 -428.4732
70.0000 -433.1988 -0.1938 0.5568
0.1637 -0.9844 -1.2110 1.7338 0.7802
0.4969 -6.1481 -428.4641
70.0200 -432.3538 0.4108 0.9292 -
0.0992 -0.8825 -1.2421 1.9198 0.7979
0.5386 -6.1363 -428.4550
70.0400 -432.6964 0.2267 0.3801 -
0.2968 -0.7607 -1.2565 2.1048 0.8153
0.5802 -6.1244 -428.4459
70.0600 -433.8715 -0.2018 -0.7845 -
0.3714 -0.6302 -1.2533 2.2887 0.8323
0.6216 -6.1124 -428.4368
70.0800 -434.5986 -0.4532 -1.4458 -
0.2939 -0.5025 -1.2316 2.4711 0.8490
0.6629 -6.1003 -428.4277
70.1000 -433.2096 -0.4746 -0.9481 -
0.0767 -0.3904 -1.1910 2.6517 0.8653
0.7040 -6.0882 -428.4186
70.1200 -430.4655 0.6335 0.2267
0.2013 -0.3047 -1.1310 2.8302 0.8811
0.7450 -6.0760 -428.4096

70.1400 -428.9282 0.5142 1.2160
0.4215 -0.2540 -1.0518 3.0065 0.8966
0.7858 -6.0636 -428.4005
70.1600 -428.4868 0.1147 1.6044
0.4952 -0.2434 -0.9536 3.1801 0.9116
0.8264 -6.0512 -428.3914
70.1800 -428.2330 0.2737 1.3862
0.3888 -0.2751 -0.8374 3.3508 0.9262
0.8668 -6.0387 -428.3823
70.2000 -428.7699 1.0128 0.2965
0.1690 -0.3471 -0.7043 3.5184 0.9404
0.9071 -6.0262 -428.3732
70.2200 -431.6092 -0.5883 -1.4122 -
0.0347 -0.4533 -0.5558 3.6825 0.9540
0.9472 -6.0135 -428.3641
70.2400 -433.5903 -1.2981 -2.2530 -
0.1206 -0.5833 -0.3935 3.8429 0.9673
0.9871 -6.0008 -428.3550
70.2600 -431.5394 -0.2952 -1.2318 -
0.0596 -0.7230 -0.2197 3.9994 0.9800
1.0268 -5.9880 -428.3459
70.2800 -427.5417 0.4519 0.6997
0.0688 -0.8581 -0.0365 4.1515 0.9923
1.0663 -5.9750 -428.3368
70.3000 -425.9355 1.7746 1.7505
0.1409 -0.9748 0.1537 4.2992 1.0040
1.1056 -5.9621 -428.3278
70.3200 -427.0166 -0.7703 1.2460
0.0759 -1.0601 0.3487 4.4420 1.0152
1.1447 -5.9490 -428.3187
70.3400 -428.2636 -0.3238 0.1745 -
0.1328 -1.1024 0.5458 4.5797 1.0259
1.1836 -5.9358 -428.3096
70.3600 -428.3554 0.4784 -0.2996 -
0.4272 -1.0916 0.7430 4.7121 1.0361
1.2223 -5.9226 -428.3005
70.3800 -428.1534 -0.1027 -0.2215 -
0.6815 -1.0215 0.9377 4.8389 1.0457
1.2607 -5.9093 -428.2914
70.4000 -427.8810 0.5318 -0.4789 -
0.7447 -0.8924 1.1278 4.9598 1.0548
1.2990 -5.8959 -428.2823
70.4200 -427.5589 0.0948 -1.1287 -
0.5213 -0.7107 1.3111 5.0746 1.0632
1.3370 -5.8824 -428.2733
70.4400 -427.0566 -0.3339 -1.2043 -
0.0444 -0.4862 1.4856 5.1830 1.0711
1.3747 -5.8688 -428.2642
70.4600 -425.6536 -0.7403 0.0188
0.5422 -0.2296 1.6495 5.2848 1.0784
1.4123 -5.8552 -428.2551

70.4800 -421.7095 -0.0354 1.6636
1.0171 0.0484 1.8009 5.3797 1.0851
1.4496 -5.8414 -428.2460
70.5000 -419.4017 1.5904 2.0813
1.1733 0.3371 1.9382 5.4674 1.0912
1.4866 -5.8276 -428.2369
70.5200 -421.5072 -0.3007 0.8158
0.9313 0.6273 2.0595 5.5477 1.0966
1.5234 -5.8138 -428.2278
70.5400 -424.2750 -0.5040 -0.7675
0.4043 0.9131 2.1627 5.6204 1.1014
1.5599 -5.7998 -428.2188
70.5600 -423.9597 -0.7364 -1.2539 -
0.2142 1.1888 2.2456 5.6851 1.1056
1.5962 -5.7858 -428.2097
70.5800 -422.4324 0.6338 -0.8509 -
0.7208 1.4482 2.3058 5.7417 1.1090
1.6322 -5.7716 -428.2006
70.6000 -421.9045 0.3555 -0.3760 -
0.9740 1.6843 2.3415 5.7899 1.1118
1.6679 -5.7574 -428.1915
70.6200 -421.9580 0.1255 -0.2270 -
0.9605 1.8895 2.3506 5.8295 1.1140
1.7034 -5.7432 -428.1825
70.6400 -421.9999 -0.3414 -0.1052 -
0.7427 2.0552 2.3320 5.8604 1.1154
1.7386 -5.7288 -428.1734
70.6600 -420.7375 -0.6624 0.3970 -
0.4091 2.1732 2.2849 5.8823 1.1161
1.7735 -5.7144 -428.1643
70.6800 -418.7594 0.5324 0.8801 -
0.0631 2.2337 2.2092 5.8954 1.1162
1.8081 -5.6999 -428.1552
70.7000 -418.9723 0.9272 0.6803
0.2228 2.2257 2.1056 5.8996 1.1156
1.8425 -5.6853 -428.1461
70.7200 -420.6281 -0.4727 0.0186
0.4348 2.1373 1.9753 5.8951 1.1142
1.8765 -5.6706 -428.1371
70.7400 -421.2483 -0.6022 -0.4445
0.6225 1.9586 1.8206 5.8820 1.1122
1.9103 -5.6559 -428.1280
70.7600 -421.3115 0.1357 -0.5921
0.8396 1.6843 1.6440 5.8604 1.1095
1.9437 -5.6411 -428.1189
70.7800 -421.4158 0.5165 -0.8484
1.0797 1.3173 1.4488 5.8305 1.1061
1.9769 -5.6262 -428.1099
70.8000 -422.6153 0.3652 -1.1418
1.2640 0.8711 1.2384 5.7925 1.1021
2.0097 -5.6112 -428.1008

70.8200 -423.9984 -1.1794 -0.5382
1.2338 0.3672 1.0164 5.7465 1.0974
2.0423 -5.5962 -428.0917
70.8400 -423.3526 -0.7935 1.4738
0.8826 -0.1676 0.7862 5.6927 1.0921
2.0745 -5.5811 -428.0826
70.8600 -421.3343 0.5153 3.4804
0.2470 -0.7041 0.5514 5.6312 1.0861
2.1064 -5.5659 -428.0736
70.8800 -421.4524 1.8192 3.1617 -
0.5568 -1.2121 0.3155 5.5622 1.0795
2.1379 -5.5506 -428.0645
70.9000 -428.7289 -0.6998 0.1769 -
1.3628 -1.6619 0.0821 5.4859 1.0724
2.1692 -5.5353 -428.0554
70.9200 -434.6270 -2.0613 -3.1604 -
1.9631 -2.0235 -0.1454 5.4025 1.0647
2.2001 -5.5199 -428.0464
70.9400 -434.3359 0.0890 -4.6698 -
2.1540 -2.2687 -0.3634 5.3121 1.0564
2.2307 -5.5044 -428.0373
70.9600 -433.2430 -0.1345 -3.7264 -
1.8310 -2.3782 -0.5689 5.2148 1.0475
2.2610 -5.4889 -428.0282
70.9800 -431.5872 -1.0480 -0.8237 -
1.1027 -2.3510 -0.7593 5.1108 1.0382
2.2909 -5.4733 -428.0192
71.0000 -426.3761 -0.6932 2.7296 -
0.2167 -2.1971 -0.9329 5.0004 1.0283
2.3205 -5.4576 -428.0101
71.0200 -420.7384 2.3421 4.8778
0.5551 -1.9305 -1.0883 4.8836 1.0180
2.3498 -5.4418 -428.0010
71.0400 -420.8907 2.0840 3.9393
1.0012 -1.5690 -1.2252 4.7606 1.0071
2.3787 -5.4260 -427.9920
71.0600 -427.2389 -1.2691 0.6327
1.0872 -1.1359 -1.3429 4.6316 0.9959
2.4072 -5.4101 -427.9829
71.0800 -430.8906 -1.6515 -2.3057
0.9044 -0.6603 -1.4413 4.4967 0.9842
2.4354 -5.3941 -427.9738
71.1000 -429.5606 -0.4191 -2.9303
0.5881 -0.1753 -1.5200 4.3562 0.9720
2.4633 -5.3780 -427.9648
71.1200 -427.4741 0.9757 -1.8004
0.3026 0.2849 -1.5788 4.2100 0.9595
2.4907 -5.3619 -427.9557
71.1400 -426.4973 0.4456 -0.6392
0.2154 0.6872 -1.6175 4.0584 0.9466
2.5179 -5.3457 -427.9467

71.1600 -426.0420 -0.3412 -0.1543
0.4211 1.0050 -1.6360 3.9016 0.9333
2.5447 -5.3295 -427.9376
71.1800 -425.6359 -0.0815 -0.1154
0.8574 1.2232 -1.6344 3.7396 0.9197
2.5711 -5.3131 -427.9285
71.2000 -425.0532 0.0764 -0.0685
1.3692 1.3358 -1.6129 3.5727 0.9058
2.5971 -5.2967 -427.9195
71.2200 -424.5106 0.1369 0.1611
1.7748 1.3429 -1.5724 3.4010 0.8915
2.6228 -5.2803 -427.9104
71.2400 -424.4066 -0.2939 0.5920
1.9039 1.2505 -1.5137 3.2248 0.8770
2.6481 -5.2638 -427.9014
71.2600 -424.4704 -0.2241 0.9529
1.6562 1.0702 -1.4380 3.0442 0.8621
2.6730 -5.2472 -427.8923
71.2800 -424.5972 0.6516 0.9044
1.0233 0.8217 -1.3467 2.8595 0.8470
2.6976 -5.2305 -427.8833
71.3000 -426.6221 0.2774 0.2669
0.1195 0.5318 -1.2413 2.6709 0.8317
2.7218 -5.2138 -427.8742
71.3200 -429.8734 -0.3602 -0.6694 -
0.8530 0.2293 -1.1235 2.4786 0.8161
2.7456 -5.1970 -427.8652
71.3400 -430.9792 -0.0058 -1.1325 -
1.6682 -0.0580 -0.9949 2.2828 0.8004
2.7690 -5.1801 -427.8561
71.3600 -431.4049 0.5947 -0.8790 -
2.1731 -0.3034 -0.8574 2.0838 0.7844
2.7921 -5.1632 -427.8471
71.3800 -431.5956 -0.2753 -0.1919 -
2.3240 -0.4835 -0.7129 1.8818 0.7682
2.8148 -5.1462 -427.8380
71.4000 -431.0475 -0.0266 0.6139 -
2.1556 -0.5845 -0.5634 1.6771 0.7519
2.8371 -5.1291 -427.8289
71.4200 -429.7366 0.0202 1.1399 -
1.7289 -0.6042 -0.4105 1.4700 0.7355
2.8590 -5.1120 -427.8199
71.4400 -429.1025 0.0484 0.7853 -
1.0918 -0.5512 -0.2558 1.2607 0.7189
2.8805 -5.0948 -427.8109
71.4600 -429.3259 0.5548 -0.4372 -
0.3191 -0.4405 -0.1006 1.0494 0.7023
2.9016 -5.0775 -427.8018
71.4800 -429.6354 -0.4931 -1.4681
0.4696 -0.2909 0.0538 0.8364 0.6855
2.9224 -5.0602 -427.7928

71.5000 -429.1947 -0.7993 -1.1385
1.1494 -0.1239 0.2066 0.6220 0.6687
2.9427 -5.0428 -427.7837
71.5200 -426.0968 0.2706 0.3615
1.6168 0.0398 0.3569 0.4063 0.6518
2.9627 -5.0254 -427.7747
71.5400 -423.5792 1.3411 1.5813
1.7818 0.1815 0.5039 0.1896 0.6348
2.9822 -5.0079 -427.7656
71.5600 -424.7743 -0.0022 1.4079
1.5985 0.2878 0.6468 -0.0279 0.6179
3.0014 -4.9903 -427.7566
71.5800 -427.6779 -0.4627 0.0961
1.1510 0.3529 0.7850 -0.2460 0.6009
3.0202 -4.9726 -427.7475
71.6000 -428.8954 -0.1548 -1.1394
0.5979 0.3753 0.9177 -0.4646 0.5839
3.0386 -4.9549 -427.7385
71.6200 -429.2997 0.3961 -1.2975
0.0880 0.3567 1.0441 -0.6833 0.5670
3.0566 -4.9372 -427.7295
71.6400 -429.4924 0.0818 -0.5316 -
0.2869 0.3031 1.1631 -0.9021 0.5501
3.0741 -4.9194 -427.7204
71.6600 -429.2750 -0.0426 0.4257 -
0.5112 0.2246 1.2738 -1.1207 0.5333
3.0913 -4.9015 -427.7114
71.6800 -428.6816 -0.2013 0.9074 -
0.6398 0.1330 1.3752 -1.3390 0.5165
3.1081 -4.8835 -427.7023
71.7000 -428.4139 0.4266 0.8589 -
0.7214 0.0419 1.4665 -1.5568 0.4998
3.1245 -4.8655 -427.6933
71.7200 -429.2252 0.3007 0.3575 -
0.7786 -0.0360 1.5469 -1.7739 0.4833
3.1405 -4.8475 -427.6843
71.7400 -430.2986 -0.3694 -0.3981 -
0.7830 -0.0917 1.6157 -1.9902 0.4668
3.1560 -4.8293 -427.6752
71.7600 -430.4710 -0.0817 -0.6613 -
0.6970 -0.1206 1.6721 -2.2056 0.4505
3.1712 -4.8112 -427.6662
71.7800 -430.2714 -0.1595 -0.3452 -
0.4974 -0.1220 1.7157 -2.4197 0.4344
3.1860 -4.7929 -427.6572
71.8000 -429.9608 0.3373 -0.0817 -
0.1989 -0.1003 1.7458 -2.6326 0.4184
3.2003 -4.7746 -427.6481
71.8200 -429.3609 -0.1347 0.1338
0.1485 -0.0629 1.7619 -2.8438 0.4026
3.2143 -4.7563 -427.6391

71.8400 -428.4647 -0.4196 0.5166
0.4621 -0.0193 1.7634 -3.0534 0.3870
3.2278 -4.7378 -427.6301
71.8600 -428.1255 0.9376 0.7981
0.6731 0.0191 1.7501 -3.2610 0.3717
3.2409 -4.7194 -427.6210
71.8800 -429.1420 -0.1685 0.4721
0.7466 0.0431 1.7217 -3.4664 0.3566
3.2536 -4.7008 -427.6120
71.9000 -430.6280 -0.5644 -0.1211
0.7031 0.0497 1.6778 -3.6694 0.3417
3.2659 -4.6822 -427.6030
71.9200 -431.4295 -0.0564 -0.6174
0.5866 0.0406 1.6183 -3.8697 0.3271
3.2778 -4.6636 -427.5940
71.9400 -431.9943 0.0691 -0.9372
0.4453 0.0219 1.5431 -4.0673 0.3128
3.2893 -4.6449 -427.5849
71.9600 -432.2907 -0.5080 -0.7549
0.2732 0.0038 1.4524 -4.2617 0.2988
3.3003 -4.6261 -427.5759
71.9800 -431.8626 -0.4478 0.1424
0.0324 -0.0017 1.3463 -4.4529 0.2851
3.3110 -4.6073 -427.5669
72.0000 -430.7690 0.1759 1.2466 -
0.2960 0.0160 1.2255 -4.6404 0.2718
3.3212 -4.5884 -427.5579
72.0200 -430.2097 0.7507 1.5916 -
0.6879 0.0639 1.0904 -4.8242 0.2588
3.3310 -4.5695 -427.5488
72.0400 -432.4361 0.3791 0.4865 -
1.0592 0.1446 0.9418 -5.0040 0.2461
3.3404 -4.5505 -427.5398
72.0600 -435.9043 -0.8897 -1.2980 -
1.2861 0.2564 0.7806 -5.1795 0.2339
3.3494 -4.5315 -427.5308
72.0800 -436.8209 -0.8365 -1.8494 -
1.2516 0.3912 0.6077 -5.3505 0.2220
3.3580 -4.5124 -427.5218
72.1000 -434.4312 -0.1852 -0.4742 -
0.9223 0.5354 0.4242 -5.5167 0.2106
3.3661 -4.4933 -427.5128
72.1200 -431.5823 1.0039 1.2734 -
0.3680 0.6726 0.2317 -5.6779 0.1996
3.3738 -4.4741 -427.5038
72.1400 -431.0854 0.4576 1.5561
0.2933 0.7856 0.0318 -5.8338 0.1890
3.3812 -4.4548 -427.4947
72.1600 -432.5840 -0.0768 0.3469
0.9466 0.8561 -0.1735 -5.9842 0.1789
3.3881 -4.4355 -427.4857

72.1800 -434.0468 -0.7606 -0.8859
1.4775 0.8687 -0.3825 -6.1289 0.1692
3.3945 -4.4161 -427.4767
72.2000 -433.9809 -0.1773 -1.0833
1.7947 0.8136 -0.5929 -6.2674 0.1601
3.4006 -4.3967 -427.4677
72.2200 -433.1594 -0.1941 -0.2004
1.8217 0.6890 -0.8027 -6.3997 0.1514
3.4063 -4.3773 -427.4587
72.2400 -432.5267 0.7933 0.8925
1.5037 0.5022 -1.0094 -6.5254 0.1433
3.4115 -4.3578 -427.4497
72.2600 -433.3277 0.3181 1.1533
0.8547 0.2683 -1.2105 -6.6444 0.1357
3.4163 -4.3382 -427.4407
72.2800 -435.7868 -0.4620 0.6635 -
0.0080 0.0065 -1.4032 -6.7563 0.1286
3.4207 -4.3186 -427.4317
72.3000 -438.1251 0.1795 -0.1001 -
0.9010 -0.2638 -1.5850 -6.8610 0.1221
3.4247 -4.2989 -427.4226
72.3200 -439.8253 -0.1662 -0.7987 -
1.6016 -0.5228 -1.7534 -6.9582 0.1162
3.4283 -4.2792 -427.4136
72.3400 -441.3872 0.6667 -1.3319 -
1.9283 -0.7508 -1.9058 -7.0476 0.1109
3.4314 -4.2595 -427.4046
72.3600 -442.1850 -0.4427 -1.4042 -
1.8095 -0.9312 -2.0399 -7.1291 0.1062
3.4342 -4.2397 -427.3956
72.3800 -441.1169 -0.8461 -0.7450 -
1.2959 -1.0567 -2.1536 -7.2024 0.1021
3.4365 -4.2198 -427.3866
72.4000 -438.4213 0.3277 0.4127 -
0.5510 -1.1271 -2.2452 -7.2673 0.0986
3.4384 -4.1999 -427.3776
72.4200 -436.3559 0.7398 1.3660
0.2103 -1.1448 -2.3133 -7.3236 0.0958
3.4400 -4.1800 -427.3686
72.4400 -436.3515 0.1949 1.5874
0.7914 -1.1138 -2.3571 -7.3711 0.0936
3.4411 -4.1600 -427.3596
72.4600 -436.9167 -0.4333 1.1088
1.0781 -1.0406 -2.3761 -7.4097 0.0922
3.4418 -4.1399 -427.3506
72.4800 -437.5486 -0.2208 0.4677
1.0531 -0.9345 -2.3705 -7.4392 0.0914
3.4421 -4.1198 -427.3416
72.5000 -438.0161 0.1569 -0.0176
0.7596 -0.8042 -2.3406 -7.4597 0.0912
3.4420 -4.0997 -427.3326

72.5200 -438.4821 -0.2634 -0.4005
0.2983 -0.6557 -2.2875 -7.4711 0.0918
3.4415 -4.0795 -427.3236
72.5400 -438.7752 0.1546 -0.5975 -
0.1808 -0.4939 -2.2123 -7.4736 0.0931
3.4406 -4.0593 -427.3146
72.5600 -438.7522 0.0493 -0.5250 -
0.5307 -0.3243 -2.1165 -7.4672 0.0951
3.4393 -4.0390 -427.3057
72.5800 -438.5253 0.0121 -0.3783 -
0.6737 -0.1521 -2.0018 -7.4521 0.0978
3.4376 -4.0187 -427.2967
72.6000 -438.1699 0.1951 -0.2599 -
0.5998 0.0167 -1.8698 -7.4285 0.1011
3.4356 -3.9983 -427.2877
72.6200 -437.5443 -0.4590 -0.1580 -
0.3512 0.1759 -1.7222 -7.3965 0.1052
3.4331 -3.9779 -427.2787
72.6400 -436.2060 0.1003 0.1359 -
0.0253 0.3175 -1.5607 -7.3564 0.1099
3.4303 -3.9575 -427.2697
72.6600 -435.0183 0.1730 0.4627
0.2750 0.4350 -1.3870 -7.3083 0.1153
3.4271 -3.9370 -427.2607
72.6800 -434.7026 -0.2645 0.5019
0.4696 0.5220 -1.2028 -7.2525 0.1212
3.4234 -3.9164 -427.2517
72.7000 -434.5715 0.2984 0.2921
0.5250 0.5721 -1.0101 -7.1892 0.1279
3.4195 -3.8959 -427.2427
72.7200 -434.5054 0.2339 0.0863
0.4511 0.5823 -0.8110 -7.1186 0.1351
3.4151 -3.8752 -427.2338
72.7400 -434.5249 -0.1184 0.0101
0.2842 0.5538 -0.6075 -7.0409 0.1429
3.4104 -3.8546 -427.2248
72.7600 -434.6199 -0.1397 -0.1369
0.0996 0.4915 -0.4022 -6.9562 0.1512
3.4053 -3.8339 -427.2158
72.7800 -434.6957 -0.0178 -0.3834 -
0.0223 0.4028 -0.1976 -6.8649 0.1601
3.3998 -3.8131 -427.2068
72.8000 -434.5534 -0.0471 -0.5458 -
0.0288 0.2967 0.0038 -6.7670 0.1696
3.3940 -3.7924 -427.1978
72.8200 -434.0519 0.2175 -0.3513
0.0681 0.1839 0.1992 -6.6628 0.1795
3.3878 -3.7715 -427.1889
72.8400 -433.4718 0.0529 0.1986
0.1845 0.0764 0.3862 -6.5525 0.1899
3.3813 -3.7507 -427.1799

72.8600 -432.8678 0.0149 0.7486
0.2159 -0.0149 0.5625 -6.4362 0.2009
3.3744 -3.7298 -427.1709
72.8800 -432.2034 -0.6282 1.0312
0.0904 -0.0799 0.7259 -6.3143 0.2122
3.3671 -3.7088 -427.1619
72.9000 -431.9008 0.7916 0.7179 -
0.1763 -0.1104 0.8748 -6.1867 0.2240
3.3595 -3.6879 -427.1530
72.9200 -432.8100 0.3103 -0.3383 -
0.4828 -0.1019 1.0074 -6.0538 0.2362
3.3516 -3.6669 -427.1440
72.9400 -434.3334 -0.0282 -1.4197 -
0.7215 -0.0541 1.1226 -5.9157 0.2488
3.3433 -3.6458 -427.1350
72.9600 -434.7874 -1.1468 -1.4902 -
0.8023 0.0305 1.2195 -5.7726 0.2618
3.3347 -3.6247 -427.1260
72.9800 -432.3007 -0.0903 -0.1902 -
0.6877 0.1448 1.2971 -5.6246 0.2751
3.3257 -3.6036 -427.1171
73.0000 -429.1548 1.0048 1.2405 -
0.4000 0.2793 1.3549 -5.4721 0.2887
3.3164 -3.5825 -427.1081
73.0200 -428.5130 0.1549 1.3578 -
0.0048 0.4226 1.3924 -5.3151 0.3027
3.3068 -3.5613 -427.0991
73.0400 -429.4008 -0.0626 0.2250
0.4267 0.5604 1.4093 -5.1538 0.3170
3.2969 -3.5401 -427.0902
73.0600 -430.2331 -0.3569 -0.9414
0.8078 0.6771 1.4053 -4.9885 0.3315
3.2866 -3.5188 -427.0812
73.0800 -429.9472 -0.4799 -1.2123
1.0609 0.7577 1.3805 -4.8194 0.3463
3.2761 -3.4975 -427.0723
73.1000 -428.7944 0.5997 -0.6213
1.1306 0.7907 1.3350 -4.6464 0.3613
3.2652 -3.4762 -427.0633
73.1200 -427.9925 0.0489 0.2431
0.9939 0.7697 1.2696 -4.4700 0.3766
3.2540 -3.4548 -427.0543
73.1400 -427.7658 -0.0895 1.0244
0.6476 0.6949 1.1853 -4.2902 0.3920
3.2424 -3.4334 -427.0454
73.1600 -427.6258 -0.1527 1.5063
0.1355 0.5727 1.0836 -4.1072 0.4077
3.2306 -3.4120 -427.0364
73.1800 -428.0089 1.1813 0.7724 -
0.4135 0.4146 0.9664 -3.9212 0.4235
3.2185 -3.3906 -427.0275

73.2000 -431.2234 0.6360 -1.0948 -
0.8399 0.2336 0.8356 -3.7323 0.4394
3.2061 -3.3691 -427.0185
73.2200 -434.1269 -1.8827 -2.3761 -
0.9952 0.0436 0.6933 -3.5409 0.4554
3.1934 -3.3476 -427.0096
73.2400 -432.8046 -1.1086 -1.4663 -
0.7986 -0.1413 0.5420 -3.3469 0.4716
3.1804 -3.3260 -427.0006
73.2600 -428.5039 0.8455 0.7628 -
0.3558 -0.3084 0.3836 -3.1507 0.4878
3.1671 -3.3045 -426.9917
73.2800 -425.9024 1.9150 2.0621
0.1473 -0.4481 0.2205 -2.9524 0.5041
3.1535 -3.2829 -426.9827
73.3000 -427.6882 0.3979 1.2814
0.5307 -0.5526 0.0549 -2.7523 0.5204
3.1397 -3.2612 -426.9738
73.3200 -430.7508 -1.1472 -0.3609
0.6698 -0.6162 -0.1112 -2.5506 0.5368
3.1255 -3.2396 -426.9648
73.3400 -431.4092 -1.4333 -0.8097
0.4963 -0.6367 -0.2754 -2.3474 0.5531
3.1111 -3.2179 -426.9559
73.3600 -429.6479 0.0422 0.1727
0.0904 -0.6156 -0.4357 -2.1429 0.5695
3.0964 -3.1962 -426.9469
73.3800 -427.8888 0.9259 0.9439 -
0.3423 -0.5582 -0.5904 -1.9374 0.5858
3.0815 -3.1745 -426.9380
73.4000 -428.5505 1.3857 0.1833 -
0.6168 -0.4704 -0.7380 -1.7309 0.6020
3.0663 -3.1527 -426.9291
73.4200 -431.6064 -0.1777 -1.5852 -
0.6507 -0.3597 -0.8769 -1.5237 0.6182
3.0508 -3.1309 -426.9201
73.4400 -433.3977 -2.2621 -2.2012 -
0.4337 -0.2363 -1.0059 -1.3158 0.6342
3.0351 -3.1091 -426.9112
73.4600 -429.9709 -0.8702 -0.6103 -
0.0350 -0.1126 -1.1237 -1.1073 0.6502
3.0191 -3.0873 -426.9022
73.4800 -424.2170 1.2200 1.8333
0.3835 -0.0014 -1.2291 -0.8985 0.6660
3.0029 -3.0654 -426.8933
73.5000 -422.3001 2.2451 2.6796
0.6468 0.0851 -1.3210 -0.6894 0.6817
2.9864 -3.0435 -426.8844
73.5200 -426.2859 0.0905 0.9988
0.6607 0.1350 -1.3986 -0.4801 0.6972
2.9697 -3.0216 -426.8754

73.5400	-430.2904	-1.6095	-1.3165	
0.4957	0.1403	-1.4610	-0.2708	0.7125
	2.9528	-2.9997	-426.8665	
73.5600	-429.6798	-0.7996	-1.9301	
0.2834	0.0970	-1.5078	-0.0617	0.7276
	2.9356	-2.9777	-426.8576	
73.5800	-427.4948	0.5141	-0.7461	
0.1553	0.0057	-1.5383	0.1472	0.7424
	2.9181	-2.9558	-426.8487	
73.6000	-426.4000	0.7968	0.5253	
0.1758	-0.1281	-1.5526	0.3556	0.7570
	2.9005	-2.9338	-426.8397	
73.6200	-426.4000	-0.0501	0.7475	
0.3353	-0.2917	-1.5510	0.5635	0.7714
	2.8826	-2.9117	-426.8308	
73.6400	-426.4000	-0.8056	0.2966	
0.5182	-0.4657	-1.5341	0.7706	0.7854
	2.8645	-2.8897	-426.8219	
73.6600	-426.3250	-0.0133	0.0570	
0.5771	-0.6282	-1.5027	0.9768	0.7991
	2.8461	-2.8677	-426.8130	
73.6800	-425.9509	-0.1487	0.2606	
0.4084	-0.7583	-1.4579	1.1819	0.8125
	2.8276	-2.8456	-426.8040	
73.7000	-425.7003	0.4248	0.5329	-
0.0081	-0.8364	-1.4008	1.3858	0.8256
	2.8088	-2.8235	-426.7951	
73.7200	-426.7017	0.3036	0.2637	-
0.5895	-0.8489	-1.3327	1.5882	0.8383
	2.7899	-2.8014	-426.7862	
73.7400	-428.3891	-0.6212	-0.2624	-
1.1960	-0.7873	-1.2552	1.7891	0.8506
	2.7707	-2.7793	-426.7773	
73.7600	-428.5748	-0.8181	-0.3177	-
1.6630	-0.6486	-1.1699	1.9882	0.8625
	2.7513	-2.7571	-426.7684	
73.7800	-426.9167	0.6724	-0.0544	-
1.8320	-0.4350	-1.0783	2.1853	0.8740
	2.7317	-2.7350	-426.7594	
73.8000	-425.3965	0.7592	-0.1248	-
1.6160	-0.1567	-0.9817	2.3803	0.8850
	2.7119	-2.7128	-426.7505	
73.8200	-425.1517	0.6014	-0.8923	-
1.0076	0.1663	-0.8812	2.5730	0.8956
	2.6920	-2.6906	-426.7416	
73.8400	-425.0509	-0.2411	-1.6593	-
0.0955	0.5076	-0.7780	2.7631	0.9056
	2.6718	-2.6684	-426.7327	
73.8600	-424.4431	-1.9718	-0.9995	
0.9249	0.8397	-0.6729	2.9505	0.9152
	2.6515	-2.6462	-426.7238	

73.8800 -419.0120 -0.2897 1.1707
1.8042 1.1359 -0.5670 3.1349 0.9242
2.6309 -2.6240 -426.7149
73.9000 -414.3032 2.0393 2.7693
2.2711 1.3707 -0.4612 3.3161 0.9327
2.6102 -2.6017 -426.7060
73.9200 -416.4625 0.4652 1.9895
2.1421 1.5241 -0.3564 3.4939 0.9407
2.5893 -2.5795 -426.6971
73.9400 -420.7201 -1.3769 -0.2420
1.4923 1.5885 -0.2531 3.6680 0.9481
2.5683 -2.5572 -426.6882
73.9600 -421.7378 -0.8193 -1.6414
0.5491 1.5673 -0.1517 3.8381 0.9548
2.5471 -2.5349 -426.6793
73.9800 -422.0725 0.6709 -1.5017 -
0.4293 1.4690 -0.0525 4.0040 0.9609
2.5257 -2.5127 -426.6704
74.0000 -422.2394 0.7855 -1.0149 -
1.1982 1.3038 0.0446 4.1655 0.9664
2.5041 -2.4904 -426.6615
74.0200 -422.3493 0.0096 -0.8507 -
1.6026 1.0830 0.1394 4.3222 0.9713
2.4824 -2.4681 -426.6526
74.0400 -422.3996 -1.0093 -0.3924 -
1.6372 0.8175 0.2320 4.4740 0.9754
2.4605 -2.4457 -426.6437
74.0600 -420.9949 -0.3824 0.6909 -
1.3766 0.5180 0.3226 4.6205 0.9789
2.4385 -2.4234 -426.6348
74.0800 -418.4843 1.0385 1.5068 -
0.9149 0.1951 0.4118 4.7614 0.9817
2.4163 -2.4011 -426.6259
74.1000 -418.6729 0.5960 0.8733 -
0.3309 -0.1412 0.5003 4.8966 0.9837
2.3940 -2.3788 -426.6170
74.1200 -421.9103 -0.5082 -0.7426
0.2899 -0.4795 0.5886 5.0256 0.9849
2.3715 -2.3564 -426.6081
74.1400 -423.0953 -1.7506 -1.4146
0.8441 -0.8078 0.6775 5.1482 0.9854
2.3489 -2.3341 -426.5992
74.1600 -419.4756 0.1791 -0.2737
1.2258 -1.1146 0.7675 5.2641 0.9851
2.3262 -2.3117 -426.5904
74.1800 -416.1728 1.6854 1.4400
1.3390 -1.3873 0.8592 5.3732 0.9839
2.3033 -2.2894 -426.5815
74.2000 -416.8311 -0.2559 1.9828
1.1298 -1.6129 0.9525 5.4750 0.9820
2.2803 -2.2670 -426.5726

74.2200 -418.6905 0.9753 0.8919
0.6473 -1.7807 1.0473 5.5694 0.9792
2.2572 -2.2446 -426.5637
74.2400 -420.8414 0.7761 -0.8653
0.0301 -1.8831 1.1436 5.6561 0.9755
2.2339 -2.2223 -426.5548
74.2600 -423.4944 -0.8042 -1.9636 -
0.5584 -1.9143 1.2414 5.7348 0.9709
2.2106 -2.1999 -426.5459
74.2800 -424.0420 -1.0855 -1.6166 -
0.9889 -1.8689 1.3400 5.8053 0.9654
2.1871 -2.1775 -426.5371
74.3000 -421.0385 0.2308 -0.0178 -
1.2311 -1.7425 1.4385 5.8675 0.9590
2.1635 -2.1552 -426.5282
74.3200 -418.6170 0.9442 1.4929 -
1.3446 -1.5317 1.5357 5.9211 0.9516
2.1398 -2.1328 -426.5193
74.3400 -419.0850 -0.1231 1.6305 -
1.3757 -1.2362 1.6305 5.9659 0.9433
2.1160 -2.1104 -426.5104
74.3600 -419.8862 0.0258 0.4833 -
1.3325 -0.8624 1.7215 6.0017 0.9339
2.0921 -2.0881 -426.5016
74.3800 -419.8982 -0.2634 -0.6476 -
1.2029 -0.4244 1.8071 6.0283 0.9236
2.0680 -2.0657 -426.4927
74.4000 -419.3849 0.6427 -1.0190 -
0.9486 0.0569 1.8857 6.0456 0.9123
2.0439 -2.0433 -426.4838
74.4200 -418.6608 -0.1091 -0.9554 -
0.5379 0.5569 1.9557 6.0534 0.9000
2.0198 -2.0210 -426.4750
74.4400 -417.4712 -0.3554 -0.5873
0.0058 1.0495 2.0152 6.0516 0.8866
1.9955 -1.9986 -426.4661
74.4600 -415.8371 -0.6361 0.3487
0.6044 1.5068 2.0624 6.0400 0.8723
1.9711 -1.9762 -426.4572
74.4800 -412.6875 -0.1474 1.2841
1.1532 1.9004 2.0954 6.0187 0.8569
1.9467 -1.9539 -426.4484
74.5000 -410.1456 1.9536 1.2142
1.5510 2.2025 2.1122 5.9875 0.8404
1.9221 -1.9315 -426.4395
74.5200 -412.7582 0.5947 -0.1137
1.7414 2.3894 2.1112 5.9465 0.8230
1.8975 -1.9092 -426.4307
74.5400 -416.7637 -1.8299 -1.4789
1.7266 2.4479 2.0908 5.8959 0.8046
1.8729 -1.8868 -426.4218

74.5600 −415.8090 −0.8872 −1.3561
1.5402 2.3769 2.0506 5.8356 0.7852
1.8481 −1.8645 −426.4129
74.5800 −413.7474 1.2001 0.1770
1.2096 2.1846 1.9912 5.7660 0.7648
1.8234 −1.8422 −426.4041
74.6000 −413.7696 −0.1375 1.4568
0.7542 1.8889 1.9140 5.6872 0.7435
1.7985 −1.8199 −426.3952
74.6200 −414.9647 0.0673 1.4628
0.1975 1.5129 1.8211 5.5995 0.7212
1.7736 −1.7976 −426.3864
74.6400 −417.1471 −0.1829 0.4398 −
0.4311 1.0820 1.7144 5.5032 0.6980
1.7486 −1.7753 −426.3775
74.6600 −421.2915 0.0978 −0.8341 −
1.0299 0.6216 1.5959 5.3985 0.6739
1.7236 −1.7530 −426.3687
74.6800 −422.6230 −0.5242 −1.5036 −
1.4589 0.1574 1.4678 5.2858 0.6489
1.6985 −1.7307 −426.3598
74.7000 −421.6996 0.2976 −1.0000 −
1.6185 −0.2853 1.3321 5.1653 0.6230
1.6734 −1.7084 −426.3510
74.7200 −420.9079 0.6574 0.1306 −
1.4835 −0.6842 1.1908 5.0375 0.5962
1.6483 −1.6861 −426.3422
74.7400 −420.7767 −0.1791 0.9392 −
1.1276 −1.0235 1.0460 4.9026 0.5686
1.6231 −1.6639 −426.3333
74.7600 −420.7071 0.4242 1.0831 −
0.6752 −1.2931 0.8999 4.7608 0.5402
1.5979 −1.6417 −426.3245
74.7800 −421.1266 0.4470 0.5031 −
0.2395 −1.4889 0.7547 4.6127 0.5109
1.5726 −1.6194 −426.3156
74.8000 −422.7749 −0.2420 −0.3438
0.1071 −1.6113 0.6123 4.4584 0.4809
1.5473 −1.5972 −426.3068
74.8200 −423.4516 −0.5229 −0.6093
0.3446 −1.6661 0.4748 4.2983 0.4501
1.5220 −1.5750 −426.2980
74.8400 −422.3432 0.2392 −0.1341
0.5130 −1.6649 0.3443 4.1327 0.4185
1.4967 −1.5528 −426.2891
74.8600 −421.2442 0.9664 0.2703
0.6743 −1.6224 0.2227 3.9620 0.3862
1.4714 −1.5307 −426.2803
74.8800 −422.3929 0.6455 −0.0815
0.8616 −1.5533 0.1115 3.7865 0.3531
1.4460 −1.5085 −426.2715

74.9000 -425.1106 -1.2455 -0.7093
1.0469 -1.4673 0.0122 3.6065 0.3194
1.4206 -1.4864 -426.2627
74.9200 -425.5625 -1.4752 -0.4124
1.1499 -1.3709 -0.0744 3.4223 0.2849
1.3953 -1.4643 -426.2538
74.9400 -422.6196 -0.3143 1.0231
1.0798 -1.2680 -0.1477 3.2344 0.2498
1.3699 -1.4422 -426.2450
74.9600 -420.3195 1.7797 1.9410
0.7652 -1.1610 -0.2078 3.0431 0.2140
1.3445 -1.4201 -426.2362
74.9800 -423.0861 0.8960 0.9946
0.2182 -1.0493 -0.2551 2.8487 0.1776
1.3192 -1.3980 -426.2274
75.0000 -428.1326 -1.5261 -0.8158 -
0.4280 -0.9284 -0.2902 2.6516 0.1405
1.2938 -1.3760 -426.2185
75.0200 -429.1481 -0.4824 -1.8419 -
0.9871 -0.7935 -0.3140 2.4521 0.1029
1.2685 -1.3539 -426.2097
75.0400 -428.0449 0.2220 -1.5623 -
1.2889 -0.6401 -0.3275 2.2507 0.0646
1.2431 -1.3319 -426.2009
75.0600 -426.7368 0.2258 -0.5800 -
1.2637 -0.4666 -0.3319 2.0478 0.0258
1.2178 -1.3099 -426.1921
75.0800 -425.6959 0.1815 0.4579 -
0.9551 -0.2776 -0.3284 1.8437 -0.0136
1.1925 -1.2880 -426.1833
75.1000 -424.7368 -0.0220 0.9207 -
0.4730 -0.0806 -0.3185 1.6387 -0.0535
1.1672 -1.2660 -426.1745
75.1200 -424.3613 -0.1431 0.6072
0.0375 0.1148 -0.3034 1.4335 -0.0939
1.1420 -1.2441 -426.1657
75.1400 -424.2527 0.5324 0.1649
0.4468 0.2985 -0.2849 1.2282 -0.1349
1.1168 -1.2222 -426.1569
75.1600 -424.2020 -0.5340 0.1462
0.6847 0.4612 -0.2643 1.0234 -0.1763
1.0916 -1.2003 -426.1481
75.1800 -424.3703 0.5507 0.3278
0.7435 0.5944 -0.2434 0.8195 -0.2182
1.0665 -1.1785 -426.1392
75.2000 -424.8716 -0.3371 0.1950
0.6759 0.6924 -0.2238 0.6168 -0.2606
1.0414 -1.1566 -426.1304
75.2200 -425.2913 -0.0380 -0.1657
0.5563 0.7526 -0.2073 0.4159 -0.3034
1.0163 -1.1348 -426.1216

75.2400 -425.6545 0.0613 -0.3869
0.4417 0.7782 -0.1954 0.2171 -0.3466
0.9913 -1.1131 -426.1128
75.2600 -425.8872 0.0175 -0.3868
0.3438 0.7763 -0.1898 0.0208 -0.3902
0.9663 -1.0913 -426.1041
75.2800 -425.7769 0.0219 -0.1024
0.2329 0.7548 -0.1918 -0.1726 -0.4342
0.9414 -1.0696 -426.0953
75.3000 -425.4414 0.1863 0.4333
0.0646 0.7227 -0.2029 -0.3628 -0.4786
0.9166 -1.0479 -426.0865
75.3200 -425.3610 0.4246 0.7688 -
0.1709 0.6899 -0.2245 -0.5493 -0.5233
0.8918 -1.0262 -426.0777
75.3400 -426.9393 -0.2611 0.4774 -
0.4343 0.6671 -0.2579 -0.7320 -0.5683
0.8671 -1.0046 -426.0689
75.3600 -428.8216 -0.2318 -0.1602 -
0.6630 0.6633 -0.3040 -0.9104 -0.6137
0.8425 -0.9829 -426.0601
75.3800 -429.2188 -0.2344 -0.6871 -
0.7766 0.6846 -0.3634 -1.0842 -0.6594
0.8179 -0.9614 -426.0513
75.4000 -429.3510 0.5039 -0.8623 -
0.7230 0.7317 -0.4359 -1.2532 -0.7053
0.7934 -0.9398 -426.0425
75.4200 -429.4000 -0.1714 -0.5908 -
0.5091 0.8006 -0.5207 -1.4171 -0.7516
0.7690 -0.9183 -426.0337
75.4400 -428.5941 -0.4246 0.1979 -
0.1999 0.8847 -0.6164 -1.5757 -0.7980
0.7447 -0.8968 -426.0250
75.4600 -427.1654 0.3958 0.9285
0.1095 0.9735 -0.7213 -1.7287 -0.8447
0.7204 -0.8753 -426.0162
75.4800 -426.8537 0.3850 0.8383
0.3350 1.0531 -0.8336 -1.8760 -0.8917
0.6963 -0.8539 -426.0074
75.5000 -428.8735 0.3234 -0.1841
0.4371 1.1081 -0.9511 -2.0172 -0.9388
0.6722 -0.8325 -425.9986
75.5200 -430.9807 -1.3969 -0.8544
0.4123 1.1269 -1.0714 -2.1523 -0.9861
0.6482 -0.8111 -425.9899
75.5400 -430.8318 -0.4960 -0.2134
0.2816 1.0996 -1.1924 -2.2809 -1.0335
0.6244 -0.7898 -425.9811
75.5600 -429.5040 -0.3360 0.9319
0.1035 1.0182 -1.3117 -2.4030 -1.0811
0.6006 -0.7685 -425.9723

75.5800 -428.6072 0.5025 1.1311 -
0.0295 0.8766 -1.4270 -2.5183 -1.1289
0.5769 -0.7472 -425.9635
75.6000 -430.0415 1.1511 0.0174 -
0.0481 0.6746 -1.5363 -2.6267 -1.1767
0.5534 -0.7260 -425.9548
75.6200 -432.8959 -0.4855 -1.4424
0.0699 0.4182 -1.6374 -2.7279 -1.2247
0.5299 -0.7048 -425.9460
75.6400 -433.9399 -0.5841 -1.7920
0.3181 0.1181 -1.7283 -2.8219 -1.2727
0.5066 -0.6837 -425.9373
75.6600 -432.5431 -0.2632 -0.6635
0.6184 -0.2101 -1.8068 -2.9084 -1.3208
0.4834 -0.6625 -425.9285
75.6800 -430.7155 0.3188 1.0311
0.8422 -0.5482 -1.8710 -2.9873 -1.3689
0.4603 -0.6415 -425.9197
75.7000 -430.3638 0.5203 1.9247
0.8424 -0.8776 -1.9190 -3.0584 -1.4171
0.4373 -0.6204 -425.9110
75.7200 -432.2157 -0.3198 1.4722
0.5173 -1.1787 -1.9488 -3.1216 -1.4652
0.4145 -0.5994 -425.9022
75.7400 -434.5435 -0.5633 0.2728 -
0.0974 -1.4308 -1.9586 -3.1768 -1.5134
0.3918 -0.5784 -425.8935
75.7600 -435.7745 0.5740 -0.6221 -
0.8508 -1.6150 -1.9466 -3.2240 -1.5615
0.3692 -0.5575 -425.8847
75.7800 -436.6941 -0.1387 -1.0513 -
1.5241 -1.7139 -1.9114 -3.2633 -1.6096
0.3468 -0.5366 -425.8760
75.8000 -437.2444 0.0512 -1.1908 -
1.9004 -1.7120 -1.8525 -3.2946 -1.6576
0.3245 -0.5158 -425.8672
75.8200 -437.0891 -0.1198 -1.0254 -
1.8667 -1.6012 -1.7708 -3.3182 -1.7056
0.3024 -0.4950 -425.8585
75.8400 -436.0677 0.1210 -0.5900 -
1.4451 -1.3904 -1.6678 -3.3342 -1.7535
0.2804 -0.4742 -425.8497
75.8600 -434.7134 0.0360 0.0316 -
0.7669 -1.0994 -1.5460 -3.3428 -1.8013
0.2586 -0.4535 -425.8410
75.8800 -433.3290 -0.6902 0.7271 -
0.0251 -0.7514 -1.4083 -3.3443 -1.8489
0.2369 -0.4328 -425.8322
75.9000 -431.5380 -0.0190 1.2853
0.5923 -0.3711 -1.2575 -3.3389 -1.8965
0.2153 -0.4122 -425.8235

75.9200	-430.2992	0.4261	1.2808	
0.9823	0.0159	-1.0967	-3.3267	-1.9438
	0.1940	-0.3916	-425.8148	
75.9400	-430.4083	-0.0201	0.3928	
1.1664	0.3826	-0.9289	-3.3081	-1.9910
	0.1728	-0.3710	-425.8060	
75.9600	-431.1131	0.2092	-0.7906	
1.2541	0.7037	-0.7570	-3.2833	-2.0381
	0.1517	-0.3505	-425.7973	
75.9800	-431.5943	-0.3713	-1.3989	
1.3574	0.9568	-0.5841	-3.2525	-2.0849
	0.1309	-0.3301	-425.7886	
76.0000	-430.7515	0.0882	-1.0907	
1.4965	1.1285	-0.4130	-3.2159	-2.1314
	0.1102	-0.3097	-425.7798	
76.0200	-428.9042	-0.2721	-0.1741	
1.6078	1.2179	-0.2461	-3.1738	-2.1778
	0.0897	-0.2893	-425.7711	
76.0400	-427.8712	0.1769	0.8174	
1.5780	1.2318	-0.0857	-3.1264	-2.2238
	0.0693	-0.2690	-425.7624	
76.0600	-427.6479	-0.2734	1.4712	
1.2759	1.1829	0.0659	-3.0739	-2.2696
	0.0492	-0.2487	-425.7537	
76.0800	-427.5198	0.1995	1.6895	
0.6313	1.0861	0.2069	-3.0166	-2.3151
	0.0292	-0.2285	-425.7449	
76.1000	-428.0713	1.1860	1.0001	-
0.2879	0.9547	0.3357	-2.9547	-2.3602
	0.0094	-0.2083	-425.7362	
76.1200	-431.6879	-0.2037	-0.4739	-
1.2749	0.8003	0.4508	-2.8883	-2.4050
	-0.0102	-0.1882	-425.7275	
76.1400	-434.6888	-0.7355	-1.6498	-
2.0794	0.6337	0.5513	-2.8178	-2.4495
	-0.0296	-0.1681	-425.7188	
76.1600	-434.2113	-0.6340	-1.5279	-
2.4887	0.4653	0.6361	-2.7432	-2.4935
	-0.0487	-0.1481	-425.7101	
76.1800	-432.5906	0.1843	-0.3626	-
2.3933	0.3034	0.7048	-2.6649	-2.5371
	-0.0677	-0.1281	-425.7014	
76.2000	-431.2636	0.5557	0.6865	-
1.8175	0.1534	0.7567	-2.5830	-2.5803
	-0.0865	-0.1081	-425.6926	
76.2200	-430.8403	0.3002	0.5742	-
0.8831	0.0176	0.7917	-2.4977	-2.6230
	-0.1051	-0.0883	-425.6839	
76.2400	-430.5736	0.0341	-0.4459	
0.2359	-0.1038	0.8104	-2.4094	-2.6653
	-0.1235	-0.0684	-425.6752	

76.2600 -430.1345 -0.2425 -1.1113
1.3341 -0.2104 0.8133 -2.3181 -2.7070
-0.1417 -0.0487 -425.6665
76.2800 -428.5846 0.0841 -0.6625
2.1802 -0.3011 0.8011 -2.2241 -2.7483
-0.1596 -0.0289 -425.6578
76.3000 -426.7553 0.4146 0.5825
2.5992 -0.3744 0.7749 -2.1278 -2.7890
-0.1773 -0.0093 -425.6491
76.3200 -426.3266 -0.2730 1.7560
2.4744 -0.4289 0.7357 -2.0293 -2.8291
-0.1948 0.0104 -425.6404
76.3400 -426.6302 -0.4552 2.2922
1.8039 -0.4616 0.6844 -1.9289 -2.8687
-0.2121 0.0299 -425.6317
76.3600 -427.2159 0.4392 1.7315
0.7244 -0.4681 0.6223 -1.8270 -2.9077
-0.2292 0.0494 -425.6230
76.3800 -429.7303 1.0627 -0.2547 -
0.5077 -0.4432 0.5509 -1.7238 -2.9461
-0.2460 0.0689 -425.6143
76.4000 -435.3505 -0.5944 -2.6947 -
1.5906 -0.3824 0.4715 -1.6197 -2.9839
-0.2626 0.0883 -425.6057
76.4200 -438.3970 -2.3229 -3.4182 -
2.2439 -0.2833 0.3859 -1.5150 -3.0210
-0.2790 0.1076 -425.5970
76.4400 -434.7865 -1.5677 -1.4670 -
2.3131 -0.1475 0.2957 -1.4099 -3.0575
-0.2951 0.1269 -425.5883
76.4600 -428.5212 1.9341 1.6860 -
1.8825 0.0138 0.2026 -1.3049 -3.0933
-0.3110 0.1462 -425.5796
76.4800 -425.7302 1.3008 3.2000 -
1.1319 0.1867 0.1085 -1.2001 -3.1285
-0.3266 0.1653 -425.5709
76.5000 -427.9320 0.7486 1.6425 -
0.2550 0.3567 0.0150 -1.0959 -3.1630
-0.3420 0.1845 -425.5622
76.5200 -430.9940 -0.9520 -1.1665
0.5848 0.5062 -0.0763 -0.9925 -3.1967
-0.3571 0.2035 -425.5535
76.5400 -431.3619 -0.8883 -2.4306
1.2648 0.6146 -0.1638 -0.8902 -3.2298
-0.3720 0.2225 -425.5449
76.5600 -428.9249 0.0859 -1.5236
1.7100 0.6611 -0.2460 -0.7893 -3.2621
-0.3867 0.2415 -425.5362
76.5800 -426.6190 0.5066 0.1324
1.8991 0.6289 -0.3216 -0.6902 -3.2937
-0.4010 0.2604 -425.5275

76.6000 -426.5524 0.0316 1.2313
1.8048 0.5119 -0.3892 -0.5931 -3.3245
-0.4151 0.2792 -425.5189
76.6200 -427.2221 -0.3633 1.5625
1.3830 0.3196 -0.4475 -0.4984 -3.3546
-0.4290 0.2979 -425.5102
76.6400 -428.1557 0.4688 1.0825
0.6921 0.0709 -0.4951 -0.4063 -3.3838
-0.4426 0.3167 -425.5015
76.6600 -430.1618 0.1784 -0.0299 -
0.1141 -0.2118 -0.5305 -0.3173 -3.4123
-0.4559 0.3353 -425.4929
76.6800 -432.4473 -0.2393 -1.0410 -
0.8279 -0.5036 -0.5526 -0.2315 -3.4399
-0.4689 0.3539 -425.4842
76.7000 -432.9550 -0.0447 -1.3159 -
1.2716 -0.7783 -0.5602 -0.1492 -3.4667
-0.4817 0.3724 -425.4755
76.7200 -432.5233 -0.3502 -1.0153 -
1.3621 -1.0112 -0.5521 -0.0706 -3.4926
-0.4942 0.3909 -425.4669
76.7400 -431.8841 0.2055 -0.3137 -
1.1627 -1.1844 -0.5275 0.0041 -3.5177
-0.5064 0.4093 -425.4582
76.7600 -431.2385 -0.0865 0.6590 -
0.8236 -1.2858 -0.4857 0.0747 -3.5418
-0.5183 0.4276 -425.4496
76.7800 -430.4027 0.0638 1.2330 -
0.5172 -1.3092 -0.4268 0.1413 -3.5651
-0.5300 0.4459 -425.4409
76.8000 -429.8352 -0.3446 1.1372 -
0.3612 -1.2559 -0.3512 0.2036 -3.5874
-0.5414 0.4641 -425.4323
76.8200 -430.0549 0.3636 0.5657 -
0.3466 -1.1356 -0.2599 0.2616 -3.6088
-0.5524 0.4823 -425.4236
76.8400 -430.8230 -0.3694 -0.2873 -
0.3849 -0.9643 -0.1542 0.3153 -3.6291
-0.5632 0.5003 -425.4150
76.8600 -431.2991 -0.2242 -1.1111 -
0.3575 -0.7604 -0.0357 0.3646 -3.6485
-0.5737 0.5184 -425.4063
76.8800 -430.2662 0.0776 -1.1814 -
0.1685 -0.5417 0.0940 0.4094 -3.6669
-0.5839 0.5363 -425.3977
76.9000 -428.2348 0.4132 -0.3789
0.1818 -0.3239 0.2330 0.4497 -3.6843
-0.5938 0.5542 -425.3890
76.9200 -427.0856 -0.1796 0.5186
0.6029 -0.1191 0.3791 0.4854 -3.7007
-0.6034 0.5720 -425.3804

76.9400 -426.6487 -0.2801 0.7017
0.9697 0.0641 0.5299 0.5165 -3.7160
-0.6126 0.5898 -425.3718
76.9600 -426.3714 -0.2508 0.2069
1.1831 0.2199 0.6829 0.5430 -3.7303
-0.6216 0.6075 -425.3631
76.9800 -426.2334 0.3511 -0.2778
1.1759 0.3463 0.8353 0.5648 -3.7435
-0.6303 0.6251 -425.3545
77.0000 -426.1394 0.2530 -0.2916
0.9230 0.4464 0.9843 0.5820 -3.7556
-0.6386 0.6427 -425.3459
77.0200 -426.1001 -0.2548 0.3030
0.4570 0.5258 1.1270 0.5943 -3.7667
-0.6467 0.6602 -425.3372
77.0400 -426.1960 -0.7957 1.0464 -
0.1302 0.5919 1.2605 0.6020 -3.7767
-0.6544 0.6776 -425.3286
77.0600 -426.4654 0.2627 1.1078 -
0.7058 0.6524 1.3818 0.6049 -3.7856
-0.6618 0.6950 -425.3200
77.0800 -427.0214 0.9309 -0.0519 -
1.1353 0.7142 1.4883 0.6030 -3.7934
-0.6689 0.7123 -425.3114
77.1000 -428.9407 0.2741 -1.6796 -
1.3236 0.7843 1.5775 0.5963 -3.8002
-0.6757 0.7295 -425.3027
77.1200 -430.5942 -1.2705 -2.3200 -
1.2260 0.8659 1.6474 0.5848 -3.8059
-0.6821 0.7467 -425.2941
77.1400 -428.8965 -1.3064 -0.9469 -
0.8795 0.9579 1.6962 0.5687 -3.8106
-0.6882 0.7637 -425.2855
77.1600 -423.6852 0.6573 1.6061 -
0.4153 1.0551 1.7228 0.5478 -3.8142
-0.6940 0.7808 -425.2769
77.1800 -420.9126 1.9555 2.8361
0.0223 1.1505 1.7263 0.5224 -3.8167
-0.6995 0.7977 -425.2683
77.2000 -423.7629 -0.0774 1.4307
0.3374 1.2352 1.7065 0.4925 -3.8183
-0.7046 0.8146 -425.2597
77.2200 -427.6930 -0.8675 -1.0578
0.5447 1.2991 1.6632 0.4585 -3.8188
-0.7094 0.8314 -425.2511
77.2400 -427.8976 -1.0457 -2.4468
0.7036 1.3324 1.5965 0.4205 -3.8184
-0.7139 0.8482 -425.2425
77.2600 -426.0553 0.4318 -1.9505
0.8641 1.3270 1.5073 0.3788 -3.8170
-0.7181 0.8649 -425.2339

77.2800 -424.6190 0.9069 -0.5412
1.0335 1.2763 1.3968 0.3337 -3.8146
-0.7219 0.8815 -425.2253
77.3000 -424.2586 -0.4947 0.6779
1.1502 1.1760 1.2669 0.2856 -3.8113
-0.7254 0.8980 -425.2167
77.3200 -424.0390 -0.1241 1.4783
1.0936 1.0261 1.1201 0.2347 -3.8071
-0.7286 0.9145 -425.2081
77.3400 -424.1849 0.1325 1.8368
0.7709 0.8310 0.9590 0.1814 -3.8020
-0.7314 0.9309 -425.1995
77.3600 -425.7190 0.3567 1.5118
0.1801 0.6006 0.7864 0.1259 -3.7959
-0.7339 0.9472 -425.1909
77.3800 -427.7039 -0.2018 0.4671 -
0.5508 0.3470 0.6052 0.0687 -3.7890
-0.7361 0.9634 -425.1823
77.4000 -429.7654 0.0262 -0.7811 -
1.1939 0.0830 0.4181 0.0101 -3.7813
-0.7380 0.9796 -425.1737
77.4200 -431.9024 0.4116 -1.6950 -
1.5111 -0.1789 0.2279 -0.0497 -3.7727
-0.7395 0.9957 -425.1651
77.4400 -432.5289 -0.7713 -1.7460 -
1.3746 -0.4281 0.0376 -0.1104 -3.7633
-0.7408 1.0118 -425.1565
77.4600 -431.0474 -0.2104 -0.7038 -
0.8190 -0.6553 -0.1501 -0.1715 -3.7530
-0.7417 1.0277 -425.1480
77.4800 -429.1707 0.6564 0.6465 -
0.0401 -0.8555 -0.3323 -0.2328 -3.7420
-0.7422 1.0436 -425.1394
77.5000 -428.7044 -0.0640 1.2318
0.6938 -1.0251 -0.5061 -0.2939 -3.7302
-0.7425 1.0595 -425.1308
77.5200 -428.7552 -0.1660 1.0639
1.1454 -1.1612 -0.6691 -0.3546 -3.7177
-0.7424 1.0752 -425.1222
77.5400 -428.8576 0.3449 0.6691
1.1959 -1.2616 -0.8189 -0.4144 -3.7045
-0.7420 1.0909 -425.1136
77.5600 -429.4897 0.4062 0.0792
0.8912 -1.3262 -0.9536 -0.4731 -3.6905
-0.7413 1.1065 -425.1051
77.5800 -431.2559 -0.2590 -0.5508
0.3575 -1.3551 -1.0716 -0.5304 -3.6758
-0.7403 1.1220 -425.0965
77.6000 -432.3939 -0.3874 -0.6754 -
0.2522 -1.3480 -1.1719 -0.5859 -3.6604
-0.7389 1.1375 -425.0879

77.6200 -432.4553 -0.1334 -0.2985 -
0.7645 -1.3046 -1.2539 -0.6393 -3.6444
-0.7373 1.1529 -425.0794
77.6400 -432.4900 0.5201 0.0587 -
1.0517 -1.2264 -1.3177 -0.6902 -3.6277
-0.7353 1.1682 -425.0708
77.6600 -432.4961 0.2953 0.0365 -
1.0475 -1.1159 -1.3637 -0.7386 -3.6104
-0.7331 1.1835 -425.0623
77.6800 -432.1952 -0.4048 -0.2592 -
0.7537 -0.9787 -1.3926 -0.7841 -3.5924
-0.7305 1.1986 -425.0537
77.7000 -431.5910 -0.0134 -0.4004 -
0.2706 -0.8211 -1.4053 -0.8266 -3.5739
-0.7276 1.2137 -425.0451
77.7200 -430.8930 0.0286 -0.1369
0.2713 -0.6487 -1.4030 -0.8658 -3.5547
-0.7244 1.2288 -425.0366
77.7400 -429.7366 0.0637 0.2765
0.7350 -0.4670 -1.3867 -0.9015 -3.5350
-0.7209 1.2437 -425.0280
77.7600 -428.6897 0.0077 0.5514
0.9902 -0.2810 -1.3578 -0.9338 -3.5148
-0.7171 1.2586 -425.0195
77.7800 -428.6524 0.6392 0.4699
0.9673 -0.0951 -1.3177 -0.9624 -3.4940
-0.7130 1.2734 -425.0109
77.8000 -429.5637 -0.2710 0.0640
0.6861 0.0874 -1.2677 -0.9872 -3.4727
-0.7087 1.2881 -425.0024
77.8200 -430.3529 -0.7453 -0.3420
0.2351 0.2649 -1.2092 -1.0083 -3.4509
-0.7040 1.3028 -424.9939
77.8400 -430.1299 -0.1906 -0.3647 -
0.2506 0.4361 -1.1440 -1.0256 -3.4286
-0.6990 1.3174 -424.9853
77.8600 -429.3545 0.4675 -0.0420 -
0.6333 0.6009 -1.0735 -1.0390 -3.4058
-0.6937 1.3319 -424.9768
77.8800 -428.9000 0.2007 0.2657 -
0.8254 0.7574 -0.9991 -1.0487 -3.3827
-0.6882 1.3463 -424.9682
77.9000 -429.2035 0.2903 0.0065 -
0.8023 0.9027 -0.9220 -1.0546 -3.3591
-0.6823 1.3607 -424.9597
77.9200 -429.7242 0.0359 -0.5076 -
0.5735 1.0318 -0.8431 -1.0569 -3.3350
-0.6762 1.3750 -424.9512
77.9400 -429.7892 -0.1569 -0.5697 -
0.2018 1.1378 -0.7635 -1.0555 -3.3107
-0.6698 1.3892 -424.9427

77.9600 -428.1171 -0.5233 0.0916
0.1813 1.2143 -0.6840 -1.0507 -3.2859
-0.6631 1.4034 -424.9341
77.9800 -426.2141 0.3869 0.9059
0.4446 1.2574 -0.6054 -1.0425 -3.2608
-0.6561 1.4174 -424.9256
78.0000 -426.0074 0.2213 1.0394
0.4926 1.2653 -0.5282 -1.0311 -3.2354
-0.6489 1.4314 -424.9171
78.0200 -426.6332 0.2072 0.5476
0.3373 1.2361 -0.4527 -1.0165 -3.2097
-0.6413 1.4454 -424.9086
78.0400 -427.4515 -0.2524 -0.1786
0.0982 1.1662 -0.3788 -0.9989 -3.1836
-0.6335 1.4592 -424.9000
78.0600 -428.5375 0.0518 -1.0240 -
0.0850 1.0519 -0.3065 -0.9785 -3.1574
-0.6254 1.4730 -424.8915
78.0800 -429.7365 0.1563 -1.7490 -
0.0881 0.8921 -0.2358 -0.9555 -3.1309
-0.6171 1.4867 -424.8830
78.1000 -429.9345 -0.9635 -1.6454
0.1390 0.6887 -0.1666 -0.9300 -3.1041
-0.6085 1.5003 -424.8745
78.1200 -427.5134 0.0481 -0.3850
0.5109 0.4481 -0.0989 -0.9022 -3.0771
-0.5996 1.5139 -424.8660
78.1400 -424.8078 1.0408 1.3351
0.8463 0.1801 -0.0328 -0.8724 -3.0500
-0.5905 1.5273 -424.8575
78.1600 -424.4199 0.0523 2.6514
0.9496 -0.1050 0.0320 -0.8406 -3.0226
-0.5810 1.5408 -424.8490
78.1800 -424.5519 -0.6911 3.1086
0.6672 -0.3947 0.0956 -0.8072 -2.9951
-0.5714 1.5541 -424.8405
78.2000 -424.7973 0.4063 2.3654 -
0.0061 -0.6720 0.1581 -0.7722 -2.9674
-0.5614 1.5673 -424.8320
78.2200 -428.1422 0.9497 0.2069 -
0.8828 -0.9208 0.2196 -0.7360 -2.9396
-0.5513 1.5805 -424.8235
78.2400 -433.4098 -0.8038 -2.5433 -
1.6877 -1.1261 0.2805 -0.6986 -2.9117
-0.5408 1.5936 -424.8150
78.2600 -434.6711 -0.4814 -4.0975 -
2.1116 -1.2738 0.3408 -0.6603 -2.8836
-0.5301 1.6067 -424.8065
78.2800 -433.3542 0.8332 -3.6110 -
1.9541 -1.3541 0.4007 -0.6213 -2.8555
-0.5192 1.6196 -424.7980

78.3000 -431.2569 0.1977 -1.8238 -
1.2772 -1.3677 0.4604 -0.5818 -2.8272
-0.5080 1.6325 -424.7895
78.3200 -428.6953 -0.9170 0.2296 -
0.3067 -1.3253 0.5199 -0.5418 -2.7989
-0.4965 1.6454 -424.7810
78.3400 -424.6726 0.2276 1.9372
0.6996 -1.2405 0.5791 -0.5017 -2.7705
-0.4848 1.6581 -424.7726
78.3600 -421.9145 1.3452 2.8238
1.5039 -1.1271 0.6378 -0.4615 -2.7421
-0.4729 1.6708 -424.7641
78.3800 -422.1329 -0.1518 2.5758
1.9341 -0.9985 0.6959 -0.4213 -2.7137
-0.4607 1.6834 -424.7556
78.4000 -423.1617 0.0075 1.5787
1.9375 -0.8648 0.7529 -0.3813 -2.6852
-0.4483 1.6959 -424.7471
78.4200 -424.2953 -0.4617 0.3788
1.5716 -0.7299 0.8080 -0.3416 -2.6568
-0.4356 1.7083 -424.7387
78.4400 -425.8350 0.2539 -0.5877
0.9734 -0.5943 0.8603 -0.3022 -2.6283
-0.4228 1.7207 -424.7302
78.4600 -427.0113 -0.2220 -1.1107
0.3075 -0.4572 0.9088 -0.2632 -2.5999
-0.4096 1.7330 -424.7217
78.4800 -427.1474 0.2669 -1.2138 -
0.2624 -0.3159 0.9523 -0.2248 -2.5714
-0.3963 1.7452 -424.7132
78.5000 -427.1882 0.2463 -0.9927 -
0.6527 -0.1670 0.9897 -0.1870 -2.5431
-0.3827 1.7574 -424.7048
78.5200 -427.1710 -0.7788 -0.3160 -
0.8494 -0.0054 1.0197 -0.1499 -2.5148
-0.3689 1.7695 -424.6963
78.5400 -425.2976 -0.5305 0.7391 -
0.8848 0.1749 1.0413 -0.1135 -2.4865
-0.3548 1.7815 -424.6879
78.5600 -422.8308 1.3144 1.3405 -
0.8356 0.3790 1.0534 -0.0780 -2.4584
-0.3406 1.7934 -424.6794
78.5800 -422.6205 0.5768 0.9730 -
0.7613 0.6055 1.0553 -0.0433 -2.4303
-0.3261 1.8052 -424.6709
78.6000 -423.7058 0.0518 -0.0100 -
0.6572 0.8450 1.0461 -0.0096 -2.4023
-0.3113 1.8170 -424.6625
78.6200 -424.4000 -0.2173 -0.8740 -
0.4760 1.0833 1.0256 0.0231 -2.3745
-0.2964 1.8287 -424.6540

78.6400 -424.1207 0.0283 -1.0955 -
0.1572 1.3036 0.9936 0.0548 -2.3467
-0.2813 1.8404 -424.6456
78.6600 -423.4736 0.1217 -0.7733
0.3107 1.4879 0.9502 0.0853 -2.3191
-0.2659 1.8519 -424.6372
78.6800 -422.5239 -0.0832 -0.1730
0.8375 1.6217 0.8954 0.1148 -2.2917
-0.2503 1.8634 -424.6287
78.7000 -420.8181 -0.1023 0.5837
1.2658 1.6930 0.8296 0.1431 -2.2644
-0.2345 1.8748 -424.6203
78.7200 -419.9003 0.1265 1.2436
1.4387 1.6923 0.7533 0.1704 -2.2373
-0.2185 1.8862 -424.6118
78.7400 -420.1349 -0.3543 1.6194
1.2553 1.6139 0.6674 0.1965 -2.2104
-0.2023 1.8975 -424.6034
78.7600 -420.7205 0.8206 1.2415
0.7418 1.4597 0.5731 0.2217 -2.1837
-0.1859 1.9087 -424.5950
78.7800 -422.4817 0.7447 -0.1889
0.0637 1.2392 0.4718 0.2459 -2.1572
-0.1693 1.9198 -424.5865
78.8000 -426.5791 -0.8637 -1.9364 -
0.5339 0.9647 0.3653 0.2692 -2.1310
-0.1525 1.9308 -424.5781
78.8200 -428.3342 -1.0528 -2.4316 -
0.8254 0.6494 0.2554 0.2916 -2.1050
-0.1354 1.9418 -424.5697
78.8400 -426.0331 0.4903 -1.1793 -
0.7178 0.3074 0.1441 0.3132 -2.0792
-0.1182 1.9527 -424.5612
78.8600 -423.2672 1.1004 0.4835 -
0.3013 -0.0480 0.0333 0.3341 -2.0537
-0.1008 1.9635 -424.5528
78.8800 -423.0790 -0.2341 1.2524
0.2412 -0.4034 -0.0752 0.3542 -2.0285
-0.0832 1.9743 -424.5444
78.9000 -423.8093 -0.0817 1.2658
0.6684 -0.7446 -0.1794 0.3738 -2.0035
-0.0654 1.9850 -424.5360
78.9200 -424.3069 -0.6741 1.3498
0.7652 -1.0587 -0.2773 0.3928 -1.9789
-0.0474 1.9956 -424.5276
78.9400 -424.3854 -0.2646 1.7342
0.4603 -1.3341 -0.3673 0.4114 -1.9545
-0.0292 2.0061 -424.5192
78.9600 -424.4437 0.7667 1.5680 -
0.1249 -1.5600 -0.4473 0.4295 -1.9305
-0.0108 2.0166 -424.5108

78.9800	-425.5075	1.4886	-0.0273	-
0.7572	-1.7260	-0.5158	0.4473	-1.9068
	0.0077	2.0270	-424.5023	
79.0000	-430.1623	-0.4152	-2.3822	-
1.2134	-1.8224	-0.5714	0.4648	-1.8834
	0.0264	2.0373	-424.4939	
79.0200	-433.4690	-2.1360	-3.3658	-
1.3117	-1.8427	-0.6130	0.4821	-1.8604
	0.0454	2.0475	-424.4855	
79.0400	-430.1650	-1.2349	-1.7240	-
0.9803	-1.7844	-0.6405	0.4993	-1.8377
	0.0645	2.0577	-424.4771	
79.0600	-423.4078	1.6570	1.0785	-
0.4065	-1.6517	-0.6539	0.5164	-1.8154
	0.0837	2.0678	-424.4687	
79.0800	-420.6873	2.8966	2.5802	
0.1603	-1.4532	-0.6541	0.5334	-1.7934
	0.1032	2.0778	-424.4603	
79.1000	-423.4088	-0.2910	1.8444	
0.5163	-1.1990	-0.6419	0.5503	-1.7718
	0.1228	2.0878	-424.4519	
79.1200	-426.5553	-2.9953	0.3308	
0.5560	-0.8994	-0.6184	0.5671	-1.7506
	0.1426	2.0976	-424.4436	
79.1400	-426.0188	-1.3850	-0.1136	
0.2557	-0.5667	-0.5850	0.5838	-1.7297
	0.1626	2.1074	-424.4352	
79.1600	-422.9305	0.4142	0.4031	-
0.2497	-0.2157	-0.5428	0.6004	-1.7093
	0.1827	2.1172	-424.4268	
79.1800	-421.3260	2.9215	0.2190	-
0.7129	0.1358	-0.4929	0.6169	-1.6892
	0.2030	2.1268	-424.4184	
79.2000	-424.0797	0.5355	-1.2104	-
0.9213	0.4687	-0.4367	0.6333	-1.6694
	0.2235	2.1364	-424.4100	
79.2200	-427.6918	-1.6217	-2.4500	-
0.7732	0.7648	-0.3754	0.6495	-1.6501
	0.2441	2.1459	-424.4016	
79.2400	-426.7402	-1.9175	-1.8921	-
0.2666	1.0114	-0.3102	0.6654	-1.6311
	0.2649	2.1554	-424.3933	
79.2600	-420.7396	0.6465	0.0085	
0.4759	1.1995	-0.2426	0.6812	-1.6125
	0.2858	2.1648	-424.3849	
79.2800	-417.1004	1.5175	1.4546	
1.2490	1.3235	-0.1738	0.6966	-1.5943
	0.3069	2.1740	-424.3765	
79.3000	-418.1716	0.1177	1.4816	
1.8448	1.3792	-0.1051	0.7118	-1.5764
	0.3281	2.1833	-424.3682	

79.3200 -419.6454 -0.8244 0.6405
2.1226 1.3650 -0.0380 0.7268 -1.5589
0.3495 2.1924 -424.3598
79.3400 -419.6652 -0.0762 0.0735
2.0439 1.2818 0.0265 0.7413 -1.5418
0.3711 2.2015 -424.3514
79.3600 -419.2436 0.0935 0.0964
1.6689 1.1371 0.0872 0.7555 -1.5250
0.3927 2.2105 -424.3431
79.3800 -419.3433 0.4201 0.0628
1.1180 0.9426 0.1431 0.7693 -1.5085
0.4145 2.2194 -424.3347
79.4000 -421.9860 -0.1241 -0.5101
0.4951 0.7129 0.1934 0.7827 -1.4924
0.4365 2.2283 -424.3263
79.4200 -424.0867 -0.9105 -0.8387 -
0.1254 0.4640 0.2373 0.7956 -1.4766
0.4586 2.2371 -424.3180
79.4400 -422.8749 -0.4038 -0.0791 -
0.7102 0.2124 0.2741 0.8080 -1.4611
0.4808 2.2458 -424.3096
79.4600 -420.7403 1.5721 0.9196 -
1.2460 -0.0225 0.3033 0.8198 -1.4460
0.5032 2.2545 -424.3013
79.4800 -421.0877 1.9932 0.7412 -
1.7201 -0.2209 0.3245 0.8311 -1.4311
0.5256 2.2630 -424.2929
79.5000 -425.4766 -0.2780 -0.5935 -
2.1128 -0.3654 0.3371 0.8416 -1.4165
0.5482 2.2715 -424.2846
79.5200 -428.3993 -3.1314 -1.2761 -
2.3819 -0.4458 0.3414 0.8514 -1.4022
0.5710 2.2800 -424.2763
79.5400 -425.1705 -0.8610 -0.0105 -
2.4703 -0.4595 0.3374 0.8605 -1.3881
0.5938 2.2883 -424.2679
79.5600 -419.9564 1.8931 1.7425 -
2.3208 -0.4112 0.3259 0.8687 -1.3743
0.6168 2.2966 -424.2596
79.5800 -419.4753 2.3933 1.6353 -
1.8783 -0.3113 0.3076 0.8760 -1.3608
0.6398 2.3048 -424.2513
79.6000 -423.6662 -0.0564 -0.7241 -
1.1348 -0.1754 0.2832 0.8824 -1.3475
0.6630 2.3130 -424.2429
79.6200 -426.5951 -1.9852 -2.8924 -
0.1471 -0.0229 0.2538 0.8877 -1.3344
0.6863 2.3210 -424.2346
79.6400 -423.2251 -1.0761 -2.4681
0.9793 0.1274 0.2202 0.8921 -1.3215
0.7097 2.3290 -424.2263

79.6600	-417.4798	1.1237	0.1313	
2.0688	0.2578	0.1833	0.8954	-1.3088
	0.7331	2.3369	-424.2179	
79.6800	-415.6681	0.7423	2.5972	
2.9029	0.3511	0.1439	0.8975	-1.2963
	0.7567	2.3448	-424.2096	
79.7000	-415.0687	-0.7663	3.1830	
3.2658	0.3907	0.1030	0.8984	-1.2839
	0.7804	2.3526	-424.2013	
79.7200	-415.0755	1.1505	1.7423	
3.0586	0.3673	0.0615	0.8981	-1.2717
	0.8042	2.3603	-424.1930	
79.7400	-419.7528	-0.1114	-0.7504	
2.3940	0.2848	0.0204	0.8965	-1.2597
	0.8280	2.3679	-424.1847	
79.7600	-424.0449	-1.1879	-2.5909	
1.4679	0.1540	-0.0198	0.8936	-1.2478
	0.8519	2.3755	-424.1764	
79.7800	-423.2628	-0.7215	-2.3240	
0.4843	-0.0121	-0.0582	0.8894	-1.2361
	0.8759	2.3830	-424.1681	
79.8000	-421.7236	0.8685	-0.5327	-
0.3860	-0.1999	-0.0942	0.8839	-1.2244
	0.9000	2.3904	-424.1597	
79.8200	-421.5269	0.3280	0.9735	-
1.0667	-0.3923	-0.1271	0.8769	-1.2129
	0.9242	2.3978	-424.1514	
79.8400	-422.3463	-0.2248	1.3920	-
1.5743	-0.5696	-0.1563	0.8686	-1.2014
	0.9484	2.4050	-424.1431	
79.8600	-423.3039	-0.5163	0.9489	-
1.9351	-0.7118	-0.1812	0.8588	-1.1900
	0.9727	2.4122	-424.1348	
79.8800	-424.2898	0.4738	0.1455	-
2.1673	-0.8040	-0.2011	0.8476	-1.1787
	0.9971	2.4194	-424.1265	
79.9000	-425.1555	-0.0734	-0.6771	-
2.2491	-0.8370	-0.2154	0.8349	-1.1674
	1.0215	2.4264	-424.1183	
79.9200	-425.1865	0.1178	-0.9813	-
2.1312	-0.8072	-0.2240	0.8206	-1.1562
	1.0459	2.4334	-424.1100	
79.9400	-424.4561	-0.2614	-0.6610	-
1.7896	-0.7169	-0.2269	0.8049	-1.1449
	1.0705	2.4404	-424.1017	
79.9600	-423.4455	0.2442	-0.0041	-
1.2641	-0.5749	-0.2244	0.7875	-1.1337
	1.0950	2.4472	-424.0934	
79.9800	-421.8635	0.0033	0.6473	-
0.6511	-0.3940	-0.2165	0.7685	-1.1225
	1.1197	2.4540	-424.0851	

80.0000 -420.1417 -0.1161 0.9549 -
0.0532 -0.1881 -0.2037 0.7478 -1.1113
1.1443 2.4607 -424.0768
80.0200 -419.8384 0.3996 0.7459
0.4765 0.0273 -0.1862 0.7254 -1.1000
1.1690 2.4673 -424.0685
80.0400 -420.0630 -0.1459 0.0406
0.9219 0.2366 -0.1645 0.7012 -1.0887
1.1938 2.4739 -424.0603
80.0600 -420.2000 -0.4038 -0.9764
1.3042 0.4246 -0.1392 0.6753 -1.0772
1.2185 2.4804 -424.0520
80.0800 -420.1802 0.1746 -1.6025
1.6192 0.5776 -0.1109 0.6475 -1.0657
1.2433 2.4868 -424.0437
80.1000 -420.1256 -0.4001 -1.0308
1.8162 0.6851 -0.0799 0.6179 -1.0541
1.2681 2.4932 -424.0355
80.1200 -418.7319 -1.4247 0.7165
1.8017 0.7422 -0.0467 0.5865 -1.0424
1.2930 2.4995 -424.0272
80.1400 -414.7058 1.2198 2.2712
1.5007 0.7493 -0.0115 0.5533 -1.0305
1.3178 2.5057 -424.0189
80.1600 -414.1841 3.4431 1.8377
0.8971 0.7104 0.0255 0.5183 -1.0185
1.3427 2.5118 -424.0107
80.1800 -421.7205 -1.2907 -0.5843
0.1032 0.6321 0.0641 0.4815 -1.0062
1.3676 2.5179 -424.0024
80.2000 -426.4787 -3.3802 -2.1962 -
0.6776 0.5220 0.1041 0.4430 -0.9938
1.3925 2.5239 -423.9942
80.2200 -423.3462 -0.6835 -1.3662 -
1.2379 0.3876 0.1454 0.4027 -0.9812
1.4174 2.5298 -423.9859
80.2400 -419.0822 2.5590 0.2984 -
1.4534 0.2377 0.1875 0.3607 -0.9683
1.4422 2.5357 -423.9777
80.2600 -419.0303 1.8155 0.6688 -
1.2719 0.0818 0.2302 0.3170 -0.9552
1.4671 2.5414 -423.9694
80.2800 -421.7646 -0.0188 -0.4300 -
0.7555 -0.0728 0.2733 0.2716 -0.9418
1.4920 2.5472 -423.9612
80.3000 -423.0249 -1.8807 -1.2103 -
0.0633 -0.2198 0.3166 0.2245 -0.9282
1.5168 2.5528 -423.9529
80.3200 -420.2591 -0.2971 -0.4234
0.6120 -0.3550 0.3600 0.1757 -0.9142
1.5417 2.5584 -423.9447

80.3400 -417.1493 1.4552 1.0576
1.0994 -0.4754 0.4034 0.1253 -0.8999
1.5665 2.5639 -423.9364
80.3600 -417.4747 0.3251 1.6729
1.2631 -0.5777 0.4467 0.0732 -0.8852
1.5913 2.5693 -423.9282
80.3800 -419.2933 -0.4594 0.9632
1.0570 -0.6589 0.4898 0.0196 -0.8702
1.6161 2.5747 -423.9200
80.4000 -420.6652 -0.1583 -0.2773
0.5747 -0.7155 0.5323 -0.0357 -0.8548
1.6408 2.5800 -423.9118
80.4200 -421.7853 0.0294 -0.9412 -
0.0233 -0.7433 0.5739 -0.0924 -0.8390
1.6655 2.5852 -423.9035
80.4400 -422.2998 -0.6180 -0.6898 -
0.5725 -0.7386 0.6140 -0.1505 -0.8228
1.6902 2.5903 -423.8953
80.4600 -421.7458 -0.0593 -0.1631 -
0.9406 -0.6972 0.6520 -0.2099 -0.8061
1.7148 2.5954 -423.8871
80.4800 -420.9320 0.0794 0.1831 -
1.0709 -0.6168 0.6871 -0.2705 -0.7890
1.7394 2.6004 -423.8789
80.5000 -420.8263 0.2487 0.3042 -
0.9834 -0.4967 0.7186 -0.3321 -0.7714
1.7639 2.6054 -423.8706
80.5200 -420.9412 0.1297 0.1650 -
0.7359 -0.3384 0.7454 -0.3946 -0.7534
1.7884 2.6102 -423.8624
80.5400 -420.9950 -0.1916 -0.2669 -
0.4005 -0.1475 0.7665 -0.4579 -0.7348
1.8128 2.6150 -423.8542
80.5600 -420.6896 0.2843 -0.7582 -
0.0242 0.0658 0.7808 -0.5218 -0.7157
1.8372 2.6198 -423.8460
80.5800 -420.0902 0.0543 -0.8064
0.3461 0.2877 0.7872 -0.5861 -0.6960
1.8615 2.6244 -423.8378
80.6000 -419.0376 -0.6875 -0.2219
0.6423 0.5029 0.7846 -0.6507 -0.6758
1.8857 2.6290 -423.8296
80.6200 -417.4568 0.3284 0.7305
0.7887 0.6961 0.7720 -0.7154 -0.6550
1.9099 2.6335 -423.8214
80.6400 -417.0620 0.2770 1.3545
0.7331 0.8541 0.7486 -0.7800 -0.6336
1.9340 2.6380 -423.8132
80.6600 -417.7595 -0.3234 1.0959
0.5063 0.9687 0.7139 -0.8444 -0.6116
1.9580 2.6424 -423.8050

80.6800 -418.7392 -0.5713 0.0028
0.2115 1.0368 0.6675 -0.9084 -0.5890
1.9819 2.6467 -423.7968
80.7000 -420.2012 0.4708 -1.3485 -
0.0419 1.0578 0.6096 -0.9718 -0.5657
2.0058 2.6509 -423.7886
80.7200 -421.4953 -0.1038 -1.9737 -
0.1628 1.0338 0.5406 -1.0344 -0.5418
2.0295 2.6551 -423.7805
80.7400 -420.8105 -0.2373 -1.0983 -
0.1168 0.9684 0.4613 -1.0961 -0.5172
2.0532 2.6592 -423.7723
80.7600 -418.5057 -0.1530 0.7298
0.0268 0.8650 0.3726 -1.1566 -0.4919
2.0768 2.6632 -423.7641
80.7800 -417.7265 0.6263 1.9938
0.1591 0.7280 0.2759 -1.2157 -0.4659
2.1003 2.6672 -423.7559
80.8000 -418.1332 -0.2314 1.7784
0.1835 0.5633 0.1723 -1.2732 -0.4391
2.1237 2.6710 -423.7477
80.8200 -418.8615 0.3525 0.3880
0.0863 0.3786 0.0632 -1.3291 -0.4117
2.1469 2.6749 -423.7396
80.8400 -420.9394 -0.0941 -1.1677 -
0.0955 0.1805 -0.0499 -1.3830 -0.3834
2.1701 2.6786 -423.7314
80.8600 -423.5205 -0.1874 -1.7117 -
0.3036 -0.0251 -0.1652 -1.4347 -0.3544
2.1932 2.6823 -423.7232
80.8800 -423.2552 -0.8836 -0.7294 -
0.4720 -0.2323 -0.2809 -1.4842 -0.3245
2.2161 2.6859 -423.7151
80.9000 -420.5764 0.7575 0.8339 -
0.5418 -0.4350 -0.3954 -1.5312 -0.2939
2.2390 2.6894 -423.7069
80.9200 -419.2347 0.8356 1.4535 -
0.4854 -0.6287 -0.5066 -1.5755 -0.2624
2.2617 2.6929 -423.6987
80.9400 -421.2203 0.2114 0.7085 -
0.3086 -0.8092 -0.6128 -1.6169 -0.2301
2.2843 2.6963 -423.6906
80.9600 -423.5798 -0.7057 -0.4867 -
0.0259 -0.9701 -0.7124 -1.6554 -0.1969
2.3068 2.6997 -423.6824
80.9800 -423.6646 0.5698 -1.2992
0.3316 -1.1044 -0.8036 -1.6906 -0.1628
2.3291 2.7029 -423.6743
81.0000 -423.0996 -0.8252 -1.3566
0.7132 -1.2051 -0.8850 -1.7226 -0.1278
2.3513 2.7061 -423.6661

81.0200 -422.3110 -0.2500 -0.5667
1.0217 -1.2664 -0.9552 -1.7510 -0.0919
2.3734 2.7092 -423.6580
81.0400 -420.6321 0.2909 0.6702
1.1455 -1.2836 -1.0130 -1.7759 -0.0551
2.3953 2.7123 -423.6498
81.0600 -419.1920 0.1789 1.5110
0.9807 -1.2522 -1.0576 -1.7971 -0.0173
2.4172 2.7153 -423.6417
81.0800 -420.0338 0.6801 1.4074
0.4876 -1.1723 -1.0884 -1.8144 0.0214
2.4388 2.7182 -423.6336
81.1000 -422.6542 -0.2727 0.4937 -
0.2457 -1.0482 -1.1051 -1.8279 0.0610
2.4603 2.7211 -423.6254
81.1200 -423.9579 -0.3281 -0.5195 -
1.0402 -0.8856 -1.1079 -1.8374 0.1016
2.4817 2.7238 -423.6173
81.1400 -424.3877 0.3870 -1.1483 -
1.6803 -0.6916 -1.0968 -1.8431 0.1431
2.5029 2.7266 -423.6092
81.1600 -424.5933 0.1184 -1.2230 -
1.9810 -0.4743 -1.0720 -1.8450 0.1856
2.5240 2.7292 -423.6010
81.1800 -423.9748 -0.3739 -0.7119 -
1.8453 -0.2431 -1.0340 -1.8430 0.2290
2.5449 2.7318 -423.5929
81.2000 -422.2351 0.6828 -0.1106 -
1.2781 -0.0095 -0.9830 -1.8374 0.2734
2.5656 2.7343 -423.5848
81.2200 -420.8107 -0.1820 -0.0207 -
0.3762 0.2122 -0.9198 -1.8281 0.3187
2.5862 2.7367 -423.5767
81.2400 -419.9099 0.3450 -0.2952
0.6868 0.4067 -0.8449 -1.8154 0.3649
2.6067 2.7391 -423.5686
81.2600 -418.9223 -0.5801 -0.3062
1.6942 0.5589 -0.7589 -1.7992 0.4121
2.6269 2.7414 -423.5604
81.2800 -416.9629 0.0328 0.3569
2.4233 0.6540 -0.6624 -1.7799 0.4601
2.6470 2.7436 -423.5523
81.3000 -414.6312 0.4272 1.4407
2.7063 0.6788 -0.5565 -1.7574 0.5090
2.6670 2.7458 -423.5442
81.3200 -414.3993 0.0949 2.0894
2.4580 0.6277 -0.4425 -1.7319 0.5588
2.6867 2.7479 -423.5361
81.3400 -415.6608 0.3048 1.6985
1.7124 0.5085 -0.3220 -1.7036 0.6094
2.7063 2.7499 -423.5280

81.3600 -417.3894 0.1887 0.4767
0.6422 0.3388 -0.1971 -1.6726 0.6609
2.7257 2.7518 -423.5199
81.3800 -420.4199 -0.3569 -1.0538 -
0.5027 0.1382 -0.0701 -1.6390 0.7131
2.7449 2.7537 -423.5118
81.4000 -422.3943 -0.1342 -2.1102 -
1.4676 -0.0738 0.0569 -1.6030 0.7662
2.7640 2.7556 -423.5037
81.4200 -422.3517 0.3256 -2.1657 -
2.0448 -0.2774 0.1816 -1.5647 0.8200
2.7828 2.7573 -423.4956
81.4400 -422.2054 -0.1130 -1.4505 -
2.1631 -0.4528 0.3017 -1.5243 0.8745
2.8015 2.7590 -423.4875
81.4600 -421.4739 -0.4265 -0.3058 -
1.8945 -0.5820 0.4151 -1.4817 0.9298
2.8200 2.7606 -423.4795
81.4800 -418.6498 0.0670 0.8825 -
1.3941 -0.6500 0.5198 -1.4373 0.9858
2.8383 2.7622 -423.4714
81.5000 -417.0000 0.2860 1.6223 -
0.8702 -0.6455 0.6144 -1.3911 1.0424
2.8564 2.7636 -423.4633
81.5200 -417.0000 0.5821 1.6530 -
0.4868 -0.5603 0.6974 -1.3433 1.0997
2.8743 2.7650 -423.4552
81.5400 -417.0000 -0.7596 1.1741 -
0.3384 -0.3911 0.7680 -1.2940 1.1576
2.8920 2.7664 -423.4471
81.5600 -417.0000 -0.0363 0.5728 -
0.4029 -0.1467 0.8255 -1.2432 1.2161
2.9095 2.7677 -423.4391
81.5800 -417.0000 0.2778 -0.3392 -
0.5261 0.1546 0.8696 -1.1912 1.2752
2.9268 2.7689 -423.4310
81.6000 -417.2592 1.2980 -1.6303 -
0.5358 0.4908 0.9000 -1.1380 1.3349
2.9439 2.7700 -423.4229
81.6200 -418.8003 -0.1718 -2.3860 -
0.3181 0.8390 0.9164 -1.0838 1.3951
2.9607 2.7711 -423.4149
81.6400 -419.4337 -2.6987 -1.5605
0.1450 1.1758 0.9189 -1.0287 1.4558
2.9774 2.7721 -423.4068
81.6600 -413.0894 0.3551 0.6729
0.7368 1.4791 0.9073 -0.9728 1.5170
2.9939 2.7730 -423.3988
81.6800 -406.7610 3.5891 2.2705
1.2737 1.7285 0.8816 -0.9161 1.5786
3.0101 2.7739 -423.3907

81.7000 -409.8673 0.8039 1.7539
1.5908 1.9046 0.8418 -0.8589 1.6407
3.0262 2.7747 -423.3827
81.7200 -415.2869 -2.6807 0.1280
1.6452 1.9904 0.7882 -0.8011 1.7032
3.0420 2.7755 -423.3746
81.7400 -414.2244 -1.3942 -0.7245
1.4716 1.9765 0.7216 -0.7428 1.7661
3.0576 2.7761 -423.3666
81.7600 -411.3334 1.7934 -0.4039
1.1464 1.8618 0.6430 -0.6842 1.8293
3.0729 2.7767 -423.3585
81.7800 -412.0767 0.7203 -0.1993
0.7695 1.6518 0.5542 -0.6253 1.8929
3.0881 2.7773 -423.3505
81.8000 -415.2695 -0.5709 -0.7249
0.4161 1.3577 0.4570 -0.5663 1.9567
3.1030 2.7777 -423.3424
81.8200 -415.9348 -0.1524 -1.0494
0.1174 0.9974 0.3537 -0.5071 2.0209
3.1176 2.7781 -423.3344
81.8400 -415.5281 -0.1668 -0.4275 -
0.1210 0.5908 0.2463 -0.4478 2.0852
3.1321 2.7785 -423.3264
81.8600 -415.0817 0.0641 0.7477 -
0.3221 0.1571 0.1370 -0.3886 2.1499
3.1463 2.7787 -423.3183
81.8800 -414.5644 -0.4307 1.5332 -
0.5254 -0.2833 0.0280 -0.3296 2.2147
3.1603 2.7789 -423.3103
81.9000 -414.3084 1.3156 1.1827 -
0.7418 -0.7103 -0.0786 -0.2708 2.2796
3.1740 2.7791 -423.3023
81.9200 -417.3658 0.2977 -0.2770 -
0.9386 -1.1058 -0.1806 -0.2123 2.3448
3.1875 2.7791 -423.2943
81.9400 -420.8865 -2.0660 -1.4891 -
1.0335 -1.4539 -0.2761 -0.1543 2.4100
3.2007 2.7791 -423.2863
81.9600 -419.7251 0.1258 -1.3772 -
0.9421 -1.7411 -0.3631 -0.0967 2.4753
3.2137 2.7791 -423.2782
81.9800 -417.0073 1.0087 -0.4430 -
0.6659 -1.9557 -0.4395 -0.0397 2.5406
3.2264 2.7789 -423.2702
82.0000 -416.4689 1.1190 0.1529 -
0.2836 -2.0902 -0.5039 0.0166 2.6060
3.2389 2.7787 -423.2622
82.0200 -417.6476 -0.4646 0.1410
0.0887 -2.1411 -0.5550 0.0722 2.6714
3.2511 2.7785 -423.2542

82.0400 -418.2512 -1.7283 0.2713
0.3280 -2.1074 -0.5925 0.1271 2.7367
3.2631 2.7782 -423.2462
82.0600 -415.7368 -0.4525 1.0296
0.3640 -1.9910 -0.6160 0.1813 2.8020
3.2748 2.7778 -423.2382
82.0800 -413.0924 2.4232 1.5444
0.1692 -1.7964 -0.6261 0.2347 2.8671
3.2863 2.7773 -423.2302
82.1000 -414.9046 0.7724 0.6757 -
0.2050 -1.5304 -0.6237 0.2874 2.9321
3.2975 2.7768 -423.2222
82.1200 -418.6601 -1.3406 -0.9022 -
0.6236 -1.2025 -0.6098 0.3394 2.9970
3.3084 2.7762 -423.2142
82.1400 -418.2867 -1.0151 -1.4357 -
0.9403 -0.8257 -0.5858 0.3907 3.0616
3.3190 2.7755 -423.2062
82.1600 -414.9685 0.8755 -0.8608 -
1.0330 -0.4174 -0.5529 0.4414 3.1260
3.3294 2.7748 -423.1982
82.1800 -413.8216 0.7475 -0.3758 -
0.8415 0.0018 -0.5127 0.4915 3.1901
3.3395 2.7740 -423.1903
82.2000 -414.0526 -0.3632 -0.4495 -
0.3934 0.4104 -0.4665 0.5410 3.2539
3.3494 2.7731 -423.1823
82.2200 -414.1761 -0.9806 -0.4371
0.1937 0.7881 -0.4157 0.5899 3.3174
3.3589 2.7722 -423.1743
82.2400 -411.3030 -0.0836 0.3285
0.7666 1.1166 -0.3619 0.6383 3.3805
3.3682 2.7712 -423.1663
82.2600 -408.5121 0.3575 1.3167
1.1726 1.3799 -0.3065 0.6863 3.4432
3.3772 2.7702 -423.1584
82.2800 -408.7313 0.0160 1.4378
1.3224 1.5646 -0.2511 0.7337 3.5055
3.3859 2.7690 -423.1504
82.3000 -409.3135 0.4171 0.3230
1.2327 1.6623 -0.1970 0.7808 3.5672
3.3943 2.7679 -423.1424
82.3200 -410.8083 0.1798 -1.0914
0.9985 1.6711 -0.1457 0.8274 3.6285
3.4024 2.7666 -423.1345
82.3400 -412.4492 -0.7916 -1.5755
0.7286 1.5949 -0.0984 0.8737 3.6892
3.4102 2.7653 -423.1265
82.3600 -411.1020 -0.1869 -0.7408
0.4715 1.4437 -0.0559 0.9197 3.7493
3.4178 2.7639 -423.1185

82.3800 -408.6081 0.5751 0.6956
0.2360 1.2325 -0.0192 0.9654 3.8088
3.4250 2.7625 -423.1106
82.4000 -409.2716 0.7544 1.3366
0.0230 0.9811 0.0109 1.0108 3.8677
3.4320 2.7610 -423.1026
82.4200 -411.6549 -0.2438 0.4799 -
0.1917 0.7121 0.0337 1.0559 3.9259
3.4386 2.7594 -423.0947
82.4400 -412.9424 -0.4150 -0.8043 -
0.4332 0.4480 0.0487 1.1008 3.9834
3.4449 2.7578 -423.0867
82.4600 -413.7822 -0.6612 -0.8354 -
0.7126 0.2100 0.0555 1.1454 4.0401
3.4510 2.7560 -423.0788
82.4800 -413.0958 -0.9320 0.5491 -
0.9959 0.0160 0.0538 1.1896 4.0961
3.4567 2.7543 -423.0709
82.5000 -409.8292 1.2306 1.7528 -
1.2300 -0.1205 0.0436 1.2335 4.1512
3.4621 2.7524 -423.0629
82.5200 -409.7065 2.4973 1.1003 -
1.3492 -0.1920 0.0251 1.2770 4.2055
3.4672 2.7505 -423.0550
82.5400 -414.9253 -1.1320 -1.3119 -
1.2860 -0.2001 -0.0006 1.3200 4.2590
3.4720 2.7486 -423.0471
82.5600 -416.9775 -1.7537 -3.2887 -
1.0006 -0.1547 -0.0321 1.3624 4.3115
3.4764 2.7466 -423.0391
82.5800 -415.0079 -0.7356 -2.7410 -
0.4809 -0.0714 -0.0676 1.4041 4.3631
3.4806 2.7445 -423.0312
82.6000 -411.9933 -0.4463 0.1389
0.1878 0.0313 -0.1048 1.4451 4.4138
3.4844 2.7423 -423.0233
82.6200 -406.6819 0.2881 3.2361
0.8365 0.1343 -0.1416 1.4852 4.4635
3.4879 2.7401 -423.0154
82.6400 -402.6398 3.1406 3.8821
1.2920 0.2181 -0.1757 1.5244 4.5121
3.4910 2.7378 -423.0074
82.6600 -407.6913 0.1851 1.4614
1.4621 0.2645 -0.2051 1.5625 4.5598
3.4939 2.7355 -422.9995
82.6800 -413.3998 -3.0530 -1.8115
1.4038 0.2602 -0.2278 1.5994 4.6063
3.4964 2.7331 -422.9916
82.7000 -413.0233 0.4076 -3.2897
1.2054 0.1982 -0.2423 1.6352 4.6518
3.4986 2.7306 -422.9837

82.7200 -412.1676 -0.1524 -2.1504
0.9431 0.0772 -0.2475 1.6696 4.6962
3.5004 2.7280 -422.9758
82.7400 -409.7766 0.1817 0.2371
0.6495 -0.0976 -0.2426 1.7027 4.7394
3.5019 2.7254 -422.9679
82.7600 -407.2750 1.0901 1.9982
0.3562 -0.3146 -0.2276 1.7343 4.7815
3.5030 2.7228 -422.9600
82.7800 -408.5240 0.3702 1.9710
0.0748 -0.5581 -0.2027 1.7643 4.8224
3.5039 2.7201 -422.9521
82.8000 -411.1629 -0.7321 0.7347 -
0.2009 -0.8100 -0.1685 1.7926 4.8620
3.5043 2.7173 -422.9442
82.8200 -412.3457 0.0083 -0.4965 -
0.4649 -1.0519 -0.1256 1.8192 4.9005
3.5045 2.7144 -422.9363
82.8400 -413.0492 0.3544 -1.0614 -
0.6932 -1.2668 -0.0744 1.8440 4.9377
3.5042 2.7115 -422.9284
82.8600 -413.0894 -0.0594 -0.9914 -
0.8497 -1.4396 -0.0157 1.8668 4.9736
3.5036 2.7085 -422.9206
82.8800 -413.0381 -0.8701 -0.4560 -
0.9259 -1.5566 0.0498 1.8876 5.0081
3.5027 2.7055 -422.9127
82.9000 -412.4224 0.1584 0.4797 -
0.9235 -1.6087 0.1213 1.9062 5.0414
3.5014 2.7024 -422.9048
82.9200 -410.1075 0.9382 0.9859 -
0.8572 -1.5913 0.1979 1.9225 5.0733
3.4998 2.6992 -422.8969
82.9400 -409.8302 1.5887 0.3431 -
0.7437 -1.5048 0.2784 1.9365 5.1038
3.4978 2.6960 -422.8890
82.9600 -412.9677 -1.2388 -0.6443 -
0.5833 -1.3517 0.3613 1.9481 5.1330
3.4954 2.6927 -422.8812
82.9800 -413.8702 -2.6529 -0.5347 -
0.3820 -1.1358 0.4451 1.9571 5.1607
3.4927 2.6893 -422.8733
83.0000 -408.4141 1.7783 0.4340 -
0.1685 -0.8625 0.5283 1.9636 5.1870
3.4896 2.6859 -422.8654
83.0200 -405.6612 2.7786 0.6322
0.0176 -0.5407 0.6088 1.9673 5.2118
3.4861 2.6825 -422.8576
83.0400 -409.3463 -0.1782 -0.4446
0.1533 -0.1817 0.6847 1.9683 5.2352
3.4823 2.6789 -422.8497

83.0600 -411.6200 -2.5162 -1.2729
0.2231 0.2015 0.7540 1.9665 5.2571
3.4780 2.6753 -422.8419
83.0800 -408.0071 0.2111 -0.5769
0.2052 0.5935 0.8146 1.9617 5.2776
3.4735 2.6717 -422.8340
83.1000 -405.4055 1.0043 0.8596
0.0965 0.9760 0.8647 1.9540 5.2965
3.4685 2.6679 -422.8262
83.1200 -405.8781 0.4667 1.3832 -
0.0627 1.3290 0.9025 1.9431 5.3139
3.4632 2.6642 -422.8183
83.1400 -406.4592 -0.4627 0.7185 -
0.1971 1.6333 0.9263 1.9292 5.3298
3.4574 2.6603 -422.8105
83.1600 -406.8474 -0.0848 -0.2155 -
0.2231 1.8705 0.9347 1.9121 5.3442
3.4513 2.6564 -422.8026
83.1800 -407.1479 0.1660 -0.8615 -
0.0884 2.0241 0.9264 1.8920 5.3571
3.4449 2.6525 -422.7948
83.2000 -407.4072 -0.0578 -1.1192
0.2197 2.0808 0.9007 1.8687 5.3684
3.4380 2.6484 -422.7870
83.2200 -407.4471 -0.0193 -0.8622
0.6558 2.0320 0.8577 1.8423 5.3783
3.4308 2.6444 -422.7791
83.2400 -406.2384 -0.2961 -0.0524
1.0952 1.8752 0.7980 1.8130 5.3866
3.4231 2.6402 -422.7713
83.2600 -405.1310 -0.4127 1.0618
1.3683 1.6135 0.7233 1.7809 5.3935
3.4151 2.6360 -422.7635
83.2800 -404.4855 0.2127 1.6621
1.3260 1.2575 0.6357 1.7460 5.3989
3.4067 2.6318 -422.7557
83.3000 -404.8167 1.9343 0.8790
0.9252 0.8281 0.5378 1.7085 5.4028
3.3979 2.6274 -422.7479
83.3200 -410.4815 -1.2322 -0.7412
0.2774 0.3534 0.4323 1.6686 5.4052
3.3887 2.6231 -422.7400
83.3400 -412.4742 -0.9873 -1.4222 -
0.4323 -0.1365 0.3218 1.6263 5.4062
3.3792 2.6186 -422.7322
83.3600 -411.4563 0.1288 -0.5676 -
1.0173 -0.6115 0.2088 1.5819 5.4057
3.3692 2.6141 -422.7244
83.3800 -411.0189 0.2887 0.5565 -
1.3316 -1.0434 0.0962 1.5355 5.4039
3.3589 2.6096 -422.7166

83.4000	-411.3770	0.6319	0.7955	-
1.2952	-1.4068	-0.0136	1.4873	5.4006
	3.3482	2.6050	-422.7088	
83.4200	-412.0062	0.1011	-0.0918	-
0.9253	-1.6814	-0.1181	1.4373	5.3959
	3.3371	2.6003	-422.7010	
83.4400	-413.6254	0.2996	-1.1702	-
0.3515	-1.8554	-0.2147	1.3858	5.3899
	3.3256	2.5956	-422.6932	
83.4600	-414.7993	-2.1597	-0.9837	
0.2540	-1.9237	-0.3013	1.3329	5.3825
	3.3137	2.5908	-422.6854	
83.4800	-411.6413	-0.0657	0.5329	
0.7167	-1.8868	-0.3762	1.2788	5.3737
	3.3015	2.5860	-422.6776	
83.5000	-407.6326	2.2423	1.6705	
0.8833	-1.7509	-0.4385	1.2236	5.3636
	3.2888	2.5811	-422.6698	
83.5200	-410.1699	0.8386	0.9110	
0.6660	-1.5287	-0.4880	1.1675	5.3522
	3.2758	2.5762	-422.6621	
83.5400	-414.8076	-2.1802	-0.6833	
0.1476	-1.2400	-0.5245	1.1107	5.3395
	3.2624	2.5712	-422.6543	
83.5600	-414.5075	-0.3939	-1.2316	-
0.4698	-0.9102	-0.5484	1.0534	5.3255
	3.2487	2.5661	-422.6465	
83.5800	-412.8903	0.2235	-0.4249	-
0.9811	-0.5653	-0.5599	0.9957	5.3102
	3.2345	2.5610	-422.6387	
83.6000	-411.7698	0.0303	0.5971	-
1.2101	-0.2305	-0.5595	0.9379	5.2937
	3.2200	2.5559	-422.6309	
83.6200	-410.8986	0.3583	0.8433	-
1.0353	0.0717	-0.5476	0.8800	5.2759
	3.2051	2.5507	-422.6232	
83.6400	-410.8864	0.4751	-0.1348	-
0.4432	0.3248	-0.5247	0.8223	5.2569
	3.1899	2.5454	-422.6154	
83.6600	-412.2210	0.1917	-1.6462	
0.4203	0.5219	-0.4919	0.7649	5.2367
	3.1742	2.5401	-422.6076	
83.6800	-412.4943	-1.4994	-1.8834	
1.3117	0.6617	-0.4503	0.7079	5.2154
	3.1583	2.5347	-422.5999	
83.7000	-408.2629	0.5526	-0.1743	
1.9842	0.7445	-0.4016	0.6515	5.1928
	3.1419	2.5293	-422.5921	
83.7200	-405.7492	0.4782	2.1372	
2.2301	0.7725	-0.3478	0.5958	5.1691
	3.1252	2.5238	-422.5844	

83.7400 -405.5532 0.2241 3.0249
1.9037 0.7525 -0.2910 0.5411 5.1442
3.1082 2.5182 -422.5766
83.7600 -405.7964 2.8626 1.3625
1.0197 0.6982 -0.2335 0.4872 5.1183
3.0908 2.5127 -422.5689
83.7800 -413.9249 -1.2910 -1.4326 -
0.1745 0.6260 -0.1774 0.4345 5.0912
3.0730 2.5070 -422.5611
83.8000 -418.5454 -3.5641 -2.7446 -
1.3436 0.5527 -0.1248 0.3831 5.0630
3.0549 2.5013 -422.5534
83.8200 -414.5880 1.2930 -1.5244 -
2.1723 0.4948 -0.0776 0.3329 5.0338
3.0365 2.4956 -422.5456
83.8400 -410.8709 2.1686 0.3232 -
2.4872 0.4674 -0.0375 0.2842 5.0035
3.0177 2.4898 -422.5379
83.8600 -412.0072 0.3564 0.7739 -
2.2176 0.4786 -0.0057 0.2370 4.9722
2.9985 2.4839 -422.5302
83.8800 -413.9689 -1.1406 -0.2242 -
1.4368 0.5255 0.0171 0.1915 4.9398
2.9791 2.4781 -422.5224
83.9000 -413.6065 -0.5698 -1.1328 -
0.3852 0.5967 0.0309 0.1477 4.9065
2.9593 2.4721 -422.5147
83.9200 -411.9422 -1.0360 -0.5247
0.6524 0.6771 0.0358 0.1057 4.8722
2.9392 2.4661 -422.5070
83.9400 -409.4194 -0.0527 1.3479
1.4575 0.7511 0.0325 0.0656 4.8368
2.9187 2.4601 -422.4993
83.9600 -405.6732 1.8998 2.4788
1.8723 0.8024 0.0217 0.0273 4.8006
2.8980 2.4540 -422.4916
83.9800 -406.3048 2.5211 1.4306
1.8170 0.8177 0.0043 -0.0090 4.7634
2.8769 2.4478 -422.4838
84.0000 -413.0402 -2.2582 -0.6947
1.4057 0.7884 -0.0186 -0.0433 4.7253
2.8555 2.4416 -422.4761
84.0200 -414.5565 -1.5493 -1.6645
0.8323 0.7108 -0.0458 -0.0757 4.6863
2.8337 2.4354 -422.4684
84.0400 -411.7251 0.8071 -1.0201
0.2904 0.5823 -0.0760 -0.1060 4.6464
2.8117 2.4291 -422.4607
84.0600 -410.8593 1.4146 -0.2923 -
0.0655 0.4032 -0.1078 -0.1343 4.6056
2.7894 2.4228 -422.4530

84.0800 -413.7997 -0.3639 -0.2136 -
0.1859 0.1799 -0.1398 -0.1606 4.5640
2.7668 2.4164 -422.4453
84.1000 -415.4348 -2.2717 0.1855 -
0.1573 -0.0747 -0.1707 -0.1848 4.5215
2.7438 2.4100 -422.4376
84.1200 -412.7124 -0.5521 1.1777 -
0.1086 -0.3423 -0.1992 -0.2070 4.4782
2.7206 2.4035 -422.4299
84.1400 -410.1294 2.8466 1.3693 -
0.1266 -0.6030 -0.2245 -0.2272 4.4341
2.6971 2.3969 -422.4222
84.1600 -413.9107 0.8378 -0.1344 -
0.2506 -0.8390 -0.2454 -0.2454 4.3892
2.6733 2.3904 -422.4146
84.1800 -419.6061 -2.4010 -1.7795 -
0.4380 -1.0356 -0.2612 -0.2617 4.3435
2.6492 2.3838 -422.4069
84.2000 -418.2051 -1.6150 -1.6102 -
0.6296 -1.1832 -0.2711 -0.2761 4.2970
2.6248 2.3771 -422.3992
84.2200 -414.2835 1.2450 0.1452 -
0.7789 -1.2752 -0.2744 -0.2885 4.2498
2.6001 2.3704 -422.3915
84.2400 -413.5065 1.3558 1.6939 -
0.8507 -1.3082 -0.2708 -0.2991 4.2018
2.5752 2.3636 -422.3838
84.2600 -414.9369 -0.3393 1.6139 -
0.8092 -1.2833 -0.2602 -0.3079 4.1531
2.5500 2.3568 -422.3762
84.2800 -416.1302 -0.1530 0.1802 -
0.6179 -1.2063 -0.2426 -0.3148 4.1037
2.5245 2.3500 -422.3685
84.3000 -416.8309 -0.3472 -1.1820 -
0.2683 -1.0857 -0.2187 -0.3200 4.0536
2.4988 2.3431 -422.3608
84.3200 -417.1962 -0.9022 -1.4723
0.2053 -0.9306 -0.1890 -0.3236 4.0028
2.4728 2.3361 -422.3532
84.3400 -415.1132 -0.4504 -0.6585
0.7034 -0.7492 -0.1544 -0.3254 3.9514
2.4465 2.3291 -422.3455
84.3600 -411.6152 0.6687 0.6285
1.0863 -0.5496 -0.1159 -0.3258 3.8993
2.4200 2.3221 -422.3379
84.3800 -411.2707 0.0133 1.4106
1.2159 -0.3380 -0.0746 -0.3246 3.8465
2.3932 2.3150 -422.3302
84.4000 -411.6188 0.0894 1.2040
1.0099 -0.1187 -0.0317 -0.3220 3.7932
2.3662 2.3079 -422.3226

84.4200 -412.5424 0.3294 0.2504
0.5183 0.1044 0.0115 -0.3181 3.7392
2.3389 2.3008 -422.3149
84.4400 -415.2463 -0.0415 -0.7925 -
0.1124 0.3260 0.0538 -0.3129 3.6847
2.3114 2.2936 -422.3073
84.4600 -416.1855 -0.9539 -1.0349 -
0.7158 0.5405 0.0939 -0.3064 3.6296
2.2837 2.2863 -422.2996
84.4800 -414.6230 0.3489 -0.3021 -
1.1397 0.7427 0.1306 -0.2988 3.5739
2.2557 2.2790 -422.2920
84.5000 -413.5005 1.0407 0.3784 -
1.2876 0.9265 0.1624 -0.2901 3.5177
2.2275 2.2717 -422.2844
84.5200 -414.1180 0.0272 0.0980 -
1.1113 1.0857 0.1883 -0.2803 3.4609
2.1991 2.2643 -422.2767
84.5400 -414.8685 -0.8225 -0.6142 -
0.6426 1.2146 0.2072 -0.2696 3.4037
2.1704 2.2569 -422.2691
84.5600 -414.2421 -0.1752 -0.7218
0.0322 1.3072 0.2185 -0.2580 3.3460
2.1415 2.2495 -422.2615
84.5800 -412.5854 0.4223 -0.1135
0.7585 1.3569 0.2216 -0.2456 3.2877
2.1124 2.2420 -422.2539
84.6000 -411.3903 0.1263 0.7444
1.3395 1.3577 0.2164 -0.2325 3.2290
2.0831 2.2345 -422.2462
84.6200 -410.4142 -0.1627 1.4559
1.5969 1.3047 0.2030 -0.2187 3.1698
2.0536 2.2269 -422.2386
84.6400 -410.4485 0.9330 1.5287
1.4343 1.1966 0.1821 -0.2044 3.1101
2.0239 2.2193 -422.2310
84.6600 -412.3229 -0.7629 0.9500
0.8737 1.0377 0.1544 -0.1895 3.0500
1.9939 2.2116 -422.2234
84.6800 -414.0701 -0.4274 0.3701
0.0623 0.8375 0.1211 -0.1743 2.9895
1.9638 2.2039 -422.2158
84.7000 -415.2336 0.5733 -0.0746 -
0.7746 0.6077 0.0834 -0.1587 2.9285
1.9334 2.1962 -422.2082
84.7200 -416.5036 0.3172 -0.7970 -
1.4096 0.3600 0.0425 -0.1428 2.8671
1.9029 2.1884 -422.2006
84.7400 -418.6542 0.6854 -2.0610 -
1.6628 0.1056 -0.0001 -0.1268 2.8053
1.8722 2.1806 -422.1930

84.7600 -420.3993 -0.5552 -3.0701 -
1.4599 -0.1458 -0.0427 -0.1105 2.7431
1.8413 2.1727 -422.1854
84.7800 -419.7834 -0.5271 -2.7553 -
0.8672 -0.3873 -0.0835 -0.0943 2.6804
1.8102 2.1648 -422.1778
84.8000 -417.4592 -0.1333 -0.8477 -
0.0567 -0.6127 -0.1209 -0.0779 2.6174
1.7789 2.1569 -422.1702
84.8200 -414.8996 -0.9216 1.9806
0.7481 -0.8158 -0.1532 -0.0617 2.5540
1.7475 2.1489 -422.1627
84.8400 -411.5212 0.4418 4.0428
1.3330 -0.9908 -0.1788 -0.0455 2.4903
1.7159 2.1409 -422.1551
84.8600 -409.9578 2.8696 3.5342
1.5406 -1.1327 -0.1964 -0.0294 2.4262
1.6841 2.1329 -422.1475
84.8800 -416.0236 -0.2306 0.3177
1.3681 -1.2362 -0.2049 -0.0135 2.3617
1.6521 2.1248 -422.1399
84.9000 -422.4540 -2.6172 -3.0313
0.9406 -1.2985 -0.2038 0.0022 2.2969
1.6200 2.1167 -422.1324
84.9200 -421.5442 -0.3292 -3.8235
0.4049 -1.3196 -0.1931 0.0177 2.2317
1.5877 2.1085 -422.1248
84.9400 -419.1620 0.2659 -2.0302 -
0.1250 -1.2996 -0.1730 0.0329 2.1662
1.5553 2.1003 -422.1172
84.9600 -417.7365 0.8538 0.5731 -
0.5903 -1.2341 -0.1443 0.0480 2.1003
1.5227 2.0921 -422.1097
84.9800 -416.7933 0.3033 2.2469 -
0.9675 -1.1160 -0.1079 0.0628 2.0342
1.4900 2.0839 -422.1021
85.0000 -416.4010 -0.1665 2.5476 -
1.2696 -0.9397 -0.0650 0.0774 1.9677
1.4571 2.0756 -422.0946
85.0200 -417.0181 -0.4251 1.8937 -
1.5001 -0.7056 -0.0170 0.0919 1.9008
1.4241 2.0672 -422.0870
85.0400 -418.0637 0.2803 0.7984 -
1.6062 -0.4246 0.0344 0.1061 1.8337
1.3909 2.0589 -422.0795
85.0600 -418.8359 0.1203 -0.5079 -
1.4998 -0.1139 0.0875 0.1203 1.7662
1.3577 2.0505 -422.0719
85.0800 -419.5209 0.0203 -1.9156 -
1.0999 0.2084 0.1405 0.1343 1.6984
1.3242 2.0420 -422.0644

85.1000 -419.6027 -0.0509 -3.0551 -
0.3963 0.5230 0.1918 0.1482 1.6304
1.2907 2.0335 -422.0568
85.1200 -418.3358 -0.3759 -2.9988
0.5348 0.8078 0.2401 0.1621 1.5620
1.2570 2.0250 -422.0493
85.1400 -416.2284 -1.1619 -0.9971
1.5226 1.0399 0.2838 0.1760 1.4933
1.2232 2.0165 -422.0418
85.1600 -411.8849 -0.9496 2.1647
2.3054 1.1976 0.3215 0.1899 1.4243
1.1893 2.0079 -422.0343
85.1800 -406.8096 2.6109 4.0640
2.6193 1.2621 0.3520 0.2038 1.3551
1.1553 1.9993 -422.0267
85.2000 -409.0374 1.8404 2.7655
2.2957 1.2252 0.3740 0.2178 1.2855
1.1211 1.9907 -422.0192
85.2200 -417.8399 -2.6492 -0.5568
1.4770 1.0998 0.3873 0.2320 1.2157
1.0869 1.9820 -422.0117
85.2400 -419.4064 -0.7933 -2.8272
0.4426 0.9085 0.3915 0.2463 1.1456
1.0525 1.9733 -422.0042
85.2600 -418.9210 0.2330 -2.6222 -
0.5261 0.6754 0.3870 0.2608 1.0753
1.0181 1.9646 -421.9967
85.2800 -418.3998 -0.0339 -0.9881 -
1.2237 0.4243 0.3737 0.2755 1.0047
0.9836 1.9558 -421.9892
85.3000 -417.8109 0.3695 0.6684 -
1.5631 0.1763 0.3519 0.2905 0.9338
0.9489 1.9470 -421.9816
85.3200 -417.4129 -0.2005 1.6346 -
1.5884 -0.0489 0.3219 0.3059 0.8627
0.9142 1.9382 -421.9741
85.3400 -417.5348 -0.0923 1.7486 -
1.4028 -0.2328 0.2842 0.3215 0.7914
0.8794 1.9293 -421.9666
85.3600 -417.9777 0.4766 0.9903 -
1.1029 -0.3636 0.2390 0.3376 0.7198
0.8445 1.9204 -421.9591
85.3800 -418.7985 0.2447 -0.4842 -
0.7308 -0.4406 0.1871 0.3540 0.6479
0.8095 1.9115 -421.9517
85.4000 -420.7637 -0.0067 -1.8510 -
0.3090 -0.4675 0.1294 0.3708 0.5759
0.7744 1.9025 -421.9442
85.4200 -421.8966 -2.0387 -1.7809
0.1340 -0.4510 0.0668 0.3880 0.5036
0.7393 1.8935 -421.9367

85.4400 -418.9767 -0.6932 0.0280
0.5322 -0.3969 0.0008 0.4057 0.4311
0.7041 1.8845 -421.9292
85.4600 -414.9174 1.9795 1.9099
0.7695 -0.3112 -0.0675 0.4238 0.3585
0.6688 1.8755 -421.9217
85.4800 -415.4523 0.9348 2.0082
0.7518 -0.2020 -0.1368 0.4423 0.2856
0.6335 1.8664 -421.9142
85.5000 -419.2320 -1.1259 0.5513
0.4975 -0.0804 -0.2056 0.4611 0.2125
0.5981 1.8573 -421.9068
85.5200 -420.7870 -1.0062 -0.8396
0.1119 0.0412 -0.2726 0.4804 0.1393
0.5627 1.8482 -421.8993
85.5400 -420.5426 0.7661 -1.1594 -
0.2513 0.1498 -0.3362 0.5000 0.0660
0.5272 1.8390 -421.8918
85.5600 -420.3083 -0.1568 -0.8432 -
0.4392 0.2321 -0.3951 0.5199 -0.0076
0.4916 1.8298 -421.8844
85.5800 -420.3254 0.4482 -0.6125 -
0.3798 0.2769 -0.4477 0.5401 -0.0812
0.4560 1.8206 -421.8769
85.6000 -420.3825 -0.6035 -0.4808 -
0.1051 0.2789 -0.4931 0.5605 -0.1550
0.4204 1.8114 -421.8695
85.6200 -420.2395 0.1488 -0.1621
0.2902 0.2374 -0.5302 0.5810 -0.2288
0.3847 1.8021 -421.8620
85.6400 -418.6768 0.2772 0.4464
0.6936 0.1556 -0.5586 0.6016 -0.3028
0.3490 1.7928 -421.8545
85.6600 -417.4145 0.3061 0.9994
0.9926 0.0394 -0.5777 0.6222 -0.3768
0.3132 1.7835 -421.8471
85.6800 -417.7048 -0.3817 1.0618
1.1020 -0.1029 -0.5874 0.6427 -0.4509
0.2774 1.7741 -421.8397
85.7000 -418.5204 -0.1916 0.6405
0.9783 -0.2615 -0.5874 0.6630 -0.5251
0.2416 1.7647 -421.8322
85.7200 -419.7426 0.2549 0.0586
0.6422 -0.4249 -0.5779 0.6830 -0.5992
0.2058 1.7553 -421.8248
85.7400 -421.9489 -0.5740 -0.6206
0.1858 -0.5800 -0.5585 0.7025 -0.6734
0.1699 1.7459 -421.8173
85.7600 -423.0994 0.1635 -1.0164 -
0.2966 -0.7126 -0.5295 0.7215 -0.7476
0.1341 1.7365 -421.8099

85.7800 -422.9228 0.1293 -0.6425 -
0.7151 -0.8088 -0.4908 0.7397 -0.8218
0.0982 1.7270 -421.8025
85.8000 -422.6156 0.0999 -0.0354 -
1.0038 -0.8576 -0.4427 0.7570 -0.8959
0.0623 1.7175 -421.7951
85.8200 -422.3999 -0.4786 0.2307 -
1.1609 -0.8510 -0.3857 0.7733 -0.9699
0.0264 1.7079 -421.7876
85.8400 -422.2194 0.2448 0.2945 -
1.1962 -0.7864 -0.3208 0.7885 -1.0439
-0.0095 1.6984 -421.7802
85.8600 -422.1021 -0.2199 0.1982 -
1.1074 -0.6668 -0.2491 0.8023 -1.1177
-0.0454 1.6888 -421.7728
85.8800 -422.0618 0.9039 -0.2486 -
0.8778 -0.5000 -0.1720 0.8147 -1.1914
-0.0813 1.6792 -421.7654
85.9000 -422.0332 -0.4103 -0.7845 -
0.5051 -0.2967 -0.0913 0.8256 -1.2650
-0.1172 1.6696 -421.7580
85.9200 -421.6618 -0.0275 -0.6715 -
0.0344 -0.0720 -0.0086 0.8347 -1.3384
-0.1530 1.6599 -421.7506
85.9400 -419.5022 0.1593 0.2069
0.4496 0.1575 0.0745 0.8421 -1.4116
-0.1889 1.6502 -421.7432
85.9600 -417.9108 0.4472 1.1736
0.8600 0.3747 0.1563 0.8475 -1.4846
-0.2247 1.6406 -421.7358
85.9800 -418.1688 -0.1906 1.2715
1.1421 0.5635 0.2351 0.8509 -1.5574
-0.2606 1.6308 -421.7284
86.0000 -418.7905 0.0762 0.3083
1.2917 0.7111 0.3094 0.8521 -1.6299
-0.2964 1.6211 -421.7210
86.0200 -419.4311 -0.3522 -0.9242
1.3405 0.8103 0.3778 0.8511 -1.7021
-0.3322 1.6113 -421.7136
86.0400 -420.2235 0.3093 -1.3612
1.3049 0.8590 0.4393 0.8477 -1.7741
-0.3679 1.6015 -421.7062
86.0600 -420.5930 -0.4639 -0.6653
1.1571 0.8591 0.4930 0.8418 -1.8457
-0.4036 1.5917 -421.6989
86.0800 -419.0683 -0.4494 0.7905
0.8603 0.8159 0.5383 0.8333 -1.9169
-0.4393 1.5819 -421.6915
86.1000 -417.0455 0.7519 1.7935
0.3904 0.7381 0.5750 0.8222 -1.9878
-0.4749 1.5721 -421.6841

86.1200 -418.2270 1.4183 1.1490 -
0.2195 0.6374 0.6027 0.8083 -2.0583
-0.5105 1.5622 -421.6767
86.1400 -423.3294 -1.2398 -0.5828 -
0.8544 0.5265 0.6217 0.7916 -2.1284
-0.5461 1.5523 -421.6694
86.1600 -425.6531 -1.3128 -1.6007 -
1.3622 0.4169 0.6320 0.7720 -2.1981
-0.5815 1.5424 -421.6620
86.1800 -424.0694 0.1858 -1.0318 -
1.6074 0.3186 0.6339 0.7495 -2.2673
-0.6170 1.5325 -421.6546
86.2000 -422.1620 0.4645 0.2390 -
1.5338 0.2375 0.6281 0.7239 -2.3360
-0.6524 1.5225 -421.6473
86.2200 -421.9418 0.3036 1.0259 -
1.1857 0.1749 0.6154 0.6952 -2.4043
-0.6877 1.5125 -421.6399
86.2400 -422.1287 -0.4603 0.8985 -
0.6748 0.1282 0.5969 0.6634 -2.4720
-0.7230 1.5026 -421.6326
86.2600 -422.3007 0.1472 0.1736 -
0.0971 0.0916 0.5740 0.6284 -2.5392
-0.7581 1.4925 -421.6252
86.2800 -422.4071 0.3193 -0.5625
0.4441 0.0566 0.5482 0.5902 -2.6058
-0.7933 1.4825 -421.6179
86.3000 -422.4859 -0.3886 -0.7998
0.8646 0.0127 0.5211 0.5488 -2.6718
-0.8283 1.4725 -421.6105
86.3200 -422.3255 0.0768 -0.2563
1.1147 -0.0517 0.4940 0.5041 -2.7373
-0.8633 1.4624 -421.6032
86.3400 -421.2302 0.0252 0.6366
1.1868 -0.1465 0.4683 0.4560 -2.8021
-0.8982 1.4523 -421.5959
86.3600 -420.4083 0.3120 1.0695
1.0927 -0.2771 0.4455 0.4046 -2.8662
-0.9330 1.4422 -421.5885
86.3800 -421.8440 0.5843 0.5372
0.8813 -0.4422 0.4266 0.3500 -2.9297
-0.9677 1.4321 -421.5812
86.4000 -424.6013 -0.8381 -0.5669
0.6099 -0.6346 0.4127 0.2920 -2.9925
-1.0023 1.4220 -421.5739
86.4200 -425.3604 -0.2470 -1.1079
0.3472 -0.8424 0.4042 0.2308 -3.0546
-1.0369 1.4118 -421.5666
86.4400 -424.9824 0.5836 -0.6836
0.1370 -1.0522 0.4015 0.1663 -3.1159
-1.0713 1.4017 -421.5592

86.4600 -424.6247 0.1329 0.2900 -
0.0291 -1.2476 0.4048 0.0988 -3.1765
-1.1057 1.3915 -421.5519
86.4800 -424.8485 -0.3101 0.9676 -
0.2098 -1.4108 0.4142 0.0282 -3.2363
-1.1399 1.3813 -421.5446
86.5000 -425.6911 0.2234 0.9159 -
0.4710 -1.5241 0.4296 -0.0452 -3.2953
-1.1740 1.3711 -421.5373
86.5200 -426.5264 -0.0470 0.4437 -
0.8212 -1.5732 0.4511 -0.1214 -3.3534
-1.2081 1.3609 -421.5300
86.5400 -427.2190 -0.5167 -0.0335 -
1.1780 -1.5501 0.4779 -0.2002 -3.4107
-1.2420 1.3506 -421.5227
86.5600 -427.8394 0.4216 -0.3702 -
1.4001 -1.4513 0.5092 -0.2814 -3.4672
-1.2758 1.3404 -421.5154
86.5800 -428.1698 0.2146 -0.8033 -
1.3667 -1.2770 0.5437 -0.3647 -3.5228
-1.3094 1.3301 -421.5081
86.6000 -428.3662 0.5529 -1.5382 -
1.0203 -1.0341 0.5799 -0.4500 -3.5774
-1.3430 1.3198 -421.5008
86.6200 -428.4839 -0.8056 -1.8237 -
0.4035 -0.7370 0.6164 -0.5370 -3.6311
-1.3764 1.3095 -421.4935
86.6400 -427.6555 -1.6904 -0.7880
0.3419 -0.4044 0.6519 -0.6255 -3.6839
-1.4097 1.2992 -421.4862
86.6600 -423.1485 0.0841 1.2966
1.0014 -0.0562 0.6850 -0.7153 -3.7356
-1.4429 1.2889 -421.4790
86.6800 -419.2616 1.7899 2.7501
1.3672 0.2883 0.7141 -0.8060 -3.7864
-1.4759 1.2785 -421.4717
86.7000 -420.6501 0.5862 2.0466
1.2948 0.6130 0.7376 -0.8976 -3.8362
-1.5088 1.2682 -421.4644
86.7200 -425.2598 -0.9766 -0.0535
0.8392 0.9098 0.7537 -0.9896 -3.8849
-1.5416 1.2578 -421.4571
86.7400 -427.0641 -0.9811 -1.3936
0.1783 1.1751 0.7605 -1.0819 -3.9325
-1.5742 1.2474 -421.4499
86.7600 -426.3384 0.1547 -1.0206 -
0.4798 1.4062 0.7564 -1.1742 -3.9790
-1.6066 1.2370 -421.4426
86.7800 -425.4648 0.5734 -0.1653 -
0.9423 1.6013 0.7398 -1.2662 -4.0245
-1.6389 1.2266 -421.4354

86.8000 -425.4469 0.2231 -0.2177 -
1.0785 1.7580 0.7093 -1.3577 -4.0688
-1.6711 1.2162 -421.4281
86.8200 -426.2819 -0.3585 -0.9216 -
0.8980 1.8731 0.6641 -1.4484 -4.1119
-1.7031 1.2058 -421.4208
86.8400 -426.9712 0.0096 -1.0462 -
0.5153 1.9439 0.6040 -1.5382 -4.1538
-1.7349 1.1953 -421.4136
86.8600 -426.0859 -0.9878 0.0657 -
0.0754 1.9668 0.5294 -1.6266 -4.1946
-1.7666 1.1849 -421.4063
86.8800 -423.7345 0.3906 1.6108
0.3041 1.9374 0.4413 -1.7137 -4.2341
-1.7981 1.1744 -421.3991
86.9000 -422.5103 1.4736 1.8729
0.5605 1.8518 0.3410 -1.7990 -4.2724
-1.8294 1.1639 -421.3919
86.9200 -425.0553 0.3087 0.2214
0.7087 1.7059 0.2302 -1.8823 -4.3094
-1.8606 1.1535 -421.3846
86.9400 -428.8609 -1.7416 -1.6955
0.8351 1.4955 0.1107 -1.9636 -4.3452
-1.8915 1.1430 -421.3774
86.9600 -429.5051 -1.5135 -1.9902
1.0159 1.2178 -0.0153 -2.0424 -4.3796
-1.9223 1.1325 -421.3702
86.9800 -427.1791 0.2322 -0.5179
1.2474 0.8748 -0.1459 -2.1186 -4.4126
-1.9530 1.1220 -421.3629
87.0000 -424.8745 0.8314 1.0566
1.4616 0.4774 -0.2789 -2.1921 -4.4444
-1.9834 1.1114 -421.3557
87.0200 -425.1534 0.3529 1.5143
1.5701 0.0419 -0.4119 -2.2625 -4.4747
-2.0136 1.1009 -421.3485
87.0400 -427.5588 0.0818 1.0351
1.4793 -0.4117 -0.5429 -2.3297 -4.5036
-2.0437 1.0904 -421.3413
87.0600 -430.0393 -0.6841 0.3512
1.1364 -0.8620 -0.6694 -2.3934 -4.5312
-2.0735 1.0798 -421.3341
87.0800 -431.4622 0.0385 -0.2075
0.5512 -1.2880 -0.7894 -2.4535 -4.5572
-2.1032 1.0693 -421.3268
87.1000 -432.6263 0.1482 -0.5174 -
0.2075 -1.6701 -0.9005 -2.5097 -4.5818
-2.1326 1.0587 -421.3196
87.1200 -433.6525 0.3150 -0.5793 -
1.0343 -1.9890 -1.0006 -2.5619 -4.6049
-2.1619 1.0482 -421.3124

87.1400 -434.7451 -0.3715 -0.3067 -
1.8097 -2.2260 -1.0873 -2.6098 -4.6265
-2.1909 1.0376 -421.3052
87.1600 -435.7778 0.2059 0.0762 -
2.4216 -2.3627 -1.1587 -2.6533 -4.6466
-2.2197 1.0270 -421.2980
87.1800 -436.2943 -0.1228 0.1919 -
2.8032 -2.3833 -1.2131 -2.6922 -4.6651
-2.2484 1.0164 -421.2908
87.2000 -436.2659 0.1711 0.1288 -
2.9151 -2.2793 -1.2493 -2.7264 -4.6821
-2.2768 1.0058 -421.2837
87.2200 -436.1523 0.1962 -0.0283 -
2.7167 -2.0545 -1.2677 -2.7557 -4.6975
-2.3049 0.9952 -421.2765
87.2400 -435.9682 -0.3727 -0.3916 -
2.1678 -1.7246 -1.2689 -2.7802 -4.7112
-2.3329 0.9846 -421.2693
87.2600 -435.3273 -0.2186 -0.9253 -
1.2693 -1.3141 -1.2542 -2.7999 -4.7234
-2.3606 0.9740 -421.2621
87.2800 -433.4758 0.5058 -1.3452 -
0.0970 -0.8523 -1.2250 -2.8147 -4.7339
-2.3881 0.9634 -421.2549
87.3000 -431.1543 -0.3280 -1.1485
1.1954 -0.3692 -1.1825 -2.8248 -4.7428
-2.4154 0.9528 -421.2478
87.3200 -428.8705 -0.2903 0.0278
2.3799 0.1052 -1.1281 -2.8302 -4.7500
-2.4425 0.9421 -421.2406
87.3400 -425.9653 0.0919 1.6685
3.1930 0.5412 -1.0629 -2.8311 -4.7556
-2.4693 0.9315 -421.2334
87.3600 -423.8425 0.4941 2.5409
3.4135 0.9110 -0.9884 -2.8276 -4.7595
-2.4959 0.9209 -421.2263
87.3800 -424.3292 0.8963 2.0012
2.9492 1.1949 -0.9056 -2.8198 -4.7617
-2.5222 0.9102 -421.2191
87.4000 -427.4933 -0.5006 0.5004
1.9318 1.3880 -0.8160 -2.8079 -4.7624
-2.5483 0.8996 -421.2119
87.4200 -430.3790 -0.3663 -1.0602
0.6357 1.4939 -0.7207 -2.7920 -4.7613
-2.5742 0.8890 -421.2048
87.4400 -431.5148 -0.0146 -1.8419 -
0.6457 1.5186 -0.6209 -2.7724 -4.7587
-2.5998 0.8783 -421.1976
87.4600 -432.3499 0.2334 -1.6391 -
1.6581 1.4694 -0.5177 -2.7491 -4.7544
-2.6252 0.8677 -421.1905

87.4800 -432.6994 0.6466 -0.9941 -
2.2402 1.3556 -0.4125 -2.7223 -4.7486
-2.6503 0.8570 -421.1834
87.5000 -431.9387 -1.0216 0.0163 -
2.3830 1.1884 -0.3065 -2.6923 -4.7411
-2.6752 0.8463 -421.1762
87.5200 -430.4098 -0.0848 1.1155 -
2.1375 0.9811 -0.2009 -2.6592 -4.7321
-2.6998 0.8357 -421.1691
87.5400 -429.5041 0.7899 1.3365 -
1.5698 0.7474 -0.0967 -2.6232 -4.7216
-2.7242 0.8250 -421.1620
87.5600 -430.0640 -0.2200 0.2659 -
0.7871 0.5005 0.0051 -2.5845 -4.7095
-2.7483 0.8144 -421.1548
87.5800 -431.2051 -0.0689 -1.1980
0.0558 0.2530 0.1037 -2.5434 -4.6959
-2.7722 0.8037 -421.1477
87.6000 -431.7970 -1.0422 -1.3858
0.7839 0.0168 0.1985 -2.4999 -4.6809
-2.7958 0.7930 -421.1406
87.6200 -429.5794 -0.5170 0.2279
1.2702 -0.1978 0.2888 -2.4544 -4.6643
-2.8191 0.7824 -421.1335
87.6400 -425.7807 0.3268 2.1121
1.4573 -0.3846 0.3738 -2.4069 -4.6463
-2.8422 0.7717 -421.1264
87.6600 -424.1602 2.5253 2.2106
1.3548 -0.5417 0.4532 -2.3578 -4.6269
-2.8651 0.7610 -421.1192
87.6800 -428.0122 0.5746 0.1158
1.0480 -0.6676 0.5263 -2.3071 -4.6061
-2.8876 0.7504 -421.1121
87.7000 -433.6243 -1.8627 -2.2628
0.6673 -0.7627 0.5927 -2.2552 -4.5839
-2.9099 0.7397 -421.1050
87.7200 -435.1435 -2.3151 -2.8019
0.3324 -0.8286 0.6520 -2.2020 -4.5604
-2.9320 0.7290 -421.0979
87.7400 -431.5431 -0.7084 -0.9303
0.0968 -0.8652 0.7040 -2.1480 -4.5355
-2.9538 0.7184 -421.0908
87.7600 -427.0832 1.6760 1.6956 -
0.0909 -0.8695 0.7485 -2.0931 -4.5092
-2.9753 0.7077 -421.0837
87.7800 -426.0358 1.0835 2.8414 -
0.3153 -0.8380 0.7855 -2.0376 -4.4817
-2.9965 0.6970 -421.0766
87.8000 -428.8196 -0.1311 1.6537 -
0.6197 -0.7675 0.8149 -1.9815 -4.4530
-3.0175 0.6864 -421.0696

87.8200 -431.9787 -0.7885 -0.4901 -
0.9639 -0.6554 0.8366 -1.9252 -4.4229
-3.0382 0.6757 -421.0625
87.8400 -432.4813 -0.5466 -1.7086 -
1.2529 -0.5010 0.8508 -1.8686 -4.3917
-3.0587 0.6651 -421.0554
87.8600 -431.6383 0.2972 -1.5779 -
1.3756 -0.3072 0.8574 -1.8118 -4.3592
-3.0789 0.6544 -421.0483
87.8800 -430.4618 0.4411 -0.7277 -
1.2619 -0.0808 0.8564 -1.7550 -4.3256
-3.0988 0.6438 -421.0413
87.9000 -429.4255 -0.0537 0.1134 -
0.9142 0.1677 0.8476 -1.6983 -4.2908
-3.1185 0.6331 -421.0342
87.9200 -428.3343 0.0397 0.5118 -
0.3878 0.4245 0.8309 -1.6416 -4.2549
-3.1379 0.6225 -421.0271
87.9400 -427.3353 -0.1939 0.5066
0.2285 0.6737 0.8062 -1.5852 -4.2179
-3.1570 0.6118 -421.0201
87.9600 -426.5998 0.0605 0.2528
0.8329 0.8992 0.7733 -1.5290 -4.1799
-3.1758 0.6012 -421.0130
87.9800 -425.9678 0.1806 -0.0705
1.3258 1.0847 0.7322 -1.4731 -4.1408
-3.1944 0.5906 -421.0059
88.0000 -425.5480 0.0753 -0.0344
1.6106 1.2148 0.6830 -1.4176 -4.1007
-3.2127 0.5799 -420.9989
88.0200 -425.3673 -0.0421 0.4048
1.6106 1.2771 0.6259 -1.3625 -4.0596
-3.2307 0.5693 -420.9918
88.0400 -425.2440 -0.1858 0.9134
1.2859 1.2655 0.5614 -1.3079 -4.0175
-3.2485 0.5587 -420.9848
88.0600 -425.2059 0.2724 0.9094
0.6629 1.1827 0.4900 -1.2537 -3.9746
-3.2660 0.5481 -420.9778
88.0800 -426.8630 0.4873 0.1734 -
0.1259 1.0419 0.4127 -1.1999 -3.9307
-3.2832 0.5375 -420.9707
88.1000 -429.4683 -0.4702 -0.7449 -
0.8756 0.8585 0.3306 -1.1465 -3.8860
-3.3002 0.5269 -420.9637
88.1200 -430.2951 0.0124 -1.1084 -
1.3864 0.6478 0.2448 -1.0935 -3.8404
-3.3168 0.5163 -420.9567
88.1400 -430.2103 0.3024 -1.0075 -
1.5333 0.4255 0.1566 -1.0409 -3.7941
-3.3333 0.5057 -420.9496

88.1600 -430.0360 0.2792 -0.6961 -
1.3349 0.2056 0.0674 -0.9887 -3.7469
-3.3494 0.4951 -420.9426
88.1800 -429.6338 -0.2410 0.0729 -
0.9173 -0.0004 -0.0218 -0.9368 -3.6991
-3.3652 0.4845 -420.9356
88.2000 -428.1285 -0.1128 1.2295 -
0.4453 -0.1833 -0.1094 -0.8853 -3.6505
-3.3808 0.4739 -420.9286
88.2200 -426.6483 0.5953 1.8328 -
0.0354 -0.3372 -0.1942 -0.8341 -3.6013
-3.3961 0.4634 -420.9215
88.2400 -426.8973 0.5950 1.0179
0.2673 -0.4628 -0.2747 -0.7832 -3.5514
-3.4112 0.4528 -420.9145
88.2600 -429.5769 -0.5152 -0.8017
0.4757 -0.5660 -0.3493 -0.7326 -3.5009
-3.4260 0.4423 -420.9075
88.2800 -431.9237 -0.6798 -2.0609
0.6368 -0.6556 -0.4165 -0.6822 -3.4498
-3.4404 0.4317 -420.9005
88.3000 -431.1825 -0.8008 -1.6835
0.7820 -0.7396 -0.4748 -0.6319 -3.3981
-3.4547 0.4212 -420.8935
88.3200 -428.1549 0.4733 -0.0196
0.8766 -0.8212 -0.5231 -0.5819 -3.3460
-3.4686 0.4107 -420.8865
88.3400 -425.7574 0.7039 1.6466
0.8510 -0.9024 -0.5606 -0.5319 -3.2933
-3.4823 0.4002 -420.8795
88.3600 -426.0414 0.0443 2.2390
0.6398 -0.9852 -0.5866 -0.4820 -3.2402
-3.4956 0.3897 -420.8726
88.3800 -427.5775 -0.1442 1.6176
0.2578 -1.0686 -0.6010 -0.4320 -3.1867
-3.5088 0.3792 -420.8656
88.4000 -429.2212 0.4206 0.2021 -
0.2117 -1.1459 -0.6035 -0.3820 -3.1327
-3.5216 0.3687 -420.8586
88.4200 -430.7387 -0.0711 -1.3874 -
0.6264 -1.2072 -0.5946 -0.3319 -3.0784
-3.5342 0.3582 -420.8516
88.4400 -432.2468 -0.2343 -2.5020 -
0.8313 -1.2417 -0.5749 -0.2817 -3.0238
-3.5464 0.3477 -420.8446
88.4600 -432.8967 -0.9943 -2.5121 -
0.7450 -1.2407 -0.5449 -0.2313 -2.9689
-3.5584 0.3373 -420.8377
88.4800 -430.6413 -0.2406 -0.9324 -
0.4193 -1.1992 -0.5057 -0.1808 -2.9137
-3.5702 0.3268 -420.8307

88.5000 -426.7399 0.5161 1.2732 -
0.0078 -1.1164 -0.4583 -0.1301 -2.8582
-3.5816 0.3164 -420.8237
88.5200 -424.9060 0.3033 2.4044
0.3188 -0.9927 -0.4040 -0.0791 -2.8026
-3.5928 0.3060 -420.8168
88.5400 -425.7261 -0.1281 1.8856
0.4264 -0.8296 -0.3444 -0.0279 -2.7467
-3.6037 0.2956 -420.8098
88.5600 -427.0793 -0.0592 0.4894
0.2978 -0.6306 -0.2809 0.0236 -2.6907
-3.6143 0.2852 -420.8029
88.5800 -427.8091 -0.4376 -0.6463
0.0087 -0.4008 -0.2149 0.0755 -2.6346
-3.6246 0.2748 -420.7959
88.6000 -428.1541 0.1236 -1.0447 -
0.3180 -0.1453 -0.1481 0.1278 -2.5783
-3.6346 0.2644 -420.7890
88.6200 -428.3668 -0.0324 -0.8944 -
0.5556 0.1303 -0.0817 0.1805 -2.5220
-3.6444 0.2540 -420.7820
88.6400 -428.2164 -0.2996 -0.7294 -
0.6310 0.4203 -0.0174 0.2336 -2.4657
-3.6539 0.2437 -420.7751
88.6600 -426.9635 -0.0966 -0.5439 -
0.5306 0.7183 0.0436 0.2872 -2.4094
-3.6631 0.2333 -420.7681
88.6800 -425.3779 0.2372 -0.0525 -
0.3016 1.0173 0.0998 0.3413 -2.3530
-3.6720 0.2230 -420.7612
88.7000 -424.1583 -0.6678 0.4699 -
0.0424 1.3090 0.1502 0.3960 -2.2968
-3.6807 0.2127 -420.7543
88.7200 -422.9434 0.4080 0.6402
0.1536 1.5829 0.1936 0.4512 -2.2406
-3.6890 0.2024 -420.7474
88.7400 -422.3056 0.2792 0.3560
0.2284 1.8272 0.2293 0.5070 -2.1845
-3.6971 0.1921 -420.7404
88.7600 -422.8840 0.3961 -0.1984
0.1944 2.0283 0.2566 0.5635 -2.1285
-3.7049 0.1818 -420.7335
88.7800 -424.0240 -0.2963 -0.5773
0.1344 2.1713 0.2754 0.6206 -2.0727
-3.7124 0.1716 -420.7266
88.8000 -424.2944 -0.3545 -0.4486
0.1335 2.2425 0.2854 0.6784 -2.0171
-3.7196 0.1613 -420.7197
88.8200 -422.9175 -0.2643 0.1884
0.2381 2.2312 0.2867 0.7368 -1.9617
-3.7266 0.1511 -420.7128

88.8400 -421.4648 0.4546 0.6700
0.4572 2.1306 0.2795 0.7959 -1.9066
-3.7332 0.1409 -420.7059
88.8600 -421.6821 0.6638 0.2973
0.7580 1.9384 0.2642 0.8556 -1.8517
-3.7396 0.1307 -420.6990
88.8800 -423.0850 -0.3012 -0.5938
1.0410 1.6564 0.2414 0.9159 -1.7971
-3.7457 0.1205 -420.6921
88.9000 -423.8988 -0.8528 -0.9138
1.1830 1.2906 0.2120 0.9767 -1.7428
-3.7515 0.1103 -420.6852
88.9200 -423.0169 0.2361 0.0162
1.0926 0.8524 0.1769 1.0380 -1.6889
-3.7570 0.1002 -420.6783
88.9400 -421.6481 0.5687 1.4110
0.7328 0.3590 0.1370 1.0997 -1.6354
-3.7623 0.0900 -420.6714
88.9600 -421.7853 1.1836 1.6349
0.1444 -0.1672 0.0931 1.1617 -1.5823
-3.7672 0.0799 -420.6645
88.9800 -425.6696 -0.6035 0.1476 -
0.5462 -0.7006 0.0463 1.2238 -1.5296
-3.7719 0.0698 -420.6576
89.0000 -429.1894 -0.7711 -1.5149 -
1.1592 -1.2150 -0.0027 1.2861 -1.4773
-3.7762 0.0597 -420.6508
89.0200 -429.3209 0.3958 -2.0448 -
1.5137 -1.6838 -0.0529 1.3483 -1.4255
-3.7803 0.0496 -420.6439
89.0400 -429.0108 0.3539 -1.6126 -
1.5309 -2.0814 -0.1034 1.4104 -1.3743
-3.7841 0.0396 -420.6370
89.0600 -428.5484 -0.3918 -0.6055 -
1.2610 -2.3860 -0.1533 1.4723 -1.3236
-3.7876 0.0295 -420.6302
89.0800 -427.3502 -0.0598 0.6857 -
0.8494 -2.5825 -0.2017 1.5338 -1.2734
-3.7908 0.0195 -420.6233
89.1000 -425.4891 -0.2494 1.7675 -
0.4897 -2.6595 -0.2480 1.5948 -1.2238
-3.7937 0.0095 -420.6164
89.1200 -424.6034 0.4689 2.0562 -
0.3413 -2.6094 -0.2918 1.6552 -1.1748
-3.7964 -0.0005 -420.6096
89.1400 -425.4228 0.0884 1.5298 -
0.4674 -2.4312 -0.3329 1.7148 -1.1265
-3.7987 -0.0105 -420.6027
89.1600 -426.7643 -0.6633 0.4198 -
0.8025 -2.1321 -0.3711 1.7737 -1.0788
-3.8007 -0.0204 -420.5959

89.1800 -427.3710 0.0111 -0.8018 -
1.1610 -1.7260 -0.4065 1.8315 -1.0318
-3.8025 -0.0304 -420.5890
89.2000 -427.5706 0.9180 -1.8804 -
1.3286 -1.2324 -0.4390 1.8883 -0.9854
-3.8040 -0.0403 -420.5822
89.2200 -427.6841 -0.0021 -2.2881 -
1.1578 -0.6755 -0.4687 1.9440 -0.9398
-3.8051 -0.0502 -420.5754
89.2400 -426.9731 -1.0984 -1.3212 -
0.6487 -0.0841 -0.4957 1.9983 -0.8950
-3.8060 -0.0601 -420.5685
89.2600 -422.7950 -0.0660 0.6249
0.0545 0.5116 -0.5200 2.0513 -0.8508
-3.8066 -0.0700 -420.5617
89.2800 -419.0268 0.7084 2.0218
0.7412 1.0814 -0.5417 2.1028 -0.8075
-3.8069 -0.0798 -420.5549
89.3000 -418.8631 0.2527 1.9647
1.2152 1.5949 -0.5609 2.1527 -0.7650
-3.8069 -0.0897 -420.5480
89.3200 -419.4722 -0.5147 0.8676
1.3707 2.0233 -0.5775 2.2009 -0.7233
-3.8066 -0.0995 -420.5412
89.3400 -420.1028 -0.1218 -0.3260
1.2431 2.3419 -0.5916 2.2473 -0.6824
-3.8060 -0.1093 -420.5344
89.3600 -420.8110 0.0210 -1.0037
0.9723 2.5337 -0.6033 2.2918 -0.6424
-3.8051 -0.1191 -420.5276
89.3800 -421.3509 -0.5259 -1.1533
0.7286 2.5875 -0.6126 2.3343 -0.6033
-3.8039 -0.1289 -420.5208
89.4000 -421.1246 0.2040 -0.9405
0.6454 2.4997 -0.6192 2.3746 -0.5651
-3.8024 -0.1386 -420.5140
89.4200 -420.1937 0.1496 -0.3130
0.7591 2.2747 -0.6230 2.4127 -0.5277
-3.8006 -0.1483 -420.5072
89.4400 -419.7029 0.6369 0.4605
0.9883 1.9288 -0.6232 2.4482 -0.4914
-3.7986 -0.1581 -420.5004
89.4600 -420.0848 -0.8709 0.7036
1.1840 1.4869 -0.6195 2.4813 -0.4559
-3.7962 -0.1678 -420.4936
89.4800 -420.7520 0.0139 0.4033
1.1924 0.9797 -0.6113 2.5115 -0.4214
-3.7935 -0.1774 -420.4868
89.5000 -421.2436 -0.6380 0.4239
0.9213 0.4383 -0.5981 2.5389 -0.3879
-3.7905 -0.1871 -420.4800

89.5200 -421.6254 0.4585 0.8677
0.3617 -0.1077 -0.5794 2.5632 -0.3554
-3.7873 -0.1967 -420.4732
89.5400 -422.2023 0.5418 0.8678 -
0.3906 -0.6309 -0.5548 2.5844 -0.3239
-3.7837 -0.2063 -420.4664
89.5600 -424.4212 -0.1563 0.1857 -
1.1881 -1.1041 -0.5236 2.6023 -0.2934
-3.7798 -0.2159 -420.4597
89.5800 -427.3493 -0.7290 -0.6889 -
1.8557 -1.5018 -0.4856 2.6168 -0.2639
-3.7757 -0.2255 -420.4529
89.6000 -428.0862 -0.1714 -1.3728 -
2.2058 -1.8017 -0.4403 2.6276 -0.2355
-3.7712 -0.2351 -420.4461
89.6200 -427.9248 0.4301 -1.6857 -
2.0957 -1.9874 -0.3873 2.6349 -0.2080
-3.7664 -0.2446 -420.4394
89.6400 -427.6029 -0.1382 -1.5066 -
1.5068 -2.0545 -0.3267 2.6383 -0.1817
-3.7613 -0.2542 -420.4326
89.6600 -426.4823 -1.1555 -0.5640 -
0.5504 -2.0106 -0.2585 2.6378 -0.1563
-3.7559 -0.2637 -420.4258
89.6800 -422.5647 -0.1307 0.9328
0.5288 -1.8682 -0.1833 2.6334 -0.1321
-3.7503 -0.2731 -420.4191
89.7000 -419.3825 0.8495 1.8882
1.4338 -1.6411 -0.1016 2.6250 -0.1088
-3.7443 -0.2826 -420.4123
89.7200 -419.6728 0.1129 1.4970
1.9037 -1.3436 -0.0143 2.6124 -0.0866
-3.7380 -0.2921 -420.4056
89.7400 -421.0001 -0.4128 0.4298
1.8096 -0.9924 0.0777 2.5957 -0.0655
-3.7314 -0.3015 -420.3988
89.7600 -421.7305 -0.2322 -0.0557
1.1705 -0.6059 0.1733 2.5748 -0.0454
-3.7245 -0.3109 -420.3921
89.7800 -421.8433 0.1849 0.2163
0.1673 -0.2043 0.2714 2.5497 -0.0263
-3.7173 -0.3203 -420.3854
89.8000 -421.9320 0.0320 0.4788 -
0.8910 0.1903 0.3709 2.5203 -0.0083
-3.7098 -0.3296 -420.3786
89.8200 -422.2927 0.2166 -0.0091 -
1.6770 0.5561 0.4707 2.4866 0.0087
-3.7020 -0.3390 -420.3719
89.8400 -423.2178 0.1532 -1.0283 -
1.9617 0.8741 0.5697 2.4487 0.0247
-3.6939 -0.3483 -420.3652

89.8600 -423.7983 -0.2049 -1.7150 -
1.6657 1.1313 0.6664 2.4066 0.0397
-3.6855 -0.3576 -420.3585
89.8800 -422.7316 0.0436 -1.5173 -
0.8720 1.3213 0.7595 2.3601 0.0537
-3.6768 -0.3669 -420.3517
89.9000 -420.3379 0.0837 -0.6618
0.2128 1.4426 0.8475 2.3094 0.0668
-3.6678 -0.3762 -420.3450
89.9200 -418.0894 -0.1546 0.4486
1.3269 1.4950 0.9289 2.2545 0.0788
-3.6585 -0.3854 -420.3383
89.9400 -415.5453 0.0094 1.5233
2.1829 1.4795 1.0022 2.1954 0.0900
-3.6489 -0.3947 -420.3316
89.9600 -413.9402 0.5707 2.0199
2.5354 1.3979 1.0657 2.1321 0.1002
-3.6390 -0.4039 -420.3249
89.9800 -415.5260 0.4976 1.4266
2.2796 1.2568 1.1182 2.0646 0.1095
-3.6288 -0.4131 -420.3182
90.0000 -419.2578 -0.5694 -0.0271
1.5111 1.0690 1.1586 1.9932 0.1179
-3.6184 -0.4222 -420.3115
90.0200 -421.2615 -0.1491 -1.2532
0.4478 0.8504 1.1861 1.9178 0.1254
-3.6076 -0.4314 -420.3048
90.0400 -422.3522 -0.0851 -1.4187 -
0.6640 0.6181 1.1997 1.8386 0.1320
-3.5966 -0.4405 -420.2981
90.0600 -422.9708 0.3866 -0.6648 -
1.6083 0.3890 1.1985 1.7558 0.1378
-3.5852 -0.4496 -420.2914
90.0800 -423.2098 0.1650 0.0840 -
2.2224 0.1799 1.1818 1.6695 0.1428
-3.5736 -0.4587 -420.2847
90.1000 -423.3622 -0.1013 0.3500 -
2.4329 0.0055 1.1489 1.5798 0.1470
-3.5617 -0.4678 -420.2781
90.1200 -423.3896 -0.2297 0.3085 -
2.2458 -0.1234 1.0994 1.4870 0.1504
-3.5495 -0.4768 -420.2714
90.1400 -423.2175 0.0160 0.2072 -
1.7137 -0.2015 1.0334 1.3914 0.1530
-3.5370 -0.4858 -420.2647
90.1600 -423.0258 0.0101 -0.0285 -
0.9254 -0.2292 0.9512 1.2931 0.1548
-3.5243 -0.4948 -420.2580
90.1800 -423.0494 -0.0103 -0.7585
0.0147 -0.2103 0.8537 1.1925 0.1560
-3.5112 -0.5038 -420.2514

90.2000 -423.2557 0.0317 -1.5687
0.9594 -0.1501 0.7424 1.0898 0.1564
-3.4980 -0.5128 -420.2447
90.2200 -423.3988 -1.1372 -1.2303
1.7200 -0.0540 0.6188 0.9853 0.1560
-3.4844 -0.5217 -420.2381
90.2400 -420.8349 -1.3980 0.8904
2.1203 0.0720 0.4849 0.8794 0.1551
-3.4705 -0.5307 -420.2314
90.2600 -415.9370 1.3158 3.1331
2.0323 0.2195 0.3427 0.7723 0.1534
-3.4564 -0.5396 -420.2248
90.2800 -415.0328 2.5697 3.1869
1.4048 0.3761 0.1946 0.6643 0.1511
-3.4421 -0.5484 -420.2181
90.3000 -422.1482 0.2566 0.4850
0.3750 0.5274 0.0431 0.5558 0.1482
-3.4274 -0.5573 -420.2115
90.3200 -428.6353 -2.5901 -2.6473 -
0.7248 0.6580 -0.1092 0.4470 0.1446
-3.4125 -0.5661 -420.2048
90.3400 -428.4674 -0.2683 -3.5842 -
1.5335 0.7526 -0.2601 0.3382 0.1405
-3.3974 -0.5750 -420.1982
90.3600 -427.1575 0.4033 -2.3170 -
1.7781 0.7983 -0.4071 0.2298 0.1358
-3.3820 -0.5838 -420.1916
90.3800 -425.7788 0.3149 -0.6256 -
1.3728 0.7862 -0.5482 0.1219 0.1305
-3.3663 -0.5925 -420.1849
90.4000 -424.5808 -0.3974 0.0692 -
0.4170 0.7117 -0.6814 0.0150 0.1247
-3.3504 -0.6013 -420.1783
90.4200 -423.3703 0.0866 0.2680
0.7871 0.5733 -0.8049 -0.0906 0.1184
-3.3342 -0.6100 -420.1717
90.4400 -422.2573 -0.2250 1.0137
1.8445 0.3713 -0.9173 -0.1948 0.1116
-3.3178 -0.6187 -420.1651
90.4600 -421.0290 -0.5412 2.4402
2.4213 0.1074 -1.0172 -0.2971 0.1042
-3.3012 -0.6274 -420.1584
90.4800 -420.1176 0.1477 3.3110
2.3368 -0.2130 -1.1036 -0.3974 0.0965
-3.2843 -0.6361 -420.1518
90.5000 -421.4060 1.0674 2.1594
1.6163 -0.5748 -1.1755 -0.4953 0.0883
-3.2671 -0.6448 -420.1452
90.5200 -427.5137 -0.7994 -0.4413
0.5158 -0.9556 -1.2322 -0.5905 0.0796
-3.2498 -0.6534 -420.1386

90.5400 -431.3000 -0.8448 -2.3514 -
0.6320 -1.3318 -1.2729 -0.6829 0.0705
-3.2322 -0.6620 -420.1320
90.5600 -431.1046 0.1439 -2.3906 -
1.5214 -1.6791 -1.2969 -0.7721 0.0611
-3.2143 -0.6706 -420.1254
90.5800 -430.6694 0.3900 -1.0340 -
1.9889 -1.9734 -1.3033 -0.8579 0.0513
-3.1963 -0.6791 -420.1188
90.6000 -430.2096 0.2863 0.3865 -
2.0211 -2.1917 -1.2915 -0.9402 0.0411
-3.1780 -0.6877 -420.1122
90.6200 -429.7814 -0.4479 0.8737 -
1.7223 -2.3162 -1.2608 -1.0188 0.0306
-3.1595 -0.6962 -420.1057
90.6400 -429.3257 -0.2274 0.6727 -
1.2571 -2.3358 -1.2111 -1.0936 0.0198
-3.1407 -0.7047 -420.0991
90.6600 -428.9046 0.0878 0.5294 -
0.8036 -2.2444 -1.1425 -1.1645 0.0087
-3.1218 -0.7132 -420.0925
90.6800 -428.5677 0.1887 0.4899 -
0.4880 -2.0394 -1.0566 -1.2315 -0.0026
-3.1026 -0.7217 -420.0859
90.7000 -428.2582 -0.0187 0.3104 -
0.3558 -1.7263 -0.9556 -1.2946 -0.0143
-3.0832 -0.7301 -420.0794
90.7200 -427.8772 -0.1907 0.0394 -
0.3801 -1.3195 -0.8420 -1.3537 -0.0261
-3.0637 -0.7385 -420.0728
90.7400 -427.3197 -0.3202 -0.2393 -
0.4930 -0.8384 -0.7185 -1.4088 -0.0382
-3.0439 -0.7469 -420.0662
90.7600 -426.6479 0.0049 -0.2662 -
0.5872 -0.3074 -0.5879 -1.4600 -0.0504
-3.0239 -0.7553 -420.0597
90.7800 -426.0016 0.7263 -0.1079 -
0.5542 0.2460 -0.4530 -1.5073 -0.0629
-3.0037 -0.7637 -420.0531
90.8000 -425.4738 -0.2295 -0.2367 -
0.3189 0.7944 -0.3166 -1.5507 -0.0755
-2.9833 -0.7720 -420.0466
90.8200 -424.9024 -0.0765 -0.5110
0.1110 1.3126 -0.1814 -1.5901 -0.0882
-2.9627 -0.7803 -420.0400
90.8400 -423.9592 -0.4258 -0.4080
0.6574 1.7772 -0.0501 -1.6258 -0.1010
-2.9420 -0.7886 -420.0335
90.8600 -421.8431 -0.0313 0.1575
1.1794 2.1666 0.0744 -1.6576 -0.1140
-2.9210 -0.7969 -420.0269

90.8800 -420.0109 0.4452 0.7935
1.5216 2.4607 0.1896 -1.6856 -0.1270
-2.8999 -0.8051 -420.0204
90.9000 -419.9497 0.1037 1.1896
1.5601 2.6429 0.2932 -1.7099 -0.1401
-2.8786 -0.8133 -420.0138
90.9200 -420.6532 -0.1557 1.0449
1.2706 2.7057 0.3834 -1.7304 -0.1532
-2.8571 -0.8215 -420.0073
90.9400 -421.5455 0.2433 0.2879
0.7464 2.6526 0.4592 -1.7474 -0.1664
-2.8354 -0.8297 -420.0008
90.9600 -422.8812 -0.0395 -0.5549
0.1531 2.4952 0.5204 -1.7608 -0.1795
-2.8136 -0.8379 -419.9943
90.9800 -424.2575 -0.2329 -1.0520 -
0.3324 2.2491 0.5670 -1.7706 -0.1926
-2.7916 -0.8460 -419.9877
91.0000 -424.6553 0.2990 -1.2697 -
0.5751 1.9320 0.5997 -1.7771 -0.2057
-2.7694 -0.8542 -419.9812
91.0200 -424.7514 0.5221 -1.2877 -
0.5291 1.5617 0.6192 -1.7802 -0.2188
-2.7471 -0.8622 -419.9747
91.0400 -424.7971 -0.4685 -0.7375 -
0.2618 1.1555 0.6267 -1.7800 -0.2317
-2.7246 -0.8703 -419.9682
91.0600 -424.2896 -0.8899 0.6273
0.0691 0.7302 0.6231 -1.7768 -0.2446
-2.7020 -0.8784 -419.9617
91.0800 -422.5960 0.0469 2.0244
0.3066 0.3021 0.6097 -1.7705 -0.2573
-2.6792 -0.8864 -419.9552
91.1000 -421.5042 1.2246 2.1213
0.3385 -0.1151 0.5878 -1.7613 -0.2699
-2.6563 -0.8944 -419.9487
91.1200 -423.9150 1.2230 0.5043
0.1553 -0.5127 0.5593 -1.7493 -0.2823
-2.6332 -0.9024 -419.9422
91.1400 -428.4814 -1.5318 -1.5141 -
0.1367 -0.8852 0.5259 -1.7347 -0.2945
-2.6100 -0.9104 -419.9357
91.1600 -430.1117 -1.0497 -2.1611 -
0.3955 -1.2271 0.4897 -1.7177 -0.3066
-2.5866 -0.9183 -419.9292
91.1800 -428.2207 0.2014 -0.9556 -
0.5170 -1.5323 0.4525 -1.6983 -0.3184
-2.5631 -0.9263 -419.9227
91.2000 -425.7764 0.2394 0.8471 -
0.4900 -1.7922 0.4160 -1.6767 -0.3299
-2.5395 -0.9342 -419.9163

91.2200 -425.2378 0.5146 1.4806 -
0.3556 -1.9978 0.3814 -1.6531 -0.3412
-2.5158 -0.9421 -419.9098
91.2400 -426.6882 0.1372 0.6565 -
0.1648 -2.1413 0.3499 -1.6276 -0.3522
-2.4919 -0.9499 -419.9033
91.2600 -428.2970 -0.5922 -0.4813
0.0181 -2.2126 0.3224 -1.6004 -0.3629
-2.4679 -0.9578 -419.8968
91.2800 -428.4042 0.1645 -0.9199
0.1261 -2.2001 0.2995 -1.5716 -0.3732
-2.4438 -0.9656 -419.8904
91.3000 -427.2116 -0.3591 -0.3898
0.0925 -2.0944 0.2813 -1.5414 -0.3832
-2.4195 -0.9734 -419.8839
91.3200 -426.0041 0.2805 0.6699 -
0.1468 -1.8914 0.2676 -1.5099 -0.3928
-2.3952 -0.9811 -419.8774
91.3400 -425.6944 -0.1389 1.5171 -
0.6002 -1.5960 0.2581 -1.4773 -0.4020
-2.3708 -0.9889 -419.8710
91.3600 -425.5632 -0.2380 1.7964 -
1.1853 -1.2223 0.2526 -1.4437 -0.4108
-2.3462 -0.9966 -419.8645
91.3800 -425.5024 0.1695 1.2979 -
1.7291 -0.7919 0.2505 -1.4093 -0.4192
-2.3216 -1.0043 -419.8581
91.4000 -426.5135 1.7192 -0.4419 -
2.0289 -0.3301 0.2513 -1.3740 -0.4271
-2.2968 -1.0120 -419.8517
91.4200 -429.3034 0.0396 -2.7283 -
1.9094 0.1370 0.2545 -1.3380 -0.4346
-2.2720 -1.0197 -419.8452
91.4400 -430.9973 -1.7536 -3.6431 -
1.2688 0.5830 0.2597 -1.3015 -0.4415
-2.2470 -1.0273 -419.8388
91.4600 -427.1847 -1.3241 -1.8595 -
0.1509 0.9827 0.2662 -1.2645 -0.4480
-2.2220 -1.0350 -419.8323
91.4800 -419.7453 1.2454 1.4378
1.1554 1.3111 0.2736 -1.2270 -0.4540
-2.1969 -1.0426 -419.8259
91.5000 -415.8004 0.9122 3.5372
2.2872 1.5438 0.2811 -1.1891 -0.4594
-2.1717 -1.0501 -419.8195
91.5200 -416.9596 -0.1686 3.0253
2.9248 1.6573 0.2882 -1.1508 -0.4643
-2.1465 -1.0577 -419.8131
91.5400 -419.1901 -0.9275 1.0165
2.9393 1.6354 0.2940 -1.1123 -0.4687
-2.1212 -1.0652 -419.8066

91.5600 -420.9817 0.6539 -0.6539
2.3687 1.4840 0.2979 -1.0735 -0.4726
-2.0958 -1.0728 -419.8002
91.5800 -422.5800 0.2260 -1.1244
1.3604 1.2272 0.2996 -1.0344 -0.4759
-2.0703 -1.0803 -419.7938
91.6000 -423.9790 -0.2928 -0.7209
0.1416 0.8958 0.2987 -0.9950 -0.4786
-2.0448 -1.0877 -419.7874
91.6200 -425.1733 -0.2463 -0.2368 -
1.0200 0.5228 0.2949 -0.9553 -0.4808
-2.0192 -1.0952 -419.7810
91.6400 -426.2147 -0.0297 -0.1911 -
1.8922 0.1423 0.2875 -0.9154 -0.4824
-1.9935 -1.1026 -419.7746
91.6600 -426.8771 0.2440 -0.5453 -
2.3515 -0.2129 0.2762 -0.8752 -0.4835
-1.9678 -1.1100 -419.7682
91.6800 -427.3222 -0.0626 -1.0480 -
2.3664 -0.5146 0.2606 -0.8348 -0.4840
-1.9421 -1.1174 -419.7618
91.7000 -427.6284 -0.5736 -1.3342 -
1.9921 -0.7430 0.2404 -0.7941 -0.4839
-1.9163 -1.1248 -419.7554
91.7200 -427.5125 -0.6571 -0.9336 -
1.3637 -0.8887 0.2155 -0.7532 -0.4833
-1.8905 -1.1321 -419.7490
91.7400 -425.2236 0.1517 0.2629 -
0.6524 -0.9481 0.1856 -0.7120 -0.4821
-1.8646 -1.1394 -419.7427
91.7600 -422.6138 0.8684 1.4559 -
0.0273 -0.9224 0.1508 -0.6707 -0.4803
-1.8387 -1.1467 -419.7363
91.7800 -422.2951 0.1407 1.6075
0.3817 -0.8163 0.1113 -0.6292 -0.4779
-1.8128 -1.1540 -419.7299
91.8000 -423.4890 -0.7173 0.7125
0.5307 -0.6411 0.0676 -0.5876 -0.4750
-1.7868 -1.1612 -419.7235
91.8200 -424.5339 -0.1266 -0.2661
0.4872 -0.4153 0.0203 -0.5458 -0.4714
-1.7608 -1.1685 -419.7172
91.8400 -424.5679 0.1584 -0.8373
0.3688 -0.1621 -0.0299 -0.5040 -0.4673
-1.7348 -1.1757 -419.7108
91.8600 -424.4306 0.1923 -1.2171
0.3049 0.0945 -0.0824 -0.4621 -0.4627
-1.7087 -1.1829 -419.7044
91.8800 -424.2033 -0.4455 -1.0242
0.3553 0.3326 -0.1363 -0.4203 -0.4575
-1.6827 -1.1900 -419.6981

91.9000	-422.8201	-0.3774	0.0417	
0.5080	0.5350	-0.1910	-0.3786	-0.4517
	-1.6566	-1.1972	-419.6917	
91.9200	-420.6562	0.5501	1.0931	
0.7055	0.6899	-0.2458	-0.3369	-0.4453
	-1.6305	-1.2043	-419.6854	
91.9400	-419.9565	0.8400	1.2560	
0.8634	0.7883	-0.2999	-0.2955	-0.4384
	-1.6044	-1.2114	-419.6790	
91.9600	-420.9661	-0.9613	0.5027	
0.9256	0.8253	-0.3527	-0.2543	-0.4309
	-1.5783	-1.2185	-419.6727	
91.9800	-422.1286	-0.3841	-0.4545	
0.8956	0.8002	-0.4035	-0.2134	-0.4228
	-1.5522	-1.2256	-419.6663	
92.0000	-422.4196	0.7004	-0.9111	
0.8139	0.7137	-0.4514	-0.1729	-0.4142
	-1.5261	-1.2326	-419.6600	
92.0200	-422.5506	0.1082	-0.6900	
0.7276	0.5693	-0.4954	-0.1328	-0.4050
	-1.5000	-1.2396	-419.6537	
92.0400	-422.6000	0.0889	-0.1400	
0.6635	0.3764	-0.5344	-0.0932	-0.3953
	-1.4738	-1.2466	-419.6474	
92.0600	-422.5698	-0.1049	0.3005	
0.6041	0.1487	-0.5673	-0.0542	-0.3850
	-1.4477	-1.2536	-419.6410	
92.0800	-422.5172	-0.6963	0.5666	
0.4905	-0.0979	-0.5930	-0.0159	-0.3742
	-1.4217	-1.2605	-419.6347	
92.1000	-422.5758	0.5615	0.6652	
0.2650	-0.3471	-0.6108	0.0218	-0.3629
	-1.3956	-1.2675	-419.6284	
92.1200	-423.6174	-0.1812	0.3411	-
0.0824	-0.5819	-0.6196	0.0586	-0.3510
	-1.3695	-1.2744	-419.6221	
92.1400	-424.8498	-0.1906	-0.1599	-
0.5202	-0.7828	-0.6186	0.0946	-0.3386
	-1.3435	-1.2812	-419.6158	
92.1600	-425.2692	-0.1062	-0.2797	-
1.0011	-0.9304	-0.6071	0.1296	-0.3256
	-1.3174	-1.2881	-419.6095	
92.1800	-425.4916	-0.2100	0.0089	-
1.4427	-1.0091	-0.5843	0.1637	-0.3122
	-1.2914	-1.2949	-419.6032	
92.2000	-425.5968	0.2116	0.1797	-
1.7436	-1.0102	-0.5499	0.1967	-0.2982
	-1.2654	-1.3018	-419.5969	
92.2200	-425.4381	0.3145	-0.1127	-
1.8136	-0.9313	-0.5035	0.2285	-0.2838
	-1.2395	-1.3086	-419.5906	

92.2400 -424.9229 -0.4477 -0.5952 -
1.5957 -0.7785 -0.4456 0.2590 -0.2688
-1.2135 -1.3153 -419.5843
92.2600 -424.3021 0.3878 -0.9509 -
1.0713 -0.5673 -0.3775 0.2883 -0.2534
-1.1876 -1.3221 -419.5780
92.2800 -423.6898 -0.1651 -1.2089 -
0.2920 -0.3194 -0.3005 0.3161 -0.2375
-1.1618 -1.3288 -419.5717
92.3000 -422.9109 0.2658 -1.1082
0.6283 -0.0589 -0.2162 0.3423 -0.2211
-1.1359 -1.3355 -419.5654
92.3200 -421.6954 -1.0320 -0.1821
1.5031 0.1912 -0.1260 0.3670 -0.2042
-1.1101 -1.3422 -419.5591
92.3400 -418.5186 -0.4012 1.4249
2.1235 0.4093 -0.0316 0.3900 -0.1869
-1.0844 -1.3489 -419.5529
92.3600 -415.5484 0.7922 2.6512
2.3012 0.5755 0.0655 0.4112 -0.1692
-1.0586 -1.3555 -419.5466
92.3800 -415.9779 0.7589 2.5265
1.9345 0.6762 0.1633 0.4306 -0.1510
-1.0330 -1.3622 -419.5403
92.4000 -419.1434 -0.5556 0.9162
1.1025 0.7102 0.2600 0.4480 -0.1324
-1.0073 -1.3688 -419.5341
92.4200 -421.7260 -0.6974 -1.1453
0.0429 0.6867 0.3536 0.4633 -0.1134
-0.9817 -1.3753 -419.5278
92.4400 -423.1541 0.4571 -2.3272 -
0.9574 0.6175 0.4423 0.4765 -0.0940
-0.9562 -1.3819 -419.5216
92.4600 -424.1369 0.0157 -2.1492 -
1.6480 0.5153 0.5242 0.4874 -0.0742
-0.9307 -1.3884 -419.5153
92.4800 -423.9364 -0.5916 -0.9676 -
1.8854 0.3939 0.5974 0.4961 -0.0539
-0.9053 -1.3949 -419.5091
92.5000 -421.7107 0.2574 0.3973 -
1.6685 0.2679 0.6602 0.5024 -0.0334
-0.8799 -1.4014 -419.5028
92.5200 -419.8017 0.4698 1.0035 -
1.0857 0.1507 0.7109 0.5064 -0.0124
-0.8546 -1.4079 -419.4966
92.5400 -419.9639 0.2758 0.5111 -
0.2876 0.0526 0.7483 0.5079 0.0089
-0.8294 -1.4143 -419.4903
92.5600 -420.6694 -0.3955 -0.4848
0.5021 -0.0185 0.7715 0.5070 0.0306
-0.8042 -1.4208 -419.4841

92.5800	-420.9585	-1.0109	-0.6113	
1.0493	-0.0570	0.7800	0.5037	0.0526
	-0.7790	-1.4272	-419.4779	
92.6000	-418.8368	-0.8010	0.6074	
1.2133	-0.0603	0.7736	0.4980	0.0749
	-0.7540	-1.4336	-419.4716	
92.6200	-416.1305	1.6875	1.8078	
0.9789	-0.0276	0.7525	0.4900	0.0976
	-0.7290	-1.4399	-419.4654	
92.6400	-416.9336	1.6335	1.3681	
0.4198	0.0375	0.7173	0.4798	0.1205
	-0.7040	-1.4463	-419.4592	
92.6600	-422.5141	-0.3731	-0.5593	-
0.2816	0.1290	0.6687	0.4675	0.1437
	-0.6792	-1.4526	-419.4530	
92.6800	-425.8978	-2.7047	-2.0394	-
0.8822	0.2400	0.6078	0.4532	0.1673
	-0.6544	-1.4589	-419.4468	
92.7000	-423.6056	-0.7545	-1.6059	-
1.1764	0.3620	0.5359	0.4372	0.1911
	-0.6297	-1.4651	-419.4406	
92.7200	-419.8492	1.3946	0.1185	-
1.1097	0.4832	0.4546	0.4194	0.2151
	-0.6051	-1.4714	-419.4344	
92.7400	-418.6419	1.0394	1.3663	-
0.7534	0.5915	0.3657	0.4003	0.2394
	-0.5805	-1.4776	-419.4282	
92.7600	-419.2436	-0.1813	1.1234	-
0.2257	0.6753	0.2710	0.3798	0.2639
	-0.5560	-1.4838	-419.4220	
92.7800	-419.8530	-0.2786	-0.0090	
0.3487	0.7248	0.1725	0.3582	0.2887
	-0.5316	-1.4900	-419.4158	
92.8000	-419.8789	-0.6432	-0.7435	
0.8484	0.7318	0.0721	0.3357	0.3136
	-0.5073	-1.4962	-419.4096	
92.8200	-419.7962	0.4287	-0.6826	
1.1778	0.6902	-0.0283	0.3126	0.3387
	-0.4831	-1.5023	-419.4034	
92.8400	-419.6851	-0.1150	-0.2715	
1.2852	0.5945	-0.1265	0.2888	0.3640
	-0.4589	-1.5084	-419.3972	
92.8600	-419.4606	-0.0885	0.2351	
1.1329	0.4431	-0.2206	0.2648	0.3894
	-0.4349	-1.5145	-419.3910	
92.8800	-419.2354	-0.2377	0.9722	
0.7123	0.2412	-0.3090	0.2405	0.4150
	-0.4109	-1.5206	-419.3849	
92.9000	-419.4642	0.3737	1.4682	
0.0903	0.0013	-0.3901	0.2162	0.4407
	-0.3870	-1.5267	-419.3787	

92.9200 -420.9241 0.2505 1.0859 -
0.6102 -0.2604 -0.4624 0.1920 0.4665
-0.3633 -1.5327 -419.3725
92.9400 -422.6479 -0.0093 -0.0755 -
1.2214 -0.5273 -0.5248 0.1681 0.4925
-0.3396 -1.5387 -419.3664
92.9600 -424.4526 -0.0800 -1.3790 -
1.5680 -0.7822 -0.5759 0.1446 0.5185
-0.3160 -1.5447 -419.3602
92.9800 -426.2396 -0.2161 -2.1263 -
1.4994 -1.0087 -0.6148 0.1216 0.5446
-0.2925 -1.5507 -419.3540
93.0000 -426.6180 -1.5406 -1.6006 -
0.9904 -1.1934 -0.6407 0.0993 0.5708
-0.2691 -1.5566 -419.3479
93.0200 -423.1753 -0.3974 0.1272 -
0.1994 -1.3253 -0.6530 0.0778 0.5970
-0.2458 -1.5625 -419.3417
93.0400 -418.9451 1.4450 1.7109
0.6104 -1.3957 -0.6516 0.0571 0.6233
-0.2226 -1.5684 -419.3356
93.0600 -418.3135 0.9058 1.7427
1.1711 -1.3973 -0.6366 0.0374 0.6497
-0.1995 -1.5743 -419.3294
93.0800 -420.4972 -0.1406 0.3875
1.3029 -1.3248 -0.6088 0.0185 0.6760
-0.1765 -1.5802 -419.3233
93.1000 -422.3332 -1.2079 -0.5906
0.9602 -1.1783 -0.5694 0.0007 0.7024
-0.1536 -1.5860 -419.3172
93.1200 -422.1785 0.1493 -0.2238
0.2194 -0.9644 -0.5199 -0.0163 0.7287
-0.1308 -1.5918 -419.3110
93.1400 -421.6409 -0.2307 0.6070 -
0.6955 -0.6953 -0.4621 -0.0322 0.7551
-0.1081 -1.5976 -419.3049
93.1600 -421.4236 0.6485 0.7612 -
1.4964 -0.3847 -0.3977 -0.0472 0.7814
-0.0855 -1.6034 -419.2988
93.1800 -422.4183 -0.2529 -0.0729 -
1.9409 -0.0466 -0.3287 -0.0611 0.8077
-0.0631 -1.6091 -419.2927
93.2000 -423.7257 0.2616 -1.1908 -
1.8928 0.3040 -0.2570 -0.0741 0.8340
-0.0407 -1.6148 -419.2866
93.2200 -423.4736 -1.1251 -1.3636 -
1.3517 0.6488 -0.1847 -0.0861 0.8602
-0.0185 -1.6205 -419.2804
93.2400 -420.0854 -0.0285 -0.2897 -
0.4708 0.9696 -0.1138 -0.0970 0.8863
0.0036 -1.6262 -419.2743

93.2600 -416.9508 0.7486 1.0263
0.5110 1.2485 -0.0461 -0.1070 0.9123
0.0256 -1.6319 -419.2682
93.2800 -416.2620 -0.4760 1.5286
1.3620 1.4672 0.0164 -0.1161 0.9383
0.0475 -1.6375 -419.2621
93.3000 -415.8927 0.0345 1.0969
1.9313 1.6084 0.0721 -0.1242 0.9641
0.0693 -1.6431 -419.2560
93.3200 -415.8565 -0.2355 0.1697
2.1672 1.6558 0.1199 -0.1315 0.9899
0.0909 -1.6487 -419.2499
93.3400 -416.8170 0.5163 -0.8586
2.1111 1.5998 0.1588 -0.1379 1.0155
0.1124 -1.6543 -419.2438
93.3600 -417.7552 -0.3324 -1.3639
1.8615 1.4405 0.1884 -0.1435 1.0410
0.1338 -1.6599 -419.2377
93.3800 -417.6605 -0.0824 -0.9240
1.4996 1.1880 0.2091 -0.1484 1.0663
0.1551 -1.6654 -419.2317
93.4000 -417.3191 -0.1002 0.0801
1.0636 0.8628 0.2216 -0.1525 1.0915
0.1763 -1.6709 -419.2256
93.4200 -417.2346 0.2367 1.0240
0.5480 0.4915 0.2267 -0.1561 1.1165
0.1973 -1.6764 -419.2195
93.4400 -417.8055 -0.0343 1.4140 -
0.0836 0.1041 0.2252 -0.1590 1.1413
0.2182 -1.6818 -419.2134
93.4600 -418.8312 0.4868 1.0479 -
0.8331 -0.2703 0.2179 -0.1615 1.1660
0.2390 -1.6873 -419.2074
93.4800 -420.9798 0.2248 0.0116 -
1.6108 -0.6056 0.2060 -0.1635 1.1904
0.2596 -1.6927 -419.2013
93.5000 -424.5050 -0.5911 -1.1562 -
2.2603 -0.8785 0.1904 -0.1652 1.2147
0.2801 -1.6981 -419.1952
93.5200 -425.9600 -0.6255 -1.7731 -
2.6006 -1.0685 0.1724 -0.1666 1.2387
0.3005 -1.7035 -419.1892
93.5400 -424.1720 -0.2190 -1.4809 -
2.4917 -1.1627 0.1534 -0.1679 1.2625
0.3207 -1.7088 -419.1831
93.5600 -421.5770 0.8187 -0.5766 -
1.9172 -1.1616 0.1347 -0.1690 1.2860
0.3408 -1.7142 -419.1771
93.5800 -420.4936 1.0260 0.0244 -
0.9825 -1.0798 0.1175 -0.1701 1.3093
0.3608 -1.7195 -419.1710

93.6000 -420.2025 -0.3264 -0.0867
0.1293 -0.9395 0.1030 -0.1711 1.3323
0.3806 -1.7248 -419.1650
93.6200 -419.8647 -1.0148 -0.0784
1.1588 -0.7644 0.0922 -0.1723 1.3550
0.4003 -1.7300 -419.1589
93.6400 -417.8443 -1.4791 1.1716
1.8575 -0.5768 0.0860 -0.1735 1.3774
0.4199 -1.7353 -419.1529
93.6600 -413.3919 1.2701 2.8054
2.0970 -0.3969 0.0854 -0.1748 1.3996
0.4393 -1.7405 -419.1469
93.6800 -411.6877 2.8190 2.7733
1.8511 -0.2413 0.0911 -0.1762 1.4214
0.4586 -1.7457 -419.1408
93.7000 -417.3017 0.5379 0.3864
1.2350 -0.1206 0.1037 -0.1778 1.4429
0.4777 -1.7509 -419.1348
93.7200 -423.3175 -3.1751 -2.3139
0.4977 -0.0400 0.1233 -0.1796 1.4640
0.4967 -1.7561 -419.1288
93.7400 -422.9056 -0.8312 -3.1998 -
0.1057 -0.0025 0.1499 -0.1815 1.4848
0.5155 -1.7612 -419.1228
93.7600 -420.5019 0.8386 -2.1118 -
0.4043 -0.0075 0.1827 -0.1836 1.5053
0.5342 -1.7663 -419.1167
93.7800 -418.6916 0.5091 -0.2240 -
0.3989 -0.0475 0.2208 -0.1857 1.5253
0.5528 -1.7714 -419.1107
93.8000 -417.7270 -0.3093 1.2427 -
0.2024 -0.1098 0.2630 -0.1878 1.5450
0.5712 -1.7765 -419.1047
93.8200 -417.0897 0.3171 1.6766
0.0251 -0.1795 0.3081 -0.1899 1.5643
0.5894 -1.7815 -419.0987
93.8400 -417.2089 0.4002 1.3544
0.1282 -0.2413 0.3548 -0.1917 1.5831
0.6075 -1.7866 -419.0927
93.8600 -418.2756 0.1342 0.7533
0.0507 -0.2816 0.4017 -0.1933 1.6015
0.6255 -1.7916 -419.0867
93.8800 -419.1004 -0.5358 0.1497 -
0.1769 -0.2912 0.4472 -0.1944 1.6195
0.6433 -1.7966 -419.0807
93.9000 -419.3331 -0.1344 -0.2241 -
0.4772 -0.2648 0.4897 -0.1951 1.6370
0.6609 -1.8015 -419.0747
93.9200 -419.4758 0.3404 -0.5032 -
0.7417 -0.1978 0.5274 -0.1951 1.6541
0.6784 -1.8065 -419.0687

93.9400 -419.4512 -0.3015 -0.4932 -
0.8735 -0.0874 0.5587 -0.1944 1.6707
0.6957 -1.8114 -419.0628
93.9600 -419.0573 0.3776 -0.2118 -
0.8348 0.0640 0.5820 -0.1928 1.6868
0.7129 -1.8163 -419.0568
93.9800 -418.4872 -0.3246 0.1183 -
0.6482 0.2499 0.5957 -0.1902 1.7024
0.7299 -1.8212 -419.0508
94.0000 -417.6787 -0.0865 0.2367 -
0.3568 0.4599 0.5982 -0.1865 1.7174
0.7468 -1.8260 -419.0448
94.0200 -416.6964 -0.1240 0.2380 -
0.0106 0.6795 0.5882 -0.1815 1.7320
0.7635 -1.8309 -419.0389
94.0400 -416.4117 0.0905 0.1221
0.3496 0.8904 0.5647 -0.1752 1.7459
0.7801 -1.8357 -419.0329
94.0600 -416.5597 0.3896 -0.2018
0.6655 1.0734 0.5266 -0.1675 1.7594
0.7965 -1.8405 -419.0269
94.0800 -416.6951 -0.3714 -0.5056
0.8718 1.2123 0.4731 -0.1583 1.7723
0.8127 -1.8453 -419.0210
94.1000 -416.3222 0.0565 -0.3449
0.9174 1.2974 0.4040 -0.1475 1.7846
0.8288 -1.8500 -419.0150
94.1200 -415.4670 -0.1577 0.3398
0.7741 1.3273 0.3194 -0.1352 1.7963
0.8447 -1.8547 -419.0091
94.1400 -415.2103 -0.0271 1.1744
0.4513 1.3062 0.2201 -0.1213 1.8074
0.8605 -1.8595 -419.0031
94.1600 -415.3577 0.0078 1.6883
0.0046 1.2422 0.1072 -0.1059 1.8180
0.8760 -1.8641 -418.9972
94.1800 -415.6604 0.3585 1.0278 -
0.4649 1.1466 -0.0180 -0.0890 1.8279
0.8915 -1.8688 -418.9912
94.2000 -418.3268 0.8468 -1.0468 -
0.8440 1.0317 -0.1536 -0.0705 1.8373
0.9067 -1.8735 -418.9853
94.2200 -422.9459 -0.9256 -3.1134 -
1.0317 0.9084 -0.2980 -0.0507 1.8460
0.9218 -1.8781 -418.9793
94.2400 -423.5024 -1.3891 -2.9309 -
0.9739 0.7848 -0.4489 -0.0295 1.8541
0.9368 -1.8827 -418.9734
94.2600 -418.9522 0.7022 -0.2285 -
0.7126 0.6661 -0.6039 -0.0070 1.8616
0.9515 -1.8873 -418.9675

94.2800 -414.6685 1.8374 2.7346 -
0.3696 0.5545 -0.7603 0.0167 1.8684
0.9661 -1.8918 -418.9616
94.3000 -414.7759 0.1455 3.7699 -
0.0767 0.4495 -0.9147 0.0415 1.8746
0.9806 -1.8964 -418.9556
94.3200 -416.5518 -0.4279 2.4143
0.0768 0.3476 -1.0632 0.0674 1.8802
0.9949 -1.9009 -418.9497
94.3400 -418.4783 0.3310 -0.2310
0.1116 0.2395 -1.2020 0.0943 1.8851
1.0090 -1.9054 -418.9438
94.3600 -420.9385 0.4779 -2.5671
0.1125 0.1113 -1.3272 0.1221 1.8893
1.0229 -1.9099 -418.9379
94.3800 -422.7717 -1.2442 -3.2427
0.1841 -0.0524 -1.4350 0.1508 1.8929
1.0367 -1.9144 -418.9320
94.4000 -421.7132 -0.8515 -1.8612
0.3996 -0.2648 -1.5219 0.1803 1.8958
1.0503 -1.9188 -418.9261
94.4200 -418.0371 0.4896 0.5295
0.7644 -0.5320 -1.5849 0.2106 1.8981
1.0637 -1.9232 -418.9202
94.4400 -415.8013 1.2322 1.9733
1.2206 -0.8490 -1.6220 0.2415 1.8997
1.0770 -1.9276 -418.9143
94.4600 -417.2460 -0.0964 1.5003
1.6634 -1.2041 -1.6321 0.2730 1.9006
1.0901 -1.9320 -418.9084
94.4800 -419.5543 -1.1510 0.0656
1.9783 -1.5803 -1.6147 0.3051 1.9009
1.1030 -1.9364 -418.9025
94.5000 -420.0299 -0.1571 -0.8977
2.0528 -1.9572 -1.5694 0.3375 1.9005
1.1158 -1.9407 -418.8966
94.5200 -420.0578 0.6080 -0.8748
1.8144 -2.3121 -1.4962 0.3702 1.8994
1.1284 -1.9450 -418.8907
94.5400 -420.0935 0.1261 -0.0957
1.2428 -2.6222 -1.3954 0.4028 1.8977
1.1409 -1.9493 -418.8848
94.5600 -420.2028 -0.1941 0.9893
0.3718 -2.8648 -1.2679 0.4353 1.8952
1.1531 -1.9536 -418.8790
94.5800 -420.4360 -0.8509 1.7238 -
0.7119 -3.0179 -1.1145 0.4674 1.8921
1.1653 -1.9578 -418.8731
94.6000 -420.9624 1.1692 1.3096 -
1.8635 -3.0604 -0.9369 0.4989 1.8883
1.1772 -1.9621 -418.8672

94.6200 -423.7982 1.1647 -0.3410 -
2.8638 -2.9724 -0.7370 0.5295 1.8839
1.1890 -1.9663 -418.8613
94.6400 -426.7827 -1.7052 -1.9776 -
3.4877 -2.7368 -0.5178 0.5591 1.8788
1.2006 -1.9705 -418.8555
94.6600 -426.4796 -1.2663 -2.2241 -
3.5493 -2.3461 -0.2830 0.5875 1.8730
1.2121 -1.9747 -418.8496
94.6800 -422.9790 0.2960 -1.0877 -
2.9780 -1.8134 -0.0371 0.6144 1.8666
1.2234 -1.9789 -418.8438
94.7000 -419.1668 1.2374 0.1559 -
1.8769 -1.1710 0.2158 0.6396 1.8594
1.2345 -1.9830 -418.8379
94.7200 -417.1063 0.3221 0.3056 -
0.4456 -0.4576 0.4711 0.6630 1.8517
1.2455 -1.9871 -418.8321
94.7400 -415.5816 -0.7902 -0.3008
1.0479 0.2876 0.7246 0.6842 1.8432
1.2563 -1.9912 -418.8262
94.7600 -413.6399 -0.2072 -0.2220
2.3098 1.0254 0.9717 0.7031 1.8342
1.2669 -1.9953 -418.8204
94.7800 -409.8625 -0.0780 0.9254
3.1196 1.7168 1.2082 0.7195 1.8244
1.2774 -1.9994 -418.8145
94.8000 -406.8459 1.0780 2.1782
3.3700 2.3266 1.4297 0.7332 1.8141
1.2877 -2.0034 -418.8087
94.8200 -406.9606 0.2621 2.5195
3.0509 2.8276 1.6317 0.7439 1.8031
1.2979 -2.0075 -418.8028
94.8400 -407.8804 -0.4230 1.5016
2.2726 3.2053 1.8100 0.7514 1.7915
1.3079 -2.0115 -418.7970
94.8600 -409.9376 1.3555 -0.5628
1.2599 3.4566 1.9605 0.7555 1.7792
1.3177 -2.0155 -418.7912
94.8800 -414.1519 -1.4468 -2.4655
0.2692 3.5830 2.0795 0.7561 1.7664
1.3274 -2.0194 -418.7854
94.9000 -415.1175 -0.9767 -2.6917 -
0.4955 3.5884 2.1638 0.7528 1.7530
1.3369 -2.0234 -418.7795
94.9200 -412.6671 0.7384 -0.9637 -
0.9682 3.4830 2.2115 0.7456 1.7390
1.3463 -2.0273 -418.7737
94.9400 -410.8083 0.7736 1.2651 -
1.2117 3.2817 2.2223 0.7345 1.7244
1.3555 -2.0313 -418.7679

94.9600	-411.0764	-0.0439	2.1590	-
1.3184	3.0007	2.1973	0.7193	1.7093
	1.3645	-2.0352	-418.7621	
94.9800	-411.7979	-0.1265	1.5435	-
1.3499	2.6542	2.1382	0.7003	1.6937
	1.3734	-2.0391	-418.7563	
95.0000	-413.3143	-0.1629	0.3268	-
1.3152	2.2560	2.0478	0.6776	1.6776
	1.3822	-2.0429	-418.7505	
95.0200	-416.0263	-0.1208	-0.9644	-
1.1633	1.8163	1.9290	0.6514	1.6610
	1.3908	-2.0468	-418.7447	
95.0400	-416.8315	-0.3989	-1.6748	-
0.8448	1.3446	1.7848	0.6218	1.6439
	1.3992	-2.0506	-418.7389	
95.0600	-416.0261	0.3796	-1.3478	-
0.3581	0.8509	1.6186	0.5893	1.6264
	1.4075	-2.0544	-418.7331	
95.0800	-415.2450	0.3730	-0.4378	
0.2042	0.3468	1.4337	0.5539	1.6084
	1.4156	-2.0582	-418.7273	
95.1000	-415.0941	-0.3470	0.5937	
0.6946	-0.1538	1.2334	0.5159	1.5901
	1.4236	-2.0620	-418.7215	
95.1200	-415.0150	0.1956	1.4289	
0.9660	-0.6385	1.0208	0.4756	1.5714
	1.4314	-2.0658	-418.7157	
95.1400	-415.2005	0.2999	1.5183	
0.9254	-1.0964	0.7992	0.4332	1.5524
	1.4391	-2.0696	-418.7099	
95.1600	-417.0307	0.0565	0.8163	
0.5986	-1.5162	0.5715	0.3888	1.5331
	1.4466	-2.0733	-418.7041	
95.1800	-419.1744	0.1809	-0.2595	
0.1013	-1.8873	0.3409	0.3429	1.5134
	1.4540	-2.0770	-418.6983	
95.2000	-420.8522	0.2877	-1.1320	-
0.4001	-2.1985	0.1103	0.2954	1.4935
	1.4612	-2.0807	-418.6926	
95.2200	-422.3219	-0.3450	-1.3689	-
0.7509	-2.4391	-0.1174	0.2468	1.4734
	1.4683	-2.0844	-418.6868	
95.2400	-422.5968	0.1393	-0.8074	-
0.8658	-2.6004	-0.3391	0.1972	1.4530
	1.4752	-2.0881	-418.6810	
95.2600	-421.4177	-0.0310	0.0742	-
0.7501	-2.6795	-0.5519	0.1469	1.4324
	1.4820	-2.0917	-418.6753	
95.2800	-420.1662	0.4062	0.6983	-
0.4643	-2.6797	-0.7533	0.0961	1.4116
	1.4887	-2.0954	-418.6695	

95.3000 -420.0413 -0.0406 0.8064 -
0.1279 -2.6085 -0.9408 0.0450 1.3907
1.4952 -2.0990 -418.6637
95.3200 -420.2603 0.4395 0.5459
0.1412 -2.4759 -1.1121 -0.0061 1.3696
1.5016 -2.1026 -418.6580
95.3400 -420.5817 -0.2584 0.1319
0.2842 -2.2928 -1.2657 -0.0571 1.3485
1.5078 -2.1062 -418.6522
95.3600 -421.2521 0.8175 -0.2112
0.3091 -2.0731 -1.4001 -0.1076 1.3272
1.5139 -2.1098 -418.6465
95.3800 -422.1240 -0.2151 -0.5397
0.2933 -1.8307 -1.5141 -0.1575 1.3060
1.5198 -2.1134 -418.6407
95.4000 -422.3453 -0.3102 -0.8585
0.2905 -1.5805 -1.6070 -0.2066 1.2846
1.5257 -2.1170 -418.6350
95.4200 -421.5255 0.0723 -0.6797
0.3046 -1.3358 -1.6783 -0.2546 1.2633
1.5313 -2.1205 -418.6292
95.4400 -420.5268 0.3207 -0.0533
0.3086 -1.1073 -1.7280 -0.3014 1.2420
1.5369 -2.1240 -418.6235
95.4600 -420.1674 -0.0977 0.6788
0.2403 -0.9012 -1.7565 -0.3469 1.2207
1.5423 -2.1275 -418.6178
95.4800 -419.9822 -0.0688 1.1475
0.0708 -0.7205 -1.7642 -0.3908 1.1994
1.5476 -2.1311 -418.6120
95.5000 -419.9008 -0.2369 0.9062 -
0.1381 -0.5649 -1.7522 -0.4331 1.1783
1.5527 -2.1345 -418.6063
95.5200 -420.6102 0.1507 -0.1268 -
0.2967 -0.4319 -1.7213 -0.4737 1.1572
1.5577 -2.1380 -418.6006
95.5400 -422.0563 0.0096 -1.2585 -
0.3200 -0.3184 -1.6728 -0.5125 1.1363
1.5626 -2.1415 -418.5948
95.5600 -422.4937 -0.7729 -1.4555 -
0.1718 -0.2200 -1.6082 -0.5494 1.1155
1.5674 -2.1449 -418.5891
95.5800 -420.4008 -0.2656 -0.2594
0.1010 -0.1334 -1.5293 -0.5843 1.0949
1.5720 -2.1484 -418.5834
95.6000 -417.9723 0.9697 1.2290
0.3784 -0.0548 -1.4379 -0.6173 1.0745
1.5765 -2.1518 -418.5777
95.6200 -417.8634 0.2048 1.6489
0.5269 0.0192 -1.3357 -0.6482 1.0543
1.5809 -2.1552 -418.5720

95.6400 -419.1673 -0.3347 0.8778
0.4713 0.0926 -1.2245 -0.6771 1.0344
1.5851 -2.1586 -418.5663
95.6600 -420.4467 -0.2965 -0.3129
0.2229 0.1673 -1.1060 -0.7038 1.0147
1.5892 -2.1620 -418.5606
95.6800 -421.2204 0.0174 -1.5219 -
0.1148 0.2428 -0.9818 -0.7283 0.9953
1.5932 -2.1654 -418.5549
95.7000 -421.8359 0.1774 -2.1648 -
0.4370 0.3164 -0.8533 -0.7507 0.9761
1.5971 -2.1688 -418.5492
95.7200 -421.8007 -1.0429 -1.3779 -
0.6845 0.3837 -0.7215 -0.7708 0.9573
1.6009 -2.1721 -418.5435
95.7400 -419.0041 0.5619 0.8711 -
0.8369 0.4394 -0.5878 -0.7888 0.9388
1.6045 -2.1755 -418.5378
95.7600 -416.0321 1.2693 2.8640 -
0.8869 0.4785 -0.4533 -0.8046 0.9207
1.6081 -2.1788 -418.5321
95.7800 -416.2550 -0.0122 2.8858 -
0.8158 0.4970 -0.3191 -0.8182 0.9030
1.6115 -2.1821 -418.5264
95.8000 -418.4295 0.0965 1.1066 -
0.6226 0.4937 -0.1860 -0.8296 0.8856
1.6148 -2.1855 -418.5207
95.8200 -420.2612 -0.3142 -1.4021 -
0.2995 0.4683 -0.0551 -0.8390 0.8686
1.6180 -2.1888 -418.5150
95.8400 -421.3116 0.4690 -3.5924
0.1591 0.4214 0.0727 -0.8462 0.8520
1.6210 -2.1921 -418.5093
95.8600 -422.0608 -0.5117 -4.1543
0.7152 0.3548 0.1964 -0.8513 0.8358
1.6240 -2.1953 -418.5036
95.8800 -421.3015 -1.6074 -2.0985
1.2790 0.2733 0.3154 -0.8544 0.8201
1.6268 -2.1986 -418.4980
95.9000 -415.0752 -0.0281 1.6286
1.7004 0.1835 0.4286 -0.8556 0.8048
1.6296 -2.2019 -418.4923
95.9200 -409.8773 2.3473 4.5453
1.8251 0.0926 0.5351 -0.8549 0.7899
1.6322 -2.2051 -418.4866
95.9400 -411.6126 1.4211 4.5736
1.5284 0.0080 0.6341 -0.8523 0.7755
1.6347 -2.2084 -418.4809
95.9600 -416.5569 -1.1599 1.9350
0.8194 -0.0630 0.7247 -0.8478 0.7615
1.6372 -2.2116 -418.4753

95.9800 -420.3472 0.1205 -1.4359 -
0.1249 -0.1129 0.8062 -0.8414 0.7479
1.6395 -2.2149 -418.4696
96.0000 -423.6172 -0.5348 -3.7799 -
1.0600 -0.1343 0.8778 -0.8333 0.7348
1.6417 -2.2181 -418.4640
96.0200 -425.5351 -1.2422 -4.0716 -
1.7617 -0.1209 0.9391 -0.8234 0.7222
1.6438 -2.2213 -418.4583
96.0400 -423.4831 -1.2510 -2.0688 -
2.1037 -0.0690 0.9895 -0.8117 0.7100
1.6458 -2.2245 -418.4526
96.0600 -418.1165 1.0884 1.0137 -
2.0763 0.0213 1.0285 -0.7982 0.6983
1.6477 -2.2277 -418.4470
96.0800 -415.2020 1.7110 2.8868 -
1.7124 0.1436 1.0560 -0.7830 0.6871
1.6495 -2.2309 -418.4413
96.1000 -416.6453 0.4528 2.2359 -
1.0746 0.2881 1.0717 -0.7661 0.6763
1.6512 -2.2341 -418.4357
96.1200 -418.7837 -1.0826 0.1884 -
0.2837 0.4421 1.0754 -0.7475 0.6659
1.6528 -2.2373 -418.4300
96.1400 -419.0204 -0.7957 -1.4233
0.5080 0.5915 1.0670 -0.7273 0.6561
1.6543 -2.2404 -418.4244
96.1600 -417.8741 0.4745 -1.5264
1.1828 0.7214 1.0465 -0.7056 0.6466
1.6557 -2.2436 -418.4188
96.1800 -416.4162 0.0057 -0.5667
1.6811 0.8175 1.0138 -0.6824 0.6376
1.6570 -2.2467 -418.4131
96.2000 -414.7505 -0.2087 0.6356
1.9418 0.8688 0.9691 -0.6578 0.6291
1.6583 -2.2499 -418.4075
96.2200 -413.0712 -0.0438 1.6799
1.8976 0.8708 0.9129 -0.6319 0.6210
1.6594 -2.2530 -418.4019
96.2400 -413.0071 0.6374 2.0432
1.5093 0.8241 0.8460 -0.6048 0.6133
1.6604 -2.2562 -418.3962
96.2600 -415.4145 -0.2408 1.2241
0.8268 0.7328 0.7696 -0.5765 0.6061
1.6614 -2.2593 -418.3906
96.2800 -418.0009 -0.1997 -0.2914
0.0256 0.6029 0.6849 -0.5472 0.5993
1.6623 -2.2624 -418.3850
96.3000 -419.9465 0.1213 -1.7230 -
0.6751 0.4416 0.5936 -0.5169 0.5929
1.6631 -2.2655 -418.3793

96.3200	-421.5041	-0.4641	-2.5627	-
1.0858	0.2564	0.4971	-0.4857	0.5869
	1.6638	-2.2687	-418.3737	
96.3400	-421.7194	-0.0582	-2.6102	-
1.1154	0.0565	0.3972	-0.4537	0.5814
	1.6644	-2.2718	-418.3681	
96.3600	-421.0426	0.3190	-1.7631	-
0.8133	-0.1482	0.2955	-0.4209	0.5762
	1.6649	-2.2749	-418.3625	
96.3800	-419.9298	-0.4312	-0.0054	-
0.3295	-0.3477	0.1935	-0.3875	0.5715
	1.6654	-2.2780	-418.3569	
96.4000	-417.7757	-0.9577	2.2648	
0.1384	-0.5318	0.0929	-0.3534	0.5671
	1.6657	-2.2811	-418.3513	
96.4200	-414.1713	0.2586	4.0742	
0.4072	-0.6912	-0.0048	-0.3189	0.5632
	1.6661	-2.2842	-418.3457	
96.4400	-412.5633	2.6618	3.7652	
0.3706	-0.8177	-0.0980	-0.2839	0.5596
	1.6663	-2.2872	-418.3401	
96.4600	-418.2174	0.8883	0.9098	
0.0527	-0.9052	-0.1854	-0.2486	0.5564
	1.6664	-2.2903	-418.3344	
96.4800	-424.9727	-2.5530	-2.4452	-
0.3977	-0.9491	-0.2658	-0.2130	0.5535
	1.6665	-2.2934	-418.3288	
96.5000	-425.0017	-0.4843	-3.7928	-
0.8032	-0.9462	-0.3381	-0.1773	0.5511
	1.6665	-2.2965	-418.3232	
96.5200	-423.2569	1.1254	-2.9119	-
0.9923	-0.8945	-0.4016	-0.1415	0.5490
	1.6665	-2.2996	-418.3176	
96.5400	-421.4870	0.3789	-1.5634	-
0.8702	-0.7947	-0.4559	-0.1058	0.5472
	1.6663	-2.3026	-418.3121	
96.5600	-420.5008	0.4055	-0.9320	-
0.4421	-0.6516	-0.5009	-0.0704	0.5458
	1.6662	-2.3057	-418.3065	
96.5800	-419.7033	-0.2145	-0.6159	
0.1421	-0.4744	-0.5368	-0.0352	0.5447
	1.6659	-2.3088	-418.3009	
96.6000	-418.5943	-1.0327	0.4182	
0.6686	-0.2742	-0.5637	-0.0006	0.5439
	1.6656	-2.3118	-418.2953	
96.6200	-416.0807	-0.0577	2.1213	
0.9598	-0.0623	-0.5818	0.0334	0.5435
	1.6652	-2.3149	-418.2897	
96.6400	-413.6551	1.1685	2.8929	
0.9156	0.1501	-0.5915	0.0667	0.5433
	1.6648	-2.3179	-418.2841	

96.6600 -414.5443 1.3550 1.5544
0.5355 0.3505 -0.5929 0.0992 0.5435
1.6643 -2.3210 -418.2785
96.6800 -420.0455 -1.1685 -0.9788 -
0.0152 0.5273 -0.5863 0.1308 0.5439
1.6637 -2.3241 -418.2729
96.7000 -423.1886 -2.4403 -2.3507 -
0.5292 0.6705 -0.5723 0.1614 0.5447
1.6631 -2.3271 -418.2674
96.7200 -420.7377 0.1368 -1.4861 -
0.8352 0.7718 -0.5514 0.1909 0.5457
1.6625 -2.3302 -418.2618
96.7400 -417.0582 1.6643 0.2834 -
0.8714 0.8270 -0.5244 0.2193 0.5469
1.6618 -2.3332 -418.2562
96.7600 -416.2833 1.0386 1.0695 -
0.6392 0.8359 -0.4920 0.2466 0.5485
1.6610 -2.3363 -418.2506
96.7800 -417.8122 -0.2438 0.4010 -
0.2007 0.7978 -0.4547 0.2726 0.5502
1.6602 -2.3393 -418.2451
96.8000 -419.1464 -0.9467 -0.4894
0.3280 0.7134 -0.4133 0.2975 0.5522
1.6593 -2.3424 -418.2395
96.8200 -418.4209 -0.4210 -0.5276
0.8066 0.5857 -0.3681 0.3210 0.5545
1.6584 -2.3455 -418.2339
96.8400 -416.5387 0.5598 0.0631
1.1279 0.4190 -0.3199 0.3433 0.5569
1.6575 -2.3485 -418.2284
96.8600 -415.8641 0.2152 0.4281
1.2113 0.2202 -0.2694 0.3643 0.5596
1.6565 -2.3516 -418.2228
96.8800 -417.1676 0.0356 0.3237
1.0153 0.0000 -0.2171 0.3841 0.5624
1.6555 -2.3546 -418.2173
96.9000 -418.5727 -1.1536 0.2457
0.5663 -0.2275 -0.1636 0.4025 0.5654
1.6544 -2.3577 -418.2117
96.9200 -418.6829 -0.4931 0.7362 -
0.0392 -0.4453 -0.1096 0.4196 0.5686
1.6532 -2.3608 -418.2061
96.9400 -418.6282 0.0729 1.1300 -
0.6486 -0.6362 -0.0556 0.4353 0.5719
1.6521 -2.3638 -418.2006
96.9600 -418.6229 1.3151 0.3007 -
1.1097 -0.7841 -0.0021 0.4497 0.5754
1.6509 -2.3669 -418.1950
96.9800 -420.8442 0.6674 -1.5904 -
1.3052 -0.8749 0.0503 0.4628 0.5790
1.6496 -2.3700 -418.1895

97.0000 -423.9825 -1.5037 -2.7699 -
1.1816 -0.9002 0.1013 0.4746 0.5828
1.6484 -2.3730 -418.1839
97.0200 -423.8009 -2.7532 -1.7819 -
0.7682 -0.8580 0.1504 0.4850 0.5866
1.6471 -2.3761 -418.1784
97.0400 -417.5307 0.0214 0.8642 -
0.2174 -0.7533 0.1972 0.4941 0.5906
1.6457 -2.3792 -418.1728
97.0600 -412.0094 3.5273 2.8596
0.2732 -0.5946 0.2412 0.5020 0.5946
1.6444 -2.3823 -418.1673
97.0800 -413.6802 1.8365 2.1846
0.5440 -0.3938 0.2816 0.5087 0.5987
1.6430 -2.3854 -418.1618
97.1000 -418.9256 -2.2120 -0.2270
0.5927 -0.1657 0.3178 0.5141 0.6029
1.6415 -2.3885 -418.1562
97.1200 -421.0026 -2.4591 -1.8355
0.4911 0.0728 0.3490 0.5185 0.6071
1.6401 -2.3916 -418.1507
97.1400 -418.6475 -0.2554 -1.5689
0.3200 0.3054 0.3749 0.5217 0.6114
1.6386 -2.3947 -418.1452
97.1600 -415.9913 1.1780 -0.3660
0.1490 0.5172 0.3948 0.5240 0.6157
1.6371 -2.3978 -418.1396
97.1800 -415.7940 0.5659 0.3863
0.0472 0.6947 0.4085 0.5252 0.6200
1.6355 -2.4009 -418.1341
97.2000 -416.1248 -0.1815 0.3083
0.0517 0.8293 0.4156 0.5255 0.6243
1.6340 -2.4040 -418.1286
97.2200 -416.2978 -0.1556 0.1701
0.1507 0.9163 0.4160 0.5251 0.6285
1.6324 -2.4071 -418.1230
97.2400 -416.0588 0.4686 0.1924
0.2969 0.9530 0.4096 0.5238 0.6328
1.6308 -2.4103 -418.1175
97.2600 -415.7445 -0.5918 0.1000
0.4467 0.9374 0.3964 0.5219 0.6370
1.6291 -2.4134 -418.1120
97.2800 -415.8228 0.1093 -0.0825
0.5663 0.8698 0.3767 0.5193 0.6412
1.6275 -2.4166 -418.1065
97.3000 -416.3222 0.2396 -0.2354
0.6070 0.7542 0.3509 0.5163 0.6453
1.6258 -2.4197 -418.1009
97.3200 -416.5970 -0.5852 -0.1840
0.5285 0.5979 0.3197 0.5128 0.6494
1.6241 -2.4229 -418.0954

97.3400 -416.1299 -0.6046 0.4852
0.3143 0.4109 0.2837 0.5089 0.6533
1.6224 -2.4260 -418.0899
97.3600 -415.4968 0.2330 1.3060
0.0072 0.2038 0.2437 0.5047 0.6572
1.6207 -2.4292 -418.0844
97.3800 -416.0617 0.8408 1.1189 -
0.3196 -0.0132 0.2005 0.5003 0.6609
1.6190 -2.4324 -418.0789
97.4000 -418.9405 -0.1967 -0.3036 -
0.5725 -0.2315 0.1547 0.4957 0.6646
1.6173 -2.4356 -418.0733
97.4200 -420.6999 -0.7243 -1.7996 -
0.6478 -0.4415 0.1072 0.4910 0.6681
1.6155 -2.4388 -418.0678
97.4400 -420.4650 0.2987 -2.2042 -
0.4750 -0.6321 0.0587 0.4863 0.6715
1.6137 -2.4420 -418.0623
97.4600 -419.8776 0.2118 -1.3964 -
0.0703 -0.7926 0.0099 0.4817 0.6747
1.6120 -2.4452 -418.0568
97.4800 -418.8345 -1.0763 0.2986
0.4258 -0.9120 -0.0385 0.4770 0.6778
1.6102 -2.4484 -418.0513
97.5000 -415.9000 -0.1030 2.0815
0.7806 -0.9796 -0.0856 0.4726 0.6807
1.6084 -2.4517 -418.0458
97.5200 -413.5190 1.4435 2.8189
0.7756 -0.9879 -0.1307 0.4683 0.6834
1.6066 -2.4549 -418.0403
97.5400 -415.5636 1.3698 1.5120
0.3389 -0.9347 -0.1731 0.4642 0.6860
1.6048 -2.4582 -418.0348
97.5600 -421.0781 -1.3398 -0.9412 -
0.4023 -0.8232 -0.2125 0.4604 0.6884
1.6029 -2.4614 -418.0293
97.5800 -423.1017 -1.5054 -2.2336 -
1.2185 -0.6613 -0.2483 0.4569 0.6905
1.6011 -2.4647 -418.0238
97.6000 -421.2759 0.0919 -1.3282 -
1.8479 -0.4590 -0.2805 0.4537 0.6925
1.5993 -2.4680 -418.0183
97.6200 -419.1486 0.9356 0.2286 -
2.0605 -0.2288 -0.3087 0.4508 0.6943
1.5975 -2.4713 -418.0128
97.6400 -418.8836 0.9599 0.3135 -
1.6901 0.0147 -0.3331 0.4483 0.6959
1.5956 -2.4746 -418.0073
97.6600 -419.2922 -0.1779 -0.9920 -
0.7652 0.2552 -0.3535 0.4462 0.6973
1.5938 -2.4779 -418.0018

97.6800	-419.5952	-1.0246	-1.6987	
0.4727	0.4756	-0.3699	0.4445	0.6984
	1.5920	-2.4812	-417.9963	
97.7000	-417.3111	-1.2300	-0.5217	
1.7240	0.6590	-0.3824	0.4430	0.6994
	1.5901	-2.4846	-417.9908	
97.7200	-412.5487	1.0870	1.4893	
2.7107	0.7899	-0.3909	0.4420	0.7001
	1.5883	-2.4879	-417.9853	
97.7400	-410.9063	1.0225	2.4243	
3.1832	0.8551	-0.3954	0.4412	0.7006
	1.5865	-2.4913	-417.9798	
97.7600	-412.8583	0.0836	1.6227	
2.9961	0.8472	-0.3958	0.4407	0.7008
	1.5847	-2.4947	-417.9743	
97.7800	-415.3694	-0.4316	0.1424	
2.2282	0.7718	-0.3921	0.4404	0.7009
	1.5828	-2.4981	-417.9688	
97.8000	-417.0382	-0.3775	-0.7204	
1.0856	0.6443	-0.3844	0.4403	0.7007
	1.5810	-2.5015	-417.9633	
97.8200	-418.4866	0.2724	-0.6588	-
0.1942	0.4821	-0.3726	0.4404	0.7003
	1.5792	-2.5049	-417.9579	
97.8400	-419.5878	-0.0693	-0.4136	-
1.3801	0.3024	-0.3566	0.4406	0.6996
	1.5774	-2.5083	-417.9524	
97.8600	-420.5814	0.5128	-0.3520	-
2.2916	0.1228	-0.3367	0.4408	0.6987
	1.5755	-2.5118	-417.9469	
97.8800	-421.1692	-0.7264	-0.1611	-
2.8429	-0.0392	-0.3129	0.4410	0.6976
	1.5737	-2.5152	-417.9414	
97.9000	-421.1370	0.0880	0.0909	-
3.0138	-0.1672	-0.2854	0.4411	0.6962
	1.5719	-2.5187	-417.9359	
97.9200	-420.9042	0.2086	0.0654	-
2.8242	-0.2492	-0.2545	0.4411	0.6946
	1.5701	-2.5222	-417.9304	
97.9400	-420.5940	-0.1376	-0.3031	-
2.3097	-0.2825	-0.2203	0.4408	0.6928
	1.5683	-2.5257	-417.9250	
97.9600	-420.2089	0.2025	-0.8223	-
1.5286	-0.2746	-0.1830	0.4402	0.6907
	1.5666	-2.5292	-417.9195	
97.9800	-419.6367	0.3085	-0.9493	-
0.5696	-0.2368	-0.1427	0.4392	0.6884
	1.5648	-2.5327	-417.9140	
98.0000	-418.6423	-0.9752	-0.1361	
0.4302	-0.1812	-0.0996	0.4378	0.6859
	1.5630	-2.5362	-417.9085	

98.0200 -415.8743 0.1800 1.0207
1.3111 -0.1209 -0.0537 0.4359 0.6832
1.5612 -2.5398 -417.9031
98.0400 -413.4811 0.9829 1.4513
1.9410 -0.0680 -0.0052 0.4333 0.6802
1.5595 -2.5433 -417.8976
98.0600 -414.0711 0.4262 0.7866
2.2456 -0.0344 0.0458 0.4302 0.6770
1.5577 -2.5469 -417.8921
98.0800 -416.3149 -0.5428 -0.2068
2.2034 -0.0292 0.0992 0.4263 0.6737
1.5560 -2.5505 -417.8866
98.1000 -417.1548 -1.1789 -0.4428
1.8494 -0.0573 0.1547 0.4215 0.6701
1.5542 -2.5541 -417.8812
98.1200 -416.3316 0.1156 0.0664
1.2909 -0.1187 0.2122 0.4160 0.6663
1.5525 -2.5577 -417.8757
98.1400 -415.4692 1.4308 0.2139
0.6834 -0.2102 0.2709 0.4094 0.6623
1.5508 -2.5614 -417.8702
98.1600 -416.8274 1.4126 -0.6923
0.1781 -0.3267 0.3304 0.4020 0.6581
1.5491 -2.5650 -417.8648
98.1800 -421.1151 -0.5633 -2.0208 -
0.1500 -0.4591 0.3898 0.3935 0.6537
1.5474 -2.5687 -417.8593
98.2000 -423.0001 -3.1797 -2.0344 -
0.2967 -0.5923 0.4481 0.3839 0.6492
1.5457 -2.5724 -417.8538
98.2200 -418.9338 -0.5073 0.1010 -
0.3150 -0.7070 0.5047 0.3731 0.6445
1.5440 -2.5760 -417.8484
98.2400 -413.6100 2.8516 2.5223 -
0.3370 -0.7840 0.5586 0.3613 0.6396
1.5423 -2.5798 -417.8429
98.2600 -413.3696 2.3761 2.8632 -
0.4915 -0.8071 0.6089 0.3482 0.6346
1.5406 -2.5835 -417.8374
98.2800 -418.6656 -0.6430 1.0460 -
0.8110 -0.7661 0.6549 0.3339 0.6294
1.5389 -2.5872 -417.8320
98.3000 -422.5691 -2.4863 -0.9625 -
1.1995 -0.6562 0.6956 0.3183 0.6241
1.5373 -2.5910 -417.8265
98.3200 -421.4362 -0.3396 -1.6033 -
1.5164 -0.4781 0.7298 0.3014 0.6186
1.5356 -2.5947 -417.8210
98.3400 -419.1240 0.6799 -1.0027 -
1.6369 -0.2370 0.7567 0.2831 0.6130
1.5340 -2.5985 -417.8156

98.3600 -418.1701 0.8931 -0.3586 -
1.4821 0.0563 0.7756 0.2636 0.6073
1.5323 -2.6023 -417.8101
98.3800 -418.1044 -0.1181 -0.3156 -
1.0531 0.3829 0.7857 0.2427 0.6015
1.5307 -2.6061 -417.8047
98.4000 -418.0482 -0.3425 -0.4375 -
0.4520 0.7192 0.7865 0.2205 0.5955
1.5290 -2.6100 -417.7992
98.4200 -417.2497 0.0801 0.1168
0.1697 1.0396 0.7774 0.1971 0.5895
1.5274 -2.6138 -417.7937
98.4400 -414.4383 -0.0488 1.1977
0.6895 1.3172 0.7580 0.1725 0.5834
1.5258 -2.6177 -417.7883
98.4600 -412.8028 0.9535 1.3603
1.0641 1.5257 0.7277 0.1467 0.5772
1.5242 -2.6215 -417.7828
98.4800 -414.8375 0.3049 0.0164
1.3047 1.6439 0.6863 0.1199 0.5709
1.5226 -2.6254 -417.7774
98.5000 -417.7925 -1.5759 -1.5232
1.4507 1.6575 0.6337 0.0921 0.5646
1.5210 -2.6293 -417.7719
98.5200 -417.6301 -1.3554 -1.6209
1.5133 1.5621 0.5704 0.0633 0.5582
1.5194 -2.6333 -417.7664
98.5400 -414.4547 0.8157 -0.0884
1.4510 1.3632 0.4973 0.0337 0.5518
1.5178 -2.6372 -417.7610
98.5600 -412.5014 0.6806 1.4858
1.1839 1.0767 0.4155 0.0034 0.5453
1.5162 -2.6411 -417.7555
98.5800 -414.5946 0.7711 1.6226
0.6601 0.7255 0.3266 -0.0275 0.5387
1.5146 -2.6451 -417.7501
98.6000 -418.1965 -0.7328 0.5178 -
0.0845 0.3369 0.2320 -0.0590 0.5322
1.5130 -2.6491 -417.7446
98.6200 -420.2573 0.0136 -0.5784 -
0.9146 -0.0607 0.1331 -0.0908 0.5256
1.5114 -2.6530 -417.7392
98.6400 -421.7491 -0.2757 -0.9808 -
1.6058 -0.4400 0.0315 -0.1229 0.5190
1.5098 -2.6570 -417.7337
98.6600 -422.4904 0.1377 -0.9403 -
1.9535 -0.7756 -0.0713 -0.1551 0.5123
1.5082 -2.6610 -417.7283
98.6800 -422.3523 -0.0639 -0.7729 -
1.8608 -1.0474 -0.1737 -0.1873 0.5057
1.5067 -2.6651 -417.7228

98.7000 -421.9131 0.0934 -0.4494 -
1.3575 -1.2439 -0.2741 -0.2195 0.4991
1.5051 -2.6691 -417.7174
98.7200 -421.3063 -0.4776 0.0693 -
0.6097 -1.3638 -0.3710 -0.2514 0.4925
1.5035 -2.6732 -417.7119
98.7400 -419.7907 0.0622 0.7902
0.1799 -1.4119 -0.4629 -0.2830 0.4859
1.5019 -2.6772 -417.7065
98.7600 -418.0985 0.2608 1.2915
0.8403 -1.3945 -0.5483 -0.3141 0.4794
1.5003 -2.6813 -417.7010
98.7800 -417.9757 1.0272 0.8216
1.2627 -1.3205 -0.6260 -0.3447 0.4729
1.4987 -2.6854 -417.6956
98.8000 -420.1690 -0.1871 -0.4812
1.4081 -1.2001 -0.6949 -0.3746 0.4664
1.4972 -2.6894 -417.6901
98.8200 -422.1714 -0.7628 -1.4095
1.2845 -1.0446 -0.7542 -0.4037 0.4599
1.4956 -2.6935 -417.6847
98.8400 -421.4089 -0.8836 -0.8696
0.9357 -0.8649 -0.8032 -0.4320 0.4535
1.4940 -2.6977 -417.6792
98.8600 -418.8077 0.9896 0.7365
0.4505 -0.6721 -0.8414 -0.4593 0.4472
1.4924 -2.7018 -417.6738
98.8800 -417.3001 0.6095 1.5343 -
0.0443 -0.4776 -0.8689 -0.4857 0.4409
1.4908 -2.7059 -417.6683
98.9000 -419.6441 0.5118 0.4424 -
0.4296 -0.2914 -0.8858 -0.5109 0.4347
1.4892 -2.7100 -417.6629
98.9200 -423.4605 -0.8781 -1.3326 -
0.6350 -0.1191 -0.8929 -0.5350 0.4286
1.4876 -2.7142 -417.6574
98.9400 -424.1300 -1.7195 -1.8277 -
0.6360 0.0374 -0.8913 -0.5580 0.4224
1.4860 -2.7183 -417.6520
98.9600 -420.7070 -0.0798 -0.4441 -
0.4636 0.1767 -0.8823 -0.5800 0.4164
1.4844 -2.7225 -417.6465
98.9800 -417.0322 2.0015 1.1643 -
0.2190 0.3015 -0.8674 -0.6011 0.4104
1.4828 -2.7266 -417.6411
99.0000 -416.6379 1.6315 1.3199
0.0038 0.4193 -0.8495 -0.6217 0.4044
1.4812 -2.7308 -417.6356

DATA:
Time-varying Shannon entropy (SE) and 95% significance level of DO modes extracted by EEMD
(shown in the 5-8 columns in the above).

Column 1: Time (kyr. BP)
Column 2: Shannon entropy of IMF2 (SE2)
Column 3: 95% significance level of SE of IMF2 (95% SL2)
Column 4: Shannon entropy of IMF3 (SE3)
Column 5: 95% significance level of SE of IMF3 (95% SL3)
Column 6: Shannon entropy of IMF4 (SE4)
Column 7: 95% significance level of SE of IMF4 (95% SL4)
Column 8: Shannon entropy of IMF5 (SE5)
Column 9: 95% significance level of SE of IMF5 (95% SL5)
Column 10: Shannon entropy of IMF6 (SE6)
Column 11: 95% significance level of SE of IMF6 (95% SL6)

Time	SE2	95% SL2	SE3	95% SL3	SE4	95% SL4	SE5	95% SL5	SE6	95% SL6
4.9900	1.4756	1.4244	1.4058	1.5505	1.6546	1.6584	1.5719	1.7296	1.4038	1.9057
5.5900	1.4497	1.4117	1.3936	1.5598	1.7435	1.6601	1.5805	1.7352	1.4493	1.8954
6.1900	1.5169	1.4185	1.3464	1.5685	1.7884	1.6586	1.4326	1.7393	1.5599	1.9034
6.7900	1.5747	1.4169	1.3552	1.5562	1.6811	1.6464	1.4169	1.7238	1.5088	1.8936
7.3900	1.4864	1.4231	1.3593	1.5517	1.6866	1.6585	1.4175	1.7253	1.5498	1.8967
7.9900	1.5905	1.4211	1.3648	1.5581	1.6879	1.6536	1.4814	1.7200	1.6067	1.8928
8.5900	1.0647	1.4200	1.3476	1.5589	1.5473	1.6194	1.2213	1.7148	1.7058	1.8983
9.1900	1.0644	1.4287	1.3612	1.5580	1.5446	1.6412	1.5542	1.7144	1.7637	1.8994
9.7900	1.0722	1.4289	1.3683	1.5523	1.5658	1.6422	1.6509	1.7223	1.4792	1.8956
10.3900	0.9855	1.4312	1.2905	1.5551	1.6729	1.6507	1.6490	1.7330	1.5931	1.9140

10.9900 0.9844 1.4229 1.1813
1.5527 1.7788 1.6329 1.5911 1.7303
1.6619 1.9104
11.5900 0.9907 1.4199 1.2745
1.5541 0.9366 1.6318 1.6903 1.7336
1.7103 1.9113
12.1900 0.9866 1.4213 1.2785
1.5561 1.2223 1.6432 1.7024 1.7270
1.6727 1.9016
12.7900 0.9781 1.4346 1.2762
1.5255 1.2400 1.6474 1.6920 1.7270
1.6519 1.9056
13.3900 0.9730 1.4219 1.2701
1.5331 1.2411 1.6442 1.6941 1.7162
1.6960 1.8998
13.9900 0.9551 1.4211 1.2675
1.5393 1.2395 1.6589 1.6637 1.7211
1.7462 1.9064
14.5900 1.4097 1.4202 1.2774
1.5427 1.2522 1.6717 1.4288 1.7183
1.7701 1.8933
15.1900 1.4795 1.4258 1.2554
1.5535 1.2494 1.6617 1.3041 1.7214
1.7553 1.8956
15.7900 1.3973 1.4186 1.2517
1.5569 1.2393 1.6555 1.2213 1.7305
1.6870 1.9081
16.3900 1.3589 1.4180 1.3781
1.5548 1.2592 1.6555 1.1453 1.7299
1.8189 1.9068
16.9900 1.2661 1.4293 1.1268
1.5544 1.2569 1.6578 0.9790 1.7239
1.8737 1.9041
17.5900 1.2158 1.4297 1.0825
1.5436 0.9051 1.6545 1.6827 1.7313
1.8172 1.9089
18.1900 1.0579 1.4181 1.0845
1.5574 1.5393 1.6640 1.6452 1.7329
1.6311 1.9032
18.7900 1.1195 1.4174 1.1625
1.5560 1.5637 1.6645 1.5542 1.7455
1.4796 1.9150
19.3900 1.1273 1.4137 1.1680
1.5415 1.6512 1.6558 1.5408 1.7406
1.4708 1.9111
19.9900 1.1292 1.4258 1.1642
1.5457 1.6436 1.6608 1.4797 1.7265
1.3594 1.9021
20.5900 1.1229 1.4239 1.1541
1.5391 1.5967 1.6494 1.4561 1.7158
1.2876 1.8969

21.1900 1.1197 1.4169 1.1602
1.5531 1.6334 1.6407 1.5442 1.7184
1.2063 1.8904
21.7900 1.2312 1.4169 1.3289
1.5448 1.6508 1.6367 1.5838 1.7402
1.6823 1.8814
22.3900 1.1114 1.4248 1.3332
1.5546 1.6247 1.6383 1.5808 1.7343
1.6458 1.8850
22.9900 1.1138 1.4324 1.2055
1.5537 1.6094 1.6536 1.5489 1.7357
1.5365 1.8873
23.5900 1.1341 1.4295 1.1962
1.5535 1.6034 1.6530 1.5571 1.7264
1.6200 1.8984
24.1900 1.1315 1.4375 1.1994
1.5522 1.6153 1.6465 1.6293 1.7340
1.6932 1.8968
24.7900 1.1232 1.4308 0.9849
1.5376 1.6451 1.6506 1.3827 1.7380
1.6764 1.8951
25.3900 1.1316 1.4369 0.9867
1.5405 1.1402 1.6629 1.4943 1.7351
1.7204 1.8943
25.9900 1.1266 1.4271 0.9868
1.5514 1.3042 1.6478 1.4815 1.7366
1.5532 1.8995
26.5900 1.1292 1.4166 0.9899
1.5577 1.2940 1.6503 1.6201 1.7389
1.6076 1.8984
27.1900 1.1348 1.4248 0.9999
1.5532 1.1841 1.6512 1.6292 1.7453
1.6694 1.8941
27.7900 1.0689 1.4306 0.9597
1.5464 1.1830 1.6489 1.6724 1.7316
1.7173 1.8972
28.3900 1.0826 1.4348 0.9598
1.5636 1.1938 1.6539 1.5160 1.7394
1.7814 1.9031
28.9900 1.1214 1.4224 0.9934
1.5475 1.3100 1.6417 1.5853 1.7211
1.8622 1.8927
29.5900 1.1187 1.4193 0.9980
1.5579 1.3219 1.6468 1.5918 1.7270
1.8767 1.8938
30.1900 1.1288 1.4176 0.9785
1.5557 1.3456 1.6545 1.7400 1.7315
1.8014 1.9043
30.7900 1.0963 1.4325 1.2631
1.5371 1.3458 1.6585 1.7105 1.7231
1.7802 1.9024

31.3900 1.0465 1.4341 1.2660
1.5458 1.2677 1.6584 1.7352 1.7361
1.9313 1.8955
31.9900 1.0579 1.4339 1.2671
1.5382 1.4476 1.6484 1.7223 1.7351
1.5980 1.8879
32.5900 1.2234 1.4213 1.2680
1.5396 1.4305 1.6506 1.7337 1.7250
1.7591 1.8897
33.1900 1.2187 1.4241 1.6300
1.5381 1.4125 1.6417 1.7246 1.7254
1.6133 1.8981
33.7900 1.2142 1.4235 1.6373
1.5434 1.5643 1.6424 1.7203 1.7345
1.7020 1.8992
34.3900 1.2110 1.4229 1.2159
1.5397 1.6523 1.6521 1.6693 1.7329
1.7711 1.8963
34.9900 1.0959 1.4182 1.0623
1.5427 1.4021 1.6406 1.6555 1.7293
1.8074 1.8972
35.5900 1.1195 1.4279 1.0691
1.5429 1.3888 1.6453 1.5896 1.7353
1.9144 1.8991
36.1900 1.2148 1.4260 1.0889
1.5478 1.2970 1.6635 1.4395 1.7349
1.8431 1.8948
36.7900 1.2101 1.4237 1.1022
1.5414 1.2972 1.6361 1.8131 1.7357
1.8749 1.9020
37.3900 1.2143 1.4285 1.2322
1.5537 1.3036 1.6310 1.7352 1.7300
1.8850 1.9035
37.9900 1.2015 1.4201 1.2596
1.5493 1.3221 1.6441 1.7509 1.7283
1.8817 1.8974
38.5900 1.1175 1.4360 1.2652
1.5472 1.3381 1.6339 1.6306 1.7354
1.8292 1.9004
39.1900 1.1144 1.4334 1.2301
1.5453 1.3305 1.6383 1.8094 1.7199
1.8074 1.9002
39.7900 1.1294 1.4315 1.2309
1.5482 1.3347 1.6549 1.8146 1.7273
1.8294 1.9086
40.3900 1.1386 1.4220 1.2140
1.5400 1.0930 1.6486 1.8455 1.7201
1.7914 1.8988
40.9900 0.9952 1.4201 1.2286
1.5545 1.7043 1.6464 1.8333 1.7305
1.8028 1.8882

41.5900 1.0063 1.4197 1.2214
1.5579 1.6297 1.6319 1.7932 1.7341
1.8129 1.8947
42.1900 1.1865 1.4199 1.2057
1.5380 1.6142 1.6414 1.6821 1.7369
1.7700 1.8963
42.7900 1.1172 1.4140 1.1903
1.5522 1.5266 1.6383 1.6366 1.7250
1.8064 1.9031
43.3900 0.9771 1.4206 0.8907
1.5393 1.0858 1.6531 1.6119 1.7271
1.7240 1.8959
43.9900 0.9843 1.4276 1.0910
1.5482 1.3132 1.6459 1.5433 1.7257
1.7624 1.9017
44.5900 0.9554 1.4313 1.1103
1.5279 1.3240 1.6374 1.5571 1.7248
1.8790 1.9018
45.1900 1.1801 1.4237 1.1130
1.5573 1.4705 1.6507 1.5317 1.7300
1.8666 1.9038
45.7900 1.2123 1.4258 1.1315
1.5537 1.5575 1.6527 1.5899 1.7291
1.8133 1.9076
46.3900 1.0568 1.4204 1.1296
1.5445 1.5776 1.6499 1.4236 1.7357
1.7663 1.8998
46.9900 1.0833 1.4168 1.1212
1.5507 1.5900 1.6475 1.4880 1.7447
1.7678 1.9018
47.5900 1.0889 1.4161 1.1318
1.5346 1.5817 1.6530 1.5287 1.7351
1.7623 1.9020
48.1900 1.1139 1.4176 1.1436
1.5505 1.5803 1.6559 1.5346 1.7362
1.7411 1.8960
48.7900 1.0817 1.4286 1.1534
1.5529 1.5860 1.6548 1.4497 1.7362
1.6289 1.9020
49.3900 1.0682 1.4225 1.3510
1.5435 1.5670 1.6674 1.6244 1.7316
1.6638 1.8919
49.9900 1.1565 1.4118 1.3568
1.5457 1.3941 1.6536 1.5641 1.7253
2.0166 1.8916
50.5900 1.0980 1.4216 1.3564
1.5479 1.5737 1.6400 1.7054 1.7223
2.0159 1.8986
51.1900 1.0701 1.4277 1.4063
1.5560 1.4300 1.6412 1.7397 1.7265
2.0102 1.9030

51.7900 1.0487 1.4247 1.4135
1.5596 1.4407 1.6522 1.6577 1.7280
1.9668 1.8990

52.3900 1.2073 1.4277 1.4097
1.5469 1.4392 1.6656 1.5872 1.7202
1.8882 1.9024

52.9900 1.0113 1.4287 1.4114
1.5537 1.4183 1.6593 1.6534 1.7346
1.8488 1.8943

53.5900 1.0139 1.4257 1.3880
1.5514 1.4259 1.6571 1.6551 1.7248
1.9548 1.9020

54.1900 1.0039 1.4376 1.4559
1.5564 1.4346 1.6617 1.6961 1.7268
1.9522 1.8961

54.7900 1.1612 1.4294 1.4860
1.5549 1.4269 1.6499 1.7630 1.7324
1.9087 1.8952

55.3900 1.1621 1.4398 1.4700
1.5566 1.3992 1.6453 1.8503 1.7256
1.9112 1.8983

55.9900 1.1091 1.4081 1.4585
1.5462 1.3739 1.6553 1.8182 1.7377
1.8937 1.9020

56.5900 1.1083 1.4302 1.4565
1.5454 1.1495 1.6376 1.7743 1.7338
1.7960 1.8987

57.1900 1.1183 1.4235 1.2508
1.5461 1.1700 1.6283 1.7840 1.7407
1.8341 1.8933

57.7900 1.1260 1.4265 1.2347
1.5454 1.3027 1.6383 1.7369 1.7432
1.8430 1.8969

58.3900 1.1169 1.4341 1.2315
1.5441 1.5186 1.6533 1.5323 1.7354
1.7035 1.9065

58.9900 1.1091 1.4328 1.2238
1.5465 1.6085 1.6526 1.5282 1.7439
1.6521 1.9020

59.5900 1.0999 1.4218 1.2457
1.5299 1.5883 1.6363 1.5044 1.7284
1.5791 1.9057

60.1900 1.1081 1.4030 1.2356
1.5378 1.5824 1.6337 1.4577 1.7181
1.6106 1.9020

60.7900 1.0923 1.4050 1.2961
1.5499 1.3086 1.6299 1.2063 1.7280
1.7353 1.8932

61.3900 1.0930 1.4235 1.4038
1.5422 1.4026 1.6308 1.3915 1.7359
1.7131 1.8950

61.9900 1.0969 1.4265 1.4075
1.5396 1.4067 1.6311 1.3680 1.7437
1.7395 1.9026
62.5900 1.3733 1.4165 1.4113
1.5512 1.4051 1.6424 1.4189 1.7381
1.7262 1.8894
63.1900 1.3470 1.4141 1.1365
1.5428 1.3757 1.6251 1.4321 1.7413
1.7312 1.9071
63.7900 1.2227 1.4086 1.1247
1.5447 1.3626 1.6480 1.4450 1.7441
1.7233 1.8986
64.3900 1.0672 1.4129 1.1194
1.5519 1.3552 1.6569 1.4732 1.7371
1.6980 1.9051
64.9900 1.3016 1.4138 1.1508
1.5520 1.3946 1.6532 1.4780 1.7496
1.6772 1.9094
65.5900 1.2960 1.4384 1.1350
1.5433 1.1053 1.6490 1.5521 1.7412
1.5343 1.9104
66.1900 1.2915 1.4324 1.1122
1.5376 1.2417 1.6501 1.5498 1.7383
1.5289 1.9175
66.7900 1.2427 1.4165 1.2233
1.5444 1.1199 1.6529 1.3464 1.7301
1.5519 1.9136
67.3900 1.2556 1.4251 1.1304
1.5404 1.1061 1.6546 1.5829 1.7349
1.3610 1.9053
67.9900 1.1210 1.4236 1.1354
1.5367 1.1841 1.6447 1.6633 1.7313
1.4267 1.9017
68.5900 1.0141 1.4261 1.1867
1.5412 1.1851 1.6476 1.6671 1.7404
1.5249 1.8981
69.1900 1.0173 1.4262 1.1836
1.5532 1.1737 1.6389 1.8212 1.7423
1.5922 1.8997
69.7900 0.9759 1.4157 1.1953
1.5619 1.1756 1.6474 1.5609 1.7378
1.6497 1.9088
70.3900 0.9773 1.4211 1.1932
1.5483 1.1823 1.6478 1.5693 1.7621
1.8331 1.9109
70.9900 0.9681 1.4212 1.1636
1.5582 1.1767 1.6579 1.5728 1.7487
1.8927 1.9116
71.5900 0.9725 1.4336 1.1683
1.5699 1.5388 1.6602 1.5805 1.7453
1.8933 1.9056

72.1900 0.9567 1.4279 1.1833
1.5668 1.4484 1.6598 1.5536 1.7471
1.9072 1.9113
72.7900 0.9339 1.4287 1.4561
1.5710 1.4504 1.6523 1.6267 1.7345
1.8745 1.9088
73.3900 0.9427 1.4273 1.3554
1.5548 1.4496 1.6602 1.6342 1.7449
1.8582 1.9029
73.9900 0.8946 1.4270 1.3631
1.5470 1.1872 1.6444 1.5939 1.7295
1.9199 1.8976
74.5900 1.0833 1.4270 1.3455
1.5449 1.1912 1.6496 1.5948 1.7532
1.8572 1.9113
75.1900 1.0866 1.4344 1.3454
1.5394 1.2077 1.6450 1.5846 1.7458
1.7376 1.8982
75.7900 0.9971 1.4322 1.3692
1.5242 1.2851 1.6385 1.7072 1.7340
1.6705 1.9046
76.3900 1.0145 1.4274 1.3884
1.5539 1.2966 1.6412 1.6088 1.7285
1.6606 1.9063
76.9900 1.2004 1.4166 1.2913
1.5541 1.2904 1.6458 1.5704 1.7241
1.4546 1.9052
77.5900 1.2102 1.4212 1.2831
1.5384 1.5177 1.6470 1.4384 1.7242
1.3876 1.8988
78.1900 1.2177 1.4220 1.2786
1.5369 1.4748 1.6582 1.6077 1.7420
1.6364 1.8997
78.7900 1.2295 1.4195 1.5070
1.5308 1.5596 1.6541 1.3935 1.7354
1.5426 1.9019
79.3900 1.2250 1.4132 1.3293
1.5406 1.5995 1.6512 1.5143 1.7382
1.8926 1.9034
79.9900 1.2063 1.4201 1.3189
1.5435 1.6062 1.6525 1.5071 1.7463
1.8327 1.8939
80.5900 1.1953 1.4210 1.3106
1.5406 1.4993 1.6544 1.6409 1.7307
1.8808 1.9032
81.1900 1.2069 1.4201 1.4160
1.5594 1.4888 1.6583 1.6658 1.7375
1.8074 1.8997
81.7900 1.1947 1.4190 1.4148
1.5560 1.4832 1.6585 1.4860 1.7410
1.7791 1.8996

82.3900 1.2876 1.4285 1.4993
1.5546 1.4720 1.6414 1.4119 1.7395
1.7806 1.8928
82.9900 1.2747 1.4370 1.3346
1.5471 1.4731 1.6468 1.4293 1.7443
1.7942 1.8891
83.5900 1.2690 1.4305 1.3302
1.5636 1.4794 1.6411 1.4229 1.7415
1.7352 1.8860
84.1900 1.2732 1.4239 1.3414
1.5569 1.6668 1.6327 1.3340 1.7373
1.6611 1.9016
84.7900 1.2788 1.4190 1.1760
1.5377 1.6718 1.6355 1.4105 1.7317
1.5770 1.8980
85.3900 1.2825 1.4036 1.1816
1.5407 1.5192 1.6379 1.4434 1.7473
1.7980 1.8930
85.9900 1.1211 1.4121 1.1778
1.5392 1.5447 1.6285 1.4525 1.7475
1.8131 1.8988
86.5900 1.1292 1.4082 1.2004
1.5578 1.4201 1.6165 1.4425 1.7377
1.8079 1.8997
87.1900 1.0940 1.4160 1.2152
1.5600 1.5426 1.6117 1.5124 1.7295
1.8326 1.9007
87.7900 1.1326 1.4204 1.2371
1.5456 1.5540 1.6230 1.6122 1.7267
1.8674 1.9023
88.3900 1.0823 1.4296 1.1843
1.5432 1.6020 1.6296 1.6208 1.7280
1.8685 1.8977
88.9900 1.0806 1.4240 1.2081
1.5540 1.6083 1.6353 1.6178 1.7267
1.8937 1.9097
89.5900 1.0865 1.4215 1.2192
1.5406 1.4973 1.6493 1.6217 1.7377
1.8898 1.9080
90.1900 1.0857 1.4144 1.2124
1.5423 1.5838 1.6439 1.5986 1.7453
1.8603 1.8960
90.7900 1.2105 1.4193 1.3603
1.5391 1.5798 1.6389 1.3329 1.7234
1.8099 1.8942
91.3900 1.4022 1.4185 1.3523
1.5314 1.5749 1.6522 1.5000 1.7345
1.6949 1.8985
91.9900 1.4243 1.4136 1.2568
1.5199 1.5090 1.6550 1.1756 1.7382
1.5045 1.8922

92.5900 1.4127 1.4159 1.2516
1.5267 1.4005 1.6575 1.2533 1.7530
1.4346 1.8956
93.1900 1.1543 1.4230 1.1730
1.5514 1.3769 1.6520 1.2434 1.7439
1.4251 1.9036
93.7900 1.2871 1.4193 1.1338
1.5413 1.3645 1.6507 1.2078 1.7385
1.7024 1.8985
94.3900 1.2835 1.4364 1.1261
1.5421 1.1313 1.6461 1.1977 1.7360
1.5889 1.9007
94.9900 1.0188 1.4296 1.2436
1.5444 1.1147 1.6387 1.1896 1.7397
1.6040 1.8990
95.5900 1.0191 1.4307 1.2403
1.5392 1.1287 1.6381 1.2053 1.7405
1.6159 1.8962
